# Genome-wide analysis of the transcriptional response to drought stress in root and leaf of common bean

**DOI:** 10.1590/1678-4685-GMB-2018-0259

**Published:** 2020-03-16

**Authors:** Wendell Jacinto Pereira, Arthur Tavares de Oliveira Melo, Alexandre Siqueira Guedes Coelho, Fabiana Aparecida Rodrigues, Sujan Mamidi, Sérgio Amorim de Alencar, Anna Cristina Lanna, Paula Arielle Mendes Ribeiro Valdisser, Claudio Brondani, Ivanildo Ramalho do Nascimento-Júnior, Tereza Cristina de Oliveira Borba, Rosana Pereira Vianello

**Affiliations:** 1 Universidade Federal de Goiás, Instituto de Ciências Biológicas, Goiânia, GO, Brazil.; 2 Universidade de Brasília, Departamento de Biologia Celular, Brasília, DF, Brazil.; 3 Universidade Federal de Goiás, Escola de Agronomia, Goiânia, GO, Brazil.; 4 EMBRAPA Soja, Rod. Carlos João Strass, s/n, Londrina, PR, Brazil.; 5 Genome Sequencing Center, HudsonAlpha Institute for Biotechnology, Huntsville, AL, USA.; 6 Universidade Católica de Brasília, Programa de Pós-Graduação em Ciências Genômicas e Biotecnologia, Brasília, DF, Brazil.; 7 EMBRAPA Arroz e Feijão, Rod. GO - 462, Km 12, Santo Antônio de Goiás, GO, Brazil.; 8 Universidade Estadual Paulista, Programa de Pós-Graduação em Genética e Melhoramento de Plantas, Jaboticabal, SP, Brazil.

**Keywords:** Phaseolus vulgaris, water deficit, functional genomics, single nucleotide polymorphism (SNP)

## Abstract

Genes related to the response to drought stress in leaf and root tissue of drought-susceptible (DS) and tolerant (DT) genotypes were characterized by RNA-Seq. In total, 54,750 transcripts, representative of 28,590 genes, were identified; of these, 1,648 were of high-fidelity (merge of 12 libraries) and described for the first time in the Andean germplasm. From the 1,239 differentially expressed genes (DEGs), 458 were identified in DT, with a predominance of genes in categories of oxidative stress, response to stimulus and kinase activity. Most genes related to oxidation-reduction terms in roots were early triggered in DT (T75) compared to DS (T150) suggestive of a mechanism of tolerance by reducing the damage from ROS. Among the KEGG enriched by DEGs up-regulated in DT leaves, two related to the formation of Sulfur-containing compounds, which are known for their involvement in tolerance to abiotic stresses, were common to all treatments. Through qPCR, 88.64% of the DEGs were validated. A total of 151,283 variants were identified and functional effects estimated for 85,780. The raw data files were submitted to the NCBI database. A transcriptome map revealed new genes and isoforms under drought. These results supports a better understanding of the drought tolerance mechanisms in beans.

## Introduction

Drought has become increasingly intense and worrisome because the worldwide demand for agricultural products is expected to increase over the coming decade (FAO Land and Water, 2013). During evolution, climate changes have imposed selective pressure on genetic diversity plays an important role in the adaptive response to environmental changes (Ravenscroft *et al.*, 2015). This directional selection pressure provides an increased allelic frequency that is most advantageous for the adaptive response (Jump *et al.*, 2009; Anderson *et al.*, 2012). The impact on farming systems has accelerated the search for cultivated varieties better adapted to actual climate change in order to reduce vulnerabilities in cropping systems (Alemayehu *et al.*, 2015; Fisher *et al.*, 2015). According to the Food and Agriculture Organization of the United Nations (FAO), the term “drought” in agriculture means an insufficient amount of water in the soil to meet plant demands at a particular time. Consequently, plants under drought stress activate different mechanisms of resistance, maintaining a balance to optimize yield and survive (Zhang, 2007).

The common bean (*Phaseolus vulgaris* L.) is a new world crop (Gentry, 1969) that is grown extensively on all continents as a monoculture or in intercropping systems, from lowland tropics to semiarid regions in temperate environments, with or without irrigation (Thung and Rao, 1999). As a legume, it is considered the major source of nutrients for millions of people in developing countries and an important source of total protein, energy and micronutrients worldwide (Petry *et al.*, 2015). Drought has a great impact on the global production of common bean, resulting in yield losses of over 60% under terminal or intermittent drought stress, with more critical effects when the soil water depletion level reaches 60-70% during the grain-filling period (White and Singh, 1991; Beebe *et al.*, 2013). In Latin America, the areas of bean growth most affected by drought are northeastern Brazil and the central and northern highlands of Mexico (Wortmann *et al.*, 1998). Lines with higher yield under drought have resulted from a cross of races from the Mesoamerican genepool, Durango and Mesoamerica (Singh *et al.*, 2011), followed by excellent adaptation of the small-seeded beans in Central America and Brazil (Beebe *et al.*, 2013). Despite global efforts, due to the highly complex plant response to drought, the precise correlation between genes and drought tolerance remains to be demonstrated.

Efforts have been made to identify the broad set of mechanisms associated with enhanced drought tolerance under water restriction in Mesoamerican dry bean genotypes (Beebe *et al.*, 2013; Rosales *et al.*, 2013; Lanna *et al.*, 2016). An increasing amount of common bean genomic data is becoming available to the scientific community, allowing for more accurate and predictive research based on genomic information. In 2014, the Andean common bean genome was made available, estimated at 587 mega base pairs (Mbp) in size, with ~27 thousand genes, of which 91% were clustered in synteny blocks with *Glycine max* (Schmutz *et al.*, 2014). More recently, the Mesoamerican genome was estimated at 549.6Mbp, with ~30 thousand genes, of which 94% were functionally annotated (Vlasova *et al.*, 2016). The identification of drought-responsive genes through expression profiling studies is favoured as more genome information becomes available. Blair *et al.* (2011) identified and characterized 4,219 unigenes from cDNA libraries prepared using contrasting genotypes under drought stress, low soil phosphorus and high soil aluminium toxicity, contributing to thousands of newly described sequences for common beans. Subsequently, Recchia *et al.* (2013), using a suppression subtractive hybridization (SSH) library, identified 1,120 differentially expressed genes (DEGs) in the roots of a drought-tolerant (DT) genotype (BAT 477) when irrigation was halted during its development. Also using BAT 477, Müller *et al.* (2014a) identified 802 differentially expressed sequence tags (ESTs) in the leaf during the flowering and grain-filling developmental stages. Based on contrasting DT (Long 22-0579) and drought-sensitive (DS; Naihua) genotypes from different gene pools, 49Mbp of unique transcriptome sequences, of which 42% were annotated, 18% were assigned to Gene Ontology (GO) terms, and 8,932 were DEGs (Wu *et al.*, 2014). Using quantitative real-time PCR (qPCR), the differential expression profiles of several drought tolerance-related genes were investigated in response to stress. Many of the identified and validated genes encode, among others, transcription factors (TFs), oxidative stress-responsive proteins and proteins related to the components of photosystem II (Recchia *et al.*, 2013; Müller *et al.*, 2014b; Wu *et al.*, 2014).

Here, we report the expression profiling of the two contrasting drought-responsive genotypes BAT 477 (DT) and Pérola (DS) using RNA sequencing (RNA-Seq) and multiple bioinformatics resources to explore the potential candidate drought-responsive genes in leaves and roots. Based on the Andean genome, new genes and isoforms were identified in this study and provide a drought transcriptome map for common bean, and more than 150,000 variants (single nucleotide polymorphism singe nucleotide polymorphism - SNP and indels were characterized according to the SNP effect. In addition, non-coding RNAs and long non-coding RNAs (lncRNAs) were identified as differentially expressed in response to drought conditions. From the Kyoto Encyclopedia of Genes and Genomes (KEGG), 70 metabolic pathways were identified for newly identified genes, and 97 metabolic pathways for DEGs. These results provide a broad overview of transcriptional regulation in response to drought, enriching the public database of genes potentially involved in abiotic stress.

## Materials and Methods

### Plant material

Two well-known genotypes of the common bean, BAT 477 and Pérola, characterized according to their contrasting drought tolerances (Lanna *et al.*, 2016), were used in the present study. The lineage BAT 477, characterized as DT, was developed at the International Center for Tropical Agriculture (CIAT, Cali, Colombia), while Pérola, characterized as DS, was developed in the EMBRAPA (Brazilian Company for Agricultural Research) breeding programme in 1994 (Guimarães *et al.*, 2002). Both lineages are of Mesoamerican origin and present growth habit type III (Terán and Singh 2002; Gonçalves *et al.*, 2009).

### Experimental conditions

Both varieties were maintained in a controlled environment (hydroponics system) with a specific nutrient solution, according to the recommendations of Martins *et al.* (2008). Briefly, the plants were grown in plastic containers with an aerated pH 6.6-balanced nutrient solution (Hewitt, 1963). The seeds were pre-germinated on moist filter paper in a dark room with controlled temperature and relative humidity. The seedlings were placed in tray supports, their roots remained completely immersed in nutritive solution, and the hydroponic system was maintained in a greenhouse at 25 °C and 60% relative humidity under natural daylight. After 15 days, the seedlings were submitted to the treatments, in which they were completely removed from the hydroponic solution (T_0_; control), 75 min (T_75_), or 150 min (T_150_). The experiment was conducted in the form of random complete blocks with five replications and in a double factorial arrangement, with two sampled genotypes and water regime as factors.

### Physiological evaluation and statistical analysis

The physiological evaluations consisted of seven treatments: 0min (T_0_; control), 25 min (T_25_), 50 min (T_50_), 75 min (T_75_), 100 min (T_100_), 125 min (T_125_) and 150 min (T_150_). In all treatments, the photosynthetic rate (μmol CO_2_ m^-2^s^-1^), stomatal conductance (mmol H_2_O m^-2^s^-1^), leaf temperature (°C) and leaf transpiration rate (mmol H_2_O m-^2^ s-^1^) were evaluated using an LI-6400 Portable Photosynthesis System model (LI-COR Inc., Lincoln, Nebraska, USA). Measurements were taken on an expanded leaf subjected to progressive water deficit treatments for the periods of 0 min, 25 min, 50 min, 75 min, 100 min, 125 min, or 150 min. The osmotic (Ψ_s_) and hydric (Ψ_w_) potentials in megapascals (MPa), were taken according to the methodology described by Bajji *et al.*, 2001. The data were submitted to ANOVA using the SAS (Statistical Analysis System v.7.0, SAS Institute Inc., Cary, North Carolina, USA) program and the treatment means were compared using Tukey’s test at 95% statistical significance.

### Library preparation and RNA-Seq

Fresh leaves and roots from three treatments (T_0_, T_75_ and T_150_) for both lineages (BAT 477 and Pérola) were sampled from 15-day-old seedlings and were stored at -80 °C, totaling twelve different RNA-Seq libraries. From the five biological replicates conducted for each treatment, two were individually collected, processed and bulked with equimolar amounts of RNA. Total RNA was extracted using a commercial PureLink^®^ RNA Mini Kit (Thermo Fisher) following the manufacturer’s instructions. RNA quantity and purity were estimated through spectrophotometry using a NanoVue Plus Spectrophotometer (General Electric Co., Boston, Massachusetts, USA), and the integrity was verified using microfluidics technology and an Agilent 2100 Bioanalyzer (General Electric). The RNA-Seq libraries were prepared using the Illumina TruSeq RNA kit (Illumina, Inc., San Diego, California, USA) following the manufacturer’s instructions and were paired-end sequenced in a technical replicate system using two Illumina platforms (GAII and HiSeq 2000).

### Pre-processing data

The paired-end reads quality was first visualized using the FastQC v.0.11.3 (Andrews, 2010). The sequences were trimmed to eliminate adapters and low-quality bases allowing a maximum of two mismatches between the adapter sequences and reads. In addition, sequences of four contiguous nucleotides with an average score Q ≤ 30 and reads shorter than 50 base pairs (bp) were trimmed. The procedures were performed using Trimmomatic v.0.35 (Bolger *et al.*, 2014). Finally, the high-quality paired-end and single-end (orphan) reads were retained for further analysis.

### *P. vulgaris* drought-specific transcriptome assembly

The high-quality reads were mapped to the reference Andean genome using TopHat v2.1.0. (Trapnell *et al.*, 2009), and duplicated reads removed using Picard tools (http://broadinstitute.github.io/picard/). The transcriptome was assembled using reads that mapped to each of those twelve RNA-Seq libraries using the Cufflinks v2.2.1 (Trapnell *et al.*, 2010; Roberts *et al.*, 2011a) in the RABT mode (Roberts *et al.*, 2011b). An alternative isoform was set when the abundance was at least 20% compared with the principal isoform (-F=0.2). The reference genome and gene annotation (GFF3 file), available at Phytozome v.11.0 for *P. vulgaris* (Schmutz *et al.*, 2014), were used to guide the assembly. In addition, reads that represent repetitive DNA sequence regions were masked, and to obtain a single representative transcriptome for all libraries, the individual assembled transcripts (12 libraries) were merged into a single file using the Cuffmerge algorithm following the default parameters. A comparative analysis was also performed using the Cuffcompare algorithm to evaluate the transcripts overlapping between the transcriptomes available in the Phytozome v.11.0 database, and the drought-specific transcriptome was assembled. Finally, reads from the drought-specific transcriptome were appropriately sampled.

### Differential gene expression (DGE) analysis

The gene expression quantification was normalized to reads per kilobase of transcripts per million reads (FPKM) using the reference genome. After that, the DEGs were identified between-sample comparison (Table 1) using the edgeR (Robinson *et al.*, 2010) Bioconductor package. The abundance of reads was quantified using HTSeq (Anders *et al.*, 2015), and only genes with counts per million (CPM) > 1 in at least two samples were kept in the analysis. The estimation of common dispersion was conducted by the function “estimateCommonDisp” from a set of all genes that did not respond to the implemented treatment (~3900 genes). This strategy was applied to improve the variation estimate without biological replicate samples, according to the edgeR manual. We defined an FDR (false discovery rate)<0.01, the log2 ratio>2, and Fold Change>4, as a threshold to judge the significance of differences in gene expression.

**Table 1:** DEGs identified in a pairwise comparison among genotypes, plant tissues and treatments across the 12 RNA-Seq libraries.

Experimental comparison	Genotypes	Tissues	Treatments	N° of DEGs	Up-regulated DEGs	Down-regulated DEGs
1	Pérola x BAT 477	Root	All	21	1	20
2	Pérola x BAT 477	Leaf	All	138	64	74
3.1	BAT 477	Root	T_0_ x T_75_	192	177	15
3.2	BAT 477	Root	T_75_ x T_150_	3	0	3
3.3	BAT 477	Root	T_0_ x T_150_	681	567	114
4.1	Pérola	Root	T_0_ x T_75_	123	110	13
4.2	Pérola	Root	T_75_ x T_150_	31	13	18
4.3	Pérola	Root	T_0_ x T_150_	403	336	67
5.1	BAT 477	Leaf	T_0_ x T_75_	2	2	0
5.2	BAT 477	Leaf	T_75_ x T_150_	14	13	1
5.3	BAT 477	Leaf	T_0_ x T_150_	251	206	45
6.1	Pérola	Leaf	T_0_ x T_75_	0	0	0
6.2	Pérola	Leaf	T_75_ x T_150_	25	25	0
6.3	Pérola	Leaf	T_0_ x T_150_	208	149	59
7	Pérola x BAT 477	Root	T_0_	46	20	26
8	Pérola x BAT 477	Root	T_75_	17	7	10
9	Pérola x BAT 477	Root	T_150_	4	1	3
10	Pérola x BAT 477	Leaf	T_0_	76	41	35
11	Pérola x BAT 477	Leaf	T_75_	97	31	66
12	Pérola x BAT 477	Leaf	T_150_	57	25	32

### Gene annotation

Gene annotation was carried out for the drought-specific transcriptome assembly and DGEs. Initially, the major transcripts for each gene were compared with the NCBI’s non-redundant protein database (NR) through the BLASTx v.2.2.30+ algorithm (Camacho *et al.*, 2009) using the standard configurations. For loci without hits in BLASTx, the comparison was made through PSI-BLAST (Altschul *et al.*, 1997). The prediction of open reading frames (ORFs) on raw reads was conducted with TransDecoder v2.0.1 (https://transdecoder.github.io/). In addition, the loci with no hit with the Andean genome were annotated *against* the Mesoamerican transcriptome and LncRNAs (Vlasova *et al.*, 2016) (http://denovo.cnag.cat/genomes/bean/) using *BLASTn* v.2.2.30+. The BLAST2GO tool v4.1 (BioBam^®,^ Valencia, Spain; Free PRO Trial, Conesa and Götz, 2008) was used for GO categorization (E-value<10^-6^, annotation cutoff=55). The GO terms with significant changes in abundance were identified using Fisher’s exact test, with FDR correction (p≤0.05*)*. The identification of genes involved in specific metabolic pathways was performed using the KEGG database (Kanehisa *et al.*, 2016). Additionally, for sequences up to 2,000bp, the Rfam database (Nawrocki *et al.*, 2015) was used to search non-coding RNA.

### Variant call and characterization procedure

The Genome Analyze Toolkit (GATK) v.3.4-46 (McKenna *et al.*, 2010) performed the calls of SNPs and indels (using “HaplotypeCaller” algorithm), as well as analysis of base quality score recalibration (*BQSR*) and indel realignment (IR). The mapping step was performed using STAR v.2.4.1 (Dobin *et al.*, 2013), and duplicate reads were identified and removed using Picard tools v1.119 (http://broadinstitute.github.io/picard/). Quality control filters were used, such as a Fisher strand (FS)>30, a quality depth (QD)<2, and a minimum of 35bp of physical distance between two consecutive SNPs according to GATK recommendations (DePristo *et al.*, 2011; Van der Auwera *et al.*, 2013). The annotation and variant prediction of those high-quality SNPs were performed using the SnpEff v.4.2 (Cingolani *et al.*, 2012) and an “in-home” genome database built from the genome Phaseolus_vulgaris_218 v.1 (Schmutz *et al.*, 2014) combined with Pvulgaris_218_v1.0.gene_exons.gff3 (Phytozome v.11.0). Putative SNP effects categorized as high were annotated on GO terms and integrated in the KEGG pathway through BLAST2GO tool v4.1.

### Differentially expressed gene validation by qPCR

To perform validation using qPCR, 15 target DEGs were evaluated using the 12 cDNA samples taken to develop the libraries. The amplifications based on the TaqMan^®^ Gene Expression assay (Thermo Fisher Scientific) were performed using the Applied Biosystems^®^ 7500 Real-Time PCR (Thermo Fisher Scientific), followed by the determination of the C_q_ (quantification cycle) values using 7500 Software v.2.3 (Thermo Fisher Scientific). The C_q_ values were subjected to the 2^-^ΔΔ^*C*T^ method (Livak and Schmittgen, 2001), and the significance was determined by ANOVA and Tukey’s test (p<0.05) with R (https://cran.r-project.org/) using the packages agricolae v.1.2-4 and multcomp v.1.4-6. A set of three reference genes was used (Elongation factor, assay ID AI20SMX; 18S ribosomal RNA, assay ID AI39QS5 and PvT197, assay ID AIRR961), and the stability assessed with the geNorm Plus application (M values) integrated into the qbase^PLUS^/qPCR data analysis software package (Biogazelle, Zulte, Belgium; Hellemans *et al.*, 2007).

### Availability of data and material

The dataset(s) supporting the conclusions of this article are available in the SRA/NCBI database repository, accession number: SRP077562 (http://www.ncbi.nlm.nih.gov/sra/SRP077562).

## Results

### Evaluation of physiological conditions

The physiological parameters measured on plants grown hydroponically exposed to a short-term water deficit indicated that the dehydration treatments triggered changes in the plant physiological behaviour at both genotypes (Table S1). The photosynthetic rate (μmol CO_2_ m^-2^ s^-1^), stomatal conductance (mol H_2_O m^-2^s^-1^) and leaf transpiration rate (mmol H_2_O m^-2^s^-1^) decreased, while the leaf temperature (°C) increased, and the internal CO_2_ concentration (μmol CO_2_ mol^-1^) varied across the dehydration process. In general, the DT presented lower values of the photosynthetic rate (*p*<0.05 at T_0_ and T_50_) and stomatal conductance (*p*<0.05 at T_50_) than did the DS. For leaf transpiration, DT presented a reduced value at T_0_ (*p*<0.05). Similar values of leaf temperature and CO_2_ concentration were obtained for both genotypes.

### Providing a drought-specific transcriptome for *P. vulgaris*

All raw sequence data generated a total of ~580,000,000 reads, totalling 5.8 Giga base pairs (Gbp) of RNA-Seq data; ~329 and ~250 millions of reads were obtained, and 73.83% and 85.57% were retained (Q30) for further assembly in HiSeq and GAII, respectively. The raw data are summarized in Table S2 and deposited in the Sequence Read Archive (SRA)/NCBI database repository through accession number SRP077562 (http://www.ncbi.nlm.nih.gov/sra/SRP077562). The coverage of the bean genome per library was ~4x, while that of the total RNA-Seq was 94.96x, and that of the coding DNA sequence (CDS) was 476.39x. The total mapped reads per library was 87.52% (SD ± 3.72%). A *P. vulgaris* drought-specific transcriptome was assembled into 28,590 gene loci with 54,750 transcripts, of which 1,648 loci (5.76%) and 23,112 isoforms were newly described based on the Andean genome.

### Newly discovered genes and functional annotation

From the 1,648 loci newly predicted, 790 showed hits with previous sequences evaluated using both BLASTx and PSI-BLAST, 858 showed no homology with the NR. From the 790 loci with BLAST hit, 1,886 GO terms (GO IDs) were identified, and 466 GO terms were directly annotated (Table S3), of which 1,301 revealed a *significantly relative abundance* compared with the previously annotated *P. vulgaris* genes, and 1,300 terms were over-represented (Table S4) in biological process (69.02%), molecular function (18.52%) and cellular component (12.45%). Through KEGG analysis 70 metabolic pathways were identified containing 77 associated enzymes (Table S5).

From the 858 loci with no hit, 58 were categorized as non-coding RNA and were classified into seven biotypes: a) gene, snRNA, snoRNA, CD-box (56.8%); b) gene, miRNA (25.3%); c) gene, snRNA, snoRNA, HACA-box (11.6%); d) intron (3.2%); e) gene (1.1%); f) gene, antisense (1.1%); and g) gene, tRNA (1.1%). From the remaining 800 loci, 261 presented high similarity with transcripts encoding proteins and 136 were lncRNAs, both identified through homology to the Mesoamerican genome (Table S6), and 403 had no hit (23 potential ORF and 380 loci without information).

### Differentially expressed genes (DEGs)

For the whole set of genes (28,590), the comparative transcriptome revealed differences in gene expression patterns between DT and DS, even under control conditions (Figure S1). For the leaves and roots, the number of expressed genes (CPM >1) was similar, although the set of genes and tissue distribution varied considerably. In total, 21,696 genes were identified for the DT, with 17,425 (80.31%) common to both tissues (leaf and root), 2,423 (11.17%) exclusive in the roots, and 1,848 (8.52%) exclusive in the leaves. For DS, a set of 21,699 genes was identified under control conditions, of which 17,484 (80.58%) were expressed in both tissues, 2,550 (11.75%) were exclusive in the roots, and 1,665 (7.67%) were exclusive in the leaves. Between the contrasting genotypes, considering the leaf tissues, 816 genes were expressed only in DT, and 692 in DS; for the roots, 489 only in DT and 675 in DS.

The gene expression level, based on fold change (FC), is presented in Table S7. Compared with the control conditions (T_0_), T_75_ showed an increase in the overall number of expressed genes in the DS (326) and DT (42) genotypes. Interestingly, within genotypes, while drought sensitivity-related genes increased in both tissues (189), drought tolerance-related genes increased in the roots (332) but decreased in the leaves. Between genotypes, an increase in the total number of genes expressed specifically in the DS genotype (an additional of 232 for leaves and 198 for roots) was observed. By contrast, in T_150_, there was an increase in the number of expressed genes in the DT genotype (301), mainly in the roots (63 genes), compared with the DS genotype (30 genes), whose genes were preferentially expressed in the leaves. Overall, between the genotypes, a higher number of genes were expressed in the leaves for the DS (900) than for the DT (653) genotype; however, in the roots, an opposite trend was noted: more genes were observed for the DT genotype (615) than for the DS (536) genotype (Figure S1). In general, a greater number of KEGG pathways linked to DEGs were observed in the comparison of T_0_ *vs* T_150_ within genotypes for the up- and down-regulated genes in both tissues and genotypes (Table S5).

**Figure 1:**
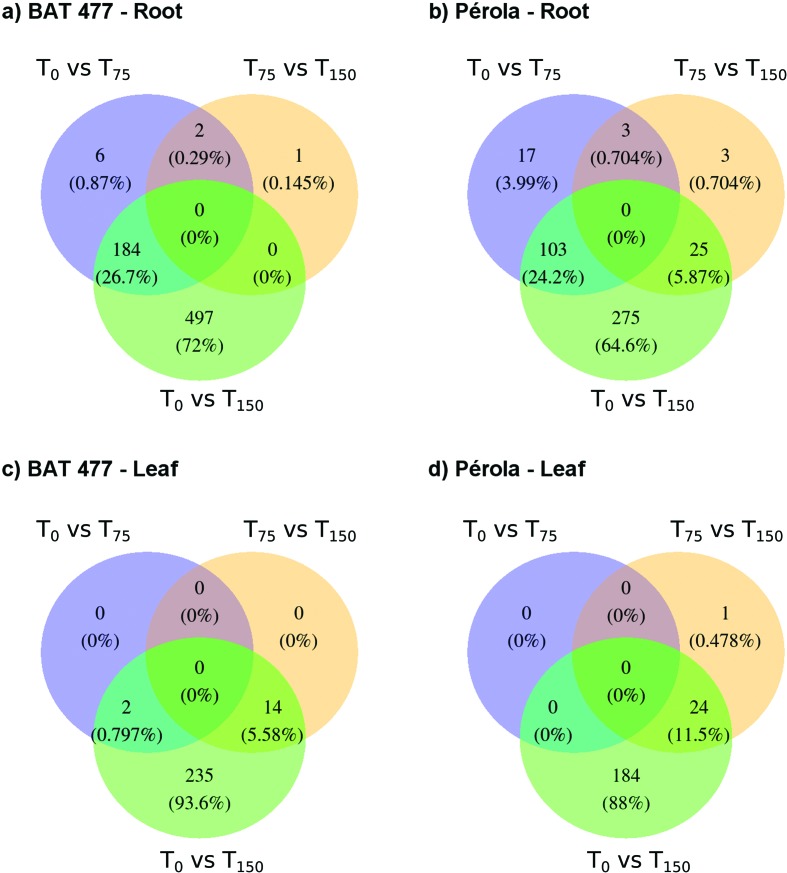
Differentially expressed genes between treatments for each genotype and tissue. (a) DEGs from BAT 477 in the root; (b) DEGs from Pérola in the root; (c) DEGs from BAT 477 in the leaf; (d) DEGs from Pérola in the leaf.

For DGE, in each pairwise comparison (Figure 1), the value of the dispersion utilized was 0.191, as estimated in edgeR. In the root tissue, in T_75_, 192 DEGs for the DT genotype and 123 for the DS were observed, of which 177 (91.71%) and 110 (89.43%) were up-regulated, respectively. In T_150_, root tissue, 681 DEGs were reported in the DT (569 up- and 112 down-regulated), of which 184 (27.02%) were also identified in T_75_. However, in the DS, 403 DEGs were identified (338 up- and 65 down-regulated), of which 103 (25.56%) were common to both treatments. For the leaf tissue in T_150_, a set of 251 DEGs were identified in the DT (82.07% up-regulated) and 208 in the DS genotype (71.64% up-regulated). For the leaf tissue, only a small portion of DEGs (5.58% for the DT genotype and 11.53% for the DS genotype) identified in T_150_ were differentially expressed in relation to T_75_. Among the DEGs in the roots, 73 in T_75_ were common to both genotypes (69 up-regulated and four down-regulated); however, in T_150_, this total was 245 (227 up- and 18 down-regulated). Of the 353 DGEs in T_150_ leaves, 106 were the same in both genotypes (92 up- and 14 down-regulated). Between the genotypes, 17 DEGs were identified in the roots in T_75_, and only four were identified in T_150_; however, in the leaves in T_75_ and T_150,_ 97 and 57 DEGs were identified, respectively.

From the DEGs, only one lncRNA was verified in the leaf tissue (DS genotype), while in the root tissue, six lncRNAs were identified that responded to drought stress with high reliability (fold change ≥ 4 and FDR < 0.01), four in the DT genotype, of which three were up-regulated, and two in the DS genotype.

### Functional annotation of differentially expressed genes (DEGs)

The GO categorization for all 1,239 DEGs was conducted with emphasis in the most representative distributions filtered by node score within GO categories (Table S3). Regarding functional annotation, in T_75_ roots, 27 enriched terms were associated with up-regulated drought tolerance-associated genes, while for the drought sensitivity-associated genes, only one term related to oxidoreductase activity was reported. No enriched terms were identified for down-regulated DEGs in the roots in T_75_ or in the leaves in T_75_ and T_150_. For the DT genotype in the long-term dehydration treatment (T_150_), 51 over-represented GO terms for up-regulated genes were identified in the roots. For the DS genotype, enriched GPO terms reported were similar to those previously found related to the T_75_ and T_150_ treatments in the DT (Table S4).

Through KEGG pathway, 97 metabolic processes were identified containing 145 associated enzymes (Table S5). In DT root tissues, 75 pathways for up-regulated genes and 47 for down-regulated genes were identified between treatments; however, in DS root tissues, these values were 49 and 24, respectively. In leaf tissue, 51 and 18 pathways for up- and down-regulated genes were reported for the DT genotype; however, for the DS genotype, these values were 55 and 14, respectively. In both comparisons for root tissue, the number of pathways exclusively represented in up-regulated DEGs in the DT genotype was 17 for T_0_ *vs* T_75_ and 28 for T_0_ *vs* T_150_; however, in the DS genotype, one and two KEGG pathways were identified in the same comparisons. In leaf tissues, four and eight pathways were exclusively identified for T_0_ vs T_150_ in the DT and DS genotypes, respectively, and another 12 were verified in DEGs from the T_75_ *vs* T_150_ comparison in the DS genotype.

In the KEGG analysis of leaf tissues, nine metabolic pathways were identified in the DT genotype. Of these, four were exclusive to this genotype (tropane, piperidine and pyridine alkaloid biosynthesis; lysine degradation; cysteine and methionine metabolism; sulphur metabolism), of which two were common to all three treatments (cysteine and methionine metabolism and sulphur metabolism). In the DS genotype, 15 pathways were identified, five of which were common to all three treatments and 10 of which were exclusive to this genotype. In the roots, 10 pathways were identified in the DS genotype and none in the DT genotype (Table S5).

### Variant call and characterization

**Figure 2:**
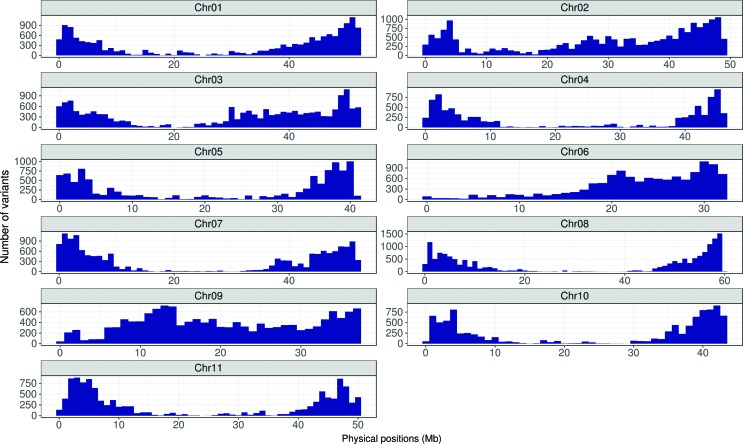
Distribution of variants (SNPs and indels) in P. vulgaris chromosomes.

The results evidenced a convergence of the numbers of SNPs and indels called, indicating that the increase in the number of steps in *BQSR* and IR analyses would not lead to quality improvement of the variants identified (Table S8). A total of 151,283 variants was identified, including 135,167 SNPs and 16,116 indels, of which 120,726 SNPs and 13,631 indels occurred in genes. The variants were placed on all *P. vulgaris* chromosomes, with one variant for every 3,413 bases (Figure 2). Furthermore, the identified variants affected 71.80% of the 27,197 loci described for the reference genome of common bean (Schmutz *et al.*, 2014). In total, 183,033 putative effects were identified and classified as modifier (49.68%) and low (29.21%), high (1.91%) and moderate (19.21%) impact types. Classification by functional class was reported for 85,780 variants and revealed 59.49% as silent, 40.04% as missense and 0.47% as nonsense. The largest proportion of the predicted effects occurred in exons (45.76%), and a significant number of effects (20.15%) was also observed in the 5’ and 3’ UTR regions, suggesting possible changes in the regulatory regions (Table 2). The Ts\Tv ratio was 1.34.

**Table 2:** Number of effects by type according to SnpEff classification.

Effect type	Count	Percentage (%)
3_prime_UTR_variant	26,765	14.48%
5_prime_UTR_premature_start_codon_gain_variant	1074	0.58%
5_prime_UTR_variant	9,041	4.89%
disruptive_inframe_deletion	317	0.17%
disruptive_inframe_insertion	223	0.12%
frameshift_variant	2,831	1.53%
inframe_deletion	166	0.09%
inframe_insertion	203	0.11%
initiator_condon_variant	8	0.01%
intergenic_region	17,465	9.46%
intron_variant	38,910	21.04%
missense_variant	34,257	18.53%
non_conding_transcript_variant	10	0.01%
splice_acceptor_variant	10	0.01%
splice_donor_variant	83	0.05%
splice_region_variant	1,815	0.98%
start_lost	66	0.04%
stop_gained	432	0.23%
stop_lost	83	0.05%
stop_retained_variant	82	0.04%
synonymous_variant	50,951	27.56%

### Validation of DEGs by qPCR

**Figure 3:**
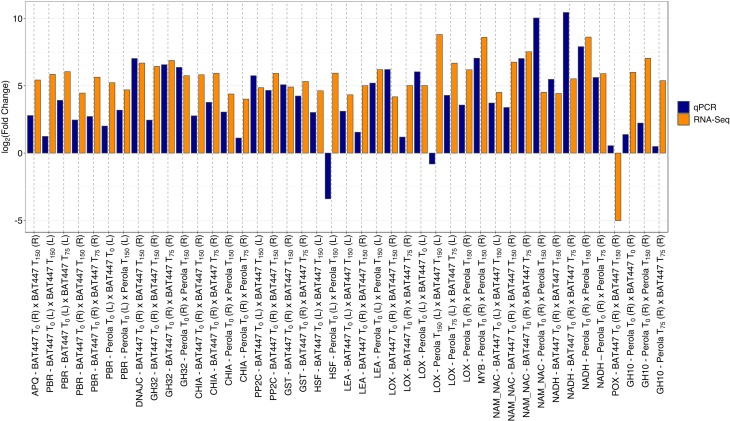
Comparisons between the gene expression profiles obtained for DEGs by RNA-Seq and qPCR analyses. Among the 15 genes chosen to be validate by qPCR, those genes differentially expressed in RNA-Seq had their values of cycle quantification (Cq) expressed as a fold-change and then compared between the analyses.

The reference genes used present high stability of expression among all samples, with the combined value below the cutoff of 1.5 (Robledo *et al.*, 2014). A set of 15 DEGs was validated through qPCR in a biological replicated experiment (Table S9). The gene expression profile obtained by qPCR was 88.64%, concordant with the results obtained in RNA-Seq analysis (Figure 3 and Figure S2). Of the 15 DEGs identified by RNA-Seq, most (60%; POX, LEA, OPDA, CWI, MYB, GST, HSTF, FHOS2C) correlated well with the qPCR results, presenting high correspondence (100%) and a strong coefficient of correlation (≥60%), which are important technical validation parameters in RNA-Seq. Among the genes with high correspondence between RNA-Seq and qPCR methodologies, those that were up-regulated in the DT genotype under greater drought stress were glutathione S-transferase (GST; Phvul.008G113700) and peroxidase (POX; Phvul.009G140700). GST presented increased expression in the leaf tissue of the DT genotype in T_0_, T_75_ and T_150_, compared to DS. The POX enzyme was up-regulated in T_0_ and T_75_ in the DT genotype, followed by weak up-regulation in subsequent T_150_ treatments in the leaves, while POX in the DS genotype showed the opposite pattern of expression, with increased up-regulation in T_150_. In the roots, POX expression gradually increased with treatment time, with significant levels of expression in T_150_ in the DT genotype.

## Discussion

Episodes of drought with a temporary decrease in water availability lead to stress and induce changes in several morphological, physiological, biochemical and molecular processes in plants (Mehrotra *et al.*, 2014). Hydroponics is a useful system for studying plant responses to abiotic stresses, including drought, in which the water deprivation occurs abruptly by removing the plant from the nutrient solution (Neves-Borges *et al.*, 2012; Tripathi *et al.*, 2016). As an advantage, hydroponic systems overcome the effects of several abiotic stresses other than water stress, such as the problems of heterogeneity and drainage (Munns *et al.*, 2010), allowing the maintenance of constant conditions such as temperature and relative humidity (Martins *et al.*, 2008), besides the ease of root collecting. A more uniform water stress can facilitate the discovery of more related genes and, consequently, the recognition of mechanisms more directly involved in the response to the environmental change. Although hydroponic and soil-grown plants induce different physiological/molecular responses, studies have shown that these responses should be the same but expressed differently over time (Munns *et al.*, 2010; Tripathi *et al.*, 2016). In the present study, despite hydroponic stress failed to predict significant differences in the osmotic potential, probably due to the short periods of dehydration, the mechanisms related to gas exchange (photosynthetic rate, transpiration rate, and stomatal conductance) were reduced to values close to zero, which may be a consequence of the decline in photosynthesis due to dehydration. Therefore, hydroponics is a reliable and valid strategy to support the progress in understanding water stress responses.

### Functional annotation of newly discovered transcripts

Approximately 3.87% of the transcripts represented newly discovered genes (5.76% of all genes) within the Andean and Mesoamerican gene pools (Schmutz *et al.*, 2014; Vlasova *et al.*, 2016). However, all the new genes described are involved directly or indirectly in the mechanisms of plants affected by water stress; the most interesting was that several reported enriched GO terms were related to abiotic stimulus and the defence response. Exploring the potential of candidate target genes that confer tolerance to drought for the implementation of breeding strategies is of great interest. Among the discovered genes, a considerable number (34 of 466 new genes with GO terms) were annotated to protein kinase (PK) GO terms (Table S3). These enzymes play a fundamental role in various regulatory mechanisms of the cell through signal transduction pathways during biotic and abiotic stress (Ho, 2015). In addition, different types of PKs not previously described for common beans were identified, including various receptor-like kinase genes (20 new for beans) that could be strongly associated with the mechanisms of the response of external stimuli such as water deficit, activating signalling networks to adapt the plant to the changing environment, as described previously (Osakabe *et al.*, 2013).

In the present study, three novel genes involved in response to abscisic acid (ABA) (XLOC_002778, XLOC_009245, XLOC_017074) and one involved in ABA biosynthesis (XLOC_011282) were identified. ABA is a well-characterized phytohormone that triggers several pathways for plant stress responses, mainly by the activation of a transcriptional regulatory network involved in drought (Tuteja, 2007). TFs were also newly reported for common beans, such as MYB (XLOC_009355) and WRKY (XLOC_002066), which have been proposed to regulate several processes, such as hormonal induction during stress and responses to diverse biotic or abiotic environments (Rushton *et al.*, 2010; Ambawat *et al.*, 2013). A less-specific TF was also identified, including GTE1 (XLOC_005373, XLOC_011757), which functions as a general TF (Duque and Chua, 2003), and an ethylene-responsive TF (XLOC_013281) differentially regulated by ethylene and various forms of abiotic stress (Fujimoto *et al.*, 2000). Genes related to jasmonic (JA), ethylene (ET) and salicylic (AS) acids were also newly described and related to the GO terms of biosynthetic process and mediated signalling pathway (XLOC_013281; XLOC_002778; XLOC_024793; XLOC_026696; XLOC_006232; XLOC_006232; XLOC_028474; XLOC_028574; XLOC_011121), in addition to auxins (XLOC_018037) and cytokinins (XLOC_001761, XLOC_019264). The role of these hormones in abiotic stress tolerance (Santino *et al.*, 2013; Miura and Tada, 2014; Kazan, 2015) and plant defence, as well as plant development and growth through efficient signalling networks and elaborate crosstalk, has been broadly reviewed (Verma *et al.*, 2016).

These findings also revealed a new class of chaperones (four new *loci*). Chaperones play an important role in protein homeostasis and, although constitutively expressed, are extremely active in the signalling of stresses such as drought, temperature extremes and salinity (Park and Seo, 2015). There are several diverse groups of chaperones sharing the property of binding to unstable substrate protein (Gupta and Tuteja, 2011; Trivedi *et al.*, 2016). The co-chaperones identified in the present study, known as DnaJ/HSP40 proteins (XLOC_010633, XLOC_028205) and p23 proteins (XLOC_010687), were described as key regulatory factors that modulate the activity of HSP70 and HSP90 chaperones, respectively (Felts and Toft, 2003; Kampinga and Craig, 2010; Qi *et al.*, 2013). Finally, several genes related to plant disease resistance in several ways were newly identified for common bean (more than 30 genes), contributing to the enrichment of the biotic stress-related gene database. This would be expected because, during drought stress, the plant becomes more predisposed to diseases/infections and susceptible to insect and pest infestations (Rosenzweig *et al.,* 2001).

### Long non-coding RNAs (lncRNAs)

Long non-coding RNAs (lncRNAs) have been identified as regulators of many fundamental biological processes, such as tissue development, as well as the response to osmotic, saline and drought stresses (Zhang *et al.*, 2014; Liu X *et al.*, 2015; Muthusamy *et al.*, 2015; Quan *et al.*, 2015; Wang *et al.*, 2015; Lu *et al.*, 2016). Many stress-responsive lncRNA transcripts have been predicted and identified in some species, such as *Medicago truncatula* (Wang *et al.*, 2015), *P. vulgaris* (Vlasova *et al.*, 2016) and wheat (Zhang *et al.*, 2016). To date, only a few plant lncRNAs with potential roles in response to drought stress have been characterized, such as *Arabidopsis* (Ben Amor *et al.,* 2009), foxtail millet (Qi *et al.*, 2013) and maize (Zhang *et al.*, 2014). In this study, lncRNAs were identified as DEGs responsive to drought stress with high reliability (fold change≥4 and FDR<0.01), of which three were specifically detected and up-regulated in the DT root tissues. The recently demonstrated function of lncRNA in cell differentiation and development (Fatica and Bozzoni, 2014) suggests that lncRNA expression could play a critical role in the molecular mechanisms underlying root development under drought stress in BAT 477, a deep-rooting genotype (Asfaw and Blair, 2012). Our results suggest that these lncRNAs could be potential targets to explore the functional role of lncRNAs in the tolerance mechanisms of bean. More recently, a genome-scale screening platform based on CRISPR-mediated interference (CRISPRi) has been used for the identification of lncRNA function in human cells (Liu *et al.*, 2017), providing a new perspective to functionally test other organisms.

### DEGs

In T_0_, a specific gene expression profile by genotype was observed, as well as considerable variation between the root and leaf tissue modulated by the activation of different genes along the dehydration periods (T_75_ - T_150_). This differential expression pattern over time demonstrated the effectiveness of the implemented stress-induced protocol. In the root tissue there was an increased number of DEGs up-regulated in the DT genotype (T_75_=108 genes; T_150_=342) compared with that in the DS genotype (T_75_=41; T_150_=111), with a predominance of enriched categories related to osmotic and redox processes, signal transduction mechanisms, TFs, and the development of cellular metabolism. Regarding physiological aspects, in dry conditions, the DT genotype develops root tissue for access to deeper layers of the soil, as an important adaptive advantage in the mechanism of bean tolerance (Lanna *et al.*, 2016), although this potential is dependent on favourable environmental conditions such as soil type (Sponchiado *et al.*, 1989). BAT 477 (DT) presents differentiated root growth, suggesting a strong effect of genetic factors controlling these traits. Asfaw and Blair (2012) identified 15 regions containing QTLs related to root development under dry conditions, and the most favourable alleles were derived from the parental BAT 477. A detailed annotation of these QTL regions, integrating information on the genes identified in this study, together with the high LD in the bean genome (Valdisser *et al.*, 2016) may be used for the selection of the traits related to root development and will be effective in breeding programmes for drought tolerance.

Plants can adapt to environments with large variations in climate conditions, which has driven the evolution of a highly flexible metabolism (Shao *et al.*, 2007). This stress leads to the formation of ROS (reactive oxygen species) such as superoxide, hydrogen peroxide (H_2_O_2_), hydroxyl radical, and singlet oxygen (Noctor and Foyer, 2016), which are regulated by a cellular antioxidant defence system consisting of enzymatic and non-enzymatic antioxidants. In this study, it was clearly observed in the DT genotype that gene expression involved in redox and related processes increased in roots in the comparison of T_0_ *vs* T_75_, immediately following the removal of the plants from the nutrient solution. Approximately 15% of enriched terms, such as oxidation-reduction, oxidoreductase activity, electron carrier activity, and iron ion binding, were directly related to these genes. This evidence suggests that oxidative stress may have been the primary factor triggering different signalling events in response to dehydration. Non-enzymatic antioxidants, such as ascorbate, GSH, proline and betaine, were also identified in the present study as DEGs.

Although the redox process was strongly activated in the roots and leaves, the associated DEGs were completely different. While in the roots, the main DEGs were peroxidases, lipoxygenases (linoleate 13s-lipoxygenase-3-chloroplastic-like), dioxygenases (gibberellin and leucoanthocyanidins), oxidases (1-aminocyclopropane-1-carboxylate), NADP-dependent malic enzymes and cytochrome P450 (CYP) proteins; in the leaves the main DEGs were cationic peroxidase, oxidases (polyphenol oxidase and long-chain alcohol oxidase), lipoxygenases (seed linoleate 9s-lipoxygenase), lysine-ketoglutarate reductase saccharopine dehydrogenase, zinc-binding alcohol dehydrogenase family protein and protein hothead-like. These DEGs identified between tissues indicated that the DT genotype triggers distinct mechanisms of drought tolerance, inducing expressive antioxidant responses.

The dehydration stress in the DT genotype drove changes in the expression of genes related to developmental and cellular growth processes associated with cell division, differentiation, and structure. Additionally, many terms related to kinases play a central role in the regulation of cell function (Krebs, 1985) and adaptive responses (Lehti-Shiu and Shiu, 2012). The overexpression of calcium-dependent protein kinase (CDPK) enhances crop stress resistance/tolerance to cold, salt and drought, such as that for rice (Wei *et al.*, 2014) and pepper (Cai *et al.*, 2015), and has been involved in specific tissue growth and development (Ivashuta *et al.*, 2005). In the root tissue of the DT genotype in T_75_, terms related to metabolic processes of polysaccharides, aminoglycans, chitin, and glucosamine were still highlighted, which are related to plant adaptation process under stress, such as restructuring of the cell wall at lower water content and secondary wall formation (Moore *et al.*, 2008; Guo *et al.*, 2013). In addition, GO terms related to chitin were linked to the same set of DEGs (four genes) in the root tissue under dehydration (T_75_ and T_150_) in the DT and DS genotypes. The enzymatic hydrolysis of chitin involves an initial cleavage by chitinases into chitin oligosaccharides and further cleavage to N-acetylglucosamine (GlcNAc) and monosaccharides by chitobiases (reviewed by Hamid *et al.*, 2013). In the present study, the enzyme chitinase (Phvul.005G155800) displayed increased expression after drought stress in both genotypes and tissues, highlighting the DT genotype in T_75_ for leaf tissue and T_150_ for root tissue. In plants, cell wall glycoproteins containing GlcNAc appear to be an endogenous substrate for plant chitinases (Dyachok *et al.*, 2002). Their important role in plant defence against biotic stress is well known; moreover, it has been demonstrated that they are expressed under normal conditions and act in response to abiotic agents (ethylene, jasmonate) and conditions (cold, drought) (Passarinho *et al.*, 2001; Ahmed *et al.*, 2012).

We also observed that most genes related to oxidation-reduction terms were up-regulated in DT roots in early stress (T_75_), but in late stress in DS roots (T_150_), suggesting that the susceptibility also seemed to be related to the late response to stress. On the other hand, the tolerance could be attributed to the ability to activate genes promoting an immediate response at the beginning of stress, increasing the chances of adaptation and minimizing and/or fighting the effects of the lack of water. These findings are supported by previous results on the physiological characterization of BAT 477 and Pérola genotypes evaluated under water deficit (Lanna *et al.*, 2016), in which the DS genotype triggered late perception and signalling pathways induced by water deficit compared with the DT genotype. In addition, the terms associated with the antioxidant system activated early in DT can be an important mechanism of tolerance in beans, inhibiting and/or reducing the damage caused by the deleterious effects of ROS to cells and tissues (Gill and Tuteja, 2010). Among the stress-inducible genes, those involved in direct protection from stress, including the synthesis of regulatory proteins such as TFs, PKs, and phosphatase, were activated early in the DT genotype. In the DS plants (commercial cultivar Pérola), several genes related to the response to biotic stimulus in roots were identified, which was expected because disease resistance is one of the main pillars of genomic breeding of common bean lines (Souza *et al.*, 2013).

### KEGG pathways of DEGs

Our data showed that, among the KEGG pathways enriched by DEGs up-regulated in the DT genotype, two were common to all treatments in leaves. These pathways are related to the regulation of sulphur-containing amino acid metabolism (cysteine and methionine) and sulphur metabolism, both of which are essential for plant growth (Hawkesford and De Kok, 2006). The findings in the present study suggested that these pathways are important in improving stress tolerance in the common bean due to the formation of many sulphur-containing defence compounds involved in plant defence signalling (Capaldi *et al.*, 2015). These processes of sulphur metabolism consist of the uptake of inorganic sulphur (sulphate) by roots, followed by sulphate reduction and assimilation in leaves, leading to the synthesis of sulphur-containing amino-acids (reviewed by Wirtz and Droux, 2005). Increased demand for reduced sulphur is seen involved in regulating essential processes in the plant, including protection against stress through the induction of S-containing compounds with cysteine as precursors for essential biomolecules (Romero *et al.*, 2014). Of interest is the antioxidant GSH, which has been a central molecule determinant of cellular redox homeostasis (Noctor *et al.*, 2012). Moreover, the biosynthesis of methionine from cysteine resulted in the hormone ethylene, polyamines, and nicotine amine, all of which are involved to varying degrees in the modulation of the plant response to stresses (Burstenbinder and Sauter, 2012; Liu JH *et al.*, 2015; Lee *et al.*, 2017).

### qPCR validation

Our results validated a high proportion of DEGs (88.64%) of the RNA-seq experiments using qPCR, with an overall moderate correlation (61%) between both methods. As the experiment was carried out in biological replicates with the same genotypes and design as used for the RNA-Seq study, this similarity was expected. Although qPCR-based methods have been considered the gold standard for measuring gene expression, these methods are negatively affected by several factors (Everaert *et al.*, 2017). In addition, differential isoform expression, which is not considered in primer design, is challenging and may interfere significantly compared with sampling different genes (Teixeira *et al.*, 2006). Of the 15 DEGs identified by RNA-Seq, 60% correlated well with the qPCR results (100% of correspondence), an important technical validation of the RNA-Seq experiments. Among those eight validated genes, the antioxidant enzymes GST and POX, which are involved in maintaining cellular redox balance (Asada, 1992; Galant *et al.*, 2011), have been up-regulated in the DT genotype in several treatments of leaves and roots. The enzyme POX, up-regulated in the leaves (T_0_, T_75_) and roots (T_150_) of the DT genotype when compared to DS, is closely associated with the response to abiotic stresses, such as drought tolerance (Rossel *et al.*, 2006). Increased expression of POX was reported in the root tissue of soybean, promoting drought tolerance (Mohammadi *et al.,* 2012), as well as in other plants (Sheoran *et al.*, 2015; Katam *et al.,* 2016). Our study also showed an up-regulation of GST in the DT genotype under all conditions in the leaf tissue. Xu *et al.* (2015) reported an overexpression of GST from tomato (LeGSTU2) in Arabidopsis, resulting in increased activity of enzymes related to antioxidant responses and improved tolerance to salinity and drought stresses. These findings demonstrate that these enzymes have a favourable impact on the drought tolerance mechanism of the common bean and could be used as targets in the search and development of more DT genotypes.

The results of RNA-Seq and qPCR for the enzyme involved in cell wall degradation, GH10 xylanase, were not perfectly correlated but were in the same direction and showed a similar trend, being up-regulated in the roots of the DT genotype in T_0_ and T_75_. In addition, according to qPCR results, this enzyme was up-regulated in DT leaves and roots in all treatments. This enzyme presents predominantly an endo-beta-1,4-xylanase activity (GO:0005975) and hydrolase activity (GO:0004553), was previously described in soybean as LOC100801147, and acts in the breakdown of hemicellulose, suggesting the disassembly of cell wall components during drought stress. The high levels of expression in the roots of the DT genotype in all treatments probably suggest that cell expansion necessary to maintain root growth under drought to absorb water in deeper soil layers and thereby maintain the water status of the plant. In leaves, although gene expression in the DT genotype was greater than that in the DS genotype, down-regulation was observed (greater than 50%), and the expression level was three times smaller than that in the root. Loss of leaf area is the most important morphological adaptation to drought in common bean (Acosta-Gallegos, 1988). Under drought stress, while the root is strategically stimulated to grow to explore the water in the soil, the leaves become reduced in cell turgor due to osmotic stress, and the mechanical power of the cell is also reduced to expand the polysaccharide network (Tenhaken, 2015).

### Genomic regions with variants of high impact

Through genome re-sequencing, numerous SNPs have been identified (Song *et al.*, 2015), providing more subsidies for genome-wide association analysis, identification of genomic regions of agronomic interest, genomic selection and studies of genetic diversity. In this study, 3,067 transcripts belonging to 2,501 genes were under high effect impact (~2%). Although this number of genes is quite low, functional annotation revealed that the affected genes were mostly involved in cellular response to stimulus, response to stress, oxidation-reduction stress, and regulation of gene expression. These genes are targets for subsequent functional studies because they can be related to the divergence resulting from the adaptive process to which the genotypes were imposed during their development process. BAT 477 is a well-known source of drought tolerance developed by CIAT (White and Castillo, 1989), whose adaptive advantage is attributed to the root systems (Sponchiado *et al.*, 1989), while the DS plant (Pérola) was developed for growing under adequate cultivation conditions. An SNP set carrying variants filtered for high impact was identified in the present study, allowing follow up of the genetic variations and their contribution to phenotypic diversity in a large and representative sample of accessions. In a recent study (Schrimpf *et al.*, 2016), variants of high impact identified in genes involved in horse fertility were assigned to putative deleterious effects and were recommended for selection against these effects, demonstrating how genetic information can be used to benefit the breeding programmes.

## Conclusions

In this study, we constructed a bean transcriptome under drought conditions and novel transcripts and novel isoforms were identified in the Andean common bean genome, contribution to improve the currently available database to the community of beans. As more genes become available, the chance to perform more refined mining will increase, which would obtain better knowledge of the control of specific routes related to the response to drought stress, which could be used for breeding drought-resistant plants. In addition, a broad genome SNP coverage improves the ability to predict target genic regions and/or genomic candidate variants, allowing more refined studies. The results of this study contribute to knowledge of the gene mechanisms, as well as their functional variants, related to two contrasting common DT bean genotypes.

## Supplementary material

The following online material is available for this article:

Figure S1 - Comparison of the number of genes expressed in leaf and root tissues, as well as between genotypes.

Figure S2 - Expression profile for the 15 genes selected for qPCR validation.

Table S1 - Physiological parameters measured on plants grown hydroponically and exposed to short-term water deficit.

Table S2 - Information of raw sequence derived from each library sequenced on the Illumina HiSeq and Illumina Genome Analyzer II.

Table S3 - Functional annotation with Blast2GO predicted for the new loci and the DEGs. This table is available at the link: https://doi.org/10.6084/m9.figshare.7471697.v1.

Table S4 - Statistical assessment of GO term enrichment.

Table S5 - KEGG metabolic pathways identified for the new loci and DEGs.

Table S6 - Description of the 397 loci with no hit in Blastx or PSI-Blast).

Table S7 - Expression values of the differential expressed genes.

Table S8 - Number of variants identified after each Base Quality Score Recalibration (BQSR) step.

Table S9 - Descriptions of TaqMan probes.
