# Supplementary material for: Genome-wide analysis of the transcriptional response to drought stress in root and leaf of common bean
Source: Genet Mol Biol. 2020 Mar 16;43(1):e20180259. doi: 10.1590/1678-4685-GMB-2018-0259 (PMC7307723; doi:10.1590/1678-4685-GMB-2018-0259)
Supplement: Supplementary file 1 [file 1415-4757-GMB-43-1-e20180259-s3.pdf]

# Supplementary Material to: "Genome-wide analysis of the transcriptional response to drought stress in root and leaf of common bean"

**Table S1** Physiological parameters measured on plants grown hydroponically and exposed to short-term water deficit. Statistical analysis presents the mean, standard deviation (SD) and coefficient of variation (CV) as a percentage of physiological analyses performed between genotypes at seven distinct times of treatments.

| Photosynthetic carbon assimilation (μmol $CO_2$ $m^{-2}s^{-1}$) | | | | | | | | | | | | | | |
|---|---|---|---|---|---|---|---|---|---|---|---|---|---|---|
| | **T0** | | **T25** | | **T50** | | **T75** | | **T100** | | **T125** | | **T150** | |
| **Genotype** | **MEAN** | **SD** | **MEAN** | **SD** | **MEAN** | **SD** | **MEAN** | **SD** | **MEAN** | **SD** | **MEAN** | **SD** | **MEAN** | **SD** |
| **BAT 477** | 10,62 aB* | 0.93 | 15,30 aA | 2.87 | 8,40 aB | 1.16 | 7,19 aB | 2.91 | 6,68 aB | 2.03 | 2,19 aC | 2.19 | 0,23 aC | 0.41 |
| **PÉROLA** | 15,75 bA | 2.05 | 14,08 aA | 1.71 | 11,88 aAB | 3.98 | 7,62 aBC | 4.53 | 4,90 aCD | 1.68 | 1,45 aD | 1.02 | 1,25 bD | 0.65 |
| **Stomatal conductance (mol $H_2O$ $m^{-2}s^{-1}$)** | | | | | | | | | | | | | | |
| | **T0** | | **T25** | | **T50** | | **T75** | | **T100** | | **T125** | | **T150** | |
| **Genotype** | **MEAN** | **SD** | **MEAN** | **SD** | **MEAN** | **SD** | **MEAN** | **SD** | **MEAN** | **SD** | **MEAN** | **SD** | **MEAN** | **SD** |
| **BAT 477** | 0,21 aB | 0.04 | 0,39 aA | 0.1 | 0,16 aBC | 0.03 | 0,14 aBCD | 0.13 | 0,09 aBCD | 0.04 | 0,04 aCD | 0.02 | 0,02 aD | 0.01 |
| **PÉROLA** | 0,38 aA | 0.14 | 0,37 aB | 0.1 | 0,28 bC | 0.05 | 0,10 aD | 0.04 | 0,06 aE | 0.02 | 0,07 aF | 0.08 | 0,02 aG | 0.01 |
| **Internal $CO_2$ concentration (μmol $CO_2$ $mol^{-1}$)** | | | | | | | | | | | | | | |
| | **T0** | | **T25** | | **T50** | | **T75** | | **T100** | | **T125** | | **T150** | |
| **Genotype** | **MEAN** | **SD** | **MEAN** | **SD** | **MEAN** | **SD** | **MEAN** | **SD** | **MEAN** | **SD** | **MEAN** | **SD** | **MEAN** | **SD** |
| **BAT 477** | 261,50 aAB | 11.12 | 292,50 aAB | 7.42 | 276,75 aAB | 6.40 | 246,25 aB | 62.10 | 229,00 aB | 71.99 | 280,25 aAB | 57.32 | 349,00 aA | 27.39 |
| **PÉROLA** | 269,50 aAB | 16.42 | 291,25 aA | 14.25 | 288,50 aAB | 23.73 | 242,25 aAB | 25.25 | 217,50 aB | 33.91 | 287,75 aAB | 65.83 | 236,25 bAB | 35.51 |
| **Leaf transpiration rate (mmol H2O $m^{-2}$ $s^{-1}$)** | | | | | | | | | | | | | | |
| | **T0** | | **T25** | | **T50** | | **T75** | | **T100** | | **T125** | | **T150** | |
| **Genotype** | **MEAN** | **SD** | **MEAN** | **SD** | **MEAN** | **SD** | **MEAN** | **SD** | **MEAN** | **SD** | **MEAN** | **SD** | **MEAN** | **SD** |
| **BAT 477** | 3,84 aB | 0.57 | 7,12 aA | 1.02 | 4,15 aB | 0.5 | 3,35 aBC | 2.43 | 2,94 aBCD | 1.03 | 1,44 aCD | 0.57 | 0,76 aD | 0.53 |
| **PÉROLA** | 5,68 bA | 1.31 | 6,93 aA | 1 | 6,04 bA | 0.78 | 2,82 aB | 1.13 | 1,87 aB | 0.48 | 2,01 aB | 2.1 | 0,70 aB | 0.23 |
| **Leaf temperature (°C)** | | | | | | | | | | | | | | |
| | **T0** | | **T25** | | **T50** | | **T75** | | **T100** | | **T125** | | **T150** | |
| **Genotype** | **MEAN** | **SD** | **MEAN** | **SD** | **MEAN** | **SD** | **MEAN** | **SD** | **MEAN** | **SD** | **MEAN** | **SD** | **MEAN** | **SD** |
| **BAT 477** | 27,02 aE | 0.26 | 28,15 aE | 0.48 | 30,10 aD | 0.43 | 30,83 aCD | 1.19 | 31,84 aC | 0.24 | 34,19 aB | 0.45 | 35,44 aA | 0.35 |
| **PÉROLA** | 26,29 bE | 0.47 | 28,28 aD | 0.46 | 29,40 aD | 0.43 | 31,25 aC | 0.43 | 32,50 bBC | 0.37 | 33,46 aB | 1.78 | 35,66 aA | 0.19 |

*The different lowercase between genotypes under the same treatment indicate statistically significant differences, and the different capital letters among treatments for the same genotype indicate statistically significant differences ($p \leq 0.05$).
