# Supplementary material for: Genome-wide analysis of the transcriptional response to drought stress in root and leaf of common bean
Source: Genet Mol Biol. 2020 Mar 16;43(1):e20180259. doi: 10.1590/1678-4685-GMB-2018-0259 (PMC7307723; doi:10.1590/1678-4685-GMB-2018-0259)
Supplement: Supplementary file 2 [file 1415-4757-GMB-43-1-e20180259-s4.pdf]

Genetics and Molecular Biology 43, 1, (2020)

Supplementary Material to: “Genome-wide analysis of the transcriptional response to drought stress in root and leaf of common bean”

Table S2 Information of raw sequence derived from each library sequenced on the Illumina HiSeq and Illumina Genome Analyzer II platforms including the total number of reads, and the paired-end (2x150bp) and single-end total resulting reads after the quality control (QC) data.

Library	Genotype	Tissue	Physiol. treatment	Nº of raw paired-end reads	Total of reads	Paired-end reads remaining	% of paired-end reads remaining	Single-end reads remaining	Total of reads remaining	% of reads remaining (Total)	Descarded reads	% of descarded reads
**Illumina Genome Analyzer II**												
Lib01	BAT 477	root	0 min	14347428	11621328	11186032	80.71%	2666004	10330824	87.51%	3697682	3.73%
Lib02	BAT 477	root	75 min	4168237	17475314	3318208	79.96%	721361	15473837	87.26%	3656788	4.50%
Lib03	BAT 477	root	150 min	17394311	20910150	13754688	78.49%	3039320	18309586	88.63%	2773852	17.92%
Lib04	BAT 477	leaf	0 min	17505097	8336474	13709575	79.61%	3218579	7357777	88.55%	2001477	20.93%
Lib05	BAT 477	leaf	75 min	12202546	35010194	9781481	78.32%	2068278	30637729	88.26%	978697	24.01%
Lib06	BAT 477	leaf	150 min	8336865	16673730	2357296	28.28%	5033085	9747677	58.46%	6926053	41.54%
Lib07	Pérola	root	0 min	6499354	12998708	5110795	78.64%	1163963	11385553	87.59%	1613155	12.41%
Lib08	Pérola	root	75 min	5810664	29610270	4689510	78.52%	951804	25912588	88.82%	1103664	42.94%
Lib09	Pérola	root	150 min	14805135	9874980	11625580	80.40%	2661428	8771316	87.81%	4239926	7.48%
Lib10	Pérola	leaf	0 min	8737657	28694856	6986533	77.97%	1500771	25038068	88.90%	1290504	31.82%
Lib11	Pérola	leaf	75 min	4937490	34788622	3969802	79.08%	831712	30548696	87.56%	2600564	13.27%
Lib12	Pérola	leaf	150 min	10455075	24405092	8205859	80.16%	1897868	21631240	87.51%	4372465	2.80%
**Total:**				**125199859**	**250399718**	**94695359**	**75.64%**	**25754173**	**215144891**	**85.92%**	**35254827**	**14.08%**
**Illumina HiSeq**												
Lib01	BAT 477	root	0 min	12122950	26664506	7924229	63.56%	2084395	19404767	74.01%	6034821	40.90%
Lib02	BAT 477	root	75 min	14135389	24817868	9179107	60.24%	2516610	17441307	73.96%	6313047	29.94%
Lib03	BAT 477	root	150 min	12891083	28393246	8665937	67.51%	2192479	21577525	72.47%	5937525	36.34%
Lib04	BAT 477	leaf	0 min	14398137	28270778	9189312	64.94%	2580275	20874824	70.28%	7376561	25.44%
Lib05	BAT 477	leaf	75 min	10782705	28796274	6804553	63.82%	2018779	20958899	73.84%	7395954	26.09%
Lib06	BAT 477	leaf	150 min	12263336	24526672	8271060	67.45%	2062933	18605053	75.86%	5921619	24.14%
Lib07	Pérola	root	0 min	17787446	35574892	11612045	65.28%	3108273	26332363	74.02%	9242529	25.98%
Lib08	Pérola	root	75 min	13332253	23220140	8474461	64.88%	2455845	17185319	74.29%	9497030	16.94%
Lib09	Pérola	root	150 min	11610070	36933750	7532519	65.47%	2120281	27436720	75.73%	6257813	26.44%
Lib10	Pérola	leaf	0 min	12408934	24245900	7474747	65.37%	2491813	17932853	72.77%	7259739	22.63%
Lib11	Pérola	leaf	75 min	18466875	25782166	12090958	67.22%	3254804	19524353	76.00%	6815721	20.91%
Lib12	Pérola	leaf	150 min	14196623	21565410	9583566	63.11%	2410393	15627885	72.78%	7837375	25.68%
**Total:**				**164395801**	**328791602**	**106802494**	**64.97%**	**29296880**	**242901868**	**73.88%**	**85889734**	**26.12%**
