# Supplementary material for: Genome-wide analysis of the transcriptional response to drought stress in root and leaf of common bean
Source: Genet Mol Biol. 2020 Mar 16;43(1):e20180259. doi: 10.1590/1678-4685-GMB-2018-0259 (PMC7307723; doi:10.1590/1678-4685-GMB-2018-0259)
Supplement: Supplementary file 3 [file 1415-4757-GMB-43-1-e20180259-s5.pdf]

Table S3 Functional annotation with Blast2GO predicted for the new loci, DEGs and DEGs under high impact predicted effects (HIPE).

Cufflinks ID	Major Transcript	Sequence Description	GO Terms	Enzymes	Kegg Maps
**NEW LOCI**					
XLOC_000084	TCONS_00000157	dna-directed rna polymerases i and iii subunit rpac2	DNA-directed RNA polymerase III complex;DNA binding;DNA-directed RNA polymerase activity;protein dimerization activity;transcription, DNA-templated	ec:2.7.7.6-RNA polymerase	Purine metabolism;Pyrimidine metabolism
XLOC_000219	TCONS_00000420	aspartate aminotransferase mitochondrial	L-aspartate:2-oxoglutarate aminotransferase activity;pyridoxal phosphate binding;L-phenylalanine:2-oxoglutarate aminotransferase activity;cellular amino acid metabolic process;biosynthetic process	ec:2.6.1.57-transaminase;ec:2.6.1.9-transaminase;ec:2.6.1.5-transaminase;ec:2.6.1.1-transaminase	Lysine biosynthesis;Phenylalanine metabolism;Tropane, piperidine and pyridine alkaloid biosynthesis;Tyrosine metabolism;Biosynthesis of antibiotics;Isoquinoline alkaloid biosynthesis;Cysteine and methionine metabolism;Novobiocin biosynthesis;Phenylalanine, tyrosine and tryptophan biosynthesisPhenylalanine metabolism;Tropane, piperidine and pyridine alkaloid biosynthesis;Tyrosine metabolism;Biosynthesis of antibiotics;Histidine metabolism;Novobiocin biosynthesis;Phenylalanine, tyrosine and tryptophan biosynthesisPhenylalanine metabolism;Ubiquinone and other terpenoid-quinone biosynthesis;Tropane, piperidine and pyridine alkaloid biosynthesis;Tyrosine metabolism;Biosynthesis of antibiotics;Isoquinoline alkaloid biosynthesis;Cysteine and methionine metabolism;Novobiocin biosynthesis;Phenylalanine, tyrosine and tryptophan biosynthesisArginine and proline metabolism;Phenylalanine metabolism;Carbon fixation in photosynthetic organisms;Tropane, piperidine and pyridine alkaloid biosynthesis;Tyrosine metabolism;Arginine biosynthesis;Alanine, aspartate and glutamate metabolism;Biosynthesis of antibiotics;Isoquinoline alkaloid biosynthesis;Cysteine and methionine metabolism;Novobiocin biosynthesis;Phenylalanine, tyrosine and tryptophan biosynthesis
XLOC_000316	TCONS_00000626	tom1-like protein 2	intracellular;intracellular protein transport		
XLOC_000351	TCONS_00000697	peroxisomal -dienoyl-reductase-like			
XLOC_000434	TCONS_00000869	cysteine-rich and transmembrane domain-containing protein a-like			
XLOC_000483	TCONS_00000970	k(+) efflux antiporter 4 isoform x1			
XLOC_000484	TCONS_00000973	hypothetical protein PHAVU_001G118000g			
XLOC_000516	TCONS_00001026	regulation of nuclear pre-mrna domain-containing protein 1b-like			
XLOC_000534	TCONS_00001052	transcription factor iiib 90 kda subunit-like isoform x2	transcription factor TFIIIB complex;zinc ion binding;TBP-class protein binding;regulation of transcription, DNA-templated;transcription		

			initiation from RNA polymerase III promoter		
XLOC_000535	TCONS_00001053	transcription factor iiib 90 kda subunit-like isoform x2	transcription factor TFIIIB complex;zinc ion binding;TBP-class protein binding;regulation of transcription, DNA-templated;transcription initiation from RNA polymerase III promoter		
XLOC_000574	TCONS_00001132	histone deacetylase	nucleus;metal ion binding;cellular process		
XLOC_000576	TCONS_00001136	hypothetical protein PHAVU_L006100g			
XLOC_000666	TCONS_00001297	abc transporter g family member 15-like	plasmodesma;membrane;ATP binding;organic phosphonate transmembrane-transporting ATPase activity;metabolic process;response to salt stress;organic phosphonate transport;organophosphate ester transport;cutin transport;petal epidermis patterning	ec:3.6.3.28;ec:3.6.1.15-phosphatase;ec:3.6.1.3-adenylpyrophosphatase;ec:3.6.1	Purine metabolism;Thiamine metabolismPurine metabolism
XLOC_000683	TCONS_00001315	disease resistance protein rga4	phosphoprotein phosphatase activity;ADP binding;protein dephosphorylation	ec:3.1.3.41-nitrophenyl phosphatase;ec:3.1;ec:3.1.3.16-phosphatase	Aminobenzoate degradationT cell receptor signaling pathway
XLOC_000774	TCONS_00001487	pre-mrna-splicing factor 18	spliceosomal complex;RNA splicing		
XLOC_000790	TCONS_00001518	protein clec16a like			
XLOC_000814	TCONS_00001558	hypothetical protein LR48_Vigan05g011100			
XLOC_000895	TCONS_00001723	syntaxin of plants protein	endoplasmic reticulum;plasma membrane;plasmodesma;SNAP receptor activity;protein transporter activity;protein targeting to membrane;membrane fusion		
XLOC_001428	TCONS_00002742	hypothetical protein VIGAN_03280800			
XLOC_001558	TCONS_00002992	myosin-j heavy chain	myosin complex;motor activity;DNA topoisomerase type I activity;ATP binding;actin filament binding;metabolic process	ec:5.99.1.2;ec:3.6.1.15-phosphatase;ec:3.6.1;ec:5.99.1	Purine metabolism;Thiamine metabolism
XLOC_001581	TCONS_00003037	hypothetical protein VIGAN_03245500			
XLOC_001720	TCONS_00003349	peroxisomal -dienoyl-reductase-like	vacuolar membrane;peroxisome;kinesin complex;microtubule;microtubule motor activity;ATP binding;microtubule binding;2,4-dienoyl-CoA reductase (NADPH) activity;microtubule-based movement;oxidation-reduction process	ec:3.6.1.15-phosphatase;ec:1.3.1.34;ec:3.6.1	Purine metabolism;Thiamine metabolism

XLOC_001761	TCONS_00003426	peptidyl-prolyl cis-trans isomerase fkbp62-like	nucleus;cytosol;cell periphery;peptidyl-prolyl cis-trans isomerase activity;phosphatidylinositol-3-phosphate binding;phosphatidylinositol-3,5-bisphosphate binding;protein peptidyl-prolyl isomerization;protein folding;response to osmotic stress;response to heat;response to cytokinin;seed germination	ec:5.2.1.8	
XLOC_001777	TCONS_00003464	glycerophosphodiester phosphodiesterase gdpd6	vacuole;glycerophosphodiester phosphodiesterase activity;glycerol metabolic process;lipid metabolic process	ec:3.1.4.46-phosphodiesterase;ec:3.1	Glycerophospholipid metabolism
XLOC_001781	TCONS_00003471	brefeldin a-inhibited guanine nucleotide-exchange protein 5	ARF guanyl-nucleotide exchange factor activity;regulation of ARF protein signal transduction;positive regulation of GTPase activity		
XLOC_001821	TCONS_00003544	ribonuclease h protein at1g65750			
XLOC_001840	TCONS_00003575	squalene synthase 1	integral component of membrane;farnesyl-diphosphate farnesyltransferase activity;squalene synthase activity;lipid biosynthetic process	ec:2.5.1.29-diphosphate synthase;ec:2.5.1.21-synthase;ec:2.5.1	Terpenoid backbone biosynthesis;Biosynthesis of antibioticsSesquiterpenoid and triterpenoid biosynthesis;Steroid biosynthesis;Biosynthesis of antibiotics
XLOC_001867	TCONS_00003614	transcription-repair-coupling factor	nucleic acid binding;helicase activity;ATP binding;DNA repair;heme biosynthetic process	ec:3.6.1.15-phosphatase;ec:3.6.1	Purine metabolism;Thiamine metabolism
XLOC_001912	TCONS_00003688	hypothetical protein VIGAN_04253900			
XLOC_002065	TCONS_00004007	fact complex subunit ssrp1-like	nucleus;DNA binding;2-alkenal reductase [NAD(P)] activity;oxidation-reduction process	ec:1.3.1.74	
XLOC_002066	TCONS_00004009	wrky transcription factor 12	transcription factor activity, sequence-specific DNA binding;sequence-specific DNA binding;regulation of transcription, DNA-templated		
XLOC_002145	TCONS_00004159	nuclease harbi1			
XLOC_002282	TCONS_00004444	transporter mch1	integral component of membrane;transmembrane transport		
XLOC_002305	TCONS_00004479	histone deacetylase hdt1	nucleic acid binding;metal ion binding		
XLOC_002484	TCONS_00004819	transcription factor pif3 isoform x1	nucleus;transcription factor activity, sequence-specific DNA binding;protein dimerization activity;regulation of transcription, DNA-templated		
XLOC_002563	TCONS_00004976	glutamate--cysteine chloroplastic	chloroplast stroma;glutamate-cysteine ligase activity;ATP binding;defense response to insect;glucose catabolic process;gluconeogenesis;glycolytic process;glutathione biosynthetic process;response to	ec:6.3.2.2-ligase	Glutathione metabolism;Cysteine and methionine metabolism

			heat;response to salt stress;indole phytoalexin biosynthetic process;defense response to bacterium, incompatible interaction;flower development;cysteine biosynthetic process;glucosinolate biosynthetic process;cellulose biosynthetic process;defense response to fungus;defense response by callose deposition in cell wall		
XLOC_002741	TCONS_00005314	atp synthase cf1 alpha partial	mitochondrion;chloroplast thylakoid membrane;proton-transporting ATP synthase complex, catalytic core F(1);ATP binding;proton-transporting ATP synthase activity, rotational mechanism;proton-transporting ATPase activity, rotational mechanism;ATP synthesis coupled proton transport;ATP hydrolysis coupled proton transport	ec:3.6.1.15-phosphatase;ec:3.6.1.3-adenylpyrophosphatase;ec:3.6.1	Purine metabolism;Thiamine metabolismPurine metabolism
XLOC_002747	TCONS_00005320	nuclease harbi1	vacuolar membrane;plasma membrane;chloroplast		
XLOC_002748	TCONS_00005321	hypothetical protein PHAVU_011G176000g	organic cyclic compound binding;heterocyclic compound binding		
XLOC_002749	TCONS_00005322	hypothetical protein PHAVU_001G154000g, partial			
XLOC_002752	TCONS_00005325	hypothetical protein PHAVU_001G048900g	protein serine/threonine kinase activity;ATP binding;polysaccharide binding;protein phosphorylation	ec:2.7.11	
XLOC_002753	TCONS_00005326	transmembrane amino acid transporter family protein	membrane		
XLOC_002756	TCONS_00005329	plasma membrane atpase 1			
XLOC_002762	TCONS_00005335	hypothetical protein (mitochondrion)			
XLOC_002763	TCONS_00005336	hypothetical protein glysoja_046184			
XLOC_002769	TCONS_00005342	hypothetical protein PHAVU_004G040400g, partial			
XLOC_002771	TCONS_00005344	catalytic region zinc cchc-type peptidase catalytic	metal ion binding		
XLOC_002776	TCONS_00005349	rna-directed dna polymerase (reverse transcriptase) chromo zinc cchc-type peptidase active site	binding;transferase activity;DNA metabolic process		

		polynucleotidyl ribonuclease h fold			
XLOC_002777	TCONS_00005350	hypothetical protein ARALYDRAFT_893970			
XLOC_002778	TCONS_00005351	ethylene-insensitive protein 2	intracellular;membrane;transporter activity;establishment of planar polarity;response to molecule of bacterial origin;lipid transport;intra-Golgi vesicle-mediated transport;response to heat;response to salt stress;positive regulation of abscisic acid-activated signaling pathway;jasmonic acid and ethylene-dependent systemic resistance, ethylene mediated signaling pathway;auxin polar transport;regulation of stomatal movement;leaf senescence;sugar mediated signaling pathway;lithium ion transport;protein import into peroxisome matrix;negative regulation of defense response;defense response to bacterium;root hair cell differentiation;cotyledon development;defense response to fungus;defense response by callose deposition in cell wall		
XLOC_002779	TCONS_00005352	hypothetical protein PHAVU_006G007700g			
XLOC_002780	TCONS_00005353	disease resistance protein (cc-nbs-lrr class) family protein	ATP binding;ADP binding		
XLOC_002781	TCONS_00005354	hypothetical protein PHAVU_006G024500g			
XLOC_002783	TCONS_00005356	esterase lipase thioesterase family protein	transferase activity, transferring acyl groups other than amino-acyl groups;metabolic process		
XLOC_002788	TCONS_00005361	rna-directed dna polymerase (reverse transcriptase) chromo zinc cchc-type peptidase active site polynucleotidyl ribonuclease h fold			
XLOC_002791	TCONS_00005364	hypothetical protein LR48_Vigan08g047200			
XLOC_002793	TCONS_00005366	hypothetical protein PHAVU_010G070700g			

XLOC_002794	TCONS_00005367	hypothetical protein PHAVU_004G102000g			
XLOC_002796	TCONS_00005369	hypothetical protein PHAVU_001G068700g			
XLOC_002797	TCONS_00005370	integrase core domain containing protein			
XLOC_002800	TCONS_00005373	serine protease chloroplastic-like	integral component of membrane;chloroplast part;peptidase activity;signal peptide processing		
XLOC_002801	TCONS_00005374	probable acyl-dehydrogenase ibr3 isoform x2			
XLOC_002803	TCONS_00005376	hypothetical protein PHAVU_001G035000g			
XLOC_002805	TCONS_00005378	hypothetical protein PHAVU_011G0958002g, partial			
XLOC_002806	TCONS_00005379	glycine-rich protein 2-like	DNA binding;zinc ion binding;regulation of transcription, DNA-templated		
XLOC_002808	TCONS_00005381	topless-related protein 4-like	regulation of transcription, DNA-templated		
XLOC_002809	TCONS_00005382	abc transporter family protein	nucleotide binding;ATPase activity, coupled to transmembrane movement of substances;single-organism transport;organic substance transport;nitrogen compound transport	ec:3.6.1.15-phosphatase;ec:3.6.1.3-adenylpyrophosphatase; ec:3.6.1	Purine metabolism;Thiamine metabolismPurine metabolism
XLOC_002810	TCONS_00005383	hypothetical protein PHAVU_001G126500g			
XLOC_002811	TCONS_00005384	disease resistance protein rga4	phosphoprotein phosphatase activity;ADP binding;protein dephosphorylation	ec:3.1.3.41-nitrophenyl phosphatase;ec:3.1;ec:3.1.3.16-phosphatase	Aminobenzoate degradationT cell receptor signaling pathway
XLOC_002812	TCONS_00005385	photosystem ii d2 protein	chloroplast;electron transporter, transferring electrons within the cyclic electron transport pathway of photosynthesis activity;photosynthetic electron transport in photosystem II		
XLOC_002813	TCONS_00005386	pre-mrna-splicing factor 18	spliceosomal complex;RNA splicing		
XLOC_002822	TCONS_00005395	photosystem i p700 apoprotein a1	plastid;photosynthetic membrane;binding;metabolic process		
XLOC_002836	TCONS_00005409	glycine-rich cell wall structural isoform x3			
XLOC_002840	TCONS_00005413	ribosomal protein s7	chloroplast;small ribosomal subunit;structural constituent of ribosome;rRNA binding;translation		

XLOC_003010	TCONS_00005733	hypothetical protein glysoja_003246			
XLOC_003011	TCONS_00005734	guanylate-binding protein 1-like			
XLOC_003305	TCONS_00006370	udp-n-acetylglucosamine transferase subunit alg14 like	N-acetylglucosaminyldiphosphodolichol N-acetylglucosaminyltransferase activity;metabolic process	ec:2.4.1.141-N-acetylglucosaminyltransferase;ec:2.4.1	Various types of N-glycan biosynthesis;N-Glycan biosynthesis
XLOC_003348	TCONS_00006442	pre-mrna-processing-splicing factor 8	spliceosomal complex;U6 snRNA binding;U5 snRNA binding;mRNA splicing, via spliceosome		
XLOC_003388	TCONS_00006518	alpha beta fold hydrolase	acylglycerol lipase activity;metabolic process	ec:3.1.1.23-lipase;ec:3.1.1.1-ali-esterase;ec:3.1.1	Glycerolipid metabolismDrug metabolism - other enzymes
XLOC_003500	TCONS_00006753	hypothetical protein PHAVU_002G130100g	ADP binding		
XLOC_003618	TCONS_00006959	copper transporter -like			
XLOC_003707	TCONS_00007106	hypothetical protein PHAVU_009G238900g			
XLOC_003713	TCONS_00007116	probable dna primase large subunit	primosome complex;DNA binding;DNA primase activity;metal ion binding;4 iron, 4 sulfur cluster binding;DNA replication, synthesis of RNA primer	ec:2.7.7.6-RNA polymerase; ec:2.7.7	Purine metabolism;Pyrimidine metabolism
XLOC_003731	TCONS_00007151	glyceraldehyde-3-phosphate dehydrogenase			
XLOC_004204	TCONS_00008106	hypothetical protein VIGAN_01070900			
XLOC_004473	TCONS_00008651	vacuolar protein sorting-associated			
XLOC_004843	TCONS_00009426	receptor-like serine threonine-protein kinase ale2	plasma membrane;protein serine/threonine kinase activity;ATP binding;protoderm histogenesis;cuticle development;protein autophosphorylation;organ formation	ec:2.7.11	
XLOC_004855	TCONS_00009462	peroxisomal acyl-coenzyme a oxidase-like protein	peroxisome;acyl-CoA dehydrogenase activity;acyl-CoA oxidase activity;flavin adenine dinucleotide binding;fatty acid beta-oxidation	ec:1.3.99.3;ec:1.3.3.6-oxidase	Fatty acid degradation;Biosynthesis of unsaturated fatty acids;alpha-Linolenic acid metabolism
XLOC_004920	TCONS_00009575	glycoside hydrolase family 79 amino-terminal domain protein	membrane;hydrolase activity, acting on glycosyl bonds;carbohydrate metabolic process		
XLOC_004975	TCONS_00009700	hypothetical protein			

		VIGAN_01263100			
XLOC_004981	TCONS_00009708	probable polygalacturonase at3g15720	extracellular region;polygalacturonase activity;carbohydrate metabolic process;cell wall organization	ec:3.2.1.15-pectin depolymerase	Pentose and glucuronate interconversions;Starch and sucrose metabolism
XLOC_005087	TCONS_00009916	hypothetical protein LR48_Vigan05g133500			
XLOC_005128	TCONS_00010004	serine threonine-protein kinase afc3	protein kinase activity;ATP binding;protein phosphorylation		
XLOC_005207	TCONS_00010158	glycogen synthase	starch synthase activity;metabolic process	ec:2.4.1.21-synthase (glycosyl-transferring);ec:2.4.1	Starch and sucrose metabolism
XLOC_005217	TCONS_00010176	PREDICTED: uncharacterized protein LOC106764048 isoform X2			
XLOC_005229	TCONS_00010196	zinc c3hc4 type (ring finger) protein	zinc ion binding		
XLOC_005273	TCONS_00010287	cpd photolyase			
XLOC_005354	TCONS_00010469	asparagine synthase	mitochondrion;double-stranded DNA binding;asparagine synthase (glutamine-hydrolyzing) activity;regulation of transcription, DNA-templated;asparagine biosynthetic process	ec:6.3.5.4-synthase (glutamine-hydrolysing)	Alanine, aspartate and glutamate metabolism
XLOC_005373	TCONS_00010513	transcription factor gte1			
XLOC_005471	TCONS_00010729	myo-inositol oxygenase	cytoplasm;iron ion binding;inositol oxygenase activity;inositol catabolic process;oxidation-reduction process	ec:1.13.99.1-oxygenase	Ascorbate and aldarate metabolism;Inositol phosphate metabolism
XLOC_005480	TCONS_00010750	inter-alpha-trypsin inhibitor heavy chain-like protein	transferase activity		
XLOC_005481	TCONS_00010751	inter-alpha-trypsin inhibitor heavy chain-like protein	NAD+ ADP-ribosyltransferase activity;glycogen phosphorylase activity;pyridoxal phosphate binding;carbohydrate metabolic process	ec:2.4.1.1-phosphorylase;ec:2.4.2.30	Starch and sucrose metabolism
XLOC_005513	TCONS_00010809	two-pore potassium channel 3-like	vacuolar membrane;integral component of membrane;voltage-gated potassium channel activity;cellular potassium ion homeostasis;potassium ion transmembrane transport		
XLOC_005582	TCONS_00010929	hypothetical protein PHAVU_002G108200g			
XLOC_005706	TCONS_00011167	hypothetical protein LR48_Vigan609s002200			
XLOC_005748	TCONS_00011255	hypothetical protein			

		VIGAN_01106500, partial			
XLOC_005913	TCONS_00011593	hypothetical protein VIGAN_01068200			
XLOC_006068	TCONS_00011895	atp-dependent zinc metalloprotease ftsh mitochondrial	integral component of membrane;metalloendopeptidase activity;ATP binding;proteolysis;iron ion transport;zinc II ion transport;magnesium ion transport;transmembrane transport	ec:3.4.24	
XLOC_006181	TCONS_00012152	luminal-binding protein 5	ATP binding;2-alkenal reductase [NAD(P)] activity;oxidation-reduction process	ec:1.3.1.74	
XLOC_006206	TCONS_00012196	hypothetical protein VIGAN_03201500			
XLOC_006208	TCONS_00012198	hypothetical protein PHAVU_005G0985002g, partial			
XLOC_006213	TCONS_00012203	ppr containing plant-like protein			
XLOC_006214	TCONS_00012204	hypothetical protein LR48_Vigan11g162500			
XLOC_006215	TCONS_00012205	hypothetical protein VIGAN_10206100			
XLOC_006223	TCONS_00012213	PREDICTED: uncharacterized protein LOC101488260, partial			
XLOC_006231	TCONS_00012221	lob domain-containing protein 4-like	carbamoyl-phosphate synthase (glutamine-hydrolyzing) activity;metabolic process;determination of bilateral symmetry;polarity specification of adaxial/abaxial axis;meristem initiation;regulation of meristem growth	ec:6.3.5.5-synthase (glutamine-hydrolysing)	Alanine, aspartate and glutamate metabolism;Pyrimidine metabolism
XLOC_006232	TCONS_00012222	l-ascorbate oxidase	extracellular region;copper ion binding;L-ascorbate oxidase activity;systemic acquired resistance;salicylic acid biosynthetic process;sterol biosynthetic process;sphingoid biosynthetic process;oxidation-reduction process	ec:1.10.3;ec:1.10.3.3-oxidase	Ascorbate and aldarate metabolism
XLOC_006233	TCONS_00012223	hypothetical protein PHAVU_011G146800g			
XLOC_006235	TCONS_00012225	rna polymerase beta subunit	chloroplast;DNA binding;DNA-directed RNA polymerase activity;transcription, DNA-templated	ec:2.7.7.6-RNA polymerase	Purine metabolism;Pyrimidine metabolism
XLOC_006236	TCONS_00012226	hypothetical chloroplast rf15	mitochondrion;chloroplast		

XLOC_006238	TCONS_00012228	trna(adenine) chloroplastic-like isoform x1	zinc ion binding;hydrolase activity;metabolic process		
XLOC_006244	TCONS_00012234	photosystem ii phosphoprotein	photosystem II;chloroplast thylakoid membrane;integral component of membrane;phosphate ion binding;photosynthesis;protein stabilization		
XLOC_006246	TCONS_00012236	ring u-box protein	zinc ion binding		
XLOC_006255	TCONS_00012245	aldo keto reductase family oxidoreductase	oxidoreductase activity;oxidation-reduction process		
XLOC_006256	TCONS_00012246	ribosomal protein l14	chloroplast;large ribosomal subunit;structural constituent of ribosome;rRNA binding;translation		
XLOC_006260	TCONS_00012250	uncharacterized mitochondrial protein g00810-like	nucleic acid binding;zinc ion binding		
XLOC_006262	TCONS_00012252	hypothetical protein VIGAN_01185400, partial			
XLOC_006263	TCONS_00012253	abc transporter c family member 2-like isoform x1			
XLOC_006265	TCONS_00012255	hypothetical protein PHAVU_L006100g			
XLOC_006266	TCONS_00012256	hypothetical protein LR48_Vigan205s006200			
XLOC_006269	TCONS_00012259	hypothetical protein PHAVU_008G128800g	organic cyclic compound binding;heterocyclic compound binding		
XLOC_006270	TCONS_00012260	ribosomal protein s14	mitochondrion;chloroplast;small ribosomal subunit;structural constituent of ribosome;rRNA binding;translation		
XLOC_006272	TCONS_00012262	hypothetical protein PHAVU_002G212100g			
XLOC_006276	TCONS_00012266	rna polymerase beta partial	chloroplast;DNA binding;DNA-directed RNA polymerase activity;transcription, DNA-templated	ec:2.7.7.6-RNA polymerase	Purine metabolism;Pyrimidine metabolism
XLOC_006278	TCONS_00012268	transmembrane protein, putative			
XLOC_006279	TCONS_00012269	ycf68 protein			
XLOC_006280	TCONS_00012270	probable dolichyl pyrophosphate man9 c2 alpha- -glucosyltransferase isoform x1	endoplasmic reticulum membrane;transferase activity, transferring hexosyl groups;metabolic process		

XLOC_006281	TCONS_00012271	wrky transcription factor 22-like	transcription factor activity, sequence-specific DNA binding;sequence-specific DNA binding;regulation of transcription, DNA-templated		
XLOC_006282	TCONS_00012272	hypothetical protein glysoja_010825			
XLOC_006284	TCONS_00012274	reverse partial	nucleus;nucleic acid binding;aspartic-type endopeptidase activity;zinc ion binding;proteolysis;DNA integration	ec:3.4.23	
XLOC_006293	TCONS_00012283	hypothetical protein glysoja_002809, partial			
XLOC_006296	TCONS_00012286	aspartate-semialdehyde dehydrogenase	mitochondrion;chloroplast stroma;N-acetyl-gamma-glutamyl-phosphate reductase activity;aspartate-semialdehyde dehydrogenase activity;protein dimerization activity;NADP binding;NAD binding;purine nucleotide biosynthetic process;methionine biosynthetic process;threonine biosynthetic process;lysine biosynthetic process via diaminopimelate;isoleucine biosynthetic process;oxidation-reduction process	ec:1.2.1.38-reductase;ec:1.2.1.11-dehydrogenase	Arginine biosynthesis;Biosynthesis of antibioticsLysine biosynthesis;Monobactam biosynthesis;Glycine, serine and threonine metabolism;Biosynthesis of antibiotics;Cysteine and methionine metabolism
XLOC_006298	TCONS_00012288	hypothetical protein glysoja_023492			
XLOC_006468	TCONS_00012620	peroxisomal -dienoyl-reductase	vacuolar membrane;peroxisome;2,4-dienoyl-CoA reductase (NADPH) activity;oxidation-reduction process	ec:1.3.1.34	
XLOC_006482	TCONS_00012647	ribonuclease h protein	catalytic activity;cellular macromolecule metabolic process;nucleic acid metabolic process		
XLOC_006612	TCONS_00012897	e3 ubiquitin-protein ligase herc1	ligase activity;metal ion binding;metabolic process		
XLOC_006730	TCONS_00013148	protein transport protein sec24-like at3g07100-like isoform x1	COPII vesicle coat;zinc ion binding;intracellular protein transport;ER to Golgi vesicle-mediated transport		
XLOC_006744	TCONS_00013176	fruit weight protein			
XLOC_006807	TCONS_00013301	pleiotropic drug resistance protein 1	integral component of membrane;ATP binding;ATPase activity;transport;metabolic process	ec:3.6.1.15-phosphatase;ec:3.6.1.3-adenylpyrophosphatase; ec:3.6.1	Purine metabolism;Thiamine metabolismPurine metabolism
XLOC_006949	TCONS_00013526	t-complex protein 1 subunit alpha	cellular protein metabolic process;single-organism process		
XLOC_006966	TCONS_00013564	pre-mrna-processing-splicing factor 8-like	spliceosomal complex;U6 snRNA binding;U5 snRNA binding;mRNA splicing, via spliceosome		

XLOC_007105	TCONS_00013857	hypothetical protein HQ43_09450			
XLOC_007105	TCONS_00013857	hypothetical protein HQ43_09450			
XLOC_007172	TCONS_00013999	pleiotropic drug resistance protein 1-like	integral component of membrane;ATP binding;ATPase activity;transport;metabolic process	ec:3.6.1.15-phosphatase;ec:3.6.1.3-adenylpyrophosphatase; ec:3.6.1	Purine metabolism;Thiamine metabolismPurine metabolism
XLOC_007185	TCONS_00014025	protein enhanced disease resistance 2-like isoform x1	lipid binding		
XLOC_007186	TCONS_00014026	pleckstrin-like and lipid-binding start domain protein	plasma membrane;lipid binding;cysteine biosynthetic process		
XLOC_007278	TCONS_00014224	pentatricopeptide repeat-containing protein at1g74750-like			
XLOC_007384	TCONS_00014450	agamous-like mads-box protein agl19			
XLOC_007414	TCONS_00014513	leukocyte receptor cluster member 1 homolog			
XLOC_007421	TCONS_00014529	dwnn a cchc-type zinc finger protein	nucleus;nucleic acid binding;zinc ion binding		
XLOC_007535	TCONS_00014761	frigida-like protein 3			
XLOC_007575	TCONS_00014830	lrr and nb-arc domain disease resistance protein			
XLOC_007745	TCONS_00015144	pentatricopeptide repeat-containing protein chloroplastic			
XLOC_007764	TCONS_00015198	asc1-like protein	endoplasmic reticulum membrane;integral component of membrane;sphingosine N-acyltransferase activity;lipid metabolic process	ec:2.3.1;ec:2.3.1.24-N-acyltransferase	Sphingolipid metabolism
XLOC_007862	TCONS_00015399	tetratricopeptide repeat protein 7b			
XLOC_007864	TCONS_00015402	ubx domain-containing protein 1	metal ion binding		
XLOC_007865	TCONS_00015404	hypothetical protein PHAVU_009G043500g	tRNA binding		
XLOC_008029	TCONS_00015793	h aca ribonucleoprotein complex subunit 1-like protein 1			
XLOC_008064	TCONS_00015856	ribonuclease h protein			
XLOC_008065	TCONS_00015857	ribonuclease h protein	catalytic activity;cellular macromolecule metabolic		

			process;nucleic acid metabolic process		
XLOC_008118	TCONS_00015985	probable cadmium zinc-transporting atpase chloroplastic	chloroplast envelope;integral component of membrane;calcium-transporting ATPase activity;ATP binding;cadmium-exporting ATPase activity;cadmium-transporting ATPase activity;zinc transporting ATPase activity;metal ion binding;cellular copper ion homeostasis;metabolic process;response to toxic substance;response to light intensity;zinc ion homeostasis;cadmium ion transmembrane transport;calcium ion transmembrane transport;zinc II ion transmembrane transport	ec:3.6.1.15-phosphatase;ec:3.6.3.8;ec:3.6.3.3;ec:3.6.1.3-adenylpyrophosphatase;ec:3.6.1;ec:3.6.3.46	Purine metabolism;Thiamine metabolismPurine metabolism
XLOC_008150	TCONS_00016046	hypothetical protein PHAVU_003G053500g			
XLOC_008186	TCONS_00016112	cysteine chloroplastic chromoplastic	cysteine synthase activity;transferase activity;cysteine biosynthetic process from serine	ec:2.5.1.47-synthase	Sulfur metabolism;Biosynthesis of antibiotics;Cysteine and methionine metabolism
XLOC_008327	TCONS_00016389	glyceraldehyde-3-phosphate dehydrogenase	chloroplast;glyceraldehyde-3-phosphate dehydrogenase (NAD+) (phosphorylating) activity;NADP binding;NAD binding;glucose metabolic process;glycolytic process;oxidation-reduction process	ec:1.2.1.59-dehydrogenase (NAD(P)+) (phosphorylating);ec:1.2.1.12-dehydrogenase (phosphorylating)	Glycolysis / Gluconeogenesis;Carbon fixation in photosynthetic organisms;Biosynthesis of antibioticsGlycolysis / Gluconeogenesis;Carbon fixation in photosynthetic organisms;Biosynthesis of antibiotics
XLOC_008370	TCONS_00016484	hypothetical protein PHAVU_005G102300g			
XLOC_008379	TCONS_00016499	pyrimidine-specific ribonucleoside hydrolase riha	xanthine dehydrogenase activity;hydrolase activity;oxidation-reduction process	ec:1.17.1;ec:1.17.1.4-dehydrogenase	Purine metabolism
XLOC_008395	TCONS_00016521	myosin-j heavy chain	myosin complex;motor activity;ATP binding;metabolic process	ec:3.6.1.15-phosphatase;ec:3.6.1	Purine metabolism;Thiamine metabolism
XLOC_008417	TCONS_00016574	respiratory burst oxidase-like protein	calcium ion binding		
XLOC_008533	TCONS_00016803	hypothetical protein GLYMA_13G109900			
XLOC_008645	TCONS_00017029	dna-directed rna polymerase chloroplastic	mitochondrion;DNA binding;DNA-directed RNA polymerase activity;transcription, DNA-templated	ec:2.7.7.6-RNA polymerase	Purine metabolism;Pyrimidine metabolism
XLOC_008646	TCONS_00017030	dna-directed rna polymerase chloroplastic	DNA binding;DNA-directed RNA polymerase activity;transcription, DNA-templated	ec:2.7.7.6-RNA polymerase	Purine metabolism;Pyrimidine metabolism
XLOC_008647	TCONS_00017031	dna-directed rna polymerase chloroplastic	mitochondrion;DNA binding;DNA-directed RNA polymerase activity;transcription, DNA-templated	ec:2.7.7.6-RNA polymerase	Purine metabolism;Pyrimidine metabolism
XLOC_008664	TCONS_00017066	PREDICTED: uncharacterize			

		d protein LOC106767492 isoform X1			
XLOC_008968	TCONS_00017704	neurofilament heavy partial			
XLOC_009024	TCONS_00017824	dna-binding protein escarola	response to hormone;floral organ development;floral whorl development;phyllome development		
XLOC_009070	TCONS_00017923	cbs domain-containing protein chloroplastic			
XLOC_009096	TCONS_00017981	peptidyl-prolyl cis-trans isomerase-like 3	peptidyl-prolyl cis-trans isomerase activity;protein peptidyl-prolyl isomerization;protein folding	ec:5.2.1.8	
XLOC_009245	TCONS_00018294	pentatricopeptide repeat-containing protein mitochondrial	mitochondrion;response to oxidative stress;response to salt stress;response to abscisic acid;mitochondrial ATP synthesis coupled electron transport		
XLOC_009305	TCONS_00018446	plastocyanin-like domain protein	electron carrier activity		
XLOC_009311	TCONS_00018452	nuclease harbi1			
XLOC_009312	TCONS_00018453	probable nucleoredoxin 1 isoform x6			
XLOC_009314	TCONS_00018455	hypothetical protein PHAVU_007G108600g			
XLOC_009315	TCONS_00018456	ribonuclease h protein at1g65750	nucleic acid binding;RNA-DNA hybrid ribonuclease activity;RNA phosphodiester bond hydrolysis, endonucleolytic	ec:3.1;ec:3.1.30;ec:3.1.26;ec:3.1.26.4	
XLOC_009317	TCONS_00018458	maturase k			
XLOC_009318	TCONS_00018459	isoflavone synthase 1	monooxygenase activity;iron ion binding;oxidoreductase activity, acting on paired donors, with incorporation or reduction of molecular oxygen;isomerase activity;heme binding;oxidation-reduction process		
XLOC_009319	TCONS_00018460	hypothetical protein PHAVU_003G053500g			
XLOC_009323	TCONS_00018464	hypothetical protein LR48_Vigan01g112400			
XLOC_009326	TCONS_00018467	gag-pol polyprotein	nucleic acid binding;zinc ion binding;DNA integration		
XLOC_009328	TCONS_00018469	hypothetical protein PHAVU_011G164600g			
XLOC_009332	TCONS_00018473	hypothetical protein PHAVU_001G104900g, partial			
XLOC_009333	TCONS_00018474	a subunit of nadh dehydrogenase	chloroplast thylakoid membrane;integral component of membrane;NADH dehydrogenase (ubiquinone)	ec:1.6.99.5;ec:1.6.99.3-dehydrogenase;ec:1.6.5.3-reductase	Oxidative phosphorylationOxidative phosphorylation

			activity;quinone binding;transport;ATP synthesis coupled electron transport	(H+-translocating)	
XLOC_009335	TCONS_00018476	hypothetical protein PHAVU_005G070100g			
XLOC_009336	TCONS_00018477	copia partial	nucleic acid binding;zinc ion binding		
XLOC_009338	TCONS_00018479	lysine-specific demethylase jmj16	nucleus;transcription factor activity, sequence-specific DNA binding;methyltransferase activity;oxidoreductase activity;protein glycosylation;gravitropism;vegetative to reproductive phase transition of meristem;glucuronoxylan metabolic process;protein desumoylation;methylation;post-translational protein modification;xylan biosynthetic process;positive regulation of transcription, DNA-templated;hydrogen peroxide biosynthetic process;oxidation-reduction process		
XLOC_009340	TCONS_00018481	PREDICTED: uncharacterized protein LOC105778352			
XLOC_009345	TCONS_00018486	copia partial	binding;transferase activity, transferring alkyl or aryl (other than methyl) groups;metabolic process		
XLOC_009347	TCONS_00018488	hypothetical protein PHAVU_002G097600g, partial			
XLOC_009348	TCONS_00018489	photosystem i p700 chlorophyll a partial	ribosome;chloroplast;photosystem I;integral component of membrane;structural constituent of ribosome;ATP binding;translation;photosynthesis		
XLOC_009349	TCONS_00018490	hypothetical protein B456_001G165700			
XLOC_009353	TCONS_00018494	probable prefoldin subunit 3			
XLOC_009354	TCONS_00018495	lysine-specific demethylase jmj16	nucleus;transcription factor activity, sequence-specific DNA binding;methyltransferase activity;oxidoreductase activity;protein glycosylation;gravitropism;vegetative to reproductive phase transition of meristem;glucuronoxylan metabolic process;protein desumoylation;methylation;post-translational protein modification;xylan biosynthetic process;positive regulation of transcription, DNA-templated;hydrogen peroxide biosynthetic process;oxidation-reduction process		

XLOC_009355	TCONS_00018496	myb transcription factor	DNA binding		
XLOC_009356	TCONS_00018497	maturase k	chloroplast;RNA binding;mRNA processing;tRNA processing;RNA splicing		
XLOC_009360	TCONS_00018501	mannose-1-phosphate guanylyltransferase 1-like	mannose-1-phosphate guanylyltransferase (GDP) activity;biosynthetic process	ec:2.7.7.22-guanylyltransferase (GDP);ec:2.7.7	Amino sugar and nucleotide sugar metabolism;Fructose and mannose metabolism
XLOC_009361	TCONS_00018502	nad h-quinone oxidoreductase subunit 2 chloroplastic isoform x2	chloroplast		
XLOC_009362	TCONS_00018503	nuclease harbi1			
XLOC_009363	TCONS_00018504	hypothetical protein PHAVU_011G004100g			
XLOC_009365	TCONS_00018506	hypothetical protein PHAVU_008G101400g	nucleotide binding;nucleic acid binding		
XLOC_009367	TCONS_00018508	k(+) efflux antiporter 4 isoform x1			
XLOC_009370	TCONS_00018511	pathogenesis-related protein pr-1	extracellular region		
XLOC_009372	TCONS_00018513	hypothetical protein PHAVU_011G148300g			
XLOC_009374	TCONS_00018515	hypothetical protein VIGAN_01410200			
XLOC_009376	TCONS_00018517	PREDICTED: uncharacterized protein LOC103931614			
XLOC_009378	TCONS_00018519	upf0057 membrane protein at4g30660-like			
XLOC_009379	TCONS_00018520	serine carboxypeptidase 24-like	carboxypeptidase activity		
XLOC_009395	TCONS_00018536	werner syndrome-like exonuclease	nucleic acid binding;3'-5' exonuclease activity;nucleic acid phosphodiester bond hydrolysis	ec:3.1	
XLOC_009397	TCONS_00018538	ribonuclease h protein at1g65750			
XLOC_009400	TCONS_00018541	PREDICTED: uncharacterized protein LOC103501196			
XLOC_009401	TCONS_00018542	photosystem i p700 apoprotein a1	photosystem I;chloroplast thylakoid membrane;integral component of membrane;magnesium ion binding;electron carrier activity;chlorophyll binding;oxidoreductase activity;4 iron, 4 sulfur cluster binding;photosynthesis;prot		

			ein-chromophore linkage;oxidation-reduction process		
XLOC_009407	TCONS_00018548	gag polyprotein			
XLOC_009413	TCONS_00018554	rna polymerase beta subunit	chloroplast;DNA binding;DNA-directed RNA polymerase activity;transcription, DNA-templated	ec:2.7.7.6-RNA polymerase	Purine metabolism;Pyrimidine metabolism
XLOC_009417	TCONS_00018558	hypothetical protein PHAVU_003G107200g			
XLOC_009421	TCONS_00018562	photosystem ii p680 chlorophyll a partial	chloroplast;photosystem;integral component of membrane;chlorophyll binding;photosynthetic electron transport chain;protein-chromophore linkage		
XLOC_009572	TCONS_00018843	zeitlupe partial			
XLOC_009795	TCONS_00019241	ccr4-not transcription complex subunit 3	nucleus;regulation of transcription, DNA-templated		
XLOC_009806	TCONS_00019258	pentatricopeptide repeat-containing protein mitochondrial-like			
XLOC_009813	TCONS_00019271	leucine-rich repeat receptor-like tyrosine-protein kinase at2g41820-like	protein kinase activity;ATP binding;protein phosphorylation		
XLOC_009814	TCONS_00019272	lrr receptor-like kinase family protein	membrane;protein kinase activity;ATP binding;protein phosphorylation		
XLOC_009829	TCONS_00019290	beta-galactosidase 17	beta-galactosidase activity;protein kinase activity;ATP binding;protein phosphorylation;vegetative to reproductive phase transition of meristem;glucuronoxylan metabolic process;protein desumoylation;xylan biosynthetic process;hydrogen peroxide biosynthetic process	ec:3.2.1.23-lactase (ambiguous); ec:3.2.1	Other glycan degradation;Glycosphingolipid biosynthesis - ganglio series;Sphingolipid metabolism;Galactose metabolism;Glycosaminoglycan degradation
XLOC_009853	TCONS_00019336	hypothetical protein PHAVU_005G084700g			
XLOC_009855	TCONS_00019339	hypothetical protein PHAVU_003G046100g			
XLOC_009875	TCONS_00019375	transcription initiation factor tfiid subunit 10			
XLOC_009889	TCONS_00019395	protein lurp-one-related 6			
XLOC_009934	TCONS_00019460	probable wrky transcription factor 12	transcription factor activity, sequence-specific DNA binding;sequence-specific DNA binding;regulation of transcription, DNA-templated		

XLOC_009935	TCONS_00019462	fact complex subunit ssrp1	nuclear euchromatin;FACT complex;DNA binding;2-alkenal reductase [NAD(P)] activity;polar nucleus fusion;vegetative to reproductive phase transition of meristem;oxidation-reduction process	ec:1.3.1.74	
XLOC_010310	TCONS_00020188	PREDICTED: uncharacterized protein LOC104810178			
XLOC_010322	TCONS_00020213	hypothetical protein PHAVU_L009500g			
XLOC_010328	TCONS_00020220	hypothetical protein PHAVU_004G0078002g, partial			
XLOC_010340	TCONS_00020245	nbs-lrr type disease resistance protein cnl-j9	ADP binding		
XLOC_010438	TCONS_00020452	abc transporter b family member 25-like isoform x2			
XLOC_010568	TCONS_00020646	pentatricopeptide repeat-containing protein at2g15690	microtubule-severing ATPase activity;metabolic process	ec:3.6.1.15-phosphatase;ec:3.6.4.3;ec:3.6.1.3-adenylpyrophosphatase;ec:3.6.1	Purine metabolism;Thiamine metabolismPurine metabolism
XLOC_010633	TCONS_00020763	chaperone protein dnaj 10-like			
XLOC_010649	TCONS_00020793	gdt1-like protein 2			
XLOC_010653	TCONS_00020802	bifunctional epoxide hydrolase 2-like	peroxisome;epoxide hydrolase activity;cis-stilbene-oxide hydrolase activity;metabolic process	ec:3.3.2.10-epoxide hydrolase;ec:3.3.2.9-epoxide hydrolase	Chloroalkane and chloroalkene degradation;Arachidonic acid metabolismMetabolism of xenobiotics by cytochrome P450
XLOC_010687	TCONS_00020869	p23 co-chaperone protein			
XLOC_010703	TCONS_00020896	nuclear pore complex protein nup107 isoform x2			
XLOC_010718	TCONS_00020916	t-complex protein 1 subunit theta-like	cytoplasm;ATP binding;unfolded protein binding;protein folding		
XLOC_010758	TCONS_00020992	PREDICTED: uncharacterized protein LOC102665818			
XLOC_010839	TCONS_00021136	dna replication complex gins protein psf2	nucleus;DNA replication		
XLOC_010897	TCONS_00021237	short-chain dehydrogenase tic chloroplastic	oxidoreductase activity;oxidation-reduction process		

XLOC_010898	TCONS_00021238	short-chain dehydrogenase tic chloroplastic	chloroplast inner membrane;calmodulin binding;oxidoreductase activity;protein transport;oxidation-reduction process		
XLOC_010936	TCONS_00021319	hypothetical protein LR48_Vigan10g088200			
XLOC_010963	TCONS_00021361	hypothetical protein PHAVU_001G035000g			
XLOC_011009	TCONS_00021428	nuclease harbi1	kinase activity;phosphorylation		
XLOC_011095	TCONS_00021580	nuclease harbi1			
XLOC_011121	TCONS_00021630	mechanosensitive ion channel protein chloroplastic-like isoform x1	plasma membrane;integral component of membrane;chloroplast membrane;ion channel activity;response to osmotic stress;jasmonic acid metabolic process;response to jasmonic acid;chloroplast fission;ion transmembrane transport		
XLOC_011259	TCONS_00021849	hypothetical protein PHAVU_008G145700g, partial			
XLOC_011260	TCONS_00021850	phospholipid-transporting atpase 8	integral component of membrane;magnesium ion binding;phospholipid-translocating ATPase activity;ATP binding;metabolic process;phospholipid translocation	ec:3.6.1.15-phosphatase;ec:3.6.3.1;ec:3.6.1.3-adenylpyrophosphatase;ec:3.6.1	Purine metabolism;Thiamine metabolismPurine metabolism
XLOC_011262	TCONS_00021852	disease resistance protein cyr1	ADP binding		
XLOC_011263	TCONS_00021853	senescence-associated protein			
XLOC_011264	TCONS_00021854	metalloendoproteinase 1			
XLOC_011271	TCONS_00021861	chaperone protein 1	ATP binding;protein metabolic process		
XLOC_011272	TCONS_00021862	hypothetical protein glysoja_045364, partial			
XLOC_011277	TCONS_00021867	oligopeptide transporter 7	methyltransferase activity;methylation;transmembrane transport		
XLOC_011282	TCONS_00021872	neoxanthin synthase	PSII associated light-harvesting complex II;chloroplast envelope;abscisic acid biosynthetic process;photoprotection;xanthophyll metabolic process;regulation of superoxide anion generation;hydrogen peroxide catabolic process		
XLOC_011283	TCONS_00021873	gag- partial			
XLOC_011284	TCONS_00021874	PREDICTED: uncharacterized protein LOC102668698			

XLOC_011286	TCONS_00021876	transcription factor scream2-like	DNA binding;protein dimerization activity		
XLOC_011289	TCONS_00021879	mitochondrial phosphate carrier protein mitochondrial-like	integral component of membrane;glutathione transferase activity;transport;metabolic process;response to salt stress;response to ethylene	ec:2.5.1.18-transferase	Glutathione metabolism;Drug metabolism - cytochrome P450;Metabolism of xenobiotics by cytochrome P450
XLOC_011290	TCONS_00021880	hypothetical protein LR48_Vigan406s007800			
XLOC_011294	TCONS_00021884	hypothetical protein PHAVU_008G035300g			
XLOC_011298	TCONS_00021888	PREDICTED: uncharacterized protein LOC102664980			
XLOC_011303	TCONS_00021893	PREDICTED: uncharacterized protein LOC106779432			
XLOC_011307	TCONS_00021897	hypothetical protein VIGAN_03201500			
XLOC_011308	TCONS_00021898	gag-pol poly	nucleic acid binding;ion binding;metabolic process		
XLOC_011309	TCONS_00021899	glycine--trna mitochondrial 1			
XLOC_011313	TCONS_00021903	hypothetical protein PHAVU_009G156200g			
XLOC_011314	TCONS_00021904	protein involved in de novo 2-like	gene silencing by RNA		
XLOC_011315	TCONS_00021905	pollen protein ole e i-like protein	structural constituent of cell wall;plant-type cell wall organization		
XLOC_011317	TCONS_00021907	hypothetical protein PHAVU_009G162400g			
XLOC_011321	TCONS_00021911	lrr receptor-like serine threonine-protein kinase gso2-like			
XLOC_011323	TCONS_00021913	nadh-plastoquinone oxidoreductase subunit 6	chloroplast thylakoid membrane;integral component of membrane;NADH dehydrogenase (ubiquinone) activity;quinone binding;transport;oxidation-reduction process	ec:1.6.99.5;ec:1.6.99.3-dehydrogenase;ec:1.6.5.3-reductase (H+-translocating)	Oxidative phosphorylationOxidative phosphorylation
XLOC_011324	TCONS_00021914	extensin-2 isoform x3			
XLOC_011326	TCONS_00021916	rna-binding protein with multiple splicing	nucleotide binding;snRNA binding;mRNA splicing, via spliceosome		
XLOC_011331	TCONS_00021921	hypothetical protein M569_00246			
XLOC_011335	TCONS_00021925	hypothetical protein			

		PHAVU_002G163900g			
XLOC_011337	TCONS_00021927	wrky family transcription factor	transcription factor activity, sequence-specific DNA binding;sequence-specific DNA binding;regulation of transcription, DNA-templated		
XLOC_011341	TCONS_00021931	lrr receptor-like serine threonine-protein kinase gso2-like			
XLOC_011343	TCONS_00021933	PREDICTED: uncharacterized protein LOC106766088			
XLOC_011344	TCONS_00021934	ribosomal protein s11	ribosome;chloroplast;structural constituent of ribosome;rRNA binding;translation		
XLOC_011346	TCONS_00021936	hypothetical protein PHAVU_L006100g	metal ion binding		
XLOC_011348	TCONS_00021938	rna polymerase beta subunit	mitochondrion;chloroplast;DNA binding;DNA-directed RNA polymerase activity;ribonucleoside binding;transcription, DNA-templated	ec:2.7.7.6-RNA polymerase	Purine metabolism;Pyrimidine metabolism
XLOC_011349	TCONS_00021939	udp-glycosyltransferase 90a1-like	transferase activity		
XLOC_011350	TCONS_00021940	nadh-plastoquinone oxidoreductase subunit 7			
XLOC_011352	TCONS_00021942	udp-glucosyl transferase isoform 1	coniferyl-alcohol glucosyltransferase activity;hydroquinone glucosyltransferase activity;metabolic process	ec:2.4.1.218; ec:2.4.1.111-glucosyltransferase;ec:2.4.1	Phenylpropanoid biosynthesis
XLOC_011355	TCONS_00021945	photosystem ii d2 partial	chloroplast;photosystem II;integral component of membrane;electron transporter, transferring electrons within the cyclic electron transport pathway of photosynthesis activity;metal ion binding;photosynthetic electron transport in photosystem II		
XLOC_011446	TCONS_00022106	hypothetical protein PHAVU_002G108200g			
XLOC_011500	TCONS_00022219	1-o-acylglucose:anthocyanin acyltransferase	integral component of membrane;serine-type carboxypeptidase activity;ATP binding;transferase activity, transferring acyl groups;ATPase activity, coupled to transmembrane movement of substances;proteolysis;transmembrane transport	ec:3.4.16;ec:3.6.1.15-phosphatase;ec:3.6.1.3-adenylpyrophosphatase; ec:3.6.1;ec:3.4.21	Purine metabolism;Thiamine metabolismPurine metabolism
XLOC_011602	TCONS_00022423	peroxisomal -2-hydroxy-acid oxidase isoform x1	FMN binding;very-long-chain-(S)-2-hydroxy-acid oxidase activity;long-chain-(S)-2-hydroxy-long-chain-acid oxidase activity;medium-chain-(S)-2-	ec:1.1.3.15-oxidase	Biosynthesis of antibiotics;Glyoxylate and dicarboxylate metabolism

			hydroxy-acid oxidase activity;oxidation-reduction process		
XLOC_011666	TCONS_00022546	dna polymerase epsilon catalytic subunit a	epsilon DNA polymerase complex;nucleotide binding;DNA binding;DNA-directed DNA polymerase activity;zinc ion binding;3'-5' exonuclease activity;DNA replication;DNA repair;DNA biosynthetic process;nucleic acid phosphodiester bond hydrolysis	ec:3.1;ec:2.7.7.7-DNA polymerase	Purine metabolism;Pyrimidine metabolism
XLOC_011667	TCONS_00022547	PREDICTED: uncharacterized protein LOC105954144	binding		
XLOC_011689	TCONS_00022593	nuclear pore complex protein nup98-nup96	nuclear pore;transport		
XLOC_011715	TCONS_00022648	protein yippee-like at5g53940	nucleus		
XLOC_011720	TCONS_00022655	dna polymerase delta small subunit	DNA binding;DNA-directed DNA polymerase activity;metal ion binding;DNA-dependent DNA replication;DNA methylation;methylation-dependent chromatin silencing;microtubule nucleation;cell proliferation;embryo sac egg cell differentiation;regulation of flower development;chromatin silencing by small RNA;floral organ formation;histone H3-K9 methylation;DNA biosynthetic process	ec:2.7.7.7-DNA polymerase	Purine metabolism;Pyrimidine metabolism
XLOC_011742	TCONS_00022706	choline transporter-like protein 2	trans-Golgi network;plasma membrane;plasmodesma;phragmoplast;integral component of membrane;sieve plate;choline transmembrane transporter activity;xylem and phloem pattern formation;phloem development;choline transport;root development;sieve element differentiation		
XLOC_011743	TCONS_00022707	choline transporter-like protein 2	trans-Golgi network;plasma membrane;plasmodesma;phragmoplast;integral component of membrane;sieve plate;choline transmembrane transporter activity;xylem and phloem pattern formation;phloem development;choline transport;root development;sieve element differentiation		
XLOC_011757	TCONS_00022736	transcription factor gte1			
XLOC_011759	TCONS_00022742	trafficking protein particle complex ii-specific	trans-Golgi network;cell plate assembly		

		subunit 130 homolog			
XLOC_011869	TCONS_00022953	inter-alpha-trypsin inhibitor heavy chain-like protein	NAD+ ADP-ribosyltransferase activity;metabolic process	ec:2.4.2.30	
XLOC_011898	TCONS_00023010	hypothetical protein LR48_Vigan05g161300			
XLOC_011997	TCONS_00023212	solanesyl diphosphate synthase	heptaprenyl diphosphate synthase activity;trans-octaprenyltranstransferase activity;all-trans-nonaprenyl-diphosphate synthase (geranyl-diphosphate specific) activity;isoprenoid biosynthetic process;photosynthesis	ec:2.5.1.30-diphosphate synthase;ec:2.5.1.85-diphosphate synthase [geranylgeranyl-diphosphate specific];ec:2.5.1.84-synthase [geranyl-diphosphate specific]	Terpenoid backbone biosynthesisTerpenoid backbone biosynthesisTerpenoid backbone biosynthesis
XLOC_012032	TCONS_00023285	nuclease harbi1	ATP binding;sequence-specific DNA binding;DNA replication initiation;regulation of DNA replication		
XLOC_012070	TCONS_00023355	extensin-2-like isoform x1			
XLOC_012138	TCONS_00023499	leucine-rich repeat receptor-like protein kinase	chloroplast;protein serine/threonine kinase activity;ATP binding;acyl-[acyl-carrier-protein] desaturase activity;protein phosphorylation;fatty acid biosynthetic process;oxidation-reduction process	ec:2.7.11;ec:1.14.19;ec:1.14.19.2-9-desaturase	Fatty acid biosynthesis;Biosynthesis of unsaturated fatty acids
XLOC_012375	TCONS_00024011	mlp-like protein 31	defense response;response to biotic stimulus		
XLOC_012376	TCONS_00024012	mlp-like protein 28	defense response;response to biotic stimulus		
XLOC_012504	TCONS_00024255	amino-terminal domain cyclin	protein kinase binding;regulation of cyclin-dependent protein serine/threonine kinase activity;regulation of transcription, DNA-templated		
XLOC_012532	TCONS_00024315	peroxisomal -2-hydroxy-acid oxidase	FMN binding;very-long-chain-(S)-2-hydroxy-acid oxidase activity;long-chain-(S)-2-hydroxy-long-chain-acid oxidase activity;medium-chain-(S)-2-hydroxy-acid oxidase activity;oxidation-reduction process	ec:1.1.3.15-oxidase	Biosynthesis of antibiotics;Glyoxylate and dicarboxylate metabolism
XLOC_012624	TCONS_00024473	dna ligase 1	nucleotide binding;DNA ligase activity;DNA repair;cellular macromolecule biosynthetic process	ec:6.5.1	
XLOC_012674	TCONS_00024585	lrr receptor-like kinase	protein kinase activity;protein phosphorylation		
XLOC_012679	TCONS_00024596	probable leucine-rich repeat receptor-like serine threonine-	transferase activity		

		protein kinase at3g14840			
XLOC_012682	TCONS_00024604	probable leucine-rich repeat receptor-like serine threonine-protein kinase at3g14840	protein kinase activity;ATP binding;protein phosphorylation		
XLOC_012683	TCONS_00024605	lrr receptor-like kinase	protein kinase activity;ATP binding;protein phosphorylation		
XLOC_012699	TCONS_00024631	lysine-specific demethylase jmj16	nucleus;transcription factor activity, sequence-specific DNA binding;methyltransferase activity;oxidoreductase activity;protein glycosylation;gravitropism;vegetative to reproductive phase transition of meristem;glucuronoxylan metabolic process;protein desumoylation;methylation; post-translational protein modification;xylan biosynthetic process;positive regulation of transcription, DNA-templated;hydrogen peroxide biosynthetic process;oxidation-reduction process		
XLOC_012706	TCONS_00024646	histone acetyltransferase hac1-like isoform x1	nucleus;transcription cofactor activity;histone acetyltransferase activity;zinc ion binding;regulation of transcription, DNA-templated;histone acetylation	ec:2.3.1.48;ec:2.3.1	
XLOC_012721	TCONS_00024689	hypothetical protein PHAVU_003G116000g, partial	nucleic acid binding;zinc ion binding		
XLOC_012735	TCONS_00024721	hypothetical protein PHAVU_005G084700g			
XLOC_012966	TCONS_00025137	beta- -galactosyltransferase 2	Golgi apparatus;integral component of membrane;galactosylxylosyl protein 3-beta-galactosyltransferase activity;protein glycosylation	ec:2.4.1.134-3-beta-galactosyltransferase;ec:2.4.1	Glycosaminoglycan biosynthesis - heparan sulfate / heparin;Glycosaminoglycan biosynthesis - chondroitin sulfate / dermatan sulfate
XLOC_012967	TCONS_00025142	beta- -galactosyltransferase 2	Golgi apparatus;integral component of membrane;galactosylxylosyl protein 3-beta-galactosyltransferase activity;protein glycosylation	ec:2.4.1.134-3-beta-galactosyltransferase;ec:2.4.1	Glycosaminoglycan biosynthesis - heparan sulfate / heparin;Glycosaminoglycan biosynthesis - chondroitin sulfate / dermatan sulfate
XLOC_013129	TCONS_00025478	leucine-rich repeat receptor-like protein kinase	protein serine/threonine kinase activity;ATP binding;protein phosphorylation	ec:2.7.11	
XLOC_013244	TCONS_00025686	zinc finger protein zpr1-like	nucleus;cytosol;zinc ion binding		
XLOC_013247	TCONS_00025689	reverse partial	transferase activity, transferring alkyl or aryl (other than methyl) groups;metabolic process		
XLOC_013252	TCONS_00025694	transmembrane			
XLOC_013255	TCONS_00025697	copia partial	nucleic acid binding;zinc ion binding		

XLOC_013256	TCONS_00025698	e3 ubiquitin-protein ligase rglg2-like	zinc ion binding		
XLOC_013258	TCONS_00025700	hypothetical protein LR48_Vigan01g134000			
XLOC_013261	TCONS_00025703	protein pat1 like 1	isomerase activity;metabolic process		
XLOC_013267	TCONS_00025709	ribonuclease h partial	oxidoreductase activity		
XLOC_013268	TCONS_00025710	PREDICTED: uncharacterized protein LOC105954144	nucleic acid binding		
XLOC_013269	TCONS_00025711	clavata3 esr gene family member			
XLOC_013275	TCONS_00025717	disease resistance protein (tir-nbs-lrr class)	ADP binding;signal transduction		
XLOC_013277	TCONS_00025719	hypothetical protein PHAVU_009G162400g			
XLOC_013278	TCONS_00025720	photosystem i p700 apoprotein a2	photosystem I;chloroplast thylakoid membrane;integral component of membrane;magnesium ion binding;electron carrier activity;chlorophyll binding;oxidoreductase activity;4 iron, 4 sulfur cluster binding;photosynthesis;protein-chromophore linkage;oxidation-reduction process		
XLOC_013280	TCONS_00025722	hypothetical protein PHAVU_005G079800g			
XLOC_013281	TCONS_00025723	ethylene-responsive transcription factor rap2-7-like	nucleus;DNA binding;transcription factor activity, sequence-specific DNA binding;transcription, DNA-templated;regulation of transcription, DNA-templated;multicellular organismal development		
XLOC_013283	TCONS_00025725	hypothetical protein PHAVU_001G081900g, partial			
XLOC_013288	TCONS_00025730	hypothetical chloroplast rf2	chloroplast stroma;ATP binding		
XLOC_013290	TCONS_00025732	pentatricopeptide repeat-containing mitochondrial			
XLOC_013296	TCONS_00025738	hydroxcinnamoyl- quinate shikimate partial	shikimate O-hydroxycinnamoyltransferase activity;metabolic process	ec:2.3.1;ec:2.3.1.133-O-hydroxycinnamoyltransferase	Stilbenoid, diarylheptanoid and gingerol biosynthesis;Flavonoid biosynthesis;Phenylpropanoid biosynthesis
XLOC_013297	TCONS_00025739	glutamyl-trna reductase chloroplastic-like	chloroplast;glutamyl-tRNA reductase activity;NADP binding;protoporphyrinogen IX biosynthetic process;chlorophyll biosynthetic process;oxidation-reduction process	ec:1.2.1.70-reductase	Porphyrin and chlorophyll metabolism

XLOC_013301	TCONS_00025743	hypothetical protein PHAVU_001G067100g			
XLOC_013302	TCONS_00025744	shikimate o-hydroxycinnamoyltransferase-like	shikimate O-hydroxycinnamoyltransferase activity;metabolic process	ec:2.3.1;ec:2.3.1.133-O-hydroxycinnamoyltransferase	Stilbenoid, diarylheptanoid and gingerol biosynthesis;Flavonoid biosynthesis;Phenylpropanoid biosynthesis
XLOC_013303	TCONS_00025745	pentatricopeptide repeat-containing protein at1g06270	plastid membrane		
XLOC_013306	TCONS_00025748	probable indole-3-acetic acid-amido synthetase	jasmonate-amino synthetase activity;response to wounding;jasmonic acid metabolic process;induced systemic resistance, jasmonic acid mediated signaling pathway		
XLOC_013307	TCONS_00025749	hypothetical protein PHAVU_002G085800g			
XLOC_013308	TCONS_00025750	probable serine threonine-protein kinase fhke-like			
XLOC_013309	TCONS_00025751	hypothetical protein LR48_Vigan05g172500	nucleic acid binding;protein dimerization activity		
XLOC_013314	TCONS_00025756	hypothetical protein Poptr_cp015			
XLOC_013320	TCONS_00025762	nadh-plastoquinone oxidoreductase subunit 2	plasma membrane;chloroplast thylakoid membrane;integral component of membrane;NADH dehydrogenase (ubiquinone) activity;quinone binding;transport;photosynthesis, light reaction;ATP synthesis coupled electron transport	ec:1.6.99.5;ec:1.6.99.3-dehydrogenase;ec:1.6.5.3-reductase (H+-translocating)	Oxidative phosphorylationOxidative phosphorylation
XLOC_013322	TCONS_00025764	hypothetical protein PHAVU_001G0336001g, partial	DNA binding		
XLOC_013342	TCONS_00025801	transcriptional adapter ada2a	nucleus;DNA binding;zinc ion binding;regulation of transcription from RNA polymerase II promoter;histone acetylation		
XLOC_013365	TCONS_00025862	pleiotropic drug resistance subfamily g protein	plasmodesma;integral component of membrane;ATP binding;ATPase activity;transport;metabolic process	ec:3.6.1.15-phosphatase;ec:3.6.1.3-adenylpyrophosphatase;ec:3.6.1	Purine metabolism;Thiamine metabolismPurine metabolism
XLOC_013397	TCONS_00025929	telomeric repeat-binding factor 1-like	metal ion binding		
XLOC_013398	TCONS_00025931	transposon tf2-1 polyprotein isoform x1			
XLOC_013426	TCONS_00025981	hypothetical protein PHAVU_003G274300g			

XLOC_013473	TCONS_00026077	6-phosphofructo-2-kinase fructose- -bisphosphatase-like	binding;organic substance metabolic process		
XLOC_013495	TCONS_00026111	hypothetical protein			
XLOC_013496	TCONS_00026113	hypothetical protein			
XLOC_013503	TCONS_00026131	lysine-specific demethylase jmj16	metal ion binding;primary metabolic process;cellular macromolecule metabolic process		
XLOC_013569	TCONS_00026229	protein zinc induced facilitator-like 1	integral component of membrane;transporter activity;transmembrane transport		
XLOC_013575	TCONS_00026238	disease resistance gene nbs-lrr family protein			
XLOC_013597	TCONS_00026284	glyceraldehyde-3-phosphate dehydrogenase a	oxidoreductase activity, acting on the aldehyde or oxo group of donors, NAD or NADP as acceptor;NADP binding;NAD binding;glucose metabolic process;oxidation-reduction process		
XLOC_013645	TCONS_00026376	histone-lysine n- h3 lysine-9 specific suvh5-like	intracellular organelle;binding;methyltransferase activity;methylation		
XLOC_013968	TCONS_00026979	rab proteins geranylgeranyl transferase component a-like	cytoplasm;transferase activity;metabolic process		
XLOC_014473	TCONS_00027964	abc transporter c family member 13	integral component of membrane;ATP binding;zinc ion binding;oxidoreductase activity;ATPase activity, coupled to transmembrane movement of substances;transmembrane transport;oxidation-reduction process	ec:3.6.1.15-phosphatase;ec:3.6.1.3-adenylpyrophosphatase; ec:3.6.1	Purine metabolism;Thiamine metabolismPurine metabolism
XLOC_014537	TCONS_00028095	protein transport protein sec24-like at3g07100-like isoform x2	COPII vesicle coat;zinc ion binding;intracellular protein transport;ER to Golgi vesicle-mediated transport		
XLOC_014550	TCONS_00028115	probable apyrase 6			
XLOC_014554	TCONS_00028121	myosin-j heavy chain	myosin complex;motor activity;ATP binding;metabolic process	ec:3.6.1.15-phosphatase;ec:3.6.1	Purine metabolism;Thiamine metabolism
XLOC_014571	TCONS_00028146	rna-binding protein ylmh	RNA binding;anatomical structure morphogenesis;cellular component organization;cell development;plant epidermis development		
XLOC_014604	TCONS_00028198	hypothetical protein			
XLOC_014614	TCONS_00028221	ring u-box superfamily protein isoform 1	zinc ion binding		
XLOC_014668	TCONS_00028326	hypothetical protein PHAVU_001G035000g			

XLOC_014752	TCONS_00028478	dna-directed rna polymerases i and iii subunit rpac1	DNA binding;DNA-directed RNA polymerase activity;protein dimerization activity;transcription, DNA-templated	ec:2.7.7.6-RNA polymerase	Purine metabolism;Pyrimidine metabolism
XLOC_014782	TCONS_00028534	hypothetical protein LR48_Vigan09g116500			
XLOC_015084	TCONS_00029103	pseudo histidine-containing phosphotransfer protein 6-like			
XLOC_015315	TCONS_00029533	ankyrin repeat protein			
XLOC_015334	TCONS_00029569	leucine--trna cytoplasmic-like isoform x1	cytosol;plasmodesma;chloroplast;aminoacyl-tRNA editing activity;leucine-tRNA ligase activity;ATP binding;leucyl-tRNA aminoacylation;regulation of translational fidelity	ec:6.1.1.4-ligase;ec:6.1.1;ec:3.1.1.1-ali-esterase	Aminoacyl-tRNA biosynthesisDrug metabolism - other enzymes
XLOC_015560	TCONS_00030012	transcriptional adapter ada2	nucleus;DNA binding;zinc ion binding;regulation of transcription from RNA polymerase II promoter;histone acetylation		
XLOC_015561	TCONS_00030013	long chain acyl-synthetase 8	endoplasmic reticulum;Golgi apparatus;chloroplast envelope;long-chain fatty acid-CoA ligase activity;long-chain fatty acid metabolic process	ec:6.2.1;ec:6.2.1.3-ligase	Fatty acid degradation;Fatty acid biosynthesis
XLOC_015568	TCONS_00030020	golgin candidate 4-like			
XLOC_015572	TCONS_00030024	hypothetical protein PHAVU_001G197200g	cysteine-type peptidase activity;ubiquitinyl hydrolase activity;ubiquitin-dependent protein catabolic process	ec:3.4	
XLOC_015576	TCONS_00030028	spermidine hydroxycinnamoyl transferase-like	shikimate O-hydroxycinnamoyltransferase activity;anthranilate N-benzoyltransferase activity;metabolic process	ec:2.3.1;ec:2.3.1.144;ec:2.3.1.133-O-hydroxycinnamoyltransferase	Stilbenoid, diarylheptanoid and gingerol biosynthesis;Flavonoid biosynthesis;Phenylpropanoid biosynthesis
XLOC_015579	TCONS_00030031	hypothetical protein LR48_Vigan09g091200			
XLOC_015580	TCONS_00030032	transcription factor bhlh086	hydrolase activity;protein dimerization activity;metabolic process		
XLOC_015581	TCONS_00030033	hypothetical protein LR48_Vigan09g091200			
XLOC_015584	TCONS_00030036	hypothetical chloroplast rf68	chloroplast		
XLOC_015585	TCONS_00030037	squalene monooxygenase	plastid;integral component of membrane;cytoplasmic membrane-bounded vesicle;squalene monooxygenase activity;flavin adenine dinucleotide binding;oxidation-reduction process	ec:1.14.13;ec:1.14.13.132	
XLOC_015586	TCONS_00030038	squalene monooxygenase	integral component of membrane;squalene monooxygenase	ec:1.14.13;ec:1.14.13.132	

			activity;flavin adenine dinucleotide binding;oxidation-reduction process		
XLOC_015591	TCONS_00030043	histone-lysine n- h3 lysine-9 specific suvh6	nucleus;chromosome;zinc ion binding;histone-lysine N-methyltransferase activity;histone binding;histone lysine methylation	ec:2.1.1;ec:2.1.1.43-N-methyltransferase	Lysine degradation
XLOC_015592	TCONS_00030044	PREDICTED: uncharacterized protein LOC106753175			
XLOC_015593	TCONS_00030045	phd finger protein partial	binding		
XLOC_015594	TCONS_00030046	hypothetical protein PHAVU_003G093300g			
XLOC_015598	TCONS_00030050	jq0280 hypothetical 12k protein (trna intron) - rice chloroplast	chloroplast		
XLOC_015605	TCONS_00030057	ubiquitin-conjugating enzyme e2-17 kda			
XLOC_015613	TCONS_00030065	hypothetical protein glysoja_001754			
XLOC_015615	TCONS_00030067	atp-citrate synthase beta chain protein 1-like			
XLOC_015620	TCONS_00030072	hypothetical protein			
XLOC_015623	TCONS_00030075	wall associated kinase-like protein	kinase activity;polysaccharide binding;phosphorylation		
XLOC_015624	TCONS_00030076	wrky transcription factor 22-like	transcription factor activity, sequence-specific DNA binding;sequence-specific DNA binding;regulation of transcription, DNA-templated		
XLOC_015628	TCONS_00030080	PREDICTED: uncharacterized protein LOC102666466			
XLOC_015629	TCONS_00030081	dvl family protein			
XLOC_015631	TCONS_00030083	hypothetical protein PHAVU_010G021000g			
XLOC_015632	TCONS_00030084	at4g21420p-like protein	nucleic acid binding;RNA-DNA hybrid ribonuclease activity;transferase activity;transport;RNA phosphodiester bond hydrolysis, endonucleolytic	ec:3.1;ec:3.1.30;ec:3.1.26;ec:3.1.26.4	
XLOC_015697	TCONS_00030224	nuclease harbi1			
XLOC_015830	TCONS_00030453	hypothetical protein LR48_Vigan694s000300			

XLOC_015899	TCONS_00030569	transcription regulatory protein snf2	helicase activity;ATP binding;histone binding;metabolic process	ec:3.6.1.15-phosphatase;ec:3.6.1	Purine metabolism;Thiamine metabolism
XLOC_016025	TCONS_00030844	u1 small nuclear ribonucleoprotein a-like			
XLOC_016038	TCONS_00030872	hypothetical protein PHAVU_007G081200g			
XLOC_016042	TCONS_00030879	hypothetical protein LR48_Vigan02g201600			
XLOC_016043	TCONS_00030880	hypothetical protein LR48_Vigan02g201600			
XLOC_016045	TCONS_00030883	hypothetical protein PHAVU_007G0816001g			
XLOC_016155	TCONS_00031111	exosome complex exonuclease rrp46 homolog isoform x1	kinesin complex;microtubule;microtubule motor activity;exonuclease activity;ATP binding;microtubule binding;mRNA splicing, via spliceosome;RNA methylation;protein targeting to mitochondrion;microtubule-based movement;protein maturation;nucleic acid phosphodiester bond hydrolysis	ec:3.1;ec:3.6.1.15-phosphatase;ec:3.6.1	Purine metabolism;Thiamine metabolism
XLOC_016161	TCONS_00031125	transcriptional activator demeter-like	catalytic activity;DNA metabolic process;single-organism process		
XLOC_016234	TCONS_00031263	hypothetical protein LR48_Vigan03g138500			
XLOC_016248	TCONS_00031288	serine threonine protein phosphatase 2a regulatory subunit b beta-like	calcium ion binding		
XLOC_016390	TCONS_00031555	hypothetical protein glysoja_007957			
XLOC_016665	TCONS_00032118	hypothetical protein PHAVU_006G036900g, partial			
XLOC_016701	TCONS_00032178	polyketide cyclase dehydrase and lipid transporter			
XLOC_016713	TCONS_00032194	hypothetical protein LR48_Vigan02g146700			
XLOC_016754	TCONS_00032274	fk506-binding protein 5-like			
XLOC_017074	TCONS_00032863	dna mismatch repair protein	nuclear chromatin;mismatch repair complex;ATP binding;mismatched DNA binding;mitotic cell		

			cycle;double-strand break repair via homologous recombination;DNA-dependent DNA replication;regulation of DNA replication;mismatch repair;DNA methylation;mitotic recombination;sister chromatid cohesion;synapsis;pollen development;embryo sac egg cell differentiation;response to abscisic acid;seed germination;seed dormancy process;regulation of cell cycle process;somatic cell DNA recombination;chromatin modification;gene silencing by RNA;meiotic DNA double-strand break formation;cotyledon development		
XLOC_017187	TCONS_00033109	cysteine-rich receptor-kinase-like protein	protein serine/threonine kinase activity;non-membrane spanning protein tyrosine kinase activity;ATP binding;peptidyl-tyrosine phosphorylation	ec:2.7.11;ec:2.7.10;ec:2.7.10.2-protein-tyrosine kinase	T cell receptor signaling pathway
XLOC_017321	TCONS_00033359	cysteine-rich receptor-like protein kinase 29	protein serine/threonine kinase activity;non-membrane spanning protein tyrosine kinase activity;ATP binding;peptidyl-tyrosine phosphorylation	ec:2.7.11;ec:2.7.10;ec:2.7.10.2-protein-tyrosine kinase	T cell receptor signaling pathway
XLOC_017342	TCONS_00033413	exosome complex exonuclease rrp46 homolog isoform x1	exonuclease activity;nucleic acid phosphodiester bond hydrolysis	ec:3.1	
XLOC_017343	TCONS_00033415	cysteine-rich receptor-kinase-like protein	nucleotide binding;protein kinase activity;protein phosphorylation		
XLOC_017516	TCONS_00033752	pentatricopeptide repeat-containing protein mitochondrial	zinc ion binding;microtubule-severing ATPase activity;metabolic process	ec:3.6.1.15-phosphatase;ec:3.6.4.3;ec:3.6.1.3-adenylpyrophosphatase; ec:3.6.1	Purine metabolism;Thiamine metabolismPurine metabolism
XLOC_017565	TCONS_00033852	zinc c3hc4 type (ring finger) protein	zinc ion binding		
XLOC_017569	TCONS_00033859	abc transporter g family member 24-like	plasma membrane;ATP binding;xenobiotic-transporting ATPase activity;drug transmembrane transport;metabolic process;xenobiotic transport	ec:3.6.1.15-phosphatase;ec:3.6.1.3-adenylpyrophosphatase; ec:3.6.1;ec:3.6.3.44	Purine metabolism;Thiamine metabolismPurine metabolism
XLOC_017620	TCONS_00033960	hypothetical protein PHAVU_003G046100g			
XLOC_017637	TCONS_00033992	magnesium transporter mrs2-2-like	integral component of membrane;magnesium ion transmembrane transporter activity;magnesium ion transport;transmembrane transport		
XLOC_017674	TCONS_00034049	ftsh extracellular	plasmodesma;chloroplast envelope;metalloendopeptidase activity;ATP	ec:3.4.24	

		protease family protein	binding;proteolysis;regulation of apoptotic process		
XLOC_017794	TCONS_00034309	signal recognition particle 43 kda chloroplastic	chloroplast thylakoid membrane;chloroplast envelope;signal recognition particle, chloroplast targeting;identical protein binding;response to blue light;response to sucrose;response to red light;regulation of proton transport;response to far red light;cysteine biosynthetic process;protein import into chloroplast thylakoid membrane;protein autophosphorylation		
XLOC_017843	TCONS_00034409	nad -binding rossmann-fold protein	tropine dehydrogenase activity;oxidation-reduction process	ec:1.1.1.206-reductase I	Tropane, piperidine and pyridine alkaloid biosynthesis
XLOC_017913	TCONS_00034541	mitogen-activated protein kinase homolog mmk2-like	intracellular;MAP kinase activity;ATP binding;MAPK cascade	ec:2.7.11;ec:2.7.11.24-protein kinase	mTOR signaling pathway
XLOC_018020	TCONS_00034803	protein mon2 homolog isoform x2	protein transport		
XLOC_018037	TCONS_00034850	auxin-responsive protein iaa16-like partial			
XLOC_018176	TCONS_00035138	myb-like protein p			
XLOC_018187	TCONS_00035163	acylphosphatase family isoform 1	acylphosphatase activity;metabolic process	ec:3.6.1.7-acetylphosphatase	Aminobenzoate degradation;Pyruvate metabolism
XLOC_018249	TCONS_00035272	PREDICTED: uncharacterized protein LOC100789825 isoform X2	chloroplast;hydrolase activity;lipid metabolic process		
XLOC_018489	TCONS_00035755	hypothetical protein PHAVU_004G124900g			
XLOC_018494	TCONS_00035760	hypothetical protein glysoja_015948			
XLOC_018498	TCONS_00035764	hypothetical protein PHAVU_007G081200g			
XLOC_018501	TCONS_00035767	hypothetical protein M569_00483, partial			
XLOC_018507	TCONS_00035773	atp synthase cf0 subunit i	plasma membrane;chloroplast thylakoid membrane;integral component of membrane;proton-transporting ATP synthase complex, coupling factor F(o);ATP binding;proton-transporting ATP synthase activity, rotational mechanism;ATP synthesis coupled proton transport	ec:3.6.1.15-phosphatase;ec:3.6.1.3-adenylpyrophosphatase;ec:3.6.1	Purine metabolism;Thiamine metabolismPurine metabolism
XLOC_018510	TCONS_00035776	hypothetical chloroplast rf1	chloroplast inner membrane;integral component of membrane;protein transport		

XLOC_018516	TCONS_00035782	endonuclease exonuclease phosphatase family protein			
XLOC_018517	TCONS_00035783	hypothetical protein PHAVU_002G227000g			
XLOC_018521	TCONS_00035787	hypothetical protein PHAVU_001G121300g			
XLOC_018523	TCONS_00035789	histone deacetylase 6	oxidoreductase activity;hydrolase activity;cellular protein metabolic process;single-organism metabolic process		
XLOC_018527	TCONS_00035793	hypothetical protein PHAVU_006G007700g			
XLOC_018528	TCONS_00035794	nadh-plastoquinone oxidoreductase subunit 2	chloroplast thylakoid membrane;integral component of membrane;NADH dehydrogenase (ubiquinone) activity;quinone binding;transport;photosynthesis, light reaction;ATP synthesis coupled electron transport	ec:1.6.99.5;ec:1.6.99.3-dehydrogenase;ec:1.6.5.3-reductase (H+-translocating)	Oxidative phosphorylationOxidative phosphorylation
XLOC_018529	TCONS_00035795	maturase	mitochondrion;chloroplast;RNA binding;mRNA processing;tRNA processing;RNA splicing		
XLOC_018532	TCONS_00035798	PREDICTED: uncharacterized protein LOC102660158			
XLOC_018533	TCONS_00035799	hypothetical protein PHAVU_003G069400g			
XLOC_018543	TCONS_00035809	dead-box atp-dependent rna helicase 7	nucleus;RNA binding;helicase activity;ATP binding;metabolic process	ec:3.6.1.15-phosphatase;ec:3.6.1	Purine metabolism;Thiamine metabolism
XLOC_018545	TCONS_00035811	uncharacterized mitochondrial protein g00810-like	catalytic activity		
XLOC_018550	TCONS_00035816	transmembrane protein, putative			
XLOC_018554	TCONS_00035820	PREDICTED: uncharacterized protein LOC102664214	nucleic acid binding;protein dimerization activity		
XLOC_018555	TCONS_00035821	hypothetical protein PHAVU_004G0453000g, partial	DNA binding		
XLOC_018561	TCONS_00035827	photosystem ii d1 partial	chloroplast;photosystem II;integral component of membrane;oxidoreductase activity;electron transporter, transferring electrons within the cyclic electron transport pathway of photosynthesis activity;metal ion binding;photosynthetic electron transport in photosystem II		

XLOC_018563	TCONS_00035829	PREDICTED: uncharacterized protein LOC105036210			
XLOC_018564	TCONS_00035830	calmodulin-binding family protein			
XLOC_018707	TCONS_00036123	serine-threonine kinase	protein kinase activity;ATP binding;protein phosphorylation		
XLOC_018947	TCONS_00036541	agamous-like 20 isoform partial	nucleus;cytoplasm;DNA binding;transcription factor activity, sequence-specific DNA binding;transcription factor binding;protein dimerization activity;protein import into nucleus, translocation;transcription, DNA-templated;regulation of transcription, DNA-templated;positive regulation of flower development;maintenance of inflorescence meristem identity		
XLOC_018960	TCONS_00036570	hypothetical protein PHAVU_008G075700g			
XLOC_018973	TCONS_00036607	probable lrr receptor-like serine threonine-protein kinase rfk1 isoform x2	protein kinase activity;phosphorylation		
XLOC_019070	TCONS_00036784	aaa-type atpase family protein	intracellular;ATP binding;microtubule-severing ATPase activity;fatty acid beta-oxidation;protein import into peroxisome matrix	ec:3.6.1.15-phosphatase;ec:3.6.4.3;ec:3.6.1.3-adenylpyrophosphatase; ec:3.6.1	Purine metabolism;Thiamine metabolismPurine metabolism
XLOC_019116	TCONS_00036870	hypothetical protein PHAVU_001G133800g			
XLOC_019216	TCONS_00037034	hypothetical protein PHAVU_L006100g	DNA binding;zinc ion binding		
XLOC_019264	TCONS_00037126	wound-induced basic protein	cytosol;response to cytokinin		
XLOC_019320	TCONS_00037227	unnamed protein product	ATP-dependent DNA helicase activity;ATP binding;DNA repair;chromatin remodeling;DNA duplex unwinding	ec:3.6.1.15-phosphatase;ec:3.6.1.3-adenylpyrophosphatase; ec:3.6.1	Purine metabolism;Thiamine metabolismPurine metabolism
XLOC_019344	TCONS_00037265	gata transcription factor 7-like	binding		
XLOC_019362	TCONS_00037292	glutamate receptor 2	integral component of membrane;ionotropic glutamate receptor activity;ion transport;ionotropic glutamate receptor signaling pathway		
XLOC_019386	TCONS_00037333	iron-sulfur cluster biosynthesis family protein	chloroplast envelope		

XLOC_019388	TCONS_00037335	nucleic acid-ob-fold-like protein			
XLOC_019446	TCONS_00037436	hypothetical protein VIGAN_03165800, partial			
XLOC_019472	TCONS_00037481	sugar transporter	integral component of membrane;substrate-specific transmembrane transporter activity;2-alkenal reductase [NAD(P)] activity;carbohydrate transport;transmembrane transport;oxidation-reduction process	ec:1.3.1.74	
XLOC_019541	TCONS_00037605	hypothetical protein PHAVU_005G084700g			
XLOC_019643	TCONS_00037802	hypothetical protein LR48_Vigan05g133500			
XLOC_020164	TCONS_00038830	ankyrin repeat and sam domain-containing protein 3-like			
XLOC_020297	TCONS_00039100	protein transport protein sec24-1	COPII vesicle coat;zinc ion binding;intracellular protein transport;ER to Golgi vesicle-mediated transport		
XLOC_020421	TCONS_00039396	phytochrome a-associated f-box protein	organic cyclic compound binding;heterocyclic compound binding;cellular protein metabolic process;system development;regulation of biological process		
XLOC_020515	TCONS_00039576	hypothetical protein PHAVU_003G177200g			
XLOC_020535	TCONS_00039627	acyl-coenzyme a oxidase peroxisomal	oxidoreductase activity		
XLOC_020664	TCONS_00039870	hedgehog isoform 2	plasma membrane;plasmodesma;integral component of membrane;hedgehog receptor activity;signal transduction;cholesterol transport		
XLOC_020667	TCONS_00039876	transcription factor hy5-like			
XLOC_020674	TCONS_00039893	g-type lectin s-receptor-like serine threonine-kinase	protein serine/threonine kinase activity;ATP binding;carbohydrate binding;protein phosphorylation;recognition of pollen	ec:2.7.11	
XLOC_020681	TCONS_00039909	dna-directed rna polymerase iii subunit rpc3	DNA binding;DNA-directed RNA polymerase activity;transcription, DNA-templated	ec:2.7.7.6-RNA polymerase	Purine metabolism;Pyrimidine metabolism
XLOC_020748	TCONS_00040036	protein transport protein sec24	COPII vesicle coat;zinc ion binding;intracellular protein transport;ER to Golgi vesicle-mediated transport		
XLOC_020761	TCONS_00040054	u6 snrna phosphodiesterase isoform x3	nucleus;nuclease activity;mRNA processing;RNA splicing;U6 snRNA 3'-end processing;nucleic acid	ec:3.1	

			phosphodiester bond hydrolysis		
XLOC_020771	TCONS_00040067	hypothetical protein PHAVU_002G203500g			
XLOC_020794	TCONS_00040116	u4 u6 small nuclear ribonucleoprotein prp31	viral nucleocapsid;U4/U6 x U5 tri-snRNP complex;spliceosomal tri-snRNP complex assembly		
XLOC_020796	TCONS_00040120	actin-related protein 2	Arp2/3 protein complex;ATP binding;multidimensional cell growth;trichome morphogenesis;Arp2/3 complex-mediated actin nucleation		
XLOC_020797	TCONS_00040123	serine hydroxymethyltransferase	transferase activity, transferring one-carbon groups;organic cyclic compound binding;heterocyclic compound binding;one-carbon metabolic process;serine family amino acid metabolic process		
XLOC_020802	TCONS_00040133	glutamate receptor 2	integral component of membrane;ionotropic glutamate receptor activity;ion transport;ionotropic glutamate receptor signaling pathway		
XLOC_020820	TCONS_00040168	trehalose-phosphate phosphatase	trehalose-phosphatase activity;trehalose biosynthetic process;protein dephosphorylation	ec:3.1.3.41-nitrophenyl phosphatase;ec:3.1;ec:3.1.3.12-trehalose 6-phosphatase	Aminobenzoate degradationStarch and sucrose metabolism
XLOC_020838	TCONS_00040209	translation elongation factor ef protein	intracellular;translation elongation factor activity;GTPase activity;GTP binding;translational elongation	ec:3.6.1.15-phosphatase;ec:3.6.1	Purine metabolism;Thiamine metabolism
XLOC_020857	TCONS_00040237	btb poz domain-containing protein at1g21780-like	cytoplasm;Cul3-RING ubiquitin ligase complex;protein binding;fatty acid beta-oxidation;intra-Golgi vesicle-mediated transport;proximal/distal pattern formation;floral organ abscission;protein import into peroxisome matrix;protein ubiquitination;flower morphogenesis		
XLOC_020863	TCONS_00040257	mads-box transcription factor 50	nucleus;DNA binding;metal ion binding;protein dimerization activity;transcription, DNA-templated;regulation of transcription, DNA-templated		
XLOC_020934	TCONS_00040382	hypothetical protein LR48_Vigan46s000800			
XLOC_021014	TCONS_00040540	ribonuclease e	RNA binding;ribonuclease activity;starch binding;RNA processing;RNA phosphodiester bond hydrolysis	ec:3.1	
XLOC_021047	TCONS_00040620	disease resistance	ADP binding;signal transduction		

		protein (tir-nbs-lrr class)			
XLOC_021310	TCONS_00041124	two-component response regulator arr2-like protein	intracellular;DNA binding;phosphorelay signal transduction system		
XLOC_021548	TCONS_00041550	rna polymerase beta subunit	plastid;nucleotidyltransferase activity		
XLOC_021549	TCONS_00041551	spfh domain band 7 family protein	nucleic acid binding;zinc ion binding;DNA integration		
XLOC_021551	TCONS_00041553	hypothetical protein PHAVU_004G108700g			
XLOC_021554	TCONS_00041556	40s ribosomal protein s2-4-like	small ribosomal subunit;RNA binding;structural constituent of ribosome;translation		
XLOC_021561	TCONS_00041563	downstream target of agl15-4	protein kinase activity;ATP binding;protein phosphorylation		
XLOC_021562	TCONS_00041564	protein lurp-one-related 15			
XLOC_021565	TCONS_00041567	hypothetical protein PHAVU_011G106500g			
XLOC_021566	TCONS_00041568	protein lurp-one-related 15-like			
XLOC_021567	TCONS_00041569	hypothetical protein LR48_Vigan04g240900			
XLOC_021572	TCONS_00041574	hypothetical protein VITISV_008145			
XLOC_021574	TCONS_00041576	hypothetical protein PHAVU_L006100g			
XLOC_021575	TCONS_00041577	hypothetical protein PHAVU_004G088700g			
XLOC_021578	TCONS_00041580	rna polymerase beta partial	chloroplast;DNA binding;DNA-directed RNA polymerase activity;transcription, DNA-templated	ec:2.7.7.6-RNA polymerase	Purine metabolism;Pyrimidine metabolism
XLOC_021579	TCONS_00041581	gdsl esterase lipase cprd49	hydrolase activity, acting on ester bonds;vegetative to reproductive phase transition of meristem;protein desumoylation;hydrogen peroxide biosynthetic process		
XLOC_021580	TCONS_00041582	atp synthase cf1 alpha partial	mitochondrial inner membrane;plasma membrane;chloroplast thylakoid membrane;proton-transporting ATP synthase complex, catalytic core F(1);ATP binding;proton-transporting ATP synthase activity, rotational mechanism;proton-transporting ATPase activity, rotational mechanism;ATP synthesis coupled proton	ec:3.6.1.15-phosphatase;ec:3.6.1.3-adenylpyrophosphatase;ec:3.6.1	Purine metabolism;Thiamine metabolismPurine metabolism

			transport;ATP hydrolysis coupled proton transport		
XLOC_021582	TCONS_00041584	hypothetical protein LR48_Vigan03g146600			
XLOC_021584	TCONS_00041586	ribosomal protein l16	mitochondrion;ribosome;chloroplast;structural constituent of ribosome;rRNA binding;translation		
XLOC_021585	TCONS_00041587	beta-glucosidase 11	beta-glucosidase activity;carbohydrate metabolic process	ec:3.2.1.21-gentiobiase; ec:3.2.1	Cyanoamino acid metabolism;Phenylpropanoid biosynthesis;Starch and sucrose metabolism
XLOC_021586	TCONS_00041588	hypothetical protein LR48_Vigan583s003200			
XLOC_021588	TCONS_00041590	cytochrome p450 family protein	monooxygenase activity;iron ion binding;oxidoreductase activity, acting on paired donors, with incorporation or reduction of molecular oxygen;heme binding;oxidation-reduction process		
XLOC_021592	TCONS_00041594	ribulose- -bisphosphate carboxylase oxygenase large partial	chloroplast;magnesium ion binding;monooxygenase activity;ribulose-bisphosphate carboxylase activity;reductive pentose-phosphate cycle;oxidation-reduction process	ec:4.1.1.39-carboxylase	Carbon fixation in photosynthetic organisms;Biosynthesis of antibiotics;Glyoxylate and dicarboxylate metabolism
XLOC_021593	TCONS_00041595	hypothetical protein PHAVU_010G029600g	ATP binding;ADP binding;signal transduction		
XLOC_021595	TCONS_00041597	hypothetical protein Csa_2G055570	mitochondrion;chloroplast		
XLOC_021596	TCONS_00041598	hypothetical protein PHAVU_001G0336001g, partial	DNA binding		
XLOC_021597	TCONS_00041599	integrase core domain containing protein			
XLOC_021598	TCONS_00041600	hypothetical protein PHAVU_009G162400g			
XLOC_021599	TCONS_00041601	rna polymerase beta subunit	chloroplast;DNA binding;DNA-directed RNA polymerase activity;transcription, DNA-templated	ec:2.7.7.6-RNA polymerase	Purine metabolism;Pyrimidine metabolism
XLOC_021601	TCONS_00041603	cysteine-rich rlk (receptor-like protein kinase) 8	binding		
XLOC_021603	TCONS_00041605	plant t8m16-150			
XLOC_021607	TCONS_00041609	pentatricopeptide repeat-containing protein at2g06000	microtubule-severing ATPase activity;metabolic process	ec:3.6.1.15-phosphatase;ec:3.6.4.3;ec:3.6.1.3-adenylpyrophosphatase; ec:3.6.1	Purine metabolism;Thiamine metabolismPurine metabolism
XLOC_021608	TCONS_00041610	hypothetical protein LR48_Vigan97s002000			

XLOC_021609	TCONS_00041611	hypothetical protein PHAVU_011G140600g	DNA binding		
XLOC_021612	TCONS_00041614	photosystem ii protein partial	chloroplast;electron transporter, transferring electrons within the cyclic electron transport pathway of photosynthesis activity;photosynthetic electron transport in photosystem II		
XLOC_021618	TCONS_00041620	ubiquitin ligase sinat3	nucleus;ubiquitin-protein transferase activity;zinc ion binding;ligase activity;ubiquitin-dependent protein catabolic process;multicellular organismal development;protein ubiquitination		
XLOC_021619	TCONS_00041621	integrase core domain containing protein			
XLOC_021624	TCONS_00041626	exocyst complex component exo70b1-like	exocyst;exocytosis		
XLOC_021625	TCONS_00041627	exocyst complex component exo70b1-like	exocyst;exocytosis		
XLOC_021627	TCONS_00041629	hypothetical protein LR48_Vigan07g170500			
XLOC_021628	TCONS_00041630	cytochrome f	chloroplast thylakoid membrane;integral component of thylakoid membrane;iron ion binding;electron carrier activity;heme binding;photosynthesis;oxidation-reduction process		
XLOC_021633	TCONS_00041635	atp synthase cf0 subunit i	plasma membrane;chloroplast thylakoid membrane;integral component of membrane;proton-transporting ATP synthase complex, coupling factor F(o);ATP binding;proton-transporting ATP synthase activity, rotational mechanism;ATP synthesis coupled proton transport	ec:3.6.1.15-phosphatase;ec:3.6.1.3-adenylpyrophosphatase;ec:3.6.1	Purine metabolism;Thiamine metabolismPurine metabolism
XLOC_021634	TCONS_00041636	endonuclease exonuclease phosphatase family protein	catalytic activity;cellular metabolic process		
XLOC_021636	TCONS_00041638	rna polymerase beta subunit	chloroplast;DNA binding;DNA-directed RNA polymerase activity;transcription, DNA-templated	ec:2.7.7.6-RNA polymerase	Purine metabolism;Pyrimidine metabolism
XLOC_021642	TCONS_00041644	gag-pol polyprotein			
XLOC_021645	TCONS_00041647	hypothetical protein GLYMA_06G042000			
XLOC_021647	TCONS_00041649	atp synthase cf1 alpha subunit			

XLOC_021649	TCONS_00041651	hypothetical protein VIGAN_03082100			
XLOC_021664	TCONS_00041666	multidrug resistance protein abc transporter family protein	integral component of membrane;ATP binding;ATPase activity, coupled to transmembrane movement of substances;metabolic process;transmembrane transport	ec:3.6.1.15-phosphatase;ec:3.6.1.3-adenylpyrophosphatase; ec:3.6.1	Purine metabolism;Thiamine metabolismPurine metabolism
XLOC_021724	TCONS_00041758	folate transporter carrier-like protein			
XLOC_021753	TCONS_00041810	guanine nucleotide-binding protein subunit beta-like protein 1 homolog	nucleus;cytoplasm;Cul4-RING E3 ubiquitin ligase complex;regulation of DNA repair;cellular response to DNA damage stimulus;pollen development;plasmodesma organization;embryo development ending in seed dormancy;response to aluminum ion;plasmodesmata-mediated intercellular transport;DNA integrity checkpoint;ovule development;acquisition of desiccation tolerance in seed;positive regulation of cell cycle arrest;response to cisplatin;regulation of root development		
XLOC_021797	TCONS_00041911	duf241 domain protein			
XLOC_022009	TCONS_00042380	cell wall invertase 2 isoform 2	sucrose alpha-glucosidase activity;carbohydrate metabolic process	ec:3.2.1.48-alpha-glucosidase; ec:3.2.1.26-invertase;ec:3.2.1	Starch and sucrose metabolismGalactose metabolism;Starch and sucrose metabolism
XLOC_022070	TCONS_00042500	ferrochelatase - chloroplastic	plastid;ferrochelatase activity;heme biosynthetic process;response to stress;macromolecule localization;regulation of programmed cell death	ec:4.99.1.1-ferro-protoporphyrin chelatase	Porphyrin and chlorophyll metabolism
XLOC_022312	TCONS_00043004	pentatricopeptide repeat-containing protein at4g17915			
XLOC_022425	TCONS_00043219	zinc finger (c3hc4-type ring finger) family protein	plasma membrane;zinc ion binding		
XLOC_022543	TCONS_00043496	4-coumarate--ligase-like 9	ligase activity;Photinus-luciferin 4-monooxygenase (ATP-hydrolyzing) activity;oxidation-reduction process	ec:1.13.12;ec:1.13.12.7	
XLOC_022572	TCONS_00043545	c2 and gram domain-containing protein at1g03370-like	lyase activity;metabolic process		
XLOC_022666	TCONS_00043755	mlo-like protein 10	integral component of membrane;calmodulin binding;defense response;response to biotic stimulus		

XLOC_022719	TCONS_00043846	lipase rog1 isoform x3			
XLOC_022735	TCONS_00043870	pentatricopeptide repeat-containing protein at5g55840	microtubule-severing ATPase activity;metabolic process	ec:3.6.1.15-phosphatase;ec:3.6.4.3;ec:3.6.1.3-adenylpyrophosphatase; ec:3.6.1	Purine metabolism;Thiamine metabolismPurine metabolism
XLOC_023069	TCONS_00044551	pentatricopeptide repeat-containing protein at2g13600-like isoform x1	microtubule-severing ATPase activity;metabolic process	ec:3.6.1.15-phosphatase;ec:3.6.4.3;ec:3.6.1.3-adenylpyrophosphatase; ec:3.6.1	Purine metabolism;Thiamine metabolismPurine metabolism
XLOC_023127	TCONS_00044665	duf241 domain protein			
XLOC_023140	TCONS_00044682	protein transport protein sec24-1	COPII vesicle coat;zinc ion binding;intracellular protein transport;ER to Golgi vesicle-mediated transport		
XLOC_023375	TCONS_00045188	chromosome region maintenance protein 1 exportin	intracellular;Ran GTPase binding;intracellular protein transport		
XLOC_023440	TCONS_00045306	hypothetical protein VIGAN_02068400			
XLOC_023474	TCONS_00045372	protein topless-like isoform x1	regulation of transcription, DNA-templated		
XLOC_023475	TCONS_00045373	topless-like protein	regulation of transcription, DNA-templated		
XLOC_023610	TCONS_00045657	protein far1-related sequence 2	zinc ion binding;regulation of transcription, DNA-templated		
XLOC_023611	TCONS_00045659	protein far1-related sequence 2	zinc ion binding;regulation of transcription, DNA-templated		
XLOC_023648	TCONS_00045724	hypothetical protein PHAVU_009G125600g			
XLOC_023693	TCONS_00045812	hat transposon superfamily isoform 2	DNA binding;protein dimerization activity		
XLOC_023694	TCONS_00045813	myosin id heavy chain-like protein			
XLOC_023743	TCONS_00045924	hypothetical protein PHAVU_009G156200g			
XLOC_023868	TCONS_00046178	protein dehydration-induced 19 homolog 6-like isoform x2			
XLOC_023878	TCONS_00046192	c2 and gram domain-containing protein	phosphatidylserine decarboxylase activity;protein kinase C activity;ligase activity;protein phosphorylation	ec:2.7.11;ec:4.1.1.65-decarboxylase;ec:2.7.11.13-kinase C	Glycerophospholipid metabolismPhosphatidylinositol signaling system
XLOC_023887	TCONS_00046208	serine threonine protein phosphatase 2a 55 kda regulatory	regulation of cellular process		

		subunit b beta isoform-like isoform x2			
XLOC_023896	TCONS_00046231	peroxisomal membrane protein pex14	peroxisomal membrane;protein import into peroxisome matrix, docking		
XLOC_024006	TCONS_00046452	protein lurp-one-related 6-like			
XLOC_024211	TCONS_00046876	upf0047 protein	chloroplast		
XLOC_024320	TCONS_00047063	photosystem i p700 apoprotein a2	photosystem I;chloroplast thylakoid membrane;integral component of membrane;magnesium ion binding;electron carrier activity;chlorophyll binding;oxidoreductase activity;4 iron, 4 sulfur cluster binding;photosynthesis;protein-chromophore linkage;oxidation-reduction process		
XLOC_024322	TCONS_00047065	ribosomal protein s15	ribosome;chloroplast;structural constituent of ribosome;translation		
XLOC_024323	TCONS_00047066	nadh-plastoquinone oxidoreductase subunit i	plasma membrane;chloroplast thylakoid membrane;iron ion binding;NADH dehydrogenase (ubiquinone) activity;quinone binding;4 iron, 4 sulfur cluster binding;photosynthesis, light reaction;oxidation-reduction process	ec:1.6.99.5;ec:1.6.99.3-dehydrogenase;ec:1.6.5.3-reductase (H+-translocating)	Oxidative phosphorylationOxidative phosphorylation
XLOC_024324	TCONS_00047067	rna polymerase beta subunit	chloroplast;DNA binding;DNA-directed RNA polymerase activity;ribonucleoside binding;transcription, DNA-templated	ec:2.7.7.6-RNA polymerase	Purine metabolism;Pyrimidine metabolism
XLOC_024325	TCONS_00047068	hypothetical protein MTR_3g073490			
XLOC_024332	TCONS_00047075	calmodulin-binding transcription activator 2-like	nucleus;DNA binding;response to cold;leaf senescence;defense response to bacterium;defense response to fungus		
XLOC_024334	TCONS_00047077	nadh-plastoquinone oxidoreductase subunit 4	chloroplast thylakoid membrane;integral component of membrane;NADH dehydrogenase (ubiquinone) activity;quinone binding;ATP synthesis coupled electron transport	ec:1.6.99.5;ec:1.6.99.3-dehydrogenase;ec:1.6.5.3-reductase (H+-translocating)	Oxidative phosphorylationOxidative phosphorylation
XLOC_024338	TCONS_00047081	pentatricopeptide repeat-containing protein at5g14080			
XLOC_024346	TCONS_00047089	hypothetical protein VITISV_005281			
XLOC_024348	TCONS_00047091	atp synthase mitochondrial f1 complex assembly factor 2	proton-transporting ATP synthase complex assembly		

XLOC_024351	TCONS_00047094	hypothetical protein LR48_Vigan561s006800			
XLOC_024359	TCONS_00047102	hypothetical protein PHAVU_005G103000g, partial	nucleotide binding;nucleic acid binding		
XLOC_024360	TCONS_00047103	hypothetical protein PHAVU_008G1333001g, partial			
XLOC_024363	TCONS_00047106	nad-dependent epimerase dehydratase family protein	mitochondrion;chloroplast thylakoid;translation initiation factor activity;rRNA processing;translational initiation;response to blue light;response to red light;photosystem II assembly;response to far red light;carotenoid biosynthetic process;isopentenyl diphosphate biosynthetic process, methylerythritol 4-phosphate pathway		
XLOC_024367	TCONS_00047110	replication factor c subunit 4	DNA replication factor C complex;nucleolus;DNA clamp loader activity;DNA-directed DNA polymerase activity;ATP binding;RNA methylation;DNA-dependent DNA replication;pyrimidine ribonucleotide biosynthetic process;embryo development ending in seed dormancy;regulation of flower development;histone modification;floral organ formation;DNA biosynthetic process	ec:3.6.1.15-phosphatase;ec:2.7.7.7-DNA polymerase;ec:3.6.1.3-adenylpyrophosphatase;ec:3.6.1	Purine metabolism;Thiamine metabolismPurine metabolism;Pyrimidine metabolismPurine metabolism
XLOC_024370	TCONS_00047113	hypothetical protein glysoja_046184			
XLOC_024377	TCONS_00047120	atp synthase cf0 subunit i	plasma membrane;chloroplast thylakoid membrane;integral component of membrane;proton-transporting ATP synthase complex, coupling factor F(o);ATP binding;proton-transporting ATP synthase activity, rotational mechanism;ATP synthesis coupled proton transport	ec:3.6.1.15-phosphatase;ec:3.6.1.3-adenylpyrophosphatase;ec:3.6.1	Purine metabolism;Thiamine metabolismPurine metabolism
XLOC_024379	TCONS_00047122	2-oxoisovalerate dehydrogenase subunit alpha mitochondrial-like	3-methyl-2-oxobutanoate dehydrogenase (2-methylpropanoyl-transferring) activity;oxidation-reduction process	ec:1.2.4.4-dehydrogenase (2-methylpropanoyl-transferring)	Propanoate metabolism;Valine, leucine and isoleucine degradation;Biosynthesis of antibiotics
XLOC_024380	TCONS_00047123	magnesium transporter nipa2	integral component of membrane;magnesium ion transmembrane transporter activity;magnesium ion transport		
XLOC_024385	TCONS_00047128	photosystem ii cp43 chlorophyll partial	photosystem II;chloroplast thylakoid membrane;integral component of membrane;chlorophyll binding;electron transporter, transferring electrons within the cyclic electron transport		

			pathway of photosynthesis activity;metal ion binding;photosynthetic electron transport in photosystem II;protein-chromophore linkage		
XLOC_024386	TCONS_00047129	hypothetical protein PHAVU_003G200800g			
XLOC_024392	TCONS_00047135	double-stranded rna-binding motif protein	double-stranded RNA binding		
XLOC_024394	TCONS_00047137	protein chloroplastic			
XLOC_024396	TCONS_00047139	envelope membrane protein	chloroplast inner membrane;integral component of membrane;hydrogen ion transmembrane transporter activity;hydrogen ion transmembrane transport		
XLOC_024398	TCONS_00047141	protein dennd6a			
XLOC_024400	TCONS_00047143	atp synthase cf1 alpha subunit	mitochondrial inner membrane;plasma membrane;chloroplast thylakoid membrane;proton-transporting ATP synthase complex, catalytic core F(1);ATP binding;proton-transporting ATP synthase activity, rotational mechanism;proton-transporting ATPase activity, rotational mechanism;ATP synthesis coupled proton transport;ATP hydrolysis coupled proton transport	ec:3.6.1.15-phosphatase;ec:3.6.1.3-adenylpyrophosphatase;ec:3.6.1	Purine metabolism;Thiamine metabolismPurine metabolism
XLOC_024402	TCONS_00047145	hypothetical protein LR48_Vigan01g305800			
XLOC_024405	TCONS_00047148	endonuclease exonuclease phosphatase family protein			
XLOC_024409	TCONS_00047152	nuclease harbi1			
XLOC_024413	TCONS_00047156	cysteine-rich rlk (receptor-like protein kinase) 8	cytoskeleton;protein complex;kinase activity;purine ribonucleoside binding;purine ribonucleotide binding;purine ribonucleoside triphosphate binding;phosphorylation;primary metabolic process;cellular macromolecule metabolic process		
XLOC_024416	TCONS_00047159	hypothetical protein PHAVU_006G105700g	nucleic acid binding		
XLOC_024418	TCONS_00047161	hypothetical protein PHAVU_005G063000g			
XLOC_024425	TCONS_00047178	vacuolar cation proton exchanger 3	membrane;cation transmembrane transporter activity;calcium ion transport;cation transmembrane transport		

XLOC_024477	TCONS_00047261	ribonuclease chloroplastic mitochondrial	mitochondrion;chloroplast;3'-5'-exoribonuclease activity;exoribonuclease II activity;rRNA processing;tRNA metabolic process;chloroplast relocation;thylakoid membrane organization;RNA phosphodiester bond hydrolysis, exonucleolytic	ec:3.1.15;ec:3.1.13;ec:3.1;ec:3.1.13.1	
XLOC_024497	TCONS_00047288	unknown			
XLOC_024539	TCONS_00047350	nbs-lrr protein	ADP binding		
XLOC_024553	TCONS_00047373	tmv resistance protein n-like isoform x1	ADP binding;signal transduction		
XLOC_024557	TCONS_00047384	hypothetical protein PHAVU_010G025500g	ATP binding;ADP binding;signal transduction		
XLOC_024765	TCONS_00047744	alpha beta fold hydrolase	chloroplast;hydrolase activity;metabolic process		
XLOC_024793	TCONS_00047785	serine acetyltransferase 5-like	cytosol;magnesium ion binding;serine O-acetyltransferase activity;terpene synthase activity;cysteine biosynthetic process from serine;protein targeting to membrane;response to wounding;response to fungus;jasmonic acid biosynthetic process;salicylic acid mediated signaling pathway;jasmonic acid mediated signaling pathway;regulation of plant-type hypersensitive response	ec:2.3.1;ec:2.3.1.30-O-acetyltransferase	Sulfur metabolism;Biosynthesis of antibiotics;Cysteine and methionine metabolism
XLOC_024795	TCONS_00047791	condensin complex subunit 1	mitotic nuclear division;chromosome condensation		
XLOC_024823	TCONS_00047830	hypothetical protein PHAVU_006G010500g, partial			
XLOC_024833	TCONS_00047843	mate efflux family protein	integral component of membrane;serine-type endopeptidase activity;drug transmembrane transporter activity;antiporter activity;proteolysis;drug transmembrane transport	ec:3.4.21	
XLOC_024853	TCONS_00047897	hypothetical protein PHAVU_006G035700g	zinc ion binding		
XLOC_024866	TCONS_00047929	acyl-activating enzyme 19	ligase activity;cell-cell signaling;virus induced gene silencing;production of ta-siRNAs involved in RNA interference;production of miRNAs involved in gene silencing by miRNA		
XLOC_024896	TCONS_00047978	phosphoprotein phosphatase	ADP binding		
XLOC_024922	TCONS_00048028	probable lrr receptor-like serine threonine-protein kinase rfk1 isoform x2	protein serine/threonine kinase activity;transmembrane receptor protein tyrosine kinase activity;ATP binding;peptidyl-tyrosine phosphorylation	ec:2.7.11;ec:2.7.10;ec:2.7.10.1	
XLOC_024949	TCONS_00048081	actin family protein	ATP binding		

XLOC_024966	TCONS_00048111	pleiotropic drug resistance protein 1-like	integral component of membrane;ATP binding;heme-transporting ATPase activity;metabolic process;heme transport	ec:3.6.3.41;ec:3.6.1.15-phosphatase;ec:3.6.1.3-adenylpyrophosphatase; ec:3.6.1	Purine metabolism;Thiamine metabolismPurine metabolism
XLOC_025065	TCONS_00048297	protein lhy	DNA binding		
XLOC_025316	TCONS_00048789	protein far1-related sequence partial	methylmalonate-semialdehyde dehydrogenase (acylating) activity;hydrolase activity, acting on glycosyl bonds;regulation of transcription, DNA-templated;oxidation-reduction process	ec:1.2.1.27-dehydrogenase (CoA-acylating)	Propanoate metabolism;Valine, leucine and isoleucine degradation
XLOC_025408	TCONS_00048984	hypothetical protein PHAVU_010G019200g, partial			
XLOC_025463	TCONS_00049099	hypothetical protein PHAVU_010G0291001g, partial	ATP binding;ADP binding;signal transduction		
XLOC_025544	TCONS_00049247	patatin group a-3	lipid metabolic process		
XLOC_025545	TCONS_00049248	patatin-like protein 2	hydrolase activity;lipid catabolic process		
XLOC_025618	TCONS_00049386	alpha-l-fucosidase 2	apoplast;1,2-alpha-L-fucosidase activity;metabolic process	ec:3.2.1.51-alpha-fucosidase;ec:3.2.1;ec:3.2.1.63	Other glycan degradation
XLOC_025660	TCONS_00049465	nucleotidyltransferase family protein isoform 4	nucleotidyltransferase activity;metabolic process		
XLOC_025687	TCONS_00049517	acyl- oxidase	acyl-CoA dehydrogenase activity;acyl-CoA oxidase activity;flavin adenine dinucleotide binding;oxidation-reduction process	ec:1.3.99.3;ec:1.3.3.6-oxidase	Fatty acid degradation;Biosynthesis of unsaturated fatty acids;alpha-Linolenic acid metabolism
XLOC_025693	TCONS_00049524	4-coumarate--ligase-like 10	plasmodesma;chloroplast stroma;apoplast;o-succinylbenzoate-CoA ligase activity;oxalate-CoA ligase activity;positive regulation of seed germination;response to nitrate;seed coat development;nitrate transport;oxalate catabolic process;para-aminobenzoic acid metabolic process;defense response to fungus	ec:6.2.1.8-ligase;ec:6.2.1.26-ligase	Glyoxylate and dicarboxylate metabolismUbiquinone and other terpenoid-quinone biosynthesis
XLOC_025694	TCONS_00049525	histone-lysine n-methyltransferase eza1-like isoform x1	PcG protein complex;histone-lysine N-methyltransferase activity;histone lysine methylation	ec:2.1.1;ec:2.1.1.43-N-methyltransferase	Lysine degradation
XLOC_025714	TCONS_00049559	mate efflux family protein	integral component of membrane;drug transmembrane transporter activity;antiporter activity;drug transmembrane transport		
XLOC_025729	TCONS_00049586	exocyst complex component sec6-like	exocyst;extracellular region;cell wall;cytosol;cytoskeleton;plasma membrane;plasmodesma;phragmoplast;SNARE		

			binding;exocytosis;pollen germination;pollen tube growth;exocyst localization		
XLOC_025787	TCONS_00049690	pentatricopeptide repeat-containing protein at2g17140			
XLOC_025825	TCONS_00049782	probable polygalacturonase	extracellular region;polygalacturonase activity;carbohydrate metabolic process;cell wall organization	ec:3.2.1.15-pectin depolymerase	Pentose and glucuronate interconversions;Starch and sucrose metabolism
XLOC_025855	TCONS_00049833	hypothetical protein PHAVU_003G068600g			
XLOC_025931	TCONS_00049965	autophagy-related protein 18b isoform x3			
XLOC_025972	TCONS_00050043	disease resistance protein (tir-nbs-lrr class)	signal transduction		
XLOC_025978	TCONS_00050060	disease resistance protein (tir-nbs-lrr class)	ADP binding;signal transduction		
XLOC_026078	TCONS_00050258	mediator of rna polymerase ii transcription subunit 15a-like	transcription cofactor activity;protein kinase activity;ATP binding;regulation of transcription, DNA-templated;protein phosphorylation		
XLOC_026125	TCONS_00050332	gag-pol partial	binding;macromolecule metabolic process;primary metabolic process		
XLOC_026127	TCONS_00050334	hypothetical protein PHAVU_010G084700g			
XLOC_026128	TCONS_00050335	hypothetical protein PHAVU_010G071400g			
XLOC_026132	TCONS_00050339	hypothetical protein LR48_Vigan845s005400			
XLOC_026133	TCONS_00050340	hypothetical protein PHAVU_011G131100g			
XLOC_026135	TCONS_00050342	hypothetical protein PHAVU_010G025200g	ATP binding;ADP binding;signal transduction		
XLOC_026136	TCONS_00050343	hypothetical protein PHAVU_010G025400g	ATP binding;ADP binding;signal transduction		
XLOC_026137	TCONS_00050344	hypothetical protein			
XLOC_026138	TCONS_00050345	disease resistance protein (tir-nbs-lrr class)	ADP binding;signal transduction		
XLOC_026144	TCONS_00050351	rna polymerase beta subunit	chloroplast;DNA binding;DNA-directed RNA polymerase activity;transcription, DNA-templated	ec:2.7.7.6-RNA polymerase	Purine metabolism;Pyrimidine metabolism

XLOC_026146	TCONS_00050353	hypothetical protein GLYMA_01G137800			
XLOC_026147	TCONS_00050354	rna-directed dna polymerase	RNA-directed DNA polymerase activity;RNA-dependent DNA replication;mRNA processing	ec:2.7.7.49	
XLOC_026149	TCONS_00050356	probable l-type lectin-domain containing receptor kinase	protein kinase activity;binding;phosphorylation		
XLOC_026153	TCONS_00050360	solute carrier family 40 protein	integral component of membrane;iron ion transmembrane transporter activity;iron ion transmembrane transport		
XLOC_026154	TCONS_00050361	hypothetical protein PHAVU_011G131100g	binding;metabolic process		
XLOC_026159	TCONS_00050366	nadh-plastoquinone oxidoreductase subunit 3	plasma membrane;chloroplast thylakoid membrane;integral component of membrane;mitochondrial membrane;proton-transporting ATP synthase complex, catalytic core F(1);respiratory chain;NADH dehydrogenase (ubiquinone) activity;proton-transporting ATP synthase activity, rotational mechanism;proton-transporting ATPase activity, rotational mechanism;quinone binding;ATP synthesis coupled proton transport;photosynthesis, light reaction;oxidation-reduction process	ec:3.6.1.15-phosphatase;ec:3.6.1.3-adenylpyrophosphatase;ec:3.6.1;ec:1.6.99.5;ec:1.6.99.3-dehydrogenase;ec:1.6.5.3-reductase (H+-translocating)	Purine metabolism;Thiamine metabolismPurine metabolismOxidative phosphorylationOxidative phosphorylation
XLOC_026160	TCONS_00050367	hypothetical protein LR48_Vigan03g139700			
XLOC_026161	TCONS_00050368	photosystem i assembly protein ycf3	chloroplast thylakoid membrane;photosynthesis		
XLOC_026165	TCONS_00050372	rna polymerase beta subunit	chloroplast;DNA binding;DNA-directed RNA polymerase activity;transcription, DNA-templated	ec:2.7.7.6-RNA polymerase	Purine metabolism;Pyrimidine metabolism
XLOC_026166	TCONS_00050373	hypothetical protein PHAVU_011G120700g			
XLOC_026173	TCONS_00050380	hypothetical protein PHAVU_011G184000g			
XLOC_026175	TCONS_00050382	hypothetical protein PHAVU_010G087400g	organic cyclic compound binding;heterocyclic compound binding		
XLOC_026179	TCONS_00050386	disease resistance protein	nucleotide binding		
XLOC_026183	TCONS_00050390	hypothetical protein PHAVU_007G108600g			

XLOC_026184	TCONS_00050391	PREDICTED: uncharacterized protein LOC105953446			
XLOC_026185	TCONS_00050392	uncharacterized mitochondrial protein g00810-like	nucleic acid binding;zinc ion binding		
XLOC_026189	TCONS_00050396	hypothetical protein CICLE_v10023190mg			
XLOC_026194	TCONS_00050401	disease resistance protein (tir-nbs-lrr class)	ADP binding;signal transduction		
XLOC_026197	TCONS_00050404	hypothetical protein PhapfoPp088	chloroplast;catalytic activity;metal ion binding;metabolic process		
XLOC_026199	TCONS_00050406	abc transporter-like family-protein	integral component of membrane;ATP binding;ATPase activity, coupled to transmembrane movement of substances;metabolic process;transmembrane transport	ec:3.6.1.15-phosphatase;ec:3.6.1.3-adenylpyrophosphatase; ec:3.6.1	Purine metabolism;Thiamine metabolismPurine metabolism
XLOC_026202	TCONS_00050409	protein far-red impaired response 1	regulation of transcription, DNA-templated		
XLOC_026246	TCONS_00050499	hypothetical protein PHAVU_011G007700g			
XLOC_026270	TCONS_00050555	hypothetical protein PHAVU_007G129600g			
XLOC_026341	TCONS_00050691	PREDICTED: uncharacterized protein LOC100527898			
XLOC_026345	TCONS_00050695	tudor pwwp mbt domain-containing isoform 2	3-hydroxyisobutyrate dehydrogenase activity;oxidation-reduction process	ec:1.1.1.31-dehydrogenase	Valine, leucine and isoleucine degradation
XLOC_026591	TCONS_00051207	hypothetical protein PHAVU_011G075800g			
XLOC_026612	TCONS_00051256	adrenodoxin-like protein mitochondrial	electron carrier activity;metal ion binding;2 iron, 2 sulfur cluster binding		
XLOC_026696	TCONS_00051403	serine acetyltransferase 5-like	cytosol;magnesium ion binding;serine O-acetyltransferase activity;terpene synthase activity;cysteine biosynthetic process from serine;protein targeting to membrane;response to wounding;response to fungus;jasmonic acid biosynthetic process;salicylic acid mediated signaling pathway;jasmonic acid mediated signaling pathway;regulation of plant-type hypersensitive response	ec:2.3.1;ec:2.3.1.30-O-acetyltransferase	Sulfur metabolism;Biosynthesis of antibiotics;Cysteine and methionine metabolism
XLOC_026772	TCONS_00051517	hypothetical protein LR48_Vigan08g111800	photorespiration		

XLOC_026788	TCONS_00051538	transmembrane e3 ubiquitin-protein ligase 1-like	integral component of membrane;zinc ion binding;ligase activity;metabolic process		
XLOC_026865	TCONS_00051656	hypothetical protein PHAVU_011G131100g			
XLOC_026949	TCONS_00051774	scarecrow-like protein 21	transcription, DNA-templated;regulation of transcription, DNA-templated		
XLOC_027015	TCONS_00051893	wd repeat-containing protein 25	Cul4-RING E3 ubiquitin ligase complex;photoperiodism, flowering		
XLOC_027091	TCONS_00052034	pentatricopeptide repeat-containing protein at1g53330	embryo development;fruit development;root development;shoot system development		
XLOC_027135	TCONS_00052113	glucan endo- -beta-glucosidase	glucan exo-1,3-beta-glucosidase activity;carbohydrate metabolic process	ec:3.2.1.58-1,3-beta-glucosidase; ec:3.2.1.21-gentiobiase; ec:3.2.1	Starch and sucrose metabolismCyanoamino acid metabolism;Phenylpropanoid biosynthesis;Starch and sucrose metabolism
XLOC_027242	TCONS_00052297	hypothetical protein PHAVU_011G194800g			
XLOC_027588	TCONS_00052954	specific tissue protein			
XLOC_027643	TCONS_00053076	suppressor of rps4-rld 1	intracellular part;regulation of transcription, DNA-templated;response to stress;negative regulation of gene expression;single-organism cellular process		
XLOC_027767	TCONS_00053359	zinc c3hc4 type (ring finger) protein	anaphase-promoting complex;protein kinase activity;ubiquitin-protein transferase activity;ATP binding;zinc ion binding;protein phosphorylation;protein ubiquitination		
XLOC_027885	TCONS_00053593	hypothetical protein glysoja_006702			
XLOC_027916	TCONS_00053639	lysine-specific demethylase jmj16	nucleus;transcription factor activity, sequence-specific DNA binding;protein glycosylation;gravitropism;vegetative to reproductive phase transition of meristem;glucuronoxylan metabolic process;protein desumoylation;post-translational protein modification;xylan biosynthetic process;positive regulation of transcription, DNA-templated;hydrogen peroxide biosynthetic process		
XLOC_027985	TCONS_00053754	hypothetical protein PHAVU_010G070700g			
XLOC_028007	TCONS_00053786	probable inactive serine threonine-protein kinase lvsg	Cul4-RING E3 ubiquitin ligase complex;protein kinase activity;ATP binding;protein phosphorylation;protein N-linked glycosylation;protein targeting to vacuole;defense		

			response to bacterium, incompatible interaction		
XLOC_028058	TCONS_00053862	protein lurp-one-related 6-like			
XLOC_028126	TCONS_00053979	protein spa1-related 3	protein kinase activity;ATP binding;ligase activity;2-alkenal reductase [NAD(P)] activity;protein phosphorylation;oxidation-reduction process	ec:1.3.1.74	
XLOC_028139	TCONS_00054008	hypothetical protein LR48_Vigan07g230300			
XLOC_028191	TCONS_00054108	lrr receptor-like	catalytic activity;metabolic process		
XLOC_028193	TCONS_00054111	lrr receptor-like	protein kinase activity;ATP binding;protein phosphorylation;regulation of cellular process		
XLOC_028205	TCONS_00054130	chaperone protein dnaj 49			
XLOC_028213	TCONS_00054151	aldehyde dehydrogenase 22a1	cytosol;oxidoreductase activity, acting on the aldehyde or oxo group of donors, NAD or NADP as acceptor;nitronate monooxygenase activity;para-aminobenzoic acid metabolic process;oxidation-reduction process	ec:1.13.12.16-monooxygenase;ec:1.13.12	Nitrogen metabolism
XLOC_028322	TCONS_00054362	serine carboxypeptidase-like protein	serine-type carboxypeptidase activity;proteolysis	ec:3.4.16;ec:3.4.21	
XLOC_028393	TCONS_00054500	e3 ubiquitin-protein ligase orthrus 2	zinc ion binding;histone binding		
XLOC_028396	TCONS_00054503	disease resistance rpp13-like protein 1			
XLOC_028397	TCONS_00054504	lrr and nb-arc domain disease resistance protein			
XLOC_028405	TCONS_00054512	abc transporter g family member 15-like isoform x1			
XLOC_028410	TCONS_00054517	maturase k	chloroplast;RNA binding;mRNA processing;tRNA processing;RNA splicing		
XLOC_028412	TCONS_00054519	hypothetical protein B456_003G094200			
XLOC_028414	TCONS_00054521	maturase k	chloroplast;RNA binding;mRNA processing;tRNA processing;RNA splicing		
XLOC_028415	TCONS_00054522	hypothetical protein PHAVU_006G0250000g, partial	DNA binding		
XLOC_028417	TCONS_00054524	probable serine threonine-	protein kinase activity;ATP binding;protein phosphorylation		

		protein kinase gcn2 isoform x1			
XLOC_028418	TCONS_00054525	hypothetical protein PHAVU_005G070100g			
XLOC_028419	TCONS_00054526	hypothetical protein VIGAN_06199800, partial			
XLOC_028421	TCONS_00054528	hypothetical protein LR48_Vigan238s000200			
XLOC_028427	TCONS_00054534	hypothetical protein VIGAN_06177900			
XLOC_028430	TCONS_00054537	hypothetical protein PHAVU_010G034700g			
XLOC_028431	TCONS_00054538	hypothetical protein glysoja_008166			
XLOC_028435	TCONS_00054542	metal transporter nramp6	membrane;transporter activity;transport		
XLOC_028436	TCONS_00054543	disease resistance-dirigent domain protein			
XLOC_028441	TCONS_00054548	hypothetical chloroplast rf68	chloroplast		
XLOC_028445	TCONS_00054552	PREDICTED: uncharacterized protein LOC106302390			
XLOC_028449	TCONS_00054556	ctp:phosphorylcholine cytidylyltransferase	choline-phosphate cytidylyltransferase activity;phosphatidylcholine biosynthetic process	ec:2.7.7.15-cytidylyltransferase	Phosphonate and phosphinate metabolism;Glycerophospholipid metabolism
XLOC_028450	TCONS_00054557	hypothetical protein GLYMA_09G051300			
XLOC_028452	TCONS_00054559	hypothetical protein PHAVU_002G055500g			
XLOC_028453	TCONS_00054560	hypothetical protein PHAVU_011G121500g			
XLOC_028454	TCONS_00054561	hypothetical protein PHAVU_011G1212001g			
XLOC_028457	TCONS_00054564	cyclic nucleotide-gated ion channel 4-like	integral component of membrane;ion channel activity;ion transmembrane transport		
XLOC_028459	TCONS_00054566	photosystem i p700 chlorophyll a apoprotein a2	photosystem I;chloroplast thylakoid membrane;integral component of membrane;magnesium ion binding;electron carrier activity;chlorophyll binding;oxidoreductase activity;4 iron, 4 sulfur		

			cluster binding;photosynthesis;protein-chromophore linkage;oxidation-reduction process		
XLOC_028461	TCONS_00054568	hypothetical protein LR48_Vigan09g073700			
XLOC_028466	TCONS_00054573	rna-binding protein 28	nucleus;nucleotide binding;RNA binding		
XLOC_028468	TCONS_00054575	pap-specific phosphatase hal2-like	inositol-1,4-bisphosphate 1-phosphatase activity;3'(2'),5'-bisphosphate nucleotidase activity;phosphotransferase activity, alcohol group as acceptor;protein dephosphorylation;sulfur compound metabolic process;phosphatidylinositol phosphorylation	ec:3.1.3.57-1-phosphatase;ec:3.1.3.41-nitrophenyl phosphatase;ec:3.1;ec:3.1.3.31;ec:3.1.3.7-nucleotidase;ec:3.1.3	Phosphatidylinositol signaling system;Inositol phosphate metabolismAminobenzoate degradationSulfur metabolism
XLOC_028471	TCONS_00054578	cysteine-rich rlk (receptor-like protein kinase) 8	nucleic acid binding;cellular metabolic process		
XLOC_028472	TCONS_00054579	ubiquitin-conjugating enzyme e2 26	ligase activity;metabolic process		
XLOC_028473	TCONS_00054580	hypothetical protein PHAVU_011G163800g			
XLOC_028474	TCONS_00054581	glucan endo- -beta-glucosidase 14	nucleus;anchored component of plasma membrane;hydrolase activity, hydrolyzing O-glycosyl compounds;carbohydrate metabolic process;protein targeting to membrane;salicylic acid mediated signaling pathway;jasmonic acid mediated signaling pathway;regulation of plant-type hypersensitive response		
XLOC_028477	TCONS_00054584	hypothetical protein PHAVU_008G173400g			
XLOC_028480	TCONS_00054587	photosystem i p700 chlorophyll a apoprotein a2	photosystem I;chloroplast thylakoid membrane;integral component of membrane;magnesium ion binding;electron carrier activity;chlorophyll binding;oxidoreductase activity;4 iron, 4 sulfur cluster binding;photosynthesis;protein-chromophore linkage;oxidation-reduction process		
XLOC_028484	TCONS_00054591	photosystem ii phosphoprotein	photosystem II;chloroplast thylakoid membrane;integral component of membrane;phosphate ion binding;photosynthesis;protein stabilization		
XLOC_028487	TCONS_00054594	disease resistance protein	ADP binding		
XLOC_028491	TCONS_00054598	PREDICTED: uncharacterized protein			

		LOC102668362, partial			
XLOC_028492	TCONS_00054599	nbs-lrr-like protein cd8	ADP binding		
XLOC_028494	TCONS_00054601	hypothetical protein PHAVU_011G201800g			
XLOC_028495	TCONS_00054602	disease resistance rpp13-like protein 1			
XLOC_028496	TCONS_00054603	nadh-plastoquinone oxidoreductase subunit partial	chloroplast thylakoid membrane;integral component of membrane;NADH dehydrogenase (ubiquinone) activity;quinone binding;transport;photosynthesis, light reaction;ATP synthesis coupled electron transport	ec:1.6.99.5;ec:1.6.99.3-dehydrogenase;ec:1.6.5.3-reductase (H+-translocating)	Oxidative phosphorylationOxidative phosphorylation
XLOC_028497	TCONS_00054604	dis3-exonuclease-like protein	exonuclease activity;nucleic acid phosphodiester bond hydrolysis	ec:3.1	
XLOC_028498	TCONS_00054605	hypothetical chloroplast rf1	chloroplast inner membrane;integral component of membrane;protein transport		
XLOC_028510	TCONS_00054623	serine threonine-protein kinase sapk7-like isoform x2	DNA binding;protein kinase activity;ATP binding;protein dimerization activity;protein phosphorylation		
XLOC_028545	TCONS_00054686	nadh dehydrogenase subunit 1	plasma membrane;chloroplast thylakoid membrane;integral component of membrane;oxidoreductase activity, acting on NAD(P)H, quinone or similar compound as acceptor;quinone binding;photosynthesis, light reaction;oxidation-reduction process		
XLOC_028551	TCONS_00054693	atp synthase alpha partial	mitochondrial inner membrane;chloroplast thylakoid membrane;proton-transporting ATP synthase complex, catalytic core F(1);ATP binding;proton-transporting ATP synthase activity, rotational mechanism;proton-transporting ATPase activity, rotational mechanism;ATP synthesis coupled proton transport;ATP hydrolysis coupled proton transport	ec:3.6.1.15-phosphatase;ec:3.6.1.3-adenylpyrophosphatase;ec:3.6.1	Purine metabolism;Thiamine metabolismPurine metabolism
XLOC_028552	TCONS_00054694	hypothetical protein GLYMA_07G099300			
XLOC_028558	TCONS_00054702	protein rst1 isoform x3	cellular protein modification process;response to abiotic stimulus;embryo development ending in seed dormancy;meristem structural organization;seed maturation;cellular macromolecule biosynthetic process;single-organism biosynthetic process;single-organism carbohydrate metabolic process;single-		

			organism cellular process;xylan metabolic process;leaf development;regulation of developmental process;regulation of cellular process;cellular component organization or biogenesis		
XLOC_028561	TCONS_00054706	potassium transporter 12			
XLOC_028572	TCONS_00054719	glycerophosphodiester phosphodiesterase gdpdl4-like isoform x2			
XLOC_028573	TCONS_00054720	nbs-lrr type disease resistance protein cnl-b19	ADP binding		
XLOC_028574	TCONS_00054722	cellular apoptosis susceptibility protein importin-alphare- isoform 1	nuclear pore;cytosol;plasmodesma;chloroplast;membrane;Ran GTPase binding;protein transporter activity;protein import into nucleus, docking;gluconeogenesis;cytoskeleton organization;response to wounding;response to fungus;jasmonic acid biosynthetic process;response to jasmonic acid;proteasomal protein catabolic process		
XLOC_028588	TCONS_00054740	sister chromatid cohesion pds5-like protein			
XLOC_028600	TCONS_00054753	protein rst1 isoform x1	protein glycosylation;cuticle hydrocarbon biosynthetic process;sister chromatid cohesion;photomorphogenesis;seed germination;embryonic pattern specification;regulation of flower development;primary shoot apical meristem specification;seed dormancy process;sugar mediated signaling pathway;vegetative to reproductive phase transition of meristem;glucuronoxylan metabolic process;regulation of cell cycle process;protein ubiquitination;lipid storage;xylan biosynthetic process;regulation of cell differentiation;cotyledon development;response to freezing;cell division		
XLOC_028601	TCONS_00054755	lysosomal alpha-mannosidase	vacuolar membrane;plant-type cell wall;apoplast;alpha-mannosidase activity;zinc ion binding;carbohydrate binding;mannose metabolic process;microtubule nucleation;cysteine biosynthetic process	ec:3.2.1.24-alpha-D-mannosidase;ec:3.2.1	Other glycan degradation
XLOC_028606	TCONS_00054761	cytochrome b6	chloroplast thylakoid membrane;integral component of membrane;iron ion		

			binding;oxidoreductase activity;heme binding;electron transporter, transferring electrons within cytochrome b6/f complex of photosystem II activity;photosynthesis;respiratory electron transport chain		
XLOC_028615	TCONS_00054771	plant invertase pectin methylesterase inhibitor	enzyme inhibitor activity;pectinesterase activity;metabolic process;negative regulation of catalytic activity	ec:3.1.1.1-ali-esterase;ec:3.1.1.11-pectin demethoxylase	Drug metabolism - other enzymesPentose and glucuronate interconversions;Starch and sucrose metabolism
XLOC_028625	TCONS_00054787	casp poptrdraft-like protein	plasma membrane;integral component of membrane		
XLOC_028639	TCONS_00054806	cytochrome b6 f complex subunit iv	chloroplast thylakoid membrane;integral component of membrane;oxidoreductase activity;electron transporter, transferring electrons within the cyclic electron transport pathway of photosynthesis activity;electron transporter, transferring electrons within cytochrome b6/f complex of photosystem II activity;photosynthetic electron transport chain		

SeqName	Sequence Description	GO Terms	Enzymes	Kegg Maps
		**DEG**		
Phvul.001G005800	hypothetical protein PHAVU_001G005800g			
Phvul.001G016600	pathogenesis-related protein 5-like			
Phvul.001G017200	type i inositol -trisphosphate 5-phosphatase 11-like	phosphatidylinositol-4,5-bisphosphate 5-phosphatase activity;nuclease activity;phosphatidylinositol-3,4,5-trisphosphate 5-phosphatase activity;phosphatidylinositol-3,5-bisphosphate 5-phosphatase activity;protein dephosphorylation;phosphatidylinositol-3-phosphate biosynthetic process;phosphatidylinositol dephosphorylation	ec:3.1.3.41-nitrophenyl phosphatase;ec:3.1;ec:3.1.3.36-5-phosphatase;ec:3.1.3	Aminobenzoate degradationInositol phosphate metabolism;Phosphatidylinositol signaling system
Phvul.001G018300	probable transcription factor kan4	DNA binding;oxidoreductase activity;single-organism process		
Phvul.001G020300	patatin-like phospholipase	hydrolase activity;lipid catabolic process		
Phvul.001G024400	serine threonine plant-type	kinase activity;phosphorylation		
Phvul.001G025300	hypothetical protein PHAVU_001G025300g			
Phvul.001G029100	hypothetical protein PHAVU_001G029100g			
Phvul.001G029200	phytosulfokines 3-like	extracellular region;growth factor activity;cell proliferation		
Phvul.001G029500	basic leucine zipper 1-like	intracellular;transcription factor activity, sequence-specific DNA binding;MAP kinase activity;sequence-specific DNA binding;MAPK cascade;regulation of transcription, DNA-templated	ec:2.7.11.24-protein kinase;ec:2.7.11	mTOR signaling pathway
Phvul.001G037100	cytochrome p450 711a1-like	iron ion binding;heme binding;aromatase activity;auxin polar transport;regulation of meristem structural organization;secondary shoot formation;oxidation-reduction process	ec:1.14.14;ec:1.14.14.1-monooxygenase	Linoleic acid metabolism;Arachidonic acid metabolism;Drug metabolism - cytochrome P450;Metabolism of xenobiotics by cytochrome P450;Caffeine metabolism;Aminobenzoate degradation;Tryptophan metabolism;Fatty acid degradation;Retinol metabolism;Steroid hormone biosynthesis
Phvul.001G038500	hypothetical protein PHAVU_001G038500g			
Phvul.001G039600	solute carrier family 25 member 44	integral component of membrane;transmembrane transport		
Phvul.001G039700	kda class ii heat shock protein	cytoplasm		
Phvul.001G039800	kda class ii heat shock protein	cytoplasm		
Phvul.001G040600	l-type lectin-domain containing receptor kinase -like	protein serine/threonine kinase activity;transmembrane receptor protein tyrosine kinase activity;ATP binding;carbohydrate binding;peptidyl-tyrosine phosphorylation	ec:2.7.10.1;ec:2.7.11;ec:2.7.10	
Phvul.001G040800	l-type lectin-domain containing	protein serine/threonine kinase activity;transmembrane receptor protein tyrosine	ec:2.7.10.1;ec:2.7.11;ec:2.7.10	

	receptor kinase -like	kinase activity;ATP binding;carbohydrate binding;peptidyl-tyrosine phosphorylation		
Phvul.001G042200	wrky family transcription factor	transcription factor activity, sequence-specific DNA binding;sequence-specific DNA binding;regulation of transcription, DNA-templated		
Phvul.001G043500	protein glutamine dumper 3-like			
Phvul.001G050400	hypothetical protein PHAVU_001G050400g			
Phvul.001G051000	hypothetical protein PHAVU_001G051000g			
Phvul.001G052100	cytochrome p450 family 71 protein	monooxygenase activity;iron ion binding;oxidoreductase activity, acting on paired donors, with incorporation or reduction of molecular oxygen;heme binding;oxidation-reduction process		
Phvul.001G054500	pleiotropic drug resistance protein 1-like	integral component of membrane;ATP binding;organic phosphonate transmembrane-transporting ATPase activity;polyamine-transporting ATPase activity;metabolic process;organic phosphonate transport;organophosphate ester transport;polyamine transmembrane transport	ec:3.6.3.31;ec:3.6.3.28;ec:3.6.1.3-adenylpyrophosphatase;ec:3.6.1;ec:3.6.1.15-phosphatase	Purine metabolismPurine metabolism;Thiamine metabolism
Phvul.001G055000	gibberellin 3-beta-dioxygenase 1-like	iron ion binding;gibberellin 3-beta-dioxygenase activity;oxidation-reduction process	ec:1.14.11.15-3beta-dioxygenase;ec:1.14.11	Diterpenoid biosynthesis
Phvul.001G059600	hypothetical protein PHAVU_001G059600g			
Phvul.001G064000	alcohol dehydrogenase 1	cytoplasm;alcohol dehydrogenase (NAD) activity;zinc ion binding;S-(hydroxymethyl)glutathione dehydrogenase activity;oxidation-reduction process	ec:1.1.1.284-dehydrogenase;ec:1.1.1.1-dehydrogenase	Methane metabolismalpha-Linolenic acid metabolism;Drug metabolism - cytochrome P450;Metabolism of xenobiotics by cytochrome P450;Naphthalene degradation;Chloroalkane and chloroalkene degradation;Biosynthesis of antibiotics;Glycolysis / Gluconeogenesis;Fatty acid degradation;Glycine, serine and threonine metabolism;Tyrosine metabolism;Retinol metabolism
Phvul.001G067400	ef hand calcium-binding family protein	calcium ion binding		
Phvul.001G071200	probable receptor-like protein kinase at1g11050	transmembrane receptor protein tyrosine kinase activity;ATP binding;peptidyl-tyrosine phosphorylation	ec:2.7.10.1;ec:2.7.11;ec:2.7.10	
Phvul.001G074200	ultraviolet-b receptor uvr8	ligase activity;metabolic process		
Phvul.001G075400	probable protein phosphatase 2c 51	protein serine/threonine phosphatase activity;metal ion binding;protein dephosphorylation	ec:3.1.3.16-phosphatase;ec:3.1.3.41-nitrophenyl phosphatase;ec:3.1	T cell receptor signaling pathwayAminobenzoate degradation
Phvul.001G076000	peroxidase 5-like	extracellular region;peroxidase activity;heme binding;metal ion binding;response to oxidative stress;hydrogen peroxide catabolic process;oxidation-reduction process	ec:1.11.1.7-lactoperoxidase	Phenylpropanoid biosynthesis

Phvul.001G076700	beta-glucosidase 12-like	beta-glucosidase activity;carbohydrate metabolic process	ec:3.2.1;ec:3.2.1.21-gentiobiase	Starch and sucrose metabolism;Cyanoamino acid metabolism;Phenylpropanoid biosynthesis
Phvul.001G077000	amino acid permease 6	plasma membrane;integral component of membrane;acidic amino acid transmembrane transporter activity;neutral amino acid transmembrane transporter activity;neutral amino acid transport;aspartate transport;tryptophan transport		
Phvul.001G081000	aluminum-activated malate transporter 10-like	malate transport		
Phvul.001G085900	transmembrane protein 45b	integral component of membrane		
Phvul.001G088200	probable wrky transcription factor 75	transcription factor activity, sequence-specific DNA binding;sequence-specific DNA binding;regulation of transcription, DNA-templated		
Phvul.001G091600	2-isopropylmalate synthase	2-isopropylmalate synthase activity;leucine biosynthetic process	ec:2.3.3.13-synthase	Valine, leucine and isoleucine biosynthesis;Pyruvate metabolism
Phvul.001G103600	protein transparent testa 12	integral component of membrane;drug transmembrane transporter activity;antiporter activity;drug transmembrane transport		
Phvul.001G108300	protein transparent testa 12	integral component of membrane;drug transmembrane transporter activity;antiporter activity;drug transmembrane transport		
Phvul.001G112400	spermidine hydroxycinnamoyl transferase-like	shikimate O-hydroxycinnamoyltransferase activity;metabolic process	ec:2.3.1;ec:2.3.1.133-O-hydroxycinnamoyl transferase	Stilbenoid, diarylheptanoid and gingerol biosynthesis;Flavonoid biosynthesis;Phenylpropanoid biosynthesis
Phvul.001G112500	spermidine hydroxycinnamoyl transferase-like	shikimate O-hydroxycinnamoyltransferase activity;metabolic process	ec:2.3.1;ec:2.3.1.133-O-hydroxycinnamoyl transferase	Stilbenoid, diarylheptanoid and gingerol biosynthesis;Flavonoid biosynthesis;Phenylpropanoid biosynthesis
Phvul.001G124700	s-adenosylmethionine-dependent methyltransferase	mitochondrion;methyltransferase activity;methylation		
Phvul.001G124800	s-adenosylmethionine-dependent methyltransferase	mitochondrion;methyltransferase activity;methylation		
Phvul.001G127700	gibberellin induced protein			
Phvul.001G128500	glucan endo- -beta- basic isoform-like	vacuole;glucan endo-1,3-beta-D-glucosidase activity;carbohydrate metabolic process;defense response	ec:3.2.1;ec:3.2.1.39-endo-1,3-beta-D-glucosidase;ec:3.2.1.21-gentiobiase	Starch and sucrose metabolismStarch and sucrose metabolism;Cyanoamino acid metabolism;Phenylpropanoid biosynthesis
Phvul.001G131000	heat stress transcription factor b-3	nucleus;transcription factor activity, sequence-specific DNA binding;sequence-specific DNA binding;regulation of transcription, DNA-templated;toxin catabolic process;response to heat		
Phvul.001G135200	probable galactinol--sucrose galactosyltransferase 2	plasmodesma;galactinol-raffinose galactosyltransferase activity;raffinose alpha-galactosidase activity;raffinose catabolic process	ec:3.2.1;ec:2.4.1;ec:2.4.1.67-galactosyltransferase;ec:3.2.1.22-melibiase	Galactose metabolismGlycosphingolipid biosynthesis - globo series;Sphingolipid metabolism;Glycerolipid metabolism;Galactose metabolism
Phvul.001G139400	-dihydroxypterocarpan 6a-monooxygenase	monooxygenase activity;iron ion binding;oxidoreductase activity, acting on paired donors, with incorporation or reduction of molecular oxygen;heme binding;oxidation-reduction process		

Phvul.001G 140000	polygalacturonase inhibiting protein	transferase activity;metabolic process		
Phvul.001G 142000	seed maturation protein lea 4	embryo development		
Phvul.001G 143100	seed maturation protein lea 4	embryo development		
Phvul.001G 143500	homeobox-leucine zipper protein hox11-like	nucleus;transcription factor activity, sequence-specific DNA binding;sequence-specific DNA binding;transcription, DNA-templated;regulation of transcription, DNA-templated		
Phvul.001G 145600	disease resistance response protein			
Phvul.001G 145700	disease resistance response protein 206			
Phvul.001G 148000	hypothetical protein PHAVU_001G148 000g			
Phvul.001G 148100	hypothetical protein PHAVU_001G148 100g			
Phvul.001G 151900	copalyldiphosphat e synthase	magnesium ion binding;ent-copalyl diphosphate synthase activity;terpene synthase activity;metabolic process	ec:5.5.1.13-diphosphate synthase	Diterpenoid biosynthesis
Phvul.001G 152100	copalyldiphosphat e synthase	chloroplast;magnesium ion binding;ent-copalyl diphosphate synthase activity;terpene synthase activity;gibberellin biosynthetic process	ec:5.5.1.13-diphosphate synthase	Diterpenoid biosynthesis
Phvul.001G 154200	sulfate transporter -like	chloroplast;integral component of membrane;secondary active sulfate transmembrane transporter activity;sulfate transmembrane transport		
Phvul.001G 154500	bi1-like protein	integral component of membrane;protein folding;response to heat;response to high light intensity;response to hydrogen peroxide		
Phvul.001G 156500	auxin-induced protein 22e-like	nucleus;transcription, DNA-templated;regulation of transcription, DNA-templated;auxin-activated signaling pathway		
Phvul.001G 156900	phospholipase a1-chloroplastic-like	hydrolase activity;lipid metabolic process		
Phvul.001G 158100	cytochrome p450 94a1-like	endoplasmic reticulum membrane;integral component of membrane;monooxygenase activity;iron ion binding;oxidoreductase activity, acting on paired donors, with incorporation or reduction of molecular oxygen;heme binding;oxidation-reduction process		
Phvul.001G 160100	ap2 domain class transcription factor	nucleus;DNA binding;transcription factor activity, sequence-specific DNA binding;transcription, DNA-templated;regulation of transcription, DNA-templated;ethylene-activated signaling pathway		
Phvul.001G 160200	ethylene-responsive transcription factor 1b	nucleus;DNA binding;transcription factor activity, sequence-specific DNA binding;transcription, DNA-templated;regulation of transcription, DNA-templated		
Phvul.001G 161000	myb-related protein myb4-like	DNA binding;response to acid chemical;response to stress;hormone-mediated signaling pathway;response to oxygen-containing compound		
Phvul.001G 162800	long-chain-alcohol oxidase fao2-like	integral component of membrane;long-chain-alcohol oxidase activity;flavin adenine dinucleotide binding;oxidation-reduction process	ec:1.1.3.20	
Phvul.001G 163400	peptide transporter	membrane;transporter activity;transport		
Phvul.001G 165200	photosystem ii reaction center psb28 chloroplastic	photosystem II oxygen evolving complex;photosynthesis		
Phvul.001G 165700	drug resistance transporter-like abc domain protein	integral component of membrane;ATP binding;ATPase activity;transport;metabolic process	ec:3.6.1.3-adenylpyrophosphatase;ec:3.6.1;ec:3.6.1.15-phosphatase	Purine metabolismPurine metabolism;Thiamine metabolism
Phvul.001G 166000	drug resistance transporter-like abc domain protein	integral component of membrane;ATP binding;organic phosphonate transmembrane-transporting ATPase activity;metabolic process;organic phosphonate transport;organophosphate ester transport	ec:3.6.3.28;ec:3.6.1.3-adenylpyrophosphatase;ec:3.6.1;ec	Purine metabolismPurine metabolism;Thiamine metabolism

			:3.6.1.15-phosphatase	
Phvul.001G166700	two-component response regulator arr22-like	intracellular;kinase activity;phosphorelay signal transduction system;phosphorylation		
Phvul.001G169300	cation h(+) antiporter 20	endoplasmic reticulum;integral component of membrane;solute:proton antiporter activity;protein targeting to vacuole;regulation of pH;response to stress;cellular potassium ion homeostasis;water homeostasis;hydrogen ion transmembrane transport		
Phvul.001G173300	glutathione s-transferase l3	cytoplasm;glutathione transferase activity;metabolic process	ec:2.5.1.18-transferase	Drug metabolism - cytochrome P450;Metabolism of xenobiotics by cytochrome P450;Glutathione metabolism
Phvul.001G174200	transcription factor asg4	DNA binding		
Phvul.001G177100	hypothetical protein PHAVU_001G177100g			
Phvul.001G180500	transporter mch1	integral component of membrane;transmembrane transport		
Phvul.001G182300	udp-glucosyltransferase family protein	transferase activity, transferring hexosyl groups;metabolic process		
Phvul.001G183300	leucoanthocyanidin dioxygenase-like protein	monooxygenase activity;iron ion binding;methyltransferase activity;oxidoreductase activity, acting on paired donors, with incorporation or reduction of molecular oxygen, 2-oxoglutarate as one donor, and incorporation of one atom each of oxygen into both donors;heme binding;methylation;oxidation-reduction process	ec:1.14.11	
Phvul.001G184600	embryonic protein dc-8-like			
Phvul.001G185700	plant t22k18-16	response to oxidative stress		
Phvul.001G187800	drug resistance transporter-like abc domain protein	integral component of membrane;ATP binding;organic phosphonate transmembrane-transporting ATPase activity;metabolic process;organic phosphonate transport;organophosphate ester transport	ec:3.6.3.28;ec:3.6.1.3-adenylpyrophosphatase;ec:3.6.1;ec:3.6.1.15-phosphatase	Purine metabolismPurine metabolism;Thiamine metabolism
Phvul.001G194800	protein yls9-like			
Phvul.001G201300	peroxidase 3-like	extracellular region;peroxidase activity;heme binding;metal ion binding;response to oxidative stress;hydrogen peroxide catabolic process;oxidation-reduction process	ec:1.11.1.7-lactoperoxidase	Phenylpropanoid biosynthesis
Phvul.001G201500	probable atp-dependent rna helicase ddx56			
Phvul.001G205900	translocator protein homolog	Golgi stack;integral component of membrane;heme binding;porphyrin-containing compound metabolic process		
Phvul.001G206000	xyloglucan 6-xylosyltransferase 2	Golgi apparatus;integral component of membrane;GTP binding;xyloglucan 6-xylosyltransferase activity;metabolic process	ec:2.4.2.39	
Phvul.001G206700	abc transporter c family member 4	integral component of membrane;ATP binding;2-alkenal reductase [NAD(P)] activity;ATPase activity, coupled to transmembrane movement of substances;transmembrane transport;oxidation-reduction process	ec:1.3.1.74;ec:3.6.1.3-adenylpyrophosphatase;ec:3.6.1;ec:3.6.1.15-phosphatase	Purine metabolismPurine metabolism;Thiamine metabolism
Phvul.001G207500	caffeoylshikimate esterase-like	plasma membrane;acylglycerol lipase activity;lipid metabolic process	ec:3.1.1.23-lipase;ec:3.1.1.1-ali-esterase;ec:3.1.1	Glycerolipid metabolismDrug metabolism - other enzymes
Phvul.001G209200	pectinesterase pectinesterase inhibitor	cell wall;enzyme inhibitor activity;pectinesterase activity;aspartyl esterase activity;cell wall modification;negative regulation of catalytic activity;pectin catabolic process	ec:3.1.1.11-pectin demethoxylase;ec:3.1.1.1-ali-esterase	Starch and sucrose metabolism;Pentose and glucuronate interconversionsDrug

				metabolism - other enzymes
Phvul.001G211200	mitochondrial carrier family protein			
Phvul.001G211300	hypothetical protein PHAVU_001G211300g	protein folding;response to heat;response to high light intensity;terpenoid biosynthetic process;response to hydrogen peroxide;positive regulation of transcription, DNA-templated		
Phvul.001G211900	alpha beta-hydrolases superfamily protein	proteasome complex;hydrolase activity;lipid metabolic process;cellular macromolecule metabolic process		
Phvul.001G215700	boron transporter 1-like	integral component of membrane;inorganic anion exchanger activity;anion transmembrane transport		
Phvul.001G215900	polygalacturonase glycoside hydrolase family protein	extracellular region;polygalacturonase activity;galacturan 1,4-alpha-galacturonidase activity;carbohydrate metabolic process;cell wall organization	ec:3.2.1.67-1,4-alpha-galacturonidase;ec:3.2.1.15-pectin depolymerase	Starch and sucrose metabolism;Pentose and glucuronate interconversionsStarch and sucrose metabolism;Pentose and glucuronate interconversions
Phvul.001G219300	suppressor of disruption of tfiis	hydrolase activity;metabolic process;response to nitrate;nitrate transport		
Phvul.001G220800	hypothetical protein PHAVU_001G220800g			
Phvul.001G223700	galactinol synthase	inositol 3-alpha-galactosyltransferase activity;galactose metabolic process	ec:2.4.1.123-3-alpha-galactosyltransferase;ec:2.4.1	Galactose metabolism
Phvul.001G226300	heat shock protein binding			
Phvul.001G231900	little protein 1			
Phvul.001G235000	transcriptional regulator superman protein	metal ion binding		
Phvul.001G246500	cation h(+) antiporter 15-like	integral component of membrane;monovalent cation:proton antiporter activity;potassium ion transport;sodium ion transport;hydrogen ion transmembrane transport		
Phvul.001G247300	cation h(+) antiporter 15-like	integral component of membrane;monovalent cation:proton antiporter activity;potassium ion transport;sodium ion transport;hydrogen ion transmembrane transport		
Phvul.001G249600	e3 ubiquitin-protein ligase rha2a	zinc ion binding		
Phvul.001G250900	duf581 family protein			
Phvul.001G255200	probable e3 ubiquitin-protein ligase xerico	catalytic activity;zinc ion binding		
Phvul.001G258900	hypothetical protein PHAVU_001G258900g	peroxisome		
Phvul.001G263200	hypothetical protein LR48_Vigan102s009700	integral component of membrane		
Phvul.001G263900	hypothetical protein PHAVU_001G263900g			
Phvul.001G264400	cysteine proteinase inhibitor b	cysteine-type endopeptidase inhibitor activity;negative regulation of endopeptidase activity		
Phvul.001G265800	pentatricopeptide repeat-containing protein mitochondrial	membrane;microtubule-severing ATPase activity;metabolic process	ec:3.6.1.3-adenylpyrophosphatase;ec:3.6.1;ec	Purine metabolismPurine metabolism;Thiamine metabolism

			:3.6.4.3;ec:3.6.1.15-phosphatase	
Phvul.002G002000	protein tify 10a-like			
Phvul.002G004100	expansin-like b1	extracellular region		
Phvul.002G007500	basic helix loop helix dna-binding family protein	protein dimerization activity		
Phvul.002G008300	epidermal patterning factor-like protein 2			
Phvul.002G009700	vq motif protein			
Phvul.002G014700	isoflavone 2 -hydroxylase	membrane;iron ion binding;heme binding;aromatase activity;oxidation-reduction process	ec:1.14.14;ec:1.14.14.1-monooxygenase	Linoleic acid metabolism;Arachidonic acid metabolism;Drug metabolism - cytochrome P450;Metabolism of xenobiotics by cytochrome P450;Caffeine metabolism;Aminobenzoate degradation;Tryptophan metabolism;Fatty acid degradation;Retinol metabolism;Steroid hormone biosynthesis
Phvul.002G017500	caffeoyl- o-methyltransferase	caffeoyl-CoA O-methyltransferase activity;metal ion binding;lignin biosynthetic process;methylation	ec:2.1.1;ec:2.1.1.104-O-methyltransferase	Phenylalanine metabolism;Stilbenoid, diarylheptanoid and gingerol biosynthesis;Flavonoid biosynthesis;Phenylpropanoid biosynthesis
Phvul.002G018100	map3k-like kinase	protein serine/threonine kinase activity;ATP binding;protein phosphorylation	ec:2.7.11	
Phvul.002G019100	heat shock factor protein hsf24-like	nucleus;transcription factor activity, sequence-specific DNA binding;sequence-specific DNA binding;response to acid chemical;regulation of transcription, DNA-templated;signal transduction;response to heat;response to organic substance;response to endoplasmic reticulum stress;cellular metabolic process;single-organism metabolic process;response to oxygen-containing compound		
Phvul.002G021800	reticulon-like protein b13	endoplasmic reticulum membrane;integral component of membrane		
Phvul.002G027900	vicilin-like antimicrobial peptides 2-2	nutrient reservoir activity		
Phvul.002G029900	plant mud21-2 protein			
Phvul.002G031100	late embryogenesis abundant domain protein lea domain			
Phvul.002G033100	isoflavone reductase	2'-hydroxyisoflavone reductase activity;oxidation-reduction process	ec:1.3.1.45-reductase	Isoflavonoid biosynthesis
Phvul.002G035800	nuclear transport factor 2 family protein	chloroplast thylakoid membrane;peptidyl-prolyl cis-trans isomerase activity;protein peptidyl-prolyl isomerization;protein folding;transport;chlorophyll catabolic process	ec:5.2.1.8	
Phvul.002G035900	ethylene-responsive transcription factor erf021-like	nucleus;DNA binding;transcription factor activity, sequence-specific DNA binding;transcription, DNA-templated;regulation of transcription, DNA-templated		
Phvul.002G043000	12-oxophytodienoate reductase 2	FMN binding;12-oxophytodienoate reductase activity;oxidation-reduction process	ec:1.3.1.42-reductase	alpha-Linolenic acid metabolism
Phvul.002G046800	sugar transport protein 13	plasma membrane;plasmodesma;integral component of membrane;high-affinity hydrogen:glucose symporter	ec:1.3.1.74	

		activity;2-alkenal reductase [NAD(P)] activity;glucose transport;hexose transmembrane transport;amino acid import;oxidation-reduction process		
Phvul.002G 048100	tryptophan synthase beta chain 1-like	tryptophan synthase activity;2-alkenal reductase [NAD(P)] activity;tryptophan biosynthetic process;oxidation-reduction process	ec:1.3.1.74;ec:4.2.1.20-synthase	Biosynthesis of antibiotics;Phenylalanine, tyrosine and tryptophan biosynthesis;Glycine, serine and threonine metabolism
Phvul.002G 049000	gibberellin 2-beta-dioxygenase 8-like	gibberellin 3-beta-dioxygenase activity;metal ion binding;oxidation-reduction process	ec:1.14.11.15-3beta-dioxygenase;ec:1.14.11	Diterpenoid biosynthesis
Phvul.002G 050700	probable zinc metallopeptidase chloroplastic isoform x1	aminopeptidase activity;protein folding;proteolysis	ec:3.4.11	
Phvul.002G 055600	g-type lectin s-receptor-like serine threonine-protein kinase rlk1-like	protein serine/threonine kinase activity;transmembrane receptor protein tyrosine kinase activity;ATP binding;carbohydrate binding;peptidyl-tyrosine phosphorylation;recognition of pollen	ec:2.7.10.1;ec:2.7.11;ec:2.7.10	
Phvul.002G 061200	acid beta-fructofuranosidase	vacuole;sucrose alpha-glucosidase activity;sucrose metabolic process	ec:3.2.1;ec:3.2.1.48-alpha-glucosidase;ec:3.2.1.26-invertase	Starch and sucrose metabolismStarch and sucrose metabolism;Galactose metabolism
Phvul.002G 061900	peptide nitrate transporter	plasma membrane;integral component of membrane;nitrate transmembrane transporter activity;oligopeptide transport;response to water deprivation;response to nitrate;nitrate transport;sterol biosynthetic process;glucosinolate biosynthetic process;plant-type cell wall cellulose metabolic process;cell wall pectin metabolic process		
Phvul.002G 063500	polygalacturonase at1g48100	extracellular region;polygalacturonase activity;galacturan 1,4-alpha-galacturonidase activity;carbohydrate metabolic process;cell wall organization	ec:3.2.1.67-1,4-alpha-galacturonidase;ec:3.2.1.15-pectin depolymerase	Starch and sucrose metabolism;Pentose and glucuronate interconversionsStarch and sucrose metabolism;Pentose and glucuronate interconversions
Phvul.002G 070300	dna (cytosine-5)-methyltransferase drm2-like isoform x1	DNA binding;transferase activity;DNA methylation		
Phvul.002G 072000	u-box domain-containing protein 19	ubiquitin-protein transferase activity;zinc ion binding;ligase activity;protein ubiquitination		
Phvul.002G 075200	udp-glycosyltransferase 74b1-like	indole-3-acetate beta-glucosyltransferase activity;thiohydroximate beta-D-glucosyltransferase activity;tryptophan catabolic process;indoleacetic acid biosynthetic process;glucosinolate biosynthetic process;defense response to bacterium;root hair elongation;defense response by callose deposition in cell wall	ec:2.4.1.121;ec:2.4.1;ec:2.4.1.195-S-beta-glucosyltransferase	Tryptophan metabolism;Glucosinolate biosynthesis
Phvul.002G 076700	metalloendoproteinase 1-like	extracellular matrix;metalloendopeptidase activity;zinc ion binding;proteolysis	ec:3.4.24	
Phvul.002G 081400	clavata3 esr -related protein 9-like	extracellular region		
Phvul.002G 083800	myb transcription factor	DNA binding		
Phvul.002G 085000	phd finger protein male sterility 1	zinc ion binding;pollen germination;pollen wall assembly;positive regulation of transcription, DNA-templated;anther wall tapetum morphogenesis;microgametogenesis		
Phvul.002G 089700	probable wrky transcription factor 72-like	transcription factor activity, sequence-specific DNA binding;sequence-specific DNA binding;regulation of transcription, DNA-templated		
Phvul.002G 093000	chaperone -domain superfamily	nucleus;cytoplasm;protein folding;response to heat;response to high light intensity;response to hydrogen peroxide		

Phvul.002G 096600	glycoside hydrolase family 17 protein	hydrolase activity, hydrolyzing O-glycosyl compounds;carbohydrate metabolic process		
Phvul.002G 099700	probable zinc transporter 10	integral component of membrane;zinc ion transmembrane transporter activity;iron ion transport;water transport;cellular response to iron ion starvation;zinc II ion transmembrane transport		
Phvul.002G 103300	hypothetical protein PHAVU_002G103 300g			
Phvul.002G 105700	protein fd-like	transcription factor activity, sequence-specific DNA binding;sequence-specific DNA binding;regulation of transcription, DNA-templated		
Phvul.002G 108200	hypothetical protein PHAVU_002G108 200g			
Phvul.002G 112600	thiosulfate sulfurtransferase chloroplastic-like	thiosulfate sulfurtransferase activity;metabolic process	ec:2.8.1.1-sulfurtransferase	Sulfur metabolism
Phvul.002G 117700	auxin efflux carrier family protein	integral component of membrane;transmembrane transport		
Phvul.002G 123500	lrr receptor-like kinase	integral component of membrane;protein serine/threonine kinase activity;ATP binding;protein phosphorylation	ec:2.7.11	
Phvul.002G 124100	hypothetical protein PHAVU_002G124 100g			
Phvul.002G 134700	sn1-specific diacylglycerol lipase alpha	hydrolase activity;lipid metabolic process		
Phvul.002G 140000	tpr repeat protein			
Phvul.002G 141600	plant mza15-19			
Phvul.002G 141900	abscisic acid receptor pyr1-like			
Phvul.002G 148700	6-phosphofructokin ase chloroplastic-like	chloroplast;6-phosphofructokinase activity;ATP binding;metal ion binding;fructose 6-phosphate metabolic process;glycolytic process;carbohydrate phosphorylation	ec:2.7.1;ec:2.7.1.1 1-phosphohexokina se	Biosynthesis of antibiotics;Pentose phosphate pathway;Methane metabolism;Glycolysis / Gluconeogenesis;Galac tose metabolism;Fructose and mannose metabolism
Phvul.002G 154900	protein iq-domain 14			
Phvul.002G 155400	protein p21-like			
Phvul.002G 156100	tubby c 2 protein			
Phvul.002G 159800	1-aminocyclopropan e-1-carboxylate synthase	1-aminocyclopropane-1-carboxylate synthase activity;pyridoxal phosphate binding;identical protein binding;ethylene biosynthetic process;response to ethylene;response to abscisic acid;fruit ripening;response to chitin;1-aminocyclopropane-1-carboxylate biosynthetic process	ec:4.4.1.14-synthase	Cysteine and methionine metabolism
Phvul.002G 160100	wrky transcription factor 22	transcription factor activity, sequence-specific DNA binding;sequence-specific DNA binding;regulation of transcription, DNA-templated		
Phvul.002G 160600	protein lurp-one-related 17			
Phvul.002G 163900	probable protein phosphatase 2c 8	protein serine/threonine phosphatase activity;metal ion binding;protein dephosphorylation	ec:3.1.3.16-phosphatase;ec:3. 1.3.41-nitrophenyl phosphatase;ec:3. 1	T cell receptor signaling pathwayAminobenzoat e degradation

Phvul.002G166600	probable methyltransferase pmt27	endomembrane system;intracellular membrane-bounded organelle;cytoplasmic part;methyltransferase activity;methylation		
Phvul.002G170200	nac domain-containing protein 29	nucleus;DNA binding;transcription, DNA-templated;regulation of transcription, DNA-templated		
Phvul.002G170800	ribonuclease 1	extracellular region;cell wall;plasma membrane;RNA binding;ribonuclease T2 activity;anthocyanin-containing compound biosynthetic process;proline transport;RNA phosphodiester bond hydrolysis, endonucleolytic	ec:3.1.31;ec:3.1.27.1;ec:3.1.27;ec:3.1	
Phvul.002G180900	bon1-associated-like protein			
Phvul.002G181200	type i inositol- -trisphosphate 5-phosphatase cvp2	phosphatidylinositol-4,5-bisphosphate 5-phosphatase activity;protein dephosphorylation;glucuronoxylan metabolic process;xylan biosynthetic process;phosphatidylinositol dephosphorylation	ec:3.1.3.41-nitrophenyl phosphatase;ec:3.1;ec:3.1.3.36-5-phosphatase	Aminobenzoate degradationInositol phosphate metabolism;Phosphatidylinositol signaling system
Phvul.002G184600	transcription factor myb108-like	DNA binding		
Phvul.002G185800	glutamate decarboxylase 1	glutamate decarboxylase activity;pyridoxal phosphate binding;glutamate metabolic process	ec:4.1.1.15-decarboxylase	Taurine and hypotaurine metabolism;beta-Alanine metabolism;Butanoate metabolism;Alanine, aspartate and glutamate metabolism
Phvul.002G186000	glutamate decarboxylase 1	glutamate decarboxylase activity;pyridoxal phosphate binding;glutamate metabolic process	ec:4.1.1.15-decarboxylase	Taurine and hypotaurine metabolism;beta-Alanine metabolism;Butanoate metabolism;Alanine, aspartate and glutamate metabolism
Phvul.002G186600	map3k-like kinase	protein serine/threonine kinase activity;ATP binding;protein phosphorylation	ec:2.7.11	
Phvul.002G187800	cytochrome p450 family 81 protein	endoplasmic reticulum membrane;integral component of membrane;iron ion binding;heme binding;isoflavone 2'-hydroxylase activity;4'-methoxyisoflavone 2'-hydroxylase activity;aromatase activity;oxidation-reduction process	ec:1.14.13.53;ec:1.14.14;ec:1.14.13;ec:1.14.13.89-2'-hydroxylase;ec:1.14.14.1-monooxygenase	Isoflavonoid biosynthesisLinoleic acid metabolism;Arachidonic acid metabolism;Drug metabolism - cytochrome P450;Metabolism of xenobiotics by cytochrome P450;Caffeine metabolism;Aminobenzoate degradation;Tryptophan metabolism;Fatty acid degradation;Retinol metabolism;Steroid hormone biosynthesis
Phvul.002G197800	stress up-regulated nod 19 protein			
Phvul.002G199000	fad-binding berberine family protein	UDP-N-acetylmuramate dehydrogenase activity;flavin adenine dinucleotide binding;oxidation-reduction process	ec:1.1.1.158	
Phvul.002G199700	cannabidiolic acid synthase-like 2-like	UDP-N-acetylmuramate dehydrogenase activity;flavin adenine dinucleotide binding;oxidation-reduction process	ec:1.1.1.158	
Phvul.002G200600	cannabidiolic acid synthase-like 2-like	UDP-N-acetylmuramate dehydrogenase activity;flavin adenine dinucleotide binding;oxidation-reduction process	ec:1.1.1.158	
Phvul.002G204900	histidine decarboxylase	cell part;histidine decarboxylase activity;pyridoxal phosphate binding;phenylalanine decarboxylase activity;carboxylic acid metabolic process	ec:4.1.1.53-decarboxylase;ec:4.1.1.22-decarboxylase	Phenylalanine metabolismHistidine metabolism

Phvul.002G208900	hypothetical protein PHAVU_002G208900g			
Phvul.002G209400	stress-induced protein sam22	defense response;response to biotic stimulus		
Phvul.002G209500	stress-induced protein sam22	defense response;response to biotic stimulus		
Phvul.002G209900	ice-like protease p20 domain protein	cysteine-type endopeptidase activity;proteolysis	ec:3.4.22;ec:3.4	
Phvul.002G210400	abc transporter g family member 11	membrane;ATP binding;organic phosphonate transmembrane-transporting ATPase activity;metabolic process;organic phosphonate transport;organophosphate ester transport	ec:3.6.3.28;ec:3.6.1.3-adenylpyrophosphatase;ec:3.6.1;ec:3.6.1.15-phosphatase	Purine metabolismPurine metabolism;Thiamine metabolism
Phvul.002G210500	abc transporter g family member 11	membrane;ATP binding;ATPase activity;metabolic process;single-organism transport;organic substance transport	ec:3.6.1.3-adenylpyrophosphatase;ec:3.6.1;ec:3.6.1.15-phosphatase	Purine metabolismPurine metabolism;Thiamine metabolism
Phvul.002G215100	serine-threonine protein plant-	integral component of membrane;protein serine/threonine kinase activity;ATP binding;oxidoreductase activity;protein phosphorylation	ec:2.7.11	
Phvul.002G218300	phosphosulfolactate synthase	catalytic activity;protein folding;response to high light intensity;heat acclimation;posttranscriptional regulation of gene expression;coenzyme M biosynthetic process;response to hydrogen peroxide		
Phvul.002G219100	( )-nerolidol synthase 1-like	plastid;magnesium ion binding;terpene synthase activity;metabolic process		
Phvul.002G220300	hydrolyzing o-glycosyl compounds hydrolase	hydrolase activity, hydrolyzing O-glycosyl compounds;carbohydrate binding;carbohydrate metabolic process		
Phvul.002G223400	internal alternative nad h-ubiquinone oxidoreductase mitochondrial-like	NADH dehydrogenase (ubiquinone) activity;oxidation-reduction process	ec:1.6.5.3-reductase (H+-translocating);ec:1.6.99.5;ec:1.6.99.3-dehydrogenase	Oxidative phosphorylationOxidative phosphorylation
Phvul.002G228400	heat stress transcription factor b-2a-like	nucleus;transcription factor activity, sequence-specific DNA binding;sequence-specific DNA binding;regulation of transcription, DNA-templated;response to heat		
Phvul.002G228700	linoleate 13s-lipoxygenase 3-chloroplastic-like	oxidoreductase activity, acting on single donors with incorporation of molecular oxygen, incorporation of two atoms of oxygen;metal ion binding;oxylipin biosynthetic process;oxidation-reduction process	ec:1.13.11	
Phvul.002G231400	kda class i heat shock protein	cytoplasm		
Phvul.002G231500	kda class i heat shock protein	cytoplasm		
Phvul.002G231600	kda class i heat shock protein	cytoplasm		
Phvul.002G231700	kda class i heat shock protein	cytoplasm		
Phvul.002G231800	kda class i heat shock protein	cytoplasm		
Phvul.002G232800	protein srg1	gibberellin 3-beta-dioxygenase activity;flavonol synthase activity;metal ion binding;flavonol biosynthetic process;oxidation-reduction process	ec:1.14.11.15-3beta-dioxygenase;ec:1.14.11;ec:1.14.11.23-synthase	Diterpenoid biosynthesisFlavonoid biosynthesis
Phvul.002G235100	duf761 domain protein			
Phvul.002G239300	papain family cysteine protease	cysteine-type peptidase activity;proteolysis	ec:3.4	
Phvul.002G240100	leucine-rich repeat family protein	kinase activity;phosphorylation		
Phvul.002G241400	tau class glutathione s-transferase	glutathione transferase activity;metabolic process	ec:2.5.1.18-transferase	Drug metabolism - cytochrome P450;Metabolism of xenobiotics by cytochrome

				P450;Glutathione metabolism
Phvul.002G 244900	sulfite exporter family protein	integral component of membrane		
Phvul.002G 249300	serine threonine-protein kinase srk2a	protein kinase activity;ATP binding;protein phosphorylation		
Phvul.002G 256900	zinc finger protein stop1 homolog	nucleic acid binding;metal ion binding		
Phvul.002G 270900	probable pectinesterase pectinesterase inhibitor 51	cell wall;enzyme inhibitor activity;pectinesterase activity;aspartyl esterase activity;cell wall modification;negative regulation of catalytic activity;pectin catabolic process	ec:3.1.1.11-pectin demethoxylase;ec:3.1.1.1-ali-esterase	Starch and sucrose metabolism;Pentose and glucuronate interconversionsDrug metabolism - other enzymes
Phvul.002G 271900	coiled-coil domain-containing protein isoform partial			
Phvul.002G 274900	hypothetical protein PHAVU_002G274900g	integral component of membrane		
Phvul.002G 280400	nfu1 iron-sulfur mitochondrial	protein folding;response to heat;response to high light intensity;response to hydrogen peroxide		
Phvul.002G 284700	saur-like auxin-responsive family protein	response to auxin		
Phvul.002G 285800	probable wrky transcription factor 75	transcription factor activity, sequence-specific DNA binding;sequence-specific DNA binding;response to ethylene;cellular response to phosphate starvation;galactolipid biosynthetic process;regulation of response to nutrient levels;cellular response to water deprivation;regulation of DNA-templated transcription in response to stress;lateral root development		
Phvul.002G 287300	clavata3 esr gene family member			
Phvul.002G 306400	inositol oxygenase 2-like	cytoplasm;iron ion binding;inositol oxygenase activity;inositol catabolic process;oxidation-reduction process	ec:1.13.99.1-oxygenase	Inositol phosphate metabolism;Ascorbate and aldarate metabolism
Phvul.002G 306800	6-phosphofructokinase 3	cytoplasm;6-phosphofructokinase activity;ATP binding;metal ion binding;fructose 6-phosphate metabolic process;glycolytic process;carbohydrate phosphorylation	ec:2.7.1;ec:2.7.1.11-phosphohexokinase	Biosynthesis of antibiotics;Pentose phosphate pathway;Methane metabolism;Glycolysis / Gluconeogenesis;Galactose metabolism;Fructose and mannose metabolism
Phvul.002G 308000	pectinesterase 53	cell wall;cytoplasm;lyase activity;pectinesterase activity;aspartyl esterase activity;response to UV;flavonoid biosynthetic process;cell wall modification	ec:3.1.1.11-pectin demethoxylase;ec:3.1.1.1-ali-esterase	Starch and sucrose metabolism;Pentose and glucuronate interconversionsDrug metabolism - other enzymes
Phvul.002G 309100	protein phosphatase 2c 56-like	protein serine/threonine phosphatase activity;metal ion binding;protein dephosphorylation	ec:3.1.3.16-phosphatase;ec:3.1.3.41-nitrophenyl phosphatase;ec:3.1	T cell receptor signaling pathwayAminobenzoate degradation
Phvul.002G 314600	heavy metal transport detoxification superfamily protein	metal ion binding;metal ion transport		
Phvul.002G 316100	zinc finger protein constans-like 16	intracellular;zinc ion binding		
Phvul.002G 316900	transcription factor bee 1-like	nucleus;transcription factor activity, sequence-specific DNA binding;protein dimerization activity;regulation of transcription, DNA-templated		

Phvul.002G317000	transcription factor myb21	DNA binding		
Phvul.002G319000	protein plant cadmium resistance 2-like			
Phvul.002G319200	protein plant cadmium resistance 2-like			
Phvul.002G320400	protein xap5 circadian timekeeper isoform x2	nucleus;response to blue light;ethylene-activated signaling pathway;regulation of photomorphogenesis;response to red light;regulation of circadian rhythm		
Phvul.002G320600	hypothetical protein PHAVU_002G320600g			
Phvul.002G322300	two-component response regulator arr2	intracellular;DNA binding;phosphorelay signal transduction system		
Phvul.002G324400	clathrin assembly protein at2g25430	clathrin-coated vesicle;1-phosphatidylinositol binding;oxidoreductase activity;clathrin binding;clathrin coat assembly		
Phvul.002G325400	disease resistance-dirigent domain protein			
Phvul.002G326600	1-aminocyclopropane-1-carboxylate oxidase	1-aminocyclopropane-1-carboxylate oxidase activity;L-ascorbic acid binding;naringenin 3-dioxygenase activity;metal ion binding;ethylene biosynthetic process;fruit ripening;oxidation-reduction process	ec:1.14.11;ec:1.14.17.4-oxidase;ec:1.14.11.9-3-dioxygenase	Cysteine and methionine metabolismFlavonoid biosynthesis
Phvul.002G326700	mate efflux family protein	integral component of membrane;drug transmembrane transporter activity;antiporter activity;drug transmembrane transport		
Phvul.002G331800	u-box domain-containing protein 21-like	ubiquitin-protein transferase activity;ligase activity;protein ubiquitination		
Phvul.002G332300	monoglyceride lipase-like	acylglycerol lipase activity;metabolic process	ec:3.1.1.23-lipase;ec:3.1.1.1-ali-esterase;ec:3.1.1	Glycerolipid metabolismDrug metabolism - other enzymes
Phvul.003G002500	hypothetical protein PHAVU_003G002500g	ADP binding		
Phvul.003G002800	hypothetical protein PHAVU_003G002800g	ADP binding		
Phvul.003G007200	dishevelled egl-10 leckstrin domain protein	intracellular;electron carrier activity;protein disulfide oxidoreductase activity;intracellular signal transduction;cell redox homeostasis;oxidation-reduction process		
Phvul.003G007300	phototropic-responsive nph3 family protein			
Phvul.003G012600	plastocyanin-like domain protein	electron carrier activity		
Phvul.003G015600	probable pectinesterase 29	cell wall;pectinesterase activity;aspartyl esterase activity;metabolic process;cell wall modification	ec:3.1.1.11-pectin demethoxylase;ec:3.1.1.1-ali-esterase	Starch and sucrose metabolism;Pentose and glucuronate interconversionsDrug metabolism - other enzymes
Phvul.003G016400	probable s-adenosylmethionine-dependent methyltransferase at5g38100	methyltransferase activity;methylation		
Phvul.003G024700	abc transporter g family member 21	membrane;ATP binding;organic phosphonate transmembrane-transporting ATPase activity;metabolic process;organic phosphonate transport;organophosphate ester transport	ec:3.6.3.28;ec:3.6.1.3-adenylpyrophosphatase;ec:3.6.1;ec:3.6.1.15-phosphatase	Purine metabolismPurine metabolism;Thiamine metabolism

Phvul.003G027000	pectinesterase-like protein	cell wall;pectinesterase activity;aspartyl esterase activity;metabolic process;cell wall modification	ec:3.1.1.11-pectin demethoxylase;ec:3.1.1.1-ali-esterase	Starch and sucrose metabolism;Pentose and glucuronate interconversionsDrug metabolism - other enzymes
Phvul.003G029400	hypothetical protein PHAVU_003G029400g	integral component of membrane		
Phvul.003G030500	s1 p1 nuclease family protein	nucleic acid binding;endonuclease activity;DNA catabolic process;nucleic acid phosphodiester bond hydrolysis	ec:3.1	
Phvul.003G031300	3-ketoacyl-synthase 6	membrane;transferase activity, transferring acyl groups other than amino-acyl groups;fatty acid biosynthetic process		
Phvul.003G034200	duf1262 family protein			
Phvul.003G036600	long chain acyl-synthetase 2	endoplasmic reticulum;plasmodesma;long-chain fatty acid-CoA ligase activity;very long-chain fatty acid-CoA ligase activity;long-chain fatty acid metabolic process;cutin biosynthetic process;lateral root formation;defense response to fungus	ec:6.2.1.3-ligase;ec:6.2.1	Fatty acid degradation;Fatty acid biosynthesis
Phvul.003G041200	cytochrome p450 78a5-like	endoplasmic reticulum;monooxygenase activity;iron ion binding;oxidoreductase activity, acting on paired donors, with incorporation or reduction of molecular oxygen;heme binding;positive regulation of cell proliferation;regulation of meristem growth;leaf formation;organ growth;regulation of growth rate;positive regulation of organ growth;floral organ development;oxidation-reduction process		
Phvul.003G044600	formiminotransferase-cyclodeaminase-like protein	folic acid binding;glutamate formimidoyltransferase activity;metabolic process	ec:2.1.2;ec:2.1.2.5-formimidoyltransferase	One carbon pool by folate;Histidine metabolism
Phvul.003G048700	transcription termination factor family protein	mitochondrion;double-stranded DNA binding;regulation of transcription, DNA-templated		
Phvul.003G049800	phytochrome kinase substrate	kinase activity;lyase activity;phosphorylation		
Phvul.003G051700	isoflavone synthase 1	monooxygenase activity;iron ion binding;oxidoreductase activity, acting on paired donors, with incorporation or reduction of molecular oxygen;isomerase activity;heme binding;oxidation-reduction process		
Phvul.003G051800	isoflavone synthase 1	monooxygenase activity;iron ion binding;oxidoreductase activity, acting on paired donors, with incorporation or reduction of molecular oxygen;isomerase activity;heme binding;oxidation-reduction process		
Phvul.003G051900	plastocyanin-like domain protein	electron carrier activity		
Phvul.003G055500	gibberellin-regulated family protein			
Phvul.003G057900	cle1 protein			
Phvul.003G058700	u-box domain-containing protein 7-like			
Phvul.003G064300	protein transparent testa 12-like	integral component of membrane;drug transmembrane transporter activity;antiporter activity;drug transmembrane transport		
Phvul.003G064500	70 kda peptidyl-prolyl isomerase-like	peptidyl-prolyl cis-trans isomerase activity;protein peptidyl-prolyl isomerization;protein folding	ec:5.2.1.8	
Phvul.003G071000	pentatricopeptide repeat-containing protein mitochondrial	chloroplast;endonuclease activity;mRNA modification;nucleic acid phosphodiester bond hydrolysis	ec:3.1	
Phvul.003G074000	isoflavone synthase 1	monooxygenase activity;iron ion binding;oxidoreductase activity, acting on paired donors, with incorporation or reduction of molecular oxygen;isomerase activity;heme binding;oxidation-reduction process		

Phvul.003G 077800	hypothetical protein PHAVU_003G077 800g	DNA binding		
Phvul.003G 078100	hypothetical protein PHAVU_003G078 100g			
Phvul.003G 079800	5 -adenylylsulfate reductase chloroplastic-like	cell;adenylyl-sulfate reductase (glutathione) activity;sulfate reduction;cell redox homeostasis	ec:1.8.4.9-reductase (glutathione)	Sulfur metabolism
Phvul.003G 087500	probable adp-ribosylation factor gtpase-activating protein agd13	vacuole;plasma membrane;phosphatidylserine decarboxylase activity;phospholipase A2 activity;metabolic process	ec:4.1.1.65-decarboxylase;ec:3.1.1.4-A2;ec:3.1.1.1-ali-esterase;ec:3.1.1	Glycerophospholipid metabolismalpha-Linolenic acid metabolism;Linoleic acid metabolism;Arachidonic acid metabolism;Ether lipid metabolism;Glycerophospholipid metabolismDrug metabolism - other enzymes
Phvul.003G 087700	web family protein at1g12150-like			
Phvul.003G 089500	transmembrane protein 64			
Phvul.003G 092100	basic 7s globulin	aspartic-type endopeptidase activity;proteolysis	ec:3.4.23	
Phvul.003G 093100	adenylate isopentenyltransferase	AMP dimethylallyltransferase activity;tRNA processing	ec:2.5.1.27-dimethylallyltransferase	Zeatin biosynthesis
Phvul.003G 096700	late embryogenesis abundant protein lea5	response to stress		
Phvul.003G 098600	tryptophan aminotransferase-related protein 4	carbon-sulfur lyase activity;metabolic process		
Phvul.003G 099100	inosine-uridine preferring nucleoside hydrolase	xanthine dehydrogenase activity;hydrolase activity;oxidation-reduction process	ec:1.17.1.4-dehydrogenase;ec:1.17.1	Purine metabolism
Phvul.003G 108400	mono di-acylglycerol amino-terminal	hydrolase activity;lipid catabolic process		
Phvul.003G 109000	pathogenesis-related protein bet v i family protein	defense response;response to biotic stimulus		
Phvul.003G 109200	stress-induced protein sam22	defense response;response to biotic stimulus		
Phvul.003G 109600	stress-induced protein sam22	defense response;response to biotic stimulus		
Phvul.003G 109800	stress-induced protein sam22	defense response;response to biotic stimulus		
Phvul.003G 118300	gibberellin 2-beta-dioxygenase 2-like	iron ion binding;gibberellin 2-beta-dioxygenase activity;oxidation-reduction process;organic substance metabolic process	ec:1.14.11.13-2beta-dioxygenase;ec:1.14.11	Diterpenoid biosynthesis
Phvul.003G 118400	alkaline neutral invertase cinv2-like	mitochondrion;chloroplast;sucrose alpha-glucosidase activity;glycopeptide alpha-N-acetylgalactosaminidase activity;ethylene biosynthetic process;regulation of seed germination;regulation of timing of transition from vegetative to reproductive phase	ec:3.2.1.97;ec:3.2.1;ec:3.2.1.48-alpha-glucosidase;ec:3.2.1.26-invertase	Starch and sucrose metabolismStarch and sucrose metabolism;Galactose metabolism
Phvul.003G 120100	double clp-n motif p-loop nucleoside triphosphate hydrolase superfamily protein			
Phvul.003G 120900	hypothetical protein	nucleus		

	PHAVU_003G120900g			
Phvul.003G121900	group 3 lea protein			
Phvul.003G122700	abc transporter b family protein	integral component of membrane;ATP binding;ATPase activity, coupled to transmembrane movement of substances;metabolic process;transmembrane transport	ec:3.6.1.3-adenylpyrophosphatase;ec:3.6.1;ec:3.6.1.15-phosphatase	Purine metabolismPurine metabolism;Thiamine metabolism
Phvul.003G124100	cytidine deoxycytidylate deaminase family protein	cytosine deaminase activity;zinc ion binding;metabolic process	ec:3.5.4.1-deaminase	Arginine and proline metabolism;Pyrimidine metabolism
Phvul.003G124300	hypothetical protein PHAVU_003G124300g	binding		
Phvul.003G126300	lysine-ketoglutarate reductase saccharopine dehydrogenase	cytosol;saccharopine dehydrogenase (NADP+, L-glutamate-forming) activity;saccharopine dehydrogenase (NADP+, L-lysine-forming) activity;response to sucrose;response to fructose;vegetative to reproductive phase transition of meristem;protein desumoylation;L-lysine catabolic process;hydrogen peroxide biosynthetic process;oxidation-reduction process	ec:1.5.1.10-dehydrogenase (NADP+, L-glutamate-forming);ec:1.5.1;ec:1.5.1.8-dehydrogenase (NADP+, L-lysine-forming)	Biosynthesis of antibiotics;Lysine degradation;Lysine biosynthesisBiosynthesis of antibiotics;Lysine degradation
Phvul.003G126600	pectinesterase pectinesterase inhibitor	cell wall;enzyme inhibitor activity;pectinesterase activity;aspartyl esterase activity;cell wall modification;negative regulation of catalytic activity;pectin catabolic process	ec:3.1.1.11-pectin demethoxylase;ec:3.1.1.1-ali-esterase	Starch and sucrose metabolism;Pentose and glucuronate interconversionsDrug metabolism - other enzymes
Phvul.003G128400	hypothetical protein PHAVU_003G128400g			
Phvul.003G129200	protein tify 10a-like			
Phvul.003G131400	auxin-binding protein abp19a	nucleus;plant-type cell wall;extracellular matrix;apoplast;manganese ion binding;nutrient reservoir activity;auxin-activated signaling pathway;stomatal complex morphogenesis;photosynthesis, light reaction;cellular cation homeostasis;defense response to bacterium;divalent metal ion transport		
Phvul.003G131500	12-oxophytodienoate reductase 3	peroxisome;FMN binding;12-oxophytodienoate reductase activity;tryptophan catabolic process;response to water deprivation;response to wounding;indoleacetic acid biosynthetic process;jasmonic acid biosynthetic process;response to ethylene;response to auxin;abscisic acid-activated signaling pathway;jasmonic acid mediated signaling pathway;hyperosmotic salinity response;oxidation-reduction process	ec:1.3.1.42-reductase	alpha-Linolenic acid metabolism
Phvul.003G133400	membrane protein			
Phvul.003G135400	heat stress transcription factor a-2-like	nucleus;transcription factor activity, sequence-specific DNA binding;sequence-specific DNA binding;regulation of transcription, DNA-templated;response to heat		
Phvul.003G135700	transcription factor bhlh041	protein dimerization activity		
Phvul.003G138800	thioredoxin superfamily protein isoform 1			
Phvul.003G138900	mitochondrial ribosomal protein l37 isoform 1	ribosome		
Phvul.003G140800	transcription factor bhlh35	DNA binding;amino acid binding;protein dimerization activity;metabolic process		
Phvul.003G146700	probable beta- -galactosyltransferase 16	Golgi apparatus;integral component of membrane;galactosyltransferase activity;protein glycosylation	ec:2.4.1	
Phvul.003G147100	zinc finger protein 2	nucleic acid binding;metal ion binding		

Phvul.003G148600	cbl-interacting serine threonine-protein kinase 21 isoform x1	protein serine/threonine kinase activity;ATP binding;protein phosphorylation;signal transduction	ec:2.7.11	
Phvul.003G148900	1-deoxy-d-xylulose-5-phosphate synthase	1-deoxy-D-xylulose-5-phosphate synthase activity;terpenoid biosynthetic process	ec:2.2.1.7-synthase	Biosynthesis of antibiotics;Terpenoid backbone biosynthesis;Thiamine metabolism
Phvul.003G154800	heat shock 70 kda protein	cell wall;plasma membrane;chloroplast;ATP binding;2-alkenal reductase [NAD(P)] activity;protein folding;response to high light intensity;heat acclimation;response to endoplasmic reticulum stress;response to hydrogen peroxide;oxidation-reduction process	ec:1.3.1.74	
Phvul.003G160900	plant msj11-3			
Phvul.003G163400	lactoylglutathione lyase glyoxalase i family protein	transferase activity;lyase activity;metabolic process		
Phvul.003G164000	protein nrt1 ptr family	cell;integral component of membrane;transporter activity;oligopeptide transport;cellular cation homeostasis;divalent metal ion transport		
Phvul.003G167700	auxin-responsive protein iaa29-like	nucleus;transcription, DNA-templated;regulation of transcription, DNA-templated;auxin-activated signaling pathway		
Phvul.003G173600	hypothetical protein PHAVU_003G173600g			
Phvul.003G176800	transcription factor divaricata	nucleus;DNA binding;transcription factor activity, sequence-specific DNA binding;regulation of transcription, DNA-templated;mRNA modification		
Phvul.003G177800	oleosin 1-like	monolayer-surrounded lipid storage body;integral component of membrane		
Phvul.003G178300	adenine nucleotide alpha hydrolases-like superfamily protein isoform 2	response to stress		
Phvul.003G181300	serine threonine-protein kinase rio2-like isoform x1			
Phvul.003G182600	adenine nucleotide alpha hydrolase superfamily protein	hydrolase activity;response to stress;metabolic process		
Phvul.003G184200	ninja-family protein afp3-like isoform x3			
Phvul.003G187200	probable lrr receptor-like serine threonine-protein kinase at1g74360	mitochondrion;integral component of membrane;MAP kinase kinase kinase activity;transmembrane receptor protein tyrosine kinase activity;ATP binding;MAPK cascade;activation of MAPKK activity;peptidyl-tyrosine phosphorylation	ec:2.7.10.1;ec:2.7.11.25;ec:2.7.11;ec:2.7.10	
Phvul.003G190400	r2r3-myb transcription factor	DNA binding		
Phvul.003G192800	protein nrt1 ptr family	membrane;transporter activity;transport		
Phvul.003G193400	peptide nitrate transporter	membrane;transporter activity;transport		
Phvul.003G199200	u-box domain-containing protein 19-like	ubiquitin-protein transferase activity;ligase activity;protein ubiquitination		
Phvul.003G207000	myosin heavy chain kinase b	transferase activity		
Phvul.003G209000	hypothetical protein PHAVU_003G209000g			
Phvul.003G209100	malate glyoxysomal	glyoxysome;malate synthase activity;glyoxylate cycle;tricarboxylic acid cycle	ec:2.3.3.9-synthase	Pyruvate metabolism;Glyoxylate

				and dicarboxylate metabolism
Phvul.003G 212600	mate efflux family protein 5-like	integral component of membrane;drug transmembrane transporter activity;antiporter activity;drug transmembrane transport		
Phvul.003G 212900	protein gar2-like			
Phvul.003G 217200	21 kda protein	enzyme inhibitor activity;pectinesterase activity;metabolic process;negative regulation of catalytic activity	ec:3.1.1.11-pectin demethoxylase;ec :3.1.1.1-ali-esterase	Starch and sucrose metabolism;Pentose and glucuronate interconversionsDrug metabolism - other enzymes
Phvul.003G 225700	cationic amino acid transporter chloroplastic-like	integral component of membrane;amino acid transmembrane transporter activity;amino acid transmembrane transport		
Phvul.003G 227500	invertase inhibitor	enzyme inhibitor activity;pectinesterase activity;metabolic process;negative regulation of catalytic activity	ec:3.1.1.11-pectin demethoxylase;ec :3.1.1.1-ali-esterase	Starch and sucrose metabolism;Pentose and glucuronate interconversionsDrug metabolism - other enzymes
Phvul.003G 228000	expansin-like b1	extracellular region		
Phvul.003G 228700	type i inositol- -trisphosphate 5-phosphatase 2	phosphatidylinositol-4,5-bisphosphate 5-phosphatase activity;inositol-polyphosphate 5-phosphatase activity;protein dephosphorylation;response to abscisic acid;seed germination;inositol trisphosphate metabolic process;inositol phosphate dephosphorylation;phosphatidylinositol dephosphorylation	ec:3.1.3.56-5-phosphatase;ec:3.1.3.41-nitrophenyl phosphatase;ec:3.1;ec:3.1.3.36-5-phosphatase;ec:3.1.3	Inositol phosphate metabolism;Phosphatidylinositol signaling systemAminobenzoate degradationInositol phosphate metabolism;Phosphatidylinositol signaling system
Phvul.003G 230300	seed maturation protein	embryo development		
Phvul.003G 233400	phytosulfokines 3-like	extracellular region;growth factor activity;cell proliferation		
Phvul.003G 235800	probable n-acetyltransferase hls1	N-acetyltransferase activity;metabolic process	ec:2.3.1	
Phvul.003G 236200	lateral organ boundaries domain protein			
Phvul.003G 238700	indole-3-acetic acid-amido synthetase			
Phvul.003G 239800	hypothetical protein PHAVU_003G239 800g			
Phvul.003G 243400	triacylglycerol lipase-like protein	feruloyl esterase activity;lipid metabolic process	ec:3.1.1.73;ec:3.1.1.1-ali-esterase	Drug metabolism - other enzymes
Phvul.003G 247100	disease resistance protein			
Phvul.003G 247400	disease resistance protein	ADP binding		
Phvul.003G 252500	probable polyol transporter 6	integral component of membrane;substrate-specific transmembrane transporter activity;transmembrane transport		
Phvul.003G 259200	cytochrome p450 84a1-like	monooxygenase activity;iron ion binding;oxidoreductase activity, acting on paired donors, with incorporation or reduction of molecular oxygen;heme binding;oxidation-reduction process		
Phvul.003G 264600	histidine kinase 1	intracellular;membrane;phosphorelay sensor kinase activity;osmosensor activity;histidine phosphotransfer kinase activity;phosphorelay signal transduction system;response to osmotic stress;response to water deprivation;stomatal complex patterning;seed maturation;peptidyl-histidine phosphorylation;signal transduction by protein phosphorylation	ec:2.7.3;ec:2.7.13.3	
Phvul.003G 267000	receptor-like protein kinase theseus 1	protein serine/threonine kinase activity;ATP binding;protein phosphorylation	ec:2.7.11	

Phvul.003G 268500	chitinase (class ib) hevein	chitinase activity;chitin binding;carbohydrate metabolic process;chitin catabolic process;cell wall macromolecule catabolic process	ec:3.2.1.14-chitodextrinase	Amino sugar and nucleotide sugar metabolism
Phvul.003G 268600	chitinase (class ib) hevein	chitinase activity;chitin binding;polysaccharide catabolic process;chitin catabolic process;defense response;cell wall macromolecule catabolic process	ec:3.2.1.14-chitodextrinase	Amino sugar and nucleotide sugar metabolism
Phvul.003G 269500	dirigent protein 19-like			
Phvul.003G 277900	late embryogenesis abundant protein			
Phvul.003G 278400	cytochrome p450 family aba 8 -hydroxylase	iron ion binding;(+)-abscisic acid 8'-hydroxylase activity;heme binding;oxidation-reduction process	ec:1.14.13;ec:1.14 .13.93-acid 8'-hydroxylase	Carotenoid biosynthesis
Phvul.003G 281200	protein strictosidine synthase-like 10-like	strictosidine synthase activity;biosynthetic process	ec:4.3.3.2-synthase	Indole alkaloid biosynthesis
Phvul.003G 283100	transcription factor jumonji family protein	catalytic activity;metabolic process		
Phvul.003G 287400	probable mannitol dehydrogenase	zinc ion binding;oxidoreductase activity;oxidation-reduction process		
Phvul.003G 287500	probable mannitol dehydrogenase	zinc ion binding;cinnamyl-alcohol dehydrogenase activity;sinapyl alcohol dehydrogenase activity;oxidation-reduction process	ec:1.1.1.195-dehydrogenase	Phenylpropanoid biosynthesis
Phvul.004G 005400	cytochrome p450 86b1-like	monooxygenase activity;iron ion binding;oxidoreductase activity, acting on paired donors, with incorporation or reduction of molecular oxygen;heme binding;oxidation-reduction process		
Phvul.004G 008900	nbs-lrr type disease resistance protein cnl-j9	ADP binding		
Phvul.004G 009000	nbs-lrr type disease resistance protein cnl-j3	ADP binding		
Phvul.004G 012800	nbs-lrr type disease resistance protein cnl-b18	ADP binding		
Phvul.004G 013400	hypothetical protein			
Phvul.004G 014600	formate dehydrogenase	mitochondrion;chloroplast;thylakoid;formate dehydrogenase (NAD+) activity;oxidoreductase activity, acting on the CH-OH group of donors, NAD or NADP as acceptor;NAD binding;oxidation-reduction process	ec:1.2.1.2-dehydrogenase	Methane metabolism;Glyoxylate and dicarboxylate metabolism
Phvul.004G 014700	formate dehydrogenase	mitochondrion;chloroplast;thylakoid;formate dehydrogenase (NAD+) activity;oxidoreductase activity, acting on the CH-OH group of donors, NAD or NADP as acceptor;NAD binding;oxidation-reduction process	ec:1.2.1.2-dehydrogenase	Methane metabolism;Glyoxylate and dicarboxylate metabolism
Phvul.004G 015500	cyclin-like f-box			
Phvul.004G 018900	alpha-dioxygenase 1-like	peroxidase activity;heme binding;dioxygenase activity;response to oxidative stress;oxidation-reduction process	ec:1.11.1.7-lactoperoxidase	Phenylpropanoid biosynthesis
Phvul.004G 021200	cytochrome p450 family 82 protein	membrane;monooxygenase activity;iron ion binding;oxidoreductase activity, acting on paired donors, with incorporation or reduction of molecular oxygen;heme binding;oxidation-reduction process		
Phvul.004G 021300	cytochrome p450 82a4-like	monooxygenase activity;iron ion binding;oxidoreductase activity, acting on paired donors, with incorporation or reduction of molecular oxygen;heme binding;oxidation-reduction process		
Phvul.004G 021400	cytochrome p450 82a4-like	monooxygenase activity;iron ion binding;oxidoreductase activity, acting on paired donors, with incorporation or reduction of molecular oxygen;heme binding;oxidation-reduction process		
Phvul.004G 030500	expansin a10	extracellular region;cell wall;membrane;plant-type cell wall organization;unidimensional cell growth;primary root development		
Phvul.004G 037700	transcription factor tcp13			
Phvul.004G 041700	cytochrome p450 85a	integral component of membrane;monooxygenase activity;iron ion binding;oxidoreductase activity, acting on paired donors, with incorporation or reduction of		

		molecular oxygen;heme binding;oxidation-reduction process		
Phvul.004G 044800	lrr receptor-like kinase family protein			
Phvul.004G 045800	ocs element-binding factor 1	intracellular;transcription factor activity, sequence-specific DNA binding;MAP kinase activity;sequence-specific DNA binding;MAPK cascade;regulation of transcription, DNA-templated	ec:2.7.11.24-protein kinase;ec:2.7.11	mTOR signaling pathway
Phvul.004G 046200	calcium-transporting atpase plasma membrane-type	cell;integral component of membrane;calcium-transporting ATPase activity;ATP binding;metal ion binding;cellular zinc ion homeostasis;metabolic process;response to nematode;calcium ion transmembrane transport	ec:3.6.3.8;ec:3.6.1.3-adenylpyrophosphatase;ec:3.6.1;ec:3.6.1.15-phosphatase	Purine metabolismPurine metabolism;Thiamine metabolism
Phvul.004G 049900	protein pns1-like	integral component of membrane		
Phvul.004G 054800	triacylglycerol lipase 2	lipase activity;defense response to insect;fatty acid beta-oxidation	ec:3.1.1	
Phvul.004G 054900	triacylglycerol lipase-like protein	hydrolase activity;lipid catabolic process		
Phvul.004G 055200	calcium-binding protein cml38	intracellular;calcium ion binding;MAPK cascade;respiratory burst involved in defense response;protein targeting to membrane;ethylene biosynthetic process;salicylic acid biosynthetic process;systemic acquired resistance, salicylic acid mediated signaling pathway;jasmonic acid mediated signaling pathway;response to chitin;regulation of hydrogen peroxide metabolic process;regulation of plant-type hypersensitive response;endoplasmic reticulum unfolded protein response;negative regulation of defense response;defense response to bacterium;defense response to fungus		
Phvul.004G 056700	lysine histidine transporter 1-like	plasma membrane;integral component of membrane;amino acid transmembrane transporter activity;amino acid transmembrane transport;amino acid import		
Phvul.004G 058400	cystinosin homolog	membrane		
Phvul.004G 058500	retrovirus-related pol polyprotein from transposon tnt 1- partial	metal ion binding;metabolic process		
Phvul.004G 061400	mads-box transcription factor pheres 2			
Phvul.004G 068400	cytochrome p450 81e8-like	integral component of membrane;iron ion binding;heme binding;isoflavone 2'-hydroxylase activity;oxidation-reduction process	ec:1.14.13;ec:1.14.13.89-2'-hydroxylase	Isoflavonoid biosynthesis
Phvul.004G 070500	mitochondrial phosphate carrier protein mitochondrial-like	integral component of membrane;glutathione transferase activity;transport;metabolic process;response to salt stress;response to ethylene	ec:2.5.1.18-transferase	Drug metabolism - cytochrome P450;Metabolism of xenobiotics by cytochrome P450;Glutathione metabolism
Phvul.004G 071700	mlo-like protein 12	integral component of membrane;calmodulin binding;defense response;response to biotic stimulus		
Phvul.004G 072900	upf0481 protein at3g47200-like			
Phvul.004G 076400	calmodulin	cytosol;phosphorylase kinase activity;calcium ion binding;protein binding;2-alkenal reductase [NAD(P)] activity;detection of calcium ion;acetyl-CoA metabolic process;protein phosphorylation;response to mechanical stimulus;regulation of photomorphogenesis;calcium-mediated signaling;oxidation-reduction process	ec:1.3.1.74;ec:2.7.11.19;ec:2.7.11.17;ec:2.7.11	
Phvul.004G 077400	nac domain-containing protein 29	nucleus;DNA binding;transcription, DNA-templated;regulation of transcription, DNA-templated;single-organism process		
Phvul.004G 080200	glutamate dehydrogenase 1	oxidoreductase activity, acting on the CH-NH2 group of donors, NAD or NADP as acceptor;cellular amino acid metabolic process;oxidation-reduction process		
Phvul.004G 085100	sucrose transport protein suc8-like	integral component of plasma membrane;sucrose transmembrane transporter activity;sucrose transport		

Phvul.004G088400	tryptophan aminotransferase-related protein 4	transaminase activity;alliin lyase activity;metabolic process	ec:4.4.1.4	
Phvul.004G090200	methionine gamma-lyase-like	cytosol;cystathionine gamma-synthase activity;methionine gamma-lyase activity;pyridoxal phosphate binding;metabolic process;protein homotetramerization	ec:2.5.1.48-gamma-synthase;ec:4.4.1.11-gamma-lyase	Sulfur metabolism;Biosynthesis of antibiotics;Cysteine and methionine metabolism;Selenocompound metabolismCysteine and methionine metabolism;Selenocompound metabolism
Phvul.004G090300	transcription factor myc2-like	protein dimerization activity		
Phvul.004G092100	ethylene-responsive transcription factor abr1-like isoform x1	nucleus;DNA binding;transcription factor activity, sequence-specific DNA binding;transcription, DNA-templated;regulation of transcription, DNA-templated		
Phvul.004G099100	lrr receptor-like serine threonine-protein kinase gso2-like	kinase activity;phosphorylation		
Phvul.004G101500	acidic endochitinase-like	extracellular space;chitinase activity;polysaccharide catabolic process;chitin catabolic process	ec:3.2.1.14-chitodextrinase	Amino sugar and nucleotide sugar metabolism
Phvul.004G103900	isoflavone 7-o-glucosyltransferase 1-like	hydroquinone glucosyltransferase activity;metabolic process	ec:2.4.1;ec:2.4.1.218	
Phvul.004G106500	f-box fbd lrr-repeat protein at1g78750-like			
Phvul.004G107700	heat shock protein 83	ATP binding;unfolded protein binding;protein folding;response to stress		
Phvul.004G108900	beta-carotene hydroxylase	integral component of membrane;iron ion binding;oxidoreductase activity;fatty acid biosynthetic process;oxidation-reduction process		
Phvul.004G111600	transporter arsb	integral component of membrane;arsenite transmembrane transporter activity;arsenite transport;transmembrane transport		
Phvul.004G111900	transporter arsb	integral component of membrane;arsenite transmembrane transporter activity;arsenite transport;transmembrane transport		
Phvul.004G114300	cytochrome p450 family 72 protein	monooxygenase activity;iron ion binding;oxidoreductase activity, acting on paired donors, with incorporation or reduction of molecular oxygen;heme binding;oxidation-reduction process		
Phvul.004G114600	oxalate-- ligase	plasmodesma;chloroplast stroma;apoplast;o-succinylbenzoate-CoA ligase activity;4-coumarate-CoA ligase activity;oxalate-CoA ligase activity;positive regulation of seed germination;response to nitrate;seed coat development;nitrate transport;oxalate catabolic process;para-aminobenzoic acid metabolic process;defense response to fungus	ec:6.2.1.8-ligase;ec:6.2.1.26-ligase;ec:6.2.1.12-ligase	Glyoxylate and dicarboxylate metabolismUbiquinone and other terpenoid-quinone biosynthesisUbiquinone and other terpenoid-quinone biosynthesis;Phenylalanine metabolism;Phenylpropanoid biosynthesis
Phvul.004G115300	lrr receptor-like serine threonine-protein kinase gso2-like			
Phvul.004G116600	lrr receptor-like serine threonine-protein kinase gso2-like			
Phvul.004G117100	low-temperature-induced 65 kda			
Phvul.004G118200	e3 ubiquitin-protein ligase atl31-like	zinc ion binding		

Phvul.004G119400	hypothetical protein PHAVU_004G119400g			
Phvul.004G123600	gibberellin 20-oxidase	iron ion binding;gibberellin 3-beta-dioxygenase activity;gibberellin-44 dioxygenase activity;oxidation-reduction process	ec:1.14.11.15-3beta-dioxygenase;ec:1.14.11.12-dioxygenase;ec:1.14.11	Diterpenoid biosynthesisDiterpenoid biosynthesis
Phvul.004G124200	transcription factor	DNA binding;protein kinase activity;ATP binding;protein dimerization activity;protein phosphorylation		
Phvul.004G129400	kda class iv heat shock protein			
Phvul.004G131400	clathrin assembly protein at1g33340	clathrin-coated vesicle;1-phosphatidylinositol binding;clathrin binding;clathrin coat assembly		
Phvul.004G134400	uncharacterized oxidoreductase at4g09670-like	trans-1,2-dihydrobenzene-1,2-diol dehydrogenase activity;oxidation-reduction process	ec:1.3.1.20-dehydrogenase	Metabolism of xenobiotics by cytochrome P450
Phvul.004G134800	upf0481 protein at3g02645			
Phvul.004G138500	kunitz type trypsin inhibitor miraculin	endopeptidase inhibitor activity;negative regulation of endopeptidase activity		
Phvul.004G138600	gram domain protein aba-responsive-like protein			
Phvul.004G141300	blue copper	electron carrier activity;root hair cell differentiation		
Phvul.004G142000	organic cation carnitine transporter 1	integral component of membrane;transmembrane transporter activity;transmembrane transport		
Phvul.004G142400	lrr receptor-like serine threonine-protein kinase gso1			
Phvul.004G142500	lrr receptor-like serine threonine-protein kinase gso1			
Phvul.004G142600	lrr receptor-like serine threonine-protein kinase gso1	transferase activity		
Phvul.004G142700	lrr receptor-like serine threonine-protein kinase gso1	transferase activity		
Phvul.004G143600	beta-amylase chloroplastic	beta-amylase activity;polysaccharide catabolic process	ec:3.2.1;ec:3.2.1.2-saccharogen amylase	Starch and sucrose metabolism
Phvul.004G148400	uncharacterized protein LOC100527740			
Phvul.004G150400	pentatricopeptide repeat-containing protein mitochondrial-like			
Phvul.004G152900	ctd small phosphatase-like protein 2	phosphoprotein phosphatase activity;protein dephosphorylation	ec:3.1.3.16-phosphatase;ec:3.1.3.41-nitrophenyl phosphatase;ec:3.1	T cell receptor signaling pathwayAminobenzoate degradation
Phvul.004G155000	receptor-like kinase	protein serine/threonine kinase activity;transmembrane receptor protein tyrosine kinase activity;ATP binding;polysaccharide binding;2-alkenal reductase [NAD(P)] activity;peptidyl-tyrosine phosphorylation;oxidation-reduction process	ec:1.3.1.74;ec:2.7.10.1;ec:2.7.11;ec:2.7.10	
Phvul.004G155200	receptor-like kinase	protein serine/threonine kinase activity;transmembrane receptor protein tyrosine kinase activity;ATP binding;polysaccharide binding;2-alkenal reductase [NAD(P)] activity;peptidyl-tyrosine phosphorylation;oxidation-reduction process	ec:1.3.1.74;ec:2.7.10.1;ec:2.7.11;ec:2.7.10	

Phvul.004G 158800	maturation-associated protein	response to stress;response to water		
Phvul.004G 160300	atp-dependent clp protease adapter protein	peptidase activity;proteolysis;protein catabolic process		
Phvul.004G 162100	multiprotein-bridging factor 1c	nucleolus;cytoplasm;transcription factor activity, sequence-specific DNA binding;transcription coactivator activity;sequence-specific DNA binding;transcription, DNA-templated;protein folding;response to heat;response to water deprivation;response to high light intensity;response to abscisic acid;ethylene-activated signaling pathway;response to endoplasmic reticulum stress;response to hydrogen peroxide;positive regulation of transcription, DNA-templated		
Phvul.004G 168400	seed linoleate 9s-lipoxygenase	oxidoreductase activity, acting on single donors with incorporation of molecular oxygen, incorporation of two atoms of oxygen;metal ion binding;oxylipin biosynthetic process;oxidation-reduction process	ec:1.13.11	
Phvul.004G 173400	vacuolar protein sorting-associated protein 32 homolog 2-like	intracellular;vacuolar transport		
Phvul.004G 175200	probable pectinesterase 53	cell wall;cytoplasm;lyase activity;pectinesterase activity;aspartyl esterase activity;response to UV;flavonoid biosynthetic process;cell wall modification	ec:3.1.1.11-pectin demethoxylase;ec:3.1.1.1-ali-esterase	Starch and sucrose metabolism;Pentose and glucuronate interconversionsDrug metabolism - other enzymes
Phvul.005G 004400	fad-linked oxidoreductase	UDP-N-acetylmuramate dehydrogenase activity;flavin adenine dinucleotide binding;oxidation-reduction process	ec:1.1.1.158	
Phvul.005G 005400	anthocyanidin -o-glucosyltransferase	transferase activity, transferring hexosyl groups;metabolic process		
Phvul.005G 005500	anthocyanidin -o-glucosyltransferase	transferase activity, transferring hexosyl groups;metabolic process		
Phvul.005G 012000	pectinesterase pectinesterase inhibitor 24	cell wall;enzyme inhibitor activity;pectinesterase activity;aspartyl esterase activity;cell wall modification;negative regulation of catalytic activity;pectin catabolic process	ec:3.1.1.11-pectin demethoxylase;ec:3.1.1.1-ali-esterase	Starch and sucrose metabolism;Pentose and glucuronate interconversionsDrug metabolism - other enzymes
Phvul.005G 014000	disease resistance rpp13-like protein 1	hydrolase activity;ADP binding;metabolic process		
Phvul.005G 014600	ubiquitin-associated ts-n domain			
Phvul.005G 014900	cysteine-rich receptor-kinase-like protein	protein serine/threonine kinase activity;ATP binding;protein phosphorylation	ec:2.7.11	
Phvul.005G 015900	wat1-related protein at5g07050-like	integral component of membrane;transmembrane transporter activity;transmembrane transport		
Phvul.005G 016400	probable carboxylesterase 15	carboxylic ester hydrolase activity;metabolic process	ec:3.1.1.1-ali-esterase	Drug metabolism - other enzymes
Phvul.005G 018500	protein radialis-like 1-like	nucleus;DNA binding		
Phvul.005G 021600	mlp-like protein 31	defense response;response to biotic stimulus		
Phvul.005G 021900	mate efflux family protein	integral component of membrane;drug transmembrane transporter activity;antiporter activity;drug transmembrane transport		
Phvul.005G 022500	polygalacturonase-like	extracellular region;polygalacturonase activity;carbohydrate metabolic process;cell wall organization	ec:3.2.1.15-pectin depolymerase	Starch and sucrose metabolism;Pentose and glucuronate interconversions
Phvul.005G 029700	dna-directed rna polymerase v subunit 5c-like	nucleus;DNA binding;DNA-directed RNA polymerase activity;transcription, DNA-templated	ec:2.7.7.6-RNA polymerase	Purine metabolism;Pyrimidine metabolism

Phvul.005G032000	subtilisin inhibitor 1	serine-type endopeptidase inhibitor activity;response to wounding;negative regulation of endopeptidase activity		
Phvul.005G034000	cytokinin dehydrogenase 6	UDP-N-acetylmuramate dehydrogenase activity;cytokinin dehydrogenase activity;flavin adenine dinucleotide binding;cytokinin metabolic process;oxidation-reduction process	ec:1.5.99.12-dehydrogenase;ec:1.1.1.158	Zeatin biosynthesis
Phvul.005G035800	late embryogenesis abundant protein d-34-like			
Phvul.005G044200	seed maturation protein pm41	cytoplasm		
Phvul.005G046200	zinc finger protein zat11-like	nucleic acid binding;metal ion binding		
Phvul.005G047200	ankyrin repeat-containing protein at3g12360			
Phvul.005G049000	plant f3o9-12 protein			
Phvul.005G051600	9-cis-epoxycarotenoid dioxygenase	chloroplast thylakoid membrane;9-cis-epoxycarotenoid dioxygenase activity;metal ion binding;abscisic acid biosynthetic process;oxidation-reduction process	ec:1.13.11;ec:1.13.11.51-dioxygenase	Carotenoid biosynthesis
Phvul.005G053000	pentatricopeptide repeat-containing protein at1g20230-like	microtubule-severing ATPase activity;metabolic process	ec:3.6.1.3-adenylpyrophosphatase;ec:3.6.1;ec:3.6.4.3;ec:3.6.1.15-phosphatase	Purine metabolismPurine metabolism;Thiamine metabolism
Phvul.005G053400	cytochrome p450 87a3	iron ion binding;heme binding;taxane 10-beta-hydroxylase activity;oxidation-reduction process	ec:1.14.13.76-10beta-hydroxylase;ec:1.14.13	Diterpenoid biosynthesis
Phvul.005G053600	cytochrome p450 87a3-like	iron ion binding;heme binding;taxane 10-beta-hydroxylase activity;oxidation-reduction process	ec:1.14.13.76-10beta-hydroxylase;ec:1.14.13	Diterpenoid biosynthesis
Phvul.005G053900	glutathione s-amino-terminal domain protein	glutathione transferase activity;lactoylglutathione lyase activity;metabolic process	ec:2.5.1.18-transferase;ec:4.4.1.5-lyase	Drug metabolism - cytochrome P450;Metabolism of xenobiotics by cytochrome P450;Glutathione metabolismPyruvate metabolism
Phvul.005G054000	glutathione s-transferase	glutathione transferase activity;metabolic process	ec:2.5.1.18-transferase	Drug metabolism - cytochrome P450;Metabolism of xenobiotics by cytochrome P450;Glutathione metabolism
Phvul.005G054100	glutathione s-transferase	glutathione transferase activity;metabolic process	ec:2.5.1.18-transferase	Drug metabolism - cytochrome P450;Metabolism of xenobiotics by cytochrome P450;Glutathione metabolism
Phvul.005G054200	glutathione s-transferase	glutathione transferase activity;metabolic process	ec:2.5.1.18-transferase	Drug metabolism - cytochrome P450;Metabolism of xenobiotics by cytochrome P450;Glutathione metabolism
Phvul.005G055000	serine threonine-protein kinase	protein serine/threonine kinase activity;transmembrane receptor protein tyrosine kinase activity;non-membrane spanning protein tyrosine kinase activity;ATP binding;peptidyl-tyrosine phosphorylation;protein autophosphorylation	ec:2.7.10.2-protein-tyrosine kinase;ec:2.7.10.1;ec:2.7.11;ec:2.7.10	T cell receptor signaling pathway
Phvul.005G058900	nitrile-specifier protein 5			
Phvul.005G059100	feruloyl ortho-hydroxylase 1	iron ion binding;flavonol synthase activity;naringenin 3-dioxygenase activity;flavonol biosynthetic process;oxidation-reduction process	ec:1.14.11;ec:1.14.11.9-3-	Flavonoid biosynthesisFlavonoid biosynthesis

			dioxygenase;ec:1.14.11.23-synthase	
Phvul.005G065100	f-box fbd lrr-repeat protein at3g14710-like			
Phvul.005G075400	protein srg1	oxidoreductase activity, acting on paired donors, with incorporation or reduction of molecular oxygen, 2-oxoglutarate as one donor, and incorporation of one atom each of oxygen into both donors;metal ion binding;oxidation-reduction process	ec:1.14.11	
Phvul.005G075500	protein srg1-like	oxidoreductase activity, acting on paired donors, with incorporation or reduction of molecular oxygen, 2-oxoglutarate as one donor, and incorporation of one atom each of oxygen into both donors;metal ion binding;oxidation-reduction process	ec:1.14.11	
Phvul.005G077000	pgr5-like protein chloroplastic			
Phvul.005G078100	lrr receptor-like kinase	transmembrane receptor protein tyrosine kinase activity;ATP binding;peptidyl-tyrosine phosphorylation	ec:2.7.10.1;ec:2.7.11;ec:2.7.10	
Phvul.005G078400	probable leucine-rich repeat receptor-like serine threonine-protein kinase at3g14840	protein kinase activity;ATP binding;protein phosphorylation		
Phvul.005G080600	gamma-glutamyl hydrolase 2-like	extracellular space;cell wall;gamma-glutamyl-peptidase activity;proteolysis;glutamine metabolic process	ec:3.4.19.9-hydrolase;ec:3.4.19	Folate biosynthesis
Phvul.005G084500	nac transcription factor-like protein	nucleus;DNA binding;transcription, DNA-templated;regulation of transcription, DNA-templated		
Phvul.005G084700	callose synthase 10-like			
Phvul.005G096300	seed biotin-containing protein sbp65			
Phvul.005G097000	nitrogen-induced cle 1			
Phvul.005G097200	transcription factor tcp4-like	signal transduction;post-embryonic development;cellular component organization;regulation of cellular metabolic process;reproductive structure development;phyllome development		
Phvul.005G099500	fasciclin-like arabinogalactan protein 13			
Phvul.005G106500	indole-3-acetic acid-amido synthetase	chloroplast;jasmonate-amino synthetase activity;response to wounding;response to salt stress;jasmonic acid metabolic process;response to abscisic acid;induced systemic resistance, jasmonic acid mediated signaling pathway		
Phvul.005G108100	wound-induced protein			
Phvul.005G108800	anthocyanin 5-aromatic acyltransferase	shikimate O-hydroxycinnamoyltransferase activity;metabolic process	ec:2.3.1;ec:2.3.1.133-O-hydroxycinnamoyl transferase	Stilbenoid, diarylheptanoid and gingerol biosynthesis;Flavonoid biosynthesis;Phenylpropanoid biosynthesis
Phvul.005G109000	malonyl-coenzyme a:anthocyanin 3-o-glucoside-6 -o-malonyltransferase	shikimate O-hydroxycinnamoyltransferase activity;anthranilate N-benzoyltransferase activity;metabolic process	ec:2.3.1;ec:2.3.1.144;ec:2.3.1.133-O-hydroxycinnamoyl transferase	Stilbenoid, diarylheptanoid and gingerol biosynthesis;Flavonoid biosynthesis;Phenylpropanoid biosynthesis
Phvul.005G109700	myb-related protein myb4-like	DNA binding		
Phvul.005G111100	hypothetical protein PHAVU_005G111100g			
Phvul.005G111400	sesquiterpene synthase	magnesium ion binding;isoprene synthase activity;metabolic process	ec:4.2.3.27-synthase	Terpenoid backbone biosynthesis
Phvul.005G111500	sesquiterpene synthase	magnesium ion binding;isoprene synthase activity;metabolic process	ec:4.2.3.27-synthase	Terpenoid backbone biosynthesis

Phvul.005G 111700	sesquiterpene synthase	magnesium ion binding;isoprene synthase activity;metabolic process	ec:4.2.3.27-synthase	Terpenoid backbone biosynthesis
Phvul.005G 112700	cysteine-rich repeat secretory protein 38-like			
Phvul.005G 116500	cellulose synthase-like protein h1	integral component of membrane;cellulose synthase (UDP-forming) activity;cellulose biosynthetic process	ec:2.4.1.12-synthase (UDP-forming);ec:2.4.1	Starch and sucrose metabolism
Phvul.005G 116600	plant t7a14-6 protein			
Phvul.005G 118900	protein faf-chloroplastic			
Phvul.005G 121600	hypothetical protein PHAVU_005G121 600g	response to karrikin		
Phvul.005G 138400	hypothetical protein PHAVU_005G138 400g			
Phvul.005G 141500	zinc finger protein zat12	metal ion binding		
Phvul.005G 145600	hypothetical protein PHAVU_005G145 600g			
Phvul.005G 147600	aldehyde dehydrogenase family 3 member f1	aldehyde dehydrogenase [NAD(P)+] activity;cellular aldehyde metabolic process;oxidation-reduction process	ec:1.2.1.5-dehydrogenase [NAD(P)+]	Drug metabolism - cytochrome P450;Metabolism of xenobiotics by cytochrome P450;beta-Alanine metabolism;Glycolysis / Gluconeogenesis;Pheny lalanine metabolism;Tyrosine metabolism;Histidine metabolism
Phvul.005G 151600	glycoside hydrolase family 1 protein	beta-glucosidase activity;carbohydrate metabolic process	ec:3.2.1;ec:3.2.1.2 1-gentiobiase	Starch and sucrose metabolism;Cyanoamin o acid metabolism;Phenylpro panoid biosynthesis
Phvul.005G 155800	endochitinase pr4-like	chitinase activity;chitin binding;polysaccharide catabolic process;chitin catabolic process;defense response;cell wall macromolecule catabolic process	ec:3.2.1.14-chitodextrinase	Amino sugar and nucleotide sugar metabolism
Phvul.005G 156700	seed linoleate 9s-lipoxygenase-2	cytoplasm;linoleate 13S-lipoxygenase activity;metal ion binding;oxylipin biosynthetic process;oxidation-reduction process	ec:1.13.11.12-13S-lipoxygenase;ec:1. 13.11	alpha-Linolenic acid metabolism;Linoleic acid metabolism
Phvul.005G 158500	cell-wall invertase	sucrose alpha-glucosidase activity;carbohydrate metabolic process	ec:3.2.1;ec:3.2.1.4 8-alpha-glucosidase;ec:3.2 .1.26-invertase	Starch and sucrose metabolismStarch and sucrose metabolism;Galactose metabolism
Phvul.005G 163100	leucine-rich repeat receptor-like protein kinase	protein serine/threonine kinase activity;ATP binding;protein phosphorylation	ec:2.7.11	
Phvul.005G 166800	duf1677 family protein			
Phvul.005G 167800	mitochondrial carnitine acylcarnitine carrier protein cacl	mitochondrion;integral component of membrane;L-ornithine transmembrane transporter activity;L-histidine transmembrane transporter activity;arginine transmembrane transporter activity;L-lysine transmembrane transporter activity;proline biosynthetic process;L-arginine import;L-histidine transmembrane transport;L-ornithine transmembrane transport;L-lysine transmembrane transport;arginine transmembrane transport		
Phvul.005G 171900	coatomer subunit beta -1-like			
Phvul.005G 173000	auxin-responsive protein iaa1	nucleus;transcription, DNA-templated;regulation of transcription, DNA-templated;auxin-activated signaling pathway		

Phvul.005G173500	gibberellin 3-beta-dioxygenase 1-like	iron ion binding;gibberellin 3-beta-dioxygenase activity;gibberellin biosynthetic process;oxidation-reduction process	ec:1.14.11.15-3beta-dioxygenase;ec:1.14.11	Diterpenoid biosynthesis
Phvul.005G173600	drug resistance transporter-like abc domain protein	integral component of membrane;ATP binding;ATPase activity;transport;metabolic process	ec:3.6.1.3-adenylpyrophosphatase;ec:3.6.1;ec:3.6.1.15-phosphatase	Purine metabolismPurine metabolism;Thiamine metabolism
Phvul.005G174400	boron transporter 4	integral component of membrane;inorganic anion exchanger activity;anion transmembrane transport		
Phvul.005G174800	late embryogenesis abundant protein group 3 protein			
Phvul.005G181300	helix loop helix dna-binding domain protein	protein dimerization activity		
Phvul.005G181700	ds12 from 2d-page of leaf	amino acid binding;metabolic process		
Phvul.006G001900	protein tify 5a-like			
Phvul.006G004800	duf868 family protein			
Phvul.006G005100	probable e3 ubiquitin-protein ligase xerico	zinc ion binding;hydrolase activity;ligase activity;abscisic acid metabolic process		
Phvul.006G007700	hypothetical protein PHAVU_006G007700g			
Phvul.006G011600	l-ascorbate oxidase	extracellular region;copper ion binding;L-ascorbate oxidase activity;oxidation-reduction process	ec:1.10.3;ec:1.10.3.3-oxidase	Ascorbate and aldarate metabolism
Phvul.006G014000	hyp o-arabinosyltransferase homolog	transferase activity;metabolic process		
Phvul.006G016900	linoleate 13s-lipoxygenase 2-chloroplastic-like	oxidoreductase activity, acting on single donors with incorporation of molecular oxygen, incorporation of two atoms of oxygen;metal ion binding;oxylipin biosynthetic process;oxidation-reduction process	ec:1.13.11	
Phvul.006G023100	nac domain-containing protein 42	nucleus;DNA binding;transcription, DNA-templated;regulation of transcription, DNA-templated;response to stress;single-organism biosynthetic process;single-organism cellular process;organonitrogen compound biosynthetic process		
Phvul.006G023900	hxxxd-type acyl-transferase family protein	shikimate O-hydroxycinnamoyltransferase activity;metabolic process	ec:2.3.1;ec:2.3.1.133-O-hydroxycinnamoyltransferase	Stilbenoid, diarylheptanoid and gingerol biosynthesis;Flavonoid biosynthesis;Phenylpropanoid biosynthesis
Phvul.006G028700	protein transparent testa 12-like	integral component of membrane;drug transmembrane transporter activity;antiporter activity;drug transmembrane transport		
Phvul.006G033000	probable alpha-mannosidase i mns5 isoform x1	membrane;mannosyl-oligosaccharide 1,2-alpha-mannosidase activity;calcium ion binding;regulation of mitotic cell cycle;metabolic process	ec:3.2.1;ec:3.2.1.24-alpha-D-mannosidase;ec:3.2.1.113-1,2-alpha-mannosidase	Other glycan degradationVarious types of N-glycan biosynthesis;N-Glycan biosynthesis
Phvul.006G033300	g-type lectin s-receptor-like serine threonine-protein kinase rlk1	protein serine/threonine kinase activity;transmembrane receptor protein tyrosine kinase activity;ATP binding;carbohydrate binding;peptidyl-tyrosine phosphorylation	ec:2.7.10.1;ec:2.7.11;ec:2.7.10	
Phvul.006G043100	duf4228 domain protein			
Phvul.006G045800	gdsl-like lipase acylhydrolase	hydrolase activity, acting on ester bonds;9,9'-dicis-carotene:quinone oxidoreductase activity;7,9,9'-tricis-neurosporene:quinone oxidoreductase activity;9,9'-di-cis-zeta-carotene desaturation to 7,9,7',9'-tetra-cis-lycopene;oxidation-reduction process	ec:1.3.5.6-desaturase	Carotenoid biosynthesis
Phvul.006G046000	small heat shock chloroplastic-like			

Phvul.006G 054200	cytochrome p450 family 71 protein	iron ion binding;heme binding;flavonoid 3',5'-hydroxylase activity;oxidation-reduction process	ec:1.14.13;ec:1.14 .13.88-3',5'-hydroxylase	Flavone and flavonol biosynthesis;Flavonoid biosynthesis
Phvul.006G 054500	cytochrome p450 family 71 protein	iron ion binding;heme binding;flavonoid 3',5'-hydroxylase activity;oxidation-reduction process	ec:1.14.13;ec:1.14 .13.88-3',5'-hydroxylase	Flavone and flavonol biosynthesis;Flavonoid biosynthesis
Phvul.006G 056300	retrotransposon-like protein 1			
Phvul.006G 058600	cellulose synthase-like protein e1	plasma membrane;integral component of membrane;cellulose synthase (UDP-forming) activity;cellulose biosynthetic process;para-aminobenzoic acid metabolic process	ec:2.4.1.12-synthase (UDP-forming);ec:2.4.1	Starch and sucrose metabolism
Phvul.006G 060700	chaperone protein dnaj chloroplastic-like			
Phvul.006G 068600	microsomal omega-3 fatty acid desaturase	endoplasmic reticulum membrane;integral component of membrane;oxidoreductase activity, acting on paired donors, with oxidation of a pair of donors resulting in the reduction of molecular oxygen to two molecules of water;unsaturated fatty acid biosynthetic process;oxidation-reduction process	ec:1.14.19	
Phvul.006G 069300	asparagine synthetase	asparagine synthase (glutamine-hydrolyzing) activity;ATP binding;glutamine metabolic process;L-asparagine biosynthetic process	ec:6.3.5.4-synthase (glutamine-hydrolysing)	Alanine, aspartate and glutamate metabolism
Phvul.006G 075600	cationic peroxidase 1-like	extracellular region;peroxidase activity;heme binding;metal ion binding;response to oxidative stress;hydrogen peroxide catabolic process;oxidation-reduction process	ec:1.11.1.7-lactoperoxidase	Phenylpropanoid biosynthesis
Phvul.006G 075900	peroxidase 4-like	extracellular region;peroxidase activity;heme binding;metal ion binding;response to oxidative stress;hydrogen peroxide catabolic process;oxidation-reduction process	ec:1.11.1.7-lactoperoxidase	Phenylpropanoid biosynthesis
Phvul.006G 078800	cytochrome p450 partial	monooxygenase activity;iron ion binding;oxidoreductase activity, acting on paired donors, with incorporation or reduction of molecular oxygen;heme binding;oxidation-reduction process		
Phvul.006G 078900	cytochrome p450 partial	endoplasmic reticulum;integral component of membrane;organelle membrane;monooxygenase activity;iron ion binding;oxidoreductase activity, acting on paired donors, with incorporation or reduction of molecular oxygen;heme binding;cyanogenic glycoside biosynthetic process;oxidation-reduction process		
Phvul.006G 079100	cytochrome p450 partial	monooxygenase activity;iron ion binding;oxidoreductase activity, acting on paired donors, with incorporation or reduction of molecular oxygen;heme binding;oxidation-reduction process		
Phvul.006G 079500	oxalate-- ligase-like	plasmodesma;chloroplast stroma;apoplast;o-succinylbenzoate-CoA ligase activity;4-coumarate-CoA ligase activity;oxalate-CoA ligase activity;positive regulation of seed germination;response to nitrate;seed coat development;nitrate transport;oxalate catabolic process;para-aminobenzoic acid metabolic process;defense response to fungus	ec:6.2.1.8-ligase;ec:6.2.1.26-ligase;ec:6.2.1.12-ligase	Glyoxylate and dicarboxylate metabolismUbiquinone and other terpenoid-quinone biosynthesisUbiquinon e and other terpenoid-quinone biosynthesis;Phenylala nine metabolism;Phenylpro panoid biosynthesis
Phvul.006G 079600	probable glutathione s-transferase para	glutathione transferase activity;glutathione metabolic process;auxin-activated signaling pathway	ec:2.5.1.18-transferase	Drug metabolism - cytochrome P450;Metabolism of xenobiotics by cytochrome P450;Glutathione metabolism
Phvul.006G 080600	60s ribosomal protein l10	ribosome;structural constituent of ribosome;translation		
Phvul.006G 080700	metal tolerance protein 4 isoform 1	integral component of membrane;cation transmembrane transporter activity;cation transmembrane transport		
Phvul.006G 083700	protein root primordium defective 1-like			

Phvul.006G 083800	probable metal-nicotianamine transporter ysl7	plasma membrane;integral component of membrane;oligopeptide transporter activity;oligopeptide transport;amino acid transport;transmembrane transport		
Phvul.006G 084300	psbq-like protein chloroplastic	photosystem II oxygen evolving complex;extrinsic component of membrane;calcium ion binding;photosynthesis		
Phvul.006G 084600	cysteine-rich receptor-like protein kinase 2-like	protein serine/threonine kinase activity;transmembrane receptor protein tyrosine kinase activity;ATP binding;peptidyl-tyrosine phosphorylation	ec:2.7.10.1;ec:2.7.11;ec:2.7.10	
Phvul.006G 084900	cysteine-rich receptor-like protein kinase 2	protein serine/threonine kinase activity;transmembrane receptor protein tyrosine kinase activity;ATP binding;peptidyl-tyrosine phosphorylation	ec:2.7.10.1;ec:2.7.11;ec:2.7.10	
Phvul.006G 096200	protein bonzai 3	calcium-dependent phospholipid binding;negative regulation of cell death		
Phvul.006G 100700	b3 domain-containing protein at5g66980 isoform x1	nucleus;DNA binding;transcription, DNA-templated;regulation of transcription, DNA-templated		
Phvul.006G 102300	pathogenesis-related protein pr-4-like	chitin binding;defense response to bacterium;defense response to fungus		
Phvul.006G 109000	acidic endochitinase-like	chitinase activity;carbohydrate metabolic process	ec:3.2.1.14-chitodextrinase	Amino sugar and nucleotide sugar metabolism
Phvul.006G 112300	non-specific lipid-transfer protein a-like	lipid binding;lipid transport		
Phvul.006G 116000	homeobox-leucine zipper protein athb-12-like	nucleus;transcription factor activity, sequence-specific DNA binding;sequence-specific DNA binding;transcription, DNA-templated;regulation of transcription, DNA-templated		
Phvul.006G 116500	permease transmembrane protein	membrane		
Phvul.006G 117300	nicotianamine synthase	nicotianamine synthase activity;nicotianamine biosynthetic process	ec:2.5.1.43	
Phvul.006G 127500	probable 3-hydroxyisobutyrate mitochondrial isoform x1	phosphogluconate dehydrogenase (decarboxylating) activity;3-hydroxyisobutyrate dehydrogenase activity;NAD binding;valine catabolic process;oxidation-reduction process	ec:1.1.1.44-dehydrogenase (NADP+-dependent, decarboxylating);ec:1.1.1.31-dehydrogenase	Biosynthesis of antibiotics;Pentose phosphate pathway;Glutathione metabolismValine, leucine and isoleucine degradation
Phvul.006G 129500	peroxidase 15	extracellular region;peroxidase activity;heme binding;metal ion binding;response to oxidative stress;hydrogen peroxide catabolic process;oxidation-reduction process	ec:1.11.1.7-lactoperoxidase	Phenylpropanoid biosynthesis
Phvul.006G 133600	ferric reduction oxidase chloroplastic-like	integral component of membrane;NAD(P)H oxidase activity;oxidation-reduction process	ec:1.6.3.1	
Phvul.006G 136800	web family protein at1g12150-like			
Phvul.006G 137300	wall associated kinase-like protein	protein kinase activity;ATP binding;polysaccharide binding;protein phosphorylation		
Phvul.006G 139100	14 kda proline-rich			
Phvul.006G 143300	tnfr cd27 30 40 95 cysteine-rich region			
Phvul.006G 146900	plant t7a14-6 protein			
Phvul.006G 148400	f-box plant-like			
Phvul.006G 150900	serine threonine-protein kinase pbs1-like	protein serine/threonine kinase activity;ATP binding;protein phosphorylation	ec:2.7.11	
Phvul.006G 153100	extracellular dermal glycoprotein	aspartic-type endopeptidase activity;transferase activity;proteolysis	ec:3.4.23	
Phvul.006G 156100	beta-amyrin synthase	cycloartenol synthase activity;metabolic process	ec:5.4.99.8-synthase	Steroid biosynthesis

Phvul.006G 166000	hypothetical protein PHAVU_006G166 000g			
Phvul.006G 168000	btb poz domain-containing protein at5g03250-like			
Phvul.006G 168600	duf584 protein			
Phvul.006G 171000	major intrinsic protein family transporter	integral component of membrane;transporter activity;transport		
Phvul.006G 173000	b-cell lymphoma 6			
Phvul.006G 173600	wat1-related protein at3g30340-like	integral component of membrane;transmembrane transporter activity;transmembrane transport		
Phvul.006G 176500	olee1-like protein	extracellular space		
Phvul.006G 178300	3 -n-debenzoyl-2 -deoxytaxol n-benzoyltransferas e	cytoplasm;transferase activity, transferring acyl groups other than amino-acyl groups;response to wounding;systemic acquired resistance;coumarin biosynthetic process;positive regulation of flavonoid biosynthetic process;response to endoplasmic reticulum stress		
Phvul.006G 187500	asparagine synthetase	asparagine synthase (glutamine-hydrolyzing) activity;metabolic process	ec:6.3.5.4-synthase (glutamine-hydrolysing)	Alanine, aspartate and glutamate metabolism
Phvul.006G 188500	polyphenol oxidase chloroplastic	catechol oxidase activity;metal ion binding;pigment biosynthetic process;oxidation-reduction process	ec:1.10.3;ec:1.10. 3.1-oxidase	Isoquinoline alkaloid biosynthesis;Tyrosine metabolism
Phvul.006G 188900	nac transcription factor 25-like	nucleus;DNA binding;transcription factor activity, sequence-specific DNA binding;transcription, DNA-templated;regulation of transcription, DNA-templated		
Phvul.006G 189100	serine carboxypeptidase-like 31	serine-type carboxypeptidase activity;proteolysis	ec:3.4.21;ec:3.4.1 6	
Phvul.006G 190400	pap-specific phosphatase hal2-like	3'(2'),5'-bisphosphate nucleotidase activity;protein dephosphorylation;sulfur compound metabolic process;phosphatidylinositol phosphorylation	ec:3.1.3.7-nucleotidase;ec:3. 1.3.41-nitrophenyl phosphatase;ec:3. 1;ec:3.1.3.31	Sulfur metabolismAminobenz oate degradation
Phvul.006G 191200	cfe protein			
Phvul.006G 194200	beta-amyrin synthase	cycloartenol synthase activity;metabolic process	ec:5.4.99.8-synthase	Steroid biosynthesis
Phvul.006G 194600	receptor-like serine threonine-protein kinase sd1-8	protein serine/threonine kinase activity;ATP binding;carbohydrate binding;protein phosphorylation;recognition of pollen	ec:2.7.11	
Phvul.006G 197200	basic form of pathogenesis-related protein 1-like	extracellular region		
Phvul.006G 205300	protein exordium-like 2			
Phvul.006G 209000	clathrin assembly protein at1g03050	clathrin-coated vesicle;1-phosphatidylinositol binding;oxidoreductase activity;clathrin binding;clathrin coat assembly		
Phvul.006G 209500	cytochrome p450 family 71 protein	monooxygenase activity;iron ion binding;oxidoreductase activity, acting on paired donors, with incorporation or reduction of molecular oxygen;heme binding;oxidation-reduction process		
Phvul.006G 210300	wat1-related protein at1g68170-like	integral component of membrane;transmembrane transporter activity;transmembrane transport		
Phvul.006G 215400	like subfamily b member 13	unfolded protein binding;protein folding		
Phvul.006G 215700	potassium channel akt2 3	plasmodesma;integral component of membrane;voltage-gated potassium channel activity;identical protein binding;myo-inositol hexakisphosphate biosynthetic process;regulation of		

		membrane potential;potassium ion transmembrane transport		
Phvul.006G 216700	ferredoxin-chloroplastic	electron carrier activity;metal ion binding;2 iron, 2 sulfur cluster binding;electron transport chain		
Phvul.007G 013000	---NA---			
Phvul.007G 018700	zeaxanthin chloroplastic-like	zeaxanthin epoxidase [overall] activity;salicylate 1-monooxygenase activity;zeaxanthin epoxidase activity;antheraxanthin epoxidase activity;oxidation-reduction process	ec:1.14.13;ec:1.14.13.1-1-monooxygenase;ec:1.14.13.90-epoxidase	Naphthalene degradation;Polycyclic aromatic hydrocarbon degradation;Dioxin degradationCarotenoid biosynthesis
Phvul.007G 021100	pentatricopeptide repeat-containing protein at2g35130			
Phvul.007G 025700	probable carboxylesterase 6	hydrolase activity;metabolic process		
Phvul.007G 031000	wall associated kinase-like protein	protein serine/threonine kinase activity;non-membrane spanning protein tyrosine kinase activity;ATP binding;peptidyl-tyrosine phosphorylation	ec:2.7.10.2-protein-tyrosine kinase;ec:2.7.11;ec:2.7.10	T cell receptor signaling pathway
Phvul.007G 032200	auxin-responsive protein iaa32	nucleus;transcription, DNA-templated;regulation of transcription, DNA-templated;auxin-activated signaling pathway		
Phvul.007G 032800	amino-acid permease bat1 homolog isoform x1	integral component of membrane;amino acid transmembrane transporter activity;amino acid transmembrane transport		
Phvul.007G 034500	mate efflux family protein	integral component of membrane;drug transmembrane transporter activity;antiporter activity;drug transmembrane transport		
Phvul.007G 036600	hypothetical protein PHAVU_007G036600g			
Phvul.007G 036900	hypothetical protein PHAVU_007G036600g	integral component of membrane		
Phvul.007G 044500	zinc finger protein zat10-like	metal ion binding		
Phvul.007G 045400	glycosyl hydrolase family 10 protein	endo-1,4-beta-xylanase activity;xylan catabolic process	ec:3.2.1.8	
Phvul.007G 046200	hypothetical protein PHAVU_007G046200g			
Phvul.007G 048500	cysteine-rich receptor-kinase-like protein	protein serine/threonine kinase activity;ATP binding;protein phosphorylation	ec:2.7.11	
Phvul.007G 048800	cysteine-rich receptor-like protein kinase 29-like	protein serine/threonine kinase activity;ATP binding;protein phosphorylation	ec:2.7.11	
Phvul.007G 049600	cysteine-rich receptor-like protein kinase 29	protein serine/threonine kinase activity;ATP binding;protein phosphorylation	ec:2.7.11	
Phvul.007G 050300	cysteine-rich receptor-kinase-like protein	protein kinase activity;ATP binding;protein phosphorylation		
Phvul.007G 050400	cysteine-rich receptor-kinase-like protein	protein serine/threonine kinase activity;ATP binding;protein phosphorylation	ec:2.7.11	
Phvul.007G 056000	haloacid dehalogenase-like hydrolase	hydrolase activity;metabolic process		
Phvul.007G 057900	late embryogenesis abundant protein d-34-like			
Phvul.007G 058500	lipid transfer protein	lipid binding;lipid transport		

Phvul.007G 061800	heat stress transcription factor a-6b-like	nucleus;transcription factor activity, sequence-specific DNA binding;sequence-specific DNA binding;regulation of transcription, DNA-templated;response to heat		
Phvul.007G 062500	meiotic recombination protein dmc1 homolog	nucleus;DNA binding;ATP binding;DNA-dependent ATPase activity;DNA repair;reciprocal meiotic recombination	ec:3.6.1.3-adenylpyrophosphatase;ec:3.6.1;ec:3.6.1.15-phosphatase	Purine metabolismPurine metabolism;Thiamine metabolism
Phvul.007G 066200	protein hothead-like	oxidoreductase activity, acting on CH-OH group of donors;mandelonitrile lyase activity;flavin adenine dinucleotide binding;oxidation-reduction process	ec:4.1.2.10-lyase	Cyanoamino acid metabolism
Phvul.007G 067700	leucine-rich repeat receptor protein kinase exs	intracellular;integral component of membrane;MAP kinase kinase kinase activity;transmembrane receptor protein tyrosine kinase activity;phosphoprotein phosphatase activity;ATP binding;2-alkenal reductase [NAD(P)] activity;MAPK cascade;activation of MAPKK activity;response to molecule of bacterial origin;protein dephosphorylation;microsporogenesis;stomatal complex morphogenesis;anther wall tapetum cell fate specification;peptidyl-tyrosine phosphorylation;oxidation-reduction process	ec:1.3.1.74;ec:3.1.3.16-phosphatase;ec:2.7.10.1;ec:2.7.11.25;ec:2.7.11;ec:2.7.10;ec:3.1.3.41-nitrophenyl phosphatase;ec:3.1	T cell receptor signaling pathwayAminobenzoate degradation
Phvul.007G 069800	low-temperature-induced 65 kda			
Phvul.007G 070000	copper transport protein family	metal ion binding;metal ion transport		
Phvul.007G 071500	cytochrome p450 family 72 protein	monooxygenase activity;iron ion binding;oxidoreductase activity, acting on paired donors, with incorporation or reduction of molecular oxygen;heme binding;oxidation-reduction process		
Phvul.007G 077300	g-type lectin s-receptor-like serine threonine-protein kinase at5g24080	protein serine/threonine kinase activity;calmodulin binding;ATP binding;carbohydrate binding;protein phosphorylation;recognition of pollen	ec:2.7.11	
Phvul.007G 082000	ethylene-responsive transcription factor erf110-like isoform x1	nucleus;DNA binding;transcription factor activity, sequence-specific DNA binding;transcription, DNA-templated;regulation of transcription, DNA-templated		
Phvul.007G 091000	isoliquiritigenin 2 -o-methyltransferase	licodione 2'-O-methyltransferase activity;quercetin 3-O-methyltransferase activity;isoliquiritigenin 2'-O-methyltransferase activity;protein dimerization activity;caffeate O-methyltransferase activity;methylation	ec:2.1.1.154;ec:2.1.1;ec:2.1.1.76-3-O-methyltransferase;ec:2.1.1.68-O-methyltransferase;ec:2.1.1.65	Flavone and flavonol biosynthesisPhenylpropanoid biosynthesis
Phvul.007G 092400	hypothetical protein PHAVU_007G092400g			
Phvul.007G 096100	phenylalanine ammonia lyase	cytoplasm;phenylalanine ammonia-lyase activity;L-phenylalanine catabolic process;cinnamic acid biosynthetic process	ec:4.3.1.25-ammonia-lyase;ec:4.3.1.24-ammonia-lyase	Phenylalanine metabolism;Phenylpropanoid biosynthesisPhenylalanine metabolism;Phenylpropanoid biosynthesis
Phvul.007G 100700	aba induced plasma membrane protein			
Phvul.007G 109000	basic 7s globulin-like	aspartic-type endopeptidase activity;transferase activity;proteolysis	ec:3.4.23	
Phvul.007G 113300	reticuline oxidase-like	UDP-N-acetylmuramate dehydrogenase activity;reticuline oxidase activity;flavin adenine dinucleotide binding;oxidation-reduction process	ec:1.21.3;ec:1.21.3.3-oxidase;ec:1.1.1.158	Isoquinoline alkaloid biosynthesis
Phvul.007G 113700	kda class iv heat shock protein	endoplasmic reticulum		
Phvul.007G 114200	peroxidase 47	extracellular region;peroxidase activity;heme binding;metal ion binding;response to oxidative stress;hydrogen peroxide catabolic process;oxidation-reduction process	ec:1.11.1.7-lactoperoxidase	Phenylpropanoid biosynthesis
Phvul.007G 116300	leucine-rich repeat receptor-	integral component of membrane;protein serine/threonine kinase activity;ATP	ec:2.7.11	

	like protein kinase pxl1	binding;vasculature development;response to molecule of bacterial origin;protein phosphorylation;signal transduction;procambium histogenesis;stomatal complex morphogenesis;stamen development		
Phvul.007G 118300	probable tyrosine-protein phosphatase at1g05000	protein tyrosine phosphatase activity;peptidyl-tyrosine dephosphorylation	ec:3.1.3.16-phosphatase;ec:3.1.3.48;ec:3.1.3.41-nitrophenyl phosphatase;ec:3.1	T cell receptor signaling pathwayAminobenzoate degradation
Phvul.007G 120200	ammonium transporter 1 member 2-like	plasma membrane;integral component of membrane;ammonium transmembrane transporter activity;myo-inositol hexakisphosphate biosynthetic process;methylammonium transport;ammonium transmembrane transport		
Phvul.007G 120600	#NOME?			
Phvul.007G 127200	phosphoenolpyruvate carboxylase kinase 1-like	protein kinase activity;ATP binding;protein phosphorylation		
Phvul.007G 127800	ethylene-responsive transcription factor 1b	nucleus;DNA binding;transcription factor activity, sequence-specific DNA binding;transcription, DNA-templated;regulation of transcription, DNA-templated;response to stimulus		
Phvul.007G 128100	ethylene-responsive transcription factor erf098-like	nucleus;DNA binding;transcription factor activity, sequence-specific DNA binding;transcription, DNA-templated;regulation of transcription, DNA-templated		
Phvul.007G 132300	myb transcription factor	DNA binding		
Phvul.007G 133600	hypothetical protein PHAVU_007G1336002g, partial			
Phvul.007G 135600	1-aminocyclopropane-1-carboxylate oxidase	1-aminocyclopropane-1-carboxylate oxidase activity;naringenin 3-dioxygenase activity;metal ion binding;oxidation-reduction process	ec:1.14.11;ec:1.14.17.4-oxidase;ec:1.14.11.9-3-dioxygenase	Cysteine and methionine metabolismFlavonoid biosynthesis
Phvul.007G 137600	ring-h2 finger protein atl70-like	zinc ion binding;ligase activity;metabolic process		
Phvul.007G 138300	oleosin 1	monolayer-surrounded lipid storage body;integral component of membrane		
Phvul.007G 149000	auxin response factor 18-like isoform x2	nucleus;DNA binding;transcription, DNA-templated;regulation of transcription, DNA-templated;auxin-activated signaling pathway		
Phvul.007G 158600	wat1-related protein at2g37460-like	integral component of membrane;transmembrane transporter activity;transmembrane transport		
Phvul.007G 160000	exocyst complex component exo70a1-like	exocyst;exocytosis		
Phvul.007G 164400	beta- -xylosyltransferase irx9	membrane;galactosylgalactosylxylosylprotein 3-beta-glucuronosyltransferase activity;glucuronoxylan metabolic process;cell wall biogenesis	ec:2.4.1.17-1-naphthol glucuronyltransferase;ec:2.4.1.135-3-beta-glucuronosyltransferase	Drug metabolism - other enzymes;Drug metabolism - cytochrome P450;Metabolism of xenobiotics by cytochrome P450;Porphyrin and chlorophyll metabolism;Starch and sucrose metabolism;Ascorbate and aldarate metabolism;Retinol metabolism;Steroid hormone biosynthesis;Pentose and glucuronate interconversionsOther types of O-glycan biosynthesis;Glycosaminoglycan biosynthesis - heparan sulfate / heparin;Glycosaminogl

				ycan biosynthesis - chondroitin sulfate / dermatan sulfate
Phvul.007G 167000	probable atp-dependent rna helicase ddx56			
Phvul.007G 170900	basic 7s globulin	aspartic-type endopeptidase activity;proteolysis	ec:3.4.23	
Phvul.007G 181100	root phototropism protein 3-like	protein ubiquitination		
Phvul.007G 183300	viral a-type inclusion	intracellular membrane-bounded organelle		
Phvul.007G 184800	late embryogenesis abundant protein d-34-like	pollen tube growth		
Phvul.007G 185700	trna(adenine) chloroplastic	zinc ion binding;hydrolase activity, acting on carbon-nitrogen (but not peptide) bonds, in cyclic amidines;deaminase activity;metabolic process		
Phvul.007G 192700	patellin-4-like	phosphatase activity;protein dephosphorylation	ec:3.1.3.41-nitrophenyl phosphatase;ec:3.1	Aminobenzoate degradation
Phvul.007G 192800	glycinin	intracellular membrane-bounded organelle;cytoplasmic part;nutrient reservoir activity		
Phvul.007G 193700	ethylene-responsive transcription factor 1b	nucleus;DNA binding;transcription factor activity, sequence-specific DNA binding;transcription, DNA-templated;regulation of transcription, DNA-templated		
Phvul.007G 193800	ethylene-responsive transcription factor 1b	nucleus;DNA binding;transcription factor activity, sequence-specific DNA binding;transcription, DNA-templated;regulation of transcription, DNA-templated		
Phvul.007G 193900	ethylene-responsive transcription factor erf098-like	nucleus;DNA binding;transcription factor activity, sequence-specific DNA binding;transcription, DNA-templated;regulation of transcription, DNA-templated		
Phvul.007G 195100	lob domain-containing protein 16-like	nucleus;viral capsid;structural molecule activity;lateral root formation		
Phvul.007G 197500	hypoxia-responsive family protein	integral component of membrane;transferase activity;metabolic process		
Phvul.007G 198700	probable protein phosphatase 2c 73-like isoform x1	protein serine/threonine phosphatase activity;protein dephosphorylation	ec:3.1.3.16-phosphatase;ec:3.1.3.41-nitrophenyl phosphatase;ec:3.1	T cell receptor signaling pathwayAminobenzoate degradation
Phvul.007G 201100	hypothetical protein PHAVU_007G201100g			
Phvul.007G 203400	galactinol synthase	inositol 3-alpha-galactosyltransferase activity;galactose metabolic process	ec:2.4.1.123-3-alpha-galactosyltransferase;ec:2.4.1	Galactose metabolism
Phvul.007G 208800	auxin-responsive aux iaa family protein	nucleus;transcription, DNA-templated;regulation of transcription, DNA-templated;auxin-activated signaling pathway		
Phvul.007G 208900	chitinase 10	chitinase activity;carbohydrate metabolic process;chitin catabolic process;cell wall macromolecule catabolic process	ec:3.2.1.14-chitodextrinase	Amino sugar and nucleotide sugar metabolism
Phvul.007G 210400	pectinesterase pectinesterase inhibitor	cell wall;enzyme inhibitor activity;pectinesterase activity;aspartyl esterase activity;cell wall modification;negative regulation of catalytic activity;pectin catabolic process	ec:3.1.1.11-pectin demethoxylase;ec:3.1.1.1-ali-esterase	Starch and sucrose metabolism;Pentose and glucuronate interconversionsDrug metabolism - other enzymes
Phvul.007G 211400	polygalacturonase glycoside hydrolase family protein	extracellular region;polygalacturonase activity;galacturan 1,4-alpha-galacturonidase activity;carbohydrate metabolic process;cell wall organization	ec:3.2.1.67-1,4-alpha-galacturonidase;ec:3.2.1.15-pectin depolymerase	Starch and sucrose metabolism;Pentose and glucuronate interconversionsStarch and sucrose

				metabolism;Pentose and glucuronate interconversions
Phvul.007G 211900	myb-related protein 305	DNA binding		
Phvul.007G 220200	ubiquitin carboxyl-terminal hydrolase 34-like			
Phvul.007G 220500	hypothetical protein PHAVU_007G220 500g			
Phvul.007G 222400	hypothetical protein PHAVU_007G222 400g			
Phvul.007G 222500	dehydration-responsive element-binding protein 1b-like	nucleus;DNA binding;transcription factor activity, sequence-specific DNA binding;transcription, DNA-templated;regulation of transcription, DNA-templated		
Phvul.007G 222900	fructose-bisphosphate cytoplasmic isozyme	fructose-bisphosphate aldolase activity;glycolytic process	ec:4.1.2.13-aldolase	Biosynthesis of antibiotics;Pentose phosphate pathway;Methane metabolism;Carbon fixation in photosynthetic organisms;Glycolysis / Gluconeogenesis;Fructose and mannose metabolism
Phvul.007G 223100	phytosulfokine precursor protein	extracellular region;growth factor activity;cell proliferation		
Phvul.007G 225200	late embryogenesis abundant partial			
Phvul.007G 228200	glycinol 4-dimethylallyltransferase-like	integral component of membrane;prenyltransferase activity;metabolic process	ec:2.5.1	
Phvul.007G 228300	glycinol 4-dimethylallyltransferase-like	integral component of membrane;prenyltransferase activity;metabolic process	ec:2.5.1	
Phvul.007G 228500	glycinol 4-dimethylallyltransferase-like	integral component of membrane;prenyltransferase activity;metabolic process	ec:2.5.1	
Phvul.007G 230600	protein trichome birefringence-like 8			
Phvul.007G 231800	transcription factor myb48	DNA binding		
Phvul.007G 239800	lectin precursor	carbohydrate binding		
Phvul.007G 242000	mitogen-activated protein kinase kinase kinase	protein serine/threonine kinase activity;ATP binding;protein phosphorylation	ec:2.7.11	
Phvul.007G 246700	probable pectinesterase pectinesterase inhibitor 7	cell wall;enzyme inhibitor activity;pectinesterase activity;aspartyl esterase activity;cell wall modification;negative regulation of catalytic activity;pectin catabolic process	ec:3.1.1.11-pectin demethoxylase;ec:3.1.1.1-ali-esterase	Starch and sucrose metabolism;Pentose and glucuronate interconversionsDrug metabolism - other enzymes
Phvul.007G 248500	gibberellin 20 oxidase 1	gibberellin 3-beta-dioxygenase activity;gibberellin-44 dioxygenase activity;oxidation-reduction process	ec:1.14.11.15-3beta-dioxygenase;ec:1.14.11.12-dioxygenase;ec:1.14.11	Diterpenoid biosynthesisDiterpenoid biosynthesis
Phvul.007G 248700	gibberellin 20 oxidase 1	metal ion binding;gibberellin-44 dioxygenase activity;oxidation-reduction process	ec:1.14.11.12-dioxygenase;ec:1.14.11	Diterpenoid biosynthesis
Phvul.007G 257300	cytochrome p450 93a3-like	iron ion binding;flavonoid 3'-monooxygenase activity;heme binding;oxidation-reduction process	ec:1.14.13.21-3'-monooxygenase;ec:1.14.13	Flavone and flavonol biosynthesis;Flavonoid biosynthesis
Phvul.007G 258900	duf761 domain protein			

Phvul.007G259400	seed maturation protein lea 4	embryo development		
Phvul.007G259500	omega-6 fatty acid endoplasmic reticulum isozyme 2	oxidoreductase activity, acting on paired donors, with oxidation of a pair of donors resulting in the reduction of molecular oxygen to two molecules of water;delta12-fatty acid dehydrogenase activity;lipid metabolic process;oxidation-reduction process	ec:1.14.99.33;ec:1.14.19	
Phvul.007G269400	cytokinin riboside 5 -monophosphate phosphoribohydrolase log7	nucleus;cytosol;hydrolase activity;metabolic process		
Phvul.007G273000	ethylene-responsive transcription factor 1b	nucleus;DNA binding;transcription factor activity, sequence-specific DNA binding;transcription, DNA-templated;regulation of transcription, DNA-templated;response to stimulus		
Phvul.007G275700	mung bean seed albumin	metal ion binding		
Phvul.007G276100	achain crystal structure of hemopexin fold protein cp4 from cow pea	metal ion binding		
Phvul.007G276200	achain crystal structure of hemopexin fold protein cp4 from cow pea	metal ion binding		
Phvul.007G276400	albumin-2 protein	metal ion binding		
Phvul.007G276500	albumin-2 protein	metal ion binding		
Phvul.007G276600	albumin-2 protein	metal ion binding		
Phvul.007G276700	achain crystal structure of hemopexin fold protein cp4 from cow pea	metal ion binding		
Phvul.007G276900	phosphatase 2c family protein	protein serine/threonine phosphatase activity;protein dephosphorylation	ec:3.1.3.16-phosphatase;ec:3.1.3.41-nitrophenyl phosphatase;ec:3.1	T cell receptor signaling pathwayAminobenzoate degradation
Phvul.007G277000	achain crystal structure of hemopexin fold protein cp4 from cow pea	metal ion binding		
Phvul.007G278800	dentin sialophospho			
Phvul.007G278900	calmodulin-like protein 11	calcium ion binding		
Phvul.007G280700	transcription factor bhlh92-like	protein dimerization activity		
Phvul.008G005200	ist1-like protein	response to stress;single-organism cellular process;regulation of biological process		
Phvul.008G007900	probable serine threonine-protein kinase at1g18390 isoform x1	protein serine/threonine kinase activity;ATP binding;protein phosphorylation	ec:2.7.11	
Phvul.008G008000	probable receptor-like protein kinase at1g67000	binding;kinase activity;phosphorylation		
Phvul.008G008100	glutamine amidotransferase	GMP synthase (glutamine-hydrolyzing) activity;transferase activity;hydrolase activity;glutamine metabolic process	ec:6.3.5.2-synthase (glutamine-hydrolysing)	Drug metabolism - other enzymes;Purine metabolism
Phvul.008G008400	protein plastid movement impaired 15-like			
Phvul.008G008800	hypothetical protein			

	PHAVU_008G008800g			
Phvul.008G012600	probable mediator of rna polymerase ii transcription subunit 37e	ATP binding;2-alkenal reductase [NAD(P)] activity;oxidation-reduction process	ec:1.3.1.74	
Phvul.008G016200	triphosphate tunel metalloenzyme 3-like			
Phvul.008G016500	bag domain protein	chaperone binding		
Phvul.008G016600	bag domain protein	chaperone binding		
Phvul.008G017100	wrky transcription factor-like protein			
Phvul.008G019600	ethylene response factor	nucleus;DNA binding;transcription factor activity, sequence-specific DNA binding;transcription, DNA-templated;regulation of transcription, DNA-templated		
Phvul.008G020100	family transcriptional	DNA binding;DNA-directed RNA polymerase activity;protein dimerization activity;transcription, DNA-templated	ec:2.7.7.6-RNA polymerase	Purine metabolism;Pyrimidine metabolism
Phvul.008G023600	hypothetical protein LR48_Vigan03g004300			
Phvul.008G024200	upf0481 plant-like protein			
Phvul.008G028000	transcription factor myb59	DNA binding		
Phvul.008G028400	serine-threonine kinase	protein kinase activity;ATP binding;protein phosphorylation		
Phvul.008G029600	serine-threonine kinase	protein kinase activity;ATP binding;protein phosphorylation		
Phvul.008G030300	hypothetical protein PHAVU_008G030300g			
Phvul.008G031900	anthocyanin 5-aromatic acyltransferase	shikimate O-hydroxycinnamoyltransferase activity;metabolic process	ec:2.3.1;ec:2.3.1.133-O-hydroxycinnamoyl transferase	Stilbenoid, diarylheptanoid and gingerol biosynthesis;Flavonoid biosynthesis;Phenylpropanoid biosynthesis
Phvul.008G032200	anthocyanin 5-aromatic acyltransferase	shikimate O-hydroxycinnamoyltransferase activity;metabolic process	ec:2.3.1;ec:2.3.1.133-O-hydroxycinnamoyl transferase	Stilbenoid, diarylheptanoid and gingerol biosynthesis;Flavonoid biosynthesis;Phenylpropanoid biosynthesis
Phvul.008G032400	anthocyanin 5-aromatic acyltransferase	malonyltransferase activity;shikimate O-hydroxycinnamoyltransferase activity;metabolic process	ec:2.3.1;ec:2.3.1.133-O-hydroxycinnamoyl transferase	Stilbenoid, diarylheptanoid and gingerol biosynthesis;Flavonoid biosynthesis;Phenylpropanoid biosynthesis
Phvul.008G036500	duf679 domain membrane protein			
Phvul.008G040500	nitrate transporter -like	membrane;nitrate transmembrane transporter activity;nitrate transport		
Phvul.008G041800	transcription factor bhlh135	protein dimerization activity		
Phvul.008G042300	o-acyltransferase wsd1-like protein	diacylglycerol O-acyltransferase activity;glycerolipid biosynthetic process	ec:2.3.1;ec:2.3.1.20-O-acyltransferase	Cutin, suberine and wax biosynthesis;Glycerolipid metabolism
Phvul.008G043400	lrr receptor-like kinase	transferase activity;2-alkenal reductase [NAD(P)] activity;oxidation-reduction process	ec:1.3.1.74	
Phvul.008G044900	p-loop nucleoside triphosphate hydrolase superfamily protein	ATP binding;hydrolase activity;metabolic process		

Phvul.008G 045100	homeobox-leucine zipper protein hat3	nucleus;transcription factor activity, sequence-specific DNA binding;sequence-specific DNA binding;transcription, DNA-templated;regulation of transcription, DNA-templated		
Phvul.008G 046500	ethylene-responsive transcription factor 1-like	nucleus;DNA binding;transcription factor activity, sequence-specific DNA binding;transcription, DNA-templated;regulation of transcription, DNA-templated		
Phvul.008G 048400	pentatricopeptide repeat-containing protein chloroplastic	microtubule-severing ATPase activity;metabolic process	ec:3.6.1.3-adenylpyrophosphatase;ec:3.6.1;ec:3.6.4.3;ec:3.6.1.15-phosphatase	Purine metabolismPurine metabolism;Thiamine metabolism
Phvul.008G 050900	helix loop helix dna-binding domain protein	2-alkenal reductase [NAD(P)] activity;protein dimerization activity;oxidation-reduction process	ec:1.3.1.74	
Phvul.008G 055200	auxin efflux carrier component 8	endoplasmic reticulum;integral component of membrane;auxin transmembrane transporter activity;pollen development;auxin homeostasis;transmembrane transport;intracellular auxin transport		
Phvul.008G 056800	type receptor kinase	protein serine/threonine kinase activity;ATP binding;protein phosphorylation	ec:2.7.11	
Phvul.008G 058400	1-aminocyclopropane-1-carboxylate synthase 1-like	1-aminocyclopropane-1-carboxylate synthase activity;pyridoxal phosphate binding;1-aminocyclopropane-1-carboxylate biosynthetic process	ec:4.4.1.14-synthase	Cysteine and methionine metabolism
Phvul.008G 062700	gibberellin 3-beta-dioxygenase 4	iron ion binding;gibberellin-44 dioxygenase activity;oxidation-reduction process	ec:1.14.11.12-dioxygenase;ec:1.14.11	Diterpenoid biosynthesis
Phvul.008G 065500	duf3245 family protein			
Phvul.008G 065800	trypsin inhibitor	extracellular region;serine-type endopeptidase inhibitor activity;peptidase activity;proteolysis;negative regulation of endopeptidase activity		
Phvul.008G 068300	(+)-neomenthol dehydrogenase	carbonyl reductase (NADPH) activity;oxidation-reduction process	ec:1.1.1.184-reductase (NADPH)	Arachidonic acid metabolism;Metabolism of xenobiotics by cytochrome P450
Phvul.008G 069400	spx domain-containing membrane protein at4g22990-like	integral component of membrane;transmembrane transport		
Phvul.008G 069500	peptidoglycan-binding domain protein			
Phvul.008G 073200	polyphenol oxidase chloroplastic	catechol oxidase activity;metal ion binding;pigment biosynthetic process;oxidation-reduction process	ec:1.10.3;ec:1.10.3.1-oxidase	Isoquinoline alkaloid biosynthesis;Tyrosine metabolism
Phvul.008G 077500	cysteine-rich receptor-kinase-like protein	protein kinase activity;ATP binding;protein phosphorylation		
Phvul.008G 079700	receptor-like protein 12	transferase activity;metabolic process		
Phvul.008G 087500	golgin subfamily a member 5-like			
Phvul.008G 087600	alanine--glyoxylate aminotransferase 2 homolog mitochondrial-like	alanine-glyoxylate transaminase activity;pyridoxal phosphate binding;metabolic process	ec:2.6.1.44-transaminase	Cysteine and methionine metabolism;Glycine, serine and threonine metabolism;Alanine, aspartate and glutamate metabolism
Phvul.008G 089400	tubby c 2 protein			
Phvul.008G 089900	short-chain dehydrogenase reductase 2a-like	3-oxoacyl-[acyl-carrier-protein] reductase (NADPH) activity;xanthoxin dehydrogenase activity;oxidation-reduction process	ec:1.1.1.288-dehydrogenase;ec:2.3.1.85-synthase;ec:1.1.1.100-reductase;ec:1.1.1.1-dehydrogenase	Carotenoid biosynthesisFatty acid biosynthesisBiosynthesis of unsaturated fatty acids;Biotin metabolism;Fatty acid biosynthesisalpha-Linolenic acid metabolism;Drug

				metabolism - cytochrome P450;Metabolism of xenobiotics by cytochrome P450;Naphthalene degradation;Chloroalkane and chloroalkene degradation;Biosynthesis of antibiotics;Glycolysis / Gluconeogenesis;Fatty acid degradation;Glycine, serine and threonine metabolism;Tyrosine metabolism;Retinol metabolism
Phvul.008G093200	lrr receptor-like kinase	integral component of membrane;protein serine/threonine kinase activity;ATP binding;protein phosphorylation	ec:2.7.11	
Phvul.008G094400	nodulation-signaling pathway 2	transcription, DNA-templated;regulation of transcription, DNA-templated		
Phvul.008G098200	alc-interacting protein			
Phvul.008G098500	leucoanthocyanidin dioxygenase-like	iron ion binding;leucocyanidin oxygenase activity;oxidation-reduction process	ec:1.14.11.19-oxygenase;ec:1.14.11	Flavonoid biosynthesis
Phvul.008G103500	gdsl esterase lipase cprd49-like	hydrolase activity, acting on ester bonds;vegetative to reproductive phase transition of meristem;protein desumoylation;hydrogen peroxide biosynthetic process		
Phvul.008G103600	gdsl esterase lipase cprd49	hydrolase activity, acting on ester bonds;vegetative to reproductive phase transition of meristem;protein desumoylation;hydrogen peroxide biosynthetic process		
Phvul.008G104600	probable envelope carrier chloroplastic	integral component of membrane;transmembrane transport		
Phvul.008G105800	hypothetical protein PHAVU_008G105800g			
Phvul.008G106500	lrr receptor-like kinase family protein	integral component of membrane;protein kinase activity;ATP binding;protein phosphorylation		
Phvul.008G108900	mitochondrial uncoupling protein 1-like	integral component of membrane;mitochondrial membrane;mitochondrial transport		
Phvul.008G109000	lrr receptor-like serine threonine-protein kinase gso1			
Phvul.008G109100	lrr receptor-like serine threonine-protein kinase gso1	transferase activity		
Phvul.008G109300	lrr receptor-like serine threonine-protein kinase gso1	transferase activity		
Phvul.008G109600	lrr receptor-like serine threonine-protein kinase gso1	transferase activity		
Phvul.008G111000	ppr superfamily protein			
Phvul.008G113700	tau class glutathione s-transferase	glutathione transferase activity;metabolic process	ec:2.5.1.18-transferase	Drug metabolism - cytochrome P450;Metabolism of xenobiotics by cytochrome P450;Glutathione metabolism
Phvul.008G121000	hypothetical protein			

	PHAVU_008G121000g, partial			
Phvul.008G123300	hypothetical protein PHAVU_008G123300g			
Phvul.008G127000	g-type lectin s-receptor-like serine threonine-kinase	protein kinase activity;ATP binding;carbohydrate binding;protein phosphorylation;recognition of pollen		
Phvul.008G127400	2og-fe oxygenase family oxidoreductase	oxidoreductase activity, acting on paired donors, with incorporation or reduction of molecular oxygen, 2-oxoglutarate as one donor, and incorporation of one atom each of oxygen into both donors;metal ion binding;oxidation-reduction process	ec:1.14.11	
Phvul.008G134100	gibberellin 20 oxidase 1	iron ion binding;oxidoreductase activity, acting on paired donors, with incorporation or reduction of molecular oxygen, 2-oxoglutarate as one donor, and incorporation of one atom each of oxygen into both donors;oxidation-reduction process	ec:1.14.11	
Phvul.008G134800	serine threonine-protein kinase oxi1-like	protein serine/threonine kinase activity;ATP binding;protein phosphorylation	ec:2.7.11	
Phvul.008G140000	hypothetical protein PHAVU_008G140000g			
Phvul.008G146400	hypothetical protein PHAVU_008G146400g, partial			
Phvul.008G155000	cytochrome p450 family 71 protein	monooxygenase activity;iron ion binding;oxidoreductase activity, acting on paired donors, with incorporation or reduction of molecular oxygen;heme binding;oxidation-reduction process		
Phvul.008G160400	transcription factor bhlh086-like	hydrolase activity;protein dimerization activity;metabolic process		
Phvul.008G164100	wat1-related protein at4g08290-like	integral component of membrane;transmembrane transporter activity;transmembrane transport		
Phvul.008G169600	aminotransferase ald1-like	L,L-diaminopimelate aminotransferase activity;pyridoxal phosphate binding;lysine biosynthetic process via diaminopimelate	ec:2.6.1.83-aminotransferase	Biosynthesis of antibiotics;Lysine biosynthesis
Phvul.008G174800	hypothetical protein PHAVU_008G174800g			
Phvul.008G175500	udp-glucosyltransferase family protein	transferase activity, transferring hexosyl groups;metabolic process		
Phvul.008G176100	protein exordium-like 2			
Phvul.008G176900	zinc finger cchc domain-containing protein 10-like			
Phvul.008G183400	hypothetical protein PHAVU_008G183400g	plasma membrane		
Phvul.008G186200	voltage-dependent t-type calcium channel subunit alpha-			
Phvul.008G186800	probable inorganic phosphate transporter 1-3	integral component of membrane;inorganic phosphate transmembrane transporter activity;phosphate ion transport;transmembrane transport		
Phvul.008G188100	hypothetical protein PHAVU_008G188100g			

Phvul.008G 188200	ribonuclease p family protein rpp14 family protein isoform 1	vacuole;ribonuclease P activity;tRNA processing;RNA phosphodiester bond hydrolysis, endonucleolytic	ec:3.1.26.5;ec:3.1.30;ec:3.1.26;ec:3.1	
Phvul.008G 191600	glycerol-3-phosphate acyltransferase 1	mitochondrion;membrane;glycerol-3-phosphate O-acyltransferase activity;glycerol-3-phosphate 2-O-acyltransferase activity;phosphatidylglycerol biosynthetic process;pollen sperm cell differentiation	ec:2.3.1;ec:2.3.1.15-1-O-acyltransferase	Glycerophospholipid metabolism;Glycerolipid metabolism
Phvul.008G 194600	nac domain-containing protein 42	nucleus;DNA binding;transcription, DNA-templated;regulation of transcription, DNA-templated;response to stress;single-organism biosynthetic process;single-organism cellular process;organonitrogen compound biosynthetic process		
Phvul.008G 207900	zinc-binding alcohol dehydrogenase family protein	zinc ion binding;S-(hydroxymethyl)glutathione dehydrogenase activity;oxidation-reduction process	ec:1.1.1.284-dehydrogenase	Methane metabolism
Phvul.008G 213800	1-aminocyclopropane-1-carboxylate oxidase	1-aminocyclopropane-1-carboxylate oxidase activity;naringenin 3-dioxygenase activity;metal ion binding;oxidation-reduction process	ec:1.14.11;ec:1.14.17.4-oxidase;ec:1.14.11.9-3-dioxygenase	Cysteine and methionine metabolismFlavonoid biosynthesis
Phvul.008G 213900	1-aminocyclopropane-1-carboxylate oxidase	1-aminocyclopropane-1-carboxylate oxidase activity;naringenin 3-dioxygenase activity;metal ion binding;oxidation-reduction process	ec:1.14.11;ec:1.14.17.4-oxidase;ec:1.14.11.9-3-dioxygenase	Cysteine and methionine metabolismFlavonoid biosynthesis
Phvul.008G 214200	1-aminocyclopropane-1-carboxylate oxidase	1-aminocyclopropane-1-carboxylate oxidase activity;L-ascorbic acid binding;naringenin 3-dioxygenase activity;metal ion binding;ethylene biosynthetic process;fruit ripening;oxidation-reduction process	ec:1.14.11;ec:1.14.17.4-oxidase;ec:1.14.11.9-3-dioxygenase	Cysteine and methionine metabolismFlavonoid biosynthesis
Phvul.008G 219800	neuronal pas domain-containing protein 4			
Phvul.008G 220400	dehydration-responsive element-binding protein 2c	nucleus;DNA binding;transcription factor activity, sequence-specific DNA binding;transcription, DNA-templated;regulation of transcription, DNA-templated		
Phvul.008G 223500	leucoanthocyanidin dioxygenase-like protein	iron ion binding;methyltransferase activity;oxidoreductase activity, acting on paired donors, with incorporation or reduction of molecular oxygen, 2-oxoglutarate as one donor, and incorporation of one atom each of oxygen into both donors;methylation;oxidation-reduction process	ec:1.14.11	
Phvul.008G 224100	phosphoinositide phospholipase c 6	intracellular;phosphatidylinositol phospholipase C activity;signal transducer activity;lipid catabolic process;intracellular signal transduction	ec:3.1.4.11-phospholipase C;ec:3.1;ec:3.1.1	Inositol phosphate metabolism;Phosphatidylinositol signaling system
Phvul.008G 225600	hypothetical protein PHAVU_008G225600g			
Phvul.008G 226200	uncharacterized acetyltransferase at3g50280	shikimate O-hydroxycinnamoyltransferase activity;anthranilate N-benzoyltransferase activity;metabolic process	ec:2.3.1;ec:2.3.1.144;ec:2.3.1.133-O-hydroxycinnamoyl transferase	Stilbenoid, diarylheptanoid and gingerol biosynthesis;Flavonoid biosynthesis;Phenylpropanoid biosynthesis
Phvul.008G 227100	glucan endo- -beta-glucosidase 12	hydrolase activity, hydrolyzing O-glycosyl compounds;carbohydrate metabolic process		
Phvul.008G 228000	kda class i heat shock			
Phvul.008G 228100	kda class i heat shock			
Phvul.008G 228900	cucumisin-like isoform x1	serine-type endopeptidase activity;proteolysis	ec:3.4.21	
Phvul.008G 229100	cucumisin-like isoform x1	serine-type endopeptidase activity;proteolysis	ec:3.4.21	
Phvul.008G 238200	hypothetical protein PHAVU_008G238200g			
Phvul.008G 242900	protein yls9			

Phvul.008G 249500	peroxidase 4	extracellular region;peroxidase activity;heme binding;metal ion binding;response to oxidative stress;hydrogen peroxide catabolic process;oxidation-reduction process	ec:1.11.1.7-lactoperoxidase	Phenylpropanoid biosynthesis
Phvul.008G 249900	cationic peroxidase 1-like	extracellular region;peroxidase activity;heme binding;metal ion binding;response to oxidative stress;hydrogen peroxide catabolic process;oxidation-reduction process	ec:1.11.1.7-lactoperoxidase	Phenylpropanoid biosynthesis
Phvul.008G 251000	wound-induced-like protein			
Phvul.008G 251400	cation h(+) antiporter 15-like	integral component of membrane;monovalent cation:proton antiporter activity;potassium ion transport;sodium ion transport;hydrogen ion transmembrane transport		
Phvul.008G 251500	duf4228 domain protein			
Phvul.008G 251700	exocyst subunit exo70 family protein	Golgi apparatus;transcription factor activity, sequence-specific DNA binding;sequence-specific DNA binding;regulation of transcription, DNA-templated		
Phvul.008G 252000	achain crystal structure of hemopexin fold protein cp4 from cow pea	metal ion binding		
Phvul.008G 256600	udp-glycosyltransferase 87a1-like	indole-3-acetate beta-glucosyltransferase activity;limonoid glucosyltransferase activity;metabolic process	ec:2.4.1.121;ec:2.4.1;ec:2.4.1.210	
Phvul.008G 259700	mitogen-activated protein kinase kinase kinase npk1-like	protein serine/threonine kinase activity;ATP binding;protein phosphorylation	ec:2.7.11	
Phvul.008G 261200	protein early flowering 4-like	nucleus;protein homodimerization activity;positive regulation of circadian rhythm		
Phvul.008G 264000	duf868 family protein			
Phvul.008G 268200	geranyl-diphosphate synthase			
Phvul.008G 270500	probable wrky transcription factor 31	transcription factor activity, sequence-specific DNA binding;sequence-specific DNA binding;regulation of transcription, DNA-templated		
Phvul.008G 273800	receptor-like protein			
Phvul.008G 275400	nucleic acid binding			
Phvul.008G 279600	cellulose synthase-like protein e1	integral component of membrane;cellulose synthase (UDP-forming) activity;cellulose biosynthetic process	ec:2.4.1.12-synthase (UDP-forming);ec:2.4.1	Starch and sucrose metabolism
Phvul.008G 279800	cellulose synthase-like protein e6	integral component of membrane;cellulose synthase (UDP-forming) activity;cellulose biosynthetic process	ec:2.4.1.12-synthase (UDP-forming);ec:2.4.1	Starch and sucrose metabolism
Phvul.008G 282200	heavy metal transport detoxification superfamily protein	metal ion binding;single-organism process		
Phvul.008G 282600	probable galactinol--sucrose galactosyltransferase 1	galactinol-raffinose galactosyltransferase activity;metabolic process	ec:2.4.1;ec:2.4.1.67-galactosyltransferase	Galactose metabolism
Phvul.008G 285200	hypothetical protein PHAVU_008G285200g			
Phvul.008G 287200	polyketide reductase	alcohol dehydrogenase (NADP+) activity;6'-deoxychalcone synthase activity;flavonoid biosynthetic process;oxidation-reduction process	ec:2.3.1.170-synthase;ec:1.1.1;ec:1.1.1.2-dehydrogenase (NADP+)	Flavonoid biosynthesisBiosynthesis of antibiotics;Glycolysis / Gluconeogenesis;Glycerolipid metabolism;Caprolactam degradation;Pentose and glucuronate interconversions

Phvul.008G 290700	vq motif-containing protein 22-like			
Phvul.009G 005300	late embryogenesis abundant	response to stress;response to water		
Phvul.009G 012700	beta-galactosidase 5	beta-galactosidase activity;carbohydrate binding;carbohydrate metabolic process	ec:3.2.1;ec:3.2.1.23-lactase (ambiguous)	Other glycan degradation;Glycosphingolipid biosynthesis - ganglio series;Sphingolipid metabolism;Galactose metabolism;Glycosaminoglycan degradation
Phvul.009G 017700	pathogenesis-related thaumatin family protein			
Phvul.009G 018300	gdsl esterase lipase exl3	lipase activity;9,9'-dicis-carotene:quinone oxidoreductase activity;7,9,9'-tricis-neurosporene:quinone oxidoreductase activity;9,9'-di-cis-zeta-carotene desaturation to 7,9,7',9'-tetra-cis-lycopene;oxidation-reduction process	ec:1.3.5.6-desaturase;ec:3.1.1	Carotenoid biosynthesis
Phvul.009G 024300	specific tissue protein			
Phvul.009G 027700	lob domain-containing protein 36-like	carbamoyl-phosphate synthase (glutamine-hydrolyzing) activity;regulation of transcription, DNA-templated;metabolic process;proximal/distal pattern formation;leaf morphogenesis;petal development	ec:6.3.5.5-synthase (glutamine-hydrolysing)	Alanine, aspartate and glutamate metabolism;Pyrimidine metabolism
Phvul.009G 042800	voltage-dependent t-type calcium channel subunit alpha-	chloroplast		
Phvul.009G 043200	wrky transcription factor 55	transcription factor activity, sequence-specific DNA binding;sequence-specific DNA binding;regulation of transcription, DNA-templated		
Phvul.009G 046500	small heat shock chloroplastic	chloroplast;protein folding;heat acclimation		
Phvul.009G 054300	hypothetical protein PHAVU_009G054300g, partial	nucleotidyltransferase activity;metabolic process		
Phvul.009G 054400	probable trehalose-phosphate phosphatase c isoform x1	trehalose-phosphatase activity;transferase activity, transferring glycosyl groups;trehalose biosynthetic process;protein dephosphorylation	ec:3.1.3.12-trehalose 6-phosphatase;ec:3.1.3.41-nitrophenyl phosphatase;ec:3.1	Starch and sucrose metabolismAminobenzoate degradation
Phvul.009G 057300	aldehyde dehydrogenase			
Phvul.009G 059900	hypothetical protein PHAVU_009G059900g			
Phvul.009G 061600	cytochrome p450 82c4-like	monooxygenase activity;iron ion binding;oxidoreductase activity, acting on paired donors, with incorporation or reduction of molecular oxygen;heme binding;oxidation-reduction process		
Phvul.009G 062600	early nodulin-55-2-like	electron carrier activity		
Phvul.009G 064900	chlorophyll a-b binding protein chloroplastic	photosystem II;chloroplast thylakoid membrane;photosystem I antenna complex;integral component of membrane;chlorophyll binding;pigment binding;metal ion binding;rRNA processing;photosynthesis, light harvesting in photosystem I;thylakoid membrane organization;photosystem II assembly;protein-chromophore linkage		
Phvul.009G 066300	uncharacterized acetyltransferase at3g50280-like	shikimate O-hydroxycinnamoyltransferase activity;metabolic process	ec:2.3.1;ec:2.3.1.133-O-hydroxycinnamoyltransferase	Stilbenoid, diarylheptanoid and gingerol biosynthesis;Flavonoid biosynthesis;Phenylpropanoid biosynthesis

Phvul.009G066900	uncharacterized acetyltransferase at3g50280-like	shikimate O-hydroxycinnamoyltransferase activity;metabolic process	ec:2.3.1;ec:2.3.1.133-O-hydroxycinnamoyltransferase	Stilbenoid, diarylheptanoid and gingerol biosynthesis;Flavonoid biosynthesis;Phenylpropanoid biosynthesis
Phvul.009G067900	probable carbohydrate esterase at4g34215			
Phvul.009G068000	transcription factor myb44-like	DNA binding		
Phvul.009G075100	branched-chain amino acid aminotransferase	L-leucine transaminase activity;L-valine transaminase activity;L-isoleucine transaminase activity;branched-chain amino acid metabolic process;toxin catabolic process	ec:2.6.1.42-transaminase	Valine, leucine and isoleucine biosynthesis;Biosynthesis of antibiotics;Valine, leucine and isoleucine degradation;Pantothenate and CoA biosynthesis;Glucosinolate biosynthesis;Cysteine and methionine metabolism
Phvul.009G075500	zinc finger ccch domain-containing protein 23-like	metal ion binding		
Phvul.009G078300	heat shock factor protein hsf30 isoform x1	nucleus;transcription factor activity, sequence-specific DNA binding;sequence-specific DNA binding;transcription regulatory region DNA binding;response to hypoxia;heat acclimation;cellular response to unfolded protein;cellular metabolic process;positive regulation of transcription, DNA-templated		
Phvul.009G080200	kda class ii heat shock protein			
Phvul.009G087700	hypothetical protein PHAVU_009G087700g			
Phvul.009G094000	eg45-like domain containing protein 2			
Phvul.009G094600	upf0481 protein			
Phvul.009G100300	flowering-promoting factor 1-like			
Phvul.009G104200	ef hand calcium-binding family protein	calcium ion binding		
Phvul.009G108300	bark storage protein a-like	catalytic activity;nucleoside metabolic process		
Phvul.009G108700	probable lactoylglutathione chloroplast	chloroplast stroma;stromule;thylakoid lumen;lactoylglutathione lyase activity;metal ion binding;dioxygenase activity;oxidation-reduction process	ec:4.4.1.5-lyase	Pyruvate metabolism
Phvul.009G109100	carotenoid (9)-cleavage dioxygenase 1-like	dioxygenase activity;oxidation-reduction process		
Phvul.009G111500	nadp-dependent malic enzyme	cytoplasm;malate dehydrogenase (decarboxylating) (NAD+) activity;malate dehydrogenase (decarboxylating) (NADP+) activity;oxaloacetate decarboxylase activity;metal ion binding;NAD binding;malate metabolic process;oxidation-reduction process	ec:1.1.1.40-dehydrogenase (oxaloacetate-decarboxylating) (NADP+);ec:4.1.1.3-decarboxylase;ec:1.1.1.39-dehydrogenase (decarboxylating);ec:1.1.1.38-dehydrogenase (oxaloacetate-decarboxylating)	Pyruvate metabolism;Carbon fixation in photosynthetic organismsPyruvate metabolismPyruvate metabolism;Carbon fixation in photosynthetic organismsPyruvate metabolism

Phvul.009G 116800	hypothetical protein PHAVU_009G116 800g			
Phvul.009G 118800	plant phospholipase-like protein			
Phvul.009G 120400	nodulin family protein			
Phvul.009G 120500	probable endo- -beta-xylanase c	endo-1,4-beta-xylanase activity;xylan catabolic process	ec:3.2.1.8	
Phvul.009G 121000	nitrate reductase	nitrate reductase (NADH) activity;heme binding;molybdenum ion binding;molybdopterin cofactor binding;nitrate reductase (NADPH) activity;nitric oxide biosynthetic process;nitrate assimilation;oxidation-reduction process	ec:1.7.1.3-reductase (NADPH);ec:1.7.1.1-reductase (NADH);ec:1.7.99.4-reductase	Nitrogen metabolismNitrogen metabolismNitrogen metabolism
Phvul.009G 125100	photosystem ii 22 kda chloroplastic	PSII associated light-harvesting complex II;chlorophyll binding;xanthophyll binding;maltose metabolic process;rRNA processing;thylakoid membrane organization;stomatal complex morphogenesis;nonphotochemical quenching;photosystem II assembly;starch biosynthetic process;cysteine biosynthetic process;positive regulation of catalytic activity		
Phvul.009G 125900	nac domain-containing protein 2	nucleus;DNA binding;transcription, DNA-templated;regulation of transcription, DNA-templated		
Phvul.009G 127900	metal transporter nramp5-like	membrane;transporter activity;transport		
Phvul.009G 131000	chalcone synthase	nucleus;endoplasmic reticulum;plant-type vacuole membrane;naringenin-chalcone synthase activity;response to gravity;anthocyanin-containing compound biosynthetic process;response to sucrose;auxin polar transport;response to UV-B	ec:2.3.1.74-synthase	Flavonoid biosynthesis
Phvul.009G 132900	2-oxoisovalerate dehydrogenase subunit alpha mitochondrial-like	3-methyl-2-oxobutanoate dehydrogenase (2-methylpropanoyl-transferring) activity;oxidation-reduction process	ec:1.2.4.4-dehydrogenase (2-methylpropanoyl-transferring)	Biosynthesis of antibiotics;Valine, leucine and isoleucine degradation;Propanoate metabolism
Phvul.009G 135300	wat1-related protein at1g70260-like	integral component of membrane;transmembrane transporter activity;transmembrane transport		
Phvul.009G 136000	glycolipid transfer protein family protein	cytoplasm;glycolipid transporter activity;glycolipid binding;glycolipid transport		
Phvul.009G 137900	ethylene-responsive transcription factor erf114	nucleus;DNA binding;transcription factor activity, sequence-specific DNA binding;transcription, DNA-templated;regulation of transcription, DNA-templated		
Phvul.009G 140700	peroxidase 4-like	extracellular region;peroxidase activity;heme binding;metal ion binding;response to oxidative stress;hydrogen peroxide catabolic process;oxidation-reduction process	ec:1.11.1.7-lactoperoxidase	Phenylpropanoid biosynthesis
Phvul.009G 142500	potassium transporter 2	nucleus;integral component of membrane;DNA binding;zinc ion binding;potassium ion transmembrane transporter activity;chloroplast organization;myo-inositol hexakisphosphate biosynthetic process;positive regulation of transcription, DNA-templated;potassium ion transmembrane transport		
Phvul.009G 143700	rho gtpase-activating protein ren1-like isoform x2	GTPase activator activity;signal transduction;positive regulation of GTPase activity		
Phvul.009G 152400	kda class i heat shock protein	cytoplasm		
Phvul.009G 152500	kda class i heat shock protein	cytoplasm		
Phvul.009G 153300	hypothetical protein PHAVU_009G153 300g	integral component of membrane		
Phvul.009G 155800	pentatricopeptide repeat-containing protein	microtubule-severing ATPase activity;metabolic process	ec:3.6.1.3-adenylpyrophosphatase;ec:3.6.1;ec	Purine metabolismPurine

			:3.6.4.3;ec:3.6.1.15-phosphatase	metabolism;Thiamine metabolism
Phvul.009G156500	cation h(+) antiporter 15	integral component of membrane;monovalent cation:proton antiporter activity;potassium ion transport;sodium ion transport;regulation of pH;plant-type cell wall modification;pollen tube growth;hydrogen ion transmembrane transport		
Phvul.009G158100	scorpion toxin-like knottin superfamily protein	defense response		
Phvul.009G160300	hypothetical protein PHAVU_009G160300g			
Phvul.009G161000	ethylene-responsive transcription factor erf114-like	nucleus;DNA binding;transcription factor activity, sequence-specific DNA binding;transcription, DNA-templated;regulation of transcription, DNA-templated		
Phvul.009G162000	transcription factor ice1-like	2-alkenal reductase [NAD(P)] activity;protein dimerization activity;oxidation-reduction process	ec:1.3.1.74	
Phvul.009G168000	hypothetical protein PHAVU_009G168000g			
Phvul.009G173900	hypothetical protein PHAVU_009G173900g			
Phvul.009G176400	lrr receptor-like serine threonine-protein kinase gso1	integral component of membrane;kinase activity;2-alkenal reductase [NAD(P)] activity;phosphorylation;oxidation-reduction process	ec:1.3.1.74	
Phvul.009G179600	transcription factor bhlh36	DNA binding;protein dimerization activity		
Phvul.009G182300	betaine aldehyde dehydrogenase	nucleotide binding;betaine-aldehyde dehydrogenase activity;aminobutyraldehyde dehydrogenase activity;1-pyrroline dehydrogenase activity;oxidation-reduction process	ec:1.2.1.19-dehydrogenase;ec:1.2.1.8-dehydrogenase;ec:1.2.1.3-dehydrogenase (NAD+)	Arginine and proline metabolism;beta-Alanine metabolismGlycine, serine and threonine metabolismArginine and proline metabolism;Chloroalkane and chloroalkene degradation;Pyruvate metabolism;Biosynthesis of antibiotics;Tryptophan metabolism;Valine, leucine and isoleucine degradation;Limonene and pinene degradation;beta-Alanine metabolism;Lysine degradation;Glycolysis / Gluconeogenesis;Fatty acid degradation;Glycerolipid metabolism;Ascorbate and aldarate metabolism;Histidine metabolism;Pentose and glucuronate interconversions
Phvul.009G186700	disulfide bond formation protein b			
Phvul.009G196200	zein-binding protein			
Phvul.009G197000	ids4-like protein	cellular response to phosphate starvation		
Phvul.009G207800	non-functional nadph-dependent	oxidoreductase activity;transferase activity, transferring acyl groups;oxidation-reduction process		

	codeinone reductase 2-like			
Phvul.009G 210900	auxin efflux carrier component 2	integral component of membrane;transmembrane transport		
Phvul.009G 211200	uncharacterized protein CPUR_03533			
Phvul.009G 224300	hypothetical protein PHAVU_009G224 300g			
Phvul.009G 239700	g-type lectin s-receptor-like serine threonine-protein kinase	protein serine/threonine kinase activity;transmembrane receptor protein tyrosine kinase activity;ATP binding;carbohydrate binding;peptidyl-tyrosine phosphorylation	ec:2.7.10.1;ec:2.7.11;ec:2.7.10	
Phvul.009G 239900	g-type lectin s-receptor-like serine threonine-protein kinase	protein serine/threonine kinase activity;transmembrane receptor protein tyrosine kinase activity;ATP binding;carbohydrate binding;peptidyl-tyrosine phosphorylation	ec:2.7.10.1;ec:2.7.11;ec:2.7.10	
Phvul.009G 240500	upf0481 protein at3g47200-like			
Phvul.009G 240600	glycoside hydrolase family 18 protein	hydrolase activity, hydrolyzing O-glycosyl compounds;carbohydrate metabolic process		
Phvul.009G 244000	isoflavone 2 -hydroxylase-like	monooxygenase activity;iron ion binding;oxidoreductase activity, acting on paired donors, with incorporation or reduction of molecular oxygen;heme binding;oxidation-reduction process		
Phvul.009G 259600	homeodomain-like superfamily protein isoform 2	transcription factor activity, sequence-specific DNA binding;sequence-specific DNA binding;circadian rhythm;response to cold;response to red or far red light;myo-inositol hexakisphosphate biosynthetic process;negative regulation of circadian rhythm;regulation of protein homodimerization activity;negative regulation of transcription, DNA-templated;positive regulation of transcription, DNA-templated;long-day photoperiodism, flowering		
Phvul.009G 261400	ring-h2 finger protein atl3	zinc ion binding		
Phvul.010G 000300	leucoanthocyanidin dioxygenase-like	iron ion binding;leucocyanidin oxygenase activity;oxidation-reduction process	ec:1.14.11.19-oxygenase;ec:1.14.11	Flavonoid biosynthesis
Phvul.010G 003400	hypothetical protein PHAVU_010G003 400g			
Phvul.010G 004000	protein nrt1 ptr family -like	membrane;transporter activity;transport		
Phvul.010G 004300	protein nrt1 ptr family -like	membrane;transporter activity;transport		
Phvul.010G 004900	disease resistance rpp13-like protein 1	hydrolase activity;ADP binding;metabolic process		
Phvul.010G 005600	plant t7h20-70 protein			
Phvul.010G 008800	glycinol 4-dimethylallyltransferase-like	plastid;integral component of membrane;prenyltransferase activity;cellular amino acid metabolic process;fatty acid metabolic process;cellular aromatic compound metabolic process;coenzyme metabolic process;sulfur compound metabolic process;lipid biosynthetic process;heterocycle metabolic process;regulation of biological process;monocarboxylic acid biosynthetic process;organic cyclic compound metabolic process	ec:2.5.1	
Phvul.010G 009400	protein nrt1 ptr family -like	integral component of membrane;transporter activity;oligopeptide transport		
Phvul.010G 012300	type 2 metallothionein	hydrolase activity;metal ion binding;metabolic process		
Phvul.010G 012900	cytochrome p450	integral component of membrane;monooxygenase activity;iron ion binding;oxidoreductase activity, acting on paired donors, with incorporation or reduction of molecular oxygen;heme binding;oxidation-reduction process		

Phvul.010G 013000	cytochrome p450	integral component of membrane;monooxygenase activity;iron ion binding;oxidoreductase activity, acting on paired donors, with incorporation or reduction of molecular oxygen;heme binding;oxidation-reduction process		
Phvul.010G 013100	isoflavone 2 -hydroxylase	integral component of membrane;iron ion binding;heme binding;isoflavone 2'-hydroxylase activity;oxidation-reduction process	ec:1.14.13;ec:1.14.13.89-2'-hydroxylase	Isoflavonoid biosynthesis
Phvul.010G 013700	verticillium wilt disease resistance protein	transferase activity;metabolic process		
Phvul.010G 014100	vinorine synthase-like	shikimate O-hydroxycinnamoyltransferase activity;metabolic process	ec:2.3.1;ec:2.3.1.133-O-hydroxycinnamoyl transferase	Stilbenoid, diarylheptanoid and gingerol biosynthesis;Flavonoid biosynthesis;Phenylpropanoid biosynthesis
Phvul.010G 016300	transcriptional regulator rabbit ears	metal ion binding		
Phvul.010G 019000	hypothetical protein PHAVU_010G019000g, partial			
Phvul.010G 019300	hypothetical protein PHAVU_010G019300g			
Phvul.010G 021000	gdsl esterase lipase 1	hydrolase activity, acting on ester bonds;9,9'-dicis-carotene:quinone oxidoreductase activity;7,9,9'-tricis-neurosporene:quinone oxidoreductase activity;9,9'-di-cis-zeta-carotene desaturation to 7,9,7',9'-tetra-cis-lycopene;oxidation-reduction process	ec:1.3.5.6-desaturase	Carotenoid biosynthesis
Phvul.010G 022000	ninja-family protein afp2-like			
Phvul.010G 031900	cysteine-rich rlk (receptor-like kinase) protein	protein serine/threonine kinase activity;ATP binding;carbohydrate binding;protein phosphorylation	ec:2.7.11	
Phvul.010G 036100	feruloyl ortho-hydroxylase 1-like	iron ion binding;oxidoreductase activity, acting on paired donors, with incorporation or reduction of molecular oxygen, 2-oxoglutarate as one donor, and incorporation of one atom each of oxygen into both donors;oxidation-reduction process	ec:1.14.11	
Phvul.010G 050500	ethylene-responsive transcription factor 13-like	nucleus;DNA binding;transcription factor activity, sequence-specific DNA binding;transcription, DNA-templated;regulation of transcription, DNA-templated;response to stress;response to abiotic stimulus;response to abscisic acid;response to jasmonic acid;ethylene-activated signaling pathway;response to chitin;regulation of programmed cell death;cellular response to acid chemical;cellular response to oxygen-containing compound		
Phvul.010G 050700	ap2-domain dna-binding protein	nucleus;DNA binding;transcription factor activity, sequence-specific DNA binding;transcription, DNA-templated;regulation of transcription, DNA-templated		
Phvul.010G 051100	uncharacterized transporter ybr287w-like	integral component of membrane;transmembrane transport		
Phvul.010G 054200	tmv resistance protein n	ADP binding;signal transduction		
Phvul.010G 054400	tmv resistance protein n-like	ADP binding;signal transduction		
Phvul.010G 056200	squamosa promoter-binding-like protein 7	nucleus;DNA binding		
Phvul.010G 057900	wrky transcription factor	transcription factor activity, sequence-specific DNA binding;sequence-specific DNA binding;regulation of transcription, DNA-templated		
Phvul.010G 063800	disease resistance response protein 206			
Phvul.010G 073500	phosphatidylethanolamine-binding protein	seed dormancy process;myo-inositol hexakisphosphate biosynthetic process;positive regulation of transcription, DNA-templated		

Phvul.010G 073900	hypothetical protein PHAVU_010G073 900g			
Phvul.010G 077200	cytochrome p450 family 71 protein	monooxygenase activity;iron ion binding;oxidoreductase activity, acting on paired donors, with incorporation or reduction of molecular oxygen;heme binding;oxidation-reduction process		
Phvul.010G 083700	myeloid leukemia factor 1			
Phvul.010G 093500	bon1-associated-like protein			
Phvul.010G 096900	f-box lrr-repeat protein at5g41630-like			
Phvul.010G 097800	math domain protein			
Phvul.010G 101500	oxidoreductase ferric-chelate reductase	integral component of membrane;oxidoreductase activity;oxidation-reduction process		
Phvul.010G 103900	1-aminocyclopropane-1-carboxylate synthase 1-like	1-aminocyclopropane-1-carboxylate synthase activity;pyridoxal phosphate binding;1-aminocyclopropane-1-carboxylate biosynthetic process	ec:4.4.1.14-synthase	Cysteine and methionine metabolism
Phvul.010G 107400	bag family molecular chaperone regulator 6	chaperone binding		
Phvul.010G 111000	mitogen-activated protein kinase kinase kinase 7-like	intracellular;MAP kinase kinase kinase activity;protein serine/threonine/tyrosine kinase activity;ATP binding;MAPK cascade;activation of MAPKK activity	ec:2.7.12.1;ec:2.7.11.25;ec:2.7.11	
Phvul.010G 111200	protein suppressor of phya-105 1	protein kinase activity;ATP binding;ligase activity;2-alkenal reductase [NAD(P)] activity;protein phosphorylation;multicellular organism development;response to light stimulus;regulation of biological process;oxidation-reduction process	ec:1.3.1.74	
Phvul.010G 117200	homeobox-leucine zipper protein athb-12-like	nucleus;transcription factor activity, sequence-specific DNA binding;sequence-specific DNA binding;transcription, DNA-templated;regulation of transcription, DNA-templated		
Phvul.010G 120200	caffeic acid o-methyltransferase	O-methyltransferase activity;quercetin 3-O-methyltransferase activity;protein dimerization activity;methylation	ec:2.1.1;ec:2.1.1.76-3-O-methyltransferase	Flavone and flavonol biosynthesis
Phvul.010G 122500	ring-h2 finger protein atl60	zinc ion binding;ligase activity;metabolic process		
Phvul.010G 124000	duf581 family protein	linoleate 13S-lipoxygenase activity;metal ion binding;oxidation-reduction process	ec:1.13.11.12-13S-lipoxygenase;ec:1.13.11	alpha-Linolenic acid metabolism;Linoleic acid metabolism
Phvul.010G 124800	gdsl esterase lipase 5-like	lipase activity;9,9'-dicis-carotene:quinone oxidoreductase activity;7,9,9'-tricis-neurosporene:quinone oxidoreductase activity;proline transport;9,9'-di-cis-zeta-carotene desaturation to 7,9,7',9'-tetra-cis-lycopene;oxidation-reduction process	ec:1.3.5.6-desaturase;ec:3.1.1	Carotenoid biosynthesis
Phvul.010G 125300	kda class iii heat shock protein	protein folding		
Phvul.010G 127500	probable inactive leucine-rich repeat receptor-like protein kinase at5g20690	intracellular;integral component of membrane;MAP kinase kinase kinase activity;transmembrane receptor protein tyrosine kinase activity;ATP binding;MAPK cascade;activation of MAPKK activity;peptidyl-tyrosine phosphorylation	ec:2.7.10.1;ec:2.7.11.25;ec:2.7.11;ec:2.7.10	
Phvul.010G 129900	auxin-binding protein abp19a	extracellular region;cell;extracellular matrix;manganese ion binding;nutrient reservoir activity;stomatal complex morphogenesis;cellular cation homeostasis;divalent metal ion transport		
Phvul.010G 135800	seed linoleate 9s-lipoxygenase	cytoplasm;oxidoreductase activity, acting on single donors with incorporation of molecular oxygen, incorporation of two atoms of oxygen;metal ion binding;oxylipin biosynthetic process;oxidation-reduction process	ec:1.13.11	
Phvul.010G 136700	disease resistance protein (tir-nbs-lrr class)	ADP binding;signal transduction		

Phvul.010G 137300	calmodulin-binding family protein			
Phvul.010G 139800	phospholipase d epsilon	membrane;phospholipase D activity;calcium ion binding;N-acylphosphatidylethanolamine-specific phospholipase D activity;lipid catabolic process;phosphatidylcholine metabolic process	ec:3.1.4.4-D;ec:3.1;ec:3.1.1	Ether lipid metabolism;Glycerophospholipid metabolism
Phvul.010G 140800	inorganic pyrophosphatase 2	pyridoxal phosphatase activity;protein dephosphorylation	ec:3.1.3.74-phosphatase;ec:3.1.3.41-nitrophenyl phosphatase;ec:3.1	Vitamin B6 metabolismAminobenzoate degradation
Phvul.010G 144200	stem 28 kda glycoprotein	acid phosphatase activity;protein dephosphorylation	ec:3.1.3.2-phosphatase;ec:3.1.3.41-nitrophenyl phosphatase;ec:3.1	Aminobenzoate degradationAminobenzoate degradation
Phvul.010G 144600	stem 28 kda glycoprotein	acid phosphatase activity;protein dephosphorylation	ec:3.1.3.2-phosphatase;ec:3.1.3.41-nitrophenyl phosphatase;ec:3.1	Aminobenzoate degradationAminobenzoate degradation
Phvul.010G 152300	probable 2-oxoglutarate-dependent dioxygenase aop1	iron ion binding;oxidoreductase activity, acting on paired donors, with incorporation or reduction of molecular oxygen, 2-oxoglutarate as one donor, and incorporation of one atom each of oxygen into both donors;oxidation-reduction process	ec:1.14.11	
Phvul.010G 152400	probable 2-oxoglutarate-dependent dioxygenase aop1	oxidoreductase activity, acting on paired donors, with incorporation or reduction of molecular oxygen, 2-oxoglutarate as one donor, and incorporation of one atom each of oxygen into both donors;metal ion binding;oxidation-reduction process	ec:1.14.11	
Phvul.010G 152800	root phototropism protein 3-like			
Phvul.010G 154300	duf761 domain protein			
Phvul.010G 154400	lactoylglutathione lyase glyoxalase i family protein	transferase activity;lyase activity;metabolic process		
Phvul.010G 155300	kda heat shock mitochondrial			
Phvul.010G 156200	palmitoyl-monogalactosyldiacylglycerol delta-7 chloroplastic	integral component of membrane;stearoyl-CoA 9-desaturase activity;fatty acid biosynthetic process;oxidation-reduction process	ec:1.14.19;ec:1.14.19.1-9-desaturase	Biosynthesis of unsaturated fatty acids
Phvul.010G 158400	helix loop helix dna-binding domain protein	2-alkenal reductase [NAD(P)] activity;protein dimerization activity;oxidation-reduction process	ec:1.3.1.74	
Phvul.010G 160700	bifunctional dihydroflavonol 4-reductase flavanone 4-reductase	cinnamoyl-CoA reductase activity;dihydrokaempferol 4-reductase activity;coenzyme binding;oxidation-reduction process	ec:1.1.1.219-4-reductase;ec:1.2.1.44-reductase	Flavonoid biosynthesisPhenylpropanoid biosynthesis
Phvul.010G 160900	cbl-interacting kinase	protein serine/threonine kinase activity;ATP binding;protein phosphorylation;signal transduction	ec:2.7.11	
Phvul.010G 161100	wrky transcription factor 40	transcription factor activity, sequence-specific DNA binding;sequence-specific DNA binding;regulation of transcription, DNA-templated		
Phvul.010G 161300	photosynthetic ndh subunit of subcomplex b chloroplastic	chloroplast thylakoid membrane;NAD(P)H dehydrogenase complex (plastoquinone);transferase activity, transferring glycosyl groups;pentose-phosphate shunt;photosynthetic electron transport in photosystem I;photosystem II assembly;carotenoid biosynthetic process		
Phvul.010G 162900	#NOME?	oxidoreductase activity, acting on CH-OH group of donors;mandelonitrile lyase activity;flavin adenine dinucleotide binding;oxidation-reduction process	ec:4.1.2.10-lyase	Cyanoamino acid metabolism
Phvul.010G 163600	hypothetical protein PHAVU_010G163600g			

Phvul.011G 008100	lrr receptor-like kinase family protein	integral component of membrane;protein kinase activity;ATP binding;oxidoreductase activity;protein phosphorylation;single-organism metabolic process;regulation of cellular process		
Phvul.011G 008600	pp890_soybn ame: full=protein propep890 short= 890 contains: ame: full=peptide ep890 flags: precursor			
Phvul.011G 012600	duf642 family protein			
Phvul.011G 014000	cytokinin dehydrogenase 6-like	UDP-N-acetylmuramate dehydrogenase activity;cytokinin dehydrogenase activity;flavin adenine dinucleotide binding;cytokinin metabolic process;oxidation-reduction process	ec:1.5.99.12-dehydrogenase;ec:1.1.1.158	Zeatin biosynthesis
Phvul.011G 015000	scarecrow-like protein 3 isoform x1	nucleus;transcription factor activity, transcription factor binding;transcription, DNA-templated;regulation of transcription, DNA-templated		
Phvul.011G 015400	hypothetical protein PHAVU_011G015 400g			
Phvul.011G 016100	small heat shock protein	mitochondrion		
Phvul.011G 016200	small heat shock protein			
Phvul.011G 022300	cinnamyl-alcohol dehydrogenase family protein	cinnamoyl-CoA reductase activity;coenzyme binding;oxidation-reduction process	ec:1.2.1.44-reductase	Phenylpropanoid biosynthesis
Phvul.011G 025800	aquaporin tip1-2-like	integral component of membrane;transporter activity;transport		
Phvul.011G 026200	hypothetical protein PHAVU_011G026 200g			
Phvul.011G 026700	proline-rich protein 4-like			
Phvul.011G 029200	hypothetical protein PHAVU_011G029 200g			
Phvul.011G 030200	lignin-forming anionic peroxidase	extracellular region;peroxidase activity;heme binding;metal ion binding;response to oxidative stress;hydrogen peroxide catabolic process;oxidation-reduction process	ec:1.11.1.7-lactoperoxidase	Phenylpropanoid biosynthesis
Phvul.011G 033800	chaperone protein dnaj chloroplastic-like			
Phvul.011G 037900	auxin-induced protein 15a-like	response to auxin		
Phvul.011G 044700	allergenic isoflavone reductase-like protein bet protein	oxidoreductase activity;oxidation-reduction process		
Phvul.011G 047800	cytochrome c biogenesis protein chloroplastic	maltose metabolic process;phosphatidylglycerol biosynthetic process;leaf morphogenesis;thylakoid membrane organization;carotenoid biosynthetic process;mRNA modification;starch biosynthetic process;isopentenyl diphosphate biosynthetic process, methylerythritol 4-phosphate pathway;cell differentiation;positive regulation of catalytic activity		
Phvul.011G 049900	myosin heavy chain kinase c			
Phvul.011G 051300	orn lys arg decarboxylase major region protein	lysine decarboxylase activity;metabolic process	ec:4.1.1.18-decarboxylase	Lysine degradation;Tropane, piperidine and pyridine alkaloid biosynthesis
Phvul.011G 055900	beta-glucosidase 12-like	beta-glucosidase activity;carbohydrate metabolic process	ec:3.2.1;ec:3.2.1.21-gentiobiase	Starch and sucrose metabolism;Cyanoamino acid metabolism;Phenylpropanoid biosynthesis

Phvul.011G 056100	beta-glucosidase 12-like	beta-glucosidase activity;carbohydrate metabolic process	ec:3.2.1;ec:3.2.1.2 1-gentiobiase	Starch and sucrose metabolism;Cyanoamin o acid metabolism;Phenylpro panoid biosynthesis
Phvul.011G 060400	copper transporter 6-like	integral component of membrane;copper ion transmembrane transporter activity;copper ion transmembrane transport		
Phvul.011G 060500	copper transporter 6-like	integral component of membrane;copper ion transmembrane transporter activity;copper ion transmembrane transport		
Phvul.011G 062900	haloacid dehalogenase-like hydrolase	hydrolase activity;metabolic process		
Phvul.011G 066800	zinc finger protein zat11-like	metal ion binding		
Phvul.011G 067200	tonoplast intrinsic protein	integral component of membrane;urea transmembrane transporter activity;water channel activity;water transport;urea transmembrane transport		
Phvul.011G 070400	hypothetical protein PHAVU_011G070 400g			
Phvul.011G 072200	lob domain-containing protein 1-like	carbamoyl-phosphate synthase (glutamine-hydrolyzing) activity;metabolic process	ec:6.3.5.5-synthase (glutamine-hydrolysing)	Alanine, aspartate and glutamate metabolism;Pyrimidine metabolism
Phvul.011G 076400	bud13 homolog	nucleus;cytosol		
Phvul.011G 077600	beta-galactosidase 8 isoform 1	cell wall;beta-galactosidase activity;carbohydrate binding;carbohydrate metabolic process	ec:3.2.1;ec:3.2.1.2 3-lactase (ambiguous)	Other glycan degradation;Glycosphin golipid biosynthesis - ganglio series;Sphingolipid metabolism;Galactose metabolism;Glycosami noglycan degradation
Phvul.011G 081800	lysm domain receptor-like kinase 4	protein kinase activity;ATP binding;protein phosphorylation		
Phvul.011G 083500	high affinity nitrate transporter	integral component of membrane;transmembrane transport		
Phvul.011G 084300	4-coumarate: ligase-like protein	peroxisome;fatty-acyl-CoA synthase activity;4-coumarate-CoA ligase activity;Photinus-luciferin 4-monooxygenase (ATP-hydrolyzing) activity;jasmonic acid biosynthetic process;oxidation-reduction process	ec:2.3.1;ec:1.13.1 2;ec:2.3.1.86-synthase;ec:1.13. 12.7;ec:6.2.1.12-ligase	Fatty acid biosynthesisUbiquinon e and other terpenoid-quinone biosynthesis;Phenylala nine metabolism;Phenylpro panoid biosynthesis
Phvul.011G 089600	gaba transporter 1-like	plasma membrane;integral component of membrane;gamma-aminobutyric acid transmembrane transporter activity;gamma-aminobutyric acid transport		
Phvul.011G 089800	gaba transporter 1-like	plasma membrane;integral component of membrane;gamma-aminobutyric acid transmembrane transporter activity;gamma-aminobutyric acid transport		
Phvul.011G 091300	stress responsive a b barrel domain protein	chloroplast		
Phvul.011G 097100	momilactone a synthase	ubiquitin-protein transferase activity;oxidoreductase activity;ligase activity;protein ubiquitination;oxidation-reduction process		
Phvul.011G 099300	e3 ubiquitin-protein ligase pub24-like	ubiquitin-protein transferase activity;ligase activity;protein ubiquitination		
Phvul.011G 102700	aquaporin sip1-1-like	integral component of membrane;transporter activity;transport		
Phvul.011G 106300	zinc finger protein constans-like 14	intracellular;zinc ion binding		
Phvul.011G 109700	myb-related transcription factor	DNA binding		

Phvul.011G 119500	helix loop helix dna-binding domain protein	DNA binding;protein dimerization activity		
Phvul.011G 120100	protein longifolia 1-like			
Phvul.011G 125300	zinc finger ccch domain-containing protein 2-like	metal ion binding		
Phvul.011G 127100	kdel-tailed cysteine endopeptidase cep1-like	cysteine-type peptidase activity;2-alkenal reductase [NAD(P)] activity;proteolysis;oxidation-reduction process	ec:1.3.1.74;ec:3.4	
Phvul.011G 128200	peroxidase p7-like	extracellular region;peroxidase activity;heme binding;metal ion binding;response to oxidative stress;hydrogen peroxide catabolic process;oxidation-reduction process	ec:1.11.1.7-lactoperoxidase	Phenylpropanoid biosynthesis
Phvul.011G 135600	seed biotin-containing protein sbp65			
Phvul.011G 136600	fatty alcohol oxidase 3 isoform 1	integral component of membrane;intracellular membrane-bounded organelle;long-chain-alcohol oxidase activity;flavin adenine dinucleotide binding;alcohol metabolic process;response to cold;oxidation-reduction process	ec:1.1.3.20	
Phvul.011G 142300	scarecrow-like protein 21	transcription, DNA-templated;regulation of transcription, DNA-templated		
Phvul.011G 142600	(-)-germacrene d synthase-like isoform x3	magnesium ion binding;casbene synthase activity;metabolic process	ec:4.2.3.8-synthase	Diterpenoid biosynthesis
Phvul.011G 147100	g-type lectin s-receptor-like serine threonine-protein kinase	protein serine/threonine kinase activity;ATP binding;carbohydrate binding;protein phosphorylation;recognition of pollen	ec:2.7.11	
Phvul.011G 147800	nac domain-containing protein 72	nucleus;DNA binding;transcription, DNA-templated;regulation of transcription, DNA-templated		
Phvul.011G 148500	hypothetical protein PHAVU_011G148500g			
Phvul.011G 150400	g-type lectin s-receptor-like serine threonine-protein kinase	protein serine/threonine kinase activity;transmembrane receptor protein tyrosine kinase activity;ATP binding;zinc ion binding;carbohydrate binding;peptidyl-tyrosine phosphorylation;recognition of pollen	ec:2.7.10.1;ec:2.7.11;ec:2.7.10	
Phvul.011G 152700	g-type lectin s-receptor-like serine threonine-protein kinase at4g27290-like	protein serine/threonine kinase activity;ATP binding;carbohydrate binding;protein phosphorylation;recognition of pollen	ec:2.7.11	
Phvul.011G 160600	clavata3 esr gene family member			
Phvul.011G 160700	clavata3 esr gene family member			
Phvul.011G 163800	ankyrin repeat protein			
Phvul.011G 167000	acidic endochitinase	hydrolase activity, hydrolyzing O-glycosyl compounds;carbohydrate metabolic process		
Phvul.011G 169500	cell division control protein 2 homolog	cyclin-dependent protein serine/threonine kinase activity;ATP binding;protein phosphorylation;cell division;regulation of cell cycle	ec:2.7.11.22;ec:2.7.11	
Phvul.011G 182900	mlp-like protein 43	defense response;response to biotic stimulus		
Phvul.011G 183000	mlp-like protein 43	defense response;response to biotic stimulus		
Phvul.011G 183300	mlp-like protein 43	defense response;response to biotic stimulus		
Phvul.011G 183600	mlp-like protein 43	defense response;response to biotic stimulus		
Phvul.011G 183800	mlp-like protein 43	defense response;response to biotic stimulus		
Phvul.011G 183900	mlp-like protein 43	defense response;response to biotic stimulus		

Phvul.011G184200	pathogenesis-related protein bet v i family protein	defense response;response to biotic stimulus		
Phvul.011G184600	udp-glycosyltransferase 74e1-like	transferase activity, transferring hexosyl groups;metabolic process		
Phvul.011G189200	12-oxophytodienoate reductase 11	FMN binding;12-oxophytodienoate reductase activity;oxidation-reduction process	ec:1.3.1.42-reductase	alpha-Linolenic acid metabolism
Phvul.011G189300	12-oxophytodienoate reductase 11	FMN binding;12-oxophytodienoate reductase activity;oxidation-reduction process	ec:1.3.1.42-reductase	alpha-Linolenic acid metabolism
Phvul.011G189900	protein zinc induced facilitator-like 1	integral component of membrane;transporter activity;transmembrane transport		
Phvul.011G191400	hypothetical protein PHAVU_011G191400g	ADP binding		
Phvul.011G191900	proline-rich receptor-like protein kinase perk12	protein tyrosine kinase activity;ATP binding;peptidyl-tyrosine phosphorylation	ec:2.7.10	
Phvul.011G192400	hypothetical protein PHAVU_011G192400g	ADP binding		
Phvul.011G193600	hypothetical protein PHAVU_011G193600g	ADP binding		
Phvul.011G193800	hypothetical protein PHAVU_011G193800g	ADP binding		
Phvul.011G193900	cysteine-rich receptor-kinase-like protein	protein kinase activity;ATP binding;protein phosphorylation		
Phvul.011G194100	hypothetical protein PHAVU_011G194000g	ADP binding		
Phvul.011G194400	cysteine-rich receptor-like protein kinase 10-like	protein serine/threonine kinase activity;non-membrane spanning protein tyrosine kinase activity;ATP binding;peptidyl-tyrosine phosphorylation	ec:2.7.10.2-protein-tyrosine kinase;ec:2.7.11;ec:2.7.10	T cell receptor signaling pathway
Phvul.011G197000	probable carboxylesterase 15	carboxylic ester hydrolase activity;metabolic process	ec:3.1.1.1-ali-esterase	Drug metabolism - other enzymes
Phvul.011G198800	nb-lrr receptor	ADP binding		
Phvul.011G201000	nbs-lrr type disease resistance protein rpg1-b	ADP binding		
Phvul.011G201700	glycoside hydrolase family 1 protein	beta-glucosidase activity;carbohydrate metabolic process	ec:3.2.1;ec:3.2.1.21-gentiobiase	Starch and sucrose metabolism;Cyanoamino acid metabolism;Phenylpropanoid biosynthesis
Phvul.011G208300	zinc finger protein 512b			
Phvul.011G211600	hypothetical protein PHAVU_011G211600g			
Phvul.011G215300	myb-like hth transcriptional regulator family	DNA binding		
Phvul.L002700	probable zinc transporter 10	integral component of membrane;zinc ion transmembrane transporter activity;zinc II ion transmembrane transport		
Phvul.L003000	adipose-regulatory protein			

Phvul.L005900	two-pore potassium channel 3-like	vacuolar membrane;integral component of membrane;voltage-gated potassium channel activity;polysaccharide catabolic process;cellular potassium ion homeostasis;potassium ion transmembrane transport		
Phvul.L006800	nbs-lrr type disease resistance protein cnl-j9	ADP binding		
Phvul.L006900	triacylglycerol lipase 2-like	lipase activity;defense response to insect;fatty acid beta-oxidation	ec:3.1.1	
Phvul.L008100	vq motif protein			
Phvul.L009500	hypothetical protein PHAVU_L009500g			
Phvul.L011500	pentatricopeptide repeat-containing protein at1g20230-like	microtubule-severing ATPase activity;metabolic process	ec:3.6.1.3-adenylpyrophosphatase;ec:3.6.1;ec:3.6.4.3;ec:3.6.1.15-phosphatase	Purine metabolismPurine metabolism;Thiamine metabolism
XLOC_000483	---NA---			
XLOC_000683	disease resistance protein rga4	phosphoprotein phosphatase activity;ADP binding;protein dephosphorylation	ec:3.1.3.16-phosphatase;ec:3.1.3.41-nitrophenyl phosphatase;ec:3.1	T cell receptor signaling pathwayAminobenzoate degradation
XLOC_000689	---NA---			
XLOC_002282	transporter mch1	integral component of membrane;transmembrane transport		
XLOC_002350	---NA---			
XLOC_002627	---NA---			
XLOC_002736	---NA---			
XLOC_002758	---NA---			
XLOC_002766	---NA---			
XLOC_002785	---NA---			
XLOC_002793	hypothetical protein PHAVU_010G070700g			
XLOC_002805	hypothetical protein PHAVU_011G0958002g, partial			
XLOC_002817	---NA---			
XLOC_002827	---NA---			
XLOC_002839	---NA---			
XLOC_003618	copper transporter -like			
XLOC_003707	hypothetical protein PHAVU_009G238900g			
XLOC_003885	---NA---			
XLOC_005217	---NA---			
XLOC_005373	transcription factor gte1			
XLOC_006160	---NA---			
XLOC_006229	---NA---			

XLOC_006239	---NA---			
XLOC_006245	---NA---			
XLOC_006252	---NA---			
XLOC_006267	---NA---			
XLOC_006277	---NA---			
XLOC_006286	---NA---			
XLOC_006298	hypothetical protein glysoja_023492			
XLOC_006748	---NA---			
XLOC_006966	pre-mrna-processing-splicing factor 8-like	spliceosomal complex;U6 snRNA binding;U5 snRNA binding;mRNA splicing, via spliceosome		
XLOC_007575	lrr and nb-arc domain disease resistance protein			
XLOC_007780	---NA---			
XLOC_007865	hypothetical protein PHAVU_009G043500g	tRNA binding		
XLOC_008029	h aca ribonucleoprotein complex subunit 1-like protein 1			
XLOC_008379	pyrimidine-specific ribonucleoside hydrolase riha	xanthine dehydrogenase activity;hydrolase activity;oxidation-reduction process	ec:1.17.1.4-dehydrogenase;ec:1.17.1	Purine metabolism
XLOC_008533	---NA---			
XLOC_009318	isoflavone synthase 1	monooxygenase activity;iron ion binding;oxidoreductase activity, acting on paired donors, with incorporation or reduction of molecular oxygen;isomerase activity;heme binding;oxidation-reduction process		
XLOC_009320	---NA---			
XLOC_009321	---NA---			
XLOC_009332	hypothetical protein PHAVU_001G104900g, partial			
XLOC_009335	hypothetical protein PHAVU_005G070100g			
XLOC_009338	---NA---			
XLOC_009355	myb transcription factor	DNA binding		
XLOC_009372	hypothetical protein PHAVU_011G148300g			
XLOC_009383	---NA---			
XLOC_009385	---NA---			
XLOC_009395	werner syndrome-like exonuclease	nucleic acid binding;3'-5' exonuclease activity;nucleic acid phosphodiester bond hydrolysis	ec:3.1	
XLOC_009404	---NA---			
XLOC_009407	---NA---			

XLOC_009410	---NA---			
XLOC_009423	---NA---			
XLOC_009463	---NA---			
XLOC_009875	transcription initiation factor tfiid subunit 10			
XLOC_010325	---NA---			
XLOC_010340	nbs-lrr type disease resistance protein cnl-j9	ADP binding		
XLOC_010758	PREDICTED: uncharacterized protein LOC102665818			
XLOC_011258	---NA---			
XLOC_011259	hypothetical protein PHAVU_008G145700g, partial			
XLOC_011273	---NA---			
XLOC_011298	PREDICTED: uncharacterized protein LOC102664980			
XLOC_011299	---NA---			
XLOC_011309	---NA---			
XLOC_011313	hypothetical protein PHAVU_009G156200g			
XLOC_011335	hypothetical protein PHAVU_002G163900g			
XLOC_011339	---NA---			
XLOC_011342	---NA---			
XLOC_011343	---NA---			
XLOC_011346	hypothetical protein PHAVU_L006100g	metal ion binding		
XLOC_011347	---NA---			
XLOC_011353	---NA---			
XLOC_011356	---NA---			
XLOC_011689	nuclear pore complex protein nup98-nup96	nuclear pore;transport		
XLOC_011757	transcription factor gte1			
XLOC_011869	inter-alpha-trypsin inhibitor heavy chain-like protein	NAD+ ADP-ribosyltransferase activity;metabolic process	ec:2.4.2.30	
XLOC_012375	mlp-like protein 31	defense response;response to biotic stimulus		
XLOC_012682	probable leucine-rich repeat receptor-like serine threonine-protein kinase at3g14840	protein kinase activity;ATP binding;protein phosphorylation		

XLOC_012683	lrr receptor-like kinase	protein kinase activity;ATP binding;protein phosphorylation		
XLOC_012735	hypothetical protein PHAVU_005G084700g			
XLOC_012777	---NA---			
XLOC_013251	retrotransposon polyprotein	catalytic activity;metabolic process		
XLOC_013252	---NA---			
XLOC_013260	---NA---			
XLOC_013266	---NA---			
XLOC_013267	ribonuclease h partial	oxidoreductase activity		
XLOC_013284	---NA---			
XLOC_013316	---NA---			
XLOC_013644	retrotransposon expressed	macromolecule metabolic process;primary metabolic process		
XLOC_013645	histone-lysine n-h3 lysine-9 specific suvh5-like	intracellular organelle;binding;methyltransferase activity;methylation		
XLOC_013810	---NA---			
XLOC_015572	hypothetical protein PHAVU_001G197200g	cysteine-type peptidase activity;thiol-dependent ubiquitinyl hydrolase activity;ubiquitin-dependent protein catabolic process	ec:3.4.19.12;ec:3.4	
XLOC_015573	---NA---			
XLOC_015575	---NA---			
XLOC_015577	---NA---			
XLOC_015620	hypothetical protein			
XLOC_015623	wall associated kinase-like protein	kinase activity;polysaccharide binding;phosphorylation		
XLOC_015625	---NA---			
XLOC_016045	hypothetical protein PHAVU_007G0816001g			
XLOC_016155	exosome complex exonuclease rrp46 homolog isoform x1	kinesin complex;microtubule;microtubule motor activity;exonuclease activity;ATP binding;microtubule binding;mRNA splicing, via spliceosome;RNA methylation;protein targeting to mitochondrion;microtubule-based movement;protein maturation;nucleic acid phosphodiester bond hydrolysis	ec:3.6.1;ec:3.1;ec:3.6.1.15-phosphatase	Purine metabolism;Thiamine metabolism
XLOC_017321	cysteine-rich receptor-like protein kinase 29	protein serine/threonine kinase activity;non-membrane spanning protein tyrosine kinase activity;ATP binding;peptidyl-tyrosine phosphorylation	ec:2.7.10.2-protein-tyrosine kinase;ec:2.7.11;ec:2.7.10	T cell receptor signaling pathway
XLOC_017342	exosome complex exonuclease rrp46 homolog isoform x1	exonuclease activity;nucleic acid phosphodiester bond hydrolysis	ec:3.1	
XLOC_017343	cysteine-rich receptor-kinase-like protein	nucleotide binding;protein kinase activity;protein phosphorylation		
XLOC_018503	---NA---			
XLOC_018504	---NA---			
XLOC_018505	---NA---			
XLOC_018527	hypothetical protein			

	PHAVU_006G007700g			
XLOC_018539	---NA---			
XLOC_018563	---NA---			
XLOC_018564	calmodulin-binding family protein			
XLOC_018567	---NA---			
XLOC_020674	g-type lectin s-receptor-like serine threonine-kinase	protein serine/threonine kinase activity;ATP binding;carbohydrate binding;protein phosphorylation;recognition of pollen	ec:2.7.11	
XLOC_020681	dna-directed rna polymerase iii subunit rpc3	DNA binding;DNA-directed RNA polymerase activity;transcription, DNA-templated	ec:2.7.7.6-RNA polymerase	Purine metabolism;Pyrimidine metabolism
XLOC_020682	---NA---			
XLOC_020771	hypothetical protein PHAVU_002G203500g			
XLOC_020934	hypothetical protein LR48_Vigan46s000800			
XLOC_021551	hypothetical protein PHAVU_004G108700g			
XLOC_021560	---NA---			
XLOC_021563	---NA---			
XLOC_021564	---NA---			
XLOC_021565	hypothetical protein PHAVU_011G106500g			
XLOC_021571	---NA---			
XLOC_021572	hypothetical protein VITISV_008145			
XLOC_021576	---NA---			
XLOC_021586	hypothetical protein LR48_Vigan583s003200			
XLOC_021600	---NA---			
XLOC_021642	gag-pol polyprotein			
XLOC_021644	---NA---			
XLOC_021652	---NA---			
XLOC_021653	---NA---			
XLOC_021905	---NA---			
XLOC_021926	---NA---			
XLOC_024008	---NA---			
XLOC_024208	---NA---			
XLOC_024325	hypothetical protein MTR_3g073490			

XLOC_024326	---NA---			
XLOC_024327	---NA---			
XLOC_024340	---NA---			
XLOC_024342	---NA---			
XLOC_024356	---NA---			
XLOC_024362	---NA---			
XLOC_024378	---NA---			
XLOC_024387	---NA---			
XLOC_024388	---NA---			
XLOC_024404	---NA---			
XLOC_024405	endonuclease exonuclease phosphatase family protein			
XLOC_024557	hypothetical protein PHAVU_010G025500g	ATP binding;ADP binding;signal transduction		
XLOC_024793	serine acetyltransferase 5-like	cytosol;magnesium ion binding;serine O-acetyltransferase activity;terpene synthase activity;cysteine biosynthetic process from serine;protein targeting to membrane;response to wounding;response to fungus;jasmonic acid biosynthetic process;salicylic acid mediated signaling pathway;jasmonic acid mediated signaling pathway;regulation of plant-type hypersensitive response	ec:2.3.1;ec:2.3.1.30-O-acetyltransferase	Sulfur metabolism;Biosynthesis of antibiotics;Cysteine and methionine metabolism
XLOC_025114	---NA---			
XLOC_025625	---NA---			
XLOC_025627	---NA---			
XLOC_025787	pentatricopeptide repeat-containing protein at2g17140			
XLOC_025825	probable polygalacturonase	extracellular region;polygalacturonase activity;carbohydrate metabolic process;cell wall organization	ec:3.2.1.15-pectin depolymerase	Starch and sucrose metabolism;Pentose and glucuronate interconversions
XLOC_026121	---NA---			
XLOC_026123	---NA---			
XLOC_026129	---NA---			
XLOC_026139	---NA---			
XLOC_026160	hypothetical protein LR48_Vigan03g139700			
XLOC_026172	---NA---			
XLOC_026190	---NA---			
XLOC_026270	---NA---			
XLOC_026888	---NA---			
XLOC_026955	---NA---			
XLOC_027632	---NA---			

XLOC_027916	lysine-specific demethylase jmj16	nucleus;transcription factor activity, sequence-specific DNA binding;protein glycosylation;gravitropism;vegetative to reproductive phase transition of meristem;glucuronoxylan metabolic process;protein desumoylation;post-translational protein modification;xylan biosynthetic process;positive regulation of transcription, DNA-templated;hydrogen peroxide biosynthetic process		
XLOC_027985	---NA---			
XLOC_028038	---NA---			
XLOC_028432	---NA---			
XLOC_028436	disease resistance-dirigent domain protein			
XLOC_028457	cyclic nucleotide-gated ion channel 4-like	integral component of membrane;ion channel activity;ion transmembrane transport		
XLOC_028476	---NA---			
XLOC_028488	---NA---			
XLOC_028495	disease resistance rpp13-like protein 1			
XLOC_028589	---NA---			
XLOC_028640	---NA---			

Transcript ID	Sequence Description	GO Terms	Enzymes	Kegg Maps
**DEGs under high impact predicted effects (HIPE)**				
Phvul.001G000600.1	Fanconi anemia group J homolog isoform X1	DNA binding;ATP-dependent DNA helicase activity;ATP binding;nucleobase-containing compound metabolic process;DNA duplex unwinding	ec:3.6.1;ec:3.6.1.3-adenylpyrophosphatase;ec:3.6.1.15-phosphatase	Purine metabolismPurine metabolism;Thiamine metabolism
Phvul.001G000600.2	Fanconi anemia group J homolog isoform X1	DNA binding;ATP-dependent DNA helicase activity;ATP binding;nucleobase-containing compound metabolic process;DNA duplex unwinding	ec:3.6.1;ec:3.6.1.3-adenylpyrophosphatase;ec:3.6.1.15-phosphatase	Purine metabolismPurine metabolism;Thiamine metabolism
Phvul.001G000800.1	12-oxophytodienoate reductase 11	intracellular;NADPH dehydrogenase activity;FMN binding;12-oxophytodienoate reductase activity;oxidation-reduction process	ec:1.3.1.42-reductase;ec:1.6.99.1	alpha-Linolenic acid metabolism
Phvul.001G000800.2	12-oxophytodienoate reductase 11	intracellular;NADPH dehydrogenase activity;FMN binding;12-oxophytodienoate reductase activity;oxidation-reduction process	ec:1.3.1.42-reductase;ec:1.6.99.1	alpha-Linolenic acid metabolism
Phvul.001G001000.1	catalase	glyoxysome;catalase activity;heme binding;metal ion binding;response to hydrogen peroxide;hydrogen peroxide catabolic process;oxidation-reduction process;cellular oxidant detoxification	ec:1.11.1.7-lactoperoxidase;ec:1.11.1.6-equilase	Phenylpropanoid biosynthesisBiosynthesis of antibiotics;Tryptophan metabolism;Glyoxylate and dicarboxylate metabolism
Phvul.001G001300.1	4-coumarate--ligase-like 5	integral component of membrane;ligase activity;Photinus-luciferin 4-monooxygenase (ATP-hydrolyzing) activity;oxidation-reduction process	ec:1.13.12;ec:1.13.12.7	
Phvul.001G001500.1	L-ascorbate oxidase homolog	plant-type cell wall;plasmodesma;integral component of membrane;copper ion binding;L-ascorbate oxidase activity;oxidoreductase activity, oxidizing metal ions;oxidation-reduction process	ec:1.10.3.3-oxidase;ec:1.10.3	Ascorbate and aldarate metabolism
Phvul.001G001800.1	hypothetical protein PHAVU_001G001800g			
Phvul.001G002800.1	Calcium-dependent kinase 29	nucleus;cytoplasm;plasma membrane;calmodulin-dependent protein kinase activity;calcium ion binding;calmodulin binding;ATP binding;calcium-dependent protein serine/threonine kinase activity;systemic acquired resistance;salicylic acid biosynthetic process;abscisic acid-activated signaling pathway;peptidyl-serine phosphorylation;intracellular signal transduction;protein autophosphorylation	ec:2.7.11;ec:2.7.11.17	
Phvul.001G003900.1	UPF0496 At1g20180-like	integral component of membrane		
Phvul.001G005400.1	transcription elongation factor (TFIIS) family ,	elongin complex;DNA binding;transcription elongation from RNA polymerase II promoter		
Phvul.001G006000.1	vacuolar sorting-associated 35B-like	late endosome;retromer complex;protein transporter activity;intracellular protein transport;retrograde transport, endosome to Golgi		
Phvul.001G008000.1	Filament-like plant 4			
Phvul.001G009000.1	transcription factor RF2a-like	nucleus;transcription factor activity, sequence-specific DNA binding;sequence-specific DNA binding;regulation of transcription, DNA-templated		
Phvul.001G009000.2	transcription factor RF2a-like	nucleus;transcription factor activity, sequence-specific DNA binding;sequence-specific DNA binding;regulation of transcription, DNA-templated		
Phvul.001G009000.3	transcription factor RF2a-like	nucleus;transcription factor activity, sequence-specific DNA binding;sequence-specific DNA binding;regulation of transcription, DNA-templated		
Phvul.001G009300.1	lysine-specific histone demethylase 1 homolog 3-like isoform X1	nucleus;integral component of membrane;DNA binding;methyltransferase activity;oxidoreductase activity;transcription, DNA-templated;methylation;oxidation-reduction process		

Phvul.001G009500.1	ACCUMULATION AND REPLICATION OF CHLOROPLASTS 6, chloroplastic	integral component of chloroplast inner membrane;protein self-association;chloroplast fission;photosystem II assembly		
Phvul.001G010000.1	ROOT PRIMORDIUM DEFECTIVE 1	hydrolase activity;metabolic process;negative regulation of cell proliferation;lateral root morphogenesis		
Phvul.001G011900.1	Peptide upstream open reading frame 5	integral component of membrane		
Phvul.001G012200.1	Inositol-pentakisphosphate 2-kinase	ATP binding;inositol pentakisphosphate 2-kinase activity;phosphorylation	ec:2.7.1.158-2-kinase	Inositol phosphate metabolism;Phosphatidylinositol signaling system
Phvul.001G012200.2	Inositol-pentakisphosphate 2-kinase	ATP binding;inositol pentakisphosphate 2-kinase activity;phosphorylation	ec:2.7.1.158-2-kinase	Inositol phosphate metabolism;Phosphatidylinositol signaling system
Phvul.001G014000.1	sugar transporter ERD6-like 6	integral component of plasma membrane;sugar:proton symporter activity;glucose transmembrane transporter activity;2-alkenal reductase [NAD(P)] activity;proton transport;glucose import;oxidation-reduction process;glucose transmembrane transport	ec:1.3.1.74	
Phvul.001G014400.1	probable LRR receptor-like serine threonine- kinase At2g16250	integral component of membrane;transmembrane receptor protein tyrosine kinase activity;ATP binding;peptidyl-tyrosine phosphorylation	ec:2.7.11;ec:2.7.10;ec:2.7.10.1	
Phvul.001G014500.1	probable methyltransferase PMT28	endosome;trans-Golgi network;integral component of membrane;iron ion binding;methyltransferase activity;FMN binding;iron-sulfur cluster binding;methylation;oxidation-reduction process		
Phvul.001G014700.1	hypothetical protein PHAVU_001G014700g	cytosol;integral component of membrane;hydroxypyruvate reductase activity;glyoxylate reductase (NADP) activity	ec:1.1.1.81-reductase;ec:1.1.1.79-reductase (NADP+)	Glycine, serine and threonine metabolism;Glyoxylate and dicarboxylate metabolismPyruvate metabolism;Glyoxylate and dicarboxylate metabolism
Phvul.001G015400.1	uncharacterized membrane At1g75140	integral component of membrane		
Phvul.001G015600.1	lysophospholipid acyltransferase LPEAT1-like isoform X2	nucleus;integral component of membrane;1-acylglycerophosphocholine O-acyltransferase activity;phosphatidylglycerol biosynthetic process	ec:2.3.1.23-O-acyltransferase;ec:2.3.1	Glycerophospholipid metabolism
Phvul.001G015600.2	lysophospholipid acyltransferase LPEAT1-like isoform X1	nucleus;integral component of membrane;1-acylglycerophosphocholine O-acyltransferase activity;phosphatidylglycerol biosynthetic process	ec:2.3.1.23-O-acyltransferase;ec:2.3.1	Glycerophospholipid metabolism
Phvul.001G015600.3	lysophospholipid acyltransferase LPEAT1-like isoform X1	nucleus;integral component of membrane;1-acylglycerophosphocholine O-acyltransferase activity;phosphatidylglycerol biosynthetic process	ec:2.3.1.23-O-acyltransferase;ec:2.3.1	Glycerophospholipid metabolism
Phvul.001G017600.1	plant MJB21-3	integral component of membrane;DNA-directed RNA polymerase activity;RNA biosynthetic process	ec:2.7.7.6-RNA polymerase	Purine metabolism;Pyrimidine metabolism
Phvul.001G018700.1	nucleobase-ascorbate transporter 3	plasma membrane;plasmodesma;integral component of membrane;transporter activity;transmembrane transport		
Phvul.001G019900.1	probable inactive heme oxygenase 2, chloroplastic	chloroplast;heme oxygenase (decyclizing) activity;heme oxidation;phytochromobilin biosynthetic process;isopentenyl diphosphate biosynthetic process, methylerythritol 4-phosphate pathway	ec:1.14.99.3	
Phvul.001G020200.1	patatin 2	cytoplasm;membrane;phospholipase activity;acylglycerol lipase activity;lipid catabolic process	ec:3.1.1.23-lipase;ec:3.1.1;ec:3.1.1.1-ali-esterase	Glycerolipid metabolismDrug metabolism - other enzymes

Phvul.001G021200.1	probable phosphatase 2C 12 isoform X1	cytosol;protein serine/threonine phosphatase activity;protein dephosphorylation	ec:3.1;ec:3.1.3.41-nitrophenyl phosphatase;ec:3.1.3.16-phosphatase	Aminobenzoate degradationT cell receptor signaling pathway
Phvul.001G023100.1	probable BOI-related E3 ubiquitin- ligase 2	nucleus;ubiquitin-protein transferase activity;zinc ion binding;hormone-mediated signaling pathway;protein ubiquitination;regulation of defense response;photoperiodism, flowering		
Phvul.001G023800.1	aceous RNase P 1, chloroplastic mitochondrial			
Phvul.001G024200.1	kinesin FRA1 isoform X1	kinesin complex;microtubule;microtubule motor activity;ATP binding;microtubule binding;ATPase activity;microtubule-based movement;metabolic process	ec:3.6.1;ec:3.6.1.3-adenylpyrophosphatase;ec:3.6.1.15-phosphatase	Purine metabolismPurine metabolism;Thiamine metabolism
Phvul.001G025100.1	probable carbohydrate esterase At4g34215			
Phvul.001G026300.1	transcription initiation factor TFIID subunit 4b isoform X1	transcription factor TFIID complex;translation initiation factor activity;protein heterodimerization activity;DNA-templated transcription, initiation;translational initiation		
Phvul.001G026300.2	transcription initiation factor TFIID subunit 4b isoform X1	transcription factor TFIID complex;translation initiation factor activity;protein heterodimerization activity;DNA-templated transcription, initiation;translational initiation		
Phvul.001G028600.1	hypothetical protein PHAVU_001G028600g, partial			
Phvul.001G029000.2	FKBP-type peptidyl-prolyl cis-trans isomerase	endoplasmic reticulum membrane;peptidyl-prolyl cis-trans isomerase activity;FK506 binding;protein peptidyl-prolyl isomerization;chaperone-mediated protein folding	ec:5.2.1.8	
Phvul.001G030200.1	PAX3- and PAX7-binding 1	U2-type post-mRNA release spliceosomal complex;RNA polymerase II core promoter proximal region sequence-specific DNA binding;transcriptional repressor activity, RNA polymerase II core promoter proximal region sequence-specific binding;negative regulation of transcription from RNA polymerase II promoter;spliceosomal complex disassembly		
Phvul.001G032900.1	EARLY FLOWERING 3			
Phvul.001G032900.2	EARLY FLOWERING 3			
Phvul.001G034600.1	calcineurin B 10	plant-type vacuole membrane;integral component of membrane;catalytic activity;calcium ion binding;polysaccharide biosynthetic process;multidimensional cell growth;cell tip growth;regulation of hormone levels;calcium-mediated signaling;hyperosmotic salinity response;regulation of potassium ion transport;anthocyanin accumulation in tissues in response to UV light;root hair elongation;cell wall organization;regulation of ion homeostasis		
Phvul.001G034600.2	calcineurin B 10	cytoplasm;membrane;calcium ion binding;metabolic process;response to abiotic stimulus;cellular component organization;cell growth;single-multicellular organism process;cellular developmental process;regulation of biological process;developmental growth involved in morphogenesis;regulation of biological quality		
Phvul.001G035000.1	DUF1230 family	chloroplast;integral component of membrane;chlorophyll catabolic process		
Phvul.001G035000.2	DUF1230 family	chloroplast;integral component of membrane;chlorophyll catabolic process		
Phvul.001G035000.3	DUF1230 family	chloroplast;integral component of membrane;chlorophyll catabolic process		
Phvul.001G035300.1	BPS1, chloroplastic			

Phvul.001G035500.1	glycine dehydrogenase (decarboxylating), mitochondrial	mitochondrion;cytosol;glycine cleavage complex;glycine dehydrogenase (decarboxylating) activity;glycine binding;glycine decarboxylation via glycine cleavage system;oxidation-reduction process	ec:1.4.4.2-dehydrogenase (aminomethyl-transferring)	Glycine, serine and threonine metabolism
Phvul.001G036300.1	BAAT acyl- thioester hydrolase carboxy-terminal	peroxisome;hydrolase activity;metabolic process		
Phvul.001G037500.1	probable magnesium transporter NIPA9 isoform X1	integral component of membrane;magnesium ion transmembrane transporter activity;magnesium ion transport;magnesium ion transmembrane transport		
Phvul.001G038700.1	F-box LRR-repeat 15-like			
Phvul.001G040200.2	hypothetical protein PHAVU_001G040200g			
Phvul.001G043000.1	LRR receptor-like serine threonine-kinase GSO1	integral component of membrane		
Phvul.001G043800.1	F-box only 13-like			
Phvul.001G045100.1	adenine nucleotide alpha hydrolase-like domain kinase	protein kinase activity;ATP binding;protein phosphorylation;response to stress		
Phvul.001G045100.2	adenine nucleotide alpha hydrolase-like domain kinase	protein kinase activity;ATP binding;protein phosphorylation;response to stress		
Phvul.001G045500.1	probable folate-biopterin transporter 2	integral component of membrane;transport		
Phvul.001G045900.1	U-box domain-containing 13			
Phvul.001G045900.2	U-box domain-containing 13			
Phvul.001G047000.1	aspartate aminotransferase	mitochondrial matrix;chloroplast stroma;chloroplast envelope;stromule;apoplast;L-aspartate:2-oxoglutarate aminotransferase activity;pyridoxal phosphate binding;identical protein binding;L-phenylalanine:2-oxoglutarate aminotransferase activity;2-oxoglutarate metabolic process;aspartate metabolic process;glutamate metabolic process;ethylene biosynthetic process	ec:2.6.1.57-transaminase;ec:2.6.1.9-transaminase;ec:2.6.1.5-transaminase;ec:2.6.1.1-transaminase	Biosynthesis of antibiotics;Tropane, piperidine and pyridine alkaloid biosynthesis;Cysteine and methionine metabolism;Novobiocin biosynthesis;Phenylalanine, tyrosine and tryptophan biosynthesis;Isoquinoline alkaloid biosynthesis;Lysine biosynthesis;Phenylalanine metabolism;Tyrosine metabolismBiosynthesis of antibiotics;Tropane, piperidine and pyridine alkaloid biosynthesis;Novobiocin biosynthesis;Phenylalanine, tyrosine and tryptophan biosynthesis;Phenylalanine metabolism;Tyrosine metabolism;Histidine metabolismUbiquinone and other terpenoid-quinone biosynthesis;Biosynthesis of antibiotics;Tropane, piperidine and pyridine alkaloid biosynthesis;Cysteine and methionine

				metabolism;Novobiocin biosynthesis;Phenylalanine, tyrosine and tryptophan biosynthesis;Isoquinoline alkaloid biosynthesis;Phenylalanine metabolism;Tyrosine metabolismArginine and proline metabolism;Biosynthesis of antibiotics;Arginine biosynthesis;Carbon fixation in photosynthetic organisms;Tropane, piperidine and pyridine alkaloid biosynthesis;Cysteine and methionine metabolism;Novobiocin biosynthesis;Phenylalanine, tyrosine and tryptophan biosynthesis;Isoquinoline alkaloid biosynthesis;Phenylalanine metabolism;Tyrosine metabolism;Alanine, aspartate and glutamate metabolism
Phvul.001G048800.2	glycerophosphodiester phosphodiesterase kinase domain-containing GDPDL2-like	integral component of membrane;protein serine/threonine kinase activity;ATP binding;polysaccharide binding;protein phosphorylation	ec:2.7.11	
Phvul.001G050000.1	uncharacterized vacuolar membrane YML018C-like isoform X1	integral component of membrane		
Phvul.001G052800.1	peptidyl-prolyl cis-trans isomerase CYP95-like isoform X1	peptidyl-prolyl cis-trans isomerase activity;protein peptidyl-prolyl isomerization;protein folding	ec:5.2.1.8	
Phvul.001G053400.1	carbon catabolite repressor 4 homolog 6 isoform X1			
Phvul.001G055000.1	gibberellin 3-beta-dioxygenase 1-like	iron ion binding;gibberellin 3-beta-dioxygenase activity;oxidation-reduction process	ec:1.14.11.15-3beta-dioxygenase;ec:1.14.11	Diterpenoid biosynthesis
Phvul.001G055100.1	tify 8 , partial	nucleus;transcription corepressor activity;response to wounding;regulation of defense response;negative regulation of nucleic acid-templated transcription;regulation of jasmonic acid mediated signaling pathway		
Phvul.001G055200.1	pentatricopeptide repeat-containing At5g11310, mitochondrial			
Phvul.001G055600.1	E3 ubiquitin- ligase At1g63170	plasma membrane;integral component of membrane;zinc ion binding;hydrolase activity;ligase activity;metabolic process		
Phvul.001G056400.1	glucan endo-1,3-beta-glucosidase 4-like	hydrolase activity, hydrolyzing O-glycosyl compounds;protein histidine kinase activity;carbohydrate metabolic process;peptidyl-histidine phosphorylation	ec:2.7.13.3	
Phvul.001G057900.1	RNA methyltransferase At5g10620	cytoplasm;methyltransferase activity;rRNA processing;methylation		

Phvul.001G058100.1	core-2 I-branching enzyme	integral component of membrane;acetylglucosaminyltransferase activity;metabolic process	ec:2.4.1	
Phvul.001G058100.2	core-2 I-branching enzyme	integral component of membrane;acetylglucosaminyltransferase activity;metabolic process	ec:2.4.1	
Phvul.001G058100.3	core-2 I-branching enzyme	integral component of membrane;acetylglucosaminyltransferase activity;metabolic process	ec:2.4.1	
Phvul.001G058900.1	plasma membrane ATPase 4	integral component of plasma membrane;intracellular membrane-bounded organelle;ATP binding;hydrogen-exporting ATPase activity, phosphorylative mechanism;metal ion binding;ATP biosynthetic process;regulation of intracellular pH;hydrogen ion transmembrane transport	ec:3.6.1;ec:3.6.1.3-adenylpyrophosphatase;ec:3.6.1.15-phosphatase;ec:3.6.3.6-ATPase	Purine metabolismPurine metabolism;Thiamine metabolismOxidative phosphorylation
Phvul.001G059500.1	pentatricopeptide repeat-containing At5g10690	cytosol;catalytic activity;mRNA modification;isopentenyl diphosphate biosynthetic process, methylerythritol 4-phosphate pathway		
Phvul.001G059700.1	DNA ligase 1-like	integral component of membrane		
Phvul.001G060000.1	two-on-two hemoglobin-3	oxygen binding;heme binding		
Phvul.001G060700.1	dihydrofolate synthetase	tetrahydrofolylpolyglutamate synthase activity;ATP binding;dihydrofolate synthase activity;one-carbon metabolic process;dihydrofolate biosynthetic process;embryo development ending in seed dormancy;tetrahydrofolylpolyglutamate biosynthetic process	ec:6.3.2.17-synthase;ec:6.3.2.12-synthase	Folate biosynthesisFolate biosynthesis
Phvul.001G060700.2	tetrahydrofolylpolyglutamate synthase	tetrahydrofolylpolyglutamate synthase activity;ATP binding;dihydrofolate synthase activity;one-carbon metabolic process;dihydrofolate biosynthetic process;embryo development ending in seed dormancy;tetrahydrofolylpolyglutamate biosynthetic process	ec:6.3.2.17-synthase;ec:6.3.2.12-synthase	Folate biosynthesisFolate biosynthesis
Phvul.001G060700.3	tetrahydrofolylpolyglutamate synthase	tetrahydrofolylpolyglutamate synthase activity;ATP binding;dihydrofolate synthase activity;dihydrofolate biosynthetic process;tetrahydrofolylpolyglutamate biosynthetic process	ec:6.3.2.17-synthase;ec:6.3.2.12-synthase	Folate biosynthesisFolate biosynthesis
Phvul.001G060700.4	dihydrofolate synthetase	tetrahydrofolylpolyglutamate synthase activity;ATP binding;dihydrofolate synthase activity;one-carbon metabolic process;dihydrofolate biosynthetic process;embryo development ending in seed dormancy;tetrahydrofolylpolyglutamate biosynthetic process	ec:6.3.2.17-synthase;ec:6.3.2.12-synthase	Folate biosynthesisFolate biosynthesis
Phvul.001G060700.5	tetrahydrofolylpolyglutamate synthase	tetrahydrofolylpolyglutamate synthase activity;ATP binding;dihydrofolate synthase activity;dihydrofolate biosynthetic process;tetrahydrofolylpolyglutamate biosynthetic process	ec:6.3.2.17-synthase;ec:6.3.2.12-synthase	Folate biosynthesisFolate biosynthesis
Phvul.001G060700.6	tetrahydrofolylpolyglutamate synthase	tetrahydrofolylpolyglutamate synthase activity;ATP binding;dihydrofolate synthase activity;one-carbon metabolic process;dihydrofolate biosynthetic process;embryo development ending in seed dormancy;tetrahydrofolylpolyglutamate biosynthetic process	ec:6.3.2.17-synthase;ec:6.3.2.12-synthase	Folate biosynthesisFolate biosynthesis
Phvul.001G061400.1	RNA polymerase sigma factor sigE, chloroplastic mitochondrial-like	chloroplast;plastid sigma factor activity;DNA binding;transcription factor activity, sequence-specific DNA binding;DNA-templated transcription, initiation;response to blue light;chloroplast organization;photosystem II assembly;myo-inositol hexakisphosphate biosynthetic process;positive regulation of transcription, DNA-templated		
Phvul.001G064800.1	ras-related RABC1	intracellular;membrane;GTPase activity;GTP binding;intracellular protein transport;nucleocytoplasmic transport;small GTPase mediated signal transduction;metabolic process	ec:3.6.1;ec:3.6.1.15-phosphatase	Purine metabolism;Thiamine metabolism

Phvul.001G066400.1	wall-associated receptor kinase-like 16 isoform X2	integral component of membrane;protein serine/threonine kinase activity;calcium ion binding;ATP binding;polysaccharide binding;protein phosphorylation	ec:2.7.11	
Phvul.001G067200.1	probable methyltransferase PMT10	integral component of membrane;S-adenosylmethionine-dependent methyltransferase activity;methylation		
Phvul.001G068400.1	pentatricopeptide repeat-containing At2g35030, mitochondrial	zinc ion binding;microtubule-severing ATPase activity;metabolic process	ec:3.6.1;ec:3.6.1.3-adenylpyrophosphatase;ec:3.6.4.3;ec:3.6.1.15-phosphatase	Purine metabolismPurine metabolism;Thiamine metabolism
Phvul.001G071400.1	probable receptor kinase At1g11050	plasma membrane;integral component of membrane;protein serine/threonine kinase activity;transmembrane receptor protein tyrosine kinase activity;ATP binding;peptidyl-tyrosine phosphorylation	ec:2.7.11;ec:2.7.10;ec:2.7.10.1	
Phvul.001G072300.1	U-box domain-containing 13			
Phvul.001G073100.1	UDP-glucuronate:xylan alpha-glucuronosyltransferase 3	endosome;trans-Golgi network;integral component of membrane;glucuronosyltransferase activity;plant-type secondary cell wall biogenesis;xylan biosynthetic process	ec:2.4.1.17-1-naphthol glucuronyltransferase	Drug metabolism - other enzymes;Drug metabolism - cytochrome P450;Metabolism of xenobiotics by cytochrome P450;Porphyrin and chlorophyll metabolism;Starch and sucrose metabolism;Ascorbate and aldarate metabolism;Retinol metabolism;Steroid hormone biosynthesis;Pentose and glucuronate interconversions
Phvul.001G074000.1	nuclear pore complex NUP85	nuclear pore outer ring;structural constituent of nuclear pore;mRNA export from nucleus;protein import into nucleus;photomorphogenesis;embryo development ending in seed dormancy;seed germination;regulation of flower development;meristem structural organization;vernalization response;maintenance of meristem identity;seed dormancy process;sugar mediated signaling pathway;vegetative to reproductive phase transition of meristem;cullin deneddylation;protein ubiquitination;histone methylation;protein deubiquitination;lipid storage;positive regulation of transcription, DNA-templated;response to freezing		
Phvul.001G074100.1	nuclear pore complex NUP85	nuclear pore outer ring;structural constituent of nuclear pore;mRNA export from nucleus;protein import into nucleus;photomorphogenesis;embryo development ending in seed dormancy;seed germination;regulation of flower development;meristem structural organization;vernalization response;maintenance of meristem identity;seed dormancy process;sugar mediated signaling pathway;vegetative to reproductive phase transition of meristem;cullin deneddylation;protein ubiquitination;histone methylation;protein deubiquitination;lipid storage;positive regulation of transcription, DNA-templated;response to freezing		
Phvul.001G074600.1	early nodulin-93-like	mitochondrion;integral component of membrane;response to ethylene		
Phvul.001G075000.1	aminoacrylate hydrolase	integral component of membrane;hydrolase activity;metabolic process		

Phvul.001G075200.1	ATPase family AAA domain-containing 1-like	integral component of membrane;ATP binding;microtubule-severing ATPase activity;metabolic process	ec:3.6.1;ec:3.6.1.3-adenylpyrophosphatase;ec:3.6.4.3;ec:3.6.1.15-phosphatase	Purine metabolismPurine metabolism;Thiamine metabolism
Phvul.001G075700.1	carbon catabolite repressor	integral component of membrane		
Phvul.001G076100.1	stress-induced receptor-like kinase	integral component of membrane;protein serine/threonine kinase activity;ATP binding;polysaccharide binding;protein phosphorylation	ec:2.7.11	
Phvul.001G076800.1	TATA-box-binding	nucleus;DNA binding;DNA-templated transcription, initiation		
Phvul.001G078300.1	haloacid dehalogenase-like hydrolase	chloroplast;metalloendopeptidase activity;trehalose-phosphatase activity;rRNA processing;proteolysis;dephosphorylation;small molecule biosynthetic process	ec:3.1;ec:3.1.3.41-nitrophenyl phosphatase;ec:3.1.3.12-trehalose 6-phosphatase;ec:3.4.24	Aminobenzoate degradationStarch and sucrose metabolism
Phvul.001G078500.1	sulfite exporter family	integral component of membrane		
Phvul.001G080200.1	Testis-expressed sequence 2	integral component of membrane		
Phvul.001G081400.1	early nodulin 3	integral component of membrane;anchored component of plasma membrane;electron carrier activity		
Phvul.001G082200.1	RING FYVE PHD zinc finger ,	zinc ion binding		
Phvul.001G082300.1	Ubiquitin-specific protease 9 isoform 1	thiol-dependent ubiquitin-specific protease activity;zinc ion binding;ubiquitin-dependent protein catabolic process;protein deubiquitination	ec:3.4;ec:3.4.19.12	
Phvul.001G083800.1	Brain 44	mitochondrial inner membrane;integral component of membrane;mitochondrial pyruvate transport		
Phvul.001G083800.2	Brain 44	mitochondrial inner membrane;integral component of membrane;mitochondrial pyruvate transport		
Phvul.001G084700.1	alpha-mannosidase-like isoform X1	vacuolar membrane;plant-type cell wall;apoplast;alpha-mannosidase activity;zinc ion binding;carbohydrate binding;mannose metabolic process;protein deglycosylation;microtubule nucleation;cysteine biosynthetic process	ec:3.2.1;ec:3.2.1.24-alpha-D-mannosidase	Other glycan degradation
Phvul.001G085900.1	transmembrane 45B	integral component of membrane		
Phvul.001G087700.1	acyltransferase At3g26840, chloroplastic	plastoglobule;integral component of membrane;diacylglycerol O-acyltransferase activity;phytol metabolic process	ec:2.3.1.20-O-acyltransferase;ec:2.3.1	Cutin, suberine and wax biosynthesis;Glycerolipid metabolism
Phvul.001G090700.1	probable S-acyltransferase 7	integral component of membrane;zinc ion binding;oxidoreductase activity;protein-cysteine S-palmitoyltransferase activity;oxidation-reduction process	ec:2.3.1.225;ec:2.3.1	
Phvul.001G093500.1	yrdC domain-containing , mitochondrial	double-stranded RNA binding		
Phvul.001G095300.1	hypothetical protein LR48_Vigan03g181500			
Phvul.001G096900.1	apyrase 2-like	integral component of membrane;hydrolase activity;metabolic process		
Phvul.001G097700.1	Eukaryotic peptide chain release factor subunit 1-3	cytoplasm;translation release factor activity, codon specific;translational termination		
Phvul.001G099000.1	DNA cross-link repair	nuclear chromosome, telomeric region;damaged DNA binding;benzoate 4-monooxygenase activity;5'-3' exodeoxyribonuclease activity;double-strand break repair via nonhomologous end joining;protection from non-homologous end joining at telomere;interstrand cross-link repair;oxidation-reduction process;nucleic acid phosphodiester bond hydrolysis	ec:3.1.15;ec:3.1.11;ec:3.1;ec:1.14.13;ec:1.14.13.12-4-monooxygenase	Aminobenzoate degradation;Benzoate degradation

Phvul.001G101800.1	transmembrane ,	integral component of membrane		
Phvul.001G102200.1	COP9 signalosome complex subunit 2	proteasome complex;cytosol;photomorphogenesis;cullin deneddylation;protein catabolic process		
Phvul.001G104000.1	probable 2-isopropylmalate synthase	2-isopropylmalate synthase activity;leucine biosynthetic process;nitrogen fixation;nodulation	ec:2.3.3.13-synthase	Valine, leucine and isoleucine biosynthesis;Pyruvate metabolism
Phvul.001G105100.1	polyribonucleotide nucleotidyltransferase	chloroplast stroma;3'-5'-exoribonuclease activity;RNA binding;polyribonucleotide nucleotidyltransferase activity;mRNA catabolic process;negative regulation of isopentenyl diphosphate biosynthetic process, methylerythritol 4-phosphate pathway;chlorophyll biosynthetic process;cellular response to phosphate starvation;carotene biosynthetic process;xanthophyll biosynthetic process;chloroplast RNA processing;RNA phosphodiester bond hydrolysis, exonucleolytic	ec:3.1.15;ec:3.1.13;ec:3.1;ec:2.7.7.8-nucleotidyltransferase	Purine metabolism;Pyrimidine metabolism
Phvul.001G105500.1	cation calcium exchanger 2-like	integral component of membrane;transmembrane transport		
Phvul.001G108000.1	co-chaperone family	mitochondrial matrix;chloroplast stroma;thylakoid;adenyl-nucleotide exchange factor activity;copper ion binding;protein homodimerization activity;chaperone binding;protein folding;regulation of catalytic activity		
Phvul.001G109100.1	PHYTOCHROME KINASE SUBSTRATE 4-like	kinase activity;phosphorylation		
Phvul.001G109600.1	HNH endonuclease domain	nucleic acid binding;endonuclease activity;response to desiccation;response to cold;response to salt stress;response to abscisic acid;nucleic acid phosphodiester bond hydrolysis	ec:3.1	
Phvul.001G110000.1	ARM repeat			
Phvul.001G110000.2	ARM repeat			
Phvul.001G111900.1	importin-5-like	cytoplasm;nuclear membrane;nuclear periphery;nuclear localization sequence binding;Ran GTPase binding;protein transporter activity;protein import into nucleus, docking;protein import into nucleus, translocation;NLS-bearing protein import into nucleus;ribosomal protein import into nucleus		
Phvul.001G113200.1	Inhibitor of Bruton tyrosine kinase	kinase activity;phosphorylation		
Phvul.001G113200.2	Inhibitor of Bruton tyrosine kinase	kinase activity;phosphorylation		
Phvul.001G113700.1	Nucleolar MIF4G domain-containing 1	nucleolus;integral component of membrane;RNA binding;mitotic recombination;embryo sac egg cell differentiation;ribosomal small subunit biogenesis		
Phvul.001G114700.3	DNA replication complex GINS PSF2	GINS complex;replication fork protection complex;3'-5' DNA helicase activity;double-strand break repair via break-induced replication;DNA duplex unwinding;mitotic DNA replication initiation	ec:3.6.1;ec:3.6.1.15-phosphatase	Purine metabolism;Thiamine metabolism
Phvul.001G114900.1	diphthine--ammonia ligase			
Phvul.001G117600.1	cytochrome f (chloroplast)	chloroplast thylakoid membrane;integral component of thylakoid membrane;iron ion binding;electron carrier activity;heme binding;photosynthesis;oxidation-reduction process		
Phvul.001G120700.1	serine carboxypeptidase-like 45	serine-type carboxypeptidase activity;transferase activity, transferring acyl groups other than amino-acyl groups;secondary metabolic process;proteolysis involved in cellular protein catabolic process	ec:3.4.21;ec:3.4.16	

Phvul.001G122400.1	exocyst complex component EXO70B1-like	exocyst;integral component of membrane;exocytosis		
Phvul.001G123200.1	microtubule-associated 70-2-like	integral component of membrane;microtubule binding;cytoskeleton organization		
Phvul.001G123900.1	ferredoxin-dependent glutamate synthase, chloroplastic	glutamate synthase (ferredoxin) activity;glutamate biosynthetic process;ammonia assimilation cycle;oxidation-reduction process	ec:1.4;ec:1.4.7.1-synthase (ferredoxin)	Nitrogen metabolism;Glyoxylate and dicarboxylate metabolism
Phvul.001G126800.1	triacylglycerol lipase SDP1	phospholipase A2 activity;sterol esterase activity;triglyceride lipase activity;lipid metabolic process	ec:3.1.1.13-esterase;ec:3.1.1;ec:3.1.1.4-A2;ec:3.1.1.3-lipase;ec:3.1.1.1-ali-esterase	Steroid biosynthesisalpha-Linolenic acid metabolism;Linoleic acid metabolism;Arachidonic acid metabolism;Ether lipid metabolism;Glycerophospholipid metabolismGlycerolipid metabolismDrug metabolism - other enzymes
Phvul.001G128200.1	disease resistance (TIR-NBS-LRR class),	nucleic acid binding;RNA-DNA hybrid ribonuclease activity;ADP binding;signal transduction;RNA phosphodiester bond hydrolysis, endonucleolytic	ec:3.1.30;ec:3.1.26;ec:3.1;ec:3.1.26.4	
Phvul.001G129200.1	DNA helicase INO80	DNA binding;helicase activity;ATP binding;metabolic process	ec:3.6.1;ec:3.6.1.15-phosphatase	Purine metabolism;Thiamine metabolism
Phvul.001G129300.1	glycerol-3-phosphate dehydrogenase [NAD(+)] GPDHC1, cytosolic	glycerol-3-phosphate dehydrogenase complex;glycerol-3-phosphate dehydrogenase [NAD+] activity;NAD binding;carbohydrate metabolic process;glycerol-3-phosphate catabolic process;oxidation-reduction process	ec:1.1.1.8-dehydrogenase (NAD+);ec:1.1.1.94-dehydrogenase [NAD(P)+]	Glycerophospholipid metabolismGlycerophospholipid metabolism
Phvul.001G129400.1	hypothetical protein PHAVU_001G129400g	DNA-templated transcription, termination		
Phvul.001G129400.2	hypothetical protein PHAVU_001G129400g	DNA-templated transcription, termination		
Phvul.001G129800.2	tRNA-splicing endonuclease subunit Sen54	tRNA-intron endonuclease complex;tRNA-intron endonuclease activity;tRNA-type intron splice site recognition and cleavage;RNA phosphodiester bond hydrolysis, endonucleolytic	ec:3.1.31;ec:3.1.27;ec:3.1.27.9;ec:3.1	
Phvul.001G130300.1	kinesin KP1	kinesin complex;microtubule;microtubule motor activity;ATP binding;microtubule binding;chromatin organization;microtubule-based movement;metabolic process;single-organism organelle organization	ec:3.6.1;ec:3.6.1.15-phosphatase	Purine metabolism;Thiamine metabolism
Phvul.001G131100.1	ABC transporter family	vacuolar membrane;integral component of membrane;ATP binding;polyamine-transporting ATPase activity;heme-transporting ATPase activity;protein N-linked glycosylation;lipid transport;heme transport;polyamine transmembrane transport	ec:3.6.1;ec:3.6.1.3-adenylpyrophosphatase;ec:3.6.1.15-phosphatase;ec:3.6.3.41;ec:3.6.3.31	Purine metabolismPurine metabolism;Thiamine metabolism
Phvul.001G131300.1	AP2-like ethylene-responsive transcription factor At2g41710 isoform X1	nucleus;DNA binding;transcription factor activity, sequence-specific DNA binding;transcription, DNA-templated;regulation of transcription, DNA-templated;multicellular organismal development		
Phvul.001G131700.1	villin-3-like isoform X1	actin filament binding;actin filament bundle assembly		
Phvul.001G131700.2	villin-3-like isoform X1	actin filament binding;actin filament bundle assembly		
Phvul.001G133600.1	disease resistance RGA1	ADP binding;defense response		
Phvul.001G134300.1	disease resistance RGA1	phosphoprotein phosphatase activity;ADP binding;protein dephosphorylation;defense response	ec:3.1;ec:3.1.3.41-nitrophenyl phosphatase;ec:3.1.3.16-phosphatase	Aminobenzoate degradationT cell receptor signaling pathway

Phvul.001G135900.1	armadillo beta-catenin-like repeat			
Phvul.001G136300.1	oligopeptide transporter OPT family	integral component of membrane;transmembrane transport		
Phvul.001G136300.2	oligopeptide transporter OPT family	integral component of membrane;transmembrane transport		
Phvul.001G137300.1	probably inactive leucine-rich repeat receptor kinase At5g06940	integral component of membrane;protein kinase activity;ATP binding;2-alkenal reductase [NAD(P)] activity;protein phosphorylation;oxidation-reduction process	ec:1.3.1.74	
Phvul.001G137800.2	ribosomal RNA processing brix domain	nucleolus;RNA binding;ribosomal large subunit assembly		
Phvul.001G137800.4	ribosomal RNA processing brix domain	nucleolus;RNA binding;ribosomal large subunit assembly		
Phvul.001G138100.1	tRNA-splicing endonuclease subunit Sen2-1-like	tRNA-intron endonuclease complex;tRNA-intron endonuclease activity;nucleic acid binding;lyase activity;tRNA-type intron splice site recognition and cleavage;RNA phosphodiester bond hydrolysis, endonucleolytic	ec:3.1.31;ec:3.1.27;ec:3.1.27.9;ec:3.1	
Phvul.001G140300.1	TBCC domain-containing 1-like	integral component of membrane;cell morphogenesis		
Phvul.001G142700.1	nudix hydrolase 23, chloroplastic	hydrolase activity;flavin-containing compound metabolic process		
Phvul.001G143000.1	WRC	nucleus;transcription, DNA-templated;developmental process		
Phvul.001G143000.2	WRC	nucleus;transcription, DNA-templated;developmental process		
Phvul.001G144100.1	E3 ubiquitin- ligase SINAT2	nucleus;cytoplasm;zinc ion binding;ligase activity;ubiquitin protein ligase activity;apoptotic process;multicellular organismal development;protein ubiquitination involved in ubiquitin-dependent protein catabolic process;proteasome-mediated ubiquitin-dependent protein catabolic process		
Phvul.001G144400.1	DNA-directed RNA polymerase subunit beta	chloroplast		
Phvul.001G144700.1	colon cancer-associated Mic1			
Phvul.001G146800.1	receptor-like cytosolic serine threonine- kinase RBK2	protein serine/threonine kinase activity;non-membrane spanning protein tyrosine kinase activity;ATP binding;peptidyl-tyrosine phosphorylation	ec:2.7.11;ec:2.7.10;ec:2.7.10.2-protein-tyrosine kinase	T cell receptor signaling pathway
Phvul.001G146900.1	probable LRR receptor-like serine threonine- kinase RKF3	plasma membrane;integral component of membrane;protein serine/threonine kinase activity;transmembrane receptor protein tyrosine kinase activity;ATP binding;peptidyl-tyrosine phosphorylation	ec:2.7.11;ec:2.7.10;ec:2.7.10.1	
Phvul.001G150700.1	Rho GTPase	integral component of mitochondrial outer membrane;GTPase activity;calcium ion binding;GTP binding;mitochondrion organization;small GTPase mediated signal transduction;metabolic process;protein transport	ec:3.6.1;ec:3.6.1.15-phosphatase	Purine metabolism;Thiamine metabolism
Phvul.001G151200.1	Ribosome biogenesis GTPase A	cell;GTPase activity;GTP binding;maltose metabolic process;phosphatidylglycerol biosynthetic process;chloroplast relocation;thylakoid membrane organization;starch biosynthetic process;isopentenyl diphosphate biosynthetic process, methylerythritol 4-phosphate pathway;ncRNA metabolic process;ribosome biogenesis	ec:3.6.1;ec:3.6.1.15-phosphatase	Purine metabolism;Thiamine metabolism
Phvul.001G151300.1	ARM REPEAT PROTEIN INTERACTING WITH ABF2-like			
Phvul.001G152000.1	fatty-acid-binding 1	mitochondrion;chloroplast envelope;fatty acid binding;intramolecular lyase activity;fatty acid metabolic process		

Phvul.001G152600.1	plant T24G3-80			
Phvul.001G154100.1	NETWORKED 1D-like			
Phvul.001G154300.1	serine threonine-kinase Nek4-like	integral component of membrane;protein serine/threonine kinase activity;ATP binding;protein phosphorylation	ec:2.7.11	
Phvul.001G154300.2	serine threonine-kinase Nek4-like	integral component of membrane;protein serine/threonine kinase activity;ATP binding;protein phosphorylation	ec:2.7.11	
Phvul.001G154300.3	serine threonine-kinase Nek4-like	integral component of membrane;protein serine/threonine kinase activity;ATP binding;protein phosphorylation	ec:2.7.11	
Phvul.001G155100.1	hypothetical protein PHAVU_001G155100g			
Phvul.001G155300.3	hypothetical protein PHAVU_001G155300g	plastid		
Phvul.001G160900.1	DUF4283 domain	nucleic acid binding;zinc ion binding		
Phvul.001G162600.1	glutathione gamma-glutamylcysteinyltransferase 1	glutathione gamma-glutamylcysteinyltransferase activity;metal ion binding;response to metal ion;phytochelatin biosynthetic process	ec:2.3.2.15	
Phvul.001G162700.1	glutathione gamma-glutamylcysteinyltransferase 3-like	glutathione gamma-glutamylcysteinyltransferase activity;metal ion binding;response to metal ion;phytochelatin biosynthetic process	ec:2.3.2.15	
Phvul.001G162800.1	long-chain-alcohol oxidase FAO2-like	integral component of membrane;long-chain-alcohol oxidase activity;flavin adenine dinucleotide binding;oxidation-reduction process	ec:1.1.3.20	
Phvul.001G163300.1	BREAST CANCER SUSCEPTIBILITY 1 homolog	plasma membrane;integral component of membrane;BRCA1-BARD1 complex;BRCA1-A complex;ubiquitin-protein transferase activity;zinc ion binding;ligase activity;double-strand break repair via homologous recombination;DNA replication;protein ubiquitination;chromatin modification;positive regulation of histone acetylation;negative regulation of histone acetylation;regulation of cell proliferation;macromolecule methylation;negative regulation of fatty acid biosynthetic process;positive regulation of transcription from RNA polymerase II promoter;leaf development;positive regulation of cell cycle arrest		
Phvul.001G163300.2	BREAST CANCER SUSCEPTIBILITY 1 like	plasma membrane;integral component of membrane;BRCA1-BARD1 complex;BRCA1-A complex;ubiquitin-protein transferase activity;zinc ion binding;ligase activity;double-strand break repair via homologous recombination;DNA replication;protein ubiquitination;chromatin modification;positive regulation of histone acetylation;negative regulation of histone acetylation;regulation of cell proliferation;macromolecule methylation;negative regulation of fatty acid biosynthetic process;positive regulation of transcription from RNA polymerase II promoter;leaf development;positive regulation of cell cycle arrest		
Phvul.001G163800.1	probable LRR receptor-like serine threonine- kinase At4g08850	integral component of membrane;protein kinase activity;ATP binding;protein phosphorylation		
Phvul.001G165300.1	Subtilisin-like protease SDD1	cell wall;external side of plasma membrane;integral component of membrane;serine-type endopeptidase activity;2-alkenal reductase [NAD(P)] activity;proteolysis;stomatal complex morphogenesis;regulation of cell proliferation;oxidation-reduction process	ec:1.3.1.74;ec:3.4.21	

Phvul.001G165500.1	ABC transporter C family member 5-like	plant-type vacuole;vacuolar membrane;integral component of membrane;ATP binding;sulfonylurea receptor activity;ATPase activity, coupled to transmembrane movement of substances;microtubule cytoskeleton organization;cytokinesis by cell plate formation;ER to Golgi vesicle-mediated transport;signal transduction;metabolic process;response to salt stress;cellular potassium ion homeostasis;amino acid import;transmembrane transport	ec:3.6.1;ec:3.6.1.3-adenylpyrophosphatase;ec:3.6.1.15-phosphatase	Purine metabolismPurine metabolism;Thiamine metabolism
Phvul.001G165700.1	drug resistance transporter-like ABC domain	plasma membrane;integral component of membrane;ATP binding;ATPase activity, coupled to transmembrane movement of substances;metabolic process;transmembrane transport	ec:3.6.1;ec:3.6.1.3-adenylpyrophosphatase;ec:3.6.1.15-phosphatase	Purine metabolismPurine metabolism;Thiamine metabolism
Phvul.001G168700.1	transcription factor	protein dimerization activity		
Phvul.001G168700.2	transcription factor	protein dimerization activity		
Phvul.001G169200.1	Histone H4	nucleosome;nucleolus;vacuolar membrane;Golgi apparatus;cytosol;plasma membrane;plasmodesma;chloroplast;thylakoid;DNA binding;protein heterodimerization activity;nucleosome assembly;response to water deprivation		
Phvul.001G169300.1	cation H(+) antiporter 20	endoplasmic reticulum;integral component of membrane;monovalent cation:proton antiporter activity;protein targeting to vacuole;regulation of pH;response to stress;cellular potassium ion homeostasis;water homeostasis;hydrogen ion transmembrane transport		
Phvul.001G170700.1	ACCELERATED CELL DEATH 6-like	integral component of membrane;regulation of defense response;cellular response to salicylic acid stimulus;regulation of salicylic acid mediated signaling pathway		
Phvul.001G170700.2	ACCELERATED CELL DEATH 6-like	integral component of membrane;regulation of defense response;cellular response to salicylic acid stimulus;regulation of salicylic acid mediated signaling pathway		
Phvul.001G171500.1	exocyst complex component EXO70A1-like	exocyst;exocytosis		
Phvul.001G172600.1	serine arginine-rich splicing factor RS2Z32-like isoform X1	nucleotide binding;nucleic acid binding;zinc ion binding		
Phvul.001G172600.2	serine arginine-rich splicing factor RS2Z32 isoform X1	nucleotide binding;nucleic acid binding;zinc ion binding		
Phvul.001G173000.1	dirigent 17-like			
Phvul.001G174800.1	flocculation FLO11-like isoform X1			
Phvul.001G182700.1	UDP-glycosyltransferase 73C6-like	intracellular membrane-bounded organelle;quercetin 3-O-glucosyltransferase activity;quercetin 7-O-glucosyltransferase activity;flavonoid biosynthetic process;flavonoid glucuronidation	ec:2.4.1	
Phvul.001G184000.1	cactus-binding carboxy-terminal, cactin			
Phvul.001G185600.1	plant T32M21-140	membrane		
Phvul.001G185600.2	plant T32M21-140	membrane		
Phvul.001G186800.1	PAN domain-containing At5g03700	Golgi apparatus;integral component of membrane;recognition of pollen		
Phvul.001G187200.1	UPF0481 At3g47200-like	integral component of membrane;response to brassinosteroid		
Phvul.001G188100.1	serine threonine-kinase D6PKL1-like	cytoplasm;plasma membrane;protein serine/threonine kinase activity;ATP	ec:2.7.11	

		binding;protein phosphorylation;intracellular signal transduction		
Phvul.001G188100.2	serine threonine-kinase D6PKL1-like	cytoplasm;plasma membrane;protein serine/threonine kinase activity;ATP binding;protein phosphorylation;intracellular signal transduction	ec:2.7.11	
Phvul.001G188100.3	serine threonine-kinase D6PKL1-like	cytoplasm;plasma membrane;protein serine/threonine kinase activity;ATP binding;protein phosphorylation;intracellular signal transduction	ec:2.7.11	
Phvul.001G189900.1	plant F3C22-140 ,			
Phvul.001G190400.1	heme-binding At3g10130, chloroplastic	chloroplast thylakoid membrane		
Phvul.001G190400.2	heme-binding At3g10130, chloroplastic	chloroplast thylakoid membrane		
Phvul.001G193000.1	embryo defective 2735			
Phvul.001G193000.2	embryo defective 2735			
Phvul.001G193000.3	embryo defective 2735			
Phvul.001G193000.4	embryo defective 2735			
Phvul.001G193100.1	B3 domain-containing At2g36080-like	nucleus;DNA binding;transcription, DNA-templated;regulation of transcription, DNA-templated		
Phvul.001G194400.1	cytokinin riboside 5 -monophosphate phosphoribohydrolase LOG7	nucleus;cytosol;hydrolase activity, hydrolyzing N-glycosyl compounds;cytokinin biosynthetic process		
Phvul.001G194700.1	syringolide-induced B13-1-9	plasmodesma;integral component of membrane;anchored component of plasma membrane;signal transducer activity;defense response;signal transduction		
Phvul.001G196100.1	dof zinc finger -like	DNA binding;regulation of transcription, DNA-templated		
Phvul.001G196200.1	trafficking particle complex subunit 8	pre-autophagosomal structure;cytoplasmic vesicle;TRAPPIII protein complex;autophagosome assembly;ER to Golgi vesicle-mediated transport;pexophagy;CVT pathway;protein localization to pre-autophagosomal structure;nucleophagy		
Phvul.001G197900.1	fasciclin-like arabinogalactan 16			
Phvul.001G198500.1	downstream neighbor of Son			
Phvul.001G198700.1	F-box At5g06550	nucleus;core promoter proximal region sequence-specific DNA binding;transferase activity;histone demethylase activity (H3-K36 specific);regulation of transcription, DNA-templated;protein targeting to mitochondrion;regulation of flower development;positive regulation of seed germination;histone H4-R3 methylation;oxidation-reduction process;histone H3-K36 demethylation	ec:1.14.11.27;ec:1.14.11	
Phvul.001G198800.1	myosin-binding 7-like	integral component of membrane		
Phvul.001G204000.1	Spermatogenesis-associated 20	chloroplast;thymidylate kinase activity;nucleotide phosphorylation	ec:2.7.4.9-kinase	Pyrimidine metabolism
Phvul.001G204500.1	CW-type zinc-finger	zinc ion binding		
Phvul.001G208500.1	histone-lysine N-methyltransferase ATX3	nucleus;zinc ion binding;histone-lysine N-methyltransferase activity;histone lysine methylation	ec:2.1.1;ec:2.1.1.43-N-methyltransferase	Lysine degradation
Phvul.001G209200.1	pectinesterase pectinesterase inhibitor	cell wall;pectinesterase activity;aspartyl esterase activity;pectinesterase inhibitor activity;cell wall modification;negative regulation of catalytic activity;pectin catabolic process	ec:3.1.1.11-pectin demethoxylase;ec:3.1.1.1-ali-esterase	Starch and sucrose metabolism;Pentose and glucuronate interconversionsDrug metabolism - other enzymes

Phvul.001G209800.3	choline ethanolaminephosphotransferase 1	integral component of membrane;phosphotransferase activity, for other substituted phosphate groups;phospholipid biosynthetic process		
Phvul.001G210400.1	Heat shock binding			
Phvul.001G210500.1	P-loop nucleoside triphosphate hydrolase superfamily	kinesin complex;microtubule;microtubule motor activity;ATP binding;microtubule binding;ATPase activity;microtubule-based movement;metabolic process	ec:3.6.1;ec:3.6.1.3-adenylpyrophosphatase;ec:3.6.1.15-phosphatase	Purine metabolismPurine metabolism;Thiamine metabolism
Phvul.001G211000.1	cellulose synthase D5	integral component of membrane;cellulose synthase (UDP-forming) activity;1,4-beta-D-xylan synthase activity;mannan synthase activity;microtubule cytoskeleton organization;double-strand break repair via homologous recombination;cytokinesis by cell plate formation;response to water deprivation;response to salt stress;leaf morphogenesis;response to cyclopentenone;cellulose biosynthetic process;regulation of cell proliferation;cell wall biogenesis;cell wall organization;mannosylation	ec:2.4.1.12-synthase (UDP-forming);ec:2.4.2.24-synthase;ec:2.4.1	Starch and sucrose metabolismAmino sugar and nucleotide sugar metabolism;Starch and sucrose metabolism
Phvul.001G212600.1	DNA replication licensing factor MCM7	nucleus;cytoplasm;MCM complex;DNA binding;DNA helicase activity;ATP binding;DNA unwinding involved in DNA replication;DNA replication initiation;DNA methylation;chromatin silencing;cell proliferation;sugar mediated signaling pathway;histone H3-K9 methylation	ec:3.6.1;ec:3.6.1.15-phosphatase	Purine metabolism;Thiamine metabolism
Phvul.001G213200.1	trichome birefringence-like 6	Golgi apparatus;integral component of membrane;O-acetyltransferase activity;cell wall organization or biogenesis	ec:2.3.1	
Phvul.001G213800.1	HIT zinc finger,PAPA-1-like conserved region isoform 1	Ino80 complex;chromatin remodeling;regulation of transcription, DNA-templated		
Phvul.001G213800.2	HIT zinc finger,PAPA-1-like conserved region isoform 1	Ino80 complex;chromatin remodeling;regulation of transcription, DNA-templated		
Phvul.001G214100.1	DEAD-box ATP-dependent RNA helicase 24	nucleic acid binding;ATP-dependent RNA helicase activity;ATP binding;RNA secondary structure unwinding	ec:3.6.1;ec:3.6.1.3-adenylpyrophosphatase;ec:3.6.1.15-phosphatase;ec:2.7.7	Purine metabolismPurine metabolism;Thiamine metabolism
Phvul.001G216500.1	WD repeat-containing 6	Cul4-RING E3 ubiquitin ligase complex		
Phvul.001G217700.1	pentatricopeptide repeat-containing At4g02750-like	microtubule-severing ATPase activity;metabolic process	ec:3.6.1;ec:3.6.1.3-adenylpyrophosphatase;ec:3.6.4.3;ec:3.6.1.15-phosphatase	Purine metabolismPurine metabolism;Thiamine metabolism
Phvul.001G218500.1	probable WRKY transcription factor 47 isoform X1	transcription factor activity, sequence-specific DNA binding;sequence-specific DNA binding;regulation of transcription, DNA-templated		
Phvul.001G218800.1	transcription factor PIF3-like	protein dimerization activity		
Phvul.001G219300.1	Haloacid dehalogenase-like hydrolase superfamily	hydrolase activity;metabolic process;response to nitrate;nitrate transport		
Phvul.001G219500.1	phosphatase 2C 32	intracellular;plasma membrane;protein serine/threonine phosphatase activity;[pyruvate dehydrogenase (lipoamide)] phosphatase activity;phospholipid binding;microtubule cytoskeleton organization;cytokinesis by cell plate formation;mRNA export from nucleus;protein dephosphorylation;cell adhesion;regulation of flower development;regulation of meristem structural organization;maintenance of meristem identity;trichome morphogenesis;post-translational protein modification;actin nucleation;positive regulation of transcription, DNA-templated;root hair cell differentiation;cell wall organization	ec:3.1;ec:3.1.3.43;ec:3.1.3.41-nitrophenylphosphatase;ec:3.1.3.16-phosphatase	Aminobenzoate degradationT cell receptor signaling pathway

Phvul.001G223300.1	glucose-6-phosphate 1-dehydrogenase 4, chloroplastic	glucose-6-phosphate dehydrogenase activity;NADP binding;glucose metabolic process;pentose-phosphate shunt;oxidation-reduction process	ec:1.1.1.49-dehydrogenase (NADP+)	Biosynthesis of antibiotics;Pentose phosphate pathway;Glutathione metabolism
Phvul.001G223800.1	chloroplast stem-loop binding of 41 kDa b, chloroplastic	vacuole;peroxisome;ribosome;plasmodesma;chloroplast stroma;chloroplast envelope;stromule;membrane;apoplast;catalytic activity;heteropolysaccharide binding;coenzyme binding;MAPK cascade;monosaccharide metabolic process;pentose-phosphate shunt;protein targeting to membrane;unsaturated fatty acid biosynthetic process;detection of biotic stimulus;response to blue light;salicylic acid biosynthetic process;photorespiration;systemic acquired resistance, salicylic acid mediated signaling pathway;jasmonic acid mediated signaling pathway;chloroplast relocation;leaf morphogenesis;thylakoid membrane organization;response to red light;response to chitin;photosystem II assembly;response to far red light;regulation of hydrogen peroxide metabolic process;regulation of plant-type hypersensitive response;chlorophyll biosynthetic process;carotenoid biosynthetic process;isopentenyl diphosphate biosynthetic process, methylerythritol 4-phosphate pathway;cell differentiation;negative regulation of defense response;plastid translation;ncRNA metabolic process;regulation of protein dephosphorylation;transcription from plastid promoter;positive regulation of catalytic activity;regulation of multi-organism process;positive regulation of translation;positive regulation of transcription, DNA-templated;defense response to fungus		
Phvul.001G224800.1	Rab9 effector with kelch motifs			
Phvul.001G224800.2	Rab9 effector with kelch motifs			
Phvul.001G224900.1	Homeobox knotted-1-like 1	nucleus;sequence-specific DNA binding;regulation of transcription, DNA-templated		
Phvul.001G225100.1	hypothetical protein PHAVU_001G225100g			
Phvul.001G226000.1	calmodulin-binding 60 G-like	calmodulin binding;single-organism cellular process;innate immune response;regulation of cellular process;defense response to other organism		
Phvul.001G226200.1	Calmodulin-binding, isoform 2, partial	calmodulin binding;signal transduction;defense response, incompatible interaction;regulation of cellular metabolic process;cellular metabolic process;regulation of innate immune response;cellular response to acid chemical;cellular response to organic substance;cellular response to oxygen-containing compound		
Phvul.001G227600.1	Serine threonine kinases,ATP binding,catalytics isoform 1	protein serine/threonine kinase activity;ATP binding;protein phosphorylation	ec:2.7.11	
Phvul.001G229700.1	nudix hydrolase 18, mitochondrial-like	hydrolase activity;metabolic process		
Phvul.001G232200.1	pyridoxal kinase	cytosol;pyridoxal kinase activity;pyridoxine biosynthetic process;pyridoxal 5'-phosphate salvage;trichoblast differentiation;phosphorylation;hyperosmotic salinity response	ec:2.7.1.35-kinase	Vitamin B6 metabolism
Phvul.001G233800.1	anaphase promoting complex subunit 2	anaphase-promoting complex;spindle;ubiquitin protein ligase binding;ubiquitin protein ligase activity;cell-cell signaling;gamete generation;megagametogenesis;virus induced gene silencing;production of ta-siRNAs involved		

		in RNA interference;regulation of DNA endoreduplication;production of miRNAs involved in gene silencing by miRNA;protein ubiquitination involved in ubiquitin-dependent protein catabolic process;regulation of cell division;regulation of unidimensional cell growth		
Phvul.001G233800.2	anaphase promoting complex subunit 2	anaphase-promoting complex;spindle;ubiquitin protein ligase binding;ubiquitin protein ligase activity;cell-cell signaling;gamete generation;megagametogenesis;virus induced gene silencing;production of ta-siRNAs involved in RNA interference;regulation of DNA endoreduplication;production of miRNAs involved in gene silencing by miRNA;protein ubiquitination involved in ubiquitin-dependent protein catabolic process;regulation of cell division;regulation of unidimensional cell growth		
Phvul.001G236700.1	stellacyanin-like	anchored component of plasma membrane;electron carrier activity		
Phvul.001G237000.2	integral membrane Yip1 family	integral component of membrane		
Phvul.001G238900.1	UDP-glycosyltransferase 76F1-like	intracellular membrane-bounded organelle;quercetin 3-O-glucosyltransferase activity;quercetin 7-O-glucosyltransferase activity;flavonoid biosynthetic process;flavonoid glucuronidation	ec:2.4.1	
Phvul.001G239800.3	Trafficking particle complex subunit 5	intracellular;ligase activity;cellulose biosynthetic process;Golgi vesicle transport		
Phvul.001G240000.1	Myb SANT-like DNA-binding domain			
Phvul.001G240900.1	26S proteasome non-ATPase regulatory subunit 7 homolog A	proteasome complex;integral component of membrane;proteasome-mediated ubiquitin-dependent protein catabolic process		
Phvul.001G241300.1	nematode resistance -like HSPRO2	defense response		
Phvul.001G241900.1	Arginyl-tRNA--transferase 1	cytoplasm;arginyltransferase activity;arginine-tRNA ligase activity;ATP binding;arginyl-tRNA aminoacylation;protein arginylation	ec:6.1.1;ec:2.3.2.8;ec:6.1.1.19-ligase	Aminoacyl-tRNA biosynthesis
Phvul.001G242800.1	DNA replication complex GINS PSF3-like	nucleus;DNA replication		
Phvul.001G243100.1	phosphoinositide-specific phospholipase C family	intracellular;phosphatidylinositol phospholipase C activity;signal transducer activity;lipid catabolic process;intracellular signal transduction	ec:3.1.1;ec:3.1;ec:3.1.4.11-phospholipase C	Inositol phosphate metabolism;Phosphatidylinositol signaling system
Phvul.001G243800.1	serine threonine-kinase CCR3	intracellular;integral component of membrane;protein serine/threonine kinase activity;ATP binding;protein homodimerization activity;protein phosphorylation;protein targeting to membrane;response to ethylene;abscisic acid-activated signaling pathway;regulation of plant-type hypersensitive response;intracellular signal transduction;negative regulation of programmed cell death	ec:2.7.11	
Phvul.001G244100.1	N-(5-phosphoribosyl)anthranilate isomerase	phosphoribosylanthranilate isomerase activity;tryptophan biosynthetic process	ec:5.3.1.24-isomerase	Biosynthesis of antibiotics;Phenylalanine, tyrosine and tryptophan biosynthesis
Phvul.001G244100.2	N-(5-phosphoribosyl)anthranilate isomerase	phosphoribosylanthranilate isomerase activity;tryptophan biosynthetic process	ec:5.3.1.24-isomerase	Biosynthesis of antibiotics;Phenylalanine, tyrosine and tryptophan biosynthesis
Phvul.001G244900.1	UDP-glucose pyrophosphorylase	cytosol;UDP-N-acetylglucosamine diphosphorylase activity;UDP-N-acetylglucosamine biosynthetic process;cellular response to phosphate starvation;galactolipid biosynthetic process	ec:2.7.7.23-diphosphorylase	Biosynthesis of antibiotics;Amino sugar and nucleotide sugar metabolism

Phvul.001G245100.1	probable inactive serine threonine-kinase bub1	kinase activity;mitotic spindle assembly checkpoint;phosphorylation		
Phvul.001G246000.1	DEAD-box ATP-dependent RNA helicase 18	RNA binding;ATP-dependent RNA helicase activity;ATP binding;RNA secondary structure unwinding	ec:3.6.1;ec:3.6.1.3-adenylpyrophosphatase;ec:3.6.1.15-phosphatase;ec:2.7.7	Purine metabolismPurine metabolism;Thiamine metabolism
Phvul.001G247500.1	Sel1 repeat	metal ion binding;metal ion transport		
Phvul.001G247800.1	PLAC8 family	integral component of membrane		
Phvul.001G250100.1	cellulose synthase-interactive			
Phvul.001G250100.2	cellulose synthase-interactive			
Phvul.001G252900.1	chaperone -like	ATP binding;heat shock protein binding;metal ion binding;unfolded protein binding;protein folding;response to heat		
Phvul.001G254100.1	helicase, IBR and zinc finger	integral component of membrane;ATP-dependent RNA helicase activity;ATP binding;zinc ion binding;poly(A) RNA binding;RNA processing	ec:3.6.1;ec:3.6.1.3-adenylpyrophosphatase;ec:3.6.1.15-phosphatase;ec:2.7.7	Purine metabolismPurine metabolism;Thiamine metabolism
Phvul.001G254200.1	transmembrane ,	integral component of membrane		
Phvul.001G254400.1	probable glucan 1,3-beta-glucosidase A	integral component of membrane;glucan exo-1,3-beta-glucosidase activity;protein binding, bridging;actin filament binding;carbohydrate metabolic process;actin filament organization	ec:3.2.1.58-1,3-beta-glucosidase;ec:3.2.1;ec:3.2.1.21-gentiobiase	Starch and sucrose metabolismStarch and sucrose metabolism;Cyanoamino acid metabolism;Phenylpropanoid biosynthesis
Phvul.001G256000.1	GLE1 isoform X1	nuclear pore;membrane;mitotic recombination;embryo sac egg cell differentiation;poly(A)+ mRNA export from nucleus;seed development		
Phvul.001G259600.1	flocculation FLO11-like			
Phvul.001G260000.1	urease	urease activity;nickel cation binding;urea catabolic process	ec:3.5.1.5-ec:3.5.1.5	urease
Phvul.001G260800.1	ribosome biogenesis GTPase	GTPase activity;GTP binding;metabolic process	ec:3.6.1;ec:3.6.1.15-phosphatase	Purine metabolism;Thiamine metabolism
Phvul.001G262100.1	alpha-ketoglutarate-dependent dioxygenase alkB	dioxygenase activity;DNA repair;oxidation-reduction process		
Phvul.001G263600.1	15-cis-zeta-carotene isomerase, chloroplastic	integral component of membrane;9,15,9'-tri-cis-zeta-carotene isomerase activity;carotene biosynthetic process		
Phvul.001G264300.1	DNA topoisomerase 3-beta isoform X1	DNA binding;DNA topoisomerase type I activity;DNA topological change;photoperiodism, flowering	ec:5.99.1.2;ec:5.99.1	
Phvul.001G264500.1	phragmoplast-associated kinesin-related	kinesin complex;microtubule;ATP binding;microtubule binding;ATP-dependent microtubule motor activity, plus-end-directed;microtubule-based movement;metabolic process	ec:3.6.1;ec:3.6.1.3-adenylpyrophosphatase;ec:3.6.4.4;ec:3.6.1.15-phosphatase	Purine metabolismPurine metabolism;Thiamine metabolism
Phvul.001G264600.1	C2H2 type zinc finger	mitochondrion;nucleic acid binding;transcription factor activity, sequence-specific DNA binding;metal ion binding;regulation of transcription, DNA-templated		
Phvul.002G002200.1	serine threonine-kinase SAPK3-like	cytoplasm;plasma membrane;protein serine/threonine kinase activity;ATP binding;protein phosphorylation;intracellular signal transduction	ec:2.7.11	
Phvul.002G002200.2	serine threonine-kinase SAPK3-like	cytoplasm;plasma membrane;protein serine/threonine kinase activity;ATP	ec:2.7.11	

		binding;protein phosphorylation;intracellular signal transduction		
Phvul.002G003300.1	regulator of nonsense transcripts 1 homolog	cytosol;plasma membrane;plasmodesma;integral component of membrane;DNA binding;helicase activity;ATP binding;zinc ion binding;nuclear-transcribed mRNA catabolic process, nonsense-mediated decay;response to wounding;response to sucrose;salicylic acid mediated signaling pathway;jasmonic acid mediated signaling pathway;sugar mediated signaling pathway;RNA interference;defense response to bacterium;long-day photoperiodism;cotyledon development	ec:3.6.1;ec:3.6.1.15-phosphatase	Purine metabolism;Thiamine metabolism
Phvul.002G003300.2	regulator of nonsense transcripts 1 homolog	cytosol;plasma membrane;plasmodesma;integral component of membrane;DNA binding;helicase activity;ATP binding;zinc ion binding;nuclear-transcribed mRNA catabolic process, nonsense-mediated decay;response to wounding;response to sucrose;salicylic acid mediated signaling pathway;jasmonic acid mediated signaling pathway;sugar mediated signaling pathway;RNA interference;defense response to bacterium;long-day photoperiodism;cotyledon development	ec:3.6.1;ec:3.6.1.15-phosphatase	Purine metabolism;Thiamine metabolism
Phvul.002G004400.1	pentatricopeptide repeat-containing At1g32415, mitochondrial-like	microtubule-severing ATPase activity;metabolic process	ec:3.6.1;ec:3.6.1.3-adenylpyrophosphatase;ec:3.6.4.3;ec:3.6.1.15-phosphatase	Purine metabolismPurine metabolism;Thiamine metabolism
Phvul.002G007900.1	Tubulin alpha-2 chain	cytoplasm;microtubule;GTPase activity;structural constituent of cytoskeleton;GTP binding;microtubule-based process;metabolic process	ec:3.6.1;ec:3.6.1.15-phosphatase	Purine metabolism;Thiamine metabolism
Phvul.002G009800.1	Histone-lysine N-methyltransferase NSD3,			
Phvul.002G009900.1	galactomannan galactosyltransferase 1-like	Golgi membrane;integral component of membrane;xyloglucan 6-xylosyltransferase activity;metabolic process;cell wall organization	ec:2.4.2.39	
Phvul.002G010100.1	transmembrane ,	integral component of membrane		
Phvul.002G010800.1	peptidyl-prolyl cis-trans isomerase CYP65	nucleus;peptidyl-prolyl cis-trans isomerase activity;ligase activity;ubiquitin-ubiquitin ligase activity;protein polyubiquitination;protein peptidyl-prolyl isomerization;protein folding	ec:5.2.1.8	
Phvul.002G011100.1	60S ribosomal L26-1	cytosolic large ribosomal subunit;structural constituent of ribosome;cytoplasmic translation;ribosomal large subunit biogenesis		
Phvul.002G011500.1	condensin-2 complex subunit G2,	nucleus;mitochondrion		
Phvul.002G012000.1	probable N-acetyltransferase HLS1	protein acetyltransferase complex;peptide alpha-N-acetyltransferase activity;N-terminal protein amino acid acetylation	ec:2.3.1.88;ec:2.3.1	
Phvul.002G012300.1	Peroxidase 51	extracellular region;peroxidase activity;heme binding;metal ion binding;response to oxidative stress;hydrogen peroxide catabolic process;oxidation-reduction process;cellular oxidant detoxification	ec:1.11.1.7-lactoperoxidase	Phenylpropanoid biosynthesis
Phvul.002G012600.1	Ribonuclease 3	nucleus;double-stranded RNA binding;ribonuclease III activity;thylakoid membrane organization;production of ta-siRNAs involved in RNA interference;isopentenyl diphosphate biosynthetic process, methylerythritol 4-phosphate pathway;primary miRNA processing;pre-miRNA processing;defense response to virus;RNA phosphodiester bond hydrolysis, endonucleolytic	ec:3.1.30;ec:3.1.26;ec:3.1;ec:3.1.26.3	
Phvul.002G013900.1	suppressor of RPS4-RLD 1-like	nucleus;perinuclear region of cytoplasm;protein complex scaffold;mitotic cell cycle;methylation-dependent chromatin silencing;RNA processing;defense response to bacterium, incompatible interaction;vernalization response;RNA		

		interference;negative regulation of defense response;post-translational protein modification;positive regulation of transcription, DNA-templated		
Phvul.002G014400.1	probable indole-3-acetic acid-amido synthetase			
Phvul.002G014800.1	cytochrome P450 81E8-like	integral component of membrane;iron ion binding;heme binding;aromatase activity;oxidation-reduction process	ec:1.14.14.1-monooxygenase;ec:1.14.14	Linoleic acid metabolism;Arachidonic acid metabolism;Drug metabolism - cytochrome P450;Metabolism of xenobiotics by cytochrome P450;Caffeine metabolism;Aminobenzoate degradation;Tryptophan metabolism;Fatty acid degradation;Retinol metabolism;Steroid hormone biosynthesis
Phvul.002G017000.1	filament-like plant 7			
Phvul.002G017400.1	F-box At5g67140	ligase activity;metabolic process		
Phvul.002G018100.1	MAP3K-like kinase	cytoplasm;protein serine/threonine kinase activity;ATP binding;regulation of mitotic cell cycle;signal transduction by protein phosphorylation;stress-activated protein kinase signaling cascade;activation of protein kinase activity;regulation of apoptotic process	ec:2.7.11	
Phvul.002G018900.1	CLK4-associating serine arginine rich			
Phvul.002G021600.1	Kinase superfamily isoform 2	nucleus;cytosol;ATP binding;calcium-dependent protein serine/threonine kinase activity;protein phosphatase binding;sucrose metabolic process;protein phosphorylation;unsaturated fatty acid biosynthetic process;response to water deprivation;response to salt stress;response to abscisic acid;stomatal movement;regulation of stomatal movement;regulation of anion channel activity;triglyceride biosynthetic process;intracellular signal transduction;growth;defense response to bacterium;hydrogen peroxide catabolic process;leaf development;regulation of reactive oxygen species metabolic process	ec:2.7.11	
Phvul.002G022500.1	nuclear pore complex NUP58 isoform X1	nuclear pore;peroxidase activity;nucleocytoplasmic transport;cellular oxidant detoxification	ec:1.11.1.7-lactoperoxidase	Phenylpropanoid biosynthesis
Phvul.002G022800.1	cytochrome P450 CYP82D47-like	iron ion binding;oxidoreductase activity, acting on paired donors, with incorporation or reduction of molecular oxygen;heme binding;oxidation-reduction process		
Phvul.002G024200.1	DUF4228 domain			
Phvul.002G026600.1	Major facilitator superfamily isoform 1	integral component of membrane;transmembrane transport		
Phvul.002G027000.1	probable Xaa-Pro aminopeptidase P	cytosol;plasma membrane;aminopeptidase activity;N-1-naphthylphthalamic acid binding;metal ion binding;protein N-linked glycosylation;proteolysis;lipid transport;intra-Golgi vesicle-mediated transport;auxin polar transport;lithium ion transport;protein import into peroxisome matrix	ec:3.4.11	
Phvul.002G029800.1	succinate dehydrogenase [ubiquinone] flavo subunit 1, mitochondrial	mitochondrial respiratory chain complex II, succinate dehydrogenase complex (ubiquinone);succinate dehydrogenase (ubiquinone) activity;flavin adenine dinucleotide binding;tricarboxylic acid	ec:1.3.99.1;ec:1.3.5.1-dehydrogenase	Carbon fixation pathways in prokaryotes;Oxidative phosphorylation;Biosynthesis of antibiotics;Citrate cycle

		cycle;mitochondrial electron transport, succinate to ubiquinone		(TCA cycle);Butanoate metabolism
Phvul.002G031200.1	ATPase family AAA domain-containing 3	ATP binding;microtubule-severing ATPase activity;metabolic process	ec:3.6.1;ec:3.6.1.3-adenylpyrophosphatase;ec:3.6.4.3;ec:3.6.1.15-phosphatase	Purine metabolismPurine metabolism;Thiamine metabolism
Phvul.002G031600.1	laccase-11-like	apoplast;copper ion binding;oxidoreductase activity, oxidizing metal ions;hydroquinone:oxygen oxidoreductase activity;lignin biosynthetic process;lignin catabolic process;oxidation-reduction process	ec:1.10.3.2;ec:1.10.3	
Phvul.002G032000.1	pentatricopeptide repeat-containing At5g66520	chloroplast;microtubule-severing ATPase activity;cytidine to uridine editing;mRNA modification;chloroplast RNA modification	ec:3.6.1;ec:3.6.1.3-adenylpyrophosphatase;ec:3.6.4.3;ec:3.6.1.15-phosphatase	Purine metabolismPurine metabolism;Thiamine metabolism
Phvul.002G033000.1	Isoflavone reductase	oxidoreductase activity;oxidation-reduction process		
Phvul.002G033200.1	isoflavone reductase	integral component of membrane;oxidoreductase activity;oxidation-reduction process		
Phvul.002G033200.2	isoflavone reductase	integral component of membrane;oxidoreductase activity;oxidation-reduction process		
Phvul.002G034200.1	heat shock with TPR ,	cellular component organization;single-organism cellular process;single-organism developmental process;anatomical structure development		
Phvul.002G034300.1	zinc finger CCCH domain-containing 55-like	integral component of membrane;nucleotide binding;RNA binding;metal ion binding		
Phvul.002G035300.1	DUF506 family			
Phvul.002G035500.1	abhydrolase domain-containing	hydrolase activity;metabolic process		
Phvul.002G035700.1	peptidyl-prolyl cis-trans isomerase FKBP53	endoplasmic reticulum membrane;peptidyl-prolyl cis-trans isomerase activity;FK506 binding;protein peptidyl-prolyl isomerization;transport;chaperone-mediated protein folding	ec:5.2.1.8	
Phvul.002G035800.1	nuclear transport factor 2 (NTF2) family	chloroplast thylakoid membrane;peptidyl-prolyl cis-trans isomerase activity;protein peptidyl-prolyl isomerization;protein folding;transport;chlorophyll catabolic process	ec:5.2.1.8	
Phvul.002G036500.1	pentatricopeptide repeat-containing At3g18110, chloroplastic	microtubule-severing ATPase activity;metabolic process	ec:3.6.1;ec:3.6.1.3-adenylpyrophosphatase;ec:3.6.4.3;ec:3.6.1.15-phosphatase	Purine metabolismPurine metabolism;Thiamine metabolism
Phvul.002G037400.1	transferring glycosyl group transferase	integral component of membrane;transferase activity;metabolic process		
Phvul.002G037800.1	tudor PWWP MBT superfamily			
Phvul.002G039600.1	SMG9			
Phvul.002G040200.1	Cellulose synthase D3	integral component of membrane;cellulose synthase (UDP-forming) activity;1,4-beta-D-xylan synthase activity;cellulose biosynthetic process;cell wall organization	ec:2.4.1.12-synthase (UDP-forming);ec:2.4.2.24-synthase;ec:2.4.1	Starch and sucrose metabolismAmino sugar and nucleotide sugar metabolism;Starch and sucrose metabolism
Phvul.002G040500.1	heat stress transcription factor A-1e-like	nucleus;transcription factor activity, sequence-specific DNA binding;sequence-specific DNA binding;regulation of transcription, DNA-templated		
Phvul.002G041400.1	PLATZ transcription factor family			
Phvul.002G041700.1	RING FYVE PHD zinc finger ,	nucleic acid binding;zinc ion binding;oxidoreductase activity;oxidation-reduction process		
Phvul.002G043800.1	NETWORKED 1A-like	transferase activity		
Phvul.002G045700.1	Chromodomain-helicase-DNA-binding 4	DNA binding;helicase activity;zinc ion binding;metabolic process	ec:3.6.1;ec:3.6.1.15-phosphatase	Purine metabolism;Thiamine metabolism

Phvul.002G047800.1	glyco membrane precursor GPI-anchored	integral component of membrane		
Phvul.002G048900.1	tyrosyl-DNA phosphodiesterase 1 isoform X1	nucleus;3'-tyrosyl-DNA phosphodiesterase activity;DNA repair;virus induced gene silencing;vegetative phase change	ec:3.1	
Phvul.002G049200.1	probable UDP-3-O-acylglucosamine N-acyltransferase 2, mitochondrial	N-acyltransferase activity;lipid A biosynthetic process	ec:2.3.1	
Phvul.002G049300.1	cysteine-rich receptor kinase 10	plasma membrane;plasmodesma;integral component of membrane;nucleic acid binding;protein serine/threonine kinase activity;ATP binding;methyltransferase activity;protein phosphorylation;defense response;methylation	ec:2.7.11	
Phvul.002G049700.1	DUF1005 family			
Phvul.002G050000.1	cytochrome c heme attachment (chloroplast)	plastid;thylakoid;integral component of membrane;heme binding;cytochrome complex assembly		
Phvul.002G050100.1	NADH-plastoquinone oxidoreductase subunit 4 (chloroplast)	chloroplast thylakoid membrane;integral component of membrane;NADH dehydrogenase (ubiquinone) activity;quinone binding;ATP synthesis coupled electron transport	ec:1.6.5.3-reductase (H+-translocating);ec:1.6.99.5;ec:1.6.99.3-dehydrogenase	Oxidative phosphorylationOxidative phosphorylation
Phvul.002G052200.1	40S ribosomal S29	integral component of membrane;cytosolic small ribosomal subunit;RNA binding;structural constituent of ribosome;zinc ion binding;translation		
Phvul.002G052400.1	probable polyamine transporter At3g19553	integral component of membrane;L-amino acid transmembrane transporter activity;polyamine transmembrane transporter activity;antiporter activity;hydrolase activity;amino acid transmembrane transport;chlorophyll catabolic process;polyamine transmembrane transport		
Phvul.002G053800.1	dnaJ homolog subfamily C member 21-like	nucleus;nucleic acid binding;zinc ion binding;protein folding		
Phvul.002G054300.1	CHROMATIN REMODELING 25	ATP binding;transferase activity;hydrolase activity;synapsis;reciprocal meiotic recombination;male meiosis;response to ionizing radiation;vegetative to reproductive phase transition of meristem;somatic cell DNA recombination;regulation of telomere maintenance;telomere maintenance in response to DNA damage;post-translational protein modification;double-strand break repair via synthesis-dependent strand annealing;positive regulation of transcription, DNA-templated		
Phvul.002G054400.1	presequence protease 1, chloroplastic mitochondrial-like	metalloendopeptidase activity;zinc ion binding;protein processing	ec:3.4.24	
Phvul.002G054700.1	Histone deacetylase 19	nucleus;NAD-dependent histone deacetylase activity (H3-K14 specific);transcription, DNA-templated;regulation of transcription, DNA-templated;histone H3 deacetylation	ec:3.5.1.98	
Phvul.002G054700.2	Histone deacetylase 19	nucleus;NAD-dependent histone deacetylase activity (H3-K14 specific);transcription, DNA-templated;regulation of transcription, DNA-templated;histone H3 deacetylation	ec:3.5.1.98	
Phvul.002G055700.1	ethylene-responsive transcription factor 1A	nucleus;DNA binding;transcription factor activity, sequence-specific DNA binding;transcription, DNA-templated;regulation of transcription, DNA-templated		
Phvul.002G057100.1	DNA mismatch repair MSH4 isoform X3	synaptonemal complex;plasmodesma;germ cell nucleus;damaged DNA binding;ATP binding;mismatched DNA binding;cytokinesis by cell plate formation;double-strand break repair;mitotic recombination;sister chromatid cohesion;mitotic nuclear division;synapsis;response to gamma		

		radiation;regulation of cell cycle process;meiotic mismatch repair involved in reciprocal meiotic recombination;regulation of telomere maintenance;multicellular organism reproduction;meiotic DNA double-strand break formation;telomere maintenance in response to DNA damage		
Phvul.002G057800.1	agenet domain			
Phvul.002G057800.2	agenet domain			
Phvul.002G058500.1	pentatricopeptide repeat-containing At1g09820			
Phvul.002G058500.2	pentatricopeptide repeat-containing At1g09820			
Phvul.002G058500.3	pentatricopeptide repeat-containing At1g09820			
Phvul.002G058700.1	AP-5 complex subunit beta-1-like			
Phvul.002G060400.1	HAT transposon superfamily	DNA binding;protein dimerization activity		
Phvul.002G061100.1	thylakoid lumenal	chloroplast thylakoid membrane		
Phvul.002G061400.1	pentatricopeptide repeat-containing At4g18975, chloroplastic	nutrient reservoir activity;mitotic cell cycle;RNA processing		
Phvul.002G061800.1	oxysterol-binding -related 2A-like isoform X1			
Phvul.002G062500.1	phosphatidylinositol phosphatidylcholine transfer SFH11-like isoform X1	intracellular;transporter activity;transport		
Phvul.002G063500.1	polygalacturonase At1g48100	extracellular region;integral component of membrane;polygalacturonase activity;galacturan 1,4-alpha-galacturonidase activity;carbohydrate metabolic process;cell wall organization	ec:3.2.1.15-pectin depolymerase;ec:3.2.1.67-1,4-alpha-galacturonidase	Starch and sucrose metabolism;Pentose and glucuronate interconversionsStarch and sucrose metabolism;Pentose and glucuronate interconversions
Phvul.002G063700.1	cysteine-rich receptor kinase 2	plasma membrane;plasmodesma;integral component of membrane;protein serine/threonine kinase activity;ATP binding;protein folding;oligopeptide transport;defense response;response to heat;response to high light intensity;response to endoplasmic reticulum stress;response to hydrogen peroxide;protein autophosphorylation	ec:2.7.11	
Phvul.002G063700.2	cysteine-rich receptor kinase 2	plasma membrane;plasmodesma;integral component of membrane;protein serine/threonine kinase activity;ATP binding;protein folding;oligopeptide transport;defense response;response to heat;response to high light intensity;response to endoplasmic reticulum stress;response to hydrogen peroxide;protein autophosphorylation	ec:2.7.11	
Phvul.002G064400.1	NSP-INTERACTING KINASE 3	integral component of membrane;protein serine/threonine kinase activity;transmembrane receptor protein tyrosine kinase activity;non-membrane spanning protein tyrosine kinase activity;ATP binding;microtubule nucleation;regulation of meristem growth;peptidyl-tyrosine phosphorylation;anther development	ec:2.7.11;ec:2.7.10;ec:2.7.10.2-protein-tyrosine kinase;ec:2.7.10.1	T cell receptor signaling pathway
Phvul.002G066000.1	transcription initiation factor IIB-2-like	nucleus;TFIIB-class transcription factor binding;double-stranded DNA binding;translation initiation factor activity;zinc ion binding;RNA polymerase binding;DNA-		

		templated transcription, initiation;regulation of transcription, DNA-templated;translational initiation;embryo development ending in seed dormancy;pollen germination;stomatal lineage progression		
Phvul.002G066300.1	plant T7N9-9			
Phvul.002G067200.1	homeobox-leucine zipper HAT5	nucleus;transcription factor activity, sequence-specific DNA binding;sequence-specific DNA binding;transcription, DNA-templated;regulation of transcription, DNA-templated		
Phvul.002G069000.1	hypothetical protein PHAVU_002G069000g	maltose metabolic process;stomatal complex morphogenesis;starch biosynthetic process;isopentenyl diphosphate biosynthetic process, methylerythritol 4-phosphate pathway;positive regulation of transcription, DNA-templated		
Phvul.002G070100.1	phosphatidylinositol 3-kinase, root isoform	pre-autophagosomal structure;endosome;peroxisome;phosphatidylinositol 3-kinase complex, class III, type I;phosphatidylinositol 3-kinase complex, class III, type II;ATP binding;1-phosphatidylinositol-3-kinase activity;autophagosome assembly;protein phosphorylation;protein N-linked glycosylation;endocytosis;response to salt stress;vegetative to reproductive phase transition of meristem;endosomal transport;protein desumoylation;pexophagy;inositol trisphosphate metabolic process;phosphatidylinositol-3-phosphate biosynthetic process;phosphatidylinositol phosphorylation;inositol phosphate dephosphorylation;phosphatidylinositol-mediated signaling;hydrogen peroxide biosynthetic process;microgametogenesis	ec:2.7.1;ec:2.7.1.137-3-kinase	Inositol phosphate metabolism;Phosphatidylinositol signaling system
Phvul.002G071800.1	DUF936 family			
Phvul.002G072100.1	aldehyde dehydrogenase family 2 member B7, mitochondrial-like	aldehyde dehydrogenase (NAD) activity;vegetative to reproductive phase transition of meristem;protein desumoylation;hydrogen peroxide biosynthetic process;oxidation-reduction process	ec:1.2.1.3-dehydrogenase (NAD+)	Arginine and proline metabolism;Chloroalkane and chloroalkene degradation;Pyruvate metabolism;Biosynthesis of antibiotics;Tryptophan metabolism;Valine, leucine and isoleucine degradation;Limonene and pinene degradation;beta-Alanine metabolism;Lysine degradation;Glycolysis / Gluconeogenesis;Fatty acid degradation;Glycerolipid metabolism;Ascorbate and aldarate metabolism;Histidine metabolism;Pentose and glucuronate interconversions
Phvul.002G076100.1	hypothetical protein PHAVU_002G076100g			
Phvul.002G076700.1	metalloendo ase 1-like	membrane;extracellular matrix;metalloendopeptidase activity;zinc ion binding;proteolysis	ec:3.4.24	
Phvul.002G077100.1	formin 8	integral component of membrane		

Phvul.002G077800.1	paired amphipathic helix Sin3-like 4 isoform X1	histone deacetylase complex;chromatin;RNA polymerase II transcription corepressor activity;histone deacetylase activity;negative regulation of transcription from RNA polymerase II promoter;histone deacetylation	ec:3.5.1.98	
Phvul.002G077800.2	paired amphipathic helix Sin3-like 4 isoform X1	histone deacetylase complex;chromatin;RNA polymerase II transcription corepressor activity;histone deacetylase activity;negative regulation of transcription from RNA polymerase II promoter;histone deacetylation	ec:3.5.1.98	
Phvul.002G078200.1	DNA topoisomerase 2-binding 1-A isoform X1	isomerase activity;metabolic process		
Phvul.002G078200.2	DNA topoisomerase 2-binding 1-A isoform X1	isomerase activity;metabolic process		
Phvul.002G078200.3	DNA topoisomerase 2-binding 1-A isoform X1	isomerase activity;metabolic process		
Phvul.002G078200.4	DNA topoisomerase 2-binding 1-A isoform X1	isomerase activity;metabolic process		
Phvul.002G078300.1	UPF0392 RCOM_0530710	Golgi apparatus;integral component of membrane		
Phvul.002G079600.1	plant F14N23-31			
Phvul.002G079600.2	plant F14N23-31			
Phvul.002G081300.1	probable leucine-rich repeat receptor kinase At1g35710	integral component of membrane;kinase activity;phosphorylation		
Phvul.002G082500.1	transmembrane ,	integral component of membrane;isopentenyl diphosphate biosynthetic process, methylerythritol 4-phosphate pathway		
Phvul.002G085500.1	myb X	integral component of membrane;cytosolic large ribosomal subunit;RNA binding;structural constituent of ribosome;translation;ribosome biogenesis		
Phvul.002G085900.1	probable methyltransferase PMT2	integral component of membrane;S-adenosylmethionine-dependent methyltransferase activity;methylation		
Phvul.002G086200.1	haloacid dehalogenase-like hydrolase domain	hydrolase activity;metabolic process		
Phvul.002G086200.2	haloacid dehalogenase-like hydrolase domain	hydrolase activity;metabolic process		
Phvul.002G089600.1	SNF1-related kinase regulatory subunit gamma-1-like	integral component of membrane;transcription factor activity, sequence-specific DNA binding;kinase activity;sequence-specific DNA binding;regulation of transcription, DNA-templated;phosphorylation		
Phvul.002G090200.1	Sporulation RMD1	mitochondrion;embryo sac egg cell differentiation;regulation of root meristem growth;regulation of DNA endoreduplication;regulation of cell division		
Phvul.002G090200.2	Sporulation RMD1	mitochondrion;embryo sac egg cell differentiation;regulation of root meristem growth;regulation of DNA endoreduplication;regulation of cell division		
Phvul.002G090200.3	Sporulation RMD1	mitochondrion;embryo sac egg cell differentiation;regulation of root meristem growth;regulation of DNA endoreduplication;regulation of cell division		
Phvul.002G090900.1	Eukaryotic translation initiation factor 5A	translation initiation factor activity;translation elongation factor activity;ribosome binding;translational initiation;translational frameshifting;positive regulation of translational elongation;positive regulation of translational termination		
Phvul.002G091300.1	DUF1336 family	regulation of anion channel activity		
Phvul.002G091400.1	probable S-acyltransferase 22	integral component of membrane;zinc ion binding;protein-cysteine S-palmitoyltransferase activity;microtubule nucleation;metabolic	ec:2.3.1.225;ec:2.3.1	

		process;plant-type cell wall organization;plant-type cell wall biogenesis;regulation of meristem growth		
Phvul.002G091700.1	O-fucosyltransferase family	cytoplasm;integral component of membrane;calcium ion binding;transferase activity, transferring glycosyl groups;metabolic process		
Phvul.002G095700.1	phytanoyl-dioxygenase domain	plasma membrane;integral component of membrane;beta-galactosidase activity;phytanoyl-CoA dioxygenase activity;oxidation-reduction process	ec:3.2.1;ec:3.2.1.23-lactase (ambiguous);ec:1.14.11.18;ec:1.14.11	Other glycan degradation;Glycosphingolipid biosynthesis - ganglio series;Sphingolipid metabolism;Galactose metabolism;Glycosaminoglycan degradation
Phvul.002G097900.1	DCD (development and cell death) domain			
Phvul.002G098200.1	TMV resistance N-like isoform X1	ADP binding;signal transduction		
Phvul.002G098200.2	TMV resistance N-like isoform X1	ADP binding;signal transduction		
Phvul.002G098500.1	trypsin family	peptidase activity;proteolysis		
Phvul.002G099000.1	probable inorganic phosphate transporter 1-9	integral component of plasma membrane;substrate-specific transmembrane transporter activity;transmembrane transport		
Phvul.002G099000.2	probable inorganic phosphate transporter 1-9	integral component of plasma membrane;substrate-specific transmembrane transporter activity;transmembrane transport		
Phvul.002G101300.1	pyruvate kinase, cytosolic isozyme	magnesium ion binding;pyruvate kinase activity;kinase activity;potassium ion binding;glycolytic process	ec:2.7.1.40-kinase	Purine metabolism;Pyruvate metabolism;Biosynthesis of antibiotics;Glycolysis / Gluconeogenesis
Phvul.002G103300.1	transmembrane ,	integral component of membrane		
Phvul.002G103300.2	transmembrane ,	integral component of membrane		
Phvul.002G104500.1	DNA-directed RNA polymerase II subunit 1	DNA-directed RNA polymerase II, core complex;RNA polymerase II activity;DNA binding;transcription from RNA polymerase II promoter	ec:2.7.7.6-RNA polymerase	Purine metabolism;Pyrimidine metabolism
Phvul.002G104700.1	L-ascorbate peroxidase 3, peroxisomal	integral component of membrane;peroxidase activity;heme binding;response to oxidative stress;oxidation-reduction process;cellular oxidant detoxification	ec:1.11.1.7-lactoperoxidase	Phenylpropanoid biosynthesis
Phvul.002G106200.1	MID1-COMPLEMENTING ACTIVITY 1-like			
Phvul.002G106200.2	MID1-COMPLEMENTING ACTIVITY 1-like			
Phvul.002G106200.3	MID1-COMPLEMENTING ACTIVITY 1-like			
Phvul.002G106200.4	MID1-COMPLEMENTING ACTIVITY 1-like			
Phvul.002G106900.1	DUF630 family			
Phvul.002G107400.1	FKBP12-interacting of 37 kDa	nucleus;regulation of alternative mRNA splicing, via spliceosome;mRNA methylation		
Phvul.002G108000.1	ferredoxin--NADP reductase, leaf isozyme, chloroplastic	chloroplast thylakoid membrane;chloroplast stroma;chloroplast envelope;thylakoid lumen;apoplast;nucleotide binding;ferredoxin-NADP+ reductase activity;protein binding;poly(U) RNA binding;electron transporter, transferring electrons within the cyclic electron transport pathway of photosynthesis activity;electron transporter, transferring electrons within the noncyclic electron transport pathway of photosynthesis activity;MAPK cascade;pentose-phosphate	ec:1.18.1.2-reductase;ec:1.18.1	Photosynthesis

		shunt;rRNA processing;protein targeting to membrane;response to cold;detection of biotic stimulus;plastid organization;salicylic acid biosynthetic process;photosynthetic electron transport in photosystem I;systemic acquired resistance, salicylic acid mediated signaling pathway;jasmonic acid mediated signaling pathway;response to chitin;photosystem II assembly;regulation of hydrogen peroxide metabolic process;regulation of plant-type hypersensitive response;glucosinolate biosynthetic process;negative regulation of defense response;positive regulation of catalytic activity;regulation of multi-organism process;defense response to fungus		
Phvul.002G109100.1	transcription initiation factor TFIID subunit 14b	integral component of membrane;regulation of transcription, DNA-templated		
Phvul.002G110800.1	histone H1	nucleosome;nucleus;nucleosomal DNA binding;nucleosome assembly		
Phvul.002G111600.1	zinc finger CCCH domain-containing 44	nucleus;DNA binding;zinc ion binding;DNA-templated transcription, initiation;histone modification		
Phvul.002G113100.1	general transcription factor 3C			
Phvul.002G114800.1	GRIP	synaptonemal complex;trans-Golgi network;protein targeting to Golgi;mitotic cell cycle;RNA processing;reciprocal meiotic recombination;gravitropism;cellulose biosynthetic process		
Phvul.002G115200.1	tRNA nucleotidyltransferase poly(A) polymerase	RNA binding;hydrolase activity;CTP:tRNA cytidylyltransferase activity;CTP:3'-cytidine-tRNA cytidylyltransferase activity;ATP:3'-cytidine-cytidine-tRNA adenylyltransferase activity;tRNA 3'-terminal CCA addition	ec:2.7.7.25;ec:2.7.7.72	
Phvul.002G117100.1	Serine threonine-kinase SRPK	protein serine/threonine kinase activity;protein serine/threonine/tyrosine kinase activity;ATP binding;protein phosphorylation	ec:2.7.11;ec:2.7.12.1	
Phvul.002G117500.1	glutamate receptor	integral component of membrane;G-protein coupled receptor activity;ionotropic glutamate receptor activity;ion transport;G-protein coupled receptor signaling pathway;ionotropic glutamate receptor signaling pathway		
Phvul.002G117500.2	glutamate receptor	integral component of membrane;G-protein coupled receptor activity;ionotropic glutamate receptor activity;ion transport;G-protein coupled receptor signaling pathway;ionotropic glutamate receptor signaling pathway		
Phvul.002G117500.3	glutamate receptor	integral component of membrane;G-protein coupled receptor activity;ionotropic glutamate receptor activity;ion transport;G-protein coupled receptor signaling pathway;ionotropic glutamate receptor signaling pathway		
Phvul.002G121700.1	---NA---			
Phvul.002G122000.1	arabinosyltransferase XEG113-like	endosome;trans-Golgi network;integral component of membrane;microtubule-severing ATPase activity;arabinosyltransferase activity;protein glycosylation;cell wall pectin biosynthetic process;root hair cell development	ec:3.6.1;ec:3.6.1.3-adenylpyrophosphatase;ec:3.6.4.3;ec:3.6.1.15-phosphatase	Purine metabolismPurine metabolism;Thiamine metabolism
Phvul.002G122600.1	long-chain-alcohol oxidase FAO4A-like	integral component of membrane;long-chain-alcohol oxidase activity;flavin adenine dinucleotide binding;oxidation-reduction process	ec:1.1.3.20	
Phvul.002G123100.1	hypothetical protein PHAVU_002G123100g			
Phvul.002G123500.1	probable LRR receptor-like serine threonine- kinase At3g47570	integral component of membrane;protein serine/threonine kinase activity;ATP binding;protein phosphorylation	ec:2.7.11	

Phvul.002G127000.1	neutral alpha-glucosidase	chloroplast;integral component of membrane;alpha-1,4-glucosidase activity;carbohydrate binding;maltose alpha-glucosidase activity;maltose metabolic process	ec:3.2.1;ec:3.2.1.20-maltase	Starch and sucrose metabolism;Galactose metabolism
Phvul.002G129100.1	probable methyltransferase PMT20	endosome;trans-Golgi network;integral component of membrane;S-adenosylmethionine-dependent methyltransferase activity;methylation		
Phvul.002G129100.2	probable methyltransferase PMT20	endosome;trans-Golgi network;integral component of membrane;S-adenosylmethionine-dependent methyltransferase activity;methylation		
Phvul.002G129600.1	disease resistance RGA4	ADP binding;defense response		
Phvul.002G129700.1	disease resistance RGA4	ADP binding;defense response		
Phvul.002G130600.1	disease resistance RGA4	ADP binding;defense response		
Phvul.002G133000.1	CSC1 At1g32090	Golgi apparatus;plasma membrane;plasmodesma;integral component of membrane;4-alpha-glucanotransferase activity;glycogen metabolic process	ec:2.4.1.25-disproportionating enzyme	Starch and sucrose metabolism
Phvul.002G133400.1	disease resistance RPM1-like	ADP binding;defense response		
Phvul.002G134200.1	extra-large guanine nucleotide-binding 3 isoform X1	GTPase activity;signal transducer activity;GTP binding;G-protein beta/gamma-subunit complex binding;G-protein coupled receptor signaling pathway;metabolic process;gravitropism;thigmotropism;response to ethylene;response to abscisic acid;response to sucrose;response to glucose;response to fructose;response to mannitol;root development;regulation of root morphogenesis	ec:3.6.1;ec:3.6.1.15-phosphatase	Purine metabolism;Thiamine metabolism
Phvul.002G134200.2	extra-large guanine nucleotide-binding 3 isoform X1	GTPase activity;signal transducer activity;GTP binding;G-protein beta/gamma-subunit complex binding;G-protein coupled receptor signaling pathway;metabolic process;gravitropism;thigmotropism;response to ethylene;response to abscisic acid;response to sucrose;response to glucose;response to fructose;response to mannitol;root development;regulation of root morphogenesis	ec:3.6.1;ec:3.6.1.15-phosphatase	Purine metabolism;Thiamine metabolism
Phvul.002G134200.3	extra-large guanine nucleotide-binding 3 isoform X1	GTPase activity;signal transducer activity;GTP binding;G-protein beta/gamma-subunit complex binding;G-protein coupled receptor signaling pathway;metabolic process;gravitropism;thigmotropism;response to ethylene;response to abscisic acid;response to sucrose;response to glucose;response to fructose;response to mannitol;root development;regulation of root morphogenesis	ec:3.6.1;ec:3.6.1.15-phosphatase	Purine metabolism;Thiamine metabolism
Phvul.002G135100.1	disease resistance RGA2-like	ADP binding;defense response		
Phvul.002G135300.1	plant F3C3-6	chloroplast stroma;leaf morphogenesis;regulation of proton transport;cell differentiation;protein autophosphorylation		
Phvul.002G135800.1	ankyrin repeat	integral component of membrane		
Phvul.002G136400.1	alpha beta hydrolase domain-containing 17B	plasma membrane;hydrolase activity;metabolic process;determination of bilateral symmetry;polarity specification of adaxial/abaxial axis;meristem initiation;regulation of meristem growth		
Phvul.002G136400.2	alpha beta superfamily hydrolase	plasma membrane;hydrolase activity;metabolic process;determination of bilateral symmetry;polarity specification of adaxial/abaxial axis;meristem initiation;regulation of meristem growth		
Phvul.002G137000.1	Homeobox-leucine zipper ROC3	nucleus;lipid binding;sequence-specific DNA binding;regulation of transcription, DNA-templated		
Phvul.002G138500.1	heat shock factor HSF8	nucleus;transcription factor activity, sequence-specific DNA binding;sequence-specific DNA		

		binding;regulation of transcription, DNA-templated;toxin catabolic process		
Phvul.002G142800.1	Transcription factor bHLH93	2-alkenal reductase [NAD(P)] activity;protein dimerization activity;oxidation-reduction process	ec:1.3.1.74	
Phvul.002G142800.2	Transcription factor bHLH93	2-alkenal reductase [NAD(P)] activity;protein dimerization activity;oxidation-reduction process	ec:1.3.1.74	
Phvul.002G142900.1	probable beta-D-xylosidase 6	vacuolar membrane;plant-type cell wall;beta-glucosidase activity;xylan 1,4-beta-xylosidase activity;alpha-L-arabinofuranosidase activity;arabinan catabolic process;xylan catabolic process	ec:3.2.1.55-end alpha-L-arabinofuranosidase;ec:3.2.1.37-1,4-beta-xylosidase;ec:3.2.1;ec:3.2.1.21-gentiobiase	Amino sugar and nucleotide sugar metabolismAmino sugar and nucleotide sugar metabolism;Starch and sucrose metabolismStarch and sucrose metabolism;Cyanoamino acid metabolism;Phenylpropanoid biosynthesis
Phvul.002G143600.1	probable cytosolic oligopeptidase A	chloroplast;metalloendopeptidase activity;metal ion binding;proteolysis;peptide metabolic process	ec:3.4.24	
Phvul.002G146600.1	U-box domain-containing 50	intracellular;MAP kinase kinase kinase activity;ubiquitin-protein transferase activity;ATP binding;ligase activity;MAPK cascade;activation of MAPKK activity;protein ubiquitination	ec:2.7.11;ec:2.7.11.25	
Phvul.002G146700.1	nucleobase-ascorbate transporter 11-like	integral component of membrane;transporter activity;transmembrane transport		
Phvul.002G147900.1	GPI ethanolamine phosphate transferase 2	integral component of endoplasmic reticulum membrane;CP2 mannose-ethanolamine phosphotransferase activity;GPI anchor biosynthetic process		
Phvul.002G148400.1	exocyst subunit exo70 family	integral component of membrane		
Phvul.002G149600.1	flavonol synthase flavanone 3-hydroxylase-like	oxidoreductase activity;metal ion binding;oxidation-reduction process		
Phvul.002G149600.2	flavonol synthase flavanone 3-hydroxylase-like	oxidoreductase activity;metal ion binding;oxidation-reduction process		
Phvul.002G149600.3	flavonol synthase flavanone 3-hydroxylase-like	oxidoreductase activity;metal ion binding;oxidation-reduction process		
Phvul.002G151500.1	MATE efflux family DTX1	plasma membrane;integral component of membrane;drug transmembrane transporter activity;antiporter activity;drug transmembrane transport;regulation of stomatal opening		
Phvul.002G151700.1	Armadillo repeat-containing kinesin 3	kinesin complex;microtubule;ATP binding;microtubule binding;ATP-dependent microtubule motor activity, plus-end-directed;microtubule-based movement;protein localization;metabolic process;cytoskeleton-dependent intracellular transport	ec:3.6.1;ec:3.6.1.3-adenylpyrophosphatase;ec:3.6.4.4;ec:3.6.1.15-phosphatase	Purine metabolismPurine metabolism;Thiamine metabolism
Phvul.002G153400.1	phospholipase D beta 1-like	membrane;phospholipase D activity;calcium ion binding;N-acylphosphatidylethanolamine-specific phospholipase D activity;lipid catabolic process;phosphatidylcholine metabolic process	ec:3.1.1;ec:3.1;ec:3.1.4.4-D	Ether lipid metabolism;Glycerophospholipid metabolism
Phvul.002G155900.1	myosin heavy chain			
Phvul.002G156500.1	vacuolar amino acid transporter 1-like	integral component of membrane;amino acid transmembrane transporter activity;amino acid transmembrane transport		
Phvul.002G157400.1	pre-16S rRNA nuclease isoform X1	nucleus;membrane;DNA binding;methyltransferase activity;zinc ion binding;oxidoreductase activity, acting on paired donors, with incorporation or reduction of molecular oxygen, 2-oxoglutarate as one donor, and incorporation of one atom each of oxygen into both donors;rRNA processing;methylation;oxidation-reduction process	ec:1.14.11	

Phvul.002G157400.2	pre-16S rRNA nuclease isoform X1	nucleus;membrane;DNA binding;methyltransferase activity;zinc ion binding;oxidoreductase activity, acting on paired donors, with incorporation or reduction of molecular oxygen, 2-oxoglutarate as one donor, and incorporation of one atom each of oxygen into both donors;rRNA processing;methylation;oxidation-reduction process	ec:1.14.11	
Phvul.002G157500.1	transcription factor jumonji ( ) domain	nucleus;DNA binding;transcription factor activity, sequence-specific DNA binding;methyltransferase activity;zinc ion binding;oxidoreductase activity, acting on paired donors, with incorporation or reduction of molecular oxygen, 2-oxoglutarate as one donor, and incorporation of one atom each of oxygen into both donors;mitotic cell cycle;regulation of transcription, DNA-templated;rRNA processing;vegetative to reproductive phase transition of meristem;glucuronoxylan metabolic process;protein desumoylation;methylation;xylan biosynthetic process;hydrogen peroxide biosynthetic process;oxidation-reduction process	ec:1.14.11	
Phvul.002G157700.1	RNase P Rpr2 Rpp21 subunit domain			
Phvul.002G157700.2	RNase P Rpr2 Rpp21 subunit domain			
Phvul.002G157700.3	RNase P Rpr2 Rpp21 subunit domain			
Phvul.002G157700.4	RNase P Rpr2 Rpp21 subunit domain			
Phvul.002G160700.1	respiratory burst oxidase homolog A	integral component of membrane;peroxidase activity;calcium ion binding;NAD(P)H oxidase activity;oxidation-reduction process;cellular oxidant detoxification	ec:1.6.3.1;ec:1.11.1.7-lactoperoxidase	Phenylpropanoid biosynthesis
Phvul.002G161000.1	AAA domain-containing	ATP binding;microtubule-severing ATPase activity;metabolic process	ec:3.6.1;ec:3.6.1.3-adenylpyrophosphatase;ec:3.6.4.3;ec:3.6.1.15-phosphatase	Purine metabolismPurine metabolism;Thiamine metabolism
Phvul.002G161400.1	WRKY transcription factor 19			
Phvul.002G164900.1	GDSL esterase lipase At4g10955-like	integral component of membrane;hydrolase activity;lipid metabolic process		
Phvul.002G164900.2	GDSL esterase lipase At4g10955-like	integral component of membrane;hydrolase activity;lipid metabolic process		
Phvul.002G167200.1	beta-galactosidase 3-like	cell wall;beta-galactosidase activity;carbohydrate binding;carbohydrate metabolic process;polyamine catabolic process;plant-type cell wall organization;phenylpropanoid metabolic process;plant-type cell wall biogenesis;regulation of meristem growth;cellular modified amino acid biosynthetic process	ec:3.2.1;ec:3.2.1.23-lactase (ambiguous)	Other glycan degradation;Glycosphingolipid biosynthesis - ganglio series;Sphingolipid metabolism;Galactose metabolism;Glycosaminoglycan degradation
Phvul.002G169000.1	transmembrane ,	integral component of membrane		
Phvul.002G169900.1	hypothetical protein PHAVU_002G169900g			
Phvul.002G171400.1	disease resistance (TIR-NBS-LRR class)	ATP binding;ADP binding;defense response;signal transduction		
Phvul.002G175000.1	U-box domain-containing 45-like	ubiquitin-protein transferase activity;ligase activity;protein ubiquitination		
Phvul.002G175400.1	pre-mRNA-splicing factor ATP-dependent RNA	spliceosomal complex;ATP-dependent RNA helicase activity;ATP binding;poly(A) RNA binding;RNA processing;sister chromatid	ec:3.6.1;ec:3.6.1.3-adenylpyrophosphata	Purine metabolismPurine

	helicase PRP1 isoform X1	cohesion;reciprocal meiotic recombination;response to gamma radiation;glucuronoxylan metabolic process;regulation of telomere maintenance;meiotic DNA double-strand break formation;telomere maintenance in response to DNA damage;meiotic chromosome segregation;xylan biosynthetic process	se;ec:3.6.1.15-phosphatase;ec:2.7.7	metabolism;Thiamine metabolism
Phvul.002G175800.1	carboxy-terminal region remorin			
Phvul.002G176300.1	probable zinc transporter DDB_G0291141	integral component of membrane;cation transmembrane transporter activity;cation transmembrane transport		
Phvul.002G176700.1	type II-like restriction endonuclease,	DNA binding;endonuclease activity;nucleic acid phosphodiester bond hydrolysis	ec:3.1	
Phvul.002G176700.2	type II-like restriction endonuclease,	DNA binding;endonuclease activity;nucleic acid phosphodiester bond hydrolysis	ec:3.1	
Phvul.002G177300.1	RING finger 141	zinc ion binding;ligase activity;metabolic process		
Phvul.002G177800.1	carboxy-terminal region remorin	plasma membrane		
Phvul.002G180400.1	ornithine aminotransferase	cytoplasm;ornithine-oxo-acid transaminase activity;zinc ion binding;pyridoxal phosphate binding;identical protein binding;proline biosynthetic process;ornithine catabolic process;fatty acid beta-oxidation;peroxisome organization;plant-type hypersensitive response;arginine catabolic process to proline via ornithine;organ senescence;arginine catabolic process to glutamate;hyperosmotic salinity response	ec:2.6.1.13-aminotransferase	Arginine and proline metabolism;Biosynthesis of antibiotics
Phvul.002G181300.2	Purple acid phosphatase 3	acid phosphatase activity;dephosphorylation	ec:3.1.3.2-phosphatase;ec:3.1;ec:3.1.3.41-nitrophenyl phosphatase	Aminobenzoate degradationAminobenzoate degradation
Phvul.002G183300.1	Transmembrane 214	integral component of membrane		
Phvul.002G183800.1	pentatricopeptide repeat-containing At4g33170-like	integral component of membrane;zinc ion binding;microtubule-severing ATPase activity;metabolic process	ec:3.6.1;ec:3.6.1.3-adenylpyrophosphatase;ec:3.6.4.3;ec:3.6.1.15-phosphatase	Purine metabolismPurine metabolism;Thiamine metabolism
Phvul.002G184800.1	serine threonine-kinase BRI1-like 2	intracellular;integral component of membrane;transmembrane receptor protein serine/threonine kinase activity;MAP kinase kinase kinase activity;transmembrane receptor protein tyrosine kinase activity;ATP binding;MAPK cascade;activation of MAPKK activity;response to molecule of bacterial origin;transmembrane receptor protein serine/threonine kinase signaling pathway;auxin-activated signaling pathway;brassinosteroid mediated signaling pathway;regulation of meristem growth;stomatal complex morphogenesis;phloem transport;leaf vascular tissue pattern formation;peptidyl-tyrosine phosphorylation;stamen development	ec:2.7.11;ec:2.7.10;ec:2.7.11.25;ec:2.7.10.1	
Phvul.002G186300.1	zinc finger CCCH domain-containing 17	metal ion binding;protein modification by small protein conjugation or removal		
Phvul.002G186600.1	MAP3K-like kinase	cytoplasm;protein serine/threonine kinase activity;ATP binding;regulation of mitotic cell cycle;signal transduction by protein phosphorylation;stress-activated protein kinase signaling cascade;activation of protein kinase activity;regulation of apoptotic process	ec:2.7.11	
Phvul.002G188100.2	translocator assembly and maintenance			
Phvul.002G188100.4	translocator assembly and maintenance			

Phvul.002G188400.1	NAD(P)-binding rossmann-fold ,			
Phvul.002G189100.1	inositol oxygenase 2-like	cytoplasm;iron ion binding;inositol oxygenase activity;inositol catabolic process;oxidation-reduction process	ec:1.13.99.1-oxygenase	Inositol phosphate metabolism;Ascorbate and aldarate metabolism
Phvul.002G189700.1	E3 ubiquitin- ligase UPL1-like isoform X1	ubiquitin-protein transferase activity;ligase activity;protein ubiquitination involved in ubiquitin-dependent protein catabolic process		
Phvul.002G190300.1	RNA-binding pno1-like	nucleolus;RNA binding		
Phvul.002G190900.1	ATP-dependent zinc metalloprotease FTSH 2, chloroplastic	mitochondrion;chloroplast thylakoid membrane;chloroplast envelope;integral component of membrane;ATP-dependent peptidase activity;metalloendopeptidase activity;ATP binding;zinc ion binding;proteolysis;thylakoid membrane organization;photoinhibition;photosystem II repair;PSII associated light-harvesting complex II catabolic process	ec:3.6.1;ec:3.6.1.3-adenylpyrophosphatase;ec:3.6.1.15-phosphatase;ec:3.4.24	Purine metabolismPurine metabolism;Thiamine metabolism
Phvul.002G193700.1	eukaryotic cytochrome b561	integral component of membrane;oxidation-reduction process		
Phvul.002G195100.1	hypothetical protein PHAVU_002G195100g			
Phvul.002G201500.1	alpha N-terminal methyltransferase 1	cytoplasm;methyltransferase activity;dolichyldiphosphatase activity;N-terminal protein amino acid methylation	ec:3.6.1;ec:3.6.1.43-dolichol diphosphatase	N-Glycan biosynthesis
Phvul.002G204200.1	agenet domain			
Phvul.002G204400.1	Auxin-induced 5NG4	plasma membrane;integral component of membrane;transmembrane transporter activity;transmembrane transport		
Phvul.002G204400.2	nodulin 21 -like transporter family	plasma membrane;integral component of membrane;transmembrane transporter activity;transmembrane transport		
Phvul.002G204400.3	nodulin 21 -like transporter family	plasma membrane;integral component of membrane;transmembrane transporter activity;transmembrane transport		
Phvul.002G204400.4	Auxin-induced 5NG4	plasma membrane;integral component of membrane;transmembrane transporter activity;transmembrane transport		
Phvul.002G205600.1	tRNA (guanine(10)-N2)-methyltransferase homolog	intracellular;nucleic acid binding;methyltransferase activity;tRNA modification;protein targeting to mitochondrion;methylation;2-methylguanosine metabolic process		
Phvul.002G206200.1	mitochondrial arginine transporter BAC2	integral component of membrane;structural constituent of ribosome;translation;transmembrane transport		
Phvul.002G206700.1	WRKY transcription factor 19			
Phvul.002G206900.1	BRASSINOSTEROID INSENSITIVE 1-associated receptor kinase 1-like	integral component of membrane;protein serine/threonine kinase activity;transmembrane receptor protein tyrosine kinase activity;ATP binding;peptidyl-tyrosine phosphorylation	ec:2.7.11;ec:2.7.10;ec:2.7.10.1	
Phvul.002G209800.1	outer envelope 64, mitochondrial-like	cytoplasm;integral component of membrane;carbon-nitrogen ligase activity, with glutamine as amido-N-donor;protein targeting to membrane;protein targeting to mitochondrion;nucleotide transport;ER to Golgi vesicle-mediated transport;metabolic process;regulation of plant-type hypersensitive response;ammonium transport;basic amino acid transport;negative regulation of programmed cell death;amino acid import;regulation of ion transport		
Phvul.002G209900.1	metacaspase-1-like isoform X1	cysteine-type endopeptidase activity;proteolysis	ec:3.4;ec:3.4.22	
Phvul.002G211500.1	probable 2-oxoglutarate Fe(II)-dependent dioxygenase	oxidoreductase activity, acting on paired donors, with incorporation or reduction of molecular oxygen, 2-oxoglutarate as one donor, and incorporation of one atom each of	ec:1.14.11	

		oxygen into both donors;metal ion binding;oxidation-reduction process		
Phvul.002G211700.1	5-oxoprolinase	cytosol;plasmodesma;5-oxoprolinase (ATP-hydrolyzing) activity;glutathione catabolic process	ec:3.5.2.9-(ATP-hydrolysing)	Glutathione metabolism
Phvul.002G211700.2	5-oxoprolinase	cytosol;plasmodesma;5-oxoprolinase (ATP-hydrolyzing) activity;glutathione catabolic process	ec:3.5.2.9-(ATP-hydrolysing)	Glutathione metabolism
Phvul.002G211700.3	5-oxoprolinase	cytosol;plasmodesma;5-oxoprolinase (ATP-hydrolyzing) activity;glutathione catabolic process	ec:3.5.2.9-(ATP-hydrolysing)	Glutathione metabolism
Phvul.002G212300.1	chaperone -domain			
Phvul.002G212700.1	ycf20	chloroplast;integral component of membrane;nonphotochemical quenching		
Phvul.002G215600.1	Heat shock 70 kDa , mitochondrial	ATP binding;unfolded protein binding;protein folding		
Phvul.002G217900.1	probably inactive leucine-rich repeat receptor kinase At5g48380	intracellular;integral component of membrane;MAP kinase kinase kinase activity;transmembrane receptor protein tyrosine kinase activity;ATP binding;MAPK cascade;activation of MAPKK activity;peptidyl-tyrosine phosphorylation	ec:2.7.11;ec:2.7.10;ec:2.7.11.25;ec:2.7.10.1	
Phvul.002G218100.1	kinesin motor catalytic domain	kinesin complex;microtubule;microtubule motor activity;ATP binding;microtubule binding;ATPase activity;microtubule-based movement;metabolic process	ec:3.6.1;ec:3.6.1.3-adenylpyrophosphatase;ec:3.6.1.15-phosphatase	Purine metabolismPurine metabolism;Thiamine metabolism
Phvul.002G218100.2	kinesin motor catalytic domain	kinesin complex;microtubule;microtubule motor activity;ATP binding;microtubule binding;ATPase activity;microtubule-based movement;metabolic process	ec:3.6.1;ec:3.6.1.3-adenylpyrophosphatase;ec:3.6.1.15-phosphatase	Purine metabolismPurine metabolism;Thiamine metabolism
Phvul.002G218400.1	hypothetical protein PHAVU_002G218400g	integral component of membrane		
Phvul.002G218400.2	hypothetical protein PHAVU_002G218400g	integral component of membrane		
Phvul.002G219400.1	pentatricopeptide repeat-containing At1g55890, mitochondrial-like			
Phvul.002G219600.1	transcription factor bHLH112-like	nucleus;core promoter sequence-specific DNA binding;transcriptional activator activity, RNA polymerase II transcription regulatory region sequence-specific binding;protein dimerization activity;transcription from RNA polymerase II promoter;positive regulation of transcription from RNA polymerase II promoter		
Phvul.002G221000.1	Transcription factor MYB39	DNA binding;response to stress;metabolic process;response to abiotic stimulus;hormone-mediated signaling pathway;cellular response to acid chemical;cellular response to oxygen-containing compound		
Phvul.002G222500.1	anaphase-promoting complex subunit 4	anaphase-promoting complex;embryo development;regulation of mitotic metaphase/anaphase transition;anaphase-promoting complex-dependent proteasomal ubiquitin-dependent protein catabolic process;plant ovule development		
Phvul.002G223300.1	GDSL esterase lipase At5g14450-like	plant-type cell wall;plasmodesma;hydrolase activity, acting on ester bonds;9,9'-dicis-carotene:quinone oxidoreductase activity;7,9,9'-tricis-neurosporene:quinone oxidoreductase activity;9,9'-di-cis-zeta-carotene desaturation to 7,9,7',9'-tetra-cis-lycopene;oxidation-reduction process	ec:1.3.5.6-desaturase	Carotenoid biosynthesis
Phvul.002G223700.1	secretory carrier-associated membrane	integral component of membrane;protein serine/threonine/tyrosine kinase activity;protein phosphorylation;protein transport	ec:2.7.12.1	
Phvul.002G223900.1	POLLEN DEFECTIVE IN GUIDANCE 1-like	integral component of membrane;multicellular organismal process;single-organism process;anatomical structure development		

Phvul.002G223900.2	POLLEN DEFECTIVE IN GUIDANCE 1-like isoform X1	integral component of membrane		
Phvul.002G224000.1	Bromodomain 4,			
Phvul.002G225200.1	hypothetical protein PHAVU_002G225200g			
Phvul.002G226200.1	splicing factor 3B subunit 2	spliceosomal complex		
Phvul.002G226900.1	RNA polymerase II C-terminal domain phosphatase-like 1	DNA-directed RNA polymerase II, holoenzyme;CTD phosphatase activity;dephosphorylation of RNA polymerase II C-terminal domain	ec:3.1;ec:3.1.3.41-nitrophenyl phosphatase;ec:3.1.3.16-phosphatase	Aminobenzoate degradationT cell receptor signaling pathway
Phvul.002G227400.1	bifunctional 3-dehydroquinate dehydratase shikimate dehydrogenase, chloroplastic-like isoform X1	3-dehydroquinate dehydratase activity;shikimate 3-dehydrogenase (NADP+) activity;NADP binding;shikimate metabolic process;oxidation-reduction process	ec:4.2.1.10-dehydratase;ec:1.1.1.282-dehydrogenase;ec:1.1.1.25-dehydrogenase	Biosynthesis of antibiotics;Phenylalanine, tyrosine and tryptophan biosynthesisBiosynthesis of antibiotics;Phenylalanine, tyrosine and tryptophan biosynthesisBiosynthesis of antibiotics;Phenylalanine, tyrosine and tryptophan biosynthesis
Phvul.002G228300.1	monothiol glutaredoxin-S17	cytosol;electron carrier activity;protein disulfide oxidoreductase activity;nuclear division;nucleolus organization;response to heat;auxin polar transport;cell redox homeostasis;oxidation-reduction process;reactive oxygen species metabolic process		
Phvul.002G228500.1	vesicle-fusing ATPase	vacuole;Golgi stack;plasma membrane;plasmodesma;ATP binding;ATPase activity;intra-Golgi vesicle-mediated transport;metabolic process;Golgi to plasma membrane protein transport;Golgi vesicle docking	ec:3.6.1;ec:3.6.1.3-adenylpyrophosphatase;ec:3.6.1.15-phosphatase	Purine metabolismPurine metabolism;Thiamine metabolism
Phvul.002G231100.1	haloacid dehalogenase superfamily			
Phvul.002G232200.1	homeobox domain	nucleus;DNA binding;transcription, DNA-templated;regulation of transcription, DNA-templated		
Phvul.002G232200.2	homeobox domain	nucleus;DNA binding;transcription, DNA-templated;regulation of transcription, DNA-templated		
Phvul.002G232500.1	DUF4283 domain	nucleic acid binding;zinc ion binding		
Phvul.002G233100.1	1-O-acylglucose:anthocyanin acyltransferase	integral component of membrane;serine-type carboxypeptidase activity;ATP binding;transferase activity, transferring acyl groups other than amino-acyl groups;ATPase activity, coupled to transmembrane movement of substances;secondary metabolic process;proteolysis involved in cellular protein catabolic process;transmembrane transport	ec:3.6.1;ec:3.6.1.3-adenylpyrophosphatase;ec:3.6.1.15-phosphatase;ec:3.4.21;ec:3.4.16	Purine metabolismPurine metabolism;Thiamine metabolism
Phvul.002G233700.1	50S ribosomal L21, mitochondrial	mitochondrial large ribosomal subunit;RNA binding;structural constituent of ribosome;translation		
Phvul.002G233800.1	probable ubiquitin-conjugating enzyme E2 24	cytoplasm;ligase activity;ubiquitin protein ligase binding;ubiquitin protein ligase activity;protein ubiquitination		
Phvul.002G235300.1	pentatricopeptide repeat-containing At4g32450, mitochondrial-like	zinc ion binding;microtubule-severing ATPase activity;metabolic process	ec:3.6.1;ec:3.6.1.3-adenylpyrophosphatase;ec:3.6.4.3;ec:3.6.1.15-phosphatase	Purine metabolismPurine metabolism;Thiamine metabolism
Phvul.002G236500.1	calcium ion-binding	Golgi apparatus;integral component of membrane;nuclear outer membrane-		

		endoplasmic reticulum membrane network;calcium ion binding		
Phvul.002G236700.1	chlorophyllide A oxygenase	chloroplast envelope;integral component of membrane;chlorophyllide a oxygenase [overall] activity;metal ion binding;2 iron, 2 sulfur cluster binding;oxidation-reduction process	ec:1.14.13.122-oxygenase;ec:1.13.12	Porphyrin and chlorophyll metabolism
Phvul.002G236800.1	plant F20M13-60	endosome;trans-Golgi network;integral component of membrane		
Phvul.002G236800.2	plant F20M13-60	endosome;trans-Golgi network;integral component of membrane		
Phvul.002G237900.1	acyl-activating enzyme 17, peroxisomal ,	integral component of membrane;acetate-CoA ligase activity;metabolic process	ec:6.2.1.1-ligase	Carbon fixation pathways in prokaryotes;Pyruvate metabolism;Biosynthesis of antibiotics;Methane metabolism;Glycolysis / Gluconeogenesis;Propanoate metabolism
Phvul.002G241200.1	transmembrane ,			
Phvul.002G242400.1	adipose-regulatory (seipin),	integral component of membrane;lipid storage		
Phvul.002G243400.1	probable dolichyl pyrophosphate Man9 c2 alpha-1,3-glucosyltransferase isoform X1	endoplasmic reticulum membrane;integral component of membrane;dolichyl pyrophosphate Man9GlcNAc2 alpha-1,3-glucosyltransferase activity;protein N-linked glycosylation;oligosaccharide-lipid intermediate biosynthetic process	ec:2.4.1	
Phvul.002G243800.1	5 -3 exonuclease family	nucleus;DNA binding;5'-3' exodeoxyribonuclease activity;DNA repair;sister chromatid cohesion;chromatin silencing by small RNA;meiotic chromosome segregation;nucleic acid phosphodiester bond hydrolysis	ec:3.1.15;ec:3.1.11;ec:3.1	
Phvul.002G246100.1	alpha beta-hydrolase superfamily			
Phvul.002G249700.1	Kinetochore Nuf2	chromosome, centromeric region;vacuolar membrane;plasma membrane;mitotic nuclear division		
Phvul.002G250000.1	phosphatidylinositol 4-phosphate 5-kinase 7-like	ATP binding;1-phosphatidylinositol-4-phosphate 5-kinase activity;phosphatidylinositol phosphorylation	ec:2.7.1.68-5-kinase;ec:2.7.1	Inositol phosphate metabolism;Phosphatidylinositol signaling system
Phvul.002G250100.2	Maternal effect embryo arrest 9	plasma membrane;pollen development;embryo development ending in seed dormancy;hydrogen peroxide catabolic process		
Phvul.002G250300.1	F-box At1g10780-like	plasmodesma;DNA replication initiation;regulation of DNA replication;cell proliferation;regulation of G2/M transition of mitotic cell cycle;histone phosphorylation		
Phvul.002G250300.2	F-box At1g10780-like	plasmodesma;DNA replication initiation;regulation of DNA replication;cell proliferation;regulation of G2/M transition of mitotic cell cycle;histone phosphorylation		
Phvul.002G254100.1	BZIP transcription factor			
Phvul.002G255300.1	transmembrane ,	endosome;trans-Golgi network;integral component of membrane		
Phvul.002G255500.1	ABC transporter D family member 1	integral component of membrane;ATP binding;ATPase activity, coupled to transmembrane movement of substances;metabolic process;transmembrane transport	ec:3.6.1;ec:3.6.1.3-adenylpyrophosphatase;ec:3.6.1.15-phosphatase	Purine metabolismPurine metabolism;Thiamine metabolism
Phvul.002G257300.1	chaperone -like	ATP binding;heat shock protein binding;metal ion binding;unfolded protein binding;protein folding;response to heat		
Phvul.002G257300.2	chaperone -like	ATP binding;heat shock protein binding;metal ion binding;unfolded protein binding;protein folding;response to heat		
Phvul.002G258100.1	cytochrome b561, DM13 and DOMON domain-containing	endosome;trans-Golgi network;integral component of membrane;monooxygenase		

	At5g54830-like isoform X1	activity;heme binding;cytochrome complex assembly;oxidation-reduction process		
Phvul.002G258300.1	sieve element occlusion			
Phvul.002G258400.1	cellulase (glycosyl hydrolase family 5)	integral component of membrane;hydrolase activity, hydrolyzing O-glycosyl compounds;carbohydrate metabolic process		
Phvul.002G258700.1	myosin heavy chain,	protein glycosylation;calcium ion transport;gravitropism		
Phvul.002G258800.1	Agglutinin receptor	integral component of membrane		
Phvul.002G260200.1	clathrin assembly At4g40080	phospholipid binding;carbohydrate metabolic process		
Phvul.002G261300.1	tRNA (cytosine(34)-C(5))-methyltransferase	membrane;RNA binding;tRNA (cytosine-5-)-methyltransferase activity;tRNA methylation	ec:2.1.1	
Phvul.002G262400.1	photosynthetic NDH subunit of lumenal location 4, chloroplastic	endoplasmic reticulum membrane;chloroplast thylakoid membrane;NAD(P)H dehydrogenase complex (plastoquinone);peptidyl-prolyl cis-trans isomerase activity;FK506 binding;NADH dehydrogenase (plastoquinone) activity;protein peptidyl-prolyl isomerization;oxidation-reduction process;chaperone-mediated protein folding	ec:1.6.99.5;ec:1.6.99.3-dehydrogenase;ec:5.2.1.8	Oxidative phosphorylation
Phvul.002G262400.2	photosynthetic NDH subunit of lumenal location 4, chloroplastic	endoplasmic reticulum membrane;chloroplast thylakoid membrane;NAD(P)H dehydrogenase complex (plastoquinone);peptidyl-prolyl cis-trans isomerase activity;FK506 binding;NADH dehydrogenase (plastoquinone) activity;protein peptidyl-prolyl isomerization;oxidation-reduction process;chaperone-mediated protein folding	ec:1.6.99.5;ec:1.6.99.3-dehydrogenase;ec:5.2.1.8	Oxidative phosphorylation
Phvul.002G262400.3	photosynthetic NDH subunit of lumenal location 4, chloroplastic	endoplasmic reticulum membrane;chloroplast thylakoid membrane;NAD(P)H dehydrogenase complex (plastoquinone);peptidyl-prolyl cis-trans isomerase activity;FK506 binding;NADH dehydrogenase (plastoquinone) activity;protein peptidyl-prolyl isomerization;oxidation-reduction process;chaperone-mediated protein folding	ec:1.6.99.5;ec:1.6.99.3-dehydrogenase;ec:5.2.1.8	Oxidative phosphorylation
Phvul.002G262600.1	Binding isoform 1	cytoplasm;plasma membrane;plasmodesma;plant-type cell wall biogenesis;cellulose biosynthetic process;anisotropic cell growth		
Phvul.002G263800.1	cytochrome P450 CYP736A12-like	integral component of membrane;iron ion binding;heme binding;flavonoid 3',5'-hydroxylase activity;secondary metabolite biosynthetic process;oxidation-reduction process	ec:1.14.13.88-3',5'-hydroxylase;ec:1.14.13	Flavone and flavonol biosynthesis;Flavonoid biosynthesis
Phvul.002G266600.1	neutral alkaline invertase 3, chloroplastic	chloroplast;sucrose alpha-glucosidase activity;glycopeptide alpha-N-acetylgalactosaminidase activity;starch metabolic process;sucrose catabolic process;cotyledon development	ec:3.2.1.48-alpha-glucosidase;ec:3.2.1;ec:3.2.1.26-invertase;ec:3.2.1.97	Starch and sucrose metabolismStarch and sucrose metabolism;Galactose metabolism
Phvul.002G267900.1	ribonucleoside-diphosphate reductase large subunit	ribonucleoside-diphosphate reductase complex;ribonucleoside-diphosphate reductase activity, thioredoxin disulfide as acceptor;ATP binding;gluconeogenesis;DNA-dependent DNA replication;cytoskeleton organization;deoxyribonucleoside triphosphate biosynthetic process;deoxyribonucleotide biosynthetic process;proteasomal protein catabolic process;oxidation-reduction process	ec:1.17.4;ec:1.17.4.1-reductase	Purine metabolism;Glutathione metabolism;Pyrimidine metabolism
Phvul.002G267900.2	ribonucleoside-diphosphate reductase large subunit	ribonucleoside-diphosphate reductase complex;ribonucleoside-diphosphate reductase activity, thioredoxin disulfide as acceptor;ATP binding;gluconeogenesis;DNA-dependent DNA replication;cytoskeleton organization;deoxyribonucleoside triphosphate biosynthetic process;deoxyribonucleotide biosynthetic process;proteasomal protein catabolic process;oxidation-reduction process	ec:1.17.4;ec:1.17.4.1-reductase	Purine metabolism;Glutathione metabolism;Pyrimidine metabolism
Phvul.002G268100.1	hypothetical protein			

	PHAVU_002G268100g			
Phvul.002G268200.1	cellulose synthase family	plasma membrane;integral component of membrane;zinc ion binding;cellulose synthase (UDP-forming) activity;cellulose biosynthetic process;cell wall organization	ec:2.4.1.12-synthase (UDP-forming);ec:2.4.1	Starch and sucrose metabolism
Phvul.002G269000.1	probable plastid-lipid-associated 7, chloroplastic			
Phvul.002G273000.1	50S ribosomal L12, chloroplastic-like	ribosome;structural constituent of ribosome;DNA-templated transcription, elongation;translation		
Phvul.002G273600.1	trichome birefringence-like 14	integral component of membrane		
Phvul.002G273900.1	E3 ubiquitin- ligase RGLG2	ubiquitin-protein transferase activity;zinc ion binding;ligase activity;response to wounding;cytokinin metabolic process;jasmonic acid mediated signaling pathway;protein ubiquitination;defense response to bacterium		
Phvul.002G274100.1	hypothetical protein PHAVU_002G274100g			
Phvul.002G274500.1	lipase-like PAD4	membrane;hydrolase activity;lipid metabolic process		
Phvul.002G275100.1	aspartic ase Asp1-like	integral component of membrane;aspartic-type endopeptidase activity;transferase activity;proteolysis;protein catabolic process	ec:3.4.23	
Phvul.002G275600.1	alternative NAD(P)H-ubiquinone oxidoreductase C1, chloroplastic mitochondrial	NADH dehydrogenase activity;oxidation-reduction process	ec:1.6.99.3-dehydrogenase	Oxidative phosphorylation
Phvul.002G275600.2	alternative NAD(P)H-ubiquinone oxidoreductase C1, chloroplastic mitochondrial	NADH dehydrogenase activity;oxidation-reduction process	ec:1.6.99.3-dehydrogenase	Oxidative phosphorylation
Phvul.002G276200.1	CBS domain-containing CBSCBSPB3-like	integral component of membrane		
Phvul.002G276300.1	hypothetical protein PHAVU_002G276300g			
Phvul.002G276500.1	chalcone isomerase	chalcone isomerase activity;flavonoid biosynthetic process	ec:5.5.1.6-isomerase	Flavonoid biosynthesis
Phvul.002G279100.1	E3 ubiquitin- ligase CHFR	nucleus;ubiquitin-protein transferase activity;zinc ion binding;ligase activity;ubiquitin-dependent protein catabolic process;sister chromatid cohesion;mitotic cell cycle checkpoint;reciprocal meiotic recombination;response to gamma radiation;protein ubiquitination;regulation of telomere maintenance;meiotic DNA double-strand break formation;telomere maintenance in response to DNA damage;meiotic chromosome segregation		
Phvul.002G279700.1	respiratory burst oxidase homolog C	integral component of membrane;peroxidase activity;calcium ion binding;oxidoreductase activity, acting on NAD(P)H, oxygen as acceptor;oxidation-reduction process;cellular oxidant detoxification	ec:1.11.1.7-lactoperoxidase	Phenylpropanoid biosynthesis
Phvul.002G280100.1	staphylococcal nuclease domain-containing 1-like	RISC complex;transcription cofactor activity;posttranscriptional gene silencing by RNA;regulation of nucleic acid-templated transcription		
Phvul.002G282000.1	signal recognition particle subunit SRP68-like isoform X1	endoplasmic reticulum;signal recognition particle, endoplasmic reticulum targeting;cytosol;signal recognition particle binding;7S RNA binding;endoplasmic reticulum		

		signal peptide binding;SRP-dependent cotranslational protein targeting to membrane		
Phvul.002G282000.2	signal recognition particle subunit SRP68-like isoform X1	endoplasmic reticulum;signal recognition particle, endoplasmic reticulum targeting;cytosol;signal recognition particle binding;7S RNA binding;endoplasmic reticulum signal peptide binding;SRP-dependent cotranslational protein targeting to membrane		
Phvul.002G284100.1	retrotransposon , , unclassified	integral component of membrane		
Phvul.002G285400.1	integrator complex subunit 9 isoform X1	integrator complex;snRNA processing		
Phvul.002G287000.1	methyl- -binding domain-containing 13-like	nucleus;methyl-CpG binding;negative regulation of transcription from RNA polymerase II promoter;methylation-dependent chromatin silencing		
Phvul.002G287900.1	probable LRR receptor-like serine threonine- kinase At1g63430	integral component of membrane;protein kinase activity;ATP binding;2-alkenal reductase [NAD(P)] activity;protein phosphorylation;oxidation-reduction process	ec:1.3.1.74	
Phvul.002G289600.1	Mitochondrial substrate carrier family B	integral component of membrane;structural constituent of ribosome;translation;transport		
Phvul.002G289600.2	Mitochondrial substrate carrier family B	integral component of membrane;structural constituent of ribosome;translation;transport		
Phvul.002G289700.1	kinesin-4-like isoform X1	kinesin complex;microtubule;microtubule motor activity;ATP binding;microtubule binding;ATPase activity;microtubule-based movement;metabolic process	ec:3.6.1;ec:3.6.1.3-adenylpyrophosphatase;ec:3.6.1.15-phosphatase	Purine metabolismPurine metabolism;Thiamine metabolism
Phvul.002G289700.2	kinesin-4-like isoform X1	kinesin complex;microtubule;microtubule motor activity;ATP binding;microtubule binding;ATPase activity;microtubule-based movement;metabolic process	ec:3.6.1;ec:3.6.1.3-adenylpyrophosphatase;ec:3.6.1.15-phosphatase	Purine metabolismPurine metabolism;Thiamine metabolism
Phvul.002G290000.1	transcription factor EGL1-like	amino acid binding;protein dimerization activity;metabolic process		
Phvul.002G291600.1	CCT motif	nucleus;ribonuclease P activity;response to light stimulus;regulation of flower development;RNA phosphodiester bond hydrolysis, endonucleolytic	ec:3.1.30;ec:3.1.26;ec:3.1;ec:3.1.26.5	
Phvul.002G293200.1	probable WRKY transcription factor 29	transcription factor activity, sequence-specific DNA binding;sequence-specific DNA binding;regulation of transcription, DNA-templated		
Phvul.002G296300.1	myosin heavy chain, cardiac muscle isoform			
Phvul.002G299100.1	probable RNA 3 -terminal phosphate cyclase	nucleolus;membrane;RNA binding;RNA-3'-phosphate cyclase activity;endoribonuclease activity;endonucleolytic cleavage of tricistronic rRNA transcript (SSU-rRNA, 5.8S rRNA, LSU-rRNA)	ec:6.5.1.4;ec:3.1	
Phvul.002G299200.1	patatin-like phospholipase	cytoplasm;membrane;phospholipase activity;acylglycerol lipase activity;lipid catabolic process	ec:3.1.1.23-lipase;ec:3.1.1;ec:3.1.1.1-ali-esterase	Glycerolipid metabolismDrug metabolism - other enzymes
Phvul.002G299500.1	centromere C	kinetochore;centromeric DNA binding;primary metabolic process;cellular macromolecule metabolic process;kinetochore assembly		
Phvul.002G300400.1	3-oxoacyl-[acyl-carrier-] synthase I, chloroplastic-like	3-oxoacyl-[acyl-carrier-protein] synthase activity;beta-ketoacyl-acyl-carrier-protein synthase II activity;fatty acid biosynthetic process	ec:2.3.1.85-synthase;ec:2.3.1.41-synthase I;ec:2.3.1.179-synthase II	Fatty acid biosynthesisBiotin metabolism;Fatty acid biosynthesisBiotin metabolism;Fatty acid biosynthesis
Phvul.002G302200.1	lupeol synthase	lupeol synthase activity;pentacyclic triterpenoid biosynthetic process	ec:5.4.99	
Phvul.002G303000.1	superkiller viralicidic activity 2-like 2	nucleus;cytosol;RNA binding;RNA helicase activity;ATP binding;mitotic cell cycle;mRNA splicing, via spliceosome;RNA catabolic process;cell adhesion;photomorphogenesis;embryo development ending in seed dormancy;seed germination;regulation of flower	ec:3.6.1;ec:3.6.1.15-phosphatase;ec:2.7.7	Purine metabolism;Thiamine metabolism

		development;meristem structural organization;trichome morphogenesis;specification of floral organ identity;seed dormancy process;sugar mediated signaling pathway;vegetative to reproductive phase transition of meristem;cullin deneddylation;protein ubiquitination;histone methylation;protein deubiquitination;lipid storage;production of siRNA involved in RNA interference;production of miRNAs involved in gene silencing by miRNA;post-translational protein modification;actin nucleation;positive regulation of transcription, DNA-templated;response to freezing		
Phvul.002G304300.1	K(+) efflux antiporter 4 isoform X1	integral component of membrane;solute:proton antiporter activity;sodium ion transport;lithium ion transport;hydrogen ion transmembrane transport		
Phvul.002G304300.2	K(+) efflux antiporter 4 isoform X1	integral component of membrane;solute:proton antiporter activity;sodium ion transport;lithium ion transport;hydrogen ion transmembrane transport		
Phvul.002G305800.1	asparagine--tRNA ligase, cytoplasmic 1	cytoplasm;nucleic acid binding;asparagine-tRNA ligase activity;ATP binding;asparaginyl-tRNA aminoacylation	ec:6.1.1;ec:6.1.1.22-ligase	Aminoacyl-tRNA biosynthesis
Phvul.002G307400.1	derlin-1	integral component of membrane;protein folding;response to heat;response to high light intensity;response to endoplasmic reticulum stress;response to hydrogen peroxide		
Phvul.002G308100.1	IQ-DOMAIN 14			
Phvul.002G308700.1	nucleotide-diphospho-sugar transferase domain	integral component of membrane;ceramide glucosyltransferase activity;glucosylceramide biosynthetic process	ec:2.4.1.80-glucosyltransferase;ec:2.4.1	Sphingolipid metabolism
Phvul.002G308800.1	pentatricopeptide repeat-containing At2g15820	chloroplast;endonuclease activity;Group II intron splicing;mRNA cis splicing, via spliceosome;photosystem I assembly;nucleic acid phosphodiester bond hydrolysis	ec:3.1	
Phvul.002G309800.1	transmembrane ,	membrane		
Phvul.002G310300.1	probable glycosyltransferase At3g07620	membrane;glucuronosyl-N-acetylglucosaminyl-proteoglycan 4-alpha-N-acetylglucosaminyltransferase activity;metabolic process	ec:2.4.1.224-4-alpha-N-acetylglucosaminyltransferase;ec:2.4.1	Glycosaminoglycan biosynthesis - heparan sulfate / heparin
Phvul.002G310300.2	probable glycosyltransferase At3g07620	membrane;glucuronosyl-N-acetylglucosaminyl-proteoglycan 4-alpha-N-acetylglucosaminyltransferase activity;metabolic process	ec:2.4.1.224-4-alpha-N-acetylglucosaminyltransferase;ec:2.4.1	Glycosaminoglycan biosynthesis - heparan sulfate / heparin
Phvul.002G312800.1	Chloroplast processing peptidase	chloroplast thylakoid membrane;integral component of membrane;mitochondrial inner membrane peptidase complex;serine-type endopeptidase activity;signal peptide processing;protein processing involved in protein targeting to mitochondrion;thylakoid membrane organization;regulation of cellular protein metabolic process;mitochondrial respiratory chain complex assembly;ncRNA metabolic process;posttranscriptional gene silencing by RNA;cellular biosynthetic process;single-organism biosynthetic process;regulation of nitrogen compound metabolic process;production of small RNA involved in gene silencing by RNA;organic substance biosynthetic process;regulation of cellular macromolecule biosynthetic process	ec:3.4.21	
Phvul.002G313300.1	serine transhydroxymethyl transferase	glycine hydroxymethyltransferase activity;methyltransferase activity;pyridoxal phosphate binding;glycine metabolic process;L-serine metabolic process;methylation;tetrahydrofolate interconversion	ec:2.1.2.1-hydroxymethyltransferase	Biosynthesis of antibiotics;Methane metabolism;One carbon pool by folate;Cyanoamino acid metabolism;Glycine, serine and threonine metabolism;Glyoxylate

				and dicarboxylate metabolism
Phvul.002G313400.1	regulator of Vps4 activity in the MVB pathway			
Phvul.002G314200.1	leucine-rich repeat extensin 4	integral component of membrane;transferase activity;metabolic process		
Phvul.002G315600.1	aluminum-activated malate transporter 9	plant-type vacuole membrane;integral component of membrane;malate transmembrane transporter activity;malate transmembrane transport		
Phvul.002G317000.1	Transcription factor MYB21	DNA binding		
Phvul.002G318800.1	endoglucanase 8-like	cellulase activity;cellulose catabolic process	ec:3.2.1.4-endo-1,4-beta-D-glucanase	Starch and sucrose metabolism
Phvul.002G320100.1	Histidine--tRNA ligase	mitochondrion;histidine-tRNA ligase activity;ATP binding;histidyl-tRNA aminoacylation;mitochondrial translation	ec:6.1.1;ec:6.1.1.21-ligase	Aminoacyl-tRNA biosynthesis
Phvul.002G321200.1	nuclear pore complex NUP155	nuclear pore;nucleolus;plasma membrane;plasmodesma;chloroplast;nucleocytoplasmic transporter activity;structural constituent of nuclear pore;RNA methylation;nucleocytoplasmic transport		
Phvul.002G323400.1	TMV resistance N-like	ADP binding;defense response		
Phvul.002G323800.1	TMV resistance N-like	ATP binding;ADP binding;defense response;signal transduction		
Phvul.002G323800.2	TMV resistance N-like	ATP binding;ADP binding;defense response;signal transduction		
Phvul.002G323800.3	TMV resistance N-like	ATP binding;ADP binding;defense response;signal transduction		
Phvul.002G324500.1	THO complex subunit 2	intracellular;phosphorelay sensor kinase activity;phosphorelay signal transduction system;signal transduction by protein phosphorylation	ec:2.7.3;ec:2.7.13.3	
Phvul.002G325900.1	poly(A) polymerase	nucleus;integral component of membrane;RNA binding;polynucleotide adenylyltransferase activity;mitotic cell cycle;mRNA polyadenylation	ec:2.7.7.19	
Phvul.002G325900.2	poly(A) polymerase	nucleus;integral component of membrane;RNA binding;polynucleotide adenylyltransferase activity;mitotic cell cycle;mRNA polyadenylation	ec:2.7.7.19	
Phvul.002G325900.3	poly(A) polymerase	nucleus;integral component of membrane;RNA binding;polynucleotide adenylyltransferase activity;mitotic cell cycle;mRNA polyadenylation	ec:2.7.7.19	
Phvul.002G328600.1	vacuolar sorting-associated ,	integral component of membrane;extrinsic component of membrane		
Phvul.002G328600.2	vacuolar sorting-associated ,	integral component of membrane;extrinsic component of membrane		
Phvul.002G330500.1	protection of telomeres 1b-like isoform X1	nuclear telomere cap complex;methyltransferase activity;telomerase inhibitor activity;G-rich strand telomeric DNA binding;telomere capping;regulation of telomere maintenance via telomerase;negative regulation of telomerase activity		
Phvul.002G333400.1	rhodanese-like domain-containing 8, chloroplastic isoform X1	integral component of membrane;transferase activity;metabolic process		
Phvul.003G002600.1	hypothetical protein PHAVU_003G002600g	ADP binding;defense response		
Phvul.003G002600.2	hypothetical protein PHAVU_003G002600g	ADP binding;defense response		
Phvul.003G005800.1	zinc finger, C3HC4 type (RING finger)	plasmodesma;zinc ion binding		
Phvul.003G007200.1	dishevelled Egl-10 leckstrin domain	intracellular;electron carrier activity;protein disulfide oxidoreductase activity;intracellular signal transduction;cell redox homeostasis;oxidation-reduction process		

Phvul.003G008000.1	CHROMATIN REMODELING 19	nucleus;DNA binding;helicase activity;ATP binding;transcription, DNA-templated;regulation of transcription, DNA-templated;cellular response to DNA damage stimulus;gene silencing by RNA;cellular response to UV-C	ec:3.6.1;ec:3.6.1.15-phosphatase	Purine metabolism;Thiamine metabolism
Phvul.003G009300.1	cytochrome P450 71D11-like	integral component of membrane;iron ion binding;oxidoreductase activity, acting on paired donors, with incorporation or reduction of molecular oxygen, NAD(P)H as one donor, and incorporation of one atom of oxygen;heme binding;secondary metabolite biosynthetic process;oxidation-reduction process	ec:1.14.13	
Phvul.003G010100.1	deSI At4g17486	integral component of membrane		
Phvul.003G013100.1	tobamovirus multiplication 3-like	integral component of membrane		
Phvul.003G013300.1	GEM 4			
Phvul.003G014300.1	anthranilate synthase beta subunit 2, chloroplastic-like	anthranilate synthase activity;metabolic process	ec:4.1.3.27-synthase	Biosynthesis of antibiotics;Phenylalanine, tyrosine and tryptophan biosynthesis
Phvul.003G016400.1	probable S-adenosylmethionine-dependent methyltransferase At5g38100	methyltransferase activity;methylation		
Phvul.003G017000.1	Two-component response regulator ARR2	nucleus;DNA binding;transcription factor activity, sequence-specific DNA binding;phosphorelay signal transduction system;transcription, DNA-templated;regulation of transcription, DNA-templated;regulation of multicellular organismal development		
Phvul.003G017500.1	WD repeat-containing 74 isoform X1	Cul4-RING E3 ubiquitin ligase complex		
Phvul.003G017500.2	WD repeat-containing 74 isoform X1	Cul4-RING E3 ubiquitin ligase complex		
Phvul.003G019100.1	E3 ubiquitin ligase RIE1	integral component of membrane;zinc ion binding;hydrolase activity;ligase activity;metabolic process		
Phvul.003G019100.2	E3 ubiquitin ligase RIE1	integral component of membrane;zinc ion binding;hydrolase activity;ligase activity;metabolic process		
Phvul.003G019300.1	tRNA:m(4)X modification enzyme TRM13 homolog	tRNA methyltransferase activity;tRNA methylation	ec:2.1.1	
Phvul.003G019400.1	VAC14 homolog	integral component of membrane;PAS complex;hydrolase activity;metabolic process;regulation of lipid kinase activity		
Phvul.003G019400.2	VAC14 homolog	integral component of membrane;PAS complex;hydrolase activity;metabolic process;regulation of lipid kinase activity		
Phvul.003G021200.1	exosome complex exonuclease RRP41			
Phvul.003G022300.1	DNA gyrase subunit A, chloroplastic mitochondrial	chromosome;nucleoid;mediator complex;DNA binding;DNA topoisomerase type II (ATP-hydrolyzing) activity;ATP binding;DNA topological change;chromosome segregation;pyrimidine ribonucleotide biosynthetic process	ec:5.99.1.3;ec:3.6.1;ec:3.6.1.3-adenylpyrophosphatase;ec:5.99.1;ec:3.6.1.15-phosphatase	Purine metabolismPurine metabolism;Thiamine metabolism
Phvul.003G025500.1	nuclear matrix constituent ,			
Phvul.003G027400.1	probable galacturonosyltransferase-like 3	Golgi apparatus;integral component of membrane;polygalacturonate 4-alpha-galacturonosyltransferase activity;polysaccharide biosynthetic process	ec:2.4.1.43-4-alpha-galacturonosyltransferase	Amino sugar and nucleotide sugar metabolism;Starch and sucrose metabolism
Phvul.003G027600.1	probable receptor kinase At1g49730	integral component of membrane;protein serine/threonine kinase activity;ATP binding;protein phosphorylation	ec:2.7.11	

Phvul.003G028000.1	probable transcription factor GLK2	nucleus;DNA binding;transcription, DNA-templated;regulation of transcription, DNA-templated		
Phvul.003G028100.1	Zn-dependent hydrolase of the beta-lactamase fold	chloroplast;hydrolase activity;photosystem II assembly;myo-inositol hexakisphosphate biosynthetic process		
Phvul.003G030200.1	endonuclease 2-like	nucleic acid binding;endonuclease activity;DNA catabolic process;nucleic acid phosphodiester bond hydrolysis	ec:3.1	
Phvul.003G030200.2	endonuclease 2-like	nucleic acid binding;endonuclease activity;DNA catabolic process;nucleic acid phosphodiester bond hydrolysis	ec:3.1	
Phvul.003G031400.1	lipid phosphate phosphatase 2-like isoform X1	integral component of plasma membrane;acid phosphatase activity;phosphatidate phosphatase activity;phospholipid metabolic process;abscisic acid-activated signaling pathway;phospholipid dephosphorylation	ec:3.1.3.4-phosphatase;ec:3.1.3.2-phosphatase;ec:3.1;ec:3.1.3.41-nitrophenyl phosphatase	Sphingolipid metabolism;Ether lipid metabolism;Glycerophospholipid metabolism;Glycerolipid metabolismAminobenzoate degradationAminobenzoate degradation
Phvul.003G031400.2	lipid phosphate phosphatase 2-like isoform X1	integral component of plasma membrane;acid phosphatase activity;phosphatidate phosphatase activity;phospholipid metabolic process;abscisic acid-activated signaling pathway;phospholipid dephosphorylation	ec:3.1.3.4-phosphatase;ec:3.1.3.2-phosphatase;ec:3.1;ec:3.1.3.41-nitrophenyl phosphatase	Sphingolipid metabolism;Ether lipid metabolism;Glycerophospholipid metabolism;Glycerolipid metabolismAminobenzoate degradationAminobenzoate degradation
Phvul.003G031400.3	lipid phosphate phosphatase 2-like isoform X1	integral component of plasma membrane;acid phosphatase activity;phosphatidate phosphatase activity;phospholipid metabolic process;abscisic acid-activated signaling pathway;phospholipid dephosphorylation	ec:3.1.3.4-phosphatase;ec:3.1.3.2-phosphatase;ec:3.1;ec:3.1.3.41-nitrophenyl phosphatase	Sphingolipid metabolism;Ether lipid metabolism;Glycerophospholipid metabolism;Glycerolipid metabolismAminobenzoate degradationAminobenzoate degradation
Phvul.003G032200.1	hypothetical protein PHAVU_003G032200g	integral component of membrane		
Phvul.003G032400.1	RAE1			
Phvul.003G032500.1	auxin efflux carrier	endoplasmic reticulum;plasma membrane;integral component of membrane;auxin efflux transmembrane transporter activity;auxin-activated signaling pathway;auxin polar transport;auxin homeostasis;auxin efflux;transmembrane transport		
Phvul.003G032900.1	ATP-dependent DNA helicase	nucleic acid binding;ATP-dependent DNA helicase activity;ATP binding;DNA repair;DNA recombination;DNA duplex unwinding	ec:3.6.1;ec:3.6.1.3-adenylpyrophosphatase;ec:3.6.1.15-phosphatase	Purine metabolismPurine metabolism;Thiamine metabolism
Phvul.003G034800.1	hypothetical protein PHAVU_003G034800g	cytoplasm;transition metal ion binding;metal ion transport;cellular transition metal ion homeostasis		
Phvul.003G035400.1	alpha-xylosidase 1	alpha-1,4-glucosidase activity;carbohydrate binding;maltose alpha-glucosidase activity;maltose metabolic process	ec:3.2.1;ec:3.2.1.20-maltase	Starch and sucrose metabolism;Galactose metabolism
Phvul.003G036700.1	root meristem growth factor 2			
Phvul.003G036900.1	UDP-glycosyltransferase 89A2-like	intracellular membrane-bounded organelle;quercetin 3-O-glucosyltransferase activity;quercetin 7-O-glucosyltransferase activity;flavonoid biosynthetic process;flavonoid glucuronidation	ec:2.4.1	

Phvul.003G037200.1	GDSL esterase lipase At2g03980-like	hydrolase activity, acting on ester bonds;9,9'-dicis-carotene:quinone oxidoreductase activity;7,9,9'-tricis-neurosporene:quinone oxidoreductase activity;9,9'-di-cis-zeta-carotene desaturation to 7,9,7',9'-tetra-cis-lycopene;oxidation-reduction process	ec:1.3.5.6-desaturase	Carotenoid biosynthesis
Phvul.003G039500.1	BIG GRAIN 1-like E			
Phvul.003G040200.1	receptor kinase 2	integral component of membrane;protein serine/threonine kinase activity;ATP binding;transition metal ion transport;response to molecule of bacterial origin;protein phosphorylation;signal transduction;stomatal complex morphogenesis;regulation of anion channel activity;stamen development	ec:2.7.11	
Phvul.003G041000.1	serine threonine-kinase dst1	chromosome, centromeric region;nucleus;mitochondrion;protein serine/threonine kinase activity;ATP binding;[tyrosine 3-monooxygenase] kinase activity;regulation of mitotic cell cycle;signal transduction by protein phosphorylation;stress-activated protein kinase signaling cascade;activation of protein kinase activity;regulation of apoptotic process	ec:2.7.11;ec:2.7.11.6	
Phvul.003G041000.2	serine threonine-kinase dst1	chromosome, centromeric region;nucleus;mitochondrion;protein serine/threonine kinase activity;ATP binding;[tyrosine 3-monooxygenase] kinase activity;regulation of mitotic cell cycle;signal transduction by protein phosphorylation;stress-activated protein kinase signaling cascade;activation of protein kinase activity;regulation of apoptotic process	ec:2.7.11;ec:2.7.11.6	
Phvul.003G041900.1	ATP-dependent RNA helicase DHX33	ATP-dependent RNA helicase activity;ATP binding;poly(A) RNA binding;RNA processing	ec:3.6.1;ec:3.6.1.3-adenylpyrophosphatase;ec:3.6.1.15-phosphatase;ec:2.7.7	Purine metabolismPurine metabolism;Thiamine metabolism
Phvul.003G042400.1	pentatricopeptide repeat-containing At1g69290			
Phvul.003G043400.1	Quasimodo2 like 2 isoform 2	S-adenosylmethionine-dependent methyltransferase activity;methylation		
Phvul.003G045600.1	NAC domain-containing 29	nucleus;DNA binding;transcription, DNA-templated;regulation of transcription, DNA-templated		
Phvul.003G045700.1	cell division cycle and apoptosis regulator 1	calcium ion binding;regulation of transcription, DNA-templated;cell division		
Phvul.003G047700.1	filament-like plant 7	cell part;regulation of biological process		
Phvul.003G050000.1	methyltransferase 13	methyltransferase activity;hydrolase activity;methylation		
Phvul.003G050400.1	serine arginine repetitive matrix 2-like	motile cilium;nucleotide binding;RNA binding;metal ion binding;microtubule cytoskeleton organization;cytokinesis by cell plate formation;cell adhesion;organ morphogenesis;trichome morphogenesis;vegetative to reproductive phase transition of meristem;protein desumoylation;regulation of chromosome organization;actin nucleation;hydrogen peroxide biosynthetic process		
Phvul.003G050900.1	subtilisin-like protease	cell wall;serine-type endopeptidase activity;proteolysis;glucosinolate biosynthetic process	ec:3.4.21	
Phvul.003G050900.2	subtilisin-like protease	cell wall;serine-type endopeptidase activity;proteolysis;glucosinolate biosynthetic process	ec:3.4.21	
Phvul.003G051300.1	probable acyl-activating enzyme 16, chloroplastic	long-chain fatty acid-CoA ligase activity;long-chain fatty acid metabolic process	ec:6.2.1.3-ligase;ec:6.2.1	Fatty acid degradation;Fatty acid biosynthesis
Phvul.003G052200.1	Ulp1 protease family, carboxy-terminal domain	cysteine-type peptidase activity;proteolysis	ec:3.4	

Phvul.003G052200.2	Ulp1 protease family, carboxy-terminal domain	cysteine-type peptidase activity;proteolysis	ec:3.4	
Phvul.003G052900.1	potassium transporter 6-like	plasma membrane;integral component of membrane;potassium ion transmembrane transporter activity;potassium ion transmembrane transport		
Phvul.003G053100.1	NHL repeat	integral component of membrane;catalytic activity;metabolic process		
Phvul.003G054100.1	transcription factor DIVARICATA-like	nucleus;DNA binding;transcription factor activity, sequence-specific DNA binding;transcription, DNA-templated;regulation of transcription, DNA-templated		
Phvul.003G054100.2	transcription factor DIVARICATA-like	nucleus;DNA binding;transcription factor activity, sequence-specific DNA binding;transcription, DNA-templated;regulation of transcription, DNA-templated		
Phvul.003G055300.1	probable inactive purple acid phosphatase 1 isoform X1	acid phosphatase activity;metal ion binding;tryptophan catabolic process;systemic acquired resistance;indoleacetic acid biosynthetic process;dephosphorylation;regulation of defense response	ec:3.1.3.2-phosphatase;ec:3.1;ec:3.1.3.41-nitrophenyl phosphatase	Aminobenzoate degradationAminobenzoate degradation
Phvul.003G055300.2	probable inactive purple acid phosphatase 1 isoform X1	acid phosphatase activity;metal ion binding;tryptophan catabolic process;systemic acquired resistance;indoleacetic acid biosynthetic process;dephosphorylation;regulation of defense response	ec:3.1.3.2-phosphatase;ec:3.1;ec:3.1.3.41-nitrophenyl phosphatase	Aminobenzoate degradationAminobenzoate degradation
Phvul.003G056300.1	pyruvate dehydrogenase E1 component subunit alpha, mitochondrial-like	nucleus;cytosol;pyruvate dehydrogenase (acetyl-transferring) activity;acetyl-CoA biosynthetic process from pyruvate;oxidation-reduction process	ec:1.2.4.1-dehydrogenase (acetyl-transferring)	Pyruvate metabolism;Biosynthesis of antibiotics;Citrate cycle (TCA cycle);Glycolysis / Gluconeogenesis
Phvul.003G057400.1	IQ-DOMAIN 1	nitrogen compound metabolic process;cellular metabolic process;primary metabolic process;single-organism process;organic substance metabolic process		
Phvul.003G057400.2	IQ-DOMAIN 1	nitrogen compound metabolic process;cellular metabolic process;primary metabolic process;single-organism process;organic substance metabolic process		
Phvul.003G057800.1	hypothetical protein PHAVU_003G057800g			
Phvul.003G061000.1	Proteasome subunit beta type-1	nucleus;cytoplasm;proteasome core complex;threonine-type endopeptidase activity;proteolysis involved in cellular protein catabolic process	ec:3.4.25	
Phvul.003G061200.1	OBP32pep,	jasmonic acid metabolic process;response to jasmonic acid		
Phvul.003G062400.1	trichome birefringence-like 2	Golgi apparatus;integral component of membrane;O-acetyltransferase activity;cell wall organization or biogenesis	ec:2.3.1	
Phvul.003G063000.1	heme-binding 2-like			
Phvul.003G063000.2	heme-binding 2-like			
Phvul.003G064600.1	BTB POZ domain-containing At1g67900-like			
Phvul.003G067800.1	pre-mRNA-splicing factor CEF1-like	DNA binding;transcription factor activity, sequence-specific DNA binding;regulation of transcription, DNA-templated;cell cycle		
Phvul.003G069200.1	alpha-amylase 3, chloroplastic	chloroplast stroma;alpha-amylase activity;calcium ion binding;glucan 1,4-alpha-maltotetraohydrolase activity;starch catabolic process;circadian rhythm	ec:3.2.1;ec:3.2.1.1-glycogenase;ec:3.2.1.60	Starch and sucrose metabolism
Phvul.003G072500.1	TMV resistance N-like isoform X1	integral component of membrane;ADP binding;defense response;signal transduction		

Phvul.003G073700.1	transcription factor VOZ1	sequence-specific DNA binding;response to symbiotic fungus;positive regulation of transcription, DNA-templated;long-day photoperiodism, flowering;positive regulation of long-day photoperiodism, flowering		
Phvul.003G073700.2	transcription factor VOZ1	sequence-specific DNA binding;response to symbiotic fungus;positive regulation of transcription, DNA-templated;long-day photoperiodism, flowering;positive regulation of long-day photoperiodism, flowering		
Phvul.003G073700.3	transcription factor VOZ1	sequence-specific DNA binding;response to symbiotic fungus;positive regulation of transcription, DNA-templated;long-day photoperiodism, flowering;positive regulation of long-day photoperiodism, flowering		
Phvul.003G073900.1	probable glucan endo-1,3-beta-glucosidase A6	glucan endo-1,3-beta-D-glucosidase activity;carbohydrate metabolic process	ec:3.2.1.39-endo-1,3-beta-D-glucosidase;ec:3.2.1;ec:3.2.1.21-gentiobiase	Starch and sucrose metabolismStarch and sucrose metabolism;Cyanoamino acid metabolism;Phenylpropanoid biosynthesis
Phvul.003G074000.1	isoflavone synthase 1	membrane;iron ion binding;oxidoreductase activity, acting on paired donors, with incorporation or reduction of molecular oxygen, NAD(P)H as one donor, and incorporation of one atom of oxygen;isomerase activity;heme binding;secondary metabolite biosynthetic process;oxidation-reduction process	ec:1.14.13	
Phvul.003G074600.1	hypothetical protein PHAVU_003G074600g			
Phvul.003G076200.1	mitotic spindle assembly checkpoint ,	nuclear envelope;mitotic spindle assembly checkpoint;gravitropism		
Phvul.003G076400.1	DEAD-box ATP-dependent RNA helicase 58, chloroplastic isoform X1	nucleic acid binding;ATP-dependent RNA helicase activity;ATP binding;RNA secondary structure unwinding	ec:3.6.1;ec:3.6.1.3-adenylpyrophosphatase;ec:3.6.1.15-phosphatase;ec:2.7.7	Purine metabolismPurine metabolism;Thiamine metabolism
Phvul.003G077300.1	cardiolipin synthase (CMP-forming), mitochondrial	mitochondrion;membrane;cardiolipin synthase activity;calcium-mediated signaling;cardiolipin biosynthetic process	ec:2.7.8	
Phvul.003G077800.1	hypothetical protein PHAVU_003G077800g	DNA binding		
Phvul.003G081300.1	leucine--tRNA ligase, mitochondrial	mitochondrion;chloroplast stroma;integral component of membrane;aminoacyl-tRNA editing activity;leucine-tRNA ligase activity;ATP binding;rRNA processing;leucyl-tRNA aminoacylation;regulation of translational fidelity;chloroplast organization;embryo development ending in seed dormancy;thylakoid membrane organization;stomatal complex morphogenesis;vegetative to reproductive phase transition of meristem;iron-sulfur cluster assembly;mRNA modification;plant ovule development	ec:6.1.1;ec:3.1.1.1-ali-esterase;ec:6.1.1.4-ligase	Drug metabolism - other enzymesAminoacyl-tRNA biosynthesis
Phvul.003G081300.2	leucine--tRNA ligase, mitochondrial	mitochondrion;chloroplast stroma;integral component of membrane;aminoacyl-tRNA editing activity;leucine-tRNA ligase activity;ATP binding;rRNA processing;leucyl-tRNA aminoacylation;regulation of translational fidelity;chloroplast organization;embryo development ending in seed dormancy;thylakoid membrane organization;stomatal complex morphogenesis;vegetative to reproductive phase transition of meristem;iron-sulfur cluster assembly;mRNA modification;plant ovule development	ec:6.1.1;ec:3.1.1.1-ali-esterase;ec:6.1.1.4-ligase	Drug metabolism - other enzymesAminoacyl-tRNA biosynthesis

Phvul.003G081300.3	leucine--tRNA ligase, mitochondrial	mitochondrion;chloroplast stroma;integral component of membrane;aminoacyl-tRNA editing activity;leucine-tRNA ligase activity;ATP binding;rRNA processing;leucyl-tRNA aminoacylation;regulation of translational fidelity;chloroplast organization;embryo development ending in seed dormancy;thylakoid membrane organization;stomatal complex morphogenesis;vegetative to reproductive phase transition of meristem;iron-sulfur cluster assembly;mRNA modification;plant ovule development	ec:6.1.1;ec:3.1.1.1-ali-esterase;ec:6.1.1.4-ligase	Drug metabolism - other enzymesAminoacyl-tRNA biosynthesis
Phvul.003G081500.1	calcium permeable stress-gated cation channel 1-like	integral component of membrane;4-alpha-glucanotransferase activity;glycogen metabolic process	ec:2.4.1.25-disproportionating enzyme	Starch and sucrose metabolism
Phvul.003G082700.1	tropinone reductase-like 3	peroxisome;oxidoreductase activity;root hair elongation;oxidation-reduction process;indolebutyric acid metabolic process;response to indolebutyric acid		
Phvul.003G083100.1	hypothetical protein PHAVU_003G083100g	catalytic activity;metabolic process		
Phvul.003G083700.1	hypothetical protein PHAVU_003G083700g	integral component of membrane		
Phvul.003G084200.1	E3 ubiquitin- ligase UPL5	ubiquitin-protein transferase activity;ligase activity;signal transduction;protein ubiquitination involved in ubiquitin-dependent protein catabolic process		
Phvul.003G084600.1	cytochrome b561 and DOMON domain-containing At5g47530-like	integral component of membrane;oxidation-reduction process		
Phvul.003G086000.1	Microtubule-associated 70-5	microtubule binding;cytoskeleton organization		
Phvul.003G086800.1	nuclear transcription factor Y subunit B-3-like	nucleus;transcription factor activity, sequence-specific DNA binding;sequence-specific DNA binding;protein heterodimerization activity;regulation of transcription, DNA-templated		
Phvul.003G087500.1	probable ADP-ribosylation factor GTPase-activating AGD13	vacuole;plasma membrane;phosphatidylserine decarboxylase activity;phospholipase A2 activity;metabolic process	ec:3.1.1;ec:4.1.1.65-decarboxylase;ec:3.1.1.4-A2;ec:3.1.1.1-ali-esterase	Glycerophospholipid metabolismalpha-Linolenic acid metabolism;Linoleic acid metabolism;Arachidonic acid metabolism;Ether lipid metabolism;Glycerophospholipid metabolismDrug metabolism - other enzymes
Phvul.003G088200.1	C2 and GRAM domain-containing At1g03370-like isoform X1	integral component of membrane;phosphatidylserine decarboxylase activity;ligase activity;metabolic process	ec:4.1.1.65-decarboxylase	Glycerophospholipid metabolism
Phvul.003G088400.1	BTB POZ domain-containing At5g47800-like			
Phvul.003G092100.1	Basic 7S globulin	aspartic-type endopeptidase activity;proteolysis;protein catabolic process	ec:3.4.23	
Phvul.003G092200.1	Basic 7S globulin	aspartic-type endopeptidase activity;proteolysis;protein catabolic process	ec:3.4.23	
Phvul.003G094200.1	hypothetical protein PHAVU_003G094200g			
Phvul.003G096500.1	Glyoxysomal fatty acid beta-oxidation multifunctional MFP-a	cell wall;nucleolus;plasmodesma;3-hydroxyacyl-CoA dehydrogenase activity;enoyl-CoA hydratase activity;long-chain-enoyl-CoA hydratase activity;peroxisome	ec:4.2.1.74-hydratase;ec:4.2.1.17-	Fatty acid degradation;Fatty acid elongationalpha-Linolenic acid

		organization;fatty acid catabolic process;toxin catabolic process;proteasome-mediated ubiquitin-dependent protein catabolic process;response to misfolded protein;oxidation-reduction process;proteasome core complex assembly	hydratase;ec:1.1.1.35-dehydrogenase	metabolism;Biosynthesis of unsaturated fatty acids;Aminobenzoate degradation;Carbon fixation pathways in prokaryotes;Biosynthesis of antibiotics;Tryptophan metabolism;Geraniol degradation;Valine, leucine and isoleucine degradation;Limonene and pinene degradation;beta-Alanine metabolism;Lysine degradation;Fatty acid degradation;Benzoate degradation;Phenylalanine metabolism;Butanoate metabolism;Fatty acid elongation;Propanoate metabolism;Caprolactam degradationCarbon fixation pathways in prokaryotes;Toluene degradation;Biosynthesis of antibiotics;Tryptophan metabolism;Geraniol degradation;Valine, leucine and isoleucine degradation;Primary bile acid biosynthesis;Lysine degradation;Fatty acid degradation;Benzoate degradation;Butanoate metabolism;Fatty acid elongation;Caprolactam degradation
Phvul.003G096600.1	sericin 1-like			
Phvul.003G097100.1	U11 U12 small nuclear ribonucleo 48 kDa			
Phvul.003G097100.2	U11 U12 small nuclear ribonucleo 48 kDa			
Phvul.003G097100.3	U11 U12 small nuclear ribonucleo 48 kDa			
Phvul.003G097100.4	U11 U12 small nuclear ribonucleo 48 kDa			
Phvul.003G098200.1	pyruvate, phosphate dikinase, chloroplastic	nucleus;cytosol;chloroplast stroma;ATP binding;kinase activity;metal ion binding;pyruvate, phosphate dikinase activity;pyruvate metabolic process;phosphorylation	ec:2.7.9.1-phosphate dikinase	Carbon fixation pathways in prokaryotes;Pyruvate metabolism;Carbon fixation in photosynthetic organisms
Phvul.003G098200.2	pyruvate, phosphate dikinase, chloroplastic	nucleus;cytosol;chloroplast stroma;ATP binding;kinase activity;metal ion binding;pyruvate, phosphate dikinase activity;pyruvate metabolic process;phosphorylation	ec:2.7.9.1-phosphate dikinase	Carbon fixation pathways in prokaryotes;Pyruvate metabolism;Carbon fixation in photosynthetic organisms
Phvul.003G099600.1	probable 1-deoxy-D-xylulose-5-phosphate synthase, chloroplastic	1-deoxy-D-xylulose-5-phosphate synthase activity;terpenoid biosynthetic process	ec:2.2.1.7-synthase	Biosynthesis of antibiotics;Terpenoid backbone biosynthesis;Thiamine metabolism

Phvul.003G100000.1	heat shock amino-terminal domain			
Phvul.003G100000.2	heat shock amino-terminal domain			
Phvul.003G103100.1	hypothetical protein PHAVU_003G103100g	zinc ion binding		
Phvul.003G105900.1	Germ cell-less -like 1	Cul3-RING ubiquitin ligase complex;ubiquitin-protein transferase activity;protein ubiquitination		
Phvul.003G106100.1	ubiquitin-like-specific protease ESD4	nucleus;endopeptidase activity;SUMO-specific protease activity;protein desumoylation	ec:3.4	
Phvul.003G108200.1	catalytic phosphatase type 2C	protein serine/threonine phosphatase activity;protein dephosphorylation	ec:3.1;ec:3.1.3.41-nitrophenyl phosphatase;ec:3.1.3.16-phosphatase	Aminobenzoate degradationT cell receptor signaling pathway
Phvul.003G108500.1	Chaperone dnaJ 49	cytosol		
Phvul.003G110600.1	transmembrane ,	integral component of membrane		
Phvul.003G114400.1	serine threonine-kinase STY8 isoform X1	intracellular;phosphorelay sensor kinase activity;MAP kinase kinase kinase activity;ATP binding;phosphorelay signal transduction system;MAPK cascade;activation of MAPKK activity;regulation of transcription, DNA-templated	ec:2.7.11;ec:2.7.11.25;ec:2.7.3;ec:2.7.13.3	
Phvul.003G116300.1	probable WRKY transcription factor 48	transcription factor activity, sequence-specific DNA binding;sequence-specific DNA binding;regulation of transcription, DNA-templated;defense response;single-organism process;localization		
Phvul.003G118000.1	serine carboxypeptidase-like 33	vacuole;integral component of membrane;serine-type carboxypeptidase activity;proteolysis involved in cellular protein catabolic process	ec:3.4.21;ec:3.4.16	
Phvul.003G118700.1	craniofacial development 1			
Phvul.003G119200.1	DNA (cytosine-5)-methyltransferase DRM2 isoform X1	methyltransferase activity;DNA methylation		
Phvul.003G119200.2	DNA (cytosine-5)-methyltransferase DRM2 isoform X1	methyltransferase activity;DNA methylation		
Phvul.003G119300.1	evolutionarily carboxy-terminal region	membrane		
Phvul.003G119300.2	evolutionarily carboxy-terminal region	membrane		
Phvul.003G119300.3	evolutionarily carboxy-terminal region	membrane		
Phvul.003G119300.4	YTH domain family 2	membrane		
Phvul.003G119300.5	YTH domain family 2	membrane		
Phvul.003G119300.6	YTH domain family 2	membrane		
Phvul.003G119400.1	importin beta-like SAD2 homolog	nuclear envelope;cytosol;Ran GTPase binding;protein transporter activity;protein import into nucleus;signal transduction		
Phvul.003G119700.1	phragmoplast orienting kinesin-1	kinesin complex;microtubule;microtubule motor activity;ATP binding;microtubule binding;ATPase activity;nuclear division;cytokinesis by cell plate formation;regulation of DNA replication;nucleolus organization;microtubule-based movement;metabolic process;regulation of G2/M transition of mitotic cell cycle	ec:3.6.1;ec:3.6.1.3-adenylpyrophosphatase;ec:3.6.1.15-phosphatase	Purine metabolismPurine metabolism;Thiamine metabolism
Phvul.003G120100.1	double Clp-N motif P-loop nucleoside triphosphate			

	hydrolase superfamily			
Phvul.003G120400.1	pleiotropic drug resistance 3-like	plasma membrane;integral component of membrane;ATP binding;ATPase activity, coupled to transmembrane movement of substances;metabolic process;transmembrane transport	ec:3.6.1;ec:3.6.1.3-adenylpyrophosphatase;ec:3.6.1.15-phosphatase	Purine metabolismPurine metabolism;Thiamine metabolism
Phvul.003G122600.1	transporter ABC domain	integral component of membrane;ATP binding;ATPase activity, coupled to transmembrane movement of substances;metabolic process;transmembrane transport	ec:3.6.1;ec:3.6.1.3-adenylpyrophosphatase;ec:3.6.1.15-phosphatase	Purine metabolismPurine metabolism;Thiamine metabolism
Phvul.003G122900.1	CHUP1, chloroplastic-like isoform X1	chloroplast outer membrane;chloroplast organization		
Phvul.003G126000.1	brefeldin A-inhibited guanine nucleotide-exchange 5	ARF guanyl-nucleotide exchange factor activity;regulation of ARF protein signal transduction;positive regulation of GTPase activity		
Phvul.003G126000.2	brefeldin A-inhibited guanine nucleotide-exchange 5	ARF guanyl-nucleotide exchange factor activity;regulation of ARF protein signal transduction;positive regulation of GTPase activity		
Phvul.003G126100.1	glucan endo-1,3-beta-glucosidase 5	integral component of membrane;anchored component of membrane;hydrolase activity, hydrolyzing O-glycosyl compounds;protein histidine kinase activity;carbohydrate metabolic process;peptidyl-histidine phosphorylation	ec:2.7.13.3	
Phvul.003G126500.1	pectinesterase pectinesterase inhibitor PPE8B	extracellular region;cell wall;integral component of membrane;pectinesterase activity;aspartyl esterase activity;pectinesterase inhibitor activity;cell wall modification;negative regulation of catalytic activity;pectin catabolic process	ec:3.1.1.11-pectin demethoxylase;ec:3.1.1.1-ali-esterase	Starch and sucrose metabolism;Pentose and glucuronate interconversionsDrug metabolism - other enzymes
Phvul.003G127000.1	1-phosphatidylinositol-3-phosphate 5-kinase FAB1B-like isoform X1	1-phosphatidylinositol-3-phosphate 5-kinase activity;ATP binding;metal ion binding;vacuole organization;pollen development;organ morphogenesis;tissue development;vegetative to reproductive phase transition of meristem;endomembrane system organization;positive regulation of organelle organization;protein desumoylation;regulation of chromosome organization;phosphatidylinositol phosphorylation;hydrogen peroxide biosynthetic process	ec:2.7.1.150-5-kinase;ec:2.7.1	Inositol phosphate metabolism;Phosphatidylinositol signaling system
Phvul.003G127000.2	1-phosphatidylinositol-3-phosphate 5-kinase FAB1B-like isoform X1	1-phosphatidylinositol-3-phosphate 5-kinase activity;ATP binding;metal ion binding;vacuole organization;pollen development;organ morphogenesis;tissue development;vegetative to reproductive phase transition of meristem;endomembrane system organization;positive regulation of organelle organization;protein desumoylation;regulation of chromosome organization;phosphatidylinositol phosphorylation;hydrogen peroxide biosynthetic process	ec:2.7.1.150-5-kinase;ec:2.7.1	Inositol phosphate metabolism;Phosphatidylinositol signaling system
Phvul.003G127200.1	phosphoinositide phosphatase SAC4	mannosyl-oligosaccharide 1,2-alpha-mannosidase activity;phosphoric ester hydrolase activity;metabolic process	ec:3.2.1;ec:3.2.1.24-alpha-D-mannosidase;ec:3.1;ec:3.2.1.113-1,2-alpha-mannosidase	Other glycan degradationVarious types of N-glycan biosynthesis;N-Glycan biosynthesis
Phvul.003G127400.1	kinesin NACK2	kinesin complex;microtubule;plasmodesma;microtubule motor activity;ATP binding;microtubule binding;ATPase activity;microtubule-based movement;male meiosis cytokinesis;metabolic process;pollen development;embryo sac cellularization;radial microtubular system formation	ec:3.6.1;ec:3.6.1.3-adenylpyrophosphatase;ec:3.6.1.15-phosphatase	Purine metabolismPurine metabolism;Thiamine metabolism
Phvul.003G127600.1	Embryogenesis-associated EMB8	integral component of membrane		

Phvul.003G128700.1	Fatty acid biosynthesis 1 isoform 1	3-oxoacyl-[acyl-carrier-protein] synthase activity;beta-ketoacyl-acyl-carrier-protein synthase II activity;very long-chain fatty acid metabolic process;sulfur amino acid metabolic process;glycine catabolic process;unsaturated fatty acid biosynthetic process;oxidoreduction coenzyme metabolic process;vitamin metabolic process;cellular amino acid biosynthetic process;aromatic amino acid family metabolic process;lipoate metabolic process;coenzyme biosynthetic process;nucleotide metabolic process;response to light stimulus;cold acclimation;jasmonic acid biosynthetic process;embryo development ending in seed dormancy;plant-type cell wall biogenesis;chlorophyll biosynthetic process;cell growth;carotenoid biosynthetic process;regulation of lipid metabolic process;isopentenyl diphosphate biosynthetic process, methylerythritol 4-phosphate pathway;photosynthesis, light reaction;secondary metabolic process;cellulose metabolic process;oxylipin biosynthetic process;cuticle development;sulfur compound biosynthetic process	ec:2.3.1.85-synthase;ec:2.3.1.41-synthase I;ec:2.3.1.179-synthase II	Fatty acid biosynthesisBiotin metabolism;Fatty acid biosynthesisBiotin metabolism;Fatty acid biosynthesis
Phvul.003G128800.1	auxin response factor 2	nucleus;DNA binding;transcription, DNA-templated;regulation of transcription, DNA-templated;auxin-activated signaling pathway		
Phvul.003G131200.1	RNI superfamily			
Phvul.003G131200.2	F-box LRR-repeat 3-like			
Phvul.003G131300.1	GDP-fucose O-fucosyltransferase	Golgi apparatus;chloroplast;integral component of membrane;transferase activity, transferring glycosyl groups;metabolic process		
Phvul.003G131500.1	12-oxophytodienoate reductase 3	peroxisome;NADPH dehydrogenase activity;FMN binding;12-oxophytodienoate reductase activity;tryptophan catabolic process;response to water deprivation;response to wounding;indoleacetic acid biosynthetic process;jasmonic acid biosynthetic process;response to ethylene;response to auxin;abscisic acid-activated signaling pathway;jasmonic acid mediated signaling pathway;oxylipin biosynthetic process;hyperosmotic salinity response;oxidation-reduction process	ec:1.3.1.42-reductase;ec:1.6.99.1	alpha-Linolenic acid metabolism
Phvul.003G133000.1	phospholipid:diacyl glycerol acyltransferase 1-like	vacuole;endoplasmic reticulum;integral component of membrane;O-acyltransferase activity;phospholipid:diacylglycerol acyltransferase activity;lipid metabolic process	ec:2.3.1.158-acyltransferase;ec:2.3.1	Glycerolipid metabolism
Phvul.003G134000.1	ribosomal L18ae family	integral component of membrane;cytosolic large ribosomal subunit;structural constituent of ribosome;translation		
Phvul.003G134000.2	ribosomal L18ae family	integral component of membrane;cytosolic large ribosomal subunit;structural constituent of ribosome;translation		
Phvul.003G134000.3	ribosomal L18ae family	integral component of membrane;cytosolic large ribosomal subunit;structural constituent of ribosome;translation		
Phvul.003G134500.1	U-box domain-containing 34	protein serine/threonine kinase activity;non-membrane spanning protein tyrosine kinase activity;ubiquitin-protein transferase activity;ATP binding;ligase activity;response to stress;protein ubiquitination;peptidyl-tyrosine phosphorylation	ec:2.7.11;ec:2.7.10;ec:2.7.10.2-protein-tyrosine kinase	T cell receptor signaling pathway
Phvul.003G135700.1	transcription factor bHLH041	protein dimerization activity		
Phvul.003G135900.1	valacyclovir hydrolase,	hydrolase activity;metabolic process		
Phvul.003G136100.1	BAAT acyl- thioester hydrolase carboxy-terminal	hydrolase activity		
Phvul.003G136600.1	DUF4408 domain	integral component of membrane		

Phvul.003G136600.2	DUF4408 domain	integral component of membrane		
Phvul.003G136700.1	zinc finger CCCH domain-containing 64	cytosol;catalytic activity;metal ion binding;nuclear-transcribed mRNA catabolic process		
Phvul.003G136700.2	zinc finger CCCH domain-containing 64	cytosol;catalytic activity;metal ion binding;nuclear-transcribed mRNA catabolic process		
Phvul.003G140600.1	Chaperone 1	protein metabolic process		
Phvul.003G140800.1	Transcription factor bHLH35	protein dimerization activity		
Phvul.003G141400.1	pathogenesis-related homeodomain -like	nucleus;DNA binding;zinc ion binding		
Phvul.003G141400.2	pathogenesis-related homeodomain -like	nucleus;DNA binding;zinc ion binding		
Phvul.003G142300.1	DUF4228 domain			
Phvul.003G143300.1	glucose-6-phosphate 1-epimerase	integral component of membrane;carbohydrate binding;glucose-6-phosphate 1-epimerase activity;carbohydrate metabolic process	ec:5.1.3.15-1-epimerase	Biosynthesis of antibiotics;Glycolysis / Gluconeogenesis
Phvul.003G145300.1	afadin alpha-actinin-binding			
Phvul.003G145400.1	NADPH--cytochrome P450 reductase	endoplasmic reticulum membrane;integral component of membrane;NADPH-hemoprotein reductase activity;iron ion binding;FMN binding;[methionine synthase] reductase activity;oxidation-reduction process	ec:1.16.1.8;ec:1.16.1;ec:1.6.2.4	
Phvul.003G147300.1	probable xyloglucan endotransglucosylase hydrolase 23	cell wall;integral component of membrane;apoplast;hydrolase activity, hydrolyzing O-glycosyl compounds;xyloglucan:xyloglucosyl transferase activity;xyloglucan endotransglucosylase activity;xyloglucan metabolic process;cell wall modification;cell wall biogenesis;primary root development	ec:2.4.1;ec:2.4.1.207	
Phvul.003G148900.1	probable 1-deoxy-D-xylulose-5-phosphate synthase 2, chloroplastic	1-deoxy-D-xylulose-5-phosphate synthase activity;terpenoid biosynthetic process	ec:2.2.1.7-synthase	Biosynthesis of antibiotics;Terpenoid backbone biosynthesis;Thiamine metabolism
Phvul.003G150000.1	alpha-rhamnosidase	integral component of membrane		
Phvul.003G150000.2	alpha-rhamnosidase			
Phvul.003G150000.3	alpha-rhamnosidase	integral component of membrane		
Phvul.003G150000.4	alpha-rhamnosidase			
Phvul.003G150000.5	alpha-rhamnosidase			
Phvul.003G150000.6	alpha-rhamnosidase	integral component of membrane		
Phvul.003G152500.1	HAD-superfamily hydrolase, subfamily IG, 5 -nucleotidase isoform 1	5'-nucleotidase activity;metal ion binding;dephosphorylation;protein maturation	ec:3.1.3.5-uridine 5'-nucleotidase;ec:3.1;ec:3.1.3.41-nitrophenyl phosphatase;ec:3.1.3.31	Purine metabolism;Nicotinate and nicotinamide metabolism;Pyrimidine metabolismAminobenzoate degradation
Phvul.003G154500.1	snRNA-activating complex subunit isoform X1	integral component of membrane		
Phvul.003G156400.1	ubiquitin carboxyl-terminal hydrolase 23	thiol-dependent ubiquitinyl hydrolase activity;ubiquitin-dependent protein catabolic process;protein deubiquitination	ec:3.4;ec:3.4.19.12	
Phvul.003G157600.1	Fip1 [V]	binding		
Phvul.003G158900.1	DCN1 5	ubiquitin ligase complex;ubiquitin conjugating enzyme binding;ubiquitin-like protein binding;cullin family protein binding;protein neddylation;positive regulation of ubiquitin-protein transferase activity		

Phvul.003G161500.1	probable receptor kinase At5g20050	plasma membrane;integral component of membrane;protein serine/threonine kinase activity;transmembrane receptor protein tyrosine kinase activity;ATP binding;response to nitrate;nitrate transport;peptidyl-tyrosine phosphorylation	ec:2.7.11;ec:2.7.10;ec:2.7.10.1	
Phvul.003G162700.1	nudix hydrolase 19, chloroplastic	cytosol;NAD+ diphosphatase activity;metal ion binding;metabolic process	ec:3.6.1;ec:3.6.1.9-diphosphatase;ec:3.6.1.22-diphosphatase	Purine metabolism;Pantothenate and CoA biosynthesis;Nicotinate and nicotinamide metabolism;Starch and sucrose metabolismNicotinate and nicotinamide metabolism
Phvul.003G163300.1	axonemal dynein light chain,	microtubule associated complex;motile cilium;microtubule-based process		
Phvul.003G163600.1	F-box kelch-repeat At1g55270-like	Cul3-RING ubiquitin ligase complex;ubiquitin-protein transferase activity;protein ubiquitination		
Phvul.003G164500.1	chaperone -like isoform X1	chloroplast;heat shock protein binding;unfolded protein binding;pentose-phosphate shunt;rRNA processing;plastid organization;photosynthetic electron transport in photosystem I;photosystem II assembly;isopentenyl diphosphate biosynthetic process, methylerythritol 4-phosphate pathway		
Phvul.003G164500.2	chaperone -like isoform X2	heat shock protein binding;unfolded protein binding;nucleobase-containing compound metabolic process;cellular component organization;glyceraldehyde-3-phosphate metabolic process;photosynthesis, light reaction;cellular component biogenesis;small molecule metabolic process		
Phvul.003G165500.1	EIN3-binding F-box 1-like			
Phvul.003G166400.1	glycoside hydrolase family 17	integral component of membrane;anchored component of membrane;hydrolase activity, hydrolyzing O-glycosyl compounds;carbohydrate metabolic process		
Phvul.003G166600.1	plastocyanin-like domain	integral component of membrane;anchored component of plasma membrane;electron carrier activity		
Phvul.003G167300.1	clathrin assembly At2g25430	clathrin-coated vesicle;1-phosphatidylinositol binding;clathrin binding;clathrin coat assembly		
Phvul.003G169100.1	kDa proline-rich	plasmodesma;chloroplast thylakoid membrane;integral component of membrane;apoplast		
Phvul.003G169300.1	BSD domain			
Phvul.003G169300.2	BSD domain			
Phvul.003G169700.1	probable glycosyltransferase At3g42180	integral component of membrane;glucuronosyl-N-acetylglucosaminyl-proteoglycan 4-alpha-N-acetylglucosaminyltransferase activity;metabolic process	ec:2.4.1.224-4-alpha-N-acetylglucosaminyltransferase;ec:2.4.1	Glycosaminoglycan biosynthesis - heparan sulfate / heparin
Phvul.003G171300.1	monogalactosyldiacylglycerol synthase 2, chloroplastic	1,2-diacylglycerol 3-beta-galactosyltransferase activity;glycolipid biosynthetic process	ec:2.4.1.46-synthase;ec:2.4.1	Glycerolipid metabolism
Phvul.003G172900.1	NUCLEAR FUSION DEFECTIVE 4-like	integral component of membrane		
Phvul.003G173700.1	scythe ubiquitin-like domain			
Phvul.003G175300.1	D111 G-patch domain	nucleic acid binding		
Phvul.003G175700.1	dicer 4 isoform X1	nucleus;cytoplasm;RISC complex;DNA binding;RNA binding;helicase activity;ribonuclease III activity;ATP binding;mitotic cell cycle;mRNA splicing, via spliceosome;DNA methylation;chromatin silencing;DNA-templated transcription, termination;cell-cell signaling;virus induced gene silencing;photomorphogenesis;embryo	ec:3.6.1;ec:3.1.30;ec:3.1.26;ec:3.1;ec:3.1.26.3;ec:3.6.1.15-phosphatase	Purine metabolism;Thiamine metabolism

		development ending in seed dormancy;seed germination;regulation of flower development;meristem structural organization;vegetative phase change;seed dormancy process;sugar mediated signaling pathway;maintenance of DNA methylation;vegetative to reproductive phase transition of meristem;production of ta-siRNAs involved in RNA interference;production of lsiRNA involved in RNA interference;protein ubiquitination;covalent chromatin modification;lipid storage;production of miRNAs involved in gene silencing by miRNA;post-translational protein modification;positive regulation of transcription, DNA-templated;response to freezing;RNA phosphodiester bond hydrolysis, endonucleolytic		
Phvul.003G176600.1	tRNA (guanine(37)-N1)-methyltransferase 2 isoform X1	nucleus;mitochondrial matrix;integral component of membrane;hydrolase activity, hydrolyzing O-glycosyl compounds;protein histidine kinase activity;tRNA (guanine(37)-N(1))-methyltransferase activity;carbohydrate metabolic process;peptidyl-histidine phosphorylation;tRNA methylation	ec:2.1.1;ec:2.1.1.228;ec:2.7.13.3	
Phvul.003G177900.1	translation initiation factor eIF-2B subunit delta-like	eukaryotic translation initiation factor 2B complex;translation initiation factor activity;guanyl-nucleotide exchange factor activity;S-methyl-5-thioribose-1-phosphate isomerase activity;translational initiation;L-methionine biosynthetic process from methylthioadenosine;positive regulation of GTPase activity;negative regulation of translational initiation	ec:5.3.1.23-isomerase	Cysteine and methionine metabolism
Phvul.003G178300.1	Adenine nucleotide alpha hydrolases-like superfamily isoform 2	response to stress		
Phvul.003G178300.2	universal stress	response to stress		
Phvul.003G178600.1	C3HC zinc finger	nucleus;zinc ion binding		
Phvul.003G179000.1	DEAD-box ATP-dependent RNA helicase 27	RNA binding;ATP-dependent RNA helicase activity;ATP binding;RNA secondary structure unwinding	ec:3.6.1;ec:3.6.1.3-adenylpyrophosphatase;ec:3.6.1.15-phosphatase;ec:2.7.7	Purine metabolismPurine metabolism;Thiamine metabolism
Phvul.003G179400.1	G patch domain-containing TGH	nucleus;primary miRNA binding;pre-miRNA binding;mitotic cell cycle;nuclear-transcribed mRNA catabolic process;phloem or xylem histogenesis;production of siRNA involved in RNA interference;production of miRNAs involved in gene silencing by miRNA		
Phvul.003G179500.1	phospholipid-transporting ATPase 9	Golgi membrane;trans-Golgi network;plasma membrane;integral component of membrane;magnesium ion binding;phospholipid-translocating ATPase activity;ATP binding;metabolic process;phospholipid translocation;Golgi vesicle budding	ec:3.6.1;ec:3.6.1.3-adenylpyrophosphatase;ec:3.6.1.15-phosphatase;ec:3.6.3.1	Purine metabolismPurine metabolism;Thiamine metabolism
Phvul.003G181800.1	PHD and RING finger domain-containing 1	zinc ion binding		
Phvul.003G181900.1	Transcription factor BIM1	protein dimerization activity		
Phvul.003G183300.1	presenilins-associated rhomboid , mitochondrial	integral component of membrane;serine-type endopeptidase activity;protein processing	ec:3.4.21	
Phvul.003G184300.1	glycerophosphodiester phosphodiesterase GDPD6	vacuole;glycerophosphodiester phosphodiesterase activity;glycerophosphocholine phosphodiesterase activity;lipid metabolic process	ec:3.1;ec:3.1.4.46-phosphodiesterase;ec:3.1.4.2-phosphodiesterase	Glycerophospholipid metabolismGlycerophospholipid metabolism
Phvul.003G185800.1	diacylglycerol kinase 1	intracellular;integral component of membrane;diacylglycerol kinase activity;calcium ion binding;ATP binding;protein	ec:2.7.1.107-kinase (ATP)	Glycerophospholipid metabolism;Glycerolipid

		kinase C-activating G-protein coupled receptor signaling pathway;phosphorylation;intracellular signal transduction		metabolism;Phosphatidylinositol signaling system
Phvul.003G186200.1	centrosomal of 112 kDa-like isoform X1			
Phvul.003G186200.2	centrosomal of 112 kDa-like isoform X1			
Phvul.003G186400.1	LTV1 homolog			
Phvul.003G186400.2	LTV1 homolog			
Phvul.003G186800.1	carboxy-terminal region remorin			
Phvul.003G186800.2	carboxy-terminal region remorin			
Phvul.003G186900.1	muscle M-line assembly unc-89 isoform X2	calmodulin binding		
Phvul.003G186900.2	muscle M-line assembly unc-89 isoform X2	calmodulin binding		
Phvul.003G186900.3	muscle M-line assembly unc-89 isoform X2	calmodulin binding		
Phvul.003G187800.1	formin 20			
Phvul.003G187800.2	formin 20			
Phvul.003G188200.1	glycosyltransferase 5	integral component of membrane;transferase activity, transferring glycosyl groups;metabolic process		
Phvul.003G188300.1	pentatricopeptide repeat-containing At5g61370, mitochondrial-like			
Phvul.003G188900.1	galactoside 2-alpha-L-fucosyltransferase-like isoform X1	integral component of membrane;Golgi cisterna membrane;galactoside 2-alpha-L-fucosyltransferase activity;protein glycosylation;fucosylation;cell wall biogenesis;cell wall organization	ec:2.4.1.69-2-alpha-L-fucosyltransferase;ec:2.4.1	Glycosphingolipid biosynthesis - globo series;Glycosphingolipid biosynthesis - lacto and neolacto series
Phvul.003G189300.1	cyclic dof factor 3-like	DNA binding;regulation of transcription, DNA-templated		
Phvul.003G190500.1	alpha beta hydrolase family	chloroplast thylakoid membrane;chloroplast envelope;integral component of membrane;hydrolase activity;MAPK cascade;regulation of transcription, DNA-templated;protein targeting to membrane;response to bacterium;systemic acquired resistance, salicylic acid mediated signaling pathway;jasmonic acid mediated signaling pathway;regulation of hydrogen peroxide metabolic process;regulation of plant-type hypersensitive response;negative regulation of defense response;regulation of protein dephosphorylation		
Phvul.003G191800.1	nucleolar and coiled-body phospho 1-like			
Phvul.003G194000.1	pentatricopeptide repeat-containing At2g45350, chloroplastic	microtubule-severing ATPase activity;metabolic process	ec:3.6.1;ec:3.6.1.3-adenylpyrophosphatase;ec:3.6.4.3;ec:3.6.1.15-phosphatase	Purine metabolismPurine metabolism;Thiamine metabolism
Phvul.003G194600.1	HIPL1 -like	integral component of membrane;oxidoreductase activity, acting on the CH-OH group of donors, quinone or similar compound as acceptor;quinone binding;carbohydrate metabolic process;oxidation-reduction process		
Phvul.003G195300.1	peroxisomal small heat shock	cellular biosynthetic process		
Phvul.003G197300.1	probable alpha-mannosidase I MNS5	endoplasmic reticulum;membrane;mannosyl-oligosaccharide 1,2-alpha-mannosidase activity;calcium ion binding;N-glycan processing;regulation of mitotic cell cycle;ER-associated ubiquitin-dependent protein	ec:3.2.1;ec:3.2.1.24-alpha-D-mannosidase;ec:3.2.1.113-1,2-alpha-mannosidase	Other glycan degradationVarious types of N-glycan biosynthesis;N-Glycan biosynthesis

		catabolic process;endoplasmic reticulum unfolded protein response;glycoprotein ERAD pathway		
Phvul.003G199600.1	Phosphoglycerate mutase family isoform 1	cytosol;phosphatase activity;leaf morphogenesis;dephosphorylation;mRNA modification;cell differentiation	ec:3.1;ec:3.1.3.41-nitrophenyl phosphatase	Aminobenzoate degradation
Phvul.003G200300.1	1-deoxy-D-xylulose 5-phosphate reductoisomerase, chloroplastic	isomerase activity;manganese ion binding;1-deoxy-D-xylulose-5-phosphate reductoisomerase activity;NADPH binding;isopentenyl diphosphate biosynthetic process, methylerythritol 4-phosphate pathway involved in terpenoid biosynthetic process;oxidation-reduction process	ec:1.1.1.267-reductoisomerase	Biosynthesis of antibiotics;Terpenoid backbone biosynthesis
Phvul.003G200600.1	dof zinc finger	membrane;DNA binding;regulation of transcription, DNA-templated;tissue development		
Phvul.003G200700.1	pantothenate kinase 2	cytosol;pantothenate kinase activity;ATP binding;protein N-linked glycosylation;coenzyme A biosynthetic process;phosphorylation	ec:2.7.1.33-kinase	Pantothenate and CoA biosynthesis
Phvul.003G201000.1	iron-sulfur cluster assembly 1-like	mitochondrial matrix;ferrous iron binding;iron-sulfur transferase activity;2 iron, 2 sulfur cluster binding;4 iron, 4 sulfur cluster binding;cellular iron ion homeostasis;[2Fe-2S] cluster assembly;protein maturation by iron-sulfur cluster transfer		
Phvul.003G201700.1	Mpp10	small nucleolar ribonucleoprotein complex;cytosol;viral nucleocapsid;small-subunit processome;Mpp10 complex;rRNA processing;protein import into nucleus		
Phvul.003G202200.1	embryo defective 2759 ,	integral component of membrane		
Phvul.003G202900.1	quinolinate synthase, chloroplastic	integral component of membrane;enzyme activator activity;quinolinate synthetase A activity;transferase activity;protein homodimerization activity;4 iron, 4 sulfur cluster binding;aerobic respiration;NAD biosynthetic process;iron-sulfur cluster assembly;quinolinate biosynthetic process;positive regulation of sulfur metabolic process;positive regulation of transferase activity		
Phvul.003G203100.1	probable LRR receptor-like serine threonine- kinase At4g26540	integral component of membrane;protein serine/threonine kinase activity;ATP binding;2-alkenal reductase [NAD(P)] activity;protein phosphorylation;oxidation-reduction process	ec:2.7.11;ec:1.3.1.74	
Phvul.003G203600.1	LATERAL ORGAN BOUNDARIES-like	carbamoyl-phosphate synthase (glutamine-hydrolyzing) activity;metabolic process;organ boundary specification between lateral organs and the meristem	ec:6.3.5.5-synthase (glutamine-hydrolysing)	Alanine, aspartate and glutamate metabolism;Pyrimidine metabolism
Phvul.003G203600.2	LATERAL ORGAN BOUNDARIES-like	carbamoyl-phosphate synthase (glutamine-hydrolyzing) activity;metabolic process;organ boundary specification between lateral organs and the meristem	ec:6.3.5.5-synthase (glutamine-hydrolysing)	Alanine, aspartate and glutamate metabolism;Pyrimidine metabolism
Phvul.003G204700.1	plant intracellular Ras-group-related LRR 9-like	hydrolase activity;metabolic process		
Phvul.003G206000.1	Manganese-dependent ADP-ribose CDP-alcohol diphosphatase	ADP-ribose diphosphatase activity;CDP-glycerol diphosphatase activity;metabolic process	ec:3.6.1;ec:3.6.1.16-diphosphatase;ec:3.6.1.13-diphosphatase	Glycerophospholipid metabolismPurine metabolism
Phvul.003G208800.1	hypothetical protein PHAVU_003G208800g	peroxisome;regulation of gene expression		
Phvul.003G209200.1	Malate synthase, glyoxysomal	glyoxysome;malate synthase activity;glyoxylate cycle;tricarboxylic acid cycle	ec:2.3.3.9-synthase	Pyruvate metabolism;Glyoxylate and dicarboxylate metabolism
Phvul.003G210800.1	probable inactive purple acid phosphatase 27	integral component of membrane;acid phosphatase activity;metal ion binding;dephosphorylation	ec:3.1.3.2-phosphatase;ec:3.1;ec:3.1.3.41-nitrophenyl phosphatase	Aminobenzoate degradationAminobenzoate degradation

Phvul.003G214700.1	probable calcium-binding CML13	calcium ion binding		
Phvul.003G216600.1	chalcone-flavanone isomerase family	chalcone isomerase activity;flavonoid biosynthetic process	ec:5.5.1.6-isomerase	Flavonoid biosynthesis
Phvul.003G217400.1	hypothetical protein PHAVU_003G217400g	integral component of membrane		
Phvul.003G217400.2	hypothetical protein PHAVU_003G217400g	integral component of membrane		
Phvul.003G218000.1	probable transaldolase	sedoheptulose-7-phosphate:D-glyceraldehyde-3-phosphate glyceronetransferase activity;carbohydrate metabolic process	ec:2.2.1.2-dihydroxyacetonetransferase	Biosynthesis of antibiotics;Pentose phosphate pathway
Phvul.003G218500.1	NRT1 PTR FAMILY -like	plasma membrane;integral component of membrane;nitrate transmembrane transporter activity;oligopeptide transport;response to water deprivation;response to nitrate;nitrate transport;sterol biosynthetic process;glucosinolate biosynthetic process;plant-type cell wall cellulose metabolic process;cell wall pectin metabolic process		
Phvul.003G218500.2	NRT1 PTR FAMILY -like	integral component of membrane;transporter activity;oligopeptide transport		
Phvul.003G222300.1	probable glycosyltransferase At5g03795	integral component of membrane;glucuronosyl-N-acetylglucosaminyl-proteoglycan 4-alpha-N-acetylglucosaminyltransferase activity;metabolic process	ec:2.4.1.224-4-alpha-N-acetylglucosaminyltransferase;ec:2.4.1	Glycosaminoglycan biosynthesis - heparan sulfate / heparin
Phvul.003G223300.1	transmembrane ,	integral component of membrane		
Phvul.003G223400.1	dual specificity phosphatase DSP8	phosphatidylinositol-4,5-bisphosphate 5-phosphatase activity;protein tyrosine phosphatase activity;protein tyrosine/serine/threonine phosphatase activity;peptidyl-tyrosine dephosphorylation;negative regulation of MAP kinase activity	ec:3.1.3;ec:3.1.3.48;ec:3.1;ec:3.1.3.41-nitrophenyl phosphatase;ec:3.1.3.36-5-phosphatase;ec:3.1.3.16-phosphatase	Aminobenzoate degradationInositol phosphate metabolism;Phosphatidylinositol signaling systemT cell receptor signaling pathway
Phvul.003G223700.1	traB domain-containing isoform X1			
Phvul.003G224900.1	ATP-dependent Clp protease proteolytic subunit-related 4, chloroplastic-like	chloroplast stroma;chloroplast envelope;serine-type endopeptidase activity;response to reactive oxygen species;proteolysis;systemic acquired resistance;regulation of gene expression;isopentenyl diphosphate biosynthetic process, methylerythritol 4-phosphate pathway;regulation of defense response;regulation of protein localization	ec:3.4.21	
Phvul.003G225000.1	light harvesting	integral component of membrane		
Phvul.003G227400.1	lon protease homolog 2, peroxisomal-like	peroxisomal matrix;ATP-dependent peptidase activity;serine-type endopeptidase activity;ATP binding;misfolded or incompletely synthesized protein catabolic process;protein processing;protein import into peroxisome matrix, docking;growth;lateral root development	ec:3.6.1;ec:3.6.1.3-adenylpyrophosphatase;ec:3.6.1.15-phosphatase;ec:3.4.21	Purine metabolismPurine metabolism;Thiamine metabolism
Phvul.003G227700.1	invertase inhibitor	cell periphery;pectinesterase activity;pectinesterase inhibitor activity;negative regulation of catalytic activity	ec:3.1.1.11-pectin demethoxylase;ec:3.1.1.1-ali-esterase	Starch and sucrose metabolism;Pentose and glucuronate interconversionsDrug metabolism - other enzymes
Phvul.003G230000.1	eyes absent homolog 2 isoform X1	protein tyrosine phosphatase activity, metal-dependent;multicellular organismal development;peptidyl-tyrosine dephosphorylation	ec:3.1.3.48;ec:3.1;ec:3.1.3.41-nitrophenyl phosphatase;ec:3.1.3.16-phosphatase	Aminobenzoate degradationT cell receptor signaling pathway
Phvul.003G230900.1	hypothetical protein PHAVU_003G230900g	nucleic acid binding;zinc ion binding		

Phvul.003G231400.1	leucine-rich repeat receptor-like serine threonine- kinase BAM1	integral component of membrane;protein kinase activity;ATP binding;protein phosphorylation		
Phvul.003G232200.1	CROWDED NUCLEI 4-like			
Phvul.003G232200.2	CROWDED NUCLEI 4-like			
Phvul.003G232500.1	transmembrane ,	integral component of membrane		
Phvul.003G232500.2	transmembrane ,	integral component of membrane		
Phvul.003G232500.3	transmembrane ,	integral component of membrane		
Phvul.003G233200.1	basic leucine zipper 43-like	transcription factor activity, sequence-specific DNA binding;transferase activity;sequence-specific DNA binding;regulation of transcription, DNA-templated;metabolic process		
Phvul.003G233300.1	hypothetical protein PHAVU_003G233300g			
Phvul.003G233900.1	hypothetical protein PHAVU_003G233900g			
Phvul.003G234200.1	unknown			
Phvul.003G239900.1	Mortality factor 4 1	histone acetyltransferase complex;chromatin remodeling;regulation of transcription, DNA-templated;histone acetylation		
Phvul.003G240100.1	probable cadmium zinc-transporting ATPase HMA1, chloroplastic	integral component of plasma membrane;chloroplast envelope;calcium-transporting ATPase activity;ATP binding;cadmium-exporting ATPase activity;cadmium-transporting ATPase activity;zinc transporting ATPase activity;metal ion binding;cellular copper ion homeostasis;metabolic process;response to toxic substance;response to light intensity;zinc ion homeostasis;cadmium ion transmembrane transport;calcium ion transmembrane transport;zinc II ion transmembrane transport	ec:3.6.1;ec:3.6.1.3-adenylpyrophosphatase;ec:3.6.1.15-phosphatase;ec:3.6.3.8;ec:3.6.3.3;ec:3.6.3.46	Purine metabolismPurine metabolism;Thiamine metabolism
Phvul.003G240300.1	oxidoreductase TDA3	oxidoreductase activity;oxidation-reduction process		
Phvul.003G241900.1	floral homeotic APETALA 2-like isoform X1	nucleus;DNA binding;transcription factor activity, sequence-specific DNA binding;transcription, DNA-templated;regulation of transcription, DNA-templated;multicellular organismal development		
Phvul.003G242300.1	PI-PLC X domain-containing	integral component of membrane;phosphoric diester hydrolase activity;lipid metabolic process	ec:3.1	
Phvul.003G243000.1	Nicotinate phosphoribosyltransferase	nicotinate-nucleotide diphosphorylase (carboxylating) activity;nicotinate phosphoribosyltransferase activity;ligase activity;NAD biosynthetic process;nicotinate nucleotide salvage	ec:2.4.2.19-diphosphorylase (carboxylating);ec:2.4.2.11	Nicotinate and nicotinamide metabolism
Phvul.003G243400.1	triacylglycerol lipase	integral component of membrane;feruloyl esterase activity;lipid metabolic process	ec:3.1.1.73;ec:3.1.1.1-ali-esterase	Drug metabolism - other enzymes
Phvul.003G244900.1	hypothetical protein PHAVU_003G244900g	intracellular;protein glycosylation;protein targeting to vacuole;vesicle-mediated transport		
Phvul.003G246000.1	CDPK-related kinase 5-like	cytoplasm;plasma membrane;calmodulin-dependent protein kinase activity;calcium ion binding;calmodulin binding;ATP binding;calcium-dependent protein serine/threonine kinase activity;abscisic acid-activated signaling pathway;peptidyl-serine phosphorylation;intracellular signal transduction;protein autophosphorylation	ec:2.7.11;ec:2.7.11.17	

Phvul.003G248400.1	hypothetical protein PHAVU_003G248400g			
Phvul.003G248600.1	GDSL esterase lipase At2g23540	hydrolase activity, acting on ester bonds;9,9'-dicis-carotene:quinone oxidoreductase activity;7,9,9'-tricis-neurosporene:quinone oxidoreductase activity;9,9'-di-cis-zeta-carotene desaturation to 7,9,7',9'-tetra-cis-lycopene;oxidation-reduction process	ec:1.3.5.6-desaturase	Carotenoid biosynthesis
Phvul.003G249100.1	DUF1162 family	integral component of membrane		
Phvul.003G250400.1	BTB POZ domain-containing At3g50780-like	peptidase activity;proteolysis;cell division		
Phvul.003G251000.1	probable calcium-binding CML41	calcium ion binding		
Phvul.003G251300.1	elongation of fatty acids 3-like	integral component of membrane		
Phvul.003G251700.1	hypothetical protein PHAVU_003G251700g			
Phvul.003G252000.1	G-box-binding factor 1	cytoplasm;transcription factor activity, sequence-specific DNA binding;sequence-specific DNA binding;regulation of transcription, DNA-templated;regulation of hydrogen peroxide metabolic process;regulation of cell aging		
Phvul.003G252600.1	polyol transporter ,	integral component of membrane		
Phvul.003G252600.2	polyol transporter ,	integral component of membrane		
Phvul.003G254800.1	Anaphase-promoting complex subunit 10	anaphase-promoting complex;nuclear body;mitotic nuclear division;gamete generation;cell proliferation;phloem or xylem histogenesis;regulation of mitotic metaphase/anaphase transition;anaphase-promoting complex-dependent proteasomal ubiquitin-dependent protein catabolic process;negative regulation of DNA endoreduplication;DNA endoreduplication;proteasome assembly;cell division;regulation of unidimensional cell growth;response to misfolded protein		
Phvul.003G255100.1	pheophytinase, chloroplastic	chloroplast envelope;1-acylglycerol-3-phosphate O-acyltransferase activity;hydrolase activity;maltose metabolic process;starch biosynthetic process;positive regulation of catalytic activity	ec:2.3.1.51-O-acyltransferase;ec:2.3.1	Glycerophospholipid metabolism;Glycerolipid metabolism
Phvul.003G255200.1	auxilin 1	transferase activity;metabolic process		
Phvul.003G255200.2	auxilin 1	transferase activity;metabolic process		
Phvul.003G255600.1	GTPase Era-like isoform X1	chloroplast;RNA binding;GTP binding;embryo development ending in seed dormancy;iron-sulfur cluster assembly;isopentenyl diphosphate biosynthetic process, methylerythritol 4-phosphate pathway;transcription from plastid promoter;protein targeting to chloroplast;positive regulation of transcription, DNA-templated		
Phvul.003G256500.1	abscisic acid 8 -hydroxylase 3-like	integral component of membrane;iron ion binding;(+)-abscisic acid 8'-hydroxylase activity;heme binding;multicellular organismal development;brassinosteroid homeostasis;sterol metabolic process;brassinosteroid biosynthetic process;oxidation-reduction process	ec:1.14.13.93-acid 8'-hydroxylase;ec:1.14.13	Carotenoid biosynthesis
Phvul.003G257300.1	AT4G36440 , partial	integral component of membrane		
Phvul.003G258600.1	amino-terminal domain micro-spherule			

Phvul.003G260500.1	NAC domain-containing 37	nucleus;integral component of membrane;DNA binding;transcription, DNA-templated;regulation of transcription, DNA-templated		
Phvul.003G263100.1	armadillo repeat only			
Phvul.003G266900.1	probable phosphatase 2C 46	integral component of membrane;protein serine/threonine phosphatase activity;metal ion binding;protein dephosphorylation	ec:3.1;ec:3.1.3.41-nitrophenyl phosphatase;ec:3.1.3.16-phosphatase	Aminobenzoate degradationT cell receptor signaling pathway
Phvul.003G267000.1	Receptor kinase THESEUS 1	integral component of membrane;protein serine/threonine kinase activity;ATP binding;protein phosphorylation	ec:2.7.11	
Phvul.003G267600.1	UPF0548 At2g17695	chloroplast envelope		
Phvul.003G267600.2	UPF0548 At2g17695	chloroplast envelope		
Phvul.003G268400.1	DUF241 domain			
Phvul.003G268600.1	Chitinase (Class Ib) Hevein	chitinase activity;chitin binding;polysaccharide catabolic process;chitin catabolic process;defense response;cell wall macromolecule catabolic process	ec:3.2.1.14-chitodextrinase	Amino sugar and nucleotide sugar metabolism
Phvul.003G269600.1	dehydrodolichyl diphosphate synthase 6-like	di-trans,poly-cis-decaprenylcistransferase activity;metabolic process	ec:2.5.1.31-synthase [(2E,6E)-farnesyl-diphosphate specific];ec:2.5.1	Terpenoid backbone biosynthesis
Phvul.003G270100.1	Syntaxin-binding 5	cytoplasm;plasma membrane;GTPase activator activity;Rab GTPase binding;syntaxin binding;regulation of exocytosis;positive regulation of GTPase activity		
Phvul.003G270100.2	Syntaxin-binding 5	cytoplasm;plasma membrane;GTPase activator activity;Rab GTPase binding;syntaxin binding;regulation of exocytosis;positive regulation of GTPase activity		
Phvul.003G272800.1	transmembrane 45B-like	integral component of membrane		
Phvul.003G274200.1	UPF0664 stress-induced	plasma membrane		
Phvul.003G274200.2	UPF0664 stress-induced	plasma membrane		
Phvul.003G274900.1	macronuclear actin	structural constituent of cytoskeleton;actin filament-based process		
Phvul.003G275200.1	glycosyltransferase family 92	integral component of membrane;transferase activity;metabolic process		
Phvul.003G275500.1	serine threonine-kinase minibrain-like isoform X1	cytosol;plasmodesma;protein serine/threonine/tyrosine kinase activity;ATP binding;protein phosphorylation;response to abscisic acid	ec:2.7.12.1	
Phvul.003G276200.1	inactive RESTRICTED TEV MOVEMENT 2-like	integral component of membrane		
Phvul.003G277700.1	E3 ubiquitin- ligase MBR1-like isoform X1	zinc ion binding;ligase activity;metabolic process		
Phvul.003G278500.1	arabinosyltransferase XEG113-like	integral component of membrane;microtubule-severing ATPase activity;arabinosyltransferase activity;metabolic process	ec:3.6.1;ec:3.6.1.3-adenylpyrophosphatase;ec:3.6.4.3;ec:3.6.1.15-phosphatase	Purine metabolismPurine metabolism;Thiamine metabolism
Phvul.003G279000.1	probable apyrase 7	integral component of membrane;hydrolase activity;multicellular organismal development;metabolic process;anatomical structure development		
Phvul.003G279000.2	probable apyrase 7	integral component of membrane;hydrolase activity;multicellular organismal development;metabolic process;anatomical structure development		
Phvul.003G280000.1	probable methyltransferase PMT20	endosome;trans-Golgi network;integral component of membrane;S-adenosylmethionine-dependent methyltransferase activity;methylation		
Phvul.003G280200.1	hypothetical protein PHAVU_003G280200g			

Phvul.003G280700.1	translin-associated X	sequence-specific DNA binding		
Phvul.003G281100.1	ASC1	endoplasmic reticulum;integral component of membrane;sphingosine N-acyltransferase activity;ceramide biosynthetic process	ec:2.3.1.24-N-acyltransferase;ec:2.3.1	Sphingolipid metabolism
Phvul.003G282200.1	histone-lysine N-methyltransferase family member SUVH9-like	nucleus;chromosome;zinc ion binding;histone-lysine N-methyltransferase activity;histone binding;histone lysine methylation	ec:2.1.1;ec:2.1.1.43-N-methyltransferase	Lysine degradation
Phvul.003G282900.1	nucleolin 1-like isoform X1	nucleotide binding;nucleic acid binding		
Phvul.003G282900.2	nucleolin 1-like isoform X1	nucleotide binding;nucleic acid binding		
Phvul.003G282900.3	nucleolin 1-like isoform X1	nucleotide binding;nucleic acid binding		
Phvul.003G282900.4	nucleolin 1-like isoform X1	nucleotide binding;nucleic acid binding		
Phvul.003G283900.1	ABC transporter B family member 2-like	integral component of membrane;ATP binding;ATPase activity, coupled to transmembrane movement of substances;metabolic process;transmembrane transport	ec:3.6.1;ec:3.6.1.3-adenylpyrophosphatase;ec:3.6.1.15-phosphatase	Purine metabolismPurine metabolism;Thiamine metabolism
Phvul.003G284800.1	PLATZ transcription factor family	integral component of membrane		
Phvul.003G285900.1	phosphoenolpyruvate carboxykinase [ATP]-like	cytosol;phosphoenolpyruvate carboxykinase (ATP) activity;ATP binding;kinase activity;gluconeogenesis;phosphorylation	ec:4.1.1;ec:4.1.1.49-carboxykinase (ATP)	Pyruvate metabolism;Biosynthesis of antibiotics;Carbon fixation in photosynthetic organisms;Citrate cycle (TCA cycle);Glycolysis / Gluconeogenesis
Phvul.003G290500.1	guanylate-binding family	GTPase activity;GTP binding;metabolic process	ec:3.6.1;ec:3.6.1.15-phosphatase	Purine metabolism;Thiamine metabolism
Phvul.003G290700.1	PLASMODESMATA CALLOSE-BINDING PROTEIN 3-like	integral component of membrane;protein histidine kinase activity;hydrolase activity;peptidyl-histidine phosphorylation	ec:2.7.13.3	
Phvul.003G292000.1	glyco 3-alpha-L-fucosyltransferase A	integral component of membrane;Golgi cisterna membrane;glycoprotein 3-alpha-L-fucosyltransferase activity;protein glycosylation;fucosylation	ec:2.4.1;ec:2.4.1.214-3-alpha-L-fucosyltransferase	Various types of N-glycan biosynthesis
Phvul.003G293100.1	pentatricopeptide repeat-containing At2g13600-like	microtubule-severing ATPase activity;metabolic process	ec:3.6.1;ec:3.6.1.3-adenylpyrophosphatase;ec:3.6.4.3;ec:3.6.1.15-phosphatase	Purine metabolismPurine metabolism;Thiamine metabolism
Phvul.003G295800.1	Autophagy-related 2	pre-autophagosomal structure;extrinsic component of membrane;autophagosome assembly;mitophagy;nucleophagy		
Phvul.003G296100.1	cell division cycle-associated 7			
Phvul.003G296300.1	Sas10 U3 ribonucleo (UTP) family ,	nucleolus;small-subunit processome;maturation of SSU-rRNA from tricistronic rRNA transcript (SSU-rRNA, 5.8S rRNA, LSU-rRNA)		
Phvul.003G296900.1	hypothetical protein PHAVU_003G296900g			
Phvul.004G004800.1	serine threonine-kinase HT1	intracellular;MAP kinase kinase kinase activity;protein serine/threonine/tyrosine kinase activity;ATP binding;MAPK cascade;activation of MAPKK activity;microtubule nucleation	ec:2.7.11;ec:2.7.11.25;ec:2.7.12.1	
Phvul.004G007700.1	NBS-LRR type disease resistance CNL-J9	ADP binding;defense response		
Phvul.004G015600.1	NBS-LRR type disease resistance CNL-B11	ADP binding;defense response		
Phvul.004G017600.1	Methyl- -binding domain-containing 9	nuclear heterochromatin;DNA binding;zinc ion binding;oxidoreductase activity;ubiquitin protein ligase activity;anatomical structure morphogenesis;maintenance of DNA methylation;vegetative to reproductive phase		

		transition of meristem;protein ubiquitination;histone acetylation;cell development;oxidation-reduction process;regulation of methylation-dependent chromatin silencing;plant epidermis development		
Phvul.004G017900.1	pentatricopeptide repeat-containing At3g61360			
Phvul.004G018700.1	Auxin-induced 5NG4	plasma membrane;integral component of membrane;transmembrane transporter activity;transmembrane transport		
Phvul.004G018900.1	alpha-dioxygenase 1-like	peroxidase activity;heme binding;dioxygenase activity;response to oxidative stress;oxidation-reduction process;cellular oxidant detoxification	ec:1.11.1.7-lactoperoxidase	Phenylpropanoid biosynthesis
Phvul.004G022600.1	Hop-interacting THI043	cellular carbohydrate metabolic process;single-organism carbohydrate metabolic process;single-organism cellular process		
Phvul.004G023100.1	ABC transporter B family member 15-like	integral component of membrane;ATP binding;ATPase activity, coupled to transmembrane movement of substances;metabolic process;transmembrane transport	ec:3.6.1;ec:3.6.1.3-adenylpyrophosphatase;ec:3.6.1.15-phosphatase	Purine metabolismPurine metabolism;Thiamine metabolism
Phvul.004G023500.1	glucose-6-phosphate isomerase, cytosolic	cytosol;glucose-6-phosphate isomerase activity;gluconeogenesis;glycolytic process;response to sucrose;flavonoid biosynthetic process;defense response to fungus, incompatible interaction;response to UV-B	ec:5.3.1.9-isomerase	Biosynthesis of antibiotics;Pentose phosphate pathway;Amino sugar and nucleotide sugar metabolism;Glycolysis / Gluconeogenesis;Starch and sucrose metabolism
Phvul.004G023500.2	glucose-6-phosphate isomerase, cytosolic	cytosol;glucose-6-phosphate isomerase activity;gluconeogenesis;glycolytic process;response to sucrose;flavonoid biosynthetic process;defense response to fungus, incompatible interaction;response to UV-B	ec:5.3.1.9-isomerase	Biosynthesis of antibiotics;Pentose phosphate pathway;Amino sugar and nucleotide sugar metabolism;Glycolysis / Gluconeogenesis;Starch and sucrose metabolism
Phvul.004G023900.1	rRNA methyltransferase ylbH	methyltransferase activity;tRNA metabolic process;chloroplast organization;embryo development ending in seed dormancy;production of ta-siRNAs involved in RNA interference;iron-sulfur cluster assembly;rRNA methylation;production of miRNAs involved in gene silencing by miRNA;transcription from plastid promoter;positive regulation of transcription, DNA-templated;defense response to virus		
Phvul.004G025000.1	probable mitochondrial chaperone BCS1-B	integral component of membrane;ATP binding;hydrolase activity;metabolic process		
Phvul.004G026100.2	hypersensitive-induced response 2	vacuolar membrane;plasmodesma;chloroplast;protein histidine kinase binding		
Phvul.004G027800.1	ABC transporter B family member 19	integral component of membrane;ATP binding;xenobiotic-transporting ATPase activity;auxin efflux transmembrane transporter activity;polysaccharide biosynthetic process;drug transmembrane transport;regulation of cell size;response to blue light;photomorphogenesis;response to auxin;multidimensional cell growth;cell tip growth;positive gravitropism;vernalization response;formation of organ boundary;response to far red light;auxin efflux;basipetal auxin transport;acropetal auxin transport;xenobiotic transport;anthocyanin accumulation in tissues in response to UV light;carpel development;stamen development;lateral root development;root hair elongation;cell wall organization	ec:3.6.1;ec:3.6.1.3-adenylpyrophosphatase;ec:3.6.1.15-phosphatase;ec:3.6.3.44	Purine metabolismPurine metabolism;Thiamine metabolism

Phvul.004G031600.2	vesicle transport v-SNARE 13	Golgi apparatus;integral component of membrane;SNARE binding;SNAP receptor activity;intracellular protein transport;vesicle-mediated transport;membrane fusion		
Phvul.004G032000.1	SNF1-related kinase catalytic subunit alpha KIN10	cytoplasm;cAMP-dependent protein kinase activity;ATP binding;protein phosphorylation;intracellular signal transduction	ec:2.7.11;ec:2.7.11.11-protein kinase	mTOR signaling pathway
Phvul.004G033900.1	hypothetical protein PHAVU_004G033900g			
Phvul.004G037700.1	transcription factor TCP13	single-organism process		
Phvul.004G037700.2	transcription factor TCP13	single-organism process		
Phvul.004G044300.1	AF306499_1NBS-LRR resistance B8	ADP binding;defense response		
Phvul.004G044900.1	LRR receptor-like kinase family	integral component of membrane		
Phvul.004G045400.1	ATP sulfurylase 2	cytosol;adenylylsulfate kinase activity;sulfate adenylyltransferase (ATP) activity;sulfate assimilation;phosphorylation	ec:2.7.1.25-kinase;ec:2.7.7.4-adenylyltransferase;ec:2.7.7	Sulfur metabolism;Purine metabolismSulfur metabolism;Purine metabolism;Biosynthesis of antibiotics;Monobactam biosynthesis;Selenocompound metabolism
Phvul.004G046100.1	6-phosphogluconate dehydrogenase, decarboxylating 3	phosphogluconate dehydrogenase (decarboxylating) activity;pentose-phosphate shunt;oxidation-reduction process	ec:1.1.1.44-dehydrogenase (NADP+-dependent, decarboxylating)	Biosynthesis of antibiotics;Pentose phosphate pathway;Glutathione metabolism
Phvul.004G046600.1	zinc finger CONSTANS-LIKE 2	nucleus;zinc ion binding;response to light stimulus;regulation of flower development		
Phvul.004G048000.1	NBS-LRR type disease resistance	ADP binding;defense response		
Phvul.004G048300.1	tryptophan synthase beta chain 2	tryptophan synthase activity;pyridoxal phosphate binding;protein homodimerization activity;L-serine hydro-lyase (adding indole, L-tryptophan-forming) activity;tryptophan biosynthetic process	ec:4.2.1;ec:4.2.1.20-synthase	Biosynthesis of antibiotics;Phenylalanine, tyrosine and tryptophan biosynthesis;Glycine, serine and threonine metabolism
Phvul.004G048500.1	zinc finger, C3HC4 type (RING finger) ,	zinc ion binding;petal formation;sepal formation		
Phvul.004G048900.1	trichome birefringence-like 19	Golgi apparatus;integral component of membrane;O-acetyltransferase activity;cell wall organization or biogenesis	ec:2.3.1	
Phvul.004G049300.1	endo-1,3(4)-beta-glucanase 2	cell surface;glucan endo-1,3-beta-D-glucosidase activity;glucan endo-1,3-beta-glucanase activity, C-3 substituted reducing group;glucan endo-1,4-beta-glucanase activity, C-3 substituted reducing group;(1->3)-beta-D-glucan catabolic process	ec:3.2.1.39-endo-1,3-beta-D-glucosidase;ec:3.2.1;ec:3.2.1.21-gentiobiase;ec:3.2.1.6	Starch and sucrose metabolismStarch and sucrose metabolism;Cyanoamino acid metabolism;Phenylpropanoid biosynthesis
Phvul.004G050900.1	membrane steroid-binding 2-like	endomembrane system;integral component of membrane;dihydrofolate reductase activity;heme binding;oxidation-reduction process	ec:1.5.1.3-reductase	One carbon pool by folate;Folate biosynthesis
Phvul.004G052800.1	bystin-like			
Phvul.004G052900.1	UBP1-associated 2A-like	nucleotide binding;nucleic acid binding		
Phvul.004G054400.1	SWI SNF complex component SNF12 homolog			
Phvul.004G054400.2	SWI SNF complex component SNF12 homolog			
Phvul.004G058100.1	TPX2 (targeting for Xklp2) family ,			

Phvul.004G058900.1	uncharacterized membrane At3g27390-like	integral component of membrane		
Phvul.004G059400.1	Mitochondrial inner membrane protease ATP23	metalloendopeptidase activity;proteolysis	ec:3.4.24	
Phvul.004G060400.1	pentatricopeptide repeat-containing At4g35850, mitochondrial	pyrimidine ribonucleotide biosynthetic process;photomorphogenesis;regulation of flower development;cullin deneddylation;histone lysine methylation		
Phvul.004G064300.1	ultraviolet-B receptor UVR8	ligase activity;protein domain specific binding;metabolic process		
Phvul.004G064500.1	probable dolichyl pyrophosphate Man9 c2 alpha-1,3-glucosyltransferase	endoplasmic reticulum membrane;integral component of membrane;dolichyl pyrophosphate Man9GlcNAc2 alpha-1,3-glucosyltransferase activity;protein N-linked glycosylation;oligosaccharide-lipid intermediate biosynthetic process	ec:2.4.1	
Phvul.004G065200.1	hypothetical protein PHAVU_004G065200g			
Phvul.004G065300.1	phosphoglycerate kinase, cytosolic-like isoform X1	phosphoglycerate kinase activity;tRNA (guanine-N7-)-methyltransferase activity;glycolytic process;tRNA modification;RNA (guanine-N7)-methylation	ec:2.1.1;ec:2.1.1.33;ec:2.7.2.3-kinase	Biosynthesis of antibiotics;Carbon fixation in photosynthetic organisms;Glycolysis / Gluconeogenesis
Phvul.004G065300.4	Phosphoglycerate kinase	phosphoglycerate kinase activity;tRNA (guanine-N7-)-methyltransferase activity;glycolytic process;tRNA modification;RNA (guanine-N7)-methylation	ec:2.1.1;ec:2.1.1.33;ec:2.7.2.3-kinase	Biosynthesis of antibiotics;Carbon fixation in photosynthetic organisms;Glycolysis / Gluconeogenesis
Phvul.004G065300.6	phosphoglycerate kinase, cytosolic-like isoform X1	phosphoglycerate kinase activity;tRNA (guanine-N7-)-methyltransferase activity;glycolytic process;tRNA modification;RNA (guanine-N7)-methylation	ec:2.1.1;ec:2.1.1.33;ec:2.7.2.3-kinase	Biosynthesis of antibiotics;Carbon fixation in photosynthetic organisms;Glycolysis / Gluconeogenesis
Phvul.004G065600.1	enhancer of polycomb homolog 2-like	Piccolo NuA4 histone acetyltransferase complex;transcription, DNA-templated;regulation of transcription from RNA polymerase II promoter		
Phvul.004G065600.2	enhancer of polycomb homolog 2-like	Piccolo NuA4 histone acetyltransferase complex;transcription, DNA-templated;regulation of transcription from RNA polymerase II promoter		
Phvul.004G065900.1	neoxanthin synthase	integral component of membrane		
Phvul.004G067500.1	INO80 complex subunit D-like	histone acetyltransferase complex;H4 histone acetyltransferase activity;histone H4 acetylation	ec:2.3.1.48;ec:2.3.1	
Phvul.004G068300.1	cytochrome P450 81E8-like	integral component of membrane;monooxygenase activity;iron ion binding;oxidoreductase activity, acting on paired donors, with incorporation or reduction of molecular oxygen;heme binding;oxidation-reduction process		
Phvul.004G068400.1	cytochrome P450 81E8-like	integral component of membrane;monooxygenase activity;iron ion binding;oxidoreductase activity, acting on paired donors, with incorporation or reduction of molecular oxygen;heme binding;oxidation-reduction process		
Phvul.004G068600.1	DUF247 domain	integral component of membrane		
Phvul.004G075000.1	transmembrane ,	mitochondrion;integral component of membrane;RNA methylation		
Phvul.004G075300.1	IQ-DOMAIN 1-like isoform X1	integral component of membrane		
Phvul.004G075300.2	IQ-DOMAIN 1-like isoform X1	integral component of membrane		
Phvul.004G075300.3	IQ-DOMAIN 1-like isoform X1	integral component of membrane		

Phvul.004G080300.1	YTH domain family 1			
Phvul.004G085100.1	sucrose transport SUC8-like	integral component of plasma membrane;sucrose transmembrane transporter activity;sucrose transport		
Phvul.004G085700.1	MATE efflux family 5	integral component of membrane;drug transmembrane transporter activity;antiporter activity;drug transmembrane transport;fatty acid catabolic process		
Phvul.004G090900.1	cell division cycle 5 isoform X2	chloroplast;DNA binding;transcription factor activity, sequence-specific DNA binding;mitotic cell cycle;regulation of transcription, DNA-templated;RNA processing;defense response signaling pathway, resistance gene-dependent;defense response signaling pathway, resistance gene-independent;defense response to bacterium;defense response to fungus;cell division		
Phvul.004G091600.1	LHCII type I chlorophyll a b-binding	photosystem I;photosystem II;chloroplast thylakoid membrane;chloroplast envelope;plastoglobule;integral component of membrane;chlorophyll binding;pigment binding;metal ion binding;response to light stimulus;photosynthesis, light harvesting in photosystem I;protein-chromophore linkage		
Phvul.004G098300.1	hypothetical protein PHAVU_004G0983001g, partial	structural constituent of cell wall;plant-type cell wall organization		
Phvul.004G102700.1	probable membrane-associated kinase regulator 6	kinase activity;response to brassinosteroid;phosphorylation		
Phvul.004G102900.1	P-loop nucleoside triphosphate hydrolase superfamily	ATP binding;microtubule-severing ATPase activity;metabolic process	ec:3.6.1;ec:3.6.1.3-adenylpyrophosphatase;ec:3.6.4.3;ec:3.6.1.15-phosphatase	Purine metabolismPurine metabolism;Thiamine metabolism
Phvul.004G104900.1	receptor 12	integral component of membrane		
Phvul.004G112000.1	mitogen-activated kinase kinase kinase YODA-like	cytoplasm;protein serine/threonine kinase activity;ATP binding;regulation of mitotic cell cycle;signal transduction by protein phosphorylation;stress-activated protein kinase signaling cascade;activation of protein kinase activity;regulation of apoptotic process	ec:2.7.11	
Phvul.004G113900.1	hypothetical protein PHAVU_004G113900g	integral component of membrane		
Phvul.004G113900.2	hypothetical protein PHAVU_004G113900g	integral component of membrane		
Phvul.004G113900.3	hypothetical protein PHAVU_004G113900g	integral component of membrane		
Phvul.004G113900.4	hypothetical protein PHAVU_004G113900g	integral component of membrane		
Phvul.004G117900.1	Methyl- -binding domain-containing 13	nucleus;methyl-CpG binding;negative regulation of transcription from RNA polymerase II promoter;methylation-dependent chromatin silencing		
Phvul.004G118600.1	phosphatase inhibitor 2 isoform X2	protein phosphatase type 1 complex;protein phosphatase inhibitor activity;regulation of signal transduction;negative regulation of catalytic activity;regulation of phosphoprotein phosphatase activity		
Phvul.004G118600.2	phosphatase inhibitor 2 isoform X2	protein phosphatase type 1 complex;protein phosphatase inhibitor activity;regulation of signal transduction;negative regulation of catalytic activity;regulation of phosphoprotein phosphatase activity		

Phvul.004G118600.3	phosphatase inhibitor 2 isoform X2	protein phosphatase type 1 complex;protein phosphatase inhibitor activity;regulation of signal transduction;negative regulation of catalytic activity;regulation of phosphoprotein phosphatase activity		
Phvul.004G120400.1	RHOMBOID 2	integral component of membrane;serine-type endopeptidase activity;protein processing	ec:3.4.21	
Phvul.004G121900.1	DETOXIFICATION 49-like	integral component of membrane;drug transmembrane transporter activity;antiporter activity;drug transmembrane transport		
Phvul.004G123200.1	DUF4378 domain			
Phvul.004G128900.1	Fructose-1,6-bisphosphatase, cytosolic	nucleus;cytosol;integral component of membrane;fructose 1,6-bisphosphate 1-phosphatase activity;starch catabolic process;sucrose biosynthetic process;response to abscisic acid;response to fructose;photosynthesis;dephosphorylation;fructose 1,6-bisphosphate metabolic process	ec:3.1;ec:3.1.3.41-nitrophenyl phosphatase;ec:3.1.3.23;ec:3.1.3.11-hexose diphosphatase	Aminobenzoate degradationBiosynthesis of antibiotics;Pentose phosphate pathway;Methane metabolism;Carbon fixation in photosynthetic organisms;Glycolysis / Gluconeogenesis;Fructose and mannose metabolism
Phvul.004G129100.1	DNAJ heat shock N-terminal domain-containing , isoform 1			
Phvul.004G131400.1	clathrin assembly At1g33340	clathrin-coated vesicle;1-phosphatidylinositol binding;clathrin binding;clathrin coat assembly		
Phvul.004G132600.1	hypothetical protein PHAVU_004G132600g			
Phvul.004G133200.1	DNA (cytosine-5)-methyltransferase 1-like	integral component of membrane;chromatin binding;DNA (cytosine-5-)-methyltransferase activity;transferase activity, transferring acyl groups;C-5 methylation of cytosine	ec:2.1.1;ec:2.1.1.37-(cytosine-5-)-methyltransferase	Cysteine and methionine metabolism
Phvul.004G133400.1	chloride channel CLC-c-like isoform X1	integral component of membrane;voltage-gated chloride channel activity;chloride transport;ion transmembrane transport;regulation of anion transmembrane transport		
Phvul.004G134300.1	TMV resistance N-like	ADP binding;defense response;signal transduction		
Phvul.004G134800.1	UPF0481 At3g02645	integral component of membrane		
Phvul.004G135100.1	disease resistance (TIR-NBS-LRR class),	ADP binding;defense response;signal transduction		
Phvul.004G136400.1	hypothetical protein PHAVU_004G136400g	nucleus		
Phvul.004G136500.1	receptor kinase HSL1	integral component of membrane;protein serine/threonine kinase activity;ATP binding;protein phosphorylation	ec:2.7.11	
Phvul.004G136900.1	hypothetical protein PHAVU_004G136900g			
Phvul.004G139400.1	dolichyl-diphosphooligosaccharide--glycosyltransferase subunit STT3B	plasma membrane;oligosaccharyltransferase complex;integral component of membrane;dolichyl-diphosphooligosaccharide-protein glycotransferase activity;protein N-linked glycosylation via asparagine;post-translational protein modification	ec:2.4.1;ec:2.4.1.119	
Phvul.004G139700.1	TMV resistance N-like	integral component of membrane;ATP binding;ADP binding;defense response;signal transduction		
Phvul.004G139800.1	TMV resistance N-like	integral component of membrane;ATP binding;ADP binding;defense response;signal transduction		
Phvul.004G139800.2	TMV resistance N-like isoform X2	integral component of membrane;ATP binding;hydrolase activity;ADP binding;defense		

		response;signal transduction;RNA phosphodiester bond hydrolysis		
Phvul.004G139800.3	TMV resistance N	integral component of membrane;ATP binding;hydrolase activity;ADP binding;defense response;signal transduction;RNA phosphodiester bond hydrolysis		
Phvul.004G140300.1	hypothetical protein PHAVU_004G140300g			
Phvul.004G142300.1	nucleotide-diphospho-sugar transferase family	integral component of membrane;transferase activity;metabolic process		
Phvul.004G142900.1	argonaute 1	translation initiation factor activity;transferase activity;translational initiation		
Phvul.004G144200.1	Cyclic nucleotide-binding transporter 1 isoform 1	integral component of membrane;voltage-gated potassium channel activity;regulation of membrane potential;potassium ion transmembrane transport		
Phvul.004G144800.1	#NOME?	endoplasmic reticulum membrane;integral component of membrane;protein C-terminal S-isoprenylcysteine carboxyl O-methyltransferase activity;C-terminal protein methylation	ec:2.1.1;ec:2.1.1.100-O-methyltransferase	Biosynthesis of antibiotics;Terpenoid backbone biosynthesis
Phvul.004G144800.2	#NOME?	endoplasmic reticulum membrane;integral component of membrane;protein C-terminal S-isoprenylcysteine carboxyl O-methyltransferase activity;C-terminal protein methylation	ec:2.1.1;ec:2.1.1.100-O-methyltransferase	Biosynthesis of antibiotics;Terpenoid backbone biosynthesis
Phvul.004G145300.1	carbohydrate-binding of the ER	kinase activity;phosphorylation		
Phvul.004G145900.1	SHOOT GRAVITROPISM 5-like	nucleic acid binding;metal ion binding		
Phvul.004G147000.1	transcription termination factor family	mitochondrion;double-stranded DNA binding;regulation of transcription, DNA-templated;response to salt stress;chloroplast organization;response to abscisic acid		
Phvul.004G147000.2	transcription termination factor family	mitochondrion;double-stranded DNA binding;regulation of transcription, DNA-templated;response to salt stress;chloroplast organization;response to abscisic acid		
Phvul.004G147700.1	PPR containing plant			
Phvul.004G148200.1	Ferrochelatase-2, chloroplastic	intracellular;integral component of membrane;ferrochelatase activity;protein targeting to membrane;heme biosynthetic process;response to wounding;regulation of plant-type hypersensitive response;methylglyoxal catabolic process to D-lactate via S-lactoyl-glutathione;endoplasmic reticulum unfolded protein response;negative regulation of programmed cell death;defense response by callose deposition	ec:4.99.1.1-ferro-protoporphyrin chelatase	Porphyrin and chlorophyll metabolism
Phvul.004G149400.1	Down syndrome critical region 3 homolog	protein transporter activity;protein transport;photoperiodism, flowering		
Phvul.004G149800.1	Polypeptide deformylase, isoform 1	chloroplast;iron ion binding;peptide deformylase activity;translation;N-terminal protein amino acid modification;co-translational protein modification	ec:3.5.1.88	
Phvul.004G149800.2	Polypeptide deformylase, isoform 1	chloroplast;iron ion binding;peptide deformylase activity;translation;N-terminal protein amino acid modification;co-translational protein modification	ec:3.5.1.88	
Phvul.004G150100.1	Ketose-bisphosphate aldolase class-II family isoform 1	phosphogluconate dehydrogenase (decarboxylating) activity;zinc ion binding;aldehyde-lyase activity;NAD binding;carbohydrate metabolic process;oxidation-reduction process	ec:1.1.1.44-dehydrogenase (NADP+-dependent, decarboxylating)	Biosynthesis of antibiotics;Pentose phosphate pathway;Glutathione metabolism
Phvul.004G150700.1	photosystem I assembly ycf3 (chloroplast)	chloroplast thylakoid membrane;photosynthesis		
Phvul.004G151100.1	zinc finger VAR3, chloroplastic	DNA-directed DNA polymerase activity;zinc ion binding;DNA biosynthetic process	ec:2.7.7.7-DNA polymerase	Purine metabolism;Pyrimidine metabolism

Phvul.004G151700.1	exonuclease 1	nucleus;DNA binding;exonuclease activity;DNA repair;nucleic acid phosphodiester bond hydrolysis	ec:3.1	
Phvul.004G155500.1	glycerophosphodiester phosphodiesterase kinase domain-containing GDPDL2-like	integral component of membrane;protein serine/threonine kinase activity;transmembrane receptor protein tyrosine kinase activity;ATP binding;peptidyl-tyrosine phosphorylation	ec:2.7.11;ec:2.7.10;ec:2.7.10.1	
Phvul.004G155900.1	SAR DEFICIENT 1-like	calmodulin binding;response to stress		
Phvul.004G156400.1	plant synaptotagmin	endoplasmic reticulum;integral component of membrane		
Phvul.004G159200.2	aspartic ase Asp1	integral component of membrane;aspartic-type endopeptidase activity;transferase activity;N-terminal protein myristoylation;proteolysis;protein catabolic process	ec:3.4.23	
Phvul.004G160000.1	probable 3-beta-hydroxysteroid-Delta(8),Delta(7)-isomerase	intracellular;plasma membrane;integral component of membrane;C-8 sterol isomerase activity;cholestenol delta-isomerase activity;acetyl-CoA metabolic process;sterol metabolic process;brassinosteroid biosynthetic process;second-messenger-mediated signaling;regulation of gene silencing by miRNA	ec:5.3.3.5-Delta-isomerase;ec:5.3.3	Steroid biosynthesis
Phvul.004G162300.1	Aldehyde dehydrogenase family 2 member C4	retinal dehydrogenase activity;aldehyde dehydrogenase (NAD) activity;coniferyl-aldehyde dehydrogenase activity;systemic acquired resistance;phenylpropanoid biosynthetic process;response to nitrate;nitrate transport;response to endoplasmic reticulum stress;oxidation-reduction process	ec:1.2.1.68-dehydrogenase;ec:1.2.1.36-dehydrogenase;ec:1.2.1.5-dehydrogenase [NAD(P)+];ec:1.2.1.3-dehydrogenase (NAD+)	Phenylpropanoid biosynthesisRetinol metabolismDrug metabolism - cytochrome P450;Metabolism of xenobiotics by cytochrome P450;beta-Alanine metabolism;Glycolysis / Gluconeogenesis;Phenylalanine metabolism;Tyrosine metabolism;Histidine metabolismArginine and proline metabolism;Chloroalkane and chloroalkene degradation;Pyruvate metabolism;Biosynthesis of antibiotics;Tryptophan metabolism;Valine, leucine and isoleucine degradation;Limonene and pinene degradation;beta-Alanine metabolism;Lysine degradation;Glycolysis / Gluconeogenesis;Fatty acid degradation;Glycerolipid metabolism;Ascorbate and aldarate metabolism;Histidine metabolism;Pentose and glucuronate interconversions
Phvul.004G164500.1	subtilisin-like protease	cell wall;Golgi apparatus;integral component of membrane;serine-type endopeptidase activity;protein glycosylation;proteolysis;ER to Golgi vesicle-mediated transport;hyperosmotic salinity response	ec:3.4.21	
Phvul.004G164500.2	subtilisin-like protease	cell wall;Golgi apparatus;integral component of membrane;serine-type endopeptidase activity;protein glycosylation;proteolysis;ER to Golgi vesicle-mediated transport;hyperosmotic salinity response	ec:3.4.21	

Phvul.004G166900.1	hypothetical protein PHAVU_004G166900g			
Phvul.004G168100.1	Elongation factor Ts	mitochondrial matrix;chloroplast part;translation elongation factor activity;isoprenoid biosynthetic process;plastid organization;mitochondrial translational elongation		
Phvul.004G169700.1	probable glycosyltransferase At3g07620	integral component of membrane;glucuronosyl-N-acetylglucosaminyl-proteoglycan 4-alpha-N-acetylglucosaminyltransferase activity;metabolic process	ec:2.4.1.224-4-alpha-N-acetylglucosaminyltransferase;ec:2.4.1	Glycosaminoglycan biosynthesis - heparan sulfate / heparin
Phvul.004G170500.1	senescence dehydration-associated			
Phvul.004G171300.1	tetraspanin family	integral component of membrane		
Phvul.004G172000.1	hypothetical protein PHAVU_004G172000g			
Phvul.004G173600.1	zinc finger CCCH domain-containing 20-like	metal ion binding		
Phvul.004G173700.1	gamma-glutamyltranspeptidase 3	integral component of membrane;gamma-glutamyltransferase activity;glutathione gamma-glutamylcysteinyltransferase activity;glutathione catabolic process	ec:2.3.2.2-glutamyl transpeptidase;ec:2.3.2.15	Taurine and hypotaurine metabolism;Glutathione metabolism;Cyanoamino acid metabolism
Phvul.004G173700.2	gamma-glutamyltranspeptidase 3	integral component of membrane;gamma-glutamyltransferase activity;glutathione gamma-glutamylcysteinyltransferase activity;glutathione catabolic process	ec:2.3.2.2-glutamyl transpeptidase;ec:2.3.2.15	Taurine and hypotaurine metabolism;Glutathione metabolism;Cyanoamino acid metabolism
Phvul.004G173700.3	gamma-glutamyltranspeptidase 3	integral component of membrane;gamma-glutamyltransferase activity;glutathione gamma-glutamylcysteinyltransferase activity;glutathione catabolic process	ec:2.3.2.2-glutamyl transpeptidase;ec:2.3.2.15	Taurine and hypotaurine metabolism;Glutathione metabolism;Cyanoamino acid metabolism
Phvul.004G174000.1	NAC domain-containing 73	nucleus;DNA binding;transcription, DNA-templated;regulation of transcription, DNA-templated		
Phvul.004G174000.2	NAC domain-containing 73	nucleus;DNA binding;transcription, DNA-templated;regulation of transcription, DNA-templated		
Phvul.004G174000.3	NAC domain-containing 73	nucleus;DNA binding;transcription, DNA-templated;regulation of transcription, DNA-templated		
Phvul.004G174700.1	arginine--tRNA ligase, cytoplasmic-like	cytoplasm;arginine-tRNA ligase activity;ATP binding;arginyl-tRNA aminoacylation	ec:6.1.1;ec:6.1.1.19-ligase	Aminoacyl-tRNA biosynthesis
Phvul.004G175000.1	DNA-directed RNA polymerase III subunit RPC2	DNA-directed RNA polymerase III complex;integral component of membrane;RNA polymerase III activity;DNA binding;ribonucleoside binding;transcription from RNA polymerase III promoter	ec:2.7.7.6-RNA polymerase	Purine metabolism;Pyrimidine metabolism
Phvul.004G175100.1	phosphoinositide 3-kinase regulatory subunit 4	nuclear pore;late endosome;phosphatidylinositol 3-kinase complex, class III, type I;phosphatidylinositol 3-kinase complex, class III, type II;nucleus-vacuole junction;Cul4-RING E3 ubiquitin ligase complex;protein serine/threonine kinase activity;ATP binding;response to superoxide;protein phosphorylation;protein targeting to vacuole;fatty acid beta-oxidation;lipid transport;intra-Golgi vesicle-mediated transport;cell death;pollen development;gravitropism;pollen germination;salicylic acid mediated signaling pathway;ethylene-activated signaling	ec:2.7.11	

		pathway;lithium ion transport;macroautophagy;protein import into peroxisome matrix;pexophagy;cellular macromolecule catabolic process;late endosome to vacuole transport;photoperiodism, flowering		
Phvul.004G175700.1	Receptor kinase HSL1	integral component of membrane;protein serine/threonine kinase activity;ATP binding;protein phosphorylation	ec:2.7.11	
Phvul.004G176100.1	DNA (cytosine-5)-methyltransferase CMT3	nucleus;chromatin binding;DNA (cytosine-5-)-methyltransferase activity;DNA replication initiation;regulation of DNA replication;methylation-dependent chromatin silencing;cell proliferation;regulation of flower development;zygote asymmetric cytokinesis in embryo sac;DNA methylation on cytosine within a CNG sequence;histone phosphorylation;chromatin silencing by small RNA;histone H3-K9 methylation;regulation of cell cycle;C-5 methylation of cytosine	ec:2.1.1;ec:2.1.1.37-(cytosine-5-)-methyltransferase	Cysteine and methionine metabolism
Phvul.004G176100.2	DNA (cytosine-5)-methyltransferase CMT3	nucleus;chromatin binding;DNA (cytosine-5-)-methyltransferase activity;DNA replication initiation;regulation of DNA replication;methylation-dependent chromatin silencing;cell proliferation;regulation of flower development;zygote asymmetric cytokinesis in embryo sac;DNA methylation on cytosine within a CNG sequence;histone phosphorylation;chromatin silencing by small RNA;histone H3-K9 methylation;regulation of cell cycle;C-5 methylation of cytosine	ec:2.1.1;ec:2.1.1.37-(cytosine-5-)-methyltransferase	Cysteine and methionine metabolism
Phvul.004G176400.1	probable RNA-dependent RNA polymerase 5 isoform X1	RNA-directed RNA polymerase activity;transcription, RNA-templated	ec:2.7.7.48	
Phvul.004G176500.1	SNW SKI-interacting -like	spliceosomal complex;nucleolus;mRNA splicing, via spliceosome;nuclear-transcribed mRNA catabolic process;N-terminal protein myristoylation;vegetative to reproductive phase transition of meristem;regulation of circadian rhythm;positive regulation of transcription, DNA-templated		
Phvul.004G178400.1	microtubule-associated TORTIFOLIA1	DNA replication initiation;regulation of DNA replication;methylation-dependent chromatin silencing;microtubule-based process;cell proliferation;regulation of flower development;chromatin silencing by small RNA;histone H3-K9 methylation;regulation of cell cycle		
Phvul.005G000400.1	DUF246 domain-containing	integral component of membrane;transferase activity, transferring glycosyl groups;metabolic process		
Phvul.005G000500.1	Deoxycytidine kinase	nucleus;thymidine kinase activity;hydrolase activity;deoxyribonucleoside monophosphate biosynthetic process;phosphorylation	ec:2.7.1.21-kinase;ec:2.7.1.145	Drug metabolism - other enzymes;Pyrimidine metabolism
Phvul.005G001300.2	splicing factor U2af large subunit B-like isoform X4	commitment complex;nuclear speck;U2-type prespliceosome;U2AF;nucleotide binding;poly-pyrimidine tract binding;pre-mRNA 3'-splice site binding;U2-type prespliceosome assembly		
Phvul.005G003000.1	hypothetical protein PHAVU_005G003000g	mitotic cell cycle;RNA processing		
Phvul.005G003600.1	phosphatidylinositol phosphatidylcholine transfer SFH13	vegetative to reproductive phase transition of meristem;protein desumoylation;hydrogen peroxide biosynthetic process		
Phvul.005G003600.2	phosphatidylinositol phosphatidylcholine transfer SFH13	vegetative to reproductive phase transition of meristem;protein desumoylation;hydrogen peroxide biosynthetic process		
Phvul.005G003600.3	phosphatidylinositol phosphatidylcholine transfer SFH13	vegetative to reproductive phase transition of meristem;protein desumoylation;hydrogen peroxide biosynthetic process		
Phvul.005G006600.1	vacuolar sorting-associated 9A-like isoform X1			

Phvul.005G008900.1	U-box domain-containing 35-like isoform X1	intracellular;MAP kinase kinase kinase activity;ubiquitin-protein transferase activity;ATP binding;ligase activity;MAPK cascade;activation of MAPKK activity;protein ubiquitination	ec:2.7.11;ec:2.7.11.25	
Phvul.005G010900.1	hypothetical protein PHAVU_005G010900g	single-organism process		
Phvul.005G011000.1	pentatricopeptide repeat-containing At3g57430, chloroplastic-like	microtubule-severing ATPase activity;metabolic process	ec:3.6.1;ec:3.6.1.3-adenylpyrophosphatase;ec:3.6.4.3;ec:3.6.1.15-phosphatase	Purine metabolismPurine metabolism;Thiamine metabolism
Phvul.005G012600.1	histone-lysine N-methyltransferase, H3 lysine-9 specific SUVH1-like	nucleus;chromosome;zinc ion binding;histone-lysine N-methyltransferase activity;histone binding;histone lysine methylation	ec:2.1.1;ec:2.1.1.43-N-methyltransferase	Lysine degradation
Phvul.005G012800.1	zinc ion-binding ,	zinc ion binding;ligase activity;metabolic process		
Phvul.005G013400.1	BTB POZ domain-containing At3g05675			
Phvul.005G014700.1	pentatricopeptide repeat-containing At1g11290	chloroplast;endonuclease activity;zinc ion binding;microtubule-severing ATPase activity;mRNA modification;nucleic acid phosphodiester bond hydrolysis	ec:3.6.1;ec:3.6.1.3-adenylpyrophosphatase;ec:3.6.4.3;ec:3.1;ec:3.6.1.15-phosphatase	Purine metabolismPurine metabolism;Thiamine metabolism
Phvul.005G015800.1	Cytochrome P450, isoform 4, partial	integral component of membrane;monooxygenase activity;iron ion binding;oxidoreductase activity, acting on paired donors, with incorporation or reduction of molecular oxygen;heme binding;multicellular organismal development;brassinosteroid homeostasis;sterol metabolic process;brassinosteroid biosynthetic process;oxidation-reduction process		
Phvul.005G016000.1	RING-H2 finger ATL52-like	integral component of membrane;zinc ion binding;hydrolase activity;ligase activity;metabolic process		
Phvul.005G016000.2	RING-H2 finger ATL52-like	integral component of membrane;zinc ion binding;hydrolase activity;ligase activity;metabolic process		
Phvul.005G016500.1	TMV resistance N-like	integral component of membrane;ATP binding;ADP binding;defense response;signal transduction		
Phvul.005G016500.2	TMV resistance N-like	integral component of membrane;ATP binding;ADP binding;defense response;signal transduction		
Phvul.005G018700.1	bifunctional purine biosynthesis purH	chloroplast stroma;stromule;IMP cyclohydrolase activity;phosphoribosylaminoimidazolecarboxamide formyltransferase activity;purine nucleotide biosynthetic process;pyrimidine ribonucleotide biosynthetic process	ec:3.5.4.10-cyclohydrolase;ec:2.1.2.3-formyltransferase	Purine metabolism;Biosynthesis of antibioticsPurine metabolism;Biosynthesis of antibiotics;One carbon pool by folate
Phvul.005G019200.1	hypothetical protein PHAVU_005G019200g	integral component of membrane		
Phvul.005G020800.1	hypothetical protein PHAVU_005G020800g	microtubule cytoskeleton organization;cytokinesis by cell plate formation		
Phvul.005G021200.1	DNA ligase 1	nucleus;mitochondrion;DNA binding;DNA ligase (ATP) activity;ATP binding;lagging strand elongation;DNA recombination;DNA ligation involved in DNA repair;DNA biosynthetic process	ec:6.5.1;ec:6.5.1.1	
Phvul.005G021800.1	Group 2,	integral component of membrane		
Phvul.005G022900.1	PHYLLO, chloroplastic isoform X1	thiamine pyrophosphate binding;2-succinyl-5-enolpyruvyl-6-hydroxy-3-cyclohexene-1-carboxylic-acid synthase activity;2-succinyl-6-hydroxy-2,4-cyclohexadiene-1-carboxylate synthase activity;cellular amino acid catabolic	ec:4.2.99.20-synthase;ec:2.2.1.9-synthase	Ubiquinone and other terpenoid-quinone biosynthesisUbiquinone and other terpenoid-quinone biosynthesis

		process;menaquinone biosynthetic process;phylloquinone biosynthetic process;photosystem I stabilization		
Phvul.005G023100.1	RNA polymerase sigma factor sigD, chloroplastic	DNA binding;transcription factor activity, sequence-specific DNA binding;sigma factor activity;DNA-templated transcription, initiation;regulation of transcription, DNA-templated;cellular carbohydrate metabolic process;single-organism carbohydrate metabolic process;single-organism cellular process		
Phvul.005G023100.4	RNA polymerase sigma factor sigD, chloroplastic	chloroplast;plastid sigma factor activity;DNA binding;transcription factor activity, sequence-specific DNA binding;maltose metabolic process;pentose-phosphate shunt;DNA-templated transcription, initiation;regulation of transcription, DNA-templated;mRNA modification;starch biosynthetic process		
Phvul.005G023100.5	RNA polymerase sigma factor sigD, chloroplastic	DNA binding;transcription factor activity, sequence-specific DNA binding;sigma factor activity;DNA-templated transcription, initiation;regulation of transcription, DNA-templated;cellular carbohydrate metabolic process;single-organism carbohydrate metabolic process;single-organism cellular process		
Phvul.005G023300.2	alpha-N-acetylglucosaminidase family	vacuole;alpha-N-acetylglucosaminidase activity;metabolic process	ec:3.2.1.50-alpha-acetylglucosaminidase;ec:3.2.1	Glycosaminoglycan degradation
Phvul.005G026000.1	calcium-binding CML19	calcium ion binding		
Phvul.005G026300.1	LATERAL ROOT PRIMORDIUM 1-like			
Phvul.005G027200.1	NBS-LRR type disease resistance	ADP binding;defense response		
Phvul.005G029000.1	F-box SKIP22-like			
Phvul.005G029400.1	E3 ubiquitin- ligase SINAT5-like	nucleus;cytoplasm;zinc ion binding;ligase activity;ubiquitin protein ligase activity;apoptotic process;multicellular organismal development;protein ubiquitination involved in ubiquitin-dependent protein catabolic process;proteasome-mediated ubiquitin-dependent protein catabolic process		
Phvul.005G029500.1	Translation initiation factor eIF-2B subunit beta	eukaryotic translation initiation factor 2B complex;translation initiation factor activity;guanyl-nucleotide exchange factor activity;transferase activity;S-methyl-5-thioribose-1-phosphate isomerase activity;translational initiation;regulation of translational initiation;L-methionine biosynthetic process from methylthioadenosine;positive regulation of GTPase activity	ec:5.3.1.23-isomerase	Cysteine and methionine metabolism
Phvul.005G032400.1	disease resistance-responsive, dirigent domain	integral component of membrane		
Phvul.005G035600.1	plasma-membrane choline transporter family	integral component of membrane;cysteine biosynthetic process		
Phvul.005G036800.1	SURP and G-patch domain-containing 1 isoform X1	RNA binding;RNA processing		
Phvul.005G037700.1	ribulose-1,5 bisphosphate carboxylase oxygenase large subunit N-methyltransferase, chloroplastic	chloroplast stroma;protein-lysine N-methyltransferase activity;[ribulose-bisphosphate carboxylase]-lysine N-methyltransferase activity;maltose metabolic process;phosphatidylglycerol biosynthetic process;aromatic amino acid family biosynthetic process;embryo development ending in seed dormancy;chloroplast relocation;thylakoid membrane organization;iron-sulfur cluster assembly;peptidyl-lysine trimethylation;peptidyl-lysine monomethylation;starch biosynthetic	ec:2.1.1;ec:2.1.1.127	

		process;isopentenyl diphosphate biosynthetic process, methylerythritol 4-phosphate pathway;ncRNA metabolic process		
Phvul.005G039000.1	30S ribosomal 2, chloroplastic	ribosome;chloroplast thylakoid membrane;chloroplast stroma;chloroplast envelope;viral nucleocapsid;nucleotide binding;nucleic acid binding;photosynthetic electron transport in photosystem I;isopentenyl diphosphate biosynthetic process, methylerythritol 4-phosphate pathway;protein targeting to chloroplast		
Phvul.005G040900.1	callose synthase 2 isoform X1	1,3-beta-D-glucan synthase complex;integral component of membrane;1,3-beta-D-glucan synthase activity;(1->3)-beta-D-glucan biosynthetic process	ec:2.4.1.34-synthase;ec:2.4.1	Starch and sucrose metabolism
Phvul.005G040900.2	callose synthase 2 isoform X1	1,3-beta-D-glucan synthase complex;integral component of membrane;1,3-beta-D-glucan synthase activity;(1->3)-beta-D-glucan biosynthetic process	ec:2.4.1.34-synthase;ec:2.4.1	Starch and sucrose metabolism
Phvul.005G042700.1	serum response factor homolog A-like	xylan metabolic process		
Phvul.005G042800.1	DUF936 family			
Phvul.005G043100.1	DUF946 family			
Phvul.005G044000.1	ATPase family AAA domain-containing 3-like	ATP binding;hydrolase activity;metabolic process		
Phvul.005G044700.1	S-adenosylmethionine-dependent methyltransferase,	methyltransferase activity;methylation		
Phvul.005G044900.1	myosin-12 isoform X1	integral component of membrane;unconventional myosin complex;motor activity;actin binding;DNA topoisomerase type I activity;signal transducer activity;ATP binding;signal transduction;response to nematode;xylem development;actin filament-based movement;cell wall macromolecule metabolic process;Golgi localization;mitochondrion localization;peroxisome localization	ec:5.99.1.2;ec:3.6.1;ec:5.99.1;ec:3.6.1.15-phosphatase	Purine metabolism;Thiamine metabolism
Phvul.005G047500.1	plant T8K14-16	determination of bilateral symmetry;organ morphogenesis;meristem initiation;xylem and phloem pattern formation;meristem maintenance;flower morphogenesis;negative regulation of biological process		
Phvul.005G048400.1	ALA-interacting subunit 3-like	integral component of membrane		
Phvul.005G049900.1	cyclin-T1-3-like	cyclin-dependent protein kinase holoenzyme complex;nucleus;cyclin-dependent protein serine/threonine kinase regulator activity;positive regulation of cyclin-dependent protein serine/threonine kinase activity;positive regulation of transcription from RNA polymerase II promoter;positive regulation of phosphorylation of RNA polymerase II C-terminal domain	ec:2.7.1	
Phvul.005G049900.2	cyclin-T1-3-like	cyclin-dependent protein kinase holoenzyme complex;nucleus;cyclin-dependent protein serine/threonine kinase regulator activity;positive regulation of cyclin-dependent protein serine/threonine kinase activity;positive regulation of transcription from RNA polymerase II promoter;positive regulation of phosphorylation of RNA polymerase II C-terminal domain	ec:2.7.1	
Phvul.005G050100.1	rRNA methylase ytqB	chloroplast;methyltransferase activity;methylation		
Phvul.005G053000.1	pentatricopeptide repeat-containing At1g20230	zinc ion binding;microtubule-severing ATPase activity;metabolic process	ec:3.6.1;ec:3.6.1.3-adenylpyrophosphatase;ec:3.6.4.3;ec:3.6.1.15-phosphatase	Purine metabolismPurine metabolism;Thiamine metabolism

Phvul.005G054000.1	glutathione S-transferase	cytoplasm;glutathione transferase activity;glutathione metabolic process;toxin catabolic process	ec:2.5.1.18-transferase	Drug metabolism - cytochrome P450;Metabolism of xenobiotics by cytochrome P450;Glutathione metabolism
Phvul.005G054100.1	glutathione S-transferase	cytoplasm;glutathione transferase activity;glutathione metabolic process;toxin catabolic process	ec:2.5.1.18-transferase	Drug metabolism - cytochrome P450;Metabolism of xenobiotics by cytochrome P450;Glutathione metabolism
Phvul.005G054600.1	translation initiation factor IF-2, chloroplastic	microtubule;chloroplast stroma;chloroplast envelope;translation initiation factor activity;microtubule motor activity;GTPase activity;ATP binding;GTP binding;microtubule binding;translational initiation;microtubule-based movement;chloroplast relocation;thylakoid membrane organization;regulation of proton transport;isopentenyl diphosphate biosynthetic process, methylerythritol 4-phosphate pathway;ncRNA metabolic process;transcription from plastid promoter;protein autophosphorylation	ec:3.6.1;ec:3.6.1.15-phosphatase	Purine metabolism;Thiamine metabolism
Phvul.005G054900.1	like subfamily C member 14	integral component of membrane		
Phvul.005G055300.1	U5 small nuclear ribonucleo 200 kDa helicase-like	viral nucleocapsid;intracellular ribonucleoprotein complex;nucleic acid binding;helicase activity;ATP binding;metabolic process	ec:3.6.1;ec:3.6.1.15-phosphatase	Purine metabolism;Thiamine metabolism
Phvul.005G055300.2	U5 small nuclear ribonucleo 200 kDa helicase-like	viral nucleocapsid;intracellular ribonucleoprotein complex;nucleic acid binding;helicase activity;ATP binding;metabolic process	ec:3.6.1;ec:3.6.1.15-phosphatase	Purine metabolism;Thiamine metabolism
Phvul.005G055500.1	ribosomal L1 domain-containing 1-like	ribosome;RNA binding		
Phvul.005G055600.1	RRNAD1-like isoform X1	methyltransferase activity;methylation		
Phvul.005G055600.2	RRNAD1-like isoform X1	methyltransferase activity;methylation		
Phvul.005G056500.1	PAT1 like 1	cytoplasmic mRNA processing body;RNA binding;isomerase activity;deadenylation-dependent decapping of nuclear-transcribed mRNA;cytoplasmic mRNA processing body assembly		
Phvul.005G058500.1	1-aminocyclopropane-1-carboxylate oxidase	iron ion binding;oxidoreductase activity;oxidation-reduction process		
Phvul.005G058700.1	2OG-Fe(II) oxygenase family oxidoreductase	oxidoreductase activity;metal ion binding;oxidation-reduction process		
Phvul.005G062300.1	disease resistance (CC-NBS-LRR class) family	ATP binding;ADP binding;defense response		
Phvul.005G062300.2	Disease resistance	metabolic process		
Phvul.005G062300.3	Disease resistance	metabolic process		
Phvul.005G062500.1	callose synthase 8	1,3-beta-D-glucan synthase complex;integral component of membrane;1,3-beta-D-glucan synthase activity;(1->3)-beta-D-glucan biosynthetic process	ec:2.4.1.34-synthase;ec:2.4.1	Starch and sucrose metabolism
Phvul.005G063500.1	Secologanin synthase	integral component of membrane;acyl-CoA oxidase activity;iron ion binding;oxidoreductase activity, acting on paired donors, with incorporation or reduction of molecular oxygen, NAD(P)H as one donor, and incorporation of one atom of oxygen;heme binding;oxidation-reduction process;11-oxo-	ec:1.3.3.6-oxidase;ec:1.14.13	alpha-Linolenic acid metabolism;Biosynthesis of unsaturated fatty acids;Fatty acid degradation

		beta-amyrin catabolic process;glycyrrhetinate biosynthetic process		
Phvul.005G063500.2	11-oxo-beta-amyrin 30-oxidase-like	integral component of membrane;acyl-CoA oxidase activity;iron ion binding;oxidoreductase activity, acting on paired donors, with incorporation or reduction of molecular oxygen, NAD(P)H as one donor, and incorporation of one atom of oxygen;heme binding;oxidation-reduction process;11-oxo-beta-amyrin catabolic process;glycyrrhetinate biosynthetic process	ec:1.3.3.6-oxidase;ec:1.14.13	alpha-Linolenic acid metabolism;Biosynthesis of unsaturated fatty acids;Fatty acid degradation
Phvul.005G064700.1	receptor-like serine threonine- kinase At1g78530	intracellular;integral component of membrane;MAP kinase kinase kinase activity;transmembrane receptor protein tyrosine kinase activity;ATP binding;MAPK cascade;activation of MAPKK activity;peptidyl-tyrosine phosphorylation	ec:2.7.11;ec:2.7.10;ec:2.7.11.25;ec:2.7.10.1	
Phvul.005G064700.2	receptor-like serine threonine- kinase At1g78530	intracellular;integral component of membrane;MAP kinase kinase kinase activity;transmembrane receptor protein tyrosine kinase activity;ATP binding;MAPK cascade;activation of MAPKK activity;peptidyl-tyrosine phosphorylation	ec:2.7.11;ec:2.7.10;ec:2.7.11.25;ec:2.7.10.1	
Phvul.005G067300.1	increased DNA methylation 1-like	membrane;DNA binding;N-acetyltransferase activity;zinc ion binding;galactosylxylosylprotein 3-beta-galactosyltransferase activity;protein glycosylation;regulation of cellular metabolic process;posttranscriptional gene silencing by RNA;histone H3 acetylation;regulation of nitrogen compound metabolic process;production of small RNA involved in gene silencing by RNA;regulation of primary metabolic process	ec:2.4.1;ec:2.4.1.134-3-beta-galactosyltransferase;ec:2.3.1	Glycosaminoglycan biosynthesis - heparan sulfate / heparin;Glycosaminoglycan biosynthesis - chondroitin sulfate / dermatan sulfate
Phvul.005G068700.1	hypothetical protein PHAVU_005G068700g			
Phvul.005G069700.1	nuclear pore complex NUP98A	nuclear pore;transport		
Phvul.005G071900.1	probable serine threonine- kinase At1g54610	plasma membrane;cyclin-dependent protein serine/threonine kinase activity;ATP binding;protein phosphorylation;regulation of cell cycle	ec:2.7.11;ec:2.7.11.22	
Phvul.005G073600.1	hypothetical protein VIGAN_07134100	integral component of membrane		
Phvul.005G075600.1	nuclear pore complex NUP98A isoform X1	nuclear pore;transport		
Phvul.005G075600.2	nuclear pore complex NUP98A isoform X1	nuclear pore;transport		
Phvul.005G076700.1	B-box zinc finger 22	nucleus;transcription factor activity, transcription factor binding;zinc ion binding;regulation of transcription, DNA-templated;photomorphogenesis		
Phvul.005G076700.2	B-box zinc finger 22	nucleus;transcription factor activity, transcription factor binding;zinc ion binding;regulation of transcription, DNA-templated;photomorphogenesis		
Phvul.005G080500.1	WW domain-binding 11-like	integral component of membrane		
Phvul.005G081000.1	NHL repeat	RNA splicing, via endonucleolytic cleavage and ligation;methionine biosynthetic process;virus induced gene silencing;vegetative phase change		
Phvul.005G082400.1	choline transporter 2	plasma membrane;plasmodesma;integral component of membrane		
Phvul.005G082600.1	Nucleolar GTP-binding 2	nucleolus;GTPase activity;GTP binding;metabolic process;ribosome biogenesis	ec:3.6.1;ec:3.6.1.15-phosphatase	Purine metabolism;Thiamine metabolism
Phvul.005G085000.1	Peroxisomal membrane 22 kDa family isoform 1	integral component of membrane		

Phvul.005G085000.2	Peroxisomal membrane 22 kDa family isoform 1	integral component of membrane		
Phvul.005G086200.1	pre-mRNA-processing-splicing factor 8	U5 snRNP;catalytic step 2 spliceosome;second spliceosomal transesterification activity;U6 snRNA binding;U1 snRNA binding;U2 snRNA binding;U5 snRNA binding;pre-mRNA intronic binding;spliceosomal tri-snRNP complex assembly		
Phvul.005G086600.1	cyclic nucleotide-gated ion channel 4	cell;integral component of membrane;voltage-gated potassium channel activity;cellular cation homeostasis;regulation of membrane potential;divalent metal ion transport;potassium ion transmembrane transport		
Phvul.005G088000.1	S-locus lectin kinase family	plasma membrane;plasmodesma;integral component of membrane;protein serine/threonine kinase activity;ATP binding;carbohydrate binding;protein phosphorylation;defense response;recognition of pollen	ec:2.7.11	
Phvul.005G088400.1	NADH dehydrogenase subunit 5, partial (mitochondrion)	mitochondrial inner membrane;integral component of membrane;NADH dehydrogenase (ubiquinone) activity;ATP synthesis coupled electron transport	ec:1.6.5.3-reductase (H+-translocating);ec:1.6.99.5;ec:1.6.99.3-dehydrogenase	Oxidative phosphorylationOxidative phosphorylation
Phvul.005G090600.1	kDa class VI heat shock	response to heat;response to high light intensity;response to hydrogen peroxide		
Phvul.005G091300.1	autophagy-related 18f isoform X1	cytoplasm;integral component of membrane;cellular response to phosphate starvation;galactolipid biosynthetic process;cellular response to water deprivation		
Phvul.005G091400.1	UHRF1-binding 1-like			
Phvul.005G093400.1	disease resistance (TIR-NBS-LRR class)	purine ribonucleoside binding;adenyl ribonucleotide binding;anion binding;response to stimulus		
Phvul.005G096400.1	transmembrane ,	integral component of membrane		
Phvul.005G096700.1	transcription factor GTE4			
Phvul.005G097500.1	E3 ubiquitin- ligase ATL41-like	integral component of membrane;zinc ion binding		
Phvul.005G098500.1	pentatricopeptide repeat-containing At1g06270	transferase activity, transferring acyl groups other than amino-acyl groups;metabolic process		
Phvul.005G098500.2	pentatricopeptide repeat-containing At1g06270	transferase activity, transferring acyl groups other than amino-acyl groups;metabolic process		
Phvul.005G099800.1	zinc finger MAGPIE	nucleic acid binding;metal ion binding		
Phvul.005G101800.1	diacylglycerol kinase domain	diacylglycerol kinase activity;ATP binding;protein kinase C-activating G-protein coupled receptor signaling pathway;phosphorylation	ec:2.7.1.107-kinase (ATP)	Glycerophospholipid metabolism;Glycerolipid metabolism;Phosphatidylinositol signaling system
Phvul.005G101800.2	diacylglycerol kinase domain	diacylglycerol kinase activity;ATP binding;protein kinase C-activating G-protein coupled receptor signaling pathway;phosphorylation	ec:2.7.1.107-kinase (ATP)	Glycerophospholipid metabolism;Glycerolipid metabolism;Phosphatidylinositol signaling system
Phvul.005G101800.3	diacylglycerol kinase domain	diacylglycerol kinase activity;ATP binding;protein kinase C-activating G-protein coupled receptor signaling pathway;phosphorylation	ec:2.7.1.107-kinase (ATP)	Glycerophospholipid metabolism;Glycerolipid metabolism;Phosphatidylinositol signaling system
Phvul.005G103200.1	probable L-type lectin-domain containing receptor kinase	integral component of membrane;protein serine/threonine kinase activity;transmembrane receptor protein tyrosine kinase activity;ATP binding;carbohydrate binding;peptidyl-tyrosine phosphorylation	ec:2.7.11;ec:2.7.10;ec:2.7.10.1	

Phvul.005G104500.1	Acyl-[acyl-carrier-]--UDP-N-acetylglucosamine O-acyltransferase	transferase activity		
Phvul.005G104900.1	pre-mRNA-processing factor 6	U4/U6 x U5 tri-snRNP complex;catalytic step 2 spliceosome;spliceosomal tri-snRNP complex assembly;RNA-directed DNA methylation;positive regulation of miRNA metabolic process;positive regulation of primary miRNA processing		
Phvul.005G107100.1	ROOT INITIATION DEFECTIVE 3	Cul4-RING E3 ubiquitin ligase complex;negative regulation of shoot apical meristem development		
Phvul.005G109300.1	equilibrative nucleotide transporter 3-like	integral component of membrane;nucleoside transmembrane transporter activity;nucleoside transmembrane transport		
Phvul.005G109300.2	equilibrative nucleotide transporter 3-like	integral component of membrane;nucleoside transmembrane transporter activity;nucleoside transmembrane transport		
Phvul.005G111500.1	probable terpene synthase 11	magnesium ion binding;isoprene synthase activity;metabolic process	ec:4.2.3.27-synthase	Terpenoid backbone biosynthesis
Phvul.005G111700.1	probable terpene synthase 11	magnesium ion binding;isoprene synthase activity;metabolic process	ec:4.2.3.27-synthase	Terpenoid backbone biosynthesis
Phvul.005G114200.2	syntaxin of plants 122	plasma membrane;endomembrane system;integral component of membrane;SNARE complex;SNARE binding;SNAP receptor activity;intracellular protein transport;exocytosis;vesicle fusion;vesicle docking		
Phvul.005G114800.1	glutamate receptor -like	integral component of membrane;G-protein coupled receptor activity;ionotropic glutamate receptor activity;ion transport;G-protein coupled receptor signaling pathway;ionotropic glutamate receptor signaling pathway		
Phvul.005G114900.1	Glutamate receptor	intracellular;plasma membrane;plasmodesma;integral component of membrane;G-protein coupled receptor activity;ionotropic glutamate receptor activity;calcium channel activity;G-protein coupled receptor signaling pathway;calcium-mediated signaling;ionotropic glutamate receptor signaling pathway;calcium ion transmembrane transport;cellular response to amino acid stimulus		
Phvul.005G116800.1	linolenate hydroperoxide lyase, chloroplastic	chloroplast envelope;monooxygenase activity;iron ion binding;oxidoreductase activity, acting on paired donors, with incorporation or reduction of molecular oxygen;heme binding;hydroperoxide dehydratase activity;tryptophan catabolic process;fatty acid metabolic process;indoleacetic acid biosynthetic process;sterol metabolic process;oxidation-reduction process	ec:4.2.1.92-dehydratase	alpha-Linolenic acid metabolism
Phvul.005G119100.1	E3 ubiquitin- ligase PUB23-like	ubiquitin-protein transferase activity;ligase activity;protein ubiquitination		
Phvul.005G120200.1	histone deacetylase 8	NAD-dependent histone deacetylase activity (H3-K14 specific);histone H3 deacetylation	ec:3.5.1.98	
Phvul.005G122400.1	capsanthin capsorubin synthase, chromoplast-like	chromoplast;oxidoreductase activity, acting on paired donors, with incorporation or reduction of molecular oxygen;capsanthin synthase activity;capsorubin synthase activity;carotenoid biosynthetic process;oxidation-reduction process	ec:5.3.99.8-synthase	Carotenoid biosynthesis
Phvul.005G123900.1	myb family transcription factor family	nucleus;DNA binding;transcription, DNA-templated;regulation of transcription, DNA-templated		
Phvul.005G124400.1	pentatricopeptide repeat-containing At5g48910-like	zinc ion binding		
Phvul.005G125800.1	flagellar radial spoke 5 isoform X2	oxidoreductase activity;oxidation-reduction process		
Phvul.005G127300.1	TIC 100	kinase activity;phosphorylation		

Phvul.005G127800.1	splicing factor U2af small subunit B-like	nucleus;nucleotide binding;RNA binding;metal ion binding		
Phvul.005G128200.1	AP-2 complex subunit alpha-1-like	clathrin adaptor complex;protein transporter activity;intracellular protein transport;vesicle-mediated transport		
Phvul.005G128200.2	AP-2 complex subunit alpha-1-like	clathrin adaptor complex;protein transporter activity;intracellular protein transport;vesicle-mediated transport		
Phvul.005G130400.1	DUF2146 family	nuclear-transcribed mRNA catabolic process, nonsense-mediated decay		
Phvul.005G130600.1	UPSTREAM OF FLC isoform X1			
Phvul.005G131000.1	serine carboxypeptidase-like	vacuole;serine-type carboxypeptidase activity;proteolysis involved in cellular protein catabolic process	ec:3.4.21;ec:3.4.16	
Phvul.005G133500.1	helicase MOM1-like isoform X1			
Phvul.005G133500.2	helicase MOM1-like isoform X1	hydrolase activity		
Phvul.005G134300.1	pleckstrin-like (PH) and lipid-binding START domain	plasma membrane;lipid binding		
Phvul.005G134500.1	auxin response factor 18	nucleus;DNA binding;transcription factor activity, sequence-specific DNA binding;miRNA binding;transcription, DNA-templated;regulation of transcription, DNA-templated;pattern specification process;auxin-activated signaling pathway;abscisic acid-activated signaling pathway;response to carbohydrate;fruit development;regulation of anthocyanin biosynthetic process;leaf development;petal development;sepal development;developmental growth;root cap development;cell division		
Phvul.005G134600.1	Serine threonine-phosphatase 6 regulatory subunit 3			
Phvul.005G136900.1	auxin response factor 4-like isoform X2	nucleus;DNA binding;transcription, DNA-templated;regulation of transcription, DNA-templated;auxin-activated signaling pathway		
Phvul.005G137600.1	interactor of constitutive active ROPs 3 isoform X2			
Phvul.005G137600.2	interactor of constitutive active ROPs 3 isoform X2			
Phvul.005G137600.3	interactor of constitutive active ROPs 3 isoform X2			
Phvul.005G137600.4	interactor of constitutive active ROPs 3 isoform X2			
Phvul.005G137800.1	V-type proton ATPase subunit a1	vacuolar proton-transporting V-type ATPase, V0 domain;plant-type vacuole;endosome;trans-Golgi network;trans-Golgi network transport vesicle membrane;integral component of membrane;proton-transporting ATPase activity, rotational mechanism;ATPase binding;vacuolar acidification;ATP synthesis coupled proton transport;ATP hydrolysis coupled proton transport;vacuolar proton-transporting V-type ATPase complex assembly	ec:3.6.1;ec:3.6.1.3-adenylpyrophosphatase;ec:3.6.1.15-phosphatase	Purine metabolismPurine metabolism;Thiamine metabolism
Phvul.005G137800.2	V-type proton ATPase subunit a1	vacuolar proton-transporting V-type ATPase, V0 domain;plant-type vacuole;endosome;trans-Golgi network;trans-Golgi network transport vesicle membrane;integral component of membrane;proton-transporting ATPase activity, rotational mechanism;ATPase binding;vacuolar acidification;ATP synthesis coupled proton transport;ATP hydrolysis coupled proton transport;vacuolar proton-transporting V-type ATPase complex assembly	ec:3.6.1;ec:3.6.1.3-adenylpyrophosphatase;ec:3.6.1.15-phosphatase	Purine metabolismPurine metabolism;Thiamine metabolism
Phvul.005G139000.1	Nfrkb, isoform 1	Ino80 complex;DNA binding;mitotic cell cycle;RNA processing;sister chromatid cohesion;synapsis;reciprocal meiotic		

		recombination;positive regulation of cell proliferation;response to gamma radiation;glucuronoxylan metabolic process;chromatin silencing by small RNA;regulation of telomere maintenance;multicellular organism reproduction;meiotic DNA double-strand break formation;telomere maintenance in response to DNA damage;xylan biosynthetic process;positive regulation of transcription, DNA-templated		
Phvul.005G141100.1	Aquaporin TIP1-3	plant-type vacuole membrane;integral component of membrane;central vacuole;water channel activity;glycerol channel activity;water transport;glycerol transport;ion transmembrane transport		
Phvul.005G141200.1	myb transcription factor	DNA binding		
Phvul.005G141200.2	myb transcription factor	DNA binding		
Phvul.005G141200.3	myb transcription factor	DNA binding		
Phvul.005G141600.2	ras-related RABE1c	intracellular;membrane;GTPase activity;GTP binding;intracellular protein transport;vesicle docking involved in exocytosis;nucleocytoplasmic transport;small GTPase mediated signal transduction;metabolic process;protein secretion	ec:3.6.1;ec:3.6.1.15-phosphatase	Purine metabolism;Thiamine metabolism
Phvul.005G141800.1	histone H2A	nucleosome;nuclear chromatin;DNA binding;protein heterodimerization activity;chromatin silencing		
Phvul.005G141900.1	histone H2A	nucleosome;nuclear chromatin;DNA binding;protein heterodimerization activity;chromatin silencing		
Phvul.005G142400.1	ACT domain-containing ACR9-like	amino acid binding;nucleotidyltransferase activity;metabolic process;response to sucrose;response to fructose		
Phvul.005G142800.1	ubiquitin-conjugating enzyme E2 7	cytoplasm;ATP binding;ligase activity;ubiquitin protein ligase binding;ubiquitin protein ligase activity;protein ubiquitination		
Phvul.005G147200.1	DUF3527 domain			
Phvul.005G148600.1	zinc finger WIP2-like	nucleic acid binding;metal ion binding		
Phvul.005G149800.1	zinc transporter ZTP29	integral component of membrane;zinc ion transmembrane transporter activity;zinc II ion transmembrane transport		
Phvul.005G150000.1	pre-mRNA-splicing factor ATP-dependent RNA helicase PRP16	spliceosomal complex;ATP-dependent RNA helicase activity;ATP binding;poly(A) RNA binding;mRNA splicing, via spliceosome	ec:3.6.1;ec:3.6.1.3-adenylpyrophosphatase;ec:3.6.1.15-phosphatase;ec:2.7.7	Purine metabolismPurine metabolism;Thiamine metabolism
Phvul.005G152000.1	probable E3 ubiquitin- ligase ARI7	ubiquitin ligase complex;cytoplasm;nucleic acid binding;zinc ion binding;ligase activity;ubiquitin conjugating enzyme binding;ubiquitin protein ligase activity;protein polyubiquitination;positive regulation of proteasomal ubiquitin-dependent protein catabolic process;protein ubiquitination involved in ubiquitin-dependent protein catabolic process		
Phvul.005G152500.1	endonuclease III homolog 1, chloroplastic-like isoform X1	nucleus;oxidized pyrimidine nucleobase lesion DNA N-glycosylase activity;DNA binding;metal ion binding;4 iron, 4 sulfur cluster binding;base-excision repair, AP site formation;nucleic acid phosphodiester bond hydrolysis	ec:3.2.2;ec:4.2.99.18;ec:3.1	
Phvul.005G152500.2	endonuclease III homolog 1, chloroplastic-like isoform X1	nucleus;oxidized pyrimidine nucleobase lesion DNA N-glycosylase activity;DNA binding;metal ion binding;4 iron, 4 sulfur cluster binding;base-excision repair, AP site formation	ec:3.2.2;ec:4.2.99.18;ec:3.1	
Phvul.005G153800.1	electron transfer flavo -ubiquinone oxidoreductase, mitochondrial	mitochondrion;electron-transferring-flavoprotein dehydrogenase activity;leucine catabolic process;methylglyoxal catabolic process to D-lactate via S-lactoyl-glutathione;oxidation-reduction process	ec:1.5.5.1	

Phvul.005G156000.1	polyadenylate-binding -interacting 3-like isoform X1	cytoplasmic stress granule;poly(A) RNA binding;regulation of cytoplasmic mRNA processing body assembly;stress granule assembly		
Phvul.005G158000.1	coatomer subunit zeta-2-like	membrane;viral capsid;protein transport		
Phvul.005G158700.1	beta-galactosidase 17	integral component of membrane;nucleic acid binding;beta-galactosidase activity;metal ion binding;vegetative to reproductive phase transition of meristem;glucuronoxylan metabolic process;protein desumoylation;xylan biosynthetic process;hydrogen peroxide biosynthetic process	ec:3.2.1;ec:3.2.1.23-lactase (ambiguous)	Other glycan degradation;Glycosphingolipid biosynthesis - ganglio series;Sphingolipid metabolism;Galactose metabolism;Glycosaminoglycan degradation
Phvul.005G160700.1	inactive kinase SELMODRAFT_444075-like	non-membrane spanning protein tyrosine kinase activity;ATP binding;hydrolase activity;peptidyl-tyrosine phosphorylation	ec:2.7.10;ec:2.7.10.2-protein-tyrosine kinase	T cell receptor signaling pathway
Phvul.005G161600.1	eukaryotic translation initiation factor 2 subunit gamma-like	intracellular;integral component of membrane;translation initiation factor activity;GTPase activity;GTP binding;formation of translation preinitiation complex	ec:3.6.1;ec:3.6.1.15-phosphatase	Purine metabolism;Thiamine metabolism
Phvul.005G163000.1	leucine-rich repeat receptor kinase At2g19210 isoform X2	integral component of membrane;protein serine/threonine kinase activity;ATP binding;protein phosphorylation	ec:2.7.11	
Phvul.005G166600.1	flavonol synthase flavanone 3-hydroxylase-like	integral component of membrane		
Phvul.005G167900.1	Kinesin-related 6	nucleus;kinesin complex;microtubule;plasma membrane;microtubule motor activity;ATP binding;microtubule binding;ATPase activity;microtubule-based movement;metabolic process	ec:3.6.1;ec:3.6.1.3-adenylpyrophosphatase;ec:3.6.1.15-phosphatase	Purine metabolismPurine metabolism;Thiamine metabolism
Phvul.005G169100.1	golgin candidate 5	zinc ion binding;microtubule-severing ATPase activity;metabolic process	ec:3.6.1;ec:3.6.1.3-adenylpyrophosphatase;ec:3.6.4.3;ec:3.6.1.15-phosphatase	Purine metabolismPurine metabolism;Thiamine metabolism
Phvul.005G169600.1	auxin-responsive IAA27-like	nucleus;transcription, DNA-templated;regulation of transcription, DNA-templated;auxin-activated signaling pathway		
Phvul.005G170900.1	ras-related RHN1-like isoform X1	intracellular;membrane;GTPase activity;GTP binding;intracellular protein transport;nucleocytoplasmic transport;small GTPase mediated signal transduction;metabolic process	ec:3.6.1;ec:3.6.1.15-phosphatase	Purine metabolism;Thiamine metabolism
Phvul.005G172800.1	E3 ubiquitin- ligase At1g63170	integral component of membrane;zinc ion binding;hydrolase activity;ligase activity;metabolic process		
Phvul.005G174500.1	plant F18G18-200	plant-type cell wall		
Phvul.005G174900.1	lys-63-specific deubiquitinase BRCC36-like	integral component of membrane		
Phvul.005G174900.2	lys-63-specific deubiquitinase BRCC36-like	integral component of membrane		
Phvul.005G175300.1	Aspartate-glutamate racemase family	amino-acid racemase activity;peptidoglycan biosynthetic process	ec:5.1.1.10-racemase	D-Arginine and D-ornithine metabolism;D-Glutamine and D-glutamate metabolism;Cysteine and methionine metabolism;Glycine, serine and threonine metabolism
Phvul.005G175500.1	nuclear pore complex NUP93A-like	nuclear pore;transferase activity;structural constituent of nuclear pore;protein import into nucleus;poly(A)+ mRNA export from nucleus;nuclear pore complex assembly		
Phvul.005G179700.1	NUCLEAR FUSION DEFECTIVE 4	nucleus;integral component of membrane		
Phvul.005G182000.1	metal transporter Nramp6-like	integral component of membrane;transporter activity;transport		

Phvul.005G182000.2	metal transporter Nramp6-like	integral component of membrane;transporter activity;transport		
Phvul.005G183700.1	AP2-like ethylene-responsive transcription factor At1g16060	nucleus;DNA binding;transcription factor activity, sequence-specific DNA binding;transcription, DNA-templated;regulation of transcription, DNA-templated;multicellular organismal development		
Phvul.005G184300.1	ribulose bisphosphate carboxylase oxygenase activase, chloroplastic	chloroplast thylakoid membrane;ATP binding;hydrolase activity;thylakoid membrane organization;carotenoid biosynthetic process		
Phvul.005G186000.1	hypothetical protein PHAVU_005G186000g	integral component of membrane		
Phvul.006G000300.1	calcium permeable stress-gated cation channel 1-like	integral component of membrane;4-alpha-glucanotransferase activity;glycogen metabolic process	ec:2.4.1.25-disproportionating enzyme	Starch and sucrose metabolism
Phvul.006G001200.1	hypothetical protein PHAVU_006G001200g			
Phvul.006G001800.1	UPF0586 C9orf41	methyltransferase activity;methylation		
Phvul.006G001800.2	UPF0586 C9orf41	methyltransferase activity;methylation		
Phvul.006G002500.1	E3 ubiquitin- ligase MARCH7-like isoform X1	integral component of membrane;zinc ion binding;ligase activity;metabolic process		
Phvul.006G002500.2	E3 ubiquitin- ligase MARCH7-like isoform X1	integral component of membrane;zinc ion binding;ligase activity;metabolic process		
Phvul.006G003000.1	serine threonine-kinase ATM-like isoform X1	transferase activity		
Phvul.006G005200.1	zinc finger CONSTANS-LIKE 9-like	intracellular;integral component of membrane;zinc ion binding		
Phvul.006G005800.1	breast cancer type 2 susceptibility	nucleus;single-stranded DNA binding;double-strand break repair via homologous recombination;regulation of transcription, DNA-templated		
Phvul.006G008800.1	anamorsin	mitochondrial intermembrane space;methyltransferase activity;electron carrier activity;metal ion binding;2 iron, 2 sulfur cluster binding;iron-sulfur cluster assembly;methylation;negative regulation of apoptotic process		
Phvul.006G009000.1	homoserine dehydrogenase	cytosol;homoserine dehydrogenase activity;kinase activity;NADP binding;methionine biosynthetic process;threonine biosynthetic process;isoleucine biosynthetic process;phosphorylation;oxidation-reduction process	ec:1.1.1.3-dehydrogenase	Biosynthesis of antibiotics;Cysteine and methionine metabolism;Lysine biosynthesis;Glycine, serine and threonine metabolism
Phvul.006G009100.1	FAM91A1 isoform X1			
Phvul.006G009100.2	FAM91A1 isoform X1			
Phvul.006G009100.3	FAM91A1			
Phvul.006G009200.1	probable plastidic glucose transporter 1 isoform X1	integral component of membrane;substrate-specific transmembrane transporter activity;carbohydrate transport;transmembrane transport		
Phvul.006G009200.2	probable plastidic glucose transporter 1 isoform X1	integral component of membrane;substrate-specific transmembrane transporter activity;carbohydrate transport;transmembrane transport		
Phvul.006G010300.1	alcohol dehydrogenase-like 7	zinc ion binding;S-(hydroxymethyl)glutathione dehydrogenase activity;oxidation-reduction process	ec:1.1.1.284-dehydrogenase	Methane metabolism

Phvul.006G010300.2	alcohol dehydrogenase-like 7	zinc ion binding;S-(hydroxymethyl)glutathione dehydrogenase activity;oxidation-reduction process	ec:1.1.1.284-dehydrogenase	Methane metabolism
Phvul.006G010900.1	non-structural maintenance of chromosomes element 1 homolog isoform X1	nucleus;Smc5-Smc6 complex;ubiquitin-protein transferase activity;double-strand break repair via homologous recombination;postreplication repair;protein ubiquitination		
Phvul.006G012800.1	Transcription factor PIF1	integral component of membrane;protein dimerization activity		
Phvul.006G013500.1	Thymidine kinase	thymidine kinase activity;ATP binding;identical protein binding;deoxyribonucleoside monophosphate biosynthetic process;phosphorylation;DNA biosynthetic process	ec:2.7.1.21-kinase;ec:2.7.1.145	Drug metabolism - other enzymes;Pyrimidine metabolism
Phvul.006G014200.1	telomerase activating Est1	meiotic nuclear division		
Phvul.006G016600.1	Myb SANT-like DNA-binding domain	DNA binding		
Phvul.006G016600.2	Myb SANT-like DNA-binding domain	DNA binding		
Phvul.006G016900.1	linoleate 13S-lipoxygenase 2-1, chloroplastic-like	oxidoreductase activity, acting on single donors with incorporation of molecular oxygen, incorporation of two atoms of oxygen;metal ion binding;oxylipin biosynthetic process;oxidation-reduction process	ec:1.13.11	
Phvul.006G019400.1	ubiquitin-associated TS-N domain ,	translation elongation factor activity;translational elongation		
Phvul.006G020900.1	CBL-interacting serine threonine-kinase 3	protein serine/threonine kinase activity;ATP binding;protein phosphorylation;signal transduction	ec:2.7.11	
Phvul.006G020900.2	CBL-interacting serine threonine-kinase 3	protein serine/threonine kinase activity;ATP binding;protein phosphorylation;signal transduction	ec:2.7.11	
Phvul.006G020900.3	CBL-interacting serine threonine-kinase 3	protein serine/threonine kinase activity;ATP binding;protein phosphorylation;signal transduction	ec:2.7.11	
Phvul.006G022300.1	cytochrome b561 domain-containing At2g30890-like	integral component of membrane;oxidation-reduction process		
Phvul.006G022400.1	VACUOLELESS1	lysosomal membrane;endosome;plant-type vacuole membrane;HOPS complex;actin binding;protein N-linked glycosylation;protein targeting to vacuole;Golgi organization;vacuole organization;vegetative to reproductive phase transition of meristem;glucuronoxylan metabolic process;endosomal transport;protein desumoylation;regulation of vacuole fusion, non-autophagic;regulation of SNARE complex assembly;xylan biosynthetic process;hydrogen peroxide biosynthetic process		
Phvul.006G027200.1	ATP-dependent RNA helicase,	ATP-dependent RNA helicase activity;RNA-DNA hybrid ribonuclease activity;ATP binding;poly(A) RNA binding;RNA processing;RNA phosphodiester bond hydrolysis, endonucleolytic	ec:3.6.1;ec:3.1.30;ec:3.6.1.3-adenylpyrophosphatase;ec:3.1.26;ec:3.1;ec:3.1.26.4;ec:3.6.1.15-phosphatase;ec:2.7.7	Purine metabolismPurine metabolism;Thiamine metabolism
Phvul.006G027400.1	pentatricopeptide repeat-containing At5g27110-like			
Phvul.006G028500.1	transcription factor PIF4-like isoform X1	protein dimerization activity		
Phvul.006G028500.2	transcription factor PIF4-like isoform X1	protein dimerization activity		
Phvul.006G032500.1	disease resistance RPP13 4			
Phvul.006G034000.1	probable rhamnose biosynthetic enzyme 1	dTDP-glucose 4,6-dehydratase activity;UDP-glucose 4,6-dehydratase activity;nucleotide-sugar metabolic process	ec:4.2.1.76-4,6-dehydratase;ec:4.2.1.46-4,6-dehydratase	Amino sugar and nucleotide sugar metabolismPolyketide sugar unit biosynthesis;Biosynthesis of

				antibiotics;Streptomycin biosynthesis;Biosynthesis of vancomycin group antibiotics
Phvul.006G034500.1	zinc finger BED domain-containing RICESLEEPER 2-like isoform X1	DNA binding;protein dimerization activity		
Phvul.006G035800.1	RNA polymerase II transcription factor SIII (elongin) subunit A	nucleus;integral component of membrane;regulation of transcription, DNA-templated		
Phvul.006G036300.1	Peptidoglycan-binding domain-containing , isoform 1			
Phvul.006G036600.1	spermidine hydroxycinnamoyl transferase-like	shikimate O-hydroxycinnamoyltransferase activity;metabolic process	ec:2.3.1;ec:2.3.1.133-O-hydroxycinnamoyltransferase	Stilbenoid, diarylheptanoid and gingerol biosynthesis;Flavonoid biosynthesis;Phenylpropanoid biosynthesis
Phvul.006G037700.1	DDB1- and CUL4-associated factor 13	nucleolus;small-subunit processome;Cul4-RING E3 ubiquitin ligase complex;mitotic cell cycle;maturation of SSU-rRNA from tricistronic rRNA transcript (SSU-rRNA, 5.8S rRNA, LSU-rRNA);pyrimidine ribonucleotide biosynthetic process		
Phvul.006G037800.1	glucose-6-phosphate phosphate translocator 1, chloroplastic-like	integral component of membrane;cyclin-dependent protein serine/threonine kinase activity;transporter activity;metabolic process;carbohydrate transmembrane transport;regulation of cell cycle	ec:2.7.11;ec:2.7.11.22	
Phvul.006G039100.1	DEAD-box ATP-dependent RNA helicase 28	cytoplasmic ribonucleoprotein granule;nucleic acid binding;ATP-dependent RNA helicase activity;ATP binding;translational initiation;chromosome segregation;regulation of gene expression;RNA secondary structure unwinding	ec:3.6.1;ec:3.6.1.3-adenylpyrophosphatase;ec:3.6.1.15-phosphatase;ec:2.7.7	Purine metabolismPurine metabolism;Thiamine metabolism
Phvul.006G040000.1	Ankyrin repeat-containing	integral component of membrane		
Phvul.006G040300.1	plastidial lipoyltransferase 2-like	cytoplasm;octanoyltransferase activity;lipoyl(octanoyl) transferase activity;lipoate biosynthetic process;protein lipoylation	ec:2.3.1.181-transferase;ec:2.3.1	Lipoic acid metabolism
Phvul.006G040300.4	Plastidial lipoyltransferase 2	chloroplast;octanoyltransferase activity;ligase activity;lipoyltransferase activity;lipoyl(octanoyl) transferase activity;lipoate biosynthetic process;protein lipoylation	ec:2.3.1.181-transferase;ec:2.3.1	Lipoic acid metabolism
Phvul.006G041000.1	probable disease resistance At4g19060	ADP binding		
Phvul.006G041200.1	Disease resistance RPP13 4	hydrolase activity;metabolic process		
Phvul.006G041600.1	ankyrin repeat	integral component of membrane		
Phvul.006G042600.1	dipeptidyl-peptidase 5-like	cytosol;chloroplast;serine-type peptidase activity;N-terminal protein myristoylation;proteolysis		
Phvul.006G042700.1	dipeptidyl-peptidase 5-like	cytosol;chloroplast;serine-type peptidase activity;N-terminal protein myristoylation;proteolysis		
Phvul.006G043300.1	Nucleolar pre-ribosomal-associated 1			
Phvul.006G044600.1	ATP synthase gamma chain, chloroplastic	chloroplast ATP synthase complex;proton-transporting ATP synthase complex, catalytic core F(1);enzyme regulator activity;proton-transporting ATP synthase activity, rotational mechanism;proton-transporting ATPase activity, rotational mechanism;ATP synthesis coupled proton transport;regulation of catalytic activity;regulation of root morphogenesis	ec:3.6.1;ec:3.6.1.3-adenylpyrophosphatase;ec:3.6.1.15-phosphatase	Purine metabolismPurine metabolism;Thiamine metabolism

Phvul.006G045800.1	GDSL-like lipase acylhydrolase	integral component of membrane;hydrolase activity, acting on ester bonds;9,9'-dicis-carotene:quinone oxidoreductase activity;7,9,9'-tricis-neurosporene:quinone oxidoreductase activity;9,9'-di-cis-zeta-carotene desaturation to 7,9,7',9'-tetra-cis-lycopene;oxidation-reduction process	ec:1.3.5.6-desaturase	Carotenoid biosynthesis
Phvul.006G049000.1	transcription initiation factor TFIID subunit 6-like	nucleus;translation initiation factor activity;protein heterodimerization activity;DNA-templated transcription, initiation;translational initiation;regulation of sequence-specific DNA binding transcription factor activity		
Phvul.006G049900.1	SWIB MDM2 domain	DNA binding		
Phvul.006G051000.1	beta-(1,2)-xylosyltransferase	Golgi membrane;Golgi medial cisterna;integral component of membrane;glycoprotein 2-beta-D-xylosyltransferase activity;protein N-linked glycosylation;N-terminal protein myristoylation;posttranslational protein targeting to membrane, translocation	ec:2.4.2.38-2-beta-D-xylosyltransferase	Various types of N-glycan biosynthesis
Phvul.006G051200.1	ankyrin repeat	integral component of membrane		
Phvul.006G051400.1	probable cytokinin riboside 5 -monophosphate phosphoribohydrolase LOGL3	lyase activity;metabolic process		
Phvul.006G052700.1	Kinesin KIF15	kinesin complex;microtubule;ATP binding;microtubule binding;ATP-dependent microtubule motor activity, plus-end-directed;microtubule-based movement;metabolic process	ec:3.6.1;ec:3.6.1.3-adenylpyrophosphatase;ec:3.6.4.4;ec:3.6.1.15-phosphatase	Purine metabolismPurine metabolism;Thiamine metabolism
Phvul.006G053000.1	heterogeneous nuclear ribonucleo 1-like	viral nucleocapsid;intracellular ribonucleoprotein complex;nucleotide binding;nucleic acid binding		
Phvul.006G053000.2	heterogeneous nuclear ribonucleo 1-like	viral nucleocapsid;intracellular ribonucleoprotein complex;nucleotide binding;nucleic acid binding		
Phvul.006G054100.1	cytochrome P450 family 71	integral component of membrane;iron ion binding;oxidoreductase activity, acting on paired donors, with incorporation or reduction of molecular oxygen, NAD(P)H as one donor, and incorporation of one atom of oxygen;heme binding;secondary metabolite biosynthetic process;oxidation-reduction process	ec:1.14.13	
Phvul.006G059200.1	probable 28S rRNA (cytosine(4447)-C(5))-methyltransferase	integral component of membrane;RNA binding;S-adenosylmethionine-dependent methyltransferase activity;rRNA processing;methylation;transmembrane transport		
Phvul.006G060800.1	vacuolar cation proton exchanger 5-like isoform X1	integral component of membrane;calcium:proton antiporter activity;calcium ion transmembrane transport		
Phvul.006G060800.2	vacuolar cation proton exchanger 5-like isoform X1	integral component of membrane;calcium:proton antiporter activity;calcium ion transmembrane transport		
Phvul.006G061200.1	oxidoreductase transition metal ion-binding	DNA-directed RNA polymerase activity;RNA biosynthetic process	ec:2.7.7.6-RNA polymerase	Purine metabolism;Pyrimidine metabolism
Phvul.006G062700.1	armadillo repeat-containing 8-like			
Phvul.006G063900.1	Helicase SKI2W	vacuole;RNA binding;RNA helicase activity;ATP binding;RNA catabolic process;potassium ion transport;sister chromatid cohesion;chromatin silencing by small RNA;response to potassium ion;meiotic chromosome segregation	ec:3.6.1;ec:3.6.1.15-phosphatase;ec:2.7.7	Purine metabolism;Thiamine metabolism
Phvul.006G065100.1	thylakoid soluble phospho TSP9	chloroplast thylakoid membrane;chloroplast envelope;rRNA processing;response to blue light;chloroplast relocation;thylakoid membrane organization;response to red light;response to far red light;photosynthesis;cellular cation homeostasis;divalent metal ion transport		

Phvul.006G067600.2	RING U-box	cytosol;integral component of membrane;zinc ion binding;ligase activity;chlorophyll catabolic process		
Phvul.006G070200.1	Zinc transporter 6, chloroplastic	plasma membrane;integral component of membrane;zinc ion transmembrane transporter activity;2-alkenal reductase [NAD(P)] activity;oxidation-reduction process;zinc II ion transmembrane transport	ec:1.3.1.74	
Phvul.006G077100.1	WNK kinase	protein kinase activity;ATP binding;protein phosphorylation		
Phvul.006G082200.1	hypothetical protein PHAVU_006G082200g	intracellular part		
Phvul.006G084600.1	cysteine-rich receptor kinase 2	plasma membrane;integral component of membrane;protein serine/threonine kinase activity;transmembrane receptor protein tyrosine kinase activity;ATP binding;peptidyl-tyrosine phosphorylation	ec:2.7.11;ec:2.7.10;ec:2.7.10.1	
Phvul.006G085300.1	P-loop nucleoside triphosphate hydrolase superfamily	helicase activity;metabolic process	ec:3.6.1;ec:3.6.1.15-phosphatase	Purine metabolism;Thiamine metabolism
Phvul.006G087200.1	plant F12P19-7			
Phvul.006G087300.1	sucrose synthase	sucrose synthase activity;sucrose metabolic process	ec:2.4.1.13-synthase;ec:2.4.1	Starch and sucrose metabolism
Phvul.006G088900.1	serine threonine-phosphatase PP1	integral component of membrane;phosphoprotein phosphatase activity;metal ion binding;protein dephosphorylation	ec:3.1;ec:3.1.3.41-nitrophenyl phosphatase;ec:3.1.3.16-phosphatase	Aminobenzoate degradationT cell receptor signaling pathway
Phvul.006G088900.2	serine threonine-phosphatase PP1	integral component of membrane;phosphoprotein phosphatase activity;metal ion binding;protein dephosphorylation	ec:3.1;ec:3.1.3.41-nitrophenyl phosphatase;ec:3.1.3.16-phosphatase	Aminobenzoate degradationT cell receptor signaling pathway
Phvul.006G090000.1	cell cycle checkpoint control RAD9A	checkpoint clamp complex;3'-5' exonuclease activity;exodeoxyribonuclease III activity;DNA replication checkpoint;DNA repair;intra-S DNA damage checkpoint;cellular response to ionizing radiation;nucleic acid phosphodiester bond hydrolysis	ec:3.1.15;ec:3.1.11;ec:3.1;ec:3.1.11.2	
Phvul.006G090000.2	cell cycle checkpoint control RAD9A	checkpoint clamp complex;3'-5' exonuclease activity;exodeoxyribonuclease III activity;DNA replication checkpoint;DNA repair;intra-S DNA damage checkpoint;cellular response to ionizing radiation;nucleic acid phosphodiester bond hydrolysis	ec:3.1.15;ec:3.1.11;ec:3.1;ec:3.1.11.2	
Phvul.006G090000.3	cell cycle checkpoint control RAD9A	checkpoint clamp complex;3'-5' exonuclease activity;exodeoxyribonuclease III activity;DNA replication checkpoint;DNA repair;intra-S DNA damage checkpoint;cellular response to ionizing radiation;nucleic acid phosphodiester bond hydrolysis	ec:3.1.15;ec:3.1.11;ec:3.1;ec:3.1.11.2	
Phvul.006G090700.1	Acyl- -binding domain-containing 4	fatty-acyl-CoA binding;N-acylglucosamine 2-epimerase activity;metabolic process	ec:5.1.3.8-2-epimerase	Amino sugar and nucleotide sugar metabolism
Phvul.006G090700.2	Acyl- -binding domain-containing 4	fatty-acyl-CoA binding;N-acylglucosamine 2-epimerase activity;metabolic process	ec:5.1.3.8-2-epimerase	Amino sugar and nucleotide sugar metabolism
Phvul.006G090800.1	indeterminate-domain 2-like	photosystem II;integral component of membrane;nucleic acid binding;metal ion binding		
Phvul.006G092700.1	uncharacterized GPI-anchored At5g19250-like	integral component of membrane		
Phvul.006G093300.1	cation calcium exchanger 5	plasma membrane;integral component of membrane;nuclear periphery;calcium:sodium antiporter activity;potassium ion transmembrane transporter activity;high-affinity potassium ion import;sodium ion transmembrane transport		
Phvul.006G094000.1	branched-chain-amino-acid aminotransferase 1	mitochondrion;catalytic activity;metabolic process		

Phvul.006G095200.1	sugar transporter ERD6-like 5 isoform X1	integral component of plasma membrane;sugar:proton symporter activity;glucose transmembrane transporter activity;2-alkenal reductase [NAD(P)] activity;proton transport;glucose import;oxidation-reduction process;glucose transmembrane transport	ec:1.3.1.74	
Phvul.006G096100.1	BONZAI 3	calcium-dependent phospholipid binding;negative regulation of cell death		
Phvul.006G096100.2	BONZAI 3	calcium-dependent phospholipid binding;negative regulation of cell death		
Phvul.006G096100.3	BONZAI 3	calcium-dependent phospholipid binding;negative regulation of cell death		
Phvul.006G096300.1	like subfamily B member 14			
Phvul.006G096300.2	like subfamily B member 14			
Phvul.006G096700.1	Transmembrane adipocyte-associated 1	integral component of membrane		
Phvul.006G096700.2	Transmembrane adipocyte-associated 1	integral component of membrane		
Phvul.006G097200.1	translation initiation factor eIF-2B subunit epsilon-like	translation initiation factor activity;nucleotidyltransferase activity;translational initiation		
Phvul.006G097200.2	translation initiation factor eIF-2B subunit epsilon-like	translation initiation factor activity;nucleotidyltransferase activity;translational initiation		
Phvul.006G099200.1	5 -3 exoribonuclease 4-like isoform X1	cytosol;nucleic acid binding;exonuclease activity;zinc ion binding;nuclear-transcribed mRNA catabolic process, exonucleolytic;DNA methylation;chromatin silencing;protein N-linked glycosylation;reciprocal meiotic recombination;cell adhesion;cell-cell signaling;positive regulation of cell proliferation;virus induced gene silencing;gravitropism;unidimensional cell growth;ethylene-activated signaling pathway;meristem initiation;trichome morphogenesis;production of ta-siRNAs involved in RNA interference;glucuronoxylan metabolic process;miRNA catabolic process;deadenylation-independent decapping of nuclear-transcribed mRNA;regulation of chromosome organization;production of miRNAs involved in gene silencing by miRNA;post-translational protein modification;actin nucleation;xylan biosynthetic process;regulation of cell differentiation;positive regulation of transcription, DNA-templated	ec:3.1	
Phvul.006G100200.1	multisubstrate pseudouridine synthase 7	nucleus;RNA binding;pseudouridylate synthase activity;pseudouridine synthase activity;karyogamy;pseudouridine synthesis;embryo sac egg cell differentiation	ec:5.4.99.12;ec:4.2.1.70-synthase	Pyrimidine metabolism
Phvul.006G100800.1	peptide-N(4)-(N-acetyl-beta-glucosaminyl)asparagine amidase	cytosol;integral component of membrane;peptide-N4-(N-acetyl-beta-glucosaminyl)asparagine amidase activity;N-terminal protein myristoylation	ec:3.5.1.52	
Phvul.006G101900.1	mediator of RNA polymerase II transcription subunit 14	plasmodesma;mediator complex;core mediator complex;RNA polymerase II transcription cofactor activity;catalytic activity;coenzyme binding;regulation of transcription from RNA polymerase II promoter;protein glycosylation;positive regulation of cell proliferation;gravitropism		
Phvul.006G103200.1	hypothetical protein PHAVU_006G103200g	nucleic acid binding;zinc ion binding		
Phvul.006G104300.1	pre-mRNA processing PRP39,	integral component of membrane;glycogenin glucosyltransferase activity;metabolic process	ec:2.4.1.186;ec:2.4.1	
Phvul.006G104300.2	pre-mRNA processing PRP39,	integral component of membrane;glycogenin glucosyltransferase activity;metabolic process	ec:2.4.1.186;ec:2.4.1	

Phvul.006G107000.1	translational activator GCN1	nucleus;transcription factor activity, sequence-specific DNA binding;DNA replication;regulation of transcription, DNA-templated		
Phvul.006G108800.1	DUF2358 family			
Phvul.006G109600.1	BTB POZ domain-containing POB1-like	nucleus;Cul3-RING ubiquitin ligase complex;ubiquitin-protein transferase activity;response to red light;protein ubiquitination		
Phvul.006G110500.1	tubulin gamma-2 chain	gamma-tubulin complex;microtubule;GTPase activity;GTP binding;microtubule nucleation;metabolic process;cytoplasmic microtubule organization	ec:3.6.1;ec:3.6.1.15-phosphatase	Purine metabolism;Thiamine metabolism
Phvul.006G110500.2	tubulin gamma-2 chain	gamma-tubulin complex;microtubule;GTPase activity;GTP binding;microtubule nucleation;metabolic process;cytoplasmic microtubule organization	ec:3.6.1;ec:3.6.1.15-phosphatase	Purine metabolism;Thiamine metabolism
Phvul.006G111200.1	extra-large G	membrane		
Phvul.006G111200.2	extra-large G			
Phvul.006G113700.1	ASPARTIC PROTEASE IN GUARD CELL 1	plant-type cell wall;integral component of membrane;aspartic-type endopeptidase activity;proteolysis;plant-type cell wall organization;protein catabolic process;cell wall modification	ec:3.4.23	
Phvul.006G113900.1	defective in meristem silencing	DNA metabolic process;chromatin modification;gene silencing by RNA;regulation of gene expression, epigenetic;single-organism metabolic process;regulation of nitrogen compound metabolic process;cellular response to stimulus;regulation of primary metabolic process;regulation of cellular macromolecule biosynthetic process		
Phvul.006G113900.2	defective in meristem silencing			
Phvul.006G114200.1	exportin-2	nuclear envelope;cytosol;plasmodesma;membrane;nuclear export signal receptor activity;Ran GTPase binding;gluconeogenesis;protein import into nucleus;protein export from nucleus;cytoskeleton organization;response to wounding;response to fungus;jasmonic acid biosynthetic process;response to jasmonic acid;proteasomal protein catabolic process		
Phvul.006G114200.2	exportin-2	nuclear envelope;cytosol;plasmodesma;membrane;nuclear export signal receptor activity;Ran GTPase binding;gluconeogenesis;protein import into nucleus;protein export from nucleus;cytoskeleton organization;response to wounding;response to fungus;jasmonic acid biosynthetic process;response to jasmonic acid;proteasomal protein catabolic process		
Phvul.006G115200.1	bifunctional fucokinase fucose pyrophosphorylase	cytoplasm;ATP binding;fucokinase activity;phosphorylation;cellulose biosynthetic process;GDP-L-fucose salvage;Golgi vesicle transport	ec:2.7.1.52-fucokinase (phosphorylating)	Amino sugar and nucleotide sugar metabolism;Fructose and mannose metabolism
Phvul.006G116100.1	auxin-responsive SAUR32-like	response to auxin		
Phvul.006G116300.1	Rho GTPase-activating gacA	signal transduction		
Phvul.006G118200.1	phytochrome B	nucleus;phosphorelay sensor kinase activity;photoreceptor activity;protein homodimerization activity;phosphorelay signal transduction system;transcription, DNA-templated;regulation of transcription, DNA-templated;detection of visible light;red, far-red light phototransduction;protein-tetrapyrrole linkage;protein-chromophore linkage;signal transduction by protein phosphorylation	ec:2.7.3;ec:2.7.13.3	
Phvul.006G118300.1	Serine threonine-kinase CTR1	intracellular;MAP kinase kinase kinase activity;ATP binding;MAPK cascade;activation	ec:2.7.11;ec:2.7.11.25	

		of MAPKK activity;regulation of transcription, DNA-templated		
Phvul.006G119900.1	nuclear poly(A) polymerase 3	nucleus;cytoplasm;integral component of membrane;RNA binding;polynucleotide adenylyltransferase activity;mRNA polyadenylation	ec:2.7.7.19	
Phvul.006G120300.1	chaperone -domain	integral component of membrane		
Phvul.006G120400.1	F-box At5g49610			
Phvul.006G121000.1	nuclear export mediator factor NEMF-like isoform X1	nucleic acid binding;zinc ion binding;nuclear-transcribed mRNA catabolic process		
Phvul.006G121600.1	YTH domain family 2	integral component of membrane		
Phvul.006G124500.1	patellin-4	nucleus;cytosol;plasma membrane;integral component of membrane;transporter activity;phosphatase activity;transport;sterol biosynthetic process;dephosphorylation;plant-type cell wall cellulose metabolic process;cell wall pectin metabolic process	ec:3.1;ec:3.1.3.41-nitrophenyl phosphatase	Aminobenzoate degradation
Phvul.006G124600.1	reticuline oxidase	integral component of membrane;oxidoreductase activity, acting on CH-OH group of donors;flavin adenine dinucleotide binding;oxidation-reduction process		
Phvul.006G125200.1	midA like, partial	ligase activity;metabolic process		
Phvul.006G125800.1	Reticuline oxidase	oxidoreductase activity, acting on CH-OH group of donors;reticuline oxidase activity;flavin adenine dinucleotide binding;oxidation-reduction process	ec:1.21.3;ec:1.21.3.3-oxidase	Isoquinoline alkaloid biosynthesis
Phvul.006G126800.1	pentatricopeptide repeat-containing At1g51965, mitochondrial	RNA splicing;response to abscisic acid		
Phvul.006G127900.1	probable receptor kinase At1g30570	integral component of membrane;protein serine/threonine kinase activity;non-membrane spanning protein tyrosine kinase activity;ATP binding;response to brassinosteroid;unidimensional cell growth;peptidyl-tyrosine phosphorylation;regulation of unidimensional cell growth	ec:2.7.11;ec:2.7.10;ec:2.7.10.2-protein-tyrosine kinase	T cell receptor signaling pathway
Phvul.006G128000.1	WPP domain-associated			
Phvul.006G128800.1	Lysosomal beta glucosidase	plasma membrane;integral component of membrane;anchored component of membrane;beta-glucosidase activity;glucan catabolic process	ec:3.2.1;ec:3.2.1.21-gentiobiase	Starch and sucrose metabolism;Cyanoamino acid metabolism;Phenylpropanoid biosynthesis
Phvul.006G131100.1	Cysteine desulfurase 2, chloroplastic	pyridoxal phosphate binding;cysteine desulfurase activity;response to reactive oxygen species;selenium compound metabolic process;cysteine metabolic process;aromatic amino acid family biosynthetic process;response to iron ion;response to selenium ion;iron incorporation into metallo-sulfur cluster;iron ion homeostasis	ec:2.8.1.7-desulfurase	Thiamine metabolism
Phvul.006G131500.1	E3 ubiquitin- ligase LIN isoform X1	ligase activity;metabolic process		
Phvul.006G132500.1	FAR1-RELATED SEQUENCE 8	zinc ion binding;regulation of transcription, DNA-templated;vegetative to reproductive phase transition of meristem;protein desumoylation;hydrogen peroxide biosynthetic process		
Phvul.006G132800.1	vesicle transporter Sec20,	integral component of membrane;single-organism process		
Phvul.006G133500.1	DNA-3-methyladenine glycosylase 1	DNA N-glycosylase activity;base-excision repair	ec:3.2.2	
Phvul.006G133800.1	MLO 13	plasma membrane;integral component of membrane;hydrolase activity, hydrolyzing O-glycosyl compounds;calmodulin		

		binding;polysaccharide catabolic process;defense response;response to biotic stimulus		
Phvul.006G135100.1	probable galacturonosyltransferase 4	Golgi membrane;polygalacturonate 4-alpha-galacturonosyltransferase activity;pectin biosynthetic process;cell wall organization	ec:2.4.1.43-4-alpha-galacturonosyltransferase	Amino sugar and nucleotide sugar metabolism;Starch and sucrose metabolism
Phvul.006G135600.1	heavy metal transport detoxification superfamily	cytoplasm;integral component of membrane;transition metal ion binding;metal ion transport;cellular transition metal ion homeostasis		
Phvul.006G136000.1	BTB POZ domain-containing At5g47800-like			
Phvul.006G136200.1	C2 and GRAM domain-containing At1g03370-like	integral component of membrane;phosphatidylserine decarboxylase activity;protein kinase C activity;ligase activity;protein phosphorylation	ec:2.7.11;ec:2.7.11.13-kinase C;ec:4.1.1.65-decarboxylase	Phosphatidylinositol signaling systemGlycerophospholipid metabolism
Phvul.006G137300.1	wall associated kinase	integral component of membrane;protein kinase activity;ATP binding;polysaccharide binding;protein phosphorylation		
Phvul.006G138200.1	Cytosolic Fe-S cluster assembly factor NARFL	nucleus;cytosol;NADH dehydrogenase activity;ferredoxin hydrogenase activity;iron-sulfur cluster binding;mitotic cell cycle;RNA processing;synapsis;reciprocal meiotic recombination;response to xenobiotic stimulus;iron-sulfur cluster assembly;meiotic DNA double-strand break formation;positive regulation of cellular process;oxidation-reduction process;response to oxygen levels	ec:1.12.7;ec:1.6.99.3-dehydrogenase;ec:1.12.7.2	Oxidative phosphorylation
Phvul.006G138500.1	TRNA--methyltransferase isoform 1	methyltransferase activity;tRNA modification;methylation;1-methylguanosine metabolic process		
Phvul.006G141000.1	mediator of RNA polymerase II transcription subunit 2-like			
Phvul.006G141900.1	thymidine kinase-like	thymidine kinase activity;ATP binding;deoxyribonucleoside monophosphate biosynthetic process;phosphorylation;DNA biosynthetic process	ec:2.7.1.21-kinase;ec:2.7.1.145	Drug metabolism - other enzymes;Pyrimidine metabolism
Phvul.006G142400.1	Inositol monophosphatase 3	cytosol;plasma membrane;integral component of membrane;magnesium ion binding;inositol monophosphate 1-phosphatase activity;L-galactose-1-phosphate phosphatase activity;inositol monophosphate 3-phosphatase activity;inositol monophosphate 4-phosphatase activity;inositol metabolic process;signal transduction;response to cold;myo-inositol hexakisphosphate biosynthetic process;methylglyoxal catabolic process to D-lactate via S-lactoyl-glutathione;glucosinolate biosynthetic process;L-ascorbic acid biosynthetic process;phosphatidylinositol phosphorylation;inositol phosphate dephosphorylation	ec:3.1.3;ec:3.1;ec:3.1.3.41-nitrophenyl phosphatase;ec:3.1.3.25-phosphatase;ec:3.1.3.23	Aminobenzoate degradationBiosynthesis of antibiotics;Streptomycin biosynthesis;Inositol phosphate metabolism;Phosphatidylinositol signaling system
Phvul.006G142400.2	Inositol monophosphatase 3	cytosol;plasma membrane;integral component of membrane;magnesium ion binding;inositol monophosphate 1-phosphatase activity;L-galactose-1-phosphate phosphatase activity;inositol monophosphate 3-phosphatase activity;inositol monophosphate 4-phosphatase activity;inositol metabolic process;signal transduction;response to cold;myo-inositol hexakisphosphate biosynthetic process;methylglyoxal catabolic process to D-lactate via S-lactoyl-glutathione;glucosinolate biosynthetic process;L-ascorbic acid biosynthetic process;phosphatidylinositol phosphorylation;inositol phosphate dephosphorylation	ec:3.1.3;ec:3.1;ec:3.1.3.41-nitrophenyl phosphatase;ec:3.1.3.25-phosphatase;ec:3.1.3.23	Aminobenzoate degradationBiosynthesis of antibiotics;Streptomycin biosynthesis;Inositol phosphate metabolism;Phosphatidylinositol signaling system
Phvul.006G143100.1	Rho GDP-dissociation inhibitor 1	cytoplasm;integral component of membrane;Rho GDP-dissociation inhibitor activity;regulation of catalytic activity		

Phvul.006G143600.1	Transcription initiation factor IIF subunit alpha	nucleus;photosystem I;integral component of membrane;DNA binding;translation initiation factor activity;catalytic activity;protein C-terminus binding;transcription initiation from RNA polymerase II promoter;translational initiation;photosynthesis;positive regulation of transcription elongation from RNA polymerase II promoter		
Phvul.006G143900.1	hypothetical protein PHAVU_006G143900g			
Phvul.006G145500.1	P-loop nucleoside triphosphate hydrolase superfamily	proteasome regulatory particle, base subcomplex;nuclear proteasome complex;cytosolic proteasome complex;ATP binding;TBP-class protein binding;proteasome-activating ATPase activity;ER-associated ubiquitin-dependent protein catabolic process;positive regulation of RNA polymerase II transcriptional preinitiation complex assembly;cell division;positive regulation of proteasomal protein catabolic process	ec:3.6.1;ec:3.6.1.3-adenylpyrophosphatase;ec:3.6.1.15-phosphatase	Purine metabolismPurine metabolism;Thiamine metabolism
Phvul.006G145900.1	39S ribosomal L46, mitochondrial	mitochondrial large ribosomal subunit;structural constituent of ribosome;regulation of flower development;histone lysine methylation		
Phvul.006G146200.1	malonyl-decarboxylase, mitochondrial	peroxisome;malonyl-CoA decarboxylase activity;fatty acid biosynthetic process	ec:4.1.1.9-decarboxylase	beta-Alanine metabolism;Propanoate metabolism
Phvul.006G147100.1	proline synthase co-transcribed bacterial homolog -like	pyridoxal phosphate binding;hydrogen peroxide catabolic process		
Phvul.006G149800.1	Myb O	proton-transporting ATP synthase complex, catalytic core F(1);DNA binding;proton-transporting ATP synthase activity, rotational mechanism;proton-transporting ATPase activity, rotational mechanism;anatomical structure morphogenesis;positive regulation of organelle organization;ATP synthesis coupled proton transport;cellular component organization;cellular macromolecule metabolic process;cell development;system development;cell wall organization or biogenesis;plant epidermis development	ec:3.6.1;ec:3.6.1.3-adenylpyrophosphatase;ec:3.6.1.15-phosphatase	Purine metabolismPurine metabolism;Thiamine metabolism
Phvul.006G151100.1	adenosine deaminase	adenosine deaminase activity;adenosine catabolic process;hypoxanthine salvage;inosine biosynthetic process	ec:3.5.4.4-deaminase	Purine metabolism
Phvul.006G151300.1	beta-glucosidase 47-like	integral component of membrane;beta-glucosidase activity;carbohydrate metabolic process	ec:3.2.1;ec:3.2.1.21-gentiobiase	Starch and sucrose metabolism;Cyanoamino acid metabolism;Phenylpropanoid biosynthesis
Phvul.006G152100.1	trichome birefringence-like 10	Golgi apparatus;integral component of membrane;O-acetyltransferase activity;cell wall organization or biogenesis	ec:2.3.1	
Phvul.006G153600.1	probable E3 ubiquitin- ligase LUL4	integral component of membrane;zinc ion binding		
Phvul.006G153800.1	RING zinc finger ,			
Phvul.006G154200.1	adenylate isopentenyltransferase	mitochondrion;AMP dimethylallyltransferase activity;tRNA dimethylallyltransferase activity;tRNA modification	ec:2.5.1.75-dimethylallyltransferase;ec:2.5.1.27-dimethylallyltransferase	Zeatin biosynthesisZeatin biosynthesis
Phvul.006G154400.1	probable serine incorporator	integral component of membrane;L-serine transmembrane transporter activity;L-serine transport		
Phvul.006G154500.1	transmembrane ,	mitochondrion;integral component of membrane;cytosolic large ribosomal subunit;RNA binding;structural constituent of ribosome;translation;ribosome biogenesis		
Phvul.006G158600.1	hypothetical protein			

	PHAVU_006G158600g			
Phvul.006G159700.1	squamosa promoter-binding 7	nucleus;integral component of membrane;DNA binding		
Phvul.006G160300.1	BRCT domain DNA repair			
Phvul.006G160500.1	serine threonine-kinase ULK4	plasmodesma;protein kinase activity;ATP binding;microtubule binding;phragmoplast assembly;protein phosphorylation;endosperm cellularization		
Phvul.006G162900.1	anaphase-promoting complex subunit 7	mitotic cell cycle;RNA processing;sister chromatid cohesion;reciprocal meiotic recombination;gamete generation;positive regulation of cell proliferation;response to xenobiotic stimulus;photomorphogenesis;response to gamma radiation;cullin deneddylation;glucuronoxylan metabolic process;histone methylation;protein deubiquitination;chromatin silencing by small RNA;regulation of telomere maintenance;regulation of DNA endoreduplication;DNA endoreduplication;meiotic DNA double-strand break formation;proteasome-mediated ubiquitin-dependent protein catabolic process;telomere maintenance in response to DNA damage;proteasome assembly;post-translational protein modification;meiotic chromosome segregation;xylan biosynthetic process;positive regulation of transcription, DNA-templated;regulation of cell division;regulation of unidimensional cell growth;response to misfolded protein		
Phvul.006G165500.1	F-box kelch-repeat At1g30090			
Phvul.006G165600.1	pyruvate dehydrogenase E1 component subunit beta-3, chloroplastic-like	pyruvate dehydrogenase (acetyl-transferring) activity;oxidation-reduction process	ec:1.2.4.1-dehydrogenase (acetyl-transferring)	Pyruvate metabolism;Biosynthesis of antibiotics;Citrate cycle (TCA cycle);Glycolysis / Gluconeogenesis
Phvul.006G166300.1	Mitochondria isoform 1	mitochondrion		
Phvul.006G166300.2	Mitochondria isoform 1	mitochondrion		
Phvul.006G166300.3	Mitochondria isoform 1	mitochondrion		
Phvul.006G166900.1	F-box SKIP19-like			
Phvul.006G167200.1	BREAST CANCER SUSCEPTIBILITY 1 homolog isoform X1	zinc ion binding;ligase activity;DNA repair		
Phvul.006G167200.2	BREAST CANCER SUSCEPTIBILITY 1 homolog isoform X1	zinc ion binding;ligase activity;DNA repair		
Phvul.006G167200.3	BREAST CANCER SUSCEPTIBILITY 1 homolog isoform X2	zinc ion binding;ligase activity;DNA repair		
Phvul.006G167200.4	BREAST CANCER SUSCEPTIBILITY 1 homolog isoform X2	zinc ion binding;ligase activity;DNA repair		
Phvul.006G167500.1	nucleoporin Ndc1-Nup	integral component of membrane;DNA binding		
Phvul.006G168000.1	BTB POZ domain-containing At5g03250-like			
Phvul.006G168000.2	BTB POZ domain-containing At5g03250-like			
Phvul.006G168000.3	BTB POZ domain-containing At5g03250-like			

Phvul.006G168000.4	BTB POZ domain-containing At5g03250-like			
Phvul.006G168000.5	BTB POZ domain-containing At5g03250-like			
Phvul.006G168100.1	PLASTID TRANSCRIPTIONALLY ACTIVE 12	nucleoid;plastid;single-organism process		
Phvul.006G168500.1	Transcription factor GTE12			
Phvul.006G168500.2	Transcription factor GTE12			
Phvul.006G172000.1	ACCELERATED CELL DEATH 6-like	integral component of membrane		
Phvul.006G172000.2	ACCELERATED CELL DEATH 6-like	integral component of membrane		
Phvul.006G173100.1	hypothetical protein PHAVU_006G173100g	integral component of membrane		
Phvul.006G174300.1	RING FYVE PHD zinc finger	integral component of membrane;zinc ion binding		
Phvul.006G174400.1	probable LRR receptor-like serine threonine- kinase At5g45780 isoform X1	integral component of membrane;protein serine/threonine kinase activity;transmembrane receptor protein tyrosine kinase activity;ATP binding;embryo sac egg cell differentiation;peptidyl-tyrosine phosphorylation	ec:2.7.11;ec:2.7.10;ec:2.7.10.1	
Phvul.006G174400.2	probable LRR receptor-like serine threonine- kinase At5g45780 isoform X1	integral component of membrane;protein serine/threonine kinase activity;transmembrane receptor protein tyrosine kinase activity;ATP binding;embryo sac egg cell differentiation;peptidyl-tyrosine phosphorylation	ec:2.7.11;ec:2.7.10;ec:2.7.10.1	
Phvul.006G177100.1	trichome birefringence-like 38	Golgi apparatus;O-acetyltransferase activity;cell wall organization or biogenesis	ec:2.3.1	
Phvul.006G177300.1	phospholipase A1-IIgamma-like	phosphatidylcholine 1-acylhydrolase activity;phosphatidylserine 1-acylhydrolase activity;1-acyl-2-lysophosphatidylserine acylhydrolase activity;lipid metabolic process	ec:3.1.1.32-A1;ec:3.1.1;ec:3.1.1.1-ali-esterase	alpha-Linolenic acid metabolism;Glycerophospholipid metabolismDrug metabolism - other enzymes
Phvul.006G178700.1	IQ-DOMAIN 1	plasma membrane;RNA splicing, via endonucleolytic cleavage and ligation;methionine biosynthetic process;photoperiodism, flowering		
Phvul.006G179000.1	probable ATP-dependent RNA helicase DHX35	spliceosomal complex;ATP-dependent RNA helicase activity;ATP binding;poly(A) RNA binding;RNA processing	ec:3.6.1;ec:3.6.1.3-adenylpyrophosphatase;ec:3.6.1.15-phosphatase;ec:2.7.7	Purine metabolismPurine metabolism;Thiamine metabolism
Phvul.006G179600.1	GDSL esterase lipase At5g45960-like	integral component of membrane;hydrolase activity, acting on ester bonds;9,9'-dicis-carotene:quinone oxidoreductase activity;7,9,9'-tricis-neurosporene:quinone oxidoreductase activity;9,9'-di-cis-zeta-carotene desaturation to 7,9,7',9'-tetra-cis-lycopene;oxidation-reduction process	ec:1.3.5.6-desaturase	Carotenoid biosynthesis
Phvul.006G179800.1	pathogenesis-related genes transcriptional activator PTI5-like	nucleus;DNA binding;transcription factor activity, sequence-specific DNA binding;transcription, DNA-templated;regulation of transcription, DNA-templated		
Phvul.006G180000.1	ras-related RABA1f-like	intracellular;membrane;GTPase activity;GTP binding;intracellular protein transport;nucleocytoplasmic transport;small GTPase mediated signal transduction;metabolic process;vesicle-mediated transport	ec:3.6.1;ec:3.6.1.15-phosphatase	Purine metabolism;Thiamine metabolism
Phvul.006G180200.1	probable leucine-rich repeat receptor kinase At5g49770	integral component of membrane;protein serine/threonine kinase activity;transmembrane receptor protein tyrosine kinase activity;ATP binding;oxidoreductase activity;peptidyl-	ec:2.7.11;ec:2.7.10;ec:2.7.10.1	

		tyrosine phosphorylation;single-organism metabolic process;regulation of cellular process		
Phvul.006G180400.1	ribosomal L31	ribosome		
Phvul.006G181400.2	guanylate-binding family	GTPase activity;GTP binding;metabolic process	ec:3.6.1;ec:3.6.1.15-phosphatase	Purine metabolism;Thiamine metabolism
Phvul.006G181400.3	guanylate-binding family	GTPase activity;GTP binding;metabolic process	ec:3.6.1;ec:3.6.1.15-phosphatase	Purine metabolism;Thiamine metabolism
Phvul.006G181900.1	transmembrane ,	integral component of membrane		
Phvul.006G183400.1	Zinc finger C-x8-C-x5-C-x3-H type family , isoform 1	metal ion binding		
Phvul.006G183800.1	glyoxysomal processing protease, glyoxysomal	peroxisome;serine-type endopeptidase activity;protein processing	ec:3.4.21	
Phvul.006G184400.1	VQ motif-containing 4	membrane		
Phvul.006G188200.1	splicing factor 3B subunit 2	U2-type spliceosomal complex;U2 snRNP;U12-type spliceosomal complex;chloroplast;precatalytic spliceosome;catalytic step 2 spliceosome;mRNA splicing, via spliceosome		
Phvul.006G188500.1	Polyphenol oxidase A1, chloroplastic	catechol oxidase activity;metal ion binding;pigment biosynthetic process;oxidation-reduction process	ec:1.10.3.1-oxidase;ec:1.10.3	Isoquinoline alkaloid biosynthesis;Tyrosine metabolism
Phvul.006G189100.1	serine carboxypeptidase-like 31	integral component of membrane;serine-type carboxypeptidase activity;proteolysis involved in cellular protein catabolic process	ec:3.4.21;ec:3.4.16	
Phvul.006G189700.1	BEST plant match is: (TAIR:) ,			
Phvul.006G189900.1	katanin p80 WD40 repeat-containing subunit B1 homolog isoform X1	microtubule;katanin complex;microtubule binding;microtubule severing		
Phvul.006G190800.1	60S ribosomal L26-1	cytosolic large ribosomal subunit;structural constituent of ribosome;cytoplasmic translation;ribosomal large subunit biogenesis		
Phvul.006G191100.1	pre-mRNA-splicing factor SYF1	Prp19 complex;prespliceosome;catalytic step 1 spliceosome;catalytic step 2 spliceosome;post-mRNA release spliceosomal complex;generation of catalytic spliceosome for first transesterification step		
Phvul.006G191500.1	pre-mRNA-splicing factor SYF1	Prp19 complex;prespliceosome;catalytic step 1 spliceosome;catalytic step 2 spliceosome;post-mRNA release spliceosomal complex;generation of catalytic spliceosome for first transesterification step		
Phvul.006G191800.1	sphingosine kinase 1 isoform X1	ceramide kinase activity;sphinganine kinase activity;lipid phosphorylation	ec:2.7.1.91-kinase;ec:2.7.1.138-kinase	Sphingolipid metabolismSphingolipid metabolism
Phvul.006G192200.1	heterogeneous nuclear ribonucleo U 1 isoform X1			
Phvul.006G192200.2	heterogeneous nuclear ribonucleo U 1 isoform X1			
Phvul.006G194300.1	G-type lectin S-receptor-like Serine Threonine-kinase	plasma membrane;plasmodesma;integral component of membrane;protein serine/threonine kinase activity;ATP binding;carbohydrate binding;ubiquitin protein ligase binding;protein phosphorylation;innate immune response;recognition of pollen	ec:2.7.11	
Phvul.006G196400.1	kinesin motor catalytic domain	kinesin complex;microtubule;microtubule motor activity;ATP binding;microtubule binding;ATPase activity;microtubule-based movement;metabolic process	ec:3.6.1;ec:3.6.1.3-adenylpyrophosphatase;ec:3.6.1.15-phosphatase	Purine metabolismPurine metabolism;Thiamine metabolism
Phvul.006G196800.1	salt stress response antifungal domain	integral component of membrane;kinase activity;phosphorylation		
Phvul.006G197000.1	probably inactive leucine-rich repeat	integral component of membrane;transmembrane receptor protein	ec:2.7.11;ec:2.7.10;ec:2.7.10.1	

	receptor kinase At5g48380	tyrosine kinase activity;ATP binding;peptidyl-tyrosine phosphorylation		
Phvul.006G198200.1	receptor kinase HAIKU2	integral component of membrane;protein serine/threonine kinase activity;ATP binding;peptidase activity;oxidoreductase activity;protein phosphorylation;endosperm development	ec:2.7.11	
Phvul.006G198600.1	probable sphingolipid transporter spinster homolog 2	integral component of membrane;transmembrane transport		
Phvul.006G199000.1	phosphoglycerate mutase 1 isoform X1	cytosol;sedoheptulose-bisphosphatase activity;cytokinin-activated signaling pathway;dephosphorylation	ec:3.1;ec:3.1.3.41-nitrophenyl phosphatase;ec:3.1.3.37-SBPase	Aminobenzoate degradationCarbon fixation in photosynthetic organisms
Phvul.006G200700.1	U3 small nucleolar RNA-associated 18 homolog	small-subunit processome;Pwp2p-containing subcomplex of 90S preribosome		
Phvul.006G206300.1	probable phosphatase 2C 9	protein serine/threonine phosphatase activity;protein dephosphorylation	ec:3.1;ec:3.1.3.41-nitrophenyl phosphatase;ec:3.1.3.16-phosphatase	Aminobenzoate degradationT cell receptor signaling pathway
Phvul.006G206300.2	probable phosphatase 2C 9	protein serine/threonine phosphatase activity;protein dephosphorylation	ec:3.1;ec:3.1.3.41-nitrophenyl phosphatase;ec:3.1.3.16-phosphatase	Aminobenzoate degradationT cell receptor signaling pathway
Phvul.006G206400.1	calmodulin-binding transcription activator 2	nucleus;integral component of membrane;transcriptional activator activity, RNA polymerase II core promoter proximal region sequence-specific binding;ligase activity;sequence-specific DNA binding;transcription from RNA polymerase II promoter;positive regulation of transcription from RNA polymerase II promoter		
Phvul.006G206600.1	potassium transporter 7	plant-type vacuole;vacuolar membrane;integral component of membrane;potassium ion transmembrane transporter activity;potassium ion transmembrane transport		
Phvul.006G206800.1	pumilio homolog 7, chloroplastic	RNA binding		
Phvul.006G207300.1	chlorophyll a-b binding CP24 10A, chloroplastic	chloroplast thylakoid membrane;photosystem II antenna complex;chloroplast envelope;plastoglobule;integral component of membrane;chlorophyll binding;pigment binding;calcium ion transport;Golgi organization;response to salt stress;photosynthesis, light harvesting in photosystem I;nonphotochemical quenching;protein-chromophore linkage;cysteine biosynthetic process;regulation of protein dephosphorylation		
Phvul.006G208400.1	UDP-glucosyltransferase family	intracellular membrane-bounded organelle;quercetin 3-O-glucosyltransferase activity;quercetin 7-O-glucosyltransferase activity;flavonoid biosynthetic process;flavonoid glucuronidation	ec:2.4.1	
Phvul.006G209700.1	cytochrome P450 family 71	integral component of membrane;iron ion binding;oxidoreductase activity, acting on paired donors, with incorporation or reduction of molecular oxygen, NAD(P)H as one donor, and incorporation of one atom of oxygen;heme binding;secondary metabolite biosynthetic process;oxidation-reduction process	ec:1.14.13	
Phvul.006G210100.1	WAT1-related At1g68170-like	plasma membrane;integral component of membrane;transmembrane transporter activity;transmembrane transport		
Phvul.006G212300.1	transmembrane 87B	Golgi apparatus;integral component of membrane;acetyl-CoA metabolic process;sterol biosynthetic process;brassinosteroid biosynthetic process		
Phvul.006G212600.1	SUPPRESSOR OF GENE SILENCING 3-like	nucleolar ribonuclease P complex;ribonuclease P activity;gene silencing by RNA;RNA	ec:3.1.30;ec:3.1.26;ec:3.1;ec:3.1.26.5	

		phosphodiester bond hydrolysis, endonucleolytic		
Phvul.006G214800.1	alpha-ketoglutarate-dependent dioxygenase ABH5	dioxygenase activity;oxidation-reduction process		
Phvul.006G215100.1	cell division control 48 homolog C	integral component of membrane;ATP binding;mitotic cell cycle;mRNA splicing, via spliceosome;photomorphogenesis;cullin deneddylation;protein ubiquitination;histone methylation;protein deubiquitination;production of siRNA involved in RNA interference;production of miRNAs involved in gene silencing by miRNA;post-translational protein modification;positive regulation of transcription, DNA-templated;cell division		
Phvul.006G215200.1	importin subunit beta-4	cytosol;nuclear membrane;nuclear periphery;nuclear localization sequence binding;Ran GTPase binding;protein transporter activity;protein import into nucleus, docking;protein import into nucleus, translocation;gluconeogenesis;NLS-bearing protein import into nucleus;ribosomal protein import into nucleus;cytoskeleton organization;proteasomal protein catabolic process		
Phvul.006G215700.1	potassium channel AKT2 3	plasmodesma;integral component of membrane;voltage-gated potassium channel activity;identical protein binding;myo-inositol hexakisphosphate biosynthetic process;regulation of membrane potential;potassium ion transmembrane transport		
Phvul.006G215900.1	DUF793 family	integral component of membrane		
Phvul.006G216100.1	T-complex 11 1	plasmodesma		
Phvul.006G216200.1	ATP-dependent DNA helicase Q-like 4A	intracellular;nucleic acid binding;ATP binding;ATP-dependent 3'-5' DNA helicase activity;DNA replication;DNA repair;DNA recombination;DNA duplex unwinding	ec:3.6.1;ec:3.6.1.3-adenylpyrophosphatase;ec:3.6.1.15-phosphatase	Purine metabolismPurine metabolism;Thiamine metabolism
Phvul.006G216900.1	MLO 4	plasma membrane;integral component of membrane;calmodulin binding;defense response;response to biotic stimulus		
Phvul.006G216900.2	MLO 4	plasma membrane;integral component of membrane;calmodulin binding;defense response;response to biotic stimulus		
Phvul.006G218600.1	Lactoylglutathione lyase glyoxalase I family	integral component of membrane;transferase activity;lyase activity;metabolic process		
Phvul.006G219400.1	octicosapeptide Phox Bem1p domain-containing			
Phvul.006G220700.1	ABC transporter F family member 4	ATP binding;organic phosphonate transmembrane-transporting ATPase activity;organic phosphonate transport;organophosphate ester transport;methylglyoxal catabolic process to D-lactate via S-lactoyl-glutathione	ec:3.6.1;ec:3.6.1.3-adenylpyrophosphatase;ec:3.6.1.15-phosphatase;ec:3.6.3.28	Purine metabolismPurine metabolism;Thiamine metabolism
Phvul.006G220700.2	ABC transporter F family member 4	ATP binding;organic phosphonate transmembrane-transporting ATPase activity;organic phosphonate transport;organophosphate ester transport;methylglyoxal catabolic process to D-lactate via S-lactoyl-glutathione	ec:3.6.1;ec:3.6.1.3-adenylpyrophosphatase;ec:3.6.1.15-phosphatase;ec:3.6.3.28	Purine metabolismPurine metabolism;Thiamine metabolism
Phvul.006G221100.1	pentatricopeptide repeat-containing At1g10270-like			
Phvul.006G221300.1	zinc finger CCCH domain-containing 5	nucleus;nucleotide binding;RNA binding;metal ion binding		
Phvul.007G002100.1	DEAD-box helicase family	helicase activity;metabolic process	ec:3.6.1;ec:3.6.1.15-phosphatase	Purine metabolism;Thiamine metabolism

Phvul.007G002300.1	sn1-specific diacylglycerol lipase beta	intracellular;integral component of membrane;hydrolase activity;fatty acid beta-oxidation;protein import into peroxisome matrix		
Phvul.007G003000.1	non-structural maintenance of chromosomes element 4 homolog A-like	nucleus;Smc5-Smc6 complex;DNA repair		
Phvul.007G004700.1	nucleolar complex 2 homolog	peroxidase activity;cellular oxidant detoxification	ec:1.11.1.7-lactoperoxidase	Phenylpropanoid biosynthesis
Phvul.007G005300.1	hypothetical protein PHAVU_007G005300g			
Phvul.007G005700.1	probable phosphatase 2C 38	integral component of membrane;protein serine/threonine phosphatase activity;metal ion binding;protein dephosphorylation	ec:3.1;ec:3.1.3.41-nitrophenyl phosphatase;ec:3.1.3.16-phosphatase	Aminobenzoate degradationT cell receptor signaling pathway
Phvul.007G005900.1	leukotriene A-4 hydrolase homolog	cytosol;plasma membrane;leukotriene-A4 hydrolase activity;zinc ion binding;peptide binding;metalloaminopeptidase activity;DNA replication;DNA methylation;proteolysis;cell proliferation;leukotriene biosynthetic process;peptide catabolic process;histone H3-K9 methylation	ec:3.3.2.6-hydrolase;ec:3.4.11	Arachidonic acid metabolism
Phvul.007G007900.1	sucrose-phosphate synthase 2			
Phvul.007G007900.2	sucrose-phosphate synthase 2			
Phvul.007G014400.1	cyclic pyranopterin monophosphate synthase accessory , mitochondrial	chloroplast;cyclic pyranopterin monophosphate synthase activity;Mo-molybdopterin cofactor biosynthetic process	ec:4.1.99.18-pyranopterin phosphate synthase	Folate biosynthesis
Phvul.007G016500.1	ACT-like tyrosine kinase family	cytosol;protein serine/threonine/tyrosine kinase activity;protein tyrosine kinase activity;ATP binding;amino acid binding;actin filament organization;chloroplast organization;peptidyl-tyrosine phosphorylation	ec:2.7.10;ec:2.7.12.1	
Phvul.007G016500.2	ACT-like tyrosine kinase family	cytosol;protein serine/threonine/tyrosine kinase activity;protein tyrosine kinase activity;ATP binding;amino acid binding;actin filament organization;chloroplast organization;peptidyl-tyrosine phosphorylation	ec:2.7.10;ec:2.7.12.1	
Phvul.007G017100.1	FAD NAD(P)-binding oxidoreductase family	zeaxanthin epoxidase [overall] activity;zeaxanthin epoxidase activity;antheraxanthin epoxidase activity;FAD binding;secondary metabolite biosynthetic process;oxidation-reduction process	ec:1.14.13.90-epoxidase;ec:1.14.13	Carotenoid biosynthesis
Phvul.007G018200.1	DUF630 family			
Phvul.007G018200.2	DUF630 family			
Phvul.007G018800.1	probable ubiquitin-conjugating enzyme E2 23	cytosol;ligase activity;ubiquitin protein ligase binding;ubiquitin protein ligase activity;postreplication repair;protein ubiquitination		
Phvul.007G019900.1	Myb family transcription factor At1g14600	nucleus;DNA binding;transcription, DNA-templated;regulation of transcription, DNA-templated		
Phvul.007G020200.1	DUF615 family			
Phvul.007G020300.1	transport Sec24-like At4g32640	COPII vesicle coat;zinc ion binding;intracellular protein transport;ER to Golgi vesicle-mediated transport		
Phvul.007G020500.1	ACCUMULATION AND REPLICATION OF CHLOROPLASTS 3	extrinsic component of plastid membrane;GTPase activity;1-phosphatidylinositol-4-phosphate 5-kinase activity;protein self-association;chloroplast fission;phosphatidylinositol phosphorylation	ec:3.6.1;ec:3.6.1.15-phosphatase;ec:2.7.1.68-5-kinase;ec:2.7.1	Purine metabolism;Thiamine metabolismInositol phosphate metabolism;Phosphatidylinositol signaling system
Phvul.007G021700.1	Germin subfamily 3 member 2	extracellular region;manganese ion binding;nutrient reservoir activity		

Phvul.007G022800.1	ribonuclease H2 subunit A	ribonuclease H2 complex;RNA binding;RNA-DNA hybrid ribonuclease activity;mismatch repair;DNA replication, removal of RNA primer;RNA phosphodiester bond hydrolysis, endonucleolytic	ec:3.1.30;ec:3.1.26;ec:3.1;ec:3.1.26.4	
Phvul.007G024600.1	RNA-directed DNA methylation 3-like isoform X2	ribosome;structural constituent of ribosome;translation elongation factor activity;translational elongation;nucleic acid metabolic process		
Phvul.007G024700.1	NUCLEAR FUSION DEFECTIVE 4-like	integral component of membrane;transmembrane transport		
Phvul.007G026100.1	Serine Threonine kinase domain	protein kinase activity;ATP binding;protein phosphorylation		
Phvul.007G026100.2	Serine Threonine kinase domain	protein kinase activity;ATP binding;protein phosphorylation		
Phvul.007G026900.1	alpha-xylosidase 1-like	alpha-1,4-glucosidase activity;carbohydrate binding;maltose alpha-glucosidase activity;maltose metabolic process	ec:3.2.1;ec:3.2.1.20-maltase	Starch and sucrose metabolism;Galactose metabolism
Phvul.007G027600.1	50S ribosomal L15, chloroplastic	plastid large ribosomal subunit;chloroplast stroma;chloroplast envelope;structural constituent of ribosome;DNA-templated transcription, elongation;translation;response to stress		
Phvul.007G028000.1	caffeoylshikimate esterase-like	membrane;acylglycerol lipase activity;lipid metabolic process	ec:3.1.1.23-lipase;ec:3.1.1;ec:3.1.1.1-ali-esterase	Glycerolipid metabolismDrug metabolism - other enzymes
Phvul.007G028000.2	caffeoylshikimate esterase-like	membrane;acylglycerol lipase activity;lipid metabolic process	ec:3.1.1.23-lipase;ec:3.1.1;ec:3.1.1.1-ali-esterase	Glycerolipid metabolismDrug metabolism - other enzymes
Phvul.007G031100.1	serine threonine-kinase	integral component of membrane;protein serine/threonine kinase activity;ATP binding;polysaccharide binding;protein phosphorylation	ec:2.7.11	
Phvul.007G032200.1	auxin-responsive IAA32	nucleus;transcription, DNA-templated;regulation of transcription, DNA-templated;auxin-activated signaling pathway		
Phvul.007G034400.1	Grap2 cyclin-D-interacting	nucleus;regulation of cell cycle		
Phvul.007G035200.1	pentatricopeptide repeat-containing At5g04780	zinc ion binding;microtubule-severing ATPase activity;metabolic process	ec:3.6.1;ec:3.6.1.3-adenylpyrophosphatase;ec:3.6.4.3;ec:3.6.1.15-phosphatase	Purine metabolismPurine metabolism;Thiamine metabolism
Phvul.007G035700.1	repressor of RNA polymerase III transcription MAF1 homolog	nucleus;cytoplasm;RNA polymerase III core binding;transcription, DNA-templated;negative regulation of transcription from RNA polymerase III promoter;transcription factor TFIIIB complex assembly		
Phvul.007G035800.1	hypothetical protein PHAVU_007G035800g			
Phvul.007G036400.1	hypothetical protein PHAVU_007G036400g, partial			
Phvul.007G036900.1	transmembrane ,	integral component of membrane		
Phvul.007G038000.1	heavy metal-associated domain	cytoplasm;transition metal ion binding;metal ion transport;cellular transition metal ion homeostasis		
Phvul.007G038300.1	phosphatidylinositol 3,4,5-trisphosphate 3-phosphatase and dual-specificity phosphatase PTEN	cytosol;protein tyrosine phosphatase activity;phosphatidylinositol-3,4,5-trisphosphate 3-phosphatase activity;phosphatidic acid binding;peptidyl-tyrosine dephosphorylation;negative regulation of MAP kinase activity;phosphatidylinositol dephosphorylation;Golgi vesicle transport	ec:3.1.3.67-3-phosphatase;ec:3.1.3.48;ec:3.1;ec:3.1.3.41-nitrophenyl phosphatase;ec:3.1.3.16-phosphatase	Inositol phosphate metabolism;Phosphatidylinositol signaling systemAminobenzoate degradationT cell receptor signaling pathway
Phvul.007G039200.1	Limonoid UDP-glucosyltransferase	intracellular membrane-bounded organelle;quercetin 3-O-glucosyltransferase activity;quercetin 7-O-glucosyltransferase activity;flavonoid biosynthetic process;flavonoid glucuronidation	ec:2.4.1	

Phvul.007G040400.1	receptor kinase At4g34220	integral component of membrane;transmembrane receptor protein tyrosine kinase activity;ATP binding;peptidyl-tyrosine phosphorylation	ec:2.7.11;ec:2.7.10;ec:2.7.10.1	
Phvul.007G040700.1	methylthioribose kinase	cytosol;identical protein binding;S-methyl-5-thioribose kinase activity;S-methyl-5-thioribose-1-phosphate isomerase activity;methionine biosynthetic process;phosphorylation	ec:2.7.1.100-kinase;ec:5.3.1.23-isomerase	Cysteine and methionine metabolismCysteine and methionine metabolism
Phvul.007G041000.1	plant basic secretory (BSP) family			
Phvul.007G041800.1	homeobox KN domain	nucleus;DNA binding;zinc ion binding;positive regulation of cell proliferation;embryo sac egg cell differentiation;post-translational protein modification;positive regulation of transcription, DNA-templated;chromosome organization		
Phvul.007G046400.1	nucleobase-ascorbate transporter 4	integral component of membrane;transporter activity;transmembrane transport		
Phvul.007G047600.1	pyruvate kinase isozyme A, chloroplastic-like	magnesium ion binding;pyruvate kinase activity;kinase activity;potassium ion binding;glycolytic process	ec:2.7.1.40-kinase	Purine metabolism;Pyruvate metabolism;Biosynthesis of antibiotics;Glycolysis / Gluconeogenesis
Phvul.007G048200.1	dentin sialophospho-like	integral component of membrane		
Phvul.007G048900.1	receptor kinase At4g00960	plasma membrane;plasmodesma;integral component of membrane;protein serine/threonine kinase activity;ATP binding;protein phosphorylation;defense response to bacterium	ec:2.7.11	
Phvul.007G050600.1	cysteine-rich receptor kinase 10	plasma membrane;plasmodesma;integral component of membrane;protein serine/threonine kinase activity;transmembrane receptor protein tyrosine kinase activity;ATP binding;defense response;peptidyl-tyrosine phosphorylation	ec:2.7.11;ec:2.7.10;ec:2.7.10.1	
Phvul.007G051000.1	cysteine-rich receptor-kinase	plasma membrane;plasmodesma;integral component of membrane;protein serine/threonine kinase activity;non-membrane spanning protein tyrosine kinase activity;ATP binding;defense response;peptidyl-tyrosine phosphorylation	ec:2.7.11;ec:2.7.10;ec:2.7.10.2-protein-tyrosine kinase	T cell receptor signaling pathway
Phvul.007G054000.1	probable ATP-dependent DNA helicase CHR12 isoform X2	helicase activity;ATP binding;histone binding;metabolic process	ec:3.6.1;ec:3.6.1.15-phosphatase	Purine metabolism;Thiamine metabolism
Phvul.007G054600.1	probable LRR receptor-like serine threonine- kinase At1g67720	kinase activity;phosphorylation		
Phvul.007G054800.1	amino-terminal domain cyclin	cyclin-dependent protein kinase holoenzyme complex;nucleus;cyclin-dependent protein serine/threonine kinase regulator activity;positive regulation of cyclin-dependent protein serine/threonine kinase activity;positive regulation of transcription from RNA polymerase II promoter;positive regulation of phosphorylation of RNA polymerase II C-terminal domain	ec:2.7.1	
Phvul.007G054800.2	amino-terminal domain cyclin	cyclin-dependent protein kinase holoenzyme complex;nucleus;cyclin-dependent protein serine/threonine kinase regulator activity;positive regulation of cyclin-dependent protein serine/threonine kinase activity;positive regulation of transcription from RNA polymerase II promoter;positive regulation of phosphorylation of RNA polymerase II C-terminal domain	ec:2.7.1	
Phvul.007G056100.1	thermospermine synthase ACAULIS5-like	cytoplasm;spermidine synthase activity;thermospermine synthase activity;polyamine biosynthetic process	ec:2.5.1.79;ec:2.5.1.16-synthase	Arginine and proline metabolism;Glutathione metabolism;beta-Alanine

				metabolism;Cysteine and methionine metabolism
Phvul.007G056400.1	primary amine oxidase-like	copper ion binding;primary amine oxidase activity;quinone binding;amine metabolic process;oxidation-reduction process	ec:1.4.3.21-oxidase	beta-Alanine metabolism;Tropane, piperidine and pyridine alkaloid biosynthesis;Isoquinoline alkaloid biosynthesis;Phenylalanine metabolism;Glycine, serine and threonine metabolism;Tyrosine metabolism
Phvul.007G056400.2	primary amine oxidase-like	copper ion binding;primary amine oxidase activity;quinone binding;amine metabolic process;oxidation-reduction process	ec:1.4.3.21-oxidase	beta-Alanine metabolism;Tropane, piperidine and pyridine alkaloid biosynthesis;Isoquinoline alkaloid biosynthesis;Phenylalanine metabolism;Glycine, serine and threonine metabolism;Tyrosine metabolism
Phvul.007G056800.1	hypothetical protein PHAVU_007G056800g			
Phvul.007G060200.1	type IV inositol polyphosphate 5-phosphatase 7-like	phosphatidylinositol-4,5-bisphosphate 5-phosphatase activity;phosphatidylinositol dephosphorylation	ec:3.1;ec:3.1.3.41-nitrophenyl phosphatase;ec:3.1.3.36-5-phosphatase	Aminobenzoate degradationInositol phosphate metabolism;Phosphatidylinositol signaling system
Phvul.007G062700.1	LRR receptor-like kinase family	intracellular;MAP kinase kinase kinase activity;ATP binding;MAPK cascade;activation of MAPKK activity	ec:2.7.11;ec:2.7.11.25	
Phvul.007G062700.2	LRR receptor-like kinase family	intracellular;MAP kinase kinase kinase activity;ATP binding;MAPK cascade;activation of MAPKK activity	ec:2.7.11;ec:2.7.11.25	
Phvul.007G066100.1	Molybdenum cofactor sulfurase	catalytic activity;metabolic process		
Phvul.007G067500.1	homeobox-leucine zipper ANTHOCYANINLESS 2 isoform X2	nucleus;lipid binding;sequence-specific DNA binding;regulation of transcription, DNA-templated		
Phvul.007G067500.2	homeobox-leucine zipper ANTHOCYANINLESS 2 isoform X2	nucleus;lipid binding;sequence-specific DNA binding;regulation of transcription, DNA-templated		
Phvul.007G068100.1	3-hexulose-6-phosphate isomerase,	glutamine-fructose-6-phosphate transaminase (isomerizing) activity;isomerase activity;carbohydrate binding;fructose 6-phosphate metabolic process;UDP-N-acetylglucosamine metabolic process;protein N-linked glycosylation	ec:2.6.1.16-transaminase (isomerizing)	Biosynthesis of antibiotics;Amino sugar and nucleotide sugar metabolism;Alanine, aspartate and glutamate metabolism
Phvul.007G068200.1	phosphatase inhibitor 2	protein phosphatase type 1 complex;protein phosphatase inhibitor activity;regulation of signal transduction;negative regulation of catalytic activity;regulation of phosphoprotein phosphatase activity		
Phvul.007G068200.2	phosphatase inhibitor 2	protein phosphatase type 1 complex;protein phosphatase inhibitor activity;regulation of signal transduction;negative regulation of catalytic activity;regulation of phosphoprotein phosphatase activity		
Phvul.007G069000.1	F-box LRR-repeat At3g48880-like			
Phvul.007G069500.1	remorin-like isoform X1			

Phvul.007G069900.1	pentatricopeptide repeat-containing At3g60050-like			
Phvul.007G069900.2	pentatricopeptide repeat-containing At3g60050-like			
Phvul.007G069900.3	pentatricopeptide repeat-containing At3g60050-like			
Phvul.007G069900.4	pentatricopeptide repeat-containing At3g60050-like			
Phvul.007G069900.5	pentatricopeptide repeat-containing At3g60050-like			
Phvul.007G070600.1	cofilin actin-depolymerizing factor	intracellular;nucleic acid binding;zinc ion binding		
Phvul.007G070700.1	actin depolymerizing factor,	nucleic acid binding;zinc ion binding		
Phvul.007G071000.1	oxalate-- ligase	plasmodesma;chloroplast stroma;apoplast;oxalate-CoA ligase activity;positive regulation of seed germination;response to nitrate;seed coat development;nitrate transport;oxalate catabolic process;para-aminobenzoic acid metabolic process;defense response to fungus	ec:6.2.1.8-ligase	Glyoxylate and dicarboxylate metabolism
Phvul.007G072400.1	C2H2-like zinc finger ,	metal ion binding		
Phvul.007G072800.1	mitogen-activated kinase kinase kinase YODA-like	cytoplasm;protein serine/threonine kinase activity;ATP binding;regulation of mitotic cell cycle;signal transduction by protein phosphorylation;stress-activated protein kinase signaling cascade;activation of protein kinase activity;regulation of apoptotic process	ec:2.7.11	
Phvul.007G073500.1	scarecrow 8	transcription, DNA-templated;regulation of transcription, DNA-templated		
Phvul.007G076700.1	S-type anion channel SLAH2-like isoform X1	cell;integral component of membrane;voltage-gated anion channel activity;cellular ion homeostasis;transmembrane transport;regulation of anion transmembrane transport		
Phvul.007G077900.1	ABC transporter C family member 10-like	integral component of membrane;ATP binding;ATPase activity, coupled to transmembrane movement of substances;metabolic process;transmembrane transport	ec:3.6.1;ec:3.6.1.3-adenylpyrophosphatase;ec:3.6.1.15-phosphatase	Purine metabolismPurine metabolism;Thiamine metabolism
Phvul.007G078300.1	chromosome condensation regulator RCC1 repeat	ligase activity;metal ion binding;metabolic process		
Phvul.007G079200.1	eukaryotic translation initiation factor 5B	intracellular;translation initiation factor activity;GTPase activity;GTP binding;translational initiation	ec:3.6.1;ec:3.6.1.15-phosphatase	Purine metabolism;Thiamine metabolism
Phvul.007G079200.2	eukaryotic translation initiation factor 5B	intracellular;translation initiation factor activity;GTPase activity;GTP binding;translational initiation	ec:3.6.1;ec:3.6.1.15-phosphatase	Purine metabolism;Thiamine metabolism
Phvul.007G080200.1	UPF0496 4-like	integral component of membrane		
Phvul.007G080800.1	sterol 3-beta-glucosyltransferase UGT80B1-like isoform X1	vacuolar membrane;sterol 3-beta-glucosyltransferase activity;flavonoid biosynthetic process;seed germination;seed coat development;sterol metabolic process;lipid glycosylation;flavonoid glucuronidation	ec:2.4.1;ec:2.4.1.173	
Phvul.007G081000.1	G-box-binding factor 4-like	nucleus;transcription factor activity, sequence-specific DNA binding;sequence-specific DNA binding;regulation of transcription, DNA-templated		
Phvul.007G081900.1	polypyrimidine tract-binding homolog 3-like	nucleus;nucleotide binding;RNA binding;mRNA processing		
Phvul.007G083500.1	GIGANTEA	nucleoplasm;starch metabolic process;regulation of transcription, DNA-		

		templated;circadian rhythm;response to cold;response to blue light;response to far red light;temperature compensation of the circadian clock;response to hydrogen peroxide;long-day photoperiodism, flowering;positive regulation of long-day photoperiodism, flowering		
Phvul.007G083500.2	GIGANTEA	nucleoplasm;starch metabolic process;regulation of transcription, DNA-templated;circadian rhythm;response to cold;response to blue light;response to far red light;temperature compensation of the circadian clock;response to hydrogen peroxide;long-day photoperiodism, flowering;positive regulation of long-day photoperiodism, flowering		
Phvul.007G083800.1	gmCK1p isoform X1	choline kinase activity;CDP-choline pathway;phosphorylation	ec:2.7.1.32-kinase	Glycerophospholipid metabolism
Phvul.007G083800.2	gmCK1p isoform X1	choline kinase activity;CDP-choline pathway;phosphorylation	ec:2.7.1.32-kinase	Glycerophospholipid metabolism
Phvul.007G084400.1	probable LRR receptor-like serine threonine- kinase At1g34110	integral component of membrane;protein serine/threonine kinase activity;ATP binding;protein phosphorylation	ec:2.7.11	
Phvul.007G085100.1	transmembrane ,	vacuole;integral component of membrane		
Phvul.007G085300.1	tRNA pseudouridine(38 39) synthase isoform X2	RNA binding;pseudouridine synthase activity;pseudouridine synthesis;tRNA processing	ec:5.4.99.12	
Phvul.007G090200.1	hypothetical protein PHAVU_007G090200g			
Phvul.007G090400.1	hypothetical protein PHAVU_007G090400g	chloroplast thylakoid membrane;integral component of membrane;phosphatidylglycerol biosynthetic process;aromatic amino acid family biosynthetic process;thylakoid membrane organization;photosystem II assembly;iron-sulfur cluster assembly;isopentenyl diphosphate biosynthetic process, methylerythritol 4-phosphate pathway;cysteine biosynthetic process;photosystem I assembly		
Phvul.007G091100.1	alpha beta fold hydrolase	integral component of membrane;hydrolase activity;metabolic process		
Phvul.007G092600.1	beta-taxilin isoform X1	syntaxin binding		
Phvul.007G093000.1	pseudouridine synthase Rlu family	RNA binding;pseudouridine synthase activity;deaminase activity;enzyme-directed rRNA pseudouridine synthesis	ec:5.4.99.12	
Phvul.007G093800.1	Transmembrane 115	Golgi apparatus;integral component of membrane;serine-type endopeptidase activity;proteolysis;calcium ion transport;retrograde vesicle-mediated transport, Golgi to ER;Golgi organization;response to salt stress	ec:3.4.21	
Phvul.007G094100.1	Para-aminobenzoate synthase	chloroplast;oxo-acid-lyase activity;4-amino-4-deoxychorismate synthase activity;para-aminobenzoic acid biosynthetic process;chorismate metabolic process;folic acid biosynthetic process	ec:2.6.1.85-synthase	Folate biosynthesis
Phvul.007G095400.1	ENHANCED DISEASE RESISTANCE 2-like	lipid binding		
Phvul.007G096000.1	synaptotagmin-3 isoform X1	endoplasmic reticulum;integral component of membrane;lipid binding;sterol biosynthetic process;pentacyclic triterpenoid biosynthetic process		
Phvul.007G096500.1	probable lysine-specific demethylase ELF6	nucleic acid binding;methyltransferase activity;oxidoreductase activity;metal ion binding;cellular protein modification process;cellular component organization;methylation;single-organism cellular process;negative regulation of post-embryonic development;negative regulation of		

		response to stimulus;response to stimulus;oxidation-reduction process;regulation of photoperiodism, flowering;negative regulation of reproductive process		
Phvul.007G097100.1	pumilio homolog 4 isoform X1	mRNA binding		
Phvul.007G097200.1	vacuolar amino acid transporter 1	integral component of membrane;amino acid transmembrane transporter activity;amino acid transmembrane transport		
Phvul.007G098800.1	exocyst complex component SEC8	exocyst;cytosol;plasma membrane;plasmodesma;glucose catabolic process;vesicle docking involved in exocytosis;pollen germination;pollen tube growth;protein transport;mucilage biosynthetic process involved in seed coat development		
Phvul.007G100700.1	ABA induced plasma membrane	integral component of membrane		
Phvul.007G101800.1	clavata3 esr-related 12 family	integral component of membrane		
Phvul.007G102000.1	transmembrane fragile-X-F-associated	integral component of membrane;ubiquitin-protein transferase activity;zinc ion binding;ligase activity;regulation of signal transduction;protein ubiquitination		
Phvul.007G102000.2	transmembrane fragile-X-F-associated	integral component of membrane;ubiquitin-protein transferase activity;zinc ion binding;ligase activity;regulation of signal transduction;protein ubiquitination		
Phvul.007G104200.1	RAB6-interacting golgin-like			
Phvul.007G105800.1	phosphatidylinositol-3-phosphatase myotubularin-1-like isoform X1	protein tyrosine phosphatase activity;peptidyl-tyrosine dephosphorylation	ec:3.1.3.48;ec:3.1;ec:3.1.3.41-nitrophenyl phosphatase;ec:3.1.3.16-phosphatase	Aminobenzoate degradationT cell receptor signaling pathway
Phvul.007G106300.1	cytochrome P450 family	integral component of membrane;iron ion binding;oxidoreductase activity, acting on paired donors, with incorporation or reduction of molecular oxygen, NAD(P)H as one donor, and incorporation of one atom of oxygen;heme binding;secondary metabolite biosynthetic process;oxidation-reduction process	ec:1.14.13	
Phvul.007G107100.1	hypothetical protein PHAVU_007G107100g			
Phvul.007G108200.1	LIGHT-DEPENDENT SHORT HYPOCOTYLS 4-like			
Phvul.007G108200.2	LIGHT-DEPENDENT SHORT HYPOCOTYLS 4-like			
Phvul.007G108700.1	hypothetical protein PHAVU_007G108700g, partial	nucleotide binding;nucleic acid binding		
Phvul.007G110400.1	U-box domain-containing 43-like	ubiquitin-protein transferase activity;ligase activity;protein ubiquitination		
Phvul.007G110600.1	EXORDIUM-like 5			
Phvul.007G110800.1	DUF506 family			
Phvul.007G111500.1	65-kDa microtubule-associated 3-like	microtubule binding;microtubule cytoskeleton organization;cytokinesis		
Phvul.007G113000.1	chlorophyll a-b binding CP26, chloroplastic	PSII associated light-harvesting complex II;photosystem I;photosystem II antenna complex;chloroplast envelope;plastoglobule;integral component of membrane;chlorophyll binding;pigment binding;metal ion binding;rRNA processing;response to high light intensity;response to sucrose;photosynthesis, light harvesting in photosystem I;regulation of proton transport;nonphotochemical		

		quenching;protein-chromophore linkage;cysteine biosynthetic process		
Phvul.007G113600.1	ATP-dependent Clp protease ATP-binding subunit clpX-like, mitochondrial	ATP binding;peptidase activity;unfolded protein binding;protein folding;proteolysis		
Phvul.007G114400.1	ribosomal lysine N-methyltransferase 3 isoform X1	protein-lysine N-methyltransferase activity;peptidyl-lysine monomethylation	ec:2.1.1	
Phvul.007G114500.1	zinc finger CCCH domain-containing 13-like isoform X1			
Phvul.007G114500.2	zinc finger CCCH domain-containing 13-like isoform X2			
Phvul.007G116400.1	ATP-dependent zinc metalloprotease	mitochondrion;plasmodesma;chloroplast envelope;integral component of membrane;ATP-dependent peptidase activity;metalloendopeptidase activity;ATP binding;microtubule-severing ATPase activity;proteolysis	ec:3.6.1;ec:3.6.1.3-adenylpyrophosphatase;ec:3.6.4.3;ec:3.6.1.15-phosphatase;ec:3.4.24	Purine metabolismPurine metabolism;Thiamine metabolism
Phvul.007G116600.1	CHROMATIN REMODELING 20 isoform X1	ATP binding;hydrolase activity;metabolic process		
Phvul.007G116600.2	CHROMATIN REMODELING 20 isoform X1	ATP binding;hydrolase activity;metabolic process		
Phvul.007G118300.1	probable tyrosine-phosphatase At1g05000	protein tyrosine phosphatase activity;peptidyl-tyrosine dephosphorylation	ec:3.1.3.48;ec:3.1;ec:3.1.3.41-nitrophenyl phosphatase;ec:3.1.3.16-phosphatase	Aminobenzoate degradationT cell receptor signaling pathway
Phvul.007G118700.1	lysine ketoglutarate reductase trans-splicing	integral component of membrane		
Phvul.007G118700.2	lysine ketoglutarate reductase trans-splicing	integral component of membrane		
Phvul.007G119900.1	plastid transcriptionally active			
Phvul.007G121700.1	probable serine threonine phosphatase 2A regulatory subunit B delta	calcium ion binding		
Phvul.007G121700.2	probable serine threonine phosphatase 2A regulatory subunit B delta	calcium ion binding		
Phvul.007G121700.3	probable serine threonine phosphatase 2A regulatory subunit B delta	calcium ion binding		
Phvul.007G123200.1	MATH and LRR domain-containing PFE0570w-like isoform X2	nuclear pore inner ring;structural constituent of nuclear pore;protein import into nucleus, docking;nuclear pore organization;root hair cell differentiation		
Phvul.007G123200.2	MATH and LRR domain-containing PFE0570w-like isoform X2	nuclear pore inner ring;structural constituent of nuclear pore;protein import into nucleus, docking;nuclear pore organization;root hair cell differentiation		
Phvul.007G124500.1	phosphate transporter PHO1	trans-Golgi network;plasma membrane;integral component of membrane;inositol monophosphate 1-phosphatase activity;inositol monophosphate 3-phosphatase activity;inositol monophosphate 4-phosphatase activity;dephosphorylation	ec:3.1.3;ec:3.1;ec:3.1.3.41-nitrophenyl phosphatase;ec:3.1.3.25-phosphatase	Aminobenzoate degradationBiosynthesis of antibiotics;Streptomycin biosynthesis;Inositol phosphate metabolism;Phosphatidylinositol signaling system
Phvul.007G124500.2	phosphate transporter PHO1	trans-Golgi network;plasma membrane;integral component of membrane;inositol	ec:3.1.3;ec:3.1;ec:3.1.3.41-nitrophenyl	Aminobenzoate degradationBiosynthesi

		monophosphate 1-phosphatase activity;inositol monophosphate 3-phosphatase activity;inositol monophosphate 4-phosphatase activity;dephosphorylation	phosphatase;ec:3.1.3.25-phosphatase	s of antibiotics;Streptomycin biosynthesis;Inositol phosphate metabolism;Phosphatidylinositol signaling system
Phvul.007G124800.1	peroxisomal membrane PMP22	integral component of membrane		
Phvul.007G125800.1	WVD2-like 1 isoform X1	integral component of membrane		
Phvul.007G126200.1	chloroplastic group IIA intron splicing facilitator CRS1, chloroplastic	RNA binding		
Phvul.007G126800.1	thioredoxin HCF164, chloroplastic	cytoplasm;protein disulfide oxidoreductase activity;oxidoreductase activity, acting on a sulfur group of donors, disulfide as acceptor;sulfate assimilation;protein folding;glycerol ether metabolic process;cellular response to oxidative stress;cell redox homeostasis;oxidation-reduction process		
Phvul.007G128900.1	probable serine threonine- kinase nek3			
Phvul.007G128900.2	probable serine threonine- kinase nek3			
Phvul.007G129000.1	Actin-related 9	Ino80 complex;chromatin remodeling;vegetative to reproductive phase transition of meristem		
Phvul.007G129300.1	replication factor-A carboxy-terminal domain			
Phvul.007G134400.1	hypothetical protein PHAVU_007G134400g			
Phvul.007G134600.1	hypothetical protein PHAVU_007G134600g	integral component of membrane		
Phvul.007G134600.2	hypothetical protein PHAVU_007G134600g	integral component of membrane		
Phvul.007G134600.3	hypothetical protein PHAVU_007G134600g	integral component of membrane		
Phvul.007G134600.4	hypothetical protein PHAVU_007G134600g	integral component of membrane		
Phvul.007G135800.1	UDP rhamnose:anthocyanidin-3-glucoside rhamnosyltransferase, partial	integral component of membrane;intracellular membrane-bounded organelle;quercetin 3-O-glucosyltransferase activity;quercetin 7-O-glucosyltransferase activity;flavonoid biosynthetic process;flavonoid glucuronidation	ec:2.4.1	
Phvul.007G136200.1	2 family	thiol-dependent ubiquitinyl hydrolase activity;ubiquitin-dependent protein catabolic process;protein deubiquitination	ec:3.4;ec:3.4.19.12	
Phvul.007G136200.2	2 family	thiol-dependent ubiquitinyl hydrolase activity;ubiquitin-dependent protein catabolic process;protein deubiquitination	ec:3.4;ec:3.4.19.12	
Phvul.007G136700.1	hypothetical protein PHAVU_007G136700g			
Phvul.007G136700.2	hypothetical protein PHAVU_007G136700g			

Phvul.007G137100.1	7-methylguanosine phosphate-specific 5 -nucleotidase A	cytosol;integral component of membrane;magnesium ion binding;5'-nucleotidase activity;dephosphorylation	ec:3.1.3.5-uridine 5'-nucleotidase;ec:3.1;ec:3.1.3.41-nitrophenyl phosphatase;ec:3.1.3.31	Purine metabolism;Nicotinate and nicotinamide metabolism;Pyrimidine metabolismAminobenzoate degradation
Phvul.007G137100.2	7-methylguanosine phosphate-specific 5 -nucleotidase A	cytosol;integral component of membrane;magnesium ion binding;5'-nucleotidase activity;dephosphorylation	ec:3.1.3.5-uridine 5'-nucleotidase;ec:3.1;ec:3.1.3.41-nitrophenyl phosphatase;ec:3.1.3.31	Purine metabolism;Nicotinate and nicotinamide metabolism;Pyrimidine metabolismAminobenzoate degradation
Phvul.007G137800.1	receptor kinase	integral component of membrane;protein kinase activity;ATP binding;protein phosphorylation		
Phvul.007G138700.1	exocyst complex component EXO70A1	exocyst;exocytosis		
Phvul.007G139200.1	DUF1635 family			
Phvul.007G141200.1	UDP-glycosyltransferase family	endosome;trans-Golgi network;integral component of membrane;transferase activity;metabolic process		
Phvul.007G141200.2	UDP-glycosyltransferase family	endosome;trans-Golgi network;integral component of membrane;transferase activity;metabolic process		
Phvul.007G141300.1	DNA-binding PD2			
Phvul.007G142900.1	alanine-tRNA ligase	mitochondrion;cytosol;chloroplast stroma;membrane;tRNA binding;DNA binding;transcription factor activity, sequence-specific DNA binding;alanine-tRNA ligase activity;ATP binding;zinc ion binding;amino acid binding;sigma factor activity;DNA-templated transcription, initiation;regulation of transcription, DNA-templated;tRNA modification;alanyl-tRNA aminoacylation;embryo development ending in seed dormancy	ec:6.1.1;ec:6.1.1.7-ligase	Aminoacyl-tRNA biosynthesis
Phvul.007G144000.1	probable U6 snRNA-associated Sm LSm4	cytoplasmic mRNA processing body;spliceosomal complex;U6 snRNP;viral nucleocapsid;spliceosomal tri-snRNP complex;U6 snRNA binding;spliceosomal complex assembly;spliceosomal snRNP assembly;nuclear-transcribed mRNA catabolic process;cytoplasmic mRNA processing body assembly		
Phvul.007G146000.1	phytochrome A-associated F-box	ubiquitin-protein transferase activity;ubiquitin-dependent protein catabolic process;regulation of photomorphogenesis;protein ubiquitination;regulation of protein localization;regulation of circadian rhythm;leaf development;photoperiodism, flowering		
Phvul.007G147000.1	magnesium proton exchanger	integral component of membrane;iron ion transport;zinc II ion transport;magnesium ion transport;transmembrane transport		
Phvul.007G147100.1	blue-light photoreceptor PHR2	lyase activity;maltose metabolic process;phosphatidylglycerol biosynthetic process;carotenoid biosynthetic process;starch biosynthetic process;isopentenyl diphosphate biosynthetic process, methylerythritol 4-phosphate pathway		
Phvul.007G147200.1	gamma-tubulin complex component 4 homolog	equatorial microtubule organizing center;gamma-tubulin complex;centrosome;spindle pole body;microtubule;plasmodesma;tubulin complex;structural constituent of cytoskeleton;gamma-tubulin binding;microtubule minus-end binding;meiotic nuclear division;regulation of flower development;histone modification;growth;cortical microtubule organization;floral organ formation;centrosome duplication;interphase microtubule nucleation by interphase microtubule organizing center;positive		

		regulation of microtubule nucleation;mitotic spindle assembly		
Phvul.007G148200.1	transcription termination factor family	mitochondrion;double-stranded DNA binding;regulation of transcription, DNA-templated		
Phvul.007G148600.1	transmembrane ,	membrane		
Phvul.007G149000.1	auxin response factor 18-like isoform X1	nucleus;DNA binding;transcription, DNA-templated;regulation of transcription, DNA-templated;auxin-activated signaling pathway		
Phvul.007G151200.1	probable pectate lyase 12	integral component of membrane;pectate lyase activity;metal ion binding;pectin catabolic process	ec:4.2.2.2-lyase	Pentose and glucuronate interconversions
Phvul.007G152700.1	hypothetical protein PHAVU_007G152700g			
Phvul.007G152800.1	UDP-glycosyltransferase 73C3-like	intracellular membrane-bounded organelle;quercetin 3-O-glucosyltransferase activity;quercetin 7-O-glucosyltransferase activity;flavonoid biosynthetic process;flavonoid glucuronidation	ec:2.4.1	
Phvul.007G154900.1	RNA polymerase I termination factor	DNA binding		
Phvul.007G154900.2	RNA polymerase I termination factor	DNA binding		
Phvul.007G154900.3	RNA polymerase I termination factor	DNA binding		
Phvul.007G154900.4	RNA polymerase I termination factor	DNA binding		
Phvul.007G155200.1	probable indole-3-pyruvate monooxygenase YUCCA5	integral component of membrane;N,N-dimethylaniline monooxygenase activity;flavin adenine dinucleotide binding;NADP binding;oxidation-reduction process	ec:1.14.13.8-monooxygenase;ec:1.14.13	Drug metabolism - cytochrome P450
Phvul.007G156300.1	zinc finger HIT domain-containing 3			
Phvul.007G156300.2	zinc finger HIT domain-containing 3			
Phvul.007G157900.1	PHD zinc finger	DNA binding;helicase activity;N-acetyltransferase activity;zinc ion binding;metabolic process	ec:3.6.1;ec:3.6.1.15-phosphatase;ec:2.3.1	Purine metabolism;Thiamine metabolism
Phvul.007G157900.2	PHD zinc finger	DNA binding;helicase activity;N-acetyltransferase activity;zinc ion binding;metabolic process	ec:3.6.1;ec:3.6.1.15-phosphatase;ec:2.3.1	Purine metabolism;Thiamine metabolism
Phvul.007G159500.1	DUF4378 domain			
Phvul.007G159700.1	UDP-glucuronic acid decarboxylase 1	integral component of membrane		
Phvul.007G161700.1	probable phosphatase 2C 38	protein serine/threonine phosphatase activity;metal ion binding;protein dephosphorylation	ec:3.1;ec:3.1.3.41-nitrophenyl phosphatase;ec:3.1.3.16-phosphatase	Aminobenzoate degradationT cell receptor signaling pathway
Phvul.007G163300.1	E3 ubiquitin- ligase UPL4-like	ubiquitin-protein transferase activity;ligase activity;protein ubiquitination involved in ubiquitin-dependent protein catabolic process		
Phvul.007G164300.1	IQ-DOMAIN 1			
Phvul.007G165100.1	nuclear transcription factor Y subunit B-5	nucleus;sequence-specific DNA binding;protein heterodimerization activity;regulation of transcription, DNA-templated		
Phvul.007G165500.1	hypothetical protein PHAVU_007G165500g			
Phvul.007G166900.1	CUGBP Elav-like family member 5	nucleotide binding;RNA binding		
Phvul.007G167200.1	peroxidase 3-like	extracellular region;plant-type cell wall;peroxidase activity;heme binding;metal ion binding;response to oxidative stress;plant-type cell wall organization;hydrogen peroxide catabolic process;oxidation-reduction process;cellular oxidant detoxification	ec:1.11.1.7-lactoperoxidase	Phenylpropanoid biosynthesis

Phvul.007G168400.1	methyl- -binding domain	nucleus;thymidine kinase activity;ATP binding;zinc ion binding;methyl-CpG binding;negative regulation of transcription from RNA polymerase II promoter;methylation-dependent chromatin silencing;deoxyribonucleoside monophosphate biosynthetic process	ec:2.7.1.21-kinase;ec:2.7.1.145	Drug metabolism - other enzymes;Pyrimidine metabolism
Phvul.007G169300.1	BTB POZ and MATH domain-containing 2-like			
Phvul.007G171800.1	Rho GTPase	integral component of mitochondrial outer membrane;GTPase activity;calcium ion binding;GTP binding;mitochondrion organization;small GTPase mediated signal transduction;metabolic process;protein transport	ec:3.6.1;ec:3.6.1.15-phosphatase	Purine metabolism;Thiamine metabolism
Phvul.007G172900.1	PHD finger ,	DNA binding;N-acetyltransferase activity;zinc ion binding;metabolic process	ec:2.3.1	
Phvul.007G173800.1	hypothetical protein PHAVU_007G173800g			
Phvul.007G174000.1	NETWORKED 1D-like	kinase activity;phosphorylation		
Phvul.007G174600.1	ETO1 1	RNA splicing, via endonucleolytic cleavage and ligation;cell-cell signaling;serine family amino acid metabolic process;methionine biosynthetic process;virus induced gene silencing;determination of bilateral symmetry;meristem initiation;vegetative phase change;meristem maintenance;production of ta-siRNAs involved in RNA interference;regulation of ethylene biosynthetic process;production of miRNAs involved in gene silencing by miRNA;cell wall modification		
Phvul.007G175700.1	guanine nucleotide-binding subunit gamma 2	heterotrimeric G-protein complex;signal transducer activity;GTP binding;G-protein coupled receptor signaling pathway;defense response to fungus, incompatible interaction;seed germination;basipetal auxin transport;acropetal auxin transport;protein prenylation;protein palmitoylation;lateral root development		
Phvul.007G175800.1	LONGIFOLIA 1			
Phvul.007G177300.1	Photosystem II reaction center W , chloroplastic	chloroplast;photosystem II;integral component of membrane;photosynthesis		
Phvul.007G179200.1	DEAD-box ATP-dependent RNA helicase 16	intracellular;nucleic acid binding;ATP-dependent RNA helicase activity;ATP binding;protein import into nucleus;RNA secondary structure unwinding	ec:3.6.1;ec:3.6.1.3-adenylpyrophosphatase;ec:3.6.1.15-phosphatase;ec:2.7.7	Purine metabolismPurine metabolism;Thiamine metabolism
Phvul.007G179200.2	DEAD-box ATP-dependent RNA helicase 16	intracellular;nucleic acid binding;ATP-dependent RNA helicase activity;ATP binding;protein import into nucleus;RNA secondary structure unwinding	ec:3.6.1;ec:3.6.1.3-adenylpyrophosphatase;ec:3.6.1.15-phosphatase;ec:2.7.7	Purine metabolismPurine metabolism;Thiamine metabolism
Phvul.007G179300.1	cyclin-H1-1	cyclin-dependent protein kinase holoenzyme complex;TFIIK complex;protein kinase activity;cyclin-dependent protein serine/threonine kinase regulator activity;mitotic cell cycle;RNA splicing, via endonucleolytic cleavage and ligation;transcription from RNA polymerase II promoter;protein phosphorylation;pyrimidine ribonucleotide biosynthetic process;response to water deprivation;regulation of stomatal movement;stomatal lineage progression;DNA endoreduplication;negative regulation of cyclin-dependent protein serine/threonine kinase activity;positive regulation of cyclin-dependent protein serine/threonine kinase activity;positive regulation of transcription from RNA polymerase II promoter;reactive oxygen species metabolic process;positive regulation of phosphorylation of RNA	ec:2.7.1	

		polymerase II C-terminal domain;stomatal opening;regulation of response to water deprivation		
Phvul.007G180200.1	probable rRNA-processing EBP2 homolog	2-alkenal reductase [NAD(P)] activity;oxidation-reduction process	ec:1.3.1.74	
Phvul.007G180200.2	probable rRNA-processing EBP2 homolog	2-alkenal reductase [NAD(P)] activity;oxidation-reduction process	ec:1.3.1.74	
Phvul.007G180300.1	mitochondrial Rho GTPase 1	integral component of mitochondrial outer membrane;GTPase activity;calcium ion binding;GTP binding;mitochondrion organization;small GTPase mediated signal transduction;metabolic process;protein transport	ec:3.6.1;ec:3.6.1.15-phosphatase	Purine metabolism;Thiamine metabolism
Phvul.007G181500.1	heavy-metal-associated domain	cytoplasm;transition metal ion binding;metal ion transport;cellular transition metal ion homeostasis		
Phvul.007G181800.1	E3 ubiquitin- ligase ATL6-like	integral component of membrane;zinc ion binding		
Phvul.007G183100.1	breast carcinoma amplified sequence 3	cytoplasm;nucleotide binding;vacuole organization;cellular response to phosphate starvation;galactolipid biosynthetic process;cellular response to water deprivation		
Phvul.007G184100.1	Na+ H+ antiporter	vacuolar membrane;plasma membrane;integral component of membrane;sodium:proton antiporter activity;regulation of pH;response to salt stress;sodium ion transmembrane transport;potassium ion homeostasis;hydrogen ion transmembrane transport		
Phvul.007G184300.1	hypothetical protein PHAVU_007G184300g			
Phvul.007G185500.1	mediator-associated 1-like			
Phvul.007G185700.1	tRNA(adenine(34)) deaminase, chloroplastic	cytosol;guanosine deaminase activity;RNA modification	ec:3.5.4.15	
Phvul.007G186000.1	zeta-carotene desaturase, chloroplastic chromoplastic	chromoplast;chloroplast envelope;carotene 7,8-desaturase activity;9,9'-dicis-carotene:quinone oxidoreductase activity;7,9,9'-tricis-neurosporene:quinone oxidoreductase activity;carotenoid biosynthetic process;9,9'-di-cis-zeta-carotene desaturation to 7,9,7',9'-tetra-cis-lycopene;oxidation-reduction process	ec:1.14.99.30;ec:1.3.5.6-desaturase	Carotenoid biosynthesis
Phvul.007G186500.1	pollen-specific SF21-like			
Phvul.007G186800.1	serine threonine-kinase Nek2-like	protein serine/threonine kinase activity;ATP binding;protein phosphorylation	ec:2.7.11	
Phvul.007G191900.1	probable methyltransferase PMT3	integral component of membrane;S-adenosylmethionine-dependent methyltransferase activity;methylation		
Phvul.007G192000.1	serine threonine-kinase 19 isoform X1	kinase activity;phosphorylation		
Phvul.007G192000.2	serine threonine-kinase 19 isoform X1	kinase activity;phosphorylation		
Phvul.007G192300.1	armadillo beta-catenin repeat	ubiquitin-protein transferase activity;ligase activity;protein ubiquitination		
Phvul.007G192300.2	armadillo beta-catenin repeat	ubiquitin-protein transferase activity;ligase activity;protein ubiquitination		
Phvul.007G193000.1	root UVB sensitive 2, chloroplastic	mitochondrion;chloroplast envelope;auxin polar transport;response to UV-B		
Phvul.007G193800.1	ethylene-responsive transcription factor 1B-like	nucleus;DNA binding;transcription factor activity, sequence-specific DNA binding;transcription, DNA-templated;regulation of transcription, DNA-templated		
Phvul.007G194400.1	CDT1 a, chloroplastic			
Phvul.007G197300.1	DNA-binding ,	DNA binding;zinc ion binding		

Phvul.007G198300.1	DUF1399 family	plasma membrane		
Phvul.007G198300.2	DUF1399 family	plasma membrane		
Phvul.007G198300.3	DUF1399 family	plasma membrane		
Phvul.007G198300.4	DUF1399 family	plasma membrane		
Phvul.007G198700.1	probable phosphatase 2C 34	protein serine/threonine phosphatase activity;protein dephosphorylation	ec:3.1;ec:3.1.3.41-nitrophenyl phosphatase;ec:3.1.3.16-phosphatase	Aminobenzoate degradationT cell receptor signaling pathway
Phvul.007G199000.1	kinesin-4-like	kinesin complex;microtubule;microtubule motor activity;ATP binding;microtubule binding;ATPase activity;microtubule-based movement;metabolic process	ec:3.6.1;ec:3.6.1.3-adenylpyrophosphatase;ec:3.6.1.15-phosphatase	Purine metabolismPurine metabolism;Thiamine metabolism
Phvul.007G199700.1	syntaxin-43-like isoform X1	endomembrane system;integral component of membrane;SNARE complex;SNARE binding;SNAP receptor activity;intracellular protein transport;vesicle fusion;vesicle docking		
Phvul.007G203100.1	alkaline neutral invertase A, mitochondrial-like	nucleus;mitochondrion;sucrose alpha-glucosidase activity;exodeoxyribonuclease III activity;glycopeptide alpha-N-acetylgalactosaminidase activity;sucrose catabolic process;DNA repair;root development	ec:3.2.1.48-alpha-glucosidase;ec:3.2.1;ec:3.2.1.26-invertase;ec:3.1.15;ec:3.1.11;ec:3.1;ec:3.1.11.2;ec:3.2.1.97	Starch and sucrose metabolismStarch and sucrose metabolism;Galactose metabolism
Phvul.007G204600.1	pentatricopeptide repeat-containing At3g16710, mitochondrial isoform X2			
Phvul.007G206100.1	titin-like isoform X3			
Phvul.007G206100.2	titin-like isoform X3			
Phvul.007G206400.1	NRT1 PTR FAMILY -like	integral component of membrane;transporter activity;transport		
Phvul.007G206500.1	flap endonuclease 1 isoform X1	nucleoplasm;nucleolus;mitochondrion;cytosol;DNA binding;endonuclease activity;exonuclease activity;metal ion binding;DNA replication;DNA repair;vegetative to reproductive phase transition of meristem;nucleic acid phosphodiester bond hydrolysis	ec:3.1	
Phvul.007G206500.2	flap endonuclease 1 isoform X2	nucleoplasm;nucleolus;mitochondrion;cytosol;DNA binding;endonuclease activity;exonuclease activity;metal ion binding;DNA replication;DNA repair;vegetative to reproductive phase transition of meristem;nucleic acid phosphodiester bond hydrolysis	ec:3.1	
Phvul.007G208500.1	Transcription factor PIF3	protein dimerization activity		
Phvul.007G209300.1	translocase subunit , chloroplastic	chloroplast thylakoid membrane;chloroplast stroma;ATP binding;protein targeting;protein import		
Phvul.007G209600.1	ATP binding cassette subfamily B4 isoform 2	integral component of membrane;ATP binding;ATPase activity, coupled to transmembrane movement of substances;metabolic process;transmembrane transport	ec:3.6.1;ec:3.6.1.3-adenylpyrophosphatase;ec:3.6.1.15-phosphatase	Purine metabolismPurine metabolism;Thiamine metabolism
Phvul.007G209900.1	hypothetical protein PHAVU_007G209900g			
Phvul.007G210600.1	squamosa promoter-binding 1	nucleus;integral component of membrane;DNA binding		
Phvul.007G211400.1	polygalacturonase At1g48100	extracellular region;polygalacturonase activity;galacturan 1,4-alpha-galacturonidase activity;carbohydrate metabolic process;cell wall organization	ec:3.2.1.15-pectin depolymerase;ec:3.2.1.67-1,4-alpha-galacturonidase	Starch and sucrose metabolism;Pentose and glucuronate interconversionsStarch and sucrose metabolism;Pentose and glucuronate interconversions

Phvul.007G211800.1	myb transcription factor	DNA binding		
Phvul.007G212600.1	probable glycerol-3-phosphate acyltransferase 3	integral component of membrane;glycerol-3-phosphate O-acyltransferase activity;phosphatase activity;glycerol-3-phosphate 2-O-acyltransferase activity;cutin biosynthetic process;dephosphorylation	ec:3.1;ec:3.1.3.41-nitrophenyl phosphatase;ec:2.3.1.15-1-O-acyltransferase;ec:2.3.1	Aminobenzoate degradationGlycerophospholipid metabolism;Glycerolipid metabolism
Phvul.007G215300.1	DNA replication regulator dpb11,	zinc ion binding		
Phvul.007G216600.1	kynurenine--oxoglutarate transaminase-like	L-aspartate:2-oxoglutarate aminotransferase activity;kynurenine-oxoglutarate transaminase activity;pyridoxal phosphate binding;cysteine-S-conjugate beta-lyase activity;L-phenylalanine:2-oxoglutarate aminotransferase activity;biosynthetic process;L-kynurenine metabolic process	ec:2.6.1.57-transaminase;ec:4.4.1.13-beta-lyase;ec:2.6.1.9-transaminase;ec:2.6.1.7-transaminase;ec:2.6.1.5-transaminase;ec:2.6.1.1-transaminase	Biosynthesis of antibiotics;Tropane, piperidine and pyridine alkaloid biosynthesis;Cysteine and methionine metabolism;Novobiocin biosynthesis;Phenylalanine, tyrosine and tryptophan biosynthesis;Isoquinoline alkaloid biosynthesis;Lysine biosynthesis;Phenylalanine metabolism;Tyrosine metabolismSelenocompound metabolismBiosynthesis of antibiotics;Tropane, piperidine and pyridine alkaloid biosynthesis;Novobiocin biosynthesis;Phenylalanine, tyrosine and tryptophan biosynthesis;Phenylalanine metabolism;Tyrosine metabolism;Histidine metabolismTryptophan metabolismUbiquinone and other terpenoid-quinone biosynthesis;Biosynthesis of antibiotics;Tropane, piperidine and pyridine alkaloid biosynthesis;Cysteine and methionine metabolism;Novobiocin biosynthesis;Phenylalanine, tyrosine and tryptophan biosynthesis;Isoquinoline alkaloid biosynthesis;Phenylalanine metabolism;Tyrosine metabolismArginine and proline metabolism;Biosynthesis of antibiotics;Arginine biosynthesis;Carbon fixation in photosynthetic organisms;Tropane, piperidine and pyridine alkaloid biosynthesis;Cysteine and methionine metabolism;Novobiocin

				biosynthesis;Phenylalanine, tyrosine and tryptophan biosynthesis;Isoquinoline alkaloid biosynthesis;Phenylalanine metabolism;Tyrosine metabolism;Alanine, aspartate and glutamate metabolism
Phvul.007G217300.1	RNI superfamily			
Phvul.007G217400.1	kinesin-4-like isoform X1	kinesin complex;microtubule;microtubule motor activity;ATP binding;microtubule binding;ATPase activity;microtubule-based movement;metabolic process	ec:3.6.1;ec:3.6.1.3-adenylpyrophosphatase;ec:3.6.1.15-phosphatase	Purine metabolismPurine metabolism;Thiamine metabolism
Phvul.007G219100.1	heavy metal-associated domain	cytoplasm;transition metal ion binding;metal ion transport;cellular transition metal ion homeostasis		
Phvul.007G220700.1	serine threonine-kinase CCR1	integral component of membrane;protein serine/threonine kinase activity;ATP binding;protein homodimerization activity;protein phosphorylation;fatty acid catabolic process	ec:2.7.11	
Phvul.007G221000.1	probable dolichyl pyrophosphate Glc1Man9 c2 alpha-1,3-glucosyltransferase	endoplasmic reticulum membrane;integral component of membrane;dolichyl pyrophosphate Man9GlcNAc2 alpha-1,3-glucosyltransferase activity;microtubule cytoskeleton organization;protein folding;protein N-linked glycosylation;oligosaccharide-lipid intermediate biosynthetic process;response to heat;response to high light intensity;response to endoplasmic reticulum stress;response to hydrogen peroxide	ec:2.4.1	
Phvul.007G221000.2	probable dolichyl pyrophosphate Glc1Man9 c2 alpha-1,3-glucosyltransferase	endoplasmic reticulum membrane;integral component of membrane;dolichyl pyrophosphate Man9GlcNAc2 alpha-1,3-glucosyltransferase activity;microtubule cytoskeleton organization;protein folding;protein N-linked glycosylation;oligosaccharide-lipid intermediate biosynthetic process;response to heat;response to high light intensity;response to endoplasmic reticulum stress;response to hydrogen peroxide	ec:2.4.1	
Phvul.007G221200.1	peptide upstream ORF ,	integral component of membrane;S-adenosylmethionine-dependent methyltransferase activity;methylation		
Phvul.007G221300.1	RING U-box	integral component of membrane;zinc ion binding		
Phvul.007G222100.1	serine threonine-kinase D6PKL1-like	cytoplasm;plasma membrane;protein serine/threonine kinase activity;ATP binding;protein phosphorylation;intracellular signal transduction	ec:2.7.11	
Phvul.007G223200.1	polyadenylation and cleavage factor homolog 4-like isoform X1	cytoplasm;mRNA cleavage factor complex;RNA polymerase II core binding;mRNA binding;metal ion binding;termination of RNA polymerase II transcription;mRNA polyadenylation;mRNA cleavage		
Phvul.007G223200.2	polyadenylation and cleavage factor homolog 4-like isoform X1	cytoplasm;mRNA cleavage factor complex;RNA polymerase II core binding;mRNA binding;metal ion binding;termination of RNA polymerase II transcription;mRNA polyadenylation;mRNA cleavage		
Phvul.007G223200.3	polyadenylation and cleavage factor homolog 4-like isoform X1	cytoplasm;mRNA cleavage factor complex;RNA polymerase II core binding;mRNA binding;metal ion binding;termination of RNA polymerase II transcription;mRNA polyadenylation;mRNA cleavage		
Phvul.007G223200.4	polyadenylation and cleavage factor homolog 4-like isoform X1	cytoplasm;mRNA cleavage factor complex;RNA polymerase II core binding;mRNA binding;metal ion binding;termination of RNA polymerase II transcription;mRNA polyadenylation;mRNA cleavage		

Phvul.007G223200.5	polyadenylation and cleavage factor homolog 4-like isoform X1	cytoplasm;mRNA cleavage factor complex;RNA polymerase II core binding;mRNA binding;metal ion binding;termination of RNA polymerase II transcription;mRNA polyadenylation;mRNA cleavage		
Phvul.007G223200.6	polyadenylation and cleavage factor homolog 4-like isoform X1	cytoplasm;mRNA cleavage factor complex;RNA polymerase II core binding;mRNA binding;metal ion binding;termination of RNA polymerase II transcription;mRNA polyadenylation;mRNA cleavage		
Phvul.007G223400.1	heat stress transcription factor A-3-like	nucleus;transcription factor activity, sequence-specific DNA binding;sequence-specific DNA binding;regulation of transcription, DNA-templated		
Phvul.007G224600.1	Myb SANT-like DNA-binding domain	response to stress;pattern specification process;regulation of cellular metabolic process;meristem development		
Phvul.007G226300.1	uncharacterized permease	plasma membrane;plastid envelope;integral component of membrane;nucleobase transmembrane transporter activity;response to UV;flavonoid biosynthetic process;nucleobase transport;pyrimidine nucleobase salvage;transmembrane transport		
Phvul.007G228400.1	glycinol 4-dimethylallyltransferase-like	integral component of membrane;prenyltransferase activity;metabolic process	ec:2.5.1	
Phvul.007G231500.1	S-adenosyl-L-methionine-dependent methyltransferase superfamily	methyltransferase activity;methylation		
Phvul.007G231500.2	S-adenosyl-L-methionine-dependent methyltransferase superfamily	methyltransferase activity;methylation		
Phvul.007G231500.3	S-adenosyl-L-methionine-dependent methyltransferase superfamily	methyltransferase activity;methylation		
Phvul.007G231500.4	S-adenosyl-L-methionine-dependent methyltransferase superfamily	methyltransferase activity;methylation		
Phvul.007G232400.1	cation H(+) antiporter 15-like	endomembrane system;integral component of membrane;monovalent cation:proton antiporter activity;regulation of pH;hydrogen ion transmembrane transport		
Phvul.007G234900.1	pentatricopeptide repeat-containing At5g04810, chloroplastic	nucleotide binding;nucleic acid binding;microtubule-severing ATPase activity;metabolic process	ec:3.6.1;ec:3.6.1.3-adenylpyrophosphatase;ec:3.6.4.3;ec:3.6.1.15-phosphatase	Purine metabolismPurine metabolism;Thiamine metabolism
Phvul.007G236300.1	ankyrin repeat domain-containing 13C-like			
Phvul.007G238000.1	hydroxyethylthiazole kinase	hydroxyethylthiazole kinase activity;phosphorylation;thiamine salvage	ec:2.7.1.50-kinase	Thiamine metabolism
Phvul.007G239200.1	thioredoxin-like 3-2, chloroplastic	cytoplasm;protein disulfide oxidoreductase activity;oxidoreductase activity, acting on a sulfur group of donors, disulfide as acceptor;sulfate assimilation;protein folding;glycerol ether metabolic process;cellular response to oxidative stress;cell redox homeostasis;oxidation-reduction process		
Phvul.007G241800.1	ethylene response factor	nucleus;DNA binding;transcription factor activity, sequence-specific DNA binding;transcription, DNA-templated;regulation of transcription, DNA-templated		
Phvul.007G242000.1	Mitogen-activated kinase kinase kinase 21,	cytoplasm;protein serine/threonine kinase activity;ATP binding;regulation of mitotic cell cycle;signal transduction by protein	ec:2.7.11	

		phosphorylation;stress-activated protein kinase signaling cascade;activation of protein kinase activity;regulation of apoptotic process		
Phvul.007G242100.1	cucumisin-like	cell wall;serine-type endopeptidase activity;proteolysis	ec:3.4.21	
Phvul.007G242200.1	serine carboxypeptidase-like 45	serine-type carboxypeptidase activity;transferase activity, transferring acyl groups other than amino-acyl groups;secondary metabolic process;proteolysis involved in cellular protein catabolic process	ec:3.4.21;ec:3.4.16	
Phvul.007G242400.1	vacuolar amino acid transporter 1	integral component of membrane;amino acid transmembrane transporter activity;amino acid transmembrane transport		
Phvul.007G242400.2	vacuolar amino acid transporter 1	integral component of membrane;amino acid transmembrane transporter activity;amino acid transmembrane transport		
Phvul.007G243100.1	CDPK-related kinase 7-like	cytoplasm;plasma membrane;calmodulin-dependent protein kinase activity;calcium ion binding;calmodulin binding;ATP binding;calcium-dependent protein serine/threonine kinase activity;abscisic acid-activated signaling pathway;peptidyl-serine phosphorylation;intracellular signal transduction;protein autophosphorylation	ec:2.7.11;ec:2.7.11.17	
Phvul.007G243200.1	ABC transporter G family member 24-like	plasma membrane;integral component of membrane;ATP binding;ATPase activity, coupled to transmembrane movement of substances;metabolic process;transmembrane transport	ec:3.6.1;ec:3.6.1.3-adenylpyrophosphatase;ec:3.6.1.15-phosphatase	Purine metabolismPurine metabolism;Thiamine metabolism
Phvul.007G243300.1	plant T8M16-80	integral component of membrane		
Phvul.007G243600.1	exportin-7 isoform X1	nuclear pore;cytoplasm;nuclear export signal receptor activity;Ran GTPase binding;protein export from nucleus		
Phvul.007G243600.2	exportin-7 isoform X1	nuclear pore;cytoplasm;nuclear export signal receptor activity;Ran GTPase binding;protein export from nucleus		
Phvul.007G243600.3	exportin-7 isoform X1	nuclear pore;cytoplasm;nuclear export signal receptor activity;Ran GTPase binding;protein export from nucleus		
Phvul.007G244000.1	RAP annotation release 2, galactose-binding-like domain	chloroplast		
Phvul.007G244700.1	transcriptional corepressor LEUNIG_HOMOLOG isoform X3	transferase activity, transferring glycosyl groups;metabolic process		
Phvul.007G245500.1	serine threonine-kinase CTR1-like isoform X1	intracellular;MAP kinase kinase kinase activity;ATP binding;MAPK cascade;activation of MAPKK activity	ec:2.7.11;ec:2.7.11.25	
Phvul.007G245800.1	serine carboxypeptidase-like	vacuole;cytosol;serine-type carboxypeptidase activity;proteolysis involved in cellular protein catabolic process	ec:3.4.21;ec:3.4.16	
Phvul.007G247800.1	condensin complex subunit 1	condensed chromosome, centromeric region;nuclear condensin complex;extracellular region;chromatin binding;hormone activity;histone binding;mitotic chromosome condensation;meiotic chromosome condensation;cell division;chromosome separation		
Phvul.007G247900.1	L-galactono-1,4-lactone dehydrogenase, mitochondrial	integral component of membrane;D-arabinono-1,4-lactone oxidase activity;galactonolactone dehydrogenase activity;L-gulonolactone oxidase activity;flavin adenine dinucleotide binding;oxidation-reduction process	ec:1.1.3.37;ec:1.1.3.8-oxidase;ec:1.3.2.3-dehydrogenase	Ascorbate and aldarate metabolismAscorbate and aldarate metabolism
Phvul.007G248100.1	probable methyltransferase PMT6	integral component of membrane;S-adenosylmethionine-dependent methyltransferase activity;methylation		
Phvul.007G252500.1	probable receptor kinase At1g67000	integral component of membrane;protein serine/threonine kinase activity;transmembrane receptor protein	ec:2.7.11;ec:2.7.10;ec:2.7.10.1	

		tyrosine kinase activity;ATP binding;peptidyl-tyrosine phosphorylation		
Phvul.007G253800.1	Guanylate kinase	guanylate kinase activity;phosphorylation;GMP metabolic process;GDP metabolic process	ec:2.7.4.8-kinase	Purine metabolism
Phvul.007G254000.1	G-type lectin S-receptor-like serine threonine- kinase SD3-1	plasma membrane;integral component of membrane;protein serine/threonine kinase activity;transmembrane receptor protein tyrosine kinase activity;ATP binding;carbohydrate binding;flower development;peptidyl-tyrosine phosphorylation;recognition of pollen	ec:2.7.11;ec:2.7.10;ec:2.7.10.1	
Phvul.007G254100.1	zinc finger CCCH domain-containing 30	metal ion binding		
Phvul.007G255800.1	hypothetical protein LR48_Vigan02g029700			
Phvul.007G255900.1	serine threonine-kinase Nek7-like isoform X1	protein serine/threonine kinase activity;ATP binding;protein phosphorylation	ec:2.7.11	
Phvul.007G260300.1	probable L-type lectin-domain containing receptor kinase	integral component of membrane;protein serine/threonine kinase activity;ATP binding;carbohydrate binding;protein phosphorylation	ec:2.7.11	
Phvul.007G261300.1	hypothetical protein PHAVU_007G261300g	histamine N-methyltransferase activity;methylation	ec:2.1.1;ec:2.1.1.8-N-methyltransferase	Histidine metabolism
Phvul.007G262000.1	DEAD-box ATP-dependent RNA helicase 37-like	nucleic acid binding;ATP-dependent RNA helicase activity;ATP binding;RNA secondary structure unwinding	ec:3.6.1;ec:3.6.1.3-adenylpyrophosphatase;ec:3.6.1.15-phosphatase;ec:2.7.7	Purine metabolismPurine metabolism;Thiamine metabolism
Phvul.007G263300.1	calmodulin-binding family			
Phvul.007G263400.1	homogentisate solanesyltransferase, chloroplastic	chloroplast envelope;integral component of membrane;homogentisate farnesyltransferase activity;homogentisate geranylgeranyltransferase activity;homogentisate solanesyltransferase activity;plastoquinone biosynthetic process;carotenoid biosynthetic process	ec:2.5.1	
Phvul.007G263400.2	homogentisate solanesyltransferase, chloroplastic	chloroplast envelope;integral component of membrane;homogentisate farnesyltransferase activity;homogentisate geranylgeranyltransferase activity;homogentisate solanesyltransferase activity;plastoquinone biosynthetic process;carotenoid biosynthetic process	ec:2.5.1	
Phvul.007G263400.3	homogentisate solanesyltransferase, chloroplastic	chloroplast envelope;integral component of membrane;homogentisate farnesyltransferase activity;homogentisate geranylgeranyltransferase activity;homogentisate solanesyltransferase activity;plastoquinone biosynthetic process;carotenoid biosynthetic process	ec:2.5.1	
Phvul.007G265400.1	prolycopene isomerase, chloroplastic	oxidoreductase activity;carotenoid isomerase activity;etioplast organization;carotenoid biosynthetic process;oxidation-reduction process		
Phvul.007G265800.1	E3 ubiquitin- ligase RHF2A	zinc ion binding;ligase activity;ubiquitin protein ligase activity;protein polyubiquitination;protein ubiquitination involved in ubiquitin-dependent protein catabolic process;proteasome-mediated ubiquitin-dependent protein catabolic process		
Phvul.007G265800.2	E3 ubiquitin- ligase RHF2A	zinc ion binding;ligase activity;ubiquitin protein ligase activity;protein polyubiquitination;protein ubiquitination involved in ubiquitin-dependent protein catabolic process;proteasome-mediated ubiquitin-dependent protein catabolic process		
Phvul.007G266600.1	EEIG1 EHBP1 amino-terminal domain			

Phvul.007G267400.1	ATP-dependent RNA helicase DHX36 isoform X1	ATP-dependent RNA helicase activity;ATP binding;poly(A) RNA binding;RNA processing;N-terminal protein myristoylation	ec:3.6.1;ec:3.6.1.3-adenylpyrophosphatase;ec:3.6.1.15-phosphatase;ec:2.7.7	Purine metabolismPurine metabolism;Thiamine metabolism
Phvul.007G267500.1	embryo defective 2016	integral component of membrane		
Phvul.007G268900.1	YLS9-like	plasmodesma;integral component of membrane;anchored component of plasma membrane;signal transducer activity;defense response;signal transduction		
Phvul.007G269100.1	Chaperone 1	protein metabolic process		
Phvul.007G270000.1	RRP5 homolog isoform X1	nucleolus;small-subunit processome;RNA binding;mRNA processing		
Phvul.007G270200.1	serine arginine-rich splicing factor 4-like	nucleotide binding;nucleic acid binding;zinc ion binding		
Phvul.007G270500.1	pre-mRNA-processing factor 19-like	ubiquitin-protein transferase activity;ligase activity;protein ubiquitination		
Phvul.007G271500.1	MATH domain-containing At5g43560-like isoform X1			
Phvul.007G271700.1	Ethylene receptor 2	endoplasmic reticulum membrane;integral component of membrane;phosphorelay sensor kinase activity;ethylene receptor activity;ethylene binding;ethylene-activated signaling pathway;negative regulation of ethylene-activated signaling pathway;peptidyl-histidine phosphorylation;signal transduction by protein phosphorylation	ec:2.7.3;ec:2.7.13.3	
Phvul.007G272000.1	plant T32M21-140			
Phvul.007G272200.1	SWI SNF-related matrix-associated actin-dependent regulator of chromatin subfamily A member 3-like 3	chloroplast envelope;nucleic acid binding;helicase activity;ATP binding;zinc ion binding;metabolic process	ec:3.6.1;ec:3.6.1.15-phosphatase	Purine metabolism;Thiamine metabolism
Phvul.007G272500.1	CASP 5B3	plasma membrane;integral component of membrane		
Phvul.007G272600.1	transcription factor bHLH104-like isoform X1	protein dimerization activity		
Phvul.007G273000.1	Ethylene-responsive transcription factor 1B	nucleus;DNA binding;transcription factor activity, sequence-specific DNA binding;transcription, DNA-templated;regulation of transcription, DNA-templated		
Phvul.007G273100.1	Serine threonine-phosphatase PP1	phosphoprotein phosphatase activity;metal ion binding;protein dephosphorylation	ec:3.1;ec:3.1.3.41-nitrophenyl phosphatase;ec:3.1.3.16-phosphatase	Aminobenzoate degradationT cell receptor signaling pathway
Phvul.007G273800.1	glycoside hydrolase family 1	beta-glucosidase activity;carbohydrate metabolic process	ec:3.2.1;ec:3.2.1.21-gentiobiase	Starch and sucrose metabolism;Cyanoamino acid metabolism;Phenylpropanoid biosynthesis
Phvul.007G274800.1	NRT1 PTR FAMILY -like	integral component of membrane;transporter activity;transport		
Phvul.007G275200.1	RNA recognition motif	commitment complex;nuclear speck;U2-type prespliceosome;U2AF;nucleotide binding;poly-pyrimidine tract binding;pre-mRNA 3'-splice site binding;U2-type prespliceosome assembly		
Phvul.007G276400.1	Hemopexin D-tyrosyl-tRNA(Tyr) deacylase	integral component of membrane;metal ion binding		
Phvul.007G276400.2	Hemopexin D-tyrosyl-tRNA(Tyr) deacylase	integral component of membrane;metal ion binding		
Phvul.007G277200.1	hypothetical protein PHAVU_007G277200g			

Phvul.007G277200.2	hypothetical protein PHAVU_007G277200g			
Phvul.007G277200.3	hypothetical protein PHAVU_007G277200g			
Phvul.007G277200.4	hypothetical protein PHAVU_007G277200g			
Phvul.007G277200.5	hypothetical protein PHAVU_007G277200g			
Phvul.007G277200.6	hypothetical protein PHAVU_007G277200g			
Phvul.007G277200.7	hypothetical protein PHAVU_007G277200g			
Phvul.007G277200.8	hypothetical protein PHAVU_007G277200g			
Phvul.007G278800.1	dentin sialophospho ,			
Phvul.007G279400.1	armadillo repeat-containing LFR	nucleus;mitotic recombination;embryo sac egg cell differentiation;flower development;leaf development		
Phvul.007G280600.1	sieve element occlusion			
Phvul.007G281000.1	DNA-binding SMUBP-2	chloroplast stroma;chloroplast envelope;DNA binding;ATP binding;hydrolase activity;chlorophyll catabolic process		
Phvul.008G000200.1	probable receptor kinase At2g39360	integral component of membrane;protein serine/threonine kinase activity;ATP binding;protein phosphorylation	ec:2.7.11	
Phvul.008G001800.1	ras-related RABA5e	intracellular;membrane;GTPase activity;GTP binding;intracellular protein transport;nucleocytoplasmic transport;small GTPase mediated signal transduction;metabolic process	ec:3.6.1;ec:3.6.1.15-phosphatase	Purine metabolism;Thiamine metabolism
Phvul.008G002200.1	E3 ubiquitin- ligase LIN-1	ubiquitin-protein transferase activity;zinc ion binding;amino acid binding;ligase activity;protein ubiquitination		
Phvul.008G002300.1	callose synthase 3-like	1,3-beta-D-glucan synthase complex;integral component of membrane;1,3-beta-D-glucan synthase activity;(1->3)-beta-D-glucan biosynthetic process	ec:2.4.1.34-synthase;ec:2.4.1	Starch and sucrose metabolism
Phvul.008G002500.1	serine threonine-kinase VRK1	protein serine/threonine kinase activity;ATP binding;protein N-linked glycosylation;Wnt signaling pathway;peptidyl-serine phosphorylation	ec:2.7.11	
Phvul.008G002600.1	superkiller viralicidic activity 2-like 2	nucleus;RNA binding;RNA helicase activity;ATP binding;mRNA splicing, via spliceosome;double-strand break repair via homologous recombination;karyogamy;RNA catabolic process;mRNA export from nucleus;protein import into nucleus;embryo sac egg cell differentiation;photomorphogenesis;regulation of flower development;maintenance of meristem identity;response to ionizing radiation;cullin deneddylation;protein ubiquitination;histone methylation;protein deubiquitination;production of siRNA involved in RNA interference;rRNA 3'-end processing;production of miRNAs involved in gene silencing by miRNA;post-translational protein modification;positive regulation of transcription, DNA-templated;cotyledon development;cell division	ec:3.6.1;ec:3.6.1.15-phosphatase;ec:2.7.7	Purine metabolism;Thiamine metabolism

Phvul.008G003600.1	transaldolase family	mitochondrion;chloroplast stroma;sedoheptulose-7-phosphate:D-glyceraldehyde-3-phosphate glyceronetransferase activity;glycolytic process;pentose-phosphate shunt;aerobic respiration;lignin biosynthetic process;shikimate biosynthetic process	ec:2.2.1.2-dihydroxyacetonetransferase	Biosynthesis of antibiotics;Pentose phosphate pathway
Phvul.008G006600.1	pentatricopeptide repeat-containing At1g20300, mitochondrial-like			
Phvul.008G006700.1	ribosomal RNA-processing	integral component of membrane;CURI complex;UTP-C complex;ribosomal small subunit assembly;rRNA processing;protein targeting to mitochondrion;embryo sac egg cell differentiation;vesicle-mediated transport		
Phvul.008G007100.1	DUF4378 domain			
Phvul.008G008300.1	lisH domain and HEAT repeat KIAA1468			
Phvul.008G008300.2	lisH domain and HEAT repeat KIAA1468			
Phvul.008G008700.1	hypothetical protein PHAVU_008G008700g			
Phvul.008G011900.1	probable mediator of RNA polymerase II transcription subunit 37c	ATP binding		
Phvul.008G012400.1	uncharacterized CRM domain-containing At3g25440, chloroplastic	RNA binding		
Phvul.008G012400.2	uncharacterized CRM domain-containing At3g25440, chloroplastic	RNA binding		
Phvul.008G012400.3	uncharacterized CRM domain-containing At3g25440, chloroplastic	RNA binding		
Phvul.008G012400.4	uncharacterized CRM domain-containing At3g25440, chloroplastic	RNA binding		
Phvul.008G012500.1	dof zinc finger -like	DNA binding;regulation of transcription, DNA-templated		
Phvul.008G012500.2	dof zinc finger -like	DNA binding;regulation of transcription, DNA-templated		
Phvul.008G013700.1	zinc-finger ,			
Phvul.008G014800.1	F-box plant			
Phvul.008G017200.1	sphingosine-1-phosphate lyase	cytoplasm;sphinganine-1-phosphate aldolase activity;carboxy-lyase activity;pyridoxal phosphate binding;fatty acid beta-oxidation;ER to Golgi vesicle-mediated transport;toxin catabolic process;sphingolipid catabolic process;proteasome-mediated ubiquitin-dependent protein catabolic process;response to misfolded protein;proteasome core complex assembly	ec:4.1.2.27-aldolase	Sphingolipid metabolism
Phvul.008G017700.1	bZIP transcription factor bZIP56 precursor	nucleic acid binding;hydrolase activity		
Phvul.008G020800.1	peptide nitrate transporter	integral component of membrane;transporter activity;transport		

Phvul.008G021200.1	trichome birefringence-like 19	Golgi apparatus;integral component of membrane;O-acetyltransferase activity;cell wall organization or biogenesis	ec:2.3.1	
Phvul.008G021300.1	hypothetical protein PHAVU_008G021300g			
Phvul.008G021500.1	cell division cycle 27 homolog B-like isoform X1	spindle;cell plate;mitotic recombination;regulation of mitotic cell cycle;embryo sac egg cell differentiation;response to auxin;leaf morphogenesis;vernalization response;root meristem specification;regulation of DNA endoreduplication;DNA endoreduplication;proteasome-mediated ubiquitin-dependent protein catabolic process;proteasome assembly;root cap development;cell division;regulation of unidimensional cell growth;response to misfolded protein		
Phvul.008G022900.1	ribosome biogenesis BRX1-like	nucleolus;RNA binding;ribosomal large subunit assembly		
Phvul.008G022900.2	ribosome biogenesis BRX1-like	nucleolus;RNA binding;ribosomal large subunit assembly		
Phvul.008G023600.1	hypothetical protein PHAVU_008G023600g, partial			
Phvul.008G026100.1	UDP-glucosyltransferase family	intracellular membrane-bounded organelle;quercetin 3-O-glucosyltransferase activity;quercetin 7-O-glucosyltransferase activity;flavonoid biosynthetic process;flavonoid glucuronidation	ec:2.4.1	
Phvul.008G026100.2	UDP-glucosyltransferase family	intracellular membrane-bounded organelle;quercetin 3-O-glucosyltransferase activity;quercetin 7-O-glucosyltransferase activity;flavonoid biosynthetic process;flavonoid glucuronidation	ec:2.4.1	
Phvul.008G026100.3	UDP-glucosyltransferase family	intracellular membrane-bounded organelle;quercetin 3-O-glucosyltransferase activity;quercetin 7-O-glucosyltransferase activity;flavonoid biosynthetic process;flavonoid glucuronidation	ec:2.4.1	
Phvul.008G026700.1	receptor kinase FERONIA	protein kinase activity;ATP binding;protein phosphorylation		
Phvul.008G027300.1	serine-threonine kinase isoform X1	protein kinase activity;ATP binding;protein phosphorylation		
Phvul.008G027700.1	ultraviolet-B receptor UVR8	ligase activity;metabolic process		
Phvul.008G028100.1	hyoscyamine 6-dioxygenase-like	metal ion binding;dioxygenase activity;oxidation-reduction process		
Phvul.008G028100.2	hyoscyamine 6-dioxygenase-like	metal ion binding;dioxygenase activity;oxidation-reduction process		
Phvul.008G029500.1	serine-threonine kinase isoform X1	protein kinase activity;ATP binding;protein phosphorylation		
Phvul.008G031100.1	receptor kinase FERONIA	protein kinase activity;ATP binding;protein phosphorylation		
Phvul.008G031200.1	disease resistance At1g50180	ADP binding;defense response		
Phvul.008G031300.1	Receptor kinase ANXUR2	protein kinase activity;ATP binding;protein phosphorylation		
Phvul.008G031500.1	ubiquitin-like domain-containing CTD phosphatase 1 isoform X1	phosphoprotein phosphatase activity;protein dephosphorylation	ec:3.1;ec:3.1.3.41-nitrophenyl phosphatase;ec:3.1.3.16-phosphatase	Aminobenzoate degradationT cell receptor signaling pathway
Phvul.008G031500.2	ubiquitin-like domain-containing CTD phosphatase 1 isoform X1	phosphoprotein phosphatase activity;protein dephosphorylation	ec:3.1;ec:3.1.3.41-nitrophenyl phosphatase;ec:3.1.3.16-phosphatase	Aminobenzoate degradationT cell receptor signaling pathway
Phvul.008G032100.1	Anthocyanin 5-aromatic acyltransferase	shikimate O-hydroxycinnamoyltransferase activity;metabolic process	ec:2.3.1;ec:2.3.1.133-O-hydroxycinnamoyltransferase	Stilbenoid, diarylheptanoid and gingerol biosynthesis;Flavonoid

				biosynthesis;Phenylpropanoid biosynthesis
Phvul.008G032100.2	Anthocyanin 5-aromatic acyltransferase	shikimate O-hydroxycinnamoyltransferase activity;metabolic process	ec:2.3.1;ec:2.3.1.133-O-hydroxycinnamoyltransferase	Stilbenoid, diarylheptanoid and gingerol biosynthesis;Flavonoid biosynthesis;Phenylpropanoid biosynthesis
Phvul.008G032200.1	Anthocyanin 5-aromatic acyltransferase	shikimate O-hydroxycinnamoyltransferase activity;metabolic process	ec:2.3.1;ec:2.3.1.133-O-hydroxycinnamoyltransferase	Stilbenoid, diarylheptanoid and gingerol biosynthesis;Flavonoid biosynthesis;Phenylpropanoid biosynthesis
Phvul.008G033900.1	Magnesium transporter MRS2-3	integral component of membrane;single-organism transport		
Phvul.008G035600.1	chaperone 3, chloroplastic	chloroplast stroma;hydrolase activity, hydrolyzing O-glycosyl compounds;ATP binding;carbohydrate metabolic process;response to heat;chloroplast organization;protein processing		
Phvul.008G039500.1	SNF1-related kinase catalytic subunit alpha KIN10	nuclear ubiquitin ligase complex;cytoplasm;calmodulin-dependent protein kinase activity;cAMP-dependent protein kinase activity;ATP binding;developmental process involved in reproduction;glucose catabolic process;protein phosphorylation;protein glycosylation;detection of nutrient;abscisic acid-activated signaling pathway;vegetative phase change;sugar mediated signaling pathway;organ senescence;intracellular signal transduction;negative regulation of apoptotic process;primary root development	ec:2.7.11;ec:2.7.11.17;ec:2.7.11.11-protein kinase	mTOR signaling pathway
Phvul.008G039500.2	SNF1-related kinase catalytic subunit alpha KIN10	nuclear ubiquitin ligase complex;cytoplasm;calmodulin-dependent protein kinase activity;cAMP-dependent protein kinase activity;ATP binding;developmental process involved in reproduction;glucose catabolic process;protein phosphorylation;protein glycosylation;detection of nutrient;abscisic acid-activated signaling pathway;vegetative phase change;sugar mediated signaling pathway;organ senescence;intracellular signal transduction;negative regulation of apoptotic process;primary root development	ec:2.7.11;ec:2.7.11.17;ec:2.7.11.11-protein kinase	mTOR signaling pathway
Phvul.008G039600.1	DUF1336 family			
Phvul.008G039600.2	DUF1336 family			
Phvul.008G039600.3	DUF1336 family			
Phvul.008G042700.1	polyvinylalcohol dehydrogenase-like			
Phvul.008G044700.1	LRR receptor-like kinase family	integral component of membrane;protein kinase activity;ATP binding;protein phosphorylation		
Phvul.008G045100.1	Homeobox-leucine zipper HAT3	nucleus;transcription factor activity, sequence-specific DNA binding;sequence-specific DNA binding;transcription, DNA-templated;regulation of transcription, DNA-templated		
Phvul.008G045400.1	cationic amino acid transporter 9, chloroplastic-like	vacuolar membrane;integral component of membrane;L-amino acid transmembrane transporter activity;antiporter activity;fatty acid beta-oxidation;protein import into peroxisome matrix;L-alpha-amino acid transmembrane transport		
Phvul.008G045400.2	cationic amino acid transporter 9, chloroplastic-like	vacuolar membrane;integral component of membrane;L-amino acid transmembrane transporter activity;antiporter activity;fatty acid beta-oxidation;protein import into peroxisome matrix;L-alpha-amino acid transmembrane transport		

Phvul.008G047900.1	subtilisin-like protease Glyma18g48580	cell wall;serine-type endopeptidase activity;proteolysis	ec:3.4.21	
Phvul.008G048900.1	cysteine protease ATG4B	cytosol;cysteine-type endopeptidase activity;autophagosome assembly;mitophagy;C-terminal protein lipidation;protein targeting to membrane;protein processing;nucleophagy;protein delipidation	ec:3.4;ec:3.4.22	
Phvul.008G048900.2	cysteine protease ATG4B	cytosol;cysteine-type endopeptidase activity;autophagosome assembly;mitophagy;C-terminal protein lipidation;protein targeting to membrane;protein processing;nucleophagy;protein delipidation	ec:3.4;ec:3.4.22	
Phvul.008G049000.1	Aldolase-type TIM barrel family isoform 1	cytoplasm;3-deoxy-8-phosphooctulonate synthase activity;biosynthetic process	ec:2.5.1.55-synthase	Lipopolysaccharide biosynthesis
Phvul.008G049100.1	Heterogeneous nuclear ribonucleo Q	viral nucleocapsid;intracellular ribonucleoprotein complex;nucleotide binding;nucleic acid binding		
Phvul.008G049100.2	Heterogeneous nuclear ribonucleo Q	viral nucleocapsid;intracellular ribonucleoprotein complex;nucleotide binding;nucleic acid binding		
Phvul.008G050200.1	acyl-coenzyme A thioesterase 9, mitochondrial	peroxisome;hydrolase activity;isomerase activity;metabolic process		
Phvul.008G051800.1	chromatin assembly factor 1 subunit FAS1	nucleus;integral component of membrane;CAF-1 complex;DNA-dependent DNA replication;DNA recombination;nucleosome assembly;multicellular organismal development;single-organism cellular process;anatomical structure development;regulation of cell cycle;regulation of macromolecule metabolic process		
Phvul.008G052000.1	AP2-like ethylene-responsive transcription factor BBM2	nucleus;DNA binding;transcription factor activity, sequence-specific DNA binding;transcription, DNA-templated;regulation of transcription, DNA-templated;multicellular organismal development		
Phvul.008G052100.1	phospholipase A(1) LCAT3	O-acyltransferase activity;lipid metabolic process	ec:2.3.1	
Phvul.008G052900.1	Membrane-associated VIPP1, chloroplastic	cytosol;plastid chromosome;chloroplast thylakoid membrane;chloroplast stroma;chloroplast envelope;thylakoid membrane organization;vesicle organization		
Phvul.008G053300.1	thioredoxin F-type, chloroplastic-like	chloroplast;protein disulfide oxidoreductase activity;oxidoreductase activity, acting on a sulfur group of donors, disulfide as acceptor;sulfate assimilation;protein folding;glycerol ether metabolic process;cellular response to oxidative stress;cell redox homeostasis;oxidation-reduction process		
Phvul.008G053400.1	aspartic ase 2	integral component of membrane;aspartic-type endopeptidase activity;transferase activity;proteolysis;protein catabolic process	ec:3.4.23	
Phvul.008G054400.1	photosystem I reaction center subunit XI, chloroplastic	chloroplast thylakoid membrane;photosystem I reaction center;chloroplast envelope;plastoglobule;integral component of membrane;rRNA processing;plastid organization;photosystem II assembly;cellular cation homeostasis;divalent metal ion transport		
Phvul.008G056700.1	subtilisin-like protease	cell wall;serine-type endopeptidase activity;proteolysis	ec:3.4.21	
Phvul.008G057500.1	golgin candidate 3-like isoform X2			
Phvul.008G059100.1	hypothetical protein PHAVU_008G059100g			
Phvul.008G059100.2	hypothetical protein			

	PHAVU_008G05910 0g			
Phvul.008G0 60200.1	RIC1 homolog			
Phvul.008G0 60300.1	E3 ubiquitin- ligase RHA1B-like	nucleus;integral component of membrane;zinc ion binding;response to brassinosteroid;response to chitin;single-organism process		
Phvul.008G0 60800.1	AT-rich interactive domain-containing 1-like	DNA binding		
Phvul.008G0 60800.2	AT-rich interactive domain-containing 1-like	DNA binding		
Phvul.008G0 60800.3	AT-rich interactive domain-containing 1-like	DNA binding		
Phvul.008G0 65600.1	Beta-amylase 7	nucleus;transcription factor activity, sequence-specific DNA binding;beta-amylase activity;palmitoyl-CoA hydrolase activity;polysaccharide catabolic process;regulation of transcription, DNA-templated;vernalization response;regulation of shoot system development	ec:3.2.1;ec:3.1.2.2-hydrolase;ec:3.1.2;ec:3.1.2.20;ec:3.2.1.2-saccharogen amylase	Biosynthesis of unsaturated fatty acids;Fatty acid elongationStarch and sucrose metabolism
Phvul.008G0 65800.1	hypothetical protein PHAVU_008G06580 0g	membrane		
Phvul.008G0 67800.1	K(+) efflux antiporter 2, chloroplastic-like	integral component of membrane;zinc ion binding;solute:proton antiporter activity;potassium ion transport;hydrogen ion transmembrane transport		
Phvul.008G0 67800.2	K(+) efflux antiporter 2, chloroplastic-like	integral component of membrane;zinc ion binding;solute:proton antiporter activity;potassium ion transport;hydrogen ion transmembrane transport		
Phvul.008G0 68800.1	hypothetical protein PHAVU_008G06880 0g	mitochondrial respiratory chain complex I;ubiquitin-dependent protein catabolic process;photorespiration;response to misfolded protein;proteasome core complex assembly		
Phvul.008G0 69600.1	disease resistance RPP13 4			
Phvul.008G0 69900.1	SET domain	protein-lysine N-methyltransferase activity;[ribulose-bisphosphate carboxylase]-lysine N-methyltransferase activity;embryo sac egg cell differentiation;peptidyl-lysine monomethylation	ec:2.1.1;ec:2.1.1.127	
Phvul.008G0 71700.1	vacuolar 8	ligase activity;metabolic process		
Phvul.008G0 71700.2	ARM repeat superfamily isoform 1	ligase activity;metabolic process		
Phvul.008G0 71700.3	ARM repeat superfamily isoform 1	ligase activity;metabolic process		
Phvul.008G0 75100.1	ankyrin repeat and zinc finger domain-containing 1	integral component of membrane;Cdc48p-Npl4p-Vms1p AAA ATPase complex;thiol-dependent ubiquitin-specific protease activity;metal ion binding;ER-associated ubiquitin-dependent protein catabolic process;nucleus-associated proteasomal ubiquitin-dependent protein catabolic process;mitochondria-associated ubiquitin-dependent protein catabolic process	ec:3.4;ec:3.4.19.12	
Phvul.008G0 75200.1	smad FHA domain	transcription factor activity, sequence-specific DNA binding;regulation of transcription, DNA-templated		
Phvul.008G0 75400.1	4-hydroxy-tetrahydrodipicolin ate synthase, chloroplastic	chloroplast;4-hydroxy-tetrahydrodipicolinate synthase;lysine biosynthetic process via diaminopimelate;diaminopimelate biosynthetic process	ec:4.3.3.7-synthase	Biosynthesis of antibiotics;Lysine biosynthesis;Monobact am biosynthesis
Phvul.008G0 75600.1	brefeldin A-inhibited guanine	trans-Golgi network;cytosol;ARF guanyl-nucleotide exchange factor activity;vesicle-mediated transport;regulation of ARF protein		

	nucleotide-exchange 2	signal transduction;positive regulation of GTPase activity		
Phvul.008G077600.1	cysteine-rich receptor-kinase	integral component of membrane;protein kinase activity;ATP binding;protein phosphorylation		
Phvul.008G079500.1	zinc transporter At3g08650	Golgi membrane;trans-Golgi network;integral component of membrane;DNA-directed DNA polymerase activity;zinc ion transmembrane transporter activity;zinc II ion transmembrane transport;DNA biosynthetic process	ec:2.7.7.7-DNA polymerase	Purine metabolism;Pyrimidine metabolism
Phvul.008G079900.1	acetyltransferase NSI	chloroplast;protein acetyltransferase complex;peptide alpha-N-acetyltransferase activity;pentose-phosphate shunt;N-terminal protein amino acid acetylation	ec:2.3.1.88;ec:2.3.1	
Phvul.008G080300.1	4-hydroxybenzoate polyprenyltransferase, mitochondrial	integral component of membrane;4-hydroxybenzoate decaprenyltransferase activity;4-hydroxybenzoate nonaprenyltransferase activity;ubiquinone biosynthetic process	ec:2.5.1.39-polyprenyltransferase;ec:2.5.1	Ubiquinone and other terpenoid-quinone biosynthesis
Phvul.008G080600.1	WEB family At1g65010, chloroplastic isoform X1			
Phvul.008G081700.1	phospholipid-transporting ATPase 1-like	plasma membrane;integral component of membrane;magnesium ion binding;phospholipid-translocating ATPase activity;ATP binding;metabolic process;phospholipid translocation	ec:3.6.1;ec:3.6.1.3-adenylpyrophosphatase;ec:3.6.1.15-phosphatase;ec:3.6.3.1	Purine metabolismPurine metabolism;Thiamine metabolism
Phvul.008G081700.2	phospholipid-transporting ATPase 1-like	plasma membrane;integral component of membrane;magnesium ion binding;phospholipid-translocating ATPase activity;ATP binding;metabolic process;phospholipid translocation	ec:3.6.1;ec:3.6.1.3-adenylpyrophosphatase;ec:3.6.1.15-phosphatase;ec:3.6.3.1	Purine metabolismPurine metabolism;Thiamine metabolism
Phvul.008G082200.1	vacuolar-sorting receptor 1-like	integral component of membrane;calcium ion binding		
Phvul.008G082600.1	transmembrane ,	integral component of membrane		
Phvul.008G083000.1	probable receptor kinase At3g55450	protein kinase activity;ATP binding;protein phosphorylation;autophagy		
Phvul.008G083900.1	transmembrane ,	plasma membrane;integral component of membrane;helicase activity;response to oxidative stress;metabolic process;pollen development	ec:3.6.1;ec:3.6.1.15-phosphatase	Purine metabolism;Thiamine metabolism
Phvul.008G084300.1	adenine guanine permease AZG1-like	integral component of membrane;purine nucleobase transmembrane transporter activity;adenine transport;guanine transport;purine nucleobase transmembrane transport		
Phvul.008G086000.1	WW domain ,			
Phvul.008G086300.1	transport SFT2	integral component of membrane;vesicle-mediated transport		
Phvul.008G089400.1	tubby C 2			
Phvul.008G089400.2	tubby C 2			
Phvul.008G093400.1	trichome birefringence-like 34	Golgi apparatus;integral component of membrane;O-acetyltransferase activity;glucuronoxylan metabolic process;xylan biosynthetic process	ec:2.3.1	
Phvul.008G094500.1	lectin-domain containing receptor kinase -like	integral component of membrane;protein serine/threonine kinase activity;transmembrane receptor protein tyrosine kinase activity;ATP binding;carbohydrate binding;peptidyl-tyrosine phosphorylation	ec:2.7.11;ec:2.7.10;ec:2.7.10.1	
Phvul.008G094700.1	ethylene-overproduction 1-like	protein binding, bridging;protein N-linked glycosylation;hormone-mediated signaling pathway;sugar mediated signaling pathway;regulation of ethylene biosynthetic process;cellular response to phosphate starvation;stem cell division;proteasome-mediated ubiquitin-dependent protein catabolic process;cotyledon		

		development;regulation of post-embryonic root development		
Phvul.008G095000.1	BAG family molecular chaperone regulator 4	chaperone binding		
Phvul.008G095300.1	receptor 12	membrane		
Phvul.008G097300.1	beta-glucosidase 11-like	beta-glucosidase activity;carbohydrate metabolic process	ec:3.2.1;ec:3.2.1.21-gentiobiase	Starch and sucrose metabolism;Cyanoamino acid metabolism;Phenylpropanoid biosynthesis
Phvul.008G098000.1	lethal(2) giant larvae homolog SRO77-like	cytoplasm;plasma membrane;integral component of membrane;GTPase activator activity;Rab GTPase binding;syntaxin binding;vesicle-mediated transport;regulation of exocytosis;positive regulation of GTPase activity		
Phvul.008G099500.1	U-box domain-containing 13-like isoform X2	ubiquitin-protein transferase activity;ligase activity;protein ubiquitination		
Phvul.008G100700.1	respiratory burst oxidase homolog H isoform X1	integral component of membrane;peroxidase activity;calcium ion binding;NAD(P)H oxidase activity;oxidation-reduction process;cellular oxidant detoxification	ec:1.6.3.1;ec:1.11.1.7-lactoperoxidase	Phenylpropanoid biosynthesis
Phvul.008G104000.1	testis-expressed sequence 10			
Phvul.008G104000.2	testis-expressed sequence 10			
Phvul.008G104600.1	probable envelope ADP,ATP carrier , chloroplastic	integral component of membrane;structural constituent of ribosome;translation;transmembrane transport		
Phvul.008G105900.1	pentatricopeptide repeat-containing At4g28010	microtubule-severing ATPase activity;metabolic process;petal formation;sepal formation	ec:3.6.1;ec:3.6.1.3-adenylpyrophosphatase;ec:3.6.4.3;ec:3.6.1.15-phosphatase	Purine metabolismPurine metabolism;Thiamine metabolism
Phvul.008G105900.2	pentatricopeptide repeat-containing At4g28010	microtubule-severing ATPase activity;metabolic process;petal formation;sepal formation	ec:3.6.1;ec:3.6.1.3-adenylpyrophosphatase;ec:3.6.4.3;ec:3.6.1.15-phosphatase	Purine metabolismPurine metabolism;Thiamine metabolism
Phvul.008G105900.3	pentatricopeptide repeat-containing At4g28010	microtubule-severing ATPase activity;metabolic process;petal formation;sepal formation	ec:3.6.1;ec:3.6.1.3-adenylpyrophosphatase;ec:3.6.4.3;ec:3.6.1.15-phosphatase	Purine metabolismPurine metabolism;Thiamine metabolism
Phvul.008G106300.1	auxin transporter 2	plasma membrane;integral component of membrane;amino acid transmembrane transporter activity;symporter activity;amino acid transmembrane transport;auxin-activated signaling pathway		
Phvul.008G107600.1	AT-rich interactive domain-containing 1-like	DNA binding		
Phvul.008G108400.1	LRR receptor-like serine threonine-kinase GSO1	integral component of membrane;transferase activity		
Phvul.008G108500.1	LRR receptor-like serine threonine-kinase GSO1			
Phvul.008G109000.1	LRR receptor-like serine threonine-kinase GSO1			
Phvul.008G111500.1	L-type lectin-domain containing receptor kinase -like	integral component of membrane;kinase activity;carbohydrate binding;phosphorylation		
Phvul.008G113600.1	glutathione S-transferase	cytoplasm;glutathione transferase activity;glutathione metabolic process;toxin catabolic process	ec:2.5.1.18-transferase	Drug metabolism - cytochrome P450;Metabolism of xenobiotics by cytochrome P450;Glutathione metabolism
Phvul.008G114800.1	DUF674 family	integral component of membrane		

Phvul.008G115500.1	wall-associated receptor kinase-like 20	integral component of membrane;protein serine/threonine kinase activity;ATP binding;polysaccharide binding;protein phosphorylation;regulation of cellular process	ec:2.7.11	
Phvul.008G115700.1	bHLH transcription factor	protein dimerization activity		
Phvul.008G119700.1	scarecrow 9	transcription factor activity, sequence-specific DNA binding;transcription, DNA-templated;regulation of transcription, DNA-templated		
Phvul.008G120800.1	hypothetical protein PHAVU_008G120800g	integral component of membrane		
Phvul.008G121100.1	hypothetical protein PHAVU_008G121100g	integral component of membrane		
Phvul.008G122400.1	kinase TMKL1	plasma membrane;plasmodesma;integral component of membrane;protein kinase activity;ATP binding;protein phosphorylation;determination of bilateral symmetry;polarity specification of adaxial/abaxial axis;meristem initiation;regulation of meristem growth		
Phvul.008G122500.1	RINT1 MAG2	cytosol;extrinsic component of endoplasmic reticulum membrane;protein targeting to vacuole;ER to Golgi vesicle-mediated transport		
Phvul.008G123700.1	SULFUR DEFICIENCY-INDUCED 1-like	regulation of sulfur utilization		
Phvul.008G125300.1	root UVB sensitive 4 isoform X2	integral component of membrane		
Phvul.008G125300.2	root UVB sensitive 4 isoform X2	integral component of membrane		
Phvul.008G125300.3	UPF0420 C16orf58	integral component of membrane		
Phvul.008G125300.4	UPF0420 C16orf58	integral component of membrane		
Phvul.008G125400.1	Transcriptional corepressor LEUNIG	membrane coat;structural molecule activity;intracellular protein transport;vesicle-mediated transport		
Phvul.008G127100.1	G-type lectin S-receptor-like Serine Threonine-kinase	plasma membrane;plasmodesma;integral component of membrane;protein serine/threonine kinase activity;ATP binding;carbohydrate binding;ubiquitin protein ligase binding;protein phosphorylation;innate immune response;recognition of pollen	ec:2.7.11	
Phvul.008G130900.1	hypothetical protein PHAVU_008G130900g			
Phvul.008G132200.1	galacturonosyltransferase 8	Golgi membrane;integral component of membrane;polygalacturonate 4-alpha-galacturonosyltransferase activity;pectin biosynthetic process;cell wall organization	ec:2.4.1.43-4-alpha-galacturonosyltransferase	Amino sugar and nucleotide sugar metabolism;Starch and sucrose metabolism
Phvul.008G133300.1	hypothetical protein PHAVU_008G1333001g, partial	oxidoreductase activity;organic cyclic compound binding;heterocyclic compound binding;response to stimulus		
Phvul.008G134400.1	U-box domain-containing 9	ubiquitin-protein transferase activity;ligase activity;transmembrane receptor protein serine/threonine kinase binding;protein ubiquitination;protein autophosphorylation;cellular response to abscisic acid stimulus		
Phvul.008G135500.1	Ubiquinone biosynthesis monooxygenase COQ6	integral component of membrane;oxidoreductase activity, acting on paired donors, with incorporation or reduction of molecular oxygen, NAD(P)H as one donor, and incorporation of one atom of oxygen;FAD binding;ubiquinone biosynthetic process;methylglyoxal catabolic process to D-lactate via S-lactoyl-glutathione;secondary	ec:1.14.13	

		metabolite biosynthetic process;oxidation-reduction process		
Phvul.008G135500.2	Ubiquinone biosynthesis monooxygenase COQ6	integral component of membrane;oxidoreductase activity, acting on paired donors, with incorporation or reduction of molecular oxygen, NAD(P)H as one donor, and incorporation of one atom of oxygen;FAD binding;ubiquinone biosynthetic process;methylglyoxal catabolic process to D-lactate via S-lactoyl-glutathione;secondary metabolite biosynthetic process;oxidation-reduction process	ec:1.14.13	
Phvul.008G136000.1	embryo defective 2752	integral component of membrane		
Phvul.008G137100.1	lipid-transfer DIR1	integral component of membrane		
Phvul.008G137400.1	SOS response-associated peptidase			
Phvul.008G137400.2	SOS response-associated peptidase			
Phvul.008G139000.1	Eukaryotic translation initiation factor 4G	translation initiation factor activity;translational initiation		
Phvul.008G139000.2	Eukaryotic translation initiation factor 4G	translation initiation factor activity;translational initiation		
Phvul.008G142800.1	hypothetical protein PHAVU_008G142800g	integral component of membrane		
Phvul.008G143400.1	scarecrow 6	transcription, DNA-templated;regulation of transcription, DNA-templated		
Phvul.008G143400.2	scarecrow 6	transcription, DNA-templated;regulation of transcription, DNA-templated		
Phvul.008G144900.1	reticulocyte binding 2 homolog b isoform X4			
Phvul.008G145200.1	guanine nucleotide exchange factor,	intracellular part;cytoskeleton organization;single-multicellular organism process;single-organism developmental process;meristem development;regulation of biological process;organic substance metabolic process;single-organism organelle organization		
Phvul.008G147900.1	sieve element occlusion			
Phvul.008G149500.1	E3 ubiquitin- ligase mib1	transferase activity;ligase activity;metabolic process		
Phvul.008G150100.1	transcription factor GTE8			
Phvul.008G150100.2	transcription factor GTE8			
Phvul.008G151700.1	10 kDa chaperonin	cytoplasm;protein folding		
Phvul.008G152400.1	RING-H2 finger ATL46-like	integral component of membrane;zinc ion binding		
Phvul.008G152400.2	RING-H2 finger ATL46-like	integral component of membrane;zinc ion binding		
Phvul.008G153100.1	GPI ethanolamine phosphate transferase 1	endoplasmic reticulum membrane;integral component of membrane;sulfuric ester hydrolase activity;mannose-ethanolamine phosphotransferase activity;GPI anchor biosynthetic process		
Phvul.008G155000.1	cytochrome P450 family 71	integral component of membrane;iron ion binding;oxidoreductase activity, acting on paired donors, with incorporation or reduction of molecular oxygen, NAD(P)H as one donor, and incorporation of one atom of oxygen;heme binding;secondary metabolite biosynthetic process;oxidation-reduction process	ec:1.14.13	
Phvul.008G155100.1	CASP 4A1	cytosol;plasma membrane;integral component of membrane;NADH dehydrogenase activity;iron-sulfur cluster binding;iron-sulfur cluster assembly	ec:1.6.99.3-dehydrogenase	Oxidative phosphorylation

Phvul.008G156800.1	zinc ion-binding			
Phvul.008G162300.1	esterase-like isoform X1	hydrolase activity, acting on ester bonds;9,9'-dicis-carotene:quinone oxidoreductase activity;7,9,9'-tricis-neurosporene:quinone oxidoreductase activity;9,9'-di-cis-zeta-carotene desaturation to 7,9,7',9'-tetra-cis-lycopene;oxidation-reduction process	ec:1.3.5.6-desaturase	Carotenoid biosynthesis
Phvul.008G162300.2	esterase-like isoform X1	hydrolase activity, acting on ester bonds;9,9'-dicis-carotene:quinone oxidoreductase activity;7,9,9'-tricis-neurosporene:quinone oxidoreductase activity;9,9'-di-cis-zeta-carotene desaturation to 7,9,7',9'-tetra-cis-lycopene;oxidation-reduction process	ec:1.3.5.6-desaturase	Carotenoid biosynthesis
Phvul.008G163100.1	long chain acyl-synthetase 9, chloroplastic-like	membrane;ligase activity;metabolic process		
Phvul.008G165800.1	seleno K	integral component of membrane;response to stress;response to abiotic stimulus;regulation of biological process;cellular response to stimulus		
Phvul.008G166800.1	OSBP(oxysterol-binding)-related 1C			
Phvul.008G167400.1	ABC transporter C family member 3-like	integral component of membrane;ATP binding;ATPase activity, coupled to transmembrane movement of substances;metabolic process;transmembrane transport	ec:3.6.1;ec:3.6.1.3-adenylpyrophosphatase;ec:3.6.1.15-phosphatase	Purine metabolismPurine metabolism;Thiamine metabolism
Phvul.008G168300.1	mediator of RNA polymerase II transcription subunit 1-like			
Phvul.008G168300.2	mediator of RNA polymerase II transcription subunit 1-like			
Phvul.008G169000.1	transcription factor TGA3-like isoform X1	transcription factor activity, sequence-specific DNA binding;sequence-specific DNA binding;transcription, DNA-templated;regulation of transcription, DNA-templated		
Phvul.008G172500.1	TPR repeat-containing thioredoxin TTL1	cell;cell redox homeostasis		
Phvul.008G173500.1	synaptonemal complex 1-like	synaptonemal complex;reciprocal meiotic recombination		
Phvul.008G173500.2	synaptonemal complex 1-like	synaptonemal complex;reciprocal meiotic recombination		
Phvul.008G173500.3	synaptonemal complex 1-like	synaptonemal complex;reciprocal meiotic recombination		
Phvul.008G173500.4	synaptonemal complex 1-like	synaptonemal complex;reciprocal meiotic recombination		
Phvul.008G173500.5	synaptonemal complex 1-like	synaptonemal complex;reciprocal meiotic recombination		
Phvul.008G174000.1	probable pectin methyltransferase QUA2	endosome;trans-Golgi network;integral component of membrane;S-adenosylmethionine-dependent methyltransferase activity;response to cytokinin;homogalacturonan biosynthetic process;methylation;root development;shoot system development		
Phvul.008G175300.1	7-deoxyloganetin glucosyltransferase-like	intracellular membrane-bounded organelle;quercetin 3-O-glucosyltransferase activity;quercetin 7-O-glucosyltransferase activity;flavonoid biosynthetic process;flavonoid glucuronidation	ec:2.4.1	
Phvul.008G175700.1	EXORDIUM-like			
Phvul.008G176300.1	serine threonine-kinase SAPK2-like	ribosome;structural constituent of ribosome;protein serine/threonine kinase activity;ATP binding;translation;protein phosphorylation;intracellular signal transduction	ec:2.7.11	

Phvul.008G176700.1	Thylakoid lumenal 29 kDa , chloroplastic	nucleus;chloroplast thylakoid membrane;thylakoid lumen;L-ascorbate peroxidase activity;heme binding;pentose-phosphate shunt;rRNA processing;response to oxidative stress;response to blue light;stomatal complex morphogenesis;response to red light;response to far red light;chlorophyll biosynthetic process;starch biosynthetic process;isopentenyl diphosphate biosynthetic process, methylerythritol 4-phosphate pathway;photosynthesis, light reaction;glucosinolate metabolic process;oxidation-reduction process;cellular oxidant detoxification	ec:1.11.1.11-peroxidase;ec:1.11.1.7-lactoperoxidase	Glutathione metabolism;Ascorbate and aldarate metabolismPhenylpropanoid biosynthesis
Phvul.008G176700.2	thylakoid lumenal 29 kDa , chloroplastic isoform X1	nucleus;chloroplast thylakoid membrane;thylakoid lumen;L-ascorbate peroxidase activity;heme binding;pentose-phosphate shunt;rRNA processing;response to oxidative stress;response to blue light;stomatal complex morphogenesis;response to red light;response to far red light;chlorophyll biosynthetic process;starch biosynthetic process;isopentenyl diphosphate biosynthetic process, methylerythritol 4-phosphate pathway;photosynthesis, light reaction;glucosinolate metabolic process;oxidation-reduction process;cellular oxidant detoxification	ec:1.11.1.11-peroxidase;ec:1.11.1.7-lactoperoxidase	Glutathione metabolism;Ascorbate and aldarate metabolismPhenylpropanoid biosynthesis
Phvul.008G176800.1	DUF246 domain-containing	cytoplasm;integral component of membrane		
Phvul.008G177300.1	phage capsid scaffolding (GPO) serine peptidase			
Phvul.008G178500.1	phosphoribosylaminoimidazole-succinocarboxamide synthase, chloroplastic	chloroplast stroma;phosphoribosylaminoimidazole carboxylase activity;phosphoribosylaminoimidazolesuccinocarboxamide synthase activity;ATP binding;'de novo' IMP biosynthetic process;toxin catabolic process;response to auxin;adenine biosynthetic process	ec:6.3.2.6-synthase;ec:4.1.1.21-carboxylase	Purine metabolism;Biosynthesis of antibioticsPurine metabolism
Phvul.008G180300.1	plant MNA5-17			
Phvul.008G181200.1	clathrin-adaptor medium chain AP-2	cytosol;clathrin adaptor complex;protein targeting to vacuole;vesicle-mediated transport		
Phvul.008G181800.1	kinesin FRA1	kinesin complex;microtubule;microtubule motor activity;ATP binding;microtubule binding;ATPase activity;microtubule-based movement;metabolic process	ec:3.6.1;ec:3.6.1.3-adenylpyrophosphatase;ec:3.6.1.15-phosphatase	Purine metabolismPurine metabolism;Thiamine metabolism
Phvul.008G183800.1	hypothetical protein PHAVU_008G183800g, partial			
Phvul.008G184500.2	glycine-rich domain-containing 2-like			
Phvul.008G185000.1	zinc finger (C3HC4-type RING finger) family	zinc ion binding		
Phvul.008G185400.1	integrase-type DNA-binding superfamily , partial	nucleus;DNA binding;transcription factor activity, sequence-specific DNA binding;transcription, DNA-templated;regulation of transcription, DNA-templated;tissue development;floral organ development		
Phvul.008G186500.1	DUF3133 family			
Phvul.008G186700.1	serine threonine-kinase 2 19-like	cytoplasm;protein serine/threonine kinase activity;ATP binding;protein phosphorylation;intracellular signal transduction	ec:2.7.11	
Phvul.008G186700.2	serine threonine-kinase 2 19-like	cytoplasm;protein serine/threonine kinase activity;ATP binding;protein phosphorylation;intracellular signal transduction	ec:2.7.11	

Phvul.008G187700.1	zinc finger with UFM1-specific peptidase domain	integral component of membrane		
Phvul.008G187700.2	zinc finger with UFM1-specific peptidase domain	membrane		
Phvul.008G187700.3	zinc finger with UFM1-specific peptidase domain	integral component of membrane		
Phvul.008G188400.1	DNA mismatch repair MUTS family	endonuclease activity;ATP binding;ATPase activity;mismatched DNA binding;mismatch repair;negative regulation of DNA recombination;nucleic acid phosphodiester bond hydrolysis	ec:3.6.1;ec:3.6.1.3-adenylpyrophosphatase;ec:3.1;ec:3.6.1.15-phosphatase	Purine metabolismPurine metabolism;Thiamine metabolism
Phvul.008G190700.1	probable galacturonosyltransferase 15	Golgi membrane;integral component of membrane;polygalacturonate 4-alpha-galacturonosyltransferase activity;pectin biosynthetic process;cell wall organization	ec:2.4.1.43-4-alpha-galacturonosyltransferase	Amino sugar and nucleotide sugar metabolism;Starch and sucrose metabolism
Phvul.008G190700.2	probable galacturonosyltransferase 15	Golgi membrane;integral component of membrane;polygalacturonate 4-alpha-galacturonosyltransferase activity;pectin biosynthetic process;cell wall organization	ec:2.4.1.43-4-alpha-galacturonosyltransferase	Amino sugar and nucleotide sugar metabolism;Starch and sucrose metabolism
Phvul.008G191200.1	neurofilament medium polypeptide-like			
Phvul.008G191200.2	neurofilament medium polypeptide-like			
Phvul.008G192100.1	pumilio homolog 2-like	RNA binding		
Phvul.008G193300.1	conserved oligomeric Golgi complex subunit 5-like	cytosol;Golgi transport complex;intra-Golgi vesicle-mediated transport		
Phvul.008G195100.1	TMV resistance N-like	ADP binding;defense response;signal transduction		
Phvul.008G195300.1	TMV resistance N	ADP binding;defense response;signal transduction		
Phvul.008G195300.2	TMV resistance N	ADP binding;defense response;signal transduction		
Phvul.008G195900.1	nonexpressor of pathogenesis-related 1			
Phvul.008G196100.1	Transmembrane 56-B	integral component of membrane		
Phvul.008G196300.1	ATP-dependent RNA helicase,	ATP-dependent RNA helicase activity;ATP binding;poly(A) RNA binding;RNA processing	ec:3.6.1;ec:3.6.1.3-adenylpyrophosphatase;ec:3.6.1.15-phosphatase;ec:2.7.7	Purine metabolismPurine metabolism;Thiamine metabolism
Phvul.008G197200.1	pentatricopeptide repeat-containing At5g16860	zinc ion binding		
Phvul.008G197300.1	hypothetical protein PHAVU_008G197300g			
Phvul.008G197400.1	pentatricopeptide repeat-containing At1g74580			
Phvul.008G197500.1	pentatricopeptide repeat-containing At5g64320, mitochondrial	integral component of membrane;thymidine kinase activity;deoxyribonucleoside monophosphate biosynthetic process	ec:2.7.1.21-kinase;ec:2.7.1.145	Drug metabolism - other enzymes;Pyrimidine metabolism
Phvul.008G197600.1	auxin response factor 6-like	nucleus;DNA binding;lipid binding;transcription, DNA-templated;regulation of transcription, DNA-templated;lipid transport;auxin-activated signaling pathway		
Phvul.008G199700.1	DEAD-box ATP-dependent RNA helicase 53-like	nucleic acid binding;ATP-dependent RNA helicase activity;ATP binding;RNA secondary structure unwinding	ec:3.6.1;ec:3.6.1.3-adenylpyrophosphatase;ec:3.6.1.15-phosphatase;ec:2.7.7	Purine metabolismPurine metabolism;Thiamine metabolism
Phvul.008G199800.1	CHROMATIN REMODELING 5	nucleus;DNA binding;ATP binding;ATP-dependent helicase activity;metabolic process	ec:3.6.1;ec:3.6.1.3-adenylpyrophosphata	Purine metabolismPurine

			se;ec:3.6.1.15-phosphatase	metabolism;Thiamine metabolism
Phvul.008G200100.1	hypothetical protein PHAVU_008G200100g			
Phvul.008G200100.2	hypothetical protein PHAVU_008G200100g			
Phvul.008G200200.1	GTP-binding At3g49725, chloroplastic	cytoplasm;GTP binding;ribosome binding		
Phvul.008G200300.1	E3 ubiquitin- ligase HERC1	ligase activity;metal ion binding;metabolic process		
Phvul.008G200400.1	homeobox leucine zipper	nucleus;DNA binding		
Phvul.008G200800.1	calcium-dependent lipid-binding (domain) family			
Phvul.008G200800.2	calcium-dependent lipid-binding (domain) family			
Phvul.008G203700.1	Nuclear control of ATPase 2	mitochondrial outer membrane;integral component of membrane;protein complex;cell adhesion;organ morphogenesis;trichome morphogenesis;vegetative to reproductive phase transition of meristem;protein desumoylation;galactolipid biosynthetic process;regulation of chromosome organization;actin nucleation;root hair cell differentiation;hydrogen peroxide biosynthetic process;cell wall organization		
Phvul.008G205100.1	urease accessory F isoform X2	nickel cation binding;heat shock protein binding;unfolded protein binding;phosphatidylinositol biosynthetic process;nitrogen compound metabolic process;positive regulation of metalloenzyme activity		
Phvul.008G205100.2	urease accessory F isoform X2	nickel cation binding;heat shock protein binding;unfolded protein binding;phosphatidylinositol biosynthetic process;nitrogen compound metabolic process;positive regulation of metalloenzyme activity		
Phvul.008G205700.2	CBL-interacting serine threonine-kinase 3	cytoplasm;integral component of membrane;protein serine/threonine kinase activity;ATP binding;protein phosphorylation;intracellular signal transduction	ec:2.7.11	
Phvul.008G206300.1	la-related 1A			
Phvul.008G206600.1	argonaute 4-like	translation initiation factor activity;translational initiation		
Phvul.008G206600.2	argonaute 4-like	translation initiation factor activity;translational initiation		
Phvul.008G210200.1	serine threonine-kinase TOUSLED-like isoform X1	chromosome;chloroplast;protein kinase activity;ATP binding;identical protein binding;protein phosphorylation;chromosome organization		
Phvul.008G210200.2	serine threonine-kinase TOUSLED-like isoform X2	chloroplast;protein kinase activity;ATP binding;identical protein binding;protein phosphorylation		
Phvul.008G210700.1	CSC1 At3g21620	integral component of membrane;4-alpha-glucanotransferase activity;glycogen metabolic process	ec:2.4.1.25-disproportionating enzyme	Starch and sucrose metabolism
Phvul.008G211500.1	lycopene beta epsilon cyclase	chloroplast;oxidoreductase activity;oxidation-reduction process		
Phvul.008G211600.1	plastid transcriptionally active			
Phvul.008G212400.1	pentatricopeptide repeat-containing At3g02650, mitochondrial			

Phvul.008G212800.1	nucleobase-ascorbate transporter 1	plasmodesma;integral component of membrane;transporter activity;transmembrane transport		
Phvul.008G212800.2	Nucleobase-ascorbate transporter 1	integral component of membrane;transporter activity;transmembrane transport		
Phvul.008G213300.1	subtilisin-like protease Glyma18g48580	cell wall;serine-type endopeptidase activity;proteolysis	ec:3.4.21	
Phvul.008G213300.2	subtilisin-like protease Glyma18g48580	cell wall;serine-type endopeptidase activity;proteolysis	ec:3.4.21	
Phvul.008G214800.1	E3 ubiquitin- ligase BRE1-like 2 isoform X1	zinc ion binding;ligase activity;protein homodimerization activity;regulation of mitotic cell cycle;leaf morphogenesis;seed dormancy process;vegetative to reproductive phase transition of meristem;histone monoubiquitination;histone H2B ubiquitination;post-translational protein modification;positive regulation of transcription, DNA-templated		
Phvul.008G215200.1	centromere kinetochore zw10 homolog	nucleus;cytoplasm;RZZ complex;ER to Golgi vesicle-mediated transport;chromosome segregation;mitotic nuclear division;mitotic spindle assembly checkpoint		
Phvul.008G215200.2	centromere kinetochore zw10 homolog	nucleus;cytoplasm;RZZ complex;ER to Golgi vesicle-mediated transport;chromosome segregation;mitotic nuclear division;mitotic spindle assembly checkpoint		
Phvul.008G215200.3	centromere kinetochore zw10 homolog	nucleus;cytoplasm;RZZ complex;ER to Golgi vesicle-mediated transport;chromosome segregation;mitotic nuclear division;mitotic spindle assembly checkpoint		
Phvul.008G215200.4	centromere kinetochore zw10 homolog	nucleus;cytoplasm;RZZ complex;ER to Golgi vesicle-mediated transport;chromosome segregation;mitotic nuclear division;mitotic spindle assembly checkpoint		
Phvul.008G217900.1	SIEVE ELEMENT OCCLUSION C			
Phvul.008G219800.1	Neuronal PAS domain-containing 4			
Phvul.008G220100.1	receptor-like kinase	integral component of membrane;protein kinase activity;ATP binding;protein phosphorylation		
Phvul.008G221400.1	peptide-N4-(N-acetyl-beta-glucosaminyl)asparagine amidase A	vacuole		
Phvul.008G221900.1	probable inactive receptor kinase At3g56050	integral component of membrane;protein kinase activity;ATP binding;protein phosphorylation		
Phvul.008G222800.1	clathrin heavy chain 2	clathrin coat of trans-Golgi network vesicle;clathrin coat of coated pit;structural molecule activity;intracellular protein transport;vesicle-mediated transport		
Phvul.008G223400.1	zinc finger CCCH domain-containing 29	metal ion binding		
Phvul.008G224800.1	ATP-binding ,	ATP binding;vernalization response;anthocyanin accumulation in tissues in response to UV light;carpel development		
Phvul.008G228400.2	Rop guanine nucleotide exchange factor 1	Rho guanyl-nucleotide exchange factor activity;positive regulation of GTPase activity		
Phvul.008G228700.1	cucumisin-like	cell wall;integral component of membrane;serine-type endopeptidase activity;proteolysis	ec:3.4.21	
Phvul.008G228700.2	cucumisin-like	cell wall;integral component of membrane;serine-type endopeptidase activity;proteolysis	ec:3.4.21	
Phvul.008G230700.1	Regulator of chromosome condensation	kinase activity;ligase activity;phosphorylation		

	repeat-containing isoform 1			
Phvul.008G232400.1	AP3-complex subunit beta-A	AP-3 adaptor complex;intracellular protein transport;Golgi to vacuole transport;regulation of intracellular pH;lytic vacuole organization		
Phvul.008G233500.1	AAR2 homolog	spliceosomal tri-snRNP complex assembly;mitotic cell cycle;protein folding;response to heat;response to high light intensity;response to hydrogen peroxide		
Phvul.008G233900.1	plastid movement impaired			
Phvul.008G234100.1	transmembrane ,	integral component of membrane		
Phvul.008G234600.1	serine threonine-kinase CDL1-like	non-membrane spanning protein tyrosine kinase activity;ATP binding;peptidyl-tyrosine phosphorylation	ec:2.7.10;ec:2.7.10.2-protein-tyrosine kinase	T cell receptor signaling pathway
Phvul.008G234800.1	probable fructokinase-4	chloroplast stroma;ribokinase activity;fructokinase activity;D-ribose metabolic process;cysteine biosynthetic process;carbohydrate phosphorylation	ec:2.7.1.15-deoxyribokinase;ec:2.7.1.4-fructokinase (phosphorylating);ec:2.7.1.1-hexokinase type IV glucokinase	Pentose phosphate pathwayAmino sugar and nucleotide sugar metabolism;Starch and sucrose metabolism;Fructose and mannose metabolismButirosin and neomycin biosynthesis;Biosynthesis of antibiotics;Streptomycin biosynthesis;Amino sugar and nucleotide sugar metabolism;Glycolysis / Gluconeogenesis;Starch and sucrose metabolism;Galactose metabolism;Fructose and mannose metabolism
Phvul.008G235900.1	nucleolar complex 4 homolog	nucleolus;Noc4p-Nop14p complex;small-subunit processome;rRNA processing;single-organism process		
Phvul.008G238100.1	potassium channel SKOR	integral component of membrane;voltage-gated potassium channel activity;regulation of membrane potential;potassium ion transmembrane transport		
Phvul.008G241300.1	sucrose synthase	sucrose synthase activity;response to hypoxia;sucrose metabolic process;galactolipid biosynthetic process;callose deposition in phloem sieve plate	ec:2.4.1.13-synthase;ec:2.4.1	Starch and sucrose metabolism
Phvul.008G245200.1	probable disease resistance At4g27220	integral component of membrane;ATP binding;ADP binding;defense response		
Phvul.008G245600.1	probable LRR receptor-like serine threonine- kinase At1g06840	integral component of membrane;protein serine/threonine kinase activity;ATP binding;protein phosphorylation	ec:2.7.11	
Phvul.008G245600.2	probable LRR receptor-like serine threonine- kinase At1g06840	integral component of membrane;protein serine/threonine kinase activity;ATP binding;protein phosphorylation	ec:2.7.11	
Phvul.008G245900.1	probable inactive receptor kinase At5g58300	integral component of membrane;protein kinase activity;ATP binding;protein phosphorylation		
Phvul.008G245900.2	probable inactive receptor kinase At5g58300	integral component of membrane;protein kinase activity;ATP binding;protein phosphorylation		
Phvul.008G247700.1	extra-large guanine nucleotide-binding 3-like	zinc ion binding		
Phvul.008G248900.1	CBL-interacting serine threonine-kinase 10	integral component of membrane;protein serine/threonine kinase activity;ATP binding;protein phosphorylation;signal transduction	ec:2.7.11	

Phvul.008G251900.1	glycolipid transfer (GLTP) family	cytoplasm;integral component of membrane;glycolipid transporter activity;glycolipid binding;glycolipid transport		
Phvul.008G252300.1	exocyst complex component EXO70B1-like	exocyst;integral component of membrane;exocytosis		
Phvul.008G252400.1	exocyst complex component EXO70B1-like	exocyst;integral component of membrane;exocytosis		
Phvul.008G252700.1	hypothetical protein PHAVU_008G252700g	integral component of membrane		
Phvul.008G253000.1	probable phosphatase 2C 60	protein serine/threonine phosphatase activity;metal ion binding;protein dephosphorylation	ec:3.1;ec:3.1.3.41-nitrophenyl phosphatase;ec:3.1.3.16-phosphatase	Aminobenzoate degradationT cell receptor signaling pathway
Phvul.008G255500.1	exocyst subunit EXO70 family	exocyst;exocytosis		
Phvul.008G256100.1	hypothetical protein PHAVU_008G256100g	integral component of membrane		
Phvul.008G256800.1	UDP-glycosyltransferase 87A1	intracellular membrane-bounded organelle;indole-3-acetate beta-glucosyltransferase activity;limonoid glucosyltransferase activity;quercetin 3-O-glucosyltransferase activity;quercetin 7-O-glucosyltransferase activity;flavonoid biosynthetic process;flavonoid glucuronidation	ec:2.4.1;ec:2.4.1.210;ec:2.4.1.121	
Phvul.008G256800.2	UDP-glycosyltransferase 87A1	intracellular membrane-bounded organelle;indole-3-acetate beta-glucosyltransferase activity;limonoid glucosyltransferase activity;quercetin 3-O-glucosyltransferase activity;quercetin 7-O-glucosyltransferase activity;flavonoid biosynthetic process;flavonoid glucuronidation	ec:2.4.1;ec:2.4.1.210;ec:2.4.1.121	
Phvul.008G257100.2	exocyst complex component EXO70B1-like	exocyst;integral component of membrane;exocytosis		
Phvul.008G257400.1	LOB domain-containing 12-like	carbamoyl-phosphate synthase (glutamine-hydrolyzing) activity;metabolic process	ec:6.3.5.5-synthase (glutamine-hydrolysing)	Alanine, aspartate and glutamate metabolism;Pyrimidine metabolism
Phvul.008G258300.1	Nop53 (60S ribosomal biogenesis)	intracellular;mRNA export from nucleus;protein import into nucleus		
Phvul.008G258400.1	Serine Threonine-kinase DDB-G0267514 ,	intracellular;MAP kinase kinase kinase activity;non-membrane spanning protein tyrosine kinase activity;ATP binding;MAPK cascade;activation of MAPKK activity;peptidyl-tyrosine phosphorylation	ec:2.7.11;ec:2.7.10;ec:2.7.11.25;ec:2.7.10.2-protein-tyrosine kinase	T cell receptor signaling pathway
Phvul.008G258500.1	ubiquitin-activating enzyme E1 1	nucleus;cytosol;integral component of membrane;ubiquitin activating enzyme activity;ubiquitin-protein transferase activity;ATP binding;protein ubiquitination;modification-dependent protein catabolic process		
Phvul.008G258500.2	ubiquitin-activating enzyme E1 1	nucleus;cytosol;integral component of membrane;ubiquitin activating enzyme activity;ubiquitin-protein transferase activity;ATP binding;protein ubiquitination;modification-dependent protein catabolic process		
Phvul.008G258500.3	ubiquitin-activating enzyme E1 1	nucleus;cytosol;integral component of membrane;ubiquitin activating enzyme activity;ubiquitin-protein transferase activity;ATP binding;protein ubiquitination;modification-dependent protein catabolic process		
Phvul.008G261000.1	double Clp-N motif P-loop nucleoside triphosphate hydrolase superfamily	ATP binding;protein metabolic process		

Phvul.008G261400.1	inositol 3-kinase-like	ATP binding;inositol 3-kinase activity;myo-inositol hexakisphosphate biosynthetic process;phosphorylation	ec:2.7.1.64-3-kinase	Inositol phosphate metabolism
Phvul.008G261900.1	zinc finger CCCH domain-containing 25	U2 snRNP;RES complex;precatalytic spliceosome;catalytic step 2 spliceosome;nucleotide binding;first spliceosomal transesterification activity;RNA binding;metal ion binding;mRNA splicing, via spliceosome;mRNA export from nucleus		
Phvul.008G262200.1	probable NAD(P)H dehydrogenase (quinone) FQR1-like 3	plasma membrane;FMN binding;oxidoreductase activity;negative regulation of transcription, DNA-templated;oxidation-reduction process		
Phvul.008G264400.1	LRR receptor-like kinase	plasma membrane;integral component of membrane;protein serine/threonine kinase activity;transmembrane receptor protein tyrosine kinase activity;ATP binding;2-alkenal reductase [NAD(P)] activity;peptidyl-tyrosine phosphorylation;oxidation-reduction process	ec:2.7.11;ec:2.7.10;ec:1.3.1.74;ec:2.7.10.1	
Phvul.008G265300.1	septum-promoting GTP-binding 1	intracellular;GTP binding;small GTPase mediated signal transduction;protein transport;vesicle-mediated transport		
Phvul.008G265500.1	hypothetical protein PHAVU_008G265500g			
Phvul.008G265600.1	hypothetical protein PHAVU_008G265600g			
Phvul.008G265600.4	hypothetical protein PHAVU_008G265600g			
Phvul.008G266800.3	reactive Intermediate Deaminase A, chloroplastic-like			
Phvul.008G266900.1	ubiquitin thioesterase OTU1	cytosol;peptidase activity;proteolysis		
Phvul.008G266900.2	ubiquitin thioesterase OTU1	cytosol;peptidase activity;proteolysis		
Phvul.008G267500.1	DEAD-box ATP-dependent RNA helicase 32	RNA binding;ATP-dependent RNA helicase activity;ATP binding;RNA secondary structure unwinding	ec:3.6.1;ec:3.6.1.3-adenylpyrophosphatase;ec:3.6.1.15-phosphatase;ec:2.7.7	Purine metabolismPurine metabolism;Thiamine metabolism
Phvul.008G268800.1	GDSL-like lipase acylhydrolase	integral component of membrane;hydrolase activity, acting on ester bonds;9,9'-dicis-carotene:quinone oxidoreductase activity;7,9,9'-tricis-neurosporene:quinone oxidoreductase activity;9,9'-di-cis-zeta-carotene desaturation to 7,9,7',9'-tetra-cis-lycopene;oxidation-reduction process	ec:1.3.5.6-desaturase	Carotenoid biosynthesis
Phvul.008G269100.1	Rab3 GTPase-activating catalytic subunit	cytoplasm;GTPase activator activity;positive regulation of GTPase activity		
Phvul.008G270100.1	U4 tri-snRNP-associated 2-like	zinc ion binding;thiol-dependent ubiquitinyl hydrolase activity;protein deubiquitination	ec:3.4;ec:3.4.19.12	
Phvul.008G270100.2	U4 tri-snRNP-associated 2-like	zinc ion binding;thiol-dependent ubiquitinyl hydrolase activity;protein deubiquitination	ec:3.4;ec:3.4.19.12	
Phvul.008G270200.1	esophageal cancer associated ,	intracellular;retromer complex;protein transporter activity;protein transport;retrograde transport, endosome to Golgi		
Phvul.008G270200.2	esophageal cancer associated ,	intracellular;retromer complex;protein transporter activity;protein transport;retrograde transport, endosome to Golgi		
Phvul.008G270600.2	aspartate carbamoyltransferase 1, chloroplastic	cytosol;chloroplast stroma;aspartate carbamoyltransferase activity;amino acid binding;urea cycle;purine nucleotide biosynthetic process;'de novo' pyrimidine nucleobase biosynthetic process;cellular amino acid metabolic process;'de novo' UMP biosynthetic process	ec:2.1.3.2-carbamoyltransferase	Alanine, aspartate and glutamate metabolism;Pyrimidine metabolism

Phvul.008G273400.1	ENHANCED DOWNY MILDEW 2-like	methyltransferase activity;zinc ion binding;methylation		
Phvul.008G273400.2	ENHANCED DOWNY MILDEW 2-like	zinc ion binding;transferase activity		
Phvul.008G273400.3	ENHANCED DOWNY MILDEW 2-like	methyltransferase activity;zinc ion binding;methylation		
Phvul.008G273400.4	ENHANCED DOWNY MILDEW 2-like	zinc ion binding;transferase activity		
Phvul.008G276100.1	receptor 12	integral component of membrane		
Phvul.008G276800.1	intracellular transport USO1-like			
Phvul.008G277500.1	cytochrome P450 family 71	integral component of membrane;iron ion binding;oxidoreductase activity, acting on paired donors, with incorporation or reduction of molecular oxygen, NAD(P)H as one donor, and incorporation of one atom of oxygen;heme binding;secondary metabolite biosynthetic process;oxidation-reduction process	ec:1.14.13	
Phvul.008G279600.1	Cellulose synthase E1	integral component of membrane;cellulose synthase (UDP-forming) activity;cellulose biosynthetic process;cell wall organization	ec:2.4.1.12-synthase (UDP-forming);ec:2.4.1	Starch and sucrose metabolism
Phvul.008G279800.1	cellulose synthase E6	integral component of membrane;cellulose synthase (UDP-forming) activity;cellulose biosynthetic process;cell wall organization	ec:2.4.1.12-synthase (UDP-forming);ec:2.4.1	Starch and sucrose metabolism
Phvul.008G280000.1	sucrase ferredoxin family	integral component of membrane		
Phvul.008G280100.1	SEC14 cytosolic factor			
Phvul.008G280600.1	DUF1296 family			
Phvul.008G281500.2	probable LRR receptor-like serine threonine- kinase At4g26540	integral component of membrane;protein serine/threonine kinase activity;ATP binding;protein phosphorylation	ec:2.7.11	
Phvul.008G282100.1	late embryogenesis abundant	integral component of membrane		
Phvul.008G282600.1	probable galactinol--sucrose galactosyltransferase 1	galactinol-raffinose galactosyltransferase activity;metabolic process	ec:2.4.1.67-galactosyltransferase;ec:2.4.1	Galactose metabolism
Phvul.008G282800.1	probable amino acid permease 7	plasma membrane;integral component of membrane;amino acid transmembrane transporter activity;amino acid transmembrane transport		
Phvul.008G282800.2	probable amino acid permease 7	plasma membrane;integral component of membrane;amino acid transmembrane transporter activity;amino acid transmembrane transport		
Phvul.008G283000.1	probable amino acid permease 7	plasma membrane;integral component of membrane;amino acid transmembrane transporter activity;amino acid transmembrane transport		
Phvul.008G283200.1	histone deacetylase 9	nucleus;NAD-dependent histone deacetylase activity (H3-K14 specific);transcription, DNA-templated;regulation of transcription, DNA-templated;histone H3 deacetylation	ec:3.5.1.98	
Phvul.008G283500.1	armadillo repeat-containing 8-like			
Phvul.008G284500.1	disease resistance At4g27190-like isoform X6	ADP binding;defense response		
Phvul.008G284900.1	nuclear pore complex NUP133	chloroplast;nuclear pore outer ring;nucleocytoplasmic transporter activity;structural constituent of nuclear pore;posttranscriptional tethering of RNA polymerase II gene DNA at nuclear periphery;regulation of transcription, DNA-templated;RNA export from nucleus;protein import into nucleus;embryo sac egg cell differentiation		
Phvul.008G286000.1	scarecrow-like transcription factor PAT1	transcription, DNA-templated;regulation of transcription, DNA-templated		

Phvul.008G286300.1	probable serine threonine- kinase WNK11	protein kinase activity;ATP binding;protein phosphorylation;organ senescence		
Phvul.008G287900.1	TBC1 domain family member 2A	hydrolase activity;metabolic process;actin filament-based movement;Golgi localization;mitochondrion localization;peroxisome localization		
Phvul.008G289500.1	phenylalanine ammonia-lyase	cytoplasm;phenylalanine ammonia-lyase activity;L-phenylalanine catabolic process;cinnamic acid biosynthetic process	ec:4.3.1.25-ammonia-lyase;ec:4.3.1.24-ammonia-lyase	Phenylalanine metabolism;Phenylpropanoid biosynthesisPhenylalanine metabolism;Phenylpropanoid biosynthesis
Phvul.008G292300.1	homoserine kinase	homoserine kinase activity;ATP binding;threonine metabolic process;phosphorylation	ec:2.7.1.39-kinase	Glycine, serine and threonine metabolism
Phvul.009G003200.1	endoglucanase 24-like	cellulase activity;cellulose catabolic process	ec:3.2.1.4-endo-1,4-beta-D-glucanase	Starch and sucrose metabolism
Phvul.009G004700.1	probable phosphoribosylformylglycinamidine synthase, chloroplastic mitochondrial	cytoplasm;phosphoribosylformylglycinamidine synthase activity;ATP binding;transferase activity;'de novo' IMP biosynthetic process;glutamine metabolic process	ec:6.3.5.3-synthase	Purine metabolism;Biosynthesis of antibiotics
Phvul.009G006300.1	canopy-1	integral component of membrane;systemic acquired resistance;response to endoplasmic reticulum stress		
Phvul.009G008200.1	xylem cysteine ase 2-like	extracellular space;cell wall;lysosome;cysteine-type endopeptidase activity;regulation of meristem growth;developmental programmed cell death;proteolysis involved in cellular protein catabolic process	ec:3.4;ec:3.4.22	
Phvul.009G009900.1	Prostaglandin E synthase 2	mitochondrion;electron carrier activity;protein disulfide oxidoreductase activity;prostaglandin-E synthase activity;protein targeting to mitochondrion;toxin catabolic process;response to cyclopentenone;cell redox homeostasis;oxidation-reduction process	ec:5.3.99.3-synthase	Arachidonic acid metabolism
Phvul.009G010900.1	hypothetical protein PHAVU_009G010900g			
Phvul.009G011200.1	NLP4-like isoform X1			
Phvul.009G012200.1	WUSCHEL-related homeobox 8-like	nucleus;DNA binding		
Phvul.009G012300.1	actin cytoskeleton-regulatory complex pan1	plasma membrane;calcium ion binding;actin filament-based movement;Golgi localization;mitochondrion localization;peroxisome localization		
Phvul.009G012400.1	U-box domain-containing 44	ubiquitin-protein transferase activity;ligase activity;protein ubiquitination		
Phvul.009G012500.1	F-box kelch-repeat At5g42350-like			
Phvul.009G012500.2	F-box kelch-repeat At5g42350-like			
Phvul.009G012500.3	F-box kelch-repeat At5g42350-like			
Phvul.009G015200.1	IQ-DOMAIN 32	intracellular part		
Phvul.009G015200.2	IQ-DOMAIN 32	intracellular part		
Phvul.009G015200.3	IQ-DOMAIN 32	intracellular part		
Phvul.009G015700.1	filament-like plant 4			
Phvul.009G015700.2	filament-like plant 4			
Phvul.009G017500.1	DUF668 family			
Phvul.009G018600.1	subtilisin-like serine protease	cell wall;serine-type endopeptidase activity;proteolysis	ec:3.4.21	

Phvul.009G019500.1	RNA pseudouridine synthase 2, chloroplastic isoform X1	cytosol;RNA binding;pseudouridine synthase activity;deaminase activity;enzyme-directed rRNA pseudouridine synthesis	ec:5.4.99.12	
Phvul.009G019500.2	RNA pseudouridine synthase 2, chloroplastic isoform X1	cytosol;RNA binding;pseudouridine synthase activity;deaminase activity;enzyme-directed rRNA pseudouridine synthesis	ec:5.4.99.12	
Phvul.009G021300.1	lysine histidine transporter-like 8	integral component of membrane;amino acid transmembrane transporter activity;amino acid transmembrane transport		
Phvul.009G021600.1	DUF630 family			
Phvul.009G021700.1	DUF241 domain			
Phvul.009G021800.1	transmembrane ,	integral component of membrane		
Phvul.009G022100.1	dihydrofolate reductase-thymidylate synthase	dihydrofolate reductase activity;thymidylate synthase activity;dTMP biosynthetic process;glycine biosynthetic process;one-carbon metabolic process;methylation;tetrahydrofolate biosynthetic process;oxidation-reduction process	ec:1.5.1.3-reductase;ec:2.1.1.45-synthase	One carbon pool by folate;Folate biosynthesisOne carbon pool by folate;Pyrimidine metabolism
Phvul.009G023500.1	transcription factor bHLH63	membrane;protein dimerization activity		
Phvul.009G025800.1	hypothetical protein PHAVU_009G025800g	zinc ion binding		
Phvul.009G026300.1	grpE homolog, mitochondrial	mitochondrial matrix;chloroplast stroma;adenyl-nucleotide exchange factor activity;copper ion binding;protein homodimerization activity;chaperone binding;protein folding;embryo development ending in seed dormancy;thylakoid membrane organization;vegetative to reproductive phase transition of meristem;iron-sulfur cluster assembly;plant ovule development;regulation of catalytic activity		
Phvul.009G026700.1	phosphopantothenate--cysteine ligase 2-like	phosphopantothenate--cysteine ligase activity;metabolic process	ec:6.3.2.5-ligase	Pantothenate and CoA biosynthesis
Phvul.009G027100.1	alkylated DNA repair alkB homolog 8	tRNA methyltransferase activity;tRNA wobble uridine modification;tRNA methylation	ec:2.1.1	
Phvul.009G027200.1	early endosome antigen 1-like isoform X1	vacuole;regulation of meristem growth		
Phvul.009G027300.1	F-box kelch-repeat At1g22040-like			
Phvul.009G028600.1	probable tRNA modification GTPase	intracellular;GTPase activity;GTP binding;tRNA modification;endocytosis	ec:3.6.1;ec:3.6.1.15-phosphatase	Purine metabolism;Thiamine metabolism
Phvul.009G030600.1	sugar transport 5-like	integral component of plasma membrane;sugar:proton symporter activity;glucose transmembrane transporter activity;proton transport;glucose import;glucose transmembrane transport		
Phvul.009G031600.1	methyl- -binding domain	nucleus;DNA binding;hydrolase activity, acting on ester bonds;metabolic process		
Phvul.009G031800.1	ROOT PRIMORDIUM DEFECTIVE 1-like	response to oxidative stress;proline transport		
Phvul.009G034100.1	TRANSPARENT TESTA 12-like	integral component of membrane;drug transmembrane transporter activity;antiporter activity;drug transmembrane transport		
Phvul.009G034200.1	MATE efflux family	integral component of membrane;drug transmembrane transporter activity;antiporter activity;drug transmembrane transport		
Phvul.009G034200.2	MATE efflux family	integral component of membrane;drug transmembrane transporter activity;antiporter activity;drug transmembrane transport		

Phvul.009G034200.3	MATE efflux family	integral component of membrane;drug transmembrane transporter activity;antiporter activity;drug transmembrane transport		
Phvul.009G034200.4	MATE efflux family	integral component of membrane;drug transmembrane transporter activity;antiporter activity;drug transmembrane transport		
Phvul.009G034900.1	E3 ubiquitin- ligase UPL1-like	ubiquitin-protein transferase activity;ligase activity;protein ubiquitination involved in ubiquitin-dependent protein catabolic process		
Phvul.009G035000.1	crt homolog 1	chloroplast;integral component of membrane;defense response to oomycetes;glutathione transport;response to cadmium ion		
Phvul.009G035500.1	BEACH domain-containing lvsC isoform X1			
Phvul.009G035500.2	BEACH domain-containing lvsC isoform X1			
Phvul.009G035500.3	BEACH domain-containing lvsC isoform X1			
Phvul.009G035500.4	BEACH domain-containing lvsC isoform X1			
Phvul.009G035500.5	BEACH domain-containing lvsC isoform X1			
Phvul.009G035500.6	BEACH domain-containing lvsC isoform X1			
Phvul.009G035900.1	TPR domain	integral component of membrane		
Phvul.009G036300.1	myb-like DNA-binding domain	nuclear telomere cap complex;double-stranded telomeric DNA binding		
Phvul.009G040100.1	thaumatin 1	integral component of membrane		
Phvul.009G041800.1	hypothetical protein PHAVU_009G041800g			
Phvul.009G041800.2	hypothetical protein PHAVU_009G041800g			
Phvul.009G042600.1	DUF3133 family			
Phvul.009G046200.1	polyadenylate-binding -interacting 12-like	nucleotide binding;nucleic acid binding		
Phvul.009G047400.1	WD repeat-containing 44			
Phvul.009G049600.1	homeobox-leucine zipper ATHB-12-like	nucleus;transcription factor activity, sequence-specific DNA binding;sequence-specific DNA binding;transcription, DNA-templated;regulation of transcription, DNA-templated		
Phvul.009G051000.1	KH domain-containing At4g18375	RNA binding		
Phvul.009G053500.1	probable plastid-lipid-associated 14, chloroplastic	protein kinase activity;ATP binding;protein phosphorylation		
Phvul.009G054100.1	polynucleotide 5 -hydroxyl-kinase NOL9-like	nucleus;ATP binding;polynucleotide 5'-hydroxyl-kinase activity;cleavage in ITS2 between 5.8S rRNA and LSU-rRNA of tricistronic rRNA transcript (SSU-rRNA, 5.8S rRNA, LSU-rRNA);phosphorylation	ec:2.7.1	
Phvul.009G054200.1	ATP-dependent zinc metalloprotease FTSH 11, chloroplastic mitochondrial-like	mitochondrion;chloroplast envelope;integral component of membrane;nucleic acid binding;ATP-dependent peptidase activity;metalloendopeptidase activity;ATP binding;proteolysis;response to heat;PSII associated light-harvesting complex II catabolic process;glucosinolate biosynthetic	ec:3.6.1;ec:3.6.1.3-adenylpyrophosphatase;ec:3.6.1.15-phosphatase;ec:3.4.24	Purine metabolismPurine metabolism;Thiamine metabolism

		process;regulation of protein localization;cell division		
Phvul.009G054700.1	cyclic nucleotide-gated ion channel 1-like	integral component of membrane;intracellular cyclic nucleotide activated cation channel activity;voltage-gated potassium channel activity;calcium ion transport;regulation of membrane potential;potassium ion transmembrane transport		
Phvul.009G054900.1	F-box kelch-repeat At3g06240-like			
Phvul.009G055000.1	3-oxoacyl-[acyl-carrier] synthase			
Phvul.009G056900.1	DUF4091 domain	transferase activity, transferring glycosyl groups;metabolic process;negative regulation of transcription, DNA-templated		
Phvul.009G057400.1	GYF domain-containing mpd2			
Phvul.009G060400.1	BEL1-like homeodomain 7	nucleus;DNA binding;regulation of transcription, DNA-templated		
Phvul.009G060400.2	BEL1-like homeodomain 7	nucleus;DNA binding;regulation of transcription, DNA-templated		
Phvul.009G060900.1	BZIP transcription factor bZIP124	intracellular;transcription factor activity, sequence-specific DNA binding;MAP kinase activity;sequence-specific DNA binding;MAPK cascade;regulation of transcription, DNA-templated	ec:2.7.11;ec:2.7.11.24-protein kinase	mTOR signaling pathway
Phvul.009G061300.1	Sec14 cytosolic factor	intracellular;integral component of membrane;peptide-aspartate beta-dioxygenase activity;transporter activity;transport;oxidation-reduction process	ec:1.14.11.16;ec:1.14.11	
Phvul.009G061900.1	Cytochrome b6-f complex subunit 7	cytochrome b6f complex;integral component of membrane		
Phvul.009G062900.2	transcription factor			
Phvul.009G062900.3	transcription factor			
Phvul.009G064300.1	BRI1 kinase inhibitor 1	kinase activity;phosphorylation		
Phvul.009G067000.1	SPla RYanodine receptor (SPRY) domain	integral component of membrane		
Phvul.009G067400.1	basic helix-loop-helix			
Phvul.009G068400.1	Chromatin modification-related EAF3	histone acetyltransferase complex;chromatin remodeling;regulation of transcription, DNA-templated;histone acetylation		
Phvul.009G069700.1	metal transporter Nramp2-like	integral component of membrane;transporter activity;transport		
Phvul.009G071400.1	carboxy-terminal domain cyclin	nucleus		
Phvul.009G071400.2	carboxy-terminal domain cyclin	nucleus		
Phvul.009G072300.1	calcium-transporting ATPase 3, endoplasmic reticulum-type	trans-Golgi network;integral component of plasma membrane;calcium-transporting ATPase activity;ATP binding;manganese-transporting ATPase activity;metal ion binding;cellular calcium ion homeostasis;intra-Golgi vesicle-mediated transport;metabolic process;root development;manganese ion homeostasis;calcium ion transmembrane transport;manganese ion transmembrane transport	ec:3.6.1;ec:3.6.1.3-adenylpyrophosphatase;ec:3.6.1.15-phosphatase;ec:3.6.3.8;ec:3.6.3.35	Purine metabolismPurine metabolism;Thiamine metabolism
Phvul.009G074200.1	RNA-binding 1-like	nucleotide binding;single-stranded RNA binding;cell proliferation		
Phvul.009G074600.1	heat shock -binding	integral component of membrane		
Phvul.009G074700.1	transmembrane ,	integral component of membrane		
Phvul.009G075500.1	zinc finger CCCH domain-containing 23-like	metal ion binding		
Phvul.009G076300.1	probable phosphatase 2C 72	protein serine/threonine phosphatase activity;protein dephosphorylation	ec:3.1;ec:3.1.3.41-nitrophenyl phosphatase;ec:3.1.3.16-phosphatase	Aminobenzoate degradationT cell receptor signaling pathway

Phvul.009G076600.1	RNA-binding KH domain	RNA binding		
Phvul.009G077800.1	alkaline phytoceramidase (APHC)	integral component of membrane;hydrolase activity, acting on carbon-nitrogen (but not peptide) bonds, in linear amides;ceramide metabolic process		
Phvul.009G078600.1	graves disease carrier	mitochondrial inner membrane;integral component of membrane;structural constituent of ribosome;coenzyme A transmembrane transporter activity;translation;coenzyme A transmembrane transport		
Phvul.009G081100.1	BIG GRAIN 1-like A			
Phvul.009G082900.1	guanine nucleotide-binding alpha-1 subunit isoform X1	GTPase activity;signal transducer activity;GTPase inhibitor activity;GTP binding;channel regulator activity;G-protein beta/gamma-subunit complex binding;metal ion binding;G-protein coupled receptor signaling pathway;metabolic process;negative regulation of GTPase activity	ec:3.6.1;ec:3.6.1.15-phosphatase	Purine metabolism;Thiamine metabolism
Phvul.009G083800.1	endoribonuclease Dicer homolog 3a isoform X1	nucleus;cytoplasm;RISC complex;DNA binding;RNA binding;ribonuclease III activity;ATP binding;production of siRNA involved in RNA interference;RNA phosphodiester bond hydrolysis, endonucleolytic	ec:3.1.30;ec:3.1.26;ec:3.1;ec:3.1.26.3	
Phvul.009G084100.1	eukaryotic translation initiation factor 3 subunit B-like	eukaryotic translation initiation factor 3 complex;eukaryotic 43S preinitiation complex;eukaryotic 48S preinitiation complex;nucleotide binding;translation initiation factor activity;translation initiation factor binding;formation of translation preinitiation complex;regulation of translational initiation		
Phvul.009G086900.1	BTB POZ domain-containing NPY1	plasma membrane;signal transducer activity;signal transduction;flower development;positive regulation of flower development;inflorescence development;basipetal auxin transport;apical protein localization;animal organ development;cotyledon development		
Phvul.009G087800.1	Sister chromatid cohesion PDS5 like B-B			
Phvul.009G088900.1	Two-component response regulator ARR12	nucleus;DNA binding;transcription factor activity, sequence-specific DNA binding;phosphorelay signal transduction system;transcription, DNA-templated;regulation of transcription, DNA-templated		
Phvul.009G089500.1	probable receptor kinase At1g80640 isoform X1	integral component of membrane;protein serine/threonine kinase activity;ATP binding;protein phosphorylation	ec:2.7.11	
Phvul.009G089500.2	probable receptor kinase At1g80640 isoform X1	integral component of membrane;protein serine/threonine kinase activity;ATP binding;protein phosphorylation	ec:2.7.11	
Phvul.009G089700.1	transcriptional regulator family ,	nucleus;regulation of transcription, DNA-templated		
Phvul.009G090100.1	cellulose synthase	plasma membrane;integral component of membrane;cellulose synthase (UDP-forming) activity;metal ion binding;cellulose biosynthetic process;cell wall organization	ec:2.4.1.12-synthase (UDP-forming);ec:2.4.1	Starch and sucrose metabolism
Phvul.009G090400.1	pentatricopeptide repeat-containing At3g54980, mitochondrial-like isoform X1			
Phvul.009G092800.1	hypothetical protein PHAVU_009G092800g			
Phvul.009G092900.1	G-type lectin S-receptor-like serine	plasma membrane;integral component of membrane;peroxidase activity;protein serine/threonine kinase	ec:2.7.11;ec:2.7.10;ec:1.11.1.7-	Phenylpropanoid biosynthesis

	threonine- kinase SD2-5	activity;transmembrane receptor protein tyrosine kinase activity;ATP binding;heme binding;carbohydrate binding;ubiquitin protein ligase binding;response to oxidative stress;peptidyl-tyrosine phosphorylation;oxidation-reduction process;cellular oxidant detoxification	lactoperoxidase;ec:2.7.10.1	
Phvul.009G093500.1	Phospholipase D alpha 1	membrane;phospholipase D activity;calcium ion binding;nucleotidyltransferase activity;N-acylphosphatidylethanolamine-specific phospholipase D activity;lipid catabolic process;phosphatidylcholine metabolic process	ec:3.1.1;ec:3.1;ec:3.1.4.4-D	Ether lipid metabolism;Glycerophospholipid metabolism
Phvul.009G097300.1	TIFY 8	nucleus;transcription corepressor activity;response to wounding;regulation of defense response;negative regulation of nucleic acid-templated transcription;regulation of jasmonic acid mediated signaling pathway		
Phvul.009G097300.2	TIFY 8	nucleus;transcription corepressor activity;response to wounding;regulation of defense response;negative regulation of nucleic acid-templated transcription;regulation of jasmonic acid mediated signaling pathway		
Phvul.009G100600.1	KAKU4 isoform X1			
Phvul.009G100700.1	COP9 signalosome complex subunit 5b-like	kinase activity;phosphorylation		
Phvul.009G102200.1	probable xyloglucan glycosyltransferase 5	integral component of membrane;glucomannan 4-beta-mannosyltransferase activity;mannosylation	ec:2.4.1.32	
Phvul.009G102400.1	Nucleolysin TIAR	nucleotide binding;nucleic acid binding		
Phvul.009G104700.1	pentatricopeptide repeat-containing At3g15130	zinc ion binding;microtubule-severing ATPase activity;metabolic process	ec:3.6.4.3	
Phvul.009G105100.1	formin 8	DNA methylation;methylation-dependent chromatin silencing;asymmetric cell division;regulation of flower development;chromatin silencing by small RNA;floral organ formation;histone H3-K9 methylation		
Phvul.009G105200.1	bromo-adjacent-like (BAH) domain	chromatin;chromatin silencing complex;nucleotide binding;transcription regulatory region sequence-specific DNA binding;chromatin binding;chromatin silencing;heterochromatin assembly		
Phvul.009G106000.1	hypothetical protein PHAVU_009G106000g			
Phvul.009G106600.1	la 1-like	nucleus;intracellular ribonucleoprotein complex;nucleotide binding;RNA binding;RNA processing;nucleocytoplasmic transport;post-embryonic development;macromolecule localization;single-organism cellular process;anatomical structure development;organic substance transport;single-organism intracellular transport		
Phvul.009G107600.1	EMBRYONIC FLOWER 1			
Phvul.009G108600.1	VAN3-binding -like isoform X1			
Phvul.009G108700.1	probable lactoylglutathione lyase, chloroplast	chloroplast stroma;stromule;thylakoid lumen;lactoylglutathione lyase activity;metal ion binding;dioxygenase activity;methylglyoxal catabolic process to D-lactate via S-lactoyl-glutathione;oxidation-reduction process	ec:4.4.1.5-lyase	Pyruvate metabolism
Phvul.009G108700.2	probable lactoylglutathione lyase, chloroplast	chloroplast stroma;stromule;thylakoid lumen;lactoylglutathione lyase activity;metal ion binding;dioxygenase activity;methylglyoxal catabolic process to D-lactate via S-lactoyl-glutathione;oxidation-reduction process	ec:4.4.1.5-lyase	Pyruvate metabolism

Phvul.009G109300.1	probable glycosyltransferase At5g03795	integral component of membrane;glucuronosyl-N-acetylglucosaminyl-proteoglycan 4-alpha-N-acetylglucosaminyltransferase activity;metabolic process	ec:2.4.1.224-4-alpha-N-acetylglucosaminyltransferase	Glycosaminoglycan biosynthesis - heparan sulfate / heparin
Phvul.009G109900.1	DUF810 family	plant-type cell wall biogenesis;cell growth;cellulose metabolic process		
Phvul.009G111700.1	tryptophan--tRNA ligase, mitochondrial isoform X1	mitochondrion;chloroplast stroma;tryptophan-tRNA ligase activity;ATP binding;tryptophanyl-tRNA aminoacylation;chloroplast organization;embryo development ending in seed dormancy;thylakoid membrane organization;vegetative to reproductive phase transition of meristem;iron-sulfur cluster assembly;plant ovule development	ec:6.1.1.2-ligase	Aminoacyl-tRNA biosynthesis
Phvul.009G111900.1	ribosomal L17 family	ribosome;chloroplast;structural constituent of ribosome;translation		
Phvul.009G115100.1	Ribonucleoside-diphosphate reductase large subunit	ribonucleoside-diphosphate reductase complex;ribonucleoside-diphosphate reductase activity, thioredoxin disulfide as acceptor;ATP binding;DNA replication;deoxyribonucleotide biosynthetic process;oxidation-reduction process	ec:1.17.4.1-reductase	Purine metabolism;Glutathione metabolism;Pyrimidine metabolism
Phvul.009G115700.1	probable galacturonosyltransferase 3	Golgi membrane;endosome;vacuolar membrane;trans-Golgi network;integral component of membrane;polygalacturonate 4-alpha-galacturonosyltransferase activity;mitotic cell cycle;DNA metabolic process;RNA processing;sister chromatid cohesion;positive regulation of cell proliferation;photomorphogenesis;embryo development ending in seed dormancy;seed germination;regulation of flower development;meristem structural organization;seed dormancy process;sugar mediated signaling pathway;vegetative to reproductive phase transition of meristem;protein ubiquitination;lipid storage;chromatin silencing by small RNA;regulation of chromosome organization;meiotic chromosome segregation;pectin biosynthetic process;positive regulation of transcription, DNA-templated;response to freezing;cell wall organization	ec:2.4.1.43-4-alpha-galacturonosyltransferase	Amino sugar and nucleotide sugar metabolism;Starch and sucrose metabolism
Phvul.009G116800.1	hypothetical protein PHAVU_009G116800g			
Phvul.009G116800.2	hypothetical protein PHAVU_009G116800g			
Phvul.009G116900.1	probable alanine--tRNA ligase, chloroplastic	mitochondrion;cytosol;chloroplast stroma;membrane;tRNA binding;DNA binding;transcription factor activity, sequence-specific DNA binding;alanine-tRNA ligase activity;ATP binding;zinc ion binding;amino acid binding;sigma factor activity;DNA-templated transcription, initiation;regulation of transcription, DNA-templated;tRNA modification;alanyl-tRNA aminoacylation;embryo development ending in seed dormancy	ec:6.1.1.7-ligase	Aminoacyl-tRNA biosynthesis
Phvul.009G117100.1	PHD and RING finger domain-containing 1	zinc ion binding;ligase activity;metabolic process		
Phvul.009G117100.2	PHD and RING finger domain-containing 1	zinc ion binding;ligase activity;metabolic process		
Phvul.009G117100.3	PHD and RING finger domain-containing 1	zinc ion binding;ligase activity;metabolic process		
Phvul.009G117100.4	PHD and RING finger domain-containing 1	zinc ion binding;ligase activity;metabolic process		

Phvul.009G117300.1	RNA polymerase sigma factor rpoD	cytoplasm;integral component of membrane;plastid sigma factor activity;DNA binding;transcription factor activity, sequence-specific DNA binding;alanine-tRNA ligase activity;ATP binding;pentose-phosphate shunt;DNA-templated transcription, initiation;regulation of transcription, DNA-templated;rRNA processing;alanyl-tRNA aminoacylation;response to blue light;chloroplast relocation;thylakoid membrane organization;response to far red light;carotenoid biosynthetic process;isopentenyl diphosphate biosynthetic process, methylerythritol 4-phosphate pathway	ec:6.1.1.7-ligase	Aminoacyl-tRNA biosynthesis
Phvul.009G120000.1	subtilisin-like protease	cell wall;membrane;serine-type endopeptidase activity;sulfur amino acid metabolic process;polysaccharide catabolic process;starch metabolic process;proteolysis;microtubule nucleation;cellular amino acid biosynthetic process;serine family amino acid metabolic process;plant-type cell wall modification;plant-type cell wall biogenesis;regulation of meristem growth;glucosinolate biosynthetic process	ec:3.4.21	
Phvul.009G121200.1	phosphatidylinositol 4-phosphate 5-kinase 1-like	1-phosphatidylinositol-4-phosphate 5-kinase activity;phosphatidylinositol phosphorylation	ec:2.7.1.68-5-kinase	Inositol phosphate metabolism;Phosphatidylinositol signaling system
Phvul.009G121200.2	phosphatidylinositol 4-phosphate 5-kinase 1-like	1-phosphatidylinositol-4-phosphate 5-kinase activity;phosphatidylinositol phosphorylation	ec:2.7.1.68-5-kinase	Inositol phosphate metabolism;Phosphatidylinositol signaling system
Phvul.009G121600.1	Serine Threonine kinase family	protein serine/threonine/tyrosine kinase activity;ATP binding;protein phosphorylation;mitotic cell cycle checkpoint;chromosome separation	ec:2.7.12.1	
Phvul.009G121600.2	Serine Threonine kinase family	protein serine/threonine/tyrosine kinase activity;ATP binding;protein phosphorylation;mitotic cell cycle checkpoint;chromosome separation	ec:2.7.12.1	
Phvul.009G121700.1	nucleic acid-binding	NAD+ ADP-ribosyltransferase activity;metal ion binding;metabolic process	ec:2.4.2.30	
Phvul.009G122500.1	pentatricopeptide repeat-containing At3g62890-like	nucleic acid binding;zinc ion binding;microtubule-severing ATPase activity;metabolic process	ec:3.6.4.3	
Phvul.009G122600.1	G-type lectin S-receptor-like serine threonine- kinase At5g35370	plasma membrane;integral component of membrane;protein serine/threonine kinase activity;calmodulin binding;ATP binding;carbohydrate binding;protein phosphorylation	ec:2.7.11	
Phvul.009G124200.1	DNA ligase 1	membrane;DNA binding		
Phvul.009G124500.1	P-loop nucleoside triphosphate hydrolase superfamily ,	nucleus;DNA repair;cell cycle		
Phvul.009G126000.1	Replication factor C subunit 5	DNA clamp loader activity;DNA-directed DNA polymerase activity;ATP binding;mRNA splicing, via spliceosome;DNA-dependent DNA replication;mitotic recombination;methylation-dependent chromatin silencing;embryo sac egg cell differentiation;regulation of flower development;RNA interference;chromatin silencing by small RNA;negative regulation of defense response;floral organ formation;histone H3-K9 methylation;DNA biosynthetic process	ec:2.7.7.7-DNA polymerase	Purine metabolism;Pyrimidine metabolism
Phvul.009G128400.1	chloroplast processing peptidase-like	chloroplast thylakoid membrane;integral component of membrane;mitochondrial inner membrane peptidase complex;serine-type endopeptidase activity;signal peptide processing;protein processing involved in protein targeting to mitochondrion;thylakoid membrane organization;mitochondrial respiratory chain complex assembly	ec:3.4.21	

Phvul.009G128400.2	chloroplast processing peptidase-like	chloroplast thylakoid membrane;integral component of membrane;mitochondrial inner membrane peptidase complex;serine-type endopeptidase activity;signal peptide processing;protein processing involved in protein targeting to mitochondrion;thylakoid membrane organization;mitochondrial respiratory chain complex assembly	ec:3.4.21	
Phvul.009G129400.1	translocase subunit SECA2, chloroplastic isoform X1	intracellular;membrane;ATP binding;protein targeting;protein import		
Phvul.009G129500.1	1-aminocyclopropane-1-carboxylate oxidase 5	1-aminocyclopropane-1-carboxylate oxidase activity;metal ion binding;iron ion transport;cellular response to iron ion starvation;response to nitrate;nitrate transport;brassinosteroid biosynthetic process;oxidation-reduction process	ec:1.14.17.4-oxidase	Cysteine and methionine metabolism
Phvul.009G130300.1	probable receptor-like serine threonine- kinase At5g57670	protein serine/threonine kinase activity;ATP binding;hydrolase activity;protein phosphorylation;response to stress	ec:2.7.11	
Phvul.009G133500.1	transmembrane ,	integral component of membrane		
Phvul.009G133900.1	thioredoxin M-type, chloroplastic-like	chloroplast;protein disulfide oxidoreductase activity;oxidoreductase activity, acting on a sulfur group of donors, disulfide as acceptor;sulfate assimilation;protein folding;glycerol ether metabolic process;cellular response to oxidative stress;cell redox homeostasis;oxidation-reduction process		
Phvul.009G136800.1	probable inactive receptor kinase At4g23740	intracellular;integral component of membrane;MAP kinase kinase kinase activity;transmembrane receptor protein tyrosine kinase activity;ATP binding;MAPK cascade;activation of MAPKK activity;peptidyl-tyrosine phosphorylation	ec:2.7.11;ec:2.7.11.25;ec:2.7.10.1	
Phvul.009G138900.1	WRKY family transcription factor	transcription factor activity, sequence-specific DNA binding;sequence-specific DNA binding;regulation of transcription, DNA-templated		
Phvul.009G139100.1	ABC transporter F family member 5	ATP binding;ATPase activity;metabolic process	ec:3.6.1.3-adenylpyrophosphatase	Purine metabolism
Phvul.009G139500.1	4-alpha-glucanotransferase, chloroplastic amyloplastic	4-alpha-glucanotransferase activity;maltose catabolic process;glycogen metabolic process;starch catabolic process;glucose metabolic process	ec:2.4.1.25-disproportionating enzyme	Starch and sucrose metabolism
Phvul.009G139600.1	VAC14 homolog	vacuolar membrane		
Phvul.009G141300.1	uncharacterized membrane At1g06890-like	integral component of membrane;carbohydrate transport		
Phvul.009G141400.1	Poly(A) RNA polymerase cid14	nucleotidyltransferase activity;metabolic process		
Phvul.009G142600.1	Exosome complex exonuclease RRP44	3'-5'-exoribonuclease activity;endonuclease activity;exoribonuclease II activity;rRNA processing;mRNA catabolic process;rRNA catabolic process;RNA phosphodiester bond hydrolysis, exonucleolytic	ec:3.1.13.1;ec:3.1.13	
Phvul.009G143300.1	, chloroplastic	copper ion binding;response to metal ion;photoperiodism, flowering		
Phvul.009G144900.1	probable rhamnogalacturonate lyase B	lyase activity;carbohydrate binding;carbohydrate metabolic process		
Phvul.009G144900.2	probable rhamnogalacturonate lyase B	lyase activity;carbohydrate binding;carbohydrate metabolic process		
Phvul.009G144900.3	probable rhamnogalacturonate lyase B	lyase activity;carbohydrate binding;carbohydrate metabolic process		
Phvul.009G149100.1	zinc finger CCCH domain-containing 38-like	metal ion binding		

Phvul.009G149800.1	phosphatidylinositol 4-kinase beta 1-like isoform X1	intracellular;membrane;1-phosphatidylinositol 4-kinase activity;phosphatidylinositol phosphorylation;phosphatidylinositol-mediated signaling	ec:2.7.1.67-4-kinase	Inositol phosphate metabolism;Phosphatidylinositol signaling system
Phvul.009G150000.1	succinate dehydrogenase subunit 7B, mitochondrial isoform X1	integral component of membrane		
Phvul.009G150100.1	plant NAC domain	nucleus;membrane;DNA binding;transcription, DNA-templated;regulation of transcription, DNA-templated		
Phvul.009G153100.1	beta-galactosidase 10	plant-type cell wall;beta-galactosidase activity;carbohydrate binding;carbohydrate metabolic process	ec:3.2.1.23-lactase (ambiguous)	Other glycan degradation;Glycosphingolipid biosynthesis - ganglio series;Sphingolipid metabolism;Galactose metabolism;Glycosaminoglycan degradation
Phvul.009G153600.1	amino acid permease 4-like	plasma membrane;integral component of membrane;amino acid transmembrane transporter activity;amino acid transmembrane transport		
Phvul.009G153700.1	Amino acid permease 2	plasma membrane;integral component of membrane;amino acid transmembrane transporter activity;amino acid transmembrane transport		
Phvul.009G153800.1	auxilin-related 2-like			
Phvul.009G153900.1	ultraviolet-B receptor UVR8 isoform X1	chromatin;chromatin binding;guanyl-nucleotide exchange factor activity;ligase activity;protein homodimerization activity;glucose catabolic process;entrainment of circadian clock;response to UV-B;positive regulation of GTPase activity		
Phvul.009G154000.1	lipase-like PAD4	integral component of membrane;hydrolase activity;lipid metabolic process		
Phvul.009G154600.1	farnesylcysteine lyase	vacuolar membrane;prenylcysteine oxidase activity;lyase activity;abscisic acid-activated signaling pathway;prenylated protein catabolic process;prenylcysteine catabolic process;hydrogen peroxide catabolic process;farnesyl diphosphate metabolic process;oxidation-reduction process	ec:1.8.3.5-oxidase	Biosynthesis of antibiotics;Terpenoid backbone biosynthesis
Phvul.009G155700.1	nuclear snf4	chloroplast stroma;chloroplast envelope;apoplast;oxidoreductase activity;pentose-phosphate shunt;leaf morphogenesis;thylakoid membrane organization;carotenoid biosynthetic process;cell differentiation;oxidation-reduction process		
Phvul.009G157000.1	DUF668 family			
Phvul.009G157600.1	ferrochelatase-2, chloroplastic-like	ferrochelatase activity;heme biosynthetic process	ec:4.99.1.1-ferro-protoporphyrin chelatase	Porphyrin and chlorophyll metabolism
Phvul.009G158900.1	calcium-binding mitochondrial carrier S -2-B-like	integral component of membrane;structural constituent of ribosome;calcium ion binding;translation;transmembrane transport		
Phvul.009G158900.3	calcium-binding mitochondrial carrier S -2-like isoform X2	integral component of membrane;structural constituent of ribosome;calcium ion binding;translation;transmembrane transport		
Phvul.009G159400.1	FRIGIDA	integral component of membrane		
Phvul.009G160400.1	DYAD			
Phvul.009G161300.1	U5 small nuclear ribonucleo 40 kDa -like			
Phvul.009G161400.1	MEI2-like 1 isoform X1	nucleotide binding;nucleic acid binding		
Phvul.009G161400.2	MEI2-like 1 isoform X1	nucleotide binding;nucleic acid binding		

Phvul.009G161400.3	MEI2-like 1 isoform X1	nucleotide binding;nucleic acid binding		
Phvul.009G161600.1	Lysosomal alpha-mannosidase	alpha-mannosidase activity;zinc ion binding;carbohydrate binding;mannose metabolic process;protein deglycosylation	ec:3.2.1.24-alpha-D-mannosidase	Other glycan degradation
Phvul.009G163500.1	probable serine threonine- kinase At1g54610	cyclin-dependent protein serine/threonine kinase activity;ATP binding;protein phosphorylation;regulation of cell cycle	ec:2.7.11.22	
Phvul.009G164400.1	tRNA nucleotidyltransferase poly(A) polymerase	RNA binding;hydrolase activity;CTP:tRNA cytidylyltransferase activity;CTP:3'-cytidine-tRNA cytidylyltransferase activity;ATP:3'-cytidine-cytidine-tRNA adenylyltransferase activity;tRNA 3'-terminal CCA addition	ec:2.7.7.72	
Phvul.009G166500.1	hypothetical protein PHAVU_009G166500g	integral component of membrane;cytolysis;defense response to Gram-negative bacterium		
Phvul.009G166500.2	hypothetical protein PHAVU_009G166500g	integral component of membrane;cytolysis;defense response to Gram-negative bacterium		
Phvul.009G166500.3	hypothetical protein PHAVU_009G166500g	integral component of membrane;cytolysis;defense response to Gram-negative bacterium		
Phvul.009G166500.4	hypothetical protein PHAVU_009G166500g			
Phvul.009G166600.1	snurportin-1			
Phvul.009G167100.1	hypothetical protein PHAVU_009G167100g	ATP binding;microtubule-severing ATPase activity	ec:3.6.4.3	
Phvul.009G168100.1	transcription factor GTE10-like			
Phvul.009G168200.1	nucleic acid-binding, OB-fold	cytosol;plastid chromosome;chloroplast stroma;nucleic acid binding;transcription from plastid promoter;positive regulation of transcription, DNA-templated		
Phvul.009G169100.1	kelch domain-containing 4	lipid transport		
Phvul.009G169400.1	callose synthase 10	1,3-beta-D-glucan synthase complex;integral component of membrane;1,3-beta-D-glucan synthase activity;(1->3)-beta-D-glucan biosynthetic process	ec:2.4.1.34-synthase	Starch and sucrose metabolism
Phvul.009G170000.1	probable UDP-N-acetylglucosamine--peptide N-acetylglucosaminyltransferase SPINDLY	transferase activity, transferring glycosyl groups;nuclear-transcribed mRNA catabolic process		
Phvul.009G170000.2	probable UDP-N-acetylglucosamine--peptide N-acetylglucosaminyltransferase SPINDLY	transferase activity, transferring glycosyl groups;nuclear-transcribed mRNA catabolic process		
Phvul.009G170500.1	Pectate lyase	pectate lyase activity;metal ion binding;pectin catabolic process	ec:4.2.2.2-lyase	Pentose and glucuronate interconversions
Phvul.009G171200.1	leucine-rich repeat receptor-like serine threonine tyrosine-kinase SOBIR1	integral component of membrane;calmodulin-dependent protein kinase activity;transmembrane receptor protein tyrosine kinase activity;ATP binding;positive regulation of cell death;peptidyl-tyrosine phosphorylation;positive regulation of defense response;negative regulation of floral organ abscission	ec:2.7.11.17;ec:2.7.10.1	
Phvul.009G172500.1	transcription factor IWS1	nucleus;DNA binding;translation elongation factor activity;transcription, DNA-templated;translational elongation		
Phvul.009G172900.1	manganese-dependent ADP-ribose CDP-alcohol	ADP-ribose diphosphatase activity;CDP-glycerol diphosphatase activity;metabolic process	ec:3.6.1.16-diphosphatase;ec:3.6.1.13-diphosphatase	Glycerophospholipid metabolismPurine metabolism

	diphosphatase-like isoform X1			
Phvul.009G172900.2	manganese-dependent ADP-ribose CDP-alcohol diphosphatase-like isoform X1	ADP-ribose diphosphatase activity;CDP-glycerol diphosphatase activity;metabolic process	ec:3.6.1.16-diphosphatase;ec:3.6.1.13-diphosphatase	Glycerophospholipid metabolismPurine metabolism
Phvul.009G175300.1	tryptophan aminotransferase-related 2-like	integral component of membrane;transaminase activity;alliin lyase activity;metabolic process	ec:4.4.1.4	
Phvul.009G176400.1	LRR receptor-like serine threonine-kinase GSO1	integral component of membrane;kinase activity;2-alkenal reductase [NAD(P)] activity;phosphorylation;oxidation-reduction process	ec:1.3.1.74	
Phvul.009G176500.1	MORC family CW-type zinc finger 3-like	plasmodesma;chloroplast envelope;ATPase activity;sister chromatid cohesion;determination of bilateral symmetry;organ morphogenesis;xylem and phloem pattern formation;chromatin silencing by small RNA;regulation of chromatin silencing;meiotic chromosome segregation;flower morphogenesis;RNA-directed DNA methylation	ec:3.6.1.3-adenylpyrophosphatase	Purine metabolism
Phvul.009G176900.1	transmembrane ,	cell;integral component of membrane;maltose metabolic process;pentose-phosphate shunt;chloroplast relocation;stomatal complex morphogenesis;mRNA modification;starch biosynthetic process;positive regulation of catalytic activity		
Phvul.009G177400.1	transmembrane ,	integral component of membrane		
Phvul.009G179300.1	EXECUTER 2, chloroplastic	thylakoid membrane;singlet oxygen-mediated programmed cell death		
Phvul.009G179300.2	EXECUTER 2, chloroplastic	thylakoid membrane;singlet oxygen-mediated programmed cell death		
Phvul.009G180300.1	probable alpha,alpha-trehalose-phosphate synthase [UDP-forming] 9	alpha,alpha-trehalose-phosphate synthase (UDP-forming) activity;trehalose-phosphatase activity;trehalose biosynthetic process;dephosphorylation	ec:2.4.1.15-synthase (UDP-forming);ec:3.1.3.12-trehalose 6-phosphatase	Starch and sucrose metabolismStarch and sucrose metabolism
Phvul.009G184500.1	probable LRR receptor-like serine threonine- kinase At1g74360	intracellular;integral component of membrane;MAP kinase kinase kinase activity;transmembrane receptor protein tyrosine kinase activity;ATP binding;MAPK cascade;activation of MAPKK activity;peptidyl-tyrosine phosphorylation	ec:2.7.11;ec:2.7.11.25;ec:2.7.10.1	
Phvul.009G185100.1	phospholipid-transporting ATPase 9	Golgi membrane;trans-Golgi network;plasma membrane;integral component of membrane;magnesium ion binding;phospholipid-translocating ATPase activity;ATP binding;metabolic process;phospholipid translocation;Golgi vesicle budding	ec:3.6.3.1	
Phvul.009G187100.1	E3 ubiquitin- ligase MARCH4-like isoform X7	integral component of membrane;zinc ion binding		
Phvul.009G187100.2	E3 ubiquitin- ligase MARCH4-like isoform X7	integral component of membrane;zinc ion binding		
Phvul.009G187300.1	probable arabinosyltransferase ARAD1	integral component of membrane;glucuronosyl-N-acetylglucosaminyl-proteoglycan 4-alpha-N-acetylglucosaminyltransferase activity;metabolic process	ec:2.4.1.224-4-alpha-N-acetylglucosaminyltransferase	Glycosaminoglycan biosynthesis - heparan sulfate / heparin
Phvul.009G189600.1	hypothetical protein PHAVU_009G189600g			
Phvul.009G191000.1	2-aminoethanethiol dioxygenase	cysteamine dioxygenase activity;anaerobic respiration;peptidyl-cysteine oxidation;detection of hypoxia	ec:1.13.11.19-dioxygenase	Taurine and hypotaurine metabolism
Phvul.009G191000.2	2-aminoethanethiol dioxygenase	cysteamine dioxygenase activity;anaerobic respiration;peptidyl-cysteine oxidation;detection of hypoxia	ec:1.13.11.19-dioxygenase	Taurine and hypotaurine metabolism

Phvul.009G191000.3	2-aminoethanethiol dioxygenase	cysteamine dioxygenase activity;anaerobic respiration;peptidyl-cysteine oxidation;detection of hypoxia	ec:1.13.11.19-dioxygenase	Taurine and hypotaurine metabolism
Phvul.009G191500.1	mitochondrial substrate carrier family E	integral component of membrane;structural constituent of ribosome;translation;transmembrane transport		
Phvul.009G193100.1	dentin sialophospho ,			
Phvul.009G193400.1	pre-mRNA-splicing factor ATP-dependent RNA helicase DHX16	spliceosomal complex;ATP-dependent RNA helicase activity;ATP binding;poly(A) RNA binding;RNA processing		
Phvul.009G196400.1	hypothetical protein PHAVU_009G196400g, partial			
Phvul.009G196600.1	heat shock family	integral component of membrane		
Phvul.009G196800.1	structural maintenance of chromosomes 3 isoform X1	chromatin;nucleus;cohesin core heterodimer;plasmodesma;chromatin binding;ATP binding;DNA repair;mitotic sister chromatid cohesion;production of ta-siRNAs involved in RNA interference;production of miRNAs involved in gene silencing by miRNA;defense response to virus		
Phvul.009G197800.1	2-alkenal reductase (NADP(+)-dependent)-like	integral component of membrane;zinc ion binding;2-alkenal reductase [NAD(P)] activity;oxidation-reduction process	ec:1.3.1.74	
Phvul.009G200200.1	acyl ACP-thioesterase	oleoyl-[acyl-carrier-protein] hydrolase activity;myristoyl-[acyl-carrier-protein] hydrolase activity;palmitoyl-[acyl-carrier-protein] hydrolase activity;fatty acid biosynthetic process	ec:3.1.2.14-hydrolase	Fatty acid biosynthesis
Phvul.009G202000.1	ninja-family 2-like isoform X1	signal transduction		
Phvul.009G202600.1	pentatricopeptide repeat-containing At2g38420, mitochondrial			
Phvul.009G203100.1	COBRA 2 precursor	plant-type cell wall;external side of plasma membrane;longitudinal side of cell surface;integral component of membrane;anchored component of plasma membrane;polysaccharide biosynthetic process;polysaccharide catabolic process;starch metabolic process;glycolytic process;calcium ion transport;water transport;hyperosmotic response;Golgi organization;pattern specification process;regulation of cell size;response to temperature stimulus;response to salt stress;response to fructose;multidimensional cell growth;auxin polar transport;cell tip growth;cellulose microfibril organization;cellulose metabolic process;anthocyanin accumulation in tissues in response to UV light;response to cadmium ion;root hair elongation		
Phvul.009G204500.1	GDP-fucose O-fucosyltransferase	cytoplasm;integral component of membrane;transferase activity, transferring glycosyl groups;metabolic process		
Phvul.009G204900.1	Poly(A) polymerase	nucleus;integral component of membrane;RNA binding;polynucleotide adenylyltransferase activity;mRNA polyadenylation	ec:2.7.7.19	
Phvul.009G205100.1	cellulose synthase A catalytic subunit 7 [UDP-forming]	plasma membrane;integral component of membrane;zinc ion binding;cellulose synthase (UDP-forming) activity;cellulose biosynthetic process;cell wall organization	ec:2.4.1.12-synthase (UDP-forming)	Starch and sucrose metabolism
Phvul.009G205200.1	Cellulose synthase A catalytic subunit 7 [UDP-forming]	plasma membrane;integral component of membrane;zinc ion binding;cellulose synthase (UDP-forming) activity;cellulose biosynthetic process;cell wall organization	ec:2.4.1.12-synthase (UDP-forming)	Starch and sucrose metabolism
Phvul.009G205400.1	hypothetical protein PHAVU_009G205400g			

Phvul.009G210000.1	glycerol-3-phosphate transporter 5	integral component of membrane;transmembrane transporter activity;nitrate transport;transmembrane transport		
Phvul.009G210800.1	transmembrane ,	plasmodesma;integral component of membrane		
Phvul.009G214800.1	hypothetical protein PHAVU_009G214800g			
Phvul.009G215600.1	endoplasmic reticulum oxidoreductin-1-like	endoplasmic reticulum;integral component of membrane;protein disulfide isomerase activity;oxidoreductase activity, acting on a sulfur group of donors, disulfide as acceptor;protein folding;oxidation-reduction process	ec:5.3.4.1	
Phvul.009G217900.1	vacuolar sorting-associated 54, chloroplastic	GARP complex;syntaxin binding;Golgi to vacuole transport;retrograde transport, endosome to Golgi		
Phvul.009G218000.1	vacuolar sorting-associated 2 homolog 2	ESCRT III complex;microtubule cytoskeleton organization;cytokinesis by cell plate formation;DNA replication;DNA methylation;vacuolar transport;cell proliferation;histone phosphorylation;histone H3-K9 methylation		
Phvul.009G218100.1	Ferrochelatase-2, chloroplastic	integral component of membrane;ferrochelatase activity;heme biosynthetic process;response to stress;macromolecule localization;regulation of programmed cell death	ec:4.99.1.1-ferro-protoporphyrin chelatase	Porphyrin and chlorophyll metabolism
Phvul.009G219000.1	cytochrome b561	integral component of membrane		
Phvul.009G219100.1	pentatricopeptide repeat-containing At3g06430, chloroplastic-like	microtubule-severing ATPase activity;rRNA binding;metabolic process;embryo sac development;pollen development;embryo development;pollen tube development	ec:3.6.4.3	
Phvul.009G219600.1	hypothetical protein PHAVU_009G219600g			
Phvul.009G220100.1	DNA binding ,	DNA binding;protein dimerization activity		
Phvul.009G222000.1	transcription initiation factor TFIID subunit 10	nucleus;DNA-templated transcription, initiation		
Phvul.009G223200.1	AT-rich interactive domain-containing 4	DNA binding;zinc ion binding		
Phvul.009G224700.1	DEAD-box ATP-dependent RNA helicase 17	intracellular;RNA binding;ATP-dependent RNA helicase activity;ATP binding;mitotic recombination;mRNA export from nucleus;protein targeting to mitochondrion;embryo sac egg cell differentiation;photomorphogenesis;cullin deneddylation;RNA secondary structure unwinding;transcription factor import into nucleus;protein maturation		
Phvul.009G226100.1	transmembrane 184 homolog DDB_G0279555 isoform X1	integral component of membrane;transporter activity;transport;fatty acid catabolic process;plant-type hypersensitive response		
Phvul.009G227400.1	acyl- N-acyltransferase (NAT) superfamily	protein acetyltransferase complex;peptide alpha-N-acetyltransferase activity;N-terminal protein amino acid acetylation	ec:2.3.1.88	
Phvul.009G227900.1	DENN domain-containing 5B	integral component of membrane		
Phvul.009G227900.2	DENN domain-containing 5B	integral component of membrane		
Phvul.009G227900.3	DENN domain-containing 5B	integral component of membrane		
Phvul.009G228000.1	growth-regulating factor	nucleus;ATP binding;transcription, DNA-templated;regulation of transcription, DNA-templated;developmental process		

Phvul.009G228500.1	replication A 70 kDa DNA-binding subunit A	nuclear chromatin;DNA binding;double-strand break repair via homologous recombination;DNA replication;mitotic recombination;sister chromatid cohesion;male meiosis I;pyrimidine ribonucleotide biosynthetic process;pollen development;embryo sac egg cell differentiation;photomorphogenesis;embryo development ending in seed dormancy;seed germination;regulation of flower development;meristem structural organization;seed dormancy process;sugar mediated signaling pathway;vegetative to reproductive phase transition of meristem;response to gamma radiation;somatic cell DNA recombination;protein ubiquitination;lipid storage;regulation of telomere maintenance;meiotic DNA double-strand break formation;telomere maintenance in response to DNA damage;post-translational protein modification;positive regulation of transcription, DNA-templated;male gamete generation;response to freezing;chiasma assembly		
Phvul.009G229900.1	probable phosphatase 2C 50 isoform X1	protein serine/threonine phosphatase activity;metal ion binding;protein dephosphorylation		
Phvul.009G229900.2	probable phosphatase 2C 50 isoform X1	protein serine/threonine phosphatase activity;metal ion binding;protein dephosphorylation		
Phvul.009G229900.3	probable phosphatase 2C 50 isoform X1	protein serine/threonine phosphatase activity;metal ion binding;protein dephosphorylation		
Phvul.009G229900.4	probable phosphatase 2C 50 isoform X1	protein serine/threonine phosphatase activity;metal ion binding;protein dephosphorylation		
Phvul.009G229900.5	probable phosphatase 2C 50 isoform X1	protein serine/threonine phosphatase activity;metal ion binding;protein dephosphorylation		
Phvul.009G229900.6	probable phosphatase 2C 50 isoform X1	protein serine/threonine phosphatase activity;metal ion binding;protein dephosphorylation		
Phvul.009G229900.7	probable phosphatase 2C 50 isoform X1	protein serine/threonine phosphatase activity;metal ion binding;protein dephosphorylation		
Phvul.009G230400.1	DNA-directed RNA polymerase II subunit RPB2	DNA-directed RNA polymerase II, core complex;cytosol;plasmodesma;RNA polymerase II activity;DNA binding;ribonucleoside binding;RNA splicing, via endonucleolytic cleavage and ligation;nuclear-transcribed mRNA catabolic process;transcription from RNA polymerase II promoter		
Phvul.009G232100.1	receptor-like serine threonine- kinase ALE2 isoform X2	integral component of membrane;protein kinase activity;ATP binding;protein phosphorylation		
Phvul.009G232100.2	receptor-like serine threonine- kinase ALE2 isoform X2	integral component of membrane;protein serine/threonine kinase activity;ATP binding;protein phosphorylation	ec:2.7.11	
Phvul.009G232100.3	receptor-like serine threonine- kinase ALE2 isoform X2	integral component of membrane;protein serine/threonine kinase activity;ATP binding;protein phosphorylation	ec:2.7.11	
Phvul.009G232500.1	beta-galactosidase	cell wall;beta-galactosidase activity;zinc ion binding;carbohydrate binding;carbohydrate metabolic process	ec:3.2.1.23-lactase (ambiguous)	Other glycan degradation;Glycosphingolipid biosynthesis - ganglio series;Sphingolipid metabolism;Galactose metabolism;Glycosaminoglycan degradation
Phvul.009G235600.1	probable inactive purple acid phosphatase 28	integral component of membrane;acid phosphatase activity;dephosphorylation	ec:3.1.3.2-phosphatase	Aminobenzoate degradation
Phvul.009G235800.1	DNA ligase 4	cytoplasm;plasmodesma;apoplast;DNA binding;DNA ligase (ATP) activity;NAD+ ADP-	ec:6.5.1.1;ec:2.4.2.30	

		ribosyltransferase activity;ATP binding;lagging strand elongation;double-strand break repair;DNA recombination;response to X-ray;DNA ligation involved in DNA repair;DNA biosynthetic process		
Phvul.009G236800.1	subtilisin-like protease	cell wall;membrane;serine-type endopeptidase activity;proteolysis	ec:3.4.21	
Phvul.009G237400.1	core-2 I-branching enzyme	integral component of membrane;acetylglucosaminyltransferase activity;metabolic process	ec:2.4.1	
Phvul.009G237900.1	pumilio homolog 6, chloroplastic	mRNA binding;nucleotide biosynthetic process		
Phvul.009G237900.2	pumilio homolog 6, chloroplastic	mRNA binding;nucleotide biosynthetic process		
Phvul.009G239300.1	adagio 1	nucleus;cytosol;membrane;SCF ubiquitin ligase complex;scavenger receptor activity;blue light photoreceptor activity;ligase activity;fatty acid beta-oxidation;receptor-mediated endocytosis;circadian rhythm;blue light signaling pathway;flower development;response to red light;protein import into peroxisome matrix;SCF-dependent proteasomal ubiquitin-dependent protein catabolic process;entrainment of circadian clock by photoperiod		
Phvul.009G239900.1	G-type lectin S-receptor-like serine threonine- kinase	integral component of membrane;protein serine/threonine kinase activity;transmembrane receptor protein tyrosine kinase activity;ATP binding;carbohydrate binding;peptidyl-tyrosine phosphorylation	ec:2.7.11;ec:2.7.10.1	
Phvul.009G240000.1	heavy metal ATPase transporter	integral component of plasma membrane;intracellular membrane-bounded organelle;nucleotide binding;zinc ion transmembrane transporter activity;cadmium-exporting ATPase activity;metal ion binding;metabolic process;cadmium ion transmembrane transport;zinc II ion transmembrane transport	ec:3.6.3.3	
Phvul.009G240000.2	heavy metal ATPase transporter	integral component of plasma membrane;intracellular membrane-bounded organelle;nucleotide binding;zinc ion transmembrane transporter activity;cadmium-exporting ATPase activity;metal ion binding;metabolic process;cadmium ion transmembrane transport;zinc II ion transmembrane transport	ec:3.6.3.3	
Phvul.009G240500.1	UPF0481 At3g47200-like	integral component of membrane		
Phvul.009G241100.1	ATP-dependent zinc metalloprotease FTSH 2, chloroplastic	mitochondrion;chloroplast thylakoid membrane;chloroplast envelope;integral component of membrane;ATP-dependent peptidase activity;metalloendopeptidase activity;ATP binding;zinc ion binding;microtubule-severing ATPase activity;proteolysis;thylakoid membrane organization;photoinhibition;photosystem II repair;PSII associated light-harvesting complex II catabolic process;cell division	ec:3.6.4.3;ec:3.4.24	
Phvul.009G242700.1	cellulose synthase 4	plasma membrane;integral component of membrane;zinc ion binding;cellulose synthase (UDP-forming) activity;plant-type secondary cell wall biogenesis;glucuronoxylan metabolic process;cellulose biosynthetic process;defense response to bacterium;xylan biosynthetic process;defense response to fungus;cell wall organization	ec:2.4.1.12-synthase (UDP-forming)	Starch and sucrose metabolism
Phvul.009G243500.1	ribulose-1,5 bisphosphate carboxylase oxygenase large subunit N-methyltransferase, chloroplastic isoform X2	plastid-encoded plastid RNA polymerase complex;plastid chromosome;chloroplast thylakoid;protein-lysine N-methyltransferase activity;[ribulose-bisphosphate carboxylase]-lysine N-methyltransferase activity;rRNA processing;tRNA metabolic process;phosphatidylglycerol biosynthetic process;chloroplast organization;thylakoid membrane organization;stomatal complex	ec:2.1.1;ec:2.1.1.127	

		morphogenesis;peptidyl-lysine monomethylation;transcription from plastid promoter;protein targeting to chloroplast;positive regulation of transcription, DNA-templated		
Phvul.009G243800.1	vacuolar-sorting receptor 6-like	integral component of membrane;calcium ion binding		
Phvul.009G244300.1	DDB1- and CUL4-associated factor 4-like isoform X1			
Phvul.009G244700.1	zinc finger, C3HC4 type (RING finger)	ubiquitin-protein transferase activity;zinc ion binding;regulation of signal transduction;protein ubiquitination		
Phvul.009G247100.1	C2 domain	endoplasmic reticulum membrane;plasma membrane;integral component of membrane;protein storage vacuole membrane;anion transport;regulation of biological process;response to stimulus;organic substance transport;nitrogen compound transport;single-organism intracellular transport		
Phvul.009G247700.1	probable phosphatase 2C 40	protein serine/threonine phosphatase activity;protein dephosphorylation		
Phvul.009G247700.2	probable phosphatase 2C 40	protein serine/threonine phosphatase activity;protein dephosphorylation		
Phvul.009G248800.1	condensin-2 complex subunit D3	condensed chromosome, centromeric region;nuclear condensin complex;chromatin binding;histone binding;mitotic chromosome condensation;meiotic chromosome condensation;chromosome separation		
Phvul.009G249600.1	sericin 1-like			
Phvul.009G250700.1	Acyl-dehydrogenase family member 10	integral component of membrane;fatty-acyl-CoA binding;acyl-CoA dehydrogenase activity;electron carrier activity;flavin adenine dinucleotide binding;oxidoreductase activity, acting on the CH-CH group of donors, with a flavin as acceptor;response to symbiotic fungus;fatty acid beta-oxidation using acyl-CoA dehydrogenase;root hair elongation;lipid homeostasis	ec:1.3.99.3	
Phvul.009G252200.1	probable polygalacturonase	extracellular region;integral component of membrane;polygalacturonase activity;carbohydrate metabolic process;cell wall organization	ec:3.2.1.15-pectin depolymerase	Starch and sucrose metabolism;Pentose and glucuronate interconversions
Phvul.009G254500.1	calmodulin-binding transcription activator 5-like	nucleus;integral component of membrane;transcriptional activator activity, RNA polymerase II core promoter proximal region sequence-specific binding;sequence-specific DNA binding;transcription from RNA polymerase II promoter;positive regulation of transcription from RNA polymerase II promoter		
Phvul.009G254500.2	calmodulin-binding transcription activator 5-like	nucleus;transcriptional activator activity, RNA polymerase II core promoter proximal region sequence-specific binding;sequence-specific DNA binding;transcription from RNA polymerase II promoter;positive regulation of transcription from RNA polymerase II promoter		
Phvul.009G254600.1	conserved oligomeric Golgi complex subunit 4-like	endoplasmic reticulum;cytosol;Golgi transport complex;retrograde transport, vesicle recycling within Golgi;retrograde vesicle-mediated transport, Golgi to ER;Golgi organization;Golgi vesicle prefusion complex stabilization		
Phvul.009G256200.1	PH-interacting isoform X1	nucleus;Cul4-RING E3 ubiquitin ligase complex;DNA binding;regulation of transcription from RNA polymerase II promoter;cytoskeleton organization;regulation of cell shape;response to hormone		
Phvul.009G257900.1	conserved oligomeric Golgi complex subunit 4	endoplasmic reticulum;cytosol;Golgi transport complex;retrograde transport, vesicle recycling within Golgi;retrograde vesicle-mediated transport, Golgi to ER;Golgi organization;Golgi vesicle prefusion complex stabilization		

Phvul.009G258800.1	caffeic acid 3-O-methyltransferase	O-methyltransferase activity;quercetin 3-O-methyltransferase activity;protein dimerization activity;methylation	ec:2.1.1;ec:2.1.1.76-3-O-methyltransferase	Flavone and flavonol biosynthesis
Phvul.009G260300.1	inactive purple acid phosphatase	hydrolase activity;metabolic process		
Phvul.009G260500.1	tyrosine-sulfated glycopeptide receptor 1	integral component of membrane;protein serine/threonine kinase activity;transmembrane receptor protein tyrosine kinase activity;ATP binding;peptidyl-tyrosine phosphorylation	ec:2.7.11;ec:2.7.10.1	
Phvul.009G260900.1	STRUBBELIG-RECEPTOR FAMILY 6	integral component of membrane;protein kinase activity;ATP binding;protein phosphorylation		
Phvul.009G260900.2	STRUBBELIG-RECEPTOR FAMILY 6	integral component of membrane;protein kinase activity;ATP binding;protein phosphorylation		
Phvul.009G261000.1	GDP-fucose O-fucosyltransferase	integral component of membrane;peptide-O-fucosyltransferase activity;signal transduction;protein O-linked fucosylation	ec:2.4.1.221-GDP-L-fucose:polypeptide fucosyltransferase	Other types of O-glycan biosynthesis
Phvul.009G261600.1	pumilio homolog 23	RNA binding;auxin homeostasis		
Phvul.009G262400.1	dnaJ homolog subfamily B member 6-like isoform X4			
Phvul.009G263000.1	exportin-T	nuclear pore;cytosol;nuclear matrix;tRNA binding;Ran GTPase binding;nucleobase-containing compound transmembrane transporter activity;mRNA export from nucleus;N-terminal protein myristoylation;protein import into nucleus;photomorphogenesis;embryo development ending in seed dormancy;seed germination;flower development;regulation of flower development;meristem initiation;seed dormancy process;sugar mediated signaling pathway;vegetative to reproductive phase transition of meristem;protein ubiquitination;lipid storage;response to freezing;tRNA re-export from nucleus		
Phvul.010G001900.1	Pyridoxal biosynthesis PDX2	cytosol;glutaminase complex;glutaminase activity;transferase activity;protein heterodimerization activity;pyridoxine metabolic process;vitamin B6 biosynthetic process;pyridoxal phosphate biosynthetic process	ec:3.5.1.2-glutaminase I	Arginine biosynthesis;D-Glutamine and D-glutamate metabolism;Alanine, aspartate and glutamate metabolism
Phvul.010G002700.1	DUF247 domain	integral component of membrane		
Phvul.010G003100.1	lactosylceramide 4-alpha-galactosyltransferase-like	integral component of membrane		
Phvul.010G003200.1	30S ribosomal S31, chloroplastic	ribosome;chloroplast stroma;chloroplast envelope;plastid translation		
Phvul.010G003300.1	DUF247 domain	integral component of membrane		
Phvul.010G004000.1	NRT1 PTR FAMILY -like	integral component of membrane;transporter activity;transport		
Phvul.010G004700.1	disease resistance RPP13 1	ADP binding;defense response		
Phvul.010G004700.2	disease resistance RPP13 1	ADP binding;defense response		
Phvul.010G005100.1	sulfoquinovosyl transferase SQD2	transferase activity, transferring glycosyl groups;metabolic process		
Phvul.010G005700.1	DUF3527 domain			
Phvul.010G005700.2	DUF3527 domain			
Phvul.010G005700.3	DUF3527 domain			
Phvul.010G006700.1	fructokinase-like 1, chloroplastic	nucleus;chloroplast nucleoid;kinase activity;phosphotransferase activity, alcohol group as acceptor;rRNA processing;tRNA metabolic process;chloroplast		

		organization;embryo development ending in seed dormancy;thylakoid membrane organization;vegetative to reproductive phase transition of meristem;iron-sulfur cluster assembly;phosphorylation;transcription from plastid promoter;positive regulation of transcription, DNA-templated;plant ovule development		
Phvul.010G007200.1	hypothetical protein PHAVU_010G007200g			
Phvul.010G007400.1	GDP-fucose O-fucosyltransferase	cytoplasm;integral component of membrane;transferase activity, transferring glycosyl groups;metabolic process		
Phvul.010G009000.1	7-deoxyloganetic acid glucosyltransferase-like	intracellular membrane-bounded organelle;cyanohydrin beta-glucosyltransferase activity;quercetin 3-O-glucosyltransferase activity;quercetin 7-O-glucosyltransferase activity;flavonoid biosynthetic process;flavonoid glucuronidation	ec:2.4.1.85-beta-glucosyltransferase	Cyanoamino acid metabolism
Phvul.010G009400.1	NRT1 PTR FAMILY -like	integral component of membrane;transporter activity;oligopeptide transport		
Phvul.010G009400.2	NRT1 PTR FAMILY -like	integral component of membrane;transporter activity;oligopeptide transport		
Phvul.010G011300.1	NAD(P)-binding rossmann-fold	chloroplast stroma;thylakoid;apoplast;copper ion binding;polysaccharide catabolic process;starch metabolic process;rRNA processing;response to cold;plant-type cell wall organization;salicylic acid biosynthetic process;defense response, incompatible interaction;chlorophyll biosynthetic process;isopentenyl diphosphate biosynthetic process, methylerythritol 4-phosphate pathway;photosynthesis, light reaction		
Phvul.010G012400.1	Mitochondrial import inner membrane translocase subunit Tim9	mitochondrial intermembrane space protein transporter complex;mitochondrial inner membrane protein insertion complex;mitochondrial protein-transporting ATPase activity;metal ion binding;RNA methylation;photorespiration;protein import into mitochondrial inner membrane;chaperone-mediated protein transport	ec:3.6.3.51	
Phvul.010G013800.1	UDP-glucose:sterol 3-O-glucosyltransferase	intracellular membrane-bounded organelle;sterol 3-beta-glucosyltransferase activity;flavonoid biosynthetic process;lipid glycosylation;flavonoid glucuronidation	ec:2.4.1.173	
Phvul.010G014200.1	hypothetical protein PHAVU_010G014200g	chloroplast thylakoid membrane		
Phvul.010G014700.1	Poly ,	integral component of membrane;catalytic activity;binding;cellular metabolic process;primary metabolic process;organic substance metabolic process		
Phvul.010G014900.1	far-red elongated hypocotyl ,			
Phvul.010G016400.1	legume-specific	integral component of membrane		
Phvul.010G016900.1	aldo-keto reductase family 4 member C9-like	alcohol dehydrogenase (NADP+) activity;oxidation-reduction process	ec:1.1.1.2-dehydrogenase (NADP+)	Biosynthesis of antibiotics;Glycolysis / Gluconeogenesis;Glycerolipid metabolism;Caprolactam degradation;Pentose and glucuronate interconversions
Phvul.010G018400.1	disease resistance LAZ5-like isoform X1	ATP binding;ADP binding;defense response;signal transduction		
Phvul.010G018600.1	myosin-binding 7 isoform X1	integral component of membrane		

Phvul.010G018900.1	G-type lectin S-receptor-like Serine Threonine-kinase	plasma membrane;plasmodesma;integral component of membrane;protein serine/threonine kinase activity;ATP binding;carbohydrate binding;ubiquitin protein ligase binding;protein phosphorylation;innate immune response;recognition of pollen	ec:2.7.11	
Phvul.010G018900.2	G-type lectin S-receptor-like Serine Threonine-kinase	plasma membrane;plasmodesma;integral component of membrane;protein serine/threonine kinase activity;ATP binding;carbohydrate binding;ubiquitin protein ligase binding;protein phosphorylation;innate immune response;recognition of pollen	ec:2.7.11	
Phvul.010G019500.1	alkane hydroxylase MAH1-like	integral component of membrane;monooxygenase activity;iron ion binding;oxidoreductase activity, acting on paired donors, with incorporation or reduction of molecular oxygen;heme binding;oxidation-reduction process		
Phvul.010G020000.1	XRI1-like isoform X1			
Phvul.010G020100.1	RNA polymerase I-specific transcription initiation factor RRN3	translation initiation factor activity;translational initiation		
Phvul.010G020200.1	DNA-directed RNA polymerase III subunit RPC8	DNA-directed RNA polymerase III complex;RNA polymerase III activity;hydrolase activity;transcription initiation from RNA polymerase III promoter;lipid metabolic process		
Phvul.010G020300.1	UBX domain-containing 1-like	cytosol;metal ion binding;protein glycosylation;protein targeting to vacuole		
Phvul.010G020700.1	hypothetical protein PHAVU_010G020700g	intracellular;GTP binding;small GTPase mediated signal transduction;protein transport		
Phvul.010G023900.1	probable copper-transporting ATPase HMA5	integral component of plasma membrane;intracellular membrane-bounded organelle;nucleotide binding;copper-exporting ATPase activity;copper ion binding;metabolic process;copper ion export	ec:3.6.3.4	
Phvul.010G026000.1	TMV resistance N-like	ADP binding;defense response;signal transduction		
Phvul.010G026600.1	disease resistance LAZ5-like isoform X1	ATP binding;ADP binding;defense response;signal transduction		
Phvul.010G026700.1	disease resistance LAZ5-like isoform X1	ATP binding;ADP binding;defense response;signal transduction		
Phvul.010G026900.1	TMV resistance N-like isoform X2	ATP binding;ADP binding;defense response;signal transduction		
Phvul.010G027100.1	disease resistance LAZ5-like isoform X1	ATP binding;ADP binding;defense response;signal transduction		
Phvul.010G027200.1	TMV resistance N-like	ATP binding;ADP binding;defense response;signal transduction		
Phvul.010G027600.1	hypothetical protein PHAVU_010G027600g	ATP binding;ADP binding;defense response;signal transduction		
Phvul.010G027800.1	disease resistance LAZ5-like isoform X1	ATP binding;ADP binding;signal transduction		
Phvul.010G027800.2	disease resistance LAZ5-like isoform X1	ATP binding;ADP binding;signal transduction		
Phvul.010G028500.1	disease resistance TAO1-like	ADP binding;defense response;signal transduction		
Phvul.010G028500.2	TMV resistance N-like	ADP binding;defense response;signal transduction		
Phvul.010G028500.3	TMV resistance N-like	ADP binding;defense response;signal transduction		
Phvul.010G028600.1	TMV resistance N-like	ATP binding;ADP binding;defense response;signal transduction		

Phvul.010G029200.1	disease resistance CSA1	ATP binding;ADP binding;defense response;signal transduction		
Phvul.010G029400.1	TMV resistance N-like	ADP binding		
Phvul.010G029600.1	disease resistance LAZ5-like isoform X1	ATP binding;ADP binding;defense response;signal transduction		
Phvul.010G031700.1	Gamma-tubulin complex component 2	equatorial microtubule organizing center;centrosome;spindle pole body;gamma-tubulin small complex;guanylate cyclase activity;structural constituent of cytoskeleton;gamma-tubulin binding;microtubule minus-end binding;cGMP biosynthetic process;meiotic nuclear division;cytoplasmic microtubule organization;centrosome duplication;interphase microtubule nucleation by interphase microtubule organizing center;positive regulation of microtubule nucleation;mitotic spindle assembly	ec:4.6.1.2-cyclase	Purine metabolism
Phvul.010G032700.1	transport SEC23	COPII vesicle coat;zinc ion binding;intracellular protein transport;ER to Golgi vesicle-mediated transport		
Phvul.010G036300.1	ubiquitin-like-conjugating enzyme ATG10	membrane;ubiquitin activating enzyme activity;autophagy;protein ubiquitination		
Phvul.010G039700.1	PREDICTED: uncharacterized protein LOC106774021 isoform X1			
Phvul.010G043700.1	Eukaryotic translation initiation factor 4G	translation initiation factor activity;protein kinase activity;ATP binding;translational initiation;protein phosphorylation		
Phvul.010G044700.1	heterogeneous nuclear ribonucleo	viral nucleocapsid;intracellular ribonucleoprotein complex;nucleotide binding;nucleic acid binding		
Phvul.010G044700.2	heterogeneous nuclear ribonucleo	viral nucleocapsid;intracellular ribonucleoprotein complex;nucleotide binding;nucleic acid binding		
Phvul.010G044700.3	heterogeneous nuclear ribonucleo	viral nucleocapsid;intracellular ribonucleoprotein complex;nucleotide binding;nucleic acid binding		
Phvul.010G045600.1	dol-P-Man:Man(7)c(2)-PP-Dol alpha-1,6-mannosyltransferase	integral component of membrane;alpha-1,6-mannosyltransferase activity;protein N-linked glycosylation;ER-associated ubiquitin-dependent protein catabolic process;mannosylation	ec:2.4.1	
Phvul.010G045600.2	dol-P-Man:Man(7)c(2)-PP-Dol alpha-1,6-mannosyltransferase	integral component of membrane;alpha-1,6-mannosyltransferase activity;protein N-linked glycosylation;ER-associated ubiquitin-dependent protein catabolic process;mannosylation	ec:2.4.1	
Phvul.010G046200.1	pentatricopeptide repeat-containing At1g15510, chloroplastic	zinc ion binding;microtubule-severing ATPase activity;RNA modification;chloroplast organization;growth	ec:3.6.4.3	
Phvul.010G048300.1	pentatricopeptide repeat-containing At4g32430, mitochondrial			
Phvul.010G048700.1	cytochrome P450 family	integral component of membrane;monooxygenase activity;iron ion binding;oxidoreductase activity, acting on paired donors, with incorporation or reduction of molecular oxygen;heme binding;oxidation-reduction process		
Phvul.010G049400.1	glycyl-tRNA synthetase alpha chain beta chain	mitochondrion;chloroplast stroma;glycine-tRNA ligase activity;ATP binding;protein dimerization activity;glycyl-tRNA aminoacylation;chloroplast organization;embryo development ending in seed dormancy;regulation of embryonic development;plant ovule development	ec:6.1.1.14-ligase	Aminoacyl-tRNA biosynthesis

Phvul.010G049800.1	phospholipase A(1) DAD1, chloroplastic-like	phosphatidylcholine 1-acylhydrolase activity;phosphatidylserine 1-acylhydrolase activity;1-acyl-2-lysophosphatidylserine acylhydrolase activity;lipid metabolic process	ec:3.1.1.32-A1	alpha-Linolenic acid metabolism;Glycerophospholipid metabolism
Phvul.010G050000.1	serine threonine-kinase D6PK-like	nucleus;cytoplasm;plasma membrane;protein serine/threonine kinase activity;ATP binding;protein phosphorylation;determination of bilateral symmetry;organ morphogenesis;meristem initiation;xylem and phloem pattern formation;meristem maintenance;xylem development;intracellular signal transduction;cell wall macromolecule metabolic process;flower morphogenesis;negative regulation of biological process	ec:2.7.11	
Phvul.010G054200.2	TMV resistance N-like	ADP binding;defense response;signal transduction		
Phvul.010G056500.1	pentatricopeptide repeat-containing At2g17670	intracellular;protein targeting to mitochondrion		
Phvul.010G057800.1	transmembrane 87B-like	integral component of membrane		
Phvul.010G058700.1	cell wall integrity and stress response component 4-like			
Phvul.010G063700.1	disease resistance At3g14460	ADP binding;defense response		
Phvul.010G065600.1	ferritin-2, chloroplastic	chloroplast;ferroxidase activity;ferric iron binding;iron ion transport;cellular iron ion homeostasis;oxidation-reduction process	ec:1.16.3.1-ceruloplasmin	Porphyrin and chlorophyll metabolism
Phvul.010G068200.1	DETOXIFICATION 53-like	integral component of membrane;drug transmembrane transporter activity;antiporter activity;drug transmembrane transport		
Phvul.010G070600.1	Transcription factor UNE10	protein dimerization activity		
Phvul.010G070600.2	transcription factor UNE10-like	protein dimerization activity		
Phvul.010G070600.3	transcription factor UNE10-like	protein dimerization activity		
Phvul.010G071600.1	Holliday junction resolvase	nucleic acid binding;hydrolase activity;rRNA processing		
Phvul.010G071900.1	Anthocyanidin 3-O-glucosyltransferase 5	intracellular membrane-bounded organelle;coniferyl-alcohol glucosyltransferase activity;quercetin 3-O-glucosyltransferase activity;quercetin 7-O-glucosyltransferase activity;flavonoid biosynthetic process;flavonoid glucuronidation	ec:2.4.1.111-glucosyltransferase	Phenylpropanoid biosynthesis
Phvul.010G072500.1	EF-hand pair	calcium ion binding		
Phvul.010G072500.2	EF-hand pair	calcium ion binding		
Phvul.010G072500.3	EF-hand pair	calcium ion binding		
Phvul.010G072700.1	Scarecrow 6	transcription, DNA-templated;regulation of transcription, DNA-templated		
Phvul.010G072900.1	scarecrow 6	transcription, DNA-templated;regulation of transcription, DNA-templated		
Phvul.010G073200.1	transcription factor bHLH140	DNA binding;DNA 5'-adenosine monophosphate hydrolase activity;DNA repair;DNA recombination;meiosis I;regulation of nucleobase-containing compound metabolic process;regulation of chromosome organization;xylan metabolic process;chromosome organization;regulation of macromolecule metabolic process;regulation of biological quality;nucleic acid phosphodiester bond hydrolysis;nuclear chromosome segregation		
Phvul.010G073300.1	receptor 12	integral component of membrane;transferase activity;metabolic process		
Phvul.010G073800.1	laccase-4-like	apoplast;copper ion binding;L-ascorbate oxidase activity;oxidoreductase activity, oxidizing metal ions;hydroquinone:oxygen oxidoreductase activity;lignin biosynthetic process;glucuronoxylan metabolic	ec:1.10.3.3-oxidase;ec:1.10.3.2	Ascorbate and aldarate metabolism

		process;xylan biosynthetic process;lignin catabolic process;oxidation-reduction process		
Phvul.010G081900.1	pentatricopeptide repeat-containing At2g22410, mitochondrial	nucleic acid binding;zinc ion binding;protein methyltransferase activity;protein methylation	ec:2.1.1	
Phvul.010G081900.2	pentatricopeptide repeat-containing At2g22410, mitochondrial	nucleic acid binding;zinc ion binding;protein methyltransferase activity;protein methylation	ec:2.1.1	
Phvul.010G084900.1	LOB domain-containing 19			
Phvul.010G086700.1	Poly(A) RNA polymerase cid11	RNA uridylyltransferase activity;nuclear-transcribed mRNA catabolic process;protein N-linked glycosylation;photoperiodism, flowering	ec:2.7.7.52	
Phvul.010G089100.1	transcription factor TCP9	integral component of membrane;biological regulation		
Phvul.010G091000.1	F-box PP2-A13	carbohydrate binding;response to sucrose;response to fructose		
Phvul.010G092500.1	E3 ubiquitin- ligase ATL59	integral component of membrane;zinc ion binding;ligase activity;metabolic process		
Phvul.010G093900.1	methionine adenosyltransferase 2 subunit beta	methionine adenosyltransferase activity;metabolic process	ec:2.5.1.6-adenosyltransferase	Cysteine and methionine metabolism
Phvul.010G094300.1	hypothetical protein PHAVU_010G094300g			
Phvul.010G094300.2	hypothetical protein PHAVU_010G094300g			
Phvul.010G094400.1	zinc finger C-x8-C-x5-C-x3-H type	metal ion binding		
Phvul.010G096400.1	myb transcription factor MIXTA	DNA binding;regulation of biological process		
Phvul.010G097800.1	MATH domain and coiled-coil domain-containing At3g58250-like			
Phvul.010G098500.1	transcription factor EGL1 isoform X1	protein dimerization activity		
Phvul.010G098500.2	transcription factor EGL1 isoform X2	protein dimerization activity		
Phvul.010G099200.1	phosphatidylinositol N-acetyglucosaminlytransferase subunit P			
Phvul.010G099500.1	signal peptide peptidase-like 4	integral component of membrane;aspartic-type endopeptidase activity;proteolysis	ec:3.4.23	
Phvul.010G100100.1	ATP-dependent helicase BRM-like	nucleus;helicase activity;ATP binding;DNA-dependent ATPase activity;transferase activity;regulation of transcription, DNA-templated;metabolic process;regulation of gene expression, epigenetic;ATP-dependent chromatin remodeling		
Phvul.010G101500.1	ferric reduction oxidase 2-like	integral component of membrane;oxidoreductase activity;oxidation-reduction process		
Phvul.010G102500.1	N6-adenosine-methyltransferase MT-A70-like	chloroplast;nuclear speck;mRNA (2'-O-methyladenosine-N6-)-methyltransferase activity;mRNA methylation	ec:2.1.1.62	
Phvul.010G103800.1	FK506-binding 4	cytoplasm;transition metal ion binding;metal ion transport;cellular transition metal ion homeostasis		
Phvul.010G104900.1	3-methyl-2-oxobutanoate hydroxymethyltransferase 1, mitochondrial-like	3-methyl-2-oxobutanoate hydroxymethyltransferase activity;methyltransferase activity;pantothenate biosynthetic process;methylation	ec:2.1.2.11-hydroxymethyltransferase	Pantothenate and CoA biosynthesis
Phvul.010G105900.1	group 1 family glycosyltransferase	endosome;trans-Golgi network;integral component of membrane;transferase activity;response to hypoxia;galactolipid biosynthetic process		

Phvul.010G107600.1	dihydroorotase, mitochondrial-like	chloroplast;dihydroorotase activity;pyrimidine nucleobase biosynthetic process	ec:3.5.2.3-carbamoylaspartic dehydrase	Pyrimidine metabolism
Phvul.010G107600.2	dihydroorotase, mitochondrial-like	chloroplast;dihydroorotase activity;pyrimidine nucleobase biosynthetic process	ec:3.5.2.3-carbamoylaspartic dehydrase	Pyrimidine metabolism
Phvul.010G107700.1	F-box kelch-repeat At3g61590	ubiquitin-protein transferase activity;lipid transport;protein ubiquitination;animal organ development		
Phvul.010G107700.2	F-box kelch-repeat At3g61590	ubiquitin-protein transferase activity;lipid transport;protein ubiquitination;animal organ development		
Phvul.010G109100.1	hypothetical protein PHAVU_010G109100g	DNA binding;protein dimerization activity		
Phvul.010G110700.1	Proline-, glutamic acid- and leucine-rich 1			
Phvul.010G110800.1	mediator of RNA polymerase II transcription subunit 19a-like			
Phvul.010G112300.1	dentin sialophospho			
Phvul.010G112700.1	Poly(A) RNA polymerase cid14	DNA-directed DNA polymerase activity;DNA biosynthetic process	ec:2.7.7.7-DNA polymerase	Purine metabolism;Pyrimidine metabolism
Phvul.010G114600.1	Embryo defective 1703, isoform 1	integral component of membrane		
Phvul.010G115100.1	kinesin light chain			
Phvul.010G115800.1	probable serine threonine- kinase WNK10 isoform X1	protein kinase activity;ATP binding;protein phosphorylation		
Phvul.010G116400.1	U-box domain-containing 44-like	ubiquitin-protein transferase activity;ligase activity;protein ubiquitination		
Phvul.010G117400.1	auxin-responsive family	response to auxin		
Phvul.010G118300.1	CBL-interacting serine threonine-kinase 9	cytoplasm;protein serine/threonine kinase activity;ATP binding;protein phosphorylation;intracellular signal transduction	ec:2.7.11	
Phvul.010G118400.1	probable L-gulonolactone oxidase 6	membrane;D-arabinono-1,4-lactone oxidase activity;L-gulonolactone oxidase activity;flavin adenine dinucleotide binding;oxidation-reduction process	ec:1.1.3.37;ec:1.1.3.8-oxidase	Ascorbate and aldarate metabolism
Phvul.010G119300.1	Granule-bound starch synthase 1, chloroplastic amyloplastic	amyloplast;chloroplast;glycogen (starch) synthase activity;response to hypoxia;myo-inositol hexakisphosphate biosynthetic process;starch biosynthetic process;galactolipid biosynthetic process	ec:2.4.1.11-synthase	Starch and sucrose metabolism
Phvul.010G120800.1	Soluble inorganic pyrophosphatase	nucleus;cytosol;membrane;magnesium ion binding;inorganic diphosphatase activity;RNA splicing, via endonucleolytic cleavage and ligation;phosphate-containing compound metabolic process;methionine biosynthetic process;lipid storage	ec:3.6.1.1-diphosphatase	Oxidative phosphorylation
Phvul.010G121700.1	transcription factor 25	cytosol		
Phvul.010G122100.1	STRUBBELIG-RECEPTOR FAMILY 6	integral component of membrane;protein kinase activity;ATP binding;protein phosphorylation		
Phvul.010G122300.1	K+-H+ exchange	mitochondrion;vacuolar membrane;plastid;integral component of membrane		
Phvul.010G123900.1	AT-rich interactive domain-containing 4-like	DNA binding;zinc ion binding		
Phvul.010G124800.1	GDSL esterase lipase 5-like	lipase activity;9,9'-dicis-carotene:quinone oxidoreductase activity;7,9,9'-tricis-neurosporene:quinone oxidoreductase activity;proline transport;9,9'-di-cis-zeta-carotene desaturation to 7,9,7',9'-tetra-cis-lycopene;oxidation-reduction process	ec:3.1.1;ec:1.3.5.6-desaturase	Carotenoid biosynthesis

Phvul.010G126600.1	auxin-repressed kDa isoform X1	membrane		
Phvul.010G126700.1	chaperone domain			
Phvul.010G127600.1	exocyst complex component EXO70A1-like	exocyst;exocytosis		
Phvul.010G131900.1	disease resistance RML1A-like	integral component of membrane;ADP binding;defense response;signal transduction		
Phvul.010G132100.1	hypothetical protein PHAVU_010G132100g			
Phvul.010G132200.1	disease resistance RML1A-like	integral component of membrane;ADP binding;defense response;signal transduction		
Phvul.010G132900.1	phospholipid:diacylglycerol acyltransferase 1-like	vacuole;endoplasmic reticulum;integral component of membrane;O-acyltransferase activity;phospholipid:diacylglycerol acyltransferase activity;lipid metabolic process	ec:2.3.1.158-acyltransferase;ec:2.3.1	Glycerolipid metabolism
Phvul.010G133700.1	hypothetical protein PHAVU_010G133700g	integral component of membrane		
Phvul.010G136000.1	telomere repeat-binding factor 4-like	nucleosome;nucleus;DNA binding;nucleosome assembly		
Phvul.010G136600.1	probable phospholipid-transporting ATPase 4 isoform X1	Golgi membrane;trans-Golgi network;plasma membrane;integral component of membrane;magnesium ion binding;phospholipid-translocating ATPase activity;ATP binding;metabolic process;phospholipid translocation;Golgi vesicle budding	ec:3.6.3.1	
Phvul.010G136600.2	probable phospholipid-transporting ATPase 4 isoform X1	Golgi membrane;trans-Golgi network;plasma membrane;integral component of membrane;magnesium ion binding;phospholipid-translocating ATPase activity;ATP binding;metabolic process;phospholipid translocation;Golgi vesicle budding	ec:3.6.3.1	
Phvul.010G138000.1	F-box RNI superfamily			
Phvul.010G138200.1	indeterminate-domain 11-like	nucleic acid binding;metal ion binding		
Phvul.010G139100.1	ROOT HAIR DEFECTIVE 3-like	endoplasmic reticulum membrane;integral component of membrane;GTPase activity;GTP binding;metabolic process		
Phvul.010G139600.1	cytochrome P450 family 90	integral component of membrane;iron ion binding;oxidoreductase activity, acting on paired donors, with incorporation or reduction of molecular oxygen, NAD(P)H as one donor, and incorporation of one atom of oxygen;heme binding;brassinosteroid homeostasis;sterol metabolic process;brassinosteroid biosynthetic process;leaf development;petal development;stamen development;oxidation-reduction process	ec:1.14.13	
Phvul.010G139800.1	phospholipase D alpha	membrane;phospholipase D activity;calcium ion binding;N-acylphosphatidylethanolamine-specific phospholipase D activity;lipid catabolic process;phosphatidylcholine metabolic process	ec:3.1.4.4-D	Ether lipid metabolism;Glycerophospholipid metabolism
Phvul.010G139800.2	phospholipase D alpha	membrane;phospholipase D activity;calcium ion binding;N-acylphosphatidylethanolamine-specific phospholipase D activity;lipid catabolic process;phosphatidylcholine metabolic process	ec:3.1.4.4-D	Ether lipid metabolism;Glycerophospholipid metabolism
Phvul.010G140200.1	inactive kinase SELMODRAFT_444075	non-membrane spanning protein tyrosine kinase activity;ATP binding;hydrolase activity;peptidyl-tyrosine phosphorylation	ec:2.7.10.2-protein-tyrosine kinase	T cell receptor signaling pathway
Phvul.010G141800.1	dentin sialophospho			
Phvul.010G142900.1	EARLY FLOWERING 3-like isoform X1	regulation of transcription, DNA-templated		
Phvul.010G142900.2	EARLY FLOWERING 3-like isoform X1			
Phvul.010G142900.3	EARLY FLOWERING 3-like isoform X1	regulation of transcription, DNA-templated		

Phvul.010G142900.4	EARLY FLOWERING 3-like isoform X1			
Phvul.010G142900.5	EARLY FLOWERING 3-like isoform X1	regulation of transcription, DNA-templated		
Phvul.010G142900.6	EARLY FLOWERING 3-like isoform X1	regulation of transcription, DNA-templated		
Phvul.010G143200.1	glucose-6-phosphate 1-dehydrogenase, chloroplastic-like	chloroplast stroma;glucose-6-phosphate dehydrogenase activity;NADP binding;glucose metabolic process;pentose-phosphate shunt, oxidative branch;response to blue light;regulation of proton transport;photosynthesis;isopentenyl diphosphate biosynthetic process, methylerythritol 4-phosphate pathway;protein autophosphorylation;oxidation-reduction process	ec:1.1.1.49-dehydrogenase (NADP+)	Biosynthesis of antibiotics;Pentose phosphate pathway;Glutathione metabolism
Phvul.010G143400.1	pentatricopeptide repeat-containing At4g26680, mitochondrial			
Phvul.010G144400.1	stress response NST1-like			
Phvul.010G144400.2	stress response NST1-like			
Phvul.010G144800.1	nuclear poly(A) polymerase 4-like isoform X2	nucleus;RNA binding;polynucleotide adenylyltransferase activity;mRNA polyadenylation	ec:2.7.7.19	
Phvul.010G145100.1	lysM domain receptor-like kinase 3 isoform X1	integral component of membrane;protein serine/threonine kinase activity;ATP binding;protein phosphorylation;microtubule nucleation	ec:2.7.11	
Phvul.010G145900.1	Casein kinase I-2	protein serine/threonine kinase activity;ATP binding;Wnt signaling pathway;peptidyl-serine phosphorylation	ec:2.7.11	
Phvul.010G147600.1	transcription factor bHLH68-like isoform X1	nucleus;core promoter sequence-specific DNA binding;transcriptional activator activity, RNA polymerase II transcription regulatory region sequence-specific binding;protein dimerization activity;transcription from RNA polymerase II promoter;positive regulation of transcription from RNA polymerase II promoter		
Phvul.010G147900.1	NRT1 PTR FAMILY -like	integral component of membrane;transporter activity;oligopeptide transport		
Phvul.010G148100.1	Thioesterase superfamily ,			
Phvul.010G149300.1	proline-rich receptor kinase PERK1	integral component of membrane;protein serine/threonine kinase activity;structural constituent of cell wall;ATP binding;protein phosphorylation	ec:2.7.11	
Phvul.010G149400.1	sorting nexin carboxy-terminal	integral component of membrane;transferase activity;phosphatidylinositol binding;metabolic process		
Phvul.010G149400.2	sorting nexin carboxy-terminal	integral component of membrane;transferase activity;phosphatidylinositol binding;metabolic process		
Phvul.010G151300.1	stress response NST1-like	cytoplasm;transition metal ion binding;metal ion transport;cellular transition metal ion homeostasis		
Phvul.010G151600.1	N-alpha-acetyltransferase 15, auxiliary subunit-like	cytoplasm;peptide alpha-N-acetyltransferase activity;N-terminal peptidyl-methionine acetylation	ec:2.3.1.88	
Phvul.010G152600.1	DUF2921 family	endosome;trans-Golgi network;integral component of membrane		
Phvul.010G152600.2	DUF2921 family	endosome;trans-Golgi network;integral component of membrane		
Phvul.010G154600.1	DUF246 domain-containing	cytoplasm;transferase activity, transferring glycosyl groups;metabolic process		
Phvul.010G155600.1	TPR repeat			
Phvul.010G157200.1	clustered mitochondria homolog	cytosol;translation initiation factor activity;translational initiation		

Phvul.010G157200.2	clustered mitochondria homolog	cytosol;translation initiation factor activity;translational initiation		
Phvul.010G157900.1	mediator of RNA polymerase II transcription subunit 15a-like isoform X1	transcription cofactor activity;regulation of transcription, DNA-templated		
Phvul.010G160600.1	DNA mismatch repair Msh6-1			
Phvul.010G160600.2	DNA mismatch repair Msh6-1			
Phvul.011G000600.1	TVP38 TMEM64 family membrane slr0305	chloroplast;integral component of membrane;myo-inositol hexakisphosphate biosynthetic process		
Phvul.011G000800.1	FKBP-type peptidyl-prolyl cis-trans isomerase	endoplasmic reticulum membrane;peptidyl-prolyl cis-trans isomerase activity;FK506 binding;protein peptidyl-prolyl isomerization;single-organism process;chaperone-mediated protein folding	ec:5.2.1.8	
Phvul.011G000800.2	FKBP-type peptidyl-prolyl cis-trans isomerase	endoplasmic reticulum membrane;peptidyl-prolyl cis-trans isomerase activity;FK506 binding;protein peptidyl-prolyl isomerization;single-organism process;chaperone-mediated protein folding	ec:5.2.1.8	
Phvul.011G001200.1	phosphoinositide phosphatase SAC1	mannosyl-oligosaccharide 1,2-alpha-mannosidase activity;phosphatidylinositol-3,5-bisphosphate 5-phosphatase activity;protein N-linked glycosylation;cell adhesion;unidimensional cell growth;plant-type cell wall biogenesis;trichome morphogenesis;glucuronoxylan metabolic process;inositol trisphosphate metabolic process;phosphatidylinositol-3-phosphate biosynthetic process;actin nucleation;xylan biosynthetic process;inositol phosphate dephosphorylation;root hair cell differentiation;cell wall organization	ec:3.1.3;ec:3.2.1.113-1,2-alpha-mannosidase	Various types of N-glycan biosynthesis;N-Glycan biosynthesis
Phvul.011G002700.1	shoot gravitropism 2 (SGR2)	plasmodesma;plant-type vacuole membrane;phosphatidylcholine 1-acylhydrolase activity;metal ion binding;metabolic process;detection of gravity;amyloplast organization;negative gravitropism	ec:3.1.1.32-A1	alpha-Linolenic acid metabolism;Glycerophospholipid metabolism
Phvul.011G002700.2	shoot gravitropism 2 (SGR2)	plasmodesma;plant-type vacuole membrane;phosphatidylcholine 1-acylhydrolase activity;metal ion binding;metabolic process;detection of gravity;amyloplast organization;negative gravitropism	ec:3.1.1.32-A1	alpha-Linolenic acid metabolism;Glycerophospholipid metabolism
Phvul.011G003700.1	hypothetical protein PHAVU_011G003700g			
Phvul.011G005800.1	MADS-box transcription factor	nucleus;DNA binding;transcription factor activity, sequence-specific DNA binding;protein dimerization activity;transcription, DNA-templated;regulation of transcription, DNA-templated;iron ion transport;cellular response to iron ion starvation;response to nitrate;vegetative to reproductive phase transition of meristem;nitrate transport;plant ovule development;lateral root development;developmental growth;root hair cell differentiation		
Phvul.011G006200.1	COP9 signalosome complex subunit 4	proteasome complex;cytosol;COP9 signalosome;NEDD8-specific protease activity;photomorphogenesis;cullin deneddylation;positive regulation of G2/M transition of mitotic cell cycle		
Phvul.011G009600.1	dentin sialophospho-like			
Phvul.011G009800.1	tRNA-dihydrouridine(47) synthase	nucleus;cytosol;phragmoplast;tRNA dihydrouridine synthase activity;flavin adenine dinucleotide binding;tRNA dihydrouridine synthesis;methylglyoxal catabolic process to D-		

	[NAD(P)(+)]-like isoform X1	lactate via S-lactoyl-glutathione;oxidation-reduction process		
Phvul.011G011000.1	RETICULATA-RELATED 4, chloroplastic-like	mitochondrion;chloroplast inner membrane;integral component of membrane		
Phvul.011G011400.1	hypothetical protein PHAVU_011G0114000g, partial	DNA binding		
Phvul.011G012000.1	aspartokinase-homoserine dehydrogenase	chloroplast;aspartate kinase activity;homoserine dehydrogenase activity;amino acid binding;NADP binding;aspartate family amino acid biosynthetic process;phosphorylation;oxidation-reduction process	ec:1.1.1.3-dehydrogenase;ec:2.7.2.4-kinase	Biosynthesis of antibiotics;Cysteine and methionine metabolism;Lysine biosynthesis;Glycine, serine and threonine metabolismBiosynthesis of antibiotics;Cysteine and methionine metabolism;Lysine biosynthesis;Monobactam biosynthesis;Glycine, serine and threonine metabolism
Phvul.011G012000.2	aspartokinase-homoserine dehydrogenase	chloroplast;aspartate kinase activity;homoserine dehydrogenase activity;amino acid binding;NADP binding;aspartate family amino acid biosynthetic process;phosphorylation;oxidation-reduction process	ec:1.1.1.3-dehydrogenase;ec:2.7.2.4-kinase	Biosynthesis of antibiotics;Cysteine and methionine metabolism;Lysine biosynthesis;Glycine, serine and threonine metabolismBiosynthesis of antibiotics;Cysteine and methionine metabolism;Lysine biosynthesis;Monobactam biosynthesis;Glycine, serine and threonine metabolism
Phvul.011G012700.1	OBERON 4	cell fate specification;embryonic pattern specification;embryonic meristem initiation		
Phvul.011G016300.1	glycerol-3-phosphate transporter 1	integral component of membrane;transmembrane transporter activity;anion transport;transmembrane transport		
Phvul.011G016600.1	DNA topoisomerase 1-like	nucleolus;replication fork protection complex;DNA binding;DNA topoisomerase type I activity;DNA topoisomerase type II (ATP-hydrolyzing) activity;2-alkenal reductase [NAD(P)] activity;DNA replication;DNA topological change;chromatin remodeling;chromosome segregation;oxidation-reduction process	ec:5.99.1.3;ec:5.99.1.2;ec:1.3.1.74	
Phvul.011G016600.2	DNA topoisomerase 1-like	nucleolus;replication fork protection complex;DNA binding;DNA topoisomerase type I activity;DNA topoisomerase type II (ATP-hydrolyzing) activity;2-alkenal reductase [NAD(P)] activity;DNA replication;DNA topological change;chromatin remodeling;chromosome segregation;oxidation-reduction process	ec:5.99.1.3;ec:5.99.1.2;ec:1.3.1.74	
Phvul.011G016900.1	bromodomain-containing DDB_G0280777			
Phvul.011G017900.1	Bromodomain adjacent to zinc finger domain 1A	zinc ion binding;oxidoreductase activity;oxidation-reduction process		
Phvul.011G017900.2	RING FYVE PHD zinc finger ,	zinc ion binding;oxidoreductase activity;oxidation-reduction process		
Phvul.011G017900.3	RING FYVE PHD zinc finger ,	zinc ion binding;oxidoreductase activity;oxidation-reduction process		
Phvul.011G017900.4	Bromodomain adjacent to zinc finger domain 1A	zinc ion binding;oxidoreductase activity;oxidation-reduction process		

Phvul.011G017900.5	Bromodomain adjacent to zinc finger domain 1A	zinc ion binding;oxidoreductase activity;oxidation-reduction process		
Phvul.011G017900.6	Bromodomain adjacent to zinc finger domain 1A	zinc ion binding;oxidoreductase activity;oxidation-reduction process		
Phvul.011G017900.7	Bromodomain adjacent to zinc finger domain 1A	zinc ion binding;oxidoreductase activity;oxidation-reduction process		
Phvul.011G017900.8	RING FYVE PHD zinc finger ,	zinc ion binding;oxidoreductase activity;oxidation-reduction process		
Phvul.011G017900.9	Bromodomain adjacent to zinc finger domain 1A	zinc ion binding;oxidoreductase activity;oxidation-reduction process		
Phvul.011G018800.1	impaired sucrose induction ,	regulation of carbohydrate metabolic process		
Phvul.011G019900.1	probable arabinosyltransferase ARAD1	Golgi apparatus;integral component of membrane;glucuronosyl-N-acetylglucosaminyl-proteoglycan 4-alpha-N-acetylglucosaminyltransferase activity;metabolic process	ec:2.4.1.224-4-alpha-N-acetylglucosaminyltransferase	Glycosaminoglycan biosynthesis - heparan sulfate / heparin
Phvul.011G020800.1	probable methionine--tRNA ligase	cytoplasm;tRNA binding;methionine-tRNA ligase activity;ATP binding;methionyl-tRNA aminoacylation	ec:6.1.1.10-ligase	Aminoacyl-tRNA biosynthesis;Selenocompound metabolism
Phvul.011G021900.1	myosin-related family			
Phvul.011G023300.1	biotin synthase	biotin synthase activity;zinc ion binding;2 iron, 2 sulfur cluster binding;4 iron, 4 sulfur cluster binding;acetyl-CoA metabolic process;microtubule nucleation;biotin biosynthetic process;regulation of meristem growth;sterol biosynthetic process;brassinosteroid biosynthetic process;anther development	ec:2.8.1.6-synthase	Biotin metabolism
Phvul.011G023300.2	biotin synthase	integral component of membrane;biotin synthase activity;zinc ion binding;2 iron, 2 sulfur cluster binding;4 iron, 4 sulfur cluster binding;acetyl-CoA metabolic process;microtubule nucleation;biotin biosynthetic process;regulation of meristem growth;sterol biosynthetic process;brassinosteroid biosynthetic process;anther development	ec:2.8.1.6-synthase	Biotin metabolism
Phvul.011G023500.1	hypothetical protein PHAVU_011G023500g			
Phvul.011G023900.1	Nucleosome-remodeling factor subunit BPTF	zinc ion binding		
Phvul.011G023900.2	Nucleosome-remodeling factor subunit BPTF	zinc ion binding		
Phvul.011G024200.1	probable S-acyltransferase 19	integral component of membrane;zinc ion binding;protein-cysteine S-palmitoyltransferase activity;metabolic process	ec:2.3.1.225	
Phvul.011G024200.2	probable S-acyltransferase 19	integral component of membrane;zinc ion binding;protein-cysteine S-palmitoyltransferase activity;metabolic process	ec:2.3.1.225	
Phvul.011G025300.1	signal recognition particle 43 kDa , chloroplastic	chloroplast thylakoid membrane;chloroplast envelope;signal recognition particle, chloroplast targeting;identical protein binding;response to blue light;response to sucrose;response to red light;regulation of proton transport;response to far red light;cysteine biosynthetic process;protein import into chloroplast thylakoid membrane;protein autophosphorylation		
Phvul.011G026500.1	Transcription factor bHLH121	intracellular;transcription factor activity, sequence-specific DNA binding;protein dimerization activity;regulation of transcription, DNA-templated;fatty acid beta-oxidation;protein import into peroxisome matrix		

Phvul.011G026800.1	Nucleoporin NUP188 like	nuclear pore inner ring;structural constituent of nuclear pore;RNA export from nucleus;protein import into nucleus		
Phvul.011G029000.1	Cytochrome P450, family 86, subfamily B, polypeptide 1 isoform 1	integral component of membrane;iron ion binding;heme binding;aromatase activity;oxidation-reduction process	ec:1.14.14.1-monooxygenase	Linoleic acid metabolism;Arachidonic acid metabolism;Drug metabolism - cytochrome P450;Metabolism of xenobiotics by cytochrome P450;Caffeine metabolism;Aminobenzoate degradation;Tryptophan metabolism;Fatty acid degradation;Retinol metabolism;Steroid hormone biosynthesis
Phvul.011G029100.1	RNI superfamily	nucleotide binding;DNA binding;DNA-directed DNA polymerase activity;3'-5' exonuclease activity;DNA replication;DNA biosynthetic process;nucleic acid phosphodiester bond hydrolysis	ec:2.7.7.7-DNA polymerase	Purine metabolism;Pyrimidine metabolism
Phvul.011G029200.1	hypothetical protein PHAVU_011G029200g	integral component of membrane		
Phvul.011G029800.1	photosystem II stability assembly factor HCF136, chloroplastic	chloroplast stromal thylakoid;chloroplast envelope;thylakoid lumen;pentose-phosphate shunt;rRNA processing;response to blue light;chloroplast relocation;thylakoid membrane organization;response to red light;photosystem II assembly;response to far red light;PSII associated light-harvesting complex II catabolic process;chlorophyll biosynthetic process;carotenoid biosynthetic process;regulation of protein dephosphorylation;hydrogen peroxide catabolic process;transcription from plastid promoter;positive regulation of transcription, DNA-templated		
Phvul.011G029800.2	photosystem II stability assembly factor HCF136, chloroplastic	chloroplast stromal thylakoid;chloroplast envelope;thylakoid lumen;pentose-phosphate shunt;rRNA processing;response to blue light;chloroplast relocation;thylakoid membrane organization;response to red light;photosystem II assembly;response to far red light;PSII associated light-harvesting complex II catabolic process;chlorophyll biosynthetic process;carotenoid biosynthetic process;regulation of protein dephosphorylation;hydrogen peroxide catabolic process;transcription from plastid promoter;positive regulation of transcription, DNA-templated		
Phvul.011G030700.1	hypothetical protein PHAVU_011G030700g	ATP binding;microtubule-severing ATPase activity;metabolic process	ec:3.6.4.3	
Phvul.011G030700.2	hypothetical protein PHAVU_011G030700g	ATP binding;microtubule-severing ATPase activity;metabolic process	ec:3.6.4.3	
Phvul.011G031400.1	DPH4 homolog			
Phvul.011G031400.2	DPH4 homolog			
Phvul.011G032300.1	DUF3067 family	chloroplast		
Phvul.011G032700.1	hypothetical protein PHAVU_011G032700g			

Phvul.011G034700.1	subtilisin-like protease	cell wall;membrane;serine-type endopeptidase activity;sulfur amino acid metabolic process;polysaccharide catabolic process;starch metabolic process;proteolysis;microtubule nucleation;cellular amino acid biosynthetic process;serine family amino acid metabolic process;plant-type cell wall modification;plant-type cell wall biogenesis;regulation of meristem growth;glucosinolate biosynthetic process	ec:3.4.21	
Phvul.011G035600.1	Kinase with adenine nucleotide alpha hydrolases-like domain, isoform 1	non-membrane spanning protein tyrosine kinase activity;ATP binding;hydrolase activity;peptidyl-tyrosine phosphorylation	ec:2.7.10.2-protein-tyrosine kinase	T cell receptor signaling pathway
Phvul.011G035600.2	Kinase with adenine nucleotide alpha hydrolases-like domain, isoform 1	non-membrane spanning protein tyrosine kinase activity;ATP binding;hydrolase activity;peptidyl-tyrosine phosphorylation	ec:2.7.10.2-protein-tyrosine kinase	T cell receptor signaling pathway
Phvul.011G037800.1	transcription initiation factor TFIID subunit 14b-like	regulation of transcription, DNA-templated;vegetative to reproductive phase transition of meristem;regulation of timing of transition from vegetative to reproductive phase;regulation of histone H4 acetylation		
Phvul.011G039100.1	fructose-bisphosphate aldolase 1, chloroplastic	fructose-bisphosphate aldolase activity;glycolytic process	ec:4.1.2.13-aldolase	Biosynthesis of antibiotics;Pentose phosphate pathway;Methane metabolism;Carbon fixation in photosynthetic organisms;Glycolysis / Gluconeogenesis;Fructose and mannose metabolism
Phvul.011G039100.2	fructose-bisphosphate aldolase 1, chloroplastic	fructose-bisphosphate aldolase activity;glycolytic process	ec:4.1.2.13-aldolase	Biosynthesis of antibiotics;Pentose phosphate pathway;Methane metabolism;Carbon fixation in photosynthetic organisms;Glycolysis / Gluconeogenesis;Fructose and mannose metabolism
Phvul.011G040300.1	MATE efflux family 4, chloroplastic	chloroplast envelope;integral component of membrane;drug transmembrane transporter activity;antiporter activity;drug transmembrane transport		
Phvul.011G040700.1	kinesin motor catalytic domain	kinesin complex;microtubule;microtubule motor activity;ATP binding;microtubule binding;zinc ion binding;ATPase activity;microtubule-based movement;metabolic process	ec:3.6.1.3-adenylpyrophosphatase	Purine metabolism
Phvul.011G040700.2	kinesin motor catalytic domain	kinesin complex;microtubule;microtubule motor activity;ATP binding;microtubule binding;zinc ion binding;ATPase activity;microtubule-based movement;metabolic process	ec:3.6.1.3-adenylpyrophosphatase	Purine metabolism
Phvul.011G040700.3	kinesin-related 11-like isoform X2	kinesin complex;microtubule;microtubule motor activity;ATP binding;microtubule binding;zinc ion binding;ATPase activity;microtubule-based movement;metabolic process	ec:3.6.1.3-adenylpyrophosphatase	Purine metabolism
Phvul.011G040800.1	salt tolerance	nucleus;transcription factor activity, transcription factor binding;zinc ion binding;regulation of transcription, DNA-templated;photomorphogenesis		
Phvul.011G040800.2	salt tolerance	nucleus;transcription factor activity, transcription factor binding;zinc ion binding;regulation of transcription, DNA-templated;photomorphogenesis		
Phvul.011G040800.3	salt tolerance	nucleus;transcription factor activity, transcription factor binding;zinc ion binding;regulation of transcription, DNA-templated;photomorphogenesis		

Phvul.011G046200.1	SWI SNF complex subunit SWI3D	nucleus;DNA binding;zinc ion binding		
Phvul.011G046300.1	BURP domain-containing 3-like	protein storage vacuole;seed development		
Phvul.011G049000.1	pentatricopeptide repeat-containing At2g15980			
Phvul.011G050100.1	extra-large guanine nucleotide-binding 1-like	GTPase activity;signal transducer activity;GTP binding;G-protein beta/gamma-subunit complex binding;G-protein coupled receptor signaling pathway;metabolic process;response to abiotic stimulus;response to abscisic acid;response to hexose;regulation of defense response;macromolecule localization;regulation of programmed cell death;innate immune response;negative regulation of biological process;cellular response to acid chemical;cellular response to organic substance;defense response to other organism;cellular response to oxygen-containing compound;single-organism intracellular transport		
Phvul.011G050100.2	extra-large GTP-binding	intracellular;GTPase activity;signal transducer activity;GTP binding;G-protein beta/gamma-subunit complex binding;protein targeting to membrane;G-protein coupled receptor signaling pathway;response to water deprivation;response to wounding;systemic acquired resistance;salicylic acid biosynthetic process;response to ethylene;response to auxin;abscisic acid-activated signaling pathway;response to sucrose;response to glucose;response to fructose;response to jasmonic acid;salicylic acid mediated signaling pathway;regulation of plant-type hypersensitive response;response to mannitol;endoplasmic reticulum unfolded protein response;negative regulation of defense response;hyperosmotic salinity response;defense response to bacterium;negative regulation of programmed cell death;Golgi vesicle transport;lateral root development;defense response by callose deposition;regulation of root morphogenesis		
Phvul.011G050100.3	extra-large guanine nucleotide-binding 1-like	GTPase activity;signal transducer activity;GTP binding;G-protein beta/gamma-subunit complex binding;response to acid chemical;defense response;G-protein coupled receptor signaling pathway;metabolic process;response to hormone;response to carbohydrate		
Phvul.011G050100.4	extra-large guanine nucleotide-binding 1-like	GTPase activity;signal transducer activity;GTP binding;G-protein beta/gamma-subunit complex binding;response to acid chemical;defense response;G-protein coupled receptor signaling pathway;metabolic process;response to hormone;response to carbohydrate;localization;cellular response to organic substance		
Phvul.011G050300.1	serine threonine-kinase SMG1-like	protein serine/threonine kinase activity;ATP binding;nuclear-transcribed mRNA catabolic process, nonsense-mediated decay;DNA repair;protein phosphorylation	ec:2.7.11	
Phvul.011G050300.2	serine threonine-kinase SMG1-like	protein serine/threonine kinase activity;ATP binding;nuclear-transcribed mRNA catabolic process, nonsense-mediated decay;DNA repair;protein phosphorylation	ec:2.7.11	
Phvul.011G050600.1	B3 domain-containing transcription factor VRN1	nucleus;DNA binding;transcription, DNA-templated;regulation of transcription, DNA-templated		
Phvul.011G050600.2	B3 domain-containing transcription factor VRN1-like isoform X2	nucleus;DNA binding;transcription, DNA-templated;regulation of transcription, DNA-templated		

Phvul.011G051400.1	orn lys arg decarboxylase major region	lysine decarboxylase activity;metabolic process	ec:4.1.1.18-decarboxylase	Lysine degradation;Tropane, piperidine and pyridine alkaloid biosynthesis
Phvul.011G051900.1	ribosome biogenesis BMS1 homolog	nucleus;hydrolase activity;mRNA export from nucleus;protein import into nucleus;ribosome biogenesis		
Phvul.011G054400.1	probable serine threonine- kinase GCN2	protein kinase activity;ATP binding;protein phosphorylation		
Phvul.011G054900.1	ATPase family AAA domain	nucleus;chromatin binding;ATP binding;ATPase activity;histone binding;negative regulation of chromatin silencing;positive regulation of transcription from RNA polymerase II promoter;cell division	ec:3.6.1.3-adenylpyrophosphatase	Purine metabolism
Phvul.011G055500.1	probable E3 ubiquitin- ligase ARI7	ubiquitin ligase complex;cytoplasm;nucleic acid binding;zinc ion binding;ligase activity;ubiquitin conjugating enzyme binding;ubiquitin protein ligase activity;protein polyubiquitination;positive regulation of proteasomal ubiquitin-dependent protein catabolic process;protein ubiquitination involved in ubiquitin-dependent protein catabolic process		
Phvul.011G056200.1	cyanogenic beta-glucosidase,	integral component of membrane;lactase activity;beta-glucosidase activity;carbohydrate metabolic process	ec:3.2.1.21-gentiobiase;ec:3.2.1.108-lactase-phlorizin hydrolase	Starch and sucrose metabolism;Cyanoamino acid metabolism;Phenylpropanoid biosynthesisGalactose metabolism
Phvul.011G056600.1	multiple C2 and transmembrane domain-containing 1-like	plasmodesma;integral component of membrane;phosphatidylserine decarboxylase activity;calcium ion binding;calcium-dependent phospholipid binding;transferase activity, transferring glycosyl groups;metabolic process	ec:4.1.1.65-decarboxylase	Glycerophospholipid metabolism
Phvul.011G057300.1	hypothetical protein PHAVU_011G057300g	plasma membrane		
Phvul.011G057500.1	LOB domain-containing 4	carbamoyl-phosphate synthase (glutamine-hydrolyzing) activity;metabolic process;determination of bilateral symmetry;polarity specification of adaxial/abaxial axis;meristem initiation;regulation of meristem growth	ec:6.3.5.5-synthase (glutamine-hydrolysing)	Alanine, aspartate and glutamate metabolism;Pyrimidine metabolism
Phvul.011G058200.1	hypothetical protein PHAVU_011G058200g	integral component of membrane		
Phvul.011G060300.1	UDP-glucose iridoid glucosyltransferase-like	intracellular membrane-bounded organelle;quercetin 3-O-glucosyltransferase activity;quercetin 7-O-glucosyltransferase activity;flavonoid biosynthetic process;flavonoid glucuronidation		
Phvul.011G061000.1	pentatricopeptide repeat-containing At4g01570			
Phvul.011G063100.1	scarecrow 14	transcription, DNA-templated;regulation of transcription, DNA-templated		
Phvul.011G064300.1	ENTH VHS family			
Phvul.011G066000.1	THO complex subunit 4A	nucleotide binding;nucleic acid binding		
Phvul.011G068900.1	3-ketoacyl-synthase 12-like	integral component of membrane;transferase activity, transferring acyl groups other than amino-acyl groups;fatty acid biosynthetic process		
Phvul.011G069600.1	receptor kinase HERK 1	integral component of membrane;protein serine/threonine kinase activity;ATP binding;protein phosphorylation	ec:2.7.11	
Phvul.011G070100.1	DNA-directed RNA polymerase III subunit rpc1	DNA-directed RNA polymerase III complex;RNA polymerase III activity;DNA binding;transcription from RNA polymerase III promoter		

Phvul.011G070400.1	transmembrane ,	integral component of membrane		
Phvul.011G071200.1	CCR4-NOT transcription complex subunit 4	CCR4-NOT complex;nucleotide binding;nucleic acid binding;ubiquitin-protein transferase activity;zinc ion binding;ligase activity;protein ubiquitination		
Phvul.011G071400.2	mitogen-activated kinase 3	intracellular;MAP kinase activity;ATP binding;MAPK cascade;activation of MAPK activity involved in osmosensory signaling pathway;response to hypoxia;regulation of transcription, DNA-templated;protein targeting to membrane;response to osmotic stress;response to cold;detection of biotic stimulus;salicylic acid biosynthetic process;abscisic acid-activated signaling pathway;systemic acquired resistance, salicylic acid mediated signaling pathway;jasmonic acid mediated signaling pathway;camalexin biosynthetic process;response to UV-B;inflorescence development;regulation of hydrogen peroxide metabolic process;regulation of plant-type hypersensitive response;stomatal complex development;photosynthesis, light reaction;negative regulation of defense response;regulation of protein dephosphorylation;defense response to bacterium;negative regulation of programmed cell death;regulation of multi-organism process;plant ovule development;defense response to fungus;priming of cellular response to stress;regulation of stomatal complex patterning;regulation of stomatal complex development	ec:2.7.11.24-protein kinase	mTOR signaling pathway
Phvul.011G071800.1	arogenate dehydratase prephenate dehydratase 2, chloroplastic-like	chloroplast stroma;prephenate dehydratase activity;amino acid binding;arogenate dehydratase activity;L-phenylalanine biosynthetic process;vernalization response;anthocyanin accumulation in tissues in response to UV light;carpel development	ec:4.2.1.91-dehydratase;ec:4.2.1.51-dehydratase	Biosynthesis of antibiotics;Phenylalanine, tyrosine and tryptophan biosynthesisBiosynthesis of antibiotics;Phenylalanine, tyrosine and tryptophan biosynthesis
Phvul.011G072000.1	interactor of constitutive active ROPs 3 isoform X2			
Phvul.011G072500.1	RNA ligase,	RNA ligase (ATP) activity;2',3'-cyclic-nucleotide 3'-phosphodiesterase activity;ATP binding;polynucleotide 5'-hydroxyl-kinase activity;tRNA splicing, via endonucleolytic cleavage and ligation;phosphorylation	ec:6.5.1.3;ec:3.1.4.37;ec:2.7.1	
Phvul.011G072500.2	RNA ligase,	RNA ligase (ATP) activity;2',3'-cyclic-nucleotide 3'-phosphodiesterase activity;ATP binding;polynucleotide 5'-hydroxyl-kinase activity;tRNA splicing, via endonucleolytic cleavage and ligation;phosphorylation	ec:6.5.1.3;ec:3.1.4.37;ec:2.7.1	
Phvul.011G074500.1	fasciclin-like arabinogalactan 11	integral component of membrane		
Phvul.011G077800.1	root cap late embryogenesis			
Phvul.011G078000.1	heat shock with TPR ,	cellular component organization;single-organism cellular process;single-organism developmental process;anatomical structure development		
Phvul.011G079600.1	SAND isoform X1	cell;calcium ion transport;cellular zinc ion homeostasis;response to nematode;vesicle-mediated transport		
Phvul.011G080000.1	serine threonine-phosphatase 6 regulatory subunit 3			
Phvul.011G080500.1	glycosyltransferase family 2	integral component of membrane;transferase activity;metabolic process		
Phvul.011G080600.1	cytokinin riboside 5 -monophosphate	nucleus;cytosol;hydrolase activity, hydrolyzing N-glycosyl compounds;cytokinin biosynthetic process		

	phosphoribohydrolase LOG1			
Phvul.011G080800.1	cyclic nucleotide-gated ion channel 15	integral component of membrane;voltage-gated potassium channel activity;regulation of membrane potential;potassium ion transmembrane transport		
Phvul.011G082700.1	P-loop NTPase domain-containing LPA1 homolog 1-like	kinase activity;hydrolase activity;phosphorylation		
Phvul.011G082700.2	P-loop NTPase domain-containing LPA1 homolog 1-like	kinase activity;hydrolase activity;phosphorylation		
Phvul.011G082800.1	hypothetical protein PHAVU_011G082800g			
Phvul.011G083800.1	cell division cycle 48 homolog	plasma membrane;ATP binding;microtubule-severing ATPase activity;cell cycle;metabolic process;cell division	ec:3.6.4.3	
Phvul.011G084000.1	ABC transporter F family member 1	transporter activity;ATP binding;ATPase activity;glucose catabolic process;transport;methylglyoxal catabolic process to D-lactate via S-lactoyl-glutathione	ec:3.6.1;ec:3.6.1.3-adenylpyrophosphatase;ec:3.6.1.15-phosphatase	Purine metabolismPurine metabolism;Thiamine metabolism
Phvul.011G084000.2	ABC transporter F family member 1	transporter activity;ATP binding;ATPase activity;glucose catabolic process;transport;methylglyoxal catabolic process to D-lactate via S-lactoyl-glutathione	ec:3.6.1.3-adenylpyrophosphatase	Purine metabolism
Phvul.011G085500.1	hypothetical protein PHAVU_011G085500g			
Phvul.011G086700.1	pheophorbide a oxygenase, chloroplastic	chloroplast thylakoid;chloroplast envelope;chlorophyllide a oxygenase [overall] activity;pheophorbide a oxygenase activity;metal ion binding;2 iron, 2 sulfur cluster binding;cell death;embryo development ending in seed dormancy;defense response to bacterium, incompatible interaction;leaf morphogenesis;thylakoid membrane organization;vegetative to reproductive phase transition of meristem;iron-sulfur cluster assembly;cell differentiation;positive regulation of transcription, DNA-templated;plant ovule development;oxidation-reduction process	ec:1.8;ec:1.14.13.122-oxygenase	Porphyrin and chlorophyll metabolism
Phvul.011G088100.1	vacuolar sorting-associated 41 homolog	late endosome;HOPS complex;CORVET complex;zinc ion binding;GTPase binding;protein targeting to vacuole;gravitropism;vesicle-mediated transport;regulation of SNARE complex assembly;vacuole fusion, non-autophagic		
Phvul.011G088300.1	aspartic ase 2	integral component of membrane;aspartic-type endopeptidase activity;transferase activity;proteolysis;protein catabolic process	ec:3.4.23	
Phvul.011G089000.1	ribulose-1,5-bisphosphate carboxylase oxygenase large subunit, partial (chloroplast)	chloroplast;magnesium ion binding		
Phvul.011G090800.1	WD repeat and FYVE domain-containing 3	RNA polymerase I transcription factor complex;transcription from RNA polymerase I promoter		
Phvul.011G090800.2	WD repeat and FYVE domain-containing 3	RNA polymerase I transcription factor complex;transcription from RNA polymerase I promoter		
Phvul.011G090800.3	WD repeat and FYVE domain-containing 3	RNA polymerase I transcription factor complex;transcription from RNA polymerase I promoter		
Phvul.011G091900.1	DNA polymerase epsilon catalytic subunit A	nucleus;nucleotide binding;DNA binding;DNA-directed DNA polymerase activity;zinc ion binding;3'-5' exonuclease activity;DNA replication;DNA biosynthetic process;nucleic acid phosphodiester bond hydrolysis	ec:3.1;ec:2.7.7.7-DNA polymerase	Purine metabolism;Pyrimidine metabolism

Phvul.011G092500.1	Fatty acyl-reductase 3	intracellular membrane-bounded organelle;fatty-acyl-CoA reductase (alcohol-forming) activity;wax biosynthetic process;suberin biosynthetic process;long-chain fatty-acyl-CoA metabolic process;oxidation-reduction process		
Phvul.011G092600.1	subtilisin-like protease	cell wall;serine-type endopeptidase activity;proteolysis	ec:3.4.21	
Phvul.011G092600.2	subtilisin-like protease	cell wall;serine-type endopeptidase activity;proteolysis	ec:3.4.21	
Phvul.011G093800.1	F-box LRR-repeat At1g67190-like			
Phvul.011G095900.1	mitochondrial fission ELM1-like	mitochondrial fission		
Phvul.011G099500.1	FAF-like, chloroplastic			
Phvul.011G099600.1	BRI1 suppressor 1 (BSU1)-like 2 isoform 1	phosphoprotein phosphatase activity;protein dephosphorylation;brassinosteroid mediated signaling pathway	ec:3.1;ec:3.1.3.41-nitrophenyl phosphatase;ec:3.1.3.16-phosphatase	Aminobenzoate degradationT cell receptor signaling pathway
Phvul.011G100500.1	replication factor C1	nucleus;DNA replication factor C complex;DNA clamp loader activity;ATP binding;resolution of meiotic recombination intermediates;DNA replication;DNA repair;response to abscisic acid;regulation of chromatin silencing;regulation of histone H3-K9 methylation	ec:3.6.1;ec:3.6.1.3-adenylpyrophosphatase;ec:3.6.1.15-phosphatase	Purine metabolismPurine metabolism;Thiamine metabolism
Phvul.011G101500.1	cellulose synthase H1	integral component of membrane;cellulose synthase (UDP-forming) activity;cellulose biosynthetic process;cell wall organization	ec:2.4.1.12-synthase (UDP-forming)	Starch and sucrose metabolism
Phvul.011G101700.1	cellulose synthase H1	integral component of membrane;cellulose synthase (UDP-forming) activity;cellulose biosynthetic process;cell wall organization	ec:2.4.1.12-synthase (UDP-forming)	Starch and sucrose metabolism
Phvul.011G101700.2	cellulose synthase H1	integral component of membrane;cellulose synthase (UDP-forming) activity;cellulose biosynthetic process;cell wall organization	ec:2.4.1.12-synthase (UDP-forming)	Starch and sucrose metabolism
Phvul.011G102500.1	L-ascorbate oxidase homolog	plant-type cell wall;plasmodesma;copper ion binding;L-ascorbate oxidase activity;oxidoreductase activity, oxidizing metal ions;oxidation-reduction process	ec:1.10.3.3-oxidase;ec:1.10.3	Ascorbate and aldarate metabolism
Phvul.011G102900.1	hypothetical protein PHAVU_011G102900g	integral component of membrane		
Phvul.011G103400.1	probable beta-1,3-galactosyltransferase 2	Golgi apparatus;integral component of membrane;galactosylxylosylprotein 3-beta-galactosyltransferase activity;protein glycosylation	ec:2.4.1.134-3-beta-galactosyltransferase	Glycosaminoglycan biosynthesis - heparan sulfate / heparin;Glycosaminoglycan biosynthesis - chondroitin sulfate / dermatan sulfate
Phvul.011G103700.1	aconitate hydratase, cytoplasmic	cell wall;vacuolar membrane;cytosol;chloroplast stroma;aconitate hydratase activity;copper ion binding;ATP binding;4 iron, 4 sulfur cluster binding;response to superoxide;gluconeogenesis;glycolytic process;citrate metabolic process;isocitrate metabolic process;protein N-linked glycosylation;ubiquitin-dependent protein catabolic process;water transport;hyperosmotic response;Golgi organization;response to temperature stimulus;response to auxin;response to carbohydrate;photorespiration;ethylene-activated signaling pathway;response to iron ion;photosynthesis;response to misfolded protein;iron ion homeostasis;proteasome core complex assembly	ec:4.2.1.3-hydratase	Carbon fixation pathways in prokaryotes;Biosynthesis of antibiotics;Citrate cycle (TCA cycle);Glyoxylate and dicarboxylate metabolism
Phvul.011G104000.1	DUF4228 domain			
Phvul.011G107700.1	beta-amylase	beta-amylase activity;polysaccharide catabolic process	ec:3.2.1.2-saccharogen amylase	Starch and sucrose metabolism
Phvul.011G109900.1	myb-related Myb4-like	DNA binding		

Phvul.011G110000.1	cyclin-D6-1	nucleus;integral component of membrane;cell cycle;regulation of cell cycle		
Phvul.011G110000.2	cyclin-D6-1	nucleus;integral component of membrane;cell cycle;regulation of cell cycle		
Phvul.011G111800.1	embryo defective 1923 ,	integral component of membrane;rRNA processing;tRNA metabolic process;chloroplast organization		
Phvul.011G112400.1	H ACA ribonucleo complex non-core subunit NAF1	viral nucleocapsid;intracellular ribonucleoprotein complex;pseudouridine synthesis;ribosome biogenesis		
Phvul.011G114500.1	hypothetical protein PHAVU_011G114500g, partial	integral component of membrane		
Phvul.011G115100.1	transmembrane E3 ubiquitin- ligase 1-like	integral component of membrane;zinc ion binding;ligase activity;ubiquitin protein ligase activity;protein polyubiquitination;protein ubiquitination involved in ubiquitin-dependent protein catabolic process;proteasome-mediated ubiquitin-dependent protein catabolic process		
Phvul.011G115600.1	transcription factor VOZ1-like	positive regulation of biological process;response to stimulus		
Phvul.011G116300.1	translation factor GUF1 homolog, organellar chromatophore	intracellular;translation elongation factor activity;GTPase activity;GTP binding;translational elongation	ec:3.6.1;ec:3.6.1.15-phosphatase	Purine metabolism;Thiamine metabolism
Phvul.011G117500.1	expansin-B3-like precursor	extracellular region;plasmodesma;hydrolase activity;syncytium formation;metabolic process;sexual reproduction		
Phvul.011G117600.1	alpha-expansin 20 precursor family	extracellular region;cell wall;integral component of membrane;plant-type cell wall organization		
Phvul.011G119200.1	probable L-type lectin-domain containing receptor kinase	integral component of membrane;protein serine/threonine kinase activity;transmembrane receptor protein tyrosine kinase activity;ATP binding;carbohydrate binding;peptidyl-tyrosine phosphorylation	ec:2.7.11;ec:2.7.10;ec:2.7.10.1	
Phvul.011G124600.1	M48 family peptidase	integral component of membrane;metalloendopeptidase activity;proteolysis	ec:3.4.24	
Phvul.011G124700.1	peroxisomal membrane 22 kDa (Mpv17 PMP22) family	mitochondrion;peroxisomal membrane;chloroplast envelope;integral component of membrane		
Phvul.011G128000.1	shaggy-related kinase eta isoform X1	protein kinase activity;ATP binding;protein phosphorylation		
Phvul.011G128800.1	multi -bridging factor 1a	cytoplasm;transcription coactivator activity;sequence-specific DNA binding;response to high light intensity;response to hydrogen peroxide;positive regulation of transcription, DNA-templated		
Phvul.011G136200.1	TMV resistance N-like	ADP binding;defense response;signal transduction		
Phvul.011G136200.2	disease resistance (TIR-NBS-LRR class),	ADP binding;defense response;signal transduction		
Phvul.011G140300.1	disease resistance (TIR-NBS-LRR class),	ADP binding;defense response;signal transduction		
Phvul.011G141900.1	Formin 5 isoform 1	cell wall;phragmoplast;integral component of membrane;actin binding;structural constituent of cell wall;endosperm development;actin filament polymerization;seed morphogenesis		
Phvul.011G142300.1	scarecrow 21	transcription, DNA-templated;regulation of transcription, DNA-templated		
Phvul.011G142300.2	scarecrow 21	transcription, DNA-templated;regulation of transcription, DNA-templated		
Phvul.011G144300.1	ACCELERATED CELL DEATH 6-like	integral component of membrane;regulation of defense response;cellular response to salicylic acid stimulus;regulation of salicylic acid mediated signaling pathway		

Phvul.011G144400.1	chromosome transmission fidelity 18 homolog	ATP binding		
Phvul.011G146000.1	mRNA cap guanine-N7 methyltransferase 1	nucleus;mRNA cap binding complex;RNA binding;mRNA (guanine-N7-)-methyltransferase activity;7-methylguanosine mRNA capping;proline transport;RNA (guanine-N7)-methylation	ec:2.1.1;ec:2.1.1.56	
Phvul.011G148800.1	homogentisate 1,2-dioxygenase	cytosol;homogentisate 1,2-dioxygenase activity;L-phenylalanine catabolic process;tyrosine metabolic process;fatty acid beta-oxidation;response to sucrose;response to fructose;chlorophyll catabolic process;protein import into peroxisome matrix;homogentisate catabolic process	ec:1.13.11.5-1,2-dioxygenase	Tyrosine metabolism;Styrene degradation
Phvul.011G149500.1	S-locus lectin kinase family	plasma membrane;plasmodesma;integral component of membrane;protein serine/threonine kinase activity;ATP binding;ubiquitin protein ligase binding;protein phosphorylation;innate immune response;recognition of pollen	ec:2.7.11	
Phvul.011G151000.1	G-type lectin S-receptor-like serine threonine- kinase At4g27290 isoform X2	plasma membrane;plasmodesma;integral component of membrane;protein serine/threonine kinase activity;ATP binding;carbohydrate binding;protein phosphorylation;defense response;recognition of pollen	ec:2.7.11	
Phvul.011G151700.1	ankyrin repeat domain-containing 13C			
Phvul.011G152300.1	G-type lectin S-receptor-like serine threonine- kinase At4g27290 isoform X1	plasma membrane;plasmodesma;integral component of membrane;protein serine/threonine kinase activity;ATP binding;protein phosphorylation;defense response;recognition of pollen	ec:2.7.11	
Phvul.011G152300.2	G-type lectin S-receptor-like serine threonine- kinase At4g27290 isoform X1	plasma membrane;plasmodesma;integral component of membrane;protein serine/threonine kinase activity;ATP binding;protein phosphorylation;defense response;recognition of pollen	ec:2.7.11	
Phvul.011G152300.3	G-type lectin S-receptor-like serine threonine- kinase At4g27290 isoform X2	plasma membrane;plasmodesma;integral component of membrane;protein serine/threonine kinase activity;ATP binding;protein phosphorylation;defense response;recognition of pollen	ec:2.7.11	
Phvul.011G152700.1	G-type lectin S-receptor-like serine threonine- kinase At4g27290 isoform X1	plasma membrane;plasmodesma;integral component of membrane;protein serine/threonine kinase activity;ATP binding;carbohydrate binding;protein phosphorylation;defense response;recognition of pollen	ec:2.7.11	
Phvul.011G158100.1	SAWADEE HOMEODOMAIN HOMOLOG 2	integral component of membrane;DNA binding;chromatin binding		
Phvul.011G158200.1	SAWADEE HOMEODOMAIN HOMOLOG 2	nucleus;integral component of membrane;DNA binding;chromatin binding		
Phvul.011G159200.1	Fiber Fb32 isoform 3			
Phvul.011G159500.1	Serine threonine-phosphatase 4 regulatory subunit 2-A,	DNA binding		
Phvul.011G159700.1	serine threonine-kinase isoform X2	plasma membrane;protein serine/threonine kinase activity;transmembrane receptor protein tyrosine kinase activity;ATP binding;signal transduction;peptidyl-tyrosine phosphorylation;innate immune response;cellular response to organic substance	ec:2.7.11;ec:2.7.10.1	
Phvul.011G161000.1	phytosulfokine receptor 2-like	chloroplast;integral component of membrane;peptide receptor activity;MAP kinase kinase kinase activity;transmembrane receptor protein tyrosine kinase activity;ATP binding;MAPK cascade;activation of MAPKK	ec:2.7.11;ec:2.7.11.25;ec:2.7.10.1	

		activity;response to wounding;peptidyl-tyrosine phosphorylation		
Phvul.011G161100.1	11-oxo-beta-amyrin 30-oxidase-like	integral component of membrane;acyl-CoA oxidase activity;iron ion binding;oxidoreductase activity, acting on paired donors, with incorporation or reduction of molecular oxygen, NAD(P)H as one donor, and incorporation of one atom of oxygen;heme binding;oxidation-reduction process;11-oxo-beta-amyrin catabolic process;glycyrrhetinate biosynthetic process	ec:1.3.3.6-oxidase;ec:1.14.13	alpha-Linolenic acid metabolism;Biosynthesis of unsaturated fatty acids;Fatty acid degradation
Phvul.011G161100.2	cytochrome P450 monooxygenase	integral component of membrane;acyl-CoA oxidase activity;iron ion binding;oxidoreductase activity, acting on paired donors, with incorporation or reduction of molecular oxygen, NAD(P)H as one donor, and incorporation of one atom of oxygen;heme binding;oxidation-reduction process;11-oxo-beta-amyrin catabolic process;glycyrrhetinate biosynthetic process	ec:1.3.3.6-oxidase;ec:1.14.13	alpha-Linolenic acid metabolism;Biosynthesis of unsaturated fatty acids;Fatty acid degradation
Phvul.011G161200.2	cytochrome P450 monooxygenase	integral component of membrane;acyl-CoA oxidase activity;iron ion binding;oxidoreductase activity, acting on paired donors, with incorporation or reduction of molecular oxygen, NAD(P)H as one donor, and incorporation of one atom of oxygen;heme binding;oxidation-reduction process;11-oxo-beta-amyrin catabolic process;glycyrrhetinate biosynthetic process	ec:1.3.3.6-oxidase;ec:1.14.13	alpha-Linolenic acid metabolism;Biosynthesis of unsaturated fatty acids;Fatty acid degradation
Phvul.011G162500.1	hypothetical protein PHAVU_011G162500g			
Phvul.011G163800.1	ankyrin repeat	integral component of membrane		
Phvul.011G164700.1	germinal center kinase 1-like isoform X1	cytoplasm;cyclin-dependent protein serine/threonine kinase activity;ATP binding;Rac GTPase binding;Rho protein signal transduction;regulation of mitotic cell cycle;cell migration;signal transduction by protein phosphorylation;actin cytoskeleton organization;stress-activated protein kinase signaling cascade;activation of protein kinase activity;regulation of apoptotic process;regulation of MAPK cascade	ec:2.7.11.22	
Phvul.011G164700.2	germinal center kinase 1-like isoform X1	cytoplasm;cyclin-dependent protein serine/threonine kinase activity;ATP binding;Rac GTPase binding;Rho protein signal transduction;regulation of mitotic cell cycle;cell migration;signal transduction by protein phosphorylation;actin cytoskeleton organization;stress-activated protein kinase signaling cascade;activation of protein kinase activity;regulation of apoptotic process;regulation of MAPK cascade	ec:2.7.11;ec:2.7.11.22	
Phvul.011G164700.3	germinal center kinase 1-like isoform X1	cytoplasm;cyclin-dependent protein serine/threonine kinase activity;ATP binding;Rac GTPase binding;Rho protein signal transduction;regulation of mitotic cell cycle;cell migration;signal transduction by protein phosphorylation;actin cytoskeleton organization;stress-activated protein kinase signaling cascade;activation of protein kinase activity;regulation of apoptotic process;regulation of MAPK cascade	ec:2.7.11.22	
Phvul.011G164700.4	germinal center kinase 1-like isoform X1	cytoplasm;cyclin-dependent protein serine/threonine kinase activity;ATP binding;Rac GTPase binding;Rho protein signal transduction;regulation of mitotic cell cycle;cell migration;signal transduction by protein phosphorylation;actin cytoskeleton organization;stress-activated protein kinase signaling cascade;activation of protein kinase activity;regulation of apoptotic process;regulation of MAPK cascade	ec:2.7.11;ec:2.7.11.22	

Phvul.011G165500.1	anaphase-promoting complex subunit 6	double-strand break repair via homologous recombination;cytokinesis by cell plate formation;DNA replication initiation;DNA methylation;methylation-dependent chromatin silencing;synapsis;reciprocal meiotic recombination;cell adhesion;gamete generation;cell proliferation;nucleotide biosynthetic process;pollen development;regulation of flower development;leaf morphogenesis;phloem or xylem histogenesis;trichome morphogenesis;regulation of G2/M transition of mitotic cell cycle;somatic cell DNA recombination;chromatin silencing by small RNA;regulation of DNA endoreduplication;DNA endoreduplication;post-translational protein modification;actin nucleation;positive regulation of transcription, DNA-templated;root hair cell differentiation;spindle assembly;regulation of cell division;regulation of unidimensional cell growth;histone H3-K9 methylation;cell wall organization		
Phvul.011G166400.1	receptor kinase	integral component of membrane;protein serine/threonine kinase activity;ATP binding;protein phosphorylation	ec:2.7.11	
Phvul.011G166600.1	hypothetical protein PHAVU_011G166600g			
Phvul.011G168400.1	S-locus lectin kinase family	plasma membrane;plasmodesma;integral component of membrane;protein serine/threonine kinase activity;ATP binding;carbohydrate binding;protein phosphorylation;defense response;recognition of pollen	ec:2.7.11	
Phvul.011G169300.1	probable LRR receptor-like serine threonine- kinase At1g56130	plasma membrane;integral component of membrane;protein serine/threonine kinase activity;transmembrane receptor protein tyrosine kinase activity;ATP binding;peptidyl-tyrosine phosphorylation	ec:2.7.11;ec:2.7.10.1	
Phvul.011G170000.1	PHD finger family	zinc ion binding		
Phvul.011G170000.2	Jade-1-like isoform X2	zinc ion binding		
Phvul.011G170200.1	amidophosphoribosyltransferase, chloroplastic-like	amidophosphoribosyltransferase activity;metal ion binding;iron-sulfur cluster binding;'de novo' IMP biosynthetic process;purine nucleobase biosynthetic process;nucleoside metabolic process	ec:2.4.2.14-phosphoribosyldiphosphate 5-amidotransferase	Purine metabolism;Biosynthesis of antibiotics;Alanine, aspartate and glutamate metabolism
Phvul.011G172100.1	hypothetical protein PHAVU_011G172100g	ADP binding;defense response		
Phvul.011G172100.2	hypothetical protein PHAVU_011G172100g	ADP binding;defense response		
Phvul.011G172200.1	tRNA (adenine(58)-N(1))-methyltransferase catalytic subunit TRMT61A	nucleus;tRNA (m1A) methyltransferase complex;tRNA (adenine-N1-)-methyltransferase activity;tRNA methylation	ec:2.1.1	
Phvul.011G172900.1	vacuolar sorting-associated 8 homolog			
Phvul.011G173800.1	disease resistance RPP13 1	ADP binding;defense response		
Phvul.011G174000.1	cationic amino acid transporter 8, vacuolar	integral component of membrane;intracellular membrane-bounded organelle;L-glutamate transmembrane transporter activity;L-lysine transmembrane transporter activity;antiporter activity;response to brassinosteroid;L-arginine import;L-glutamate import;L-lysine transmembrane transport		

Phvul.011G174800.1	obg-like ATPase 1	chloroplast stroma;ATP binding;GTP binding;ATPase activity;ribosome binding;ribosomal large subunit binding;heme biosynthetic process	ec:3.6.1.3-adenylpyrophosphatase	Purine metabolism
Phvul.011G175400.1	cysteine-rich RLK (receptor-like kinase)	integral component of membrane;protein kinase activity;ATP binding;protein phosphorylation		
Phvul.011G175400.2	cysteine-rich RLK (receptor-like kinase)	integral component of membrane;protein kinase activity;ATP binding;protein phosphorylation		
Phvul.011G175600.1	cysteine-rich RLK (receptor-like kinase)	integral component of membrane;protein kinase activity;ATP binding;protein phosphorylation		
Phvul.011G177600.1	guanine nucleotide exchange factor,	nucleus;cytosol;plasma membrane;extrinsic component of membrane;endoplasmic reticulum exit site;guanyl-nucleotide exchange factor activity;cytokinesis by cell plate formation;acetyl-CoA metabolic process;microtubule nucleation;small GTPase mediated signal transduction;determination of bilateral symmetry;meristem initiation;regulation of meristem growth;trichome morphogenesis;sterol biosynthetic process;brassinosteroid biosynthetic process;vesicle-mediated transport;positive regulation of GTPase activity;actin nucleation		
Phvul.011G179000.1	cyclin-B3-1 isoform X2	nucleus		
Phvul.011G179600.1	MYND finger	zinc ion binding		
Phvul.011G179600.3	MYND finger	zinc ion binding		
Phvul.011G180100.1	strawberry notch-like	zinc ion binding;regulation of transcription, DNA-templated		
Phvul.011G181200.1	exosome complex component RRP43-like	nuclear exosome (RNase complex);cytoplasmic exosome (RNase complex);cytosol;exonuclease activity;AU-rich element binding;exonucleolytic trimming to generate mature 3'-end of 5.8S rRNA from tricistronic rRNA transcript (SSU-rRNA, 5.8S rRNA, LSU-rRNA);nuclear-transcribed mRNA catabolic process, exonucleolytic, 3'-5';U1 snRNA 3'-end processing;U4 snRNA 3'-end processing;U5 snRNA 3'-end processing;transcription factor import into nucleus;exonucleolytic nuclear-transcribed mRNA catabolic process involved in deadenylation-dependent decay;nuclear mRNA surveillance;nuclear polyadenylation-dependent rRNA catabolic process;nuclear polyadenylation-dependent tRNA catabolic process;nuclear polyadenylation-dependent mRNA catabolic process		
Phvul.011G181600.1	resistance KR4	ADP binding;defense response		
Phvul.011G182300.1	probable leucine-rich repeat receptor kinase At1g35710	integral component of membrane;protein kinase activity;ATP binding;protein phosphorylation		
Phvul.011G186500.1	SWIM zinc finger family	zinc ion binding		
Phvul.011G186900.1	calcium and calcium calmodulin-dependent serine threonine- kinase	cytoplasm;integral component of membrane;calmodulin-dependent protein kinase activity;calcium ion binding;calmodulin binding;ATP binding;calcium-dependent protein serine/threonine kinase activity;response to symbiont;nodulation;peptidyl-serine phosphorylation;intracellular signal transduction;protein autophosphorylation	ec:2.7.11;ec:2.7.11.17	
Phvul.011G190200.1	histone-lysine N-methyltransferase, H3 lysine-9 specific SUVH1-like	nucleus;chromosome;zinc ion binding;histone-lysine N-methyltransferase activity;histone binding;histone lysine methylation	ec:2.1.1.43-N-methyltransferase	Lysine degradation
Phvul.011G192800.1	cilia- and flagella-associated 20			

Phvul.011G193000.1	hypothetical protein PHAVU_011G193000g	ADP binding;defense response		
Phvul.011G194000.1	hypothetical protein PHAVU_011G194000g	ADP binding;defense response		
Phvul.011G194300.1	cysteine-rich receptor kinase 10	plasma membrane;plasmodesma;integral component of membrane;protein serine/threonine kinase activity;ATP binding;protein phosphorylation;defense response	ec:2.7.11	
Phvul.011G194300.2	cysteine-rich receptor kinase 10	plasma membrane;plasmodesma;integral component of membrane;protein serine/threonine kinase activity;ATP binding;protein phosphorylation;defense response	ec:2.7.11	
Phvul.011G194600.1	cysteine-rich receptor kinase 25	plasma membrane;plasmodesma;integral component of membrane;protein serine/threonine kinase activity;non-membrane spanning protein tyrosine kinase activity;ATP binding;defense response;peptidyl-tyrosine phosphorylation	ec:2.7.11;ec:2.7.10.2-protein-tyrosine kinase	T cell receptor signaling pathway
Phvul.011G195200.1	hypothetical protein PHAVU_011G195200g	ADP binding;defense response		
Phvul.011G197900.1	disease resistance RPP13 1	ADP binding;defense response		
Phvul.011G200400.1	probable receptor kinase At1g67000 isoform X1	integral component of membrane;protein serine/threonine kinase activity;ATP binding;polysaccharide binding;protein phosphorylation	ec:2.7.11	
Phvul.011G200900.1	disease resistance RPP13 1	ADP binding;defense response		
Phvul.011G201100.1	disease resistance RPP13 1	ADP binding;defense response		
Phvul.011G201200.1	retinoblastoma-related 1	nucleus;cytosol;DNA binding;transcription factor binding;G1/S transition of mitotic cell cycle;regulation of cell growth;cell fate specification;regulation of gene expression by genetic imprinting;transcription, DNA-templated;regulation of transcription from RNA polymerase II promoter;asymmetric cell division;embryo sac development;trichome morphogenesis;generative cell differentiation;regulation of DNA endoreduplication;DNA endoreduplication;leaf development;regulation of nuclear division;regulation of stem cell population maintenance		
Phvul.011G201200.2	retinoblastoma-related 1	nucleus;cytosol;DNA binding;transcription factor binding;G1/S transition of mitotic cell cycle;regulation of cell growth;cell fate specification;regulation of gene expression by genetic imprinting;transcription, DNA-templated;regulation of transcription from RNA polymerase II promoter;asymmetric cell division;embryo sac development;trichome morphogenesis;generative cell differentiation;regulation of DNA endoreduplication;DNA endoreduplication;leaf development;regulation of nuclear division;regulation of stem cell population maintenance		
Phvul.011G201700.2	glycoside hydrolase family 1	integral component of membrane;beta-glucosidase activity;carbohydrate metabolic process	ec:3.2.1;ec:3.2.1.21-gentiobiase	Starch and sucrose metabolism;Cyanoamino acid metabolism;Phenylpropanoid biosynthesis
Phvul.011G201800.1	hypothetical protein PHAVU_011G201800g			

Phvul.011G202100.1	disease resistance At3g14460	ADP binding;defense response		
Phvul.011G204500.1	leginsulin 1	nutrient reservoir activity;pathogenesis		
Phvul.011G206500.1	amidohydrolase ,	endoplasmic reticulum;perinuclear region of cytoplasm;hydrolase activity, acting on carbon-nitrogen (but not peptide) bonds;regulation of mitotic cell cycle;metabolic process		
Phvul.011G206600.1	hypothetical protein PHAVU_011G206600g	mitochondrion;structural constituent of ribosome		
Phvul.011G206600.2	hypothetical protein PHAVU_011G206600g	mitochondrion;structural constituent of ribosome		
Phvul.011G206800.1	beta-galactosidase	beta-galactosidase complex;beta-galactosidase activity;carbohydrate binding;carbohydrate metabolic process	ec:3.2.1;ec:3.2.1.23-lactase (ambiguous)	Other glycan degradation;Glycosphingolipid biosynthesis - ganglio series;Sphingolipid metabolism;Galactose metabolism;Glycosaminoglycan degradation
Phvul.011G210000.2	chaperone dnaJ 10-like isoform X1			
Phvul.011G212400.1	FACT complex subunit SSRP1	nuclear euchromatin;FACT complex;DNA binding;transcription factor activity, sequence-specific DNA binding;2-alkenal reductase [NAD(P)] activity;RNA splicing, via endonucleolytic cleavage and ligation;DNA methylation;methylation-dependent chromatin silencing;transcription from RNA polymerase II promoter;cell-cell signaling;virus induced gene silencing;determination of bilateral symmetry;organ morphogenesis;meristem initiation;vegetative phase change;xylem and phloem pattern formation;meristem maintenance;vegetative to reproductive phase transition of meristem;production of ta-siRNAs involved in RNA interference;production of miRNAs involved in gene silencing by miRNA;flower morphogenesis;oxidation-reduction process	ec:1.3.1.74	
Phvul.011G215500.1	organic cation carnitine transporter	plant-type vacuole membrane;integral component of membrane;substrate-specific transmembrane transporter activity;transmembrane transport		
Phvul.011G215800.1	Eukaryotic translation initiation factor 2D	translation initiation factor activity;translational initiation		
Phvul.L000500.1	transmembrane ,	integral component of membrane;nucleotide binding;nucleic acid binding		
Phvul.L000800.1	probable receptor kinase At1g67000	integral component of membrane;protein kinase activity;ATP binding;polysaccharide binding;protein phosphorylation		
Phvul.L004300.1	pentatricopeptide repeat-containing At5g40400			
Phvul.L004400.1	truncated NBS-LRR resistance isoforms JA68, JA76, and JA80	ADP binding;defense response		
Phvul.L008300.1	Cellulose synthase E1	integral component of membrane;cellulose synthase (UDP-forming) activity;cellulose biosynthetic process;cell wall organization	ec:2.4.1.12-synthase (UDP-forming);ec:2.4.1	Starch and sucrose metabolism
Phvul.L008400.1	Magnesium transporter MRS2-3	integral component of membrane;magnesium ion transmembrane transporter activity;magnesium ion transport;magnesium ion transmembrane transport		
Phvul.L009100.1	transcription factor bHLH91-like	DNA binding;protein dimerization activity		
Phvul.L009100.2	transcription factor bHLH91-like	DNA binding;protein dimerization activity		

Phvul.L009100.3	transcription factor bHLH91-like	DNA binding;protein dimerization activity		
Phvul.L009500.1	hypothetical protein PHAVU_L009500g			
