# Supplementary material for: Genome-wide analysis of the transcriptional response to drought stress in root and leaf of common bean
Source: Genet Mol Biol. 2020 Mar 16;43(1):e20180259. doi: 10.1590/1678-4685-GMB-2018-0259 (PMC7307723; doi:10.1590/1678-4685-GMB-2018-0259)
Supplement: Supplementary file 4 [file 1415-4757-GMB-43-1-e20180259-s6.pdf]

Genetics and Molecular Biology 43, 1, (2020)

Supplementary Material to: "Genome-wide analysis of the transcriptional response to drought stress in root and leaf of common bean Genome-wide analysis of the transcriptional response to drought stress in root and leaf of common bean"

Table S4 Statistical assessment of GO term enrichment for the new loci, DEGs and genes under high impact SNP effects (HISE) using the Fisher's Exact Test in combination with a robust False Discovery Rate (FDR) correction for multiple testing (<0.05). $ BP: Biological Process; CC: Cellular Component; MF: Molecular Function

Over/Under*	GO ID	GO Name	GO Category$	FDR	P-value	Test	Ref	Non Annot Test	Non Annot Reference
			**NEW LOCI**						
OVER	GO:0000003	reproduction	BP	3.67E-20	7.47E-22	24	70	442	27127
OVER	GO:0000059	protein import into nucleus, docking	BP	4.34E-02	1.68E-02	1	0	465	27197
OVER	GO:0000060	protein import into nucleus, translocation	BP	4.34E-02	1.68E-02	1	0	465	27197
OVER	GO:0000075	cell cycle checkpoint	BP	4.34E-02	1.68E-02	1	0	465	27197
OVER	GO:0000096	sulfur amino acid metabolic process	BP	1.43E-08	1.03E-09	8	13	458	27184
OVER	GO:0000097	sulfur amino acid biosynthetic process	BP	5.88E-09	3.92E-10	8	11	458	27186
OVER	GO:0000109	nucleotide-excision repair complex	CT	4.34E-02	1.68E-02	1	0	465	27197
OVER	GO:0000126**	transcription factor TFIIIB complex*	CT	1.35E-03	2.83E-04	2	0	464	27197
OVER	GO:0000145**	exocyst*	CT	4.34E-02	1.25E-02	3	26	463	27171
OVER	GO:0000149	SNARE binding	MF	4.34E-02	1.68E-02	1	0	465	27197
OVER	GO:0000151	ubiquitin ligase complex	CT	3.99E-02	1.03E-02	5	73	461	27124
OVER	GO:0000165	MAPK cascade	BP	4.34E-02	1.68E-02	1	0	465	27197
OVER	GO:0000166	nucleotide binding	MF	4.34E-13	1.56E-14	110	2973	356	24224
OVER	GO:0000226	microtubule cytoskeleton organization	BP	2.93E-02	7.41E-03	2	6	464	27191
OVER	GO:0000228	nuclear chromosome	CT	3.27E-03	7.09E-04	3	8	463	27189
OVER	GO:0000244	spliceosomal tri-snRNP complex assembly	BP	4.34E-02	1.68E-02	1	0	465	27197
OVER	GO:0000271	polysaccharide biosynthetic process	BP	2.88E-02	7.16E-03	7	125	459	27072
OVER	GO:0000278	mitotic cell cycle	BP	1.17E-02	2.71E-03	3	14	463	27183
OVER	GO:0000287	magnesium ion binding	MF	4.28E-04	7.28E-05	9	96	457	27101
OVER	GO:0000375	RNA splicing, via transesterification reactions	BP	1.23E-05	1.53E-06	5	8	461	27189
OVER	GO:0000377	RNA splicing, via transesterification reactions with bulged adenosine as nucleophile	BP	1.23E-05	1.53E-06	5	8	461	27189
OVER	GO:0000387	spliceosomal snRNP assembly	BP	4.34E-02	1.68E-02	1	0	465	27197
OVER	GO:0000398	mRNA splicing, via spliceosome	BP	1.23E-05	1.53E-06	5	8	461	27189
OVER	GO:0000413	protein peptidyl-prolyl isomerization	BP	1.35E-03	2.83E-04	2	0	464	27197
OVER	GO:0000741	karyogamy	BP	4.34E-02	1.68E-02	1	0	465	27197
OVER	GO:0000790	nuclear chromatin	CT	1.35E-03	2.83E-04	2	0	464	27197
OVER	GO:0000791	euchromatin	CT	4.34E-02	1.68E-02	1	0	465	27197
OVER	GO:0000819	sister chromatid segregation	BP	7.31E-03	1.66E-03	2	2	464	27195
OVER	GO:0000904	cell morphogenesis involved in differentiation	BP	1.35E-03	2.83E-04	2	0	464	27197

Over/Under*	GO ID	GO Name	GO Category$	FDR	P-value	Test	Ref	Non Annot Test	Non Annot Reference
OVER	GO:0000911	cytokinesis by cell plate formation	BP	4.34E-02	1.68E-02	1	0	465	27197
OVER	GO:0000919	cell plate assembly	BP	4.34E-02	1.68E-02	1	0	465	27197
OVER	GO:0001101	response to acid chemical	BP	4.88E-14	1.58E-15	10	4	456	27193
OVER	GO:0001676	long-chain fatty acid metabolic process	BP	4.34E-02	1.68E-02	1	0	465	27197
OVER	GO:0001736	establishment of planar polarity	BP	4.34E-02	1.68E-02	1	0	465	27197
OVER	GO:0001738	morphogenesis of a polarized epithelium	BP	4.34E-02	1.68E-02	1	0	465	27197
OVER	GO:0001871	pattern binding	MF	1.75E-03	3.70E-04	3	6	463	27191
OVER	GO:0001882	nucleoside binding	MF	1.29E-09	7.63E-11	93	2670	373	24527
OVER	GO:0001883	purine nucleoside binding	MF	5.32E-09	3.52E-10	91	2662	375	24535
OVER	GO:0002009	morphogenesis of an epithelium	BP	4.34E-02	1.68E-02	1	0	465	27197
OVER	GO:0002161**	aminoacyl-tRNA editing activity*	MF	4.34E-02	1.68E-02	1	0	465	27197
OVER	GO:0002213	defense response to insect	BP	4.34E-02	1.68E-02	1	0	465	27197
OVER	GO:0002218	activation of innate immune response	BP	4.34E-02	1.68E-02	1	0	465	27197
OVER	GO:0002237	response to molecule of bacterial origin	BP	4.34E-02	1.68E-02	1	0	465	27197
OVER	GO:0002252	immune effector process	BP	1.35E-03	2.83E-04	2	0	464	27197
OVER	GO:0002253	activation of immune response	BP	4.34E-02	1.68E-02	1	0	465	27197
OVER	GO:0002376	immune system process	BP	1.15E-03	2.03E-04	9	111	457	27086
OVER	GO:0002682	regulation of immune system process	BP	7.35E-07	7.95E-08	4	0	462	27197
OVER	GO:0002684	positive regulation of immune system process	BP	4.34E-02	1.68E-02	1	0	465	27197
OVER	GO:0003002	regionalization	BP	1.69E-08	1.33E-09	5	0	461	27197
OVER	GO:0003006	developmental process involved in reproduction	BP	2.66E-38	2.22E-40	23	1	443	27196
OVER	GO:0003676	nucleic acid binding	MF	7.97E-13	2.93E-14	92	2267	374	24930
OVER	GO:0003677**	DNA binding*	MF	8.13E-05	1.23E-05	49	1464	417	25733
OVER	GO:0003723	RNA binding	MF	1.67E-05	2.07E-06	21	356	445	26841
OVER	GO:0003774	motor activity	MF	4.34E-02	1.59E-02	5	82	461	27115
OVER	GO:0003824	catalytic activity	MF	1.89E-26	2.60E-28	244	7554	222	19643
OVER	GO:0003863	3-methyl-2-oxobutanoate dehydrogenase (2-methylpropanoyl-transferring) activity	MF	4.34E-02	1.68E-02	1	0	465	27197
OVER	GO:0003887	DNA-directed DNA polymerase activity	MF	2.93E-02	7.39E-03	3	21	463	27176
OVER	GO:0003899	DNA-directed 5'-3' RNA polymerase activity	MF	8.82E-15	2.64E-16	17	46	449	27151
OVER	GO:0003950	NAD+ ADP-ribosyltransferase activity	MF	1.70E-02	4.06E-03	2	4	464	27193
OVER	GO:0003954	NADH dehydrogenase activity	MF	1.97E-08	1.59E-09	8	14	458	27183
OVER	GO:0003973	(S)-2-hydroxy-acid oxidase activity	MF	1.35E-03	2.83E-04	2	0	464	27197
OVER	GO:0003995	acyl-CoA dehydrogenase activity	MF	4.34E-02	1.41E-02	2	9	464	27188
OVER	GO:0003997	acyl-CoA oxidase activity	MF	2.28E-02	5.62E-03	2	5	464	27192
OVER	GO:0003998	acylphosphatase activity	MF	4.34E-02	1.68E-02	1	0	465	27197
OVER	GO:0004012	phospholipid-translocating ATPase activity	MF	4.34E-02	1.68E-02	1	0	465	27197
OVER	GO:0004069**	L-aspartate:2-oxoglutarate aminotransferase activity*	MF	4.34E-02	1.68E-02	1	0	465	27197

Over/Under*	GO ID	GO Name	GO Category$	FDR	P-value	Test	Ref	Non Annot Test	Non Annot Reference
OVER	GO:0004073**	aspartate-semialdehyde dehydrogenase activity*	MF	4.34E-02	1.68E-02	1	0	465	27197
OVER	GO:0004088	carbamoyl-phosphate synthase (glutamine-hydrolyzing) activity	MF	4.34E-02	1.68E-02	1	0	465	27197
OVER	GO:0004105**	choline-phosphate cytidylyltransferase activity*	MF	4.34E-02	1.68E-02	1	0	465	27197
OVER	GO:0004124**	cysteine synthase activity*	MF	4.34E-02	1.68E-02	1	0	465	27197
OVER	GO:0004301**	epoxide hydrolase activity*	MF	4.34E-02	1.68E-02	1	0	465	27197
OVER	GO:0004310	farnesyl-diphosphate farnesyltransferase activity	MF	4.34E-02	1.68E-02	1	0	465	27197
OVER	GO:0004311	farnesyltranstransferase activity	MF	4.34E-02	1.68E-02	1	0	465	27197
OVER	GO:0004338**	glucan exo-1,3-beta-glucosidase activity*	MF	4.34E-02	1.68E-02	1	0	465	27197
OVER	GO:0004364**	glutathione transferase activity*	MF	4.34E-02	1.68E-02	1	0	465	27197
OVER	GO:0004365**	glyceraldehyde-3-phosphate dehydrogenase (NAD+) (phosphorylating) activity*	MF	4.34E-02	1.68E-02	1	0	465	27197
OVER	GO:0004441	inositol-1,4-bisphosphate 1-phosphatase activity	MF	4.34E-02	1.68E-02	1	0	465	27197
OVER	GO:0004467	long-chain fatty acid-CoA ligase activity	MF	4.34E-02	1.68E-02	1	0	465	27197
OVER	GO:0004497	monooxygenase activity	MF	1.81E-04	2.91E-05	7	46	459	27151
OVER	GO:0004506	squalene monooxygenase activity	MF	3.68E-02	9.43E-03	2	7	464	27190
OVER	GO:0004518	nuclease activity	MF	2.63E-04	4.29E-05	10	112	456	27085
OVER	GO:0004523**	RNA-DNA hybrid ribonuclease activity*	MF	3.68E-02	9.43E-03	2	7	464	27190
OVER	GO:0004527	exonuclease activity	MF	1.09E-04	1.67E-05	6	27	460	27170
OVER	GO:0004540	ribonuclease activity	MF	3.39E-02	8.62E-03	4	44	462	27153
OVER	GO:0004577**	N-acetylglucosaminyldiphosphodolichol N-acetylglucosaminyltransferase activity*	MF	4.34E-02	1.68E-02	1	0	465	27197
OVER	GO:0004674	protein serine/threonine kinase activity	MF	5.76E-08	4.96E-09	10	37	456	27160
OVER	GO:0004697	protein kinase C activity	MF	4.34E-02	1.68E-02	1	0	465	27197
OVER	GO:0004702	signal transducer, downstream of receptor, with serine/threonine kinase activity	MF	4.34E-02	1.68E-02	1	0	465	27197
OVER	GO:0004707	MAP kinase activity	MF	4.34E-02	1.68E-02	1	0	465	27197
OVER	GO:0004713	protein tyrosine kinase activity	MF	3.27E-05	4.75E-06	3	0	463	27197
OVER	GO:0004714	transmembrane receptor protein tyrosine kinase activity	MF	4.34E-02	1.68E-02	1	0	465	27197
OVER	GO:0004715	non-membrane spanning protein tyrosine kinase activity	MF	1.35E-03	2.83E-04	2	0	464	27197
OVER	GO:0004805	trehalose-phosphatase activity	MF	4.34E-02	1.68E-02	1	0	465	27197
OVER	GO:0004823	leucine-tRNA ligase activity	MF	4.34E-02	1.68E-02	1	0	465	27197
OVER	GO:0004854	xanthine dehydrogenase activity	MF	4.34E-02	1.68E-02	1	0	465	27197
OVER	GO:0005057	signal transducer activity, downstream of receptor	MF	4.34E-02	1.68E-02	1	0	465	27197
OVER	GO:0005215	transporter activity	MF	7.35E-07	7.63E-08	38	821	428	26376
OVER	GO:0005216**	ion channel activity*	MF	1.65E-02	3.91E-03	5	57	461	27140
OVER	GO:0005385	zinc ion transmembrane transporter activity	MF	4.34E-02	1.68E-02	1	0	465	27197
OVER	GO:0005388**	calcium-transporting ATPase activity*	MF	4.34E-02	1.68E-02	1	0	465	27197
OVER	GO:0005484**	SNAP receptor activity*	MF	4.34E-02	1.68E-02	1	0	465	27197

Over/Under*	GO ID	GO Name	GO Category$	FDR	P-value	Test	Ref	Non Annot Test	Non Annot Reference
OVER	GO:0005488	binding	MF	4.05E-38	3.76E-40	289	8635	177	18562
UNDER	GO:0005515	protein binding	MF	2.21E-02	5.35E-03	30	2796	436	24401
OVER	GO:0005524	ATP binding	MF	3.27E-05	4.38E-06	73	2443	393	24754
OVER	GO:0005548	phospholipid transporter activity	MF	4.34E-02	1.68E-02	1	0	465	27197
OVER	GO:0005576**	extracellular region*	CT	3.62E-04	6.13E-05	8	72	458	27125
OVER	GO:0005622	intracellular	CT	3.43E-72	1.44E-74	192	2457	274	24740
OVER	GO:0005623	cell	CT	2.50E-72	9.74E-75	198	2630	268	24567
OVER	GO:0005634	nucleus	CT	3.03E-10	1.52E-11	44	774	422	26423
OVER	GO:0005657	replication fork	CT	1.35E-03	2.50E-04	3	5	463	27192
OVER	GO:0005681**	spliceosomal complex*	CT	2.17E-04	3.53E-05	4	8	462	27189
OVER	GO:0005694	chromosome	CT	2.14E-02	5.18E-03	6	88	460	27109
OVER	GO:0005719**	nuclear euchromatin*	CT	4.34E-02	1.68E-02	1	0	465	27197
OVER	GO:0005737	cytoplasm	CT	3.46E-75	1.14E-77	135	973	331	26224
OVER	GO:0005739	mitochondrion	CT	5.43E-13	1.96E-14	18	75	448	27122
OVER	GO:0005773	vacuole	CT	1.08E-07	9.52E-09	6	5	460	27192
OVER	GO:0005774**	vacuolar membrane*	CT	2.70E-06	3.12E-07	5	5	461	27192
OVER	GO:0005777**	peroxisome*	CT	1.38E-04	2.16E-05	5	16	461	27181
OVER	GO:0005794	Golgi apparatus	CT	4.09E-04	6.94E-05	6	36	460	27161
OVER	GO:0005802**	trans-Golgi network*	CT	3.27E-05	4.75E-06	3	0	463	27197
OVER	GO:0005829**	cytosol*	CT	7.71E-09	5.26E-10	9	19	457	27178
OVER	GO:0005856	cytoskeleton	CT	3.31E-04	5.60E-05	8	71	458	27126
OVER	GO:0005871**	kinesin complex*	CT	1.35E-03	2.83E-04	2	0	464	27197
OVER	GO:0005875	microtubule associated complex	CT	4.34E-02	1.67E-02	2	10	464	27187
OVER	GO:0005885**	Arp2/3 protein complex*	CT	4.34E-02	1.68E-02	1	0	465	27197
OVER	GO:0005886	plasma membrane	CT	6.02E-21	1.15E-22	22	46	444	27151
OVER	GO:0005911	cell-cell junction	CT	1.20E-18	2.76E-20	11	0	455	27197
OVER	GO:0005938	cell cortex	CT	4.34E-02	1.25E-02	3	26	463	27171
OVER	GO:0005975	carbohydrate metabolic process	BP	6.71E-03	1.51E-03	24	684	442	26513
OVER	GO:0005976	polysaccharide metabolic process	BP	3.73E-02	9.58E-03	8	166	458	27031
OVER	GO:0005996	monosaccharide metabolic process	BP	3.83E-03	8.39E-04	5	39	461	27158
OVER	GO:0006006	glucose metabolic process	BP	7.12E-04	1.23E-04	4	12	462	27185
OVER	GO:0006066	alcohol metabolic process	BP	4.34E-02	1.51E-02	3	28	463	27169
OVER	GO:0006082	organic acid metabolic process	BP	2.03E-07	1.89E-08	26	398	440	26799
OVER	GO:0006091	generation of precursor metabolites and energy	BP	4.17E-12	1.66E-13	20	116	446	27081
OVER	GO:0006094	gluconeogenesis	BP	2.93E-02	7.41E-03	2	6	464	27191
OVER	GO:0006119	oxidative phosphorylation	BP	2.97E-06	3.46E-07	6	12	460	27185
OVER	GO:0006139	nucleobase-containing compound metabolic process	BP	1.52E-22	2.40E-24	106	2036	360	25161
OVER	GO:0006163	purine nucleotide metabolic process	BP	1.40E-08	1.00E-09	17	136	449	27061
OVER	GO:0006164	purine nucleotide biosynthetic process	BP	2.89E-05	3.78E-06	9	64	457	27133
OVER	GO:0006259	DNA metabolic process	BP	4.70E-05	6.92E-06	17	265	449	26932
OVER	GO:0006260	DNA replication	BP	1.41E-03	2.96E-04	7	69	459	27128
OVER	GO:0006261	DNA-dependent DNA replication	BP	7.88E-06	9.54E-07	5	7	461	27190

Over/Under*	GO ID	GO Name	GO Category$	FDR	P-value	Test	Ref	Non Annot Test	Non Annot Reference
OVER	GO:0006275	regulation of DNA replication	BP	2.93E-02	7.41E-03	2	6	464	27191
OVER	GO:0006304	DNA modification	BP	4.34E-02	1.17E-02	2	8	464	27189
OVER	GO:0006305	DNA alkylation	BP	4.34E-02	1.17E-02	2	8	464	27189
OVER	GO:0006306	DNA methylation	BP	4.34E-02	1.17E-02	2	8	464	27189
OVER	GO:0006312	mitotic recombination	BP	4.34E-02	1.68E-02	1	0	465	27197
OVER	GO:0006325	chromatin organization	BP	1.48E-04	2.34E-05	9	82	457	27115
OVER	GO:0006346	methylation-dependent chromatin silencing	BP	4.34E-02	1.68E-02	1	0	465	27197
OVER	GO:0006351	transcription, DNA-templated	BP	6.32E-06	7.62E-07	49	1321	417	25876
OVER	GO:0006355	regulation of transcription, DNA-templated	BP	4.34E-02	1.26E-02	33	1216	433	25981
OVER	GO:0006383	transcription from RNA polymerase III promoter	BP	7.31E-03	1.66E-03	2	2	464	27195
OVER	GO:0006384	transcription initiation from RNA polymerase III promoter	BP	1.35E-03	2.83E-04	2	0	464	27197
OVER	GO:0006396	RNA processing	BP	4.17E-08	3.53E-09	17	149	449	27048
OVER	GO:0006397	mRNA processing	BP	7.22E-10	4.10E-11	11	29	455	27168
OVER	GO:0006399	tRNA metabolic process	BP	3.45E-02	8.78E-03	6	99	460	27098
OVER	GO:0006417	regulation of translation	BP	2.93E-02	7.41E-03	2	6	464	27191
OVER	GO:0006429	leucyl-tRNA aminoacylation	BP	4.34E-02	1.68E-02	1	0	465	27197
OVER	GO:0006461	protein complex assembly	BP	3.13E-04	5.23E-05	6	34	460	27163
OVER	GO:0006464	cellular protein modification process	BP	4.15E-08	3.50E-09	60	1513	406	25684
OVER	GO:0006470	protein dephosphorylation	BP	4.67E-03	1.03E-03	4	23	462	27174
OVER	GO:0006473	protein acetylation	BP	3.27E-05	4.75E-06	3	0	463	27197
OVER	GO:0006475	internal protein amino acid acetylation	BP	3.27E-05	4.75E-06	3	0	463	27197
OVER	GO:0006486	protein glycosylation	BP	2.86E-05	3.72E-06	8	47	458	27150
OVER	GO:0006520	cellular amino acid metabolic process	BP	3.72E-04	6.31E-05	14	226	452	26971
OVER	GO:0006534	cysteine metabolic process	BP	8.41E-09	5.79E-10	7	6	459	27191
OVER	GO:0006535	cysteine biosynthetic process from serine	BP	1.75E-03	3.70E-04	3	6	463	27191
OVER	GO:0006563	L-serine metabolic process	BP	1.60E-02	3.77E-03	3	16	463	27181
OVER	GO:0006566	threonine metabolic process	BP	4.34E-02	1.68E-02	1	0	465	27197
OVER	GO:0006605	protein targeting	BP	8.19E-12	3.33E-13	13	32	453	27165
OVER	GO:0006606	protein import into nucleus	BP	2.93E-02	7.41E-03	2	6	464	27191
OVER	GO:0006612	protein targeting to membrane	BP	3.42E-05	4.98E-06	5	11	461	27186
OVER	GO:0006623	protein targeting to vacuole	BP	4.34E-02	1.68E-02	1	0	465	27197
OVER	GO:0006625	protein targeting to peroxisome	BP	7.35E-07	7.95E-08	4	0	462	27197
OVER	GO:0006631	fatty acid metabolic process	BP	4.34E-02	1.38E-02	5	79	461	27118
OVER	GO:0006635	fatty acid beta-oxidation	BP	1.35E-03	2.50E-04	3	5	463	27192
OVER	GO:0006656	phosphatidylcholine biosynthetic process	BP	4.34E-02	1.68E-02	1	0	465	27197
OVER	GO:0006714	sesquiterpenoid metabolic process	BP	4.34E-02	1.68E-02	1	0	465	27197
OVER	GO:0006720	isoprenoid metabolic process	BP	4.58E-02	1.78E-02	3	30	463	27167
OVER	GO:0006723	cuticle hydrocarbon biosynthetic process	BP	4.34E-02	1.68E-02	1	0	465	27197
OVER	GO:0006725	cellular aromatic compound metabolic process	BP	8.23E-24	1.26E-25	111	2128	355	25069
OVER	GO:0006753	nucleoside phosphate metabolic process	BP	1.16E-07	1.04E-08	18	183	448	27014

Over/Under*	GO ID	GO Name	GO Category$	FDR	P-value	Test	Ref	Non Annot Test	Non Annot Reference
OVER	GO:0006754	ATP biosynthetic process	BP	3.25E-05	4.27E-06	8	48	458	27149
OVER	GO:0006778	porphyrin-containing compound metabolic process	BP	4.34E-02	1.25E-02	3	26	463	27171
OVER	GO:0006779	porphyrin-containing compound biosynthetic process	BP	3.27E-02	8.29E-03	3	22	463	27175
OVER	GO:0006782	protoporphyrinogen IX biosynthetic process	BP	4.34E-02	1.68E-02	1	0	465	27197
OVER	GO:0006783	heme biosynthetic process	BP	1.75E-03	3.70E-04	3	6	463	27191
OVER	GO:0006790	sulfur compound metabolic process	BP	2.89E-05	3.78E-06	9	64	457	27133
OVER	GO:0006793	phosphorus metabolic process	BP	1.54E-06	1.73E-07	58	1599	408	25598
OVER	GO:0006796	phosphate-containing compound metabolic process	BP	1.51E-06	1.69E-07	58	1596	408	25601
OVER	GO:0006807	nitrogen compound metabolic process	BP	3.06E-24	4.40E-26	130	2779	336	24418
OVER	GO:0006810	transport	BP	1.24E-19	2.63E-21	79	1326	387	25871
OVER	GO:0006811	ion transport	BP	3.35E-07	3.16E-08	26	409	440	26788
OVER	GO:0006812	cation transport	BP	2.61E-05	3.32E-06	20	337	446	26860
OVER	GO:0006816	calcium ion transport	BP	1.35E-03	2.83E-04	2	0	464	27197
OVER	GO:0006818	hydrogen transport	BP	7.55E-07	8.21E-08	10	52	456	27145
OVER	GO:0006829	zinc II ion transport	BP	1.35E-03	2.83E-04	2	0	464	27197
OVER	GO:0006869	lipid transport	BP	3.27E-03	7.09E-04	3	8	463	27189
OVER	GO:0006873	cellular ion homeostasis	BP	2.28E-02	5.62E-03	2	5	464	27192
OVER	GO:0006875	cellular metal ion homeostasis	BP	2.28E-02	5.62E-03	2	5	464	27192
OVER	GO:0006878	cellular copper ion homeostasis	BP	4.34E-02	1.68E-02	1	0	465	27197
OVER	GO:0006886	intracellular protein transport	BP	8.62E-11	4.18E-12	20	141	446	27056
OVER	GO:0006888	ER to Golgi vesicle-mediated transport	BP	1.38E-04	2.16E-05	5	16	461	27181
OVER	GO:0006891	intra-Golgi vesicle-mediated transport	BP	1.18E-02	2.74E-03	2	3	464	27194
OVER	GO:0006913	nucleocytoplasmic transport	BP	4.34E-02	1.17E-02	2	8	464	27189
OVER	GO:0006950	response to stress	BP	9.19E-07	1.01E-07	29	528	437	26669
OVER	GO:0006952	defense response	BP	3.58E-05	5.22E-06	14	178	452	27019
OVER	GO:0006955	immune response	BP	1.15E-03	2.03E-04	9	111	457	27086
OVER	GO:0006970	response to osmotic stress	BP	8.49E-12	3.68E-13	7	0	459	27197
OVER	GO:0006996	organelle organization	BP	3.55E-11	1.65E-12	22	169	444	27028
OVER	GO:0006997	nucleus organization	BP	4.34E-02	1.68E-02	1	0	465	27197
OVER	GO:0007010	cytoskeleton organization	BP	7.67E-03	1.74E-03	4	27	462	27170
OVER	GO:0007020	microtubule nucleation	BP	3.83E-03	8.40E-04	2	1	464	27196
OVER	GO:0007031	peroxisome organization	BP	2.17E-04	3.53E-05	4	8	462	27189
OVER	GO:0007049	cell cycle	BP	1.35E-03	2.60E-04	6	47	460	27150
OVER	GO:0007059	chromosome segregation	BP	1.70E-02	4.06E-03	2	4	464	27193
OVER	GO:0007062	sister chromatid cohesion	BP	1.35E-03	2.83E-04	2	0	464	27197
OVER	GO:0007127	meiosis I	BP	4.34E-02	1.68E-02	1	0	465	27197
OVER	GO:0007129	synapsis	BP	4.34E-02	1.68E-02	1	0	465	27197
OVER	GO:0007154	cell communication	BP	1.01E-05	1.23E-06	26	500	440	26697
OVER	GO:0007164	establishment of tissue polarity	BP	4.34E-02	1.68E-02	1	0	465	27197
OVER	GO:0007165	signal transduction	BP	4.77E-06	5.67E-07	24	416	442	26781
OVER	GO:0007166	cell surface receptor signaling pathway	BP	1.35E-03	2.83E-04	2	0	464	27197

**Over/Under***	**GO ID**	**GO Name**	**GO Category$**	**FDR**	**P-value**	**Test**	**Ref**	**Non Annot Test**	**Non Annot Reference**
OVER	GO:0007215	glutamate receptor signaling pathway	BP	1.35E-03	2.83E-04	2	0	464	27197
OVER	GO:0007267	cell-cell signaling	BP	4.34E-02	1.68E-02	1	0	465	27197
OVER	GO:0007275	multicellular organism development	BP	4.34E-41	3.37E-43	31	18	435	27179
OVER	GO:0007276	gamete generation	BP	1.35E-03	2.83E-04	2	0	464	27197
OVER	GO:0007292	female gamete generation	BP	1.35E-03	2.83E-04	2	0	464	27197
OVER	GO:0007389	pattern specification process	BP	4.00E-10	2.21E-11	6	0	460	27197
OVER	GO:0007568	aging	BP	1.35E-03	2.83E-04	2	0	464	27197
OVER	GO:0008017	microtubule binding	MF	7.31E-03	1.66E-03	2	2	464	27195
OVER	GO:0008023	transcription elongation factor complex	CT	4.34E-02	1.68E-02	1	0	465	27197
OVER	GO:0008033	tRNA processing	BP	4.34E-02	1.38E-02	4	51	462	27146
OVER	GO:0008092	cytoskeletal protein binding	MF	4.58E-02	1.78E-02	3	30	463	27167
OVER	GO:0008104	protein localization	BP	1.11E-11	4.89E-13	25	215	441	26982
OVER	GO:0008134	transcription factor binding	MF	6.70E-03	1.51E-03	3	11	463	27186
OVER	GO:0008137**	NADH dehydrogenase (ubiquinone) activity*	MF	1.97E-08	1.59E-09	8	14	458	27183
OVER	GO:0008152	metabolic process	BP	3.54E-61	1.80E-63	315	8006	151	19191
OVER	GO:0008184**	glycogen phosphorylase activity*	MF	4.34E-02	1.68E-02	1	0	465	27197
OVER	GO:0008270	zinc ion binding	MF	2.39E-12	9.15E-14	41	575	425	26622
OVER	GO:0008299	isoprenoid biosynthetic process	BP	4.58E-02	1.78E-02	3	30	463	27167
OVER	GO:0008324	cation transmembrane transporter activity	MF	8.06E-06	9.79E-07	17	227	449	26970
OVER	GO:0008374	O-acyltransferase activity	MF	6.58E-04	1.13E-04	5	24	461	27173
OVER	GO:0008380	RNA splicing	BP	1.70E-14	5.19E-16	12	11	454	27186
OVER	GO:0008408	3'-5' exonuclease activity	MF	1.81E-02	4.37E-03	3	17	463	27180
OVER	GO:0008422	beta-glucosidase activity	MF	1.35E-03	2.83E-04	2	0	464	27197
OVER	GO:0008441	3'(2'),5'-bisphosphate nucleotidase activity	MF	4.34E-02	1.68E-02	1	0	465	27197
OVER	GO:0008442	3-hydroxyisobutyrate dehydrogenase activity	MF	4.34E-02	1.68E-02	1	0	465	27197
OVER	GO:0008447	L-ascorbate oxidase activity	MF	4.34E-02	1.68E-02	1	0	465	27197
OVER	GO:0008536	Ran GTPase binding	MF	1.35E-03	2.83E-04	2	0	464	27197
OVER	GO:0008551**	cadmium-exporting ATPase activity*	MF	4.34E-02	1.68E-02	1	0	465	27197
OVER	GO:0008559**	xenobiotic-transporting ATPase activity*	MF	4.34E-02	1.68E-02	1	0	465	27197
OVER	GO:0008568	microtubule-severing ATPase activity	MF	4.00E-10	2.21E-11	6	0	460	27197
OVER	GO:0008622**	epsilon DNA polymerase complex*	CT	4.34E-02	1.68E-02	1	0	465	27197
OVER	GO:0008652	cellular amino acid biosynthetic process	BP	4.60E-04	7.84E-05	9	97	457	27100
OVER	GO:0008670**	2,4-dienoyl-CoA reductase (NADPH) activity*	MF	1.35E-03	2.83E-04	2	0	464	27197
OVER	GO:0008756**	o-succinylbenzoate-CoA ligase activity*	MF	4.34E-02	1.68E-02	1	0	465	27197
OVER	GO:0008859	exoribonuclease II activity	MF	4.34E-02	1.68E-02	1	0	465	27197
OVER	GO:0008905	mannose-phosphate guanylyltransferase activity	MF	4.34E-02	1.68E-02	1	0	465	27197
OVER	GO:0008928**	mannose-1-phosphate guanylyltransferase (GDP) activity*	MF	4.34E-02	1.68E-02	1	0	465	27197
OVER	GO:0009001**	serine O-acetyltransferase activity*	MF	2.93E-02	7.41E-03	2	6	464	27191

Over/Under*	GO ID	GO Name	GO Category$	FDR	P-value	Test	Ref	Non Annot Test	Non Annot Reference
OVER	GO:0009011	starch synthase activity	MF	4.34E-02	1.68E-02	1	0	465	27197
OVER	GO:0009056	catabolic process	BP	7.92E-03	1.81E-03	12	251	454	26946
OVER	GO:0009058	biosynthetic process	BP	7.51E-14	2.47E-15	106	2732	360	24465
OVER	GO:0009059	macromolecule biosynthetic process	BP	9.32E-09	6.44E-10	76	2057	390	25140
OVER	GO:0009062	fatty acid catabolic process	BP	1.35E-03	2.50E-04	3	5	463	27192
OVER	GO:0009069	serine family amino acid metabolic process	BP	8.84E-07	9.64E-08	8	27	458	27170
OVER	GO:0009070	serine family amino acid biosynthetic process	BP	7.11E-08	6.19E-09	7	10	459	27187
OVER	GO:0009088	threonine biosynthetic process	BP	4.34E-02	1.68E-02	1	0	465	27197
OVER	GO:0009100	glycoprotein metabolic process	BP	2.86E-05	3.72E-06	8	47	458	27150
OVER	GO:0009101	glycoprotein biosynthetic process	BP	2.86E-05	3.72E-06	8	47	458	27150
OVER	GO:0009116	nucleoside metabolic process	BP	4.54E-07	4.36E-08	16	157	450	27040
OVER	GO:0009117	nucleotide metabolic process	BP	7.39E-08	6.45E-09	18	177	448	27020
OVER	GO:0009119	ribonucleoside metabolic process	BP	8.60E-08	7.54E-09	16	137	450	27060
OVER	GO:0009123	nucleoside monophosphate metabolic process	BP	2.75E-08	2.28E-09	16	125	450	27072
OVER	GO:0009124	nucleoside monophosphate biosynthetic process	BP	1.47E-04	2.31E-05	8	62	458	27135
OVER	GO:0009126	purine nucleoside monophosphate metabolic process	BP	1.88E-08	1.49E-09	16	121	450	27076
OVER	GO:0009127	purine nucleoside monophosphate biosynthetic process	BP	9.74E-05	1.49E-05	8	58	458	27139
OVER	GO:0009141	nucleoside triphosphate metabolic process	BP	1.22E-08	8.57E-10	16	116	450	27081
OVER	GO:0009142	nucleoside triphosphate biosynthetic process	BP	4.88E-05	7.25E-06	8	52	458	27145
OVER	GO:0009144	purine nucleoside triphosphate metabolic process	BP	1.09E-08	7.65E-10	16	115	450	27082
OVER	GO:0009145	purine nucleoside triphosphate biosynthetic process	BP	4.88E-05	7.25E-06	8	52	458	27145
OVER	GO:0009150	purine ribonucleotide metabolic process	BP	6.54E-08	5.65E-09	16	134	450	27063
OVER	GO:0009152	purine ribonucleotide biosynthetic process	BP	1.47E-04	2.31E-05	8	62	458	27135
OVER	GO:0009156	ribonucleoside monophosphate biosynthetic process	BP	1.21E-04	1.86E-05	8	60	458	27137
OVER	GO:0009161	ribonucleoside monophosphate metabolic process	BP	2.25E-08	1.85E-09	16	123	450	27074
OVER	GO:0009163	nucleoside biosynthetic process	BP	1.95E-04	3.15E-05	8	65	458	27132
OVER	GO:0009165	nucleotide biosynthetic process	BP	1.87E-05	2.33E-06	10	78	456	27119
OVER	GO:0009167	purine ribonucleoside monophosphate metabolic process	BP	1.88E-08	1.49E-09	16	121	450	27076
OVER	GO:0009168	purine ribonucleoside monophosphate biosynthetic process	BP	9.74E-05	1.49E-05	8	58	458	27139
OVER	GO:0009199	ribonucleoside triphosphate metabolic process	BP	1.09E-08	7.65E-10	16	115	450	27082
OVER	GO:0009201	ribonucleoside triphosphate biosynthetic process	BP	4.88E-05	7.25E-06	8	52	458	27145
OVER	GO:0009205	purine ribonucleoside triphosphate metabolic process	BP	1.09E-08	7.65E-10	16	115	450	27082
OVER	GO:0009206	purine ribonucleoside triphosphate biosynthetic process	BP	4.88E-05	7.25E-06	8	52	458	27145

Over/Under*	GO ID	GO Name	GO Category[$]	FDR	P-value	Test	Ref	Non Annot Test	Non Annot Reference
OVER	GO:0009259	ribonucleotide metabolic process	BP	1.40E-08	1.00E-09	17	136	449	27061
OVER	GO:0009260	ribonucleotide biosynthetic process	BP	2.89E-05	3.78E-06	9	64	457	27133
OVER	GO:0009266	response to temperature stimulus	BP	1.69E-08	1.33E-09	5	0	461	27197
OVER	GO:0009314	response to radiation	BP	6.79E-04	1.17E-04	6	40	460	27157
OVER	GO:0009403	toxin biosynthetic process	BP	4.34E-02	1.68E-02	1	0	465	27197
OVER	GO:0009404	toxin metabolic process	BP	4.34E-02	1.68E-02	1	0	465	27197
OVER	GO:0009408	response to heat	BP	3.27E-05	4.75E-06	3	0	463	27197
OVER	GO:0009409	response to cold	BP	1.35E-03	2.83E-04	2	0	464	27197
OVER	GO:0009416	response to light stimulus	BP	6.79E-04	1.17E-04	6	40	460	27157
OVER	GO:0009503	thylakoid light-harvesting complex	CT	4.34E-02	1.68E-02	1	0	465	27197
OVER	GO:0009505	plant-type cell wall	CT	3.27E-05	4.75E-06	3	0	463	27197
OVER	GO:0009506**	plasmodesma*	CT	1.20E-18	2.76E-20	11	0	455	27197
OVER	GO:0009507	chloroplast	CT	5.41E-135	3.23E-138	89	24	377	27173
OVER	GO:0009517**	PSII associated light-harvesting complex II*	CT	4.34E-02	1.68E-02	1	0	465	27197
OVER	GO:0009521	photosystem	CT	1.38E-08	9.81E-10	12	54	454	27143
OVER	GO:0009522**	photosystem I*	CT	5.85E-06	7.02E-07	6	14	460	27183
OVER	GO:0009523**	photosystem II*	CT	1.99E-03	4.21E-04	5	33	461	27164
OVER	GO:0009524**	phragmoplast*	CT	3.27E-05	4.75E-06	3	0	463	27197
OVER	GO:0009526	plastid envelope	CT	2.30E-20	4.54E-22	12	0	454	27197
OVER	GO:0009528	plastid inner membrane	CT	7.35E-07	7.95E-08	4	0	462	27197
OVER	GO:0009532	plastid stroma	CT	7.35E-07	7.95E-08	4	0	462	27197
OVER	GO:0009534	chloroplast thylakoid	CT	6.77E-54	3.85E-56	31	0	435	27197
OVER	GO:0009535	chloroplast thylakoid membrane	CT	3.71E-52	2.44E-54	30	0	436	27197
OVER	GO:0009536	plastid	CT	1.73E-138	5.16E-142	94	32	372	27165
OVER	GO:0009553	embryo sac development	BP	3.27E-05	4.75E-06	3	0	463	27197
OVER	GO:0009555	pollen development	BP	1.35E-03	2.83E-04	2	0	464	27197
OVER	GO:0009559	embryo sac central cell differentiation	BP	4.34E-02	1.68E-02	1	0	465	27197
OVER	GO:0009560	embryo sac egg cell differentiation	BP	1.35E-03	2.83E-04	2	0	464	27197
OVER	GO:0009561	megagametogenesis	BP	3.27E-05	4.75E-06	3	0	463	27197
OVER	GO:0009570**	chloroplast stroma*	CT	7.35E-07	7.95E-08	4	0	462	27197
OVER	GO:0009579	thylakoid	CT	1.06E-37	1.17E-39	36	57	430	27140
OVER	GO:0009605	response to external stimulus	BP	5.21E-18	1.25E-19	15	14	451	27183
OVER	GO:0009606	tropism	BP	7.35E-07	7.95E-08	4	0	462	27197
OVER	GO:0009607	response to biotic stimulus	BP	5.70E-11	2.71E-12	14	50	452	27147
OVER	GO:0009611	response to wounding	BP	7.22E-07	7.13E-08	5	3	461	27194
OVER	GO:0009615	response to virus	BP	4.34E-02	1.68E-02	1	0	465	27197
OVER	GO:0009616	virus induced gene silencing	BP	4.34E-02	1.68E-02	1	0	465	27197
OVER	GO:0009617	response to bacterium	BP	7.22E-07	7.13E-08	5	3	461	27194
OVER	GO:0009620	response to fungus	BP	7.36E-10	4.23E-11	7	3	459	27194
OVER	GO:0009626	plant-type hypersensitive response	BP	3.27E-05	4.75E-06	3	0	463	27197
OVER	GO:0009627	systemic acquired resistance	BP	4.34E-02	1.68E-02	1	0	465	27197
OVER	GO:0009628	response to abiotic stimulus	BP	1.76E-17	4.33E-19	19	44	447	27153

Over/Under*	GO ID	GO Name	GO Category$	FDR	P-value	Test	Ref	Non Annot Test	Non Annot Reference
OVER	GO:0009629	response to gravity	BP	7.35E-07	7.95E-08	4	0	462	27197
OVER	GO:0009630	gravitropism	BP	7.35E-07	7.95E-08	4	0	462	27197
OVER	GO:0009636	response to toxic substance	BP	4.34E-02	1.68E-02	1	0	465	27197
OVER	GO:0009637	response to blue light	BP	1.35E-03	2.83E-04	2	0	464	27197
OVER	GO:0009639	response to red or far red light	BP	3.27E-05	4.75E-06	3	0	463	27197
OVER	GO:0009640	photomorphogenesis	BP	4.34E-02	1.68E-02	1	0	465	27197
OVER	GO:0009642	response to light intensity	BP	4.34E-02	1.68E-02	1	0	465	27197
OVER	GO:0009648	photoperiodism	BP	4.34E-02	1.68E-02	1	0	465	27197
OVER	GO:0009651	response to salt stress	BP	1.69E-08	1.33E-09	5	0	461	27197
OVER	GO:0009653	anatomical structure morphogenesis	BP	1.63E-10	7.99E-12	11	24	455	27173
OVER	GO:0009657	plastid organization	BP	7.31E-03	1.66E-03	2	2	464	27195
OVER	GO:0009658	chloroplast organization	BP	1.35E-03	2.83E-04	2	0	464	27197
OVER	GO:0009663	plasmodesma organization	BP	4.34E-02	1.68E-02	1	0	465	27197
OVER	GO:0009682	induced systemic resistance	BP	4.34E-02	1.68E-02	1	0	465	27197
OVER	GO:0009687	abscisic acid metabolic process	BP	4.34E-02	1.68E-02	1	0	465	27197
OVER	GO:0009688	abscisic acid biosynthetic process	BP	4.34E-02	1.68E-02	1	0	465	27197
OVER	GO:0009694	jasmonic acid metabolic process	BP	1.69E-08	1.33E-09	5	0	461	27197
OVER	GO:0009695	jasmonic acid biosynthetic process	BP	3.27E-05	4.75E-06	3	0	463	27197
OVER	GO:0009696	salicylic acid metabolic process	BP	4.34E-02	1.68E-02	1	0	465	27197
OVER	GO:0009697	salicylic acid biosynthetic process	BP	4.34E-02	1.68E-02	1	0	465	27197
OVER	GO:0009700	indole phytoalexin biosynthetic process	BP	4.34E-02	1.68E-02	1	0	465	27197
OVER	GO:0009706**	chloroplast inner membrane*	CT	7.35E-07	7.95E-08	4	0	462	27197
OVER	GO:0009719	response to endogenous stimulus	BP	1.56E-12	5.93E-14	13	27	453	27170
OVER	GO:0009723	response to ethylene	BP	1.35E-03	2.83E-04	2	0	464	27197
OVER	GO:0009725	response to hormone	BP	1.56E-12	5.93E-14	13	27	453	27170
OVER	GO:0009735	response to cytokinin	BP	1.35E-03	2.83E-04	2	0	464	27197
OVER	GO:0009737	response to abscisic acid	BP	3.27E-05	4.75E-06	3	0	463	27197
OVER	GO:0009738	abscisic acid-activated signaling pathway	BP	4.34E-02	1.68E-02	1	0	465	27197
OVER	GO:0009743	response to carbohydrate	BP	3.27E-05	4.75E-06	3	0	463	27197
OVER	GO:0009744	response to sucrose	BP	4.34E-02	1.68E-02	1	0	465	27197
OVER	GO:0009751	response to salicylic acid	BP	3.27E-05	4.75E-06	3	0	463	27197
OVER	GO:0009753	response to jasmonic acid	BP	4.00E-10	2.21E-11	6	0	460	27197
OVER	GO:0009755	hormone-mediated signaling pathway	BP	1.69E-08	1.33E-09	5	0	461	27197
OVER	GO:0009756	carbohydrate mediated signaling	BP	1.35E-03	2.83E-04	2	0	464	27197
OVER	GO:0009767	photosynthetic electron transport chain	BP	7.11E-08	6.19E-09	7	10	459	27187
OVER	GO:0009772	photosynthetic electron transport in photosystem II	BP	7.22E-07	7.13E-08	5	3	461	27194
OVER	GO:0009787	regulation of abscisic acid-activated signaling pathway	BP	4.34E-02	1.68E-02	1	0	465	27197
OVER	GO:0009789	positive regulation of abscisic acid-activated signaling pathway	BP	4.34E-02	1.68E-02	1	0	465	27197
OVER	GO:0009790	embryo development	BP	1.50E-10	7.30E-12	8	5	458	27192
OVER	GO:0009791	post-embryonic development	BP	5.76E-38	5.86E-40	22	0	444	27197
OVER	GO:0009793	embryo development ending in seed dormancy	BP	8.49E-12	3.68E-13	7	0	459	27197

Over/Under*	GO ID	GO Name	GO Category$	FDR	P-value	Test	Ref	Non Annot Test	Non Annot Reference
OVER	GO:0009798	axis specification	BP	4.34E-02	1.68E-02	1	0	465	27197
OVER	GO:0009799	specification of symmetry	BP	4.34E-02	1.68E-02	1	0	465	27197
OVER	GO:0009814	defense response, incompatible interaction	BP	7.35E-07	7.95E-08	4	0	462	27197
OVER	GO:0009816	defense response to bacterium, incompatible interaction	BP	1.35E-03	2.83E-04	2	0	464	27197
OVER	GO:0009825	multidimensional cell growth	BP	4.34E-02	1.68E-02	1	0	465	27197
OVER	GO:0009826	unidimensional cell growth	BP	4.34E-02	1.68E-02	1	0	465	27197
OVER	GO:0009838	abscission	BP	4.34E-02	1.68E-02	1	0	465	27197
OVER	GO:0009845	seed germination	BP	7.35E-07	7.95E-08	4	0	462	27197
OVER	GO:0009846	pollen germination	BP	4.34E-02	1.68E-02	1	0	465	27197
OVER	GO:0009853	photorespiration	BP	4.34E-02	1.68E-02	1	0	465	27197
OVER	GO:0009855	determination of bilateral symmetry	BP	4.34E-02	1.68E-02	1	0	465	27197
OVER	GO:0009860	pollen tube growth	BP	4.34E-02	1.68E-02	1	0	465	27197
OVER	GO:0009861	jasmonic acid and ethylene-dependent systemic resistance	BP	4.34E-02	1.68E-02	1	0	465	27197
OVER	GO:0009863	salicylic acid mediated signaling pathway	BP	3.27E-05	4.75E-06	3	0	463	27197
OVER	GO:0009864	induced systemic resistance, jasmonic acid mediated signaling pathway	BP	4.34E-02	1.68E-02	1	0	465	27197
OVER	GO:0009867	jasmonic acid mediated signaling pathway	BP	7.35E-07	7.95E-08	4	0	462	27197
OVER	GO:0009871	jasmonic acid and ethylene-dependent systemic resistance, ethylene mediated signaling pathway	BP	4.34E-02	1.68E-02	1	0	465	27197
OVER	GO:0009873	ethylene-activated signaling pathway	BP	4.34E-02	1.68E-02	1	0	465	27197
OVER	GO:0009880	embryonic pattern specification	BP	4.34E-02	1.68E-02	1	0	465	27197
OVER	GO:0009887	animal organ morphogenesis	BP	4.34E-02	1.68E-02	1	0	465	27197
OVER	GO:0009888	tissue development	BP	6.50E-17	1.67E-18	10	0	456	27197
OVER	GO:0009889	regulation of biosynthetic process	BP	4.97E-03	1.10E-03	37	1228	429	25969
OVER	GO:0009890	negative regulation of biosynthetic process	BP	1.70E-02	4.06E-03	2	4	464	27193
OVER	GO:0009891	positive regulation of biosynthetic process	BP	3.03E-04	5.04E-05	4	9	462	27188
OVER	GO:0009892	negative regulation of metabolic process	BP	2.70E-06	3.12E-07	5	5	461	27192
OVER	GO:0009893	positive regulation of metabolic process	BP	3.03E-04	5.04E-05	4	9	462	27188
OVER	GO:0009902	chloroplast relocation	BP	4.34E-02	1.68E-02	1	0	465	27197
OVER	GO:0009908	flower development	BP	1.75E-13	6.11E-15	8	0	458	27197
OVER	GO:0009909	regulation of flower development	BP	7.35E-07	7.95E-08	4	0	462	27197
OVER	GO:0009911	positive regulation of flower development	BP	4.34E-02	1.68E-02	1	0	465	27197
OVER	GO:0009914	hormone transport	BP	4.34E-02	1.68E-02	1	0	465	27197
OVER	GO:0009926	auxin polar transport	BP	4.34E-02	1.68E-02	1	0	465	27197
OVER	GO:0009932	cell tip growth	BP	4.34E-02	1.68E-02	1	0	465	27197
OVER	GO:0009933	meristem structural organization	BP	3.27E-05	4.75E-06	3	0	463	27197
OVER	GO:0009941	chloroplast envelope	CT	1.20E-18	2.76E-20	11	0	455	27197
OVER	GO:0009943	adaxial/abaxial axis specification	BP	4.34E-02	1.68E-02	1	0	465	27197

Over/Under*	GO ID	GO Name	GO Category$	FDR	P-value	Test	Ref	Non Annot Test	Non Annot Reference
OVER	GO:0009944	polarity specification of adaxial/abaxial axis	BP	4.34E-02	1.68E-02	1	0	465	27197
OVER	GO:0009954	proximal/distal pattern formation	BP	4.34E-02	1.68E-02	1	0	465	27197
OVER	GO:0009955	adaxial/abaxial pattern specification	BP	4.34E-02	1.68E-02	1	0	465	27197
OVER	GO:0009967	positive regulation of signal transduction	BP	4.34E-02	1.68E-02	1	0	465	27197
OVER	GO:0009987	cellular process	BP	1.61E-47	1.11E-49	258	6277	208	20920
OVER	GO:0010014	meristem initiation	BP	1.35E-03	2.83E-04	2	0	464	27197
OVER	GO:0010015	root morphogenesis	BP	4.34E-02	1.68E-02	1	0	465	27197
OVER	GO:0010016	shoot system morphogenesis	BP	1.35E-03	2.83E-04	2	0	464	27197
OVER	GO:0010020	chloroplast fission	BP	4.34E-02	1.68E-02	1	0	465	27197
OVER	GO:0010026	trichome differentiation	BP	4.34E-02	1.68E-02	1	0	465	27197
OVER	GO:0010029	regulation of seed germination	BP	4.34E-02	1.68E-02	1	0	465	27197
OVER	GO:0010030	positive regulation of seed germination	BP	4.34E-02	1.68E-02	1	0	465	27197
OVER	GO:0010033	response to organic substance	BP	2.16E-16	5.69E-18	16	27	450	27170
OVER	GO:0010051	xylem and phloem pattern formation	BP	1.35E-03	2.83E-04	2	0	464	27197
OVER	GO:0010053	root epidermal cell differentiation	BP	4.34E-02	1.68E-02	1	0	465	27197
OVER	GO:0010054	trichoblast differentiation	BP	4.34E-02	1.68E-02	1	0	465	27197
OVER	GO:0010065	primary meristem tissue development	BP	4.34E-02	1.68E-02	1	0	465	27197
OVER	GO:0010068	protoderm histogenesis	BP	4.34E-02	1.68E-02	1	0	465	27197
OVER	GO:0010072	primary shoot apical meristem specification	BP	4.34E-02	1.68E-02	1	0	465	27197
OVER	GO:0010073	meristem maintenance	BP	1.35E-03	2.83E-04	2	0	464	27197
OVER	GO:0010074	maintenance of meristem identity	BP	4.34E-02	1.68E-02	1	0	465	27197
OVER	GO:0010075	regulation of meristem growth	BP	4.34E-02	1.68E-02	1	0	465	27197
OVER	GO:0010077	maintenance of inflorescence meristem identity	BP	4.34E-02	1.68E-02	1	0	465	27197
OVER	GO:0010087	phloem or xylem histogenesis	BP	1.35E-03	2.83E-04	2	0	464	27197
OVER	GO:0010088	phloem development	BP	1.35E-03	2.83E-04	2	0	464	27197
OVER	GO:0010090	trichome morphogenesis	BP	4.34E-02	1.68E-02	1	0	465	27197
OVER	GO:0010114	response to red light	BP	1.35E-03	2.83E-04	2	0	464	27197
OVER	GO:0010117	photoprotection	BP	4.34E-02	1.68E-02	1	0	465	27197
OVER	GO:0010118	stomatal movement	BP	4.34E-02	1.68E-02	1	0	465	27197
OVER	GO:0010119	regulation of stomatal movement	BP	4.34E-02	1.68E-02	1	0	465	27197
OVER	GO:0010150	leaf senescence	BP	1.35E-03	2.83E-04	2	0	464	27197
OVER	GO:0010154	fruit development	BP	3.53E-15	1.01E-16	9	0	457	27197
OVER	GO:0010155	regulation of proton transport	BP	4.34E-02	1.68E-02	1	0	465	27197
OVER	GO:0010162	seed dormancy process	BP	3.27E-05	4.75E-06	3	0	463	27197
OVER	GO:0010167	response to nitrate	BP	4.34E-02	1.68E-02	1	0	465	27197
OVER	GO:0010182	sugar mediated signaling pathway	BP	1.35E-03	2.83E-04	2	0	464	27197
OVER	GO:0010197	polar nucleus fusion	BP	4.34E-02	1.68E-02	1	0	465	27197
OVER	GO:0010207	photosystem II assembly	BP	4.34E-02	1.68E-02	1	0	465	27197
OVER	GO:0010214	seed coat development	BP	4.34E-02	1.68E-02	1	0	465	27197
OVER	GO:0010218	response to far red light	BP	1.35E-03	2.83E-04	2	0	464	27197

**Over/Under***	**GO ID**	**GO Name**	**GO Category$**	**FDR**	**P-value**	**Test**	**Ref**	**Non Annot Test**	**Non Annot Reference**
OVER	GO:0010227	floral organ abscission	BP	4.34E-02	1.68E-02	1	0	465	27197
OVER	GO:0010228	vegetative to reproductive phase transition of meristem	BP	3.53E-15	1.01E-16	9	0	457	27197
OVER	GO:0010267	production of ta-siRNAs involved in RNA interference	BP	4.34E-02	1.68E-02	1	0	465	27197
OVER	GO:0010351	lithium ion transport	BP	4.34E-02	1.68E-02	1	0	465	27197
OVER	GO:0010363	regulation of plant-type hypersensitive response	BP	3.27E-05	4.75E-06	3	0	463	27197
OVER	GO:0010383	cell wall polysaccharide metabolic process	BP	8.49E-12	3.68E-13	7	0	459	27197
OVER	GO:0010410	hemicellulose metabolic process	BP	8.49E-12	3.68E-13	7	0	459	27197
OVER	GO:0010413	glucuronoxylan metabolic process	BP	4.00E-10	2.21E-11	6	0	460	27197
OVER	GO:0010431	seed maturation	BP	7.35E-07	7.95E-08	4	0	462	27197
OVER	GO:0010467	gene expression	BP	5.25E-11	2.48E-12	79	1952	387	25245
OVER	GO:0010468	regulation of gene expression	BP	4.79E-03	1.05E-03	37	1223	429	25974
OVER	GO:0010496	intercellular transport	BP	4.34E-02	1.68E-02	1	0	465	27197
OVER	GO:0010497	plasmodesmata-mediated intercellular transport	BP	4.34E-02	1.68E-02	1	0	465	27197
OVER	GO:0010556	regulation of macromolecule biosynthetic process	BP	4.97E-03	1.10E-03	37	1228	429	25969
OVER	GO:0010557	positive regulation of macromolecule biosynthetic process	BP	3.03E-04	5.04E-05	4	9	462	27188
OVER	GO:0010558	negative regulation of macromolecule biosynthetic process	BP	1.70E-02	4.06E-03	2	4	464	27193
OVER	GO:0010564	regulation of cell cycle process	BP	9.07E-04	1.58E-04	3	4	463	27193
OVER	GO:0010604	positive regulation of macromolecule metabolic process	BP	3.03E-04	5.04E-05	4	9	462	27188
OVER	GO:0010605	negative regulation of macromolecule metabolic process	BP	2.70E-06	3.12E-07	5	5	461	27192
OVER	GO:0010608	posttranscriptional regulation of gene expression	BP	2.93E-02	7.41E-03	2	6	464	27191
OVER	GO:0010628	positive regulation of gene expression	BP	3.03E-04	5.04E-05	4	9	462	27188
OVER	GO:0010629	negative regulation of gene expression	BP	1.43E-06	1.58E-07	5	4	461	27193
OVER	GO:0010638	positive regulation of organelle organization	BP	4.34E-02	1.68E-02	1	0	465	27197
OVER	GO:0010647	positive regulation of cell communication	BP	4.34E-02	1.68E-02	1	0	465	27197
OVER	GO:0010876	lipid localization	BP	2.17E-04	3.53E-05	4	8	462	27189
OVER	GO:0010941	regulation of cell death	BP	1.69E-08	1.33E-09	5	0	461	27197
OVER	GO:0012505	endomembrane system	CT	2.84E-07	2.65E-08	16	151	450	27046
OVER	GO:0012506	vesicle membrane	CT	3.17E-04	5.34E-05	5	20	461	27177
OVER	GO:0012507	ER to Golgi transport vesicle membrane	CT	1.87E-05	2.34E-06	5	9	461	27188
OVER	GO:0014070	response to organic cyclic compound	BP	7.35E-07	7.95E-08	4	0	462	27197
OVER	GO:0015031	protein transport	BP	1.60E-11	7.07E-13	24	199	442	26998
OVER	GO:0015074	DNA integration	BP	3.27E-05	4.75E-06	3	0	463	27197
OVER	GO:0015075	ion transmembrane transporter activity	MF	3.11E-06	3.64E-07	22	346	444	26851
OVER	GO:0015077	monovalent inorganic cation transmembrane transporter activity	MF	1.28E-03	2.27E-04	10	139	456	27058

Over/Under*	GO ID	GO Name	GO Category$	FDR	P-value	Test	Ref	Non Annot Test	Non Annot Reference
OVER	GO:0015078	hydrogen ion transmembrane transporter activity	MF	8.45E-04	1.47E-04	9	106	457	27091
OVER	GO:0015085	calcium ion transmembrane transporter activity	MF	4.34E-02	1.68E-02	1	0	465	27197
OVER	GO:0015086	cadmium ion transmembrane transporter activity	MF	4.34E-02	1.68E-02	1	0	465	27197
OVER	GO:0015095**	magnesium ion transmembrane transporter activity*	MF	1.35E-03	2.83E-04	2	0	464	27197
OVER	GO:0015220**	choline transmembrane transporter activity*	MF	1.35E-03	2.83E-04	2	0	464	27197
OVER	GO:0015232	heme transporter activity	MF	4.34E-02	1.68E-02	1	0	465	27197
OVER	GO:0015267	channel activity	MF	1.65E-02	3.91E-03	5	57	461	27140
OVER	GO:0015399	primary active transmembrane transporter activity	MF	1.60E-09	9.64E-11	17	115	449	27082
OVER	GO:0015405	P-P-bond-hydrolysis-driven transmembrane transporter activity	MF	1.60E-09	9.64E-11	17	115	449	27082
OVER	GO:0015416**	ATPase-coupled organic phosphonate transmembrane transporter activity*	MF	4.34E-02	1.68E-02	1	0	465	27197
OVER	GO:0015434	cadmium-transporting ATPase activity	MF	4.34E-02	1.68E-02	1	0	465	27197
OVER	GO:0015439	heme-transporting ATPase activity	MF	4.34E-02	1.68E-02	1	0	465	27197
OVER	GO:0015604	organic phosphonate transmembrane transporter activity	MF	4.34E-02	1.68E-02	1	0	465	27197
OVER	GO:0015629	actin cytoskeleton	CT	5.32E-03	1.18E-03	4	24	462	27173
OVER	GO:0015631	tubulin binding	MF	7.31E-03	1.66E-03	2	2	464	27195
OVER	GO:0015633	zinc-transporting ATPase activity	MF	4.34E-02	1.68E-02	1	0	465	27197
OVER	GO:0015645	fatty acid ligase activity	MF	4.34E-02	1.68E-02	1	0	465	27197
OVER	GO:0015672	monovalent inorganic cation transport	BP	2.57E-06	2.93E-07	12	96	454	27101
OVER	GO:0015691	cadmium ion transport	BP	4.34E-02	1.68E-02	1	0	465	27197
OVER	GO:0015693	magnesium ion transport	BP	3.27E-05	4.75E-06	3	0	463	27197
OVER	GO:0015706	nitrate transport	BP	4.34E-02	1.68E-02	1	0	465	27197
OVER	GO:0015716	organic phosphonate transport	BP	4.34E-02	1.68E-02	1	0	465	27197
OVER	GO:0015748	organophosphate ester transport	BP	1.35E-03	2.83E-04	2	0	464	27197
OVER	GO:0015850	organic hydroxy compound transport	BP	4.34E-02	1.68E-02	1	0	465	27197
OVER	GO:0015871	choline transport	BP	1.35E-03	2.83E-04	2	0	464	27197
OVER	GO:0015886	heme transport	BP	4.34E-02	1.68E-02	1	0	465	27197
OVER	GO:0015914	phospholipid transport	BP	4.34E-02	1.68E-02	1	0	465	27197
OVER	GO:0015918	sterol transport	BP	4.34E-02	1.68E-02	1	0	465	27197
OVER	GO:0015926	glucosidase activity	MF	3.27E-03	7.09E-04	3	8	463	27189
OVER	GO:0015935**	small ribosomal subunit*	CT	1.75E-03	3.70E-04	3	6	463	27191
OVER	GO:0015979	photosynthesis	BP	1.59E-20	3.10E-22	27	99	439	27098
OVER	GO:0015980	energy derivation by oxidation of organic compounds	BP	1.09E-05	1.34E-06	7	27	459	27170
OVER	GO:0015985	energy coupled proton transport, down electrochemical gradient	BP	1.14E-07	1.02E-08	8	19	458	27178
OVER	GO:0015986	ATP synthesis coupled proton transport	BP	1.14E-07	1.02E-08	8	19	458	27178
OVER	GO:0015988	energy coupled proton transmembrane transport, against electrochemical gradient	BP	8.57E-03	1.97E-03	4	28	462	27169

Over/Under*	GO ID	GO Name	GO Category$	FDR	P-value	Test	Ref	Non Annot Test	Non Annot Reference
OVER	GO:0015991	ATP hydrolysis coupled proton transport	BP	8.57E-03	1.97E-03	4	28	462	27169
OVER	GO:0015992	proton transport	BP	7.55E-07	8.21E-08	10	52	456	27145
OVER	GO:0016020	membrane	CT	5.07E-20	1.06E-21	104	2140	362	25057
OVER	GO:0016021	integral component of membrane	CT	5.83E-24	8.71E-26	67	793	399	26404
OVER	GO:0016032	viral process	BP	4.34E-02	1.68E-02	1	0	465	27197
OVER	GO:0016042	lipid catabolic process	BP	9.10E-04	1.59E-04	4	13	462	27184
OVER	GO:0016043	cellular component organization	BP	3.69E-15	1.07E-16	35	328	431	26869
OVER	GO:0016049	cell growth	BP	1.35E-03	2.83E-04	2	0	464	27197
OVER	GO:0016051	carbohydrate biosynthetic process	BP	3.55E-03	7.74E-04	10	164	456	27033
OVER	GO:0016053	organic acid biosynthetic process	BP	1.56E-04	2.48E-05	13	179	453	27018
OVER	GO:0016054	organic acid catabolic process	BP	3.01E-03	6.46E-04	4	20	462	27177
OVER	GO:0016070	RNA metabolic process	BP	3.70E-12	1.45E-13	71	1551	395	25646
OVER	GO:0016071	mRNA metabolic process	BP	2.63E-09	1.67E-10	11	34	455	27163
OVER	GO:0016106	sesquiterpenoid biosynthetic process	BP	4.34E-02	1.68E-02	1	0	465	27197
OVER	GO:0016108	tetraterpenoid metabolic process	BP	4.34E-02	1.17E-02	2	8	464	27189
OVER	GO:0016116	carotenoid metabolic process	BP	4.34E-02	1.17E-02	2	8	464	27189
OVER	GO:0016122	xanthophyll metabolic process	BP	4.34E-02	1.68E-02	1	0	465	27197
OVER	GO:0016143	S-glycoside metabolic process	BP	4.34E-02	1.68E-02	1	0	465	27197
OVER	GO:0016144	S-glycoside biosynthetic process	BP	4.34E-02	1.68E-02	1	0	465	27197
OVER	GO:0016168	chlorophyll binding	MF	1.58E-08	1.14E-09	7	7	459	27190
OVER	GO:0016180	snRNA processing	BP	4.34E-02	1.68E-02	1	0	465	27197
OVER	GO:0016192	vesicle-mediated transport	BP	7.01E-04	1.21E-04	10	128	456	27069
OVER	GO:0016246	RNA interference	BP	4.34E-02	1.68E-02	1	0	465	27197
OVER	GO:0016310	phosphorylation	BP	9.52E-04	1.67E-04	43	1362	423	25835
OVER	GO:0016311	dephosphorylation	BP	5.32E-03	1.18E-03	4	24	462	27173
OVER	GO:0016312	inositol bisphosphate phosphatase activity	MF	4.34E-02	1.68E-02	1	0	465	27197
OVER	GO:0016405	CoA-ligase activity	MF	4.34E-02	1.68E-02	1	0	465	27197
OVER	GO:0016412	serine O-acyltransferase activity	MF	2.93E-02	7.41E-03	2	6	464	27191
OVER	GO:0016413	O-acetyltransferase activity	MF	2.93E-02	7.41E-03	2	6	464	27191
OVER	GO:0016441	posttranscriptional gene silencing	BP	4.34E-02	1.68E-02	1	0	465	27197
OVER	GO:0016444	somatic cell DNA recombination	BP	4.34E-02	1.68E-02	1	0	465	27197
OVER	GO:0016458	gene silencing	BP	2.11E-05	2.67E-06	4	3	462	27194
OVER	GO:0016459**	myosin complex*	CT	1.60E-02	3.77E-03	3	16	463	27181
OVER	GO:0016462	pyrophosphatase activity	MF	4.18E-09	2.73E-10	38	659	428	26538
OVER	GO:0016469	proton-transporting two-sector ATPase complex	CT	3.82E-05	5.60E-06	8	50	458	27147
OVER	GO:0016491	oxidoreductase activity	MF	5.47E-06	6.53E-07	57	1628	409	25569
OVER	GO:0016558	protein import into peroxisome matrix	BP	7.35E-07	7.95E-08	4	0	462	27197
OVER	GO:0016560	protein import into peroxisome matrix, docking	BP	4.34E-02	1.68E-02	1	0	465	27197
OVER	GO:0016569	covalent chromatin modification	BP	4.52E-06	5.36E-07	7	23	459	27174
OVER	GO:0016570	histone modification	BP	4.52E-06	5.36E-07	7	23	459	27174
OVER	GO:0016571	histone methylation	BP	1.17E-02	2.71E-03	3	14	463	27183

Over/Under*	GO ID	GO Name	GO Category$	FDR	P-value	Test	Ref	Non Annot Test	Non Annot Reference
OVER	GO:0016573	histone acetylation	BP	3.27E-05	4.75E-06	3	0	463	27197
OVER	GO:0016620	oxidoreductase activity, acting on the aldehyde or oxo group of donors, NAD or NADP as acceptor	MF	1.50E-04	2.37E-05	6	29	460	27168
OVER	GO:0016627	oxidoreductase activity, acting on the CH-CH group of donors	MF	6.60E-07	6.41E-08	9	37	457	27160
OVER	GO:0016628	oxidoreductase activity, acting on the CH-CH group of donors, NAD or NADP as acceptor	MF	4.18E-09	2.71E-10	7	5	459	27192
OVER	GO:0016634	oxidoreductase activity, acting on the CH-CH group of donors, oxygen as acceptor	MF	3.68E-02	9.43E-03	2	7	464	27190
OVER	GO:0016651	oxidoreductase activity, acting on NAD(P)H	MF	1.74E-06	1.96E-07	9	43	457	27154
OVER	GO:0016655	oxidoreductase activity, acting on NAD(P)H, quinone or similar compound as acceptor	MF	5.46E-09	3.62E-10	9	18	457	27179
OVER	GO:0016703	oxidoreductase activity, acting on single donors with incorporation of molecular oxygen, incorporation of one atom of oxygen (internal monooxygenases or internal mixed function oxidases)	MF	4.34E-02	1.41E-02	2	9	464	27188
OVER	GO:0016726	oxidoreductase activity, acting on CH or CH2 groups, NAD or NADP as acceptor	MF	4.34E-02	1.68E-02	1	0	465	27197
OVER	GO:0016740	transferase activity	MF	8.70E-10	5.02E-11	97	2822	369	24375
OVER	GO:0016765	transferase activity, transferring alkyl or aryl (other than methyl) groups	MF	1.45E-02	3.39E-03	5	55	461	27142
OVER	GO:0016772	transferase activity, transferring phosphorus-containing groups	MF	1.53E-06	1.71E-07	58	1598	408	25599
OVER	GO:0016779	nucleotidyltransferase activity	MF	6.57E-16	1.81E-17	25	131	441	27066
OVER	GO:0016787	hydrolase activity	MF	2.06E-09	1.25E-10	84	2320	382	24877
OVER	GO:0016788	hydrolase activity, acting on ester bonds	MF	1.43E-02	3.35E-03	19	525	447	26672
OVER	GO:0016791	phosphatase activity	MF	4.34E-02	1.47E-02	4	52	462	27145
OVER	GO:0016803	ether hydrolase activity	MF	4.34E-02	1.68E-02	1	0	465	27197
OVER	GO:0016817	hydrolase activity, acting on acid anhydrides	MF	2.63E-09	1.68E-10	39	678	427	26519
OVER	GO:0016818	hydrolase activity, acting on acid anhydrides, in phosphorus-containing anhydrides	MF	2.07E-09	1.26E-10	39	671	427	26526
OVER	GO:0016820	hydrolase activity, acting on acid anhydrides, catalyzing transmembrane movement of substances	MF	1.15E-09	6.66E-11	17	112	449	27085
OVER	GO:0016874	ligase activity	MF	5.56E-06	6.66E-07	16	195	450	27002
OVER	GO:0016877	ligase activity, forming carbon-sulfur bonds	MF	1.70E-02	4.06E-03	2	4	464	27193
OVER	GO:0016878	acid-thiol ligase activity	MF	4.34E-02	1.68E-02	1	0	465	27197
OVER	GO:0016887	ATPase activity	MF	1.38E-09	8.21E-11	29	377	437	26820
OVER	GO:0016899	oxidoreductase activity, acting on the CH-OH group of donors, oxygen as acceptor	MF	2.28E-02	5.62E-03	2	5	464	27192
OVER	GO:0016903	oxidoreductase activity, acting on the aldehyde or oxo group of donors	MF	1.12E-04	1.72E-05	7	42	459	27155
OVER	GO:0016926	protein desumoylation	BP	4.00E-10	2.21E-11	6	0	460	27197

Over/Under*	GO ID	GO Name	GO Category$	FDR	P-value	Test	Ref	Non Annot Test	Non Annot Reference
OVER	GO:0017016	Ras GTPase binding	MF	1.35E-03	2.83E-04	2	0	464	27197
OVER	GO:0017025**	TBP-class protein binding*	MF	1.35E-03	2.83E-04	2	0	464	27197
OVER	GO:0017038	protein import	BP	1.23E-06	1.36E-07	7	18	459	27179
OVER	GO:0017069	snRNA binding	MF	3.27E-05	4.75E-06	3	0	463	27197
OVER	GO:0017070**	U6 snRNA binding*	MF	1.35E-03	2.83E-04	2	0	464	27197
OVER	GO:0017076	purine nucleotide binding	MF	5.94E-09	3.98E-10	91	2673	375	24524
OVER	GO:0017111	nucleoside-triphosphatase activity	MF	2.93E-09	1.88E-10	38	650	428	26547
OVER	GO:0018022	peptidyl-lysine methylation	BP	1.17E-02	2.71E-03	3	14	463	27183
OVER	GO:0018108	peptidyl-tyrosine phosphorylation	BP	3.27E-05	4.75E-06	3	0	463	27197
OVER	GO:0018130	heterocycle biosynthetic process	BP	2.13E-10	1.06E-11	66	1515	400	25682
OVER	GO:0018193	peptidyl-amino acid modification	BP	3.93E-18	9.17E-20	17	25	449	27172
OVER	GO:0018205	peptidyl-lysine modification	BP	8.04E-13	2.98E-14	12	18	454	27179
OVER	GO:0018208	peptidyl-proline modification	BP	1.35E-03	2.83E-04	2	0	464	27197
OVER	GO:0018212	peptidyl-tyrosine modification	BP	3.27E-05	4.75E-06	3	0	463	27197
OVER	GO:0018298	protein-chromophore linkage	BP	1.90E-09	1.15E-10	7	4	459	27193
OVER	GO:0018393	internal peptidyl-lysine acetylation	BP	3.27E-05	4.75E-06	3	0	463	27197
OVER	GO:0018394	peptidyl-lysine acetylation	BP	3.27E-05	4.75E-06	3	0	463	27197
OVER	GO:0018958	phenol-containing compound metabolic process	BP	4.34E-02	1.68E-02	1	0	465	27197
OVER	GO:0019012	virion	CT	4.34E-02	1.68E-02	1	0	465	27197
OVER	GO:0019013**	viral nucleocapsid*	CT	4.34E-02	1.68E-02	1	0	465	27197
OVER	GO:0019028	viral capsid	CT	4.34E-02	1.68E-02	1	0	465	27197
OVER	GO:0019199	transmembrane receptor protein kinase activity	MF	4.34E-02	1.68E-02	1	0	465	27197
OVER	GO:0019219	regulation of nucleobase-containing compound metabolic process	BP	9.70E-03	2.24E-03	36	1223	430	25974
OVER	GO:0019222	regulation of metabolic process	BP	1.16E-03	2.06E-04	40	1244	426	25953
OVER	GO:0019253	reductive pentose-phosphate cycle	BP	4.34E-02	1.68E-02	1	0	465	27197
OVER	GO:0019318	hexose metabolic process	BP	2.24E-03	4.77E-04	5	34	461	27163
OVER	GO:0019319	hexose biosynthetic process	BP	3.68E-02	9.43E-03	2	7	464	27190
OVER	GO:0019344	cysteine biosynthetic process	BP	8.41E-09	5.79E-10	7	6	459	27191
OVER	GO:0019395	fatty acid oxidation	BP	1.35E-03	2.50E-04	3	5	463	27192
OVER	GO:0019438	aromatic compound biosynthetic process	BP	7.35E-11	3.54E-12	67	1501	399	25696
OVER	GO:0019538	protein metabolic process	BP	2.25E-08	1.84E-09	86	2530	380	24667
OVER	GO:0019637	organophosphate metabolic process	BP	4.53E-07	4.32E-08	21	277	445	26920
OVER	GO:0019684	photosynthesis, light reaction	BP	5.35E-12	2.14E-13	14	40	452	27157
OVER	GO:0019685	photosynthesis, dark reaction	BP	4.34E-02	1.68E-02	1	0	465	27197
OVER	GO:0019693	ribose phosphate metabolic process	BP	1.23E-07	1.11E-08	17	162	449	27035
OVER	GO:0019750	chloroplast localization	BP	4.34E-02	1.68E-02	1	0	465	27197
OVER	GO:0019752	carboxylic acid metabolic process	BP	1.95E-07	1.80E-08	26	397	440	26800
OVER	GO:0019757	glycosinolate metabolic process	BP	4.34E-02	1.68E-02	1	0	465	27197
OVER	GO:0019758	glycosinolate biosynthetic process	BP	4.34E-02	1.68E-02	1	0	465	27197
OVER	GO:0019760	glucosinolate metabolic process	BP	4.34E-02	1.68E-02	1	0	465	27197

Over/Under*	GO ID	GO Name	GO Category$	FDR	P-value	Test	Ref	Non Annot Test	Non Annot Reference
OVER	GO:0019761	glucosinolate biosynthetic process	BP	4.34E-02	1.68E-02	1	0	465	27197
OVER	GO:0019783	ubiquitin-like protein-specific protease activity	MF	4.34E-02	1.68E-02	1	0	465	27197
OVER	GO:0019827	stem cell population maintenance	BP	4.34E-02	1.68E-02	1	0	465	27197
OVER	GO:0019829	cation-transporting ATPase activity	MF	1.08E-08	7.51E-10	9	20	457	27177
OVER	GO:0019843**	rRNA binding*	MF	4.61E-04	7.89E-05	5	22	461	27175
OVER	GO:0019866	organelle inner membrane	CT	4.76E-05	7.02E-06	7	36	459	27161
OVER	GO:0019899	enzyme binding	MF	3.27E-02	8.29E-03	3	22	463	27175
OVER	GO:0019915	lipid storage	BP	4.34E-02	1.68E-02	1	0	465	27197
OVER	GO:0019953	sexual reproduction	BP	1.35E-03	2.83E-04	2	0	464	27197
OVER	GO:0021700	developmental maturation	BP	1.69E-08	1.33E-09	5	0	461	27197
OVER	GO:0022402	cell cycle process	BP	3.17E-04	5.34E-05	5	20	461	27177
OVER	GO:0022412	cellular process involved in reproduction in multicellular organism	BP	1.35E-03	2.83E-04	2	0	464	27197
OVER	GO:0022414	reproductive process	BP	3.67E-20	7.47E-22	24	70	442	27127
OVER	GO:0022607	cellular component assembly	BP	3.45E-03	7.50E-04	8	107	458	27090
OVER	GO:0022611	dormancy process	BP	3.27E-05	4.75E-06	3	0	463	27197
OVER	GO:0022615	protein to membrane docking	BP	4.34E-02	1.68E-02	1	0	465	27197
OVER	GO:0022622	root system development	BP	1.69E-08	1.33E-09	5	0	461	27197
OVER	GO:0022803	passive transmembrane transporter activity	MF	1.65E-02	3.91E-03	5	57	461	27140
OVER	GO:0022804	active transmembrane transporter activity	MF	1.63E-06	1.83E-07	19	250	447	26947
OVER	GO:0022838	substrate-specific channel activity	MF	1.65E-02	3.91E-03	5	57	461	27140
OVER	GO:0022853	active ion transmembrane transporter activity	MF	1.19E-06	1.31E-07	10	55	456	27142
OVER	GO:0022857	transmembrane transporter activity	MF	3.92E-07	3.72E-08	32	597	434	26600
OVER	GO:0022890	inorganic cation transmembrane transporter activity	MF	8.14E-05	1.23E-05	14	193	452	27004
OVER	GO:0022891	substrate-specific transmembrane transporter activity	MF	1.85E-06	2.09E-07	23	363	443	26834
OVER	GO:0022892	substrate-specific transporter activity	MF	4.72E-07	4.55E-08	26	417	440	26780
OVER	GO:0022900	electron transport chain	BP	1.25E-13	4.20E-15	14	28	452	27169
OVER	GO:0022904	respiratory electron transport chain	BP	9.08E-07	9.94E-08	7	17	459	27180
OVER	GO:0023014	signal transduction by protein phosphorylation	BP	4.34E-02	1.68E-02	1	0	465	27197
OVER	GO:0023052	signaling	BP	1.44E-06	1.60E-07	25	416	441	26781
OVER	GO:0023056	positive regulation of signaling	BP	4.34E-02	1.68E-02	1	0	465	27197
OVER	GO:0030001	metal ion transport	BP	4.88E-02	1.90E-02	8	189	458	27008
OVER	GO:0030003	cellular cation homeostasis	BP	2.28E-02	5.62E-03	2	5	464	27192
OVER	GO:0030004	cellular monovalent inorganic cation homeostasis	BP	4.34E-02	1.68E-02	1	0	465	27197
OVER	GO:0030007	cellular potassium ion homeostasis	BP	4.34E-02	1.68E-02	1	0	465	27197
OVER	GO:0030054	cell junction	CT	3.81E-15	1.12E-16	11	5	455	27192
OVER	GO:0030117	membrane coat	CT	6.14E-03	1.38E-03	5	44	461	27153
OVER	GO:0030120	vesicle coat	CT	3.17E-04	5.34E-05	5	20	461	27177

Over/Under*	GO ID	GO Name	GO Category$	FDR	P-value	Test	Ref	Non Annot Test	Non Annot Reference
OVER	GO:0030127**	COPII vesicle coat*	CT	1.87E-05	2.34E-06	5	9	461	27188
OVER	GO:0030133	transport vesicle	CT	1.38E-04	2.16E-05	5	16	461	27181
OVER	GO:0030134	ER to Golgi transport vesicle	CT	1.87E-05	2.34E-06	5	9	461	27188
OVER	GO:0030135	coated vesicle	CT	3.17E-04	5.34E-05	5	20	461	27177
OVER	GO:0030148	sphingolipid biosynthetic process	BP	4.34E-02	1.68E-02	1	0	465	27197
OVER	GO:0030154	cell differentiation	BP	6.50E-17	1.67E-18	10	0	456	27197
OVER	GO:0030247	polysaccharide binding	MF	1.75E-03	3.70E-04	3	6	463	27191
OVER	GO:0030258	lipid modification	BP	1.31E-02	3.06E-03	4	32	462	27165
OVER	GO:0030261	chromosome condensation	BP	4.34E-02	1.68E-02	1	0	465	27197
OVER	GO:0030301	cholesterol transport	BP	4.34E-02	1.68E-02	1	0	465	27197
OVER	GO:0030422	production of siRNA involved in RNA interference	BP	4.34E-02	1.68E-02	1	0	465	27197
OVER	GO:0030529	intracellular ribonucleoprotein complex	CT	2.93E-02	7.28E-03	14	369	452	26828
OVER	GO:0030532	small nuclear ribonucleoprotein complex	CT	4.34E-02	1.68E-02	1	0	465	27197
OVER	GO:0030554	adenyl nucleotide binding	MF	4.04E-10	2.25E-11	89	2447	377	24750
OVER	GO:0030623**	U5 snRNA binding*	MF	1.35E-03	2.83E-04	2	0	464	27197
OVER	GO:0030658	transport vesicle membrane	CT	1.38E-04	2.16E-05	5	16	461	27181
OVER	GO:0030659	cytoplasmic vesicle membrane	CT	3.17E-04	5.34E-05	5	20	461	27177
OVER	GO:0030662	coated vesicle membrane	CT	3.17E-04	5.34E-05	5	20	461	27177
OVER	GO:0030838	positive regulation of actin filament polymerization	BP	4.34E-02	1.68E-02	1	0	465	27197
OVER	GO:0030894	replisome	CT	1.35E-03	2.83E-04	2	0	464	27197
OVER	GO:0031047	gene silencing by RNA	BP	7.35E-07	7.95E-08	4	0	462	27197
OVER	GO:0031048	chromatin silencing by small RNA	BP	4.34E-02	1.68E-02	1	0	465	27197
OVER	GO:0031050	dsRNA fragmentation	BP	4.34E-02	1.68E-02	1	0	465	27197
OVER	GO:0031090	organelle membrane	CT	4.10E-14	1.31E-15	21	100	445	27097
OVER	GO:0031109	microtubule polymerization or depolymerization	BP	3.83E-03	8.40E-04	2	1	464	27196
OVER	GO:0031123	RNA 3'-end processing	BP	4.34E-02	1.68E-02	1	0	465	27197
OVER	GO:0031224	intrinsic component of membrane	CT	2.46E-21	4.42E-23	68	921	398	26276
OVER	GO:0031225	anchored component of membrane	CT	4.34E-02	1.68E-02	1	0	465	27197
OVER	GO:0031267	small GTPase binding	MF	1.35E-03	2.83E-04	2	0	464	27197
OVER	GO:0031323	regulation of cellular metabolic process	BP	2.25E-03	4.80E-04	39	1241	427	25956
OVER	GO:0031324	negative regulation of cellular metabolic process	BP	1.70E-02	4.06E-03	2	4	464	27193
OVER	GO:0031325	positive regulation of cellular metabolic process	BP	3.03E-04	5.04E-05	4	9	462	27188
OVER	GO:0031326	regulation of cellular biosynthetic process	BP	4.97E-03	1.10E-03	37	1228	429	25969
OVER	GO:0031327	negative regulation of cellular biosynthetic process	BP	1.70E-02	4.06E-03	2	4	464	27193
OVER	GO:0031328	positive regulation of cellular biosynthetic process	BP	3.03E-04	5.04E-05	4	9	462	27188
OVER	GO:0031334	positive regulation of protein complex assembly	BP	4.34E-02	1.68E-02	1	0	465	27197
OVER	GO:0031347	regulation of defense response	BP	1.69E-08	1.33E-09	5	0	461	27197
OVER	GO:0031348	negative regulation of defense response	BP	4.34E-02	1.68E-02	1	0	465	27197

Over/Under*	GO ID	GO Name	GO Category$	FDR	P-value	Test	Ref	Non Annot Test	Non Annot Reference
OVER	GO:0031349	positive regulation of defense response	BP	4.34E-02	1.68E-02	1	0	465	27197
OVER	GO:0031360	intrinsic component of thylakoid membrane	CT	4.34E-02	1.68E-02	1	0	465	27197
OVER	GO:0031361**	integral component of thylakoid membrane*	CT	4.34E-02	1.68E-02	1	0	465	27197
OVER	GO:0031410	cytoplasmic vesicle	CT	9.07E-05	1.38E-05	6	26	460	27171
OVER	GO:0031461	cullin-RING ubiquitin ligase complex	CT	1.23E-05	1.53E-06	5	8	461	27189
OVER	GO:0031463**	Cul3-RING ubiquitin ligase complex*	CT	4.34E-02	1.68E-02	1	0	465	27197
OVER	GO:0031503	protein complex localization	BP	4.34E-02	1.68E-02	1	0	465	27197
OVER	GO:0031519**	PcG protein complex*	CT	4.34E-02	1.68E-02	1	0	465	27197
OVER	GO:0031570	DNA integrity checkpoint	BP	4.34E-02	1.68E-02	1	0	465	27197
OVER	GO:0031647	regulation of protein stability	BP	1.35E-03	2.83E-04	2	0	464	27197
OVER	GO:0031966**	mitochondrial membrane*	CT	4.34E-02	1.14E-02	4	48	462	27149
OVER	GO:0031967	organelle envelope	CT	7.97E-13	2.91E-14	18	77	448	27120
OVER	GO:0031969	chloroplast membrane	CT	1.69E-08	1.33E-09	5	0	461	27197
OVER	GO:0031974	membrane-enclosed lumen	CT	1.74E-02	4.19E-03	5	58	461	27139
OVER	GO:0031975	envelope	CT	2.34E-11	1.06E-12	18	98	448	27099
OVER	GO:0031976	plastid thylakoid	CT	6.77E-54	3.85E-56	31	0	435	27197
OVER	GO:0031981	nuclear lumen	CT	5.62E-03	1.25E-03	5	43	461	27154
OVER	GO:0031982	vesicle	CT	9.07E-05	1.38E-05	6	26	460	27171
OVER	GO:0031984	organelle subcompartment	CT	1.33E-53	7.95E-56	34	5	432	27192
OVER	GO:0032259	methylation	BP	1.94E-07	1.78E-08	11	57	455	27140
OVER	GO:0032266	phosphatidylinositol-3-phosphate binding	MF	4.34E-02	1.68E-02	1	0	465	27197
OVER	GO:0032268	regulation of cellular protein metabolic process	BP	3.63E-02	9.26E-03	3	23	463	27174
OVER	GO:0032273	positive regulation of protein polymerization	BP	4.34E-02	1.68E-02	1	0	465	27197
OVER	GO:0032300**	mismatch repair complex*	CT	4.34E-02	1.68E-02	1	0	465	27197
OVER	GO:0032392	DNA geometric change	BP	4.34E-02	1.68E-02	1	0	465	27197
OVER	GO:0032440**	2-alkenal reductase [NAD(P)] activity*	MF	1.69E-08	1.33E-09	5	0	461	27197
OVER	GO:0032501	multicellular organismal process	BP	1.67E-28	2.25E-30	33	90	433	27107
OVER	GO:0032502	developmental process	BP	6.00E-38	6.28E-40	34	43	432	27154
OVER	GO:0032504	multicellular organism reproduction	BP	1.69E-08	1.33E-09	5	0	461	27197
OVER	GO:0032508	DNA duplex unwinding	BP	4.34E-02	1.68E-02	1	0	465	27197
OVER	GO:0032549	ribonucleoside binding	MF	1.28E-09	7.54E-11	93	2669	373	24528
OVER	GO:0032550	purine ribonucleoside binding	MF	5.32E-09	3.52E-10	91	2662	375	24535
OVER	GO:0032553	ribonucleotide binding	MF	2.42E-09	1.48E-10	93	2692	373	24505
OVER	GO:0032555	purine ribonucleotide binding	MF	5.32E-09	3.52E-10	91	2662	375	24535
OVER	GO:0032559	adenyl ribonucleotide binding	MF	4.00E-10	2.11E-11	89	2443	377	24754
OVER	GO:0032774	RNA biosynthetic process	BP	3.41E-06	4.02E-07	50	1323	416	25874
OVER	GO:0032787	monocarboxylic acid metabolic process	BP	2.96E-09	1.91E-10	18	139	448	27058
OVER	GO:0032870	cellular response to hormone stimulus	BP	1.69E-08	1.33E-09	5	0	461	27197
OVER	GO:0032928	regulation of superoxide anion generation	BP	4.34E-02	1.68E-02	1	0	465	27197
OVER	GO:0032991	macromolecular complex	CT	4.24E-19	9.14E-21	65	936	401	26261

Over/Under*	GO ID	GO Name	GO Category$	FDR	P-value	Test	Ref	Non Annot Test	Non Annot Reference
OVER	GO:0033036	macromolecule localization	BP	2.18E-15	6.12E-17	30	223	436	26974
OVER	GO:0033037	polysaccharide localization	BP	1.35E-03	2.83E-04	2	0	464	27197
OVER	GO:0033177	proton-transporting two-sector ATPase complex, proton-transporting domain	CT	4.00E-02	1.03E-02	3	24	463	27173
OVER	GO:0033178	proton-transporting two-sector ATPase complex, catalytic domain	CT	4.61E-04	7.89E-05	5	22	461	27175
OVER	GO:0033365	protein localization to organelle	BP	4.47E-07	4.25E-08	9	35	457	27162
OVER	GO:0033554	cellular response to stress	BP	9.29E-04	1.63E-04	11	160	455	27037
OVER	GO:0033609	oxalate metabolic process	BP	4.34E-02	1.68E-02	1	0	465	27197
OVER	GO:0033611	oxalate catabolic process	BP	4.34E-02	1.68E-02	1	0	465	27197
OVER	GO:0033692	cellular polysaccharide biosynthetic process	BP	2.88E-02	7.16E-03	7	125	459	27072
OVER	GO:0033961	cis-stilbene-oxide hydrolase activity	MF	4.34E-02	1.68E-02	1	0	465	27197
OVER	GO:0033993	response to lipid	BP	3.27E-05	4.75E-06	3	0	463	27197
OVER	GO:0034050	host programmed cell death induced by symbiont	BP	3.27E-05	4.75E-06	3	0	463	27197
OVER	GO:0034061	DNA polymerase activity	MF	6.06E-03	1.35E-03	4	25	462	27172
OVER	GO:0034062	5'-3' RNA polymerase activity	MF	3.45E-14	1.08E-15	17	51	449	27146
OVER	GO:0034204	lipid translocation	BP	4.34E-02	1.68E-02	1	0	465	27197
OVER	GO:0034220	ion transmembrane transport	BP	3.55E-08	2.98E-09	15	109	451	27088
OVER	GO:0034248	regulation of cellular amide metabolic process	BP	2.93E-02	7.41E-03	2	6	464	27191
OVER	GO:0034285	response to disaccharide	BP	4.34E-02	1.68E-02	1	0	465	27197
OVER	GO:0034314	Arp2/3 complex-mediated actin nucleation	BP	4.34E-02	1.68E-02	1	0	465	27197
OVER	GO:0034330	cell junction organization	BP	4.34E-02	1.68E-02	1	0	465	27197
OVER	GO:0034357	photosynthetic membrane	CT	1.00E-36	1.14E-38	35	55	431	27142
OVER	GO:0034440	lipid oxidation	BP	1.35E-03	2.50E-04	3	5	463	27192
OVER	GO:0034470	ncRNA processing	BP	2.07E-03	4.37E-04	7	74	459	27123
OVER	GO:0034472	snRNA 3'-end processing	BP	4.34E-02	1.68E-02	1	0	465	27197
OVER	GO:0034477	U6 snRNA 3'-end processing	BP	4.34E-02	1.68E-02	1	0	465	27197
OVER	GO:0034504	protein localization to nucleus	BP	2.93E-02	7.41E-03	2	6	464	27191
OVER	GO:0034613	cellular protein localization	BP	5.08E-11	2.39E-12	21	154	445	27043
OVER	GO:0034622	cellular macromolecular complex assembly	BP	5.43E-03	1.21E-03	7	89	459	27108
OVER	GO:0034629	cellular protein complex localization	BP	4.34E-02	1.68E-02	1	0	465	27197
OVER	GO:0034637	cellular carbohydrate biosynthetic process	BP	2.26E-02	5.48E-03	8	150	458	27047
OVER	GO:0034641	cellular nitrogen compound metabolic process	BP	3.82E-21	7.09E-23	118	2566	348	24631
OVER	GO:0034645	cellular macromolecule biosynthetic process	BP	6.05E-09	4.07E-10	76	2054	390	25143
OVER	GO:0034654	nucleobase-containing compound biosynthetic process	BP	7.29E-10	4.17E-11	62	1415	404	25782
OVER	GO:0034660	ncRNA metabolic process	BP	8.00E-03	1.82E-03	8	124	458	27073
OVER	GO:0034968	histone lysine methylation	BP	1.17E-02	2.71E-03	3	14	463	27183
OVER	GO:0035101**	FACT complex*	CT	4.34E-02	1.68E-02	1	0	465	27197
OVER	GO:0035194	posttranscriptional gene silencing by RNA	BP	4.34E-02	1.68E-02	1	0	465	27197
OVER	GO:0035195	gene silencing by miRNA	BP	4.34E-02	1.68E-02	1	0	465	27197

Over/Under*	GO ID	GO Name	GO Category$	FDR	P-value	Test	Ref	Non Annot Test	Non Annot Reference
OVER	GO:0035196	production of miRNAs involved in gene silencing by miRNA	BP	4.34E-02	1.68E-02	1	0	465	27197
OVER	GO:0035235	ionotropic glutamate receptor signaling pathway	BP	1.35E-03	2.83E-04	2	0	464	27197
OVER	GO:0035250	UDP-galactosyltransferase activity	MF	1.35E-03	2.83E-04	2	0	464	27197
OVER	GO:0035266	meristem growth	BP	4.34E-02	1.68E-02	1	0	465	27197
OVER	GO:0035639	purine ribonucleoside triphosphate binding	MF	1.81E-04	2.90E-05	75	2662	391	24535
OVER	GO:0035670	plant-type ovary development	BP	4.34E-02	1.68E-02	1	0	465	27197
OVER	GO:0035821	modification of morphology or physiology of other organism	BP	4.34E-02	1.68E-02	1	0	465	27197
OVER	GO:0036094	small molecule binding	MF	1.97E-13	6.94E-15	112	3019	354	24178
OVER	GO:0036211	protein modification process	BP	4.15E-08	3.50E-09	60	1513	406	25684
OVER	GO:0036422	heptaprenyl diphosphate synthase activity	MF	4.34E-02	1.68E-02	1	0	465	27197
OVER	GO:0036442	hydrogen-exporting ATPase activity	MF	1.72E-04	2.76E-05	5	17	461	27180
OVER	GO:0036459	thiol-dependent ubiquitinyl hydrolase activity	MF	4.34E-02	1.68E-02	1	0	465	27197
OVER	GO:0040007	growth	BP	3.27E-05	4.75E-06	3	0	463	27197
OVER	GO:0040008	regulation of growth	BP	4.34E-02	1.68E-02	1	0	465	27197
OVER	GO:0040029	regulation of gene expression, epigenetic	BP	7.31E-03	1.66E-03	2	2	464	27195
OVER	GO:0042138	meiotic DNA double-strand break formation	BP	4.34E-02	1.68E-02	1	0	465	27197
OVER	GO:0042168	heme metabolic process	BP	3.27E-03	7.09E-04	3	8	463	27189
OVER	GO:0042170	plastid membrane	CT	4.00E-10	2.21E-11	6	0	460	27197
OVER	GO:0042221	response to chemical	BP	2.17E-14	6.68E-16	22	110	444	27087
OVER	GO:0042278	purine nucleoside metabolic process	BP	5.42E-08	4.65E-09	16	132	450	27065
OVER	GO:0042301	phosphate ion binding	MF	1.35E-03	2.83E-04	2	0	464	27197
OVER	GO:0042335	cuticle development	BP	1.35E-03	2.83E-04	2	0	464	27197
OVER	GO:0042393	histone binding	MF	6.70E-03	1.51E-03	3	11	463	27186
OVER	GO:0042435	indole-containing compound biosynthetic process	BP	4.34E-02	1.68E-02	1	0	465	27197
OVER	GO:0042440	pigment metabolic process	BP	1.59E-02	3.73E-03	4	34	462	27163
OVER	GO:0042451	purine nucleoside biosynthetic process	BP	1.21E-04	1.86E-05	8	60	458	27137
OVER	GO:0042455	ribonucleoside biosynthetic process	BP	1.95E-04	3.15E-05	8	65	458	27132
OVER	GO:0042537	benzene-containing compound metabolic process	BP	3.27E-05	4.75E-06	3	0	463	27197
OVER	GO:0042546	cell wall biogenesis	BP	3.64E-07	3.44E-08	6	7	460	27190
OVER	GO:0042554	superoxide anion generation	BP	4.34E-02	1.68E-02	1	0	465	27197
OVER	GO:0042579	microbody	CT	1.38E-04	2.16E-05	5	16	461	27181
OVER	GO:0042623	ATPase activity, coupled	MF	3.16E-11	1.45E-12	26	248	440	26949
OVER	GO:0042625	ATPase coupled ion transmembrane transporter activity	MF	5.20E-07	5.02E-08	10	49	456	27148
OVER	GO:0042626	ATPase activity, coupled to transmembrane movement of substances	MF	5.49E-10	3.07E-11	17	106	449	27091
OVER	GO:0042651	thylakoid membrane	CT	9.91E-38	1.07E-39	30	23	436	27174
OVER	GO:0042742	defense response to bacterium	BP	7.22E-07	7.13E-08	5	3	461	27194
OVER	GO:0042743	hydrogen peroxide metabolic process	BP	8.49E-12	3.68E-13	7	0	459	27197

Over/Under*	GO ID	GO Name	GO Category$	FDR	P-value	Test	Ref	Non Annot Test	Non Annot Reference
OVER	GO:0042744	hydrogen peroxide catabolic process	BP	4.34E-02	1.68E-02	1	0	465	27197
OVER	GO:0042773	ATP synthesis coupled electron transport	BP	2.97E-06	3.46E-07	6	12	460	27185
OVER	GO:0042908	xenobiotic transport	BP	4.34E-02	1.68E-02	1	0	465	27197
OVER	GO:0042910	xenobiotic transporter activity	MF	4.34E-02	1.68E-02	1	0	465	27197
OVER	GO:0042981	regulation of apoptotic process	BP	4.34E-02	1.68E-02	1	0	465	27197
OVER	GO:0043067	regulation of programmed cell death	BP	1.69E-08	1.33E-09	5	0	461	27197
OVER	GO:0043167	ion binding	MF	3.35E-26	4.70E-28	175	4402	291	22795
OVER	GO:0043168	anion binding	MF	1.26E-09	7.36E-11	102	3044	364	24153
OVER	GO:0043169	cation binding	MF	7.03E-16	1.95E-17	76	1454	390	25743
OVER	GO:0043170	macromolecule metabolic process	BP	3.13E-21	5.71E-23	164	4394	302	22803
OVER	GO:0043207	response to external biotic stimulus	BP	3.56E-16	9.59E-18	11	3	455	27194
OVER	GO:0043225	ATPase-coupled anion transmembrane transporter activity	MF	4.34E-02	1.68E-02	1	0	465	27197
OVER	GO:0043226	organelle	CT	9.34E-87	1.68E-89	172	1497	294	25700
OVER	GO:0043227	membrane-bounded organelle	CT	1.20E-98	1.08E-101	162	1043	304	26154
OVER	GO:0043228	non-membrane-bounded organelle	CT	7.12E-04	1.24E-04	22	512	444	26685
OVER	GO:0043229	intracellular organelle	CT	9.34E-87	1.68E-89	172	1497	294	25700
OVER	GO:0043231	intracellular membrane-bounded organelle	CT	3.34E-94	4.00E-97	157	1030	309	26167
OVER	GO:0043232	intracellular non-membrane-bounded organelle	CT	7.12E-04	1.24E-04	22	512	444	26685
OVER	GO:0043233	organelle lumen	CT	1.74E-02	4.19E-03	5	58	461	27139
OVER	GO:0043234	protein complex	CT	1.08E-22	1.68E-24	45	342	421	26855
OVER	GO:0043288	apocarotenoid metabolic process	BP	4.34E-02	1.68E-02	1	0	465	27197
OVER	GO:0043289	apocarotenoid biosynthetic process	BP	4.34E-02	1.68E-02	1	0	465	27197
OVER	GO:0043331	response to dsRNA	BP	4.34E-02	1.68E-02	1	0	465	27197
OVER	GO:0043412	macromolecule modification	BP	2.25E-08	1.84E-09	62	1565	404	25632
OVER	GO:0043413	macromolecule glycosylation	BP	2.86E-05	3.72E-06	8	47	458	27150
OVER	GO:0043414	macromolecule methylation	BP	2.86E-03	6.13E-04	6	56	460	27141
OVER	GO:0043436	oxoacid metabolic process	BP	1.95E-07	1.80E-08	26	397	440	26800
OVER	GO:0043492	ATPase activity, coupled to movement of substances	MF	7.17E-11	3.43E-12	18	106	448	27091
OVER	GO:0043531**	ADP binding*	MF	2.20E-06	2.50E-07	22	338	444	26859
OVER	GO:0043543	protein acylation	BP	2.83E-04	4.63E-05	3	2	463	27195
OVER	GO:0043547	positive regulation of GTPase activity	BP	4.34E-02	1.68E-02	1	0	465	27197
OVER	GO:0043572	plastid fission	BP	4.34E-02	1.68E-02	1	0	465	27197
OVER	GO:0043574	peroxisomal transport	BP	7.35E-07	7.95E-08	4	0	462	27197
OVER	GO:0043596	nuclear replication fork	CT	4.34E-02	1.68E-02	1	0	465	27197
OVER	GO:0043601	nuclear replisome	CT	4.34E-02	1.68E-02	1	0	465	27197
OVER	GO:0043623	cellular protein complex assembly	BP	2.37E-04	3.87E-05	6	32	460	27165
OVER	GO:0043628	ncRNA 3'-end processing	BP	4.34E-02	1.68E-02	1	0	465	27197
OVER	GO:0043649	dicarboxylic acid catabolic process	BP	4.34E-02	1.68E-02	1	0	465	27197
OVER	GO:0043687	post-translational protein modification	BP	9.52E-06	1.16E-06	4	2	462	27195

Over/Under*	GO ID	GO Name	GO Category$	FDR	P-value	Test	Ref	Non Annot Test	Non Annot Reference
OVER	GO:0043891	glyceraldehyde-3-phosphate dehydrogenase (NAD(P)+) (phosphorylating) activity	MF	4.34E-02	1.68E-02	1	0	465	27197
OVER	GO:0043933	macromolecular complex subunit organization	BP	1.19E-02	2.78E-03	7	104	459	27093
OVER	GO:0044003	modification by symbiont of host morphology or physiology	BP	4.34E-02	1.68E-02	1	0	465	27197
OVER	GO:0044036	cell wall macromolecule metabolic process	BP	1.27E-04	1.97E-05	7	43	459	27154
OVER	GO:0044038	cell wall macromolecule biosynthetic process	BP	2.44E-09	1.53E-10	6	1	460	27196
OVER	GO:0044085	cellular component biogenesis	BP	1.88E-06	2.13E-07	15	155	451	27042
OVER	GO:0044089	positive regulation of cellular component biogenesis	BP	4.34E-02	1.68E-02	1	0	465	27197
OVER	GO:0044237	cellular metabolic process	BP	3.86E-39	3.12E-41	214	4997	252	22200
OVER	GO:0044238	primary metabolic process	BP	9.54E-30	1.26E-31	212	5697	254	21500
OVER	GO:0044242	cellular lipid catabolic process	BP	1.35E-03	2.50E-04	3	5	463	27192
OVER	GO:0044248	cellular catabolic process	BP	1.73E-02	4.15E-03	9	175	457	27022
OVER	GO:0044249	cellular biosynthetic process	BP	7.95E-15	2.35E-16	102	2482	364	24715
OVER	GO:0044255	cellular lipid metabolic process	BP	1.25E-02	2.93E-03	11	232	455	26965
OVER	GO:0044260	cellular macromolecule metabolic process	BP	4.91E-21	9.26E-23	153	3961	313	23236
OVER	GO:0044262	cellular carbohydrate metabolic process	BP	2.28E-02	5.55E-03	10	218	456	26979
OVER	GO:0044267	cellular protein metabolic process	BP	2.89E-09	1.85E-10	78	2098	388	25099
OVER	GO:0044271	cellular nitrogen compound biosynthetic process	BP	2.64E-10	1.32E-11	78	1980	388	25217
OVER	GO:0044272	sulfur compound biosynthetic process	BP	2.20E-05	2.79E-06	8	45	458	27152
OVER	GO:0044281	small molecule metabolic process	BP	3.70E-12	1.44E-13	44	668	422	26529
OVER	GO:0044282	small molecule catabolic process	BP	1.50E-04	2.37E-05	6	29	460	27168
OVER	GO:0044283	small molecule biosynthetic process	BP	6.70E-04	1.15E-04	13	210	453	26987
OVER	GO:0044391	ribosomal subunit	CT	9.59E-03	2.21E-03	4	29	462	27168
OVER	GO:0044422	organelle part	CT	3.03E-63	1.45E-65	88	384	378	26813
OVER	GO:0044423	virion part	CT	4.34E-02	1.68E-02	1	0	465	27197
OVER	GO:0044424	intracellular part	CT	2.81E-80	7.56E-83	185	1992	281	25205
OVER	GO:0044425	membrane part	CT	3.71E-24	5.43E-26	79	1104	387	26093
OVER	GO:0044426	cell wall part	CT	1.35E-03	2.83E-04	2	0	464	27197
OVER	GO:0044427	chromosomal part	CT	2.49E-02	6.17E-03	5	64	461	27133
OVER	GO:0044428	nuclear part	CT	1.98E-11	8.83E-13	16	69	450	27128
OVER	GO:0044430	cytoskeletal part	CT	1.84E-03	3.88E-04	6	51	460	27146
OVER	GO:0044433	cytoplasmic vesicle part	CT	5.53E-04	9.47E-05	5	23	461	27174
OVER	GO:0044434	chloroplast part	CT	6.00E-79	1.79E-81	45	0	421	27197
OVER	GO:0044435	plastid part	CT	1.14E-80	2.74E-83	46	0	420	27197
OVER	GO:0044436	thylakoid part	CT	2.48E-36	2.90E-38	35	57	431	27140
OVER	GO:0044437	vacuolar part	CT	2.70E-06	3.12E-07	5	5	461	27192
OVER	GO:0044444	cytoplasmic part	CT	1.51E-84	3.15E-87	129	703	337	26494
OVER	GO:0044446	intracellular organelle part	CT	3.03E-63	1.45E-65	88	384	378	26813
OVER	GO:0044448	cell cortex part	CT	4.34E-02	1.25E-02	3	26	463	27171
OVER	GO:0044454	nuclear chromosome part	CT	2.83E-04	4.63E-05	3	2	463	27195

Over/Under*	GO ID	GO Name	GO Category$	FDR	P-value	Test	Ref	Non Annot Test	Non Annot Reference
OVER	GO:0044462	external encapsulating structure part	CT	1.35E-03	2.83E-04	2	0	464	27197
OVER	GO:0044464	cell part	CT	2.50E-72	9.74E-75	198	2630	268	24567
OVER	GO:0044699	single-organism process	BP	2.78E-34	3.33E-36	174	3738	292	23459
OVER	GO:0044700	single organism signaling	BP	1.44E-06	1.60E-07	25	416	441	26781
OVER	GO:0044702	single organism reproductive process	BP	2.07E-10	1.02E-11	15	69	451	27128
OVER	GO:0044707	single-multicellular organism process	BP	4.34E-41	3.37E-43	32	22	434	27175
OVER	GO:0044710	single-organism metabolic process	BP	1.02E-18	2.23E-20	111	2477	355	24720
OVER	GO:0044711	single-organism biosynthetic process	BP	6.22E-10	3.51E-11	38	611	428	26586
OVER	GO:0044712	single-organism catabolic process	BP	1.16E-03	2.06E-04	8	87	458	27110
OVER	GO:0044723	single-organism carbohydrate metabolic process	BP	1.70E-04	2.70E-05	16	266	450	26931
OVER	GO:0044728	DNA methylation or demethylation	BP	4.34E-02	1.17E-02	2	8	464	27189
OVER	GO:0044743	protein transmembrane import into intracellular organelle	BP	2.70E-05	3.47E-06	5	10	461	27187
OVER	GO:0044744	protein targeting to nucleus	BP	2.93E-02	7.41E-03	2	6	464	27191
OVER	GO:0044763	single-organism cellular process	BP	1.30E-21	2.29E-23	97	1791	369	25406
OVER	GO:0044764	multi-organism cellular process	BP	4.34E-02	1.68E-02	1	0	465	27197
OVER	GO:0044765	single-organism transport	BP	6.56E-14	2.14E-15	29	238	437	26959
OVER	GO:0044767	single-organism developmental process	BP	3.97E-38	3.56E-40	34	42	432	27155
OVER	GO:0044769	ATPase activity, coupled to transmembrane movement of ions, rotational mechanism	MF	1.55E-07	1.41E-08	8	20	458	27177
OVER	GO:0045010	actin nucleation	BP	4.34E-02	1.68E-02	1	0	465	27197
OVER	GO:0045036	protein targeting to chloroplast	BP	4.34E-02	1.68E-02	1	0	465	27197
OVER	GO:0045038	protein import into chloroplast thylakoid membrane	BP	4.34E-02	1.68E-02	1	0	465	27197
OVER	GO:0045087	innate immune response	BP	1.15E-03	2.03E-04	9	111	457	27086
OVER	GO:0045088	regulation of innate immune response	BP	7.35E-07	7.95E-08	4	0	462	27197
OVER	GO:0045089	positive regulation of innate immune response	BP	4.34E-02	1.68E-02	1	0	465	27197
OVER	GO:0045143	homologous chromosome segregation	BP	4.34E-02	1.68E-02	1	0	465	27197
OVER	GO:0045156	electron transporter, transferring electrons within the cyclic electron transport pathway of photosynthesis activity	MF	2.19E-08	1.78E-09	6	3	460	27194
OVER	GO:0045158	electron transporter, transferring electrons within cytochrome b6/f complex of photosystem II activity	MF	1.35E-03	2.83E-04	2	0	464	27197
OVER	GO:0045184	establishment of protein localization	BP	2.76E-11	1.26E-12	24	205	442	26992
OVER	GO:0045216	cell-cell junction organization	BP	4.34E-02	1.68E-02	1	0	465	27197
OVER	GO:0045229	external encapsulating structure organization	BP	4.34E-02	1.38E-02	5	79	461	27118
OVER	GO:0045259	proton-transporting ATP synthase complex	CT	1.14E-07	1.02E-08	8	19	458	27178
OVER	GO:0045261**	proton-transporting ATP synthase complex, catalytic core F(1)*	CT	2.70E-05	3.47E-06	5	10	461	27187

Over/Under*	GO ID	GO Name	GO Category$	FDR	P-value	Test	Ref	Non Annot Test	Non Annot Reference
OVER	GO:0045263**	proton-transporting ATP synthase complex, coupling factor F(o)*	CT	4.25E-03	9.33E-04	3	9	463	27188
OVER	GO:0045289	luciferin monooxygenase activity	MF	4.34E-02	1.68E-02	1	0	465	27197
OVER	GO:0045332	phospholipid translocation	BP	4.34E-02	1.68E-02	1	0	465	27197
OVER	GO:0045333	cellular respiration	BP	7.12E-06	8.60E-07	7	25	459	27172
OVER	GO:0045491	xylan metabolic process	BP	8.49E-12	3.68E-13	7	0	459	27197
OVER	GO:0045492	xylan biosynthetic process	BP	4.00E-10	2.21E-11	6	0	460	27197
OVER	GO:0045595	regulation of cell differentiation	BP	4.34E-02	1.68E-02	1	0	465	27197
OVER	GO:0045787	positive regulation of cell cycle	BP	4.34E-02	1.68E-02	1	0	465	27197
OVER	GO:0045893	positive regulation of transcription, DNA-templated	BP	6.20E-05	9.37E-06	4	5	462	27192
OVER	GO:0045935	positive regulation of nucleobase-containing compound metabolic process	BP	6.20E-05	9.37E-06	4	5	462	27192
OVER	GO:0046034	ATP metabolic process	BP	7.07E-09	4.80E-10	16	111	450	27086
OVER	GO:0046128	purine ribonucleoside metabolic process	BP	5.42E-08	4.65E-09	16	132	450	27065
OVER	GO:0046129	purine ribonucleoside biosynthetic process	BP	1.21E-04	1.86E-05	8	60	458	27137
OVER	GO:0046148	pigment biosynthetic process	BP	1.07E-02	2.47E-03	4	30	462	27167
OVER	GO:0046189	phenol-containing compound biosynthetic process	BP	4.34E-02	1.68E-02	1	0	465	27197
OVER	GO:0046217	indole phytoalexin metabolic process	BP	4.34E-02	1.68E-02	1	0	465	27197
OVER	GO:0046364	monosaccharide biosynthetic process	BP	3.68E-02	9.43E-03	2	7	464	27190
OVER	GO:0046390	ribose phosphate biosynthetic process	BP	2.89E-05	3.78E-06	9	64	457	27133
OVER	GO:0046394	carboxylic acid biosynthetic process	BP	1.56E-04	2.48E-05	13	179	453	27018
OVER	GO:0046395	carboxylic acid catabolic process	BP	3.01E-03	6.46E-04	4	20	462	27177
OVER	GO:0046470	phosphatidylcholine metabolic process	BP	4.34E-02	1.68E-02	1	0	465	27197
OVER	GO:0046482	para-aminobenzoic acid metabolic process	BP	1.35E-03	2.83E-04	2	0	464	27197
OVER	GO:0046483	heterocycle metabolic process	BP	4.37E-22	7.25E-24	108	2136	358	25061
OVER	GO:0046501	protoporphyrinogen IX metabolic process	BP	4.34E-02	1.68E-02	1	0	465	27197
OVER	GO:0046519	sphingoid metabolic process	BP	4.34E-02	1.68E-02	1	0	465	27197
OVER	GO:0046520	sphingoid biosynthetic process	BP	4.34E-02	1.68E-02	1	0	465	27197
OVER	GO:0046540**	U4/U6 x U5 tri-snRNP complex*	CT	4.34E-02	1.68E-02	1	0	465	27197
OVER	GO:0046658**	anchored component of plasma membrane*	CT	4.34E-02	1.68E-02	1	0	465	27197
OVER	GO:0046777	protein autophosphorylation	BP	1.35E-03	2.83E-04	2	0	464	27197
OVER	GO:0046785	microtubule polymerization	BP	3.83E-03	8.40E-04	2	1	464	27196
OVER	GO:0046872	metal ion binding	MF	4.16E-16	1.13E-17	76	1439	390	25758
OVER	GO:0046907	intracellular transport	BP	3.16E-10	1.60E-11	20	153	446	27044
OVER	GO:0046914	transition metal ion binding	MF	2.08E-08	1.68E-09	48	1045	418	26152
OVER	GO:0046933	proton-transporting ATP synthase activity, rotational mechanism	MF	1.97E-08	1.59E-09	8	14	458	27183
OVER	GO:0046961	proton-transporting ATPase activity, rotational mechanism	MF	1.72E-04	2.76E-05	5	17	461	27180
OVER	GO:0046983	protein dimerization activity	MF	3.42E-03	7.43E-04	12	225	454	26972

Over/Under*	GO ID	GO Name	GO Category$	FDR	P-value	Test	Ref	Non Annot Test	Non Annot Reference
OVER	GO:0047077**	Photinus-luciferin 4-monooxygenase (ATP-hydrolyzing) activity*	MF	4.34E-02	1.68E-02	1	0	465	27197
OVER	GO:0047172	shikimate O-hydroxycinnamoyltransferase activity	MF	3.27E-05	4.75E-06	3	0	463	27197
OVER	GO:0047209**	coniferyl-alcohol glucosyltransferase activity*	MF	4.34E-02	1.68E-02	1	0	465	27197
OVER	GO:0047220**	galactosylxylosylprotein 3-beta-galactosyltransferase activity*	MF	1.35E-03	2.83E-04	2	0	464	27197
OVER	GO:0047372	acylglycerol lipase activity	MF	4.34E-02	1.68E-02	1	0	465	27197
OVER	GO:0047513	1,2-alpha-L-fucosidase activity	MF	4.34E-02	1.68E-02	1	0	465	27197
OVER	GO:0047672	anthranilate N-benzoyltransferase activity	MF	4.34E-02	1.68E-02	1	0	465	27197
OVER	GO:0048037	cofactor binding	MF	2.16E-03	4.59E-04	21	517	445	26680
OVER	GO:0048038	quinone binding	MF	2.59E-09	1.63E-10	9	16	457	27181
OVER	GO:0048193	Golgi vesicle transport	BP	8.84E-06	1.08E-06	7	26	459	27171
OVER	GO:0048229	gametophyte development	BP	7.35E-07	7.95E-08	4	0	462	27197
OVER	GO:0048285	organelle fission	BP	1.81E-02	4.37E-03	3	17	463	27180
OVER	GO:0048316	seed development	BP	1.75E-13	6.11E-15	8	0	458	27197
OVER	GO:0048364	root development	BP	1.69E-08	1.33E-09	5	0	461	27197
OVER	GO:0048366	leaf development	BP	1.69E-08	1.33E-09	5	0	461	27197
OVER	GO:0048367	shoot system development	BP	4.37E-22	7.45E-24	13	0	453	27197
OVER	GO:0048437	floral organ development	BP	1.69E-08	1.33E-09	5	0	461	27197
OVER	GO:0048438	floral whorl development	BP	1.35E-03	2.83E-04	2	0	464	27197
OVER	GO:0048439	flower morphogenesis	BP	4.34E-02	1.68E-02	1	0	465	27197
OVER	GO:0048440	carpel development	BP	4.34E-02	1.68E-02	1	0	465	27197
OVER	GO:0048444	floral organ morphogenesis	BP	1.35E-03	2.83E-04	2	0	464	27197
OVER	GO:0048449	floral organ formation	BP	1.35E-03	2.83E-04	2	0	464	27197
OVER	GO:0048467	gynoecium development	BP	4.34E-02	1.68E-02	1	0	465	27197
OVER	GO:0048468	cell development	BP	7.35E-07	7.95E-08	4	0	462	27197
OVER	GO:0048469	cell maturation	BP	4.34E-02	1.68E-02	1	0	465	27197
OVER	GO:0048475	coated membrane	CT	6.14E-03	1.38E-03	5	44	461	27153
OVER	GO:0048481	plant ovule development	BP	4.34E-02	1.68E-02	1	0	465	27197
OVER	GO:0048507	meristem development	BP	1.69E-08	1.33E-09	5	0	461	27197
OVER	GO:0048508	embryonic meristem development	BP	1.35E-03	2.83E-04	2	0	464	27197
OVER	GO:0048509	regulation of meristem development	BP	4.34E-02	1.68E-02	1	0	465	27197
OVER	GO:0048513	animal organ development	BP	4.34E-02	1.68E-02	1	0	465	27197
OVER	GO:0048518	positive regulation of biological process	BP	3.50E-12	1.35E-13	10	9	456	27188
OVER	GO:0048519	negative regulation of biological process	BP	1.12E-07	9.98E-09	7	11	459	27186
OVER	GO:0048522	positive regulation of cellular process	BP	4.35E-08	3.69E-09	7	9	459	27188
OVER	GO:0048523	negative regulation of cellular process	BP	5.39E-03	1.20E-03	3	10	463	27187
OVER	GO:0048532	anatomical structure arrangement	BP	3.27E-05	4.75E-06	3	0	463	27197
OVER	GO:0048573	photoperiodism, flowering	BP	4.34E-02	1.68E-02	1	0	465	27197
OVER	GO:0048580	regulation of post-embryonic development	BP	1.69E-08	1.33E-09	5	0	461	27197

Over/Under*	GO ID	GO Name	GO Category$	FDR	P-value	Test	Ref	Non Annot Test	Non Annot Reference
OVER	GO:0048582	positive regulation of post-embryonic development	BP	1.35E-03	2.83E-04	2	0	464	27197
OVER	GO:0048583	regulation of response to stimulus	BP	2.54E-07	2.36E-08	7	13	459	27184
OVER	GO:0048584	positive regulation of response to stimulus	BP	1.35E-03	2.83E-04	2	0	464	27197
OVER	GO:0048588	developmental cell growth	BP	4.34E-02	1.68E-02	1	0	465	27197
OVER	GO:0048589	developmental growth	BP	1.35E-03	2.83E-04	2	0	464	27197
OVER	GO:0048608	reproductive structure development	BP	5.76E-38	5.86E-40	22	0	444	27197
OVER	GO:0048609	multicellular organismal reproductive process	BP	1.69E-08	1.33E-09	5	0	461	27197
OVER	GO:0048638	regulation of developmental growth	BP	4.34E-02	1.68E-02	1	0	465	27197
OVER	GO:0048645	animal organ formation	BP	4.34E-02	1.68E-02	1	0	465	27197
OVER	GO:0048646	anatomical structure formation involved in morphogenesis	BP	3.27E-05	4.75E-06	3	0	463	27197
OVER	GO:0048700	acquisition of desiccation tolerance in seed	BP	4.34E-02	1.68E-02	1	0	465	27197
OVER	GO:0048729	tissue morphogenesis	BP	4.34E-02	1.68E-02	1	0	465	27197
OVER	GO:0048731	system development	BP	5.36E-45	3.85E-47	26	0	440	27197
OVER	GO:0048764	trichoblast maturation	BP	4.34E-02	1.68E-02	1	0	465	27197
OVER	GO:0048765	root hair cell differentiation	BP	4.34E-02	1.68E-02	1	0	465	27197
OVER	GO:0048825	cotyledon development	BP	3.27E-05	4.75E-06	3	0	463	27197
OVER	GO:0048827	phyllome development	BP	8.49E-12	3.68E-13	7	0	459	27197
OVER	GO:0048831	regulation of shoot system development	BP	7.35E-07	7.95E-08	4	0	462	27197
OVER	GO:0048856	anatomical structure development	BP	3.97E-38	3.56E-40	34	42	432	27155
OVER	GO:0048868	pollen tube development	BP	4.34E-02	1.68E-02	1	0	465	27197
OVER	GO:0048869	cellular developmental process	BP	2.44E-09	1.53E-10	10	24	456	27173
OVER	GO:0048878	chemical homeostasis	BP	2.28E-02	5.62E-03	2	5	464	27192
OVER	GO:0050136	NADH dehydrogenase (quinone) activity	MF	1.97E-08	1.59E-09	8	14	458	27183
OVER	GO:0050203**	oxalate-CoA ligase activity*	MF	4.34E-02	1.68E-02	1	0	465	27197
OVER	GO:0050291**	sphingosine N-acyltransferase activity*	MF	4.34E-02	1.68E-02	1	0	465	27197
OVER	GO:0050347	trans-octaprenyltranstransferase activity	MF	4.34E-02	1.68E-02	1	0	465	27197
OVER	GO:0050356**	tropine dehydrogenase activity*	MF	4.34E-02	1.68E-02	1	0	465	27197
OVER	GO:0050505	hydroquinone glucosyltransferase activity	MF	4.34E-02	1.68E-02	1	0	465	27197
OVER	GO:0050661**	NADP binding*	MF	2.22E-02	5.37E-03	4	38	462	27159
OVER	GO:0050665	hydrogen peroxide biosynthetic process	BP	4.00E-10	2.21E-11	6	0	460	27197
OVER	GO:0050734	hydroxycinnamoyltransferase activity	MF	3.27E-05	4.75E-06	3	0	463	27197
OVER	GO:0050737	O-hydroxycinnamoyltransferase activity	MF	3.27E-05	4.75E-06	3	0	463	27197
OVER	GO:0050776	regulation of immune response	BP	7.35E-07	7.95E-08	4	0	462	27197
OVER	GO:0050778	positive regulation of immune response	BP	4.34E-02	1.68E-02	1	0	465	27197
OVER	GO:0050789	regulation of biological process	BP	2.27E-11	1.03E-12	75	1749	391	25448
OVER	GO:0050793	regulation of developmental process	BP	1.75E-13	6.11E-15	8	0	458	27197
OVER	GO:0050794	regulation of cellular process	BP	2.59E-09	1.63E-10	69	1737	397	25460
OVER	GO:0050801	ion homeostasis	BP	2.28E-02	5.62E-03	2	5	464	27192

Over/Under*	GO ID	GO Name	GO Category[$]	FDR	P-value	Test	Ref	Non Annot Test	Non Annot Reference
OVER	GO:0050821	protein stabilization	BP	1.35E-03	2.83E-04	2	0	464	27197
OVER	GO:0050826	response to freezing	BP	4.34E-02	1.68E-02	1	0	465	27197
OVER	GO:0050832	defense response to fungus	BP	2.11E-05	2.67E-06	4	3	462	27194
OVER	GO:0050896	response to stimulus	BP	1.84E-16	4.78E-18	62	976	404	26221
OVER	GO:0051020	GTPase binding	MF	3.83E-03	8.40E-04	2	1	464	27196
OVER	GO:0051052	regulation of DNA metabolic process	BP	2.44E-03	5.22E-04	3	7	463	27190
OVER	GO:0051094	positive regulation of developmental process	BP	1.35E-03	2.83E-04	2	0	464	27197
OVER	GO:0051169	nuclear transport	BP	4.34E-02	1.17E-02	2	8	464	27189
OVER	GO:0051170	nuclear import	BP	2.93E-02	7.41E-03	2	6	464	27191
OVER	GO:0051171	regulation of nitrogen compound metabolic process	BP	3.21E-03	6.90E-04	38	1229	428	25968
OVER	GO:0051172	negative regulation of nitrogen compound metabolic process	BP	1.70E-02	4.06E-03	2	4	464	27193
OVER	GO:0051173	positive regulation of nitrogen compound metabolic process	BP	3.03E-04	5.04E-05	4	9	462	27188
OVER	GO:0051179	localization	BP	1.28E-21	2.23E-23	83	1342	383	25855
OVER	GO:0051181	cofactor transport	BP	4.34E-02	1.68E-02	1	0	465	27197
OVER	GO:0051184	cofactor transporter activity	MF	4.34E-02	1.68E-02	1	0	465	27197
OVER	GO:0051234	establishment of localization	BP	4.28E-20	8.83E-22	80	1332	386	25865
OVER	GO:0051239	regulation of multicellular organismal process	BP	4.00E-10	2.21E-11	6	0	460	27197
OVER	GO:0051240	positive regulation of multicellular organismal process	BP	1.35E-03	2.83E-04	2	0	464	27197
OVER	GO:0051246	regulation of protein metabolic process	BP	4.34E-02	1.14E-02	3	25	463	27172
OVER	GO:0051252	regulation of RNA metabolic process	BP	4.34E-02	1.26E-02	33	1216	433	25981
OVER	GO:0051254	positive regulation of RNA metabolic process	BP	6.20E-05	9.37E-06	4	5	462	27192
OVER	GO:0051258	protein polymerization	BP	4.34E-02	1.38E-02	3	27	463	27170
OVER	GO:0051276	chromosome organization	BP	1.91E-05	2.40E-06	11	98	455	27099
OVER	GO:0051301	cell division	BP	7.31E-03	1.66E-03	2	2	464	27195
OVER	GO:0051345	positive regulation of hydrolase activity	BP	4.34E-02	1.68E-02	1	0	465	27197
OVER	GO:0051495	positive regulation of cytoskeleton organization	BP	4.34E-02	1.68E-02	1	0	465	27197
OVER	GO:0051536	iron-sulfur cluster binding	MF	5.50E-05	8.23E-06	8	53	458	27144
OVER	GO:0051539**	4 iron, 4 sulfur cluster binding*	MF	1.71E-07	1.56E-08	7	12	459	27185
OVER	GO:0051540	metal cluster binding	MF	5.50E-05	8.23E-06	8	53	458	27144
OVER	GO:0051567	histone H3-K9 methylation	BP	4.34E-02	1.68E-02	1	0	465	27197
OVER	GO:0051601	exocyst localization	BP	4.34E-02	1.68E-02	1	0	465	27197
OVER	GO:0051607	defense response to virus	BP	4.34E-02	1.68E-02	1	0	465	27197
OVER	GO:0051640	organelle localization	BP	4.34E-02	1.68E-02	1	0	465	27197
OVER	GO:0051641	cellular localization	BP	3.99E-12	1.57E-13	23	166	443	27031
OVER	GO:0051644	plastid localization	BP	4.34E-02	1.68E-02	1	0	465	27197
OVER	GO:0051649	establishment of localization in cell	BP	4.57E-11	2.13E-12	21	153	445	27044
OVER	GO:0051656	establishment of organelle localization	BP	4.34E-02	1.68E-02	1	0	465	27197
OVER	GO:0051667	establishment of plastid localization	BP	4.34E-02	1.68E-02	1	0	465	27197
OVER	GO:0051701	interaction with host	BP	4.34E-02	1.68E-02	1	0	465	27197

Over/Under*	GO ID	GO Name	GO Category$	FDR	P-value	Test	Ref	Non Annot Test	Non Annot Reference
OVER	GO:0051707	response to other organism	BP	3.56E-16	9.59E-18	11	3	455	27194
OVER	GO:0051716	cellular response to stimulus	BP	1.84E-07	1.69E-08	32	576	434	26621
OVER	GO:0051726	regulation of cell cycle	BP	6.84E-03	1.54E-03	4	26	462	27171
OVER	GO:0051817	modification of morphology or physiology of other organism involved in symbiotic interaction	BP	4.34E-02	1.68E-02	1	0	465	27197
OVER	GO:0051996**	squalene synthase activity*	MF	4.34E-02	1.68E-02	1	0	465	27197
OVER	GO:0052018	modulation by symbiont of RNA levels in host	BP	4.34E-02	1.68E-02	1	0	465	27197
OVER	GO:0052249	modulation of RNA levels in other organism involved in symbiotic interaction	BP	4.34E-02	1.68E-02	1	0	465	27197
OVER	GO:0052314	phytoalexin metabolic process	BP	4.34E-02	1.68E-02	1	0	465	27197
OVER	GO:0052315	phytoalexin biosynthetic process	BP	4.34E-02	1.68E-02	1	0	465	27197
OVER	GO:0052386	cell wall thickening	BP	1.35E-03	2.83E-04	2	0	464	27197
OVER	GO:0052482	defense response by cell wall thickening	BP	1.35E-03	2.83E-04	2	0	464	27197
OVER	GO:0052542	defense response by callose deposition	BP	1.35E-03	2.83E-04	2	0	464	27197
OVER	GO:0052543	callose deposition in cell wall	BP	1.35E-03	2.83E-04	2	0	464	27197
OVER	GO:0052544	defense response by callose deposition in cell wall	BP	1.35E-03	2.83E-04	2	0	464	27197
OVER	GO:0052545	callose localization	BP	1.35E-03	2.83E-04	2	0	464	27197
OVER	GO:0052745	inositol phosphate phosphatase activity	MF	4.34E-02	1.68E-02	1	0	465	27197
OVER	GO:0052852**	very-long-chain-(S)-2-hydroxy-acid oxidase activity*	MF	1.35E-03	2.83E-04	2	0	464	27197
OVER	GO:0052853**	long-chain-(S)-2-hydroxy-long-chain-acid oxidase activity*	MF	1.35E-03	2.83E-04	2	0	464	27197
OVER	GO:0052854	medium-chain-(S)-2-hydroxy-acid oxidase activity	MF	1.35E-03	2.83E-04	2	0	464	27197
OVER	GO:0052923	all-trans-nonaprenyl-diphosphate synthase (geranyl-diphosphate specific) activity	MF	4.34E-02	1.68E-02	1	0	465	27197
OVER	GO:0055035	plastid thylakoid membrane	CT	3.71E-52	2.44E-54	30	0	436	27197
OVER	GO:0055044	symplast	CT	1.20E-18	2.76E-20	11	0	455	27197
OVER	GO:0055065	metal ion homeostasis	BP	2.28E-02	5.62E-03	2	5	464	27192
OVER	GO:0055067	monovalent inorganic cation homeostasis	BP	4.34E-02	1.68E-02	1	0	465	27197
OVER	GO:0055069	zinc ion homeostasis	BP	4.34E-02	1.68E-02	1	0	465	27197
OVER	GO:0055070	copper ion homeostasis	BP	4.34E-02	1.68E-02	1	0	465	27197
OVER	GO:0055075	potassium ion homeostasis	BP	4.34E-02	1.68E-02	1	0	465	27197
OVER	GO:0055080	cation homeostasis	BP	2.28E-02	5.62E-03	2	5	464	27192
OVER	GO:0055082	cellular chemical homeostasis	BP	2.28E-02	5.62E-03	2	5	464	27192
OVER	GO:0055085	transmembrane transport	BP	2.60E-06	2.98E-07	33	692	433	26505
OVER	GO:0055086	nucleobase-containing small molecule metabolic process	BP	7.35E-07	7.80E-08	18	211	448	26986
OVER	GO:0055114	oxidation-reduction process	BP	3.71E-10	1.89E-11	63	1415	403	25782
OVER	GO:0060255	regulation of macromolecule metabolic process	BP	2.25E-03	4.80E-04	39	1241	427	25956
OVER	GO:0060429	epithelium development	BP	4.34E-02	1.68E-02	1	0	465	27197
OVER	GO:0060560	developmental growth involved in morphogenesis	BP	4.34E-02	1.68E-02	1	0	465	27197
OVER	GO:0060918	auxin transport	BP	4.34E-02	1.68E-02	1	0	465	27197
OVER	GO:0061024	membrane organization	BP	2.33E-02	5.76E-03	3	19	463	27178

Over/Under*	GO ID	GO Name	GO Category$	FDR	P-value	Test	Ref	Non Annot Test	Non Annot Reference
OVER	GO:0061458	reproductive system development	BP	5.76E-38	5.86E-40	22	0	444	27197
OVER	GO:0061647	histone H3-K9 modification	BP	4.34E-02	1.68E-02	1	0	465	27197
OVER	GO:0065001	specification of axis polarity	BP	4.34E-02	1.68E-02	1	0	465	27197
OVER	GO:0065002	intracellular protein transmembrane transport	BP	2.70E-05	3.47E-06	5	10	461	27187
OVER	GO:0065003	macromolecular complex assembly	BP	6.10E-03	1.36E-03	7	91	459	27106
OVER	GO:0065007	biological regulation	BP	1.15E-12	4.29E-14	81	1875	385	25322
OVER	GO:0065008	regulation of biological quality	BP	2.29E-03	4.88E-04	10	154	456	27043
OVER	GO:0070013	intracellular organelle lumen	CT	1.74E-02	4.19E-03	5	58	461	27139
OVER	GO:0070085	glycosylation	BP	1.34E-04	2.08E-05	8	61	458	27136
OVER	GO:0070192	chromosome organization involved in meiotic cell cycle	BP	4.34E-02	1.68E-02	1	0	465	27197
OVER	GO:0070271	protein complex biogenesis	BP	3.13E-04	5.23E-05	6	34	460	27163
OVER	GO:0070546	L-phenylalanine aminotransferase activity	MF	4.34E-02	1.68E-02	1	0	465	27197
OVER	GO:0070567	cytidylyltransferase activity	MF	4.34E-02	1.68E-02	1	0	465	27197
OVER	GO:0070574	cadmium ion transmembrane transport	BP	4.34E-02	1.68E-02	1	0	465	27197
OVER	GO:0070588	calcium ion transmembrane transport	BP	4.34E-02	1.68E-02	1	0	465	27197
OVER	GO:0070589	cellular component macromolecule biosynthetic process	BP	2.44E-09	1.53E-10	6	1	460	27196
OVER	GO:0070592	cell wall polysaccharide biosynthetic process	BP	4.00E-10	2.21E-11	6	0	460	27197
OVER	GO:0070646	protein modification by small protein removal	BP	4.00E-10	2.21E-11	6	0	460	27197
OVER	GO:0070647	protein modification by small protein conjugation or removal	BP	4.97E-06	5.91E-07	10	66	456	27131
OVER	GO:0070727	cellular macromolecule localization	BP	7.61E-12	3.07E-13	22	154	444	27043
OVER	GO:0070838	divalent metal ion transport	BP	1.72E-04	2.76E-05	5	17	461	27180
OVER	GO:0070887	cellular response to chemical stimulus	BP	8.49E-12	3.68E-13	7	0	459	27197
OVER	GO:0070918	production of small RNA involved in gene silencing by RNA	BP	4.34E-02	1.68E-02	1	0	465	27197
OVER	GO:0071156	regulation of cell cycle arrest	BP	4.34E-02	1.68E-02	1	0	465	27197
OVER	GO:0071158	positive regulation of cell cycle arrest	BP	4.34E-02	1.68E-02	1	0	465	27197
OVER	GO:0071215	cellular response to abscisic acid stimulus	BP	4.34E-02	1.68E-02	1	0	465	27197
OVER	GO:0071229	cellular response to acid chemical	BP	1.69E-08	1.33E-09	5	0	461	27197
OVER	GO:0071310	cellular response to organic substance	BP	8.49E-12	3.68E-13	7	0	459	27197
OVER	GO:0071322	cellular response to carbohydrate stimulus	BP	1.35E-03	2.83E-04	2	0	464	27197
OVER	GO:0071359	cellular response to dsRNA	BP	4.34E-02	1.68E-02	1	0	465	27197
OVER	GO:0071369	cellular response to ethylene stimulus	BP	4.34E-02	1.68E-02	1	0	465	27197
OVER	GO:0071395	cellular response to jasmonic acid stimulus	BP	7.35E-07	7.95E-08	4	0	462	27197
OVER	GO:0071396	cellular response to lipid	BP	4.34E-02	1.68E-02	1	0	465	27197
OVER	GO:0071407	cellular response to organic cyclic compound	BP	7.35E-07	7.95E-08	4	0	462	27197
OVER	GO:0071446	cellular response to salicylic acid stimulus	BP	3.27E-05	4.75E-06	3	0	463	27197

Over/Under*	GO ID	GO Name	GO Category$	FDR	P-value	Test	Ref	Non Annot Test	Non Annot Reference
OVER	GO:0071495	cellular response to endogenous stimulus	BP	1.69E-08	1.33E-09	5	0	461	27197
OVER	GO:0071554	cell wall organization or biogenesis	BP	3.27E-05	4.72E-06	12	128	454	27069
OVER	GO:0071555	cell wall organization	BP	4.34E-02	1.38E-02	5	79	461	27118
OVER	GO:0071577	zinc II ion transmembrane transport	BP	4.34E-02	1.68E-02	1	0	465	27197
OVER	GO:0071702	organic substance transport	BP	9.50E-12	4.15E-13	30	321	436	26876
OVER	GO:0071704	organic substance metabolic process	BP	2.86E-30	3.51E-32	218	5927	248	21270
OVER	GO:0071806	protein transmembrane transport	BP	2.70E-05	3.47E-06	5	10	461	27187
OVER	GO:0071822	protein complex subunit organization	BP	1.35E-03	2.60E-04	6	47	460	27150
OVER	GO:0071840	cellular component organization or biogenesis	BP	4.71E-18	1.11E-19	41	376	425	26821
OVER	GO:0071897	DNA biosynthetic process	BP	3.61E-05	5.27E-06	4	4	462	27193
OVER	GO:0071944	cell periphery	CT	8.90E-14	2.96E-15	26	185	440	27012
OVER	GO:0072329	monocarboxylic acid catabolic process	BP	1.35E-03	2.50E-04	3	5	463	27192
OVER	GO:0072330	monocarboxylic acid biosynthetic process	BP	2.22E-03	4.71E-04	7	75	459	27122
OVER	GO:0072507	divalent inorganic cation homeostasis	BP	4.34E-02	1.68E-02	1	0	465	27197
OVER	GO:0072509	divalent inorganic cation transmembrane transporter activity	MF	1.81E-02	4.37E-03	3	17	463	27180
OVER	GO:0072511	divalent inorganic cation transport	BP	2.15E-04	3.48E-05	5	18	461	27179
OVER	GO:0072521	purine-containing compound metabolic process	BP	2.66E-08	2.21E-09	17	144	449	27053
OVER	GO:0072522	purine-containing compound biosynthetic process	BP	5.46E-05	8.14E-06	9	71	457	27126
OVER	GO:0072593	reactive oxygen species metabolic process	BP	2.55E-08	2.11E-09	7	8	459	27189
OVER	GO:0072594	establishment of protein localization to organelle	BP	9.39E-08	8.28E-09	9	28	457	27169
OVER	GO:0072596	establishment of protein localization to chloroplast	BP	4.34E-02	1.68E-02	1	0	465	27197
OVER	GO:0072598	protein localization to chloroplast	BP	4.34E-02	1.68E-02	1	0	465	27197
OVER	GO:0072657	protein localization to membrane	BP	1.04E-03	1.84E-04	5	27	461	27170
OVER	GO:0072662	protein localization to peroxisome	BP	7.35E-07	7.95E-08	4	0	462	27197
OVER	GO:0072663	establishment of protein localization to peroxisome	BP	7.35E-07	7.95E-08	4	0	462	27197
OVER	GO:0072665	protein localization to vacuole	BP	4.34E-02	1.68E-02	1	0	465	27197
OVER	GO:0072666	establishment of protein localization to vacuole	BP	4.34E-02	1.68E-02	1	0	465	27197
OVER	GO:0072718	response to cisplatin	BP	4.34E-02	1.68E-02	1	0	465	27197
OVER	GO:0080008**	Cul4-RING E3 ubiquitin ligase complex*	CT	3.27E-05	4.75E-06	3	0	463	27197
OVER	GO:0080025	phosphatidylinositol-3,5-bisphosphate binding	MF	4.34E-02	1.68E-02	1	0	465	27197
OVER	GO:0080051	cutin transport	BP	4.34E-02	1.68E-02	1	0	465	27197
OVER	GO:0080085**	signal recognition particle, chloroplast targeting*	CT	4.34E-02	1.68E-02	1	0	465	27197
OVER	GO:0080090	regulation of primary metabolic process	BP	3.42E-03	7.43E-04	38	1240	428	25957
OVER	GO:0080123	jasmonate-amino synthetase activity	MF	4.34E-02	1.68E-02	1	0	465	27197

Over/Under*	GO ID	GO Name	GO Category$	FDR	P-value	Test	Ref	Non Annot Test	Non Annot Reference
OVER	GO:0080130	L-phenylalanine:2-oxoglutarate aminotransferase activity	MF	4.34E-02	1.68E-02	1	0	465	27197
OVER	GO:0080134	regulation of response to stress	BP	2.44E-09	1.53E-10	6	1	460	27196
OVER	GO:0080135	regulation of cellular response to stress	BP	3.34E-06	3.92E-07	4	1	462	27196
OVER	GO:0080172	petal epidermis patterning	BP	4.34E-02	1.68E-02	1	0	465	27197
OVER	GO:0090068	positive regulation of cell cycle process	BP	4.34E-02	1.68E-02	1	0	465	27197
OVER	GO:0090150	establishment of protein localization to membrane	BP	1.04E-03	1.84E-04	5	27	461	27170
OVER	GO:0090304	nucleic acid metabolic process	BP	2.72E-17	6.76E-19	89	1803	377	25394
OVER	GO:0090305	nucleic acid phosphodiester bond hydrolysis	BP	1.35E-17	3.26E-19	11	1	455	27196
OVER	GO:0090322	regulation of superoxide metabolic process	BP	4.34E-02	1.68E-02	1	0	465	27197
OVER	GO:0090351	seedling development	BP	7.35E-07	7.95E-08	4	0	462	27197
OVER	GO:0090407	organophosphate biosynthetic process	BP	2.03E-05	2.55E-06	12	120	454	27077
OVER	GO:0090421	embryonic meristem initiation	BP	4.34E-02	1.68E-02	1	0	465	27197
OVER	GO:0090501	RNA phosphodiester bond hydrolysis	BP	3.34E-06	3.92E-07	4	1	462	27196
OVER	GO:0090502	RNA phosphodiester bond hydrolysis, endonucleolytic	BP	1.35E-03	2.83E-04	2	0	464	27197
OVER	GO:0090503	RNA phosphodiester bond hydrolysis, exonucleolytic	BP	4.34E-02	1.68E-02	1	0	465	27197
OVER	GO:0090558	plant epidermis development	BP	3.27E-05	4.75E-06	3	0	463	27197
OVER	GO:0090567	reproductive shoot system development	BP	1.75E-13	6.11E-15	8	0	458	27197
OVER	GO:0090576	RNA polymerase III transcription factor complex	CT	1.35E-03	2.83E-04	2	0	464	27197
OVER	GO:0090603	sieve element differentiation	BP	1.35E-03	2.83E-04	2	0	464	27197
OVER	GO:0090626	plant epidermis morphogenesis	BP	4.34E-02	1.68E-02	1	0	465	27197
OVER	GO:0090627	plant epidermal cell differentiation	BP	4.34E-02	1.68E-02	1	0	465	27197
OVER	GO:0090662	ATP hydrolysis coupled transmembrane transport	BP	8.57E-03	1.97E-03	4	28	462	27169
OVER	GO:0090693	plant organ senescence	BP	1.35E-03	2.83E-04	2	0	464	27197
OVER	GO:0090696	post-embryonic plant organ development	BP	1.35E-03	2.83E-04	2	0	464	27197
OVER	GO:0090697	post-embryonic plant organ morphogenesis	BP	1.35E-03	2.83E-04	2	0	464	27197
OVER	GO:0090698	post-embryonic plant morphogenesis	BP	3.27E-05	4.75E-06	3	0	463	27197
OVER	GO:0097035	regulation of membrane lipid distribution	BP	4.34E-02	1.68E-02	1	0	465	27197
OVER	GO:0097159	organic cyclic compound binding	MF	6.27E-30	8.06E-32	208	5504	258	21693
OVER	GO:0097164	ammonium ion metabolic process	BP	2.28E-02	5.62E-03	2	5	464	27192
OVER	GO:0097218**	sieve plate*	CT	1.35E-03	2.83E-04	2	0	464	27197
OVER	GO:0097305	response to alcohol	BP	3.27E-05	4.75E-06	3	0	463	27197
OVER	GO:0097306	cellular response to alcohol	BP	4.34E-02	1.68E-02	1	0	465	27197
OVER	GO:0097367	carbohydrate derivative binding	MF	6.21E-10	3.49E-11	95	2713	371	24484
OVER	GO:0097435	supramolecular fiber organization	BP	3.27E-03	7.09E-04	3	8	463	27189
OVER	GO:0097439	acquisition of desiccation tolerance	BP	4.34E-02	1.68E-02	1	0	465	27197
OVER	GO:0097525	spliceosomal snRNP complex	CT	4.34E-02	1.68E-02	1	0	465	27197
OVER	GO:0097526	spliceosomal tri-snRNP complex	CT	4.34E-02	1.68E-02	1	0	465	27197

**Over/Under***	**GO ID**	**GO Name**	**GO Category$**	**FDR**	**P-value**	**Test**	**Ref**	**Non Annot Test**	**Non Annot Reference**
OVER	GO:0097659	nucleic acid-templated transcription	BP	6.32E-06	7.62E-07	49	1321	417	25876
OVER	GO:0097708	intracellular vesicle	CT	9.07E-05	1.38E-05	6	26	460	27171
OVER	GO:0097747	RNA polymerase activity	MF	3.45E-14	1.08E-15	17	51	449	27146
OVER	GO:0098542	defense response to other organism	BP	2.15E-11	9.64E-13	8	3	458	27194
OVER	GO:0098586	cellular response to virus	BP	4.34E-02	1.68E-02	1	0	465	27197
OVER	GO:0098588	bounding membrane of organelle	CT	5.28E-07	5.12E-08	11	64	455	27133
OVER	GO:0098655	cation transmembrane transport	BP	1.55E-07	1.40E-08	13	87	453	27110
OVER	GO:0098660	inorganic ion transmembrane transport	BP	3.12E-07	2.93E-08	12	76	454	27121
OVER	GO:0098662	inorganic cation transmembrane transport	BP	3.12E-07	2.93E-08	12	76	454	27121
OVER	GO:0098727	maintenance of cell number	BP	4.34E-02	1.68E-02	1	0	465	27197
OVER	GO:0098771	inorganic ion homeostasis	BP	2.28E-02	5.62E-03	2	5	464	27192
OVER	GO:0098791	Golgi subcompartment	CT	1.35E-03	2.50E-04	3	5	463	27192
OVER	GO:0098796	membrane protein complex	CT	1.09E-14	3.29E-16	26	167	440	27030
OVER	GO:0098805	whole membrane	CT	3.03E-08	2.53E-09	11	46	455	27151
OVER	GO:0098807	chloroplast thylakoid membrane protein complex	CT	4.34E-02	1.68E-02	1	0	465	27197
OVER	GO:0098813	nuclear chromosome segregation	BP	1.18E-02	2.74E-03	2	3	464	27194
OVER	GO:0099023	tethering complex	CT	4.34E-02	1.38E-02	3	27	463	27170
OVER	GO:0099131	ATP hydrolysis coupled ion transmembrane transport	BP	8.57E-03	1.97E-03	4	28	462	27169
OVER	GO:0099132	ATP hydrolysis coupled cation transmembrane transport	BP	8.57E-03	1.97E-03	4	28	462	27169
OVER	GO:0099402	plant organ development	BP	4.37E-22	7.45E-24	13	0	453	27197
OVER	GO:0099568	cytoplasmic region	CT	4.34E-02	1.25E-02	3	26	463	27171
OVER	GO:0101005	ubiquitinyl hydrolase activity	MF	4.34E-02	1.68E-02	1	0	465	27197
OVER	GO:1900140	regulation of seedling development	BP	4.34E-02	1.68E-02	1	0	465	27197
OVER	GO:1901135	carbohydrate derivative metabolic process	BP	6.46E-09	4.37E-10	26	328	440	26869
OVER	GO:1901137	carbohydrate derivative biosynthetic process	BP	5.42E-08	4.64E-09	18	173	448	27024
OVER	GO:1901265	nucleoside phosphate binding	MF	4.34E-13	1.56E-14	110	2973	356	24224
OVER	GO:1901293	nucleoside phosphate biosynthetic process	BP	2.73E-05	3.51E-06	10	82	456	27115
OVER	GO:1901360	organic cyclic compound metabolic process	BP	4.37E-22	7.31E-24	111	2242	355	24955
OVER	GO:1901362	organic cyclic compound biosynthetic process	BP	1.26E-09	7.41E-11	67	1628	399	25569
OVER	GO:1901363	heterocyclic compound binding	MF	6.27E-30	7.92E-32	208	5503	258	21694
OVER	GO:1901419	regulation of response to alcohol	BP	4.34E-02	1.68E-02	1	0	465	27197
OVER	GO:1901421	positive regulation of response to alcohol	BP	4.34E-02	1.68E-02	1	0	465	27197
OVER	GO:1901564	organonitrogen compound metabolic process	BP	1.85E-08	1.46E-09	47	1005	419	26192
OVER	GO:1901566	organonitrogen compound biosynthetic process	BP	2.49E-06	2.84E-07	35	760	431	26437
OVER	GO:1901575	organic substance catabolic process	BP	9.22E-03	2.12E-03	11	222	455	26975
OVER	GO:1901576	organic substance biosynthetic process	BP	4.10E-14	1.31E-15	103	2571	363	24626
OVER	GO:1901605	alpha-amino acid metabolic process	BP	2.14E-04	3.46E-05	10	109	456	27088

Over/Under*	GO ID	GO Name	GO Category$	FDR	P-value	Test	Ref	Non Annot Test	Non Annot Reference
OVER	GO:1901607	alpha-amino acid biosynthetic process	BP	4.03E-05	5.92E-06	9	68	457	27129
OVER	GO:1901615	organic hydroxy compound metabolic process	BP	1.18E-02	2.75E-03	4	31	462	27166
OVER	GO:1901617	organic hydroxy compound biosynthetic process	BP	2.28E-02	5.62E-03	2	5	464	27192
OVER	GO:1901657	glycosyl compound metabolic process	BP	4.54E-07	4.36E-08	16	157	450	27040
OVER	GO:1901659	glycosyl compound biosynthetic process	BP	3.23E-05	4.24E-06	9	65	457	27132
OVER	GO:1901678	iron coordination entity transport	BP	4.34E-02	1.68E-02	1	0	465	27197
OVER	GO:1901698	response to nitrogen compound	BP	1.35E-03	2.83E-04	2	0	464	27197
OVER	GO:1901699	cellular response to nitrogen compound	BP	4.34E-02	1.68E-02	1	0	465	27197
OVER	GO:1901700	response to oxygen-containing compound	BP	3.09E-17	7.77E-19	12	4	454	27193
OVER	GO:1901701	cellular response to oxygen-containing compound	BP	4.00E-10	2.21E-11	6	0	460	27197
OVER	GO:1901981	phosphatidylinositol phosphate binding	MF	4.34E-02	1.68E-02	1	0	465	27197
OVER	GO:1902578	single-organism localization	BP	1.25E-13	4.24E-15	29	245	437	26952
OVER	GO:1902580	single-organism cellular localization	BP	7.22E-07	7.15E-08	7	16	459	27181
OVER	GO:1902582	single-organism intracellular transport	BP	7.22E-07	7.15E-08	7	16	459	27181
OVER	GO:1902589	single-organism organelle organization	BP	9.39E-08	8.28E-09	9	28	457	27169
OVER	GO:1902593	single-organism nuclear import	BP	2.93E-02	7.41E-03	2	6	464	27191
OVER	GO:1902600	hydrogen ion transmembrane transport	BP	2.79E-06	3.24E-07	9	46	457	27151
OVER	GO:1902644	tertiary alcohol metabolic process	BP	4.34E-02	1.68E-02	1	0	465	27197
OVER	GO:1902645	tertiary alcohol biosynthetic process	BP	4.34E-02	1.68E-02	1	0	465	27197
OVER	GO:1902680	positive regulation of RNA biosynthetic process	BP	6.20E-05	9.37E-06	4	5	462	27192
OVER	GO:1902905	positive regulation of supramolecular fiber organization	BP	4.34E-02	1.68E-02	1	0	465	27197
OVER	GO:1902936	phosphatidylinositol bisphosphate binding	MF	4.34E-02	1.68E-02	1	0	465	27197
OVER	GO:1903409	reactive oxygen species biosynthetic process	BP	4.00E-10	2.21E-11	6	0	460	27197
OVER	GO:1903506	regulation of nucleic acid-templated transcription	BP	4.34E-02	1.26E-02	33	1216	433	25981
OVER	GO:1903508	positive regulation of nucleic acid-templated transcription	BP	6.20E-05	9.37E-06	4	5	462	27192
OVER	GO:1905392	plant organ morphogenesis	BP	3.27E-05	4.75E-06	3	0	463	27197
OVER	GO:1905393	plant organ formation	BP	1.35E-03	2.83E-04	2	0	464	27197
OVER	GO:1990077**	primosome complex*	CT	4.34E-02	1.68E-02	1	0	465	27197
OVER	GO:1990234	transferase complex	CT	2.46E-02	6.07E-03	7	121	459	27076
OVER	GO:1990391	DNA repair complex	CT	1.35E-03	2.83E-04	2	0	464	27197
OVER	GO:1990904	ribonucleoprotein complex	CT	2.93E-02	7.28E-03	14	369	452	26828
OVER	GO:2000026	regulation of multicellular organismal development	BP	4.00E-10	2.21E-11	6	0	460	27197
OVER	GO:2000112	regulation of cellular macromolecule biosynthetic process	BP	4.97E-03	1.10E-03	37	1228	429	25969
OVER	GO:2000113	negative regulation of cellular macromolecule biosynthetic process	BP	1.70E-02	4.06E-03	2	4	464	27193

Over/Under*	GO ID	GO Name	GO Category$	FDR	P-value	Test	Ref	Non Annot Test	Non Annot Reference
OVER	GO:2000241	regulation of reproductive process	BP	7.35E-07	7.95E-08	4	0	462	27197
OVER	GO:2000243	positive regulation of reproductive process	BP	4.34E-02	1.68E-02	1	0	465	27197
OVER	GO:2000280	regulation of root development	BP	4.34E-02	1.68E-02	1	0	465	27197
OVER	GO:2000377	regulation of reactive oxygen species metabolic process	BP	4.34E-02	1.68E-02	1	0	465	27197
OVER	GO:2001141	regulation of RNA biosynthetic process	BP	4.34E-02	1.26E-02	33	1216	433	25981

* over/under-represented genes: Over means enriched DEGs for a particular category; under means fewer DEGs for a particular category, both of them associated with p-value.
** Most specific enriched term determined using the function "Reduce to most specific terms" implemented in BLAST2GO.
Test: Number of genes from the set of new loci annotated with the GO ID
Ref: Number of genes in the reference subset (all genes) annotated with the GO ID
Non Annot Test: Number of genes in the tested subset (new loci) not annotated with the GO ID
Non Annot Reference: Number of genes in the reference subset (all genes) not annotated with the GO ID
obs. The "all gene" set represents the junction of the 27197 gene in the *P.vulgaris* genome plus the 466 new loci full annotated.

Over/ Under *	Comparison	Comparison Description	Representative set**	GO ID	GO Name	GO	FDR	Test	Ref	Non Annot Test	Non Annot Reference
**DEGs**											
OVER	**Comp 2**	Pérola all samples of leaves vs BAT 477 all samples of leaves	All genes	GO:0009607	response to biotic stimulus	BP	1.23E-05	7	57	94	27505
OVER	**Comp 2**	Pérola all samples of leaves vs BAT 477 all samples of leaves	All genes	GO:0006952	defense response	BP	9.06E-04	8	184	93	27378
OVER	**Comp 2**	Pérola all samples of leaves vs BAT 477 all samples of leaves	All genes	GO:0008219	cell death	BP	3.35E-02	8	366	93	27196
OVER	**Comp 2**	Pérola all samples of leaves vs BAT 477 all samples of leaves	All genes	GO:0012501	programmed cell death	BP	3.35E-02	8	346	93	27216
OVER	**Comp 2**	Pérola all samples of leaves vs BAT 477 all samples of leaves	All genes	GO:0043167	ion binding	MF	2.50E-02	34	4543	67	23019
OVER	**Comp 2**	Pérola all samples of leaves vs BAT 477 all samples of leaves	All genes	GO:0005488	binding	MF	3.35E-02	52	8872	49	18690
OVER	**Comp 2**	Pérola all samples of leaves vs BAT 477 all samples of leaves	All genes	GO:0043531	ADP binding	MF	3.35E-02	8	352	93	27210
OVER	**Comp 3.1**	BAT 477, root in T0 vs BAT 477, root in T75	All genes	GO:0008152	metabolic process	BP	3.65E-05	90	8231	92	19250
OVER	**Comp 3.1**	BAT 477, root in T0 vs BAT 477, root in T75	All genes	GO:0055114	oxidation-reduction process	BP	3.65E-05	30	1448	152	26033
OVER	**Comp 3.1**	BAT 477, root in T0 vs BAT 477, root in T75	All genes	GO:0044710	single-organism metabolic process	BP	1.29E-04	40	2548	142	24933
OVER	**Comp 3.1**	BAT 477, root in T0 vs BAT 477, root in T75	All genes	GO:0006032	chitin catabolic process	BP	3.60E-04	4	10	178	27471
OVER	**Comp 3.1**	BAT 477, root in T0 vs BAT 477, root in T75	All genes	GO:0046348	amino sugar catabolic process	BP	3.60E-04	4	10	178	27471
OVER	**Comp 3.1**	BAT 477, root in T0 vs BAT 477, root in T75	All genes	GO:1901072	glucosamine-containing compound catabolic process	BP	3.60E-04	4	10	178	27471
OVER	**Comp 3.1**	BAT 477, root in T0 vs BAT 477, root in T75	All genes	GO:1901071	glucosamine-containing compound metabolic process	BP	3.60E-04	4	10	178	27471
OVER	**Comp 3.1**	BAT 477, root in T0 vs BAT 477, root in T75	All genes	GO:0006040	amino sugar metabolic process	BP	3.60E-04	4	10	178	27471
OVER	**Comp 3.1**	BAT 477, root in T0 vs BAT 477, root in T75	All genes	GO:0006030	chitin metabolic process	BP	3.60E-04	4	10	178	27471
OVER	**Comp 3.1**	BAT 477, root in T0 vs BAT 477, root in T75	All genes	GO:0006026	aminoglycan catabolic process	BP	3.60E-04	4	10	178	27471

OVER	**Comp 3.1**	BAT 477, root in T0 vs BAT 477, root in T75	All genes	GO:0006022	aminoglycan metabolic process	BP	4.60E-04	4	11	178	27470
OVER	**Comp 3.1**	BAT 477, root in T0 vs BAT 477, root in T75	All genes	GO:0071554	cell wall organization or biogenesis	BP	7.97E-04	8	132	174	27349
OVER	**Comp 3.1**	BAT 477, root in T0 vs BAT 477, root in T75	All genes	GO:1901136	carbohydrate derivative catabolic process	BP	1.75E-03	4	17	178	27464
OVER	**Comp 3.1**	BAT 477, root in T0 vs BAT 477, root in T75	All genes	GO:0044699	single-organism process	BP	2.37E-03	48	3864	134	23617
OVER	**Comp 3.1**	BAT 477, root in T0 vs BAT 477, root in T75	All genes	GO:1901565	organonitrogen compound catabolic process	BP	1.02E-02	4	29	178	27452
OVER	**Comp 3.1**	BAT 477, root in T0 vs BAT 477, root in T75	All genes	GO:0016998	cell wall macromolecule catabolic process	BP	2.33E-02	4	38	178	27443
OVER	**Comp 3.1**	BAT 477, root in T0 vs BAT 477, root in T75	All genes	GO:0010468	regulation of gene expression	BP	2.60E-02	20	1240	162	26241
OVER	**Comp 3.1**	BAT 477, root in T0 vs BAT 477, root in T75	All genes	GO:0051252	regulation of RNA metabolic process	BP	2.60E-02	20	1229	162	26252
OVER	**Comp 3.1**	BAT 477, root in T0 vs BAT 477, root in T75	All genes	GO:0006355	regulation of transcription, DNA-templated	BP	2.60E-02	20	1229	162	26252
OVER	**Comp 3.1**	BAT 477, root in T0 vs BAT 477, root in T75	All genes	GO:0019219	regulation of nucleobase-containing compound metabolic process	BP	2.60E-02	20	1239	162	26242
OVER	**Comp 3.1**	BAT 477, root in T0 vs BAT 477, root in T75	All genes	GO:1903506	regulation of nucleic acid-templated transcription	BP	2.60E-02	20	1229	162	26252
OVER	**Comp 3.1**	BAT 477, root in T0 vs BAT 477, root in T75	All genes	GO:0031326	regulation of cellular biosynthetic process	BP	2.60E-02	20	1245	162	26236
OVER	**Comp 3.1**	BAT 477, root in T0 vs BAT 477, root in T75	All genes	GO:2001141	regulation of RNA biosynthetic process	BP	2.60E-02	20	1229	162	26252
OVER	**Comp 3.1**	BAT 477, root in T0 vs BAT 477, root in T75	All genes	GO:2000112	regulation of cellular macromolecule biosynthetic process	BP	2.60E-02	20	1245	162	26236

OVER	**Comp 3.1**	BAT 477, root in T0 vs BAT 477, root in T75	All genes	GO:0009889	regulation of biosynthetic process	BP	2.60E-02	20	1245	162	26236
OVER	**Comp 3.1**	BAT 477, root in T0 vs BAT 477, root in T75	All genes	GO:0051171	regulation of nitrogen compound metabolic process	BP	2.60E-02	20	1247	162	26234
OVER	**Comp 3.1**	BAT 477, root in T0 vs BAT 477, root in T75	All genes	GO:0044036	cell wall macromolecule metabolic process	BP	3.06E-02	4	46	178	27435
OVER	**Comp 3.1**	BAT 477, root in T0 vs BAT 477, root in T75	All genes	GO:0031323	regulation of cellular metabolic process	BP	4.02E-02	20	1260	162	26221
OVER	**Comp 3.1**	BAT 477, root in T0 vs BAT 477, root in T75	All genes	GO:0080090	regulation of primary metabolic process	BP	4.02E-02	20	1258	162	26223
OVER	**Comp 3.1**	BAT 477, root in T0 vs BAT 477, root in T75	All genes	GO:0060255	regulation of macromolecule metabolic process	BP	4.02E-02	20	1260	162	26221
OVER	**Comp 3.1**	BAT 477, root in T0 vs BAT 477, root in T75	All genes	GO:0019222	regulation of metabolic process	BP	4.02E-02	20	1264	162	26217
OVER	**Comp 3.1**	BAT 477, root in T0 vs BAT 477, root in T75	All genes	GO:0016705	oxidoreductase activity, acting on paired donors, with incorporation or reduction of molecular oxygen	MF	1.17E-07	20	442	162	27039
OVER	**Comp 3.1**	BAT 477, root in T0 vs BAT 477, root in T75	All genes	GO:0051213	dioxygenase activity	MF	1.07E-05	12	181	170	27300
OVER	**Comp 3.1**	BAT 477, root in T0 vs BAT 477, root in T75	All genes	GO:0016706	oxidoreductase activity, acting on paired donors, with incorporation or reduction of molecular oxygen, 2-oxoglutarate as one donor, and incorporat	MF	3.65E-05	10	136	172	27345

					ion of one atom each of oxygen into both donors						
OVER	**Comp 3.1**	BAT 477, root in T0 vs BAT 477, root in T75	All genes	GO:0016491	oxidoreductase activity	MF	1.05E-04	31	1654	151	25827
OVER	**Comp 3.1**	BAT 477, root in T0 vs BAT 477, root in T75	All genes	GO:0003824	catalytic activity	MF	1.32E-04	84	7714	98	19767
OVER	**Comp 3.1**	BAT 477, root in T0 vs BAT 477, root in T75	All genes	GO:0004568	chitinase activity	MF	3.60E-04	4	10	178	27471
OVER	**Comp 3.1**	BAT 477, root in T0 vs BAT 477, root in T75	All genes	GO:0020037	heme binding	MF	1.34E-02	11	397	171	27084
OVER	**Comp 3.1**	BAT 477, root in T0 vs BAT 477, root in T75	All genes	GO:0046906	tetrapyrrole binding	MF	1.73E-02	11	411	171	27070
OVER	**Comp 3.1**	BAT 477, root in T0 vs BAT 477, root in T75	All genes	GO:0005506	iron ion binding	MF	4.02E-02	9	332	173	27149
OVER	**Comp 3.3**	BAT 477, root in T0 vs BAT 477, root in T150	All genes	GO:0055114	oxidation-reduction process	BP	2.06E-07	71	1407	524	25661
OVER	**Comp 3.3**	BAT 477, root in T0 vs BAT 477, root in T150	All genes	GO:0008152	metabolic process	BP	1.29E-04	238	8083	357	18985
OVER	**Comp 3.3**	BAT 477, root in T0 vs BAT 477, root in T150	All genes	GO:0044710	single-organism metabolic process	BP	2.07E-04	94	2494	501	24574
OVER	**Comp 3.3**	BAT 477, root in T0 vs BAT 477, root in T150	All genes	GO:0071554	cell wall organization or biogenesis	BP	7.07E-04	14	126	581	26942
OVER	**Comp 3.3**	BAT 477, root in T0 vs BAT 477, root in T150	All genes	GO:0055085	transmembrane transport	BP	2.61E-03	35	690	560	26378
OVER	**Comp 3.3**	BAT 477, root in T0 vs BAT 477, root in T150	All genes	GO:0010556	regulation of macromolecule biosynthetic process	BP	5.08E-03	50	1215	545	25853
OVER	**Comp 3.3**	BAT 477, root in T0 vs BAT 477, root in T150	All genes	GO:0006355	regulation of transcription, DNA-templated	BP	5.08E-03	50	1199	545	25869
OVER	**Comp 3.3**	BAT 477, root in T0 vs BAT 477, root in T150	All genes	GO:2000112	regulation of cellular macromolecule biosynthetic process	BP	5.08E-03	50	1215	545	25853
OVER	**Comp 3.3**	BAT 477, root in T0 vs BAT 477, root in T150	All genes	GO:0009889	regulation of biosynthetic process	BP	5.08E-03	50	1215	545	25853
OVER	**Comp 3.3**	BAT 477, root in T0 vs BAT 477, root in T150	All genes	GO:0010468	regulation of gene expression	BP	5.08E-03	50	1210	545	25858
OVER	**Comp 3.3**	BAT 477, root in T0 vs BAT 477, root in T150	All genes	GO:0051252	regulation of RNA metabolic process	BP	5.08E-03	50	1199	545	25869
OVER	**Comp 3.3**	BAT 477, root in T0 vs BAT 477, root in T150	All genes	GO:0019219	regulation of nucleobase-	BP	5.08E-03	50	1209	545	25859

					containing compound metabolic process						
OVER	**Comp 3.3**	BAT 477, root in T0 vs BAT 477, root in T150	All genes	GO:0031326	regulation of cellular biosynthetic process	BP	5.08E-03	50	1215	545	25853
OVER	**Comp 3.3**	BAT 477, root in T0 vs BAT 477, root in T150	All genes	GO:2001141	regulation of RNA biosynthetic process	BP	5.08E-03	50	1199	545	25869
OVER	**Comp 3.3**	BAT 477, root in T0 vs BAT 477, root in T150	All genes	GO:0051171	regulation of nitrogen compound metabolic process	BP	5.08E-03	50	1217	545	25851
OVER	**Comp 3.3**	BAT 477, root in T0 vs BAT 477, root in T150	All genes	GO:0060255	regulation of macromolecule metabolic process	BP	7.14E-03	50	1230	545	25838
OVER	**Comp 3.3**	BAT 477, root in T0 vs BAT 477, root in T150	All genes	GO:0019222	regulation of metabolic process	BP	7.14E-03	50	1234	545	25834
OVER	**Comp 3.3**	BAT 477, root in T0 vs BAT 477, root in T150	All genes	GO:0031323	regulation of cellular metabolic process	BP	7.14E-03	50	1230	545	25838
OVER	**Comp 3.3**	BAT 477, root in T0 vs BAT 477, root in T150	All genes	GO:0080090	regulation of primary metabolic process	BP	7.14E-03	50	1228	545	25840
OVER	**Comp 3.3**	BAT 477, root in T0 vs BAT 477, root in T150	All genes	GO:0044699	single-organism process	BP	8.01E-03	119	3793	476	23275
OVER	**Comp 3.3**	BAT 477, root in T0 vs BAT 477, root in T150	All genes	GO:0071555	cell wall organization	BP	8.01E-03	9	75	586	26993
OVER	**Comp 3.3**	BAT 477, root in T0 vs BAT 477, root in T150	All genes	GO:0045229	external encapsulating structure organization	BP	8.01E-03	9	75	586	26993
OVER	**Comp 3.3**	BAT 477, root in T0 vs BAT 477, root in T150	All genes	GO:0046348	amino sugar catabolic process	BP	1.19E-02	4	10	591	27058
OVER	**Comp 3.3**	BAT 477, root in T0 vs BAT 477, root in T150	All genes	GO:0006351	transcription, DNA-templated	BP	1.19E-02	51	1319	544	25749
OVER	**Comp 3.3**	BAT 477, root in T0 vs BAT 477, root in T150	All genes	GO:1901072	glucosamine-containing compound catabolic process	BP	1.19E-02	4	10	591	27058
OVER	**Comp 3.3**	BAT 477, root in T0 vs BAT 477, root in T150	All genes	GO:1901071	glucosamine-containing compound	BP	1.19E-02	4	10	591	27058

					metabolic process						
OVER	**Comp 3.3**	BAT 477, root in T0 vs BAT 477, root in T150	All genes	GO:0006026	aminoglycan catabolic process	BP	1.19E-02	4	10	591	27058
OVER	**Comp 3.3**	BAT 477, root in T0 vs BAT 477, root in T150	All genes	GO:0006032	chitin catabolic process	BP	1.19E-02	4	10	591	27058
OVER	**Comp 3.3**	BAT 477, root in T0 vs BAT 477, root in T150	All genes	GO:0097659	nucleic acid-templated transcription	BP	1.19E-02	51	1319	544	25749
OVER	**Comp 3.3**	BAT 477, root in T0 vs BAT 477, root in T150	All genes	GO:0032774	RNA biosynthetic process	BP	1.19E-02	51	1322	544	25746
OVER	**Comp 3.3**	BAT 477, root in T0 vs BAT 477, root in T150	All genes	GO:0006040	amino sugar metabolic process	BP	1.19E-02	4	10	591	27058
OVER	**Comp 3.3**	BAT 477, root in T0 vs BAT 477, root in T150	All genes	GO:0006030	chitin metabolic process	BP	1.19E-02	4	10	591	27058
OVER	**Comp 3.3**	BAT 477, root in T0 vs BAT 477, root in T150	All genes	GO:0042545	cell wall modification	BP	1.19E-02	8	65	587	27003
UNDER	**Comp 3.3**	BAT 477, root in T0 vs BAT 477, root in T150	All genes	GO:0043603	cellular amide metabolic process	BP	1.30E-02	1	533	594	26535
OVER	**Comp 3.3**	BAT 477, root in T0 vs BAT 477, root in T150	All genes	GO:0006022	aminoglycan metabolic process	BP	1.38E-02	4	11	591	27057
UNDER	**Comp 3.3**	BAT 477, root in T0 vs BAT 477, root in T150	All genes	GO:1901566	organonitrogen compound biosynthetic process	BP	1.49E-02	4	791	591	26277
UNDER	**Comp 3.3**	BAT 477, root in T0 vs BAT 477, root in T150	All genes	GO:0006518	peptide metabolic process	BP	1.66E-02	1	505	594	26563
UNDER	**Comp 3.3**	BAT 477, root in T0 vs BAT 477, root in T150	All genes	GO:0043604	amide biosynthetic process	BP	1.68E-02	1	516	594	26552
UNDER	**Comp 3.3**	BAT 477, root in T0 vs BAT 477, root in T150	All genes	GO:0043043	peptide biosynthetic process	BP	2.30E-02	1	496	594	26572
OVER	**Comp 3.3**	BAT 477, root in T0 vs BAT 477, root in T150	All genes	GO:0009607	response to biotic stimulus	BP	2.30E-02	7	57	588	27011
UNDER	**Comp 3.3**	BAT 477, root in T0 vs BAT 477, root in T150	All genes	GO:0006412	translation	BP	2.30E-02	1	488	594	26580
OVER	**Comp 3.3**	BAT 477, root in T0 vs BAT 477, root in T150	All genes	GO:0034654	nucleobase-containing compound biosynthetic process	BP	2.87E-02	52	1425	543	25643
OVER	**Comp 3.3**	BAT 477, root in T0 vs BAT 477, root in T150	All genes	GO:0008283	cell proliferation	BP	3.51E-02	3	6	592	27062
OVER	**Comp 3.3**	BAT 477, root in T0 vs BAT 477, root in T150	All genes	GO:1901136	carbohydrate derivative	BP	4.34E-02	4	17	591	27051

					catabolic process						
UNDER	**Comp 3.3**	BAT 477, root in T0 vs BAT 477, root in T150	All genes	GO:0005622	intracellular	CC	3.59E-04	25	2624	570	24444
UNDER	**Comp 3.3**	BAT 477, root in T0 vs BAT 477, root in T150	All genes	GO:0005737	cytoplasm	CC	1.11E-03	5	1103	590	25965
UNDER	**Comp 3.3**	BAT 477, root in T0 vs BAT 477, root in T150	All genes	GO:0005623	cell	CC	1.07E-02	34	2794	561	24274
UNDER	**Comp 3.3**	BAT 477, root in T0 vs BAT 477, root in T150	All genes	GO:0044464	cell part	CC	1.07E-02	34	2794	561	24274
UNDER	**Comp 3.3**	BAT 477, root in T0 vs BAT 477, root in T150	All genes	GO:0044444	cytoplasmic part	CC	1.18E-02	4	828	591	26240
UNDER	**Comp 3.3**	BAT 477, root in T0 vs BAT 477, root in T150	All genes	GO:0044424	intracellular part	CC	1.25E-02	24	2153	571	24915
UNDER	**Comp 3.3**	BAT 477, root in T0 vs BAT 477, root in T150	All genes	GO:0043229	intracellular organelle	CC	1.34E-02	16	1653	579	25415
UNDER	**Comp 3.3**	BAT 477, root in T0 vs BAT 477, root in T150	All genes	GO:0043226	organelle	CC	1.34E-02	16	1653	579	25415
UNDER	**Comp 3.3**	BAT 477, root in T0 vs BAT 477, root in T150	All genes	GO:0044446	intracellular organelle part	CC	3.03E-02	1	471	594	26597
UNDER	**Comp 3.3**	BAT 477, root in T0 vs BAT 477, root in T150	All genes	GO:0044422	organelle part	CC	3.03E-02	1	471	594	26597
OVER	**Comp 3.3**	BAT 477, root in T0 vs BAT 477, root in T150	All genes	GO:0016705	oxidoreductase activity, acting on paired donors, with incorporation or reduction of molecular oxygen	MF	7.26E-11	41	421	554	26647
OVER	**Comp 3.3**	BAT 477, root in T0 vs BAT 477, root in T150	All genes	GO:0016706	oxidoreductase activity, acting on paired donors, with incorporation or reduction of molecular oxygen, 2-oxoglutarate as one donor, and incorporation of one atom each of oxygen into both donors	MF	7.61E-09	21	125	574	26943
OVER	**Comp 3.3**	BAT 477, root in T0 vs BAT 477, root in T150	All genes	GO:0051213	dioxygenase activity	MF	7.61E-09	24	169	571	26899
OVER	**Comp 3.3**	BAT 477, root in T0 vs BAT 477, root in T150	All genes	GO:0016491	oxidoreductase activity	MF	3.79E-06	74	1611	521	25457

OVER	**Comp 3.3**	BAT 477, root in T0 vs BAT 477, root in T150	All genes	GO:0003824	catalytic activity	MF	1.38E-04	225	7573	370	19495
OVER	**Comp 3.3**	BAT 477, root in T0 vs BAT 477, root in T150	All genes	GO:0020037	heme binding	MF	1.00E-03	25	383	570	26685
OVER	**Comp 3.3**	BAT 477, root in T0 vs BAT 477, root in T150	All genes	GO:0046906	tetrapyrrole binding	MF	1.53E-03	25	397	570	26671
OVER	**Comp 3.3**	BAT 477, root in T0 vs BAT 477, root in T150	All genes	GO:0005506	iron ion binding	MF	4.10E-03	21	320	574	26748
OVER	**Comp 3.3**	BAT 477, root in T0 vs BAT 477, root in T150	All genes	GO:0005215	transporter activity	MF	7.14E-03	37	822	558	26246
OVER	**Comp 3.3**	BAT 477, root in T0 vs BAT 477, root in T150	All genes	GO:0015297	antiporter activity	MF	1.07E-02	10	98	585	26970
OVER	**Comp 3.3**	BAT 477, root in T0 vs BAT 477, root in T150	All genes	GO:0004568	chitinase activity	MF	1.19E-02	4	10	591	27058
OVER	**Comp 3.3**	BAT 477, root in T0 vs BAT 477, root in T150	All genes	GO:0005102	receptor binding	MF	1.21E-02	3	3	592	27065
OVER	**Comp 3.3**	BAT 477, root in T0 vs BAT 477, root in T150	All genes	GO:0008083	growth factor activity	MF	1.21E-02	3	3	592	27065
OVER	**Comp 3.3**	BAT 477, root in T0 vs BAT 477, root in T150	All genes	GO:0009055	electron carrier activity	MF	1.25E-02	25	497	570	26571
OVER	**Comp 3.3**	BAT 477, root in T0 vs BAT 477, root in T150	All genes	GO:0004659	prenyltransferase activity	MF	1.65E-02	5	23	590	27045
OVER	**Comp 3.3**	BAT 477, root in T0 vs BAT 477, root in T150	All genes	GO:0052689	carboxylic ester hydrolase activity	MF	2.76E-02	12	168	583	26900
OVER	**Comp 3.3**	BAT 477, root in T0 vs BAT 477, root in T150	All genes	GO:0015291	secondary active transmembrane transporter activity	MF	2.76E-02	10	121	585	26947
OVER	**Comp 3.3**	BAT 477, root in T0 vs BAT 477, root in T150	All genes	GO:0022857	transmembrane transporter activity	MF	3.34E-02	27	602	568	26466
OVER	**Comp 4.1**	Pérola, root in T0 vs Pérola, root in T75	All genes	GO:0016705	oxidoreductase activity, acting on paired donors, with incorporation or reduction of molecular oxygen	MF	3.49E-04	13	449	106	27095
OVER	**Comp 4.3**	Pérola, root in T0 vs Pérola, root in T150	All genes	GO:0055114	oxidation-reduction process	BP	4.10E-08	53	1425	308	25877
OVER	**Comp 4.3**	Pérola, root in T0 vs Pérola, root in T150	All genes	GO:0008152	metabolic process	BP	5.46E-07	164	8157	197	19145
OVER	**Comp 4.3**	Pérola, root in T0 vs Pérola, root in T150	All genes	GO:0044710	single-organism metabolic process	BP	5.27E-05	66	2522	295	24780
OVER	**Comp 4.3**	Pérola, root in T0 vs Pérola, root in T150	All genes	GO:0044699	single-organism process	BP	2.44E-04	86	3826	275	23476

OVER	**Comp 4.3**	Pérola, root in T0 vs Pérola, root in T150	All genes	GO:0009607	response to biotic stimulus	BP	4.49E-02	6	58	355	27244
OVER	**Comp 4.3**	Pérola, root in T0 vs Pérola, root in T150	All genes	GO:0016705	oxidoreductase activity, acting on paired donors, with incorporation or reduction of molecular oxygen	MF	3.91E-10	31	431	330	26871
OVER	**Comp 4.3**	Pérola, root in T0 vs Pérola, root in T150	All genes	GO:0016491	oxidoreductase activity	MF	5.02E-07	55	1630	306	25672
OVER	**Comp 4.3**	Pérola, root in T0 vs Pérola, root in T150	All genes	GO:0020037	heme binding	MF	1.74E-05	22	386	339	26916
OVER	**Comp 4.3**	Pérola, root in T0 vs Pérola, root in T150	All genes	GO:0046906	tetrapyrrole binding	MF	2.63E-05	22	400	339	26902
OVER	**Comp 4.3**	Pérola, root in T0 vs Pérola, root in T150	All genes	GO:0003824	catalytic activity	MF	4.47E-05	149	7649	212	19653
OVER	**Comp 4.3**	Pérola, root in T0 vs Pérola, root in T150	All genes	GO:0005506	iron ion binding	MF	5.27E-05	19	322	342	26980
OVER	**Comp 4.3**	Pérola, root in T0 vs Pérola, root in T150	All genes	GO:0051213	dioxygenase activity	MF	9.27E-05	14	179	347	27123
OVER	**Comp 4.3**	Pérola, root in T0 vs Pérola, root in T150	All genes	GO:0016706	oxidoreductase activity, acting on paired donors, with incorporation or reduction of molecular oxygen, 2-oxoglutarate as one donor, and incorporation of one atom each of oxygen into both donors	MF	1.59E-04	12	134	349	27168
OVER	**Comp 4.3**	Pérola, root in T0 vs Pérola, root in T150	All genes	GO:0009055	electron carrier activity	MF	4.46E-04	22	500	339	26802
OVER	**Comp 5.3**	BAT 477, leaf in T0 vs BAT 477, leaf in T150	All genes	GO:0009790	embryo development	BP	1.07E-02	4	9	244	27406
OVER	**Comp 5.3**	BAT 477, leaf in T0 vs BAT 477, leaf in T150	All genes	GO:0048856	anatomical structure development	BP	1.84E-02	6	70	242	27345
OVER	**Comp 5.3**	BAT 477, leaf in T0 vs BAT 477, leaf in T150	All genes	GO:0032502	developmental process	BP	1.84E-02	6	71	242	27344

OVER	**Comp 5.3**	BAT 477, leaf in T0 vs BAT 477, leaf in T150	All genes	GO:0044767	single-organism developmental process	BP	1.84E-02	6	70	242	27345
OVER	**Comp 5.3**	BAT 477, leaf in T0 vs BAT 477, leaf in T150	All genes	GO:0007275	multicellular organism development	BP	1.84E-02	5	44	243	27371
OVER	**Comp 5.3**	BAT 477, leaf in T0 vs BAT 477, leaf in T150	All genes	GO:0044707	single-multicellular organism process	BP	2.58E-02	5	49	243	27366
UNDER	**Comp 5.3**	BAT 477, leaf in T0 vs BAT 477, leaf in T150	All genes	GO:0032549	ribonucleoside binding	MF	1.07E-02	7	2755	241	24660
UNDER	**Comp 5.3**	BAT 477, leaf in T0 vs BAT 477, leaf in T150	All genes	GO:0032550	purine ribonucleoside binding	MF	1.07E-02	7	2746	241	24669
UNDER	**Comp 5.3**	BAT 477, leaf in T0 vs BAT 477, leaf in T150	All genes	GO:0001883	purine nucleoside binding	MF	1.07E-02	7	2746	241	24669
UNDER	**Comp 5.3**	BAT 477, leaf in T0 vs BAT 477, leaf in T150	All genes	GO:0035639	purine ribonucleoside triphosphate binding	MF	1.07E-02	7	2730	241	24685
UNDER	**Comp 5.3**	BAT 477, leaf in T0 vs BAT 477, leaf in T150	All genes	GO:0001882	nucleoside binding	MF	1.07E-02	7	2756	241	24659
UNDER	**Comp 5.3**	BAT 477, leaf in T0 vs BAT 477, leaf in T150	All genes	GO:0032555	purine ribonucleotide binding	MF	1.07E-02	7	2746	241	24669
UNDER	**Comp 5.3**	BAT 477, leaf in T0 vs BAT 477, leaf in T150	All genes	GO:0017076	purine nucleotide binding	MF	1.07E-02	7	2757	241	24658
UNDER	**Comp 5.3**	BAT 477, leaf in T0 vs BAT 477, leaf in T150	All genes	GO:0097367	carbohydrate derivative binding	MF	1.84E-02	8	2800	240	24615
UNDER	**Comp 5.3**	BAT 477, leaf in T0 vs BAT 477, leaf in T150	All genes	GO:0005524	ATP binding	MF	2.58E-02	7	2509	241	24906
UNDER	**Comp 5.3**	BAT 477, leaf in T0 vs BAT 477, leaf in T150	All genes	GO:0030554	adenyl nucleotide binding	MF	2.58E-02	7	2529	241	24886
UNDER	**Comp 5.3**	BAT 477, leaf in T0 vs BAT 477, leaf in T150	All genes	GO:0032559	adenyl ribonucleotide binding	MF	2.58E-02	7	2525	241	24890
OVER	**Comp 5.3**	BAT 477, leaf in T0 vs BAT 477, leaf in T150	All genes	GO:0004097	catechol oxidase activity	MF	4.20E-02	2	1	246	27414
OVER	**Comp 11**	Pérola, leaf in T75 vs BAT 477, leaf in T75	All genes	GO:0009607	response to biotic stimulus	BP	4.27E-06	7	57	80	27519
OVER	**Comp 11**	Pérola, leaf in T75 vs BAT 477, leaf in T75	All genes	GO:0006952	defense response	BP	2.85E-04	8	184	79	27392
OVER	**Comp 11**	Pérola, leaf in T75 vs BAT 477, leaf in T75	All genes	GO:0006468	protein phosphorylation	BP	1.85E-03	16	1286	71	26290
OVER	**Comp 11**	Pérola, leaf in T75 vs BAT 477, leaf in T75	All genes	GO:0016310	phosphorylation	BP	3.08E-03	16	1389	71	26187

OVER	**Comp 11**	Pérola, leaf in T75 vs BAT 477, leaf in T75	All genes	GO:0036211	protein modification process	BP	5.55E-03	16	1557	71	26019
OVER	**Comp 11**	Pérola, leaf in T75 vs BAT 477, leaf in T75	All genes	GO:0043412	macromolecule modification	BP	6.18E-03	16	1611	71	25965
OVER	**Comp 11**	Pérola, leaf in T75 vs BAT 477, leaf in T75	All genes	GO:0006796	phosphate-containing compound metabolic process	BP	6.80E-03	16	1638	71	25938
OVER	**Comp 11**	Pérola, leaf in T75 vs BAT 477, leaf in T75	All genes	GO:0006793	phosphorus metabolic process	BP	6.80E-03	16	1641	71	25935
OVER	**Comp 11**	Pérola, leaf in T75 vs BAT 477, leaf in T75	All genes	GO:0006950	response to stress	BP	7.75E-03	9	548	78	27028
OVER	**Comp 11**	Pérola, leaf in T75 vs BAT 477, leaf in T75	All genes	GO:0016740	transferase activity	MF	6.63E-04	26	2893	61	24683
OVER	**Comp 11**	Pérola, leaf in T75 vs BAT 477, leaf in T75	All genes	GO:0004672	protein kinase activity	MF	1.85E-03	16	1290	71	26286
OVER	**Comp 11**	Pérola, leaf in T75 vs BAT 477, leaf in T75	All genes	GO:0016773	phosphotransferase activity, alcohol group as acceptor	MF	3.08E-03	16	1420	71	26156
OVER	**Comp 11**	Pérola, leaf in T75 vs BAT 477, leaf in T75	All genes	GO:0016301	kinase activity	MF	3.08E-03	16	1420	71	26156
OVER	**Comp 11**	Pérola, leaf in T75 vs BAT 477, leaf in T75	All genes	GO:0030554	adenyl nucleotide binding	MF	3.08E-03	22	2514	65	25062
OVER	**Comp 11**	Pérola, leaf in T75 vs BAT 477, leaf in T75	All genes	GO:0032559	adenyl ribonucleotide binding	MF	3.08E-03	22	2510	65	25066
OVER	**Comp 11**	Pérola, leaf in T75 vs BAT 477, leaf in T75	All genes	GO:0005488	binding	MF	4.40E-03	48	8876	39	18700
OVER	**Comp 11**	Pérola, leaf in T75 vs BAT 477, leaf in T75	All genes	GO:0032549	ribonucleoside binding	MF	5.55E-03	22	2740	65	24836
OVER	**Comp 11**	Pérola, leaf in T75 vs BAT 477, leaf in T75	All genes	GO:0001883	purine nucleoside binding	MF	5.55E-03	22	2731	65	24845
OVER	**Comp 11**	Pérola, leaf in T75 vs BAT 477, leaf in T75	All genes	GO:0032550	purine ribonucleoside binding	MF	5.55E-03	22	2731	65	24845
OVER	**Comp 11**	Pérola, leaf in T75 vs BAT 477, leaf in T75	All genes	GO:0001882	nucleoside binding	MF	5.55E-03	22	2741	65	24835
OVER	**Comp 11**	Pérola, leaf in T75 vs BAT 477, leaf in T75	All genes	GO:0032555	purine ribonucleotide binding	MF	5.55E-03	22	2731	65	24845
OVER	**Comp 11**	Pérola, leaf in T75 vs BAT 477, leaf in T75	All genes	GO:0005524	ATP binding	MF	5.55E-03	21	2495	66	25081
OVER	**Comp 11**	Pérola, leaf in T75 vs BAT 477, leaf in T75	All genes	GO:0017076	purine nucleotide binding	MF	5.55E-03	22	2742	65	24834
OVER	**Comp 11**	Pérola, leaf in T75 vs BAT 477, leaf in T75	All genes	GO:0032553	ribonucleotide binding	MF	5.94E-03	22	2763	65	24813

OVER	**Comp 11**	Pérola, leaf in T75 vs BAT 477, leaf in T75	All genes	GO:0097367	carbohydrate derivative binding	MF	6.18E-03	22	2786	65	24790
OVER	**Comp 11**	Pérola, leaf in T75 vs BAT 477, leaf in T75	All genes	GO:0016772	transferase activity, transferring phosphorus-containing groups	MF	6.80E-03	16	1640	71	25936
OVER	**Comp 11**	Pérola, leaf in T75 vs BAT 477, leaf in T75	All genes	GO:0043167	ion binding	MF	7.36E-03	30	4547	57	23029
OVER	**Comp 11**	Pérola, leaf in T75 vs BAT 477, leaf in T75	All genes	GO:0035639	purine ribonucleoside triphosphate binding	MF	1.03E-02	21	2716	66	24860
OVER	**Comp 11**	Pérola, leaf in T75 vs BAT 477, leaf in T75	All genes	GO:0043168	anion binding	MF	1.19E-02	23	3123	64	24453
OVER	**Comp 11**	Pérola, leaf in T75 vs BAT 477, leaf in T75	All genes	GO:1901265	nucleoside phosphate binding	MF	2.12E-02	22	3061	65	24515
OVER	**Comp 11**	Pérola, leaf in T75 vs BAT 477, leaf in T75	All genes	GO:0000166	nucleotide binding	MF	2.12E-02	22	3061	65	24515
OVER	**Comp 11**	Pérola, leaf in T75 vs BAT 477, leaf in T75	All genes	GO:0036094	small molecule binding	MF	2.43E-02	22	3109	65	24467
OVER	**Comp 11**	Pérola, leaf in T75 vs BAT 477, leaf in T75	All genes	GO:0003824	catalytic activity	MF	4.49E-02	40	7758	47	19818
OVER	**Comp 12**	Pérola, leaf in T150 vs BAT 477, leaf in T150	All genes	GO:0008219	cell death	BP	4.63E-03	7	367	43	27246
OVER	**Comp 12**	Pérola, leaf in T150 vs BAT 477, leaf in T150	All genes	GO:0009607	response to biotic stimulus	BP	4.63E-03	4	60	46	27553
OVER	**Comp 12**	Pérola, leaf in T150 vs BAT 477, leaf in T150	All genes	GO:0012501	programmed cell death	BP	4.63E-03	7	347	43	27266
OVER	**Comp 12**	Pérola, leaf in T150 vs BAT 477, leaf in T150	All genes	GO:0006952	defense response	BP	1.68E-02	5	187	45	27426
OVER	**Comp 12**	Pérola, leaf in T150 vs BAT 477, leaf in T150	All genes	GO:0006915	apoptotic process	BP	2.18E-02	6	344	44	27269
OVER	**Comp 12**	Pérola, leaf in T150 vs BAT 477, leaf in T150	All genes	GO:0043531	ADP binding	MF	4.63E-03	7	353	43	27260
OVER	**Comp 12**	Pérola, leaf in T150 vs BAT 477, leaf in T150	All genes	GO:0016838	carbon-oxygen lyase activity, acting on phosphates	MF	2.47E-02	3	39	47	27574
OVER	**Comp 12**	Pérola, leaf in T150 vs BAT 477, leaf in T150	All genes	GO:0043167	ion binding	MF	2.47E-02	20	4557	30	23056
OVER	**Comp 12**	Pérola, leaf in T150 vs BAT 477, leaf in T150	All genes	GO:0010333	terpene synthase activity	MF	2.47E-02	3	37	47	27576
OVER	**Comp 3.1**	BAT 477, root in T0 vs BAT 477, root in T75	Up-regulated DEGs	GO:0055114	oxidation-reduction process	BP	1.19E-04	28	1450	139	26046
OVER	**Comp 3.1**	BAT 477, root in T0 vs BAT 477, root in T75	Up-regulated DEGs	GO:0044710	single-organism metabolic process	BP	2.92E-04	37	2551	130	24945

OVER	**Comp 3.1**	BAT 477, root in T0 vs BAT 477, root in T75	Up-regulated DEGs	GO:0006032	chitin catabolic process	BP	2.92E-04	4	10	163	27486
OVER	**Comp 3.1**	BAT 477, root in T0 vs BAT 477, root in T75	Up-regulated DEGs	GO:1901071	glucosamine-containing compound metabolic process	BP	2.92E-04	4	10	163	27486
OVER	**Comp 3.1**	BAT 477, root in T0 vs BAT 477, root in T75	Up-regulated DEGs	GO:0008152	metabolic process	BP	2.92E-04	81	8240	86	19256
OVER	**Comp 3.1**	BAT 477, root in T0 vs BAT 477, root in T75	Up-regulated DEGs	GO:0046348	amino sugar catabolic process	BP	2.92E-04	4	10	163	27486
OVER	**Comp 3.1**	BAT 477, root in T0 vs BAT 477, root in T75	Up-regulated DEGs	GO:0006040	amino sugar metabolic process	BP	2.92E-04	4	10	163	27486
OVER	**Comp 3.1**	BAT 477, root in T0 vs BAT 477, root in T75	Up-regulated DEGs	GO:0006030	chitin metabolic process	BP	2.92E-04	4	10	163	27486
OVER	**Comp 3.1**	BAT 477, root in T0 vs BAT 477, root in T75	Up-regulated DEGs	GO:1901072	glucosamine-containing compound catabolic process	BP	2.92E-04	4	10	163	27486
OVER	**Comp 3.1**	BAT 477, root in T0 vs BAT 477, root in T75	Up-regulated DEGs	GO:0006026	aminoglycan catabolic process	BP	2.92E-04	4	10	163	27486
OVER	**Comp 3.1**	BAT 477, root in T0 vs BAT 477, root in T75	Up-regulated DEGs	GO:0006022	aminoglycan metabolic process	BP	3.70E-04	4	11	163	27485
OVER	**Comp 3.1**	BAT 477, root in T0 vs BAT 477, root in T75	Up-regulated DEGs	GO:1901136	carbohydrate derivative catabolic process	BP	1.31E-03	4	17	163	27479
OVER	**Comp 3.1**	BAT 477, root in T0 vs BAT 477, root in T75	Up-regulated DEGs	GO:0044699	single-organism process	BP	2.84E-03	45	3867	122	23629
OVER	**Comp 3.1**	BAT 477, root in T0 vs BAT 477, root in T75	Up-regulated DEGs	GO:0071554	cell wall organization or biogenesis	BP	3.95E-03	7	133	160	27363
OVER	**Comp 3.1**	BAT 477, root in T0 vs BAT 477, root in T75	Up-regulated DEGs	GO:1901565	organonitrogen compound catabolic process	BP	6.95E-03	4	29	163	27467
OVER	**Comp 3.1**	BAT 477, root in T0 vs BAT 477, root in T75	Up-regulated DEGs	GO:0016998	cell wall macromolecule catabolic process	BP	1.67E-02	4	38	163	27458
OVER	**Comp 3.1**	BAT 477, root in T0 vs BAT 477, root in T75	Up-regulated DEGs	GO:0044036	cell wall macromolecule metabolic process	BP	3.09E-02	4	46	163	27450

OVER	**Comp 3.1**	BAT 477, root in T0 vs BAT 477, root in T75	Up-regulated DEGs	GO:0016705	oxidoreductase activity, acting on paired donors, with incorporation or reduction of molecular oxygen	MF	1.55E-06	18	444	149	27052
OVER	**Comp 3.1**	BAT 477, root in T0 vs BAT 477, root in T75	Up-regulated DEGs	GO:0051213	dioxygenase activity	MF	2.55E-04	10	183	157	27313
OVER	**Comp 3.1**	BAT 477, root in T0 vs BAT 477, root in T75	Up-regulated DEGs	GO:0016491	oxidoreductase activity	MF	2.55E-04	29	1656	138	25840
OVER	**Comp 3.1**	BAT 477, root in T0 vs BAT 477, root in T75	Up-regulated DEGs	GO:0004568	chitinase activity	MF	2.92E-04	4	10	163	27486
OVER	**Comp 3.1**	BAT 477, root in T0 vs BAT 477, root in T75	Up-regulated DEGs	GO:0003824	catalytic activity	MF	4.94E-04	76	7722	91	19774
OVER	**Comp 3.1**	BAT 477, root in T0 vs BAT 477, root in T75	Up-regulated DEGs	GO:0016706	oxidoreductase activity, acting on paired donors, with incorporation or reduction of molecular oxygen, 2-oxoglutarate as one donor, and incorporation of one atom each of oxygen into both donors	MF	6.10E-04	8	138	159	27358
OVER	**Comp 3.1**	BAT 477, root in T0 vs BAT 477, root in T75	Up-regulated DEGs	GO:0020037	heme binding	MF	6.46E-03	11	397	156	27099
OVER	**Comp 3.1**	BAT 477, root in T0 vs BAT 477, root in T75	Up-regulated DEGs	GO:0046906	tetrapyrrole binding	MF	7.99E-03	11	411	156	27085
OVER	**Comp 3.1**	BAT 477, root in T0 vs BAT 477, root in T75	Up-regulated DEGs	GO:0005506	iron ion binding	MF	3.09E-02	9	332	158	27164
OVER	**Comp 3.1**	BAT 477, root in T0 vs BAT 477, root in T75	Up-regulated DEGs	GO:0009055	electron carrier activity	MF	4.32E-02	11	511	156	26985
OVER	**Comp 3.3**	BAT 477, root in T0 vs BAT 477, root in T150	Up-regulated DEGs	GO:0055114	oxidation-reduction process	BP	8.55E-04	53	1425	431	25754
OVER	**Comp 3.3**	BAT 477, root in T0 vs BAT 477, root in T150	Up-regulated DEGs	GO:0010556	regulation of macromol	BP	1.57E-03	45	1220	439	25959

					ecule biosynthetic process						
OVER	**Comp 3.3**	BAT 477, root in T0 vs BAT 477, root in T150	Up-regulated DEGs	GO:0008152	metabolic process	BP	1.57E-03	194	8127	290	19052
OVER	**Comp 3.3**	BAT 477, root in T0 vs BAT 477, root in T150	Up-regulated DEGs	GO:0031323	regulation of cellular metabolic process	BP	1.57E-03	45	1235	439	25944
OVER	**Comp 3.3**	BAT 477, root in T0 vs BAT 477, root in T150	Up-regulated DEGs	GO:0010468	regulation of gene expression	BP	1.57E-03	45	1215	439	25964
OVER	**Comp 3.3**	BAT 477, root in T0 vs BAT 477, root in T150	Up-regulated DEGs	GO:0051252	regulation of RNA metabolic process	BP	1.57E-03	45	1204	439	25975
OVER	**Comp 3.3**	BAT 477, root in T0 vs BAT 477, root in T150	Up-regulated DEGs	GO:0080090	regulation of primary metabolic process	BP	1.57E-03	45	1233	439	25946
OVER	**Comp 3.3**	BAT 477, root in T0 vs BAT 477, root in T150	Up-regulated DEGs	GO:0006355	regulation of transcription, DNA-templated	BP	1.57E-03	45	1204	439	25975
OVER	**Comp 3.3**	BAT 477, root in T0 vs BAT 477, root in T150	Up-regulated DEGs	GO:1903506	regulation of nucleic acid-templated transcription	BP	1.57E-03	45	1204	439	25975
OVER	**Comp 3.3**	BAT 477, root in T0 vs BAT 477, root in T150	Up-regulated DEGs	GO:0019219	regulation of nucleobase-containing compound metabolic process	BP	1.57E-03	45	1214	439	25965
OVER	**Comp 3.3**	BAT 477, root in T0 vs BAT 477, root in T150	Up-regulated DEGs	GO:0031326	regulation of cellular biosynthetic process	BP	1.57E-03	45	1220	439	25959
OVER	**Comp 3.3**	BAT 477, root in T0 vs BAT 477, root in T150	Up-regulated DEGs	GO:0060255	regulation of macromolecule metabolic process	BP	1.57E-03	45	1235	439	25944
OVER	**Comp 3.3**	BAT 477, root in T0 vs BAT 477, root in T150	Up-regulated DEGs	GO:2001141	regulation of RNA biosynthetic process	BP	1.57E-03	45	1204	439	25975
OVER	**Comp 3.3**	BAT 477, root in T0 vs BAT 477, root in T150	Up-regulated DEGs	GO:2000112	regulation of cellular macromolecule biosynthetic process	BP	1.57E-03	45	1220	439	25959
OVER	**Comp 3.3**	BAT 477, root in T0 vs BAT 477, root in T150	Up-regulated DEGs	GO:0009889	regulation of biosynthetic process	BP	1.57E-03	45	1220	439	25959

OVER	**Comp 3.3**	BAT 477, root in T0 vs BAT 477, root in T150	Up-regulated DEGs	GO:0051171	regulation of nitrogen compound metabolic process	BP	1.57E-03	45	1222	439	25957
OVER	**Comp 3.3**	BAT 477, root in T0 vs BAT 477, root in T150	Up-regulated DEGs	GO:0019222	regulation of metabolic process	BP	1.58E-03	45	1239	439	25940
OVER	**Comp 3.3**	BAT 477, root in T0 vs BAT 477, root in T150	Up-regulated DEGs	GO:0097659	nucleic acid-templated transcription	BP	3.89E-03	46	1324	438	25855
OVER	**Comp 3.3**	BAT 477, root in T0 vs BAT 477, root in T150	Up-regulated DEGs	GO:0006351	transcription, DNA-templated	BP	3.89E-03	46	1324	438	25855
OVER	**Comp 3.3**	BAT 477, root in T0 vs BAT 477, root in T150	Up-regulated DEGs	GO:0032774	RNA biosynthetic process	BP	3.89E-03	46	1327	438	25852
OVER	**Comp 3.3**	BAT 477, root in T0 vs BAT 477, root in T150	Up-regulated DEGs	GO:0044710	single-organism metabolic process	BP	6.41E-03	73	2515	411	24664
OVER	**Comp 3.3**	BAT 477, root in T0 vs BAT 477, root in T150	Up-regulated DEGs	GO:0006032	chitin catabolic process	BP	7.09E-03	4	10	480	27169
OVER	**Comp 3.3**	BAT 477, root in T0 vs BAT 477, root in T150	Up-regulated DEGs	GO:0046348	amino sugar catabolic process	BP	7.09E-03	4	10	480	27169
OVER	**Comp 3.3**	BAT 477, root in T0 vs BAT 477, root in T150	Up-regulated DEGs	GO:1901072	glucosamine-containing compound catabolic process	BP	7.09E-03	4	10	480	27169
OVER	**Comp 3.3**	BAT 477, root in T0 vs BAT 477, root in T150	Up-regulated DEGs	GO:1901071	glucosamine-containing compound metabolic process	BP	7.09E-03	4	10	480	27169
OVER	**Comp 3.3**	BAT 477, root in T0 vs BAT 477, root in T150	Up-regulated DEGs	GO:0006040	amino sugar metabolic process	BP	7.09E-03	4	10	480	27169
OVER	**Comp 3.3**	BAT 477, root in T0 vs BAT 477, root in T150	Up-regulated DEGs	GO:0006030	chitin metabolic process	BP	7.09E-03	4	10	480	27169
OVER	**Comp 3.3**	BAT 477, root in T0 vs BAT 477, root in T150	Up-regulated DEGs	GO:0006026	aminoglycan catabolic process	BP	7.09E-03	4	10	480	27169
OVER	**Comp 3.3**	BAT 477, root in T0 vs BAT 477, root in T150	Up-regulated DEGs	GO:0034654	nucleobase-containing compound biosynthetic process	BP	7.44E-03	47	1430	437	25749
OVER	**Comp 3.3**	BAT 477, root in T0 vs BAT 477, root in T150	Up-regulated DEGs	GO:0006022	aminoglycan metabolic process	BP	8.63E-03	4	11	480	27168

OVER	**Comp 3.3**	BAT 477, root in T0 vs BAT 477, root in T150	Up-regulated DEGs	GO:0009607	response to biotic stimulus	BP	9.59E-03	7	57	477	27122
OVER	**Comp 3.3**	BAT 477, root in T0 vs BAT 477, root in T150	Up-regulated DEGs	GO:0019438	aromatic compound biosynthetic process	BP	2.39E-02	47	1521	437	25658
OVER	**Comp 3.3**	BAT 477, root in T0 vs BAT 477, root in T150	Up-regulated DEGs	GO:0008283	cell proliferation	BP	2.94E-02	3	6	481	27173
OVER	**Comp 3.3**	BAT 477, root in T0 vs BAT 477, root in T150	Up-regulated DEGs	GO:1901136	carbohydrate derivative catabolic process	BP	3.05E-02	4	17	480	27162
OVER	**Comp 3.3**	BAT 477, root in T0 vs BAT 477, root in T150	Up-regulated DEGs	GO:0018130	heterocycle biosynthetic process	BP	3.27E-02	47	1534	437	25645
OVER	**Comp 3.3**	BAT 477, root in T0 vs BAT 477, root in T150	Up-regulated DEGs	GO:0071554	cell wall organization or biogenesis	BP	4.94E-02	9	131	475	27048
OVER	**Comp 3.3**	BAT 477, root in T0 vs BAT 477, root in T150	Up-regulated DEGs	GO:0016070	RNA metabolic process	BP	4.94E-02	47	1575	437	25604
UNDER	**Comp 3.3**	BAT 477, root in T0 vs BAT 477, root in T150	Up-regulated DEGs	GO:1901566	organonitrogen compound biosynthetic process	BP	4.94E-02	3	792	481	26387
UNDER	**Comp 3.3**	BAT 477, root in T0 vs BAT 477, root in T150	Up-regulated DEGs	GO:0005622	intracellular	CC	3.37E-03	21	2628	463	24551
UNDER	**Comp 3.3**	BAT 477, root in T0 vs BAT 477, root in T150	Up-regulated DEGs	GO:0005737	cytoplasm	CC	4.30E-03	4	1104	480	26075
UNDER	**Comp 3.3**	BAT 477, root in T0 vs BAT 477, root in T150	Up-regulated DEGs	GO:0005623	cell	CC	7.09E-03	25	2803	459	24376
UNDER	**Comp 3.3**	BAT 477, root in T0 vs BAT 477, root in T150	Up-regulated DEGs	GO:0044464	cell part	CC	7.09E-03	25	2803	459	24376
OVER	**Comp 3.3**	BAT 477, root in T0 vs BAT 477, root in T150	Up-regulated DEGs	GO:0016705	oxidoreductase activity, acting on paired donors, with incorporation or reduction of molecular oxygen	MF	1.31E-05	29	433	455	26746
OVER	**Comp 3.3**	BAT 477, root in T0 vs BAT 477, root in T150	Up-regulated DEGs	GO:0051213	dioxygenase activity	MF	9.85E-05	17	176	467	27003
OVER	**Comp 3.3**	BAT 477, root in T0 vs BAT 477, root in T150	Up-regulated DEGs	GO:0016706	oxidoreductase activity, acting on paired donors, with	MF	3.42E-04	14	132	470	27047

					incorporation or reduction of molecular oxygen, 2-oxoglutarate as one donor, and incorporation of one atom each of oxygen into both donors						
OVER	**Comp 3.3**	BAT 477, root in T0 vs BAT 477, root in T150	Up-regulated DEGs	GO:0016491	oxidoreductase activity	MF	1.57E-03	55	1630	429	25549
OVER	**Comp 3.3**	BAT 477, root in T0 vs BAT 477, root in T150	Up-regulated DEGs	GO:0003824	catalytic activity	MF	6.50E-03	177	7621	307	19558
OVER	**Comp 3.3**	BAT 477, root in T0 vs BAT 477, root in T150	Up-regulated DEGs	GO:0004568	chitinase activity	MF	7.09E-03	4	10	480	27169
OVER	**Comp 3.3**	BAT 477, root in T0 vs BAT 477, root in T150	Up-regulated DEGs	GO:0005102	receptor binding	MF	8.35E-03	3	3	481	27176
OVER	**Comp 3.3**	BAT 477, root in T0 vs BAT 477, root in T150	Up-regulated DEGs	GO:0008083	growth factor activity	MF	8.35E-03	3	3	481	27176
OVER	**Comp 3.3**	BAT 477, root in T0 vs BAT 477, root in T150	Up-regulated DEGs	GO:0015297	antiporter activity	MF	9.39E-03	9	99	475	27080
OVER	**Comp 3.3**	BAT 477, root in T0 vs BAT 477, root in T150	Up-regulated DEGs	GO:0020037	heme binding	MF	2.53E-02	18	390	466	26789
OVER	**Comp 3.3**	BAT 477, root in T0 vs BAT 477, root in T150	Up-regulated DEGs	GO:0046906	tetrapyrrole binding	MF	3.40E-02	18	404	466	26775
OVER	**Comp 3.3**	BAT 477, root in T0 vs BAT 477, root in T150	Up-regulated DEGs	GO:0015291	secondary active transmembrane transporter activity	MF	3.40E-02	9	122	475	27057
OVER	**Comp 3.3**	BAT 477, root in T0 vs BAT 477, root in T150	Up-regulated DEGs	GO:0003700	transcription factor activity, sequence-specific DNA binding	MF	4.72E-02	24	634	460	26545
OVER	**Comp 3.3**	BAT 477, root in T0 vs BAT 477, root in T150	Up-regulated DEGs	GO:0001071	nucleic acid binding transcription factor activity	MF	4.72E-02	24	634	460	26545
OVER	**Comp 4.1**	Pérola, root in T0 vs Pérola, root in T75	Up-regulated DEGs	GO:0016705	oxidoreductase activity, acting on paired donors, with	MF	3.91E-02	10	452	96	27105

					incorporation or reduction of molecular oxygen						
OVER	**Comp 4.3**	Pérola, root in T0 vs Pérola, root in T150	Up-regulated DEGs	GO:0055114	oxidation-reduction process	BP	2.72E-07	46	1432	257	25928
OVER	**Comp 4.3**	Pérola, root in T0 vs Pérola, root in T150	Up-regulated DEGs	GO:0008152	metabolic process	BP	1.25E-06	141	8180	162	19180
OVER	**Comp 4.3**	Pérola, root in T0 vs Pérola, root in T150	Up-regulated DEGs	GO:0044710	single-organism metabolic process	BP	2.82E-04	56	2532	247	24828
OVER	**Comp 4.3**	Pérola, root in T0 vs Pérola, root in T150	Up-regulated DEGs	GO:0044699	single-organism process	BP	1.78E-03	72	3840	231	23520
OVER	**Comp 4.3**	Pérola, root in T0 vs Pérola, root in T150	Up-regulated DEGs	GO:0071554	cell wall organization or biogenesis	BP	3.86E-02	8	132	295	27228
OVER	**Comp 4.3**	Pérola, root in T0 vs Pérola, root in T150	Up-regulated DEGs	GO:0006032	chitin catabolic process	BP	4.47E-02	3	11	300	27349
OVER	**Comp 4.3**	Pérola, root in T0 vs Pérola, root in T150	Up-regulated DEGs	GO:0010556	regulation of macromolecule biosynthetic process	BP	4.47E-02	28	1237	275	26123
OVER	**Comp 4.3**	Pérola, root in T0 vs Pérola, root in T150	Up-regulated DEGs	GO:0010468	regulation of gene expression	BP	4.47E-02	28	1232	275	26128
OVER	**Comp 4.3**	Pérola, root in T0 vs Pérola, root in T150	Up-regulated DEGs	GO:0046348	amino sugar catabolic process	BP	4.47E-02	3	11	300	27349
OVER	**Comp 4.3**	Pérola, root in T0 vs Pérola, root in T150	Up-regulated DEGs	GO:0051252	regulation of RNA metabolic process	BP	4.47E-02	28	1221	275	26139
OVER	**Comp 4.3**	Pérola, root in T0 vs Pérola, root in T150	Up-regulated DEGs	GO:0006355	regulation of transcription, DNA-templated	BP	4.47E-02	28	1221	275	26139
OVER	**Comp 4.3**	Pérola, root in T0 vs Pérola, root in T150	Up-regulated DEGs	GO:1901072	glucosamine-containing compound catabolic process	BP	4.47E-02	3	11	300	27349
OVER	**Comp 4.3**	Pérola, root in T0 vs Pérola, root in T150	Up-regulated DEGs	GO:0019219	regulation of nucleobase-containing compound metabolic process	BP	4.47E-02	28	1231	275	26129
OVER	**Comp 4.3**	Pérola, root in T0 vs Pérola, root in T150	Up-regulated DEGs	GO:1903506	regulation of nucleic acid-templated	BP	4.47E-02	28	1221	275	26139

					transcription						
OVER	**Comp 4.3**	Pérola, root in T0 vs Pérola, root in T150	Up-regulated DEGs	GO:0031326	regulation of cellular biosynthetic process	BP	4.47E-02	28	1237	275	26123
OVER	**Comp 4.3**	Pérola, root in T0 vs Pérola, root in T150	Up-regulated DEGs	GO:1901071	glucosamine-containing compound metabolic process	BP	4.47E-02	3	11	300	27349
OVER	**Comp 4.3**	Pérola, root in T0 vs Pérola, root in T150	Up-regulated DEGs	GO:0006040	amino sugar metabolic process	BP	4.47E-02	3	11	300	27349
OVER	**Comp 4.3**	Pérola, root in T0 vs Pérola, root in T150	Up-regulated DEGs	GO:2001141	regulation of RNA biosynthetic process	BP	4.47E-02	28	1221	275	26139
OVER	**Comp 4.3**	Pérola, root in T0 vs Pérola, root in T150	Up-regulated DEGs	GO:2000112	regulation of cellular macromolecule biosynthetic process	BP	4.47E-02	28	1237	275	26123
OVER	**Comp 4.3**	Pérola, root in T0 vs Pérola, root in T150	Up-regulated DEGs	GO:0009889	regulation of biosynthetic process	BP	4.47E-02	28	1237	275	26123
OVER	**Comp 4.3**	Pérola, root in T0 vs Pérola, root in T150	Up-regulated DEGs	GO:0006030	chitin metabolic process	BP	4.47E-02	3	11	300	27349
OVER	**Comp 4.3**	Pérola, root in T0 vs Pérola, root in T150	Up-regulated DEGs	GO:0006026	aminoglycan catabolic process	BP	4.47E-02	3	11	300	27349
OVER	**Comp 4.3**	Pérola, root in T0 vs Pérola, root in T150	Up-regulated DEGs	GO:0051171	regulation of nitrogen compound metabolic process	BP	4.47E-02	28	1239	275	26121
OVER	**Comp 4.3**	Pérola, root in T0 vs Pérola, root in T150	Up-regulated DEGs	GO:0031323	regulation of cellular metabolic process	BP	4.52E-02	28	1252	275	26108
OVER	**Comp 4.3**	Pérola, root in T0 vs Pérola, root in T150	Up-regulated DEGs	GO:0080090	regulation of primary metabolic process	BP	4.52E-02	28	1250	275	26110
OVER	**Comp 4.3**	Pérola, root in T0 vs Pérola, root in T150	Up-regulated DEGs	GO:0060255	regulation of macromolecule metabolic process	BP	4.52E-02	28	1252	275	26108
OVER	**Comp 4.3**	Pérola, root in T0 vs Pérola, root in T150	Up-regulated DEGs	GO:0019222	regulation of metabolic process	BP	4.52E-02	28	1256	275	26104
OVER	**Comp 4.3**	Pérola, root in T0 vs Pérola, root in T150	Up-regulated DEGs	GO:0006022	aminoglycan metabolic process	BP	4.60E-02	3	12	300	27348

OVER	**Comp 4.3**	Pérola, root in T0 vs Pérola, root in T150	Up-regulated DEGs	GO:0016705	oxidoreductase activity, acting on paired donors, with incorporation or reduction of molecular oxygen	MF	3.96E-08	26	436	277	26924
OVER	**Comp 4.3**	Pérola, root in T0 vs Pérola, root in T150	Up-regulated DEGs	GO:0016491	oxidoreductase activity	MF	1.25E-06	48	1637	255	25723
OVER	**Comp 4.3**	Pérola, root in T0 vs Pérola, root in T150	Up-regulated DEGs	GO:0003824	catalytic activity	MF	1.05E-04	128	7670	175	19690
OVER	**Comp 4.3**	Pérola, root in T0 vs Pérola, root in T150	Up-regulated DEGs	GO:0051213	dioxygenase activity	MF	1.33E-04	13	180	290	27180
OVER	**Comp 4.3**	Pérola, root in T0 vs Pérola, root in T150	Up-regulated DEGs	GO:0016706	oxidoreductase activity, acting on paired donors, with incorporation or reduction of molecular oxygen, 2-oxoglutarate as one donor, and incorporation of one atom each of oxygen into both donors	MF	2.82E-04	11	135	292	27225
OVER	**Comp 4.3**	Pérola, root in T0 vs Pérola, root in T150	Up-regulated DEGs	GO:0020037	heme binding	MF	2.82E-04	18	390	285	26970
OVER	**Comp 4.3**	Pérola, root in T0 vs Pérola, root in T150	Up-regulated DEGs	GO:0046906	tetrapyrrole binding	MF	3.81E-04	18	404	285	26956
OVER	**Comp 4.3**	Pérola, root in T0 vs Pérola, root in T150	Up-regulated DEGs	GO:0005506	iron ion binding	MF	1.78E-03	15	326	288	27034
OVER	**Comp 4.3**	Pérola, root in T0 vs Pérola, root in T150	Up-regulated DEGs	GO:0009055	electron carrier activity	MF	1.86E-02	17	505	286	26855
OVER	**Comp 4.3**	Pérola, root in T0 vs Pérola, root in T150	Up-regulated DEGs	GO:0004568	chitinase activity	MF	4.47E-02	3	11	300	27349
OVER	**Comp 4.3**	Pérola, root in T0 vs Pérola, root in T150	Up-regulated DEGs	GO:0030170	pyridoxal phosphate binding	MF	4.52E-02	6	85	297	27275
OVER	**Comp 5.3**	BAT 477, leaf in T0 vs BAT 477, leaf in T150	Up-regulated DEGs	GO:0009790	embryo development	BP	6.78E-03	4	9	202	27448

OVER	**Comp 2**	Pérola all samples of leaves vs BAT 477 all samples of leaves	Down-regulated DEGs	GO:0009607	response to biotic stimulus	BP	8.59E-04	5	59	54	27545
OVER	**Comp 2**	Pérola all samples of leaves vs BAT 477 all samples of leaves	Down-regulated DEGs	GO:0006952	defense response	BP	2.29E-03	6	186	53	27418
OVER	**Comp 2**	Pérola all samples of leaves vs BAT 477 all samples of leaves	Down-regulated DEGs	GO:0016310	phosphorylation	BP	2.36E-03	13	1392	46	26212
OVER	**Comp 2**	Pérola all samples of leaves vs BAT 477 all samples of leaves	Down-regulated DEGs	GO:0006468	protein phosphorylation	BP	2.43E-03	12	1290	47	26314
OVER	**Comp 2**	Pérola all samples of leaves vs BAT 477 all samples of leaves	Down-regulated DEGs	GO:0006796	phosphate-containing compound metabolic process	BP	4.70E-03	13	1641	46	25963
OVER	**Comp 2**	Pérola all samples of leaves vs BAT 477 all samples of leaves	Down-regulated DEGs	GO:0006793	phosphorus metabolic process	BP	4.70E-03	13	1644	46	25960
OVER	**Comp 2**	Pérola all samples of leaves vs BAT 477 all samples of leaves	Down-regulated DEGs	GO:0006464	cellular protein modification process	BP	1.03E-02	12	1561	47	26043
OVER	**Comp 2**	Pérola all samples of leaves vs BAT 477 all samples of leaves	Down-regulated DEGs	GO:0036211	protein modification process	BP	1.03E-02	12	1561	47	26043
OVER	**Comp 2**	Pérola all samples of leaves vs BAT 477 all samples of leaves	Down-regulated DEGs	GO:0043412	macromolecule modification	BP	1.33E-02	12	1615	47	25989
OVER	**Comp 2**	Pérola all samples of leaves vs BAT 477 all samples of leaves	Down-regulated DEGs	GO:0043167	ion binding	MF	2.29E-03	25	4552	34	23052
OVER	**Comp 2**	Pérola all samples of leaves vs BAT 477 all samples of leaves	Down-regulated DEGs	GO:0030554	adenyl nucleotide binding	MF	2.29E-03	18	2518	41	25086
OVER	**Comp 2**	Pérola all samples of leaves vs BAT 477 all samples of leaves	Down-regulated DEGs	GO:0032559	adenyl ribonucleotide binding	MF	2.29E-03	18	2514	41	25090
OVER	**Comp 2**	Pérola all samples of leaves vs BAT 477 all samples of leaves	Down-regulated DEGs	GO:0032549	ribonucleoside binding	MF	2.36E-03	18	2744	41	24860
OVER	**Comp 2**	Pérola all samples of leaves vs BAT 477 all samples of leaves	Down-regulated DEGs	GO:0001883	purine nucleoside binding	MF	2.36E-03	18	2735	41	24869
OVER	**Comp 2**	Pérola all samples of leaves vs BAT 477 all samples of leaves	Down-regulated DEGs	GO:1901265	nucleoside phosphate binding	MF	2.36E-03	19	3064	40	24540
OVER	**Comp 2**	Pérola all samples of leaves vs BAT 477 all samples of leaves	Down-regulated DEGs	GO:0000166	nucleotide binding	MF	2.36E-03	19	3064	40	24540
OVER	**Comp 2**	Pérola all samples of leaves vs BAT 477 all samples of leaves	Down-regulated DEGs	GO:0016772	transferase activity, transferring phosphorus-containing groups	MF	2.36E-03	14	1642	45	25962
OVER	**Comp 2**	Pérola all samples of leaves vs BAT 477 all samples of leaves	Down-regulated DEGs	GO:0032550	purine ribonucleo	MF	2.36E-03	18	2735	41	24869

					side binding						
OVER	**Comp 2**	Pérola all samples of leaves vs BAT 477 all samples of leaves	Down-regulated DEGs	GO:0001882	nucleoside binding	MF	2.36E-03	18	2745	41	24859
OVER	**Comp 2**	Pérola all samples of leaves vs BAT 477 all samples of leaves	Down-regulated DEGs	GO:0032555	purine ribonucleotide binding	MF	2.36E-03	18	2735	41	24869
OVER	**Comp 2**	Pérola all samples of leaves vs BAT 477 all samples of leaves	Down-regulated DEGs	GO:0005524	ATP binding	MF	2.36E-03	17	2499	42	25105
OVER	**Comp 2**	Pérola all samples of leaves vs BAT 477 all samples of leaves	Down-regulated DEGs	GO:0016301	kinase activity	MF	2.36E-03	13	1423	46	26181
OVER	**Comp 2**	Pérola all samples of leaves vs BAT 477 all samples of leaves	Down-regulated DEGs	GO:0017076	purine nucleotide binding	MF	2.36E-03	18	2746	41	24858
OVER	**Comp 2**	Pérola all samples of leaves vs BAT 477 all samples of leaves	Down-regulated DEGs	GO:0032553	ribonucleotide binding	MF	2.36E-03	18	2767	41	24837
OVER	**Comp 2**	Pérola all samples of leaves vs BAT 477 all samples of leaves	Down-regulated DEGs	GO:0097367	carbohydrate derivative binding	MF	2.37E-03	18	2790	41	24814
OVER	**Comp 2**	Pérola all samples of leaves vs BAT 477 all samples of leaves	Down-regulated DEGs	GO:0043168	anion binding	MF	2.43E-03	19	3127	40	24477
OVER	**Comp 2**	Pérola all samples of leaves vs BAT 477 all samples of leaves	Down-regulated DEGs	GO:0036094	small molecule binding	MF	2.43E-03	19	3112	40	24492
OVER	**Comp 2**	Pérola all samples of leaves vs BAT 477 all samples of leaves	Down-regulated DEGs	GO:0004672	protein kinase activity	MF	2.43E-03	12	1294	47	26310
OVER	**Comp 2**	Pérola all samples of leaves vs BAT 477 all samples of leaves	Down-regulated DEGs	GO:0005488	binding	MF	2.76E-03	35	8889	24	18715
OVER	**Comp 2**	Pérola all samples of leaves vs BAT 477 all samples of leaves	Down-regulated DEGs	GO:0035639	purine ribonucleoside triphosphate binding	MF	4.70E-03	17	2720	42	24884
OVER	**Comp 2**	Pérola all samples of leaves vs BAT 477 all samples of leaves	Down-regulated DEGs	GO:0016773	phosphotransferase activity, alcohol group as acceptor	MF	5.05E-03	12	1424	47	26180
OVER	**Comp 2**	Pérola all samples of leaves vs BAT 477 all samples of leaves	Down-regulated DEGs	GO:0097159	organic cyclic compound binding	MF	6.80E-03	26	5686	33	21918
OVER	**Comp 2**	Pérola all samples of leaves vs BAT 477 all samples of leaves	Down-regulated DEGs	GO:1901363	heterocyclic compound binding	MF	6.80E-03	26	5685	33	21919
OVER	**Comp 2**	Pérola all samples of leaves vs BAT 477 all samples of leaves	Down-regulated DEGs	GO:0016740	transferase activity	MF	9.23E-03	17	2902	42	24702
OVER	**Comp 2**	Pérola all samples of leaves vs BAT 477 all samples of leaves	Down-regulated DEGs	GO:0043531	ADP binding	MF	1.15E-02	6	354	53	27250
OVER	**Comp 3.3**	BAT 477, root in T0 vs BAT 477, root in T150	Down-regulated DEGs	GO:0055114	oxidation-reduction process	BP	1.98E-02	18	1460	93	26092

OVER	**Comp 3.3**	BAT 477, root in T0 vs BAT 477, root in T150	Down-regulated DEGs	GO:0016020	membrane	CC	4.11E-02	22	2222	89	25330
OVER	**Comp 3.3**	BAT 477, root in T0 vs BAT 477, root in T150	Down-regulated DEGs	GO:0016705	oxidoreductase activity, acting on paired donors, with incorporation or reduction of molecular oxygen	MF	1.21E-03	12	450	99	27102
OVER	**Comp 3.3**	BAT 477, root in T0 vs BAT 477, root in T150	Down-regulated DEGs	GO:0016706	oxidoreductase activity, acting on paired donors, with incorporation or reduction of molecular oxygen, 2-oxoglutarate as one donor, and incorporation of one atom each of oxygen into both donors	MF	3.55E-03	7	139	104	27413
OVER	**Comp 3.3**	BAT 477, root in T0 vs BAT 477, root in T150	Down-regulated DEGs	GO:0051213	dioxygenase activity	MF	1.48E-02	7	186	104	27366
OVER	**Comp 3.3**	BAT 477, root in T0 vs BAT 477, root in T150	Down-regulated DEGs	GO:0016491	oxidoreductase activity	MF	2.63E-02	19	1666	92	25886
OVER	**Comp 11**	Pérola, leaf in T75 vs BAT 477, leaf in T75	Down-regulated DEGs	GO:0009607	response to biotic stimulus	BP	1.02E-03	5	59	56	27543
OVER	**Comp 11**	Pérola, leaf in T75 vs BAT 477, leaf in T75	Down-regulated DEGs	GO:0006952	defense response	BP	1.18E-03	6	186	55	27416
OVER	**Comp 11**	Pérola, leaf in T75 vs BAT 477, leaf in T75	Down-regulated DEGs	GO:0006468	protein phosphorylation	BP	1.18E-03	13	1289	48	26313
OVER	**Comp 11**	Pérola, leaf in T75 vs BAT 477, leaf in T75	Down-regulated DEGs	GO:0016310	phosphorylation	BP	1.82E-03	13	1392	48	26210
OVER	**Comp 11**	Pérola, leaf in T75 vs BAT 477, leaf in T75	Down-regulated DEGs	GO:0006464	cellular protein modification process	BP	3.74E-03	13	1560	48	26042
OVER	**Comp 11**	Pérola, leaf in T75 vs BAT 477, leaf in T75	Down-regulated DEGs	GO:0036211	protein modification process	BP	3.74E-03	13	1560	48	26042

OVER	**Comp 11**	Pérola, leaf in T75 vs BAT 477, leaf in T75	Down-regulated DEGs	GO:0043412	macromolecule modification	BP	4.79E-03	13	1614	48	25988
OVER	**Comp 11**	Pérola, leaf in T75 vs BAT 477, leaf in T75	Down-regulated DEGs	GO:0006796	phosphate-containing compound metabolic process	BP	5.27E-03	13	1641	48	25961
OVER	**Comp 11**	Pérola, leaf in T75 vs BAT 477, leaf in T75	Down-regulated DEGs	GO:0006793	phosphorus metabolic process	BP	5.27E-03	13	1644	48	25958
OVER	**Comp 11**	Pérola, leaf in T75 vs BAT 477, leaf in T75	Down-regulated DEGs	GO:0006950	response to stress	BP	2.10E-02	7	550	54	27052
OVER	**Comp 11**	Pérola, leaf in T75 vs BAT 477, leaf in T75	Down-regulated DEGs	GO:0032549	ribonucleoside binding	MF	1.18E-03	19	2743	42	24859
OVER	**Comp 11**	Pérola, leaf in T75 vs BAT 477, leaf in T75	Down-regulated DEGs	GO:0001883	purine nucleoside binding	MF	1.18E-03	19	2734	42	24868
OVER	**Comp 11**	Pérola, leaf in T75 vs BAT 477, leaf in T75	Down-regulated DEGs	GO:0032550	purine ribonucleoside binding	MF	1.18E-03	19	2734	42	24868
OVER	**Comp 11**	Pérola, leaf in T75 vs BAT 477, leaf in T75	Down-regulated DEGs	GO:0001882	nucleoside binding	MF	1.18E-03	19	2744	42	24858
OVER	**Comp 11**	Pérola, leaf in T75 vs BAT 477, leaf in T75	Down-regulated DEGs	GO:0004672	protein kinase activity	MF	1.18E-03	13	1293	48	26309
OVER	**Comp 11**	Pérola, leaf in T75 vs BAT 477, leaf in T75	Down-regulated DEGs	GO:0005524	ATP binding	MF	1.18E-03	18	2498	43	25104
OVER	**Comp 11**	Pérola, leaf in T75 vs BAT 477, leaf in T75	Down-regulated DEGs	GO:0032555	purine ribonucleotide binding	MF	1.18E-03	19	2734	42	24868
OVER	**Comp 11**	Pérola, leaf in T75 vs BAT 477, leaf in T75	Down-regulated DEGs	GO:0030554	adenyl nucleotide binding	MF	1.18E-03	19	2517	42	25085
OVER	**Comp 11**	Pérola, leaf in T75 vs BAT 477, leaf in T75	Down-regulated DEGs	GO:0017076	purine nucleotide binding	MF	1.18E-03	19	2745	42	24857
OVER	**Comp 11**	Pérola, leaf in T75 vs BAT 477, leaf in T75	Down-regulated DEGs	GO:0032553	ribonucleotide binding	MF	1.18E-03	19	2766	42	24836
OVER	**Comp 11**	Pérola, leaf in T75 vs BAT 477, leaf in T75	Down-regulated DEGs	GO:0032559	adenyl ribonucleotide binding	MF	1.18E-03	19	2513	42	25089
OVER	**Comp 11**	Pérola, leaf in T75 vs BAT 477, leaf in T75	Down-regulated DEGs	GO:0097367	carbohydrate derivative binding	MF	1.24E-03	19	2789	42	24813
OVER	**Comp 11**	Pérola, leaf in T75 vs BAT 477, leaf in T75	Down-regulated DEGs	GO:0043168	anion binding	MF	1.56E-03	20	3126	41	24476
OVER	**Comp 11**	Pérola, leaf in T75 vs BAT 477, leaf in T75	Down-regulated DEGs	GO:0016740	transferase activity	MF	1.82E-03	19	2900	42	24702
OVER	**Comp 11**	Pérola, leaf in T75 vs BAT 477, leaf in T75	Down-regulated DEGs	GO:0016773	phosphotransferase activity,	MF	2.00E-03	13	1423	48	26179

					alcohol group as acceptor						
OVER	**Comp 11**	Pérola, leaf in T75 vs BAT 477, leaf in T75	Down-regulated DEGs	GO:0016301	kinase activity	MF	2.00E-03	13	1423	48	26179
OVER	**Comp 11**	Pérola, leaf in T75 vs BAT 477, leaf in T75	Down-regulated DEGs	GO:0035639	purine ribonucleoside triphosphate binding	MF	2.51E-03	18	2719	43	24883
OVER	**Comp 11**	Pérola, leaf in T75 vs BAT 477, leaf in T75	Down-regulated DEGs	GO:0043167	ion binding	MF	2.71E-03	24	4553	37	23049
OVER	**Comp 11**	Pérola, leaf in T75 vs BAT 477, leaf in T75	Down-regulated DEGs	GO:1901265	nucleoside phosphate binding	MF	2.98E-03	19	3064	42	24538
OVER	**Comp 11**	Pérola, leaf in T75 vs BAT 477, leaf in T75	Down-regulated DEGs	GO:0000166	nucleotide binding	MF	2.98E-03	19	3064	42	24538
OVER	**Comp 11**	Pérola, leaf in T75 vs BAT 477, leaf in T75	Down-regulated DEGs	GO:0005488	binding	MF	3.14E-03	36	8888	25	18714
OVER	**Comp 11**	Pérola, leaf in T75 vs BAT 477, leaf in T75	Down-regulated DEGs	GO:0036094	small molecule binding	MF	3.42E-03	19	3112	42	24490
OVER	**Comp 11**	Pérola, leaf in T75 vs BAT 477, leaf in T75	Down-regulated DEGs	GO:0097159	organic cyclic compound binding	MF	4.42E-03	27	5685	34	21917
OVER	**Comp 11**	Pérola, leaf in T75 vs BAT 477, leaf in T75	Down-regulated DEGs	GO:1901363	heterocyclic compound binding	MF	4.42E-03	27	5684	34	21918
OVER	**Comp 11**	Pérola, leaf in T75 vs BAT 477, leaf in T75	Down-regulated DEGs	GO:0016772	transferase activity, transferring phosphorus-containing groups	MF	5.27E-03	13	1643	48	25959
OVER	**Comp 12**	Pérola, leaf in T150 vs BAT 477, leaf in T150	Down-regulated DEGs	GO:0006915	apoptotic process	BP	3.41E-02	5	345	26	27287
OVER	**Comp 12**	Pérola, leaf in T150 vs BAT 477, leaf in T150	Down-regulated DEGs	GO:0008219	cell death	BP	3.41E-02	5	369	26	27263
OVER	**Comp 12**	Pérola, leaf in T150 vs BAT 477, leaf in T150	Down-regulated DEGs	GO:0009607	response to biotic stimulus	BP	3.41E-02	3	61	28	27571
OVER	**Comp 12**	Pérola, leaf in T150 vs BAT 477, leaf in T150	Down-regulated DEGs	GO:0006952	defense response	BP	3.41E-02	4	188	27	27444
OVER	**Comp 12**	Pérola, leaf in T150 vs BAT 477, leaf in T150	Down-regulated DEGs	GO:0012501	programmed cell death	BP	3.41E-02	5	349	26	27283
OVER	**Comp 12**	Pérola, leaf in T150 vs BAT 477, leaf in T150	Down-regulated DEGs	GO:0043531	ADP binding	MF	8.72E-03	6	354	25	27278

* over/under-represented genes: Over means enriched DEGs for a particular category; under means fewer DEGs for a particular category, both of them associated with p-value.

** The “all gene” set represents the junction of the 27197 gene in the *P.vulgaris* genome plus the 466 new loci full annotated.

Test: Number of genes from the set of new loci annotated with the GO ID
Ref: Number of genes in the reference subset (all genes) annotated with the GO ID
Non Annot Test: Number of genes in the tested subset (DEGs) not annotated with the GO ID
Non Annot Reference: Number of genes in the reference subset (all genes) not annotated with the GO ID

Over/Under*	GO ID	GO Name	GO Category$	FDR	P-value	Test	Ref	Non Annot Test	Non Annot Reference
**DEGs and genes under high impact SNP effects (HISE)**									
OVER	GO:0043412	macromolecule modification	BP	4.32E-05	3.49E-07	207	1420	2294	23742
OVER	GO:0006915	apoptotic process	BP	7.86E-05	7.05E-07	61	289	2440	24873
OVER	GO:0012501	programmed cell death	BP	9.55E-05	8.85E-07	61	293	2440	24869
OVER	GO:0008219	cell death	BP	1.59E-04	1.67E-06	63	311	2438	24851
OVER	GO:0044763	single-organism cellular process	BP	1.59E-04	1.63E-06	231	1657	2270	23505
OVER	GO:0002376	immune system process	BP	1.81E-04	2.33E-06	28	92	2473	25070
OVER	GO:0006464	cellular protein modification process	BP	1.81E-04	2.32E-06	197	1376	2304	23786
OVER	GO:0045087	innate immune response	BP	1.81E-04	2.33E-06	28	92	2473	25070
OVER	GO:0006955	immune response	BP	1.81E-04	2.33E-06	28	92	2473	25070
OVER	GO:0036211	protein modification process	BP	1.81E-04	2.32E-06	197	1376	2304	23786
OVER	GO:0016310	phosphorylation	BP	3.51E-04	5.36E-06	177	1228	2324	23934
OVER	GO:0006796	phosphate-containing compound metabolic process	BP	3.86E-04	6.11E-06	203	1451	2298	23711
OVER	GO:0006793	phosphorus metabolic process	BP	3.90E-04	6.30E-06	203	1454	2298	23708
OVER	GO:0051716	cellular response to stimulus	BP	6.77E-04	1.11E-05	88	520	2413	24642
OVER	GO:0006468	protein phosphorylation	BP	7.42E-04	1.24E-05	164	1138	2337	24024
OVER	GO:0043170	macromolecule metabolic process	BP	0.001427	2.52E-05	488	4070	2013	21092
OVER	GO:0009987	cellular process	BP	0.001503	2.70E-05	677	5858	1824	19304
OVER	GO:0051274	beta-glucan biosynthetic process	BP	0.0031414	6.01E-05	16	44	2485	25118
OVER	GO:0051273	beta-glucan metabolic process	BP	0.0037291	7.47E-05	16	45	2485	25117
OVER	GO:0009250	glucan biosynthetic process	BP	0.0044744	9.23E-05	16	46	2485	25116
OVER	GO:0006310	DNA recombination	BP	0.006101	1.28E-04	17	53	2484	25109
OVER	GO:0007154	cell communication	BP	0.0069118	1.53E-04	74	452	2427	24710
OVER	GO:0071704	organic substance metabolic process	BP	0.0076062	1.71E-04	631	5514	1870	19648
OVER	GO:0044260	cellular macromolecule metabolic process	BP	0.0111671	2.57E-04	435	3679	2066	21483
OVER	GO:0044700	single organism signaling	BP	0.0118854	2.91E-04	63	378	2438	24784
OVER	GO:0007018	microtubule-based movement	BP	0.0118854	2.95E-04	16	52	2485	25110
OVER	GO:0006928	movement of cell or subcellular component	BP	0.0118854	2.95E-04	16	52	2485	25110
OVER	GO:0007165	signal transduction	BP	0.0118854	2.84E-04	63	377	2438	24785
OVER	GO:0023052	signaling	BP	0.0118854	2.91E-04	63	378	2438	24784
OVER	GO:0044238	primary metabolic process	BP	0.0121666	3.08E-04	606	5303	1895	19859

OVER	GO:0007017	microtubule-based process	BP	0.0130426	3.35E-04	21	81	2480	25081
OVER	GO:0030244	cellulose biosynthetic process	BP	0.0232183	6.11E-04	12	34	2489	25128
OVER	GO:0006259	DNA metabolic process	BP	0.0278705	7.42E-04	43	239	2458	24923
OVER	GO:0030243	cellulose metabolic process	BP	0.0280064	7.54E-04	12	35	2489	25127
OVER	GO:0043631	RNA polyadenylation	BP	0.031295	8.61E-04	4	2	2497	25160
OVER	GO:0019538	protein metabolic process	BP	0.0494184	0.0014187	282	2334	2219	22828
UNDER	GO:0030529	intracellular ribonucleoprotein complex	CC	1.81E-04	2.29E-06	11	372	2490	24790
UNDER	GO:1990904	ribonucleoprotein complex	CC	1.81E-04	2.29E-06	11	372	2490	24790
UNDER	GO:0005840	ribosome	CC	2.91E-04	3.92E-06	10	344	2491	24818
UNDER	GO:0044444	cytoplasmic part	CC	0.0031533	6.13E-05	44	788	2457	24374
UNDER	GO:0043228	non-membrane-bounded organelle	CC	0.0461362	0.0013107	28	506	2473	24656
UNDER	GO:0043232	intracellular non-membrane-bounded organelle	CC	0.0461362	0.0013107	28	506	2473	24656
OVER	GO:0036094	small molecule binding	MF	5.16E-17	1.54E-20	432	2699	2069	22463
OVER	GO:1901265	nucleoside phosphate binding	MF	6.48E-17	5.81E-20	424	2659	2077	22503
OVER	GO:0000166	nucleotide binding	MF	6.48E-17	5.81E-20	424	2659	2077	22503
OVER	GO:0032549	ribonucleoside binding	MF	6.94E-17	1.52E-19	387	2375	2114	22787
OVER	GO:0043168	anion binding	MF	6.94E-17	1.67E-19	429	2717	2072	22445
OVER	GO:0035639	purine ribonucleoside triphosphate binding	MF	6.94E-17	2.01E-19	384	2353	2117	22809
OVER	GO:0097367	carbohydrate derivative binding	MF	6.94E-17	1.03E-19	393	2415	2108	22747
OVER	GO:0017076	purine nucleotide binding	MF	6.94E-17	1.09E-19	388	2376	2113	22786
OVER	GO:0032553	ribonucleotide binding	MF	6.94E-17	2.08E-19	389	2396	2112	22766
OVER	GO:0001882	nucleoside binding	MF	6.94E-17	2.06E-19	387	2376	2114	22786
OVER	GO:0032550	purine ribonucleoside binding	MF	1.25E-16	4.88E-19	384	2369	2117	22793
OVER	GO:0001883	purine nucleoside binding	MF	1.25E-16	4.88E-19	384	2369	2117	22793
OVER	GO:0032555	purine ribonucleotide binding	MF	1.25E-16	4.88E-19	384	2369	2117	22793
OVER	GO:0030554	adenyl nucleotide binding	MF	1.04E-15	4.57E-18	356	2180	2145	22982
OVER	GO:0005524	ATP binding	MF	1.04E-15	4.64E-18	354	2162	2147	23000
OVER	GO:0032559	adenyl ribonucleotide binding	MF	2.36E-15	1.13E-17	354	2178	2147	22984
OVER	GO:0043167	ion binding	MF	7.15E-11	3.63E-13	547	4030	1954	21132
OVER	GO:0005488	binding	MF	4.11E-09	2.21E-11	958	7966	1543	17196
OVER	GO:0097159	organic cyclic compound binding	MF	7.34E-09	4.39E-11	647	5065	1854	20097
OVER	GO:1901363	heterocyclic compound binding	MF	7.34E-09	4.37E-11	647	5064	1854	20098

OVER	GO:0005515	protein binding	MF	2.65E-08	1.66E-10	352	2474	2149	22688
OVER	GO:0004386	helicase activity	MF	1.68E-06	1.11E-08	47	168	2454	24994
OVER	GO:0017111	nucleoside-triphosphatase activity	MF	3.73E-06	2.57E-08	107	581	2394	24581
OVER	GO:0016462	pyrophosphatase activity	MF	4.23E-06	3.04E-08	108	589	2393	24573
OVER	GO:0016818	hydrolase activity, acting on acid anhydrides, in phosphorus-containing anhydrides	MF	5.51E-06	4.12E-08	109	601	2392	24561
OVER	GO:0016817	hydrolase activity, acting on acid anhydrides	MF	9.01E-06	7.01E-08	109	608	2392	24554
OVER	GO:0099600	transmembrane receptor activity	MF	5.14E-05	4.46E-07	32	105	2469	25057
OVER	GO:0004888	transmembrane signaling receptor activity	MF	5.14E-05	4.46E-07	32	105	2469	25057
OVER	GO:0008026	ATP-dependent helicase activity	MF	1.49E-04	1.48E-06	29	95	2472	25067
OVER	GO:0070035	purine NTP-dependent helicase activity	MF	1.49E-04	1.48E-06	29	95	2472	25067
OVER	GO:0016772	transferase activity, transferring phosphorus-containing groups	MF	1.81E-04	2.11E-06	206	1450	2295	23712
UNDER	GO:0003735	structural constituent of ribosome	MF	2.91E-04	3.92E-06	10	344	2491	24818
OVER	GO:0043531	ADP binding	MF	3.11E-04	4.28E-06	60	300	2441	24862
OVER	GO:0016740	transferase activity	MF	3.33E-04	4.68E-06	333	2586	2168	22576
OVER	GO:0038023	signaling receptor activity	MF	3.36E-04	4.83E-06	32	119	2469	25043
OVER	GO:0004872	receptor activity	MF	3.51E-04	5.44E-06	32	120	2469	25042
OVER	GO:0060089	molecular transducer activity	MF	3.51E-04	5.44E-06	32	120	2469	25042
OVER	GO:0003824	catalytic activity	MF	3.51E-04	5.45E-06	804	6994	1697	18168
OVER	GO:0016301	kinase activity	MF	7.66E-04	1.31E-05	178	1258	2323	23904
OVER	GO:0016773	phosphotransferase activity, alcohol group as acceptor	MF	0.0011536	2.00E-05	177	1259	2324	23903
OVER	GO:0004672	protein kinase activity	MF	0.0017268	3.15E-05	162	1144	2339	24018
UNDER	GO:0005198	structural molecule activity	MF	0.0019691	3.65E-05	15	390	2486	24772
OVER	GO:0016887	ATPase activity	MF	0.0027202	5.12E-05	62	344	2439	24818
OVER	GO:0035251	UDP-glucosyltransferase activity	MF	0.0037291	7.47E-05	16	45	2485	25117
OVER	GO:0046527	glucosyltransferase activity	MF	0.0044744	9.23E-05	16	46	2485	25116
OVER	GO:0004871	signal transducer activity	MF	0.0065966	1.40E-04	43	221	2458	24941
OVER	GO:0016759	cellulose synthase activity	MF	0.0066951	1.46E-04	12	28	2489	25134
OVER	GO:0016760	cellulose synthase (UDP-forming) activity	MF	0.0066951	1.46E-04	12	28	2489	25134
OVER	GO:0042623	ATPase activity, coupled	MF	0.0078836	1.79E-04	44	230	2457	24932
OVER	GO:0003777	microtubule motor activity	MF	0.0118854	2.95E-04	16	52	2485	25110
OVER	GO:0016755	transferase activity, transferring amino-acyl groups	MF	0.0121666	3.09E-04	4	1	2497	25161
OVER	GO:0070566	adenylyltransferase activity	MF	0.0213444	5.55E-04	5	4	2496	25158

OVER	GO:0004652	polynucleotide adenylyltransferase activity	MF	0.031295	8.61E-04	4	2	2497	25160
OVER	GO:0016787	hydrolase activity	MF	0.0428452	0.0011916	262	2142	2239	23020

* over/under-represented genes: Over means enriched DEGs for a particular category; under means fewer DEGs for a particular category, both of them associated with p-value.

Test: Number of genes in the tested subset (genes under high-impact SNPs effects) annotated with the GO ID

Ref: Number of genes in the reference subset (all genes) annotated with the GO ID

Non Annot Test: Number of genes in the tested subset (genes under high-impact SNPs effects) not annotated with the GO ID

Non Annot Reference: Number of genes in the reference subset (all genes) not annotated with the GO ID
