# Supplementary material for: Genome-wide analysis of the transcriptional response to drought stress in root and leaf of common bean
Source: Genet Mol Biol. 2020 Mar 16;43(1):e20180259. doi: 10.1590/1678-4685-GMB-2018-0259 (PMC7307723; doi:10.1590/1678-4685-GMB-2018-0259)
Supplement: Supplementary file 5 [file 1415-4757-GMB-43-1-e20180259-s7.pdf]

Supplementary Material to: "Genome-wide analysis of the transcriptional response to drought stress in root and leaf of common bean"

Table S5 KEGG metabolic pathways identified for the new loci and DEGs in different tissues, genotypes and treatments.

Pathway	Seqs in Pathway	Enzyme	Enzyme ID	Seqs of Enzyme	Seqs	Pathway ID
**NEW LOCI**						
Alanine, aspartate and glutamate metabolism	3	transaminase	ec:2.6.1.1	1	XLOC_000219	map00250
Alanine, aspartate and glutamate metabolism	3	synthase (glutamine-hydrolysing)	ec:6.3.5.4	1	XLOC_005354	map00250
Alanine, aspartate and glutamate metabolism	3	synthase (glutamine-hydrolysing)	ec:6.3.5.5	1	XLOC_006231	map00250
alpha-Linolenic acid metabolism	2	oxidase	ec:1.3.3.6	2	XLOC_004855, XLOC_025687	map00592
Amino sugar and nucleotide sugar metabolism	1	guanylyltransferase (GDP)	ec:2.7.7.22	1	XLOC_009360	map00520
Aminoacyl-tRNA biosynthesis	1	ligase	ec:6.1.1.4	1	XLOC_015334	map00970
Aminobenzoate degradation	5	acetylphosphatase	ec:3.6.1.7	1	XLOC_018187	map00627
Aminobenzoate degradation	5	nitrophenyl phosphatase	ec:3.1.3.41	4	XLOC_020820, XLOC_002811, XLOC_028468, XLOC_000683	map00627
Arachidonic acid metabolism	1	epoxide hydrolase	ec:3.3.2.10	1	XLOC_010653	map00590
Arginine and proline metabolism	1	transaminase	ec:2.6.1.1	1	XLOC_000219	map00330
Arginine biosynthesis	2	transaminase	ec:2.6.1.1	1	XLOC_000219	map00220
Arginine biosynthesis	2	reductase	ec:1.2.1.38	1	XLOC_006296	map00220
Ascorbate and aldarate metabolism	2	oxygenase	ec:1.13.99.1	1	XLOC_005471	map00053
Ascorbate and aldarate metabolism	2	oxidase	ec:1.10.3.3	1	XLOC_006232	map00053
Biosynthesis of antibiotics	11	transaminase	ec:2.6.1.57	1	XLOC_000219	map01130
Biosynthesis of antibiotics	11	transaminase	ec:2.6.1.5	1	XLOC_000219	map01130
Biosynthesis of antibiotics	11	transaminase	ec:2.6.1.9	1	XLOC_000219	map01130
Biosynthesis of antibiotics	11	transaminase	ec:2.6.1.1	1	XLOC_000219	map01130
Biosynthesis of antibiotics	11	synthase	ec:2.5.1.21	1	XLOC_001840	map01130
Biosynthesis of antibiotics	11	diphosphate synthase	ec:2.5.1.29	1	XLOC_001840	map01130
Biosynthesis of antibiotics	11	dehydrogenase	ec:1.2.1.11	1	XLOC_006296	map01130
Biosynthesis of antibiotics	11	reductase	ec:1.2.1.38	1	XLOC_006296	map01130
Biosynthesis of antibiotics	11	synthase	ec:2.5.1.47	1	XLOC_008186	map01130
Biosynthesis of antibiotics	11	dehydrogenase (phosphorylating)	ec:1.2.1.12	1	XLOC_008327	map01130
Biosynthesis of antibiotics	11	dehydrogenase (NAD(P)+) (phosphorylating)	ec:1.2.1.59	1	XLOC_008327	map01130
Biosynthesis of antibiotics	11	oxidase	ec:1.1.3.15	2	XLOC_012532, XLOC_011602	map01130
Biosynthesis of antibiotics	11	carboxylase	ec:4.1.1.39	1	XLOC_021592	map01130
Biosynthesis of antibiotics	11	dehydrogenase (2-methylpropanoyl-transferring)	ec:1.2.4.4	1	XLOC_024379	map01130
Biosynthesis of antibiotics	11	O-acetyltransferase	ec:2.3.1.30	2	XLOC_026696, XLOC_024793	map01130
Biosynthesis of unsaturated fatty acids	3	oxidase	ec:1.3.3.6	2	XLOC_004855, XLOC_025687	map01040
Biosynthesis of unsaturated fatty acids	3	9-desaturase	ec:1.14.19.2	1	XLOC_012138	map01040
Carbon fixation in photosynthetic organisms	3	transaminase	ec:2.6.1.1	1	XLOC_000219	map00710
Carbon fixation in photosynthetic organisms	3	dehydrogenase (phosphorylating)	ec:1.2.1.12	1	XLOC_008327	map00710

Carbon fixation in photosynthetic organisms	3	dehydrogenase (NAD(P)+) (phosphorylating)	ec:1.2.1.59	1	XLOC_008327	map00710
Carbon fixation in photosynthetic organisms	3	carboxylase	ec:4.1.1.39	1	XLOC_021592	map00710
Chloroalkane and chloroalkene degradation	1	epoxide hydrolase	ec:3.3.2.10	1	XLOC_010653	map00625
Cyanoamino acid metabolism	2	gentiobiase	ec:3.2.1.21	2	XLOC_027135, XLOC_021585	map00460
Cysteine and methionine metabolism	6	transaminase	ec:2.6.1.57	1	XLOC_000219	map00270
Cysteine and methionine metabolism	6	transaminase	ec:2.6.1.5	1	XLOC_000219	map00270
Cysteine and methionine metabolism	6	transaminase	ec:2.6.1.1	1	XLOC_000219	map00270
Cysteine and methionine metabolism	6	ligase	ec:6.3.2.2	1	XLOC_002563	map00270
Cysteine and methionine metabolism	6	dehydrogenase	ec:1.2.1.11	1	XLOC_006296	map00270
Cysteine and methionine metabolism	6	synthase	ec:2.5.1.47	1	XLOC_008186	map00270
Cysteine and methionine metabolism	6	O-acetyltransferase	ec:2.3.1.30	2	XLOC_026696, XLOC_024793	map00270
Drug metabolism - cytochrome P450	1	transferase	ec:2.5.1.18	1	XLOC_011289	map00982
Drug metabolism - other enzymes	3	ali-esterase	ec:3.1.1.1	3	XLOC_003388, XLOC_015334, XLOC_028615	map00983
Fatty acid biosynthesis	2	9-desaturase	ec:1.14.19.2	1	XLOC_012138	map00061
Fatty acid biosynthesis	2	ligase	ec:6.2.1.3	1	XLOC_015561	map00061
Fatty acid degradation	3	oxidase	ec:1.3.3.6	2	XLOC_004855, XLOC_025687	map00071
Fatty acid degradation	3	ligase	ec:6.2.1.3	1	XLOC_015561	map00071
Flavonoid biosynthesis	3	O-hydroxycinnamoyltransferase	ec:2.3.1.133	3	XLOC_015576, XLOC_013302, XLOC_013296	map00941
Fructose and mannose metabolism	1	guanylyltransferase (GDP)	ec:2.7.7.22	1	XLOC_009360	map00051
Galactose metabolism	2	lactase (ambiguous)	ec:3.2.1.23	1	XLOC_009829	map00052
Galactose metabolism	2	invertase	ec:3.2.1.26	1	XLOC_022009	map00052
Glutathione metabolism	2	ligase	ec:6.3.2.2	1	XLOC_002563	map00480
Glutathione metabolism	2	transferase	ec:2.5.1.18	1	XLOC_011289	map00480
Glycerolipid metabolism	1	lipase	ec:3.1.1.23	1	XLOC_003388	map00561
Glycerophospholipid metabolism	3	phosphodiesterase	ec:3.1.4.46	1	XLOC_001777	map00564
Glycerophospholipid metabolism	3	decarboxylase	ec:4.1.1.65	1	XLOC_023878	map00564
Glycerophospholipid metabolism	3	cytidylyltransferase	ec:2.7.7.15	1	XLOC_028449	map00564
Glycine, serine and threonine metabolism	1	dehydrogenase	ec:1.2.1.11	1	XLOC_006296	map00260
Glycolysis / Gluconeogenesis	1	dehydrogenase (phosphorylating)	ec:1.2.1.12	1	XLOC_008327	map00010
Glycolysis / Gluconeogenesis	1	dehydrogenase (NAD(P)+) (phosphorylating)	ec:1.2.1.59	1	XLOC_008327	map00010
Glycosaminoglycan biosynthesis - chondroitin sulfate / dermatan sulfate	2	3-beta-galactosyltransferase	ec:2.4.1.134	2	XLOC_012966, XLOC_012967	map00532
Glycosaminoglycan biosynthesis - heparan sulfate / heparin	2	3-beta-galactosyltransferase	ec:2.4.1.134	2	XLOC_012966, XLOC_012967	map00534
Glycosaminoglycan degradation	1	lactase (ambiguous)	ec:3.2.1.23	1	XLOC_009829	map00531
Glycosphingolipid biosynthesis - ganglio series	1	lactase (ambiguous)	ec:3.2.1.23	1	XLOC_009829	map00604
Glyoxylate and dicarboxylate metabolism	4	oxidase	ec:1.1.3.15	2	XLOC_012532, XLOC_011602	map00630
Glyoxylate and dicarboxylate metabolism	4	carboxylase	ec:4.1.1.39	1	XLOC_021592	map00630
Glyoxylate and dicarboxylate metabolism	4	ligase	ec:6.2.1.8	1	XLOC_025693	map00630
Histidine metabolism	1	transaminase	ec:2.6.1.9	1	XLOC_000219	map00340
Inositol phosphate metabolism	2	oxygenase	ec:1.13.99.1	1	XLOC_005471	map00562
Inositol phosphate metabolism	2	1-phosphatase	ec:3.1.3.57	1	XLOC_028468	map00562
Isoquinoline alkaloid biosynthesis	1	transaminase	ec:2.6.1.57	1	XLOC_000219	map00950

Isoquinoline alkaloid biosynthesis	1	transaminase	ec:2.6.1.5	1	XLOC_000219	map00950
Isoquinoline alkaloid biosynthesis	1	transaminase	ec:2.6.1.1	1	XLOC_000219	map00950
Lysine biosynthesis	2	transaminase	ec:2.6.1.57	1	XLOC_000219	map00300
Lysine biosynthesis	2	dehydrogenase	ec:1.2.1.11	1	XLOC_006296	map00300
Lysine degradation	2	N-methyltransferase	ec:2.1.1.43	2	XLOC_025694, XLOC_015591	map00310
Metabolism of xenobiotics by cytochrome P450	2	epoxide hydrolase	ec:3.3.2.9	1	XLOC_010653	map00980
Metabolism of xenobiotics by cytochrome P450	2	transferase	ec:2.5.1.18	1	XLOC_011289	map00980
Monobactam biosynthesis	1	dehydrogenase	ec:1.2.1.11	1	XLOC_006296	map00261
mTOR signaling pathway	1	protein kinase	ec:2.7.11.24	1	XLOC_017913	map04150
N-Glycan biosynthesis	1	N-acetylglucosaminyltransferase	ec:2.4.1.141	1	XLOC_003305	map00510
Nitrogen metabolism	1	monooxygenase	ec:1.13.12.16	1	XLOC_028213	map00910
Novobiocin biosynthesis	1	transaminase	ec:2.6.1.57	1	XLOC_000219	map00401
Novobiocin biosynthesis	1	transaminase	ec:2.6.1.5	1	XLOC_000219	map00401
Novobiocin biosynthesis	1	transaminase	ec:2.6.1.9	1	XLOC_000219	map00401
Novobiocin biosynthesis	1	transaminase	ec:2.6.1.1	1	XLOC_000219	map00401
Other glycan degradation	3	lactase (ambiguous)	ec:3.2.1.23	1	XLOC_009829	map00511
Other glycan degradation	3	alpha-fucosidase	ec:3.2.1.51	1	XLOC_025618	map00511
Other glycan degradation	3	alpha-D-mannosidase	ec:3.2.1.24	1	XLOC_028601	map00511
Oxidative phosphorylation	8	dehydrogenase	ec:1.6.99.3	8	XLOC_013320, XLOC_026159, XLOC_028496, XLOC_018528, XLOC_009333, XLOC_011323, XLOC_024323, XLOC_024334	map00190
Oxidative phosphorylation	8	reductase (H+-translocating)	ec:1.6.5.3	8	XLOC_013320, XLOC_026159, XLOC_028496, XLOC_018528, XLOC_009333, XLOC_011323, XLOC_024323, XLOC_024334	map00190
Pentose and glucuronate interconversions	3	pectin depolymerase	ec:3.2.1.15	2	XLOC_004981, XLOC_025825	map00040
Pentose and glucuronate interconversions	3	pectin demethoxylase	ec:3.1.1.11	1	XLOC_028615	map00040
Phenylalanine metabolism	1	transaminase	ec:2.6.1.57	1	XLOC_000219	map00360
Phenylalanine metabolism	1	transaminase	ec:2.6.1.5	1	XLOC_000219	map00360
Phenylalanine metabolism	1	transaminase	ec:2.6.1.9	1	XLOC_000219	map00360
Phenylalanine metabolism	1	transaminase	ec:2.6.1.1	1	XLOC_000219	map00360
Phenylalanine, tyrosine and tryptophan biosynthesis	1	transaminase	ec:2.6.1.57	1	XLOC_000219	map00400
Phenylalanine, tyrosine and tryptophan biosynthesis	1	transaminase	ec:2.6.1.5	1	XLOC_000219	map00400
Phenylalanine, tyrosine and tryptophan biosynthesis	1	transaminase	ec:2.6.1.9	1	XLOC_000219	map00400
Phenylalanine, tyrosine and tryptophan biosynthesis	1	transaminase	ec:2.6.1.1	1	XLOC_000219	map00400
Phenylpropanoid biosynthesis	6	glucosyltransferase	ec:2.4.1.111	1	XLOC_011352	map00940
Phenylpropanoid biosynthesis	6	O-hydroxycinnamoyltransferase	ec:2.3.1.133	3	XLOC_015576, XLOC_013302, XLOC_013296	map00940
Phenylpropanoid biosynthesis	6	gentiobiase	ec:3.2.1.21	2	XLOC_027135, XLOC_021585	map00940
Phosphatidylinositol signaling system	2	kinase C	ec:2.7.11.13	1	XLOC_023878	map04070
Phosphatidylinositol signaling system	2	1-phosphatase	ec:3.1.3.57	1	XLOC_028468	map04070
Phosphonate and phosphinate metabolism	1	cytidylyltransferase	ec:2.7.7.15	1	XLOC_028449	map00440
Porphyrin and chlorophyll metabolism	2	reductase	ec:1.2.1.70	1	XLOC_013297	map00860

Porphyrin and chlorophyll metabolism	2	ferro-protoporphyrin chelatase	ec:4.99.1.1	1	XLOC_022070	map00860
Propanoate metabolism	2	dehydrogenase (2-methylpropanoyl-transferring)	ec:1.2.4.4	1	XLOC_024379	map00640
Propanoate metabolism	2	dehydrogenase (CoA-acylating)	ec:1.2.1.27	1	XLOC_025316	map00640
Purine metabolism	58	RNA polymerase	ec:2.7.7.6	17	XLOC_006235, XLOC_021599, XLOC_011348, XLOC_008645, XLOC_006276, XLOC_009413, XLOC_003713, XLOC_008647, XLOC_026144, XLOC_021636, XLOC_026165, XLOC_008646, XLOC_000084, XLOC_024324, XLOC_014752, XLOC_021578, XLOC_020681,	map00230
Purine metabolism	58	phosphatase	ec:3.6.1.15	38	XLOC_006807, XLOC_019070, XLOC_008118, XLOC_018507, XLOC_021633, XLOC_016155, XLOC_001867, XLOC_017516, XLOC_020838, XLOC_011500, XLOC_028551, XLOC_018543, XLOC_001720, XLOC_026159, XLOC_001558, XLOC_011260, XLOC_014554, XLOC_021580, XLOC_026199, XLOC_023069, XLOC_002809, XLOC_007172, XLOC_024377, XLOC_024966, XLOC_024400, XLOC_021664, XLOC_008395, XLOC_019320, XLOC_021607, XLOC_010568, XLOC_013365, XLOC_014473, XLOC_015899, XLOC_000666, XLOC_002741, XLOC_022735, XLOC_017569, XLOC_024367, ,	map00230

Purine metabolism	58	adenylpyrophosphatase	ec:3.6.1.3	29	XLOC_007764, XLOC_019070, XLOC_008118, XLOC_018507, XLOC_021633, XLOC_017516, XLOC_011500, XLOC_028551, XLOC_026159, XLOC_011260, XLOC_021580, XLOC_026199, XLOC_023069, XLOC_002809, XLOC_007172, XLOC_024377, XLOC_024966, XLOC_024400, XLOC_021664, XLOC_019320, XLOC_021607, XLOC_010568, XLOC_013365, XLOC_014473, XLOC_000666, XLOC_002741, XLOC_022735, XLOC_017569, XLOC_024367	map00230
Purine metabolism	58	dehydrogenase	ec:1.17.1.4	1	XLOC_008379	map00230
Purine metabolism	58	DNA polymerase	ec:2.7.7.7	3	XLOC_011720, XLOC_011666, XLOC_024367	map00230
Pyrimidine metabolism	21	synthase (glutamine-hydrolysing)	ec:6.3.5.5	1	XLOC_006231	map00240
Pyrimidine metabolism	21	RNA polymerase	ec:2.7.7.6	17	XLOC_006235, XLOC_021599, XLOC_011348, XLOC_008645, XLOC_006276, XLOC_009413, XLOC_003713, XLOC_008647, XLOC_026144, XLOC_021636, XLOC_026165, XLOC_008646, XLOC_000084, XLOC_024324, XLOC_014752, XLOC_021578, XLOC_020681,	map00240
Pyrimidine metabolism	21	DNA polymerase	ec:2.7.7.7	3	XLOC_011720, XLOC_011666, XLOC_024367	map00240
Pyruvate metabolism	1	acetylphosphatase	ec:3.6.1.7	1	XLOC_018187	map00620
Sesquiterpenoid and triterpenoid biosynthesis	1	synthase	ec:2.5.1.21	1	XLOC_001840	map00909
Sphingolipid metabolism	2	N-acyltransferase	ec:2.3.1.24	1	XLOC_007764	map00600
Sphingolipid metabolism	2	lactase (ambiguous)	ec:3.2.1.23	1	XLOC_009829	map00600
Starch and sucrose metabolism	9	pectin depolymerase	ec:3.2.1.15	2	XLOC_004981, XLOC_025825	map00500
Starch and sucrose metabolism	9	synthase (glycosyl-transferring)	ec:2.4.1.21	1	XLOC_005207	map00500
Starch and sucrose metabolism	9	phosphorylase	ec:2.4.1.1	1	XLOC_005481	map00500
Starch and sucrose metabolism	9	trehalose 6-phosphatase	ec:3.1.3.12	1	XLOC_020820	map00500
Starch and sucrose metabolism	9	alpha-glucosidase	ec:3.2.1.48	1	XLOC_022009	map00500
Starch and sucrose metabolism	9	invertase	ec:3.2.1.26	1	XLOC_022009	map00500
Starch and sucrose metabolism	9	1,3-beta-glucosidase	ec:3.2.1.58	1	XLOC_027135	map00500
Starch and sucrose metabolism	9	gentiobiase	ec:3.2.1.21	2	XLOC_027135, XLOC_021585	map00500
Starch and sucrose metabolism	9	pectin demethoxylase	ec:3.1.1.11	1	XLOC_028615	map00500

Steroid biosynthesis	1	synthase	ec:2.5.1.21	1	XLOC_001840	map00100
Stilbenoid, diarylheptanoid and gingerol biosynthesis	3	O-hydroxycinnamoyltransferase	ec:2.3.1.133	3	XLOC_015576, XLOC_013302, XLOC_013296	map00945
Sulfur metabolism	4	synthase	ec:2.5.1.47	1	XLOC_008186	map00920
Sulfur metabolism	4	O-acetyltransferase	ec:2.3.1.30	2	XLOC_026696, XLOC_024793	map00920
Sulfur metabolism	4	nucleotidase	ec:3.1.3.7	1	XLOC_028468	map00920
T cell receptor signaling pathway	4	phosphatase	ec:3.1.3.16	2	XLOC_002811, XLOC_000683	map04660
T cell receptor signaling pathway	4	protein-tyrosine kinase	ec:2.7.10.2	2	XLOC_017321, XLOC_017187	map04660
Terpenoid backbone biosynthesis	2	diphosphate synthase	ec:2.5.1.29	1	XLOC_001840	map00900
Terpenoid backbone biosynthesis	2	diphosphate synthase [geranylgeranyl-diphosphate specific]	ec:2.5.1.85	1	XLOC_011997	map00900
Terpenoid backbone biosynthesis	2	synthase [geranyl-diphosphate specific]	ec:2.5.1.84	1	XLOC_011997	map00900
Terpenoid backbone biosynthesis	2	diphosphate synthase	ec:2.5.1.30	1	XLOC_011997	map00900
Thiamine metabolism	38	phosphatase	ec:3.6.1.15	38	XLOC_006807, XLOC_019070, XLOC_008118, XLOC_018507, XLOC_021633, XLOC_016155, XLOC_001867, XLOC_017516, XLOC_020838, XLOC_011500, XLOC_028551, XLOC_018543, XLOC_001720, XLOC_026159, XLOC_001558, XLOC_011260, XLOC_014554, XLOC_021580, XLOC_026199, XLOC_023069, XLOC_002809, XLOC_007172, XLOC_024377, XLOC_024966, XLOC_024400, XLOC_021664, XLOC_008395, XLOC_019320, XLOC_021607, XLOC_010568, XLOC_013365, XLOC_014473, XLOC_015899, XLOC_000666, XLOC_002741, XLOC_022735, XLOC_017569, XLOC_024367	map00730
Tropane, piperidine and pyridine alkaloid biosynthesis	2	transaminase	ec:2.6.1.57	1	XLOC_000219	map00960
Tropane, piperidine and pyridine alkaloid biosynthesis	2	transaminase	ec:2.6.1.5	1	XLOC_000219	map00960
Tropane, piperidine and pyridine alkaloid biosynthesis	2	transaminase	ec:2.6.1.9	1	XLOC_000219	map00960
Tropane, piperidine and pyridine alkaloid biosynthesis	2	transaminase	ec:2.6.1.1	1	XLOC_000219	map00960
Tropane, piperidine and pyridine alkaloid biosynthesis	2	reductase I	ec:1.1.1.206	1	XLOC_017843	map00960
Tyrosine metabolism	1	transaminase	ec:2.6.1.57	1	XLOC_000219	map00350
Tyrosine metabolism	1	transaminase	ec:2.6.1.5	1	XLOC_000219	map00350
Tyrosine metabolism	1	transaminase	ec:2.6.1.9	1	XLOC_000219	map00350
Tyrosine metabolism	1	transaminase	ec:2.6.1.1	1	XLOC_000219	map00350

Ubiquinone and other terpenoid-quinone biosynthesis	2	transaminase	ec:2.6.1.5	1	XLOC_000219	map00130
Ubiquinone and other terpenoid-quinone biosynthesis	2	ligase	ec:6.2.1.26	1	XLOC_025693	map00130
Valine, leucine and isoleucine degradation	3	dehydrogenase (2-methylpropanoyl-transferring)	ec:1.2.4.4	1	XLOC_024379	map00280
Valine, leucine and isoleucine degradation	3	dehydrogenase (CoA-acylating)	ec:1.2.1.27	1	XLOC_025316	map00280
Valine, leucine and isoleucine degradation	3	dehydrogenase	ec:1.1.1.31	1	XLOC_026345	map00280
Various types of N-glycan biosynthesis	1	N-acetylglucosaminyltransferase	ec:2.4.1.141	1	XLOC_003305	map00513

$^{\$}$ **D-G:** Down regulated/ **U-R**: Up regulated

Comparison	Comparison ID	Gene Set$^{\$}$	Pathway	Enzyme	Enzyme ID	Pathway ID	Seqs
			**DEG**				
**Comp1**	Pérola all samples of root vs BAT 477 all samples of root	D-G	Purine metabolism	RNA polymerase	ec:2.7.7.6	map00230	XLOC_020681
**Comp1**	Pérola all samples of root vs BAT 477 all samples of root	D-G	Various types of N-glycan biosynthesis	1,2-alpha-mannosidase	ec:3.2.1.113	map00513	Phvul.006G033000
**Comp1**	Pérola all samples of root vs BAT 477 all samples of root	D-G	Other glycan degradation	alpha-D-mannosidase	ec:3.2.1.24	map00511	Phvul.006G033000
**Comp1**	Pérola all samples of root vs BAT 477 all samples of root	D-G	N-Glycan biosynthesis	1,2-alpha-mannosidase	ec:3.2.1.113	map00510	Phvul.006G033000
**Comp1**	Pérola all samples of root vs BAT 477 all samples of root	D-G	T cell receptor signaling pathway	protein-tyrosine kinase	ec:2.7.10.2	map04660	XLOC_017321
**Comp1**	Pérola all samples of root vs BAT 477 all samples of root	D-G	Pyrimidine metabolism	RNA polymerase	ec:2.7.7.6	map00240	XLOC_020681
**Comp2**	Pérola all samples of leaves vs BAT 477 all samples of leaves	D-G	Purine metabolism	RNA polymerase	ec:2.7.7.6	map00230	XLOC_020681
**Comp2**	Pérola all samples of leaves vs BAT 477 all samples of leaves	D-G	Amino sugar and nucleotide sugar metabolism	chitodextrinase	ec:3.2.1.14	map00520	Phvul.004G101500
**Comp2**	Pérola all samples of leaves vs BAT 477 all samples of leaves	D-G	Various types of N-glycan biosynthesis	1,2-alpha-mannosidase	ec:3.2.1.113	map00513	Phvul.006G033000
**Comp2**	Pérola all samples of leaves vs BAT 477 all samples of leaves	D-G	Other glycan degradation	alpha-D-mannosidase	ec:3.2.1.24	map00511	Phvul.006G033000
**Comp2**	Pérola all samples of leaves vs BAT 477 all samples of leaves	D-G	N-Glycan biosynthesis	1,2-alpha-mannosidase	ec:3.2.1.113	map00510	Phvul.006G033000
**Comp2**	Pérola all samples of leaves vs BAT 477 all samples of leaves	D-G	Carotenoid biosynthesis	desaturase	ec:1.3.5.6	map00906	Phvul.010G021000
**Comp2**	Pérola all samples of leaves vs BAT 477 all samples of leaves	D-G	Diterpenoid biosynthesis	10beta-hydroxylase	ec:1.14.13.76	map00904	Phvul.005G053600,Phvul.005G053400
**Comp2**	Pérola all samples of leaves vs BAT 477 all samples of leaves	D-G	Starch and sucrose metabolism	gentiobiase,endo-1,3-beta-D-glucosidase	ec:3.2.1.21,ec:3.2.1.39	map00500	Phvul.001G128500
**Comp2**	Pérola all samples of leaves vs BAT 477 all samples of leaves	D-G	Cyanoamino acid metabolism	gentiobiase	ec:3.2.1.21	map00460	Phvul.001G128500
**Comp2**	Pérola all samples of leaves vs BAT 477 all samples of leaves	D-G	T cell receptor signaling pathway	protein-tyrosine kinase	ec:2.7.10.2	map04660	XLOC_017321
**Comp2**	Pérola all samples of leaves vs BAT 477 all samples of leaves	D-G	Steroid biosynthesis	synthase	ec:5.4.99.8	map00100	Phvul.006G156100
**Comp2**	Pérola all samples of leaves vs BAT 477 all samples of leaves	D-G	Stilbenoid, diarylheptanoid and gingerol biosynthesis	O-hydroxycinnamoyl transferase	ec:2.3.1.133	map00945	Phvul.001G112400,Phvul.010G014100
**Comp2**	Pérola all samples of leaves vs BAT 477 all samples of leaves	D-G	Flavonoid biosynthesis	O-hydroxycinnamoyl transferase	ec:2.3.1.133	map00941	Phvul.001G112400,Phvul.010G014100
**Comp2**	Pérola all samples of leaves vs BAT 477 all samples of leaves	D-G	Phenylpropanoid biosynthesis	O-hydroxycinnamoyl transferase,gentiobiase	ec:2.3.1.133,ec:3.2.1.21	map00940	Phvul.001G112400,Phvul.010G014100,Phvul.001G128500
**Comp2**	Pérola all samples of leaves vs BAT 477 all samples of leaves	D-G	Pyrimidine metabolism	RNA polymerase	ec:2.7.7.6	map00240	XLOC_020681
**Comp2**	Pérola all samples of leaves vs BAT 477 all samples of leaves	U-R	Sulfur metabolism	O-acetyltransferase	ec:2.3.1.30	map00920	XLOC_024793

**Comp2**	Pérola all samples of leaves vs BAT 477 all samples of leaves	U-R	Purine metabolism	adenylpyrophosphatase,phosphatase	ec:3.6.1.3, ec:3.6.1.15	map00230	Phvul.001G166000
**Comp2**	Pérola all samples of leaves vs BAT 477 all samples of leaves	U-R	Biosynthesis of antibiotics	O-acetyltransferase	ec:2.3.1.30	map01130	XLOC_024793
**Comp2**	Pérola all samples of leaves vs BAT 477 all samples of leaves	U-R	Diterpenoid biosynthesis	synthase	ec:4.2.3.8	map00904	Phvul.011G142600
**Comp2**	Pérola all samples of leaves vs BAT 477 all samples of leaves	U-R	Lysine degradation	decarboxylase	ec:4.1.1.18	map00310	Phvul.011G051300
**Comp2**	Pérola all samples of leaves vs BAT 477 all samples of leaves	U-R	Tropane, piperidine and pyridine alkaloid biosynthesis	decarboxylase	ec:4.1.1.18	map00960	Phvul.011G051300
**Comp2**	Pérola all samples of leaves vs BAT 477 all samples of leaves	U-R	Cysteine and methionine metabolism	O-acetyltransferase	ec:2.3.1.30	map00270	XLOC_024793
**Comp2**	Pérola all samples of leaves vs BAT 477 all samples of leaves	U-R	Starch and sucrose metabolism	synthase (UDP-forming)	ec:2.4.1.12	map00500	Phvul.005G116500
**Comp2**	Pérola all samples of leaves vs BAT 477 all samples of leaves	U-R	Fatty acid degradation	ligase	ec:6.2.1.3	map00071	Phvul.003G036600
**Comp2**	Pérola all samples of leaves vs BAT 477 all samples of leaves	U-R	Fatty acid biosynthesis	ligase	ec:6.2.1.3	map00061	Phvul.003G036600
**Comp2**	Pérola all samples of leaves vs BAT 477 all samples of leaves	U-R	Flavone and flavonol biosynthesis	3',5'-hydroxylase	ec:1.14.13.88	map00944	Phvul.006G054500
**Comp2**	Pérola all samples of leaves vs BAT 477 all samples of leaves	U-R	Flavonoid biosynthesis	3',5'-hydroxylase	ec:1.14.13.88	map00941	Phvul.006G054500
**Comp2**	Pérola all samples of leaves vs BAT 477 all samples of leaves	U-R	Thiamine metabolism	phosphatase	ec:3.6.1.15	map00730	Phvul.001G166000
**Comp3.1**	BAT 477, root in T0 vs BAT 477, root in T75	D-G	Drug metabolism - other enzymes	ali-esterase	ec:3.1.1.1	map00983	Phvul.003G126600
**Comp3.1**	BAT 477, root in T0 vs BAT 477, root in T75	D-G	Diterpenoid biosynthesis	3beta-dioxygenase,dioxygenase	ec:1.14.11.15,ec:1.14.11.12	map00904	Phvul.004G123600,Phvul.008G062700
**Comp3.1**	BAT 477, root in T0 vs BAT 477, root in T75	D-G	Starch and sucrose metabolism	1,4-alpha-galacturonidase,alpha-glucosidase,pectin demethoxylase,invertase,pectin depolymerase	ec:3.2.1.67,ec:3.2.1.48,ec:3.1.1.11,ec:3.2.1.26,ec:3.2.1.15	map00500	Phvul.007G211400,Phvul.002G061200,Phvul.003G126600
**Comp3.1**	BAT 477, root in T0 vs BAT 477, root in T75	D-G	Galactose metabolism	invertase	ec:3.2.1.26	map00052	Phvul.002G061200
**Comp3.1**	BAT 477, root in T0 vs BAT 477, root in T75	D-G	mTOR signaling pathway	protein kinase	ec:2.7.11.24	map04150	Phvul.004G045800
**Comp3.1**	BAT 477, root in T0 vs BAT 477, root in T75	D-G	Pentose and glucuronate interconversions	1,4-alpha-galacturonidase,pectin demethoxylase,pectin depolymerase	ec:3.2.1.67,ec:3.1.1.11,ec:3.2.1.15	map00040	Phvul.007G211400,Phvul.003G126600
**Comp3.1**	BAT 477, root in T0 vs BAT 477, root in T75	U-R	alpha-Linolenic acid metabolism	reductase,13S-lipoxygenase,A2	ec:1.3.1.42,ec:1.13.11.12,ec:3.1.1.4	map00592	Phvul.011G189300,Phvul.003G131500,Phvul.005G156700,Phvul.003G087500
**Comp3.1**	BAT 477, root in T0 vs BAT 477, root in T75	U-R	Biosynthesis of unsaturated fatty acids	9-desaturase	ec:1.14.19.1	map01040	Phvul.010G156200

**Comp3.1**	BAT 477, root in T0 vs BAT 477, root in T75	U-R	Linoleic acid metabolism	monooxygenase,13S-lipoxygenase,A2	ec:1.14.14.1,ec:1.13.11.12,ec:3.1.1.4	map00591	Phvul.002G014700,Phvul.005G156700,Phvul.003G087500
**Comp3.1**	BAT 477, root in T0 vs BAT 477, root in T75	U-R	Arachidonic acid metabolism	reductase (NADPH),monooxygenase,A2	ec:1.1.1.184,ec:1.14.14.1,ec:3.1.1.4	map00590	Phvul.008G068300,Phvul.002G014700,Phvul.003G087500
**Comp3.1**	BAT 477, root in T0 vs BAT 477, root in T75	U-R	Taurine and hypotaurine metabolism	decarboxylase	ec:4.1.1.15	map00430	Phvul.002G185800
**Comp3.1**	BAT 477, root in T0 vs BAT 477, root in T75	U-R	Sulfur metabolism	reductase (glutathione)	ec:1.8.4.9	map00920	Phvul.003G079800
**Comp3.1**	BAT 477, root in T0 vs BAT 477, root in T75	U-R	Drug metabolism - other enzymes	ali-esterase	ec:3.1.1.1	map00983	Phvul.003G217200,Phvul.003G087500,Phvul.003G243400,Phvul.001G209200,Phvul.007G210400, Phvul.011G197000,Phvul.005G016400
**Comp3.1**	BAT 477, root in T0 vs BAT 477, root in T75	U-R	Drug metabolism - cytochrome P450	monooxygenase,transferase	ec:1.14.14.1,ec:2.5.1.18	map00982	Phvul.002G014700,Phvul.005G054000,Phvul.008G113700
**Comp3.1**	BAT 477, root in T0 vs BAT 477, root in T75	U-R	Metabolism of xenobiotics by cytochrome P450	reductase (NADPH),monooxygenase,dehydrogenase,transferase	ec:1.1.1.184,ec:1.14.14.1,ec:1.3.1.20,ec:2.5.1.18	map00980	Phvul.008G068300,Phvul.002G014700,Phvul.004G134400,Phvul.005G054000,Phvul.008G113700
**Comp3.1**	BAT 477, root in T0 vs BAT 477, root in T75	U-R	Arginine and proline metabolism	deaminase	ec:3.5.4.1	map00330	Phvul.003G124100
**Comp3.1**	BAT 477, root in T0 vs BAT 477, root in T75	U-R	Caffeine metabolism	monooxygenase	ec:1.14.14.1	map00232	Phvul.002G014700
**Comp3.1**	BAT 477, root in T0 vs BAT 477, root in T75	U-R	Aminobenzoate degradation	phosphatase,monooxygenase,nitrophenyl phosphatase	ec:3.1.3.2,ec:1.14.14.1,ec:3.1.3.41	map00627	Phvul.010G144200,Phvul.010G144600,Phvul.002G014700,Phvul.007G198700,Phvul.009G054400
**Comp3.1**	BAT 477, root in T0 vs BAT 477, root in T75	U-R	Purine metabolism	adenylpyrophosphatase,phosphatase,dehydrogenase	ec:3.6.1.3,ec:3.6.1.15,ec:1.17.1.4	map00230	Phvul.009G155800,Phvul.001G187800,Phvul.005G173600,Phvul.003G099100

**Comp3.1**	BAT 477, root in T0 vs BAT 477, root in T75	U-R	Pyruvate metabolism	decarboxylase,lyase,dehydrogenase (decarboxylating), dehydrogenase (oxaloacetate-decarboxylating),dehydrogenase (oxaloacetate-decarboxylating) (NADP+)	ec:4.1.1.3, ec:4.4.1.5, ec:1.1.1.39,ec:1.1.1.38,ec:1.1.1.40	map00620	Phvul.009G111500,Phvul.009G108700
**Comp3.1**	BAT 477, root in T0 vs BAT 477, root in T75	U-R	Oxidative phosphorylation	reductase (H+-translocating),dehydrogenase	ec:1.6.5.3, ec:1.6.99.3	map00190	Phvul.002G223400
**Comp3.1**	BAT 477, root in T0 vs BAT 477, root in T75	U-R	Biosynthesis of antibiotics	phosphohexokinase	ec:2.7.1.11	map01130	Phvul.002G148700,Phvul.002G306800
**Comp3.1**	BAT 477, root in T0 vs BAT 477, root in T75	U-R	Pentose phosphate pathway	phosphohexokinase	ec:2.7.1.11	map00030	Phvul.002G148700,Phvul.002G306800
**Comp3.1**	BAT 477, root in T0 vs BAT 477, root in T75	U-R	Methane metabolism	phosphohexokinase	ec:2.7.1.11	map00680	Phvul.002G148700,Phvul.002G306800
**Comp3.1**	BAT 477, root in T0 vs BAT 477, root in T75	U-R	Amino sugar and nucleotide sugar metabolism	chitodextrinase	ec:3.2.1.14	map00520	Phvul.007G208900,Phvul.005G155800,Phvul.003G268600,Phvul.003G268500
**Comp3.1**	BAT 477, root in T0 vs BAT 477, root in T75	U-R	Glutathione metabolism	transferase	ec:2.5.1.18	map00480	Phvul.005G054000,Phvul.008G113700
**Comp3.1**	BAT 477, root in T0 vs BAT 477, root in T75	U-R	Tryptophan metabolism	monooxygenase	ec:1.14.14.1	map00380	Phvul.002G014700
**Comp3.1**	BAT 477, root in T0 vs BAT 477, root in T75	U-R	Carbon fixation in photosynthetic organisms	dehydrogenase (decarboxylating), dehydrogenase (oxaloacetate-decarboxylating) (NADP+)	ec:1.1.1.39,ec:1.1.1.40	map00710	Phvul.009G111500
**Comp3.1**	BAT 477, root in T0 vs BAT 477, root in T75	U-R	Carotenoid biosynthesis	desaturase,dioxygenase	ec:1.3.5.6, ec:1.13.11.51	map00906	Phvul.006G045800,Phvul.005G051600
**Comp3.1**	BAT 477, root in T0 vs BAT 477, root in T75	U-R	Diterpenoid biosynthesis	3beta-dioxygenase	ec:1.14.11.15	map00904	Phvul.002G232800,Phvul.002G049000
**Comp3.1**	BAT 477, root in T0 vs BAT 477, root in T75	U-R	beta-Alanine metabolism	decarboxylase	ec:4.1.1.15	map00410	Phvul.002G185800
**Comp3.1**	BAT 477, root in T0 vs BAT 477, root in T75	U-R	Terpenoid backbone biosynthesis	synthase	ec:4.2.3.27	map00900	Phvul.005G111700
**Comp3.1**	BAT 477, root in T0 vs BAT 477, root in T75	U-R	Cysteine and methionine metabolism	synthase,oxidase	ec:4.4.1.14,ec:1.14.17.4	map00270	Phvul.002G159800,Phvul.008G213900,Phvul.007G135600,Phvul.008G213800
**Comp3.1**	BAT 477, root in T0 vs BAT 477, root in T75	U-R	Glycolysis / Gluconeogenesis	phosphohexokinase	ec:2.7.1.11	map00010	Phvul.002G148700,Phvul

							.002G306800
**Comp3.1**	BAT 477, root in T0 vs BAT 477, root in T75	U-R	Starch and sucrose metabolism	alpha-glucosidase,trehalose 6-phosphatase,pectin demethoxylase,invertase,pectin depolymerase	ec:3.2.1.48,ec:3.1.3.12,ec:3.1.1.11,ec:3.2.1.26,ec:3.2.1.15	map00500	Phvul.005G158500,Phvul.009G054400,Phvul.003G217200,Phvul.001G209200,Phvul.007G210400,XLOC_025825
**Comp3.1**	BAT 477, root in T0 vs BAT 477, root in T75	U-R	Ether lipid metabolism	A2	ec:3.1.1.4	map00565	Phvul.003G087500
**Comp3.1**	BAT 477, root in T0 vs BAT 477, root in T75	U-R	Glycerophospholipid metabolism	decarboxylase,A2	ec:4.1.1.65,ec:3.1.1.4	map00564	Phvul.003G087500
**Comp3.1**	BAT 477, root in T0 vs BAT 477, root in T75	U-R	Fatty acid degradation	monooxygenase	ec:1.14.14.1	map00071	Phvul.002G014700
**Comp3.1**	BAT 477, root in T0 vs BAT 477, root in T75	U-R	Phenylalanine metabolism	decarboxylase,O-methyltransferase	ec:4.1.1.53,ec:2.1.1.104	map00360	Phvul.002G204900,Phvul.002G017500
**Comp3.1**	BAT 477, root in T0 vs BAT 477, root in T75	U-R	T cell receptor signaling pathway	phosphatase,protein-tyrosine kinase	ec:3.1.3.16,ec:2.7.10.2	map04660	Phvul.007G198700,Phvul.007G031000
**Comp3.1**	BAT 477, root in T0 vs BAT 477, root in T75	U-R	Butanoate metabolism	decarboxylase	ec:4.1.1.15	map00650	Phvul.002G185800
**Comp3.1**	BAT 477, root in T0 vs BAT 477, root in T75	U-R	Stilbenoid, diarylheptanoid and gingerol biosynthesis	O-methyltransferase	ec:2.1.1.104	map00945	Phvul.002G017500
**Comp3.1**	BAT 477, root in T0 vs BAT 477, root in T75	U-R	Flavonoid biosynthesis	O-methyltransferase,synthase,oxygenase,3-dioxygenase	ec:2.1.1.104,ec:1.14.11.23,ec:1.14.11.19,ec:1.14.11.9	map00941	Phvul.002G017500,Phvul.002G232800,Phvul.008G098500,Phvul.008G213900,Phvul.007G135600,Phvul.008G213800
**Comp3.1**	BAT 477, root in T0 vs BAT 477, root in T75	U-R	Phenylpropanoid biosynthesis	O-methyltransferase,lactoperoxidase	ec:2.1.1.104,ec:1.11.1.7	map00940	Phvul.002G017500,Phvul.006G075900,Phvul.006G075600
**Comp3.1**	BAT 477, root in T0 vs BAT 477, root in T75	U-R	Alanine, aspartate and glutamate metabolism	decarboxylase	ec:4.1.1.15	map00250	Phvul.002G185800
**Comp3.1**	BAT 477, root in T0 vs BAT 477, root in T75	U-R	Galactose metabolism	phosphohexokinase,invertase	ec:2.7.1.11,ec:3.2.1.26	map00052	Phvul.002G148700,Phvul.002G306800,Phvul.005G158500
**Comp3.1**	BAT 477, root in T0 vs BAT 477, root in T75	U-R	Fructose and mannose metabolism	phosphohexokinase	ec:2.7.1.11	map00051	Phvul.002G148700,Phvul.002G306800
**Comp3.1**	BAT 477, root in T0 vs BAT 477, root in T75	U-R	Histidine metabolism	decarboxylase	ec:4.1.1.22	map00340	Phvul.002G204900
**Comp3.1**	BAT 477, root in T0 vs BAT 477, root in T75	U-R	Retinol metabolism	monooxygenase	ec:1.14.14.1	map00830	Phvul.002G014700
**Comp3.1**	BAT 477, root in T0 vs BAT 477, root in T75	U-R	Pyrimidine metabolism	deaminase	ec:3.5.4.1	map00240	Phvul.003G124100

**Comp3.1**	BAT 477, root in T0 vs BAT 477, root in T75	U-R	Thiamine metabolism	phosphatase	ec:3.6.1.15	map00730	Phvul.009G155800,Phvul.001G187800,Phvul.005G173600
**Comp3.1**	BAT 477, root in T0 vs BAT 477, root in T75	U-R	Folate biosynthesis	hydrolase	ec:3.4.19.9	map00790	Phvul.005G080600
**Comp3.1**	BAT 477, root in T0 vs BAT 477, root in T75	U-R	Steroid hormone biosynthesis	monooxygenase	ec:1.14.14.1	map00140	Phvul.002G014700
**Comp3.1**	BAT 477, root in T0 vs BAT 477, root in T75	U-R	Pentose and glucuronate interconversions	pectin demethoxylase,pectin depolymerase	ec:3.1.1.11,ec:3.2.1.15	map00040	Phvul.003G217200,Phvul.001G209200,Phvul.007G210400,XLOC_025825
**Comp3.2**	BAT 477, root in T75 vs BAT 477, root in T150	D-G	Starch and sucrose metabolism	gentiobiase	ec:3.2.1.21	map00500	Phvul.001G076700
**Comp3.2**	BAT 477, root in T75 vs BAT 477, root in T150	D-G	Cyanoamino acid metabolism	gentiobiase	ec:3.2.1.21	map00460	Phvul.001G076700
**Comp3.2**	BAT 477, root in T75 vs BAT 477, root in T150	D-G	Phenylpropanoid biosynthesis	gentiobiase	ec:3.2.1.21	map00940	Phvul.001G076700
**Comp3.3**	BAT 477, root in T0 vs BAT 477, root in T150	D-G	alpha-Linolenic acid metabolism	dehydrogenase	ec:1.1.1.1	map00592	Phvul.008G089900
**Comp3.3**	BAT 477, root in T0 vs BAT 477, root in T150	D-G	Biosynthesis of unsaturated fatty acids	reductase	ec:1.1.1.100	map01040	Phvul.008G089900
**Comp3.3**	BAT 477, root in T0 vs BAT 477, root in T150	D-G	Drug metabolism - other enzymes	ali-esterase	ec:3.1.1.1	map00983	Phvul.003G126600,Phvul.002G308000,Phvul.002G270900,Phvul.004G175200
**Comp3.3**	BAT 477, root in T0 vs BAT 477, root in T150	D-G	Drug metabolism - cytochrome P450	dehydrogenase	ec:1.1.1.1	map00982	Phvul.008G089900
**Comp3.3**	BAT 477, root in T0 vs BAT 477, root in T150	D-G	Metabolism of xenobiotics by cytochrome P450	dehydrogenase	ec:1.1.1.1	map00980	Phvul.008G089900
**Comp3.3**	BAT 477, root in T0 vs BAT 477, root in T150	D-G	Aminobenzoate degradation	nitrophenyl phosphatase	ec:3.1.3.41	map00627	Phvul.003G228700,XLOC_000683,Phvul.002G181200,Phvul.007G276900,Phvul.007G067700
**Comp3.3**	BAT 477, root in T0 vs BAT 477, root in T150	D-G	Purine metabolism	adenylpyrophosphatase,phosphatase	ec:3.6.1.3,ec:3.6.1.15	map00230	Phvul.003G122700,Phvul.001G054500
**Comp3.3**	BAT 477, root in T0 vs BAT 477, root in T150	D-G	Naphthalene degradation	dehydrogenase	ec:1.1.1.1	map00626	Phvul.008G089900
**Comp3.3**	BAT 477, root in T0 vs BAT 477, root in T150	D-G	Chloroalkane and chloroalkene degradation	dehydrogenase	ec:1.1.1.1	map00625	Phvul.008G089900
**Comp3.3**	BAT 477, root in T0 vs BAT 477, root in T150	D-G	Biotin metabolism	reductase	ec:1.1.1.100	map00780	Phvul.008G089900
**Comp3.3**	BAT 477, root in T0 vs BAT 477, root in T150	D-G	Biosynthesis of antibiotics	aldolase,dehydrogenase	ec:4.1.2.13,ec:1.1.1.1	map01130	Phvul.007G222900,Phvul.008G089900
**Comp3.3**	BAT 477, root in T0 vs BAT 477, root in T150	D-G	Pentose phosphate pathway	aldolase	ec:4.1.2.13	map00030	Phvul.007G222900

**Comp3.3**	BAT 477, root in T0 vs BAT 477, root in T150	D-G	Methane metabolism	aldolase	ec:4.1.2.13	map00680	Phvul.007G222900
**Comp3.3**	BAT 477, root in T0 vs BAT 477, root in T150	D-G	Amino sugar and nucleotide sugar metabolism	chitodextrinase	ec:3.2.1.14	map00520	Phvul.006G109000
**Comp3.3**	BAT 477, root in T0 vs BAT 477, root in T150	D-G	Carbon fixation in photosynthetic organisms	aldolase	ec:4.1.2.13	map00710	Phvul.007G222900
**Comp3.3**	BAT 477, root in T0 vs BAT 477, root in T150	D-G	Zeatin biosynthesis	dimethylallyltransferase	ec:2.5.1.27	map00908	Phvul.003G093100
**Comp3.3**	BAT 477, root in T0 vs BAT 477, root in T150	D-G	Other glycan degradation	lactase (ambiguous)	ec:3.2.1.23	map00511	Phvul.011G077600
**Comp3.3**	BAT 477, root in T0 vs BAT 477, root in T150	D-G	Carotenoid biosynthesis	dehydrogenase	ec:1.1.1.288	map00906	Phvul.008G089900
**Comp3.3**	BAT 477, root in T0 vs BAT 477, root in T150	D-G	Diterpenoid biosynthesis	3beta-dioxygenase,dioxygenase	ec:1.14.11.15,ec:1.14.11.12	map00904	Phvul.007G248500,Phvul.004G123600,Phvul.007G248700
**Comp3.3**	BAT 477, root in T0 vs BAT 477, root in T150	D-G	Glycosphingolipid biosynthesis - ganglio series	lactase (ambiguous)	ec:3.2.1.23	map00604	Phvul.011G077600
**Comp3.3**	BAT 477, root in T0 vs BAT 477, root in T150	D-G	Cysteine and methionine metabolism	transaminase	ec:2.6.1.44	map00270	Phvul.008G087600
**Comp3.3**	BAT 477, root in T0 vs BAT 477, root in T150	D-G	Sphingolipid metabolism	lactase (ambiguous)	ec:3.2.1.23	map00600	Phvul.011G077600
**Comp3.3**	BAT 477, root in T0 vs BAT 477, root in T150	D-G	Glycolysis / Gluconeogenesis	aldolase,dehydrogenase	ec:4.1.2.13,ec:1.1.1.1	map00010	Phvul.007G222900,Phvul.008G089900
**Comp3.3**	BAT 477, root in T0 vs BAT 477, root in T150	D-G	Starch and sucrose metabolism	1,4-alpha-galacturonidase,alpha-glucosidase,pectin demethoxylase,invertase,pectin depolymerase	ec:3.2.1.67,ec:3.2.1.48,ec:3.1.1.11,ec:3.2.1.26,ec:3.2.1.15	map00500	Phvul.002G063500,Phvul.007G211400,Phvul.001G215900,Phvul.002G061200,Phvul.003G126600,Phvul.002G308000,Phvul.002G270900,Phvul.004G175200
**Comp3.3**	BAT 477, root in T0 vs BAT 477, root in T150	D-G	Ether lipid metabolism	D	ec:3.1.4.4	map00565	Phvul.010G139800
**Comp3.3**	BAT 477, root in T0 vs BAT 477, root in T150	D-G	Glycerophospholipid metabolism	D	ec:3.1.4.4	map00564	Phvul.010G139800
**Comp3.3**	BAT 477, root in T0 vs BAT 477, root in T150	D-G	Fatty acid degradation	dehydrogenase	ec:1.1.1.1	map00071	Phvul.008G089900
**Comp3.3**	BAT 477, root in T0 vs BAT 477, root in T150	D-G	Inositol phosphate metabolism	5-phosphatase	ec:3.1.3.36,ec:3.1.3.56	map00562	Phvul.003G228700,Phvul.002G181200
**Comp3.3**	BAT 477, root in T0 vs BAT 477, root in T150	D-G	Isoquinoline alkaloid biosynthesis	oxidase	ec:1.21.3.3	map00950	Phvul.007G113300
**Comp3.3**	BAT 477, root in T0 vs BAT 477, root in T150	D-G	Phenylalanine metabolism	ammonia-lyase	ec:4.3.1.24,ec:4.3.1.25	map00360	Phvul.007G096100
**Comp3.3**	BAT 477, root in T0 vs BAT 477, root in T150	D-G	T cell receptor signaling pathway	phosphatase	ec:3.1.3.16	map04660	XLOC_000683,Phvul.007G276900,Phvul.007G067700

**Comp3.3**	BAT 477, root in T0 vs BAT 477, root in T150	D-G	Glycine, serine and threonine metabolism	dehydrogenase,transaminase	ec:1.1.1.1, ec:2.6.1.44	map00260	Phvul.008G089900,Phvul.008G087600
**Comp3.3**	BAT 477, root in T0 vs BAT 477, root in T150	D-G	Phosphatidylinositol signaling system	5-phosphatase	ec:3.1.3.36,ec:3.1.3.56	map04070	Phvul.003G228700,Phvul.002G181200
**Comp3.3**	BAT 477, root in T0 vs BAT 477, root in T150	D-G	Fatty acid biosynthesis	synthase,reductase	ec:2.3.1.85,ec:1.1.1.100	map00061	Phvul.008G089900
**Comp3.3**	BAT 477, root in T0 vs BAT 477, root in T150	D-G	Flavone and flavonol biosynthesis	3'-monooxygenase,3',5'-hydroxylase	ec:1.14.13.21,ec:1.14.13.88	map00944	Phvul.007G257300,Phvul.006G054200
**Comp3.3**	BAT 477, root in T0 vs BAT 477, root in T150	D-G	Flavonoid biosynthesis	3'-monooxygenase,synthase,3-dioxygenase,3',5'-hydroxylase	ec:1.14.13.21,ec:1.14.11.23,ec:1.14.11.9, ec:1.14.13.88	map00941	Phvul.007G257300,Phvul.005G059100,Phvul.006G054200
**Comp3.3**	BAT 477, root in T0 vs BAT 477, root in T150	D-G	Phenylpropanoid biosynthesis	lactoperoxidase,ammonia-lyase,reductase	ec:1.11.1.7,ec:4.3.1.24,ec:4.3.1.25,ec:1.2.1.44	map00940	Phvul.001G201300,Phvul.007G114200,Phvul.007G096100,Phvul.011G022300
**Comp3.3**	BAT 477, root in T0 vs BAT 477, root in T150	D-G	Tyrosine metabolism	dehydrogenase	ec:1.1.1.1	map00350	Phvul.008G089900
**Comp3.3**	BAT 477, root in T0 vs BAT 477, root in T150	D-G	Alanine, aspartate and glutamate metabolism	transaminase	ec:2.6.1.44	map00250	Phvul.008G087600
**Comp3.3**	BAT 477, root in T0 vs BAT 477, root in T150	D-G	Galactose metabolism	invertase,lactase (ambiguous)	ec:3.2.1.26,ec:3.2.1.23	map00052	Phvul.002G061200,Phvul.011G077600
**Comp3.3**	BAT 477, root in T0 vs BAT 477, root in T150	D-G	Fructose and mannose metabolism	aldolase	ec:4.1.2.13	map00051	Phvul.007G222900
**Comp3.3**	BAT 477, root in T0 vs BAT 477, root in T150	D-G	mTOR signaling pathway	protein kinase	ec:2.7.11.24	map04150	Phvul.004G045800
**Comp3.3**	BAT 477, root in T0 vs BAT 477, root in T150	D-G	Retinol metabolism	dehydrogenase	ec:1.1.1.1	map00830	Phvul.008G089900
**Comp3.3**	BAT 477, root in T0 vs BAT 477, root in T150	D-G	Thiamine metabolism	phosphatase	ec:3.6.1.15	map00730	Phvul.003G122700,Phvul.001G054500
**Comp3.3**	BAT 477, root in T0 vs BAT 477, root in T150	D-G	Glycosaminoglycan degradation	lactase (ambiguous)	ec:3.2.1.23	map00531	Phvul.011G077600
**Comp3.3**	BAT 477, root in T0 vs BAT 477, root in T150	D-G	Pentose and glucuronate interconversions	1,4-alpha-galacturonidase,pectin demethoxylase,pectin depolymerase	ec:3.2.1.67,ec:3.1.1.11,ec:3.2.1.15	map00040	Phvul.002G063500,Phvul.007G211400,Phvul.001G215900,Phvul.003G126600,Phvul.002G308000, Phvul.002G270900,Phvul.004G175200

**Comp3.3**	BAT 477, root in T0 vs BAT 477, root in T150	U-R	alpha-Linolenic acid metabolism	dehydrogenase,reductase,13S-lipoxygenase,A2	ec:1.1.1.1,ec:1.3.1.42,ec:1.13.11.12,ec:3.1.1.4	map00592	Phvul.001G064000,Phvul.011G189200,Phvul.011G189300,Phvul.003G131500,Phvul.005G156700,Phvul.010G124000,Phvul.003G087500
**Comp3.3**	BAT 477, root in T0 vs BAT 477, root in T150	U-R	Biosynthesis of unsaturated fatty acids	9-desaturase	ec:1.14.19.1	map01040	Phvul.010G156200
**Comp3.3**	BAT 477, root in T0 vs BAT 477, root in T150	U-R	Linoleic acid metabolism	monooxygenase,13S-lipoxygenase,A2	ec:1.14.14.1,ec:1.13.11.12,ec:3.1.1.4	map00591	Phvul.002G014700,Phvul.005G156700,Phvul.010G124000,Phvul.003G087500
**Comp3.3**	BAT 477, root in T0 vs BAT 477, root in T150	U-R	Arachidonic acid metabolism	reductase (NADPH),monooxygenase,A2	ec:1.1.1.184,ec:1.14.14.1,ec:3.1.1.4	map00590	Phvul.008G068300,Phvul.002G014700,Phvul.003G087500
**Comp3.3**	BAT 477, root in T0 vs BAT 477, root in T150	U-R	Taurine and hypotaurine metabolism	decarboxylase	ec:4.1.1.15	map00430	Phvul.002G185800
**Comp3.3**	BAT 477, root in T0 vs BAT 477, root in T150	U-R	Sulfur metabolism	nucleotidase,gamma-synthase,reductase (glutathione)	ec:3.1.3.7,ec:2.5.1.48,ec:1.8.4.9	map00920	Phvul.006G190400,Phvul.004G090200,Phvul.003G079800
**Comp3.3**	BAT 477, root in T0 vs BAT 477, root in T150	U-R	Drug metabolism - other enzymes	synthase (glutamine-hydrolysing),ali-esterase,1-naphthol glucuronyltransferase	ec:6.3.5.2,ec:3.1.1.1,ec:2.4.1.17	map00983	Phvul.008G008100,Phvul.003G217200,Phvul.003G087500,Phvul.003G243400,Phvul.001G207500,Phvul.002G332300,Phvul.001G209200,Phvul.007G210400,Phvul.011G197000,Phvul.005G016400,Phvul.003G015600,Phvul.003G027000,Phvul.007G164400

**Comp3.3**	BAT 477, root in T0 vs BAT 477, root in T150	U-R	Drug metabolism - cytochrome P450	dehydrogenase,monooxygenase,transferase,1-naphthol glucuronyltransferase	ec:1.1.1.1,ec:1.14.14.1,ec:2.5.1.18,ec:2.4.1.17	map00982	Phvul.001G064000,Phvul.002G014700,Phvul.005G053900,Phvul.002G241400,Phvul.004G070500,Phvul.005G054100,Phvul.001G173300,Phvul.005G054200,Phvul.005G054000,Phvul.008G113700,Phvul.006G079600,Phvul.007G164400
**Comp3.3**	BAT 477, root in T0 vs BAT 477, root in T150	U-R	Metabolism of xenobiotics by cytochrome P450	reductase (NADPH),dehydrogenase,monooxygenase,transferase,1-naphthol glucuronyltransferase	ec:1.1.1.184,ec:1.1.1.1,ec:1.14.14.1,ec:1.3.1.20,ec:2.5.1.18,ec:2.4.1.17	map00980	Phvul.008G068300,Phvul.001G064000,Phvul.002G014700,Phvul.004G134400,Phvul.005G053900,Phvul.002G241400,Phvul.004G070500,Phvul.005G054100,Phvul.001G173300,Phvul.005G054200,Phvul.005G054000,Phvul.008G113700,Phvul.006G079600,Phvul.007G164400
**Comp3.3**	BAT 477, root in T0 vs BAT 477, root in T150	U-R	Arginine and proline metabolism	deaminase	ec:3.5.4.1	map00330	Phvul.003G124100
**Comp3.3**	BAT 477, root in T0 vs BAT 477, root in T150	U-R	Caffeine metabolism	monooxygenase	ec:1.14.14.1	map00232	Phvul.002G014700
**Comp3.3**	BAT 477, root in T0 vs BAT 477, root in T150	U-R	Aminobenzoate degradation	phosphatase,monooxygenase,nitrophenyl phosphatase	ec:3.1.3.2,ec:1.14.14.1,ec:3.1.3.41	map00627	Phvul.010G144200,Phvul.010G144600,Phvul.002G014700,Phvul.004G152900,Phvul.007G192700,Phvul.007G198700,Phvul.007G118300,Phvul.006G190400,Phvul.009G054400,Phvul.002G163900,Phvul.001G075400

**Comp3.3**	BAT 477, root in T0 vs BAT 477, root in T150	U-R	Purine metabolism	adenylpyrophosphatase,phosphatase,RNA polymerase,synthase (glutamine-hydrolysing),dehydrogenase	ec:3.6.1.3,ec:3.6.1.15,ec:2.7.7.6,ec:6.3.5.2,ec:1.17.1.4	map00230	Phvul.003G024700,Phvul.007G062500,Phvul.002G210400,Phvul.002G210500,Phvul.009G155800,Phvul.004G046200,Phvul.001G206700,Phvul.005G053000,Phvul.001G265800,Phvul.L011500,Phvul.001G187800,Phvul.001G165700,Phvul.005G173600,XLOC_016155,Phvul.005G029700,Phvul.008G008100,Phvul.003G099100
**Comp3.3**	BAT 477, root in T0 vs BAT 477, root in T150	U-R	Naphthalene degradation	dehydrogenase,1-monooxygenase	ec:1.1.1.1,ec:1.14.13.1	map00626	Phvul.001G064000,Phvul.007G018700
**Comp3.3**	BAT 477, root in T0 vs BAT 477, root in T150	U-R	Chloroalkane and chloroalkene degradation	dehydrogenase	ec:1.1.1.1	map00625	Phvul.001G064000
**Comp3.3**	BAT 477, root in T0 vs BAT 477, root in T150	U-R	Polycyclic aromatic hydrocarbon degradation	1-monooxygenase	ec:1.14.13.1	map00624	Phvul.007G018700
**Comp3.3**	BAT 477, root in T0 vs BAT 477, root in T150	U-R	Valine, leucine and isoleucine biosynthesis	synthase	ec:2.3.3.13	map00290	Phvul.001G091600
**Comp3.3**	BAT 477, root in T0 vs BAT 477, root in T150	U-R	Dioxin degradation	1-monooxygenase	ec:1.14.13.1	map00621	Phvul.007G018700
**Comp3.3**	BAT 477, root in T0 vs BAT 477, root in T150	U-R	Ubiquinone and other terpenoid-quinone biosynthesis	ligase	ec:6.2.1.26,ec:6.2.1.12	map00130	Phvul.004G114600,Phvul.006G079500
**Comp3.3**	BAT 477, root in T0 vs BAT 477, root in T150	U-R	Pyruvate metabolism	synthase,decarboxylase,lyase,dehydrogenase (decarboxylating), dehydrogenase (oxaloacetate-decarboxylating),dehydrogenase (oxaloacetate-decarboxylating) (NADP+)	ec:2.3.3.13,ec:4.1.1.3,ec:4.4.1.5,ec:1.1.1.39,ec:1.1.1.38,ec:1.1.1.40	map00620	Phvul.001G091600,Phvul.009G111500,Phvul.005G053900,Phvul.009G108700
**Comp3.3**	BAT 477, root in T0 vs BAT 477, root in T150	U-R	Oxidative phosphorylation	reductase (H+-translocating),dehydrogenase	ec:1.6.5.3,ec:1.6.99.3	map00190	Phvul.002G223400
**Comp3.3**	BAT 477, root in T0 vs BAT 477, root in T150	U-R	Biosynthesis of antibiotics	dehydrogenase,gamma-synthase,phosphohexokinase	ec:1.1.1.1,ec:2.5.1.48,ec:2.7.1.11	map01130	Phvul.001G064000,Phvul.004G090200,Phvul.002G148700,Ph

							vul.002G306800
**Comp3.3**	BAT 477, root in T0 vs BAT 477, root in T150	U-R	Pentose phosphate pathway	phosphohexokinase	ec:2.7.1.11	map00030	Phvul.002G148700,Phvul.002G306800
**Comp3.3**	BAT 477, root in T0 vs BAT 477, root in T150	U-R	Methane metabolism	phosphohexokinase,dehydrogenase	ec:2.7.1.11,ec:1.1.1.284	map00680	Phvul.002G148700,Phvul.002G306800,Phvul.008G207900,Phvul.001G064000
**Comp3.3**	BAT 477, root in T0 vs BAT 477, root in T150	U-R	Amino sugar and nucleotide sugar metabolism	chitodextrinase	ec:3.2.1.14	map00520	Phvul.007G208900,Phvul.005G155800,Phvul.003G268600,Phvul.003G268500
**Comp3.3**	BAT 477, root in T0 vs BAT 477, root in T150	U-R	Glutathione metabolism	transferase	ec:2.5.1.18	map00480	Phvul.005G053900,Phvul.002G241400,Phvul.004G070500,Phvul.005G054100,Phvul.001G173300,Phvul.005G054200,Phvul.005G054000,Phvul.008G113700,Phvul.006G079600
**Comp3.3**	BAT 477, root in T0 vs BAT 477, root in T150	U-R	Tryptophan metabolism	monooxygenase,S-beta-glucosyltransferase	ec:1.14.14.1,ec:2.4.1.195	map00380	Phvul.002G014700,Phvul.002G075200
**Comp3.3**	BAT 477, root in T0 vs BAT 477, root in T150	U-R	Carbon fixation in photosynthetic organisms	dehydrogenase (decarboxylating), dehydrogenase (oxaloacetate-decarboxylating) (NADP+)	ec:1.1.1.39,ec:1.1.1.40	map00710	Phvul.009G111500
**Comp3.3**	BAT 477, root in T0 vs BAT 477, root in T150	U-R	Other types of O-glycan biosynthesis	3-beta-glucuronosyltransferase	ec:2.4.1.135	map00514	Phvul.007G164400
**Comp3.3**	BAT 477, root in T0 vs BAT 477, root in T150	U-R	Other glycan degradation	lactase (ambiguous)	ec:3.2.1.23	map00511	Phvul.009G012700
**Comp3.3**	BAT 477, root in T0 vs BAT 477, root in T150	U-R	Carotenoid biosynthesis	desaturase,dioxygenase,epoxidase	ec:1.3.5.6,ec:1.13.11.51,ec:1.14.13.90	map00906	Phvul.006G045800,Phvul.005G051600,Phvul.007G018700
**Comp3.3**	BAT 477, root in T0 vs BAT 477, root in T150	U-R	Diterpenoid biosynthesis	diphosphate synthase,3beta-dioxygenase,2beta-dioxygenase	ec:5.5.1.13,ec:1.14.11.15,ec:1.14.11.13	map00904	Phvul.001G152100,Phvul.001G151900,Phvul.002G232800,Phvul.002G049000,Phvul.003G118300

**Comp3.3**	BAT 477, root in T0 vs BAT 477, root in T150	U-R	beta-Alanine metabolism	decarboxylase	ec:4.1.1.15	map00410	Phvul.002G185800
**Comp3.3**	BAT 477, root in T0 vs BAT 477, root in T150	U-R	Glucosinolate biosynthesis	S-beta-glucosyltransferase	ec:2.4.1.195	map00966	Phvul.002G075200
**Comp3.3**	BAT 477, root in T0 vs BAT 477, root in T150	U-R	Terpenoid backbone biosynthesis	synthase	ec:4.2.3.27	map00900	Phvul.005G111700
**Comp3.3**	BAT 477, root in T0 vs BAT 477, root in T150	U-R	Porphyrin and chlorophyll metabolism	1-naphthol glucuronyltransferase	ec:2.4.1.17	map00860	Phvul.007G164400
**Comp3.3**	BAT 477, root in T0 vs BAT 477, root in T150	U-R	Glycosphingolipid biosynthesis - ganglio series	lactase (ambiguous)	ec:3.2.1.23	map00604	Phvul.009G012700
**Comp3.3**	BAT 477, root in T0 vs BAT 477, root in T150	U-R	Cysteine and methionine metabolism	synthase,gamma-lyase,gamma-synthase,oxidase	ec:4.4.1.14,ec:4.4.1.11,ec:2.5.1.48,ec:1.14.17.4	map00270	Phvul.002G159800,Phvul.008G058400,Phvul.010G103900,Phvul.004G090200,Phvul.002G326600,Phvul.008G213900,Phvul.007G135600,Phvul.008G213800,Phvul.008G214200
**Comp3.3**	BAT 477, root in T0 vs BAT 477, root in T150	U-R	Sphingolipid metabolism	lactase (ambiguous)	ec:3.2.1.23	map00600	Phvul.009G012700
**Comp3.3**	BAT 477, root in T0 vs BAT 477, root in T150	U-R	Glycolysis / Gluconeogenesis	dehydrogenase,phosphohexokinase	ec:1.1.1.1,ec:2.7.1.11	map00010	Phvul.001G064000,Phvul.002G148700,Phvul.002G306800
**Comp3.3**	BAT 477, root in T0 vs BAT 477, root in T150	U-R	Starch and sucrose metabolism	saccharogen amylase,alpha-glucosidase,trehalose 6-phosphatase,gentiobiase,pectin demethoxylase,invertase,1-naphthol glucuronyltransferase,synthase (UDP-forming),pectin depolymerase	ec:3.2.1.2,ec:3.2.1.48,ec:3.1.3.12,ec:3.2.1.21,ec:3.1.1.11,ec:3.2.1.26,ec:2.4.1.17,ec:2.4.1.12,ec:3.2.1.15	map00500	Phvul.004G143600,Phvul.005G158500,Phvul.009G054400,Phvul.005G151600,Phvul.003G217200,Phvul.001G209200,Phvul.003G015600,Phvul.003G027000,Phvul.007G210400,Phvul.007G164400,Phvul.006G058600,Phvul.005G022500,XLOC_025825
**Comp3.3**	BAT 477, root in T0 vs BAT 477, root in T150	U-R	Ether lipid metabolism	A2	ec:3.1.1.4	map00565	Phvul.003G087500
**Comp3.3**	BAT 477, root in T0 vs BAT 477, root in T150	U-R	Glycerophospholipid metabolism	decarboxylase,1-O-acyltransferase,A2	ec:4.1.1.65,ec:2.3.1.15,ec:3.1.1.4	map00564	Phvul.003G087500,Phvul.008G191600
**Comp3.3**	BAT 477, root in T0 vs BAT 477, root in T150	U-R	Cutin, suberine and wax biosynthesis	O-acyltransferase	ec:2.3.1.20	map00073	Phvul.008G042300

**Comp3.3**	BAT 477, root in T0 vs BAT 477, root in T150	U-R	Fatty acid degradation	dehydrogenase,monooxygenase	ec:1.1.1.1,ec:1.14.14.1	map00071	Phvul.001G064000,Phvul.002G014700
**Comp3.3**	BAT 477, root in T0 vs BAT 477, root in T150	U-R	Inositol phosphate metabolism	phospholipase C	ec:3.1.4.11	map00562	Phvul.008G224100
**Comp3.3**	BAT 477, root in T0 vs BAT 477, root in T150	U-R	Glycerolipid metabolism	1-O-acyltransferase,lipase,O-acyltransferase	ec:2.3.1.15,ec:3.1.1.23,ec:2.3.1.20	map00561	Phvul.008G191600,Phvul.001G207500,Phvul.002G332300,Phvul.008G042300
**Comp3.3**	BAT 477, root in T0 vs BAT 477, root in T150	U-R	Cyanoamino acid metabolism	lyase,gentiobiase	ec:4.1.2.10,ec:3.2.1.21	map00460	Phvul.010G162900,Phvul.005G151600
**Comp3.3**	BAT 477, root in T0 vs BAT 477, root in T150	U-R	Phenylalanine metabolism	decarboxylase,O-methyltransferase,ligase	ec:4.1.1.53,ec:2.1.1.104,ec:6.2.1.12	map00360	Phvul.002G204900,Phvul.002G017500,Phvul.004G114600,Phvul.006G079500
**Comp3.3**	BAT 477, root in T0 vs BAT 477, root in T150	U-R	T cell receptor signaling pathway	phosphatase,protein-tyrosine kinase	ec:3.1.3.16,ec:2.7.10.2	map04660	Phvul.004G152900,Phvul.007G198700,Phvul.002G163900,Phvul.001G075400,Phvul.007G118300, Phvul.011G194400,Phvul.005G055000,Phvul.007G031000
**Comp3.3**	BAT 477, root in T0 vs BAT 477, root in T150	U-R	Glycine, serine and threonine metabolism	dehydrogenase	ec:1.1.1.1	map00260	Phvul.001G064000
**Comp3.3**	BAT 477, root in T0 vs BAT 477, root in T150	U-R	Phosphatidylinositol signaling system	phospholipase C	ec:3.1.4.11	map04070	Phvul.008G224100
**Comp3.3**	BAT 477, root in T0 vs BAT 477, root in T150	U-R	Butanoate metabolism	decarboxylase	ec:4.1.1.15	map00650	Phvul.002G185800
**Comp3.3**	BAT 477, root in T0 vs BAT 477, root in T150	U-R	Stilbenoid, diarylheptanoid and gingerol biosynthesis	O-methyltransferase,O-hydroxycinnamoyltransferase	ec:2.1.1.104,ec:2.3.1.133	map00945	Phvul.002G017500,Phvul.008G031900,Phvul.006G023900,Phvul.001G112400,Phvul.009G066900, Phvul.005G109000,Phvul.009G066300
**Comp3.3**	BAT 477, root in T0 vs BAT 477, root in T150	U-R	Isoflavonoid biosynthesis	reductase,2'-hydroxylase	ec:1.3.1.45,ec:1.14.13.89	map00943	Phvul.002G033100,Phvul.004G068400
**Comp3.3**	BAT 477, root in T0 vs BAT 477, root in T150	U-R	Selenocompound metabolism	gamma-lyase,gamma-synthase	ec:4.4.1.11,ec:2.5.1.48	map00450	Phvul.004G090200

**Comp3.3**	BAT 477, root in T0 vs BAT 477, root in T150	U-R	Flavonoid biosynthesis	O-methyltransferase,synthase,oxygenase,4-reductase,3-dioxygenase,O-hydroxycinnamoyltransferase	ec:2.1.1.104,ec:1.14.11.23,ec:1.14.11.19,ec:1.1.1.219,ec:1.14.11.9,ec:2.3.1.133	map00941	Phvul.002G017500,Phvul.002G232800,Phvul.008G098500,Phvul.010G000300,Phvul.010G160700, Phvul.002G326600,Phvul.008G213900,Phvul.007G135600,Phvul.008G213800,Phvul.008G214200, Phvul.008G031900,Phvul.006G023900,Phvul.001G112400,Phvul.009G066900,Phvul.005G109000, Phvul.009G066300
**Comp3.3**	BAT 477, root in T0 vs BAT 477, root in T150	U-R	Phenylpropanoid biosynthesis	dehydrogenase,O-methyltransferase,lactoperoxidase,reductase,O-hydroxycinnamoyltransferase,gentiobiase,ligase	ec:1.1.1.195,ec:2.1.1.104,ec:1.11.1.7,ec:1.2.1.44,ec:2.3.1.133,ec:3.2.1.21,ec:6.2.1.12	map00940	Phvul.003G287500,Phvul.002G017500,Phvul.009G140700,Phvul.006G075900,Phvul.011G128200, Phvul.008G249500,Phvul.006G075600,Phvul.010G160700,Phvul.008G031900,Phvul.006G023900, Phvul.001G112400,Phvul.009G066900,Phvul.005G109000,Phvul.009G066300,Phvul.005G151600, Phvul.004G114600,Phvul.006G079500
**Comp3.3**	BAT 477, root in T0 vs BAT 477, root in T150	U-R	Tyrosine metabolism	dehydrogenase	ec:1.1.1.1	map00350	Phvul.001G064000
**Comp3.3**	BAT 477, root in T0 vs BAT 477, root in T150	U-R	Alanine, aspartate and glutamate metabolism	synthase (glutamine-hydrolysing),decarboxylase	ec:6.3.5.5, ec:4.1.1.15	map00250	Phvul.009G027700,Phvul.011G072200,Phvul.002G185800
**Comp3.3**	BAT 477, root in T0 vs BAT 477, root in T150	U-R	Ascorbate and aldarate metabolism	1-naphthol glucuronyltransferase	ec:2.4.1.17	map00053	Phvul.007G164400

**Comp3.3**	BAT 477, root in T0 vs BAT 477, root in T150	U-R	Galactose metabolism	phosphohexokinase,3-alpha-galactosyltransferase,invertase,lactase (ambiguous)	ec:2.7.1.11,ec:2.4.1.123,ec:3.2.1.26,ec:3.2.1.23	map00052	Phvul.002G148700,Phvul.002G306800,Phvul.007G203400,Phvul.005G158500,Phvul.009G012700
**Comp3.3**	BAT 477, root in T0 vs BAT 477, root in T150	U-R	Fructose and mannose metabolism	phosphohexokinase	ec:2.7.1.11	map00051	Phvul.002G148700,Phvul.002G306800
**Comp3.3**	BAT 477, root in T0 vs BAT 477, root in T150	U-R	Histidine metabolism	decarboxylase	ec:4.1.1.22	map00340	Phvul.002G204900
**Comp3.3**	BAT 477, root in T0 vs BAT 477, root in T150	U-R	mTOR signaling pathway	protein kinase	ec:2.7.11.24	map04150	Phvul.001G029500
**Comp3.3**	BAT 477, root in T0 vs BAT 477, root in T150	U-R	Retinol metabolism	dehydrogenase,monooxygenase,1-naphthol glucuronyltransferase	ec:1.1.1.1,ec:1.14.14.1,ec:2.4.1.17	map00830	Phvul.001G064000,Phvul.002G014700,Phvul.007G164400
**Comp3.3**	BAT 477, root in T0 vs BAT 477, root in T150	U-R	Pyrimidine metabolism	deaminase,RNA polymerase,synthase (glutamine-hydrolysing)	ec:3.5.4.1,ec:2.7.7.6,ec:6.3.5.5	map00240	Phvul.003G124100,Phvul.005G029700,Phvul.009G027700,Phvul.011G072200
**Comp3.3**	BAT 477, root in T0 vs BAT 477, root in T150	U-R	Thiamine metabolism	phosphatase	ec:3.6.1.15	map00730	Phvul.003G024700,Phvul.007G062500,XLOC_016155,Phvul.002G210400,Phvul.002G210500,Phvul.009G155800,Phvul.004G046200,Phvul.001G206700,Phvul.005G053000,Phvul.001G265800,Phvul.L011500,Phvul.001G187800,Phvul.001G165700,Phvul.005G173600
**Comp3.3**	BAT 477, root in T0 vs BAT 477, root in T150	U-R	Folate biosynthesis	hydrolase	ec:3.4.19.9	map00790	Phvul.005G080600
**Comp3.3**	BAT 477, root in T0 vs BAT 477, root in T150	U-R	Steroid hormone biosynthesis	monooxygenase,1-naphthol glucuronyltransferase	ec:1.14.14.1,ec:2.4.1.17	map00140	Phvul.002G014700,Phvul.007G164400
**Comp3.3**	BAT 477, root in T0 vs BAT 477, root in T150	U-R	Glyoxylate and dicarboxylate metabolism	ligase	ec:6.2.1.8	map00630	Phvul.004G114600,Phvul.006G079500
**Comp3.3**	BAT 477, root in T0 vs BAT 477, root in T150	U-R	Glycosaminoglycan biosynthesis - heparan sulfate / heparin	3-beta-glucuronosyltransferase	ec:2.4.1.135	map00534	Phvul.007G164400

**Comp3.3**	BAT 477, root in T0 vs BAT 477, root in T150	U-R	Glycosaminoglycan biosynthesis - chondroitin sulfate / dermatan sulfate	3-beta-glucuronosyltransferase	ec:2.4.1.135	map00532	Phvul.007G164400
**Comp3.3**	BAT 477, root in T0 vs BAT 477, root in T150	U-R	Glycosaminoglycan degradation	lactase (ambiguous)	ec:3.2.1.23	map00531	Phvul.009G012700
**Comp3.3**	BAT 477, root in T0 vs BAT 477, root in T150	U-R	Pentose and glucuronate interconversions	pectin demethoxylase,1-naphthol glucuronyltransferase,pectin depolymerase	ec:3.1.1.11,ec:2.4.1.17,ec:3.2.1.15	map00040	Phvul.003G217200,Phvul.001G209200,Phvul.003G015600,Phvul.003G027000,Phvul.007G210400,Phvul.007G164400,Phvul.005G022500,XLOC_025825
**Comp4.1**	Pérola, root in T0 vs Pérola, root in T75	D-G	Linoleic acid metabolism	monooxygenase	ec:1.14.14.1	map00591	Phvul.002G187800
**Comp4.1**	Pérola, root in T0 vs Pérola, root in T75	D-G	Arachidonic acid metabolism	monooxygenase	ec:1.14.14.1	map00590	Phvul.002G187800
**Comp4.1**	Pérola, root in T0 vs Pérola, root in T75	D-G	Drug metabolism - cytochrome P450	monooxygenase	ec:1.14.14.1	map00982	Phvul.002G187800
**Comp4.1**	Pérola, root in T0 vs Pérola, root in T75	D-G	Metabolism of xenobiotics by cytochrome P450	monooxygenase	ec:1.14.14.1	map00980	Phvul.002G187800
**Comp4.1**	Pérola, root in T0 vs Pérola, root in T75	D-G	Caffeine metabolism	monooxygenase	ec:1.14.14.1	map00232	Phvul.002G187800
**Comp4.1**	Pérola, root in T0 vs Pérola, root in T75	D-G	Aminobenzoate degradation	monooxygenase	ec:1.14.14.1	map00627	Phvul.002G187800
**Comp4.1**	Pérola, root in T0 vs Pérola, root in T75	D-G	Tryptophan metabolism	monooxygenase	ec:1.14.14.1	map00380	Phvul.002G187800
**Comp4.1**	Pérola, root in T0 vs Pérola, root in T75	D-G	Starch and sucrose metabolism	1,4-alpha-galacturonidase,alpha-glucosidase,invertase,pectin depolymerase	ec:3.2.1.67,ec:3.2.1.48,ec:3.2.1.26,ec:3.2.1.15	map00500	Phvul.007G211400,Phvul.002G061200
**Comp4.1**	Pérola, root in T0 vs Pérola, root in T75	D-G	Fatty acid degradation	monooxygenase	ec:1.14.14.1	map00071	Phvul.002G187800
**Comp4.1**	Pérola, root in T0 vs Pérola, root in T75	D-G	Flavone and flavonol biosynthesis	3'-monooxygenase	ec:1.14.13.21	map00944	Phvul.007G257300
**Comp4.1**	Pérola, root in T0 vs Pérola, root in T75	D-G	Isoflavonoid biosynthesis	2'-hydroxylase	ec:1.14.13.89	map00943	Phvul.002G187800
**Comp4.1**	Pérola, root in T0 vs Pérola, root in T75	D-G	Flavonoid biosynthesis	3'-monooxygenase	ec:1.14.13.21	map00941	Phvul.007G257300
**Comp4.1**	Pérola, root in T0 vs Pérola, root in T75	D-G	Galactose metabolism	invertase	ec:3.2.1.26	map00052	Phvul.002G061200
**Comp4.1**	Pérola, root in T0 vs Pérola, root in T75	D-G	Retinol metabolism	monooxygenase	ec:1.14.14.1	map00830	Phvul.002G187800
**Comp4.1**	Pérola, root in T0 vs Pérola, root in T75	D-G	Steroid hormone biosynthesis	monooxygenase	ec:1.14.14.1	map00140	Phvul.002G187800
**Comp4.1**	Pérola, root in T0 vs Pérola, root in T75	D-G	Pentose and glucuronate interconversions	1,4-alpha-galacturonidase,pectin depolymerase	ec:3.2.1.67,ec:3.2.1.15	map00040	Phvul.007G211400
**Comp4.1**	Pérola, root in T0 vs Pérola, root in T75	U-R	alpha-Linolenic acid metabolism	reductase,13S-lipoxygenase	ec:1.3.1.42,ec:1.13.11.12	map00592	Phvul.011G189300,Phvul.003G13150

							0,Phvul.010G124000
**Comp4.1**	Pérola, root in T0 vs Pérola, root in T75	U-R	Linoleic acid metabolism	monooxygenase,13S-lipoxygenase	ec:1.14.14.1,ec:1.13.11.12	map00591	Phvul.002G014700,Phvul.010G124000
**Comp4.1**	Pérola, root in T0 vs Pérola, root in T75	U-R	Arachidonic acid metabolism	monooxygenase	ec:1.14.14.1	map00590	Phvul.002G014700
**Comp4.1**	Pérola, root in T0 vs Pérola, root in T75	U-R	Taurine and hypotaurine metabolism	decarboxylase	ec:4.1.1.15	map00430	Phvul.002G185800
**Comp4.1**	Pérola, root in T0 vs Pérola, root in T75	U-R	Sulfur metabolism	sulfurtransferase	ec:2.8.1.1	map00920	Phvul.002G112600
**Comp4.1**	Pérola, root in T0 vs Pérola, root in T75	U-R	Drug metabolism - other enzymes	synthase (glutamine-hydrolysing),ali-esterase	ec:6.3.5.2,ec:3.1.1.1	map00983	Phvul.008G008100,Phvul.007G210400
**Comp4.1**	Pérola, root in T0 vs Pérola, root in T75	U-R	Drug metabolism - cytochrome P450	monooxygenase,transferase	ec:1.14.14.1,ec:2.5.1.18	map00982	Phvul.002G014700,Phvul.005G054000
**Comp4.1**	Pérola, root in T0 vs Pérola, root in T75	U-R	Metabolism of xenobiotics by cytochrome P450	monooxygenase,transferase	ec:1.14.14.1,ec:2.5.1.18	map00980	Phvul.002G014700,Phvul.005G054000
**Comp4.1**	Pérola, root in T0 vs Pérola, root in T75	U-R	Arginine and proline metabolism	deaminase	ec:3.5.4.1	map00330	Phvul.003G124100
**Comp4.1**	Pérola, root in T0 vs Pérola, root in T75	U-R	Caffeine metabolism	monooxygenase	ec:1.14.14.1	map00232	Phvul.002G014700
**Comp4.1**	Pérola, root in T0 vs Pérola, root in T75	U-R	Aminobenzoate degradation	phosphatase,monooxygenase,nitrophenyl phosphatase	ec:3.1.3.2,ec:1.14.14.1,ec:3.1.3.41	map00627	Phvul.010G144200,Phvul.010G144600,Phvul.002G014700
**Comp4.1**	Pérola, root in T0 vs Pérola, root in T75	U-R	Purine metabolism	synthase (glutamine-hydrolysing)	ec:6.3.5.2	map00230	Phvul.008G008100
**Comp4.1**	Pérola, root in T0 vs Pérola, root in T75	U-R	Amino sugar and nucleotide sugar metabolism	chitodextrinase	ec:3.2.1.14	map00520	Phvul.005G155800,Phvul.003G268500
**Comp4.1**	Pérola, root in T0 vs Pérola, root in T75	U-R	Glutathione metabolism	transferase	ec:2.5.1.18	map00480	Phvul.005G054000
**Comp4.1**	Pérola, root in T0 vs Pérola, root in T75	U-R	Tryptophan metabolism	monooxygenase	ec:1.14.14.1	map00380	Phvul.002G014700
**Comp4.1**	Pérola, root in T0 vs Pérola, root in T75	U-R	Carotenoid biosynthesis	desaturase,dioxygenase	ec:1.3.5.6,ec:1.13.11.51	map00906	Phvul.006G045800,Phvul.005G051600
**Comp4.1**	Pérola, root in T0 vs Pérola, root in T75	U-R	Diterpenoid biosynthesis	3beta-dioxygenase	ec:1.14.11.15	map00904	Phvul.002G049000
**Comp4.1**	Pérola, root in T0 vs Pérola, root in T75	U-R	beta-Alanine metabolism	decarboxylase	ec:4.1.1.15	map00410	Phvul.002G185800
**Comp4.1**	Pérola, root in T0 vs Pérola, root in T75	U-R	Terpenoid backbone biosynthesis	synthase	ec:4.2.3.27	map00900	Phvul.005G111700
**Comp4.1**	Pérola, root in T0 vs Pérola, root in T75	U-R	Cysteine and methionine metabolism	synthase,oxidase	ec:4.4.1.14,ec:1.14.17.4	map00270	Phvul.002G159800,Phvul.007G135600
**Comp4.1**	Pérola, root in T0 vs Pérola, root in T75	U-R	Starch and sucrose metabolism	pectin demethoxylase,pectin depolymerase	ec:3.1.1.11,ec:3.2.1.15	map00500	Phvul.007G210400,XLOC_025825

**Comp4.1**	Pérola, root in T0 vs Pérola, root in T75	U-R	Fatty acid degradation	monooxygenase	ec:1.14.14.1	map00071	Phvul.002G014700
**Comp4.1**	Pérola, root in T0 vs Pérola, root in T75	U-R	T cell receptor signaling pathway	protein-tyrosine kinase	ec:2.7.10.2	map04660	Phvul.007G031000
**Comp4.1**	Pérola, root in T0 vs Pérola, root in T75	U-R	Butanoate metabolism	decarboxylase	ec:4.1.1.15	map00650	Phvul.002G185800
**Comp4.1**	Pérola, root in T0 vs Pérola, root in T75	U-R	Isoflavonoid biosynthesis	reductase	ec:1.3.1.45	map00943	Phvul.002G033100
**Comp4.1**	Pérola, root in T0 vs Pérola, root in T75	U-R	Flavonoid biosynthesis	oxygenase,3-dioxygenase	ec:1.14.11.19,ec:1.14.11.9	map00941	Phvul.008G098500,Phvul.007G135600
**Comp4.1**	Pérola, root in T0 vs Pérola, root in T75	U-R	Phenylpropanoid biosynthesis	lactoperoxidase	ec:1.11.1.7	map00940	Phvul.001G076000,Phvul.006G075600
**Comp4.1**	Pérola, root in T0 vs Pérola, root in T75	U-R	Alanine, aspartate and glutamate metabolism	decarboxylase	ec:4.1.1.15	map00250	Phvul.002G185800
**Comp4.1**	Pérola, root in T0 vs Pérola, root in T75	U-R	Retinol metabolism	monooxygenase	ec:1.14.14.1	map00830	Phvul.002G014700
**Comp4.1**	Pérola, root in T0 vs Pérola, root in T75	U-R	Pyrimidine metabolism	deaminase	ec:3.5.4.1	map00240	Phvul.003G124100
**Comp4.1**	Pérola, root in T0 vs Pérola, root in T75	U-R	Steroid hormone biosynthesis	monooxygenase	ec:1.14.14.1	map00140	Phvul.002G014700
**Comp4.1**	Pérola, root in T0 vs Pérola, root in T75	U-R	Pentose and glucuronate interconversions	pectin demethoxylase,pectin depolymerase	ec:3.1.1.11,ec:3.2.1.15	map00040	Phvul.007G210400,XLOC_025825
**Comp4.2**	Pérola, root in T75 vs Pérola, root in T150	D-G	Purine metabolism	RNA polymerase	ec:2.7.7.6	map00230	XLOC_020681
**Comp4.2**	Pérola, root in T75 vs Pérola, root in T150	D-G	Various types of N-glycan biosynthesis	1,2-alpha-mannosidase	ec:3.2.1.113	map00513	Phvul.006G033000
**Comp4.2**	Pérola, root in T75 vs Pérola, root in T150	D-G	Other glycan degradation	alpha-D-mannosidase	ec:3.2.1.24	map00511	Phvul.006G033000
**Comp4.2**	Pérola, root in T75 vs Pérola, root in T150	D-G	N-Glycan biosynthesis	1,2-alpha-mannosidase	ec:3.2.1.113	map00510	Phvul.006G033000
**Comp4.2**	Pérola, root in T75 vs Pérola, root in T150	D-G	Carotenoid biosynthesis	desaturase	ec:1.3.5.6	map00906	Phvul.010G124800
**Comp4.2**	Pérola, root in T75 vs Pérola, root in T150	D-G	T cell receptor signaling pathway	protein-tyrosine kinase	ec:2.7.10.2	map04660	XLOC_017321
**Comp4.2**	Pérola, root in T75 vs Pérola, root in T150	D-G	Pyrimidine metabolism	RNA polymerase	ec:2.7.7.6	map00240	XLOC_020681
**Comp4.3**	Pérola, root in T0 vs Pérola, root in T150	D-G	Linoleic acid metabolism	monooxygenase	ec:1.14.14.1	map00591	Phvul.001G037100
**Comp4.3**	Pérola, root in T0 vs Pérola, root in T150	D-G	Arachidonic acid metabolism	monooxygenase	ec:1.14.14.1	map00590	Phvul.001G037100
**Comp4.3**	Pérola, root in T0 vs Pérola, root in T150	D-G	Drug metabolism - cytochrome P450	monooxygenase	ec:1.14.14.1	map00982	Phvul.001G037100
**Comp4.3**	Pérola, root in T0 vs Pérola, root in T150	D-G	Metabolism of xenobiotics by cytochrome P450	monooxygenase	ec:1.14.14.1	map00980	Phvul.001G037100
**Comp4.3**	Pérola, root in T0 vs Pérola, root in T150	D-G	Caffeine metabolism	monooxygenase	ec:1.14.14.1	map00232	Phvul.001G037100
**Comp4.3**	Pérola, root in T0 vs Pérola, root in T150	D-G	Aminobenzoate degradation	monooxygenase	ec:1.14.14.1	map00627	Phvul.001G037100
**Comp4.3**	Pérola, root in T0 vs Pérola, root in T150	D-G	Purine metabolism	RNA polymerase	ec:2.7.7.6	map00230	XLOC_020681
**Comp4.3**	Pérola, root in T0 vs Pérola, root in T150	D-G	Tryptophan metabolism	monooxygenase	ec:1.14.14.1	map00380	Phvul.001G037100
**Comp4.3**	Pérola, root in T0 vs Pérola, root in T150	D-G	Various types of N-glycan biosynthesis	1,2-alpha-mannosidase	ec:3.2.1.113	map00513	Phvul.006G033000

**Comp4.3**	Pérola, root in T0 vs Pérola, root in T150	D-G	Other glycan degradation	alpha-D-mannosidase	ec:3.2.1.24	map00511	Phvul.006G033000
**Comp4.3**	Pérola, root in T0 vs Pérola, root in T150	D-G	N-Glycan biosynthesis	1,2-alpha-mannosidase	ec:3.2.1.113	map00510	Phvul.006G033000
**Comp4.3**	Pérola, root in T0 vs Pérola, root in T150	D-G	Carotenoid biosynthesis	desaturase	ec:1.3.5.6	map00906	Phvul.010G124800
**Comp4.3**	Pérola, root in T0 vs Pérola, root in T150	D-G	Starch and sucrose metabolism	alpha-glucosidase,invertase	ec:3.2.1.48,ec:3.2.1.26	map00500	Phvul.002G061200
**Comp4.3**	Pérola, root in T0 vs Pérola, root in T150	D-G	Fatty acid degradation	monooxygenase	ec:1.14.14.1	map00071	Phvul.001G037100
**Comp4.3**	Pérola, root in T0 vs Pérola, root in T150	D-G	Flavone and flavonol biosynthesis	3'-monooxygenase	ec:1.14.13.21	map00944	Phvul.007G257300
**Comp4.3**	Pérola, root in T0 vs Pérola, root in T150	D-G	Flavonoid biosynthesis	3'-monooxygenase	ec:1.14.13.21	map00941	Phvul.007G257300
**Comp4.3**	Pérola, root in T0 vs Pérola, root in T150	D-G	Galactose metabolism	invertase	ec:3.2.1.26	map00052	Phvul.002G061200
**Comp4.3**	Pérola, root in T0 vs Pérola, root in T150	D-G	mTOR signaling pathway	protein kinase	ec:2.7.11.24	map04150	Phvul.004G045800
**Comp4.3**	Pérola, root in T0 vs Pérola, root in T150	D-G	Retinol metabolism	monooxygenase	ec:1.14.14.1	map00830	Phvul.001G037100
**Comp4.3**	Pérola, root in T0 vs Pérola, root in T150	D-G	Pyrimidine metabolism	RNA polymerase	ec:2.7.7.6	map00240	XLOC_020681
**Comp4.3**	Pérola, root in T0 vs Pérola, root in T150	D-G	Steroid hormone biosynthesis	monooxygenase	ec:1.14.14.1	map00140	Phvul.001G037100
**Comp4.3**	Pérola, root in T0 vs Pérola, root in T150	U-R	alpha-Linolenic acid metabolism	reductase,13S-lipoxygenase	ec:1.3.1.42,ec:1.13.11.12	map00592	Phvul.011G189200,Phvul.011G189300,Phvul.003G131500,Phvul.005G156700,Phvul.010G124000
**Comp4.3**	Pérola, root in T0 vs Pérola, root in T150	U-R	Biosynthesis of unsaturated fatty acids	9-desaturase	ec:1.14.19.1	map01040	Phvul.010G156200
**Comp4.3**	Pérola, root in T0 vs Pérola, root in T150	U-R	Linoleic acid metabolism	monooxygenase,13S-lipoxygenase	ec:1.14.14.1,ec:1.13.11.12	map00591	Phvul.002G014700,Phvul.005G156700,Phvul.010G124000
**Comp4.3**	Pérola, root in T0 vs Pérola, root in T150	U-R	Arachidonic acid metabolism	reductase (NADPH),monooxygenase	ec:1.1.1.184,ec:1.14.14.1	map00590	Phvul.008G068300,Phvul.002G014700
**Comp4.3**	Pérola, root in T0 vs Pérola, root in T150	U-R	Taurine and hypotaurine metabolism	decarboxylase	ec:4.1.1.15	map00430	Phvul.002G185800,Phvul.002G186000
**Comp4.3**	Pérola, root in T0 vs Pérola, root in T150	U-R	Sulfur metabolism	reductase (glutathione)	ec:1.8.4.9	map00920	Phvul.003G079800
**Comp4.3**	Pérola, root in T0 vs Pérola, root in T150	U-R	Drug metabolism - other enzymes	synthase (glutamine-hydrolysing),ali-esterase	ec:6.3.5.2, ec:3.1.1.1	map00983	Phvul.008G008100,Phvul.003G217200,Phvul.003G243400,Phvul.005G012000,Phvul.001G209200,Phvul.007G210400,Phvul.011G197000,Phvul.007G246700

**Comp4.3**	Pérola, root in T0 vs Pérola, root in T150	U-R	Drug metabolism - cytochrome P450	monooxygenase,transferase	ec:1.14.14.1,ec:2.5.1.18	map00982	Phvul.002G014700,Phvul.005G054000
**Comp4.3**	Pérola, root in T0 vs Pérola, root in T150	U-R	Metabolism of xenobiotics by cytochrome P450	reductase (NADPH),monooxygenase,dehydrogenase,transferase	ec:1.1.1.184,ec:1.14.14.1,ec:1.3.1.20,ec:2.5.1.18	map00980	Phvul.008G068300,Phvul.002G014700,Phvul.004G134400,Phvul.005G054000
**Comp4.3**	Pérola, root in T0 vs Pérola, root in T150	U-R	Arginine and proline metabolism	deaminase	ec:3.5.4.1	map00330	Phvul.003G124100
**Comp4.3**	Pérola, root in T0 vs Pérola, root in T150	U-R	Caffeine metabolism	monooxygenase	ec:1.14.14.1	map00232	Phvul.002G014700
**Comp4.3**	Pérola, root in T0 vs Pérola, root in T150	U-R	Aminobenzoate degradation	phosphatase,monooxygenase,nitrophenyl phosphatase	ec:3.1.3.2,ec:1.14.14.1,ec:3.1.3.41	map00627	Phvul.010G144200,Phvul.010G144600,Phvul.002G014700,Phvul.004G152900,Phvul.007G198700,Phvul.007G118300,Phvul.009G054400
**Comp4.3**	Pérola, root in T0 vs Pérola, root in T150	U-R	Purine metabolism	adenylpyrophosphatase,phosphatase,RNA polymerase,synthase (glutamine-hydrolysing),dehydrogenase	ec:3.6.1.3,ec:3.6.1.15,ec:2.7.7.6,ec:6.3.5.2,ec:1.17.1.4	map00230	Phvul.001G265800,Phvul.001G187800,Phvul.001G165700,Phvul.005G173600,Phvul.005G029700,Phvul.008G008100,XLOC_008379
**Comp4.3**	Pérola, root in T0 vs Pérola, root in T150	U-R	Pyruvate metabolism	decarboxylase,lyase,dehydrogenase (decarboxylating),dehydrogenase (oxaloacetate-decarboxylating),dehydrogenase (oxaloacetate-decarboxylating) (NADP+)	ec:4.1.1.3,ec:4.4.1.5,ec:1.1.1.39,ec:1.1.1.38,ec:1.1.1.40	map00620	Phvul.009G111500,Phvul.009G108700
**Comp4.3**	Pérola, root in T0 vs Pérola, root in T150	U-R	Oxidative phosphorylation	reductase (H+-translocating),dehydrogenase	ec:1.6.5.3,ec:1.6.99.3	map00190	Phvul.002G223400
**Comp4.3**	Pérola, root in T0 vs Pérola, root in T150	U-R	Biosynthesis of antibiotics	synthase	ec:4.2.1.20,ec:2.2.1.7	map01130	Phvul.002G048100,Phvul.003G148900
**Comp4.3**	Pérola, root in T0 vs Pérola, root in T150	U-R	Amino sugar and nucleotide sugar metabolism	chitodextrinase	ec:3.2.1.14	map00520	Phvul.007G208900,Phvul.005G155800,Phvul.003G268500
**Comp4.3**	Pérola, root in T0 vs Pérola, root in T150	U-R	Glutathione metabolism	transferase	ec:2.5.1.18	map00480	Phvul.005G054000
**Comp4.3**	Pérola, root in T0 vs Pérola, root in T150	U-R	Tryptophan metabolism	monooxygenase	ec:1.14.14.1	map00380	Phvul.002G014700

**Comp4.3**	Pérola, root in T0 vs Pérola, root in T150	U-R	Carbon fixation in photosynthetic organisms	dehydrogenase (decarboxylating), dehydrogenase (oxaloacetate-decarboxylating) (NADP+)	ec:1.1.1.39,ec:1.1.1.40	map00710	Phvul.009G111500
**Comp4.3**	Pérola, root in T0 vs Pérola, root in T150	U-R	Zeatin biosynthesis	dehydrogenase	ec:1.5.99.12	map00908	Phvul.011G014000
**Comp4.3**	Pérola, root in T0 vs Pérola, root in T150	U-R	Carotenoid biosynthesis	desaturase,dioxygenase	ec:1.3.5.6,ec:1.13.11.51	map00906	Phvul.006G045800,Phvul.005G051600
**Comp4.3**	Pérola, root in T0 vs Pérola, root in T150	U-R	Diterpenoid biosynthesis	3beta-dioxygenase	ec:1.14.11.15	map00904	Phvul.002G232800,Phvul.002G049000
**Comp4.3**	Pérola, root in T0 vs Pérola, root in T150	U-R	beta-Alanine metabolism	decarboxylase	ec:4.1.1.15	map00410	Phvul.002G185800,Phvul.002G186000
**Comp4.3**	Pérola, root in T0 vs Pérola, root in T150	U-R	Terpenoid backbone biosynthesis	synthase	ec:2.2.1.7,ec:4.2.3.27	map00900	Phvul.003G148900,Phvul.005G111500,Phvul.005G111400,Phvul.005G111700
**Comp4.3**	Pérola, root in T0 vs Pérola, root in T150	U-R	Cysteine and methionine metabolism	synthase,oxidase	ec:4.4.1.14,ec:1.14.17.4	map00270	Phvul.002G159800,Phvul.008G058400,Phvul.008G213900,Phvul.007G135600,Phvul.008G213800
**Comp4.3**	Pérola, root in T0 vs Pérola, root in T150	U-R	Starch and sucrose metabolism	alpha-glucosidase,trehalose 6-phosphatase,gentiobiase,pectin demethoxylase,invertase,pectin depolymerase	ec:3.2.1.48,ec:3.1.3.12,ec:3.2.1.21,ec:3.1.1.11,ec:3.2.1.26,ec:3.2.1.15	map00500	Phvul.005G158500,Phvul.009G054400,Phvul.011G055900,Phvul.011G056100,Phvul.011G201700,Phvul.003G217200,Phvul.005G012000,Phvul.007G246700,Phvul.001G209200,Phvul.007G210400,XLOC_025825
**Comp4.3**	Pérola, root in T0 vs Pérola, root in T150	U-R	Fatty acid degradation	monooxygenase	ec:1.14.14.1	map00071	Phvul.002G014700
**Comp4.3**	Pérola, root in T0 vs Pérola, root in T150	U-R	Inositol phosphate metabolism	phospholipase C,oxygenase	ec:3.1.4.11,ec:1.13.99.1	map00562	Phvul.008G224100,Phvul.002G306400
**Comp4.3**	Pérola, root in T0 vs Pérola, root in T150	U-R	Phenylalanine, tyrosine and tryptophan biosynthesis	synthase	ec:4.2.1.20	map00400	Phvul.002G048100
**Comp4.3**	Pérola, root in T0 vs Pérola, root in T150	U-R	Cyanoamino acid metabolism	gentiobiase	ec:3.2.1.21	map00460	Phvul.011G055900,Phvul.011G05610

							0,Phvul.011G201700
**Comp4.3**	Pérola, root in T0 vs Pérola, root in T150	U-R	Phenylalanine metabolism	decarboxylase	ec:4.1.1.53	map00360	Phvul.002G204900
**Comp4.3**	Pérola, root in T0 vs Pérola, root in T150	U-R	T cell receptor signaling pathway	phosphatase,protein-tyrosine kinase	ec:3.1.3.16,ec:2.7.10.2	map04660	Phvul.004G152900,Phvul.007G198700,Phvul.007G118300,Phvul.011G194400,Phvul.005G055000,Phvul.007G031000
**Comp4.3**	Pérola, root in T0 vs Pérola, root in T150	U-R	Glycine, serine and threonine metabolism	synthase	ec:4.2.1.20	map00260	Phvul.002G048100
**Comp4.3**	Pérola, root in T0 vs Pérola, root in T150	U-R	Phosphatidylinositol signaling system	phospholipase C	ec:3.1.4.11	map04070	Phvul.008G224100
**Comp4.3**	Pérola, root in T0 vs Pérola, root in T150	U-R	Butanoate metabolism	decarboxylase	ec:4.1.1.15	map00650	Phvul.002G185800,Phvul.002G186000
**Comp4.3**	Pérola, root in T0 vs Pérola, root in T150	U-R	Stilbenoid, diarylheptanoid and gingerol biosynthesis	O-hydroxycinnamoyltransferase	ec:2.3.1.133	map00945	Phvul.006G023900,Phvul.009G066900
**Comp4.3**	Pérola, root in T0 vs Pérola, root in T150	U-R	Isoflavonoid biosynthesis	reductase,2'-hydroxylase	ec:1.3.1.45,ec:1.14.13.89	map00943	Phvul.002G033100,Phvul.004G068400,Phvul.010G013100
**Comp4.3**	Pérola, root in T0 vs Pérola, root in T150	U-R	Flavonoid biosynthesis	synthase,oxygenase,3-dioxygenase,O-hydroxycinnamoyltransferase	ec:1.14.11.23,ec:1.14.11.19,ec:1.14.11.9,ec:2.3.1.133	map00941	Phvul.002G232800,Phvul.008G098500,Phvul.010G000300,Phvul.008G213900,Phvul.007G135600,Phvul.008G213800,Phvul.006G023900,Phvul.009G066900
**Comp4.3**	Pérola, root in T0 vs Pérola, root in T150	U-R	Phenylpropanoid biosynthesis	lactoperoxidase,O-hydroxycinnamoyltransferase,gentiobiase	ec:1.11.1.7,ec:2.3.1.133,ec:3.2.1.21	map00940	Phvul.001G076000,Phvul.006G075900,Phvul.011G030200,Phvul.006G075600,Phvul.006G023900,Phvul.009G066900,Phvul.011G055900,Phvul.011G056100,Phvul.011G201700
**Comp4.3**	Pérola, root in T0 vs Pérola, root in T150	U-R	Alanine, aspartate and glutamate metabolism	decarboxylase	ec:4.1.1.15	map00250	Phvul.002G185800,Phvul.002G186000
**Comp4.3**	Pérola, root in T0 vs Pérola, root in T150	U-R	Ascorbate and aldarate metabolism	oxidase,oxygenase	ec:1.10.3.3,ec:1.13.99.1	map00053	Phvul.006G011600,Phvul.002G306400

**Comp4.3**	Pérola, root in T0 vs Pérola, root in T150	U-R	Galactose metabolism	galactosyltransferase,invertase	ec:2.4.1.67,ec:3.2.1.26	map00052	Phvul.008G282600,Phvul.005G158500
**Comp4.3**	Pérola, root in T0 vs Pérola, root in T150	U-R	Histidine metabolism	decarboxylase	ec:4.1.1.22	map00340	Phvul.002G204900
**Comp4.3**	Pérola, root in T0 vs Pérola, root in T150	U-R	Retinol metabolism	monooxygenase	ec:1.14.14.1	map00830	Phvul.002G014700
**Comp4.3**	Pérola, root in T0 vs Pérola, root in T150	U-R	Pyrimidine metabolism	deaminase,RNA polymerase	ec:3.5.4.1, ec:2.7.7.6	map00240	Phvul.003G124100,Phvul.005G029700
**Comp4.3**	Pérola, root in T0 vs Pérola, root in T150	U-R	Thiamine metabolism	synthase,phosphatase	ec:2.2.1.7, ec:3.6.1.15	map00730	Phvul.003G148900,Phvul.001G265800,Phvul.001G187800,Phvul.001G165700,Phvul.005G173600
**Comp4.3**	Pérola, root in T0 vs Pérola, root in T150	U-R	Steroid hormone biosynthesis	monooxygenase	ec:1.14.14.1	map00140	Phvul.002G014700
**Comp4.3**	Pérola, root in T0 vs Pérola, root in T150	U-R	Pentose and glucuronate interconversions	pectin demethoxylase,pectin depolymerase	ec:3.1.1.11,ec:3.2.1.15	map00040	Phvul.003G217200,Phvul.005G012000,Phvul.007G246700,Phvul.001G209200,Phvul.007G210400, XLOC_025825
**Comp5.2**	BAT 477, leaves in T75 vs BAT 477, leaves in T150	D-G	Sulfur metabolism	gamma-synthase,reductase (glutathione)	ec:2.5.1.48,ec:1.8.4.9	map00920	Phvul.004G090200,Phvul.003G079800
**Comp5.2**	BAT 477, leaves in T75 vs BAT 477, leaves in T150	D-G	Biosynthesis of antibiotics	gamma-synthase	ec:2.5.1.48	map01130	Phvul.004G090200
**Comp5.2**	BAT 477, leaves in T75 vs BAT 477, leaves in T150	D-G	Methane metabolism	dehydrogenase	ec:1.1.1.284	map00680	Phvul.008G207900
**Comp5.2**	BAT 477, leaves in T75 vs BAT 477, leaves in T150	D-G	Cysteine and methionine metabolism	gamma-lyase,gamma-synthase	ec:4.4.1.11,ec:2.5.1.48	map00270	Phvul.004G090200
**Comp5.2**	BAT 477, leaves in T75 vs BAT 477, leaves in T150	D-G	Isoquinoline alkaloid biosynthesis	oxidase	ec:1.10.3.1	map00950	Phvul.006G188500
**Comp5.2**	BAT 477, leaves in T75 vs BAT 477, leaves in T150	D-G	Selenocompound metabolism	gamma-lyase,gamma-synthase	ec:4.4.1.11,ec:2.5.1.48	map00450	Phvul.004G090200
**Comp5.2**	BAT 477, leaves in T75 vs BAT 477, leaves in T150	D-G	Tyrosine metabolism	oxidase	ec:1.10.3.1	map00350	Phvul.006G188500
**Comp5.3**	BAT 477, leaves in T0 vs BAT 477, leaves in T150	D-G	Aminobenzoate degradation	nitrophenyl phosphatase	ec:3.1.3.41	map00627	Phvul.010G140800
**Comp5.3**	BAT 477, leaves in T0 vs BAT 477, leaves in T150	D-G	Purine metabolism	RNA polymerase	ec:2.7.7.6	map00230	Phvul.008G020100
**Comp5.3**	BAT 477, leaves in T0 vs BAT 477, leaves in T150	D-G	Nitrogen metabolism	reductase (NADH),reductase (NADPH),reductase	ec:1.7.1.1, ec:1.7.1.3, ec:1.7.99.4	map00910	Phvul.009G121000
**Comp5.3**	BAT 477, leaves in T0 vs BAT 477, leaves in T150	D-G	Carotenoid biosynthesis	desaturase	ec:1.3.5.6	map00906	Phvul.009G018300

**Comp5.3**	BAT 477, leaves in T0 vs BAT 477, leaves in T150	D-G	Diterpenoid biosynthesis	diphosphate synthase,3beta-dioxygenase,dioxygenase	ec:5.5.1.13,ec:1.14.11.15,ec:1.14.11.12	map00904	Phvul.001G152100,Phvul.004G123600
**Comp5.3**	BAT 477, leaves in T0 vs BAT 477, leaves in T150	D-G	Starch and sucrose metabolism	synthase (UDP-forming)	ec:2.4.1.12	map00500	Phvul.008G279600
**Comp5.3**	BAT 477, leaves in T0 vs BAT 477, leaves in T150	D-G	Isoquinoline alkaloid biosynthesis	oxidase	ec:1.10.3.1	map00950	Phvul.008G073200
**Comp5.3**	BAT 477, leaves in T0 vs BAT 477, leaves in T150	D-G	Vitamin B6 metabolism	phosphatase	ec:3.1.3.74	map00750	Phvul.010G140800
**Comp5.3**	BAT 477, leaves in T0 vs BAT 477, leaves in T150	D-G	Stilbenoid, diarylheptanoid and gingerol biosynthesis	O-hydroxycinnamoyl transferase	ec:2.3.1.133	map00945	Phvul.006G023900
**Comp5.3**	BAT 477, leaves in T0 vs BAT 477, leaves in T150	D-G	Flavonoid biosynthesis	O-hydroxycinnamoyl transferase	ec:2.3.1.133	map00941	Phvul.006G023900
**Comp5.3**	BAT 477, leaves in T0 vs BAT 477, leaves in T150	D-G	Phenylpropanoid biosynthesis	O-hydroxycinnamoyl transferase	ec:2.3.1.133	map00940	Phvul.006G023900
**Comp5.3**	BAT 477, leaves in T0 vs BAT 477, leaves in T150	D-G	Tyrosine metabolism	oxidase	ec:1.10.3.1	map00350	Phvul.008G073200
**Comp5.3**	BAT 477, leaves in T0 vs BAT 477, leaves in T150	D-G	Pyrimidine metabolism	RNA polymerase	ec:2.7.7.6	map00240	Phvul.008G020100
**Comp5.3**	BAT 477, leaves in T0 vs BAT 477, leaves in T150	U-R	alpha-Linolenic acid metabolism	dehydrogenase,reductase	ec:1.1.1.1,ec:1.3.1.42	map00592	Phvul.001G064000,Phvul.002G043000
**Comp5.3**	BAT 477, leaves in T0 vs BAT 477, leaves in T150	U-R	Sulfur metabolism	gamma-synthase,reductase (glutathione)	ec:2.5.1.48,ec:1.8.4.9	map00920	Phvul.004G090200,Phvul.003G079800
**Comp5.3**	BAT 477, leaves in T0 vs BAT 477, leaves in T150	U-R	Drug metabolism - other enzymes	ali-esterase	ec:3.1.1.1	map00983	Phvul.003G217200,Phvul.002G332300
**Comp5.3**	BAT 477, leaves in T0 vs BAT 477, leaves in T150	U-R	Drug metabolism - cytochrome P450	dehydrogenase,transferase	ec:1.1.1.1,ec:2.5.1.18	map00982	Phvul.001G064000,Phvul.005G054200,Phvul.006G079600
**Comp5.3**	BAT 477, leaves in T0 vs BAT 477, leaves in T150	U-R	Metabolism of xenobiotics by cytochrome P450	dehydrogenase,transferase	ec:1.1.1.1,ec:2.5.1.18	map00980	Phvul.001G064000,Phvul.005G054200,Phvul.006G079600
**Comp5.3**	BAT 477, leaves in T0 vs BAT 477, leaves in T150	U-R	Arginine and proline metabolism	dehydrogenase (NAD+),dehydrogenase	ec:1.2.1.3,ec:1.2.1.19	map00330	Phvul.009G182300
**Comp5.3**	BAT 477, leaves in T0 vs BAT 477, leaves in T150	U-R	Aminobenzoate degradation	nitrophenyl phosphatase	ec:3.1.3.41	map00627	Phvul.002G163900,Phvul.001G075400,Phvul.002G309100
**Comp5.3**	BAT 477, leaves in T0 vs BAT 477, leaves in T150	U-R	Purine metabolism	adenylpyrophosphatase,phosphatase	ec:3.6.1.3,ec:3.6.1.15	map00230	Phvul.008G048400,Phvul.004G046200
**Comp5.3**	BAT 477, leaves in T0 vs BAT 477, leaves in T150	U-R	Naphthalene degradation	dehydrogenase	ec:1.1.1.1	map00626	Phvul.001G064000
**Comp5.3**	BAT 477, leaves in T0 vs BAT 477, leaves in T150	U-R	Chloroalkane and chloroalkene degradation	dehydrogenase,dehydrogenase (NAD+)	ec:1.1.1.1,ec:1.2.1.3	map00625	Phvul.001G064000,Phvul.009G182300

**Comp5.3**	BAT 477, leaves in T0 vs BAT 477, leaves in T150	U-R	Ubiquinone and other terpenoid-quinone biosynthesis	ligase	ec:6.2.1.26,ec:6.2.1.12	map00130	Phvul.006G079500,Phvul.011G084300
**Comp5.3**	BAT 477, leaves in T0 vs BAT 477, leaves in T150	U-R	Pyruvate metabolism	dehydrogenase (NAD+),synthase	ec:1.2.1.3, ec:2.3.3.9	map00620	Phvul.009G182300,Phvul.003G209100
**Comp5.3**	BAT 477, leaves in T0 vs BAT 477, leaves in T150	U-R	Biosynthesis of antibiotics	dehydrogenase (NADP+),dehydrogenase,dehydrogenase (NADP+, L-glutamate-forming),gamma-synthase,dehydrogenase (NAD+),dehydrogenase (NADP+, L-lysine-forming)	ec:1.1.1.2, ec:1.1.1.1, ec:1.5.1.10,ec:2.5.1.48,ec:1.2.1.3,ec:1.5.1.8	map01130	Phvul.008G287200,Phvul.001G064000,Phvul.003G126300,Phvul.004G090200,Phvul.009G182300
**Comp5.3**	BAT 477, leaves in T0 vs BAT 477, leaves in T150	U-R	Methane metabolism	dehydrogenase	ec:1.2.1.2, ec:1.1.1.284	map00680	Phvul.004G014700,Phvul.008G207900,Phvul.001G064000
**Comp5.3**	BAT 477, leaves in T0 vs BAT 477, leaves in T150	U-R	Glutathione metabolism	transferase	ec:2.5.1.18	map00480	Phvul.005G054200,Phvul.006G079600
**Comp5.3**	BAT 477, leaves in T0 vs BAT 477, leaves in T150	U-R	Tryptophan metabolism	dehydrogenase (NAD+)	ec:1.2.1.3	map00380	Phvul.009G182300
**Comp5.3**	BAT 477, leaves in T0 vs BAT 477, leaves in T150	U-R	Valine, leucine and isoleucine degradation	dehydrogenase (NAD+)	ec:1.2.1.3	map00280	Phvul.009G182300
**Comp5.3**	BAT 477, leaves in T0 vs BAT 477, leaves in T150	U-R	One carbon pool by folate	formimidoyltransferase	ec:2.1.2.5	map00670	Phvul.003G044600
**Comp5.3**	BAT 477, leaves in T0 vs BAT 477, leaves in T150	U-R	Carotenoid biosynthesis	dioxygenase	ec:1.13.11.51	map00906	Phvul.005G051600
**Comp5.3**	BAT 477, leaves in T0 vs BAT 477, leaves in T150	U-R	Diterpenoid biosynthesis	3beta-dioxygenase	ec:1.14.11.15	map00904	Phvul.002G232800
**Comp5.3**	BAT 477, leaves in T0 vs BAT 477, leaves in T150	U-R	Limonene and pinene degradation	dehydrogenase (NAD+)	ec:1.2.1.3	map00903	Phvul.009G182300
**Comp5.3**	BAT 477, leaves in T0 vs BAT 477, leaves in T150	U-R	beta-Alanine metabolism	dehydrogenase (NAD+),dehydrogenase	ec:1.2.1.3, ec:1.2.1.19	map00410	Phvul.009G182300
**Comp5.3**	BAT 477, leaves in T0 vs BAT 477, leaves in T150	U-R	Lysine degradation	dehydrogenase (NADP+, L-glutamate-forming),dehydrogenase (NAD+),dehydrogenase (NADP+, L-lysine-forming)	ec:1.5.1.10,ec:1.2.1.3,ec:1.5.1.8	map00310	Phvul.003G126300,Phvul.009G182300
**Comp5.3**	BAT 477, leaves in T0 vs BAT 477, leaves in T150	U-R	Glycosphingolipid biosynthesis - globo series	melibiase	ec:3.2.1.22	map00603	Phvul.001G135200
**Comp5.3**	BAT 477, leaves in T0 vs BAT 477, leaves in T150	U-R	Cysteine and methionine metabolism	gamma-lyase,gamma-synthase	ec:4.4.1.11,ec:2.5.1.48	map00270	Phvul.004G090200
**Comp5.3**	BAT 477, leaves in T0 vs BAT 477, leaves in T150	U-R	Sphingolipid metabolism	melibiase	ec:3.2.1.22	map00600	Phvul.001G135200

**Comp5.3**	BAT 477, leaves in T0 vs BAT 477, leaves in T150	U-R	Glycolysis / Gluconeogenesis	dehydrogenase (NADP+),dehydrogenase,dehydrogenase (NAD+)	ec:1.1.1.2, ec:1.1.1.1, ec:1.2.1.3	map00010	Phvul.008G287200,Phvul.001G064000,Phvul.009G182300
**Comp5.3**	BAT 477, leaves in T0 vs BAT 477, leaves in T150	U-R	Starch and sucrose metabolism	alpha-glucosidase,pectin demethoxylase,invertase	ec:3.2.1.48,ec:3.1.1.11,ec:3.2.1.26	map00500	Phvul.003G118400,Phvul.003G217200
**Comp5.3**	BAT 477, leaves in T0 vs BAT 477, leaves in T150	U-R	Fatty acid degradation	dehydrogenase,dehydrogenase (NAD+)	ec:1.1.1.1, ec:1.2.1.3	map00071	Phvul.001G064000,Phvul.009G182300
**Comp5.3**	BAT 477, leaves in T0 vs BAT 477, leaves in T150	U-R	Glycerolipid metabolism	dehydrogenase (NADP+),dehydrogenase (NAD+),melibiase,lipase	ec:1.1.1.2, ec:1.2.1.3, ec:3.2.1.22,ec:3.1.1.23	map00561	Phvul.008G287200,Phvul.009G182300,Phvul.001G135200,Phvul.002G332300
**Comp5.3**	BAT 477, leaves in T0 vs BAT 477, leaves in T150	U-R	Cyanoamino acid metabolism	lyase	ec:4.1.2.10	map00460	Phvul.007G066200
**Comp5.3**	BAT 477, leaves in T0 vs BAT 477, leaves in T150	U-R	Isoquinoline alkaloid biosynthesis	oxidase	ec:1.10.3.1	map00950	Phvul.006G188500
**Comp5.3**	BAT 477, leaves in T0 vs BAT 477, leaves in T150	U-R	Lysine biosynthesis	dehydrogenase (NADP+, L-glutamate-forming)	ec:1.5.1.10	map00300	Phvul.003G126300
**Comp5.3**	BAT 477, leaves in T0 vs BAT 477, leaves in T150	U-R	Phenylalanine metabolism	decarboxylase,ligase	ec:4.1.1.53,ec:6.2.1.12	map00360	Phvul.002G204900,Phvul.011G084300,Phvul.006G079500
**Comp5.3**	BAT 477, leaves in T0 vs BAT 477, leaves in T150	U-R	T cell receptor signaling pathway	phosphatase	ec:3.1.3.16	map04660	Phvul.002G163900,Phvul.001G075400,Phvul.002G309100
**Comp5.3**	BAT 477, leaves in T0 vs BAT 477, leaves in T150	U-R	Glycine, serine and threonine metabolism	dehydrogenase	ec:1.1.1.1, ec:1.2.1.8	map00260	Phvul.001G064000,Phvul.009G182300
**Comp5.3**	BAT 477, leaves in T0 vs BAT 477, leaves in T150	U-R	Fatty acid biosynthesis	synthase	ec:2.3.1.86	map00061	Phvul.011G084300
**Comp5.3**	BAT 477, leaves in T0 vs BAT 477, leaves in T150	U-R	Stilbenoid, diarylheptanoid and gingerol biosynthesis	O-hydroxycinnamoyl transferase	ec:2.3.1.133	map00945	Phvul.008G032200,Phvul.008G032400,Phvul.005G108800,Phvul.008G226200
**Comp5.3**	BAT 477, leaves in T0 vs BAT 477, leaves in T150	U-R	Selenocompound metabolism	gamma-lyase,gamma-synthase	ec:4.4.1.11,ec:2.5.1.48	map00450	Phvul.004G090200
**Comp5.3**	BAT 477, leaves in T0 vs BAT 477, leaves in T150	U-R	Flavonoid biosynthesis	synthase,oxygenase,O-hydroxycinnamoyl transferase	ec:1.14.11.23,ec:1.14.11.19,ec:2.3.1.133, ec:2.3.1.170	map00941	Phvul.002G232800,Phvul.008G098500,Phvul.008G032200,Phvul.008G032400,Phvul.005G108800, Phvul.008G226200,Phvul.008G287200

**Comp5.3**	BAT 477, leaves in T0 vs BAT 477, leaves in T150	U-R	Phenylpropanoid biosynthesis	lactoperoxidase,O-hydroxycinnamoyltransferase,ligase	ec:1.11.1.7,ec:2.3.1.133,ec:6.2.1.12	map00940	Phvul.004G018900,Phvul.006G129500,Phvul.006G075600,Phvul.008G032200,Phvul.008G032400, Phvul.005G108800,Phvul.008G226200,Phvul.011G084300,Phvul.006G079500
**Comp5.3**	BAT 477, leaves in T0 vs BAT 477, leaves in T150	U-R	Tyrosine metabolism	dehydrogenase,oxidase	ec:1.1.1.1, ec:1.10.3.1	map00350	Phvul.001G064000,Phvul.006G188500
**Comp5.3**	BAT 477, leaves in T0 vs BAT 477, leaves in T150	U-R	Alanine, aspartate and glutamate metabolism	synthase (glutamine-hydrolysing)	ec:6.3.5.4	map00250	Phvul.006G069300
**Comp5.3**	BAT 477, leaves in T0 vs BAT 477, leaves in T150	U-R	Ascorbate and aldarate metabolism	dehydrogenase (NAD+)	ec:1.2.1.3	map00053	Phvul.009G182300
**Comp5.3**	BAT 477, leaves in T0 vs BAT 477, leaves in T150	U-R	Galactose metabolism	3-alpha-galactosyltransferase,galactosyltransferase,invertase, melibiase	ec:2.4.1.123,ec:2.4.1.67,ec:3.2.1.26,ec:3.2.1.22	map00052	Phvul.007G203400,Phvul.001G135200,Phvul.003G118400
**Comp5.3**	BAT 477, leaves in T0 vs BAT 477, leaves in T150	U-R	Caprolactam degradation	dehydrogenase (NADP+)	ec:1.1.1.2	map00930	Phvul.008G287200
**Comp5.3**	BAT 477, leaves in T0 vs BAT 477, leaves in T150	U-R	Histidine metabolism	formimidoyltransferase,decarboxylase,dehydrogenase (NAD+)	ec:2.1.2.5, ec:4.1.1.22,ec:1.2.1.3	map00340	Phvul.003G044600,Phvul.002G204900,Phvul.009G182300
**Comp5.3**	BAT 477, leaves in T0 vs BAT 477, leaves in T150	U-R	Retinol metabolism	dehydrogenase	ec:1.1.1.1	map00830	Phvul.001G064000
**Comp5.3**	BAT 477, leaves in T0 vs BAT 477, leaves in T150	U-R	Thiamine metabolism	phosphatase	ec:3.6.1.15	map00730	Phvul.008G048400,Phvul.004G046200
**Comp5.3**	BAT 477, leaves in T0 vs BAT 477, leaves in T150	U-R	Glyoxylate and dicarboxylate metabolism	dehydrogenase,ligase,synthase	ec:1.2.1.2, ec:6.2.1.8, ec:2.3.3.9	map00630	Phvul.004G014700,Phvul.006G079500,Phvul.003G209100
**Comp5.3**	BAT 477, leaves in T0 vs BAT 477, leaves in T150	U-R	Pentose and glucuronate interconversions	dehydrogenase (NADP+),dehydrogenase (NAD+),pectin demethoxylase	ec:1.1.1.2, ec:1.2.1.3, ec:3.1.1.11	map00040	Phvul.008G287200,Phvul.009G182300,Phvul.003G217200
**Comp6.2**	Pérola, leaves in T75 vs Pérola, leaves in T150	U-R	Pyruvate metabolism	synthase	ec:2.3.3.9	map00620	Phvul.003G209100
**Comp6.2**	Pérola, leaves in T75 vs Pérola, leaves in T150	U-R	Biosynthesis of antibiotics	dehydrogenase (NADP+, L-glutamate-forming),dehydrogenase (NADP+, L-lysine-forming),dehydrogenase (2-methylpropanoyl-transferring)	ec:1.5.1.10,ec:1.5.1.8,ec:1.2.4.4	map01130	Phvul.003G126300,Phvul.009G132900

**Comp6.2**	Pérola, leaves in T75 vs Pérola, leaves in T150	U-R	Methane metabolism	dehydrogenase	ec:1.2.1.2, ec:1.1.1.284	map00680	Phvul.004G014600,Phvul.008G207900
**Comp6.2**	Pérola, leaves in T75 vs Pérola, leaves in T150	U-R	Valine, leucine and isoleucine degradation	dehydrogenase (2-methylpropanoyl-transferring)	ec:1.2.4.4	map00280	Phvul.009G132900
**Comp6.2**	Pérola, leaves in T75 vs Pérola, leaves in T150	U-R	Lysine degradation	dehydrogenase (NADP+, L-glutamate-forming),dehydrogenase (NADP+, L-lysine-forming)	ec:1.5.1.10,ec:1.5.1.8	map00310	Phvul.003G126300
**Comp6.2**	Pérola, leaves in T75 vs Pérola, leaves in T150	U-R	Cyanoamino acid metabolism	lyase	ec:4.1.2.10	map00460	Phvul.007G066200
**Comp6.2**	Pérola, leaves in T75 vs Pérola, leaves in T150	U-R	Isoquinoline alkaloid biosynthesis	oxidase	ec:1.10.3.1	map00950	Phvul.006G188500
**Comp6.2**	Pérola, leaves in T75 vs Pérola, leaves in T150	U-R	Lysine biosynthesis	dehydrogenase (NADP+, L-glutamate-forming)	ec:1.5.1.10	map00300	Phvul.003G126300
**Comp6.2**	Pérola, leaves in T75 vs Pérola, leaves in T150	U-R	Tyrosine metabolism	oxidase	ec:1.10.3.1	map00350	Phvul.006G188500
**Comp6.2**	Pérola, leaves in T75 vs Pérola, leaves in T150	U-R	Alanine, aspartate and glutamate metabolism	synthase (glutamine-hydrolysing)	ec:6.3.5.4	map00250	Phvul.006G187500
**Comp6.2**	Pérola, leaves in T75 vs Pérola, leaves in T150	U-R	Propanoate metabolism	dehydrogenase (2-methylpropanoyl-transferring)	ec:1.2.4.4	map00640	Phvul.009G132900
**Comp6.2**	Pérola, leaves in T75 vs Pérola, leaves in T150	U-R	Glyoxylate and dicarboxylate metabolism	dehydrogenase,synthase	ec:1.2.1.2, ec:2.3.3.9	map00630	Phvul.004G014600,Phvul.003G209100
**Comp6.3**	Pérola, leaves in T0 vs Pérola, leaves in T150	D-G	Drug metabolism - cytochrome P450	dehydrogenase [NAD(P)+]	ec:1.2.1.5	map00982	Phvul.005G147600
**Comp6.3**	Pérola, leaves in T0 vs Pérola, leaves in T150	D-G	Metabolism of xenobiotics by cytochrome P450	dehydrogenase [NAD(P)+]	ec:1.2.1.5	map00980	Phvul.005G147600
**Comp6.3**	Pérola, leaves in T0 vs Pérola, leaves in T150	D-G	Nitrogen metabolism	reductase (NADH),reductase (NADPH),reductase	ec:1.7.1.1, ec:1.7.1.3, ec:1.7.99.4	map00910	Phvul.009G121000
**Comp6.3**	Pérola, leaves in T0 vs Pérola, leaves in T150	D-G	Zeatin biosynthesis	dehydrogenase	ec:1.5.99.12	map00908	Phvul.005G034000
**Comp6.3**	Pérola, leaves in T0 vs Pérola, leaves in T150	D-G	Carotenoid biosynthesis	desaturase	ec:1.3.5.6	map00906	Phvul.009G018300
**Comp6.3**	Pérola, leaves in T0 vs Pérola, leaves in T150	D-G	Diterpenoid biosynthesis	3beta-dioxygenase	ec:1.14.11.15	map00904	Phvul.005G173500
**Comp6.3**	Pérola, leaves in T0 vs Pérola, leaves in T150	D-G	Indole alkaloid biosynthesis	synthase	ec:4.3.3.2	map00901	Phvul.003G281200
**Comp6.3**	Pérola, leaves in T0 vs Pérola, leaves in T150	D-G	beta-Alanine metabolism	dehydrogenase [NAD(P)+]	ec:1.2.1.5	map00410	Phvul.005G147600
**Comp6.3**	Pérola, leaves in T0 vs Pérola, leaves in T150	D-G	Glycolysis / Gluconeogenesis	dehydrogenase [NAD(P)+]	ec:1.2.1.5	map00010	Phvul.005G147600
**Comp6.3**	Pérola, leaves in T0 vs Pérola, leaves in T150	D-G	Isoquinoline alkaloid biosynthesis	oxidase	ec:1.10.3.1	map00950	Phvul.008G073200
**Comp6.3**	Pérola, leaves in T0 vs Pérola, leaves in T150	D-G	Phenylalanine metabolism	dehydrogenase [NAD(P)+]	ec:1.2.1.5	map00360	Phvul.005G147600
**Comp6.3**	Pérola, leaves in T0 vs Pérola, leaves in T150	D-G	Steroid biosynthesis	synthase	ec:5.4.99.8	map00100	Phvul.006G156100

**Comp6.3**	Pérola, leaves in T0 vs Pérola, leaves in T150	D-G	Tyrosine metabolism	dehydrogenase [NAD(P)+],oxidase	ec:1.2.1.5, ec:1.10.3.1	map00350	Phvul.005G147600,Phvul.008G073200
**Comp6.3**	Pérola, leaves in T0 vs Pérola, leaves in T150	D-G	Histidine metabolism	dehydrogenase [NAD(P)+]	ec:1.2.1.5	map00340	Phvul.005G147600
**Comp6.3**	Pérola, leaves in T0 vs Pérola, leaves in T150	U-R	Sulfur metabolism	gamma-synthase,reductase (glutathione)	ec:2.5.1.48,ec:1.8.4.9	map00920	Phvul.004G090200,Phvul.003G079800
**Comp6.3**	Pérola, leaves in T0 vs Pérola, leaves in T150	U-R	Drug metabolism - other enzymes	ali-esterase	ec:3.1.1.1	map00983	Phvul.003G227500,Phvul.002G332300
**Comp6.3**	Pérola, leaves in T0 vs Pérola, leaves in T150	U-R	Drug metabolism - cytochrome P450	transferase	ec:2.5.1.18	map00982	Phvul.006G079600
**Comp6.3**	Pérola, leaves in T0 vs Pérola, leaves in T150	U-R	Metabolism of xenobiotics by cytochrome P450	transferase	ec:2.5.1.18	map00980	Phvul.006G079600
**Comp6.3**	Pérola, leaves in T0 vs Pérola, leaves in T150	U-R	Arginine and proline metabolism	dehydrogenase (NAD+),dehydrogenase	ec:1.2.1.3, ec:1.2.1.19	map00330	Phvul.009G182300
**Comp6.3**	Pérola, leaves in T0 vs Pérola, leaves in T150	U-R	Aminobenzoate degradation	nitrophenyl phosphatase	ec:3.1.3.41	map00627	Phvul.001G017200,Phvul.002G163900
**Comp6.3**	Pérola, leaves in T0 vs Pérola, leaves in T150	U-R	Purine metabolism	adenylpyrophosphatase,phosphatase	ec:3.6.1.3, ec:3.6.1.15	map00230	Phvul.004G046200
**Comp6.3**	Pérola, leaves in T0 vs Pérola, leaves in T150	U-R	Chloroalkane and chloroalkene degradation	dehydrogenase (NAD+)	ec:1.2.1.3	map00625	Phvul.009G182300
**Comp6.3**	Pérola, leaves in T0 vs Pérola, leaves in T150	U-R	Valine, leucine and isoleucine biosynthesis	transaminase	ec:2.6.1.42	map00290	Phvul.009G075100
**Comp6.3**	Pérola, leaves in T0 vs Pérola, leaves in T150	U-R	Ubiquinone and other terpenoid-quinone biosynthesis	ligase	ec:6.2.1.26,ec:6.2.1.12	map00130	Phvul.006G079500,Phvul.011G084300
**Comp6.3**	Pérola, leaves in T0 vs Pérola, leaves in T150	U-R	Pyruvate metabolism	dehydrogenase (NAD+),synthase	ec:1.2.1.3, ec:2.3.3.9	map00620	Phvul.009G182300,Phvul.003G209100
**Comp6.3**	Pérola, leaves in T0 vs Pérola, leaves in T150	U-R	Biosynthesis of antibiotics	dehydrogenase (NADP+),dehydrogenase (NADP+, L-glutamate-forming),gamma-synthase,transaminase,dehydrogenase (NAD+),dehydrogenase (NADP+-dependent, decarboxylating),dehydrogenase (NADP+, L-lysine-forming),dehydrogenase (2-methylpropanoyl-transferring)	ec:1.1.1.2, ec:1.5.1.10,ec:2.5.1.48,ec:2.6.1.42,ec:1.2.1.3,ec:1.1.1.44,ec:1.5.1.8,ec:1.2.4.4	map01130	Phvul.008G287200,Phvul.003G126300,Phvul.004G090200,Phvul.009G075100,Phvul.009G182300, Phvul.006G127500,Phvul.009G132900

**Comp6.3**	Pérola, leaves in T0 vs Pérola, leaves in T150	U-R	Pentose phosphate pathway	dehydrogenase (NADP+-dependent, decarboxylating)	ec:1.1.1.44	map00030	Phvul.006G127500
**Comp6.3**	Pérola, leaves in T0 vs Pérola, leaves in T150	U-R	Methane metabolism	dehydrogenase	ec:1.2.1.2, ec:1.1.1.284	map00680	Phvul.004G014600,Phvul.008G207900
**Comp6.3**	Pérola, leaves in T0 vs Pérola, leaves in T150	U-R	Glutathione metabolism	transferase,dehydrogenase (NADP+-dependent, decarboxylating)	ec:2.5.1.18,ec:1.1.1.44	map00480	Phvul.006G079600,Phvul.006G127500
**Comp6.3**	Pérola, leaves in T0 vs Pérola, leaves in T150	U-R	Tryptophan metabolism	dehydrogenase (NAD+)	ec:1.2.1.3	map00380	Phvul.009G182300
**Comp6.3**	Pérola, leaves in T0 vs Pérola, leaves in T150	U-R	Valine, leucine and isoleucine degradation	transaminase,dehydrogenase (NAD+),dehydrogenase,dehydrogenase (2-methylpropanoyl-transferring)	ec:2.6.1.42,ec:1.2.1.3,ec:1.1.1.31,ec:1.2.4.4	map00280	Phvul.009G075100,Phvul.009G182300,Phvul.006G127500,Phvul.009G132900
**Comp6.3**	Pérola, leaves in T0 vs Pérola, leaves in T150	U-R	Pantothenate and CoA biosynthesis	transaminase	ec:2.6.1.42	map00770	Phvul.009G075100
**Comp6.3**	Pérola, leaves in T0 vs Pérola, leaves in T150	U-R	Carotenoid biosynthesis	acid 8'-hydroxylase	ec:1.14.13.93	map00906	Phvul.003G278400
**Comp6.3**	Pérola, leaves in T0 vs Pérola, leaves in T150	U-R	Diterpenoid biosynthesis	3beta-dioxygenase	ec:1.14.11.15	map00904	Phvul.001G055000
**Comp6.3**	Pérola, leaves in T0 vs Pérola, leaves in T150	U-R	Limonene and pinene degradation	dehydrogenase (NAD+)	ec:1.2.1.3	map00903	Phvul.009G182300
**Comp6.3**	Pérola, leaves in T0 vs Pérola, leaves in T150	U-R	beta-Alanine metabolism	dehydrogenase (NAD+),dehydrogenase	ec:1.2.1.3, ec:1.2.1.19	map00410	Phvul.009G182300
**Comp6.3**	Pérola, leaves in T0 vs Pérola, leaves in T150	U-R	Glucosinolate biosynthesis	transaminase	ec:2.6.1.42	map00966	Phvul.009G075100
**Comp6.3**	Pérola, leaves in T0 vs Pérola, leaves in T150	U-R	Lysine degradation	dehydrogenase (NADP+, L-glutamate-forming),dehydrogenase (NAD+),dehydrogenase (NADP+, L-lysine-forming)	ec:1.5.1.10,ec:1.2.1.3,ec:1.5.1.8	map00310	Phvul.003G126300,Phvul.009G182300
**Comp6.3**	Pérola, leaves in T0 vs Pérola, leaves in T150	U-R	Glycosphingolipid biosynthesis - globo series	melibiase	ec:3.2.1.22	map00603	Phvul.001G135200
**Comp6.3**	Pérola, leaves in T0 vs Pérola, leaves in T150	U-R	Cysteine and methionine metabolism	gamma-lyase,gamma-synthase,transaminase	ec:4.4.1.11,ec:2.5.1.48,ec:2.6.1.42	map00270	Phvul.004G090200,Phvul.009G075100
**Comp6.3**	Pérola, leaves in T0 vs Pérola, leaves in T150	U-R	Sphingolipid metabolism	melibiase	ec:3.2.1.22	map00600	Phvul.001G135200
**Comp6.3**	Pérola, leaves in T0 vs Pérola, leaves in T150	U-R	Glycolysis / Gluconeogenesis	dehydrogenase (NADP+),dehydrogenase (NAD+)	ec:1.1.1.2, ec:1.2.1.3	map00010	Phvul.008G287200,Phvul.009G182300
**Comp6.3**	Pérola, leaves in T0 vs Pérola, leaves in T150	U-R	Starch and sucrose metabolism	alpha-glucosidase,pectin demethoxylase,invertase	ec:3.2.1.48,ec:3.1.1.11,ec:3.2.1.26	map00500	Phvul.003G118400,Phvul.003G227500

**Comp6.3**	Pérola, leaves in T0 vs Pérola, leaves in T150	U-R	Fatty acid degradation	dehydrogenase (NAD+)	ec:1.2.1.3	map00071	Phvul.009G182300
**Comp6.3**	Pérola, leaves in T0 vs Pérola, leaves in T150	U-R	Inositol phosphate metabolism	5-phosphatase	ec:3.1.3.36	map00562	Phvul.001G017200
**Comp6.3**	Pérola, leaves in T0 vs Pérola, leaves in T150	U-R	Glycerolipid metabolism	dehydrogenase (NADP+),dehydrogenase (NAD+),melibiase,lipase	ec:1.1.1.2,ec:1.2.1.3,ec:3.2.1.22,ec:3.1.1.23	map00561	Phvul.008G287200,Phvul.009G182300,Phvul.001G135200,Phvul.002G332300
**Comp6.3**	Pérola, leaves in T0 vs Pérola, leaves in T150	U-R	Cyanoamino acid metabolism	lyase	ec:4.1.2.10	map00460	Phvul.007G066200
**Comp6.3**	Pérola, leaves in T0 vs Pérola, leaves in T150	U-R	Isoquinoline alkaloid biosynthesis	oxidase	ec:1.10.3.1	map00950	Phvul.006G188500
**Comp6.3**	Pérola, leaves in T0 vs Pérola, leaves in T150	U-R	Lysine biosynthesis	dehydrogenase (NADP+, L-glutamate-forming)	ec:1.5.1.10	map00300	Phvul.003G126300
**Comp6.3**	Pérola, leaves in T0 vs Pérola, leaves in T150	U-R	Phenylalanine metabolism	ligase	ec:6.2.1.12	map00360	Phvul.011G084300,Phvul.006G079500
**Comp6.3**	Pérola, leaves in T0 vs Pérola, leaves in T150	U-R	T cell receptor signaling pathway	phosphatase	ec:3.1.3.16	map04660	Phvul.002G163900
**Comp6.3**	Pérola, leaves in T0 vs Pérola, leaves in T150	U-R	Glycine, serine and threonine metabolism	dehydrogenase	ec:1.2.1.8	map00260	Phvul.009G182300
**Comp6.3**	Pérola, leaves in T0 vs Pérola, leaves in T150	U-R	Steroid biosynthesis	synthase	ec:5.4.99.8	map00100	Phvul.006G194200
**Comp6.3**	Pérola, leaves in T0 vs Pérola, leaves in T150	U-R	Phosphatidylinositol signaling system	5-phosphatase	ec:3.1.3.36	map04070	Phvul.001G017200
**Comp6.3**	Pérola, leaves in T0 vs Pérola, leaves in T150	U-R	Fatty acid biosynthesis	synthase	ec:2.3.1.86	map00061	Phvul.011G084300
**Comp6.3**	Pérola, leaves in T0 vs Pérola, leaves in T150	U-R	Stilbenoid, diarylheptanoid and gingerol biosynthesis	O-hydroxycinnamoyl transferase	ec:2.3.1.133	map00945	Phvul.001G112500,Phvul.008G032200,Phvul.008G032400,Phvul.001G112400,Phvul.005G108800
**Comp6.3**	Pérola, leaves in T0 vs Pérola, leaves in T150	U-R	Selenocompound metabolism	gamma-lyase,gamma-synthase	ec:4.4.1.11,ec:2.5.1.48	map00450	Phvul.004G090200
**Comp6.3**	Pérola, leaves in T0 vs Pérola, leaves in T150	U-R	Flavonoid biosynthesis	synthase,O-hydroxycinnamoyl transferase	ec:2.3.1.74,ec:2.3.1.133,ec:2.3.1.170	map00941	Phvul.009G131000,Phvul.001G112500,Phvul.008G032200,Phvul.008G032400,Phvul.001G112400,Phvul.005G108800,Phvul.008G287200
**Comp6.3**	Pérola, leaves in T0 vs Pérola, leaves in T150	U-R	Phenylpropanoid biosynthesis	O-hydroxycinnamoyl transferase,ligase	ec:2.3.1.133,ec:6.2.1.12	map00940	Phvul.001G112500,Phvul.008G032200,Phvul.008G032400,Phvul.001G112400,Phvul.005G108800,Phvul.011G0

							84300,Phvul.006G079500
**Comp6.3**	Pérola, leaves in T0 vs Pérola, leaves in T150	U-R	Tyrosine metabolism	oxidase	ec:1.10.3.1	map00350	Phvul.006G188500
**Comp6.3**	Pérola, leaves in T0 vs Pérola, leaves in T150	U-R	Alanine, aspartate and glutamate metabolism	synthase (glutamine-hydrolysing)	ec:6.3.5.4	map00250	Phvul.006G069300
**Comp6.3**	Pérola, leaves in T0 vs Pérola, leaves in T150	U-R	Propanoate metabolism	dehydrogenase (2-methylpropanoyl-transferring)	ec:1.2.4.4	map00640	Phvul.009G132900
**Comp6.3**	Pérola, leaves in T0 vs Pérola, leaves in T150	U-R	Ascorbate and aldarate metabolism	dehydrogenase (NAD+)	ec:1.2.1.3	map00053	Phvul.009G182300
**Comp6.3**	Pérola, leaves in T0 vs Pérola, leaves in T150	U-R	Galactose metabolism	3-alpha-galactosyltransferase,galactosyltransferase,invertase,melibiase	ec:2.4.1.123,ec:2.4.1.67,ec:3.2.1.26,ec:3.2.1.22	map00052	Phvul.001G223700,Phvul.001G135200,Phvul.003G118400
**Comp6.3**	Pérola, leaves in T0 vs Pérola, leaves in T150	U-R	Caprolactam degradation	dehydrogenase (NADP+)	ec:1.1.1.2	map00930	Phvul.008G287200
**Comp6.3**	Pérola, leaves in T0 vs Pérola, leaves in T150	U-R	Histidine metabolism	dehydrogenase (NAD+)	ec:1.2.1.3	map00340	Phvul.009G182300
**Comp6.3**	Pérola, leaves in T0 vs Pérola, leaves in T150	U-R	Thiamine metabolism	phosphatase	ec:3.6.1.15	map00730	Phvul.004G046200
**Comp6.3**	Pérola, leaves in T0 vs Pérola, leaves in T150	U-R	Glyoxylate and dicarboxylate metabolism	dehydrogenase,ligase,synthase	ec:1.2.1.2, ec:6.2.1.8, ec:2.3.3.9	map00630	Phvul.004G014600,Phvul.006G079500,Phvul.003G209100
**Comp6.3**	Pérola, leaves in T0 vs Pérola, leaves in T150	U-R	Pentose and glucuronate interconversions	dehydrogenase (NADP+),dehydrogenase (NAD+),pectin demethoxylase	ec:1.1.1.2, ec:1.2.1.3, ec:3.1.1.11	map00040	Phvul.008G287200,Phvul.009G182300,Phvul.003G227500
**Comp7**	Pérola, root in T0 vs BAT 477, root in T0	D-G	Purine metabolism	RNA polymerase	ec:2.7.7.6	map00230	XLOC_020681
**Comp7**	Pérola, root in T0 vs BAT 477, root in T0	D-G	Various types of N-glycan biosynthesis	1,2-alpha-mannosidase	ec:3.2.1.113	map00513	Phvul.006G033000
**Comp7**	Pérola, root in T0 vs BAT 477, root in T0	D-G	Other glycan degradation	alpha-D-mannosidase	ec:3.2.1.24	map00511	Phvul.006G033000
**Comp7**	Pérola, root in T0 vs BAT 477, root in T0	D-G	N-Glycan biosynthesis	1,2-alpha-mannosidase	ec:3.2.1.113	map00510	Phvul.006G033000
**Comp7**	Pérola, root in T0 vs BAT 477, root in T0	D-G	Carotenoid biosynthesis	desaturase	ec:1.3.5.6	map00906	Phvul.010G124800
**Comp7**	Pérola, root in T0 vs BAT 477, root in T0	D-G	Pyrimidine metabolism	RNA polymerase	ec:2.7.7.6	map00240	XLOC_020681
**Comp8**	Pérola, root in T75 vs BAT 477, root in T75	D-G	Various types of N-glycan biosynthesis	1,2-alpha-mannosidase	ec:3.2.1.113	map00513	Phvul.006G033000
**Comp8**	Pérola, root in T75 vs BAT 477, root in T75	D-G	Other glycan degradation	alpha-D-mannosidase	ec:3.2.1.24	map00511	Phvul.006G033000
**Comp8**	Pérola, root in T75 vs BAT 477, root in T75	D-G	N-Glycan biosynthesis	1,2-alpha-mannosidase	ec:3.2.1.113	map00510	Phvul.006G033000
**Comp8**	Pérola, root in T75 vs BAT 477, root in T75	D-G	T cell receptor signaling pathway	protein-tyrosine kinase	ec:2.7.10.2	map04660	XLOC_017321

**Comp9**	Pérola, root in T150 vs BAT 477, root in T150	D-G	Starch and sucrose metabolism	gentiobiase	ec:3.2.1.21	map00500	Phvul.001G076700
**Comp9**	Pérola, root in T150 vs BAT 477, root in T150	D-G	Cyanoamino acid metabolism	gentiobiase	ec:3.2.1.21	map00460	Phvul.001G076700
**Comp9**	Pérola, root in T150 vs BAT 477, root in T150	D-G	Phenylpropanoid biosynthesis	gentiobiase	ec:3.2.1.21	map00940	Phvul.001G076700
**Comp1**	Pérola, leaves in T0 vs BAT 477, leaves in T0	D-G	Amino sugar and nucleotide sugar metabolism	chitodextrinase	ec:3.2.1.14	map00520	Phvul.004G101500
**Comp1**	Pérola, leaves in T0 vs BAT 477, leaves in T0	D-G	Various types of N-glycan biosynthesis	1,2-alpha-mannosidase	ec:3.2.1.113	map00513	Phvul.006G033000
**Comp1**	Pérola, leaves in T0 vs BAT 477, leaves in T0	D-G	Other glycan degradation	alpha-D-mannosidase	ec:3.2.1.24	map00511	Phvul.006G033000
**Comp1**	Pérola, leaves in T0 vs BAT 477, leaves in T0	D-G	N-Glycan biosynthesis	1,2-alpha-mannosidase	ec:3.2.1.113	map00510	Phvul.006G033000
**Comp1**	Pérola, leaves in T0 vs BAT 477, leaves in T0	D-G	Carotenoid biosynthesis	desaturase	ec:1.3.5.6	map00906	Phvul.010G021000
**Comp1**	Pérola, leaves in T0 vs BAT 477, leaves in T0	D-G	Diterpenoid biosynthesis	10beta-hydroxylase	ec:1.14.13.76	map00904	Phvul.005G053400
**Comp1**	Pérola, leaves in T0 vs BAT 477, leaves in T0	D-G	Starch and sucrose metabolism	gentiobiase,endo-1,3-beta-D-glucosidase	ec:3.2.1.21,ec:3.2.1.39	map00500	Phvul.001G128500
**Comp1**	Pérola, leaves in T0 vs BAT 477, leaves in T0	D-G	Cyanoamino acid metabolism	gentiobiase	ec:3.2.1.21	map00460	Phvul.001G128500
**Comp1**	Pérola, leaves in T0 vs BAT 477, leaves in T0	D-G	Steroid biosynthesis	synthase	ec:5.4.99.8	map00100	Phvul.006G156100
**Comp1**	Pérola, leaves in T0 vs BAT 477, leaves in T0	D-G	Stilbenoid, diarylheptanoid and gingerol biosynthesis	O-hydroxycinnamoyl transferase	ec:2.3.1.133	map00945	Phvul.010G014100
**Comp1**	Pérola, leaves in T0 vs BAT 477, leaves in T0	D-G	Flavonoid biosynthesis	O-hydroxycinnamoyl transferase	ec:2.3.1.133	map00941	Phvul.010G014100
**Comp1**	Pérola, leaves in T0 vs BAT 477, leaves in T0	D-G	Phenylpropanoid biosynthesis	O-hydroxycinnamoyl transferase,gentiobiase	ec:2.3.1.133,ec:3.2.1.21	map00940	Phvul.010G014100,Phvul.001G128500
**Comp1**	Pérola, leaves in T0 vs BAT 477, leaves in T0	U-R	Sulfur metabolism	O-acetyltransferase	ec:2.3.1.30	map00920	XLOC_024793
**Comp1**	Pérola, leaves in T0 vs BAT 477, leaves in T0	U-R	Biosynthesis of antibiotics	O-acetyltransferase	ec:2.3.1.30	map01130	XLOC_024793
**Comp1**	Pérola, leaves in T0 vs BAT 477, leaves in T0	U-R	Diterpenoid biosynthesis	synthase	ec:4.2.3.8	map00904	Phvul.011G142600
**Comp1**	Pérola, leaves in T0 vs BAT 477, leaves in T0	U-R	Cysteine and methionine metabolism	O-acetyltransferase	ec:2.3.1.30	map00270	XLOC_024793
**Comp1**	Pérola, leaves in T0 vs BAT 477, leaves in T0	U-R	Starch and sucrose metabolism	synthase (UDP-forming)	ec:2.4.1.12	map00500	Phvul.005G116500
**Comp1**	Pérola, leaves in T0 vs BAT 477, leaves in T0	U-R	Flavone and flavonol biosynthesis	3',5'-hydroxylase	ec:1.14.13.88	map00944	Phvul.006G054500
**Comp1**	Pérola, leaves in T0 vs BAT 477, leaves in T0	U-R	Flavonoid biosynthesis	3',5'-hydroxylase	ec:1.14.13.88	map00941	Phvul.006G054500
**Comp11**	Pérola, leaves in T75 vs BAT 477, leaves in T75	D-G	Biosynthesis of antibiotics	aminotransferase	ec:2.6.1.83	map01130	Phvul.008G169600
**Comp11**	Pérola, leaves in T75 vs BAT 477, leaves in T75	D-G	Carotenoid biosynthesis	desaturase	ec:1.3.5.6	map00906	Phvul.010G021000
**Comp11**	Pérola, leaves in T75 vs BAT 477, leaves in T75	D-G	Diterpenoid biosynthesis	10beta-hydroxylase	ec:1.14.13.76	map00904	Phvul.005G053400

**Comp11**	Pérola, leaves in T75 vs BAT 477, leaves in T75	D-G	Starch and sucrose metabolism	gentiobiase,endo-1,3-beta-D-glucosidase	ec:3.2.1.21,ec:3.2.1.39	map00500	Phvul.001G128500
**Comp11**	Pérola, leaves in T75 vs BAT 477, leaves in T75	D-G	Cyanoamino acid metabolism	gentiobiase	ec:3.2.1.21	map00460	Phvul.001G128500
**Comp11**	Pérola, leaves in T75 vs BAT 477, leaves in T75	D-G	Lysine biosynthesis	aminotransferase	ec:2.6.1.83	map00300	Phvul.008G169600
**Comp11**	Pérola, leaves in T75 vs BAT 477, leaves in T75	D-G	Steroid biosynthesis	synthase	ec:5.4.99.8	map00100	Phvul.006G156100
**Comp11**	Pérola, leaves in T75 vs BAT 477, leaves in T75	D-G	Stilbenoid, diarylheptanoid and gingerol biosynthesis	O-hydroxycinnamoyl transferase	ec:2.3.1.133	map00945	Phvul.010G014100,Phvul.005G109000
**Comp11**	Pérola, leaves in T75 vs BAT 477, leaves in T75	D-G	Flavone and flavonol biosynthesis	3-O-methyltransferase	ec:2.1.1.76	map00944	Phvul.007G091000,Phvul.010G120200
**Comp11**	Pérola, leaves in T75 vs BAT 477, leaves in T75	D-G	Flavonoid biosynthesis	O-hydroxycinnamoyl transferase	ec:2.3.1.133	map00941	Phvul.010G014100,Phvul.005G109000
**Comp11**	Pérola, leaves in T75 vs BAT 477, leaves in T75	D-G	Phenylpropanoid biosynthesis	O-methyltransferase,lactoperoxidase,O-hydroxycinnamoyl transferase,gentiobiase	ec:2.1.1.68,ec:1.11.1.7,ec:2.3.1.133,ec:3.2.1.21	map00940	Phvul.007G091000,Phvul.008G249900,Phvul.010G014100,Phvul.005G109000,Phvul.001G128500
**Comp11**	Pérola, leaves in T75 vs BAT 477, leaves in T75	U-R	Sulfur metabolism	O-acetyltransferase	ec:2.3.1.30	map00920	XLOC_024793
**Comp11**	Pérola, leaves in T75 vs BAT 477, leaves in T75	U-R	Biosynthesis of antibiotics	O-acetyltransferase	ec:2.3.1.30	map01130	XLOC_024793
**Comp11**	Pérola, leaves in T75 vs BAT 477, leaves in T75	U-R	Diterpenoid biosynthesis	synthase	ec:4.2.3.8	map00904	Phvul.011G142600
**Comp11**	Pérola, leaves in T75 vs BAT 477, leaves in T75	U-R	Cysteine and methionine metabolism	O-acetyltransferase	ec:2.3.1.30	map00270	XLOC_024793
**Comp11**	Pérola, leaves in T75 vs BAT 477, leaves in T75	U-R	Starch and sucrose metabolism	synthase (UDP-forming)	ec:2.4.1.12	map00500	Phvul.008G279800,Phvul.005G116500
**Comp12**	Pérola, leaves in T150 vs BAT 477, leaves in T150	D-G	Amino sugar and nucleotide sugar metabolism	chitodextrinase	ec:3.2.1.14	map00520	Phvul.004G101500
**Comp12**	Pérola, leaves in T150 vs BAT 477, leaves in T150	D-G	Carotenoid biosynthesis	desaturase	ec:1.3.5.6	map00906	Phvul.010G021000
**Comp12**	Pérola, leaves in T150 vs BAT 477, leaves in T150	D-G	Diterpenoid biosynthesis	3beta-dioxygenase	ec:1.14.11.15	map00904	Phvul.001G055000
**Comp12**	Pérola, leaves in T150 vs BAT 477, leaves in T150	D-G	Stilbenoid, diarylheptanoid and gingerol biosynthesis	O-hydroxycinnamoyl transferase	ec:2.3.1.133	map00945	Phvul.001G112400,Phvul.010G014100
**Comp12**	Pérola, leaves in T150 vs BAT 477, leaves in T150	D-G	Flavonoid biosynthesis	O-hydroxycinnamoyl transferase	ec:2.3.1.133	map00941	Phvul.001G112400,Phvul.010G014100
**Comp12**	Pérola, leaves in T150 vs BAT 477, leaves in T150	D-G	Phenylpropanoid biosynthesis	O-hydroxycinnamoyl transferase	ec:2.3.1.133	map00940	Phvul.001G112400,Phvul.010G014100
**Comp12**	Pérola, leaves in T150 vs BAT 477, leaves in T150	U-R	Sulfur metabolism	O-acetyltransferase	ec:2.3.1.30	map00920	XLOC_024793

**Comp12**	Pérola, leaves in T150 vs BAT 477, leaves in T150	U-R	Biosynthesis of antibiotics	O-acetyltransferase	ec:2.3.1.30	map01130	XLOC_024793
**Comp12**	Pérola, leaves in T150 vs BAT 477, leaves in T150	U-R	Diterpenoid biosynthesis	synthase	ec:4.2.3.8	map00904	Phvul.011G142600
**Comp12**	Pérola, leaves in T150 vs BAT 477, leaves in T150	U-R	Lysine degradation	decarboxylase	ec:4.1.1.18	map00310	Phvul.011G051300
**Comp12**	Pérola, leaves in T150 vs BAT 477, leaves in T150	U-R	Tropane, piperidine and pyridine alkaloid biosynthesis	decarboxylase	ec:4.1.1.18	map00960	Phvul.011G051300
**Comp12**	Pérola, leaves in T150 vs BAT 477, leaves in T150	U-R	Cysteine and methionine metabolism	O-acetyltransferase	ec:2.3.1.30	map00270	XLOC_024793
**Comp12**	Pérola, leaves in T150 vs BAT 477, leaves in T150	U-R	Starch and sucrose metabolism	synthase (UDP-forming)	ec:2.4.1.12	map00500	Phvul.005G116500
