# Supplementary material for: Genome-wide analysis of the transcriptional response to drought stress in root and leaf of common bean
Source: Genet Mol Biol. 2020 Mar 16;43(1):e20180259. doi: 10.1590/1678-4685-GMB-2018-0259 (PMC7307723; doi:10.1590/1678-4685-GMB-2018-0259)
Supplement: Supplementary file 6 [file 1415-4757-GMB-43-1-e20180259-s8.pdf]

Supplementary Material to: "Genome-wide analysis of the transcriptional response to drought stress in root and leaf of common"

Table S6 Description of the 397 loci with no hit in BLASTX or PSI-BLAST (non-redundant-NR database – NCBI) against the Andean genome, which showed homology with the Mesoamerican transcriptome and IncRNAs reported by Vlasova et al. (2016).

Loci	Major isoform	ID correlated with Vlasova et al. (2016)	Length	Hits	e-Value	similarity (mean)
XLOC_000092	TCONS_00000173	XLOC_001391	3711	20	0.0E0	97 %
XLOC_000275	TCONS_00000547	XLOC_000582	687	1	1.3E-119	100 %
XLOC_000464	TCONS_00000929	XLOC_003134	732	1	4.7E-179	98 %
XLOC_000533	TCONS_00001051	PHASIBEAM10F013671	207	7	4.8E-104	96 %
XLOC_000846	TCONS_00001627	PHASIBEAM10F021701	327	1	3.4E-167	99 %
XLOC_000919	TCONS_00001767	PHASIBEAM10F009746	737	1	0.0E0	99 %
XLOC_001530	TCONS_00002934	XLOC_001496	907	1	2.2E-153	95 %
XLOC_001801	TCONS_00003505	PHASIBEAM10F003778	828	3	3.8E-61	92 %
XLOC_001911	TCONS_00003685	PHASIBEAM10F020645	1001	2	0.0E0	92 %
XLOC_002171	TCONS_00004214	XLOC_002151	405	1	0.0E0	100 %
XLOC_002249	TCONS_00004380	PHASIBEAM10F014340	2247	2	0.0E0	98 %
XLOC_002350	TCONS_00004569	PHASIBEAM10F027640	1135	1	1.4E-66	84 %
XLOC_002593	TCONS_00005021	XLOC_000460	322	3	1.2E-151	96 %
XLOC_002737	TCONS_00005310	PHASIBEAM10F010234	574	1	4.0E-114	97 %
XLOC_002743	TCONS_00005316	PHASIBEAM10F015802	523	1	6.2E-87	100 %
XLOC_002744	TCONS_00005317	PHASIBEAM10F015802	861	1	3.1E-47	96 %
XLOC_002745	TCONS_00005318	PHASIBEAM10F015802	235	1	1.8E-14	97 %
XLOC_002750	TCONS_00005323	XLOC_003743	380	1	1.1E-8	84 %
XLOC_002751	TCONS_00005324	XLOC_003743	467	1	1.3E-13	90 %
XLOC_002754	TCONS_00005327	PHASIBEAM10F000578	96	6	5.8E-40	90 %
XLOC_002758	TCONS_00005331	PHASIBEAM10F029682	989	1	1.5E-120	89 %
XLOC_002765	TCONS_00005338	XLOC_002274	125	2	2.2E-55	97 %
XLOC_002768	TCONS_00005341	PHASIBEAM10F029779	588	20	5.6E-73	87 %
XLOC_002773	TCONS_00005346	PHASIBEAM10F000005	89	1	4.1E-36	97 %
XLOC_002775	TCONS_00005348	PHASIBEAM10F000420	99	20	1.0E-22	96 %
XLOC_002782	TCONS_00005355	PHASIBEAM10F020645	1032	5	1.3E-86	85 %
XLOC_002787	TCONS_00005360	PHASIBEAM10F018523	710	1	5.4E-59	100 %
XLOC_002790	TCONS_00005363	XLOC_001999	735	4	5.8E-24	89 %
XLOC_002795	TCONS_00005368	PHASIBEAM10F004152	745	3	0.0E0	93 %
XLOC_002798	TCONS_00005371	PHASIBEAM10F000430	165	1	2.4E-66	96 %
XLOC_002799	TCONS_00005372	XLOC_003182	493	2	1.1E-29	90 %
XLOC_002807	TCONS_00005380	PHASIBEAM10F026081	94	3	3.4E-37	96 %
XLOC_002818	TCONS_00005391	PHASIBEAM10F020785	1824	4	0.0E0	100 %
XLOC_002823	TCONS_00005396	PHASIBEAM10F000619	629	15	0.0E0	98 %
XLOC_002828	TCONS_00005401	PHASIBEAM10F014340	1724	7	0.0E0	98 %
XLOC_002830	TCONS_00005403	PHASIBEAM10F009749	770	2	0.0E0	100 %
XLOC_002835	TCONS_00005408	PHASIBEAM10F026136	287	1	5.1E-145	98 %
XLOC_002838	TCONS_00005411	PHASIBEAM10F022276	233	2	9.6E-72	98 %
XLOC_002842	TCONS_00005415	PHASIBEAM10F002018	1278	4	0.0E0	99 %
XLOC_003203	TCONS_00006151	PHASIBEAM10F030395	1303	7	0.0E0	93 %
XLOC_003751	TCONS_00007187	XLOC_001720	373	2	2.3E-179	99 %
XLOC_004074	TCONS_00007836	PHASIBEAM10F013753	449	1	0.0E0	100 %
XLOC_004462	TCONS_00008629	XLOC_001769	107	1	1.8E-50	100 %
XLOC_004617	TCONS_00008958	PHASIBEAM10F006639	1640	4	0.0E0	89 %
XLOC_004635	TCONS_00008985	XLOC_002995	454	10	0.0E0	92 %

Loci	Major isoform	ID correlated with Vlasova et al. (2016)	Length	Hits	e-Value	similarity (mean)
XLOC_005197	TCONS_00010135	PHASIBEAM10F026508	698	10	0.0E0	96 %
XLOC_005382	TCONS_00010529	PHASIBEAM10F006064	1138	2	0.0E0	97 %
XLOC_005502	TCONS_00010790	XLOC_002991	731	2	0.0E0	98 %
XLOC_005820	TCONS_00011398	PHASIBEAM10F005210	1003	1	0.0E0	99 %
XLOC_006160	TCONS_00012103	PHASIBEAM10F012829	364	6	0.0E0	89 %
XLOC_006211	TCONS_00012201	XLOC_002801	241	4	1.6E-119	98 %
XLOC_006216	TCONS_00012206	XLOC_002803	1272	9	0.0E0	99 %
XLOC_006226	TCONS_00012216	PHASIBEAM10F026449	100	1	6.1E-40	96 %
XLOC_006227	TCONS_00012217	PHASIBEAM10F000796	445	1	9.7E-20	94 %
XLOC_006240	TCONS_00012230	XLOC_003822	823	1	1.6E-119	100 %
XLOC_006241	TCONS_00012231	XLOC_001250	714	3	0.0E0	98 %
XLOC_006242	TCONS_00012232	XLOC_001250	559	3	1.1E-94	93 %
XLOC_006243	TCONS_00012233	PHASIBEAM10F009038	2203	1	2.2E-62	98 %
XLOC_006251	TCONS_00012241	PHASIBEAM10F026888	303	2	8.8E-158	100 %
XLOC_006252	TCONS_00012242	XLOC_001917	518	2	0.0E0	99 %
XLOC_006253	TCONS_00012243	XLOC_001917	474	2	9.7E-65	98 %
XLOC_006258	TCONS_00012248	XLOC_002270	314	6	9.3E-148	96 %
XLOC_006259	TCONS_00012249	PHASIBEAM10F006031	598	1	1.2E-104	100 %
XLOC_006261	TCONS_00012251	PHASIBEAM10F018097	325	7	4.4E-156	92 %
XLOC_006267	TCONS_00012257	XLOC_001382	509	2	1.3E-98	99 %
XLOC_006285	TCONS_00012275	PHASIBEAM10F027457	302	20	1.6E-90	87 %
XLOC_006287	TCONS_00012277	XLOC_001980	667	1	0.0E0	95 %
XLOC_006292	TCONS_00012282	PHASIBEAM10F010736	688	5	0.0E0	92 %
XLOC_006297	TCONS_00012287	PHASIBEAM10F029340	436	1	9.4E-20	98 %
XLOC_006717	TCONS_00013124	PHASIBEAM10F018097	291	7	1.9E-109	90 %
XLOC_006748	TCONS_00013184	PHASIBEAM10F013660	538	20	2.9E-110	95 %
XLOC_006778	TCONS_00013243	XLOC_003739	2015	2	0.0E0	99 %
XLOC_007104	TCONS_00013848	XLOC_002084	6573	20	0.0E0	93 %
XLOC_007106	TCONS_00013859	XLOC_002084	4711	20	0.0E0	87 %
XLOC_007107	TCONS_00013872	XLOC_002105	1721	20	2.8E-70	86 %
XLOC_007389	TCONS_00014457	PHASIBEAM10F019346	686	1	0.0E0	99 %
XLOC_007614	TCONS_00014902	PHASIBEAM10F014896	2060	8	0.0E0	90 %
XLOC_007780	TCONS_00015234	PHASIBEAM10F004219	2304	1	0.0E0	99 %
XLOC_007883	TCONS_00015464	XLOC_002137	833	1	2.8E-82	86 %
XLOC_008365	TCONS_00016473	PHASIBEAM10F028931	326	4	5.6E-170	96 %
XLOC_008366	TCONS_00016474	PHASIBEAM10F027378	429	8	0.0E0	96 %
XLOC_008570	TCONS_00016875	XLOC_001883	635	2	0.0E0	95 %
XLOC_008676	TCONS_00017101	XLOC_002084	5039	20	0.0E0	93 %
XLOC_008794	TCONS_00017368	XLOC_000437	914	5	0.0E0	98 %
XLOC_008824	TCONS_00017427	XLOC_000435	1651	16	0.0E0	99 %
XLOC_008859	TCONS_00017496	PHASIBEAM10F006639	1610	4	0.0E0	89 %
XLOC_009313	TCONS_00018454	PHASIBEAM10F015956	848	1	0.0E0	96 %
XLOC_009321	TCONS_00018462	PHASIBEAM10F003361	1090	1	0.0E0	99 %
XLOC_009329	TCONS_00018470	PHASIBEAM10F006561	331	2	4.4E-171	97 %
XLOC_009331	TCONS_00018472	PHASIBEAM10F019673	117	2	5.7E-51	97 %
XLOC_009341	TCONS_00018482	PHASIBEAM10F023326	689	1	0.0E0	100 %
XLOC_009343	TCONS_00018484	XLOC_003376	915	5	4.9E-125	97 %
XLOC_009352	TCONS_00018493	XLOC_003095	798	2	0.0E0	99 %
XLOC_009377	TCONS_00018518	PHASIBEAM10F029129	990	1	1.9E-119	98 %
XLOC_009381	TCONS_00018522	XLOC_001611	428	14	1.1E-63	89 %
XLOC_009382	TCONS_00018523	XLOC_001611	1497	20	1.2E-58	91 %
XLOC_009383	TCONS_00018524	XLOC_002084	2390	20	1.8E-68	85 %
XLOC_009384	TCONS_00018525	XLOC_000967	1344	20	3.6E-73	83 %

Loci	Major isoform	ID correlated with Vlasova et al. (2016)	Length	Hits	e-Value	similarity (mean)
XLOC_009385	TCONS_00018526	XLOC_001611	1806	20	3.8E-74	85 %
XLOC_009386	TCONS_00018527	XLOC_001611	1277	4	9.0E-119	87 %
XLOC_009389	TCONS_00018530	PHASIBEAM10F006094	335	1	1.9E-60	84 %
XLOC_009391	TCONS_00018532	PHASIBEAM10F026449	64	1	4.4E-24	98 %
XLOC_009392	TCONS_00018533	XLOC_001611	127	4	2.9E-54	96 %
XLOC_009393	TCONS_00018534	PHASIBEAM10F001885	598	2	7.1E-97	92 %
XLOC_009394	TCONS_00018535	XLOC_003105	299	1	1.1E-156	100 %
XLOC_009398	TCONS_00018539	PHASIBEAM10F022340	769	2	0.0E0	93 %
XLOC_009408	TCONS_00018549	PHASIBEAM10F017388	411	2	9.5E-149	98 %
XLOC_009416	TCONS_00018557	PHASIBEAM10F004871	426	1	3.6E-133	100 %
XLOC_009420	TCONS_00018561	PHASIBEAM10F004202	251	2	5.9E-109	96 %
XLOC_009422	TCONS_00018563	PHASIBEAM10F004271	157	1	4.7E-78	100 %
XLOC_009424	TCONS_00018565	PHASIBEAM10F004316	106	1	8.5E-44	98 %
XLOC_009463	TCONS_00018624	PHASIBEAM10F007250	2330	2	0.0E0	94 %
XLOC_010095	TCONS_00019771	PHASIBEAM10F013366	1583	2	0.0E0	99 %
XLOC_010325	TCONS_00020216	PHASIBEAM10F024339	480	3	0.0E0	98 %
XLOC_010396	TCONS_00020369	PHASIBEAM10F010980	900	1	7.2E-24	100 %
XLOC_010697	TCONS_00020886	PHASIBEAM10F017049	542	1	1.4E-103	92 %
XLOC_010754	TCONS_00020986	XLOC_003939	1250	2	0.0E0	99 %
XLOC_011257	TCONS_00021847	PHASIBEAM10F023419	156	1	2.2E-76	100 %
XLOC_011261	TCONS_00021851	PHASIBEAM10F026444	193	1	2.3E-12	100 %
XLOC_011267	TCONS_00021857	PHASIBEAM10F013329	683	4	5.1E-69	94 %
XLOC_011274	TCONS_00021864	XLOC_002687	139	2	4.2E-63	98 %
XLOC_011275	TCONS_00021865	PHASIBEAM10F019256	414	1	0.0E0	96 %
XLOC_011276	TCONS_00021866	PHASIBEAM10F004902	112	1	9.0E-49	98 %
XLOC_011280	TCONS_00021870	PHASIBEAM10F026450	259	1	2.5E-18	98 %
XLOC_011281	TCONS_00021871	PHASIBEAM10F004664	465	1	1.9E-131	99 %
XLOC_011292	TCONS_00021882	PHASIBEAM10F013152	705	1	0.0E0	100 %
XLOC_011297	TCONS_00021887	PHASIBEAM10F006719	500	1	1.6E-112	86 %
XLOC_011305	TCONS_00021895	PHASIBEAM10F012052	659	2	0.0E0	100 %
XLOC_011310	TCONS_00021900	XLOC_004064	811	3	0.0E0	95 %
XLOC_011316	TCONS_00021906	PHASIBEAM10F014660	898	5	1.2E-41	95 %
XLOC_011325	TCONS_00021915	XLOC_000960	862	2	0.0E0	99 %
XLOC_011330	TCONS_00021920	XLOC_003260	363	1	0.0E0	100 %
XLOC_011334	TCONS_00021924	PHASIBEAM10F027695	283	1	3.0E-132	96 %
XLOC_011347	TCONS_00021937	PHASIBEAM10F008329	1005	1	4.6E-51	86 %
XLOC_011354	TCONS_00021944	PHASIBEAM10F026447	95	1	3.4E-42	98 %
XLOC_012008	TCONS_00023226	PHASIBEAM10F022334	419	4	0.0E0	92 %
XLOC_012025	TCONS_00023271	PHASIBEAM10F016538	395	8	0.0E0	99 %
XLOC_012057	TCONS_00023329	XLOC_002305	539	1	4.4E-178	99 %
XLOC_012453	TCONS_00024169	XLOC_002681	601	2	0.0E0	94 %
XLOC_012554	TCONS_00024357	XLOC_000317	1245	4	1.9E-105	93 %
XLOC_012591	TCONS_00024414	XLOC_000312	767	1	0.0E0	99 %
XLOC_012625	TCONS_00024474	PHASIBEAM10F020115	810	12	0.0E0	99 %
XLOC_012728	TCONS_00024711	PHASIBEAM10F017575	2620	15	0.0E0	91 %
XLOC_012777	TCONS_00024810	PHASIBEAM10F006639	1177	10	2.4E-94	94 %
XLOC_013062	TCONS_00025330	PHASIBEAM10F000619	226	3	3.2E-111	97 %
XLOC_013138	TCONS_00025489	XLOC_003086	780	1	0.0E0	99 %
XLOC_013249	TCONS_00025691	XLOC_003157	555	2	6.3E-122	90 %
XLOC_013250	TCONS_00025692	XLOC_000262	268	1	2.9E-122	96 %
XLOC_013264	TCONS_00025706	PHASIBEAM10F000940	566	1	0.0E0	100 %
XLOC_013265	TCONS_00025707	PHASIBEAM10F000940	532	3	0.0E0	96 %
XLOC_013266	TCONS_00025708	PHASIBEAM10F000940	1253	1	0.0E0	100 %

Loci	Major isoform	ID correlated with Vlasova et al. (2016)	Length	Hits	e-Value	similarity (mean)
XLOC_013271	TCONS_00025713	PHASIBEAM10F020114	897	1	4.1E-56	100 %
XLOC_013272	TCONS_00025714	PHASIBEAM10F008718	110	1	4.1E-47	97 %
XLOC_013276	TCONS_00025718	PHASIBEAM10F022683	418	1	8.3E-80	97 %
XLOC_013282	TCONS_00025724	PHASIBEAM10F017960	641	1	6.4E-38	100 %
XLOC_013284	TCONS_00025726	PHASIBEAM10F003923	337	7	1.6E-170	89 %
XLOC_013286	TCONS_00025728	XLOC_000178	1566	11	1.4E-152	93 %
XLOC_013287	TCONS_00025729	XLOC_003181	565	2	1.4E-103	93 %
XLOC_013289	TCONS_00025731	PHASIBEAM10F025406	628	1	0.0E0	99 %
XLOC_013291	TCONS_00025733	XLOC_003974	476	2	0.0E0	99 %
XLOC_013294	TCONS_00025736	PHASIBEAM10F002497	247	3	1.7E-79	96 %
XLOC_013300	TCONS_00025742	PHASIBEAM10F016275	224	5	1.5E-84	91 %
XLOC_013305	TCONS_00025747	XLOC_002374	779	2	4.5E-70	98 %
XLOC_013310	TCONS_00025752	XLOC_001122	1113	1	0.0E0	99 %
XLOC_013311	TCONS_00025753	XLOC_001122	547	1	1.9E-62	97 %
XLOC_013312	TCONS_00025754	PHASIBEAM10F007920	211	1	6.3E-108	100 %
XLOC_013313	TCONS_00025755	PHASIBEAM10F007964	1499	3	3.4E-24	97 %
XLOC_013317	TCONS_00025759	PHASIBEAM10F022680	327	3	1.6E-150	94 %
XLOC_013318	TCONS_00025760	PHASIBEAM10F012570	494	4	5.2E-167	89 %
XLOC_013319	TCONS_00025761	PHASIBEAM10F016269	146	2	7.4E-61	97 %
XLOC_013394	TCONS_00025926	PHASIBEAM10F030373	733	8	2.4E-107	91 %
XLOC_013427	TCONS_00025983	XLOC_001257	205	2	6.7E-48	97 %
XLOC_013448	TCONS_00026016	PHASIBEAM10F000252	460	5	4.1E-113	90 %
XLOC_013460	TCONS_00026053	PHASIBEAM10F023821	634	20	0.0E0	95 %
XLOC_013664	TCONS_00026411	XLOC_001351	1711	1	3.0E-20	100 %
XLOC_013782	TCONS_00026623	XLOC_002351	438	1	1.3E-152	99 %
XLOC_013810	TCONS_00026685	PHASIBEAM10F016877	211	1	3.0E-86	99 %
XLOC_014431	TCONS_00027872	XLOC_001056	927	1	0.0E0	99 %
XLOC_014665	TCONS_00028322	PHASIBEAM10F027406	448	2	0.0E0	93 %
XLOC_014669	TCONS_00028327	PHASIBEAM10F027405	628	2	1.4E-34	100 %
XLOC_014736	TCONS_00028448	XLOC_001361	961	1	0.0E0	98 %
XLOC_014781	TCONS_00028532	PHASIBEAM10F026110	2393	3	0.0E0	99 %
XLOC_015563	TCONS_00030015	PHASIBEAM10F017819	252	8	1.3E-115	83 %
XLOC_015565	TCONS_00030017	PHASIBEAM10F009441	1485	2	2.6E-15	86 %
XLOC_015569	TCONS_00030021	PHASIBEAM10F000853	232	16	2.0E-78	95 %
XLOC_015570	TCONS_00030022	PHASIBEAM10F008878	763	11	0.0E0	96 %
XLOC_015571	TCONS_00030023	PHASIBEAM10F008865	100	5	6.1E-45	99 %
XLOC_015574	TCONS_00030026	PHASIBEAM10F001708	1039	1	0.0E0	99 %
XLOC_015578	TCONS_00030030	PHASIBEAM10F022276	326	1	9.7E-148	95 %
XLOC_015582	TCONS_00030034	XLOC_002052	1414	2	0.0E0	98 %
XLOC_015583	TCONS_00030035	PHASIBEAM10F016269	348	1	5.2E-91	98 %
XLOC_015587	TCONS_00030039	PHASIBEAM10F018097	1174	11	0.0E0	91 %
XLOC_015589	TCONS_00030041	PHASIBEAM10F020183	351	1	3.4E-28	94 %
XLOC_015595	TCONS_00030047	PHASIBEAM10F009970	1043	1	0.0E0	99 %
XLOC_015597	TCONS_00030049	XLOC_001852	253	20	2.2E-103	93 %
XLOC_015603	TCONS_00030055	PHASIBEAM10F002690	523	1	6.9E-12	97 %
XLOC_015604	TCONS_00030056	PHASIBEAM10F028074	81	1	1.7E-29	97 %
XLOC_015606	TCONS_00030058	PHASIBEAM10F015352	811	3	0.0E0	99 %
XLOC_015609	TCONS_00030061	PHASIBEAM10F014858	131	5	1.1E-63	100 %
XLOC_015612	TCONS_00030064	PHASIBEAM10F020766	493	1	8.6E-180	99 %
XLOC_015616	TCONS_00030068	PHASIBEAM10F008071	830	2	0.0E0	99 %
XLOC_015619	TCONS_00030071	PHASIBEAM10F029354	329	4	2.7E-163	96 %
XLOC_015622	TCONS_00030074	XLOC_001138	461	1	1.6E-42	77 %
XLOC_015627	TCONS_00030079	PHASIBEAM10F001262	411	1	0.0E0	98 %

Loci	Major isoform	ID correlated with Vlasova et al. (2016)	Length	Hits	e-Value	similarity (mean)
XLOC_015700	TCONS_00030228	PHASIBEAM10F006232	1349	3	0.0E0	99 %
XLOC_016275	TCONS_00031340	PHASIBEAM10F016298	1512	14	0.0E0	88 %
XLOC_016344	TCONS_00031457	PHASIBEAM10F004767	1160	6	0.0E0	98 %
XLOC_016347	TCONS_00031462	XLOC_000787	694	1	0.0E0	100 %
XLOC_016429	TCONS_00031639	PHASIBEAM10F002987	867	7	0.0E0	100 %
XLOC_016478	TCONS_00031749	PHASIBEAM10F006840	816	1	0.0E0	100 %
XLOC_016543	TCONS_00031867	PHASIBEAM10F017631	1485	2	0.0E0	100 %
XLOC_016552	TCONS_00031883	PHASIBEAM10F001098	415	2	3.2E-24	93 %
XLOC_017133	TCONS_00032996	PHASIBEAM10F006223	1756	1	0.0E0	99 %
XLOC_017926	TCONS_00034570	PHASIBEAM10F006821	204	2	2.2E-102	100 %
XLOC_018049	TCONS_00034877	PHASIBEAM10F011964	1192	2	0.0E0	100 %
XLOC_018175	TCONS_00035137	XLOC_002680	283	2	8.4E-138	97 %
XLOC_018386	TCONS_00035543	PHASIBEAM10F006411	768	5	0.0E0	97 %
XLOC_018412	TCONS_00035609	PHASIBEAM10F028796	267	2	8.0E-128	95 %
XLOC_018491	TCONS_00035757	XLOC_001666	706	2	0.0E0	99 %
XLOC_018492	TCONS_00035758	PHASIBEAM10F028173	349	1	0.0E0	100 %
XLOC_018493	TCONS_00035759	PHASIBEAM10F001639	487	2	1.1E-24	94 %
XLOC_018509	TCONS_00035775	PHASIBEAM10F024670	304	7	1.8E-10	97 %
XLOC_018512	TCONS_00035778	XLOC_003851	267	1	7.9E-133	98 %
XLOC_018520	TCONS_00035786	XLOC_003572	871	1	0.0E0	100 %
XLOC_018525	TCONS_00035791	PHASIBEAM10F004830	152	1	9.9E-70	98 %
XLOC_018526	TCONS_00035792	PHASIBEAM10F002779	489	8	3.7E-44	100 %
XLOC_018535	TCONS_00035801	PHASIBEAM10F013118	144	1	1.0E-19	93 %
XLOC_018538	TCONS_00035804	XLOC_002983	340	2	1.5E-66	87 %
XLOC_018539	TCONS_00035805	PHASIBEAM10F027070	865	3	1.1E-86	85 %
XLOC_018540	TCONS_00035806	PHASIBEAM10F006316	99	1	2.2E-39	95 %
XLOC_018541	TCONS_00035807	PHASIBEAM10F026443	89	1	6.8E-34	95 %
XLOC_018547	TCONS_00035813	XLOC_002485	738	1	4.0E-120	99 %
XLOC_018556	TCONS_00035822	PHASIBEAM10F019109	263	1	6.1E-129	98 %
XLOC_018557	TCONS_00035823	PHASIBEAM10F016057	560	1	0.0E0	99 %
XLOC_018562	TCONS_00035828	PHASIBEAM10F019553	746	1	4.1E-105	99 %
XLOC_018565	TCONS_00035831	XLOC_003136	410	1	1.5E-12	97 %
XLOC_018566	TCONS_00035832	XLOC_003135	862	3	0.0E0	97 %
XLOC_018567	TCONS_00035833	PHASIBEAM10F022768	785	1	2.8E-37	98 %
XLOC_018597	TCONS_00035887	XLOC_000556	349	1	3.7E-147	94 %
XLOC_018962	TCONS_00036579	PHASIBEAM10F021291	778	4	4.0E-155	90 %
XLOC_019071	TCONS_00036785	PHASIBEAM10F028931	305	4	1.2E-141	96 %
XLOC_019468	TCONS_00037474	PHASIBEAM10F028070	645	1	0.0E0	100 %
XLOC_019575	TCONS_00037667	XLOC_004051	452	1	3.7E-163	97 %
XLOC_019717	TCONS_00037910	XLOC_003415	295	3	1.1E-136	94 %
XLOC_019746	TCONS_00037969	PHASIBEAM10F022816	1182	2	5.2E-91	99 %
XLOC_019777	TCONS_00038024	PHASIBEAM10F015023	1015	5	9.3E-113	95 %
XLOC_020237	TCONS_00038985	XLOC_000011	437	1	0.0E0	99 %
XLOC_020366	TCONS_00039247	PHASIBEAM10F011565	1691	6	0.0E0	94 %
XLOC_020457	TCONS_00039469	XLOC_001626	355	2	2.2E-159	98 %
XLOC_020491	TCONS_00039541	XLOC_002332	3199	4	0.0E0	85 %
XLOC_020570	TCONS_00039697	PHASIBEAM10F030359	263	9	3.8E-96	87 %
XLOC_020626	TCONS_00039799	PHASIBEAM10F007697	497	4	1.1E-173	93 %
XLOC_020682	TCONS_00039911	PHASIBEAM10F012516	609	7	1.7E-18	93 %
XLOC_021017	TCONS_00040556	XLOC_001002	1266	6	2.3E-179	96 %
XLOC_021296	TCONS_00041096	XLOC_001556	872	3	0.0E0	98 %
XLOC_021394	TCONS_00041270	PHASIBEAM10F008454	674	1	8.2E-82	99 %
XLOC_021553	TCONS_00041555	XLOC_001732	452	11	0.0E0	95 %

Loci	Major isoform	ID correlated with Vlasova et al. (2016)	Length	Hits	e-Value	similarity (mean)
XLOC_021557	TCONS_00041559	PHASIBEAM10F021291	1085	4	1.6E-150	98 %
XLOC_021558	TCONS_00041560	PHASIBEAM10F021291	777	2	0.0E0	97 %
XLOC_021560	TCONS_00041562	XLOC_001626	615	2	2.3E-176	97 %
XLOC_021569	TCONS_00041571	PHASIBEAM10F006170	783	1	6.4E-9	97 %
XLOC_021571	TCONS_00041573	XLOC_001658	147	8	4.4E-68	98 %
XLOC_021606	TCONS_00041608	PHASIBEAM10F014622	169	2	1.4E-83	100 %
XLOC_021616	TCONS_00041618	XLOC_003698	2300	4	0.0E0	97 %
XLOC_021617	TCONS_00041619	PHASIBEAM10F000430	381	2	0.0E0	88 %
XLOC_021626	TCONS_00041628	PHASIBEAM10F024252	722	4	0.0E0	97 %
XLOC_021629	TCONS_00041631	PHASIBEAM10F001661	561	4	3.4E-35	93 %
XLOC_021635	TCONS_00041637	PHASIBEAM10F008067	1559	4	1.6E-52	91 %
XLOC_021638	TCONS_00041640	XLOC_003509	178	11	2.5E-76	95 %
XLOC_021639	TCONS_00041641	PHASIBEAM10F010033	135	1	6.8E-51	93 %
XLOC_021641	TCONS_00041643	XLOC_001001	1005	2	0.0E0	100 %
XLOC_021644	TCONS_00041646	PHASIBEAM10F022053	1423	4	5.5E-7	94 %
XLOC_021648	TCONS_00041650	XLOC_003139	156	1	1.7E-77	100 %
XLOC_021650	TCONS_00041652	PHASIBEAM10F017572	176	2	4.5E-29	99 %
XLOC_021654	TCONS_00041656	XLOC_001556	679	2	0.0E0	94 %
XLOC_021656	TCONS_00041658	XLOC_001556	1149	3	0.0E0	98 %
XLOC_021666	TCONS_00041668	PHASIBEAM10F018221	245	1	4.4E-125	99 %
XLOC_021905	TCONS_00042125	PHASIBEAM10F010955	2904	1	0.0E0	99 %
XLOC_021926	TCONS_00042169	PHASIBEAM10F021265	1286	3	0.0E0	99 %
XLOC_022039	TCONS_00042428	XLOC_001611	5245	20	0.0E0	91 %
XLOC_022207	TCONS_00042784	XLOC_003271	1555	7	0.0E0	94 %
XLOC_022508	TCONS_00043405	XLOC_000967	333	3	2.5E-34	90 %
XLOC_023065	TCONS_00044538	XLOC_003399	1458	8	0.0E0	94 %
XLOC_023212	TCONS_00044840	PHASIBEAM10F013045	478	1	1.3E-23	98 %
XLOC_023367	TCONS_00045162	XLOC_001611	5198	20	0.0E0	91 %
XLOC_023807	TCONS_00046046	XLOC_000967	270	2	5.6E-25	97 %
XLOC_023990	TCONS_00046414	XLOC_004133	878	3	0.0E0	86 %
XLOC_024008	TCONS_00046455	XLOC_000706	2683	4	0.0E0	98 %
XLOC_024019	TCONS_00046475	XLOC_000705	242	2	9.3E-122	99 %
XLOC_024208	TCONS_00046871	PHASIBEAM10F013947	267	3	6.2E-129	94 %
XLOC_024317	TCONS_00047060	PHASIBEAM10F017575	971	10	0.0E0	94 %
XLOC_024318	TCONS_00047061	PHASIBEAM10F002497	120	2	3.5E-53	98 %
XLOC_024319	TCONS_00047062	PHASIBEAM10F020510	505	1	1.3E-63	84 %
XLOC_024328	TCONS_00047071	PHASIBEAM10F024522	891	1	0.0E0	99 %
XLOC_024329	TCONS_00047072	PHASIBEAM10F024518	149	4	7.5E-71	99 %
XLOC_024330	TCONS_00047073	XLOC_003398	905	2	0.0E0	99 %
XLOC_024331	TCONS_00047074	PHASIBEAM10F013006	495	1	1.2E-148	99 %
XLOC_024335	TCONS_00047078	PHASIBEAM10F010953	749	1	1.1E-145	95 %
XLOC_024337	TCONS_00047080	PHASIBEAM10F027025	800	8	0.0E0	99 %
XLOC_024339	TCONS_00047082	PHASIBEAM10F026720	186	2	1.6E-78	98 %
XLOC_024340	TCONS_00047083	XLOC_002105	1403	7	8.1E-80	81 %
XLOC_024343	TCONS_00047086	PHASIBEAM10F012018	328	1	3.4E-157	100 %
XLOC_024350	TCONS_00047093	XLOC_000384	441	1	0.0E0	99 %
XLOC_024352	TCONS_00047095	PHASIBEAM10F017230	243	1	1.2E-125	100 %
XLOC_024354	TCONS_00047097	PHASIBEAM10F014865	410	4	9.5E-149	99 %
XLOC_024358	TCONS_00047101	PHASIBEAM10F013440	141	1	4.2E-68	100 %
XLOC_024362	TCONS_00047105	PHASIBEAM10F013424	659	1	3.9E-55	100 %
XLOC_024364	TCONS_00047107	PHASIBEAM10F026094	206	1	8.0E-102	100 %
XLOC_024366	TCONS_00047109	XLOC_002659	714	1	1.2E-35	81 %
XLOC_024368	TCONS_00047111	PHASIBEAM10F010992	346	1	2.5E-64	95 %

Loci	Major isoform	ID correlated with Vlasova et al. (2016)	Length	Hits	e-Value	similarity (mean)
XLOC_024369	TCONS_00047112	PHASIBEAM10F003249	523	3	2.5E-11	96 %
XLOC_024374	TCONS_00047117	PHASIBEAM10F001452	418	1	0.0E0	100 %
XLOC_024375	TCONS_00047118	XLOC_000418	614	1	0.0E0	99 %
XLOC_024376	TCONS_00047119	PHASIBEAM10F001529	410	1	1.9E-26	98 %
XLOC_024381	TCONS_00047124	XLOC_002084	882	13	5.8E-164	89 %
XLOC_024382	TCONS_00047125	XLOC_002084	606	13	1.9E-142	91 %
XLOC_024383	TCONS_00047126	XLOC_002084	938	20	0.0E0	89 %
XLOC_024384	TCONS_00047127	XLOC_002730	730	2	1.4E-119	100 %
XLOC_024389	TCONS_00047132	XLOC_003578	558	4	0.0E0	98 %
XLOC_024397	TCONS_00047140	XLOC_000707	1279	6	0.0E0	96 %
XLOC_024399	TCONS_00047142	XLOC_000705	1211	2	0.0E0	99 %
XLOC_024401	TCONS_00047144	PHASIBEAM10F025477	1239	2	1.9E-125	99 %
XLOC_024404	TCONS_00047147	PHASIBEAM10F022543	298	10	2.6E-103	85 %
XLOC_024406	TCONS_00047149	XLOC_002105	714	8	1.5E-79	84 %
XLOC_024407	TCONS_00047150	XLOC_000967	693	12	0.0E0	94 %
XLOC_024408	TCONS_00047151	XLOC_000967	1316	20	0.0E0	86 %
XLOC_024468	TCONS_00047249	XLOC_000488	1358	1	0.0E0	99 %
XLOC_024876	TCONS_00047952	XLOC_002662	255	8	5.9E-129	97 %
XLOC_024953	TCONS_00048089	PHASIBEAM10F022917	2466	10	0.0E0	98 %
XLOC_025542	TCONS_00049245	XLOC_002239	255	1	3.8E-76	94 %
XLOC_025780	TCONS_00049678	XLOC_003664	209	8	3.8E-105	99 %
XLOC_025784	TCONS_00049685	PHASIBEAM10F026324	3126	12	0.0E0	95 %
XLOC_025808	TCONS_00049740	PHASIBEAM10F016298	435	9	0.0E0	97 %
XLOC_025835	TCONS_00049801	PHASIBEAM10F021008	287	3	2.8E-33	86 %
XLOC_026007	TCONS_00050118	PHASIBEAM10F017125	733	1	0.0E0	98 %
XLOC_026019	TCONS_00050150	PHASIBEAM10F008171	168	4	8.5E-81	97 %
XLOC_026120	TCONS_00050327	PHASIBEAM10F000430	247	2	1.7E-69	89 %
XLOC_026122	TCONS_00050329	PHASIBEAM10F014177	1235	2	3.6E-13	89 %
XLOC_026126	TCONS_00050333	PHASIBEAM10F002475	437	2	3.0E-119	93 %
XLOC_026142	TCONS_00050349	PHASIBEAM10F027606	943	1	0.0E0	99 %
XLOC_026145	TCONS_00050352	PHASIBEAM10F021701	166	1	1.1E-49	96 %
XLOC_026151	TCONS_00050358	PHASIBEAM10F026572	800	7	1.0E-36	96 %
XLOC_026152	TCONS_00050359	PHASIBEAM10F026573	1038	1	9.1E-143	97 %
XLOC_026155	TCONS_00050362	XLOC_002859	518	2	2.5E-180	99 %
XLOC_026156	TCONS_00050363	PHASIBEAM10F014203	580	2	4.1E-99	96 %
XLOC_026162	TCONS_00050369	XLOC_003125	176	1	2.1E-27	97 %
XLOC_026163	TCONS_00050370	XLOC_003125	394	1	1.1E-167	97 %
XLOC_026164	TCONS_00050371	PHASIBEAM10F003830	100	1	1.7E-40	96 %
XLOC_026170	TCONS_00050377	PHASIBEAM10F025719	145	6	1.2E-68	99 %
XLOC_026171	TCONS_00050378	PHASIBEAM10F002555	743	1	2.3E-142	89 %
XLOC_026172	TCONS_00050379	PHASIBEAM10F005999	1394	1	8.4E-50	88 %
XLOC_026174	TCONS_00050381	XLOC_002662	696	2	3.6E-150	97 %
XLOC_026177	TCONS_00050384	PHASIBEAM10F026324	753	7	0.0E0	99 %
XLOC_026178	TCONS_00050385	PHASIBEAM10F027958	194	1	3.9E-15	88 %
XLOC_026188	TCONS_00050395	PHASIBEAM10F000394	566	1	5.1E-108	99 %
XLOC_026192	TCONS_00050399	PHASIBEAM10F004475	460	1	0.0E0	99 %
XLOC_026193	TCONS_00050400	PHASIBEAM10F004370	979	1	0.0E0	94 %
XLOC_026201	TCONS_00050408	PHASIBEAM10F019358	1378	1	8.1E-70	88 %
XLOC_026321	TCONS_00050655	PHASIBEAM10F017682	648	1	0.0E0	90 %
XLOC_026428	TCONS_00050872	XLOC_000600	897	2	0.0E0	97 %
XLOC_026583	TCONS_00051199	PHASIBEAM10F020801	2029	3	0.0E0	96 %
XLOC_026590	TCONS_00051206	PHASIBEAM10F029210	980	2	4.2E-111	97 %
XLOC_026594	TCONS_00051211	PHASIBEAM10F014852	1049	2	1.4E-36	94 %

Loci	Major isoform	ID correlated with Vlasova et al. (2016)	Length	Hits	e-Value	similarity (mean)
XLOC_026639	TCONS_00051302	XLOC_003584	200	1	2.2E-87	98 %
XLOC_026685	TCONS_00051384	XLOC_000227	559	1	0.0E0	98 %
XLOC_026888	TCONS_00051689	PHASIBEAM10F005477	1334	5	0.0E0	96 %
XLOC_026955	TCONS_00051788	PHASIBEAM10F014646	1110	6	2.8E-123	92 %
XLOC_027693	TCONS_00053181	PHASIBEAM10F024222	346	4	2.3E-134	92 %
XLOC_027978	TCONS_00053743	PHASIBEAM10F014093	864	2	0.0E0	95 %
XLOC_028038	TCONS_00053825	PHASIBEAM10F011207	458	4	3.1E-129	94 %
XLOC_028403	TCONS_00054510	PHASIBEAM10F022680	194	3	7.7E-77	95 %
XLOC_028407	TCONS_00054514	PHASIBEAM10F019952	449	1	4.5E-23	88 %
XLOC_028413	TCONS_00054520	PHASIBEAM10F026443	99	1	1.3E-41	97 %
XLOC_028416	TCONS_00054523	PHASIBEAM10F005489	260	1	4.8E-110	95 %
XLOC_028425	TCONS_00054532	PHASIBEAM10F029210	221	1	2.7E-12	100 %
XLOC_028433	TCONS_00054540	PHASIBEAM10F018389	110	3	5.3E-46	96 %
XLOC_028434	TCONS_00054541	XLOC_003909	934	2	1.8E-114	89 %
XLOC_028442	TCONS_00054549	PHASIBEAM10F021483	581	11	0.0E0	97 %
XLOC_028443	TCONS_00054550	XLOC_003060	2039	1	0.0E0	96 %
XLOC_028444	TCONS_00054551	XLOC_002647	636	2	2.7E-101	100 %
XLOC_028451	TCONS_00054558	XLOC_002453	1678	12	2.1E-86	93 %
XLOC_028455	TCONS_00054562	PHASIBEAM10F000394	601	1	2.2E-27	100 %
XLOC_028463	TCONS_00054570	PHASIBEAM10F023047	70	1	1.1E-25	97 %
XLOC_028464	TCONS_00054571	XLOC_002270	448	1	1.7E-156	91 %
XLOC_028465	TCONS_00054572	PHASIBEAM10F000430	160	2	1.0E-74	91 %
XLOC_028467	TCONS_00054574	PHASIBEAM10F016441	595	3	1.6E-63	92 %
XLOC_028469	TCONS_00054576	PHASIBEAM10F022276	432	1	7.9E-140	87 %
XLOC_028475	TCONS_00054582	PHASIBEAM10F013373	349	15	5.6E-41	92 %
XLOC_028485	TCONS_00054592	PHASIBEAM10F016267	832	1	1.0E-61	97 %
XLOC_028489	TCONS_00054596	PHASIBEAM10F010794	710	8	0.0E0	90 %
XLOC_028507	TCONS_00054619	PHASIBEAM10F016260	578	1	0.0E0	92 %
XLOC_028509	TCONS_00054622	PHASIBEAM10F008291	784	2	0.0E0	98 %
XLOC_028514	TCONS_00054634	PHASIBEAM10F016260	637	1	0.0E0	92 %
XLOC_028539	TCONS_00054674	PHASIBEAM10F011992	1158	9	2.4E-64	93 %
XLOC_028559	TCONS_00054703	PHASIBEAM10F016267	778	1	2.1E-63	98 %
XLOC_028589	TCONS_00054741	PHASIBEAM10F010599	779	3	0.0E0	99 %
XLOC_028595	TCONS_00054747	PHASIBEAM10F017494	970	2	0.0E0	100 %
