# Supplementary material for: Genome-wide analysis of the transcriptional response to drought stress in root and leaf of common bean
Source: Genet Mol Biol. 2020 Mar 16;43(1):e20180259. doi: 10.1590/1678-4685-GMB-2018-0259 (PMC7307723; doi:10.1590/1678-4685-GMB-2018-0259)
Supplement: Supplementary file 7 [file 1415-4757-GMB-43-1-e20180259-s1.pdf]

Supplementary Material to: "Genome-wide analysis of the transcriptional response to drought stress in root and leaf of common bean"

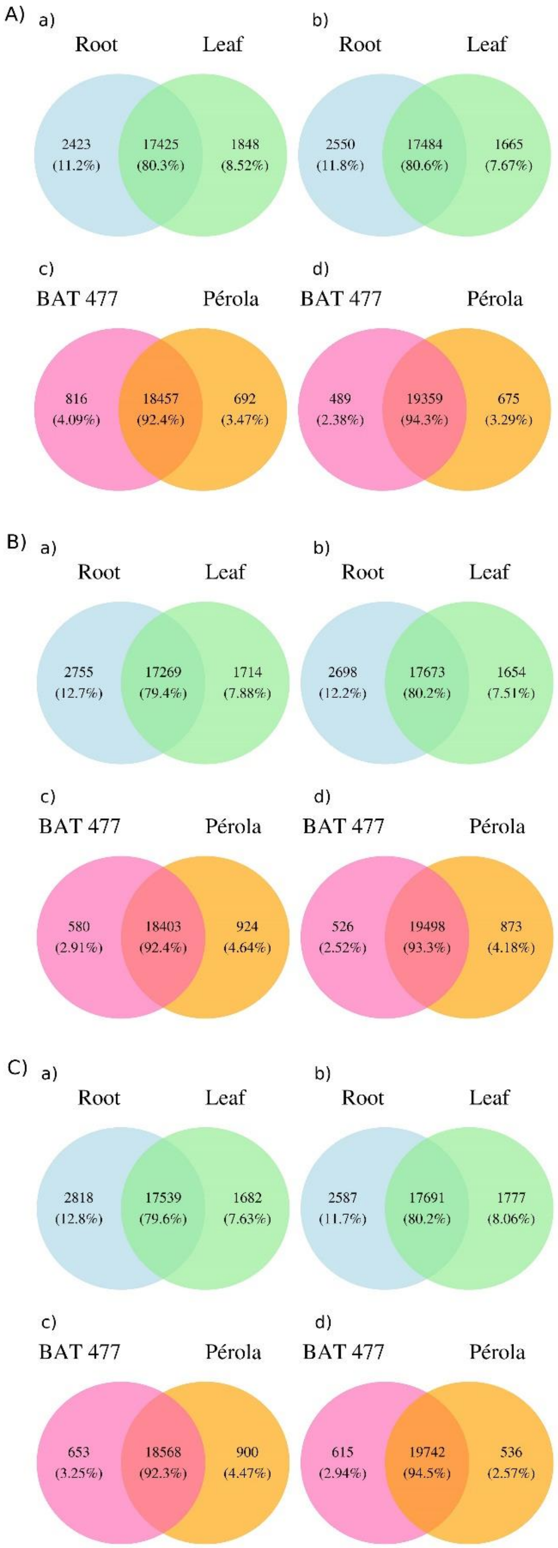

Figure S1 - Comparison of the number of genes expressed in leaf and root tissues, as well as between genotypes, in the control condition (A), after 75 min of physiological stress (B) and after 150 min of physiological stress (C). Comparisons were performed with treatments between the leaf and root of the BAT 477 genotype (a), between the leaf and root of the Pérola genotype (b), between BAT 477 and Pérola considering the leaf tissue (c) and between BAT 477 and Pérola considering the root tissue (d).
