# Supplementary material for: Genome-wide analysis of the transcriptional response to drought stress in root and leaf of common bean
Source: Genet Mol Biol. 2020 Mar 16;43(1):e20180259. doi: 10.1590/1678-4685-GMB-2018-0259 (PMC7307723; doi:10.1590/1678-4685-GMB-2018-0259)
Supplement: Supplementary file 8 [file 1415-4757-GMB-43-1-e20180259-s9.pdf]

Supplementary Material to: "Genome-wide analysis of the transcriptional response to drought stress in root and leaf of common"

Table S7 Expression values of the differential expressed genes based on fold change (FC) for each experimental comparison.

**Experimental comparison**	**Gene ID**	**Correspondent ID (Phytozome)**	**log_2(FC)[£]**	**log_2(CPM)[€]**	**FDR* (all significant at <0.01)**
comp01	XLOC_006966	new loci	-5.79	1.15	0.0078
comp01	XLOC_010340	new loci	-5.72	1.59	0.0067
comp01	XLOC_011757	new loci	-10.45	2.12	0.0004
comp01	XLOC_017321	new loci	-6.8	0.11	0.0056
comp01	XLOC_020681	new loci	-10.01	0.52	0.0013
comp01	XLOC_020682	new loci	-9.78	0.64	0.002
comp01	XLOC_021563	new loci	-9.19	-1.09	0.0078
comp01	XLOC_026888	new loci	-10.72	1.34	0.0002
comp01	XLOC_027632	new loci	-11.26	0.91	0
comp01	XLOC_028038	new loci	-9.74	0.19	0.002
comp01	XLOC_007882	Phvul.003G002500	-4.8	4.23	0.0087
comp01	XLOC_008018	Phvul.003G030500	-7.49	0.21	0.0011
comp01	XLOC_006456	Phvul.003G034200	-6.16	0.27	0.0067
comp01	XLOC_009518	Phvul.004G021200	-9.32	2.99	0
comp01	XLOC_010597	Phvul.004G058400	-6.11	1.69	0.0014
comp01	XLOC_009701	Phvul.004G058500	-9.71	-0.54	0.002
comp01	XLOC_010701	Phvul.004G076400	-10.16	7.42	0
comp01	XLOC_011726	Phvul.005G077000	-5.53	3.98	0.002
comp01	XLOC_014622	Phvul.006G033000	-11	1.19	0.0001
comp01	XLOC_022491	Phvul.009G158100	-6.56	0.11	0.002
comp01	XLOC_027984	Phvul.011G125300	5.7	0.92	0.0071
comp02	XLOC_000483	new loci	11.18	0.85	0
comp02	XLOC_002350	new loci	-10.18	-0.14	0.0001
comp02	XLOC_002827	new loci	10.72	1.61	0
comp02	XLOC_005373	new loci	-9.65	-0.33	0.0006
comp02	XLOC_006966	new loci	-5.81	1.15	0.0011
comp02	XLOC_007865	new loci	10.65	1.62	0
comp02	XLOC_009332	new loci	8.96	-0.83	0.0031
comp02	XLOC_009335	new loci	-9.18	-0.68	0.0018
comp02	XLOC_009463	new loci	8.38	1.5	0
comp02	XLOC_010325	new loci	9.75	0.44	0.0004
comp02	XLOC_010340	new loci	-11.32	1.59	0
comp02	XLOC_011258	new loci	10.15	0.91	0.0001
comp02	XLOC_011259	new loci	9.7	0.19	0.0005
comp02	XLOC_011309	new loci	-8.71	-1.29	0.006
comp02	XLOC_011313	new loci	10.3	1.09	0.0001
comp02	XLOC_011346	new loci	8.94	0.11	0.0032
comp02	XLOC_011347	new loci	9.42	0.54	0.001
comp02	XLOC_011356	new loci	-8.59	-1.55	0.0081
comp02	XLOC_011689	new loci	9.13	0.18	0.0019
comp02	XLOC_011757	new loci	-9.08	2.12	0
comp02	XLOC_011869	new loci	10.82	1.3	0

Experimental comparison	Gene ID	Correspondent ID (Phytozome)	log$_2$(FC)[£]	log$_2$(CPM)[€]	FDR* (all significant at <0.01)
comp02	XLOC_012375	new loci	-8.85	-0.31	0.0043
comp02	XLOC_012682	new loci	-10.71	0.33	0
comp02	XLOC_012683	new loci	-8.71	-1.46	0.006
comp02	XLOC_012735	new loci	8.56	-0.99	0.0087
comp02	XLOC_012777	new loci	4.72	0.96	0.0092
comp02	XLOC_013284	new loci	-11.29	1.14	0
comp02	XLOC_013644	new loci	5.95	-0.04	0.0015
comp02	XLOC_013645	new loci	5.98	-0.03	0.0014
comp02	XLOC_015572	new loci	-8.98	-1.09	0.003
comp02	XLOC_015577	new loci	-9.62	-0.05	0.0006
comp02	XLOC_015623	new loci	-6.88	-0.27	0.0009
comp02	XLOC_017321	new loci	-8.95	0.11	0.0031
comp02	XLOC_018539	new loci	9.95	1.34	0.0002
comp02	XLOC_018563	new loci	-5.69	1.18	0.0011
comp02	XLOC_020674	new loci	9.41	2.17	0.001
comp02	XLOC_020681	new loci	-9.93	0.52	0.0002
comp02	XLOC_020682	new loci	-10.33	0.64	0.0001
comp02	XLOC_021571	new loci	9.46	-0.45	0.0009
comp02	XLOC_021572	new loci	7.88	1.09	0
comp02	XLOC_021653	new loci	-6.19	-0.27	0.0055
comp02	XLOC_024208	new loci	5.15	1.68	0.0015
comp02	XLOC_024326	new loci	-8.59	-1.47	0.0081
comp02	XLOC_024327	new loci	-9.49	-0.36	0.0009
comp02	XLOC_024378	new loci	9.53	-0.66	0.0008
comp02	XLOC_024793	new loci	11.85	1.63	0
comp02	XLOC_026888	new loci	-10.86	1.34	0
comp02	XLOC_026955	new loci	10.36	0.65	0.0001
comp02	XLOC_028038	new loci	-9.52	0.19	0.0008
comp02	XLOC_028457	new loci	6.02	-0.87	0.0055
comp02	XLOC_028488	new loci	8.8	-0.12	0.0045
comp02	XLOC_028495	new loci	-5.76	-0.1	0.004
comp02	XLOC_028589	new loci	8.92	-0.4	0.0035
comp02	XLOC_000191	Phvul.001G040600	-5.02	1.05	0.0023
comp02	XLOC_000573	Phvul.001G112400	-5.02	6.22	0.0018
comp02	XLOC_000650	Phvul.001G128500	-4.58	6.36	0.0032
comp02	XLOC_002206	Phvul.001G166000	8.7	-1.19	0.006
comp02	XLOC_003411	Phvul.002G108200	7.04	0.93	0.0004
comp02	XLOC_005531	Phvul.002G200600	-4.76	2.81	0.0024
comp02	XLOC_003974	Phvul.002G228700	5.85	5.78	0.0001
comp02	XLOC_004436	Phvul.002G319000	-5.54	6.59	0.0002
comp02	XLOC_006470	Phvul.003G036600	9.42	-0.31	0.001
comp02	XLOC_006733	Phvul.003G089500	7.4	2.83	0
comp02	XLOC_007001	Phvul.003G138800	9.13	0.54	0.0019
comp02	XLOC_007002	Phvul.003G138900	11.45	2.83	0
comp02	XLOC_010333	Phvul.004G008900	-4.56	1.75	0.0073
comp02	XLOC_009518	Phvul.004G021200	-9.26	2.99	0
comp02	XLOC_009635	Phvul.004G044800	-5.08	1.87	0.0016
comp02	XLOC_010581	Phvul.004G054800	6.4	1.56	0.0001

**Experimental comparison**	**Gene ID**	**Correspondent ID (Phytozome)**	**$log_2(FC)$[£]**	**$log_2(CPM)$[€]**	**FDR* (all significant at <0.01)**
comp02	XLOC_010582	Phvul.004G054900	9.31	-0.87	0.0013
comp02	XLOC_010617	Phvul.004G061400	8.56	-0.57	0.0087
comp02	XLOC_010701	Phvul.004G076400	-10.92	7.42	0
comp02	XLOC_009909	Phvul.004G101500	-5.04	4.48	0.0011
comp02	XLOC_010110	Phvul.004G142400	9.48	5.56	0
comp02	XLOC_010111	Phvul.004G142500	13.37	3.05	0
comp02	XLOC_010112	Phvul.004G142600	9.42	6.68	0
comp02	XLOC_010113	Phvul.004G142700	12.72	2.42	0
comp02	XLOC_011124	Phvul.004G155000	-4.5	4.33	0.0043
comp02	XLOC_012336	Phvul.005G014000	5.34	1.16	0.0017
comp02	XLOC_012374	Phvul.005G021600	-9.24	-0.7	0.0015
comp02	XLOC_011579	Phvul.005G047200	-9.02	1.81	0
comp02	XLOC_011611	Phvul.005G053400	-7.3	0.05	0.0003
comp02	XLOC_011613	Phvul.005G053600	-8.59	-1.55	0.0081
comp02	XLOC_011726	Phvul.005G077000	-5.27	3.98	0.0008
comp02	XLOC_012719	Phvul.005G084700	8.02	1.61	0
comp02	XLOC_011828	Phvul.005G099500	6.64	4.44	0
comp02	XLOC_011904	Phvul.005G116500	8.98	1.93	0
comp02	XLOC_014622	Phvul.006G033000	-5.7	1.19	0.0022
comp02	XLOC_014719	Phvul.006G054500	5.82	0.46	0.0011
comp02	XLOC_013793	Phvul.006G084600	-4.66	2	0.0057
comp02	XLOC_013796	Phvul.006G084900	-4.67	1.87	0.0045
comp02	XLOC_014940	Phvul.006G096200	-5.48	-0.2	0.0084
comp02	XLOC_015235	Phvul.006G156100	-8.95	1.64	0
comp02	XLOC_017318	Phvul.007G048800	7.37	2.57	0.0001
comp02	XLOC_018469	Phvul.007G277000	8.8	1.39	0.0045
comp02	XLOC_018697	Phvul.008G028400, Phvul.008G028500	-6.67	1.35	0.0002
comp02	XLOC_018904	Phvul.008G065500	-9.36	-0.25	0.0011
comp02	XLOC_018970	Phvul.008G077500	-6.75	2.25	0.0001
comp02	XLOC_020445	Phvul.008G079700	-6.91	1.68	0.0001
comp02	XLOC_020490	Phvul.008G089400	-6.81	2.13	0.0002
comp02	XLOC_019145	Phvul.008G109300	9.16	-0.8	0.0018
comp02	XLOC_019148	Phvul.008G109600	7.78	0.84	0
comp02	XLOC_020675	Phvul.008G127000	13.81	3.57	0
comp02	XLOC_019339	Phvul.008G146400	9.34	-0.63	0.0011
comp02	XLOC_020858	Phvul.008G160400	4.69	2.85	0.0035
comp02	XLOC_019543	Phvul.008G188200	6.62	2.44	0.0001
comp02	XLOC_021207	Phvul.008G227100	5.02	2.55	0.0016
comp02	XLOC_021218	Phvul.008G229100	-8.65	3.79	0
comp02	XLOC_021349	Phvul.008G256600	-4.3	2.27	0.0095
comp02	XLOC_019956	Phvul.008G270500	-6.37	0.82	0.0004
comp02	XLOC_023458	Phvul.009G087700	7.37	2.83	0
comp02	XLOC_022504	Phvul.009G160300	-9.09	-0.76	0.0021
comp02	XLOC_022896	Phvul.009G239700	-4.33	3.81	0.0073
comp02	XLOC_024207	Phvul.009G239900	-4.63	4.8	0.003
comp02	XLOC_022900	Phvul.009G240500	-5.95	1.89	0.0008
comp02	XLOC_024439	Phvul.010G004300	8.96	1.63	0.0031

Experimental comparison	Gene ID	Correspondent ID (Phytozome)	$log_2(FC)$[£]	$log_2(CPM)$[€]	FDR* (all significant at <0.01)
comp02	XLOC_024444	Phvul.010G004900	-5.23	1.73	0.0011
comp02	XLOC_025378	Phvul.010G013700	-4.54	0.78	0.0078
comp02	XLOC_024498	Phvul.010G014100	-11.29	0.95	0
comp02	XLOC_025405	Phvul.010G019000	-9.47	-0.72	0.0009
comp02	XLOC_024530	Phvul.010G021000	-7.18	1.46	0
comp02	XLOC_024581	Phvul.010G031900	-4.84	0.15	0.0085
comp02	XLOC_025696	Phvul.010G077200	-9.12	-1.13	0.002
comp02	XLOC_025152	Phvul.010G136700	-9.59	2.88	0
comp02	XLOC_026461	Phvul.011G051300	5.85	-0.19	0.0019
comp02	XLOC_027739	Phvul.011G076400	-4.94	5.03	0.0014
comp02	XLOC_026951	Phvul.011G142600	6.31	2.98	0.0001
comp02	XLOC_028253	Phvul.011G182900	-13.08	2.7	0
comp02	XLOC_028254	Phvul.011G183000	-6.46	5.22	0.0001
comp02	XLOC_027165	Phvul.011G183300	-5.3	1.2	0.0011
comp02	XLOC_027171	Phvul.011G183900	5.4	2.93	0.0006
comp02	XLOC_027218	Phvul.011G191400	-6.6	1.31	0.0004
comp02	XLOC_027234	Phvul.011G193800	5.77	2.41	0.0003
comp02	XLOC_027235	Phvul.011G193900	6.85	2.02	0
comp02	XLOC_027313	Phvul.011G208300	-5.16	0.57	0.0019
comp02	XLOC_028638	Phvul.L006800	-4.84	1.24	0.0041
comp02	XLOC_028499	Phvul.L006900	11.17	0.84	0
comp02	XLOC_028555	Phvul.L009500	-7.83	2.45	0
Comp03.1	XLOC_002282	new loci	5.9	2.94	0.0014
Comp03.1	XLOC_002736	new loci	9.29	1.01	0.0018
Comp03.1	XLOC_007575	new loci	8.73	0.82	0.0064
Comp03.1	XLOC_007780	new loci	5.07	1.49	0.0068
Comp03.1	XLOC_008029	new loci	8.92	0.67	0.004
Comp03.1	XLOC_011298	new loci	5.03	0.95	0.0085
Comp03.1	XLOC_011343	new loci	8.76	0.99	0.0059
Comp03.1	XLOC_017343	new loci	4.28	3.71	0.0076
Comp03.1	XLOC_021652	new loci	8.79	0.69	0.0051
Comp03.1	XLOC_025825	new loci	7.48	2.18	0.0005
Comp03.1	XLOC_026139	new loci	5.13	3.15	0.0045
Comp03.1	XLOC_026270	new loci	8.54	-0.09	0.0081
Comp03.1	XLOC_027985	new loci	12.31	3.78	0
Comp03.1	XLOC_001456	Phvul.001G020300	6.3	5.7	0.0001
Comp03.1	XLOC_000186	Phvul.001G039700	5.97	6.48	0.0003
Comp03.1	XLOC_001580	Phvul.001G042200	9.24	3.62	0.002
Comp03.1	XLOC_000668	Phvul.001G131000	4.37	4.57	0.0068
Comp03.1	XLOC_000736	Phvul.001G143500	-4.94	1.4	0.0037
Comp03.1	XLOC_002111	Phvul.001G148000	4.45	2.91	0.0077
Comp03.1	XLOC_002292	Phvul.001G183300	4.53	4.19	0.0049
Comp03.1	XLOC_002314	Phvul.001G187800	6.39	1.33	0.0007
Comp03.1	XLOC_001043	Phvul.001G205900	5.43	7.01	0.0013
Comp03.1	XLOC_002429	Phvul.001G209200	9.01	0.12	0.0036
Comp03.1	XLOC_001179	Phvul.001G231900	5.04	3.81	0.007
Comp03.1	XLOC_002650	Phvul.001G255200	6.79	6	0.0021
Comp03.1	XLOC_004544	Phvul.002G009700	4.13	5.93	0.0087

Experimental comparison	Gene ID	Correspondent ID (Phytozome)	$log_2(FC)$[£]	$log_2(CPM)$[€]	FDR* (all significant at <0.01)
Comp03.1	XLOC_002924	Phvul.002G014700	5.47	3.65	0.0012
Comp03.1	XLOC_004583	Phvul.002G017500	4.23	6.21	0.0076
Comp03.1	XLOC_003080	Phvul.002G046800	5.04	7.83	0.0013
Comp03.1	XLOC_003088	Phvul.002G049000	10.58	4.39	0.0001
Comp03.1	XLOC_004803	Phvul.002G061200	-5.37	4.92	0.0006
Comp03.1	XLOC_004851	Phvul.002G072000	5.52	6.14	0.0005
Comp03.1	XLOC_003553	Phvul.002G141600	5.8	2.3	0.0013
Comp03.1	XLOC_005259	Phvul.002G148700	4.36	2.5	0.0097
Comp03.1	XLOC_005297	Phvul.002G156100	8.98	1.18	0.0037
Comp03.1	XLOC_005316	Phvul.002G159800	5.42	3.88	0.0007
Comp03.1	XLOC_003644	Phvul.002G160100	6.33	1.03	0.0059
Comp03.1	XLOC_003702	Phvul.002G170800	5.78	5.78	0.0006
Comp03.1	XLOC_003768	Phvul.002G185800	4.8	6.64	0.0021
Comp03.1	XLOC_005555	Phvul.002G204900	5.11	3.68	0.002
Comp03.1	XLOC_003887	Phvul.002G209900	5.28	0.92	0.0076
Comp03.1	XLOC_003955	Phvul.002G223400	4.69	4.09	0.0037
Comp03.1	XLOC_003974	Phvul.002G228700	5.85	5.78	0.0003
Comp03.1	XLOC_003998	Phvul.002G232800	8.54	1.47	0.0081
Comp03.1	XLOC_004194	Phvul.002G271900	6.9	5.16	0.0001
Comp03.1	XLOC_004259	Phvul.002G285800	6.61	3.25	0.0003
Comp03.1	XLOC_006070	Phvul.002G306800	4.3	5.17	0.0076
Comp03.1	XLOC_006360	Phvul.003G012600	7.27	4.06	0.0001
Comp03.1	XLOC_006376	Phvul.003G016400	5.23	1.13	0.0048
Comp03.1	XLOC_006536	Phvul.003G051800	5.86	4.94	0.0003
Comp03.1	XLOC_008161	Phvul.003G055500	5.63	1.56	0.0039
Comp03.1	XLOC_008262	Phvul.003G074000	5.75	7.82	0.0003
Comp03.1	XLOC_006675	Phvul.003G079800	4.58	3.94	0.0068
Comp03.1	XLOC_006719	Phvul.003G087500	7.26	2.4	0.0007
Comp03.1	XLOC_008378	Phvul.003G099100	4.36	4.6	0.0073
Comp03.1	XLOC_008432	Phvul.003G109200	5.79	4.43	0.0004
Comp03.1	XLOC_006889	Phvul.003G120100	5.05	2.62	0.0031
Comp03.1	XLOC_008502	Phvul.003G124100	4.27	5.34	0.0071
Comp03.1	XLOC_006933	Phvul.003G126600	-5.25	2.84	0.0091
Comp03.1	XLOC_008534	Phvul.003G131500	6.05	6.57	0.0002
Comp03.1	XLOC_008555	Phvul.003G135700	10.72	2.4	0.0001
Comp03.1	XLOC_007045	Phvul.003G147100	8.95	0.31	0.0039
Comp03.1	XLOC_008713	Phvul.003G167700	4.79	2.88	0.0042
Comp03.1	XLOC_007272	Phvul.003G193400	5.36	4.13	0.0069
Comp03.1	XLOC_008916	Phvul.003G207000	5.27	1.54	0.0057
Comp03.1	XLOC_008923	Phvul.003G209000	9.09	6.16	0.0029
Comp03.1	XLOC_008941	Phvul.003G212600	6.23	3.01	0.0005
Comp03.1	XLOC_007402	Phvul.003G217200	4.96	3	0.0069
Comp03.1	XLOC_009014	Phvul.003G228000	4.32	6.03	0.0069
Comp03.1	XLOC_007492	Phvul.003G233400	4.92	5.35	0.0019
Comp03.1	XLOC_009058	Phvul.003G238700	5.46	3.02	0.0024
Comp03.1	XLOC_007554	Phvul.003G243400	5.68	3.32	0.0007
Comp03.1	XLOC_007606	Phvul.003G252500	7.73	3.57	0.0003
Comp03.1	XLOC_007655	Phvul.003G259200	4.65	2.76	0.0048

Experimental comparison	Gene ID	Correspondent ID (Phytozome)	log_2(FC)[£]	log_2(CPM)[€]	FDR* (all significant at <0.01)
Comp03.1	XLOC_009180	Phvul.003G268500	6.55	4.26	0.0005
Comp03.1	XLOC_009181	Phvul.003G268600	4.25	5.46	0.0077
Comp03.1	XLOC_009483	Phvul.004G013400	4.41	3.05	0.0076
Comp03.1	XLOC_009520	Phvul.004G021400	4.91	6.43	0.0017
Comp03.1	XLOC_009642	Phvul.004G045800	-5.06	3	0.0021
Comp03.1	XLOC_009680	Phvul.004G055200	4.3	5.13	0.0069
Comp03.1	XLOC_010686	Phvul.004G072900	5.09	1.82	0.0049
Comp03.1	XLOC_010783	Phvul.004G090300	8.83	1.2	0.0049
Comp03.1	XLOC_010789	Phvul.004G092100	6.17	6.35	0.0001
Comp03.1	XLOC_010014	Phvul.004G123600	-4.44	3.1	0.0069
Comp03.1	XLOC_010063	Phvul.004G134400	5.03	3.41	0.002
Comp03.1	XLOC_011021	Phvul.004G134800	5.9	3.78	0.0003
Comp03.1	XLOC_011055	Phvul.004G142000	-5.1	3.44	0.0015
Comp03.1	XLOC_012343	Phvul.005G015900	-4.85	4.64	0.002
Comp03.1	XLOC_011431	Phvul.005G016400	4.29	6.61	0.0069
Comp03.1	XLOC_011600	Phvul.005G051600	5.19	8.2	0.0009
Comp03.1	XLOC_011616	Phvul.005G054000	7.17	2.97	0.0009
Comp03.1	XLOC_011734	Phvul.005G080600	4.17	7.09	0.008
Comp03.1	XLOC_011754	Phvul.005G084500	4.46	8.3	0.0048
Comp03.1	XLOC_012872	Phvul.005G111700	5.51	3.57	0.0008
Comp03.1	XLOC_012056	Phvul.005G145600	4.54	2.75	0.0086
Comp03.1	XLOC_013102	Phvul.005G155800	5.64	7.16	0.0004
Comp03.1	XLOC_012103	Phvul.005G156700	9.46	1.4	0.0012
Comp03.1	XLOC_013113	Phvul.005G158500	5.23	5.94	0.0009
Comp03.1	XLOC_012185	Phvul.005G173000	5.78	7.34	0.0003
Comp03.1	XLOC_013188	Phvul.005G173600	4.61	7.75	0.0034
Comp03.1	XLOC_014485	Phvul.006G001900	6.94	1.79	0.0015
Comp03.1	XLOC_013350	Phvul.006G004800	5.11	3.85	0.0014
Comp03.1	XLOC_014603	Phvul.006G028700	8	3.79	0
Comp03.1	XLOC_013600	Phvul.006G045800	5.48	6.39	0.0022
Comp03.1	XLOC_013746	Phvul.006G075600	5.9	7.18	0.0002
Comp03.1	XLOC_013747	Phvul.006G075900	6.13	3.42	0.0082
Comp03.1	XLOC_013758	Phvul.006G078800	4.87	5.86	0.0019
Comp03.1	XLOC_013759	Phvul.006G078900	4.68	3.63	0.0036
Comp03.1	XLOC_013761	Phvul.006G079100	5.28	0.17	0.0076
Comp03.1	XLOC_013767	Phvul.006G080600	5.05	6.17	0.0013
Comp03.1	XLOC_013787	Phvul.006G083700	4.69	3.86	0.0039
Comp03.1	XLOC_014876	Phvul.006G083800	6.41	3.25	0.0003
Comp03.1	XLOC_015019	Phvul.006G112300	4.38	2.53	0.0089
Comp03.1	XLOC_014051	Phvul.006G137300	4.24	5.9	0.0075
Comp03.1	XLOC_014082	Phvul.006G143300	5.29	1.5	0.0043
Comp03.1	XLOC_015402	Phvul.006G189100	5.16	1.36	0.0069
Comp03.1	XLOC_014335	Phvul.006G194600	5.06	4.83	0.0015
Comp03.1	XLOC_015531	Phvul.006G215400	6.83	1.25	0.002
Comp03.1	XLOC_014444	Phvul.006G216700	4.41	6.74	0.0076
Comp03.1	XLOC_015771	Phvul.007G025700	4.35	4.34	0.0072
Comp03.1	XLOC_017232	Phvul.007G031000	6.51	2.29	0.0042
Comp03.1	XLOC_017238	Phvul.007G032200	4.51	2.38	0.0092

Experimental comparison	Gene ID	Correspondent ID (Phytozome)	$log_2(FC)^£$	$log_2(CPM)^€$	FDR* (all significant at <0.01)
Comp03.1	XLOC_015798	Phvul.007G032800	4.27	5.13	0.0076
Comp03.1	XLOC_017251	Phvul.007G034500	6.76	3.54	0.0002
Comp03.1	XLOC_015864	Phvul.007G045400	-4.21	2.55	0.0092
Comp03.1	XLOC_017332	Phvul.007G050300	5.04	2.9	0.0032
Comp03.1	XLOC_015991	Phvul.007G071500	8.66	0.22	0.0069
Comp03.1	XLOC_017546	Phvul.007G092400	5.43	2.46	0.0022
Comp03.1	XLOC_016304	Phvul.007G132300	8.51	1.12	0.0087
Comp03.1	XLOC_016318	Phvul.007G135600	4.33	5.31	0.0068
Comp03.1	XLOC_018073	Phvul.007G195100	4.29	3.44	0.0084
Comp03.1	XLOC_016643	Phvul.007G198700	4.55	6.05	0.0069
Comp03.1	XLOC_016657	Phvul.007G201100	5.1	2.68	0.0029
Comp03.1	XLOC_018143	Phvul.007G208900	4.54	4.18	0.0069
Comp03.1	XLOC_018152	Phvul.007G210400	10.13	2	0.0003
Comp03.1	XLOC_016702	Phvul.007G211400	-4.21	4.38	0.0077
Comp03.1	XLOC_016775	Phvul.007G222500	5.95	3.98	0.0004
Comp03.1	XLOC_016808	Phvul.007G228500	4.72	3.68	0.0037
Comp03.1	XLOC_016875	Phvul.007G242000	8.89	0.56	0.0045
Comp03.1	XLOC_016955	Phvul.007G259500	5.43	3.23	0.0013
Comp03.1	XLOC_017043	Phvul.007G275700	4.33	2.24	0.0089
Comp03.1	XLOC_018466	Phvul.007G276200	4.54	5	0.0086
Comp03.1	XLOC_017050	Phvul.007G277000	5.5	1.82	0.0012
Comp03.1	XLOC_018647	Phvul.008G016600	4.38	5.58	0.0068
Comp03.1	XLOC_018658	Phvul.008G019600	-5.09	0.65	0.0069
Comp03.1	XLOC_020188	Phvul.008G023600	5.86	2.33	0.0012
Comp03.1	XLOC_018785	Phvul.008G043400	10.81	3.62	0.0001
Comp03.1	XLOC_020378	Phvul.008G062700	-5.02	0.43	0.0076
Comp03.1	XLOC_020387	Phvul.008G068300	6.28	1.48	0.0009
Comp03.1	XLOC_019087	Phvul.008G098200	6.48	4.88	0.0001
Comp03.1	XLOC_020537	Phvul.008G098500	5.76	6.31	0.0003
Comp03.1	XLOC_019110	Phvul.008G103600	-4.47	3.2	0.0051
Comp03.1	XLOC_019143	Phvul.008G109100	4.49	7.04	0.0045
Comp03.1	XLOC_020614	Phvul.008G113700	4.7	6.77	0.0031
Comp03.1	XLOC_020939	Phvul.008G175500	7.9	4.15	0
Comp03.1	XLOC_021042	Phvul.008G194600	5.09	2.26	0.0068
Comp03.1	XLOC_019663	Phvul.008G213800	4.33	4.94	0.0068
Comp03.1	XLOC_019664	Phvul.008G213900	4.95	2.05	0.0042
Comp03.1	XLOC_019720	Phvul.008G223500	5.6	6.42	0.0004
Comp03.1	XLOC_021267	Phvul.008G238200	4.4	4.66	0.0076
Comp03.1	XLOC_021953	Phvul.009G054300	9.76	1.98	0.0006
Comp03.1	XLOC_023283	Phvul.009G054400	4.68	3.36	0.0051
Comp03.1	XLOC_022077	Phvul.009G078300	4.58	6.64	0.0051
Comp03.1	XLOC_023458	Phvul.009G087700	-4.8	2.83	0.0049
Comp03.1	XLOC_023493	Phvul.009G094000	6.44	4.39	0.0001
Comp03.1	XLOC_023568	Phvul.009G108700	5.43	4.37	0.0014
Comp03.1	XLOC_022233	Phvul.009G111500	4.21	7.63	0.0076
Comp03.1	XLOC_022274	Phvul.009G118800	4.8	4.56	0.0025
Comp03.1	XLOC_022385	Phvul.009G137900	4.27	4.29	0.008
Comp03.1	XLOC_022461	Phvul.009G152500	4.64	4.82	0.0076

Experimental comparison	Gene ID	Correspondent ID (Phytozome)	$log_2(FC)^£$	$log_2(CPM)^€$	FDR* (all significant at <0.01)
Comp03.1	XLOC_023784	Phvul.009G155800	8.58	3.07	0.0077
Comp03.1	XLOC_023812	Phvul.009G162000	6.18	3.78	0.0076
Comp03.1	XLOC_025333	Phvul.010G003400	4.34	4.84	0.0068
Comp03.1	XLOC_025354	Phvul.010G008800	4.87	6.69	0.0019
Comp03.1	XLOC_025630	Phvul.010G063800	5	6.61	0.0015
Comp03.1	XLOC_025020	Phvul.010G111000	7.42	1.77	0.0005
Comp03.1	XLOC_025154	Phvul.010G137300	5.73	2.27	0.0032
Comp03.1	XLOC_025194	Phvul.010G144200	5.45	6.84	0.0005
Comp03.1	XLOC_025196	Phvul.010G144600	6.87	3.5	0.0001
Comp03.1	XLOC_026071	Phvul.010G156200	4.7	4.61	0.0069
Comp03.1	XLOC_027424	Phvul.011G012600	8.98	1.33	0.0037
Comp03.1	XLOC_026618	Phvul.011G081800	-4.43	1.97	0.0079
Comp03.1	XLOC_026695	Phvul.011G099300	10.66	2.1	0.0001
Comp03.1	XLOC_026860	Phvul.011G127100	6.97	3.69	0.0001
Comp03.1	XLOC_026949	Phvul.011G142300	4.46	5.86	0.0049
Comp03.1	XLOC_026983	Phvul.011G147800	4.23	7.56	0.0076
Comp03.1	XLOC_027057	Phvul.011G160600	-4.87	0.56	0.0071
Comp03.1	XLOC_027170	Phvul.011G183800	5.28	4.42	0.0076
Comp03.1	XLOC_027207	Phvul.011G189300	7.35	4.01	0.0001
Comp03.1	XLOC_027211	Phvul.011G189900	4.48	4.9	0.0054
Comp03.1	XLOC_027249	Phvul.011G197000	9.87	3.7	0.0005
Comp03.1	XLOC_028531	Phvul.L008100	4.74	4.05	0.0047
Comp03.2	XLOC_000376	Phvul.001G076700	-5.54	8.13	0.0033
Comp03.2	XLOC_017043	Phvul.007G275700	-6.47	2.24	0.0022
Comp03.2	XLOC_017050	Phvul.007G277000	-11.28	1.82	0.0001
Comp03.3	XLOC_000683	new loci	-5.82	-0.73	0.0033
Comp03.3	XLOC_000689	new loci	5.53	1.47	0.0005
Comp03.3	XLOC_002282	new loci	7.33	2.94	0
Comp03.3	XLOC_002627	new loci	6.92	0.77	0.0002
Comp03.3	XLOC_002736	new loci	10.12	1.01	0.0001
Comp03.3	XLOC_002758	new loci	5.76	-0.24	0.0038
Comp03.3	XLOC_002766	new loci	9.03	-0.35	0.0009
Comp03.3	XLOC_002785	new loci	11.29	0.92	0
Comp03.3	XLOC_002793	new loci	5.59	-0.85	0.0058
Comp03.3	XLOC_002805	new loci	10.4	0.5	0
Comp03.3	XLOC_002817	new loci	5.14	1.03	0.0012
Comp03.3	XLOC_002839	new loci	9.27	-0.5	0.0005
Comp03.3	XLOC_003618	new loci	9.63	-0.31	0.0002
Comp03.3	XLOC_003707	new loci	8.74	-0.86	0.0017
Comp03.3	XLOC_003885	new loci	9.4	-0.22	0.0004
Comp03.3	XLOC_005217	new loci	6.88	1.08	0.0001
Comp03.3	XLOC_006229	new loci	9.71	-0.18	0.0002
Comp03.3	XLOC_006239	new loci	9.12	-0.72	0.0007
Comp03.3	XLOC_006245	new loci	5.04	0.64	0.0028
Comp03.3	XLOC_006252	new loci	-4.99	0	0.0068
Comp03.3	XLOC_006277	new loci	4.7	-0.14	0.0067
Comp03.3	XLOC_006286	new loci	8.92	-0.49	0.0011
Comp03.3	XLOC_007575	new loci	9.78	0.82	0.0001

Experimental comparison	Gene ID	Correspondent ID (Phytozome)	$log_2(FC)^£$	$log_2(CPM)^€$	FDR* (all significant at <0.01)
Comp03.3	XLOC_007780	new loci	5.65	1.49	0.0005
Comp03.3	XLOC_008029	new loci	10.66	0.67	0
Comp03.3	XLOC_008533	new loci	9.95	-0.04	0.0001
Comp03.3	XLOC_009318	new loci	4.93	-0.21	0.0075
Comp03.3	XLOC_009320	new loci	5.87	-0.72	0.0028
Comp03.3	XLOC_009321	new loci	8.08	-1.02	0.0078
Comp03.3	XLOC_009355	new loci	4.26	4.47	0.0024
Comp03.3	XLOC_009372	new loci	5.11	1.06	0.0016
Comp03.3	XLOC_009383	new loci	9.36	-0.52	0.0004
Comp03.3	XLOC_009385	new loci	8.72	-0.63	0.0018
Comp03.3	XLOC_009404	new loci	8.66	-0.98	0.002
Comp03.3	XLOC_009407	new loci	10.75	0.34	0
Comp03.3	XLOC_009410	new loci	10.65	0.62	0
Comp03.3	XLOC_009423	new loci	8.45	-1.43	0.0032
Comp03.3	XLOC_011273	new loci	9.05	-0.7	0.0008
Comp03.3	XLOC_011298	new loci	5.64	0.95	0.0007
Comp03.3	XLOC_011299	new loci	8.35	-1.21	0.0041
Comp03.3	XLOC_011335	new loci	6.07	0.15	0.0005
Comp03.3	XLOC_011339	new loci	9.18	-0.03	0.0006
Comp03.3	XLOC_011342	new loci	10.28	0.34	0
Comp03.3	XLOC_011343	new loci	10.97	0.99	0
Comp03.3	XLOC_011353	new loci	8.72	-0.99	0.0018
Comp03.3	XLOC_013251	new loci	8.87	-0.72	0.0013
Comp03.3	XLOC_013260	new loci	8.87	-0.87	0.0013
Comp03.3	XLOC_013266	new loci	6.9	1.23	0.0002
Comp03.3	XLOC_013267	new loci	5.91	-0.3	0.0025
Comp03.3	XLOC_013810	new loci	4.85	1.25	0.0016
Comp03.3	XLOC_015573	new loci	8.74	-1.13	0.0017
Comp03.3	XLOC_015575	new loci	9.66	-0.19	0.0002
Comp03.3	XLOC_016045	new loci	6.09	3.37	0
Comp03.3	XLOC_016155	new loci	8.27	-0.98	0.0049
Comp03.3	XLOC_017342	new loci	8.48	-0.91	0.0029
Comp03.3	XLOC_017343	new loci	4.38	3.71	0.002
Comp03.3	XLOC_018503	new loci	8.8	-0.62	0.0015
Comp03.3	XLOC_018504	new loci	8.42	-1.23	0.0035
Comp03.3	XLOC_018505	new loci	8.99	-0.85	0.001
Comp03.3	XLOC_018527	new loci	9.78	-0.18	0.0001
Comp03.3	XLOC_018567	new loci	9.41	-0.39	0.0004
Comp03.3	XLOC_020771	new loci	5.51	0.2	0.0017
Comp03.3	XLOC_020934	new loci	8.82	-0.34	0.0014
Comp03.3	XLOC_021551	new loci	9.48	-0.56	0.0003
Comp03.3	XLOC_021564	new loci	-4.94	0.41	0.0014
Comp03.3	XLOC_021565	new loci	-5.83	0.1	0.0005
Comp03.3	XLOC_021576	new loci	8.99	-1.12	0.001
Comp03.3	XLOC_021586	new loci	9.2	-0.77	0.0006
Comp03.3	XLOC_021600	new loci	8.85	-1.31	0.0013
Comp03.3	XLOC_021642	new loci	10.83	0.72	0
Comp03.3	XLOC_021644	new loci	8.85	-1.24	0.0013

Experimental comparison	Gene ID	Correspondent ID (Phytozome)	log_2(FC)[£]	log_2(CPM)[€]	FDR* (all significant at <0.01)
Comp03.3	XLOC_021652	new loci	10.43	0.69	0
Comp03.3	XLOC_021926	new loci	4.99	3.54	0.0005
Comp03.3	XLOC_024325	new loci	8.03	-1	0.0085
Comp03.3	XLOC_024340	new loci	4.51	0.73	0.0054
Comp03.3	XLOC_024356	new loci	9.59	-0.2	0.0002
Comp03.3	XLOC_024362	new loci	6.67	0.35	0.0004
Comp03.3	XLOC_024387	new loci	11.44	1.31	0
Comp03.3	XLOC_024388	new loci	8.35	-1.16	0.0041
Comp03.3	XLOC_024404	new loci	9.25	-0.55	0.0005
Comp03.3	XLOC_024405	new loci	10.2	0.23	0
Comp03.3	XLOC_025114	new loci	4.61	0.4	0.0055
Comp03.3	XLOC_025625	new loci	8.66	-1.01	0.002
Comp03.3	XLOC_025627	new loci	8.35	-0.93	0.0041
Comp03.3	XLOC_025787	new loci	9.01	-0.48	0.0009
Comp03.3	XLOC_025825	new loci	8.23	2.18	0
Comp03.3	XLOC_026121	new loci	8.31	-1.25	0.0045
Comp03.3	XLOC_026123	new loci	9.29	-0.55	0.0005
Comp03.3	XLOC_026129	new loci	9.31	-0.84	0.0005
Comp03.3	XLOC_026139	new loci	5.59	3.15	0.0003
Comp03.3	XLOC_026160	new loci	4.57	2.45	0.0017
Comp03.3	XLOC_026172	new loci	8.82	-0.74	0.0014
Comp03.3	XLOC_026190	new loci	-5.53	0.79	0.0067
Comp03.3	XLOC_026270	new loci	9.25	-0.09	0.0005
Comp03.3	XLOC_027985	new loci	13.57	3.78	0
Comp03.3	XLOC_028432	new loci	8.03	-0.92	0.0085
Comp03.3	XLOC_028476	new loci	9.43	-0.23	0.0003
Comp03.3	XLOC_000030	Phvul.001G005800	4.52	2.83	0.0017
Comp03.3	XLOC_001436	Phvul.001G016600	8.8	-0.49	0.0015
Comp03.3	XLOC_001456	Phvul.001G020300	6.09	5.7	0
Comp03.3	XLOC_000125	Phvul.001G025300	4.03	1.51	0.0072
Comp03.3	XLOC_001508	Phvul.001G029200	5.12	3.3	0.0023
Comp03.3	XLOC_000142	Phvul.001G029500	3.99	1.44	0.0097
Comp03.3	XLOC_000181	Phvul.001G038500	5.2	0.57	0.0019
Comp03.3	XLOC_000186	Phvul.001G039700	6.11	6.48	0
Comp03.3	XLOC_000192	Phvul.001G040800	4.47	3.27	0.0018
Comp03.3	XLOC_001580	Phvul.001G042200	10.44	3.62	0
Comp03.3	XLOC_001591	Phvul.001G043500	-4.49	2.11	0.0021
Comp03.3	XLOC_000253	Phvul.001G054500	-5.72	-0.85	0.0042
Comp03.3	XLOC_000305	Phvul.001G064000	4.82	-0.15	0.0098
Comp03.3	XLOC_001744	Phvul.001G075400	5.2	7.44	0.0003
Comp03.3	XLOC_000378	Phvul.001G077000	3.95	5.6	0.0054
Comp03.3	XLOC_000401	Phvul.001G081000	-4.66	2.66	0.0012
Comp03.3	XLOC_000427	Phvul.001G085900	-3.76	4.24	0.0088
Comp03.3	XLOC_001808	Phvul.001G088200	3.77	5.41	0.008
Comp03.3	XLOC_001823	Phvul.001G091600	4.38	0.34	0.0074
Comp03.3	XLOC_001889	Phvul.001G103600	5.12	3.27	0.0009
Comp03.3	XLOC_000551	Phvul.001G108300	5.47	3.02	0.0004
Comp03.3	XLOC_000573	Phvul.001G112400	3.97	6.22	0.0048

Experimental comparison	Gene ID	Correspondent ID (Phytozome)	$log_2(FC)$[£]	$log_2(CPM)$[€]	FDR* (all significant at <0.01)
Comp03.3	XLOC_000629	Phvul.001G124700	4.54	3.56	0.002
Comp03.3	XLOC_000630	Phvul.001G124800	4.92	6.34	0.0005
Comp03.3	XLOC_000668	Phvul.001G131000	5.55	4.57	0.0001
Comp03.3	XLOC_000712	Phvul.001G139400	-4.48	0.93	0.0052
Comp03.3	XLOC_002068	Phvul.001G140000	3.92	2.22	0.0071
Comp03.3	XLOC_000736	Phvul.001G143500	-7.5	1.4	0
Comp03.3	XLOC_002095	Phvul.001G145600	5.44	4.88	0.0002
Comp03.3	XLOC_002111	Phvul.001G148000	5.6	2.91	0.0002
Comp03.3	XLOC_002112	Phvul.001G148100	6.19	0.59	0.0004
Comp03.3	XLOC_000772	Phvul.001G151900	8.56	1.73	0
Comp03.3	XLOC_000773	Phvul.001G152100	4.02	3.97	0.0047
Comp03.3	XLOC_002157	Phvul.001G156500	4.74	5.93	0.0007
Comp03.3	XLOC_000797	Phvul.001G156900	4.04	7.14	0.0041
Comp03.3	XLOC_000813	Phvul.001G160100	6.2	1.51	0.0001
Comp03.3	XLOC_000815	Phvul.001G160200	3.91	3.13	0.0073
Comp03.3	XLOC_000821	Phvul.001G161000	4.38	3.81	0.002
Comp03.3	XLOC_000843	Phvul.001G165200	-6.45	5.27	0.0008
Comp03.3	XLOC_002203	Phvul.001G165700	5.46	2.89	0.0002
Comp03.3	XLOC_002212	Phvul.001G166700	6.23	0.07	0.0012
Comp03.3	XLOC_000863	Phvul.001G169300	4.31	2.63	0.003
Comp03.3	XLOC_000883	Phvul.001G173300	5.32	0.09	0.0014
Comp03.3	XLOC_000920	Phvul.001G180500	4.59	5.79	0.0012
Comp03.3	XLOC_000932	Phvul.001G182300	5.66	1.64	0.0004
Comp03.3	XLOC_002292	Phvul.001G183300	4.73	4.19	0.0009
Comp03.3	XLOC_002314	Phvul.001G187800	5.99	1.33	0.0006
Comp03.3	XLOC_002388	Phvul.001G201300	-3.85	3.92	0.007
Comp03.3	XLOC_002389	Phvul.001G201500	9.74	-0.07	0.0002
Comp03.3	XLOC_001043	Phvul.001G205900	6.69	7.01	0
Comp03.3	XLOC_001048	Phvul.001G206700	5.95	0.49	0.0006
Comp03.3	XLOC_002421	Phvul.001G207500	4.05	1.76	0.0083
Comp03.3	XLOC_002429	Phvul.001G209200	8.82	0.12	0.0014
Comp03.3	XLOC_002466	Phvul.001G215900	-5.45	1.47	0.0004
Comp03.3	XLOC_001149	Phvul.001G226300	3.77	4.4	0.0092
Comp03.3	XLOC_001179	Phvul.001G231900	6.16	3.81	0.0001
Comp03.3	XLOC_002552	Phvul.001G235000	-5.04	0.31	0.002
Comp03.3	XLOC_002611	Phvul.001G247300	5.79	0.37	0.0005
Comp03.3	XLOC_002628	Phvul.001G249600	3.75	5.31	0.0084
Comp03.3	XLOC_001274	Phvul.001G250900	-3.97	4.64	0.0061
Comp03.3	XLOC_002650	Phvul.001G255200	7.6	6	0
Comp03.3	XLOC_002678	Phvul.001G258900	6.48	4.05	0
Comp03.3	XLOC_002712	Phvul.001G265800	5.09	3.67	0.0004
Comp03.3	XLOC_004521	Phvul.002G004100	6.07	-0.47	0.0017
Comp03.3	XLOC_004535	Phvul.002G008300	4.25	1.07	0.0072
Comp03.3	XLOC_004544	Phvul.002G009700	4.3	5.93	0.002
Comp03.3	XLOC_002924	Phvul.002G014700	6.2	3.65	0
Comp03.3	XLOC_004583	Phvul.002G017500	4.87	6.21	0.0005
Comp03.3	XLOC_004604	Phvul.002G021800	6.57	2.8	0.0005
Comp03.3	XLOC_003024	Phvul.002G033100	5.42	1.98	0.0003

Experimental comparison	Gene ID	Correspondent ID (Phytozome)	$log_2(FC)^£$	$log_2(CPM)^€$	FDR* (all significant at <0.01)
Comp03.3	XLOC_004673	Phvul.002G035900	4.01	2.95	0.0071
Comp03.3	XLOC_003080	Phvul.002G046800	6.01	7.83	0
Comp03.3	XLOC_003088	Phvul.002G049000	10.38	4.39	0
Comp03.3	XLOC_003121	Phvul.002G055600	5.28	2.76	0.0003
Comp03.3	XLOC_004803	Phvul.002G061200	-6.58	4.92	0
Comp03.3	XLOC_004806	Phvul.002G061900	-4.83	6.97	0.0006
Comp03.3	XLOC_004817	Phvul.002G063500	-4.04	2.54	0.0054
Comp03.3	XLOC_004846	Phvul.002G070300	4.34	0.41	0.0082
Comp03.3	XLOC_004851	Phvul.002G072000	5.84	6.14	0.0001
Comp03.3	XLOC_004864	Phvul.002G075200	4.85	6.13	0.0006
Comp03.3	XLOC_004896	Phvul.002G081400	-5.42	0.72	0.0093
Comp03.3	XLOC_003286	Phvul.002G085000	5.33	-0.15	0.0027
Comp03.3	XLOC_004936	Phvul.002G089700	4.95	-0.37	0.0071
Comp03.3	XLOC_003371	Phvul.002G099700	-5.24	4.67	0.0002
Comp03.3	XLOC_004996	Phvul.002G103300	8.2	2.93	0.0059
Comp03.3	XLOC_005081	Phvul.002G117700	4.37	6.09	0.0018
Comp03.3	XLOC_003553	Phvul.002G141600	6.98	2.3	0
Comp03.3	XLOC_005259	Phvul.002G148700	4.49	2.5	0.0021
Comp03.3	XLOC_003619	Phvul.002G154900	3.91	4.37	0.0065
Comp03.3	XLOC_003621	Phvul.002G155400	4.29	3.69	0.0025
Comp03.3	XLOC_005297	Phvul.002G156100	10.3	1.18	0
Comp03.3	XLOC_005316	Phvul.002G159800	5.1	3.88	0.0004
Comp03.3	XLOC_003644	Phvul.002G160100	6.2	1.03	0.0013
Comp03.3	XLOC_005321	Phvul.002G160600	4.56	6.03	0.0012
Comp03.3	XLOC_005337	Phvul.002G163900	4.47	3.75	0.0024
Comp03.3	XLOC_005348	Phvul.002G166600	4.97	0.85	0.0022
Comp03.3	XLOC_003702	Phvul.002G170800	5.93	5.78	0.0001
Comp03.3	XLOC_005428	Phvul.002G181200	-4.02	3.04	0.0048
Comp03.3	XLOC_003768	Phvul.002G185800	4.25	6.64	0.0024
Comp03.3	XLOC_005555	Phvul.002G204900	6.1	3.68	0
Comp03.3	XLOC_005579	Phvul.002G209400	5.17	5.34	0.0003
Comp03.3	XLOC_003883	Phvul.002G209500	5.8	5.61	0.0001
Comp03.3	XLOC_003887	Phvul.002G209900	5.98	0.92	0.0006
Comp03.3	XLOC_003890	Phvul.002G210400	4.54	5.28	0.0013
Comp03.3	XLOC_003891	Phvul.002G210500	4.53	6.31	0.0069
Comp03.3	XLOC_003915	Phvul.002G215100	4.45	0.82	0.0064
Comp03.3	XLOC_003943	Phvul.002G220300	8.94	-0.48	0.0011
Comp03.3	XLOC_003955	Phvul.002G223400	5.76	4.09	0.0001
Comp03.3	XLOC_003974	Phvul.002G228700	6.44	5.78	0
Comp03.3	XLOC_003990	Phvul.002G231400	5.6	4.47	0.0014
Comp03.3	XLOC_003998	Phvul.002G232800	8.74	1.47	0.0017
Comp03.3	XLOC_005730	Phvul.002G240100	-5.53	-0.23	0.0067
Comp03.3	XLOC_004045	Phvul.002G241400	4.49	7.77	0.0013
Comp03.3	XLOC_004126	Phvul.002G256900	5.45	0.97	0.0007
Comp03.3	XLOC_005889	Phvul.002G270900	-4.09	5.13	0.0038
Comp03.3	XLOC_004194	Phvul.002G271900	7.82	5.16	0
Comp03.3	XLOC_005904	Phvul.002G274900	6.25	0.12	0.0012
Comp03.3	XLOC_004254	Phvul.002G284700	3.79	4.07	0.0084

Experimental comparison	Gene ID	Correspondent ID (Phytozome)	$log_2(FC)^£$	$log_2(CPM)^€$	FDR* (all significant at <0.01)
Comp03.3	XLOC_004259	Phvul.002G285800	7.57	3.25	0
Comp03.3	XLOC_006070	Phvul.002G306800	4.49	5.17	0.0016
Comp03.3	XLOC_004372	Phvul.002G308000	-4.67	2.88	0.0013
Comp03.3	XLOC_004411	Phvul.002G314600	-5.07	1.09	0.0014
Comp03.3	XLOC_006113	Phvul.002G316900	4.85	2.97	0.0019
Comp03.3	XLOC_004423	Phvul.002G317000	5	4.64	0.0007
Comp03.3	XLOC_004438	Phvul.002G319200	8.74	-0.89	0.0017
Comp03.3	XLOC_004442	Phvul.002G320400	9.03	0.3	0.0009
Comp03.3	XLOC_006136	Phvul.002G322300	4.63	1.22	0.0019
Comp03.3	XLOC_004459	Phvul.002G324400	5.45	0.5	0.0011
Comp03.3	XLOC_006169	Phvul.002G326600	4.29	7.09	0.0021
Comp03.3	XLOC_004494	Phvul.002G332300	4.59	5.4	0.0017
Comp03.3	XLOC_007906	Phvul.003G007300	-5.09	2.95	0.0052
Comp03.3	XLOC_006360	Phvul.003G012600	8.8	4.06	0
Comp03.3	XLOC_006370	Phvul.003G015600	9.97	0.05	0.0001
Comp03.3	XLOC_006376	Phvul.003G016400	5.11	1.13	0.0016
Comp03.3	XLOC_007993	Phvul.003G024700	5.66	-0.63	0.0048
Comp03.3	XLOC_006420	Phvul.003G027000	10.34	0.31	0
Comp03.3	XLOC_006522	Phvul.003G048700	4.49	3.94	0.0016
Comp03.3	XLOC_006536	Phvul.003G051800	6.61	4.94	0
Comp03.3	XLOC_006537	Phvul.003G051900	5.9	1.35	0.0004
Comp03.3	XLOC_008161	Phvul.003G055500	7.3	1.56	0
Comp03.3	XLOC_006568	Phvul.003G057900	-5.03	0.83	0.0013
Comp03.3	XLOC_008176	Phvul.003G058700	10.48	0.5	0
Comp03.3	XLOC_006632	Phvul.003G071000	8.24	1.07	0.0054
Comp03.3	XLOC_008262	Phvul.003G074000	6.27	7.82	0
Comp03.3	XLOC_008278	Phvul.003G077800	4.4	1.7	0.0037
Comp03.3	XLOC_006675	Phvul.003G079800	6.99	3.94	0
Comp03.3	XLOC_006719	Phvul.003G087500	7.6	2.4	0
Comp03.3	XLOC_006721	Phvul.003G087700	4.86	1.8	0.0011
Comp03.3	XLOC_006750	Phvul.003G093100	-4.01	2.63	0.0058
Comp03.3	XLOC_008375	Phvul.003G098600	6.58	-0.35	0.0005
Comp03.3	XLOC_008378	Phvul.003G099100	5.02	4.6	0.0005
Comp03.3	XLOC_009346	Phvul.003G108400	4.88	0.15	0.0043
Comp03.3	XLOC_008430	Phvul.003G109000	4	6.17	0.0045
Comp03.3	XLOC_008432	Phvul.003G109200	7.28	4.43	0
Comp03.3	XLOC_008435	Phvul.003G109600	7.24	0.63	0.0001
Comp03.3	XLOC_006839	Phvul.003G109800	6.13	-0.09	0.0015
Comp03.3	XLOC_006882	Phvul.003G118300	4.54	0.74	0.0035
Comp03.3	XLOC_006889	Phvul.003G120100	5.5	2.62	0.0003
Comp03.3	XLOC_006894	Phvul.003G120900	4.41	2.5	0.0022
Comp03.3	XLOC_008496	Phvul.003G121900	6.03	3.57	0.0019
Comp03.3	XLOC_006905	Phvul.003G122700	-5.02	-0.26	0.0032
Comp03.3	XLOC_008502	Phvul.003G124100	4.79	5.34	0.0007
Comp03.3	XLOC_006933	Phvul.003G126600	-4.59	2.84	0.0048
Comp03.3	XLOC_006942	Phvul.003G128400	-8.88	0.45	0.0012
Comp03.3	XLOC_008532	Phvul.003G131400	6.71	5.75	0.0004
Comp03.3	XLOC_008534	Phvul.003G131500	6.88	6.57	0

Experimental comparison	Gene ID	Correspondent ID (Phytozome)	$log_2(FC)$[£]	$log_2(CPM)$[€]	FDR* (all significant at <0.01)
Comp03.3	XLOC_008555	Phvul.003G135700	11.69	2.4	0
Comp03.3	XLOC_007012	Phvul.003G140800	4.22	5.16	0.0028
Comp03.3	XLOC_008609	Phvul.003G146700	3.9	3.08	0.0071
Comp03.3	XLOC_007133	Phvul.003G164000	5.02	5.66	0.0008
Comp03.3	XLOC_008713	Phvul.003G167700	4.78	2.88	0.0011
Comp03.3	XLOC_007173	Phvul.003G173600	5.71	1.94	0.0042
Comp03.3	XLOC_008772	Phvul.003G176800	-4.72	3.71	0.0035
Comp03.3	XLOC_008783	Phvul.003G178300	-3.76	3.23	0.009
Comp03.3	XLOC_007212	Phvul.003G181300	10.53	0.78	0
Comp03.3	XLOC_007309	Phvul.003G199200	3.83	7.06	0.0072
Comp03.3	XLOC_008916	Phvul.003G207000	6.19	1.54	0.0002
Comp03.3	XLOC_008923	Phvul.003G209000	10.49	6.16	0
Comp03.3	XLOC_008941	Phvul.003G212600	6.65	3.01	0
Comp03.3	XLOC_007402	Phvul.003G217200	7.81	3	0
Comp03.3	XLOC_007442	Phvul.003G225700	-3.96	4.46	0.0053
Comp03.3	XLOC_009014	Phvul.003G228000	5.67	6.03	0.0001
Comp03.3	XLOC_007463	Phvul.003G228700	-3.96	7.58	0.0049
Comp03.3	XLOC_007492	Phvul.003G233400	5.63	5.35	0.0001
Comp03.3	XLOC_009047	Phvul.003G235800	-5.3	2.3	0.0005
Comp03.3	XLOC_009058	Phvul.003G238700	6.62	3.02	0
Comp03.3	XLOC_007554	Phvul.003G243400	6.35	3.32	0
Comp03.3	XLOC_007576	Phvul.003G247100	5.51	0.28	0.0071
Comp03.3	XLOC_009094	Phvul.003G247400	3.81	5.04	0.0073
Comp03.3	XLOC_007606	Phvul.003G252500	8.97	3.57	0
Comp03.3	XLOC_007655	Phvul.003G259200	4.76	2.76	0.0011
Comp03.3	XLOC_007682	Phvul.003G264600	-4.33	4.83	0.002
Comp03.3	XLOC_009172	Phvul.003G267000	-3.86	2.28	0.0083
Comp03.3	XLOC_009180	Phvul.003G268500	7.28	4.26	0
Comp03.3	XLOC_009181	Phvul.003G268600	4.96	5.46	0.0005
Comp03.3	XLOC_009186	Phvul.003G269500	3.88	4.94	0.0069
Comp03.3	XLOC_009252	Phvul.003G283100	4.37	5.95	0.0018
Comp03.3	XLOC_007797	Phvul.003G287400	8.69	2.45	0.0019
Comp03.3	XLOC_007798	Phvul.003G287500	4.81	5.69	0.0011
Comp03.3	XLOC_009449	Phvul.004G005400	-5.17	4.12	0.0012
Comp03.3	XLOC_009483	Phvul.004G013400	4.83	3.05	0.0008
Comp03.3	XLOC_010374	Phvul.004G015500	9.1	-0.18	0.0007
Comp03.3	XLOC_009520	Phvul.004G021400	5.27	6.43	0.0002
Comp03.3	XLOC_009608	Phvul.004G037700	4.49	6.08	0.0022
Comp03.3	XLOC_009642	Phvul.004G045800	-5.17	3	0.0004
Comp03.3	XLOC_010527	Phvul.004G046200	4.16	4.04	0.004
Comp03.3	XLOC_010549	Phvul.004G049900	4.59	2.95	0.0044
Comp03.3	XLOC_009680	Phvul.004G055200	4.01	5.13	0.0047
Comp03.3	XLOC_010588	Phvul.004G056700	4.06	3.1	0.007
Comp03.3	XLOC_010656	Phvul.004G068400	5.64	4.41	0.0001
Comp03.3	XLOC_009753	Phvul.004G070500	4.71	3.94	0.0014
Comp03.3	XLOC_010686	Phvul.004G072900	5.42	1.82	0.0005
Comp03.3	XLOC_010724	Phvul.004G080200	3.75	8.47	0.0083
Comp03.3	XLOC_009847	Phvul.004G090200	4.77	4.38	0.0011

Experimental comparison	Gene ID	Correspondent ID (Phytozome)	$log_2(FC)^£$	$log_2(CPM)^€$	FDR* (all significant at <0.01)
Comp03.3	XLOC_010783	Phvul.004G090300	10.48	1.2	0
Comp03.3	XLOC_010789	Phvul.004G092100	6.97	6.35	0
Comp03.3	XLOC_010866	Phvul.004G106500	-4.11	0.78	0.0076
Comp03.3	XLOC_010878	Phvul.004G108900	4.38	5.44	0.0019
Comp03.3	XLOC_009959	Phvul.004G111600	-4.39	3.64	0.0018
Comp03.3	XLOC_010913	Phvul.004G114300	6.64	-0.15	0.0005
Comp03.3	XLOC_009968	Phvul.004G114600	4.86	7.46	0.0005
Comp03.3	XLOC_010921	Phvul.004G115300	4.69	4	0.0012
Comp03.3	XLOC_009986	Phvul.004G119400	6.42	0.44	0.0008
Comp03.3	XLOC_010014	Phvul.004G123600	-4.77	3.1	0.0011
Comp03.3	XLOC_010966	Phvul.004G124200	4.19	0.74	0.0076
Comp03.3	XLOC_011005	Phvul.004G131400	-5.19	3.14	0.0004
Comp03.3	XLOC_010063	Phvul.004G134400	5.41	3.41	0.0002
Comp03.3	XLOC_011021	Phvul.004G134800	5.79	3.78	0.0001
Comp03.3	XLOC_010087	Phvul.004G138500	4.15	8.13	0.0029
Comp03.3	XLOC_011037	Phvul.004G138600	5.03	4.82	0.0004
Comp03.3	XLOC_010108	Phvul.004G141300	5.39	-0.82	0.0086
Comp03.3	XLOC_011055	Phvul.004G142000	-6.94	3.44	0
Comp03.3	XLOC_011065	Phvul.004G143600	4.54	8.8	0.0012
Comp03.3	XLOC_011088	Phvul.004G148400	3.86	6.34	0.0065
Comp03.3	XLOC_010164	Phvul.004G152900	9.82	0.38	0.0001
Comp03.3	XLOC_010254	Phvul.004G173400	10.37	0.56	0
Comp03.3	XLOC_010265	Phvul.004G175200	-4.23	4.41	0.0026
Comp03.3	XLOC_011382	Phvul.005G005400	4.32	1.41	0.0045
Comp03.3	XLOC_011383	Phvul.005G005500	6.33	0.16	0.001
Comp03.3	XLOC_011420	Phvul.005G014600	4.17	1.4	0.0048
Comp03.3	XLOC_012340	Phvul.005G014900	3.84	4.98	0.0071
Comp03.3	XLOC_012343	Phvul.005G015900	-6.13	4.64	0
Comp03.3	XLOC_011431	Phvul.005G016400	4.4	6.61	0.0017
Comp03.3	XLOC_011457	Phvul.005G021900	-4.35	4.4	0.0019
Comp03.3	XLOC_011459	Phvul.005G022500	7.87	0.86	0
Comp03.3	XLOC_011486	Phvul.005G029700	6.11	0.02	0.0016
Comp03.3	XLOC_012451	Phvul.005G035800	5.48	-0.44	0.0075
Comp03.3	XLOC_011600	Phvul.005G051600	5.84	8.2	0
Comp03.3	XLOC_011609	Phvul.005G053000	4.74	1.31	0.0024
Comp03.3	XLOC_012537	Phvul.005G053900	6.45	3.51	0
Comp03.3	XLOC_011616	Phvul.005G054000	10.06	2.97	0
Comp03.3	XLOC_011617	Phvul.005G054100	7.75	1.16	0
Comp03.3	XLOC_012538	Phvul.005G054200	10.12	1.88	0.0001
Comp03.3	XLOC_012545	Phvul.005G055000	3.87	5.42	0.0067
Comp03.3	XLOC_012568	Phvul.005G058900	-3.75	5.67	0.0084
Comp03.3	XLOC_012570	Phvul.005G059100	-3.75	4.98	0.0083
Comp03.3	XLOC_011663	Phvul.005G065100	4.75	1.82	0.0013
Comp03.3	XLOC_012654	Phvul.005G075400	4.17	2.92	0.0039
Comp03.3	XLOC_012655	Phvul.005G075500	4.95	5.51	0.0005
Comp03.3	XLOC_012673	Phvul.005G078100	4.33	5.3	0.002
Comp03.3	XLOC_012676	Phvul.005G078400	7.13	2.45	0.0001
Comp03.3	XLOC_011734	Phvul.005G080600	4.66	7.09	0.0009

Experimental comparison	Gene ID	Correspondent ID (Phytozome)	$log_2(FC)^£$	$log_2(CPM)^€$	FDR* (all significant at <0.01)
Comp03.3	XLOC_011754	Phvul.005G084500	5.75	8.3	0.0001
Comp03.3	XLOC_012779	Phvul.005G097000	-4.19	1.01	0.0092
Comp03.3	XLOC_011828	Phvul.005G099500	-3.77	4.44	0.0083
Comp03.3	XLOC_012850	Phvul.005G108100	8.87	0.68	0.0013
Comp03.3	XLOC_012857	Phvul.005G109000	4.45	5.48	0.0083
Comp03.3	XLOC_012872	Phvul.005G111700	6.44	3.57	0
Comp03.3	XLOC_012877	Phvul.005G112700	-4.51	0.6	0.0041
Comp03.3	XLOC_011905	Phvul.005G116600	4.71	4.5	0.0009
Comp03.3	XLOC_012915	Phvul.005G121600	8.16	1.83	0.0065
Comp03.3	XLOC_012056	Phvul.005G145600	5.16	2.75	0.0006
Comp03.3	XLOC_013076	Phvul.005G151600	5.48	-1.02	0.0075
Comp03.3	XLOC_013102	Phvul.005G155800	5.82	7.16	0.0001
Comp03.3	XLOC_012103	Phvul.005G156700	11.29	1.4	0
Comp03.3	XLOC_013113	Phvul.005G158500	5.93	5.94	0
Comp03.3	XLOC_012155	Phvul.005G167800	3.97	3.76	0.0072
Comp03.3	XLOC_012177	Phvul.005G171900	4.58	5.52	0.0013
Comp03.3	XLOC_012185	Phvul.005G173000	7.26	7.34	0
Comp03.3	XLOC_013188	Phvul.005G173600	4.82	7.75	0.0006
Comp03.3	XLOC_013191	Phvul.005G174800	6.08	4.03	0.0002
Comp03.3	XLOC_012236	Phvul.005G181700	6.27	7.37	0.0001
Comp03.3	XLOC_014485	Phvul.006G001900	7.1	1.79	0.0002
Comp03.3	XLOC_013350	Phvul.006G004800	5.3	3.85	0.0002
Comp03.3	XLOC_013366	Phvul.006G007700	8.38	-1.06	0.0038
Comp03.3	XLOC_013409	Phvul.006G014000	-4.13	1.42	0.007
Comp03.3	XLOC_014547	Phvul.006G016900	5.25	5.77	0.0005
Comp03.3	XLOC_014583	Phvul.006G023100	9.22	3.8	0.0006
Comp03.3	XLOC_013461	Phvul.006G023900	7.06	2.78	0
Comp03.3	XLOC_014603	Phvul.006G028700	9.49	3.79	0
Comp03.3	XLOC_013524	Phvul.006G033300	5.48	1.08	0.0075
Comp03.3	XLOC_013583	Phvul.006G043100	4.24	6.33	0.0024
Comp03.3	XLOC_013600	Phvul.006G045800	5.11	6.39	0.0012
Comp03.3	XLOC_014717	Phvul.006G054200	-4.14	4.4	0.0034
Comp03.3	XLOC_014748	Phvul.006G058600	4.59	3.56	0.0016
Comp03.3	XLOC_014761	Phvul.006G060700	4.39	4.61	0.002
Comp03.3	XLOC_013746	Phvul.006G075600	7.17	7.18	0
Comp03.3	XLOC_013747	Phvul.006G075900	7.93	3.42	0
Comp03.3	XLOC_013758	Phvul.006G078800	4.73	5.86	0.0008
Comp03.3	XLOC_013759	Phvul.006G078900	4.42	3.63	0.0019
Comp03.3	XLOC_013763	Phvul.006G079500	5.41	4.84	0.0002
Comp03.3	XLOC_013764	Phvul.006G079600	6.63	4.73	0
Comp03.3	XLOC_013767	Phvul.006G080600	5.87	6.17	0
Comp03.3	XLOC_014864	Phvul.006G080700	4	4.56	0.0048
Comp03.3	XLOC_013787	Phvul.006G083700	5.15	3.86	0.0003
Comp03.3	XLOC_014876	Phvul.006G083800	7.1	3.25	0
Comp03.3	XLOC_013790	Phvul.006G084300	4.82	2.12	0.0098
Comp03.3	XLOC_013796	Phvul.006G084900	4.75	1.87	0.0059
Comp03.3	XLOC_013859	Phvul.006G100700	5.1	0.23	0.0024
Comp03.3	XLOC_013868	Phvul.006G102300	4.36	5.14	0.0021

Experimental comparison	Gene ID	Correspondent ID (Phytozome)	log_2(FC)[£]	log_2(CPM)[€]	FDR* (all significant at <0.01)
Comp03.3	XLOC_015008	Phvul.006G109000	-4.27	3.17	0.0028
Comp03.3	XLOC_015019	Phvul.006G112300	4.48	2.53	0.0025
Comp03.3	XLOC_013951	Phvul.006G117300	-6.35	2.34	0.0001
Comp03.3	XLOC_014037	Phvul.006G133600	-3.75	7.2	0.0086
Comp03.3	XLOC_014051	Phvul.006G137300	4.02	5.9	0.0043
Comp03.3	XLOC_015152	Phvul.006G139100	-4.1	3.59	0.004
Comp03.3	XLOC_014082	Phvul.006G143300	5.82	1.5	0.0003
Comp03.3	XLOC_015186	Phvul.006G146900	8.66	-0.88	0.002
Comp03.3	XLOC_015293	Phvul.006G168000	4.57	3.88	0.0013
Comp03.3	XLOC_015304	Phvul.006G171000	4.01	5.62	0.0046
Comp03.3	XLOC_014230	Phvul.006G173600	4.92	1.59	0.0012
Comp03.3	XLOC_015337	Phvul.006G176500	5.84	0.5	0.0008
Comp03.3	XLOC_014252	Phvul.006G178300	-4.79	2.93	0.0008
Comp03.3	XLOC_015402	Phvul.006G189100	5.97	1.36	0.0003
Comp03.3	XLOC_015407	Phvul.006G190400	4.98	5.24	0.0005
Comp03.3	XLOC_015410	Phvul.006G191200	4.33	6.08	0.0022
Comp03.3	XLOC_014335	Phvul.006G194600	5.82	4.83	0.0001
Comp03.3	XLOC_015484	Phvul.006G205300	9.07	4.15	0.0008
Comp03.3	XLOC_015501	Phvul.006G209000	3.85	1.81	0.01
Comp03.3	XLOC_014408	Phvul.006G210300	4.36	3.68	0.0021
Comp03.3	XLOC_015531	Phvul.006G215400	7.45	1.25	0.0001
Comp03.3	XLOC_014437	Phvul.006G215700	4.33	4.79	0.0022
Comp03.3	XLOC_014444	Phvul.006G216700	4.72	6.74	0.0012
Comp03.3	XLOC_015733	Phvul.007G018700	11.07	1.05	0
Comp03.3	XLOC_015771	Phvul.007G025700	4.77	4.34	0.0008
Comp03.3	XLOC_017232	Phvul.007G031000	8.11	2.29	0
Comp03.3	XLOC_017238	Phvul.007G032200	5.62	2.38	0.0002
Comp03.3	XLOC_015798	Phvul.007G032800	4.4	5.13	0.0018
Comp03.3	XLOC_017251	Phvul.007G034500	8.58	3.54	0
Comp03.3	XLOC_015864	Phvul.007G045400	-6.68	2.55	0
Comp03.3	XLOC_017304	Phvul.007G046200	9.18	-0.89	0.0006
Comp03.3	XLOC_017315	Phvul.007G048500	4.83	4.75	0.0031
Comp03.3	XLOC_017325	Phvul.007G049600	4.04	1.45	0.0075
Comp03.3	XLOC_017332	Phvul.007G050300	5.1	2.9	0.0007
Comp03.3	XLOC_017333	Phvul.007G050400	5.81	4	0.0001
Comp03.3	XLOC_017369	Phvul.007G057900	8.99	-0.75	0.001
Comp03.3	XLOC_017375	Phvul.007G058500	4.18	3.77	0.0037
Comp03.3	XLOC_017389	Phvul.007G062500	7.22	2.16	0
Comp03.3	XLOC_015978	Phvul.007G067700	-4.12	2.04	0.0054
Comp03.3	XLOC_017427	Phvul.007G070000	-6.03	2.27	0.0003
Comp03.3	XLOC_015991	Phvul.007G071500	8.82	0.22	0.0014
Comp03.3	XLOC_016049	Phvul.007G082000	4.49	4.46	0.0016
Comp03.3	XLOC_017546	Phvul.007G092400	6.51	2.46	0
Comp03.3	XLOC_016114	Phvul.007G096100	-4.81	3.15	0.0008
Comp03.3	XLOC_017633	Phvul.007G109000	4.43	4.57	0.0017
Comp03.3	XLOC_016196	Phvul.007G113300	-5.08	0.29	0.0018
Comp03.3	XLOC_016200	Phvul.007G114200	-4.41	7.1	0.0016
Comp03.3	XLOC_016212	Phvul.007G116300	-4.48	2.77	0.0018

Experimental comparison	Gene ID	Correspondent ID (Phytozome)	$log_2(FC)^£$	$log_2(CPM)^€$	FDR* (all significant at <0.01)
Comp03.3	XLOC_017682	Phvul.007G118300	5.61	-0.01	0.0054
Comp03.3	XLOC_017691	Phvul.007G120200	-5.19	5.01	0.0003
Comp03.3	XLOC_017695	Phvul.007G120600	4.43	1.07	0.0034
Comp03.3	XLOC_017731	Phvul.007G128100	5.93	1.07	0.0004
Comp03.3	XLOC_016304	Phvul.007G132300	11.35	1.12	0
Comp03.3	XLOC_016318	Phvul.007G135600	5.3	5.31	0.0002
Comp03.3	XLOC_016391	Phvul.007G149000	-3.81	4.43	0.0084
Comp03.3	XLOC_016441	Phvul.007G158600	-4.21	4.32	0.0027
Comp03.3	XLOC_017909	Phvul.007G164400	5.26	0.58	0.0017
Comp03.3	XLOC_016486	Phvul.007G167000	4.63	3.89	0.0011
Comp03.3	XLOC_016569	Phvul.007G184800	5.43	0.87	0.0011
Comp03.3	XLOC_016612	Phvul.007G192700	7.01	0.19	0.0002
Comp03.3	XLOC_016613	Phvul.007G192800	8.35	-0.4	0.0041
Comp03.3	XLOC_018065	Phvul.007G193700	10.68	0.44	0
Comp03.3	XLOC_018066	Phvul.007G193800	4.03	2.16	0.0052
Comp03.3	XLOC_018067	Phvul.007G193900	5.93	-0.87	0.0024
Comp03.3	XLOC_018073	Phvul.007G195100	4.53	3.44	0.0015
Comp03.3	XLOC_016636	Phvul.007G197500	3.82	5.79	0.0072
Comp03.3	XLOC_016643	Phvul.007G198700	5.55	6.05	0.0002
Comp03.3	XLOC_016657	Phvul.007G201100	6.02	2.68	0.0001
Comp03.3	XLOC_016666	Phvul.007G203400	4.84	8.26	0.0006
Comp03.3	XLOC_016689	Phvul.007G208800	4.14	1.51	0.0085
Comp03.3	XLOC_018143	Phvul.007G208900	4.4	4.18	0.0027
Comp03.3	XLOC_018152	Phvul.007G210400	11.86	2	0
Comp03.3	XLOC_016702	Phvul.007G211400	-4.76	4.38	0.0007
Comp03.3	XLOC_016707	Phvul.007G211900	5.23	2.24	0.0004
Comp03.3	XLOC_016775	Phvul.007G222500	5.76	3.98	0.0001
Comp03.3	XLOC_018203	Phvul.007G222900	-6.02	5.13	0
Comp03.3	XLOC_016777	Phvul.007G223100	8.69	0.16	0.0019
Comp03.3	XLOC_016805	Phvul.007G228200	5.01	0.01	0.0061
Comp03.3	XLOC_016806	Phvul.007G228300	4.64	-0.05	0.0052
Comp03.3	XLOC_016808	Phvul.007G228500	4.69	3.68	0.001
Comp03.3	XLOC_018237	Phvul.007G230600	6.69	-0.26	0.0004
Comp03.3	XLOC_018243	Phvul.007G231800	5.59	0.58	0.0008
Comp03.3	XLOC_016875	Phvul.007G242000	9.53	0.56	0.0003
Comp03.3	XLOC_018326	Phvul.007G248500	-3.88	3.17	0.0069
Comp03.3	XLOC_016906	Phvul.007G248700	-5.6	1.28	0.0004
Comp03.3	XLOC_016946	Phvul.007G257300	-3.78	2.7	0.01
Comp03.3	XLOC_018388	Phvul.007G259400	4.86	6.7	0.0007
Comp03.3	XLOC_016955	Phvul.007G259500	5.77	3.23	0.0001
Comp03.3	XLOC_017009	Phvul.007G269400	4.33	2.95	0.0025
Comp03.3	XLOC_017028	Phvul.007G273000	4.77	2.87	0.0027
Comp03.3	XLOC_018466	Phvul.007G276200	5.33	5	0.0004
Comp03.3	XLOC_017045	Phvul.007G276400	4.23	5.27	0.0036
Comp03.3	XLOC_018468	Phvul.007G276700	4.51	4.95	0.0014
Comp03.3	XLOC_017049	Phvul.007G276900	-5.63	1.41	0.0002
Comp03.3	XLOC_018479	Phvul.007G278900	4.45	5.4	0.0015
Comp03.3	XLOC_020109	Phvul.008G007900	3.76	6.1	0.0082

Experimental comparison	Gene ID	Correspondent ID (Phytozome)	$log_2(FC)^£$	$log_2(CPM)^€$	FDR* (all significant at <0.01)
Comp03.3	XLOC_020111	Phvul.008G008100	3.68	7.83	0.0098
Comp03.3	XLOC_018603	Phvul.008G008400	-5.5	5.34	0.0002
Comp03.3	XLOC_018605	Phvul.008G008800	3.76	3.4	0.0095
Comp03.3	XLOC_018625	Phvul.008G012600	3.77	3.32	0.0093
Comp03.3	XLOC_020148	Phvul.008G016200	8.08	2.28	0.0078
Comp03.3	XLOC_020154	Phvul.008G017100	9.62	0.46	0.0002
Comp03.3	XLOC_018658	Phvul.008G019600	-4.52	0.65	0.004
Comp03.3	XLOC_020188	Phvul.008G023600	7.02	2.33	0
Comp03.3	XLOC_020192	Phvul.008G024200	5.32	1.11	0.0006
Comp03.3	XLOC_020213	Phvul.008G028000	4.31	0.69	0.0056
Comp03.3	XLOC_018706	Phvul.008G029600, Phvul.008G029700	3.77	4.96	0.0092
Comp03.3	XLOC_020224	Phvul.008G031900	4.56	1.79	0.0026
Comp03.3	XLOC_020240	Phvul.008G036500	8.48	-0.95	0.0029
Comp03.3	XLOC_020260	Phvul.008G040500	6.07	2.56	0
Comp03.3	XLOC_018780	Phvul.008G042300	4.32	2.74	0.0024
Comp03.3	XLOC_018785	Phvul.008G043400	13.24	3.62	0
Comp03.3	XLOC_018791	Phvul.008G045100	4.19	3.05	0.0045
Comp03.3	XLOC_020295	Phvul.008G046500	8.42	-0.77	0.0035
Comp03.3	XLOC_020340	Phvul.008G055200	-8.48	0.5	0.0032
Comp03.3	XLOC_018852	Phvul.008G058400	7.02	2.58	0
Comp03.3	XLOC_018907	Phvul.008G065800	5.34	2.66	0.0003
Comp03.3	XLOC_020387	Phvul.008G068300	7.07	1.48	0
Comp03.3	XLOC_020395	Phvul.008G069500	-4.24	4.42	0.0026
Comp03.3	XLOC_019030	Phvul.008G087600	-4.01	5.42	0.0044
Comp03.3	XLOC_020493	Phvul.008G089900	-4.19	1.75	0.0058
Comp03.3	XLOC_019087	Phvul.008G098200	6.78	4.88	0
Comp03.3	XLOC_020537	Phvul.008G098500	6.81	6.31	0
Comp03.3	XLOC_019110	Phvul.008G103600	-7.28	3.2	0
Comp03.3	XLOC_020571	Phvul.008G104600	9.24	-0.43	0.0005
Comp03.3	XLOC_019143	Phvul.008G109100	4.56	7.04	0.0011
Comp03.3	XLOC_020614	Phvul.008G113700	5.92	6.77	0
Comp03.3	XLOC_019228	Phvul.008G123300	-3.93	2.84	0.0075
Comp03.3	XLOC_019281	Phvul.008G134800	4.92	4.93	0.0005
Comp03.3	XLOC_019389	Phvul.008G155000	4.26	4.18	0.0033
Comp03.3	XLOC_020885	Phvul.008G164100	-6.65	-0.24	0.0005
Comp03.3	XLOC_020933	Phvul.008G174800	6.01	-0.03	0.002
Comp03.3	XLOC_020939	Phvul.008G175500	9.5	4.15	0
Comp03.3	XLOC_019489	Phvul.008G176100	9.93	2.87	0.0001
Comp03.3	XLOC_020944	Phvul.008G176900	4.46	3.05	0.0024
Comp03.3	XLOC_020998	Phvul.008G186800	6.23	-0.21	0.0012
Comp03.3	XLOC_021006	Phvul.008G188100	3.71	7.34	0.009
Comp03.3	XLOC_019563	Phvul.008G191600	5.98	0.62	0.0006
Comp03.3	XLOC_021042	Phvul.008G194600	6.7	2.26	0
Comp03.3	XLOC_019637	Phvul.008G207900	4.6	6.59	0.0086
Comp03.3	XLOC_019663	Phvul.008G213800	4.74	4.94	0.0008
Comp03.3	XLOC_019664	Phvul.008G213900	5.67	2.05	0.0002
Comp03.3	XLOC_019666	Phvul.008G214200	4.07	8.15	0.0042

Experimental comparison	Gene ID	Correspondent ID (Phytozome)	log_2(FC)[£]	log_2(CPM)[€]	FDR* (all significant at <0.01)
Comp03.3	XLOC_019720	Phvul.008G223500	6.19	6.42	0
Comp03.3	XLOC_021190	Phvul.008G224100	5.35	1.71	0.0009
Comp03.3	XLOC_019729	Phvul.008G225600	4.23	4.49	0.0027
Comp03.3	XLOC_021212	Phvul.008G228100	6.49	5.94	0.0006
Comp03.3	XLOC_021267	Phvul.008G238200	5.56	4.66	0.0002
Comp03.3	XLOC_021293	Phvul.008G242900	4.84	4.09	0.0007
Comp03.3	XLOC_021316	Phvul.008G249500	4.15	4.64	0.0032
Comp03.3	XLOC_019864	Phvul.008G251000	-3.87	3.02	0.008
Comp03.3	XLOC_019868	Phvul.008G251400	9.7	-0.29	0.0002
Comp03.3	XLOC_021324	Phvul.008G251500	4.68	2.1	0.0021
Comp03.3	XLOC_021395	Phvul.008G264000	6.3	0.31	0.001
Comp03.3	XLOC_021442	Phvul.008G273800	9.69	2.88	0.0002
Comp03.3	XLOC_019981	Phvul.008G275400	4.14	3.73	0.0038
Comp03.3	XLOC_020029	Phvul.008G285200	-4.49	1.01	0.0062
Comp03.3	XLOC_021532	Phvul.008G290700	4.44	4.68	0.0017
Comp03.3	XLOC_021740	Phvul.009G012700	3.72	5.82	0.0089
Comp03.3	XLOC_021773	Phvul.009G017700	-4.7	3.54	0.0011
Comp03.3	XLOC_023153	Phvul.009G027700	5.42	0.73	0.0081
Comp03.3	XLOC_021953	Phvul.009G054300	11.87	1.98	0
Comp03.3	XLOC_023283	Phvul.009G054400	5.85	3.36	0.0001
Comp03.3	XLOC_021963	Phvul.009G057300	5.06	2.33	0.0006
Comp03.3	XLOC_023321	Phvul.009G061600	-5.68	4.49	0.0001
Comp03.3	XLOC_021994	Phvul.009G062600	4.25	1.06	0.0065
Comp03.3	XLOC_023337	Phvul.009G064900	5.14	5.68	0.0022
Comp03.3	XLOC_023346	Phvul.009G066300	4.65	3.91	0.001
Comp03.3	XLOC_023350	Phvul.009G066900	4.78	4.98	0.0007
Comp03.3	XLOC_022077	Phvul.009G078300	5.45	6.64	0.0002
Comp03.3	XLOC_023458	Phvul.009G087700	-4.97	2.83	0.0009
Comp03.3	XLOC_023493	Phvul.009G094000	7.56	4.39	0
Comp03.3	XLOC_022151	Phvul.009G094600	-5.19	1.39	0.0006
Comp03.3	XLOC_023547	Phvul.009G104200	-4.54	2.16	0.002
Comp03.3	XLOC_022217	Phvul.009G108300	4.11	2.23	0.0075
Comp03.3	XLOC_023568	Phvul.009G108700	6.8	4.37	0
Comp03.3	XLOC_022233	Phvul.009G111500	4.82	7.63	0.0006
Comp03.3	XLOC_023600	Phvul.009G116800	5.67	1.27	0.0012
Comp03.3	XLOC_022274	Phvul.009G118800	5.2	4.56	0.0003
Comp03.3	XLOC_022311	Phvul.009G125900	3.85	7.51	0.0065
Comp03.3	XLOC_023656	Phvul.009G127900	-5.49	7.34	0.0001
Comp03.3	XLOC_023689	Phvul.009G136000	-3.97	5.23	0.0048
Comp03.3	XLOC_022385	Phvul.009G137900	5.08	4.29	0.0004
Comp03.3	XLOC_023712	Phvul.009G140700	4.91	5.47	0.0005
Comp03.3	XLOC_022408	Phvul.009G142500	-5.16	4.5	0.0008
Comp03.3	XLOC_022461	Phvul.009G152500	4.13	4.82	0.0085
Comp03.3	XLOC_023784	Phvul.009G155800	9.1	3.07	0.0007
Comp03.3	XLOC_022481	Phvul.009G156500	10.31	0.19	0
Comp03.3	XLOC_023804	Phvul.009G161000	5.51	0.72	0.0071
Comp03.3	XLOC_023812	Phvul.009G162000	9.08	3.78	0
Comp03.3	XLOC_022546	Phvul.009G168000	4.51	2.88	0.0025

Experimental comparison	Gene ID	Correspondent ID (Phytozome)	$log_2(FC)^£$	$log_2(CPM)^€$	FDR* (all significant at <0.01)
Comp03.3	XLOC_022600	Phvul.009G179600	7.12	1.96	0.0001
Comp03.3	XLOC_023934	Phvul.009G186700	4.42	1.61	0.0054
Comp03.3	XLOC_022682	Phvul.009G197000	-4.08	3.35	0.0055
Comp03.3	XLOC_022726	Phvul.009G207800	8.82	0.71	0.0014
Comp03.3	XLOC_022747	Phvul.009G210900	4.93	2.19	0.0038
Comp03.3	XLOC_022748	Phvul.009G211200	4.16	4.58	0.0034
Comp03.3	XLOC_024213	Phvul.009G240500	9.05	0.36	0.0008
Comp03.3	XLOC_024231	Phvul.009G244000	4.07	4.94	0.0039
Comp03.3	XLOC_023001	Phvul.009G259600	-3.89	5.04	0.0065
Comp03.3	XLOC_025319	Phvul.010G000300	4.56	5.93	0.0012
Comp03.3	XLOC_025333	Phvul.010G003400	4.22	4.84	0.0028
Comp03.3	XLOC_025336	Phvul.010G004000	4.76	2.04	0.0015
Comp03.3	XLOC_025354	Phvul.010G008800	5.22	6.69	0.0002
Comp03.3	XLOC_024471	Phvul.010G009400	3.87	5.17	0.0066
Comp03.3	XLOC_025390	Phvul.010G016300	-3.93	2.24	0.0079
Comp03.3	XLOC_025489	Phvul.010G036100	-5.82	4.23	0.0001
Comp03.3	XLOC_024687	Phvul.010G051100	-6.16	-0.23	0.0015
Comp03.3	XLOC_025594	Phvul.010G057900	4.51	5.42	0.0013
Comp03.3	XLOC_025630	Phvul.010G063800	5.88	6.61	0
Comp03.3	XLOC_024930	Phvul.010G097800	4.53	0.49	0.0051
Comp03.3	XLOC_025822	Phvul.010G101500	-5.43	4.78	0.0001
Comp03.3	XLOC_025831	Phvul.010G103900	6.33	-0.39	0.001
Comp03.3	XLOC_025020	Phvul.010G111000	7.94	1.77	0
Comp03.3	XLOC_025075	Phvul.010G122500	4.21	4.03	0.0028
Comp03.3	XLOC_025084	Phvul.010G124000	4.52	4.63	0.0013
Comp03.3	XLOC_025930	Phvul.010G125300	4.32	5.41	0.0045
Comp03.3	XLOC_025154	Phvul.010G137300	6.36	2.27	0.0002
Comp03.3	XLOC_026006	Phvul.010G139800	-4.18	1.95	0.005
Comp03.3	XLOC_025194	Phvul.010G144200	6.42	6.84	0
Comp03.3	XLOC_025196	Phvul.010G144600	8.58	3.5	0
Comp03.3	XLOC_026054	Phvul.010G152300	-3.71	5.92	0.0092
Comp03.3	XLOC_025244	Phvul.010G152400	-5.01	2.17	0.0007
Comp03.3	XLOC_026058	Phvul.010G152800	4.3	4.44	0.0031
Comp03.3	XLOC_025254	Phvul.010G154300	9.31	-0.87	0.0005
Comp03.3	XLOC_025259	Phvul.010G155300	8.97	0.11	0.001
Comp03.3	XLOC_026071	Phvul.010G156200	4.89	4.61	0.0013
Comp03.3	XLOC_025290	Phvul.010G160700	4.49	3.39	0.0018
Comp03.3	XLOC_025292	Phvul.010G161300	5.08	6.53	0.0004
Comp03.3	XLOC_026105	Phvul.010G162900	4.63	0.27	0.0079
Comp03.3	XLOC_027424	Phvul.011G012600	11.15	1.33	0
Comp03.3	XLOC_026272	Phvul.011G015000	8.31	-0.78	0.0045
Comp03.3	XLOC_027444	Phvul.011G015400	5.49	0.31	0.001
Comp03.3	XLOC_027474	Phvul.011G022300	-4.37	1.07	0.0052
Comp03.3	XLOC_027493	Phvul.011G025800	6.21	0.05	0.0012
Comp03.3	XLOC_026329	Phvul.011G026200, Phvul.011G026300	-4.26	3.33	0.0068
Comp03.3	XLOC_026343	Phvul.011G029200	-5.77	-0.29	0.0038
Comp03.3	XLOC_026446	Phvul.011G047800	4.53	5.56	0.0014

Experimental comparison	Gene ID	Correspondent ID (Phytozome)	$log_2(FC)$[£]	$log_2(CPM)$[€]	FDR* (all significant at <0.01)
Comp03.3	XLOC_026518	Phvul.011G060400	-5.02	4	0.0005
Comp03.3	XLOC_027687	Phvul.011G066800	9.62	-0.29	0.0002
Comp03.3	XLOC_027689	Phvul.011G067200	4.59	2.45	0.0059
Comp03.3	XLOC_027705	Phvul.011G070400	6.42	0.88	0.0002
Comp03.3	XLOC_026570	Phvul.011G072200	4.94	2.72	0.0024
Comp03.3	XLOC_026597	Phvul.011G077600	-4.04	4.05	0.0043
Comp03.3	XLOC_026618	Phvul.011G081800	-6.04	1.97	0.0001
Comp03.3	XLOC_027818	Phvul.011G089600	4.69	0.79	0.0034
Comp03.3	XLOC_027819	Phvul.011G089800	4.03	4.91	0.0047
Comp03.3	XLOC_026695	Phvul.011G099300	11.74	2.1	0
Comp03.3	XLOC_026810	Phvul.011G119500	5.86	2.6	0.0008
Comp03.3	XLOC_027963	Phvul.011G120100	5.56	-0.95	0.0062
Comp03.3	XLOC_027984	Phvul.011G125300	10.45	0.92	0
Comp03.3	XLOC_026860	Phvul.011G127100	7.41	3.69	0
Comp03.3	XLOC_027997	Phvul.011G128200	3.97	4.58	0.0058
Comp03.3	XLOC_026949	Phvul.011G142300	5.74	5.86	0.0001
Comp03.3	XLOC_026983	Phvul.011G147800	5.24	7.56	0.0002
Comp03.3	XLOC_026986	Phvul.011G148500	4.82	4.45	0.0007
Comp03.3	XLOC_026997	Phvul.011G150400	4.98	3.52	0.0006
Comp03.3	XLOC_027011	Phvul.011G152700	4.56	0.32	0.0064
Comp03.3	XLOC_027057	Phvul.011G160600	-6.45	0.56	0.0002
Comp03.3	XLOC_027058	Phvul.011G160700	-3.84	4.17	0.0072
Comp03.3	XLOC_027079	Phvul.011G163800	4.73	1.46	0.0021
Comp03.3	XLOC_028170	Phvul.011G169500	5.37	1.85	0.0005
Comp03.3	XLOC_027206	Phvul.011G189200	10.49	0.52	0
Comp03.3	XLOC_027207	Phvul.011G189300	9.71	4.01	0
Comp03.3	XLOC_027211	Phvul.011G189900	6.07	4.9	0
Comp03.3	XLOC_028284	Phvul.011G191900	8.77	-0.88	0.0016
Comp03.3	XLOC_028291	Phvul.011G194400	4.4	4.19	0.0018
Comp03.3	XLOC_027249	Phvul.011G197000	12.09	3.7	0
Comp03.3	XLOC_028384	Phvul.011G215300	4.23	0.63	0.0088
Comp03.3	XLOC_028567	Phvul.L002700	-4.05	3.23	0.0045
Comp03.3	XLOC_028570	Phvul.L003000	5.46	1.26	0.0007
Comp03.3	XLOC_028622	Phvul.L005900	8.8	-0.27	0.0015
Comp03.3	XLOC_028531	Phvul.L008100	5.35	4.05	0.0003
Comp03.3	XLOC_028634	Phvul.L011500	4.89	-0.27	0.0083
Comp04.1	XLOC_002736	new loci	9.18	1.01	0.0033
Comp04.1	XLOC_009372	new loci	9.2	1.06	0.0033
Comp04.1	XLOC_013810	new loci	5.25	1.25	0.0049
Comp04.1	XLOC_025825	new loci	10.64	2.18	0.0002
Comp04.1	XLOC_027985	new loci	7.43	3.78	0.0007
Comp04.1	XLOC_001456	Phvul.001G020300	6.03	5.7	0.0003
Comp04.1	XLOC_000186	Phvul.001G039700	6.43	6.48	0.0002
Comp04.1	XLOC_001674	Phvul.001G059600	4.68	4.61	0.0066
Comp04.1	XLOC_001721	Phvul.001G071200	5.21	3.61	0.0025
Comp04.1	XLOC_000374	Phvul.001G076000	4.97	3.44	0.0061
Comp04.1	XLOC_002095	Phvul.001G145600	4.57	4.88	0.0055
Comp04.1	XLOC_002168	Phvul.001G158100	5.95	1.94	0.0047

Experimental comparison	Gene ID	Correspondent ID (Phytozome)	$log_2(FC)^£$	$log_2(CPM)^€$	FDR* (all significant at <0.01)
Comp04.1	XLOC_000815	Phvul.001G160200	6.89	3.13	0.0002
Comp04.1	XLOC_000821	Phvul.001G161000	4.6	3.81	0.0061
Comp04.1	XLOC_002190	Phvul.001G163400	-8.62	-0.74	0.0087
Comp04.1	XLOC_002351	Phvul.001G194800	4.87	4.46	0.0034
Comp04.1	XLOC_001124	Phvul.001G220800	-8.79	-0.84	0.0058
Comp04.1	XLOC_001179	Phvul.001G231900	5.59	3.81	0.0093
Comp04.1	XLOC_002650	Phvul.001G255200	5.81	6	0.0058
Comp04.1	XLOC_004544	Phvul.002G009700	5.43	5.93	0.001
Comp04.1	XLOC_002924	Phvul.002G014700	5.14	3.65	0.0042
Comp04.1	XLOC_004586	Phvul.002G018100	5.95	2	0.0016
Comp04.1	XLOC_003024	Phvul.002G033100	5.98	1.98	0.0046
Comp04.1	XLOC_003080	Phvul.002G046800	4.67	7.83	0.0042
Comp04.1	XLOC_003088	Phvul.002G049000	7.48	4.39	0.0007
Comp04.1	XLOC_004803	Phvul.002G061200	-4.84	4.92	0.0033
Comp04.1	XLOC_003349	Phvul.002G096600	4.41	5.01	0.0069
Comp04.1	XLOC_005051	Phvul.002G112600	4.4	7.89	0.0065
Comp04.1	XLOC_005316	Phvul.002G159800	4.98	3.88	0.0039
Comp04.1	XLOC_003644	Phvul.002G160100	7.15	1.03	0.0013
Comp04.1	XLOC_003768	Phvul.002G185800	5.29	6.64	0.0013
Comp04.1	XLOC_003780	Phvul.002G187800	-8.69	-0.62	0.0075
Comp04.1	XLOC_005677	Phvul.002G228400	6.04	4.79	0.0003
Comp04.1	XLOC_003991	Phvul.002G231500	5.17	6.44	0.0042
Comp04.1	XLOC_004194	Phvul.002G271900	5.25	5.16	0.0033
Comp04.1	XLOC_004254	Phvul.002G284700	5.61	4.07	0.0009
Comp04.1	XLOC_004259	Phvul.002G285800	5.99	3.25	0.0015
Comp04.1	XLOC_006194	Phvul.002G331800	6.72	1.94	0.0003
Comp04.1	XLOC_006360	Phvul.003G012600	7.16	4.06	0.0002
Comp04.1	XLOC_006536	Phvul.003G051800	6.04	4.94	0.0003
Comp04.1	XLOC_008262	Phvul.003G074000	5.59	7.82	0.0007
Comp04.1	XLOC_008430	Phvul.003G109000	4.48	6.17	0.0058
Comp04.1	XLOC_008432	Phvul.003G109200	5.68	4.43	0.001
Comp04.1	XLOC_008435	Phvul.003G109600	8.78	0.63	0.0061
Comp04.1	XLOC_008502	Phvul.003G124100	4.67	5.34	0.0049
Comp04.1	XLOC_006942	Phvul.003G128400	-9.25	0.45	0.0028
Comp04.1	XLOC_008534	Phvul.003G131500	5.32	6.57	0.0013
Comp04.1	XLOC_008941	Phvul.003G212600	5.26	3.01	0.0039
Comp04.1	XLOC_007492	Phvul.003G233400	5.53	5.35	0.001
Comp04.1	XLOC_009058	Phvul.003G238700	5.24	3.02	0.0058
Comp04.1	XLOC_009180	Phvul.003G268500	5.89	4.26	0.0053
Comp04.1	XLOC_010789	Phvul.004G092100	5.5	6.35	0.001
Comp04.1	XLOC_011021	Phvul.004G134800	6.84	3.78	0.0003
Comp04.1	XLOC_011055	Phvul.004G142000	-6.12	3.44	0.0003
Comp04.1	XLOC_011570	Phvul.005G046200	5.38	3.01	0.0016
Comp04.1	XLOC_011600	Phvul.005G051600	4.5	8.2	0.0056
Comp04.1	XLOC_011616	Phvul.005G054000	9.31	2.97	0.0025
Comp04.1	XLOC_011828	Phvul.005G099500	-8.87	4.44	0.0054
Comp04.1	XLOC_012872	Phvul.005G111700	5.63	3.57	0.002
Comp04.1	XLOC_013102	Phvul.005G155800	4.63	7.16	0.0047

Experimental comparison	Gene ID	Correspondent ID (Phytozome)	$log_2(FC)$[£]	$log_2(CPM)$[€]	FDR* (all significant at <0.01)
Comp04.1	XLOC_012185	Phvul.005G173000	5.15	7.34	0.0017
Comp04.1	XLOC_013191	Phvul.005G174800	5.39	4.03	0.0039
Comp04.1	XLOC_014603	Phvul.006G028700	6.65	3.79	0.0003
Comp04.1	XLOC_013583	Phvul.006G043100	4.99	6.33	0.0023
Comp04.1	XLOC_013600	Phvul.006G045800	5.35	6.39	0.0049
Comp04.1	XLOC_013746	Phvul.006G075600	5.34	7.18	0.0012
Comp04.1	XLOC_013758	Phvul.006G078800	6.11	5.86	0.0004
Comp04.1	XLOC_014876	Phvul.006G083800	5.17	3.25	0.0047
Comp04.1	XLOC_015019	Phvul.006G112300	5.8	2.53	0.0016
Comp04.1	XLOC_014109	Phvul.006G148400	5.29	1.55	0.0032
Comp04.1	XLOC_015337	Phvul.006G176500	8.81	0.5	0.0058
Comp04.1	XLOC_014444	Phvul.006G216700	5.21	6.74	0.0062
Comp04.1	XLOC_017232	Phvul.007G031000	6.46	2.29	0.0049
Comp04.1	XLOC_017251	Phvul.007G034500	8.87	3.54	0.0055
Comp04.1	XLOC_017633	Phvul.007G109000	4.34	4.57	0.0093
Comp04.1	XLOC_016318	Phvul.007G135600	4.98	5.31	0.0033
Comp04.1	XLOC_016446	Phvul.007G160000	4.58	4	0.0058
Comp04.1	XLOC_016486	Phvul.007G167000	4.79	3.89	0.0053
Comp04.1	XLOC_016657	Phvul.007G201100	8.12	2.68	0.0002
Comp04.1	XLOC_018152	Phvul.007G210400	9.49	2	0.0016
Comp04.1	XLOC_016702	Phvul.007G211400	-4.42	4.38	0.0069
Comp04.1	XLOC_016775	Phvul.007G222500	4.9	3.98	0.0073
Comp04.1	XLOC_016946	Phvul.007G257300	-5.06	2.7	0.003
Comp04.1	XLOC_016955	Phvul.007G259500	5.94	3.23	0.0048
Comp04.1	XLOC_017043	Phvul.007G275700	8.62	2.24	0.0093
Comp04.1	XLOC_017050	Phvul.007G277000	8.81	1.82	0.0058
Comp04.1	XLOC_017069	Phvul.007G280700	4.73	6.64	0.0039
Comp04.1	XLOC_020092	Phvul.008G005200	6.51	3.12	0.0046
Comp04.1	XLOC_020111	Phvul.008G008100	4.88	7.83	0.0028
Comp04.1	XLOC_020340	Phvul.008G055200	-8.64	0.5	0.0081
Comp04.1	XLOC_019087	Phvul.008G098200	7.21	4.88	0.0002
Comp04.1	XLOC_020537	Phvul.008G098500	4.81	6.31	0.0046
Comp04.1	XLOC_020575	Phvul.008G105800	6.57	0.78	0.0042
Comp04.1	XLOC_019281	Phvul.008G134800	4.28	4.93	0.0098
Comp04.1	XLOC_020939	Phvul.008G175500	7.11	4.15	0.0004
Comp04.1	XLOC_021042	Phvul.008G194600	5.19	2.26	0.0055
Comp04.1	XLOC_019720	Phvul.008G223500	5.37	6.42	0.0013
Comp04.1	XLOC_021212	Phvul.008G228100	5.02	5.94	0.0097
Comp04.1	XLOC_021953	Phvul.009G054300	9.77	1.98	0.001
Comp04.1	XLOC_022077	Phvul.009G078300	5.88	6.64	0.0006
Comp04.1	XLOC_023493	Phvul.009G094000	4.77	4.39	0.0047
Comp04.1	XLOC_022274	Phvul.009G118800	4.66	4.56	0.0047
Comp04.1	XLOC_022461	Phvul.009G152500	4.75	4.82	0.0058
Comp04.1	XLOC_022546	Phvul.009G168000	4.85	2.88	0.0093
Comp04.1	XLOC_024213	Phvul.009G240500	6.42	0.36	0.0053
Comp04.1	XLOC_023011	Phvul.009G261400	4.64	4.38	0.0049
Comp04.1	XLOC_025354	Phvul.010G008800	4.34	6.69	0.0081
Comp04.1	XLOC_025489	Phvul.010G036100	-4.76	4.23	0.0085

Experimental comparison	Gene ID	Correspondent ID (Phytozome)	log_2(FC)[£]	log_2(CPM)[€]	FDR* (all significant at <0.01)
Comp04.1	XLOC_024709	Phvul.010G054400	9.09	0.1	0.0039
Comp04.1	XLOC_025630	Phvul.010G063800	4.68	6.61	0.0042
Comp04.1	XLOC_025084	Phvul.010G124000	5.46	4.63	0.0015
Comp04.1	XLOC_025194	Phvul.010G144200	4.84	6.84	0.0034
Comp04.1	XLOC_025196	Phvul.010G144600	8.84	3.5	0.0058
Comp04.1	XLOC_027424	Phvul.011G012600	9.57	1.33	0.0015
Comp04.1	XLOC_026618	Phvul.011G081800	-5.51	1.97	0.0019
Comp04.1	XLOC_026624	Phvul.011G083500	-4.53	1.77	0.0094
Comp04.1	XLOC_026695	Phvul.011G099300	8.62	2.1	0.0093
Comp04.1	XLOC_026810	Phvul.011G119500	9.47	2.6	0.0017
Comp04.1	XLOC_026860	Phvul.011G127100	7.04	3.69	0.0005
Comp04.1	XLOC_026949	Phvul.011G142300	4.61	5.86	0.0056
Comp04.1	XLOC_026986	Phvul.011G148500	4.46	4.45	0.0087
Comp04.1	XLOC_027207	Phvul.011G189300	5.19	4.01	0.0055
Comp04.1	XLOC_028531	Phvul.L008100	5.02	4.05	0.0037
Comp04.2	XLOC_002827	new loci	9.32	1.61	0.0055
Comp04.2	XLOC_006966	new loci	-6.22	1.15	0.0083
Comp04.2	XLOC_007865	new loci	9.45	1.62	0.0038
Comp04.2	XLOC_011757	new loci	-9.56	2.12	0.0034
Comp04.2	XLOC_017321	new loci	-9.95	0.11	0.0016
Comp04.2	XLOC_018539	new loci	9.13	1.34	0.0075
Comp04.2	XLOC_018564	new loci	-9.21	0.44	0.0065
Comp04.2	XLOC_020681	new loci	-9.26	0.52	0.006
Comp04.2	XLOC_026888	new loci	-9.84	1.34	0.0018
Comp04.2	XLOC_027632	new loci	-11.2	0.91	0
Comp04.2	XLOC_008018	Phvul.003G030500	-10.62	0.21	0.0002
Comp04.2	XLOC_009477	Phvul.004G012800	-5.93	3.36	0.0018
Comp04.2	XLOC_009518	Phvul.004G021200	-12.58	2.99	0
Comp04.2	XLOC_010701	Phvul.004G076400	-10.94	7.42	0
Comp04.2	XLOC_011726	Phvul.005G077000	-6.88	3.98	0.0001
Comp04.2	XLOC_011828	Phvul.005G099500	11.44	4.44	0
Comp04.2	XLOC_014622	Phvul.006G033000	-9.62	1.19	0.0033
Comp04.2	XLOC_017318	Phvul.007G048800	5.57	2.57	0.006
Comp04.2	XLOC_019142	Phvul.008G109000	13.29	4	0
Comp04.2	XLOC_019143	Phvul.008G109100	16.08	7.04	0
Comp04.2	XLOC_019871	Phvul.008G252000	5.49	4.18	0.0038
Comp04.2	XLOC_022283	Phvul.009G120500	5.94	4.41	0.0016
Comp04.2	XLOC_022491	Phvul.009G158100	-6.62	0.11	0.0038
Comp04.2	XLOC_025489	Phvul.010G036100	5.21	4.23	0.0069
Comp04.2	XLOC_024709	Phvul.010G054400	-6.41	0.1	0.0059
Comp04.2	XLOC_025091	Phvul.010G124800	-4.78	3.12	0.0099
Comp04.2	XLOC_026331	Phvul.011G026700	-5.99	8.95	0.006
Comp04.2	XLOC_027739	Phvul.011G076400	-5.18	5.03	0.0034
Comp04.2	XLOC_027168	Phvul.011G183600	9.57	1.51	0.0034
Comp04.2	XLOC_027170	Phvul.011G183800	9.36	4.42	0.0048
Comp04.2	XLOC_027232	Phvul.011G193600	6.07	2.65	0.0023
Comp04.3	XLOC_000689	new loci	4.93	1.47	0.0073
Comp04.3	XLOC_002282	new loci	5.56	2.94	0.0017

Experimental comparison	Gene ID	Correspondent ID (Phytozome)	$log_2(FC)^£$	$log_2(CPM)^€$	FDR* (all significant at <0.01)
Comp04.3	XLOC_002736	new loci	9.78	1.01	0.0003
Comp04.3	XLOC_002758	new loci	8.27	-0.24	0.0082
Comp04.3	XLOC_002785	new loci	8.17	0.92	0.0097
Comp04.3	XLOC_002805	new loci	8.46	0.5	0.0054
Comp04.3	XLOC_002827	new loci	9.49	1.61	0.0006
Comp04.3	XLOC_003885	new loci	8.2	-0.22	0.0089
Comp04.3	XLOC_006277	new loci	8.24	-0.14	0.0082
Comp04.3	XLOC_006966	new loci	-6.85	1.15	0.0007
Comp04.3	XLOC_007575	new loci	5.83	0.82	0.0039
Comp04.3	XLOC_007780	new loci	6.89	1.49	0.0003
Comp04.3	XLOC_007865	new loci	9.62	1.62	0.0004
Comp04.3	XLOC_008029	new loci	8.43	0.67	0.0058
Comp04.3	XLOC_008379	new loci	4.93	0.77	0.0081
Comp04.3	XLOC_009355	new loci	3.94	4.47	0.0093
Comp04.3	XLOC_009372	new loci	9.09	1.06	0.0012
Comp04.3	XLOC_009410	new loci	8.87	0.62	0.0022
Comp04.3	XLOC_009875	new loci	-6.28	-0.09	0.0028
Comp04.3	XLOC_010325	new loci	8.27	0.44	0.0082
Comp04.3	XLOC_010340	new loci	-6.41	1.59	0.0006
Comp04.3	XLOC_011258	new loci	8.91	0.91	0.0019
Comp04.3	XLOC_011298	new loci	9.41	0.95	0.0006
Comp04.3	XLOC_011313	new loci	5.6	1.09	0.0062
Comp04.3	XLOC_011342	new loci	8.52	0.34	0.0046
Comp04.3	XLOC_011343	new loci	9.15	0.99	0.0011
Comp04.3	XLOC_011757	new loci	-9.77	2.12	0.0003
Comp04.3	XLOC_011869	new loci	8.6	1.3	0.0041
Comp04.3	XLOC_013316	new loci	8.7	-0.28	0.0031
Comp04.3	XLOC_015577	new loci	-8.27	-0.05	0.0082
Comp04.3	XLOC_015620	new loci	9.43	0.46	0.0006
Comp04.3	XLOC_017343	new loci	5.63	3.71	0.0003
Comp04.3	XLOC_018539	new loci	9.3	1.34	0.0008
Comp04.3	XLOC_018563	new loci	-5.84	1.18	0.0021
Comp04.3	XLOC_018564	new loci	-9.29	0.44	0.0008
Comp04.3	XLOC_020681	new loci	-9.2	0.52	0.001
Comp04.3	XLOC_020682	new loci	-9.39	0.64	0.0007
Comp04.3	XLOC_020771	new loci	8.55	0.2	0.0046
Comp04.3	XLOC_021560	new loci	5.89	0.83	0.0033
Comp04.3	XLOC_021563	new loci	-8.58	-1.09	0.0043
Comp04.3	XLOC_021642	new loci	8.75	0.72	0.0028
Comp04.3	XLOC_021652	new loci	9.03	0.69	0.0014
Comp04.3	XLOC_021905	new loci	5.91	0.56	0.0032
Comp04.3	XLOC_021926	new loci	4.26	3.54	0.0063
Comp04.3	XLOC_024008	new loci	-5.19	1.81	0.0007
Comp04.3	XLOC_024342	new loci	8.27	-0.96	0.0082
Comp04.3	XLOC_024387	new loci	5.37	1.31	0.0099
Comp04.3	XLOC_024405	new loci	8.46	0.23	0.0054
Comp04.3	XLOC_024557	new loci	4.02	3.05	0.0082
Comp04.3	XLOC_025825	new loci	10.76	2.18	0

Experimental comparison	Gene ID	Correspondent ID (Phytozome)	$log_2(FC)^£$	$log_2(CPM)^€$	FDR* (all significant at <0.01)
Comp04.3	XLOC_026139	new loci	4.57	3.15	0.0041
Comp04.3	XLOC_026160	new loci	4.3	2.45	0.0063
Comp04.3	XLOC_026888	new loci	-10.03	1.34	0.0002
Comp04.3	XLOC_027632	new loci	-9.37	0.91	0.0007
Comp04.3	XLOC_027985	new loci	9.11	3.78	0
Comp04.3	XLOC_028038	new loci	-9.14	0.19	0.0012
Comp04.3	XLOC_028436	new loci	4.62	1.61	0.0056
Comp04.3	XLOC_001456	Phvul.001G020300	6.66	5.7	0
Comp04.3	XLOC_000175	Phvul.001G037100	-4.38	0.46	0.0077
Comp04.3	XLOC_001567	Phvul.001G039600	-4.46	0.6	0.0073
Comp04.3	XLOC_000186	Phvul.001G039700	5.58	6.48	0.0003
Comp04.3	XLOC_001636	Phvul.001G051000	6.48	4.55	0
Comp04.3	XLOC_001640	Phvul.001G052100	8.17	-0.96	0.0097
Comp04.3	XLOC_001721	Phvul.001G071200	5.58	3.61	0.0003
Comp04.3	XLOC_000374	Phvul.001G076000	6.59	3.44	0
Comp04.3	XLOC_000427	Phvul.001G085900	-4.21	4.24	0.0049
Comp04.3	XLOC_000668	Phvul.001G131000	4.61	4.57	0.0021
Comp04.3	XLOC_002095	Phvul.001G145600	4.35	4.88	0.0039
Comp04.3	XLOC_002157	Phvul.001G156500	4.15	5.93	0.0055
Comp04.3	XLOC_002168	Phvul.001G158100	6.04	1.94	0.0006
Comp04.3	XLOC_000815	Phvul.001G160200	6.18	3.13	0.0002
Comp04.3	XLOC_000821	Phvul.001G161000	5.05	3.81	0.0009
Comp04.3	XLOC_002203	Phvul.001G165700	5.5	2.89	0.0007
Comp04.3	XLOC_000920	Phvul.001G180500	4.43	5.79	0.0034
Comp04.3	XLOC_002292	Phvul.001G183300	4.21	4.19	0.0053
Comp04.3	XLOC_002314	Phvul.001G187800	10.47	1.33	0
Comp04.3	XLOC_002351	Phvul.001G194800	5	4.46	0.0008
Comp04.3	XLOC_002414	Phvul.001G206000	4.85	4.18	0.0013
Comp04.3	XLOC_002429	Phvul.001G209200	5.74	0.12	0.0046
Comp04.3	XLOC_001179	Phvul.001G231900	5.73	3.81	0.0012
Comp04.3	XLOC_002650	Phvul.001G255200	5.66	6	0.0015
Comp04.3	XLOC_001332	Phvul.001G263900	4.34	3.29	0.0069
Comp04.3	XLOC_002712	Phvul.001G265800	4.57	3.67	0.0027
Comp04.3	XLOC_002854	Phvul.002G002000	4.24	8.69	0.0041
Comp04.3	XLOC_002885	Phvul.002G007500	4.36	5.42	0.0033
Comp04.3	XLOC_004544	Phvul.002G009700	6.13	5.93	0
Comp04.3	XLOC_002924	Phvul.002G014700	5.54	3.65	0.0005
Comp04.3	XLOC_004586	Phvul.002G018100	5.7	2	0.0012
Comp04.3	XLOC_003024	Phvul.002G033100	5.87	1.98	0.0009
Comp04.3	XLOC_004672	Phvul.002G035800	4.65	3.03	0.0088
Comp04.3	XLOC_003080	Phvul.002G046800	5.52	7.83	0.0002
Comp04.3	XLOC_004743	Phvul.002G048100	3.96	6.83	0.008
Comp04.3	XLOC_003088	Phvul.002G049000	7.22	4.39	0.0001
Comp04.3	XLOC_003121	Phvul.002G055600	5.04	2.76	0.0013
Comp04.3	XLOC_004803	Phvul.002G061200	-4.77	4.92	0.0012
Comp04.3	XLOC_004851	Phvul.002G072000	5.61	6.14	0.0002
Comp04.3	XLOC_003349	Phvul.002G096600	4.83	5.01	0.0011
Comp04.3	XLOC_003411	Phvul.002G108200	8.46	0.93	0.0054

Experimental comparison	Gene ID	Correspondent ID (Phytozome)	log_2(FC)[£]	log_2(CPM)[€]	FDR* (all significant at <0.01)
Comp04.3	XLOC_005113	Phvul.002G123500	4.45	3.96	0.0029
Comp04.3	XLOC_005185	Phvul.002G134700	-5.68	0.66	0.0006
Comp04.3	XLOC_003553	Phvul.002G141600	7.77	2.3	0
Comp04.3	XLOC_005297	Phvul.002G156100	9.2	1.18	0.001
Comp04.3	XLOC_005316	Phvul.002G159800	5.77	3.88	0.0002
Comp04.3	XLOC_003644	Phvul.002G160100	6.23	1.03	0.0014
Comp04.3	XLOC_003702	Phvul.002G170800	4.75	5.78	0.0022
Comp04.3	XLOC_003765	Phvul.002G184600	4.33	4.24	0.0042
Comp04.3	XLOC_003768	Phvul.002G185800	6.21	6.64	0
Comp04.3	XLOC_003770	Phvul.002G186000	4.28	4.91	0.0042
Comp04.3	XLOC_005461	Phvul.002G186600	5.92	0.29	0.003
Comp04.3	XLOC_005521	Phvul.002G199000	-4.1	2.57	0.0092
Comp04.3	XLOC_005527	Phvul.002G199700	4.69	2.25	0.0029
Comp04.3	XLOC_005555	Phvul.002G204900	4.61	3.68	0.0028
Comp04.3	XLOC_003881	Phvul.002G208900, Phvul.002G209000	4.7	1.89	0.0079
Comp04.3	XLOC_003915	Phvul.002G215100	8.27	0.82	0.0082
Comp04.3	XLOC_003955	Phvul.002G223400	4.31	4.09	0.0044
Comp04.3	XLOC_005677	Phvul.002G228400	5.43	4.79	0.0003
Comp04.3	XLOC_003974	Phvul.002G228700	5.01	5.78	0.0007
Comp04.3	XLOC_003998	Phvul.002G232800	8.57	1.47	0.0043
Comp04.3	XLOC_005708	Phvul.002G235100	5.06	4.76	0.0007
Comp04.3	XLOC_004088	Phvul.002G249300	8.57	0	0.0043
Comp04.3	XLOC_004194	Phvul.002G271900	5.43	5.16	0.0006
Comp04.3	XLOC_004254	Phvul.002G284700	5.25	4.07	0.0006
Comp04.3	XLOC_004259	Phvul.002G285800	6.36	3.25	0.0003
Comp04.3	XLOC_004361	Phvul.002G306400	4.61	2.26	0.0033
Comp04.3	XLOC_004459	Phvul.002G324400	8.97	0.5	0.0016
Comp04.3	XLOC_006194	Phvul.002G331800	5.5	1.94	0.002
Comp04.3	XLOC_007882	Phvul.003G002500	-6.03	4.23	0.0001
Comp04.3	XLOC_006360	Phvul.003G012600	7.42	4.06	0
Comp04.3	XLOC_006376	Phvul.003G016400	5.1	1.13	0.0052
Comp04.3	XLOC_008018	Phvul.003G030500	-8.39	0.21	0.0067
Comp04.3	XLOC_006522	Phvul.003G048700	4.03	3.94	0.0081
Comp04.3	XLOC_006536	Phvul.003G051800	5.65	4.94	0.0002
Comp04.3	XLOC_006537	Phvul.003G051900	5.47	1.35	0.0083
Comp04.3	XLOC_008161	Phvul.003G055500	4.92	1.56	0.0075
Comp04.3	XLOC_008176	Phvul.003G058700	8.27	0.5	0.0082
Comp04.3	XLOC_008262	Phvul.003G074000	6.03	7.82	0.0001
Comp04.3	XLOC_008281	Phvul.003G078100	4.46	2.08	0.0059
Comp04.3	XLOC_006675	Phvul.003G079800	4.53	3.94	0.0043
Comp04.3	XLOC_006721	Phvul.003G087700	5.56	1.8	0.0017
Comp04.3	XLOC_008430	Phvul.003G109000	5.05	6.17	0.0006
Comp04.3	XLOC_008432	Phvul.003G109200	5.86	4.43	0.0002
Comp04.3	XLOC_006889	Phvul.003G120100	5.63	2.62	0.0005
Comp04.3	XLOC_008502	Phvul.003G124100	5.8	5.34	0.0001
Comp04.3	XLOC_006942	Phvul.003G128400	-6.59	0.45	0.0012
Comp04.3	XLOC_008523	Phvul.003G129200	3.91	6.93	0.0087

Experimental comparison	Gene ID	Correspondent ID (Phytozome)	$log_2(FC)^£$	$log_2(CPM)^€$	FDR* (all significant at <0.01)
Comp04.3	XLOC_008534	Phvul.003G131500	6.2	6.57	0
Comp04.3	XLOC_008555	Phvul.003G135700	11.13	2.4	0
Comp04.3	XLOC_007001	Phvul.003G138800	8.65	0.54	0.0036
Comp04.3	XLOC_007002	Phvul.003G138900	10.93	2.83	0
Comp04.3	XLOC_007055	Phvul.003G148900	5.24	3.89	0.0013
Comp04.3	XLOC_007133	Phvul.003G164000	5.26	5.66	0.0015
Comp04.3	XLOC_008713	Phvul.003G167700	4.56	2.88	0.0038
Comp04.3	XLOC_008772	Phvul.003G176800	-4.37	3.71	0.0074
Comp04.3	XLOC_007212	Phvul.003G181300	5.62	0.78	0.0059
Comp04.3	XLOC_008801	Phvul.003G182600	5.18	2.6	0.001
Comp04.3	XLOC_008941	Phvul.003G212600	5.4	3.01	0.0008
Comp04.3	XLOC_007402	Phvul.003G217200	4.54	3	0.0068
Comp04.3	XLOC_009014	Phvul.003G228000	3.94	6.03	0.0087
Comp04.3	XLOC_007492	Phvul.003G233400	6.32	5.35	0
Comp04.3	XLOC_009058	Phvul.003G238700	5.87	3.02	0.0008
Comp04.3	XLOC_007554	Phvul.003G243400	4.19	3.32	0.0066
Comp04.3	XLOC_007606	Phvul.003G252500	5.91	3.57	0.0007
Comp04.3	XLOC_007655	Phvul.003G259200	4.05	2.76	0.0092
Comp04.3	XLOC_009180	Phvul.003G268500	5.83	4.26	0.001
Comp04.3	XLOC_010334	Phvul.004G009000, Phvul.004G009100	-5.05	3.42	0.0008
Comp04.3	XLOC_009477	Phvul.004G012800	-6.17	3.36	0.0001
Comp04.3	XLOC_009483	Phvul.004G013400	4.14	3.05	0.0079
Comp04.3	XLOC_010374	Phvul.004G015500	5.51	-0.18	0.0075
Comp04.3	XLOC_009518	Phvul.004G021200	-9.59	2.99	0.0004
Comp04.3	XLOC_009520	Phvul.004G021400	6.04	6.43	0.0001
Comp04.3	XLOC_009642	Phvul.004G045800	-5.47	3	0.0004
Comp04.3	XLOC_009680	Phvul.004G055200	5.38	5.13	0.0003
Comp04.3	XLOC_010597	Phvul.004G058400	-6.37	1.69	0.0001
Comp04.3	XLOC_009701	Phvul.004G058500	-9.47	-0.54	0.0006
Comp04.3	XLOC_010656	Phvul.004G068400	5.4	4.41	0.0008
Comp04.3	XLOC_010676	Phvul.004G071700	4.52	5.98	0.0022
Comp04.3	XLOC_010701	Phvul.004G076400	-10.73	7.42	0
Comp04.3	XLOC_010724	Phvul.004G080200	4.11	8.47	0.0055
Comp04.3	XLOC_009819	Phvul.004G085100	4.54	2.84	0.0068
Comp04.3	XLOC_010783	Phvul.004G090300	9.09	1.2	0.0012
Comp04.3	XLOC_010789	Phvul.004G092100	6.32	6.35	0
Comp04.3	XLOC_009962	Phvul.004G111900	5.37	4.27	0.0099
Comp04.3	XLOC_010063	Phvul.004G134400	4.86	3.41	0.0013
Comp04.3	XLOC_011021	Phvul.004G134800	8.02	3.78	0
Comp04.3	XLOC_010087	Phvul.004G138500	3.92	8.13	0.0083
Comp04.3	XLOC_011037	Phvul.004G138600	4.25	4.82	0.0046
Comp04.3	XLOC_011055	Phvul.004G142000	-5.61	3.44	0.0002
Comp04.3	XLOC_011088	Phvul.004G148400	4.14	6.34	0.0056
Comp04.3	XLOC_010164	Phvul.004G152900	8.4	0.38	0.0062
Comp04.3	XLOC_011124	Phvul.004G155000	8.37	4.33	0.0067
Comp04.3	XLOC_011157	Phvul.004G160300	-4.4	7.01	0.0049
Comp04.3	XLOC_010254	Phvul.004G173400	8.73	0.56	0.0029

Experimental comparison	Gene ID	Correspondent ID (Phytozome)	$log_2(FC)^£$	$log_2(CPM)^€$	FDR* (all significant at <0.01)
Comp04.3	XLOC_012283	Phvul.005G004400	3.88	5.62	0.0094
Comp04.3	XLOC_012323	Phvul.005G012000	5.74	0.52	0.0046
Comp04.3	XLOC_012340	Phvul.005G014900	3.98	4.98	0.0079
Comp04.3	XLOC_011486	Phvul.005G029700	8.27	0.02	0.0082
Comp04.3	XLOC_011570	Phvul.005G046200	4.69	3.01	0.0028
Comp04.3	XLOC_011600	Phvul.005G051600	4.48	8.2	0.0024
Comp04.3	XLOC_011616	Phvul.005G054000	10.22	2.97	0.0001
Comp04.3	XLOC_012545	Phvul.005G055000	4.09	5.42	0.0065
Comp04.3	XLOC_012655	Phvul.005G075500	3.9	5.51	0.0093
Comp04.3	XLOC_011726	Phvul.005G077000	-7.04	3.98	0
Comp04.3	XLOC_011754	Phvul.005G084500	4.18	8.3	0.0048
Comp04.3	XLOC_012779	Phvul.005G097000	-4.31	1.01	0.0062
Comp04.3	XLOC_012869	Phvul.005G111400	6.06	3.36	0.0003
Comp04.3	XLOC_012870	Phvul.005G111500	4.62	6.38	0.0017
Comp04.3	XLOC_012872	Phvul.005G111700	6.66	3.57	0
Comp04.3	XLOC_013102	Phvul.005G155800	5.02	7.16	0.0007
Comp04.3	XLOC_012103	Phvul.005G156700	9.82	1.4	0.0003
Comp04.3	XLOC_013113	Phvul.005G158500	5.02	5.94	0.0007
Comp04.3	XLOC_012185	Phvul.005G173000	5.71	7.34	0.0001
Comp04.3	XLOC_013188	Phvul.005G173600	5.34	7.75	0.0003
Comp04.3	XLOC_014485	Phvul.006G001900	5.74	1.79	0.0012
Comp04.3	XLOC_013350	Phvul.006G004800	5.45	3.85	0.0004
Comp04.3	XLOC_014518	Phvul.006G011600	4.02	7.66	0.0068
Comp04.3	XLOC_013461	Phvul.006G023900	5.39	2.78	0.0028
Comp04.3	XLOC_014603	Phvul.006G028700	7.13	3.79	0
Comp04.3	XLOC_014622	Phvul.006G033000	-10.58	1.19	0
Comp04.3	XLOC_013583	Phvul.006G043100	5.11	6.33	0.0006
Comp04.3	XLOC_013600	Phvul.006G045800	6.1	6.39	0.0005
Comp04.3	XLOC_013746	Phvul.006G075600	6.64	7.18	0
Comp04.3	XLOC_013747	Phvul.006G075900	4.94	3.42	0.0078
Comp04.3	XLOC_013758	Phvul.006G078800	8.12	5.86	0
Comp04.3	XLOC_013759	Phvul.006G078900	7.6	3.63	0
Comp04.3	XLOC_013761	Phvul.006G079100	8.95	0.17	0.0017
Comp04.3	XLOC_013767	Phvul.006G080600	5.45	6.17	0.0003
Comp04.3	XLOC_013787	Phvul.006G083700	5.18	3.86	0.0007
Comp04.3	XLOC_014876	Phvul.006G083800	6.32	3.25	0.0001
Comp04.3	XLOC_015019	Phvul.006G112300	5.94	2.53	0.0005
Comp04.3	XLOC_013951	Phvul.006G117300	-4.26	2.34	0.0052
Comp04.3	XLOC_015140	Phvul.006G136800	4.45	1.34	0.0079
Comp04.3	XLOC_014051	Phvul.006G137300	4.42	5.9	0.0028
Comp04.3	XLOC_014082	Phvul.006G143300	6.18	1.5	0.0005
Comp04.3	XLOC_014125	Phvul.006G153100	4.1	7.95	0.0057
Comp04.3	XLOC_015293	Phvul.006G168000	5	3.88	0.001
Comp04.3	XLOC_015337	Phvul.006G176500	8.34	0.5	0.0071
Comp04.3	XLOC_015402	Phvul.006G189100	4.97	1.36	0.0068
Comp04.3	XLOC_014335	Phvul.006G194600	4.66	4.83	0.0018
Comp04.3	XLOC_015505	Phvul.006G209500	-5.08	2.71	0.0026
Comp04.3	XLOC_015531	Phvul.006G215400	5.18	1.25	0.0043

Experimental comparison	Gene ID	Correspondent ID (Phytozome)	log_2(FC)[£]	log_2(CPM)[€]	FDR* (all significant at <0.01)
Comp04.3	XLOC_014444	Phvul.006G216700	6.56	6.74	0.0002
Comp04.3	XLOC_017132	Phvul.007G013000	-4.2	2.3	0.0063
Comp04.3	XLOC_015771	Phvul.007G025700	4.52	4.34	0.0028
Comp04.3	XLOC_017232	Phvul.007G031000	6.6	2.29	0.0006
Comp04.3	XLOC_017238	Phvul.007G032200	4.94	2.38	0.0022
Comp04.3	XLOC_017251	Phvul.007G034500	11.45	3.54	0
Comp04.3	XLOC_017318	Phvul.007G048800	11.2	2.57	0
Comp04.3	XLOC_017332	Phvul.007G050300	5.18	2.9	0.0015
Comp04.3	XLOC_015991	Phvul.007G071500	5.58	0.22	0.0065
Comp04.3	XLOC_017546	Phvul.007G092400	4.38	2.46	0.0063
Comp04.3	XLOC_017633	Phvul.007G109000	4.88	4.57	0.0011
Comp04.3	XLOC_017682	Phvul.007G118300	8.6	-0.01	0.0041
Comp04.3	XLOC_017691	Phvul.007G120200	-4.1	5.01	0.0066
Comp04.3	XLOC_016274	Phvul.007G127200	-4.29	0.92	0.0093
Comp04.3	XLOC_017731	Phvul.007G128100	9.29	1.07	0.0008
Comp04.3	XLOC_017754	Phvul.007G133600	-8.23	-0.53	0.0089
Comp04.3	XLOC_016318	Phvul.007G135600	6.5	5.31	0
Comp04.3	XLOC_016441	Phvul.007G158600	-3.91	4.32	0.0089
Comp04.3	XLOC_016446	Phvul.007G160000	4.48	4	0.0033
Comp04.3	XLOC_016486	Phvul.007G167000	5.17	3.89	0.0007
Comp04.3	XLOC_016507	Phvul.007G170900	4.21	2.99	0.0068
Comp04.3	XLOC_018073	Phvul.007G195100	4.61	3.44	0.0024
Comp04.3	XLOC_016643	Phvul.007G198700	5.28	6.05	0.0009
Comp04.3	XLOC_016657	Phvul.007G201100	7.86	2.68	0
Comp04.3	XLOC_018143	Phvul.007G208900	4.51	4.18	0.0044
Comp04.3	XLOC_018152	Phvul.007G210400	10.26	2	0.0001
Comp04.3	XLOC_016707	Phvul.007G211900	4.52	2.24	0.0069
Comp04.3	XLOC_016763	Phvul.007G220500	4.14	3.58	0.0071
Comp04.3	XLOC_016775	Phvul.007G222500	6.24	3.98	0.0001
Comp04.3	XLOC_018243	Phvul.007G231800	8.52	0.58	0.0046
Comp04.3	XLOC_016896	Phvul.007G246700	4.79	1.31	0.0099
Comp04.3	XLOC_016946	Phvul.007G257300	-4.14	2.7	0.0064
Comp04.3	XLOC_016955	Phvul.007G259500	6.94	3.23	0.0001
Comp04.3	XLOC_017043	Phvul.007G275700	11.58	2.24	0
Comp04.3	XLOC_017045	Phvul.007G276400	5.52	5.27	0.0006
Comp04.3	XLOC_017050	Phvul.007G277000	11.19	1.82	0
Comp04.3	XLOC_017069	Phvul.007G280700	5.05	6.64	0.0006
Comp04.3	XLOC_020092	Phvul.008G005200	7.76	3.12	0
Comp04.3	XLOC_020109	Phvul.008G007900	4.53	6.1	0.0021
Comp04.3	XLOC_020110	Phvul.008G008000	5.45	1.29	0.0022
Comp04.3	XLOC_020111	Phvul.008G008100	5.29	7.83	0.0003
Comp04.3	XLOC_018603	Phvul.008G008400	-4.34	5.34	0.0043
Comp04.3	XLOC_018625	Phvul.008G012600	4.15	3.32	0.0069
Comp04.3	XLOC_018647	Phvul.008G016600	4.24	5.58	0.0052
Comp04.3	XLOC_018658	Phvul.008G019600	-4.75	0.65	0.0056
Comp04.3	XLOC_020188	Phvul.008G023600	5.83	2.33	0.0006
Comp04.3	XLOC_018697	Phvul.008G028400, Phvul.008G028500	-5.8	1.35	0.0024

**Experimental comparison**	**Gene ID**	**Correspondent ID (Phytozome)**	**log2(FC)[£]**	**log2(CPM)[€]**	**FDR* (all significant at <0.01)**
Comp04.3	XLOC_018785	Phvul.008G043400	5.21	3.62	0.0011
Comp04.3	XLOC_018791	Phvul.008G045100	4.88	3.05	0.0019
Comp04.3	XLOC_020340	Phvul.008G055200	-9.01	0.5	0.0015
Comp04.3	XLOC_018852	Phvul.008G058400	4.53	2.58	0.0083
Comp04.3	XLOC_018907	Phvul.008G065800	5.46	2.66	0.0007
Comp04.3	XLOC_020387	Phvul.008G068300	9.94	1.48	0.0002
Comp04.3	XLOC_019029	Phvul.008G087500	-5.17	-0.35	0.0055
Comp04.3	XLOC_020490	Phvul.008G089400	4.82	2.13	0.0036
Comp04.3	XLOC_019087	Phvul.008G098200	8.19	4.88	0
Comp04.3	XLOC_020537	Phvul.008G098500	7.31	6.31	0
Comp04.3	XLOC_020575	Phvul.008G105800	5.89	0.78	0.0033
Comp04.3	XLOC_020591	Phvul.008G108900	4.74	4.33	0.0015
Comp04.3	XLOC_019142	Phvul.008G109000	13.46	4	0
Comp04.3	XLOC_019143	Phvul.008G109100	12	7.04	0
Comp04.3	XLOC_019156	Phvul.008G111000	5	2.24	0.0029
Comp04.3	XLOC_020675	Phvul.008G127000	8.8	3.57	0.0026
Comp04.3	XLOC_019249	Phvul.008G127400	4.21	3.26	0.0064
Comp04.3	XLOC_020714	Phvul.008G134100	-4.92	5.05	0.0013
Comp04.3	XLOC_020858	Phvul.008G160400	10.47	2.85	0
Comp04.3	XLOC_020939	Phvul.008G175500	8.17	4.15	0
Comp04.3	XLOC_020998	Phvul.008G186800	8.2	-0.21	0.0089
Comp04.3	XLOC_019543	Phvul.008G188200	4.91	2.44	0.0028
Comp04.3	XLOC_021042	Phvul.008G194600	4.71	2.26	0.0077
Comp04.3	XLOC_019663	Phvul.008G213800	5.65	4.94	0.0002
Comp04.3	XLOC_019664	Phvul.008G213900	5.46	2.05	0.0013
Comp04.3	XLOC_019720	Phvul.008G223500	6.67	6.42	0
Comp04.3	XLOC_021190	Phvul.008G224100	6.39	1.71	0.001
Comp04.3	XLOC_019729	Phvul.008G225600	4.5	4.49	0.0028
Comp04.3	XLOC_021325	Phvul.008G251700	6.05	1.19	0.0022
Comp04.3	XLOC_019871	Phvul.008G252000	7.44	4.18	0
Comp04.3	XLOC_021417	Phvul.008G268200	4.49	2.08	0.0092
Comp04.3	XLOC_019981	Phvul.008G275400	4.08	3.73	0.0075
Comp04.3	XLOC_020011	Phvul.008G282600	4.07	5.96	0.0063
Comp04.3	XLOC_020029	Phvul.008G285200	-5.86	1.01	0.001
Comp04.3	XLOC_023133	Phvul.009G024300	-4.2	1.31	0.0073
Comp04.3	XLOC_021953	Phvul.009G054300	10.21	1.98	0.0001
Comp04.3	XLOC_023283	Phvul.009G054400	4.52	3.36	0.0041
Comp04.3	XLOC_023350	Phvul.009G066900	4.34	4.98	0.0039
Comp04.3	XLOC_023353	Phvul.009G067900	3.92	4.16	0.0092
Comp04.3	XLOC_022077	Phvul.009G078300	5.3	6.64	0.0006
Comp04.3	XLOC_023493	Phvul.009G094000	5.54	4.39	0.0003
Comp04.3	XLOC_023568	Phvul.009G108700	4.48	4.37	0.0041
Comp04.3	XLOC_023571	Phvul.009G109100	-4.59	-0.26	0.0068
Comp04.3	XLOC_022233	Phvul.009G111500	4.54	7.63	0.002
Comp04.3	XLOC_022274	Phvul.009G118800	4.25	4.56	0.0047
Comp04.3	XLOC_022283	Phvul.009G120500	8.81	4.41	0
Comp04.3	XLOC_023645	Phvul.009G125100	-4	10.03	0.0079
Comp04.3	XLOC_022408	Phvul.009G142500	-4.17	4.5	0.0068

Experimental comparison	Gene ID	Correspondent ID (Phytozome)	$log_2(FC)$[£]	$log_2(CPM)$[€]	FDR* (all significant at <0.01)
Comp04.3	XLOC_022460	Phvul.009G152400	5.56	3.04	0.0068
Comp04.3	XLOC_022491	Phvul.009G158100	-6.97	0.11	0.0006
Comp04.3	XLOC_023812	Phvul.009G162000	6.17	3.78	0.0017
Comp04.3	XLOC_022583	Phvul.009G176400	4.54	4.06	0.0026
Comp04.3	XLOC_022600	Phvul.009G179600	8.82	1.96	0.0024
Comp04.3	XLOC_022682	Phvul.009G197000	-4.39	3.35	0.0042
Comp04.3	XLOC_022747	Phvul.009G210900	5.23	2.19	0.0042
Comp04.3	XLOC_022901	Phvul.009G240600	-4.47	4.2	0.0026
Comp04.3	XLOC_025319	Phvul.010G000300	5.14	5.93	0.0006
Comp04.3	XLOC_025336	Phvul.010G004000	4.79	2.04	0.0032
Comp04.3	XLOC_025354	Phvul.010G008800	5.55	6.69	0.0002
Comp04.3	XLOC_024491	Phvul.010G013000	5.6	2.81	0.0005
Comp04.3	XLOC_025376	Phvul.010G013100	11.14	2.53	0
Comp04.3	XLOC_025407	Phvul.010G019300	5.05	3.61	0.0062
Comp04.3	XLOC_025563	Phvul.010G050500	5.01	4.11	0.0008
Comp04.3	XLOC_025564	Phvul.010G050700	4.32	4.33	0.0041
Comp04.3	XLOC_024708	Phvul.010G054200	7.48	2.65	0.0001
Comp04.3	XLOC_025630	Phvul.010G063800	4.11	6.61	0.0059
Comp04.3	XLOC_025020	Phvul.010G111000	6.49	1.77	0.0002
Comp04.3	XLOC_025084	Phvul.010G124000	6.91	4.63	0
Comp04.3	XLOC_025091	Phvul.010G124800	-5.06	3.12	0.0008
Comp04.3	XLOC_025194	Phvul.010G144200	6.16	6.84	0
Comp04.3	XLOC_025196	Phvul.010G144600	11.87	3.5	0
Comp04.3	XLOC_026058	Phvul.010G152800	4.39	4.44	0.0092
Comp04.3	XLOC_026071	Phvul.010G156200	5.5	4.61	0.0012
Comp04.3	XLOC_025279	Phvul.010G158400	4.98	3.72	0.0071
Comp04.3	XLOC_025291	Phvul.010G161100	4.11	6.83	0.0057
Comp04.3	XLOC_026245	Phvul.011G008100	5.06	1.71	0.0056
Comp04.3	XLOC_027401	Phvul.011G008600	4.08	3.75	0.007
Comp04.3	XLOC_027424	Phvul.011G012600	9.18	1.33	0.001
Comp04.3	XLOC_027433	Phvul.011G014000	4.07	4.34	0.0071
Comp04.3	XLOC_027444	Phvul.011G015400	8.57	0.31	0.0043
Comp04.3	XLOC_027493	Phvul.011G025800	8.2	0.05	0.0089
Comp04.3	XLOC_026349	Phvul.011G030200	6.1	1.53	0.0006
Comp04.3	XLOC_026446	Phvul.011G047800	4.29	5.56	0.0048
Comp04.3	XLOC_027605	Phvul.011G049900	3.94	5.79	0.0087
Comp04.3	XLOC_026490	Phvul.011G055900	4.31	8.34	0.0035
Comp04.3	XLOC_026492	Phvul.011G056100	4.17	7.17	0.0048
Comp04.3	XLOC_026519	Phvul.011G060500	-5.91	3.73	0.0065
Comp04.3	XLOC_026528	Phvul.011G062900	4.91	7.76	0.0008
Comp04.3	XLOC_027705	Phvul.011G070400	5.51	0.88	0.0075
Comp04.3	XLOC_027739	Phvul.011G076400	-4.78	5.03	0.0012
Comp04.3	XLOC_026618	Phvul.011G081800	-5.05	1.97	0.001
Comp04.3	XLOC_026624	Phvul.011G083500	-4.5	1.77	0.0038
Comp04.3	XLOC_026695	Phvul.011G099300	10.71	2.1	0
Comp04.3	XLOC_026759	Phvul.011G109700	4.2	5.83	0.0047
Comp04.3	XLOC_026810	Phvul.011G119500	9.4	2.6	0.0006
Comp04.3	XLOC_026860	Phvul.011G127100	8.23	3.69	0

Experimental comparison	Gene ID	Correspondent ID (Phytozome)	$log_2(FC)^£$	$log_2(CPM)^€$	FDR* (all significant at <0.01)
Comp04.3	XLOC_026913	Phvul.011G136600	-5.21	4.28	0.0016
Comp04.3	XLOC_026949	Phvul.011G142300	6.18	5.86	0
Comp04.3	XLOC_026983	Phvul.011G147800	3.89	7.56	0.0093
Comp04.3	XLOC_026986	Phvul.011G148500	4.72	4.45	0.0021
Comp04.3	XLOC_026997	Phvul.011G150400	5.61	3.52	0.0007
Comp04.3	XLOC_027168	Phvul.011G183600	9.74	1.51	0.0003
Comp04.3	XLOC_027170	Phvul.011G183800	9.53	4.42	0.0005
Comp04.3	XLOC_027171	Phvul.011G183900	5.72	2.93	0.0048
Comp04.3	XLOC_027174	Phvul.011G184200	-4.66	5.16	0.0015
Comp04.3	XLOC_027177	Phvul.011G184600	-4.65	0.92	0.0043
Comp04.3	XLOC_027206	Phvul.011G189200	8.17	0.52	0.0097
Comp04.3	XLOC_027207	Phvul.011G189300	6.63	4.01	0.0001
Comp04.3	XLOC_027211	Phvul.011G189900	3.91	4.9	0.0095
Comp04.3	XLOC_027236	Phvul.011G194000, Phvul.011G194100	5.52	1.97	0.0019
Comp04.3	XLOC_028291	Phvul.011G194400	4.37	4.19	0.0039
Comp04.3	XLOC_027249	Phvul.011G197000	9.61	3.7	0.0004
Comp04.3	XLOC_027261	Phvul.011G198800	-4.3	1.42	0.0062
Comp04.3	XLOC_027277	Phvul.011G201700	4.06	5	0.0068
Comp04.3	XLOC_028570	Phvul.L003000	4.96	1.26	0.0069
Comp04.3	XLOC_028531	Phvul.L008100	5	4.05	0.0012
Comp05.1	XLOC_001043	Phvul.001G205900	6.68	7.01	0.0006
Comp05.1	XLOC_015984	Phvul.007G069800	6.16	7.31	0.0012
Comp05.2	XLOC_006748	new loci	-9.58	2.29	0.0036
Comp05.2	XLOC_002707	Phvul.001G264400	8.16	3	0
Comp05.2	XLOC_003990	Phvul.002G231400	5.66	4.47	0.0014
Comp05.2	XLOC_006445	Phvul.003G031300	5.63	3.96	0.002
Comp05.2	XLOC_006675	Phvul.003G079800	6.03	3.94	0.0035
Comp05.2	XLOC_008496	Phvul.003G121900	5.85	3.57	0.0022
Comp05.2	XLOC_009847	Phvul.004G090200	4.98	4.38	0.0089
Comp05.2	XLOC_010989	Phvul.004G129400	7.01	1.38	0.0013
Comp05.2	XLOC_014301	Phvul.006G188500	5.83	6.31	0.0013
Comp05.2	XLOC_017662	Phvul.007G113700	5.77	3.93	0.0014
Comp05.2	XLOC_019637	Phvul.008G207900	5.7	6.59	0.0013
Comp05.2	XLOC_019741	Phvul.008G228000	6.82	3.06	0.0006
Comp05.2	XLOC_023245	Phvul.009G046500	11.71	1.82	0
Comp05.2	XLOC_027984	Phvul.011G125300	9.53	0.92	0.0039
Comp05.3	XLOC_006160	new loci	-6.35	-0.04	0.0017
Comp05.3	XLOC_006298	new loci	-6.83	0.32	0.0005
Comp05.3	XLOC_006748	new loci	-10.22	2.29	0.0001
Comp05.3	XLOC_009338	new loci	-8.52	0.73	0.0063
Comp05.3	XLOC_026190	new loci	-4.85	0.79	0.0057
Comp05.3	XLOC_001481	Phvul.001G024400	-5.73	1.51	0.0071
Comp05.3	XLOC_001507	Phvul.001G029100	4.19	4.19	0.0076
Comp05.3	XLOC_000186	Phvul.001G039700	7.29	6.48	0
Comp05.3	XLOC_000187	Phvul.001G039800	7.81	4.89	0
Comp05.3	XLOC_000231	Phvul.001G050400	-6.61	0.83	0.0002
Comp05.3	XLOC_001674	Phvul.001G059600	4.16	4.61	0.0077

Experimental comparison	Gene ID	Correspondent ID (Phytozome)	log_2(FC)[£]	log_2(CPM)[€]	FDR* (all significant at <0.01)
Comp05.3	XLOC_000305	Phvul.001G064000	8.89	-0.15	0.0028
Comp05.3	XLOC_001707	Phvul.001G067400	4.19	5.06	0.0072
Comp05.3	XLOC_001744	Phvul.001G075400	6.19	7.44	0
Comp05.3	XLOC_002045	Phvul.001G135200	5.65	8.14	0.0001
Comp05.3	XLOC_002080	Phvul.001G142000	4.79	4.09	0.0018
Comp05.3	XLOC_002085	Phvul.001G143100	4.59	5.72	0.0027
Comp05.3	XLOC_002095	Phvul.001G145600	10.49	4.88	0
Comp05.3	XLOC_000773	Phvul.001G152100	-8.76	3.97	0.0037
Comp05.3	XLOC_002147	Phvul.001G154500	4.86	2.43	0.0029
Comp05.3	XLOC_000797	Phvul.001G156900	4.28	7.14	0.0049
Comp05.3	XLOC_000813	Phvul.001G160100	6.35	1.51	0.0027
Comp05.3	XLOC_000830	Phvul.001G162800	4.35	5.81	0.0045
Comp05.3	XLOC_000886	Phvul.001G174200	-5.22	4.13	0.0006
Comp05.3	XLOC_002266	Phvul.001G177100	6.58	4.59	0
Comp05.3	XLOC_000947	Phvul.001G184600	7.89	3.77	0
Comp05.3	XLOC_000952	Phvul.001G185700	10.17	0.73	0.0001
Comp05.3	XLOC_001043	Phvul.001G205900	8.6	7.01	0
Comp05.3	XLOC_002440	Phvul.001G211300	7.69	2.33	0.0001
Comp05.3	XLOC_001090	Phvul.001G215700	-4.67	5.08	0.0028
Comp05.3	XLOC_001116	Phvul.001G219300	-4.59	4.09	0.0035
Comp05.3	XLOC_001149	Phvul.001G226300	5.74	4.4	0.0002
Comp05.3	XLOC_001179	Phvul.001G231900	5.89	3.81	0.0002
Comp05.3	XLOC_001252	Phvul.001G246500	9.34	0.52	0.0009
Comp05.3	XLOC_002650	Phvul.001G255200	4.9	6	0.0012
Comp05.3	XLOC_001329	Phvul.001G263200	5.98	2.38	0.0008
Comp05.3	XLOC_002707	Phvul.001G264400	12.77	3	0
Comp05.3	XLOC_002947	Phvul.002G019100	5.38	7.3	0.0003
Comp05.3	XLOC_004604	Phvul.002G021800	7.18	2.8	0
Comp05.3	XLOC_004634	Phvul.002G029900	8.61	3.48	0
Comp05.3	XLOC_004642	Phvul.002G031100	4.81	1.84	0.0042
Comp05.3	XLOC_004716	Phvul.002G043000	4.82	5.31	0.0037
Comp05.3	XLOC_004758	Phvul.002G050700	5.42	4.83	0.0003
Comp05.3	XLOC_004851	Phvul.002G072000	4.64	6.14	0.0029
Comp05.3	XLOC_003242	Phvul.002G076700	-4.91	1.39	0.0045
Comp05.3	XLOC_003281	Phvul.002G083800	-4.79	0.9	0.0066
Comp05.3	XLOC_003331	Phvul.002G093000	5.32	3.21	0.0005
Comp05.3	XLOC_004996	Phvul.002G103300	5.81	2.93	0.0013
Comp05.3	XLOC_005117	Phvul.002G124100	-4.67	4.4	0.0024
Comp05.3	XLOC_005214	Phvul.002G140000	4.34	5.59	0.0046
Comp05.3	XLOC_005337	Phvul.002G163900	6.87	3.75	0.0001
Comp05.3	XLOC_003698	Phvul.002G170200	6.06	3.92	0.0014
Comp05.3	XLOC_003702	Phvul.002G170800	6.16	5.78	0
Comp05.3	XLOC_003833	Phvul.002G197800	5.14	6.69	0.0006
Comp05.3	XLOC_005555	Phvul.002G204900	4.64	3.68	0.0038
Comp05.3	XLOC_003931	Phvul.002G218300	7.37	4.56	0
Comp05.3	XLOC_003990	Phvul.002G231400	9.64	4.47	0
Comp05.3	XLOC_003991	Phvul.002G231500	6.18	6.44	0
Comp05.3	XLOC_003992	Phvul.002G231600	7.2	6.1	0

Experimental comparison	Gene ID	Correspondent ID (Phytozome)	log_2(FC)[£]	log_2(CPM)[€]	FDR* (all significant at <0.01)
Comp05.3	XLOC_003993	Phvul.002G231700	7.76	5.61	0
Comp05.3	XLOC_003994	Phvul.002G231800	5.57	3.03	0.0003
Comp05.3	XLOC_003998	Phvul.002G232800	4.39	1.47	0.0083
Comp05.3	XLOC_004034	Phvul.002G239300	5.61	2.96	0.0004
Comp05.3	XLOC_004194	Phvul.002G271900	7.31	5.16	0
Comp05.3	XLOC_005934	Phvul.002G280400	8.35	2.7	0
Comp05.3	XLOC_004259	Phvul.002G285800	11.11	3.25	0
Comp05.3	XLOC_005973	Phvul.002G287300	-4.26	3.38	0.0068
Comp05.3	XLOC_006077	Phvul.002G309100	4.45	5.26	0.0037
Comp05.3	XLOC_004423	Phvul.002G317000	4.58	4.64	0.0031
Comp05.3	XLOC_004443	Phvul.002G320600	4.7	5.89	0.0022
Comp05.3	XLOC_006158	Phvul.002G325400	-8.66	1.79	0.0046
Comp05.3	XLOC_004494	Phvul.002G332300	5.71	5.4	0.0002
Comp05.3	XLOC_006445	Phvul.003G031300	4.61	3.96	0.0029
Comp05.3	XLOC_008099	Phvul.003G044600	4.32	5.94	0.0055
Comp05.3	XLOC_008210	Phvul.003G064300	4.25	5.95	0.0099
Comp05.3	XLOC_008212	Phvul.003G064500	4.98	5.2	0.0009
Comp05.3	XLOC_006675	Phvul.003G079800	7.96	3.94	0
Comp05.3	XLOC_008361	Phvul.003G096700	4.44	4.53	0.0046
Comp05.3	XLOC_008478	Phvul.003G118400	4.96	7.65	0.0009
Comp05.3	XLOC_008496	Phvul.003G121900	5.5	3.57	0.0005
Comp05.3	XLOC_008510	Phvul.003G126300	4.79	7.65	0.0014
Comp05.3	XLOC_008553	Phvul.003G135400	6.2	2.86	0.0002
Comp05.3	XLOC_008653	Phvul.003G154800	7.22	6.43	0
Comp05.3	XLOC_007116	Phvul.003G160900	5.26	6.55	0.0005
Comp05.3	XLOC_008779	Phvul.003G177800	7	2.43	0.0001
Comp05.3	XLOC_007228	Phvul.003G184200	4.85	2.82	0.0025
Comp05.3	XLOC_008825	Phvul.003G187200	4.23	5.84	0.0069
Comp05.3	XLOC_008923	Phvul.003G209000	4.1	6.16	0.0077
Comp05.3	XLOC_007359	Phvul.003G209100	4.9	6.33	0.0012
Comp05.3	XLOC_007402	Phvul.003G217200	8.78	3	0.0035
Comp05.3	XLOC_007475	Phvul.003G230300	5.98	1.26	0.0065
Comp05.3	XLOC_007528	Phvul.003G239800	5.68	4.59	0.0035
Comp05.3	XLOC_007606	Phvul.003G252500	4.09	3.57	0.0095
Comp05.3	XLOC_009487	Phvul.004G014700	5.17	2.51	0.0013
Comp05.3	XLOC_009502	Phvul.004G018900	6	7.05	0.0004
Comp05.3	XLOC_009519	Phvul.004G021300	-9.65	0.72	0.0004
Comp05.3	XLOC_010510	Phvul.004G041700	-5.09	1.93	0.0025
Comp05.3	XLOC_010527	Phvul.004G046200	4.72	4.04	0.0032
Comp05.3	XLOC_010549	Phvul.004G049900	4.47	2.95	0.0068
Comp05.3	XLOC_009680	Phvul.004G055200	5.05	5.13	0.0083
Comp05.3	XLOC_010708	Phvul.004G077400	5.59	4.26	0.0004
Comp05.3	XLOC_009847	Phvul.004G090200	13.05	4.38	0
Comp05.3	XLOC_010783	Phvul.004G090300	6.19	1.2	0.0038
Comp05.3	XLOC_010789	Phvul.004G092100	7.37	6.35	0
Comp05.3	XLOC_010873	Phvul.004G107700	4.14	9.08	0.0069
Comp05.3	XLOC_009977	Phvul.004G117100	6.36	4.03	0.0002
Comp05.3	XLOC_010934	Phvul.004G118200	8.92	0.17	0.0027

Experimental comparison	Gene ID	Correspondent ID (Phytozome)	$log_2(FC)$[£]	$log_2(CPM)$[€]	FDR* (all significant at <0.01)
Comp05.3	XLOC_010014	Phvul.004G123600	-6.07	3.1	0.0002
Comp05.3	XLOC_010989	Phvul.004G129400	11.07	1.38	0
Comp05.3	XLOC_010087	Phvul.004G138500	4.53	8.13	0.0028
Comp05.3	XLOC_011088	Phvul.004G148400	4.39	6.34	0.0045
Comp05.3	XLOC_010153	Phvul.004G150400	-5.08	0.35	0.0089
Comp05.3	XLOC_011146	Phvul.004G158800	7.87	2.74	0
Comp05.3	XLOC_010197	Phvul.004G162100	4.33	6.04	0.0046
Comp05.3	XLOC_011206	Phvul.004G168400	-4.09	4.28	0.0084
Comp05.3	XLOC_012436	Phvul.005G032000	6.05	2.09	0.0014
Comp05.3	XLOC_011560	Phvul.005G044200	8.81	1.06	0.0033
Comp05.3	XLOC_011600	Phvul.005G051600	4.71	8.2	0.0018
Comp05.3	XLOC_012538	Phvul.005G054200	8.75	1.88	0.0039
Comp05.3	XLOC_012655	Phvul.005G075500	5.92	5.51	0.0001
Comp05.3	XLOC_011754	Phvul.005G084500	4.51	8.3	0.0029
Comp05.3	XLOC_011821	Phvul.005G096300	7.03	2.08	0.0005
Comp05.3	XLOC_012850	Phvul.005G108100	8.53	0.68	0.0063
Comp05.3	XLOC_012856	Phvul.005G108800	4.5	3.42	0.0077
Comp05.3	XLOC_012861	Phvul.005G109700	5.71	1.46	0.0034
Comp05.3	XLOC_012866	Phvul.005G111100	-4.87	0.04	0.0077
Comp05.3	XLOC_012016	Phvul.005G138400	5.21	3.5	0.0016
Comp05.3	XLOC_013160	Phvul.005G166800	-4.8	2.54	0.0027
Comp05.3	XLOC_012155	Phvul.005G167800	6.01	3.76	0.0001
Comp05.3	XLOC_013191	Phvul.005G174800	7.12	4.03	0
Comp05.3	XLOC_013220	Phvul.005G181300	4.56	2.71	0.0069
Comp05.3	XLOC_014496	Phvul.006G005100	5.99	5.7	0.0001
Comp05.3	XLOC_013461	Phvul.006G023900	-4.37	2.78	0.0074
Comp05.3	XLOC_013602	Phvul.006G046000	7.91	5.48	0
Comp05.3	XLOC_014806	Phvul.006G069300	4.62	9.48	0.0022
Comp05.3	XLOC_013746	Phvul.006G075600	5.26	7.18	0.0051
Comp05.3	XLOC_013763	Phvul.006G079500	8.55	4.84	0
Comp05.3	XLOC_013764	Phvul.006G079600	7.95	4.73	0
Comp05.3	XLOC_015037	Phvul.006G116000	10.02	4.99	0
Comp05.3	XLOC_015038	Phvul.006G116500	-5.04	0.47	0.0099
Comp05.3	XLOC_013951	Phvul.006G117300	-8.69	2.34	0.0045
Comp05.3	XLOC_015099	Phvul.006G129500	5.79	1.7	0.0009
Comp05.3	XLOC_014037	Phvul.006G133600	-4.18	7.2	0.0065
Comp05.3	XLOC_014194	Phvul.006G166000	9.34	1.42	0.0009
Comp05.3	XLOC_015295	Phvul.006G168600	5.85	3.39	0.0008
Comp05.3	XLOC_015321	Phvul.006G173000	4.29	4.04	0.0087
Comp05.3	XLOC_014301	Phvul.006G188500	6.96	6.31	0
Comp05.3	XLOC_014303	Phvul.006G188900	5.47	2.79	0.0008
Comp05.3	XLOC_015746	Phvul.007G021100	4.32	2.57	0.0077
Comp05.3	XLOC_017251	Phvul.007G034500	8.32	3.54	0
Comp05.3	XLOC_017298	Phvul.007G044500	4.31	5.79	0.0058
Comp05.3	XLOC_015943	Phvul.007G061800	6.76	3.14	0.0002
Comp05.3	XLOC_017406	Phvul.007G066200	7.01	1.21	0.0005
Comp05.3	XLOC_015984	Phvul.007G069800	8.4	7.31	0
Comp05.3	XLOC_016021	Phvul.007G077300	4.2	3.41	0.0083

**Experimental comparison**	**Gene ID**	**Correspondent ID (Phytozome)**	**log_2(FC)[£]**	**log_2(CPM)[€]**	**FDR* (all significant at <0.01)**
Comp05.3	XLOC_016049	Phvul.007G082000	8.51	4.46	0
Comp05.3	XLOC_017592	Phvul.007G100700	6.86	5.28	0
Comp05.3	XLOC_017662	Phvul.007G113700	10.47	3.93	0
Comp05.3	XLOC_017729	Phvul.007G127800	4.45	4.37	0.0046
Comp05.3	XLOC_017781	Phvul.007G138300	8.66	-0.88	0.0046
Comp05.3	XLOC_016486	Phvul.007G167000	5.42	3.89	0.0024
Comp05.3	XLOC_017998	Phvul.007G181100	-7.07	1.1	0.0003
Comp05.3	XLOC_016666	Phvul.007G203400	4.31	8.26	0.0046
Comp05.3	XLOC_018192	Phvul.007G220200	4.47	5.39	0.0039
Comp05.3	XLOC_016774	Phvul.007G222400	5.07	4.5	0.0008
Comp05.3	XLOC_016775	Phvul.007G222500	4.95	3.98	0.0019
Comp05.3	XLOC_018216	Phvul.007G225200	7.9	4.59	0
Comp05.3	XLOC_016866	Phvul.007G239800	4.47	3.9	0.0039
Comp05.3	XLOC_018388	Phvul.007G259400	7.53	6.7	0
Comp05.3	XLOC_016955	Phvul.007G259500	4.76	3.23	0.0035
Comp05.3	XLOC_018465	Phvul.007G276100	5.7	5.65	0.0012
Comp05.3	XLOC_018466	Phvul.007G276200	4.98	5	0.0009
Comp05.3	XLOC_017045	Phvul.007G276400	6.61	5.27	0
Comp05.3	XLOC_017046	Phvul.007G276500	5.91	6.52	0.0001
Comp05.3	XLOC_017047	Phvul.007G276600	5.59	2.47	0.0003
Comp05.3	XLOC_018469	Phvul.007G277000	-8.4	1.39	0.0083
Comp05.3	XLOC_017059	Phvul.007G278800	-4.82	4.38	0.0089
Comp05.3	XLOC_018646	Phvul.008G016500	4.87	5.81	0.0013
Comp05.3	XLOC_018647	Phvul.008G016600	4.32	5.58	0.0049
Comp05.3	XLOC_020173	Phvul.008G020100	-4.31	2.47	0.0069
Comp05.3	XLOC_018722	Phvul.008G032200	4.31	6.13	0.0052
Comp05.3	XLOC_020227	Phvul.008G032400	4.6	4.31	0.0033
Comp05.3	XLOC_018777	Phvul.008G041800	-5.16	1.82	0.002
Comp05.3	XLOC_018785	Phvul.008G043400	5.82	3.62	0.0006
Comp05.3	XLOC_020307	Phvul.008G048400	5.05	2.42	0.0018
Comp05.3	XLOC_020317	Phvul.008G050900	4.4	2	0.0099
Comp05.3	XLOC_020394	Phvul.008G069400	-4.34	6.06	0.0045
Comp05.3	XLOC_020416	Phvul.008G073200	-5.23	3.52	0.0006
Comp05.3	XLOC_020537	Phvul.008G098500	7.56	6.31	0
Comp05.3	XLOC_019109	Phvul.008G103500	-4.2	6.49	0.0062
Comp05.3	XLOC_020644	Phvul.008G121000	5.7	1.83	0.0035
Comp05.3	XLOC_019389	Phvul.008G155000	4.19	4.18	0.0077
Comp05.3	XLOC_019489	Phvul.008G176100	4.18	2.87	0.0086
Comp05.3	XLOC_020995	Phvul.008G186200	8.19	5.97	0
Comp05.3	XLOC_019637	Phvul.008G207900	7.44	6.59	0
Comp05.3	XLOC_019705	Phvul.008G220400	4.92	3.95	0.0019
Comp05.3	XLOC_021203	Phvul.008G226200	4.57	5	0.0035
Comp05.3	XLOC_019741	Phvul.008G228000	8.15	3.06	0
Comp05.3	XLOC_021212	Phvul.008G228100	6.4	5.94	0
Comp05.3	XLOC_021370	Phvul.008G259700	5.2	5.35	0.0006
Comp05.3	XLOC_021381	Phvul.008G261200	4.47	0.88	0.0093
Comp05.3	XLOC_021477	Phvul.008G279600	-4.27	3.55	0.0068
Comp05.3	XLOC_021509	Phvul.008G287200	6.41	7.09	0.0002

Experimental comparison	Gene ID	Correspondent ID (Phytozome)	log_2(FC)[£]	log_2(CPM)[€]	FDR* (all significant at <0.01)
Comp05.3	XLOC_023046	Phvul.009G005300	9.22	0.49	0.0012
Comp05.3	XLOC_021779	Phvul.009G018300	-4.62	2.54	0.0033
Comp05.3	XLOC_021896	Phvul.009G042800	5.13	3.21	0.0069
Comp05.3	XLOC_023245	Phvul.009G046500	11.75	1.82	0
Comp05.3	XLOC_022020	Phvul.009G068000	4.23	5.29	0.0064
Comp05.3	XLOC_022066	Phvul.009G075500	5.67	6.13	0.0004
Comp05.3	XLOC_022077	Phvul.009G078300	5.4	6.64	0.0003
Comp05.3	XLOC_023416	Phvul.009G080200	5.8	5.4	0.0001
Comp05.3	XLOC_022176	Phvul.009G100300	-5.71	1.86	0.0005
Comp05.3	XLOC_022286	Phvul.009G121000	-5.49	7.7	0.0002
Comp05.3	XLOC_022413	Phvul.009G143700	-4.67	1.55	0.0077
Comp05.3	XLOC_022460	Phvul.009G152400	8.11	3.04	0
Comp05.3	XLOC_022461	Phvul.009G152500	7.58	4.82	0
Comp05.3	XLOC_022546	Phvul.009G168000	4.75	2.88	0.0034
Comp05.3	XLOC_023873	Phvul.009G173900	4.45	1.87	0.0081
Comp05.3	XLOC_022614	Phvul.009G182300	5.39	8.41	0.0003
Comp05.3	XLOC_025422	Phvul.010G022000	4.88	3.05	0.0025
Comp05.3	XLOC_025582	Phvul.010G056200	-8.46	-0.66	0.0072
Comp05.3	XLOC_024811	Phvul.010G073500	4.56	3.16	0.0046
Comp05.3	XLOC_025735	Phvul.010G083700	5.42	4.79	0.0003
Comp05.3	XLOC_025790	Phvul.010G093500	5.54	3.82	0.0013
Comp05.3	XLOC_025803	Phvul.010G096900	4.64	0.99	0.0051
Comp05.3	XLOC_025845	Phvul.010G107400	6.6	7.04	0
Comp05.3	XLOC_025861	Phvul.010G111200	4.12	6.12	0.0074
Comp05.3	XLOC_025890	Phvul.010G117200	4.86	7.16	0.0012
Comp05.3	XLOC_025075	Phvul.010G122500	4.92	4.03	0.0075
Comp05.3	XLOC_025930	Phvul.010G125300	6.68	5.41	0
Comp05.3	XLOC_026011	Phvul.010G140800, Phvul.010G140900	-4.34	4.74	0.0046
Comp05.3	XLOC_026064	Phvul.010G154400	4.88	6.87	0.0017
Comp05.3	XLOC_025259	Phvul.010G155300	9.34	0.11	0.0009
Comp05.3	XLOC_025306	Phvul.010G163600	6.27	1.83	0.0008
Comp05.3	XLOC_026275	Phvul.011G016100	4.69	6.6	0.0019
Comp05.3	XLOC_026276	Phvul.011G016200	4.54	2.22	0.0046
Comp05.3	XLOC_026365	Phvul.011G033800	4.69	4.71	0.0027
Comp05.3	XLOC_026395	Phvul.011G037900	-5.88	2.67	0.0002
Comp05.3	XLOC_027784	Phvul.011G084300	5.38	7.38	0.0003
Comp05.3	XLOC_026682	Phvul.011G097100	4.94	3.89	0.002
Comp05.3	XLOC_027899	Phvul.011G106300	4.47	1.93	0.0077
Comp05.3	XLOC_026810	Phvul.011G119500	7.35	2.6	0
Comp05.3	XLOC_027984	Phvul.011G125300	9.57	0.92	0.0005
Comp05.3	XLOC_026860	Phvul.011G127100	8.72	3.69	0.0042
Comp05.3	XLOC_026983	Phvul.011G147800	7.39	7.56	0
Comp05.3	XLOC_026986	Phvul.011G148500	4.64	4.45	0.0038
Comp05.3	XLOC_027100	Phvul.011G167000	4.88	1.04	0.0035
Comp05.3	XLOC_028324	Phvul.011G201000	-5.81	1.14	0.0062
Comp05.3	XLOC_027333	Phvul.011G211600	7.62	4.35	0
Comp05.3	XLOC_028499	Phvul.L006900	-4.57	0.84	0.0081

Experimental comparison	Gene ID	Correspondent ID (Phytozome)	$log_2(FC)$[£]	$log_2(CPM)$[€]	FDR* (all significant at <0.01)
Comp05.3	XLOC_028531	Phvul.L008100	5.17	4.05	0.0043
Comp06.2	XLOC_001074	Phvul.001G211900	9.4	-0.53	0.0056
Comp06.2	XLOC_002707	Phvul.001G264400	5.84	3	0.0025
Comp06.2	XLOC_004634	Phvul.002G029900	5.48	3.48	0.0025
Comp06.2	XLOC_003931	Phvul.002G218300	4.93	4.56	0.0062
Comp06.2	XLOC_006445	Phvul.003G031300	4.93	3.96	0.0063
Comp06.2	XLOC_008496	Phvul.003G121900	5.36	3.57	0.003
Comp06.2	XLOC_008510	Phvul.003G126300	5.77	7.65	0.0016
Comp06.2	XLOC_008696	Phvul.003G163400	5.12	5.25	0.0061
Comp06.2	XLOC_007359	Phvul.003G209100	4.99	6.33	0.0057
Comp06.2	XLOC_007475	Phvul.003G230300	5.42	1.26	0.0062
Comp06.2	XLOC_010367	Phvul.004G014600	5.48	2.83	0.0054
Comp06.2	XLOC_010852	Phvul.004G103900	5.92	2.28	0.003
Comp06.2	XLOC_009962	Phvul.004G111900	4.98	4.27	0.0062
Comp06.2	XLOC_014296	Phvul.006G187500	5.77	8.02	0.0017
Comp06.2	XLOC_014301	Phvul.006G188500	4.87	6.31	0.0062
Comp06.2	XLOC_017406	Phvul.007G066200	10.64	1.21	0.0004
Comp06.2	XLOC_018216	Phvul.007G225200	4.75	4.59	0.0091
Comp06.2	XLOC_019518	Phvul.008G183400	9.22	-0.46	0.0063
Comp06.2	XLOC_019637	Phvul.008G207900	5.38	6.59	0.0025
Comp06.2	XLOC_019741	Phvul.008G228000	6.78	3.06	0.0004
Comp06.2	XLOC_022357	Phvul.009G132900	5.6	4.58	0.0025
Comp06.2	XLOC_022465	Phvul.009G153300	6.38	0.21	0.0062
Comp06.2	XLOC_025341	Phvul.010G005600	5.86	3.45	0.0022
Comp06.2	XLOC_026064	Phvul.010G154400	5.75	6.87	0.0017
Comp06.2	XLOC_026682	Phvul.011G097100	7.03	3.89	0.0002
Comp06.3	XLOC_005373	new loci	-6.03	-0.33	0.0052
Comp06.3	XLOC_006267	new loci	-6.26	1.21	0.0032
Comp06.3	XLOC_009335	new loci	5.87	-0.68	0.008
Comp06.3	XLOC_013252	new loci	6.49	1.98	0.0005
Comp06.3	XLOC_015625	new loci	8.54	-1.2	0.0073
Comp06.3	XLOC_028640	new loci	-6.8	1.44	0.0009
Comp06.3	XLOC_001440	Phvul.001G017200	5.78	-0.56	0.0097
Comp06.3	XLOC_001443	Phvul.001G018300	-5.45	2.7	0.002
Comp06.3	XLOC_000231	Phvul.001G050400	-8.42	0.83	0.0092
Comp06.3	XLOC_001651	Phvul.001G055000	6.34	1.31	0.0027
Comp06.3	XLOC_001738	Phvul.001G074200	9.44	1.87	0.0009
Comp06.3	XLOC_000573	Phvul.001G112400	5.82	6.22	0.0004
Comp06.3	XLOC_001929	Phvul.001G112500	5.26	4.74	0.0008
Comp06.3	XLOC_002010	Phvul.001G127700	-4.21	3.66	0.0094
Comp06.3	XLOC_000668	Phvul.001G131000	5.46	4.57	0.0012
Comp06.3	XLOC_002045	Phvul.001G135200	4.63	8.14	0.0026
Comp06.3	XLOC_002085	Phvul.001G143100	4.69	5.72	0.0024
Comp06.3	XLOC_002146	Phvul.001G154200	-4.93	6.13	0.0013
Comp06.3	XLOC_002147	Phvul.001G154500	5.43	2.43	0.0008
Comp06.3	XLOC_000830	Phvul.001G162800	4.79	5.81	0.0018
Comp06.3	XLOC_000843	Phvul.001G165200	-5.51	5.27	0.0004
Comp06.3	XLOC_000886	Phvul.001G174200	-5.49	4.13	0.0005

Experimental comparison	Gene ID	Correspondent ID (Phytozome)	$log_2(FC)$[£]	$log_2(CPM)$[€]	FDR* (all significant at <0.01)
Comp06.3	XLOC_000947	Phvul.001G184600	5.15	3.77	0.0009
Comp06.3	XLOC_000952	Phvul.001G185700	9.79	0.73	0.0004
Comp06.3	XLOC_001043	Phvul.001G205900	4.52	7.01	0.0035
Comp06.3	XLOC_002439	Phvul.001G211200	4.49	2.51	0.0055
Comp06.3	XLOC_002440	Phvul.001G211300	6.51	2.33	0.0001
Comp06.3	XLOC_001074	Phvul.001G211900	9.63	-0.53	0.0006
Comp06.3	XLOC_001090	Phvul.001G215700	-5.26	5.08	0.0015
Comp06.3	XLOC_001116	Phvul.001G219300	-5.11	4.09	0.002
Comp06.3	XLOC_002496	Phvul.001G223700	6.25	5.22	0.0001
Comp06.3	XLOC_001252	Phvul.001G246500	6.85	0.52	0.0008
Comp06.3	XLOC_001329	Phvul.001G263200	4.44	2.38	0.0069
Comp06.3	XLOC_002707	Phvul.001G264400	8.78	3	0
Comp06.3	XLOC_004604	Phvul.002G021800	7.13	2.8	0
Comp06.3	XLOC_002995	Phvul.002G027900	9.55	-0.33	0.0007
Comp06.3	XLOC_004634	Phvul.002G029900	6.46	3.48	0.0001
Comp06.3	XLOC_004642	Phvul.002G031100	5.06	1.84	0.002
Comp06.3	XLOC_003281	Phvul.002G083800	-5.2	0.9	0.0054
Comp06.3	XLOC_003404	Phvul.002G105700	5.32	0.49	0.0029
Comp06.3	XLOC_005117	Phvul.002G124100	-7.75	4.4	0
Comp06.3	XLOC_005224	Phvul.002G141900	-4.67	4.49	0.0034
Comp06.3	XLOC_005337	Phvul.002G163900	5.75	3.75	0.0003
Comp06.3	XLOC_003698	Phvul.002G170200	5.1	3.92	0.0027
Comp06.3	XLOC_003702	Phvul.002G170800	4.43	5.78	0.0045
Comp06.3	XLOC_005426	Phvul.002G180900	4.9	1	0.0049
Comp06.3	XLOC_003833	Phvul.002G197800	5.59	6.69	0.0003
Comp06.3	XLOC_003915	Phvul.002G215100	9.31	0.82	0.0012
Comp06.3	XLOC_003931	Phvul.002G218300	7.66	4.56	0
Comp06.3	XLOC_003990	Phvul.002G231400	6.07	4.47	0.0002
Comp06.3	XLOC_003992	Phvul.002G231600	4.22	6.1	0.0069
Comp06.3	XLOC_003993	Phvul.002G231700	4.49	5.61	0.0039
Comp06.3	XLOC_004034	Phvul.002G239300	5.36	2.96	0.0008
Comp06.3	XLOC_004194	Phvul.002G271900	4.32	5.16	0.0057
Comp06.3	XLOC_005934	Phvul.002G280400	5.46	2.7	0.0011
Comp06.3	XLOC_004259	Phvul.002G285800	6.23	3.25	0.0005
Comp06.3	XLOC_004419	Phvul.002G316100	-4.95	4.57	0.0014
Comp06.3	XLOC_004465	Phvul.002G326700	-5.18	1.12	0.0099
Comp06.3	XLOC_004494	Phvul.002G332300	4.22	5.4	0.0069
Comp06.3	XLOC_007905	Phvul.003G007200	4.63	4.43	0.0031
Comp06.3	XLOC_007906	Phvul.003G007300	-5.46	2.95	0.001
Comp06.3	XLOC_006435	Phvul.003G029400	4.97	1.85	0.0064
Comp06.3	XLOC_006445	Phvul.003G031300	5.43	3.96	0.0005
Comp06.3	XLOC_008080	Phvul.003G041200	-5.27	4.16	0.0031
Comp06.3	XLOC_008132	Phvul.003G049800	-6.03	2.34	0.0008
Comp06.3	XLOC_008212	Phvul.003G064500	4.52	5.2	0.0036
Comp06.3	XLOC_006675	Phvul.003G079800	6.96	3.94	0.0002
Comp06.3	XLOC_008341	Phvul.003G092100	-4.26	4.9	0.0068
Comp06.3	XLOC_008361	Phvul.003G096700	4.3	4.53	0.006
Comp06.3	XLOC_008430	Phvul.003G109000	4.63	6.17	0.0046

**Experimental comparison**	**Gene ID**	**Correspondent ID (Phytozome)**	**$log_2(FC)^£$**	**$log_2(CPM)^€$**	**FDR* (all significant at <0.01)**
Comp06.3	XLOC_008478	Phvul.003G118400	4.1	7.65	0.0092
Comp06.3	XLOC_008496	Phvul.003G121900	5.99	3.57	0.0002
Comp06.3	XLOC_008510	Phvul.003G126300	5.49	7.65	0.0004
Comp06.3	XLOC_006971	Phvul.003G133400	5.18	2.52	0.0031
Comp06.3	XLOC_008618	Phvul.003G148600	-4.77	2.61	0.0037
Comp06.3	XLOC_008653	Phvul.003G154800	6.22	6.43	0.0001
Comp06.3	XLOC_008696	Phvul.003G163400	5.85	5.25	0.0003
Comp06.3	XLOC_008779	Phvul.003G177800	5.43	2.43	0.0008
Comp06.3	XLOC_008844	Phvul.003G190400	-9.65	0.9	0.0006
Comp06.3	XLOC_008854	Phvul.003G192800	-4.49	3.72	0.0047
Comp06.3	XLOC_007359	Phvul.003G209100	4.24	6.33	0.0066
Comp06.3	XLOC_007378	Phvul.003G212900	4.99	1.96	0.0062
Comp06.3	XLOC_007456	Phvul.003G227500	8.41	0.03	0.0097
Comp06.3	XLOC_007475	Phvul.003G230300	8.09	1.26	0
Comp06.3	XLOC_007505	Phvul.003G236200	-4.47	6.47	0.0046
Comp06.3	XLOC_007528	Phvul.003G239800	4.42	4.59	0.0061
Comp06.3	XLOC_009094	Phvul.003G247400	5.27	5.04	0.0009
Comp06.3	XLOC_009228	Phvul.003G278400	6.09	1.99	0.0004
Comp06.3	XLOC_007765	Phvul.003G281200	-5.09	3.34	0.0013
Comp06.3	XLOC_010367	Phvul.004G014600	4.77	2.83	0.0035
Comp06.3	XLOC_009571	Phvul.004G030500	-5.79	4.03	0.0003
Comp06.3	XLOC_010527	Phvul.004G046200	4.67	4.04	0.0029
Comp06.3	XLOC_010549	Phvul.004G049900	4.5	2.95	0.0047
Comp06.3	XLOC_009847	Phvul.004G090200	6.93	4.38	0
Comp06.3	XLOC_010789	Phvul.004G092100	5.09	6.35	0.001
Comp06.3	XLOC_010852	Phvul.004G103900	5.84	2.28	0.0005
Comp06.3	XLOC_009962	Phvul.004G111900	4.29	4.27	0.0061
Comp06.3	XLOC_009977	Phvul.004G117100	5.51	4.03	0.0004
Comp06.3	XLOC_010989	Phvul.004G129400	6.08	1.38	0.0013
Comp06.3	XLOC_010153	Phvul.004G150400	-8.39	0.35	0.0097
Comp06.3	XLOC_011125	Phvul.004G155100, Phvul.004G155200	4.23	3.2	0.0096
Comp06.3	XLOC_011146	Phvul.004G158800	5.54	2.74	0.0005
Comp06.3	XLOC_012357	Phvul.005G018500	-4.27	1.58	0.0099
Comp06.3	XLOC_012436	Phvul.005G032000	6.22	2.09	0.0009
Comp06.3	XLOC_011510	Phvul.005G034000	-6.13	3.26	0.0004
Comp06.3	XLOC_012515	Phvul.005G049000	-4.38	1.65	0.0094
Comp06.3	XLOC_011821	Phvul.005G096300	4.25	2.08	0.0097
Comp06.3	XLOC_011823	Phvul.005G097200, Phvul.005G097300	-4.46	3.49	0.005
Comp06.3	XLOC_011857	Phvul.005G106500	-4.84	1.8	0.0054
Comp06.3	XLOC_012856	Phvul.005G108800	5.46	3.42	0.0007
Comp06.3	XLOC_012861	Phvul.005G109700	4.67	1.46	0.0058
Comp06.3	XLOC_012906	Phvul.005G118900	-7.03	2.71	0.0005
Comp06.3	XLOC_012016	Phvul.005G138400	4.47	3.5	0.0047
Comp06.3	XLOC_012033	Phvul.005G141500	4.24	4.64	0.0069
Comp06.3	XLOC_012066	Phvul.005G147600	-4.21	2.91	0.0097
Comp06.3	XLOC_013160	Phvul.005G166800	-6.8	2.54	0.0001

Experimental comparison	Gene ID	Correspondent ID (Phytozome)	$log_2(FC)$[£]	$log_2(CPM)$[€]	FDR* (all significant at <0.01)
Comp06.3	XLOC_012189	Phvul.005G173500	-4.97	2.01	0.004
Comp06.3	XLOC_012195	Phvul.005G174400	-5.02	3.63	0.0015
Comp06.3	XLOC_013191	Phvul.005G174800	4.65	4.03	0.0029
Comp06.3	XLOC_013602	Phvul.006G046000	5.13	5.48	0.0009
Comp06.3	XLOC_013647	Phvul.006G056300	-6.18	3.56	0.0002
Comp06.3	XLOC_014802	Phvul.006G068600	-4.1	5.82	0.0094
Comp06.3	XLOC_014806	Phvul.006G069300	4.2	9.48	0.007
Comp06.3	XLOC_013763	Phvul.006G079500	7.46	4.84	0
Comp06.3	XLOC_013764	Phvul.006G079600	6.81	4.73	0
Comp06.3	XLOC_015037	Phvul.006G116000	7.25	4.99	0
Comp06.3	XLOC_014013	Phvul.006G127500	4.48	4.49	0.0045
Comp06.3	XLOC_014118	Phvul.006G150900	5.95	1.47	0.0009
Comp06.3	XLOC_014125	Phvul.006G153100	4.65	7.95	0.0026
Comp06.3	XLOC_015235	Phvul.006G156100	-5.29	1.64	0.0011
Comp06.3	XLOC_014194	Phvul.006G166000	5.41	1.42	0.0035
Comp06.3	XLOC_015295	Phvul.006G168600	6.2	3.39	0.0002
Comp06.3	XLOC_014301	Phvul.006G188500	7.48	6.31	0
Comp06.3	XLOC_014331	Phvul.006G194200	4.34	5.4	0.0054
Comp06.3	XLOC_015814	Phvul.007G036600	6.09	1.88	0.0012
Comp06.3	XLOC_015815	Phvul.007G036900	5.13	1.68	0.0029
Comp06.3	XLOC_017362	Phvul.007G056000	4.07	8.54	0.0097
Comp06.3	XLOC_017375	Phvul.007G058500	5.58	3.77	0.0005
Comp06.3	XLOC_015943	Phvul.007G061800	4.43	3.14	0.0084
Comp06.3	XLOC_017406	Phvul.007G066200	7.71	1.21	0.0001
Comp06.3	XLOC_015984	Phvul.007G069800	5.56	7.31	0.0003
Comp06.3	XLOC_016049	Phvul.007G082000	5.05	4.46	0.0012
Comp06.3	XLOC_017592	Phvul.007G100700	4.7	5.28	0.0023
Comp06.3	XLOC_017662	Phvul.007G113700	7.8	3.93	0
Comp06.3	XLOC_017691	Phvul.007G120200	-4.49	5.01	0.0043
Comp06.3	XLOC_017777	Phvul.007G137600	5.62	0.48	0.004
Comp06.3	XLOC_017998	Phvul.007G181100	-4.77	1.1	0.0097
Comp06.3	XLOC_016566	Phvul.007G183300	-4.19	4.07	0.0091
Comp06.3	XLOC_018216	Phvul.007G225200	7.39	4.59	0
Comp06.3	XLOC_016951	Phvul.007G258900	-4.15	4.33	0.0094
Comp06.3	XLOC_018388	Phvul.007G259400	5.52	6.7	0.0004
Comp06.3	XLOC_018711	Phvul.008G030300	-4.35	3.37	0.0069
Comp06.3	XLOC_018722	Phvul.008G032200	4.23	6.13	0.0069
Comp06.3	XLOC_020227	Phvul.008G032400	4.19	4.31	0.0084
Comp06.3	XLOC_018785	Phvul.008G043400	4.66	3.62	0.0046
Comp06.3	XLOC_020340	Phvul.008G055200	-9.06	0.5	0.0022
Comp06.3	XLOC_020346	Phvul.008G056800	4.51	3.6	0.0085
Comp06.3	XLOC_020416	Phvul.008G073200	-7.41	3.52	0
Comp06.3	XLOC_020517	Phvul.008G094400	4.36	3.44	0.0069
Comp06.3	XLOC_019518	Phvul.008G183400	9.45	-0.46	0.0009
Comp06.3	XLOC_020995	Phvul.008G186200	5.67	5.97	0.0003
Comp06.3	XLOC_019637	Phvul.008G207900	7.43	6.59	0
Comp06.3	XLOC_021169	Phvul.008G219800	5.53	4.23	0.0017
Comp06.3	XLOC_019741	Phvul.008G228000	12.63	3.06	0

Experimental comparison	Gene ID	Correspondent ID (Phytozome)	$log_2(FC)^£$	$log_2(CPM)^€$	FDR* (all significant at <0.01)
Comp06.3	XLOC_021212	Phvul.008G228100	4.41	5.94	0.0046
Comp06.3	XLOC_021293	Phvul.008G242900	4.82	4.09	0.0038
Comp06.3	XLOC_020009	Phvul.008G282200	-7.13	0.66	0.0004
Comp06.3	XLOC_021509	Phvul.008G287200	4.57	7.09	0.0083
Comp06.3	XLOC_021779	Phvul.009G018300	-5.64	2.54	0.0009
Comp06.3	XLOC_021896	Phvul.009G042800	4.42	3.21	0.005
Comp06.3	XLOC_023245	Phvul.009G046500	7.11	1.82	0.0004
Comp06.3	XLOC_021963	Phvul.009G057300	8.44	2.33	0.0092
Comp06.3	XLOC_021978	Phvul.009G059900	-5.5	0.78	0.0051
Comp06.3	XLOC_023383	Phvul.009G075100	4.25	7.04	0.0071
Comp06.3	XLOC_023416	Phvul.009G080200	5.23	5.4	0.0007
Comp06.3	XLOC_022176	Phvul.009G100300	-7.41	1.86	0.0003
Comp06.3	XLOC_022282	Phvul.009G120400	-5.1	1.49	0.0036
Comp06.3	XLOC_022286	Phvul.009G121000	-6.58	7.7	0
Comp06.3	XLOC_023669	Phvul.009G131000	8.63	-1.07	0.006
Comp06.3	XLOC_022357	Phvul.009G132900	5.1	4.58	0.0012
Comp06.3	XLOC_023684	Phvul.009G135300	-9.64	0.32	0.0006
Comp06.3	XLOC_022465	Phvul.009G153300	10.01	0.21	0.0003
Comp06.3	XLOC_023804	Phvul.009G161000	8.84	0.72	0.0037
Comp06.3	XLOC_022614	Phvul.009G182300	4.44	8.41	0.0041
Comp06.3	XLOC_024133	Phvul.009G224300	-4.89	1.68	0.0048
Comp06.3	XLOC_025341	Phvul.010G005600	4.68	3.45	0.0032
Comp06.3	XLOC_024487	Phvul.010G012300	4.87	2.77	0.0022
Comp06.3	XLOC_024490	Phvul.010G012900	4.94	3.43	0.0015
Comp06.3	XLOC_024811	Phvul.010G073500	5.2	3.16	0.0011
Comp06.3	XLOC_025672	Phvul.010G073900	8.94	-0.91	0.003
Comp06.3	XLOC_025735	Phvul.010G083700	4.98	4.79	0.0013
Comp06.3	XLOC_025845	Phvul.010G107400	5.49	7.04	0.0004
Comp06.3	XLOC_025075	Phvul.010G122500	5.57	4.03	0.0008
Comp06.3	XLOC_025100	Phvul.010G127500	4.64	1.48	0.009
Comp06.3	XLOC_025959	Phvul.010G129900	-6.55	5.47	0
Comp06.3	XLOC_026064	Phvul.010G154400	6.91	6.87	0
Comp06.3	XLOC_026094	Phvul.010G160900	-4.72	4.73	0.0047
Comp06.3	XLOC_025306	Phvul.010G163600	6.13	1.83	0.0004
Comp06.3	XLOC_026395	Phvul.011G037900	-6.28	2.67	0.0004
Comp06.3	XLOC_027581	Phvul.011G044700	5.25	0.39	0.0094
Comp06.3	XLOC_027784	Phvul.011G084300	4.62	7.38	0.0027
Comp06.3	XLOC_026682	Phvul.011G097100	7.67	3.89	0
Comp06.3	XLOC_026713	Phvul.011G102700	-4.36	6.08	0.0051
Comp06.3	XLOC_027899	Phvul.011G106300	4.41	1.93	0.0077
Comp06.3	XLOC_027984	Phvul.011G125300	9.24	0.92	0.0014
Comp06.3	XLOC_026910	Phvul.011G135600	4.92	0.75	0.0056
Comp06.3	XLOC_027333	Phvul.011G211600	5.49	4.35	0.0004
Comp06.3	XLOC_028531	Phvul.L008100	4.22	4.05	0.009
Comp07	XLOC_002827	new loci	9.35	1.61	0.0029
Comp07	XLOC_006748	new loci	-7.26	2.29	0.0012
Comp07	XLOC_006966	new loci	-5.91	1.15	0.0074
Comp07	XLOC_007865	new loci	9.63	1.62	0.0016

**Experimental comparison**	**Gene ID**	**Correspondent ID (Phytozome)**	**$log_2(FC)^£$**	**$log_2(CPM)^€$**	**FDR* (all significant at <0.01)**
Comp07	XLOC_010340	new loci	-6.38	1.59	0.0025
Comp07	XLOC_011757	new loci	-9.75	2.12	0.0012
Comp07	XLOC_011869	new loci	9.49	1.3	0.0024
Comp07	XLOC_018539	new loci	8.92	1.34	0.0078
Comp07	XLOC_018563	new loci	-9.76	1.18	0.0012
Comp07	XLOC_020681	new loci	-9.19	0.52	0.0044
Comp07	XLOC_020682	new loci	-9.37	0.64	0.0029
Comp07	XLOC_021560	new loci	6.65	0.83	0.0025
Comp07	XLOC_026888	new loci	-10.01	1.34	0.0007
Comp07	XLOC_027632	new loci	-9.35	0.91	0.0029
Comp07	XLOC_027916	new loci	-9.07	0.28	0.0059
Comp07	XLOC_028038	new loci	-9.12	0.19	0.0051
Comp07	XLOC_005185	Phvul.002G134700	-6.6	0.66	0.0014
Comp07	XLOC_005754	Phvul.002G244900	5.55	3.23	0.0012
Comp07	XLOC_007882	Phvul.003G002500	-5.63	4.23	0.0007
Comp07	XLOC_006456	Phvul.003G034200	-9.35	0.27	0.0029
Comp07	XLOC_006918	Phvul.003G124300	8.99	-0.44	0.007
Comp07	XLOC_007002	Phvul.003G138900	11.15	2.83	0
Comp07	XLOC_010334	Phvul.004G009000, Phvul.004G009100	-4.88	3.42	0.0051
Comp07	XLOC_009518	Phvul.004G021200	-6.53	2.99	0.0061
Comp07	XLOC_010597	Phvul.004G058400	-6.34	1.69	0.0005
Comp07	XLOC_009701	Phvul.004G058500	-9.45	-0.54	0.0025
Comp07	XLOC_010701	Phvul.004G076400	-10.7	7.42	0
Comp07	XLOC_011726	Phvul.005G077000	-6.02	3.98	0.0005
Comp07	XLOC_011828	Phvul.005G099500	4.9	4.44	0.0041
Comp07	XLOC_014622	Phvul.006G033000	-10.56	1.19	0.0002
Comp07	XLOC_019142	Phvul.008G109000	9.94	4	0.0007
Comp07	XLOC_019143	Phvul.008G109100	7.44	7.04	0.0001
Comp07	XLOC_020858	Phvul.008G160400	10.73	2.85	0.0001
Comp07	XLOC_019871	Phvul.008G252000	9.64	4.18	0
Comp07	XLOC_023133	Phvul.009G024300	-6.85	1.31	0.0005
Comp07	XLOC_023458	Phvul.009G087700	11.27	2.83	0
Comp07	XLOC_022283	Phvul.009G120500	8.62	4.41	0
Comp07	XLOC_022491	Phvul.009G158100	-9.98	0.11	0.0007
Comp07	XLOC_022901	Phvul.009G240600	-4.97	4.2	0.0031
Comp07	XLOC_025407	Phvul.010G019300	5.83	3.61	0.0044
Comp07	XLOC_024708	Phvul.010G054200	9.22	2.65	0
Comp07	XLOC_025091	Phvul.010G124800	-5.07	3.12	0.0031
Comp07	XLOC_027739	Phvul.011G076400	-4.85	5.03	0.0045
Comp07	XLOC_027168	Phvul.011G183600	9.97	1.51	0.0007
Comp07	XLOC_027224	Phvul.011G192400	5.41	1.84	0.003
Comp07	XLOC_027236	Phvul.011G194000, Phvul.011G194100	6.18	1.97	0.0011
Comp08	XLOC_011757	new loci	-9.19	2.12	0.0091
Comp08	XLOC_017321	new loci	-9.59	0.11	0.0042
Comp08	XLOC_026888	new loci	-9.47	1.34	0.005
Comp08	XLOC_027632	new loci	-10.83	0.91	0.0001

Experimental comparison	Gene ID	Correspondent ID (Phytozome)	$log_2(FC)^£$	$log_2(CPM)^€$	FDR* (all significant at <0.01)
Comp08	XLOC_008018	Phvul.003G030500	-10.25	0.21	0.0006
Comp08	XLOC_009518	Phvul.004G021200	-12.21	2.99	0
Comp08	XLOC_010701	Phvul.004G076400	-12.18	7.42	0
Comp08	XLOC_011726	Phvul.005G077000	-5.9	3.98	0.0022
Comp08	XLOC_011828	Phvul.005G099500	11.27	4.44	0
Comp08	XLOC_014622	Phvul.006G033000	-9.26	1.19	0.008
Comp08	XLOC_019142	Phvul.008G109000	12.73	4	0
Comp08	XLOC_019143	Phvul.008G109100	15.65	7.04	0
Comp08	XLOC_019871	Phvul.008G252000	5.62	4.18	0.0047
Comp08	XLOC_022283	Phvul.009G120500	5.9	4.41	0.0022
Comp08	XLOC_027739	Phvul.011G076400	-5.14	5.03	0.0054
Comp08	XLOC_027168	Phvul.011G183600	9.4	1.51	0.0057
Comp08	XLOC_027232	Phvul.011G193600	5.76	2.65	0.0074
Comp09	XLOC_000376	Phvul.001G076700	-5.57	8.13	0.0029
Comp09	XLOC_017043	Phvul.007G275700	-6.38	2.24	0.002
Comp09	XLOC_017050	Phvul.007G277000	-11.34	1.82	0.0001
Comp09	XLOC_027984	Phvul.011G125300	6.38	0.92	0.0083
Comp10	XLOC_000483	new loci	10.73	0.85	0.0001
Comp10	XLOC_002827	new loci	9.4	1.61	0.0021
Comp10	XLOC_005373	new loci	-9.26	-0.33	0.0027
Comp10	XLOC_006966	new loci	-6.53	1.15	0.0036
Comp10	XLOC_007865	new loci	9.43	1.62	0.0019
Comp10	XLOC_009463	new loci	7.63	1.5	0.0002
Comp10	XLOC_010340	new loci	-9.67	1.59	0.001
Comp10	XLOC_010758	new loci	4.7	2.9	0.0069
Comp10	XLOC_011757	new loci	-8.48	2.12	0
Comp10	XLOC_011869	new loci	9.96	1.3	0.0004
Comp10	XLOC_012682	new loci	-8.91	0.33	0.0056
Comp10	XLOC_013284	new loci	-10.74	1.14	0.0001
Comp10	XLOC_013644	new loci	9.28	-0.04	0.0027
Comp10	XLOC_015623	new loci	-9.3	-0.27	0.0025
Comp10	XLOC_018563	new loci	-6.37	1.18	0.0048
Comp10	XLOC_020682	new loci	-8.94	0.64	0.0053
Comp10	XLOC_021571	new loci	8.98	-0.45	0.0048
Comp10	XLOC_021572	new loci	10.65	1.09	0.0001
Comp10	XLOC_024793	new loci	10.45	1.63	0.0001
Comp10	XLOC_026888	new loci	-9.53	1.34	0.0014
Comp10	XLOC_026955	new loci	8.84	0.65	0.0069
Comp10	XLOC_000650	Phvul.001G128500	-4.9	6.36	0.0028
Comp10	XLOC_001043	Phvul.001G205900	-5.02	7.01	0.003
Comp10	XLOC_004851	Phvul.002G072000	-4.52	6.14	0.0087
Comp10	XLOC_003411	Phvul.002G108200	9.18	0.93	0.003
Comp10	XLOC_003974	Phvul.002G228700	6	5.78	0.0003
Comp10	XLOC_004436	Phvul.002G319000	-5.01	6.59	0.0023
Comp10	XLOC_007886	Phvul.003G002800, Phvul.003G002900	5.42	5.26	0.0036
Comp10	XLOC_006733	Phvul.003G089500	7.11	2.83	0.0006
Comp10	XLOC_007002	Phvul.003G138900	10.1	2.83	0.0003

**Experimental comparison**	**Gene ID**	**Correspondent ID (Phytozome)**	**log$_2$(FC)$^{£}$**	**log$_2$(CPM)$^{€}$**	**FDR*<br>(all significant at <0.01)**
Comp10	XLOC_009518	Phvul.004G021200	-7.49	2.99	0.0003
Comp10	XLOC_010581	Phvul.004G054800	7.06	1.56	0.0002
Comp10	XLOC_010701	Phvul.004G076400	-10.2	7.42	0
Comp10	XLOC_009909	Phvul.004G101500	-5.92	4.48	0.0003
Comp10	XLOC_010110	Phvul.004G142400	8.99	5.56	0
Comp10	XLOC_010111	Phvul.004G142500	11.99	3.05	0
Comp10	XLOC_010112	Phvul.004G142600	9.81	6.68	0
Comp10	XLOC_010113	Phvul.004G142700	11.01	2.42	0
Comp10	XLOC_011206	Phvul.004G168400	4.42	4.28	0.0097
Comp10	XLOC_012336	Phvul.005G014000	5.35	1.16	0.0042
Comp10	XLOC_011579	Phvul.005G047200	-10.97	1.81	0
Comp10	XLOC_011611	Phvul.005G053400	-9.65	0.05	0.001
Comp10	XLOC_011726	Phvul.005G077000	-5.12	3.98	0.0026
Comp10	XLOC_012719	Phvul.005G084700	9.52	1.61	0.0014
Comp10	XLOC_011828	Phvul.005G099500	7.05	4.44	0
Comp10	XLOC_011904	Phvul.005G116500	9.33	1.93	0.0025
Comp10	XLOC_014622	Phvul.006G033000	-9.22	1.19	0.0029
Comp10	XLOC_014719	Phvul.006G054500	6.62	0.46	0.0025
Comp10	XLOC_013796	Phvul.006G084900	-8.98	1.87	0.0048
Comp10	XLOC_015235	Phvul.006G156100	-8.58	1.64	0
Comp10	XLOC_017318	Phvul.007G048800	9.14	2.57	0.0033
Comp10	XLOC_018697	Phvul.008G028400,<br>Phvul.008G028500	-6.47	1.35	0.004
Comp10	XLOC_018970	Phvul.008G077500	-10.11	2.25	0.0003
Comp10	XLOC_020445	Phvul.008G079700	-10.22	1.68	0.0002
Comp10	XLOC_020490	Phvul.008G089400	-8.84	2.13	0.0069
Comp10	XLOC_019148	Phvul.008G109600	6.52	0.84	0.0029
Comp10	XLOC_020675	Phvul.008G127000	11.97	3.57	0
Comp10	XLOC_020858	Phvul.008G160400	5.14	2.85	0.0039
Comp10	XLOC_019543	Phvul.008G188200	6.32	2.44	0.0044
Comp10	XLOC_021207	Phvul.008G227100	6.54	2.55	0.0003
Comp10	XLOC_021216	Phvul.008G228900	4.96	0.73	0.0087
Comp10	XLOC_021218	Phvul.008G229100	-8.42	3.79	0
Comp10	XLOC_023458	Phvul.009G087700	9.33	2.83	0
Comp10	XLOC_023989	Phvul.009G196200	5.08	2.17	0.0087
Comp10	XLOC_024498	Phvul.010G014100	-9.24	0.95	0.0028
Comp10	XLOC_024530	Phvul.010G021000	-9.08	1.46	0.0039
Comp10	XLOC_025152	Phvul.010G136700	-10.42	2.88	0.0001
Comp10	XLOC_027739	Phvul.011G076400	-4.73	5.03	0.0048
Comp10	XLOC_026951	Phvul.011G142600	6.65	2.98	0.0001
Comp10	XLOC_026983	Phvul.011G147800	-4.79	7.56	0.0042
Comp10	XLOC_028253	Phvul.011G182900	-11.62	2.7	0
Comp10	XLOC_027171	Phvul.011G183900	5.87	2.93	0.0003
Comp10	XLOC_027234	Phvul.011G193800	5.72	2.41	0.0028
Comp10	XLOC_027235	Phvul.011G193900	6.4	2.02	0.0038
Comp10	XLOC_028499	Phvul.L006900	10.56	0.84	0.0001
Comp10	XLOC_028555	Phvul.L009500	-6.08	2.45	0.0028
Comp11	XLOC_000483	new loci	8.78	0.85	0.0074

Experimental comparison	Gene ID	Correspondent ID (Phytozome)	$log_2(FC)^£$	$log_2(CPM)^€$	FDR* (all significant at <0.01)
Comp11	XLOC_002350	new loci	-9.1	-0.14	0.0037
Comp11	XLOC_006966	new loci	-5.39	1.15	0.0088
Comp11	XLOC_007865	new loci	8.76	1.62	0.0074
Comp11	XLOC_009395	new loci	-5.15	0.07	0.0099
Comp11	XLOC_009463	new loci	9.69	1.5	0.0009
Comp11	XLOC_010340	new loci	-9.75	1.59	0.0008
Comp11	XLOC_011757	new loci	-10.1	2.12	0.0003
Comp11	XLOC_011869	new loci	8.74	1.3	0.0077
Comp11	XLOC_012682	new loci	-8.69	0.33	0.0094
Comp11	XLOC_013284	new loci	-9.25	1.14	0.0025
Comp11	XLOC_020682	new loci	-8.69	0.64	0.0094
Comp11	XLOC_021572	new loci	8.87	1.09	0.0059
Comp11	XLOC_024208	new loci	4.96	1.68	0.0075
Comp11	XLOC_024327	new loci	-8.79	-0.36	0.0074
Comp11	XLOC_024793	new loci	10.43	1.63	0.0001
Comp11	XLOC_026888	new loci	-9.52	1.34	0.0014
Comp11	XLOC_026955	new loci	9.01	0.65	0.0042
Comp11	XLOC_000191	Phvul.001G040600	-5.48	1.05	0.0023
Comp11	XLOC_000650	Phvul.001G128500	-5.81	6.36	0.0003
Comp11	XLOC_002096	Phvul.001G145700	-4.6	3.35	0.0063
Comp11	XLOC_005579	Phvul.002G209400	-4.85	5.34	0.0059
Comp11	XLOC_003883	Phvul.002G209500	-4.88	5.61	0.0034
Comp11	XLOC_003974	Phvul.002G228700	7.05	5.78	0.0001
Comp11	XLOC_004436	Phvul.002G319000	-5.83	6.59	0.0002
Comp11	XLOC_006535	Phvul.003G051700	-4.65	9.83	0.0056
Comp11	XLOC_006733	Phvul.003G089500	6.32	2.83	0.0025
Comp11	XLOC_007002	Phvul.003G138900	9.58	2.83	0.0012
Comp11	XLOC_009226	Phvul.003G277900	-4.77	2.32	0.0076
Comp11	XLOC_009518	Phvul.004G021200	-10.92	2.99	0
Comp11	XLOC_009635	Phvul.004G044800	-6.79	1.87	0.0001
Comp11	XLOC_010581	Phvul.004G054800	9.96	1.56	0.0005
Comp11	XLOC_010701	Phvul.004G076400	-10.68	7.42	0
Comp11	XLOC_009837	Phvul.004G088400	-4.84	2.05	0.0069
Comp11	XLOC_009895	Phvul.004G099100	-4.38	3.78	0.0088
Comp11	XLOC_009974	Phvul.004G116600	-5.21	1.85	0.0025
Comp11	XLOC_010110	Phvul.004G142400	11.39	5.56	0
Comp11	XLOC_010111	Phvul.004G142500	12.11	3.05	0
Comp11	XLOC_010112	Phvul.004G142600	10.8	6.68	0
Comp11	XLOC_010113	Phvul.004G142700	11.46	2.42	0
Comp11	XLOC_011124	Phvul.004G155000	-5.22	4.33	0.0012
Comp11	XLOC_011579	Phvul.005G047200	-7.63	1.81	0.0003
Comp11	XLOC_011611	Phvul.005G053400	-8.97	0.05	0.0051
Comp11	XLOC_011726	Phvul.005G077000	-5.37	3.98	0.0016
Comp11	XLOC_012719	Phvul.005G084700	9.87	1.61	0.0006
Comp11	XLOC_011828	Phvul.005G099500	6.75	4.44	0.001
Comp11	XLOC_012857	Phvul.005G109000	-4.4	5.48	0.0077
Comp11	XLOC_011904	Phvul.005G116500	10.11	1.93	0.0003
Comp11	XLOC_012134	Phvul.005G163100	-4.59	3	0.006

Experimental comparison	Gene ID	Correspondent ID (Phytozome)	$log_2(FC)$[£]	$log_2(CPM)$[€]	FDR* (all significant at <0.01)
Comp11	XLOC_013793	Phvul.006G084600	-5.29	2	0.0057
Comp11	XLOC_013796	Phvul.006G084900	-5.39	1.87	0.003
Comp11	XLOC_015235	Phvul.006G156100	-9.67	1.64	0.001
Comp11	XLOC_015440	Phvul.006G197200	-6.51	4.75	0.0001
Comp11	XLOC_017318	Phvul.007G048800	9.59	2.57	0.0011
Comp11	XLOC_016088	Phvul.007G091000	-5.17	2.35	0.003
Comp11	XLOC_018697	Phvul.008G028400, Phvul.008G028500	-5.62	1.35	0.0053
Comp11	XLOC_020285	Phvul.008G044900	-4.86	5.6	0.0029
Comp11	XLOC_018970	Phvul.008G077500	-7.54	2.25	0
Comp11	XLOC_020445	Phvul.008G079700	-7.36	1.68	0.0001
Comp11	XLOC_020490	Phvul.008G089400	-9.44	2.13	0.0016
Comp11	XLOC_019058	Phvul.008G093200	-5.05	2.91	0.0025
Comp11	XLOC_019131	Phvul.008G106500	-5.01	1.61	0.0042
Comp11	XLOC_019148	Phvul.008G109600	9.76	0.84	0.0008
Comp11	XLOC_020675	Phvul.008G127000	12.79	3.57	0
Comp11	XLOC_020742	Phvul.008G140000	-6.62	2.23	0.0002
Comp11	XLOC_020858	Phvul.008G160400	10.45	2.85	0.0001
Comp11	XLOC_019450	Phvul.008G169600	-5.54	1.29	0.0025
Comp11	XLOC_019543	Phvul.008G188200	9.69	2.44	0.0009
Comp11	XLOC_021207	Phvul.008G227100	5.59	2.55	0.0022
Comp11	XLOC_021218	Phvul.008G229100	-9.06	3.79	0
Comp11	XLOC_021317	Phvul.008G249900	-8.79	1.57	0.0074
Comp11	XLOC_021349	Phvul.008G256600	-4.67	2.27	0.0093
Comp11	XLOC_021479	Phvul.008G279800	4.43	5.31	0.0075
Comp11	XLOC_021896	Phvul.009G042800	-4.91	3.21	0.0058
Comp11	XLOC_023228	Phvul.009G043200	-4.88	2.17	0.0042
Comp11	XLOC_022896	Phvul.009G239700	-5	3.81	0.0021
Comp11	XLOC_024207	Phvul.009G239900	-5.31	4.8	0.001
Comp11	XLOC_022900	Phvul.009G240500	-7.48	1.89	0.0005
Comp11	XLOC_024444	Phvul.010G004900	-5.19	1.73	0.0058
Comp11	XLOC_024498	Phvul.010G014100	-9.05	0.95	0.0042
Comp11	XLOC_025405	Phvul.010G019000	-8.82	-0.72	0.0072
Comp11	XLOC_024530	Phvul.010G021000	-7.77	1.46	0.0002
Comp11	XLOC_024581	Phvul.010G031900	-6.95	0.15	0.0018
Comp11	XLOC_025907	Phvul.010G120200	-5.22	3.56	0.0082
Comp11	XLOC_025152	Phvul.010G136700	-11.2	2.88	0
Comp11	XLOC_027739	Phvul.011G076400	-5.42	5.03	0.001
Comp11	XLOC_026951	Phvul.011G142600	5.59	2.98	0.0016
Comp11	XLOC_028253	Phvul.011G182900	-11.08	2.7	0
Comp11	XLOC_028254	Phvul.011G183000	-9.82	5.22	0.0007
Comp11	XLOC_027165	Phvul.011G183300	-5.64	1.2	0.0049
Comp11	XLOC_027171	Phvul.011G183900	5.58	2.93	0.0016
Comp11	XLOC_027218	Phvul.011G191400	-9.5	1.31	0.0015
Comp11	XLOC_027234	Phvul.011G193800	5.56	2.41	0.0016
Comp11	XLOC_027235	Phvul.011G193900	10.6	2.02	0.0001
Comp11	XLOC_028638	Phvul.L006800	-5.75	1.24	0.0037
Comp11	XLOC_028499	Phvul.L006900	9.45	0.84	0.0016

Experimental comparison	Gene ID	Correspondent ID (Phytozome)	$log_2(FC)^{£}$	$log_2(CPM)^{€}$	FDR* (all significant at <0.01)
Comp11	XLOC_028555	Phvul.L009500	-10.74	2.45	0.0001
Comp12	XLOC_002827	new loci	9.44	1.61	0.002
Comp12	XLOC_006748	new loci	-9.17	2.29	0.0041
Comp12	XLOC_007865	new loci	8.95	1.62	0.0065
Comp12	XLOC_009335	new loci	-8.98	-0.68	0.0062
Comp12	XLOC_009463	new loci	9	1.5	0.006
Comp12	XLOC_010340	new loci	-9.79	1.59	0.0009
Comp12	XLOC_011258	new loci	9.13	0.91	0.0045
Comp12	XLOC_011313	new loci	9.02	1.09	0.0058
Comp12	XLOC_012682	new loci	-9.56	0.33	0.0017
Comp12	XLOC_018539	new loci	8.83	1.34	0.0088
Comp12	XLOC_024208	new loci	6.35	1.68	0.0018
Comp12	XLOC_024793	new loci	9.76	1.63	0.001
Comp12	XLOC_001651	Phvul.001G055000	-6.24	1.31	0.0073
Comp12	XLOC_000573	Phvul.001G112400	-5.4	6.22	0.0019
Comp12	XLOC_005531	Phvul.002G200600	-5.09	2.81	0.0031
Comp12	XLOC_003938	Phvul.002G219100	-5.23	0.9	0.0058
Comp12	XLOC_003974	Phvul.002G228700	5.38	5.78	0.0016
Comp12	XLOC_004436	Phvul.002G319000	-5.77	6.59	0.0004
Comp12	XLOC_006733	Phvul.003G089500	9.85	2.83	0.0009
Comp12	XLOC_007002	Phvul.003G138900	9.9	2.83	0.0008
Comp12	XLOC_010333	Phvul.004G008900	-5.83	1.75	0.0053
Comp12	XLOC_009477	Phvul.004G012800	-4.8	3.36	0.0093
Comp12	XLOC_009518	Phvul.004G021200	-10.8	2.99	0.0001
Comp12	XLOC_010701	Phvul.004G076400	-11.43	7.42	0
Comp12	XLOC_009909	Phvul.004G101500	-5.75	4.48	0.0007
Comp12	XLOC_010110	Phvul.004G142400	8.16	5.56	0
Comp12	XLOC_010111	Phvul.004G142500	10.88	3.05	0.0001
Comp12	XLOC_010112	Phvul.004G142600	6.84	6.68	0.0001
Comp12	XLOC_010113	Phvul.004G142700	10.75	2.42	0.0001
Comp12	XLOC_011579	Phvul.005G047200	-9.89	1.81	0.0008
Comp12	XLOC_011726	Phvul.005G077000	-5.39	3.98	0.002
Comp12	XLOC_012719	Phvul.005G084700	6.47	1.61	0.0053
Comp12	XLOC_011904	Phvul.005G116500	8.52	1.93	0
Comp12	XLOC_018027	Phvul.007G185700	5.25	3.53	0.0036
Comp12	XLOC_018697	Phvul.008G028400, Phvul.008G028500	-9.83	1.35	0.0009
Comp12	XLOC_020675	Phvul.008G127000	11.47	3.57	0
Comp12	XLOC_019543	Phvul.008G188200	6	2.44	0.0042
Comp12	XLOC_021218	Phvul.008G229100	-8.52	3.79	0
Comp12	XLOC_019956	Phvul.008G270500	-10.39	0.82	0.0002
Comp12	XLOC_024444	Phvul.010G004900	-6.81	1.73	0.0019
Comp12	XLOC_024498	Phvul.010G014100	-10.34	0.95	0.0002
Comp12	XLOC_024530	Phvul.010G021000	-6.18	1.46	0.002
Comp12	XLOC_025146	Phvul.010G135800	5.47	7.94	0.0053
Comp12	XLOC_025152	Phvul.010G136700	-8.26	2.88	0.0001
Comp12	XLOC_026461	Phvul.011G051300	8.88	-0.19	0.0079
Comp12	XLOC_027739	Phvul.011G076400	-4.72	5.03	0.0062

Experimental comparison	Gene ID	Correspondent ID (Phytozome)	$log_2(FC)^£$	$log_2(CPM)^€$	FDR* (all significant at <0.01)
Comp12	XLOC_027830	Phvul.011G091300	-9.48	0.11	0.0019
Comp12	XLOC_026951	Phvul.011G142600	6.39	2.98	0.0017
Comp12	XLOC_028072	Phvul.011G147100	-9.39	0.27	0.0021
Comp12	XLOC_028253	Phvul.011G182900	-11.66	2.7	0
Comp12	XLOC_028254	Phvul.011G183000	-7.5	5.22	0.0004
Comp12	XLOC_027165	Phvul.011G183300	-6.91	1.2	0.0016
Comp12	XLOC_027218	Phvul.011G191400	-8.87	1.31	0.0084
Comp12	XLOC_027234	Phvul.011G193800	5.85	2.41	0.002
Comp12	XLOC_027235	Phvul.011G193900	6.13	2.02	0.0017
Comp12	XLOC_027313	Phvul.011G208300	-5.6	0.57	0.0088
Comp12	XLOC_028555	Phvul.L009500	-9.88	2.45	0.0008

$^£log_2(FC) = log_2$ (Fold Change)

$^€log_2(CPM) = log_2$ (Counts per million)

*FDR = False Discovery Rate *at 1%*
