# Supplementary material for: Genome-wide analysis of the transcriptional response to drought stress in root and leaf of common bean
Source: Genet Mol Biol. 2020 Mar 16;43(1):e20180259. doi: 10.1590/1678-4685-GMB-2018-0259 (PMC7307723; doi:10.1590/1678-4685-GMB-2018-0259)
Supplement: Supplementary file 9 [file 1415-4757-GMB-43-1-e20180259-s10.pdf]

Supplementary Material to: “Genome-wide analysis of the transcriptional response to drought stress in root and leaf of common”

Table S8. Number of variants identified after each Base Quality Score Recalibration (BQSR) step.

**Variants \ Haplotype caller step**		**Without BQSR**	**First BQSR**	**Second BQSR**
BAT 477	SNPs	119,371	119,573	119,572
	Insertions	7,141	6,596	6,591
	Deletions	7,560	7,260	7,261
Pérola	SNPs	96,061	96,198	96,192
	Insertions	6,180	5,643	5,638
	Deletions	6,697	6,411	6,412
All	SNPs	134,870	135,175	135,167
	Insertions	8,194	7,656	7,652
	Deletions	8,749	8,462	8,464
**Total**		**151,813**	**151,293**	**151,283**
