# Supplementary material for: Genome-wide analysis of the transcriptional response to drought stress in root and leaf of common bean
Source: Genet Mol Biol. 2020 Mar 16;43(1):e20180259. doi: 10.1590/1678-4685-GMB-2018-0259 (PMC7307723; doi:10.1590/1678-4685-GMB-2018-0259)
Supplement: Supplementary file 10 [file 1415-4757-GMB-43-1-e20180259-s11.pdf]

Supplementary Material to: “Genome-wide analysis of the transcriptional response to drought stress in root and leaf of common”

Table S9 Description of TaqMan probes with identification of their efficiency of amplification and correspondent annotation based on BioMart tool of Phytozome v.12.

ID	Probe ID*	Efficiency of amplif. (%)	Gene ID	Description	PFAM Description	Panther Description	EC ID	EC Description	GO ID	SpTrembl ID
**AQP**	AI0IXEL	94.43	Phvul.006G171000	(M=11) K09874 - aquaporin NIP	Major intrinsic protein	AQUAPORIN TRANSPORTER			GO:0016020, GO:0006810, GO:0005215	V7BSF7
**PBR**	AII1MZI	92.41	Phvul.001G205900	(M=1) KOG3797 - Peripheral-type benzodiazepine receptor and related proteins	TspO/MBR family	PERIPHERAL-TYPE BENZODIAZEPINE RECEPTOR			GO:0016021	V7D0Q0
**DNAJC**	AI39RW9	93.65	Phvul.006G060700	(M=22) PTHR24078 - DNAJ HOMOLOG SUBFAMILY C MEMBER	DnaJ domain	DNAJ HOMOLOG SUBFAMILY C MEMBER				V7BNS8
**GH32**	AIKAK5Q	105.81	Phvul.005G158500	(M=12) K01193 - beta-fructofuranosidase	Glycosyl hydrolases family 32 C terminal, Glycosyl hydrolases family 32 N-terminal domain	FAMILY NOT NAMED	3.2.1.26	Beta-fructofuranosidase.		V7C0T3
**CHIA**	AIX0015	103.83	Phvul.005G155800	(M=2) PTHR22595:SF7 - CLASS IV CHITINASE, Insoluble isoenzyme CWINV1-Related	Chitin recognition protein, Chitinase class I	CHITINASE-RELATED	3.2.1.14	Chitinase.	GO:0008061, GO:0016998, GO:0006032, GO:0004568, GO:0008061, GO:0016998, GO:0006032, GO:0004568	V7C0P5
**PP2C**	AIHSOTA	93.12	Phvul.001G075400	(M=9) PTHR13832//PTHR13832:SF115 - PROTEIN PHOSPHATASE 2C // SUBFAMILY NOT NAMED	Protein phosphatase 2C	PROTEIN PHOSPHATASE 2C	3.1.3.16	Phosphoprotein phosphatase.	GO:0003824, GO:0006470, GO:0004722	V7CW57
**GST**	AIWR2VX	98.3	Phvul.008G113700	(M=14) PTHR11260:SF16 - GLUTATHIONE S-TRANSFERASE	Glutathione S-transferase, C-terminal domain, Glutathione S-transferase, N-terminal domain	GLUTATHIONE S-TRANSFERASE, GST, SUPERFAMILY, GST DOMAIN CONTAINING	2.5.1.18	Glutathione transferase.	GO:0005515	V7B3P4
**HSF**	AI70MFX	94.46	Phvul.007G061800	(M=30) PTHR10015 - HEAT SHOCK TRANSCRIPTION FACTOR	HSF-type DNA-binding	HEAT SHOCK TRANSCRIPTION FACTOR			GO:0043565, GO:0006355, GO:0005634, GO:0003700, GO:0009408	V7BEI8
**LEA**	AI20TQ1	90.69	Phvul.007G259400	(M=5) PF03760 - Late embryogenesis abundant (LEA) group 1	Late embryogenesis abundant (LEA) group 1				GO:0009790	V7BM85
**LOX**	AICSV4E	94.21	Phvul.002G228700	(M=31) 1.13.11.12 - Linoleate 13S-lipoxygenase.	PLAT/LH2 domain, Lipoxygenase	LIPOXYGENASE	1.13.11.12	Linoleate 13S-lipoxygenase.	GO:0005515, GO:0055114,	V7CPZ8

ID	Probe ID*	Efficiency of amplif. (%)	Gene ID	Description	PFAM Description	Panther Description	EC ID	EC Description	GO ID	SpTrembl ID
									GO:0046872, GO:0016702, GO:0005515, GO:0055114, GO:0046872, GO:0016702	
**MYB**	AIY9Y8D	91.26	Phvul.002G184600	(M=74) K09422 - myb proto-oncogene protein, plant	Myb-like DNA-binding domain	MYB-LIKE DNA-BINDING PROTEIN MYB				V7CKV2
**NAM_NAC**	AIS08C9	92.44	Phvul.005G084500	(M=90) PF02365 - No apical meristem (NAM) protein, NAC Domain-containing protein 9-related	No apical meristem (NAM) protein	FAMILY NOT NAMED			GO:0006355, GO:0003677	V7BX33
**NADH**	AIVI4PP	96.18	Phvul.003G131500	(M=11) 1.3.1.42 - 12-oxophytodienoate reductase.	NADH:flavin oxidoreductase / NADH oxidase family	NADH OXIDOREDUCTASE-RELATED	1.3.1.42	12-oxophytodienoate reductase.	GO:0055114, GO:0016491, GO:0010181	V7CB53
**POX**	AIT96JH	98.71	Phvul.009G140700	(M=88) 1.11.1.7 - Peroxidase.	Peroxidase	FAMILY NOT NAMED	1.11.1.7	Peroxidase.	GO:0055114, GO:0020037, GO:0006979, GO:0004601	V7AVB3
**GH10**	AIBJXX6	95.19	Phvul.009G120500	(M=7) PF00331 - Glycosyl hydrolase family 10	Glycosyl hydrolase family 10	FAMILY NOT NAMED	3.2.1.8	Endo-1,4-beta-xylanase.	GO:0005975, GO:0004553	V7AYM8

* Available at Thermo Fisher Scientific™
