# Supplementary material for: Genome-wide analysis of the transcriptional response to drought stress in root and leaf of common bean
Source: Genet Mol Biol. 2020 Mar 16;43(1):e20180259. doi: 10.1590/1678-4685-GMB-2018-0259 (PMC7307723; doi:10.1590/1678-4685-GMB-2018-0259)
Supplement: Supplementary file 11 [file 1415-4757-GMB-43-1-e20180259-s2.pdf]

Genetics and Molecular Biology 43, 1, (2020)

Supplementary Material to: "Genome-wide analysis of the transcriptional response to drought stress in root and leaf of common bean"

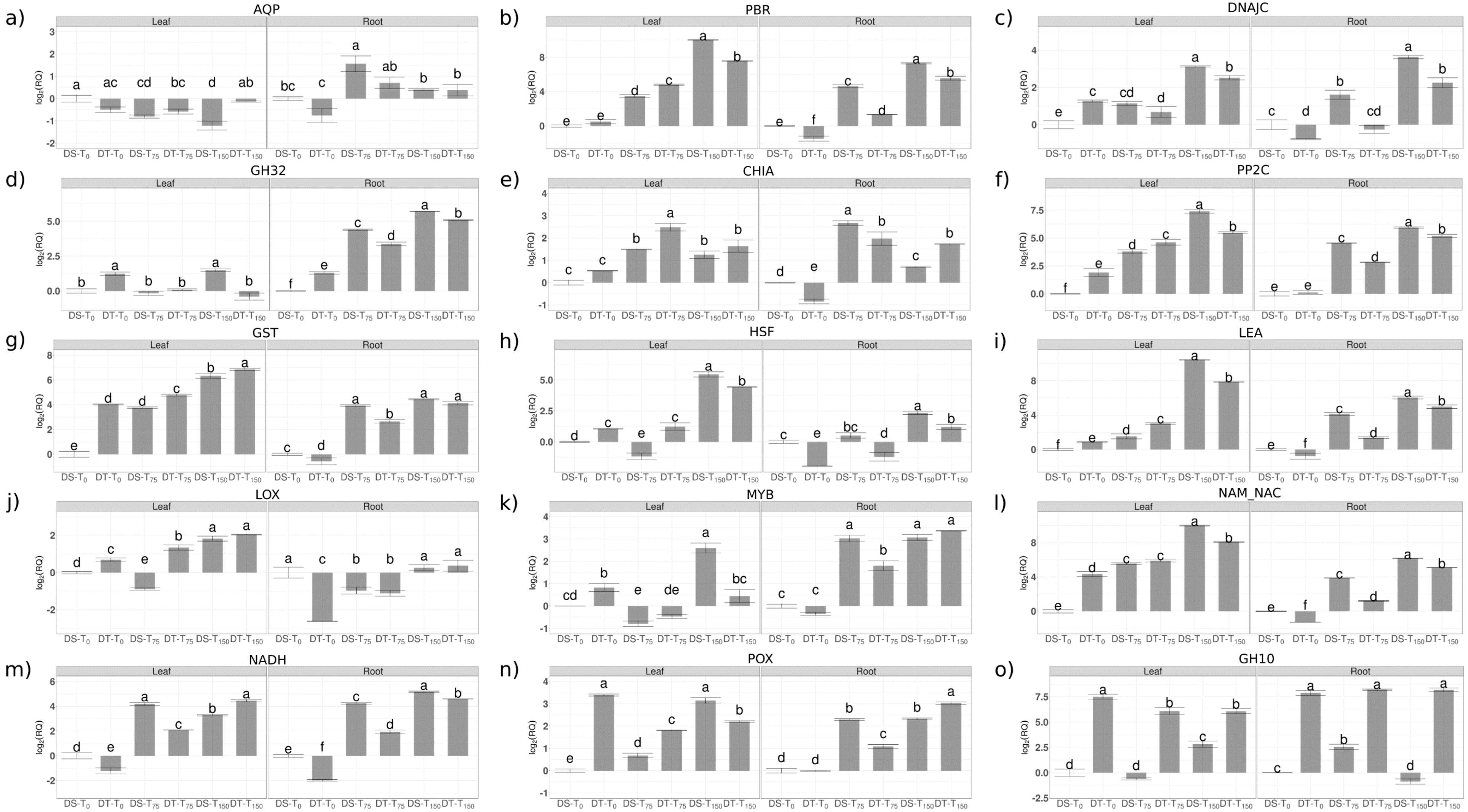

Figure S2 - Expression profile for the 15 genes selected for qPCR validation.
